# Box–Behnken Design Optimization of High-Pressure Processed Bitter Melon (*Momordica charantia*) Leaf Extract Enhancing Phytochemicals, Anticancer, and Anti-Inflammatory Activities

**DOI:** 10.3390/ijms27114945

**Published:** 2026-05-29

**Authors:** Kongsakon Kulchim, Sukan Braspaiboon, Pornsiri Pitchakarn, Arisa Imsumran, Pensiri Buacheen, Tanongsak Laowanitwattana, Piya Temviriyanukul, Kongthawat Chairatvit, Ariyaphong Wongnoppavich

**Affiliations:** 1 Graduate/Ph.D. Program in Biochemistry, Faculty of Medicine, Chiang Mai University, Chiang Mai 50200, Thailand; kongsakon_kulchim@cmu.ac.th; 2 Department of Biochemistry, Faculty of Medicine, Chiang Mai University, Chiang Mai 50200, Thailand; pornsiri.p@cmu.ac.th (P.P.); arisa.bonness@cmu.ac.th (A.I.); pensiri.bua@cmu.ac.th (P.B.); tanongsak.l@cmu.ac.th (T.L.); 3 Faculty of Agro-Industry, Chiang Mai University, Chiang Mai 50100, Thailand; sukan.bras@cmu.ac.th; 4 Institute of Nutrition, Mahidol University, Salaya, Nakhon Pathom 73170, Thailand; piya.tem@mahidol.ac.th; 5 Department of Oral Biology, Faculty of Dentistry, Mahidol University, Bangkok 10400, Thailand; kongthawat.cha@mahidol.ac.th

**Keywords:** anticancer, anti-inflammation, high-pressure processing, JNK signaling, *Momordica charantia*, non-thermal extraction, phytometabolomics, response surface methodology

## Abstract

Bitter Melon Leaf Extract (BMLE) possesses potential anticancer and anti-inflammatory properties; however, conventional extraction methods restrict phytochemical yield and bioactivity. Here, we optimized extraction using High-Pressure Processing (HPP) with Box–Behnken Design (BBD) and Response Surface Methodology (RSM). The optimized extract (O-BMLE) demonstrated significantly higher total flavonoid content (27.7 vs. 8.7 mg RE/g) and FRAP antioxidant capacity (96.5 vs. 71.2 μmol TE/g) compared to conventional BMLE. Additionally, O-BMLE exhibited enhanced cytotoxicity (A549 IC_50_: 58.7 vs. 147 μg/mL) and selectivity (SI: 5.03 vs. 2.60) against A549, HepG2, and SKOV3 cancer cells while showing minimal effects on 3T3-L1 fibroblasts. In LPS-stimulated RAW264.7 macrophages, O-BMLE selectively inhibited JNK phosphorylation without affecting NF-κB phosphorylation, resulting in suppression of iNOS, COX-2, IL-1β, IL-6, and TNF-α expression as well as nitric oxide production. HPLC analysis revealed equivalent momordicine-I levels (~28 mg/g) between extracts. In contrast, HPLC-qTOF-MS profiling revealed that O-BMLE was enriched in stearidonic acid (66% increase in relative abundance), 4-hydroxybenzoic acid (19.5%), monolinolenin, 6-gingerol, and pedunculoside, which are compounds linked to JNK inhibition, antioxidant activity, and cytokine suppression. These results indicate that HPP-BBD/RSM optimization selectively modifies the bitter melon leaf metabolome, thereby enhancing anticancer and anti-inflammatory activities independently of momordicine-I content alone. O-BMLE may therefore serve as a promising candidate for the development of functional foods and nutraceuticals targeting inflammation-associated cancers.

## 1. Introduction

Inflammation is an important physiological process of the host in response to tissue damage and infection. While the inflammatory response is typically transient, failure to resolve it can result in chronic inflammation. Chronic inflammation is a major contributing factor in the development and progression of various diseases, including metabolic disorders, cardiovascular diseases, neurodegenerative diseases, and cancer [1]. Under chronic inflammatory conditions, macrophages secrete high levels of pro-inflammatory cytokines, including tumor necrosis factor-α (TNF-α), interleukin (IL)-1β, and IL-6. These cytokines further amplify inflammation by activating inducible nitric oxide synthase (iNOS), which generates nitric oxide (NO), a reactive signaling molecule involved in vasodilation, tissue injury, pain modulation, and activation of downstream inflammatory pathways. Concurrently, prostaglandin production during inflammation is mediated by cyclooxygenase-2 (COX-2). The expression of these cytokines and inflammatory enzymes is regulated by intracellular signaling pathways, such as nuclear factor-kappa B (NF-κB) and mitogen-activated protein kinase (MAPK) [2,3,4]. Persistent inflammation is also linked to oxidative stress and free radical-mediated cellular damage, leading to DNA damage and genomic instability that can promote carcinogenesis. Notably, chronic inflammation creates a microenvironment that supports tumor initiation, promotion, and progression, illustrating the close link between inflammatory dysregulation and cancer development [5]. Given these connections, natural plant extracts and bioactive phytochemicals that modulate inflammatory responses and exhibit anticancer effects represent promising alternative strategies for preventing or treating inflammation-related cancer.

Bitter melon (*Momordica charantia* L.), locally known in Thailand as “Mara Khee Nok”, is a plant widely distributed in tropical and subtropical regions, including Thailand. It belongs to the Cucurbitaceae family and is commonly incorporated into the daily diet. In traditional medicine, several parts of this plant have been used to treat conditions such as diabetes mellitus, obesity, bacterial and viral infection, toothache, diarrhea, fever, and inflammation symptoms [6,7]. Previous studies have reported that bitter melon contains a diversity of phytochemical constituents, including cucurbitane-type triterpenoids, cucurbitane-type triterpene glycosides, saponins, flavonoids, and phenolic compounds, with leaves and vines especially rich in cucurbitane-type triterpenoids [8,9]. Recent research has demonstrated multiple biological activities of bitter melon extracts and isolated compounds, such as antidiabetic [10,11], antioxidant [12], anticancer [13,14], and anti-inflammatory activities [9,15,16]. Owing to these properties, bitter melon has gained increasing scientific interest as a promising natural source for the development of functional foods, nutraceuticals, and therapeutic agents. However, the biological efficacy of bitter melon extracts is significantly affected by the extraction process, which determines both the quantity and quality of phytochemical profiles [17].

In the development of functional foods and nutraceuticals from plant materials, the extraction process is recognized as a critical step [18]. Conventional extraction methods, such as maceration and Soxhlet extraction, have long been used to isolate bioactive compounds. However, these methods are limited by low efficiency, long processing times, high organic solvent consumption, and the risk of degrading heat-sensitive compounds due to high temperature [17]. Therefore, the development of improved extraction techniques that maximize phytochemical preservation while maintaining or enhancing biological efficacy remains an important challenge.

High-pressure processing (HPP) has emerged as a promising non-thermal extraction technology capable of overcoming the limitations of conventional extraction methods. HPP disrupts plant cell walls through high hydrostatic pressure, thereby enhancing the release of intracellular phytochemicals without the need for high temperatures. This process is also considered environmentally sustainable and has been shown to reduce the use of organic solvents [19,20]. Previous studies have demonstrated that HPP can enhance extraction efficiency, increase phenolic and flavonoid contents, and improve the biological activities of various plant materials [21,22]. Although HPP offers several advantages, optimization of HPP conditions, including pressure, extraction time, and solvent concentration and ratio, is essential, as these factors can significantly affect both the yield and bioactivity of the extracted compounds.

To overcome these challenges, statistical modeling approaches, such as response surface methodology (RSM), are widely employed to determine optimal HPP conditions. RSM enables the evaluation of relationships among multiple extraction factors and response variables [23]. The Box–Behnken Design (BBD), a widely used RSM approach, facilitates efficient optimization by exploring interactions among extraction factors while requiring fewer experimental runs than full factorial designs [24]. These statistical approaches are frequently utilized in the food and pharmaceutical industries to improve extraction efficiency while minimizing resource consumption, processing time, and operational costs. Furthermore, these methods have been successfully applied to optimize the extraction of bioactive compounds from several plant materials, such as Thai fermented soybean, *Porphyra* sp., and *Diplazium esculentum* (Retz.) [18,19,25]. In addition, BBD–RSM has been applied to optimize ultrasound-assisted extraction of bitter melon fruits, resulting in enhanced antioxidant activity and increased phenolic compounds and charantin content, a triterpenoid bioactive compound in bitter melon [26]. Despite increasing interest in bitter melon leaves, few studies have integrated HPP with BBD–RSM optimization to enhance both antioxidant and anti-inflammatory activities, followed by a comprehensive evaluation of anticancer properties across multiple cancer cell lines. Additionally, there is limited information on whether optimized extracts exhibit improved selectivity toward cancer cells over normal cells or exert stronger effects on inflammatory signaling at the molecular level.

In the present study, HPP extraction of bitter melon leaves was optimized using BBD combined with RSM, employing anticancer activity in A549 lung cancer cells and inhibition of NO production in lipopolysaccharide (LPS)-stimulated RAW264.7 macrophages as biological responses. The optimized bitter melon leaf extract (O-BMLE) was then compared with conventional (non-optimized) bitter melon leaf extract (BMLE) in terms of phytochemical profiles (total phenolic content (TPC), total flavonoid content (TFC), and HPLC-qTOF-MS-based metabolomic profiling), antioxidant capacity (ferric reducing antioxidant power (FRAP) and oxygen radical absorbance capacity (ORAC) assay), anti-inflammatory effects (modulation of cytokine expression, c-Jun N-terminal kinase (JNK) and NF-κB pathways, iNOS, and COX-2), and selective anticancer activity across A549, HepG2, SKOV3, LNCaP, and 3T3-L1 cells. This integrated approach aimed to elucidate how HPP–BBD/RSM-driven process optimization reshapes the bitter melon leaf metabolome and enhances anticancer and anti-inflammatory activities, thereby supporting the development of functional foods and nutraceuticals targeting inflammation and cancers.

## 2. Results

### 2.1. Cytotoxicity and Anti-Inflammatory Activity of BMLE

**Figure 1:**
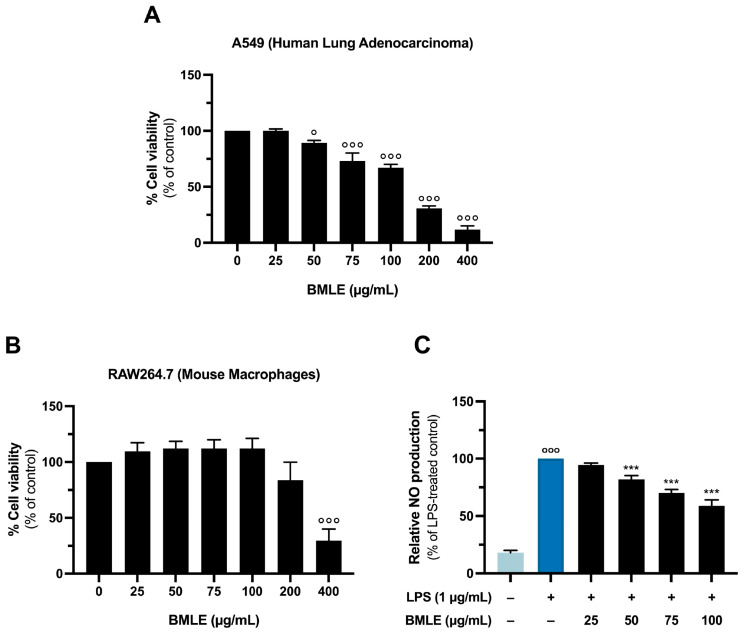
BMLE showed dose-dependent cytotoxicity against A549 lung adenocarcinoma cells and potent inhibition of NO production in LPS-stimulated RAW264.7 macrophages. (**A**) A549 cells and (**B**) RAW264.7 macrophages were treated with BMLE (0–400 µg/mL) for 48 h. Cell viability was determined using the SRB assay. (**C**) RAW264.7 macrophages were pre-treated with non-toxic concentrations of BMLE for 1 h, followed by LPS stimulation (1 µg/mL) for 24 h. NO levels in the supernatant were measured by Griess reagent. Data represent mean ± SD (*n* = 3 independent experiments). ° *p* < 0.05 and °°° *p* < 0.001 compared to non-treated control (vehicle control). *** *p* < 0.001 compared to LPS-treated control.

The cytotoxicity of BMLE was initially evaluated in A549 human lung adenocarcinoma cells and RAW264.7 mouse macrophages using the sulforhodamine B (SRB) assay. Subsequently, its inhibitory effect on NO production was assessed in LPS-stimulated RAW264.7 macrophages. As shown in Figure 1A, BMLE significantly reduced A549 cell viability in a dose-dependent manner, with a 50% inhibitory concentration (IC_50_) of 147 µg/mL after 48 h of treatment. Treatment with 25 µg/mL did not significantly affect cell viability compared to the control group, whereas stronger cytotoxic effects were observed at 200 and 400 µg/mL. In RAW264.7 cells, BMLE concentrations of 25 to 100 µg/mL did not exhibit significant cytotoxicity, with cell viability remaining above 100% after 48 h of treatment. However, higher concentrations of 200 and 400 µg/mL reduced cell viability, with a significant decrease observed at 400 µg/mL. The IC_50_ value for RAW264.7 cells was approximately 342 µg/mL (Figure 1B).

To evaluate the anti-inflammatory activity of BMLE, non-cytotoxic concentrations (≤100 µg/mL) were applied to RAW264.7 macrophages to assess NO production in LPS-stimulated cells. NO, a key pro-inflammatory mediator, regulates inflammatory responses and serves as a biomarker for preliminary screening of anti-inflammatory compounds. As shown in Figure 1C, LPS stimulation significantly increased NO production compared to the untreated control group. BMLE treatment significantly suppressed LPS-induced NO production in a dose-dependent manner. At concentrations of 50, 75, and 100 µg/mL, BMLE reduced NO levels to approximately 80%, 70%, and 60%, respectively, relative to the LPS-treated group (Figure 1C). These results indicate that BMLE exhibits cytotoxicity against A549 lung cancer cells and anti-inflammatory activity by inhibiting NO production in LPS-stimulated macrophages.

### 2.2. Optimization of BMLE by HPP Using BBD and RSM

BMLE has demonstrated a cytotoxic effect against A549 lung cancer cells and anti-inflammatory activity by inhibiting NO production in LPS-stimulated RAW264.7 macrophages. However, conventional extraction methods still present several limitations. In this study, HPP, in combination with BBD and RSM, was employed to optimize BMLE extraction and further enhance its anticancer and anti-inflammatory activities.

The BBD was initially employed to evaluate the effects of four independent variables: extraction pressure, extraction time, ethanol concentration (%EtOH), and liquid-to-solid (L:S) ratio, on the anticancer activity of the extracts against A549 lung cancer cells. Twenty-nine experimental runs were generated using the BBD model. The percentage inhibition of A549 cell growth ranged from 12.31 ± 1.21% to 73.39 ± 1.78 (Supplementary Table S1). Regression analysis and analysis of variance (ANOVA) indicated that %EtOH was the only statistically significant factor (*p* = 0.0006), contributing most to the model and exerting a strong positive effect on the response, whereas its quadratic term was not significant (Supplementary Table S2), suggesting a predominantly linear positive effect within the investigated range. Consistently, response surface and contour plots showed a progressive increase in % inhibition with increasing %EtOH toward the upper end of the design space (Supplementary Figure S1). Given the dominant influence of %EtOH and its monotonic effect within the tested range, this factor was fixed at 90% for subsequent optimization, as this level corresponded to conditions associated with maximal predicted and observed anticancer activity in the initial design.

In the initial experimental design, the pressure range was set at 300–500 MPa, which corresponds to the mid-range of operating conditions commonly used in HPP and extraction. This range was selected based on preliminary trials that demonstrated robust instrument performance and satisfactory extraction while maintaining reasonable energy-reasonable window [27]. Within this interval, both pressure and extraction time showed only moderate, though less pronounced, effects on A549 inhibition compared to %EtOH. These effects were evident in the regression coefficients but contributed less statistically to the model (Supplementary Table S2). To more thoroughly characterize the pressure–response relationship and to test whether comparable activity might be achieved at lower pressures, potentially reducing energy demand at an industrial scale, as well as to assess whether higher pressures could further enhance extraction, the pressure domain in the subsequent three-factor BBD was broadened to 100–600 MPa [19,22,28]. Therefore, in this second design, seventeen experimental runs were generated using the BBD model, pressure (A), extraction time (B), and L:S ratio (C) were independent variables to further refine the extraction conditions.

**Figure 2:**
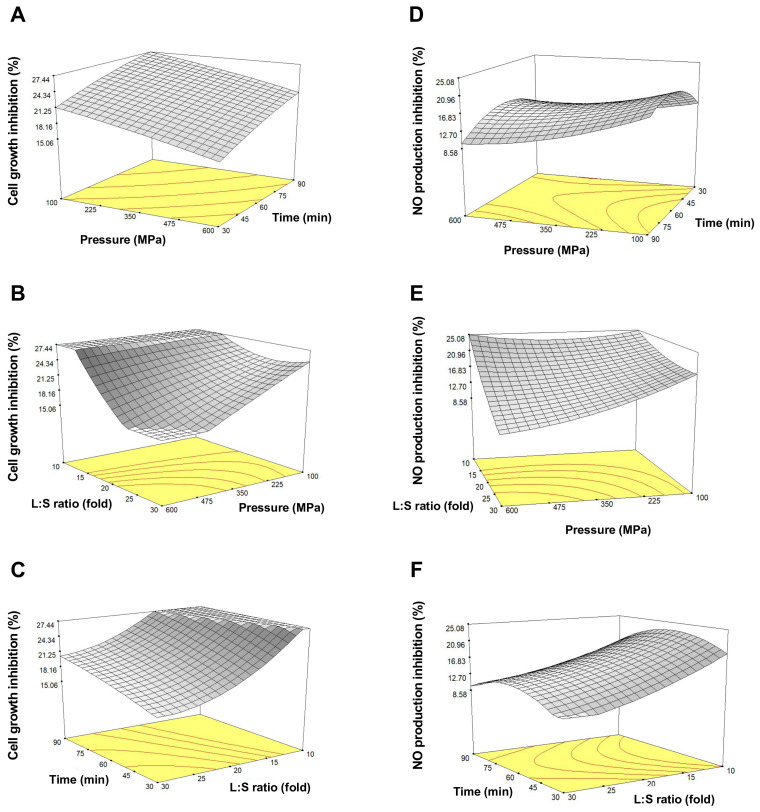
Response Surface and Contour Plots Illustrate How HPP Parameters Interactively Influence Biological Activities of BMLE During BBD Optimization. (**A**–**C**) Effects on A549 cell growth inhibition (%) (**D**–**F**). Effects on NO production inhibition (%) in LPS-stimulated RAW264.7 macrophages. Independent variables as indicated in Table 2 and Table 3: pressure (A; 100–600 MPa), extraction time (B; 30–90 min), and L:S ratio (C; 10–30-fold).

The responses measured were % inhibition of A549 cell growth (Response 1) and % inhibition of NO production in LPS-stimulated RAW264.7 macrophages (Response 2), as detailed in Table 1. Results showed that % inhibition of A549 cell growth ranged from 11.50 ± 2.12% to 39.00 ± 5.20%, while % inhibition of NO production ranged from 11.35 ± 5.28% to 26.62 ± 5.41%, depending on extraction conditions. These findings indicate that extraction parameters influence the biological activities of BMLE. The statistical significance of the model and its regression coefficients were determined by ANOVA (Table 2 and Table 3). For % inhibition of A549 cell growth, the model was statistically significant (*p* = 0.0307), suggesting that the selected variables accounted for the variation in the response. The L:S ratio significantly affected anticancer activity (*p* = 0.0016), while pressure and extraction time had moderate but non-significant effects. Similarly, the model for % inhibition of NO production was significant (*p* = 0.0392). The L:S ratio significantly influenced NO inhibition (*p* = 0.0034), and extraction time (B^2^) also had a significant effect (*p* = 0.0404). The lack-of-fit test for both models was not significant, confirming model adequacy. Contour plots illustrate the interactions between extraction factors on responses (Figure 2). For anticancer activity (Figure 2A–C), increasing the L:S ratio decreased cancer cell growth inhibition. For anti-inflammatory activity (Figure 2D–F), NO production inhibition was significantly influenced by extraction time and L:S ratio, indicating that optimal extraction conditions are required for maximum anti-inflammatory activity.

**Figure 3:**
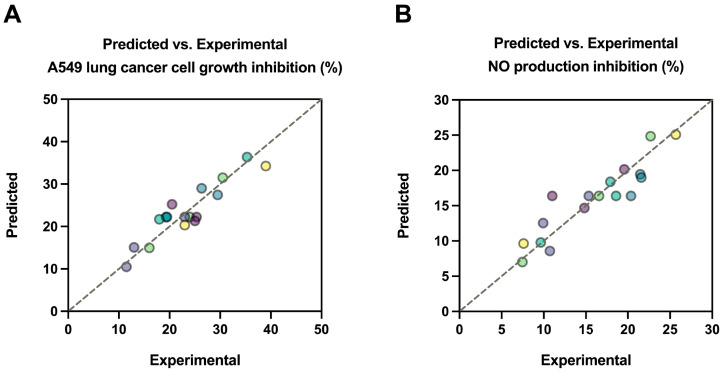
BBD–RSM Model Predictions Accurately Match Experimental Values, Validating the Optimization Process. (**A**) Predicted vs. experimental A549 lung cancer cell growth inhibition (%). (**B**) Predicted vs. experimental NO production inhibition (%) in LPS-stimulated RAW264.7 macrophages. Data points represent 17 experimental runs from BBD (*n* = 3). Solid line indicates perfect prediction (y = x); dashed lines show 95% confidence interval. Model fit: R^2^ ≈ 0.80 for both responses.

Plots comparing model-predicted and experimental values for both responses are shown in Figure 3, indicating a strong correlation with R^2^ values of approximately 0.8. The ANOVA analysis for the optimization equations is presented as Equations (1) and (2), which can guide the selection of optimal extraction parameters. These results demonstrate the effectiveness of BBD and RSM for process optimization, and the optimized conditions were further used in subsequent experiments.

Equations for variable optimization:

A549 cell growth inhibition (%) = 32.29633 + 0.02564A + 0.20486B − 1.42625C − 5.2 × 10^−6^A2 − 5.92593 × 10^−4^B2 + 0.034667C2 + 1.66667 × 10^−5^AB − 1.75 × 10^−3^AC − 1.66667 × 10^−3^BC (1)

NO production inhibition (%) = 13.14361 + 5.95924 × 10^−3^A + 0.59681B − 0.88111C + 2.92391 × 10^−5^A2 − 4.6875 × 10^−3^B2 + 0.014538C2 − 2.35507 × 10^−4^AB − 1.18207 × 10^−3^AC + 3.05707 × 10^−3^BC (2)

where A, B, and C are the independent variables, including pressure (MPa), extraction time (min), and L:S (fold), respectively. Based on these equations, the optimized HPP conditions were established as 600 MPa pressure, 62 min extraction time, and a L:S ratio of 10. Under these parameters, bitter melon leaves were re-extracted using HPP and used in subsequent experiments to evaluate whether this statistical optimization translated into enhanced functional properties. The O-BMLE was then compared with conventional BMLE obtained by 80% ethanol maceration to assess improvements in phytochemical content as well as anticancer and anti-inflammatory activities as detailed in the following sections.

### 2.3. Phytochemical Composition and Antioxidant Activity of O-BMLE and BMLE

To determine the impact of optimized extraction conditions on the phytochemical compositions of the BMLE, TPC, TFC, and antioxidant activity were compared between O-BMLE and BMLE. The results are summarized in Table 4. The TPC of O-BMLE was slightly lower than that of BMLE, but this difference was not statistically significant (15.99 ± 1.53 mg of GAE/g extract and 19.64 ± 4.90 mg of GAE/g extract, respectively). In contrast, TFC was significantly higher in O-BMLE compared to BMLE, with values of 27.70 ± 2.40 mg of RE/g extract and 8.71 ± 1.92 mg of RE/g extract, respectively. These results suggest that the optimization process did not significantly affect phenolic content but did significantly increase the flavonoid content in BMLE.

The antioxidant activities of O-BMLE and BMLE were further assessed using FRAP and ORAC assays. In the FRAP assay, O-BMLE exhibited significantly greater antioxidant capacity (96.53 ± 12.96 µmol TE/g extract) compared to BMLE (71.22 ± 11.06 µmol TE/g extract). Similarly, the ORAC assay showed that O-BMLE had a higher antioxidant value (330.40 ± 31.04 µmol TE/g extract) than BMLE (298.95 ± 20.90 µmol TE/g extract), although this difference was not statistically significant. Overall, these results demonstrate that optimized extraction conditions significantly increased both flavonoid content and antioxidant capacity in BMLE, while maintaining phenolic compound levels. The increased flavonoid content may contribute to the enhanced biological activities observed in the optimized extract.

### 2.4. Cytotoxic Effects and Selectivity of O-BMLE and BMLE in Cancer Cells and Non-Cancerous Cells

**Figure 4:**
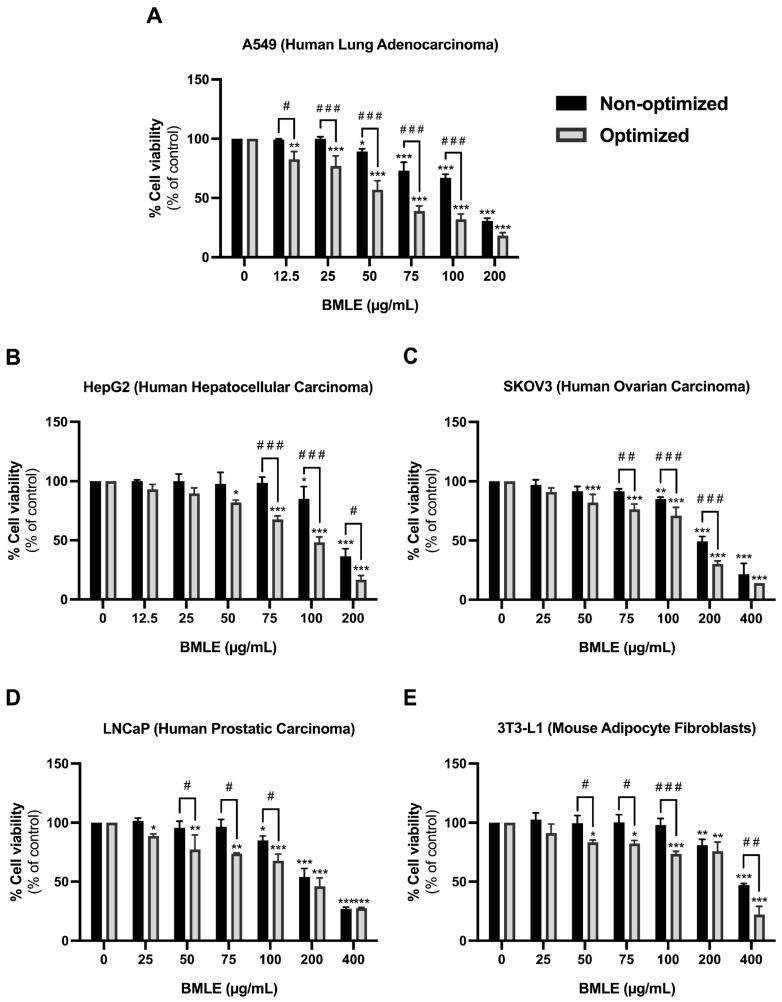
O-BMLE exhibits significantly greater cytotoxicity and improved selectivity against cancer cells compared to BMLE. Cells were treated with O-BMLE or BMLE (0–400 μg/mL; 48 h) followed by SRB assay. (**A**) A549 lung cancer cells, (**B**) HepG2 liver cancer cells, (**C**) SKOV3 ovarian cancer cells, (**D**) LNCaP prostate cancer cells, and (**E**) 3T3-L1 mouse fibroblasts. O-BMLE achieved A549 IC_50_ 58.7 μg/mL (SI 5.03) vs. BMLE 147 μg/mL (SI 2.60). Data represent mean ± SD (*n* = 3). * *p* < 0.05, ** *p* < 0.01, and *** *p* < 0.001 vs. untreated control; # *p* < 0.05, ## *p* < 0.01, and ### *p* < 0.001 O-BMLE vs. BMLE corresponding concentrations.

Following the identification of the optimal extraction parameters in Section 2.2, we needed to validate whether the statistical optimization translated into enhanced biological efficacy. While the initial design targeted A549 lung cancer cells, we further extended the investigation to evaluate the cytotoxic effects of O-BMLE compared to BMLE across a broader panel of human cancer cell lines, including HepG2 (human hepatocellular carcinoma), SKOV3 (human ovarian carcinoma), and LNCaP (human prostatic carcinoma) cells. To assess whether the optimization process also improved the safety profile of the extract, the selectivity of O-BMLE was further examined using non-cancerous 3T3-L1 fibroblast cells as a non-transformed reference cell line. As shown in Figure 4, both O-BMLE and BMLE reduced cell viability in a dose-dependent manner across all tested cancer cell lines. The IC_50_ values for each extract and cell line are presented in Table 2. O-BMLE exhibited significantly stronger cytotoxic effects than BMLE, as indicated by lower IC_50_ values. These results suggest that optimizing the extraction process enhanced anticancer activity across several cancer cell models. However, this enhancement was not observed in LNCaP cells, where IC_50_ values did not differ significantly between extracts. Cytotoxicity in 3T3-L1 fibroblast cells was also evaluated. BMLE was cytotoxic at higher concentrations (200 and 400 µg/mL), while O-BMLE showed slight cytotoxicity starting at 50 µg/mL (Figure 4E). The IC_50_ values for O-BMLE and BMLE in 3T3-L1 cells were 295.14 ± 27.82 µg/mL and 381.40 ± 11.41 µg/mL, respectively, suggesting lower cytotoxicity toward non-cancerous cells compared with cancer cells. The selectivity index (SI) was calculated using 3T3-L1 cells as the normal reference. O-BMLE demonstrated improved selectivity toward certain cancer cells compared with BMLE as shown in Table 5. Specifically, the SI value of O-BMLE in A549 cells was 5.03, approximately twice that of BMLE (2.60). Similarly, SI values for HepG2 and SKOV3 cells were higher for O-BMLE. No significant difference in SI values was observed between extracts in LNCaP. Overall, these results indicate that the optimized extraction process enhanced both cytotoxic activity and selective toxicity toward cancer cells, particularly in A549 cells. This improvement in selectivity may be associated with changes in phytochemical composition, especially the increased flavonoid content observed in O-BMLE.

### 2.5. Inhibitory Effect of O-BMLE on NO Production in LPS-Stimulated RAW264.7 Macrophages

**Figure 5:**
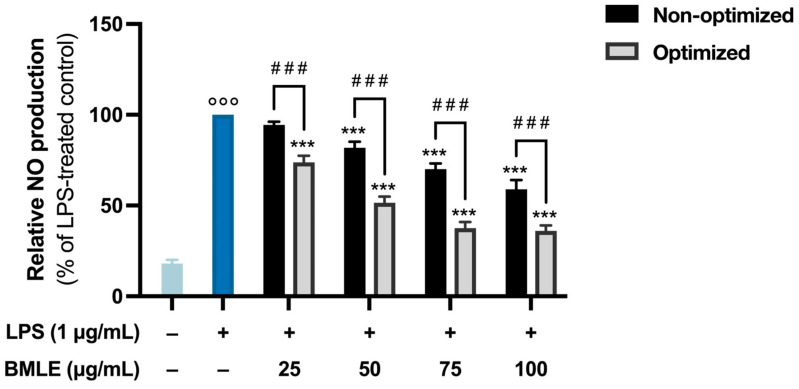
O-BMLE demonstrates significantly greater inhibition of NO production compared to BMLE in LPS-stimulated RAW264.7 macrophages. Cells were pretreated with non-cytotoxic concentrations of O-BMLE or BMLE (25–100 μg/mL; 1 h), followed by LPS stimulation (1 μg/mL; 24 h). NO levels in supernatants were measured by Griess reagent assay. Data represent mean ± SD (*n* = 3 independent experiments). °°° *p* < 0.001 vs. untreated control (without extract); *** *p* < 0.001 vs. LPS-treated control; ### *p* < 0.001 O-BMLE vs. BMLE.

In addition to enhancing anticancer activity, the second objective of the HPP optimization was to maximize the anti-inflammatory potential of the extract, as modeled by the inhibition of NO production (Response 2) in Section 2.2. This section presents a detailed comparative analysis of the anti-inflammatory effects between O-BMLE and BMLE in LPS-stimulated RAW264.7 macrophages. Evaluating these extracts across a concentration gradient was conducted to determine whether the optimized extraction conditions, which significantly increased flavonoid levels, consistently resulted in greater suppression of inflammatory mediators. As shown in Figure 5, pre-treatment with O-BMLE and BMLE significantly suppressed NO production in a dose-dependent manner compared to the LPS-treated group. At 25 µg/mL, O-BMLE decreased NO production to approximately 70%, whereas BMLE showed no significant difference from the LPS-treated group. At 50, 75, and 100 µg/mL, O-BMLE decreased NO levels to approximately 50%, 37%, and 35%, respectively, whereas BMLE reduced NO production to approximately 80%, 70%, and 55% at the same concentrations. O-BMLE consistently exhibited a significantly stronger inhibitory effect on NO production than BMLE at all concentrations (### *p* < 0.001) (Figure 5). These results suggest that the optimization process significantly enhanced the anti-inflammatory activity of BMLE by improving its ability to suppress NO levels in activated macrophages.

### 2.6. Effect of O-BMLE on Pro-Inflammatory Cytokine Gene Expressions

Pro-inflammatory cytokines, including IL-1β, IL-6, and TNF-α, are upregulated and secreted during chronic inflammation and play important roles in promoting cancer development and progression. Thus, further investigation of the anti-inflammatory activity of O-BMLE at the molecular level was necessary to confirm its biological relevance. In addition, excessive NO levels have been associated with upregulation of IL-1β, IL-6, and TNF-α. Accordingly, these cytokines were selected as representative markers to evaluate the anti-inflammatory activity of O-BMLE.

**Figure 6:**
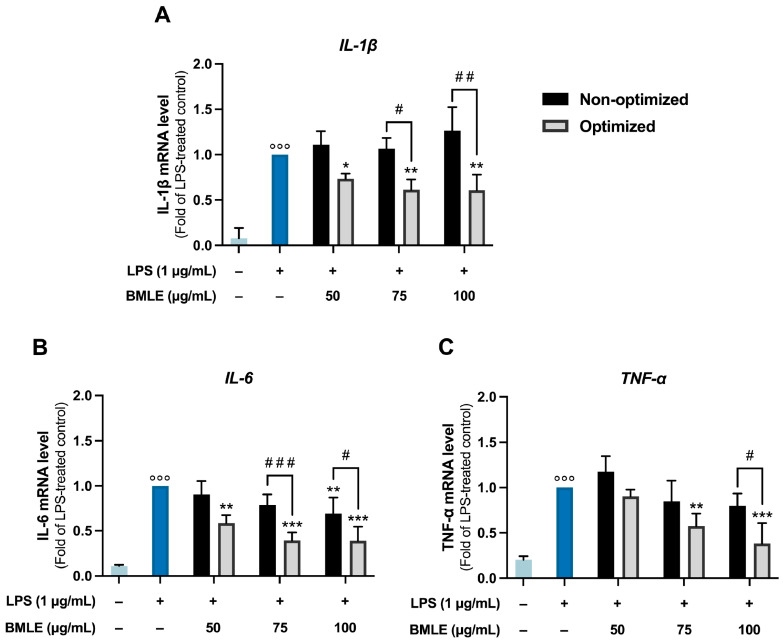
O-BMLE exhibits significantly greater suppression of pro-inflammatory cytokine mRNA expression compared to conventional BMLE in LPS-stimulated RAW264.7 macrophages. Effects on (**A**) IL-1β, (**B**) IL-6, and (**C**) TNF-α. Cells were pretreated with non-cytotoxic concentrations of O-BMLE or BMLE (50–100 μg/mL; 1 h), followed by LPS stimulation (1 μg/mL; 24 h). mRNA levels were quantified by RT-qPCR (2^−ΔΔCT^ method, GAPDH-normalized). Data represent mean ± SD (*n* = 3). °°° *p* < 0.001 vs. untreated control (without extract); * *p* < 0.05, ** *p* < 0.01, and *** *p* < 0.001 vs. LPS-treated control; # *p* < 0.05, ## *p* < 0.01, and ### *p* < 0.001 O-BMLE vs. BMLE.

The effects of O-BMLE and BMLE on the mRNA expression levels of IL-1β, IL-6, and TNF-α were examined using RT-qPCR as shown in Figure 6. LPS stimulation markedly increased mRNA expression of these cytokines compared to the untreated control group. Pre-treatment with O-BMLE significantly suppressed IL-1β and IL-6 mRNA expression at all tested concentrations (50, 75, and 100 µg/mL). O-BMLE also significantly suppressed TNF-α mRNA expression at 75 and 100 µg/mL. However, BMLE did not significantly affect mRNA expression of IL-1β, IL-6, and TNF-α, except for a significant reduction in IL-6 expression at 100 µg/mL. At higher concentrations, O-BMLE exhibited a significantly stronger inhibitory effect on mRNA expression of three cytokines compared to BMLE (Figure 6). Taken together, these results indicate that O-BMLE suppresses production of key pro-inflammatory cytokines more effectively than BMLE.

### 2.7. Effect of O-BMLE on Pro-Inflammatory Cytokine Production

**Figure 7:**
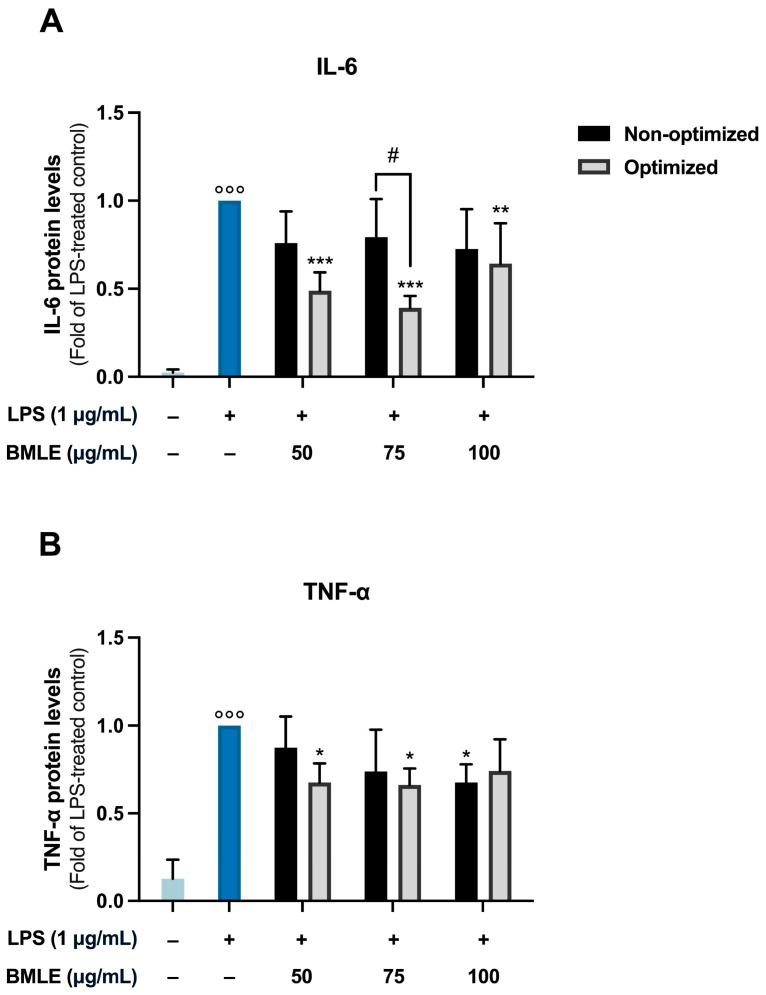
O-BMLE demonstrates significantly greater suppression of IL-6 and TNF-α protein secretion compared to BMLE in LPS-stimulated RAW264.7 macrophages. Effects on (**A**) IL-6 and (**B**) TNF-α production. Cells were pretreated with O-BMLE or BMLE (50–100 μg/mL; 1 h), followed by LPS stimulation (1 μg/mL; 24 h). Cytokine levels in supernatants were quantified by ELISA. Data represent mean ± SD (*n* = 3). °°° *p* < 0.001 vs. untreated control (without extract); * *p* < 0.05, ** *p* < 0.01, and *** *p* < 0.001 vs. LPS-treated control; # *p* < 0.05 O-BMLE vs. BMLE.

To determine whether inhibitory effects of O-BMLE at the transcriptional level corresponded to the protein expression, the secretion of pro-inflammatory cytokines IL-6 and TNF-α was measured using ELISA as shown in Figure 7. LPS stimulation markedly increased protein levels of IL-6 and TNF-α compared to the untreated control group, consistent with RT-qPCR results. Pre-treatment with O-BMLE significantly reduced IL-6 protein levels at all tested concentrations, with the strongest effect at 75 µg/mL. BMLE did not significantly inhibit IL-6 production compared to the LPS-treated group at most concentrations. O-BMLE demonstrated a significantly greater inhibitory effect on IL-6 production than BMLE, particularly at 75 µg/mL (Figure 7A). O-BMLE also slightly but significantly reduced TNF-α production at 50 and 75 µg/mL, but not at 100 µg/mL. BMLE produced a slight reduction in TNF-α levels; however, no significant difference between O-BMLE and BMLE was observed at any concentrations (Figure 7B). Overall, these results indicate that O-BMLE effectively suppresses the production of key pro-inflammatory cytokines at the protein level, particularly IL-6, consistent with its effects at the mRNA level.

### 2.8. Effect of O-BMLE on the Activation and Expression of Inflammatory Proteins

The molecular mechanisms underlying the anti-inflammatory effects of O-BMLE and BMLE were subsequently investigated by examining key intracellular signaling pathways using Western blot analysis. The NF-κB and JNK pathways primarily regulate pro-inflammatory mediators, including iNOS, COX-2, IL-6, TNF-α, and IL-1β. Suppression of these signaling cascades is therefore considered a critical mechanism for anti-inflammatory activity.

**Figure 8:**
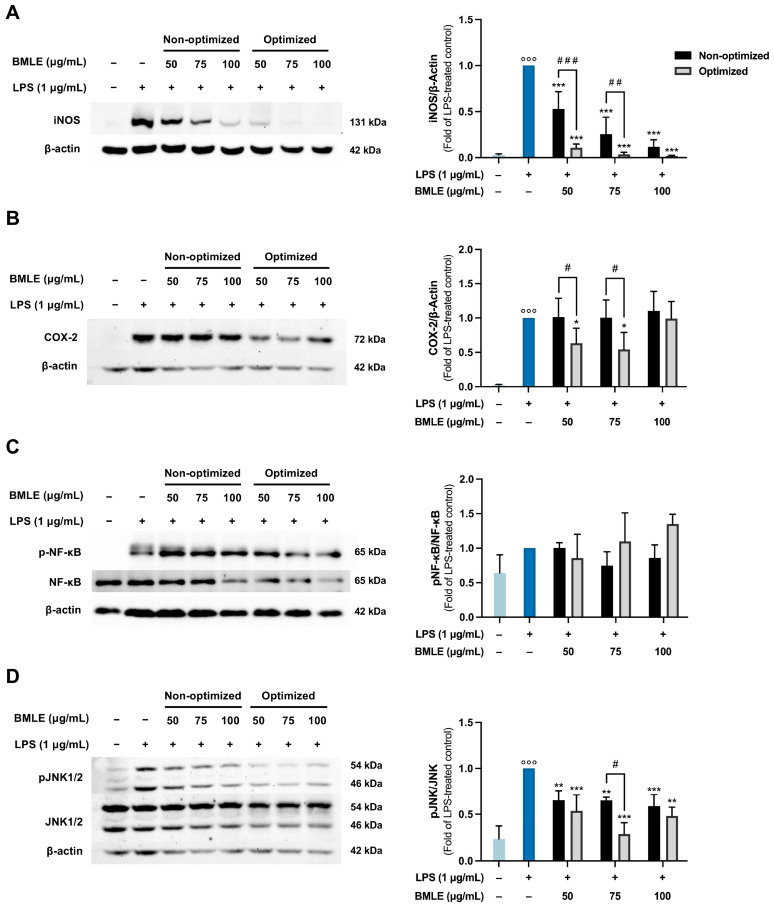
O-BMLE selectively suppresses iNOS, COX-2, and JNK phosphorylation while sparing NF-κB activation in LPS-stimulated RAW264.7 macrophages. Representative Western immunoblot analysis and densitometric quantification of (**A**) iNOS, (**B**) COX-2, (**C**) phosphorylated NF-κB p65 (p-p65)/total p65 ratio, and (**D**) phosphorylated JNK (p-JNK)/total JNK ratio. Cells were pretreated with O-BMLE or BMLE (50–100 μg/mL; 1 h), followed by LPS stimulation (1 μg/mL; 24 h). β-actin served as loading control; band intensities normalized to β-actin. Data represent mean ± SD (*n* = 3). °°° *p* < 0.001 vs. untreated control (without extract); * *p* < 0.05, ** *p* < 0.01, and *** *p* < 0.001 vs. LPS-treated control; # *p* < 0.05, ## *p* < 0.01, and ### *p* < 0.001 O-BMLE vs. BMLE.

As shown in Figure 8, LPS stimulation significantly increased protein expression levels of iNOS and COX-2 compared to the untreated control. Treatment with both O-BMLE and BMLE significantly reduced iNOS expression in a dose-dependent manner relative to the LPS-treated group. O-BMLE exhibited a stronger inhibitory effect than BMLE, particularly at 50 and 75 µg/mL. At 100 µg/mL, both extracts markedly reduced iNOS expression, with no significant difference between them (Figure 8A). Regarding COX-2 expression, O-BMLE showed a slightly yet significantly stronger inhibitory effect, especially at lower concentrations (50 and 75 µg/mL), whereas BMLE did not inhibit COX-2 expression. At the highest concentration, O-BMLE upregulated COX-2 expression (Figure 8B). To investigate upstream signaling, phosphorylation levels of NF-κB and JNK were measured by Western blotting. LPS induced NF-κB phosphorylation, but treatment with either extract at all tested concentrations did not suppress pNF-κB levels (Figure 8C). These findings suggest that the observed anti-inflammatory effects are unlikely to be mediated primarily or directly through the inhibition of NF-κB phosphorylation. In contrast, JNK signaling was significantly affected. LPS-induced phosphorylation of JNK was reduced by both BMLE and O-BMLE in a dose-dependent manner (Figure 8D). O-BMLE demonstrated a stronger inhibitory effect on pJNK than BMLE, particularly at 75 µg/mL, suggesting that suppression of the JNK pathway is a major mechanism underlying the observed anti-inflammatory activity. These findings are consistent with RT-qPCR and ELISA results, which showed reductions in IL-6, TNF-α, and IL-1β production. Since JNK signaling regulates transcription of these cytokines as well as iNOS and COX-2, its inhibition likely contributes to the overall suppression of inflammatory responses.

### 2.9. Comparison of Momordicine-I Content Between O-BMLE and BMLE

**Figure 9:**
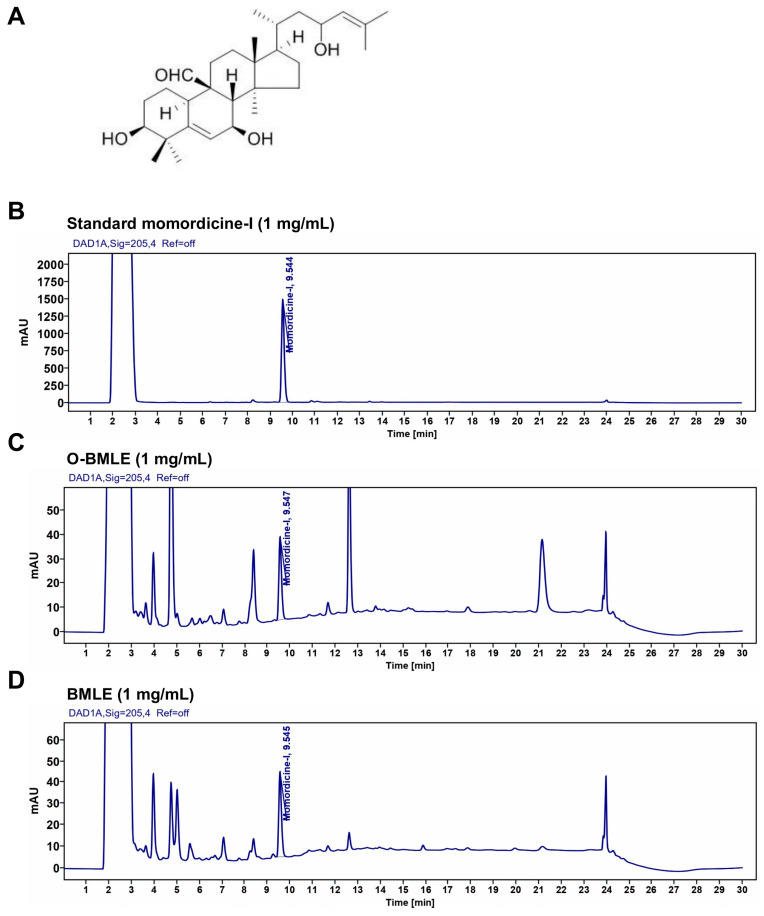
HPLC analysis confirms equivalent MM-I content between O-BMLE and BMLE (~28 mg/g extract). (**A**) Chemical structure of MM-I. Representative HPLC chromatograms of (**B**) MM-I standard, (**C**) O-BMLE, and (**D**) BMLE. Extracts and standard were prepared at 1 mg/mL in HPLC-grade methanol, filtered (0.22 μm), and analyzed using Agilent 1260 Infinity II system with ZORBAX Eclipse Plus C18 column (Agilent Technologies, Santa Clara, CA, USA) (UV 205 nm). Quantification based on external standard curve (mean ± SD, *n* = 3).

Cucurbitane-type triterpenoids are the major bioactive compounds in bitter melon leaves, and momordicine-I (MM-I) is considered a key bioactive compound due to its reported anti-inflammatory activity. We hypothesized that the enhanced anti-inflammatory activity of O-BMLE is due to increased MM-I content. To test this, MM-I content in O-BMLE was determined and quantified by HPLC analysis. As shown in Figure 9, MM-I was identified in both extracts by its retention time of approximately 9.54 min relative to the standard. Quantitative analysis revealed that O-BMLE contained 27.14 ± 2.38 mg/g extract of MM-I, while BMLE contained 28.93 ± 5.91 mg/g extract. No significant difference in MM-I content was observed between the two extracts (Table 6). These results suggest that the enhanced anti-inflammatory activity of O-BMLE is unlikely to be due solely to increased MM-I concentration. Instead, the improvement may result from changes in other bioactive compounds or synergistic interactions among phytochemicals enriched during the optimization process.

### 2.10. Phytometabolomic Profiling of O-BMLE and BMLE

Since the concentration of MM-I did not differ significantly between O-BMLE and BMLE, phytometabolomic profiling of both extracts was performed using HPLC-qTOF-MS/MS to identify additional bioactive compounds potentially responsible for the enhanced anti-inflammatory activity. The analysis revealed distinct metabolite profiles for O-BMLE and BMLE. O-BMLE tentatively contained 680 compounds in positive ionization mode and 590 in negative ionization mode, while BMLE contained 613 and 550 compounds, respectively (Supplementary Data). Metabolites in each extract were tentatively identified under both ionization modes based on a library match score of 90% or higher. O-BMLE included 48 and 42 tentatively identified metabolites with a library score of 90% or higher in positive and negative ionization modes, respectively, (Supplementary Tables S3 and S4) while BMLE included 37 and 38 compounds, respectively (Supplementary Tables S5 and S6). In positive mode, both extracts contained primary metabolites, including amino acids (phenylalanine, isoleucine, and L-tryptophan), sugar, and lipid-related compounds, particularly fatty acids and glycerol derivatives such as stearidonic acid and monolinolenin. O-BMLE exhibited higher levels of stearidonic acid (27.83%) and monolinolenin (6.17%) compared to BMLE (16.74% and 2.98%, respectively). Several bioactive secondary metabolites were detected exclusively or at higher concentrations in O-BMLE, including erucamide (14.56%), 6-gingerol (5.69%), and leiocarposide (1.03%), and increased levels of pinoresinol (1.22%). In negative mode, O-BMLE and BMLE also showed markedly different metabolite profiles. O-BMLE was enriched in several phenolic acids and phenolic compounds, including 4-hydroxybenzoic acid (19.51%), vanillic acid (2.18%), and esculin (2.19%). In contrast, BMLE showed relatively higher levels of oxidized fatty acids (oxylipins), such as 13S-hydroxy-9Z,11E,15Z-octadecatrienoic acid and 13-keto-octadecadienoic acid, which together accounted for more than half of the total relative abundance. Several triterpenoid- and saponin-related secondary metabolites, including pedunculoside, ginsenoside Rh1, chikusetsusaponin IVa, and asiatic acid, were also detected. O-BMLE contained higher levels of pedunculoside compared to BMLE, whereas BMLE exhibited greater diversity of these compounds.

## 3. Discussion

Conventional extraction of bitter melon leaves has demonstrated anticancer activity in various cancer cell types and anti-inflammatory properties [16,29,30]. However, these extraction methods are limited by prolonged extraction times, excessive solvent usage, and potential degradation of heat-sensitive bioactive compounds. To overcome these limitations, it is essential to employ extraction technologies that enhance phytochemical content and biological activity.

This study utilized BBD combined with RSM to optimize the HPP-assisted extraction conditions for bitter melon leaves. Optimal parameters, 600 MPa pressure, 62 min extraction time, and a L:S ratio of 10, were determined by maximizing inhibition of NO production and A549 cancer cell growth. The O-BMLE produced under these conditions showed significant improvements in phytochemical content, antioxidant capacity, and both anticancer and anti-inflammatory activities compared to BMLE. The enhancements possibly result from the disruption of plant cell walls by HPP, which facilitates the release of intracellular phytochemicals into the extraction solvent [20]. Previous studies have identified pressure as one of the most critical factors influencing extraction efficiency; increased pressure enhances solvent penetration and solubility of phytochemical constituents [31], consistent with the present findings that the highest pressure yielded the most effective extract. Furthermore, these results align with earlier research demonstrating that high hydrostatic pressure significantly increased the yield of bioactive compounds such as saponins from *Momordica charantia*. extracts [32]. Overall, this study demonstrates that high pressure, in combination with a lower L:S ratio, significantly influences the biological responses of O-BMLE.

The antioxidant capacities of O-BMLE and BMLE showed clear differences. O-BMLE exhibited a significantly higher FRAP value (35% increase), indicating improved electron transfer-based reducing capacity [33]. This enhancement is likely attributable to 3.2-fold increase in TFC, as flavonoids possess strong reducing activity and are highly responsive in electron transfer-based assays, such as FRAP [34]. In contrast, no significant difference was observed in the ORAC assay, which evaluates hydrogen atom transfer (HAT)-based peroxyl radical scavenging rather than electron transfer [33]. Previous research reported that ultrasound-assisted extraction of lemongrass (*Cymbopogon citratus*) yields higher ORAC values than conventional extraction methods, whereas conventional extraction yields higher FRAP values [35]. This supports the understanding that different antioxidant assays may not change in parallel. The selective improvement in FRAP observed in O-BMLE suggests that the optimization process enriched compounds, most likely flavonoids, that enhance reducing power rather than those primarily involved in hydrogen atom donation to neutralize free radicals. Furthermore, the TPC values did not differ significantly between extracts, indicating that overall phenolic compounds remained stable after optimization. This observation agrees with previous evidence that TPC does not always correlate directly with biological activities or antioxidant capacity [36]. The selective elevation in TFC may be explained by the L:S ratio used during extraction, as lower ratios have been shown to increase flavonoid yield [37]. Thus, optimizing this parameter likely enhanced flavonoid contents. The elevated TFC may also explain the improved anticancer and anti-inflammatory activities of O-BMLE. Taken together, these findings suggest that HPP-assisted optimization selectively enhanced flavonoid content, thereby improving the electron transfer-based reducing capacity of O-BMLE.

O-BMLE exhibits enhanced cytotoxicity against several cancer cell types, including A549 lung cancer cells, HepG2 liver cancer cells, SKOV3 ovarian cancer cells, and LNCaP prostate cancer cells, compared to BMLE. Importantly, O-BMLE showed high selectivity toward cancer cells, as both extracts were non-toxic to 3T3-L1 mouse fibroblasts at concentrations up to 200 μg/mL, suggesting a favorable safety profile. Nonetheless, interpretations of selectivity are limited by species differences and the lack of tissue matched normal cells, warranting validation in primary human cells. Further evaluation using primary human cells, or tissue-match normal cell lines, (e.g., human lung epithelial cells) is necessary to confirm the therapeutic potential suggested by these results. The enhanced anticancer effects of O-BMLE are likely due to increased flavonoid concentrations resulting from the optimization. Flavonoids are well-documented for their anticancer properties, including modulation of ROS-related pathways, disruption of cell cycle progression, induction of apoptosis and autophagy, and suppression of cancer cell invasiveness [38]. In addition, the higher cytotoxicity of O-BMLE may be linked to cucurbitane-type triterpenoids, major bioactive constituents of bitter melon that have demonstrated anticancer effects in various cancer models [9,11,13,14]. Despite a lower IC_50_ with O-BMLE, LNCaP cells did not exhibit a markedly improved selectivity index. This limited enhancement may be due to specific molecular characteristics of LNCaP cells, which are androgen-sensitive and rely predominantly on androgen receptor (AR) signaling rather than oxidative stress pathways for survival and proliferation [39]. If the bioactive compounds in O-BMLE do not strongly interfere with AR-regulated signaling, their selectivity toward LNCaP cells may be reduced. Furthermore, a previous study has shown that LNCaP cells tend to undergo cell cycle arrest rather than apoptosis after phytochemical exposure, resulting in moderate growth inhibition without strong cytotoxic selectivity [40], which may explain the limited improvement in selectivity observed in this study.

Our study highlights that O-BMLE exerts potent anti-inflammatory activity in LPS-stimulated RAW264.7 macrophages through selective inhibition of JNK/MAPK signaling. The results showed that O-BMLE markedly reduced phosphorylated JNK, with no significant effect on pNF-κB levels. Interestingly, despite its limited effect on NF-κB activation, O-BMLE significantly suppressed the expression and secretion of major pro-inflammatory cytokines and mediators, including IL-1β, IL-6, TNF-α, and NO. These findings are consistent with previous studies in LPS-stimulated RAW264.7 macrophages demonstrating that bitter melon reduces the expression of pro-inflammatory mediators (IL-1β, IL-6, TNF-α, COX-2, and iNOS) [15,16]. The anti-inflammatory effect of O-BMLE is likely driven primarily by inhibition of JNK activation. JNK functions as a key upstream regulator of c-Jun/AP-1, which is essential for the transcription of numerous inflammatory genes. The promoters of IL-1β, IL-6, TNF-α, and iNOS genes contain AP-1 binding elements [41,42]. Consequently, suppression of JNK phosphorylation is expected to reduce c-Jun activation and AP-1 DNA-binding activity, thereby downregulating these pro-inflammatory cytokines and mediators, even if NF-κB phosphorylation remains unchanged. This mechanism is consistent with earlier reports showing that JNK inhibition effectively reduces iNOS expression and NO synthesis in LPS-stimulated macrophages, independently of NF-κB signaling [43]. Although pNF-κB levels were unchanged, it is important to note that NF-κB activity is regulated at multiple levels, including nuclear translocation, chromatin accessibility, coactivator recruitment, and functional cooperation with AP-1.

JNK signaling is functionally interconnected with NF-κB pathways through multiple layers of regulatory crosstalk. Previous studies have demonstrated that inhibition of JNK phosphorylation can attenuate the transcriptional activity of both AP-1 and NF-κB, as these transcription factors often share common upstream signaling cascades and co-regulatory mechanisms [44]. JNK-mediated phosphorylation of c-Jun is essential for AP-1 activation, and AP-1 can cooperate with NF-κB at the promoter regions of various pro-inflammatory genes. A decrease in AP-1 activity alone may be sufficient to attenuate NF-κB-driven transcription [45]. Therefore, suppression of JNK signaling may indirectly impair NF-κB-driven gene expression, even without altering NF-κB phosphorylation status. This is consistent with previous findings that inhibition of JNK reduces the expression of iNOS, TNF-α, and COX-2 by modulating NF-κB/AP-1 transcriptional activity [46]. Furthermore, many inflammatory stimuli simultaneously activate JNK, AP-1, and NF-κB signaling pathways, and pharmacological inhibition of JNK has been shown to suppress both AP-1 activation and NF-κB-dependent transcription [47]. These observations support the notion that JNK functions as a critical upstream regulator coordinating multiple transcriptional networks involved in inflammation. Taken together, the present findings suggest that the anti-inflammatory effects of O-BMLE are primarily mediated through inhibition of JNK phosphorylation, leading to suppression of AP-1 activity and subsequent attenuation of NF-κB-dependent gene expression. This mechanism may explain the observed reduction in pro-inflammatory mediators despite the unchanged pNF-κB levels. In addition, the strong suppression of NO production observed in this study suggests that O-BMLE not only downregulates iNOS transcription but also affects post-transcriptional regulation. JNK signaling is also known to control the stability of cytokine and iNOS mRNAs through AU-rich element binding proteins, such as TTP and HuR [48,49]. Therefore, inhibition of the JNK pathway may contribute to both reduced transcription and decreased mRNA stability of inflammatory mediators. Collectively, these findings indicate that compounds in O-BMLE are promising JNK/MAPK-targeting anti-inflammatory agents with a mechanism distinct from that of classical NF-κB inhibitors.

Although MM-I is identified as a major cucurbitane-type triterpenoid in bitter melon and is frequently referred to for both its anticancer and anti-inflammatory properties [9,14], quantitative analysis showed that MM-I levels were not significantly different between BMLE and O-BMLE. Therefore, the enhanced biological activities observed in O-BMLE do not arise from increased MM-I content. The optimization process does not substantially affect the extraction efficiency of highly hydrophobic triterpenoids, such as MM-I, as their solubility and stability remain relatively constant under moderate extraction modifications. Consequently, the biological enhancement of O-BMLE is most likely driven by alterations in other metabolite groups rather than cucurbitane triterpenoids. Phytometabolomic profiling supports this interpretation by showing clear differences in both primary and secondary metabolites following optimization. In positive ion mode, O-BMLE contained higher amounts of lipid-derived metabolites, especially stearidonic acid and monolinolenin, compared with BMLE. Several secondary metabolites were also enriched or uniquely detected in O-BMLE, such as 6-gingerol, and pinoresinol. Notably, stearidonic acid, monolinolenin, 6-gingerol, and pinoresinol are recognized for their antioxidant, anticancer, anti-inflammatory activities, and may contribute to the enhanced effects of O-BMLE [50,51,52,53,54]. In negative ion mode, O-BMLE showed higher levels of phenolic acids, particularly 4-hydroxybenzoic acid, vanillic acid, and esculin, all of which have ROS-reducing and cytokine suppressing properties [55,56,57]. Both extracts contained triterpenoid- and saponin-related compounds, but O-BMLE had a higher amount of pedunculoside. These metabolites, although different from cucurbitane-type triterpenoids, have been reported to modulate the MAPK or NF-κB pathways [58] and may act together with other enriched compounds to enhance the anti-inflammatory activity of O-BMLE. Thus, the combination of these metabolites likely acts synergistically to enhance antioxidant, anticancer, and anti-inflammatory activities providing a more potent biological effect than the conventional extract.

This study presents a novel application of HPP-assisted extraction of bitter melon leaves, while most prior research has focused on the fruit. By integrating BBD and RSM with biological response assessments, the results demonstrate that optimization significantly improves phytochemical profiles and biological activities, including antioxidant, anticancer, and anti-inflammatory effects. These findings highlight the substantial impact of extraction optimization on enhancing bioactivities. Furthermore, mechanistic and phytometabolomic analyses provide new insight into how optimization enhances biological activities at both molecular and metabolite levels. Nevertheless, several limitations should be acknowledged. The current research is based on in vitro experiments; therefore, in vivo studies are necessary to confirm the safety, pharmacokinetics, and therapeutic potential of O-BMLE. The experiments utilized crude extracts containing a mixture of phytochemicals, and the specific compounds responsible for the enhanced biological activities have not yet been fully identified. In addition, cytotoxicity assays were conducted on a limited panel of cancer cell lines. Broader testing across various cancer cell types, such as drug-resistant and inflammation-induced models of cancer progression, is warranted. Finally, further investigation into the molecular mechanisms of action and the combination with standard therapies is recommended to substantiate the anticancer potential of bitter melon leaves.

## 4. Materials and Methods

### 4.1. Chemicals, Reagents, and Antibodies

Dulbecco’s Modified Eagle Medium (DMEM), Roswell Park Memorial Institute-1640 Medium (RPMI-1640), penicillin–streptomycin antibiotics, and 0.5% trypsin-EDTA were purchased from Gibco (Grand Island, NY, USA). Fetal bovine serum (FBS) was obtained from Hyclone (Logan, UT, USA). LPS, Griess reagent, and SRB dye powder were purchased from Sigma-Aldrich (St. Louis, MO, USA). Anti-COX-2, anti-phospho-SAPK/JNK (Thr183/Tyr185), anti-SAPK/JNK, anti-phospho NF-κB p65 (Ser536), and anti-NF-κB p65 antibodies were obtained from Cell Signaling Technology (Danvers, MA, USA). Anti-iNOS and anti-β-actin antibodies were purchased from Sigma-Aldrich (St. Louis, MO, USA). Mammalian cell lysis buffer was obtained from Gold Biotechnology (St. Louis, MO, USA). AmershamTM Protran Premium 0.2-μm nitrocellulose (NC) membranes for Western blotting were supplied by Cytiva (Dassel, Germany). HaltTM Protease Inhibitor Cocktail (100×) was obtained from Thermo Fisher Scientific (Waltham, MA, USA). Horseradish peroxidase (HRP)-conjugated goat anti-rabbit and goat anti-mouse IgG antibodies, as well as ClarityTM Western ECL substrate, were purchased from Bio-Rad Laboratories (Hercules, CA, USA). An HPLC standard, including MM-I (HPLC purity ≥ 98%), was obtained from ChemFaces (Wuhan, Hubei, China). TRIzol reagent was purchased from Invitrogen (Carlsbad, CA, USA). All primers for RT-qPCR were purchased from Bio Basic Inc. (Markham, ON, Canada).

### 4.2. Bitter Melon Leaf Collection and Conventional Extraction

Fresh *Momordica charantia* (bitter melon) leaves were collected from the Suthep Subdistrict, Mueang Chiang Mai District, Chiang Mai Province, Thailand, in August 2024. The extraction process involved soaking the dried leaves in 80% ethanol at a weight-to-volume ratio of 10:100 overnight with continuous stirring. This extraction step was repeated twice. The BMLE was then filtered and concentrated using a rotary evaporator. The concentrated extract was subsequently lyophilized to obtain crude BMLE powder. The extraction yield of BMLE was approximately 24.26%. The BMLE powder was stored at −20 °C until use, and a stock solution was prepared at a concentration of 50 mg/mL in DMSO.

### 4.3. Cell Lines and Cell Culture

Human cancer cell lines, including A549 (lung adenocarcinoma), LNCaP (prostatic carcinoma), HepG2 (hepatocellular carcinoma), and SKOV3 (ovarian carcinoma), as well as 3T3-L1 mouse fibroblasts, were obtained from the American Type Culture Collection (ATCC, Manassas, VA, USA). A549, HepG2, SKOV3, and 3T3-L1 cells were cultured in DMEM, whereas LNCaP cells were maintained in RPMI-1640 medium. All culture media were supplemented with 10% FBS, L-glutamine, and 1% penicillin/streptomycin antibiotics, and cells were incubated at 37 °C in a humidified atmosphere containing 5% CO_2_. Cells were subcultured upon reaching approximately 80% confluence and used for subsequent experiments.

RAW264.7, a murine macrophage cell line, was purchased from CLS Cell Lines Service (Eppelheim, Germany) and cultured in DMEM supplemented with 10% FBS, L-glutamine, 1% penicillin/streptomycin antibiotics in Corning^®^ 60 mm ultra-low attachment culture dishes (Corning, NY, USA) at 37 °C in a humidified atmosphere containing 5% CO_2_. RAW264.7 cells were subcultured every three days.

### 4.4. Cytotoxicity Testing

The cytotoxicity of BMLE was initially evaluated in A549 and RAW264.7 cells using the SRB assay [59]. A549 cells (4000 cells/well) and RAW264.7 cells (20,000 cells/well) were seeded into 96-well plates and incubated at 37 °C, 5% CO_2_ overnight. Cells were then treated with various concentrations of BMLE (0–400 μg/mL) for 48 h. At the end of the treatment period, cells were fixed with 10% (*w*/*v*) cold trichloroacetic acid (TCA) for 1 h at 4 °C. The TCA solution was then discarded, and the plates were gently rinsed with running tap water and air-dried at room temperature. Subsequently, 0.057% (*w*/*v*) SRB solution was added to each well and incubated at room temperature for 30 min. The SRB solution was removed, and the cells were washed three times with 1% (*v*/*v*) acetic acid to remove unbound dye. The plates were then air-dried. The bound dye was dissolved in 10 mM Tris base, and absorbance was measured at 510 nm using a microplate reader (BioTek Synergy H1, BioTek Instruments, Winooski, VT, USA). The percentage of cell viability was calculated as

Cell viability (%) = (Absorbance of treatment group/Absorbance of control group) × 100

After optimization of the extraction process, the cytotoxic effects of the O-BMLE were compared with those of BMLE in A549 cells and extended to other human cancer cell lines, including LNCaP, HepG2, and SKOV3 cells. Normal 3T3-L1 mouse fibroblasts were included to assess selectivity toward cancer cells. LNCaP (3000 cells/well), HepG2 (5000 cells/well), SKOV3 (4000 cells/well), and 3T3-L1 cells (3000 cells/well) were seeded into 96-well plates and incubated at 37 °C, 5% CO_2_ overnight. The cells were then treated with O-BMLE (0–400 μg/mL) for 48 h. Cytotoxicity was determined using the SRB assay as described above. The inhibitory concentration of 50% (IC_50_) values of BMLE and O-BMLE for each cell line were calculated and used to determine the selectivity index (SI) according to the following formula:

SI = IC_50_ of extract (normal cells)/IC_50_ of extract (cancer cells)

In addition, cytotoxicity of O-BMLE was further evaluated in RAW264.7 cells to determine non-toxic doses for further anti-inflammatory experiments.

### 4.5. Determination of NO Production

RAW264.7 macrophages (20,000 cells/well) were seeded into a 96-well plate and incubated overnight. Next, the cells were pretreated with non-toxic concentrations (25, 50, 75, and 100 µg/mL) of BMLE or O-BMLE for 1 h. Inflammation was subsequently induced by adding LPS to each well at a final concentration of 1 µg/mL, followed by incubation for 24 h. At the end of incubation, culture supernatants were collected to determine NO production using the Griess reaction assay, according to the previously described protocols [60]. Briefly, 100 µL of culture supernatant was mixed with 100 µL of Griess reagent solution and incubated at room temperature in the dark for 10 min. The absorbance was measured at 540 nm. In parallel, the treated cells were subjected to the SRB assay to confirm that the observed effects were not due to cytotoxicity.

### 4.6. Box–Behnken Design and Response Surface Methodology for Optimization of High-Pressure Processing

In the initial optimization step, four independent variables were investigated for the HPP of BMLE using HPP equipment (BaoTou-Kefa High-Pressure Technology Co., Ltd., Baotou, China): extraction pressure (X_1_, MPa), extraction time (X_2_, min), ethanol concentration (X_3_, %), and L:S ratio (X_4_, fold). The selected levels were as follows: pressure (300, 400, and 500 MPa); time (30, 60, and 90 min); ethanol concentration (50, 72.5, and 95%); and L:S ratio (5, 10, and 15). Using a four-factor, 29 experimental runs were generated by a three-level BBD. Cytotoxicity against A549 cells was used as the primary endpoint to evaluate the experimental fractions (Supplementary Table S1).

Following the initial optimization, a second BBD was conducted using three independent variables: pressure (X_1_: 100, 350, and 600 MPa); time (X_2_: 30, 60, and 90 min); and L:S ratio (X_3_: 10, 20, and 30-fold) (Table 7). This three-factor BBD generated 17 experimental runs (Table 8). The responses evaluated in this stage included anti-cancer activity (cytotoxicity in A549 cells) and anti-inflammatory activity (NO production in LPS-stimulated RAW264.7 macrophages). All experiments were performed three times. RSM was employed to fit the experimental data to a second-order polynomial model, and a quadratic regression model was used to estimate linear, quadratic, and interaction effects.

### 4.7. Determination of Total Phenolic Content and Total Flavonoid Content

The TPC of BMLE and O-BMLE was determined using the Folin–Ciocalteu assay, following an established protocol from a previous study [61]. Briefly, 20 µL of extract solution was mixed with 100 µL of 10% (*v*/*v*) Folin–Ciocalteu reagent and incubated at room temperature for 5 min. Subsequently, 80 µL of 7.5% (*w*/*v*) sodium carbonate solution was added, and the mixture was incubated at room temperature in the dark for 30 min. The absorbance was measured at 765 nm using a microplate reader. Gallic acid was used as the standard to generate a calibration curve (0–100 µg/mL), and results were expressed as milligrams of gallic acid equivalents per gram of extract (mg GAE/g extract).

The TFC was determined using the aluminum chloride colorimetric method [62]. Briefly, each extract solution was mixed with 10% (*w*/*v*) aluminum chloride solution and incubated at room temperature for 15 min in the dark. The absorbance was measured at 532 nm using a microplate reader. Rutin was used as the calibration standard (0–100 µg/mL), and results were expressed as milligrams of rutin equivalents per gram of extract (mg RE/g extract). All measurements were performed in triplicate.

### 4.8. Determination of Antioxidant Activity

Antioxidant activity of both BMLE and O-BMLE was assessed using FRAP and ORAC assay, as described in the method used of previous study [63]. Trolox was used as a standard. The antioxidant capacity was reported as micromoles of Trolox equivalent per gram of extract (µmol TE/g). All measurements were performed in triplicate with the SynergyTM HT 96-well UV–visible microplate reader and Gen 5 data analysis software (BioTek Instruments, Inc., Winooski, VT, USA).

### 4.9. Reverse Transcription-Quantitative Polymerase Chain Reaction (RT-qPCR)

Following the indicated treatments, total RNA was extracted from the cells using TRIzol reagent (Carlsbad, CA, USA). RNA concentration and purity were determined with a NanoDrop 8 spectrophotometer (Thermo Fisher Scientific, Waltham, MA, USA). The extracted RNA was reverse-transcribed into cDNA using ReverTra AceTM qPCR RT Master Mix (TOYOBO, Osaka, Japan). Quantitative PCR was then used to assess mRNA expression levels of TNF-α, IL-1β, and IL-6, using THUNDERBIRDTM Next SYBR qPCR Mix (TOYOBO, Osaka, Japan) and gene-specific primers. Expression was normalized to GAPDH and compared to the LPS-treated control group using the 2^−ΔΔCT^ method. All experiments were performed in triplicate and within each independent experiment. Primer sequences are provided in Supplementary Table S7.

### 4.10. Enzyme-Linked Immunosorbent Assay (ELISA)

The levels of TNF-α and IL-6 secreted by LPS-stimulated RAW264.7 macrophages into culture media of each treatment group were measured using a sandwich ELISA according to the manufacturer’s guidelines (ELISA MAXTM Deluxe Set, BioLegend, San Diego, CA, USA).

### 4.11. Western Immunoblotting

RAW264.7 macrophages from each treatment group were lysed using a mammalian cell lysis buffer supplemented with protease inhibitor. Protein concentrations were determined using the Bradford assay. Equal amounts of proteins (20 μg per sample) were separated by 10% SDS-PAGE. Proteins were then electrotransferred onto nitrocellulose membranes (Cytiva, Dassel, Germany). The membranes were probed overnight at 4 °C with specific primary antibodies, including anti-phospho-NF-kB p65 (Ser536) (1:1000), anti-NF-kB p65 (1:2000), anti-phospho-SAPK/JNK (Thr183/Tyr185) (1:2000), anti-SAPK/JNK (1:5000), anti-COX-2 (1:2000), anti-iNOS (1:2000), and anti-β-actin (1:10,000). After washing, the membranes were incubated with the appropriate HRP-conjugated secondary antibodies for 2 h at room temperature. Protein bands were visualized using a chemiluminescence detection system and captured with the iBrightTM CL-1500 Imaging System (Thermo Fisher Scientific, Waltham, MA, USA). Band intensities were quantified using ImageJ software version 1.54g (National Institutes of Health, Bethesda, MD, USA) and normalized to β-actin levels.

### 4.12. High-Performance Liquid Chromatography (HPLC) Analysis

The content of MM-I in O-BMLE and BMLE was determined using HPLC. Dried extracts were dissolved in HPLC-grade methanol at 1 mg/mL and sonicated for 15 min to ensure complete dissolution. The solutions were filtered through a 0.22 µm nylon membrane filter before injection. HPLC analysis was performed using an Agilent 1260 Infinity II HPLC system (Agilent Technologies, Santa Clara, CA, USA) equipped with a UV-Vis diode-array detector (DAD). A ZORBAX Eclipse Plus C18 column (4.6 mm × 250 mm, 5 µm particle size) was used for separation, and the column temperature was maintained at 35 °C. The mobile phase consisted of solvent A (0.05% phosphoric acid in HPLC water) and solvent B (acetonitrile). A linear gradient from 50% to 100% solvent B was applied over 10 min, followed by isocratic elution at 100% solvent B for 30 min. The flow rate was set at 1.0 mL/min, and the injection volume was 10 µL. Detection was carried out at 205 nm. MM-I was identified by comparing its retention time and UV absorption spectrum with those of an authentic reference standard. Quantification was performed using an external calibration curve constructed from standard solutions at different concentrations. The content of MM-I was expressed as mg/g of extract.

### 4.13. Non-Targeted Metabolomic Profiling Using HPLC-qTOF-MS

Nontargeted metabolomic analysis of O-BMLE and BMLE was conducted based on a previously reported method [64], with minor modifications to suit the present study. For sample preparation, each dried extract was reconstituted in 0.1% (*v*/*v*) DMSO and filtered through a 0.22 µm membrane filter to remove particulate matter prior to injection. Chromatographic separation was achieved using an Acclaim™ RSLC 120 C18 column (100 mm × 2.1 mm, 2.2 µm particle size, 120 Å pore size; Thermo Fisher Scientific, Waltham, MA, USA) coupled to a high-performance liquid chromatography–quadrupole time-of-flight mass spectrometry (HPLC-qTOF-MS) system (TripleTOF^®^ 6600; AB Sciex, Framingham, MA, USA). The mobile phase consisted of solvent A (0.1% formic acid in water) and solvent B (0.1% formic acid in acetonitrile). A linear gradient program was applied over a total run time of 30 min at a constant flow rate of 0.4 mL/min.

Mass spectrometric detection was performed in both positive and negative electrospray ionization (ESI+) and (ESI−) modes, with full-scan acquisition across an m/z range of 100–1000. Instrumental parameters were optimized to obtain high-resolution spectra with accurate mass measurement. Raw LC–MS data were processed using SCIEX OS software (version 3.3.0; AB Sciex). All putative metabolites detected from both modes were identified by matching their MS/MS fragmentation patterns against the High-Resolution MS/MS Spectral Library and the National Institute of Standards and Technology (NIST) database.

### 4.14. Statistical Analysis

Statistical analysis for the BBD and RSM was performed using Design-Expert version 13 (Stat-Ease Inc., Minneapolis, MN, USA) for experimental design, regression modeling, and graphical analysis. Specifically, analysis of variance (ANOVA) was conducted to evaluate the significance of the model and regression coefficients (*p* < 0.05). Three-dimensional response surface plots were generated to illustrate the relationships between independent variables and measured responses.

All other experiments were performed in triplicate (*n* = 3) within three independent experiments, and the results are presented as mean ± standard deviation (SD), calculated from these three independent experiments. The overall differences among treatment groups were analyzed using a one-way ANOVA followed by Tukey’s post hoc test using GraphPad Prism version 10 (GraphPad Software, Boston, MA, USA) or Student’s *t*-test. Statistical significance was defined as *p*-value < 0.05.

## 5. Conclusions

In summary, the present study demonstrates that HPP combined with BBD and RSM optimization significantly enhances both the extraction efficiency and biological activities of BMLE. The O-BMLE exhibited increased phytochemical content, improved antioxidant capacity, greater anticancer effects with no observed toxicity to normal cells, and strong anti-inflammatory activity through selective inhibition of JNK signaling and reduction of pro-inflammatory mediators, compared to the conventional extract. Phytometabolomic analysis indicated that these enhanced activities were not due to MM-I alone but rather were associated with alterations in other bioactive metabolites enriched following optimization. Overall, these results underscore the potential of O-BMLE, or its principal bioactive constituents, for use as functional food ingredients, nutraceuticals, herbal supplements, or as alternative natural agents for the prevention or management of inflammation-related disorders and cancer.

## Figures and Tables

**Table 1:** Independent Variables (pressure (A), extraction time (B), and L:S ratio (C)) and dependent variables (% inhibition of A549 cell growth and % inhibition of NO production) of BMLE derived from the BBD.

Run	X_1_: Pressure (MPa)	X_2_: Time (Min)	X_3_: L:S Ratio (Fold)	Response 1 Anti-Cancer (% Inhibition of A549 Cell Growth)	Response 2 Anti-Inflammation (% Inhibition of NO Production)
1	600	30	20	13.00 ± 2.83	16.76 ± 7.06
2	100	60	10	30.50 ± 10.61	14.26 ± 6.14
3	350	90	10	35.33 ± 2.08	25.16 ± 5.03
4	100	30	20	25.00 ± 4.24	12.80 ± 4.69
5	600	90	20	18.00 ± 1.41	22.33 ± 3.69
6	350	30	30	16.00 ± 1.41	26.62 ± 5.41
7	600	60	10	39.00 ± 5.20	24.66 ± 7.20
8	600	60	30	11.50 ± 2.12	15.80 ± 7.17
9	350	90	30	23.00 ± 5.29	24.99 ± 4.98
10	350	60	20	25.33 ± 0.58	24.46 ± 9.23
11	350	60	20	24.00 ± 4.24	16.96 ± 13.42
12	350	60	20	19.50 ± 0.71	11.35 ± 5.28
13	100	90	20	29.50 ± 2.12	16.45 ± 7.70
14	100	60	30	20.50 ± 0.71	21.42 ± 8.57
15	350	30	10	26.33 ± 10.12	24.14 ± 5.31
16	350	60	20	19.33 ± 3.79	17.57 ± 1.47
17	350	60	20	23.00 ± 4.58	16.04 ± 0.76

**Table 2:** Analysis of Variance, Regression Coefficients, and *p*-Value of the Second-Order Polynomial Models for % Inhibition of A549 Cell Growth.

Anti-Cancer Activity (% Inhibition of A549 Cell Growth)						
Source	Sum of Squares	DF	Mean Square	F-Value	*p* Value	Significant
**Model**	734.64	9	81.63	4.46	0.0307	*
**A: Pressure**	72.00	1	72.00	3.93	0.0877	
**B: Time**	81.28	1	81.28	4.44	0.0731	
**C: L/S ratio**	452.50	1	452.50	24.73	0.0016	**
**A^2^**	0.44	1	0.44	0.024	0.8805	
**B^2^**	1.20	1	1.20	0.065	0.8054	
**C^2^**	50.60	1	50.60	2.76	0.1403	
**AB**	0.063	1	0.063	3.415 × 10^−3^	0.9550	
**AC**	76.56	1	76.56	4.18	0.0801	
**BC**	1.00	1	1.00	0.055	0.8219	
**Residual**	128.11	7	18.30			
**Lack of Fit**	98.91	3	32.97	4.52	0.0897	
**Pure Error**	29.20	4	7.30			
**Cor Total**	862.75	16				
**R^2^**					0.8515	
**Adjusted R^2^**					0.6606	

Differences in responses were evaluated using ANOVA at a 95% confidence level. Statistical analyses were conducted using Design-Expert software (version 13). * *p* < 0.05, ** *p* < 0.01.

**Table 3:** Analysis of Variance, Regression Coefficients, and *p*-Value of the Second-Order Polynomial Models for % Inhibition of NO Production in LPS-Stimulated RAW264.7 Macrophages.

Anti-Inflammatory Activity (% Inhibition of NO Production)						
Source	Sum of Squares	DF	Mean Square	F-Value	*p* Value	Significant
**Model**	434.19	9	48.24	4.05	0.0392	*
**A: Pressure**	64.36	1	64.36	5.41	0.0530	
**B: Time**	1.22	1	1.22	0.10	0.7581	
**C: L/S ratio**	224.63	1	224.63	18.88	0.0034	**
**A^2^**	14.06	1	14.06	1.18	0.3130	
**B^2^**	74.94	1	74.94	6.30	0.0404	*
**C^2^**	8.90	1	8.90	0.75	0.4158	
**AB**	12.48	1	12.48	1.05	0.3399	
**AC**	34.93	1	34.93	2.94	0.1304	
**BC**	3.36	1	3.36	0.28	0.6114	
**Residual**	83.29	7	11.90			
**Lack of Fit**	32.32	3	10.77	0.85	0.5364	
**Pure Error**	50.97	4	12.74			
**Cor Total**	517.48	16				
**R^2^**					0.8390	
**Adjusted R^2^**					0.6321	

Differences in responses were evaluated using ANOVA at a 95% confidence level. Statistical analyses were conducted using Design-Expert software (version 13). * *p* < 0.05, ** *p* < 0.01.

**Table 4:** The Comparison of TPC, TFC, and Antioxidant Activity between O-BMLE and BMLE.

Assays	O-BMLE	BMLE
**Total phenolic content** (mg of GAE/g extract)	15.99 ± 1.53	19.64 ± 4.90
**Total flavonoid content** (mg of RE/g extract)	27.70 ± 2.40 ***	8.71 ± 1.92
**Antioxidant** FRAP (μmol of TE/g extract) ORAC (μmol of TE/g extract)	96.53 ± 12.96 * 330.40 ± 31.04	71.22 ± 11.06 298.95 ± 20.90

Results are shown as the mean ± standard deviation (SD) of triplicate experiments (*n* = 3). Statistical analysis was performed using an independent *t*-test to compare the optimized condition and the conventional extraction of BMLE. * *p* < 0.05, *** *p* < 0.001; GAE: gallic acid equivalent; RE: rutin equivalent; TE: Trolox equivalent.

**Table 5:** Cytotoxicity and Selectivity Index Analysis of O-BMLE and BMLE in Cancer and Normal Cell Lines.

Cell Lines	IC_50_ Values (μg/mL)		Selectivity Index	
	O-BMLE	BMLE	O-BMLE	BMLE
**Cancer cells**				
A549	58.67 ± 8.61 **	146.65 ± 2.55	5.03	2.60
HepG2	99.55 ± 8.06 **	182.14 ± 18.81	2.96	2.09
SKOV3	135.82 ± 9.40 **	195.77 ± 3.75	2.17	1.95
LNCaP	182.05 ± 24.73	215.71 ± 38.39	1.62	1.76
**Normal cells**				
3T3-L1	295.14 ± 27.82	381.40 ± 11.41	-	-

IC_50_ values are shown as the mean ± SD of triplicate experiments (*n* = 3). SI is calculated by IC_50_ of extract in 3T3-L1 cells/IC_50_ of extract in cancer cells. Statistical analysis was performed using an independent *t*-test to compare the optimized condition and the conventional extraction of BMLE. ** *p* < 0.01, and ns: not statistically significant compared to BMLE.

**Table 6:** Determination and Quantification of MM-I in O-BMLE and BMLE Using HPLC.

Compound	O-BMLE	BMLE
Momordicine-I (mg/g extract)	27.14 ± 2.38	28.93 ± 5.91

Results are shown as the mean ± SD of triplicate experiments (*n* = 3). Statistical analysis was performed using an independent *t*-test to compare the optimized condition and the conventional extraction of BMLE.

**Table 7:** BBD Matrix with Independent Variables and Actual Levels.

Factors	Units	Actual Levels		
		−1	0	+1
Pressure (X_1_)	MPa	100	350	600
Time (X_2_)	Min	30	60	90
L:S ratio (X_3_)	Fold	10	20	30

**Table 8:** The Total of 17 Actual Running Experiments Generated by BBD.

Run	X_1_: Pressure (MPa)	X_2_: Time (Min)	X_3_: L:S Ratio (Fold)
1	600	30	20
2	100	60	10
3	350	90	10
4	100	30	20
5	600	90	20
6	350	30	30
7	600	60	10
8	600	60	30
9	350	90	30
10	350	60	20
11	350	60	20
12	350	60	20
13	100	90	20
14	100	60	30
15	350	30	10
16	350	60	20
17	350	60	20

## Data Availability

The original contributions presented in this study are included in the article/Supplementary Material. Further inquiries can be directed to the corresponding authors.

## Supplementary Materials

The following supporting information can be downloaded at: https://www.mdpi.com/article/10.3390/ijms27114945/s1.
