# Supplementary material for: Box–Behnken Design Optimization of High-Pressure Processed Bitter Melon (Momordica charantia) Leaf Extract Enhancing Phytochemicals, Anticancer, and Anti-Inflammatory Activities
Source: Int J Mol Sci. 2026 May 29;27(11):4945. doi: 10.3390/ijms27114945 (PMC13256669; doi:10.3390/ijms27114945)

(BMLE-negative mode)

25_11_20_12_CBML_N.wiff - CBML_N

Data File	25_11_20_12_CBML_N.wiff	Result Table	CBML-N_CE40
Acquisition Date	2025-11-20T17:03:00	Algorithm Used	MQ4
Acquisition Method	Neg_DIA_20min_250723_001.dam	Instrument Name	TripleTOF 6600
Project		Processing Method	

Extracted Ion Chromatogram

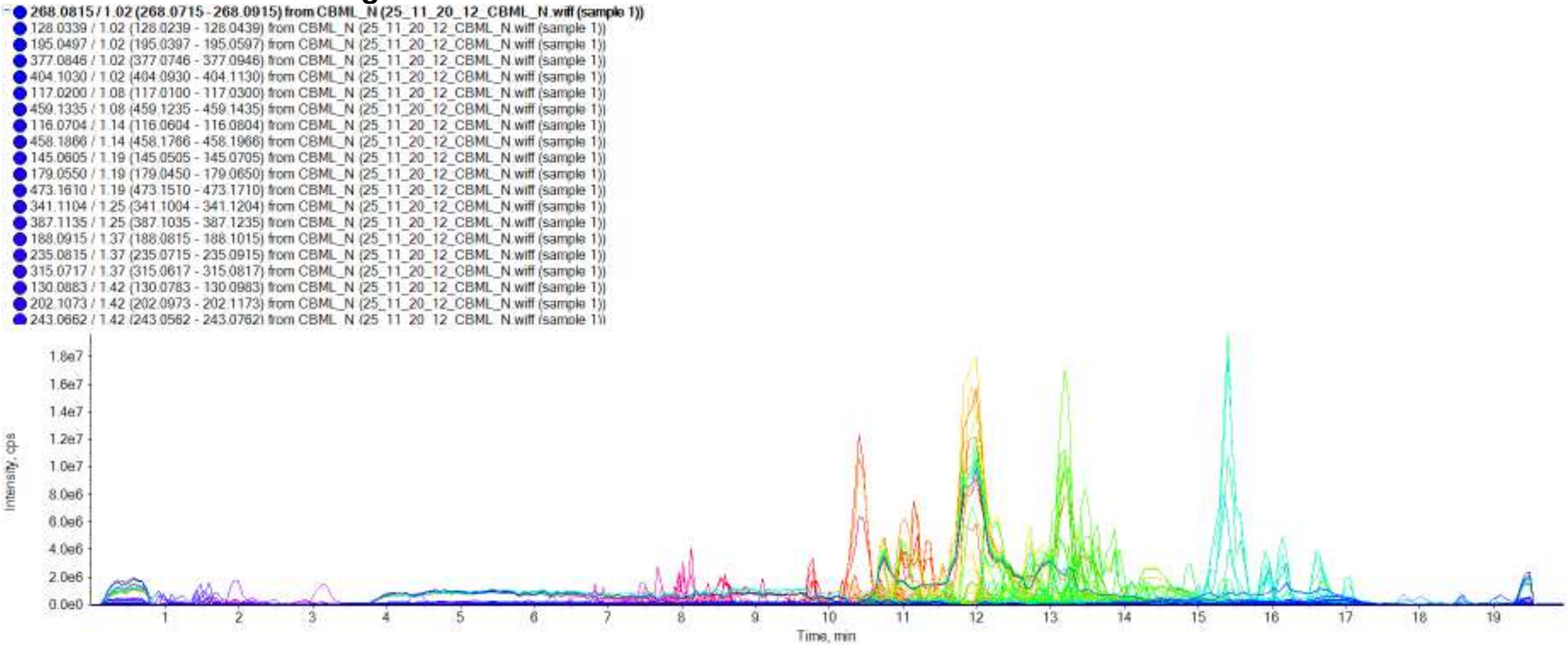

Summary

#	Analyte Peak Name	Mass Error Confidence	Fragment Mass Error Confidence	RT Confidence	Isotope Confidence	Library Confidence	Formula Confidence	Ion Ratio Confidence	Sample Name
2	128.0339 / 1.02	○	○	○	○	✓	○	○	CBML_N
3	195.0497 / 1.02	○	○	○	○	✓	○	○	CBML_N
6	117.0200 / 1.08	○	○	○	○	✓	○	○	CBML_N
8	116.0704 / 1.14	○	○	○	○	✓	○	○	CBML_N
11	179.0550 / 1.19	○	○	○	○	✓	○	○	CBML_N
13	341.1104 / 1.25	○	○	○	○	✓	○	○	CBML_N
15	188.0915 / 1.37	○	○	○	○	✓	○	○	CBML_N
18	130.0883 / 1.42	○	○	○	○	✓	○	○	CBML_N
19	202.1073 / 1.42	○	○	○	○	●	○	○	CBML_N
20	243.0662 / 1.42	○	○	○	○	✓	○	○	CBML_N
26	282.0863 / 1.54	○	○	○	○	✓	○	○	CBML_N
29	299.0810 / 1.59	○	○	○	○	●	○	○	CBML_N
32	175.0599 / 1.54 [M+FA-H]-	○	○	○	○	✓	○	○	CBML_N
34	134.0496 / 1.65	○	○	○	○	✓	○	○	CBML_N
38	206.0811 / 1.76	○	○	○	○	✓	○	○	CBML_N
43	218.1027 / 1.65 [M+AcO-H]-	○	○	○	○	✓	○	○	CBML_N
46	147.0445 / 1.93	○	○	○	○	●	○	○	CBML_N
49	164.0769 / 1.99	○	○	○	○	✓	○	○	CBML_N
52	415.1595 / 2.05	○	○	○	○	✓	○	○	CBML_N
57	192.0658 / 2.22	○	○	○	○	●	○	○	CBML_N

61	138.0543 / 2.39					✓			CBML_N
63	401.1788 / 2.44					▲			CBML_N
66	307.1384 / 2.56					●			CBML_N
67	206.0809 / 2.62					✓			CBML_N
68	327.1064 / 2.62					●			CBML_N
69	153.0179 / 2.67					✓			CBML_N
70	167.0330 / 2.67					✓			CBML_N
77	399.1664 / 2.90					●			CBML_N
78	275.1125 / 3.07					▲			CBML_N
79	203.0886 / 3.13					✓			CBML_N
80	397.1489 / 3.13					▲			CBML_N
83	121.0284 / 3.24					✓			CBML_N
91	339.0703 / 4.83					✓			CBML_N
93	274.8785 / 5.00					●			CBML_N
98	281.1382 / 5.23					●			CBML_N
104	537.1247 / 5.80					▲			CBML_N
106	401.1790 / 5.91					●			CBML_N
108	421.1610 / 6.25					▲			CBML_N
109	431.1920 / 6.25					✓			CBML_N
116	193.0488 / 6.71					✓			CBML_N
117	949.3568 / 6.71					●			CBML_N
119	345.1073 / 6.88					●			CBML_N
120	247.1536 / 6.93					✓			CBML_N

122	551.2332 / 6.99					✓			CBML_N
123	595.1299 / 7.05					✓			CBML_N
126	609.1444 / 7.28					✓			CBML_N
129	187.0957 / 7.45					✓			CBML_N
132	361.2209 / 7.45					▲			CBML_N
135	463.0886 / 7.50					✓			CBML_N
137	271.1533 / 7.62					✓			CBML_N
146	447.0963 / 7.96					✓			CBML_N
147	187.1020 / 8.01					✓			CBML_N
150	144.0446 / 8.13					✓			CBML_N
151	243.1328 / 8.13					●			CBML_N
153	231.1598 / 8.24					✓			CBML_N
154	373.1857 / 8.24					●			CBML_N
155	173.1191 / 8.36					✓			CBML_N
156	478.1970 / 8.36					●			CBML_N
158	381.1546 / 8.41 [M-H]-					●			CBML_N
159	417.1308 / 8.41 [M+Cl]-					●			CBML_N
160	343.2128 / 8.53					●			CBML_N
163	113.0595 / 8.64					●			CBML_N
171	665.3546 / 8.92					✓			CBML_N
173	263.1303 / 9.09					✓			CBML_N
177	329.1591 / 9.38					▲			CBML_N
180	147.0443 / 9.49					✓			CBML_N

185	225.1128 / 9.78								CBML_N
190	745.3439 / 9.78								CBML_N
193	213.1116 / 9.89								CBML_N
197	777.4173 / 9.95 [2M-H]-								CBML_N
203	609.2714 / 10.17								CBML_N
210	593.2624 / 10.34								CBML_N
217	645.3626 / 10.51								CBML_N
226	388.2046 / 10.69								CBML_N
230	777.4202 / 10.69								CBML_N
239	227.1271 / 10.91								CBML_N
241	329.2314 / 10.91								CBML_N
247	811.4116 / 11.03								CBML_N
250	527.3354 / 11.20								CBML_N
255	287.2205 / 11.25								CBML_N
258	195.1373 / 11.37								CBML_N
259	239.1271 / 11.42								CBML_N
262	679.3694 / 11.42								CBML_N
272	669.3755 / 11.59								CBML_N
275	329.2308 / 11.65								CBML_N
294	577.2671 / 12.39								CBML_N
302	669.3763 / 12.73								CBML_N
303	679.4159 / 12.73								CBML_N
309	309.2051 / 12.84								CBML_N

325	601.3735 / 13.19	●	●	●	●	▲	●	●	CBML_N
329	571.2882 / 13.24	●	●	●	●	✓	●	●	CBML_N
348	209.1156 / 13.70	●	●	●	●	✓	●	●	CBML_N
354	581.2985 / 13.87	●	●	●	●	▲	●	●	CBML_N
355	483.2768 / 13.92	●	●	●	●	✓	●	●	CBML_N
362	785.4693 / 14.09	●	●	●	●	🔴	●	●	CBML_N
367	509.2888 / 14.27	●	●	●	●	✓	●	●	CBML_N
376	309.2044 / 14.49	●	●	●	●	✓	●	●	CBML_N
378	695.4022 / 14.21 [M+AcO-H]-	●	●	●	●	✓	●	●	CBML_N
380	279.1941 / 14.55	●	●	●	●	✓	●	●	CBML_N
383	599.3187 / 14.61	●	●	●	●	🔴	●	●	CBML_N
387	601.3718 / 14.72	●	●	●	●	✓	●	●	CBML_N
391	291.1955 / 14.83	●	●	●	●	🔴	●	●	CBML_N
399	365.2676 / 15.00	●	●	●	●	✓	●	●	CBML_N
405	391.2226 / 15.06	●	●	●	●	✓	●	●	CBML_N
407	517.3546 / 15.12	●	●	●	●	🔴	●	●	CBML_N
411	275.2002 / 15.17 [M-H2O-H]-	●	●	●	●	✓	●	●	CBML_N
412	293.2112 / 15.74 [M-H]-	●	●	●	●	✓	●	●	CBML_N
413	865.5004 / 15.23	●	●	●	●	🔴	●	●	CBML_N
414	295.2166 / 15.35	●	●	●	●	🔴	●	●	CBML_N
417	293.2237 / 15.40	●	●	●	●	✓	●	●	CBML_N
418	487.3412 / 15.40	●	●	●	●	✓	●	●	CBML_N
419	511.3052 / 15.40	●	●	●	●	✓	●	●	CBML_N

427	601.3741 / 15.80 [M-H2O-H]-					●			CBML_N
437	291.1983 / 15.97					✓			CBML_N
440	699.3816 / 16.08					●			CBML_N
441	295.2357 / 16.14					✓			CBML_N
442	517.3611 / 16.14					▲			CBML_N
455	573.3777 / 16.77 [M+AcO-H]-					●			CBML_N
457	293.2188 / 16.60					✓			CBML_N
461	505.2998 / 16.71					●			CBML_N
462	585.3797 / 16.71					✓			CBML_N
467	793.5150 / 16.77					✓			CBML_N
474	321.2420 / 16.88 [M-H]-					▲			CBML_N
477	601.3736 / 16.94					✓			CBML_N
480	853.4794 / 16.65 [M+Cl]-					●			CBML_N
482	847.4942 / 16.99					●			CBML_N
487	831.5051 / 17.16					✓			CBML_N
488	289.1796 / 17.22					●			CBML_N
492	269.2123 / 17.28 [M-H]-					✓			CBML_N
494	365.2708 / 17.28					✓			CBML_N
507	263.1999 / 17.50					▲			CBML_N
509	277.2273 / 17.79					✓			CBML_N
511	271.2357 / 18.02					✓			CBML_N
515	253.2175 / 18.13					✓			CBML_N
516	297.2422 / 18.13					✓			CBML_N

519	279.2338 / 18.30	●	●	●	●	✓	●	●	CBML_N
523	255.2415 / 18.58	●	●	●	●	▲	●	●	CBML_N
525	402.2987 / 18.70	●	●	●	●	●	●	●	CBML_N
528	311.2930 / 18.81	●	●	●	●	▲	●	●	CBML_N
531	339.3261 / 18.93	●	●	●	●	✓	●	●	CBML_N
532	255.2392 / 19.10	●	●	●	●	▲	●	●	CBML_N
534	281.2476 / 19.15 [M-H2O-H]-	●	●	●	●	✓	●	●	CBML_N
538	355.3208 / 19.21	●	●	●	●	✓	●	●	CBML_N
542	765.5134 / 19.27	●	●	●	●	✓	●	●	CBML_N
550	831.5075 / 19.32	●	●	●	●	●	●	●	CBML_N

#	Analyte Peak Name	Original Filename
2	128.0339 / 1.02	D:\SCIEX OS Data\MUIN_Mook\Data\2025_11\CE_40\25_11_20_12_CBML_N.wiff
3	195.0497 / 1.02	D:\SCIEX OS Data\MUIN_Mook\Data\2025_11\CE_40\25_11_20_12_CBML_N.wiff
6	117.0200 / 1.08	D:\SCIEX OS Data\MUIN_Mook\Data\2025_11\CE_40\25_11_20_12_CBML_N.wiff
8	116.0704 / 1.14	D:\SCIEX OS Data\MUIN_Mook\Data\2025_11\CE_40\25_11_20_12_CBML_N.wiff
11	179.0550 / 1.19	D:\SCIEX OS Data\MUIN_Mook\Data\2025_11\CE_40\25_11_20_12_CBML_N.wiff
13	341.1104 / 1.25	D:\SCIEX OS Data\MUIN_Mook\Data\2025_11\CE_40\25_11_20_12_CBML_N.wiff
15	188.0915 / 1.37	D:\SCIEX OS Data\MUIN_Mook\Data\2025_11\CE_40\25_11_20_12_CBML_N.wiff
18	130.0883 / 1.42	D:\SCIEX OS Data\MUIN_Mook\Data\2025_11\CE_40\25_11_20_12_CBML_N.wiff
19	202.1073 / 1.42	D:\SCIEX OS Data\MUIN_Mook\Data\2025_11\CE_40\25_11_20_12_CBML_N.wiff
20	243.0662 / 1.42	D:\SCIEX OS Data\MUIN_Mook\Data\2025_11\CE_40\25_11_20_12_CBML_N.wiff
26	282.0863 / 1.54	D:\SCIEX OS Data\MUIN_Mook\Data\2025_11\CE_40\25_11_20_12_CBML_N.wiff
29	299.0810 / 1.59	D:\SCIEX OS Data\MUIN_Mook\Data\2025_11\CE_40\25_11_20_12_CBML_N.wiff
32	175.0599 / 1.54 [M+FA-H]-	D:\SCIEX OS Data\MUIN_Mook\Data\2025_11\CE_40\25_11_20_12_CBML_N.wiff
34	134.0496 / 1.65	D:\SCIEX OS Data\MUIN_Mook\Data\2025_11\CE_40\25_11_20_12_CBML_N.wiff
38	206.0811 / 1.76	D:\SCIEX OS Data\MUIN_Mook\Data\2025_11\CE_40\25_11_20_12_CBML_N.wiff
43	218.1027 / 1.65 [M+AcO-H]-	D:\SCIEX OS Data\MUIN_Mook\Data\2025_11\CE_40\25_11_20_12_CBML_N.wiff
46	147.0445 / 1.93	D:\SCIEX OS Data\MUIN_Mook\Data\2025_11\CE_40\25_11_20_12_CBML_N.wiff
49	164.0769 / 1.99	D:\SCIEX OS Data\MUIN_Mook\Data\2025_11\CE_40\25_11_20_12_CBML_N.wiff
52	415.1595 / 2.05	D:\SCIEX OS Data\MUIN_Mook\Data\2025_11\CE_40\25_11_20_12_CBML_N.wiff
57	192.0658 / 2.22	D:\SCIEX OS Data\MUIN_Mook\Data\2025_11\CE_40\25_11_20_12_CBML_N.wiff
61	138.0543 / 2.39	D:\SCIEX OS Data\MUIN_Mook\Data\2025_11\CE_40\25_11_20_12_CBML_N.wiff
63	401.1788 / 2.44	D:\SCIEX OS Data\MUIN_Mook\Data\2025_11\CE_40\25_11_20_12_CBML_N.wiff
66	307.1384 / 2.56	D:\SCIEX OS Data\MUIN_Mook\Data\2025_11\CE_40\25_11_20_12_CBML_N.wiff
67	206.0809 / 2.62	D:\SCIEX OS Data\MUIN_Mook\Data\2025_11\CE_40\25_11_20_12_CBML_N.wiff

68	327.1064 / 2.62	D:\SCIEX OS Data\MUIN_Mook\Data\2025_11\CE_40\25_11_20_12_CBML_N.wiff
69	153.0179 / 2.67	D:\SCIEX OS Data\MUIN_Mook\Data\2025_11\CE_40\25_11_20_12_CBML_N.wiff
70	167.0330 / 2.67	D:\SCIEX OS Data\MUIN_Mook\Data\2025_11\CE_40\25_11_20_12_CBML_N.wiff
77	399.1664 / 2.90	D:\SCIEX OS Data\MUIN_Mook\Data\2025_11\CE_40\25_11_20_12_CBML_N.wiff
78	275.1125 / 3.07	D:\SCIEX OS Data\MUIN_Mook\Data\2025_11\CE_40\25_11_20_12_CBML_N.wiff
79	203.0886 / 3.13	D:\SCIEX OS Data\MUIN_Mook\Data\2025_11\CE_40\25_11_20_12_CBML_N.wiff
80	397.1489 / 3.13	D:\SCIEX OS Data\MUIN_Mook\Data\2025_11\CE_40\25_11_20_12_CBML_N.wiff
83	121.0284 / 3.24	D:\SCIEX OS Data\MUIN_Mook\Data\2025_11\CE_40\25_11_20_12_CBML_N.wiff
91	339.0703 / 4.83	D:\SCIEX OS Data\MUIN_Mook\Data\2025_11\CE_40\25_11_20_12_CBML_N.wiff
93	274.8785 / 5.00	D:\SCIEX OS Data\MUIN_Mook\Data\2025_11\CE_40\25_11_20_12_CBML_N.wiff
98	281.1382 / 5.23	D:\SCIEX OS Data\MUIN_Mook\Data\2025_11\CE_40\25_11_20_12_CBML_N.wiff
104	537.1247 / 5.80	D:\SCIEX OS Data\MUIN_Mook\Data\2025_11\CE_40\25_11_20_12_CBML_N.wiff
106	401.1790 / 5.91	D:\SCIEX OS Data\MUIN_Mook\Data\2025_11\CE_40\25_11_20_12_CBML_N.wiff
108	421.1610 / 6.25	D:\SCIEX OS Data\MUIN_Mook\Data\2025_11\CE_40\25_11_20_12_CBML_N.wiff
109	431.1920 / 6.25	D:\SCIEX OS Data\MUIN_Mook\Data\2025_11\CE_40\25_11_20_12_CBML_N.wiff
116	193.0488 / 6.71	D:\SCIEX OS Data\MUIN_Mook\Data\2025_11\CE_40\25_11_20_12_CBML_N.wiff
117	949.3568 / 6.71	D:\SCIEX OS Data\MUIN_Mook\Data\2025_11\CE_40\25_11_20_12_CBML_N.wiff
119	345.1073 / 6.88	D:\SCIEX OS Data\MUIN_Mook\Data\2025_11\CE_40\25_11_20_12_CBML_N.wiff
120	247.1536 / 6.93	D:\SCIEX OS Data\MUIN_Mook\Data\2025_11\CE_40\25_11_20_12_CBML_N.wiff
122	551.2332 / 6.99	D:\SCIEX OS Data\MUIN_Mook\Data\2025_11\CE_40\25_11_20_12_CBML_N.wiff
123	595.1299 / 7.05	D:\SCIEX OS Data\MUIN_Mook\Data\2025_11\CE_40\25_11_20_12_CBML_N.wiff
126	609.1444 / 7.28	D:\SCIEX OS Data\MUIN_Mook\Data\2025_11\CE_40\25_11_20_12_CBML_N.wiff
129	187.0957 / 7.45	D:\SCIEX OS Data\MUIN_Mook\Data\2025_11\CE_40\25_11_20_12_CBML_N.wiff
132	361.2209 / 7.45	D:\SCIEX OS Data\MUIN_Mook\Data\2025_11\CE_40\25_11_20_12_CBML_N.wiff
135	463.0886 / 7.50	D:\SCIEX OS Data\MUIN_Mook\Data\2025_11\CE_40\25_11_20_12_CBML_N.wiff
137	271.1533 / 7.62	D:\SCIEX OS Data\MUIN_Mook\Data\2025_11\CE_40\25_11_20_12_CBML_N.wiff
146	447.0963 / 7.96	D:\SCIEX OS Data\MUIN_Mook\Data\2025_11\CE_40\25_11_20_12_CBML_N.wiff
147	187.1020 / 8.01	D:\SCIEX OS Data\MUIN_Mook\Data\2025_11\CE_40\25_11_20_12_CBML_N.wiff
150	144.0446 / 8.13	D:\SCIEX OS Data\MUIN_Mook\Data\2025_11\CE_40\25_11_20_12_CBML_N.wiff
151	243.1328 / 8.13	D:\SCIEX OS Data\MUIN_Mook\Data\2025_11\CE_40\25_11_20_12_CBML_N.wiff
153	231.1598 / 8.24	D:\SCIEX OS Data\MUIN_Mook\Data\2025_11\CE_40\25_11_20_12_CBML_N.wiff
154	373.1857 / 8.24	D:\SCIEX OS Data\MUIN_Mook\Data\2025_11\CE_40\25_11_20_12_CBML_N.wiff
155	173.1191 / 8.36	D:\SCIEX OS Data\MUIN_Mook\Data\2025_11\CE_40\25_11_20_12_CBML_N.wiff
156	478.1970 / 8.36	D:\SCIEX OS Data\MUIN_Mook\Data\2025_11\CE_40\25_11_20_12_CBML_N.wiff
158	381.1546 / 8.41 [M-H]-	D:\SCIEX OS Data\MUIN_Mook\Data\2025_11\CE_40\25_11_20_12_CBML_N.wiff
159	417.1308 / 8.41 [M+Cl]-	D:\SCIEX OS Data\MUIN_Mook\Data\2025_11\CE_40\25_11_20_12_CBML_N.wiff
160	343.2128 / 8.53	D:\SCIEX OS Data\MUIN_Mook\Data\2025_11\CE_40\25_11_20_12_CBML_N.wiff
163	113.0595 / 8.64	D:\SCIEX OS Data\MUIN_Mook\Data\2025_11\CE_40\25_11_20_12_CBML_N.wiff
171	665.3546 / 8.92	D:\SCIEX OS Data\MUIN_Mook\Data\2025_11\CE_40\25_11_20_12_CBML_N.wiff
173	263.1303 / 9.09	D:\SCIEX OS Data\MUIN_Mook\Data\2025_11\CE_40\25_11_20_12_CBML_N.wiff
177	329.1591 / 9.38	D:\SCIEX OS Data\MUIN_Mook\Data\2025_11\CE_40\25_11_20_12_CBML_N.wiff
180	147.0443 / 9.49	D:\SCIEX OS Data\MUIN_Mook\Data\2025_11\CE_40\25_11_20_12_CBML_N.wiff
185	225.1128 / 9.78	D:\SCIEX OS Data\MUIN_Mook\Data\2025_11\CE_40\25_11_20_12_CBML_N.wiff
190	745.3439 / 9.78	D:\SCIEX OS Data\MUIN_Mook\Data\2025_11\CE_40\25_11_20_12_CBML_N.wiff
193	213.1116 / 9.89	D:\SCIEX OS Data\MUIN_Mook\Data\2025_11\CE_40\25_11_20_12_CBML_N.wiff

197	777.4173 / 9.95 [2M-H]-	D:\SCIEX OS Data\MUIN_Mook\Data\2025_11\CE_40\25_11_20_12_CBML_N.wiff
203	609.2714 / 10.17	D:\SCIEX OS Data\MUIN_Mook\Data\2025_11\CE_40\25_11_20_12_CBML_N.wiff
210	593.2624 / 10.34	D:\SCIEX OS Data\MUIN_Mook\Data\2025_11\CE_40\25_11_20_12_CBML_N.wiff
217	645.3626 / 10.51	D:\SCIEX OS Data\MUIN_Mook\Data\2025_11\CE_40\25_11_20_12_CBML_N.wiff
226	388.2046 / 10.69	D:\SCIEX OS Data\MUIN_Mook\Data\2025_11\CE_40\25_11_20_12_CBML_N.wiff
230	777.4202 / 10.69	D:\SCIEX OS Data\MUIN_Mook\Data\2025_11\CE_40\25_11_20_12_CBML_N.wiff
239	227.1271 / 10.91	D:\SCIEX OS Data\MUIN_Mook\Data\2025_11\CE_40\25_11_20_12_CBML_N.wiff
241	329.2314 / 10.91	D:\SCIEX OS Data\MUIN_Mook\Data\2025_11\CE_40\25_11_20_12_CBML_N.wiff
247	811.4116 / 11.03	D:\SCIEX OS Data\MUIN_Mook\Data\2025_11\CE_40\25_11_20_12_CBML_N.wiff
250	527.3354 / 11.20	D:\SCIEX OS Data\MUIN_Mook\Data\2025_11\CE_40\25_11_20_12_CBML_N.wiff
255	287.2205 / 11.25	D:\SCIEX OS Data\MUIN_Mook\Data\2025_11\CE_40\25_11_20_12_CBML_N.wiff
258	195.1373 / 11.37	D:\SCIEX OS Data\MUIN_Mook\Data\2025_11\CE_40\25_11_20_12_CBML_N.wiff
259	239.1271 / 11.42	D:\SCIEX OS Data\MUIN_Mook\Data\2025_11\CE_40\25_11_20_12_CBML_N.wiff
262	679.3694 / 11.42	D:\SCIEX OS Data\MUIN_Mook\Data\2025_11\CE_40\25_11_20_12_CBML_N.wiff
272	669.3755 / 11.59	D:\SCIEX OS Data\MUIN_Mook\Data\2025_11\CE_40\25_11_20_12_CBML_N.wiff
275	329.2308 / 11.65	D:\SCIEX OS Data\MUIN_Mook\Data\2025_11\CE_40\25_11_20_12_CBML_N.wiff
294	577.2671 / 12.39	D:\SCIEX OS Data\MUIN_Mook\Data\2025_11\CE_40\25_11_20_12_CBML_N.wiff
302	669.3763 / 12.73	D:\SCIEX OS Data\MUIN_Mook\Data\2025_11\CE_40\25_11_20_12_CBML_N.wiff
303	679.4159 / 12.73	D:\SCIEX OS Data\MUIN_Mook\Data\2025_11\CE_40\25_11_20_12_CBML_N.wiff
309	309.2051 / 12.84	D:\SCIEX OS Data\MUIN_Mook\Data\2025_11\CE_40\25_11_20_12_CBML_N.wiff
325	601.3735 / 13.19	D:\SCIEX OS Data\MUIN_Mook\Data\2025_11\CE_40\25_11_20_12_CBML_N.wiff
329	571.2882 / 13.24	D:\SCIEX OS Data\MUIN_Mook\Data\2025_11\CE_40\25_11_20_12_CBML_N.wiff
348	209.1156 / 13.70	D:\SCIEX OS Data\MUIN_Mook\Data\2025_11\CE_40\25_11_20_12_CBML_N.wiff
354	581.2985 / 13.87	D:\SCIEX OS Data\MUIN_Mook\Data\2025_11\CE_40\25_11_20_12_CBML_N.wiff
355	483.2768 / 13.92	D:\SCIEX OS Data\MUIN_Mook\Data\2025_11\CE_40\25_11_20_12_CBML_N.wiff
362	785.4693 / 14.09	D:\SCIEX OS Data\MUIN_Mook\Data\2025_11\CE_40\25_11_20_12_CBML_N.wiff
367	509.2888 / 14.27	D:\SCIEX OS Data\MUIN_Mook\Data\2025_11\CE_40\25_11_20_12_CBML_N.wiff
376	309.2044 / 14.49	D:\SCIEX OS Data\MUIN_Mook\Data\2025_11\CE_40\25_11_20_12_CBML_N.wiff
378	695.4022 / 14.21 [M+AcO-H]-	D:\SCIEX OS Data\MUIN_Mook\Data\2025_11\CE_40\25_11_20_12_CBML_N.wiff
380	279.1941 / 14.55	D:\SCIEX OS Data\MUIN_Mook\Data\2025_11\CE_40\25_11_20_12_CBML_N.wiff
383	599.3187 / 14.61	D:\SCIEX OS Data\MUIN_Mook\Data\2025_11\CE_40\25_11_20_12_CBML_N.wiff
387	601.3718 / 14.72	D:\SCIEX OS Data\MUIN_Mook\Data\2025_11\CE_40\25_11_20_12_CBML_N.wiff
391	291.1955 / 14.83	D:\SCIEX OS Data\MUIN_Mook\Data\2025_11\CE_40\25_11_20_12_CBML_N.wiff
399	365.2676 / 15.00	D:\SCIEX OS Data\MUIN_Mook\Data\2025_11\CE_40\25_11_20_12_CBML_N.wiff
405	391.2226 / 15.06	D:\SCIEX OS Data\MUIN_Mook\Data\2025_11\CE_40\25_11_20_12_CBML_N.wiff
407	517.3546 / 15.12	D:\SCIEX OS Data\MUIN_Mook\Data\2025_11\CE_40\25_11_20_12_CBML_N.wiff
411	275.2002 / 15.17 [M-H2O-H]-	D:\SCIEX OS Data\MUIN_Mook\Data\2025_11\CE_40\25_11_20_12_CBML_N.wiff
412	293.2112 / 15.74 [M-H]-	D:\SCIEX OS Data\MUIN_Mook\Data\2025_11\CE_40\25_11_20_12_CBML_N.wiff
413	865.5004 / 15.23	D:\SCIEX OS Data\MUIN_Mook\Data\2025_11\CE_40\25_11_20_12_CBML_N.wiff
414	295.2166 / 15.35	D:\SCIEX OS Data\MUIN_Mook\Data\2025_11\CE_40\25_11_20_12_CBML_N.wiff
417	293.2237 / 15.40	D:\SCIEX OS Data\MUIN_Mook\Data\2025_11\CE_40\25_11_20_12_CBML_N.wiff
418	487.3412 / 15.40	D:\SCIEX OS Data\MUIN_Mook\Data\2025_11\CE_40\25_11_20_12_CBML_N.wiff
419	511.3052 / 15.40	D:\SCIEX OS Data\MUIN_Mook\Data\2025_11\CE_40\25_11_20_12_CBML_N.wiff
427	601.3741 / 15.80 [M-H2O-H]-	D:\SCIEX OS Data\MUIN_Mook\Data\2025_11\CE_40\25_11_20_12_CBML_N.wiff
437	291.1983 / 15.97	D:\SCIEX OS Data\MUIN_Mook\Data\2025_11\CE_40\25_11_20_12_CBML_N.wiff

440	699.3816 / 16.08	D:\SCIEX OS Data\MUIN_Mook\Data\2025_11\CE_40\25_11_20_12_CBML_N.wiff
441	295.2357 / 16.14	D:\SCIEX OS Data\MUIN_Mook\Data\2025_11\CE_40\25_11_20_12_CBML_N.wiff
442	517.3611 / 16.14	D:\SCIEX OS Data\MUIN_Mook\Data\2025_11\CE_40\25_11_20_12_CBML_N.wiff
455	573.3777 / 16.77 [M+AcO-H]-	D:\SCIEX OS Data\MUIN_Mook\Data\2025_11\CE_40\25_11_20_12_CBML_N.wiff
457	293.2188 / 16.60	D:\SCIEX OS Data\MUIN_Mook\Data\2025_11\CE_40\25_11_20_12_CBML_N.wiff
461	505.2998 / 16.71	D:\SCIEX OS Data\MUIN_Mook\Data\2025_11\CE_40\25_11_20_12_CBML_N.wiff
462	585.3797 / 16.71	D:\SCIEX OS Data\MUIN_Mook\Data\2025_11\CE_40\25_11_20_12_CBML_N.wiff
467	793.5150 / 16.77	D:\SCIEX OS Data\MUIN_Mook\Data\2025_11\CE_40\25_11_20_12_CBML_N.wiff
474	321.2420 / 16.88 [M-H]-	D:\SCIEX OS Data\MUIN_Mook\Data\2025_11\CE_40\25_11_20_12_CBML_N.wiff
477	601.3736 / 16.94	D:\SCIEX OS Data\MUIN_Mook\Data\2025_11\CE_40\25_11_20_12_CBML_N.wiff
480	853.4794 / 16.65 [M+Cl]-	D:\SCIEX OS Data\MUIN_Mook\Data\2025_11\CE_40\25_11_20_12_CBML_N.wiff
482	847.4942 / 16.99	D:\SCIEX OS Data\MUIN_Mook\Data\2025_11\CE_40\25_11_20_12_CBML_N.wiff
487	831.5051 / 17.16	D:\SCIEX OS Data\MUIN_Mook\Data\2025_11\CE_40\25_11_20_12_CBML_N.wiff
488	289.1796 / 17.22	D:\SCIEX OS Data\MUIN_Mook\Data\2025_11\CE_40\25_11_20_12_CBML_N.wiff
492	269.2123 / 17.28 [M-H]-	D:\SCIEX OS Data\MUIN_Mook\Data\2025_11\CE_40\25_11_20_12_CBML_N.wiff
494	365.2708 / 17.28	D:\SCIEX OS Data\MUIN_Mook\Data\2025_11\CE_40\25_11_20_12_CBML_N.wiff
507	263.1999 / 17.50	D:\SCIEX OS Data\MUIN_Mook\Data\2025_11\CE_40\25_11_20_12_CBML_N.wiff
509	277.2273 / 17.79	D:\SCIEX OS Data\MUIN_Mook\Data\2025_11\CE_40\25_11_20_12_CBML_N.wiff
511	271.2357 / 18.02	D:\SCIEX OS Data\MUIN_Mook\Data\2025_11\CE_40\25_11_20_12_CBML_N.wiff
515	253.2175 / 18.13	D:\SCIEX OS Data\MUIN_Mook\Data\2025_11\CE_40\25_11_20_12_CBML_N.wiff
516	297.2422 / 18.13	D:\SCIEX OS Data\MUIN_Mook\Data\2025_11\CE_40\25_11_20_12_CBML_N.wiff
519	279.2338 / 18.30	D:\SCIEX OS Data\MUIN_Mook\Data\2025_11\CE_40\25_11_20_12_CBML_N.wiff
523	255.2415 / 18.58	D:\SCIEX OS Data\MUIN_Mook\Data\2025_11\CE_40\25_11_20_12_CBML_N.wiff
525	402.2987 / 18.70	D:\SCIEX OS Data\MUIN_Mook\Data\2025_11\CE_40\25_11_20_12_CBML_N.wiff
528	311.2930 / 18.81	D:\SCIEX OS Data\MUIN_Mook\Data\2025_11\CE_40\25_11_20_12_CBML_N.wiff
531	339.3261 / 18.93	D:\SCIEX OS Data\MUIN_Mook\Data\2025_11\CE_40\25_11_20_12_CBML_N.wiff
532	255.2392 / 19.10	D:\SCIEX OS Data\MUIN_Mook\Data\2025_11\CE_40\25_11_20_12_CBML_N.wiff
534	281.2476 / 19.15 [M-H2O-H]-	D:\SCIEX OS Data\MUIN_Mook\Data\2025_11\CE_40\25_11_20_12_CBML_N.wiff
538	355.3208 / 19.21	D:\SCIEX OS Data\MUIN_Mook\Data\2025_11\CE_40\25_11_20_12_CBML_N.wiff
542	765.5134 / 19.27	D:\SCIEX OS Data\MUIN_Mook\Data\2025_11\CE_40\25_11_20_12_CBML_N.wiff
550	831.5075 / 19.32	D:\SCIEX OS Data\MUIN_Mook\Data\2025_11\CE_40\25_11_20_12_CBML_N.wiff

#	Analyte Peak Name	Sample Type	Component Name	Component Type	Component Group Name	Expected RT	Area	Retention Time	Retention Time Delta (min)	Formula	Precursor Mass
2	128.0339 / 1.02	Unknown	128.0339 / 1.02	Quantifiers	[No data for] Component Group Name	1.02	1.673e+06	1.02	N/A	{129.04063}	128.034
3	195.0497 / 1.02	Unknown	195.0497 / 1.02	Quantifiers	[No data for] Component Group Name	1.02	1.074e+07	0.90	N/A	{196.05646}	195.050
6	117.0200 / 1.08	Unknown	117.0200 / 1.08	Quantifiers	[No data for] Component Group Name	1.08	4.519e+06	1.08	N/A	{118.02668}	117.020
8	116.0704 / 1.14	Unknown	116.0704 / 1.14	Quantifiers	[No data for] Component Group	1.14	1.579e+06	1.22	N/A	{117.07710}	116.070

					Name						
11	179.0550 / 1.19	Unknown	179.0550 / 1.19	Quantifiers	[No data for] Component Group Name	1.20	2.368e+06	1.18	N/A	{180.06168}	179.055
13	341.1104 / 1.25	Unknown	341.1104 / 1.25	Quantifiers	[No data for] Component Group Name	1.25	7.278e+06	1.20	N/A	{342.11708}	341.110
15	188.0915 / 1.37	Unknown	188.0915 / 1.37	Quantifiers	[No data for] Component Group Name	1.37	1.232e+06	1.30	N/A	{189.09827}	188.092
18	130.0883 / 1.42	Unknown	130.0883 / 1.42	Quantifiers	[No data for] Component Group Name	1.42	4.789e+06	1.42	N/A	{131.09507}	130.088
19	202.1073 / 1.42	Unknown	202.1073 / 1.42	Quantifiers	[No data for] Component Group Name	1.42	1.119e+06	1.44	N/A	{203.11404}	202.107
20	243.0662 / 1.42	Unknown	243.0662 / 1.42	Quantifiers	[No data for] Component Group Name	1.42	7.017e+06	1.42	N/A	{244.07291}	243.066
26	282.0863 / 1.54	Unknown	282.0863 / 1.54	Quantifiers	[No data for] Component Group Name	1.54	7.943e+06	1.51	N/A	{283.09301}	282.086
29	299.0810 / 1.59	Unknown	299.0810 / 1.59	Quantifiers	[No data for] Component Group Name	1.59	1.241e+07	1.59	N/A	{300.08768}	299.081
32	175.0599 / 1.54 [M+FA-H]-	Unknown	175.0599 / 1.54 [M+FA-H]-	Qualifiers	129.0549 / 1.65	1.54	1.388e+06	1.54	N/A	{130.06115}	175.060
34	134.0496 / 1.65	Unknown	134.0496 / 1.65	Quantifiers	[No data for] Component Group Name	1.65	1.164e+07	1.67	N/A	{135.05637}	134.050
38	206.0811 / 1.76	Unknown	206.0811 / 1.76	Quantifiers	[No data for] Component Group Name	1.76	2.757e+06	1.73	N/A	{207.08782}	206.081
43	218.1027 / 1.65 [M+AcO-H]-	Unknown	218.1027 / 1.65 [M+AcO-H]-	Quantifiers	218.1027 / 1.65	1.65	1.793e+06	1.65	N/A	{159.08828}	218.103
46	147.0445 / 1.93	Unknown	147.0445 / 1.93	Quantifiers	[No data for] Component Group Name	1.93	4.181e+06	1.95	N/A	{148.05122}	147.044
49	164.0769 / 1.99	Unknown	164.0769 / 1.99	Quantifiers	[No data for] Component Group Name	1.99	2.307e+07	1.95	N/A	{165.08362}	164.077
52	415.1595 / 2.05	Unknown	415.1595 / 2.05	Quantifiers	[No data for] Component Group Name	2.05	3.474e+06	1.96	N/A	{416.16626}	415.160
57	192.0658 / 2.22	Unknown	192.0658 / 2.22	Quantifiers	[No data for] Component Group	2.22	1.582e+06	2.24	N/A	{193.07249}	192.066

					Name						
61	138.0543 / 2.39	Unknown	138.0543 / 2.39	Quantifiers	[No data for] Component Group Name	2.39	6.325e+05	2.41	N/A	{139.06101}	138.054
63	401.1788 / 2.44	Unknown	401.1788 / 2.44	Quantifiers	[No data for] Component Group Name	2.45	4.479e+05	2.45	N/A	{402.18552}	401.179
66	307.1384 / 2.56	Unknown	307.1384 / 2.56	Quantifiers	[No data for] Component Group Name	2.56	1.030e+06	2.60	N/A	{308.14508}	307.138
67	206.0809 / 2.62	Unknown	206.0809 / 2.62	Quantifiers	[No data for] Component Group Name	2.62	1.199e+06	2.63	N/A	{207.08766}	206.081
68	327.1064 / 2.62	Unknown	327.1064 / 2.62	Quantifiers	[No data for] Component Group Name	2.62	4.786e+05	2.64	N/A	{328.11314}	327.106
69	153.0179 / 2.67	Unknown	153.0179 / 2.67	Quantifiers	[No data for] Component Group Name	2.67	6.323e+05	2.69	N/A	{154.02459}	153.018
70	167.0330 / 2.67	Unknown	167.0330 / 2.67	Quantifiers	[No data for] Component Group Name	2.67	6.316e+05	2.69	N/A	{168.03978}	167.033
77	399.1664 / 2.90	Unknown	399.1664 / 2.90	Quantifiers	[No data for] Component Group Name	2.90	5.133e+06	2.88	N/A	{400.17312}	399.166
78	275.1125 / 3.07	Unknown	275.1125 / 3.07	Quantifiers	[No data for] Component Group Name	3.07	1.701e+06	3.06	N/A	{276.11920}	275.112
79	203.0886 / 3.13	Unknown	203.0886 / 3.13	Quantifiers	[No data for] Component Group Name	3.13	2.249e+07	3.13	N/A	{204.09531}	203.089
80	397.1489 / 3.13	Unknown	397.1489 / 3.13	Quantifiers	[No data for] Component Group Name	3.13	1.338e+06	3.14	N/A	{398.15560}	397.149
83	121.0284 / 3.24	Unknown	121.0284 / 3.24	Quantifiers	[No data for] Component Group Name	3.24	2.706e+06	3.32	N/A	{122.03513}	121.028
91	339.0703 / 4.83	Unknown	339.0703 / 4.83	Quantifiers	[No data for] Component Group Name	4.83	2.259e+06	4.81	N/A	{340.07700}	339.070
93	274.8785 / 5.00	Unknown	274.8785 / 5.00	Quantifiers	[No data for] Component Group Name	5.00	3.818e+06	5.35	N/A	{275.88521}	274.878
98	281.1382 / 5.23	Unknown	281.1382 / 5.23	Quantifiers	[No data for] Component Group Name	5.23	7.519e+06	5.11	N/A	{282.14491}	281.138

104	537.1247 / 5.80	Unknown	537.1247 / 5.80	Quantifiers	[No data for] Component Group Name	5.80	1.622e+06	5.80	N/A	{538.13143}	537.125
106	401.1790 / 5.91	Unknown	401.1790 / 5.91	Quantifiers	[No data for] Component Group Name	5.91	6.748e+06	5.92	N/A	{402.18574}	401.179
108	421.1610 / 6.25	Unknown	421.1610 / 6.25	Quantifiers	[No data for] Component Group Name	6.25	1.472e+06	6.25	N/A	{422.16773}	421.161
109	431.1920 / 6.25	Unknown	431.1920 / 6.25	Quantifiers	[No data for] Component Group Name	6.25	5.573e+06	6.25	N/A	{432.19876}	431.192
116	193.0488 / 6.71	Unknown	193.0488 / 6.71	Quantifiers	[No data for] Component Group Name	6.71	4.589e+06	6.65	N/A	{194.05552}	193.049
117	949.3568 / 6.71	Unknown	949.3568 / 6.71	Quantifiers	[No data for] Component Group Name	6.71	1.009e+06	6.72	N/A	{950.36357}	949.357
119	345.1073 / 6.88	Unknown	345.1073 / 6.88	Quantifiers	[No data for] Component Group Name	6.88	2.681e+06	6.88	N/A	{346.11401}	345.107
120	247.1536 / 6.93	Unknown	247.1536 / 6.93	Quantifiers	[No data for] Component Group Name	6.93	3.147e+06	6.90	N/A	{248.16029}	247.154
122	551.2332 / 6.99	Unknown	551.2332 / 6.99	Quantifiers	[No data for] Component Group Name	6.99	8.559e+06	7.01	N/A	{552.23995}	551.233
123	595.1299 / 7.05	Unknown	595.1299 / 7.05	Quantifiers	[No data for] Component Group Name	7.05	3.937e+06	7.05	N/A	{596.13662}	595.130
126	609.1444 / 7.28	Unknown	609.1444 / 7.28	Quantifiers	[No data for] Component Group Name	7.28	2.497e+06	7.28	N/A	{610.15108}	609.144
129	187.0957 / 7.45	Unknown	187.0957 / 7.45	Quantifiers	[No data for] Component Group Name	7.45	3.786e+06	7.46	N/A	{188.10243}	187.096
132	361.2209 / 7.45	Unknown	361.2209 / 7.45	Quantifiers	[No data for] Component Group Name	7.45	5.083e+06	7.54	N/A	{362.22764}	361.221
135	463.0886 / 7.50	Unknown	463.0886 / 7.50	Quantifiers	[No data for] Component Group Name	7.50	1.040e+07	7.51	N/A	{464.09529}	463.089
137	271.1533 / 7.62	Unknown	271.1533 / 7.62	Quantifiers	[No data for] Component Group Name	7.62	1.193e+07	7.67	N/A	{272.16008}	271.153
146	447.0963 / 7.96	Unknown	447.0963 / 7.96	Quantifiers	[No data for]	7.96	9.416e+06	7.97	N/A	{448.10299}	447.096

					Component Group Name						
147	187.1020 / 8.01	Unknown	187.1020 / 8.01	Quantifiers	[No data for] Component Group Name	8.01	1.435e+07	8.01	N/A	{188.10875}	187.102
150	144.0446 / 8.13	Unknown	144.0446 / 8.13	Quantifiers	[No data for] Component Group Name	8.13	3.260e+06	8.11	N/A	{145.05136}	144.045
151	243.1328 / 8.13	Unknown	243.1328 / 8.13	Quantifiers	[No data for] Component Group Name	8.13	4.450e+07	8.12	N/A	{244.13949}	243.133
153	231.1598 / 8.24	Unknown	231.1598 / 8.24	Quantifiers	[No data for] Component Group Name	8.24	6.117e+06	8.26	N/A	{232.16655}	231.160
154	373.1857 / 8.24	Unknown	373.1857 / 8.24	Quantifiers	[No data for] Component Group Name	8.24	4.462e+06	8.24	N/A	{374.19245}	373.186
155	173.1191 / 8.36	Unknown	173.1191 / 8.36	Quantifiers	[No data for] Component Group Name	8.36	7.236e+06	8.36	N/A	{174.12587}	173.119
156	478.1970 / 8.36	Unknown	478.1970 / 8.36	Quantifiers	[No data for] Component Group Name	8.36	2.616e+06	8.36	N/A	{479.20375}	478.197
158	381.1546 / 8.41 [M-H]-	Unknown	381.1546 / 8.41 [M-H]-	Quantifiers	381.1546 / 8.41	8.41	4.622e+06	8.41	N/A	{382.16131}	381.155
159	417.1308 / 8.41 [M+Cl]-	Unknown	417.1308 / 8.41 [M+Cl]-	Qualifiers	381.1546 / 8.41	8.41	2.897e+06	8.41	N/A	{382.16081}	417.131
160	343.2128 / 8.53	Unknown	343.2128 / 8.53	Quantifiers	[No data for] Component Group Name	8.53	3.540e+07	8.56	N/A	{344.21957}	343.213
163	113.0595 / 8.64	Unknown	113.0595 / 8.64	Quantifiers	[No data for] Component Group Name	8.64	8.449e+06	8.46	N/A	{114.06621}	113.059
171	665.3546 / 8.92	Unknown	665.3546 / 8.92	Quantifiers	[No data for] Component Group Name	8.92	4.007e+06	8.91	N/A	{666.36136}	665.355
173	263.1303 / 9.09	Unknown	263.1303 / 9.09	Quantifiers	[No data for] Component Group Name	9.09	1.028e+07	9.09	N/A	{264.13700}	263.130
177	329.1591 / 9.38	Unknown	329.1591 / 9.38	Quantifiers	[No data for] Component Group Name	9.38	1.205e+07	9.35	N/A	{330.16580}	329.159
180	147.0443 / 9.49	Unknown	147.0443 / 9.49	Quantifiers	[No data for] Component Group Name	9.49	2.683e+06	9.47	N/A	{148.05103}	147.044
185	225.1128 / 9.78	Unknown	225.1128 / 9.78	Quantifiers	[No data for]	9.78	1.117e+07	9.78	N/A	{226.11956}	225.113

					Component Group Name						
190	745.3439 / 9.78	Unknown	745.3439 / 9.78	Quantifiers	[No data for] Component Group Name	9.78	5.728e+06	9.80	N/A	{746.35060}	745.344
193	213.1116 / 9.89	Unknown	213.1116 / 9.89	Quantifiers	[No data for] Component Group Name	9.89	2.961e+06	9.89	N/A	{214.11835}	213.112
197	777.4173 / 9.95 [2M-H]-	Unknown	777.4173 / 9.95 [2M-H]-	Qualifiers	388.2040 / 9.95	9.95	2.826e+06	9.96	N/A	{389.21176}	777.417
203	609.2714 / 10.17	Unknown	609.2714 / 10.17	Quantifiers	[No data for] Component Group Name	10.17	1.066e+07	10.17	N/A	{610.27809}	609.271
210	593.2624 / 10.34	Unknown	593.2624 / 10.34	Quantifiers	[No data for] Component Group Name	10.34	6.372e+06	10.36	N/A	{594.26913}	593.262
217	645.3626 / 10.51	Unknown	645.3626 / 10.51	Quantifiers	[No data for] Component Group Name	10.52	1.780e+07	10.55	N/A	{646.36933}	645.363
226	388.2046 / 10.69	Unknown	388.2046 / 10.69	Quantifiers	[No data for] Component Group Name	10.69	1.701e+07	10.74	N/A	{389.21130}	388.205
230	777.4202 / 10.69	Unknown	777.4202 / 10.69	Quantifiers	[No data for] Component Group Name	10.69	2.786e+07	10.73	N/A	{778.42695}	777.420
239	227.1271 / 10.91	Unknown	227.1271 / 10.91	Quantifiers	[No data for] Component Group Name	10.91	1.004e+07	10.79	N/A	{228.13383}	227.127
241	329.2314 / 10.91	Unknown	329.2314 / 10.91	Quantifiers	[No data for] Component Group Name	10.91	1.689e+07	10.89	N/A	{330.23818}	329.231
247	811.4116 / 11.03	Unknown	811.4116 / 11.03	Quantifiers	[No data for] Component Group Name	11.03	4.678e+06	11.03	N/A	{812.41833}	811.412
250	527.3354 / 11.20	Unknown	527.3354 / 11.20	Quantifiers	[No data for] Component Group Name	11.20	8.467e+06	11.20	N/A	{528.34211}	527.335
255	287.2205 / 11.25	Unknown	287.2205 / 11.25	Quantifiers	[No data for] Component Group Name	11.25	2.751e+06	11.28	N/A	{288.22723}	287.221
258	195.1373 / 11.37	Unknown	195.1373 / 11.37	Quantifiers	[No data for] Component Group Name	11.37	4.573e+06	11.39	N/A	{196.14402}	195.137
259	239.1271 / 11.42	Unknown	239.1271 / 11.42	Quantifiers	[No data for] Component Group Name	11.42	5.179e+06	11.42	N/A	{240.13383}	239.127

262	679.3694 / 11.42	Unknown	679.3694 / 11.42	Quantifiers	[No data for] Component Group Name	11.42	4.155e+06	11.42	N/A	{680.37612}	679.369
272	669.3755 / 11.59	Unknown	669.3755 / 11.59	Quantifiers	[No data for] Component Group Name	11.59	2.043e+06	11.60	N/A	{670.38219}	669.375
275	329.2308 / 11.65	Unknown	329.2308 / 11.65	Quantifiers	[No data for] Component Group Name	11.65	7.232e+06	11.61	N/A	{330.23749}	329.231
294	577.2671 / 12.39	Unknown	577.2671 / 12.39	Quantifiers	[No data for] Component Group Name	12.39	1.063e+07	12.41	N/A	{578.27387}	577.267
302	669.3763 / 12.73	Unknown	669.3763 / 12.73	Quantifiers	[No data for] Component Group Name	12.73	9.605e+06	12.70	N/A	{670.38301}	669.376
303	679.4159 / 12.73	Unknown	679.4159 / 12.73	Quantifiers	[No data for] Component Group Name	12.73	5.316e+07	12.71	N/A	{680.42258}	679.416
309	309.2051 / 12.84	Unknown	309.2051 / 12.84	Quantifiers	[No data for] Component Group Name	12.85	1.405e+07	12.85	N/A	{310.21187}	309.205
325	601.3735 / 13.19	Unknown	601.3735 / 13.19	Quantifiers	[No data for] Component Group Name	13.19	3.589e+07	13.17	N/A	{602.38022}	601.373
329	571.2882 / 13.24	Unknown	571.2882 / 13.24	Quantifiers	[No data for] Component Group Name	13.24	2.712e+07	13.24	N/A	{572.29492}	571.288
348	209.1156 / 13.70	Unknown	209.1156 / 13.70	Quantifiers	[No data for] Component Group Name	13.70	2.840e+06	13.70	N/A	{210.12232}	209.116
354	581.2985 / 13.87	Unknown	581.2985 / 13.87	Quantifiers	[No data for] Component Group Name	13.87	3.468e+06	13.87	N/A	{582.30526}	581.299
355	483.2768 / 13.92	Unknown	483.2768 / 13.92	Quantifiers	[No data for] Component Group Name	13.92	3.754e+07	13.94	N/A	{484.28349}	483.277
362	785.4693 / 14.09	Unknown	785.4693 / 14.09	Quantifiers	[No data for] Component Group Name	14.10	5.156e+06	14.11	N/A	{786.47604}	785.469
367	509.2888 / 14.27	Unknown	509.2888 / 14.27	Quantifiers	[No data for] Component Group Name	14.27	1.519e+07	14.27	N/A	{510.29551}	509.289
376	309.2044 / 14.49	Unknown	309.2044 / 14.49	Quantifiers	[No data for] Component Group Name	14.49	2.872e+06	14.51	N/A	{310.21109}	309.204
378	695.4022 / 14.21	Unknown	695.4022 / 14.21	Qualifiers	617.3696 / 14.49	14.21	7.563e+06	14.18	N/A	{636.38775}	695.402

	**[M+AcO-H]-**		**[M+AcO-H]-**									

380	279.1941 / 14.55	Unknown	279.1941 / 14.55	Quantifiers	[No data for] Component Group Name	14.55	2.966e+06	14.48	N/A	{280.20080}	279.194
383	599.3187 / 14.61	Unknown	599.3187 / 14.61	Quantifiers	[No data for] Component Group Name	14.61	4.836e+06	14.62	N/A	{600.32544}	599.319
387	601.3718 / 14.72	Unknown	601.3718 / 14.72	Quantifiers	[No data for] Component Group Name	14.72	6.496e+06	14.78	N/A	{602.37853}	601.372
391	291.1955 / 14.83	Unknown	291.1955 / 14.83	Quantifiers	[No data for] Component Group Name	14.83	9.562e+06	14.84	N/A	{292.20220}	291.195
399	365.2676 / 15.00	Unknown	365.2676 / 15.00	Quantifiers	[No data for] Component Group Name	15.00	5.934e+06	15.19	N/A	{366.27435}	365.268
405	391.2226 / 15.06	Unknown	391.2226 / 15.06	Quantifiers	[No data for] Component Group Name	15.06	2.576e+06	15.03	N/A	{392.22937}	391.223
407	517.3546 / 15.12	Unknown	517.3546 / 15.12	Quantifiers	[No data for] Component Group Name	15.12	1.353e+07	15.12	N/A	{518.36132}	517.355
411	275.2002 / 15.17 [M-H2O-H]-	Unknown	275.2002 / 15.17 [M-H2O-H]-	Qualifiers	339.2161 / 15.57	15.18	7.347e+06	15.24	N/A	{294.21751}	275.200
412	293.2112 / 15.74 [M-H]-	Unknown	293.2112 / 15.74 [M-H]-	Qualifiers	339.2161 / 15.57	15.74	1.736e+08	15.41	N/A	{294.21789}	293.211
413	865.5004 / 15.23	Unknown	865.5004 / 15.23	Quantifiers	[No data for] Component Group Name	15.23	8.124e+06	15.25	N/A	{866.50712}	865.500
414	295.2166 / 15.35	Unknown	295.2166 / 15.35	Quantifiers	[No data for] Component Group Name	15.35	1.409e+07	15.33	N/A	{296.22334}	295.217
417	293.2237 / 15.40	Unknown	293.2237 / 15.40	Quantifiers	[No data for] Component Group Name	15.40	2.727e+08	15.39	N/A	{294.23047}	293.224
418	487.3412 / 15.40	Unknown	487.3412 / 15.40	Quantifiers	[No data for] Component Group Name	15.40	1.612e+07	15.42	N/A	{488.34794}	487.341
419	511.3052 / 15.40	Unknown	511.3052 / 15.40	Quantifiers	[No data for] Component Group Name	15.40	4.157e+07	15.43	N/A	{512.31189}	511.305
427	601.3741 / 15.80 [M-H2O-H]-	Unknown	601.3741 / 15.80 [M-H2O-H]-	Quantifiers	601.3741 / 15.80	15.80	1.509e+07	15.84	N/A	{620.39136}	601.374
437	291.1983 / 15.97	Unknown	291.1983 / 15.97	Quantifiers	[No data for] Component Group Name	15.97	2.562e+07	15.96	N/A	{292.20502}	291.198
440	699.3816 / 16.08	Unknown	699.3816 / 16.08	Quantifiers	[No data for]	16.08	3.149e+06	16.11	N/A	{700.38835}	699.382

					Component Group Name						
441	295.2357 / 16.14	Unknown	295.2357 / 16.14	Quantifiers	[No data for] Component Group Name	16.14	4.835e+07	16.14	N/A	{296.24247}	295.236
442	517.3611 / 16.14	Unknown	517.3611 / 16.14	Quantifiers	[No data for] Component Group Name	16.14	3.564e+07	16.12	N/A	{518.36780}	517.361
455	573.3777 / 16.77 [M+AcO-H]-	Unknown	573.3777 / 16.77 [M+AcO-H]-	Qualifiers	513.3568 / 16.54	16.77	1.953e+06	16.74	N/A	{514.36326}	573.378
457	293.2188 / 16.60	Unknown	293.2188 / 16.60	Quantifiers	[No data for] Component Group Name	16.60	4.283e+07	16.63	N/A	{294.22550}	293.219
461	505.2998 / 16.71	Unknown	505.2998 / 16.71	Quantifiers	[No data for] Component Group Name	16.71	3.718e+06	16.70	N/A	{506.30651}	505.300
462	585.3797 / 16.71	Unknown	585.3797 / 16.71	Quantifiers	[No data for] Component Group Name	16.71	7.288e+06	16.71	N/A	{586.38640}	585.380
467	793.5150 / 16.77	Unknown	793.5150 / 16.77	Quantifiers	[No data for] Component Group Name	16.77	2.093e+07	16.85	N/A	{794.52168}	793.515
474	321.2420 / 16.88 [M-H]-	Unknown	321.2420 / 16.88 [M-H]-	Quantifiers	321.2420 / 16.88	16.88	1.789e+06	16.85	N/A	{322.24872}	321.242
477	601.3736 / 16.94	Unknown	601.3736 / 16.94	Quantifiers	[No data for] Component Group Name	16.94	2.681e+06	16.91	N/A	{602.38033}	601.374
480	853.4794 / 16.65 [M+Cl]-	Unknown	853.4794 / 16.65 [M+Cl]-	Qualifiers	817.5049 / 16.94	16.65	2.277e+06	16.63	N/A	{818.50944}	853.479
482	847.4942 / 16.99	Unknown	847.4942 / 16.99	Quantifiers	[No data for] Component Group Name	16.99	1.032e+07	16.97	N/A	{848.50090}	847.494
487	831.5051 / 17.16	Unknown	831.5051 / 17.16	Quantifiers	[No data for] Component Group Name	17.16	1.889e+06	17.61	N/A	{832.51185}	831.505
488	289.1796 / 17.22	Unknown	289.1796 / 17.22	Quantifiers	[No data for] Component Group Name	17.22	6.109e+05	17.25	N/A	{290.18629}	289.180
492	269.2123 / 17.28 [M-H]-	Unknown	269.2123 / 17.28 [M-H]-	Quantifiers	269.2123 / 17.28	17.28	3.160e+06	17.27	N/A	{270.21901}	269.212
494	365.2708 / 17.28	Unknown	365.2708 / 17.28	Quantifiers	[No data for] Component Group Name	17.28	2.756e+06	17.28	N/A	{366.27756}	365.271
507	263.1999 / 17.50	Unknown	263.1999 / 17.50	Quantifiers	[No data for] Component Group Name	17.51	1.867e+05	17.49	N/A	{264.20665}	263.200

509	277.2273 / 17.79	Unknown	277.2273 / 17.79	Quantifiers	[No data for] Component Group Name	17.79	6.305e+06	17.79	N/A	{278.23405}	277.227
511	271.2357 / 18.02	Unknown	271.2357 / 18.02	Quantifiers	[No data for] Component Group Name	18.02	7.800e+06	18.01	N/A	{272.24243}	271.236
515	253.2175 / 18.13	Unknown	253.2175 / 18.13	Quantifiers	[No data for] Component Group Name	18.13	1.819e+06	18.14	N/A	{254.22424}	253.218
516	297.2422 / 18.13	Unknown	297.2422 / 18.13	Quantifiers	[No data for] Component Group Name	18.13	6.228e+05	18.11	N/A	{298.24895}	297.242
519	279.2338 / 18.30	Unknown	279.2338 / 18.30	Quantifiers	[No data for] Component Group Name	18.30	1.302e+06	18.29	N/A	{280.24054}	279.234
523	255.2415 / 18.58	Unknown	255.2415 / 18.58	Quantifiers	[No data for] Component Group Name	18.58	6.470e+06	18.57	N/A	{256.24826}	255.242
525	402.2987 / 18.70	Unknown	402.2987 / 18.70	Quantifiers	[No data for] Component Group Name	18.70	2.082e+05	18.67	N/A	{403.30543}	402.299
528	311.2930 / 18.81	Unknown	311.2930 / 18.81	Quantifiers	[No data for] Component Group Name	18.81	3.348e+05	18.83	N/A	{312.29968}	311.293
531	339.3261 / 18.93	Unknown	339.3261 / 18.93	Quantifiers	[No data for] Component Group Name	18.93	3.180e+06	18.94	N/A	{340.33288}	339.326
532	255.2392 / 19.10	Unknown	255.2392 / 19.10	Quantifiers	[No data for] Component Group Name	19.10	1.008e+07	19.09	N/A	{256.24595}	255.239
534	281.2476 / 19.15 [M-H2O-H]-	Unknown	281.2476 / 19.15 [M-H2O-H]-	Qualifiers	299.2583 / 19.04	19.15	1.707e+06	19.13	N/A	{300.26491}	281.248
538	355.3208 / 19.21	Unknown	355.3208 / 19.21	Quantifiers	[No data for] Component Group Name	19.21	8.799e+05	19.21	N/A	{356.32755}	355.321
542	765.5134 / 19.27	Unknown	765.5134 / 19.27	Quantifiers	[No data for] Component Group Name	19.27	5.704e+05	19.24	N/A	{766.52009}	765.513
550	831.5075 / 19.32	Unknown	831.5075 / 19.32	Quantifiers	[No data for] Component Group Name	19.32	4.896e+06	19.35	N/A	{832.51422}	831.507

#	Analyte Peak Name	Found At Mass	Library Hit	Library Score
2	128.0339 / 1.02	128.0340	DL-Pyroglutamic acid (NIST) [Smart Confirmation]	81.6
3	195.0497 / 1.02	195.0546	D-Gluconic acid (NIST) [Smart Confirmation]	89.1
6	117.0200 / 1.08	117.0205	丁二酸 Amber Acid [Smart Confirmation]	76.0

8	116.0704 / 1.14	116.0703	3-Aminopentanoic acid (NIST) [Smart Confirmation]	95.1
11	179.0550 / 1.19	179.0550	D-甘露糖 D-(+)-Mannose [Smart Confirmation]	97.4
13	341.1104 / 1.25	341.1100	Melibiose (NIST) [Smart Confirmation]	96.2
15	188.0915 / 1.37	188.0915	8-Hydroxyquinoline-5-carboxylic acid (NIST) [Smart Confirmation]	87.6
18	130.0883 / 1.42	130.0891	trans-4-Hydroxy-L-proline (NIST) [Smart Confirmation]	86.9
19	202.1073 / 1.42	202.1071	1-(2,8-Dihydroxyquinolin-5-yl)ethan-1-one (NIST) [Smart Confirmation]	24.1
20	243.0662 / 1.42	243.0666	尿苷 Uridine [Smart Confirmation]	73.6
26	282.0863 / 1.54	282.0867	乌苷guanosine [Smart Confirmation]	84.3
29	299.0810 / 1.59	299.0821	5-Nitro-2-(3-phenylpropylamino)benzoic acid (NIST) [Smart Confirmation]	27.1
32	175.0599 / 1.54 [M+FA-H]-	175.0600	2-Isopropylmalic acid (NIST) [Smart Confirmation]	86.1
34	134.0496 / 1.65	134.0498	腺嘌呤Adenine [Smart Confirmation]	97.9
38	206.0811 / 1.76	206.0812	N-Acetyl-L-phenylalanine (NIST) [Smart Confirmation]	98.0
43	218.1027 / 1.65 [M+AcO-H]-	218.1027	Pantothenic acid (NIST) [Smart Confirmation]	100.0
46	147.0445 / 1.93	147.0447	肉桂酸 cinnamic acid [Smart Confirmation]	32.6
49	164.0769 / 1.99	164.0775	苯丙氨酸Phenprobamate [Smart Confirmation]	96.6
52	415.1595 / 2.05	415.1597	大豆苷 Daidzin [Smart Confirmation]	91.9
57	192.0658 / 2.22	192.0658	4-(Butylamino)benzoic acid (NIST) [Smart Confirmation]	38.5
61	138.0543 / 2.39	138.0546	5-Amino-2-methoxyphenol (NIST) [Smart Confirmation]	100.0
63	401.1788 / 2.44	401.1793	Trandolaprilat (NIST) [Smart Confirmation]	50.1
66	307.1384 / 2.56	307.1380	Bisdemethoxycurcumin (NIST) [Smart Confirmation]	46.0
67	206.0809 / 2.62	206.0808	N-Acetyl-L-phenylalanine (NIST) [Smart Confirmation]	91.2
68	327.1064 / 2.62	327.1063	草夹竹桃苷 Androsin [Smart Confirmation]	44.6
69	153.0179 / 2.67	153.0180	2,6-Dihydroxybenzoic acid (NIST) [Smart Confirmation]	98.0
70	167.0330 / 2.67	167.0335	香草酸Vanillic acid [Smart Confirmation]	83.9
77	399.1664 / 2.90	399.1667	Methanone, 6-hydroxy-1-2-(4-morpholinyl)ethyl-1H-indol-3-yl-1-naphthalenyl- (NIST) [Smart Confirmation]	45.6
78	275.1125 / 3.07	275.1122	Stearidonic acid (NIST) [Smart Confirmation]	57.6
79	203.0886 / 3.13	203.0887	色氨酸 L-Tryptophan [Smart Confirmation]	98.6
80	397.1489 / 3.13	397.1488	6-3-(1-Adamantyl)-4-hydroxyphenyl-2-naphthalenecarboxylic acid (NIST) [Smart Confirmation]	69.3
83	121.0284 / 3.24	121.0285	Benzoic acid (NIST) [Smart Confirmation]	88.6
91	339.0703 / 4.83	339.0703	Esculin (NIST) [Smart Confirmation]	97.2
93	274.8785 / 5.00	274.8787	2-Hydroxy-5-bromobenzophenone (NIST) [Smart Confirmation]	29.5
98	281.1382 / 5.23	281.1381	7-Hydroxy-3-(4-methoxyphenyl)-4-methylcoumarin (NIST) [Smart Confirmation]	29.8
104	537.1247 / 5.80	537.1239	穗花杉双黄酮 Amentoflavone [Smart Confirmation]	62.5
106	401.1790 / 5.91	401.1790	Trandolaprilat (NIST) [Smart Confirmation]	30.4
108	421.1610 / 6.25	421.1617	Dioctyl sulfosuccinate (NIST) [Smart Confirmation]	50.8
109	431.1920 / 6.25	431.1919	Naphthofluorescein (NIST) [Smart Confirmation]	78.4
116	193.0488 / 6.71	193.0489	异阿魏酸 Isoferulic acid [Smart Confirmation]	97.8
117	949.3568 / 6.71	949.3565	Rebaudioside C (NIST) [Smart Confirmation]	26.8
119	345.1073 / 6.88	345.1073	银杏酸C15:1 Ginkgolic Acid (C15:1) [Smart Confirmation]	40.3
120	247.1536 / 6.93	247.1535	Asp-Asp (NIST) [Smart Confirmation]	89.2
122	551.2332 / 6.99	551.2335	远志口山酮Ⅸ PolygalaxanthoneⅨ [Smart Confirmation]	93.9

123	595.1299 / 7.05	595.1297	Peltatoside (NIST) [Smart Confirmation]	99.4
126	609.1444 / 7.28	609.1449	芦丁 Rutin [Smart Confirmation]	97.8
129	187.0957 / 7.45	187.0960	Azelaic acid (NIST) [Smart Confirmation]	76.9
132	361.2209 / 7.45	361.2206	Piretanide (NIST) [Smart Confirmation]	52.2
135	463.0886 / 7.50	463.0889	金丝桃苷 Hyperin [Smart Confirmation]	98.7
137	271.1533 / 7.62	271.1536	DL-.beta.-Hydroxypalmitic acid (NIST) [Smart Confirmation]	87.4
146	447.0963 / 7.96	447.0968	紫云英苷 Astragalin [Smart Confirmation]	99.1
147	187.1020 / 8.01	187.1029	Azelaic acid (NIST) [Smart Confirmation]	98.4
150	144.0446 / 8.13	144.0448	Quinolin-8-ol (NIST) [Smart Confirmation]	73.6
151	243.1328 / 8.13	243.1327	1,11-Undecanedicarboxylic acid (NIST) [Smart Confirmation]	27.1
153	231.1598 / 8.24	231.1598	(.+-.)-Camphor-10-sulfonic acid (NIST) [Smart Confirmation]	94.1
154	373.1857 / 8.24	373.1857	京尼平苷酸 Geniposidic acid [Smart Confirmation]	38.4
155	173.1191 / 8.36	173.1192	L-Theanine (NIST) [Smart Confirmation]	73.4
156	478.1970 / 8.36	478.1975	1-Oleoyl-sn-glycero-3-phosphoethanolamine (NIST) [Smart Confirmation]	21.6
158	381.1546 / 8.41 [M-H]-	381.1545	Ibuprofen .beta.-D-glucuronide (NIST) [Smart Confirmation]	25.8
159	417.1308 / 8.41 [M+Cl]-	417.1307	Farnesyl pyrophosphate (NIST) [Smart Confirmation]	39.7
160	343.2128 / 8.53	343.2146	19(20)-Epoxy-4Z,7Z,10Z,13Z,16Z-docosapentaenoic acid (NIST) [Smart Confirmation]	42.4
163	113.0595 / 8.64	113.0595	Methimazole (NIST) [Smart Confirmation]	34.7
171	665.3546 / 8.92	665.3546	1-(1,2-Dioctanoylphosphatidyl)inositol-5-phosphate (NIST) [Smart Confirmation]	87.7
173	263.1303 / 9.09	263.1304	(+)-Abscisic acid (NIST) [Smart Confirmation]	97.7
177	329.1591 / 9.38	329.1592	cis-7,10,13,16,19-Docosapentaenoic acid (NIST) [Smart Confirmation]	64.6
180	147.0443 / 9.49	147.0443	肉桂酸 cinnamic acid [Smart Confirmation]	99.5
185	225.1128 / 9.78	225.1133	Benzoic acid, 4-benzoyl (NIST) [Smart Confirmation]	46.3
190	745.3439 / 9.78	745.3442	1-palmitoyl-2-linoleoyl-sn-glycero-3-phospho-(1'-rac-glycerol) (NIST) [Smart Confirmation]	36.0
193	213.1116 / 9.89	213.1118	p-Bromophenylacetic acid (NIST) [Smart Confirmation]	82.9
197	777.4173 / 9.95 [2M-H]-	777.4174	太子参环肽B Heterophyllin B [Smart Confirmation]	20.3
203	609.2714 / 10.17	609.2715	1-(1,2-Dihexanoylphosphatidyl)inositol-5-phosphate (NIST) [Smart Confirmation]	69.5
210	593.2624 / 10.34	593.2625	2',6'-Dihydroxy-4-methoxychalcone-4'-O-neohesperidoside (NIST) [Smart Confirmation]	20.2
217	645.3626 / 10.51	645.3624	N-Lauroyl-D-erythro-sphingosylphosphorylcholine (NIST) [Smart Confirmation]	23.2
226	388.2046 / 10.69	388.2049	Epiprogoitrin (NIST) [Smart Confirmation]	60.3
230	777.4202 / 10.69	777.4197	太子参环肽B Heterophyllin B [Smart Confirmation]	42.3
239	227.1271 / 10.91	227.1273	Butanedioic acid, 2-(4,4-dimethyl-2-methylenepentyl)- (NIST) [Smart Confirmation]	99.1
241	329.2314 / 10.91	329.2318	4,2'-Dihydroxy-3,4',6'-trimethoxychalcone (NIST) [Smart Confirmation]	68.2
247	811.4116 / 11.03	811.4124	柴胡皂苷B4 Saikosaponin B4 [Smart Confirmation]	92.7
250	527.3354 / 11.20	527.3352	茯苓酸 Pachymic acid [Smart Confirmation]	98.7
255	287.2205 / 11.25	287.2204	Ciprofibrate (NIST) [Smart Confirmation]	80.9
258	195.1373 / 11.37	195.1372	1,3-Dimethyluric acid (NIST) [Smart Confirmation]	63.8
259	239.1271 / 11.42	239.1272	Picloram (NIST) [Smart Confirmation]	71.3
262	679.3694 / 11.42	679.3696	Dipyridamole mono-O-.beta.-D-glucuronide (NIST) [Smart Confirmation]	46.3
272	669.3755 / 11.59	669.3754	Monensin (NIST) [Smart Confirmation]	73.8
275	329.2308 / 11.65	329.2309	4,2'-Dihydroxy-3,4',6'-trimethoxychalcone (NIST) [Smart Confirmation]	48.9
294	577.2671 / 12.39	577.2674	Isorhoifolin (NIST) [Smart Confirmation]	21.1

302	669.3763 / 12.73	669.3764	Monensin (NIST) [Smart Confirmation]	20.6
303	679.4159 / 12.73	679.4205	Dipyridamole mono-O-.beta.-D-glucuronide (NIST) [Smart Confirmation]	31.5
309	309.2051 / 12.84	309.2052	13-Hydroperoxy-9Z,11E,15Z-octadecatrienoic acid (NIST) [Smart Confirmation]	26.4
325	601.3735 / 13.19	601.3736	Garcinol (NIST) [Smart Confirmation]	62.3
329	571.2882 / 13.24	571.2884	1-Hexadecanoyl-sn-glycero-3-phospho-(1'-myo-inositol) (NIST) [Smart Confirmation]	72.0
348	209.1156 / 13.70	209.1163	Jasmonic acid (NIST) [Smart Confirmation]	79.0
354	581.2985 / 13.87	581.2981	Naringin dihydrochalcone (NIST) [Smart Confirmation]	58.2
355	483.2768 / 13.92	483.2785	1-Palmitoyl-2-hydroxy-sn-glycero-3-phospho-(1'-rac-glycerol) (NIST) [Smart Confirmation]	99.1
362	785.4693 / 14.09	785.4691	Echinacoside (NIST) [Smart Confirmation]	23.8
367	509.2888 / 14.27	509.2892	1-Oleoyl-2-hydroxy-sn-glycero-3-phospho-(1'-rac-glycerol) (NIST) [Smart Confirmation]	98.6
376	309.2044 / 14.49	309.2046	13S-Hydroperoxy-6Z,9Z,11E-octadecatrienoic acid (NIST) [Smart Confirmation]	91.8
378	695.4022 / 14.21 [M+AcO-H]-	695.4021	长梗冬青苷 Pedunculoside +HCOOH [Smart Confirmation]	92.4
380	279.1941 / 14.55	279.1943	亚油酸 Linoleic acid [Smart Confirmation]	100.0
383	599.3187 / 14.61	599.3190	1-Octadecanoyl-sn-glycero-3-phospho-(1'-myo-inositol) (NIST) [Smart Confirmation]	48.6
387	601.3718 / 14.72	601.3720	Garcinol (NIST) [Smart Confirmation]	73.5
391	291.1955 / 14.83	291.1956	3-Bromo-5-phenylsalicylic acid (NIST) [Smart Confirmation]	36.6
399	365.2676 / 15.00	365.2675	cis-15-Tetracosenoic acid (NIST) [Smart Confirmation]	79.1
405	391.2226 / 15.06	391.2228	1-Hexadecanoyl-sn-glycero-2,3-cyclic-phosphate (NIST) [Smart Confirmation]	78.0
407	517.3546 / 15.12	517.3551	Gossypol (NIST) [Smart Confirmation]	47.4
411	275.2002 / 15.17 [M-H2O-H]-	275.2000	Stearidonic acid (NIST) [Smart Confirmation]	71.1
412	293.2112 / 15.74 [M-H]-	293.2230	13S-Hydroxy-9Z,11E,15Z-octadecatrienoic acid (NIST) [Smart Confirmation]	97.8
413	865.5004 / 15.23	865.5005	1,2-Dioctadecanoyl-sn-glycero-3-phospho-(1'-myo-inositol) (NIST) [Smart Confirmation]	25.8
414	295.2166 / 15.35	295.2165	4-Hydroxycyclofenil (NIST) [Smart Confirmation]	20.3
417	293.2237 / 15.40	293.2230	13S-Hydroxy-9Z,11E,15Z-octadecatrienoic acid (NIST) [Smart Confirmation]	97.8
418	487.3412 / 15.40	487.3415	积雪草酸 Asiatic acid [Smart Confirmation]	100.0
419	511.3052 / 15.40	511.3058	1-Octadecanoyl-sn-glycero-3-phospho-(1'-sn-glycerol) (NIST) [Smart Confirmation]	76.2
427	601.3741 / 15.80 [M-H2O-H]-	601.3743	Garcinol (NIST) [Smart Confirmation]	24.3
437	291.1983 / 15.97	291.1993	Monastrol (NIST) [Smart Confirmation]	88.1
440	699.3816 / 16.08	699.3814	拟人参皂苷RT5 Pseudoginsenoside-RT5 +HCOOH [Smart Confirmation]	34.2
441	295.2357 / 16.14	295.2358	13R-Hydroxy-9Z,11E-octadecadienoic acid (NIST) [Smart Confirmation]	97.1
442	517.3611 / 16.14	517.3639	西伯利亚远志糖A5Sibiricose A5 [Smart Confirmation]	61.7
455	573.3777 / 16.77 [M+AcO-H]-	573.3781	川楝素 Toosendanin [Smart Confirmation]	35.1
457	293.2188 / 16.60	293.2202	13-Keto-9Z,11E-octadecadienoic acid (NIST) [Smart Confirmation]	98.2
461	505.2998 / 16.71	505.2998	Quercetin 3-O-.beta.-D-glucose-6'-acetate (NIST) [Smart Confirmation]	20.3
462	585.3797 / 16.71	585.3798	1-(1,2-Dioctanoylphosphatidyl)inositol (NIST) [Smart Confirmation]	76.1
467	793.5150 / 16.77	793.5156	竹节参皂苷IVa Chikusetsusponin IVa [Smart Confirmation]	100.0
474	321.2420 / 16.88 [M-H]-	321.2421	8,11-Tridecadienoic acid, 13-(3-pentyl-2-oxiranyl)-, (8Z,11Z)- (NIST) [Smart Confirmation]	68.0
477	601.3736 / 16.94	601.3738	Garcinol (NIST) [Smart Confirmation]	88.8
480	853.4794 / 16.65 [M+Cl]-	853.4794	重楼皂苷Ⅰ PolyphyllinⅠ [Smart Confirmation]	32.3
482	847.4942 / 16.99	847.4948	G1F1,6 (NIST) [Smart Confirmation]	34.6
487	831.5051 / 17.16	831.5045	B-Pentasaccharide (NIST) [Smart Confirmation]	87.7

488	289.1796 / 17.22	289.1795	表儿茶素 Epicatechin [Smart Confirmation]	30.9
492	269.2123 / 17.28 [M-H]-	269.2129	Lucidin (NIST) [Smart Confirmation]	79.6
494	365.2708 / 17.28	365.2710	17-Phenyltrinor-8-iso-prostaglandin E2 (NIST) [Smart Confirmation]	88.9
507	263.1999 / 17.50	263.1999	Asp-Met (NIST) [Smart Confirmation]	57.3
509	277.2273 / 17.79	277.2284	Pinolenic acid (NIST) [Smart Confirmation]	97.1
511	271.2357 / 18.02	271.2354	16-Hydroxyhexadecanoic acid (NIST) [Smart Confirmation]	85.6
515	253.2175 / 18.13	253.2181	.DELTA.2-cis-Hexadecenoic acid (NIST) [Smart Confirmation]	97.3
516	297.2422 / 18.13	297.2425	3-Oxostearic acid (NIST) [Smart Confirmation]	78.8
519	279.2338 / 18.30	279.2341	亚油酸 Linoleic acid [Smart Confirmation]	100.0
523	255.2415 / 18.58	255.2448	2,2',4'-Trihydroxychalcone (NIST) [Smart Confirmation]	69.2
525	402.2987 / 18.70	402.2988	N-Oleoyl-N-methyltaurine (NIST) [Smart Confirmation]	22.8
528	311.2930 / 18.81	311.2932	Benzenesulfonic acid, 4-undecyl- (NIST) [Smart Confirmation]	65.2
531	339.3261 / 18.93	339.3265	15S-Hydroperoxy-11Z,13E-eicosadienoic acid (NIST) [Smart Confirmation]	85.3
532	255.2392 / 19.10	255.2401	2,2',4'-Trihydroxychalcone (NIST) [Smart Confirmation]	69.5
534	281.2476 / 19.15 [M-H2O-H]-	281.2477	1,4-D-Xylobiose (NIST) [Smart Confirmation]	70.9
538	355.3208 / 19.21	355.3208	9S,11R-Dihydroxy-15-oxoprostanoic acid (NIST) [Smart Confirmation]	80.5
542	765.5134 / 19.27	765.5132	1,2-Dilinolenoyl-sn-glycero-3-phospho-(1'-rac-glycerol) (NIST) [Smart Confirmation]	87.1
550	831.5075 / 19.32	831.5087	B-Pentasaccharide (NIST) [Smart Confirmation]	20.1

End of Table

128.0339 / 1.02 (Mass/FragMass/RT/Isotope/Library/Formula/Ion Ratio)

Retention Time: 1.02 minutes
Precursor m/z : 128.0339
Fit (%) 100.0% **RFit (%)** 81.6%

Exp RT: 1.02 minutes
Analyte Name:
128.0339 / 1.02

Collision Energy = 35 ± 15 eV

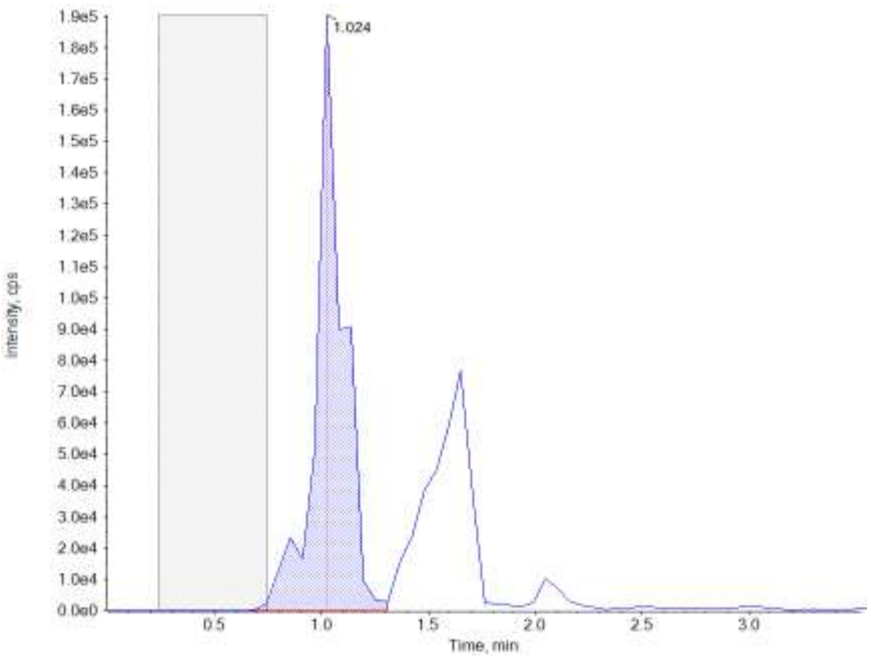

Acquired / Library MSMS

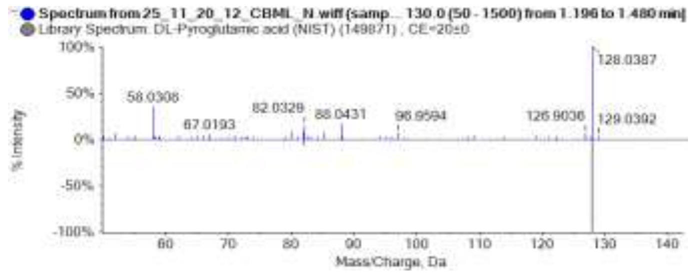

Acquired / Theoretical MS

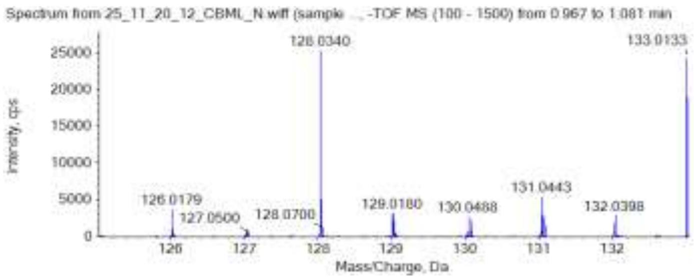

195.0497 / 1.02 (Mass/FragMass/RT/Isotope/Library/Formula/Ion Ratio)

**Retention Time:** 0.90 minutes<br>**Precursor m/z :** 195.0497<br>**Fit (%)** 100.0% **RFit (%)** 89.1%	**Exp RT:** 1.02 minutes<br>**Analyte Name:**<br>195.0497 / 1.02

Collision Energy = 35 ± 15 eV

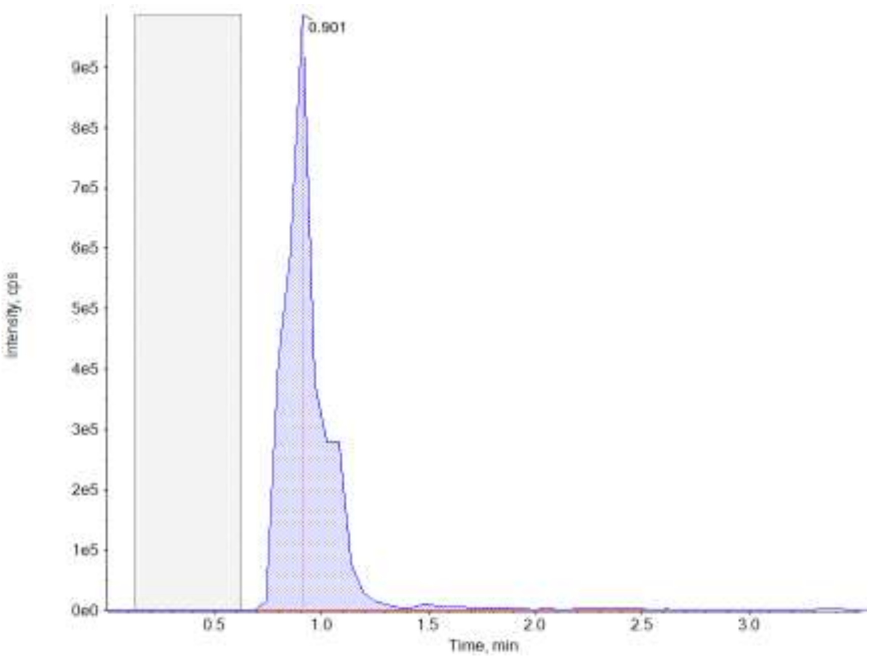

Acquired / Library MSMS

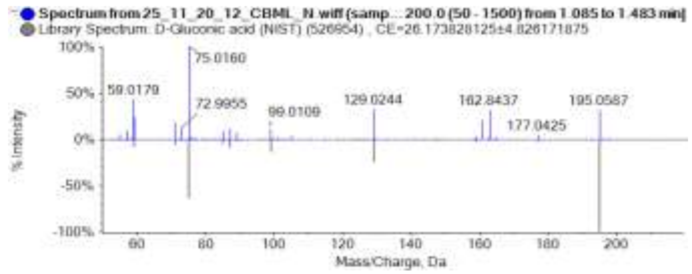

Acquired / Theoretical MS

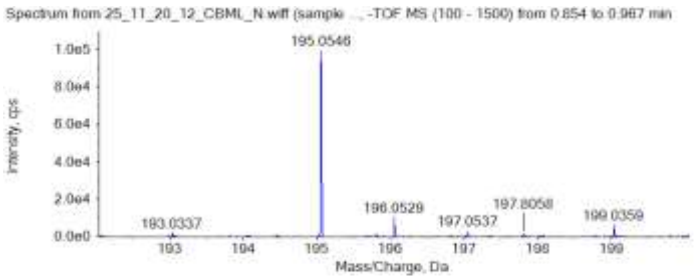

117.0200 / 1.08 (Mass/FragMass/RT/Isotope/Library/Formula/Ion Ratio)

Retention Time: 1.08 minutes
Precursor m/z : 117.0200
Fit (%) 99.6% **RFit (%)** 77.9%

Exp RT: 1.08 minutes
Analyte Name:
117.0200 / 1.08

Collision Energy = 35 ± 15 eV

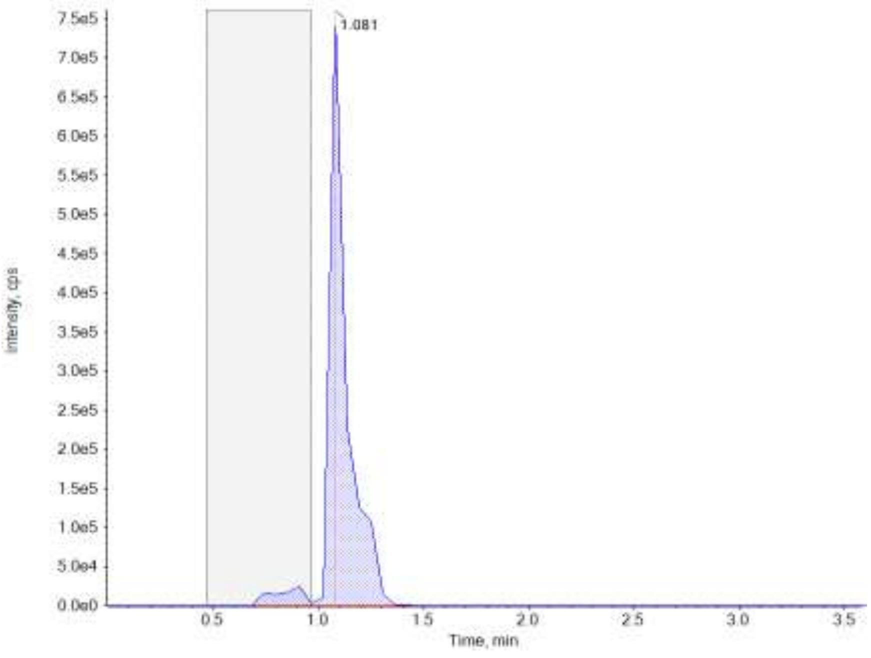

Acquired / Library MSMS

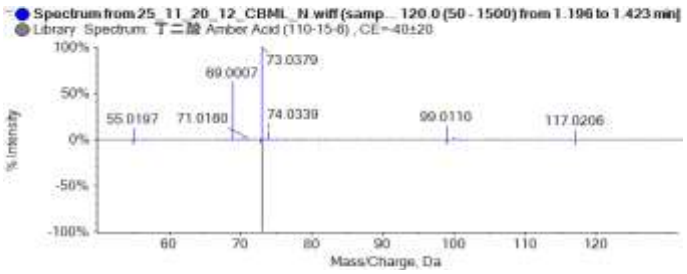

Acquired / Theoretical MS

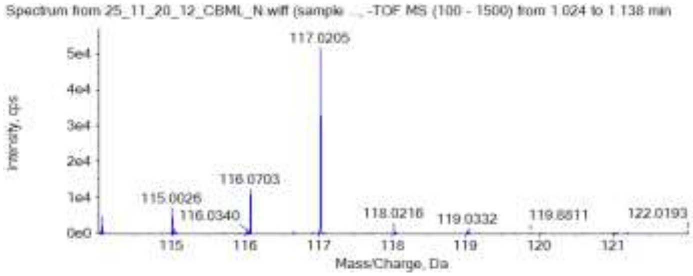

116.0704 / 1.14 (Mass/FragMass/RT/Isotope/Library/Formula/Ion Ratio)

Retention Time: 1.22 minutes
Precursor m/z : 116.0704
Fit (%) 97.9% **RFit (%)** 98.0%

Exp RT: 1.14 minutes
Analyte Name:
116.0704 / 1.14

Collision Energy = 35 ± 15 eV

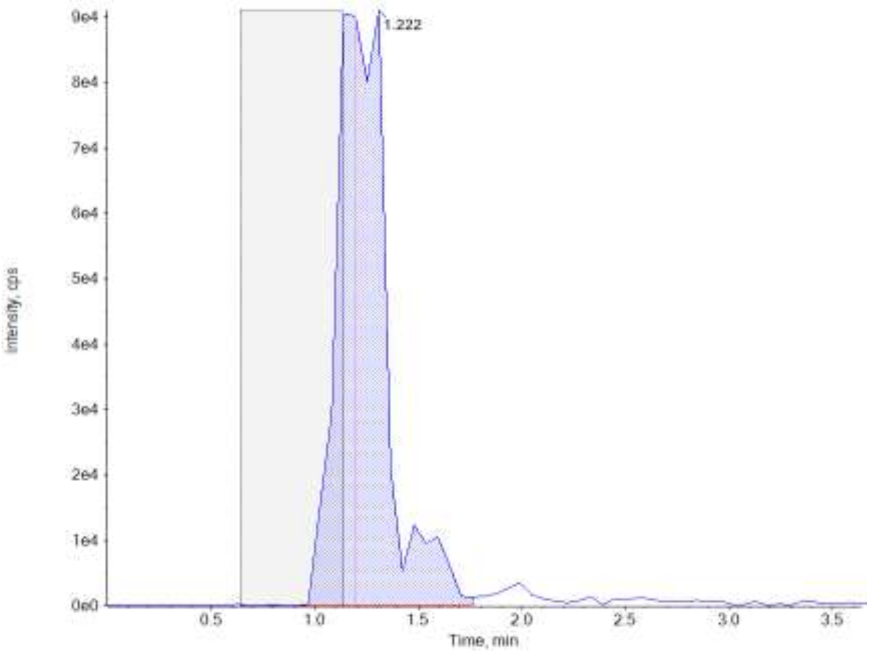

Acquired / Library MSMS

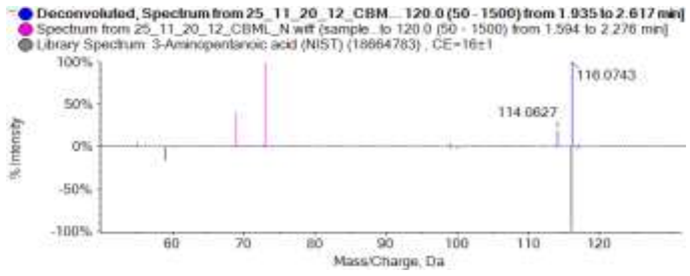

Acquired / Theoretical MS

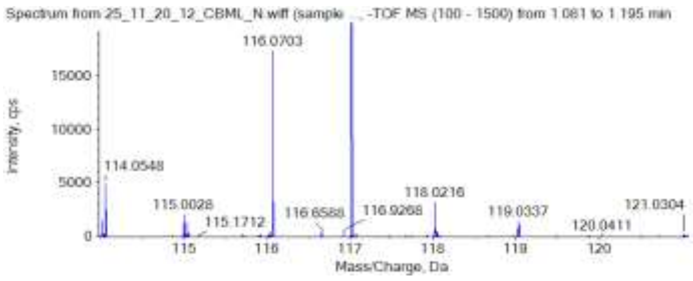

179.0550 / 1.19 (Mass/FragMass/RT/Isotope/Library/Formula/Ion Ratio)

Retention Time: 1.18 minutes
Precursor m/z : 179.0550
Fit (%) 98.1% **RFit (%)** 99.4%

Exp RT: 1.20 minutes
Analyte Name:
179.0550 / 1.19

Collision Energy = 35 ± 15 eV

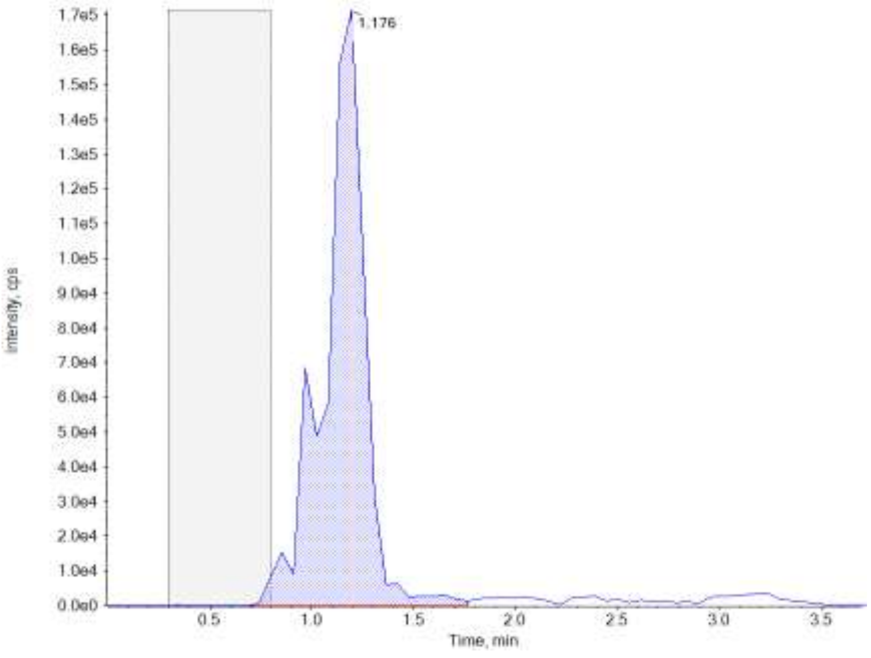

Acquired / Library MSMS

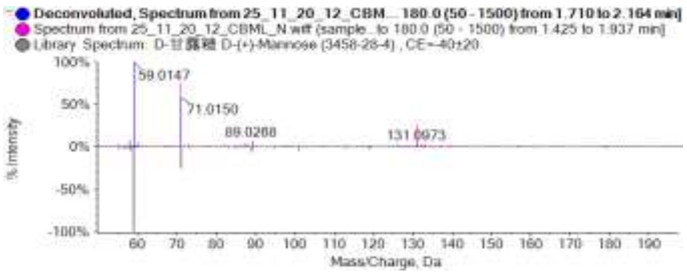

Acquired / Theoretical MS

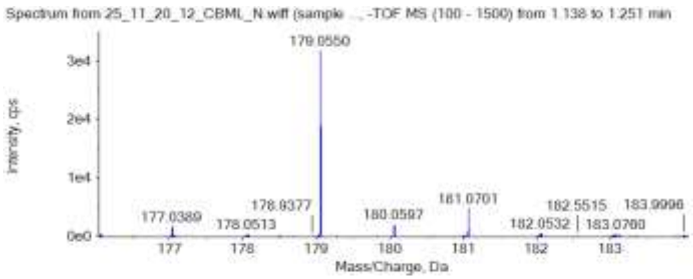

341.1104 / 1.25 (Mass/FragMass/RT/Isotope/Library/Formula/Ion Ratio)

Retention Time: 1.20 minutes
Precursor m/z : 341.1104
Fit (%) 100.0% **RFit (%)** 96.2%

Exp RT: 1.25 minutes
Analyte Name:
341.1104 / 1.25

Collision Energy = 35 ± 15 eV

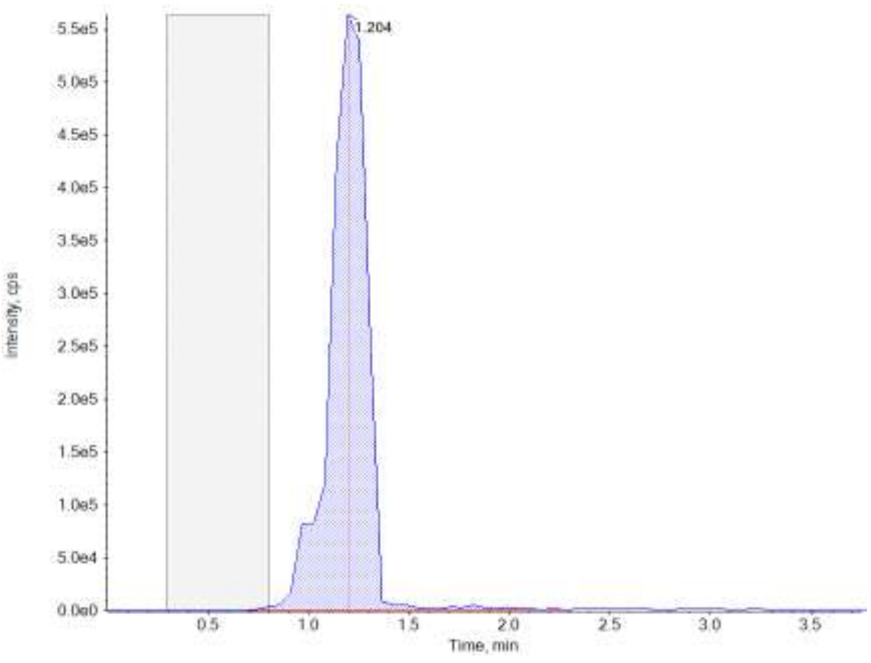

Acquired / Library MSMS

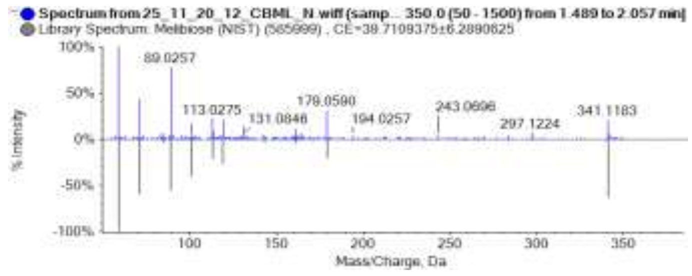

Acquired / Theoretical MS

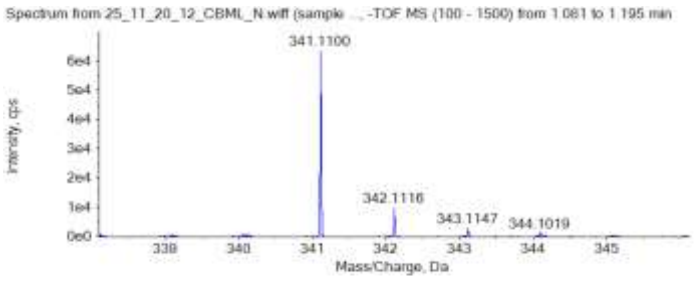

Created with SCIEX OS 3.3.0
Printed:12/9/2025 2:25:51 PM

188.0915 / 1.37 (Mass/FragMass/RT/Isotope/Library/Formula/Ion Ratio)

Retention Time: 1.30 minutes
Precursor m/z : 188.0915
Fit (%) 100.0% **RFit (%)** 87.6%

Exp RT: 1.37 minutes
Analyte Name:
188.0915 / 1.37

Collision Energy = 35 ± 15 eV

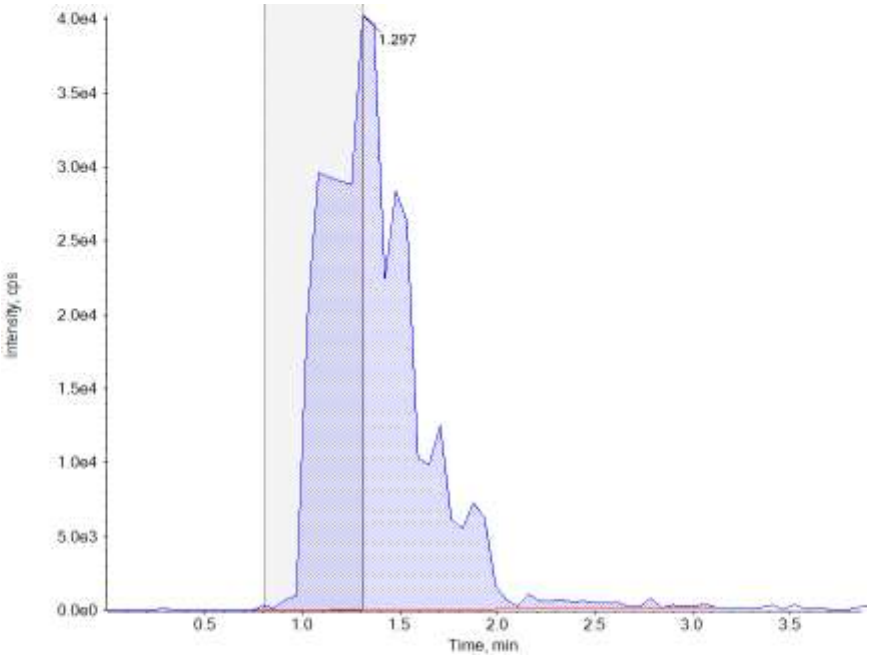

Acquired / Library MSMS

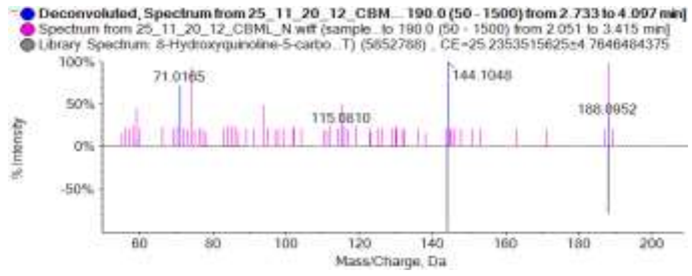

Acquired / Theoretical MS

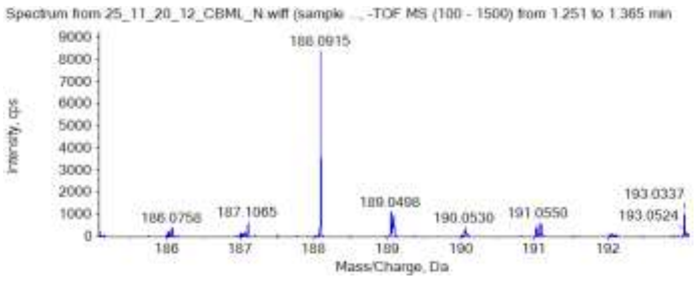

Created with SCIEX OS 3.3.0
Printed:12/9/2025 2:25:51 PM

130.0883 / 1.42 (Mass/FragMass/RT/Isotope/Library/Formula/Ion Ratio)

**Retention Time:** 1.42 minutes	**Exp RT:** 1.42 minutes
**Precursor m/z :** 130.0883	**Analyte Name:**
**Fit (%)** 96.3% **RFit (%)** 94.5%	130.0883 / 1.42

Collision Energy = 35 ± 15 eV

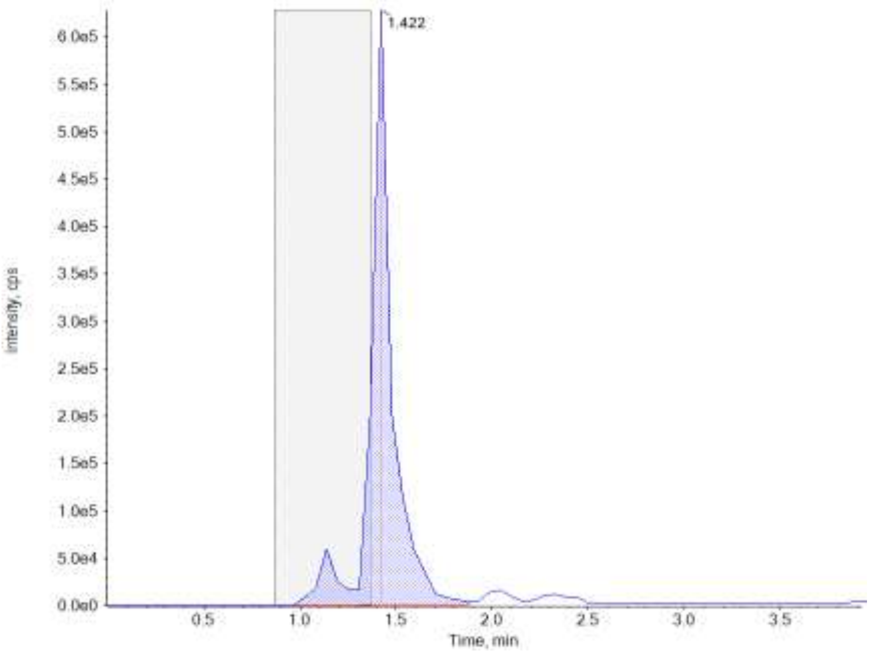

Acquired / Library MSMS

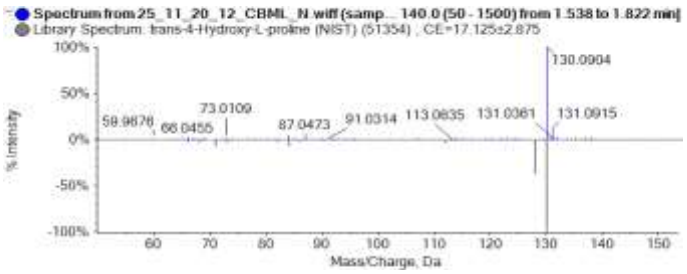

Acquired / Theoretical MS

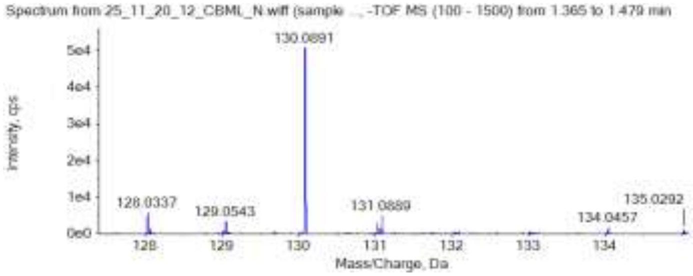

202.1073 / 1.42 (Mass/FragMass/RT/Isotope/Library/Formula/Ion Ratio)

Retention Time: 1.44 minutes
Precursor m/z : 202.1073
Fit (%) 83.3% **RFit (%)** 51.7%

Exp RT: 1.42 minutes
Analyte Name:
202.1073 / 1.42

Collision Energy = 35 ± 15 eV

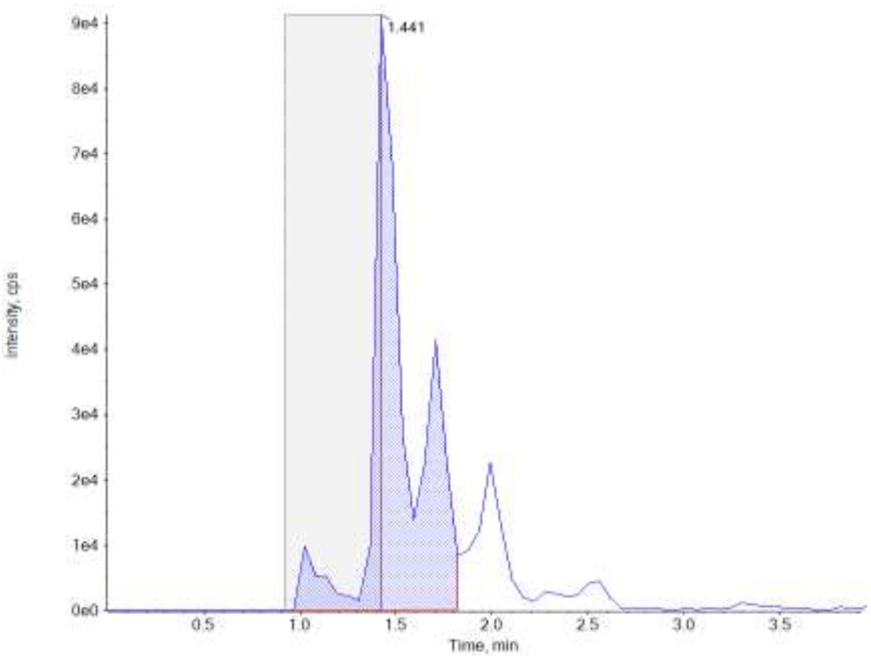

Acquired / Library MSMS

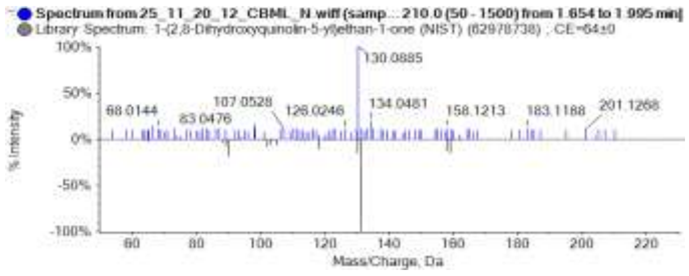

Acquired / Theoretical MS

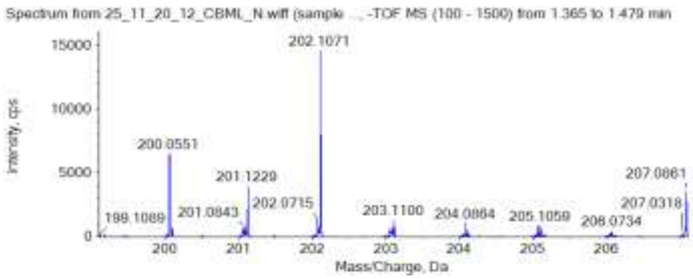

243.0662 / 1.42 (Mass/FragMass/RT/Isotope/Library/Formula/Ion Ratio)

Retention Time: 1.42 minutes
Precursor m/z : 243.0662
Fit (%) 83.9% **RFit (%)** 97.0%

Exp RT: 1.42 minutes
Analyte Name:
243.0662 / 1.42

Collision Energy = 35 ± 15 eV

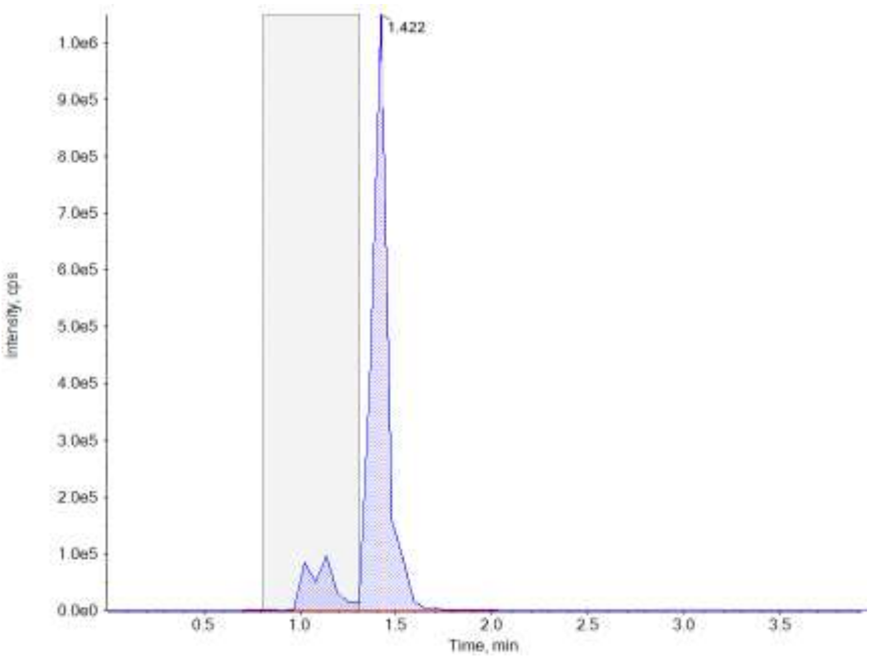

Acquired / Library MSMS

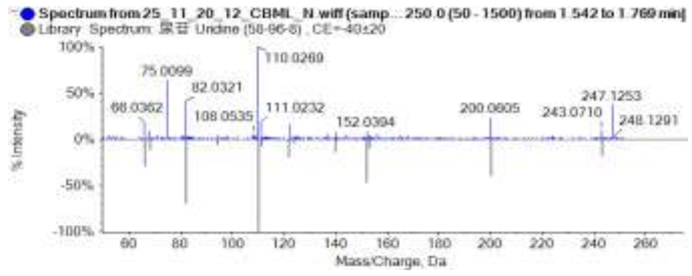

Acquired / Theoretical MS

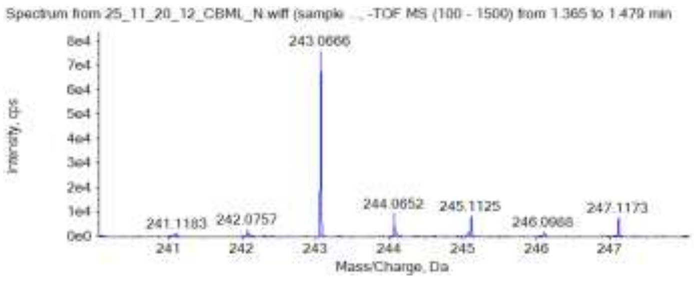

282.0863 / 1.54 (Mass/FragMass/RT/Isotope/Library/Formula/Ion Ratio)

Retention Time: 1.51 minutes
Precursor m/z : 282.0863
Fit (%) 100.0% **RFit (%)** 84.3%

Exp RT: 1.54 minutes
Analyte Name:
282.0863 / 1.54

Collision Energy = 35 ± 15 eV

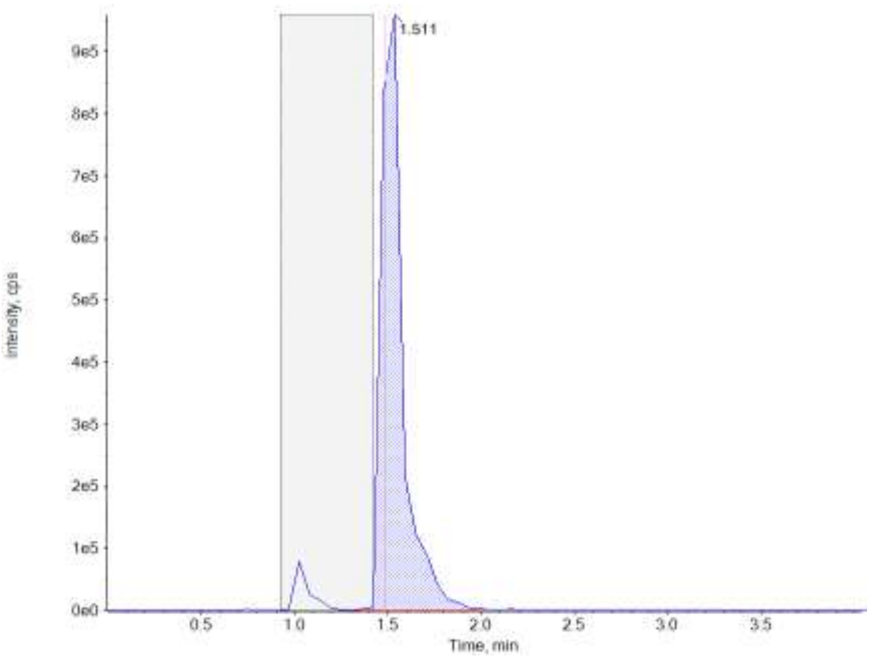

Acquired / Library MSMS

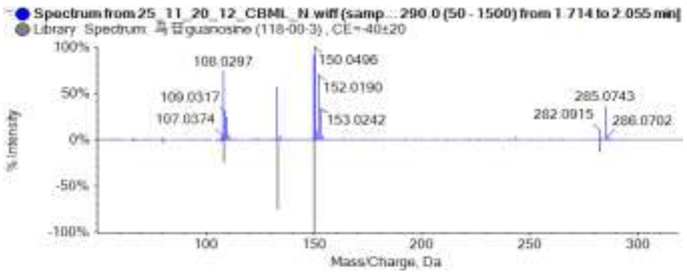

Acquired / Theoretical MS

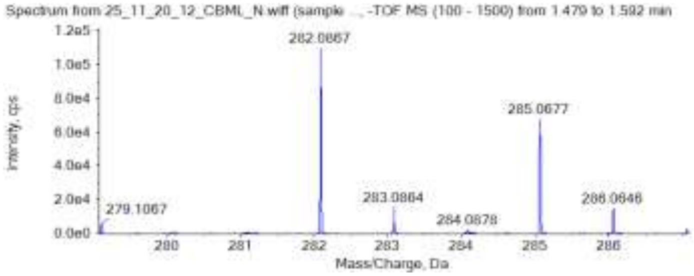

299.0810 / 1.59 (Mass/FragMass/RT/Isotope/Library/Formula/Ion Ratio)

Retention Time: 1.59 minutes
Precursor m/z : 299.0810
Fit (%) 46.0% **RFit (%)** 93.2%

Exp RT: 1.59 minutes
Analyte Name:
299.0810 / 1.59

Collision Energy = 35 ± 15 eV

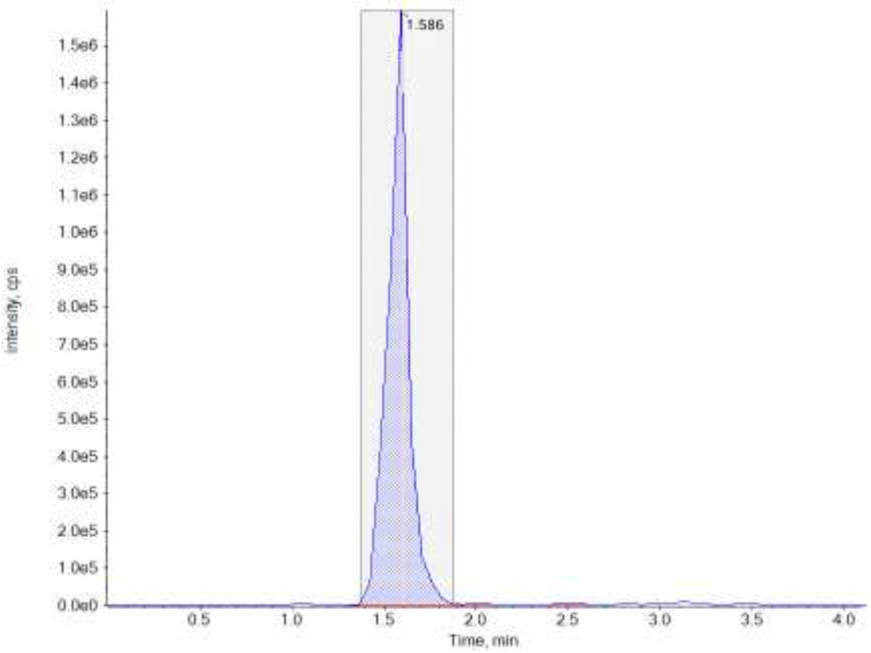

Acquired / Library MSMS

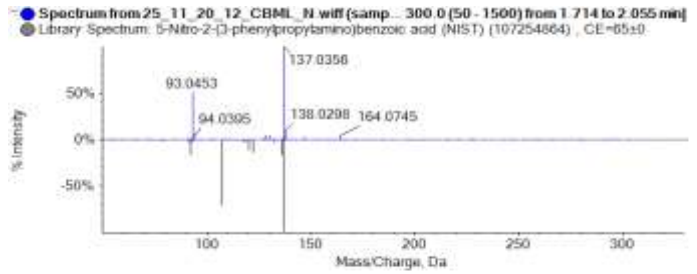

Acquired / Theoretical MS

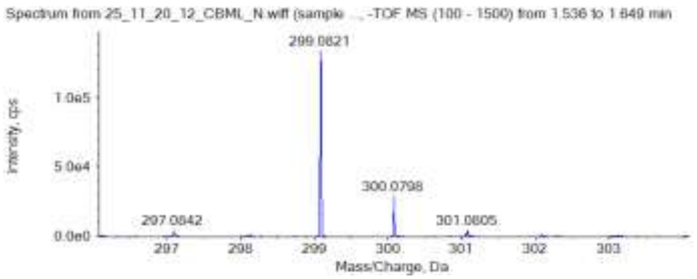

175.0599 / 1.54 [M+FA-H]- (Mass/FragMass/RT/Isotope/Library/Formula/Ion Ratio)

**Retention Time:** 1.54 minutes	**Exp RT:** 1.54 minutes
**Precursor m/z :** 175.0599	**Analyte Name:**
**Fit (%)** 98.1% **RFit (%)** 91.1%	175.0599 / 1.54 [M+FA-H]-

Collision Energy = 35 ± 15 eV

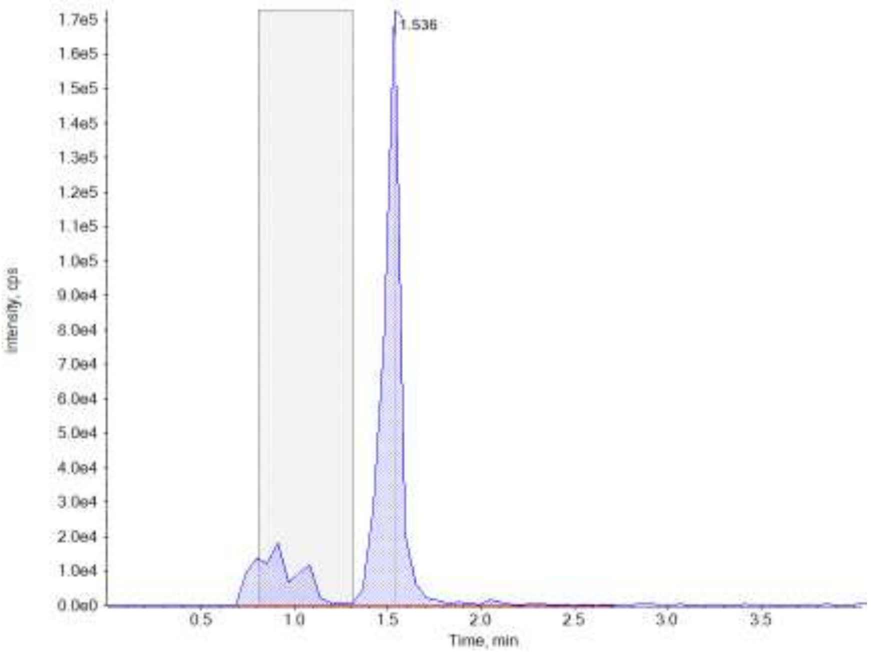

Acquired / Library MSMS

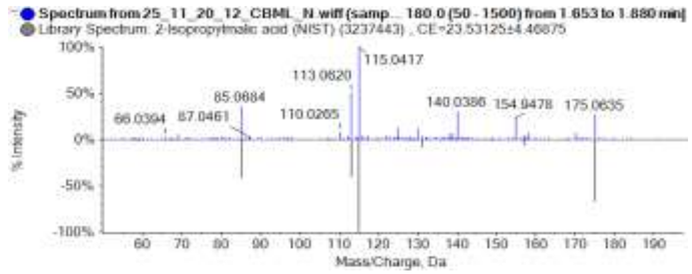

Acquired / Theoretical MS

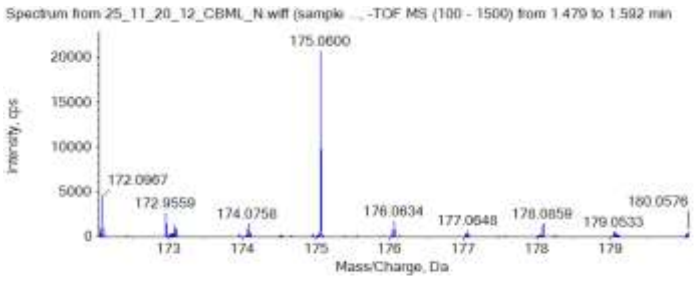

134.0496 / 1.65 (Mass/FragMass/RT/Isotope/Library/Formula/Ion Ratio)

Retention Time: 1.67 minutes | **Exp RT:** 1.65 minutes
Precursor m/z : 134.0496 | **Analyte Name:**
Fit (%) 97.9% **RFit (%)** 100.0% | 134.0496 / 1.65

Collision Energy = 35 ± 15 eV

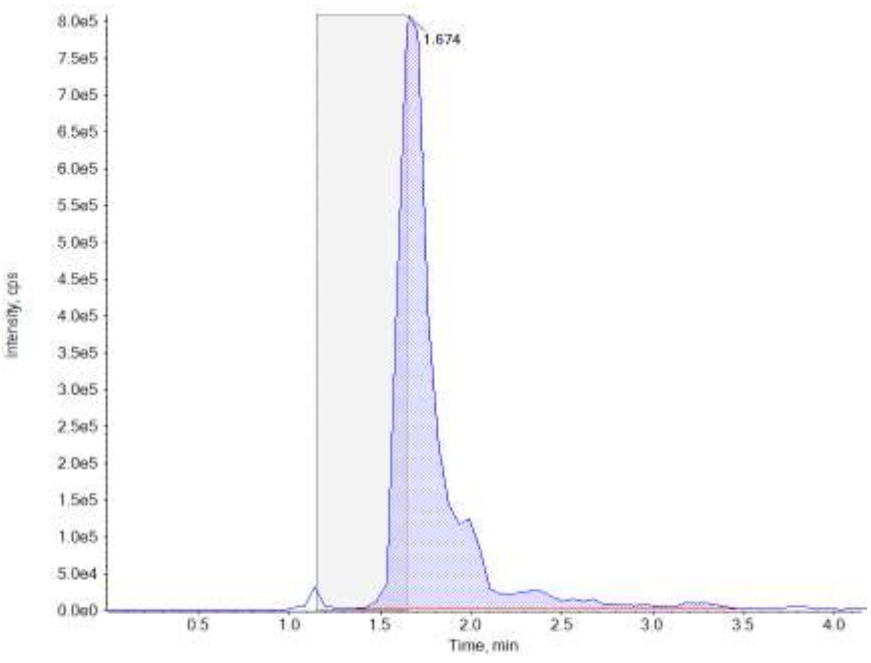

Acquired / Library MSMS

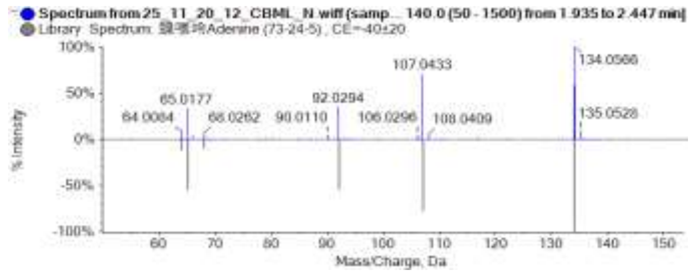

Acquired / Theoretical MS

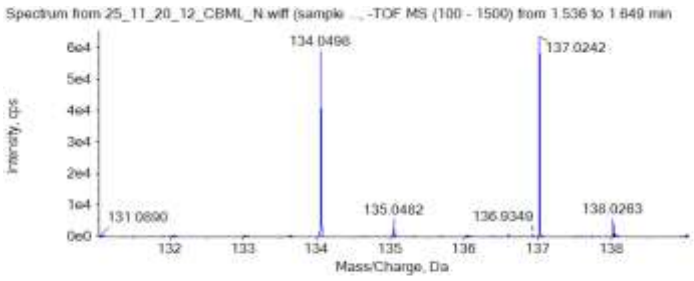

206.0811 / 1.76 (Mass/FragMass/RT/Isotope/Library/Formula/Ion Ratio)

Retention Time: 1.73 minutes
Precursor m/z : 206.0811
Fit (%) 99.7% **RFit (%)** 98.4%

Exp RT: 1.76 minutes
Analyte Name:
206.0811 / 1.76

Collision Energy = 35 ± 15 eV

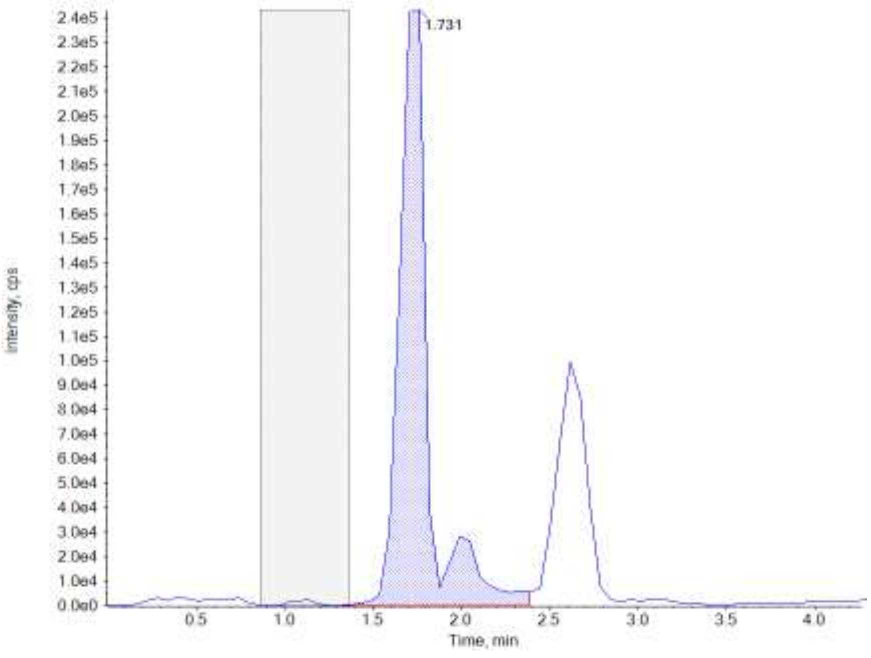

Acquired / Library MSMS

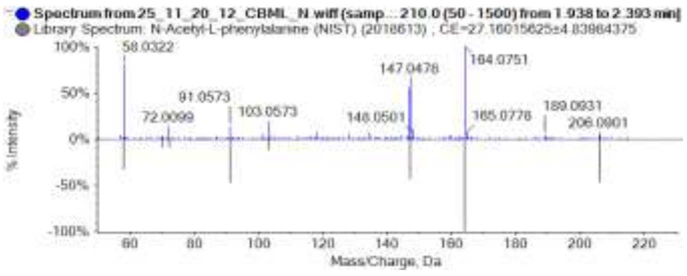

Acquired / Theoretical MS

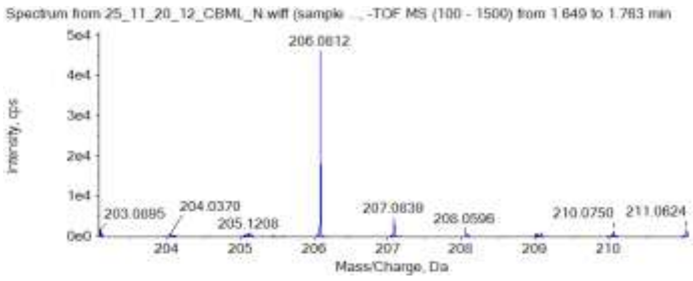

218.1027 / 1.65 [M+AcO-H]- (Mass/FragMass/RT/Isotope/Library/Formula/Ion Ratio)

Retention Time: 1.65 minutes
Precursor m/z : 218.1027
Fit (%) 100.0% **RFit (%)** 100.0%

Exp RT: 1.65 minutes
Analyte Name:
218.1027 / 1.65 [M+AcO-H]-

Collision Energy = 35 ± 15 eV

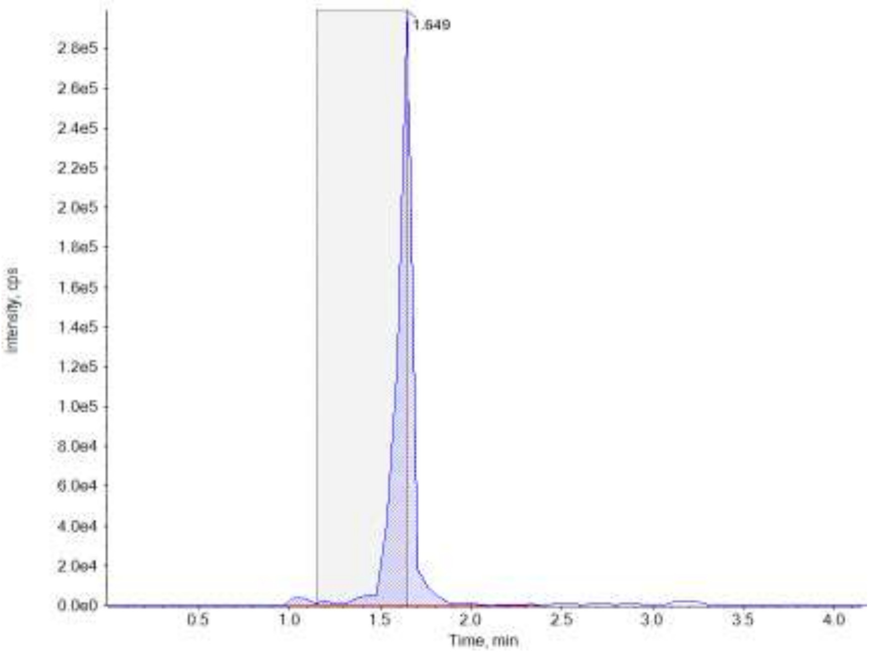

Acquired / Library MSMS

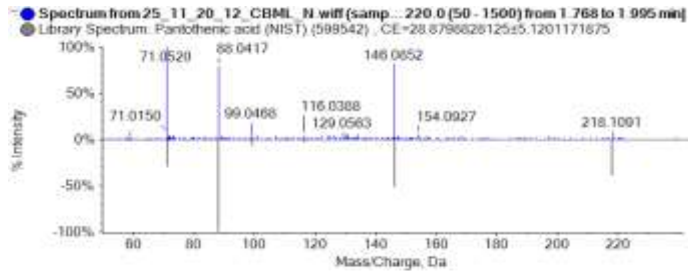

Acquired / Theoretical MS

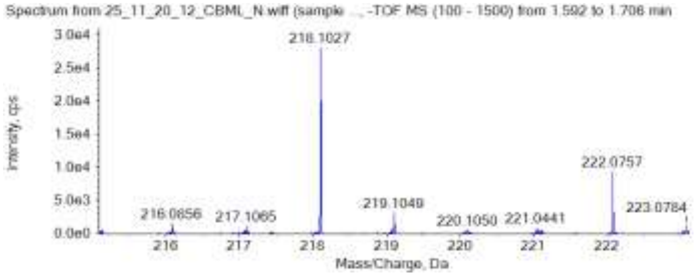

Created with SCIEX OS 3.3.0
Printed:12/9/2025 2:25:51 PM

147.0445 / 1.93 (Mass/FragMass/RT/Isotope/Library/Formula/Ion Ratio)

Retention Time: 1.95 minutes **Exp RT:** 1.93 minutes
Precursor m/z : 147.0445 **Analyte Name:**
Fit (%) 32.6% **RFit (%)** 100.0% 147.0445 / 1.93

Collision Energy = 35 ± 15 eV

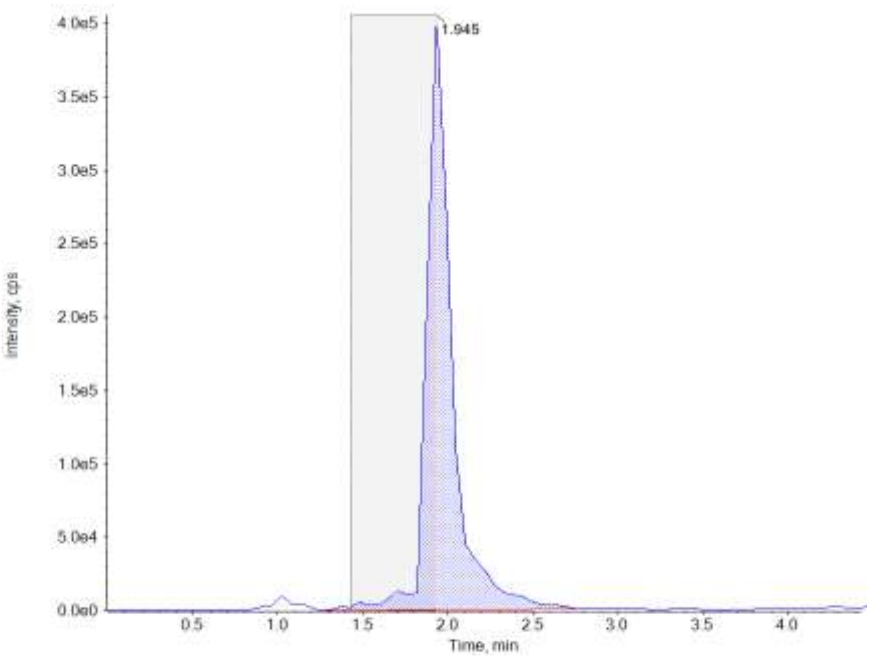

Acquired / Library MSMS

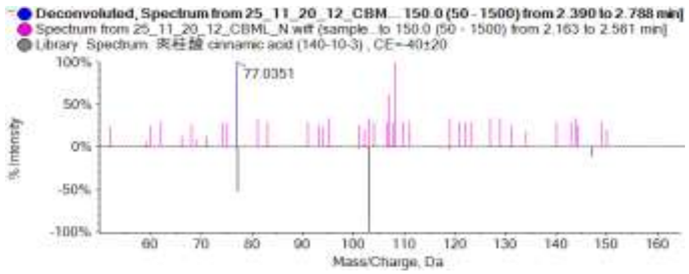

Acquired / Theoretical MS

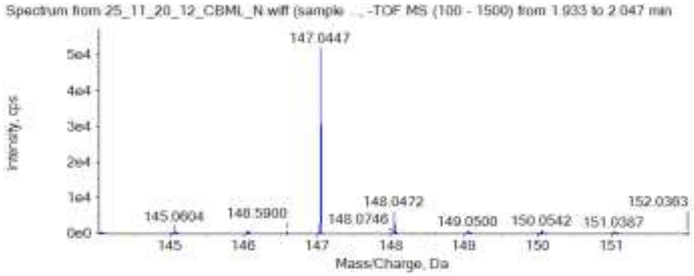

164.0769 / 1.99 (Mass/FragMass/RT/Isotope/Library/Formula/Ion Ratio)

**Retention Time:** 1.95 minutes<br>**Precursor m/z :** 164.0769<br>**Fit (%)** 100.0% **RFit (%)** 96.6%	**Exp RT:** 1.99 minutes<br>**Analyte Name:**<br>164.0769 / 1.99

	**Collision Energy = 35 ± 15 eV**	
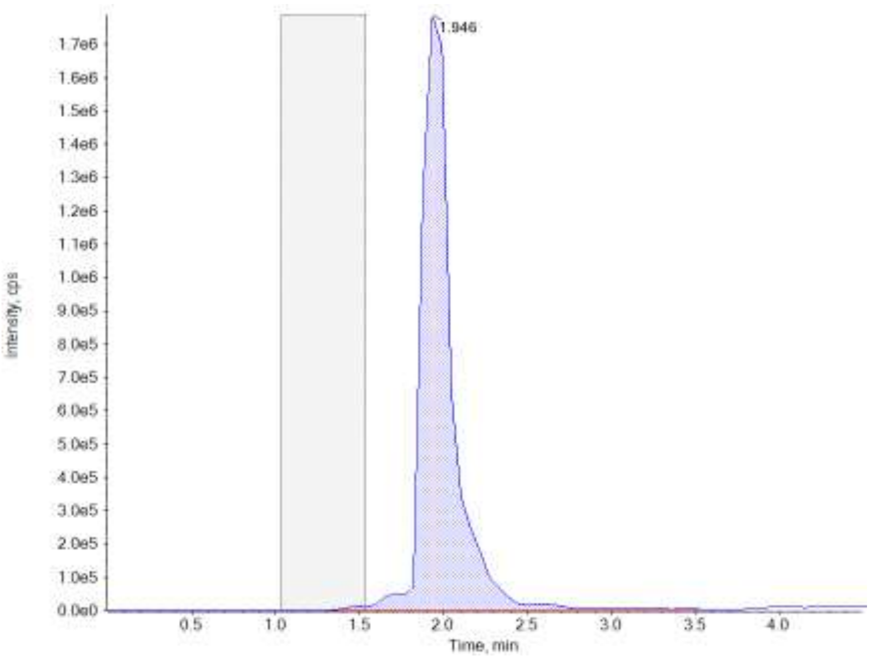	**Acquired / Library MSMS**	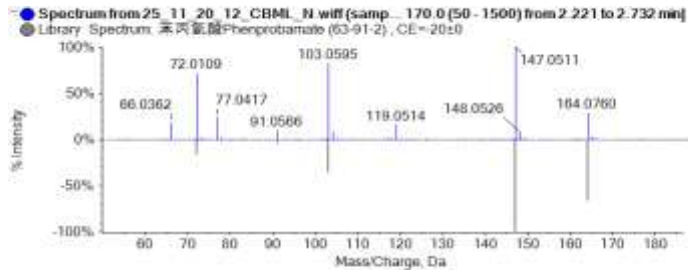
	**Acquired / Theoretical MS**	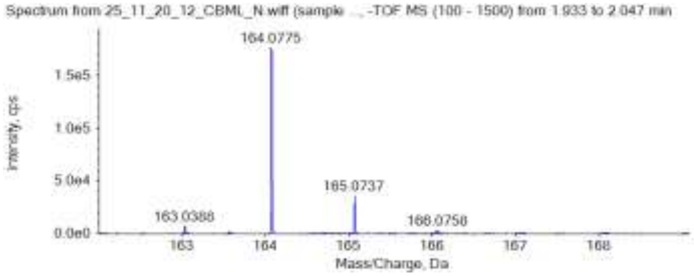

415.1595 / 2.05 (Mass/FragMass/RT/Isotope/Library/Formula/Ion Ratio)

**Retention Time:** 1.96 minutes	**Exp RT:** 2.05 minutes
**Precursor m/z :** 415.1595	**Analyte Name:**
**Fit (%)** 91.9% **RFit (%)** 100.0%	415.1595 / 2.05

	**Collision Energy = 35 ± 15 eV**

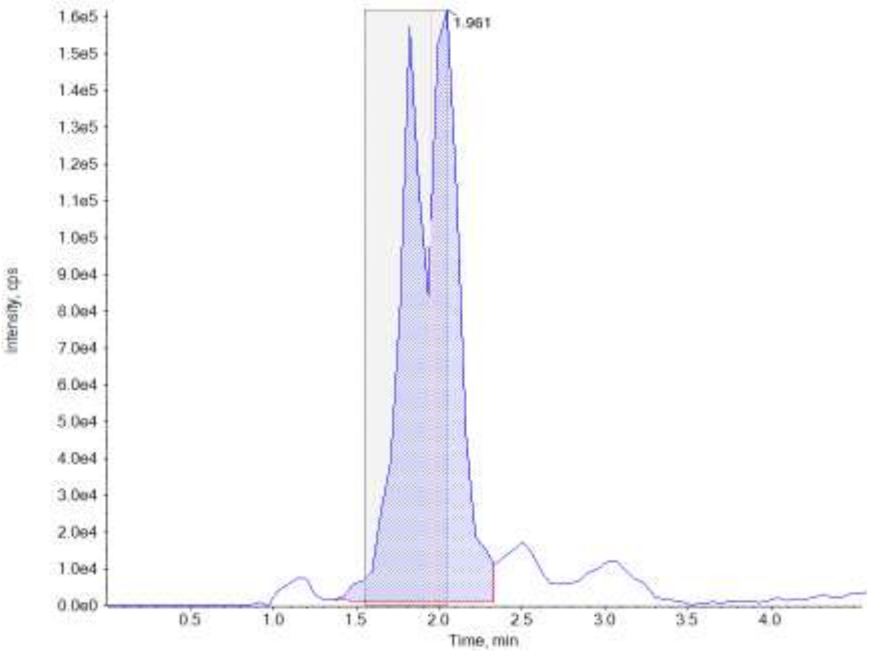

Acquired / Library MSMS

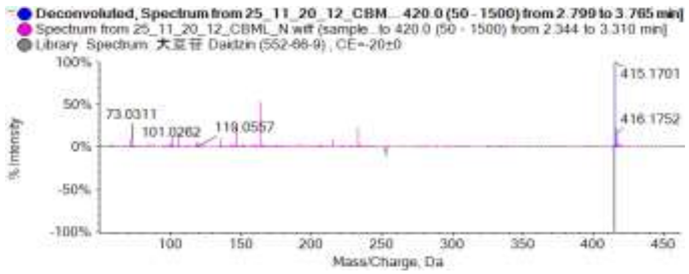

Acquired / Theoretical MS

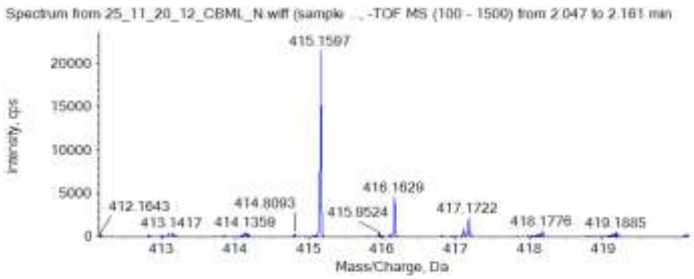

192.0658 / 2.22 (Mass/FragMass/RT/Isotope/Library/Formula/Ion Ratio)

Retention Time: 2.24 minutes
Precursor m/z : 192.0658
Fit (%) 92.9% **RFit (%)** 63.0%

Exp RT: 2.22 minutes
Analyte Name:
192.0658 / 2.22

Collision Energy = 35 ± 15 eV

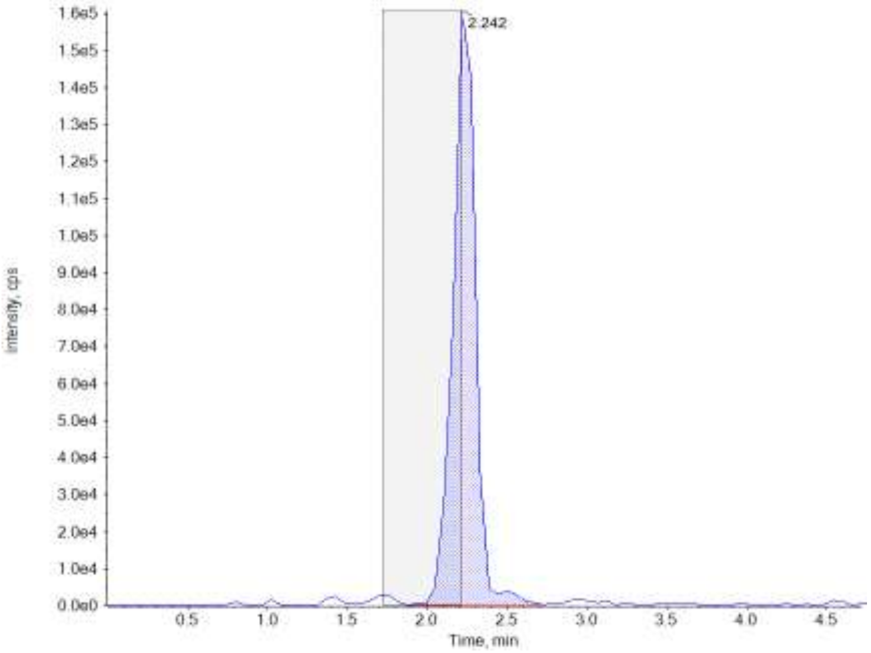

Acquired / Library MSMS

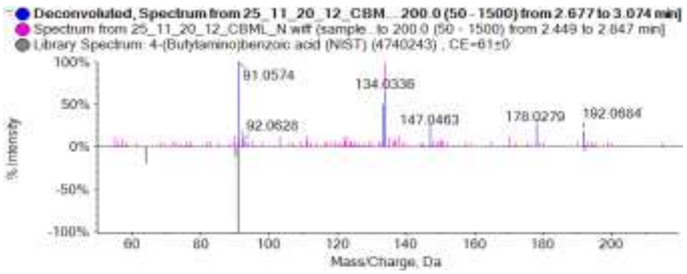

Acquired / Theoretical MS

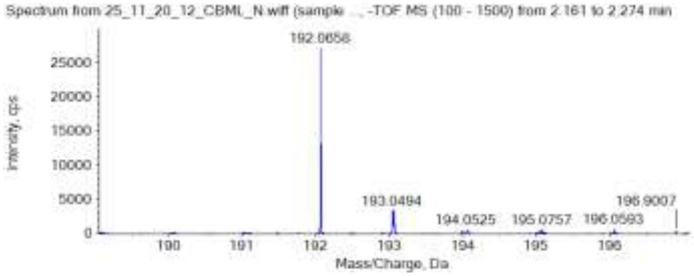

138.0543 / 2.39 (Mass/FragMass/RT/Isotope/Library/Formula/Ion Ratio)

Retention Time: 2.41 minutes
Precursor m/z : 138.0543
Fit (%) 100.0% **RFit (%)** 100.0%

Exp RT: 2.39 minutes
Analyte Name:
138.0543 / 2.39

Collision Energy = 35 ± 15 eV

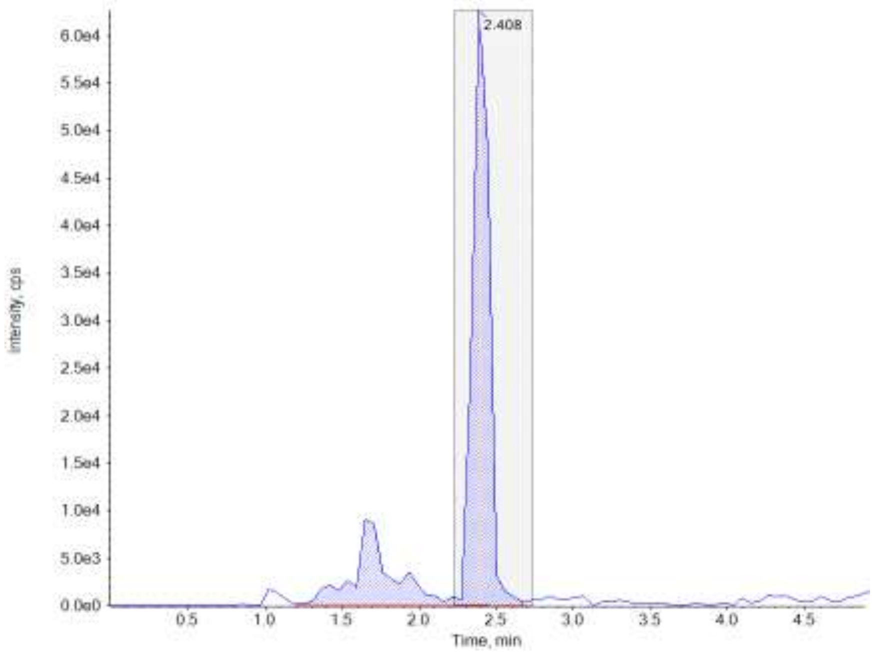

Acquired / Library MSMS

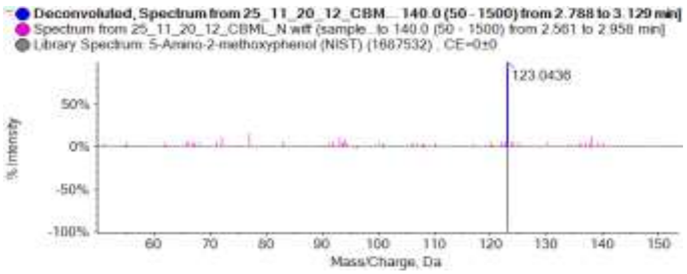

Acquired / Theoretical MS

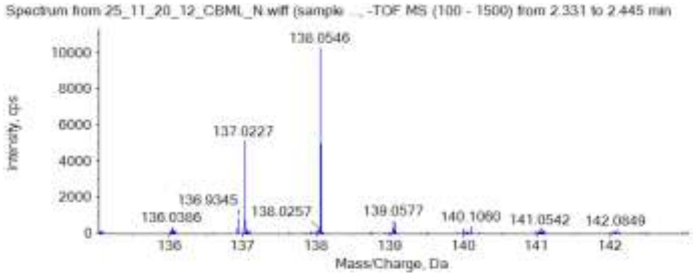

401.1788 / 2.44 (Mass/FragMass/RT/Isotope/Library/Formula/Ion Ratio)

**Retention Time:** 2.45 minutes<br>**Precursor m/z :** 401.1788<br>**Fit (%)** 83.0% **RFit (%)** 88.1%	**Exp RT:** 2.45 minutes<br>**Analyte Name:**<br>401.1788 / 2.44

		**Collision Energy = 35 ± 15 eV**
	**Acquired / Library MSMS**	
	**Acquired / Theoretical MS**	

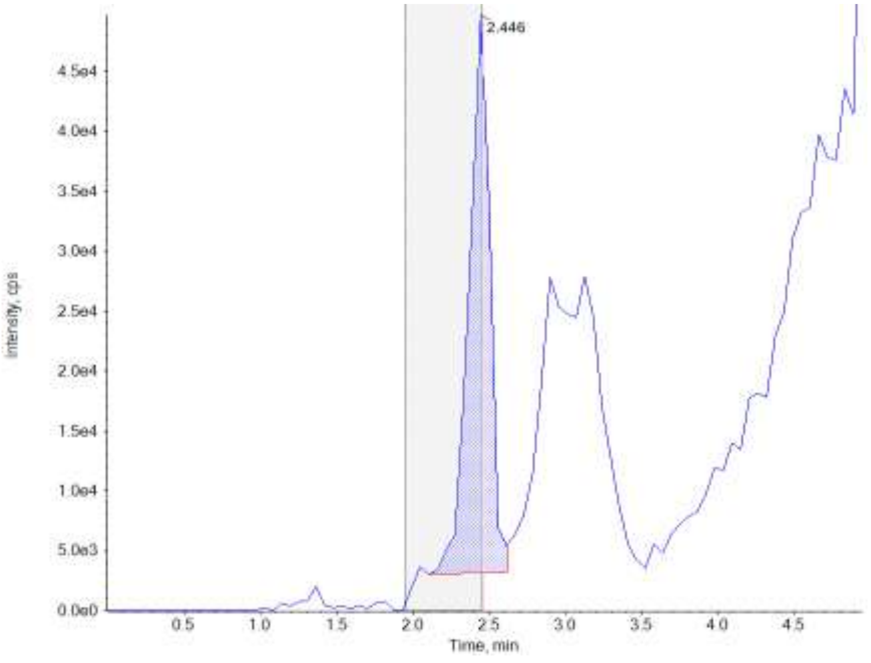

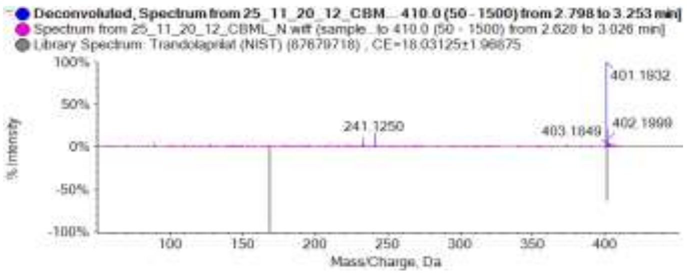

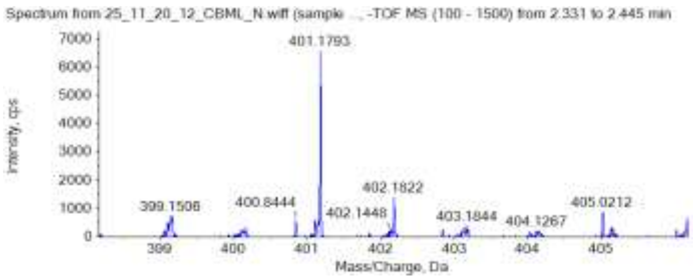

SCIEX

Created with SCIEX OS 3.3.0
Printed:12/9/2025 2:25:51 PM

307.1384 / 2.56 (Mass/FragMass/RT/Isotope/Library/Formula/Ion Ratio)

Retention Time: 2.60 minutes **Exp RT:** 2.56 minutes
Precursor m/z : 307.1384 **Analyte Name:**
Fit (%) 53.5% **RFit (%)** 98.5% 307.1384 / 2.56

Collision Energy = 35 ± 15 eV

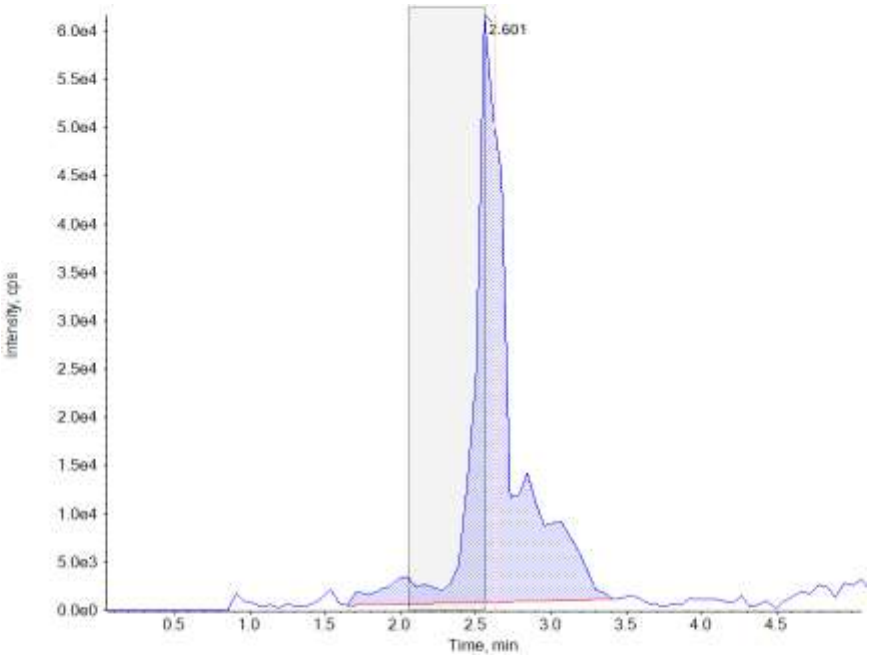

Acquired / Library MSMS

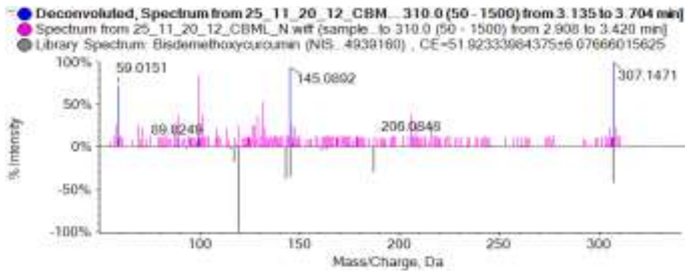

Acquired / Theoretical MS

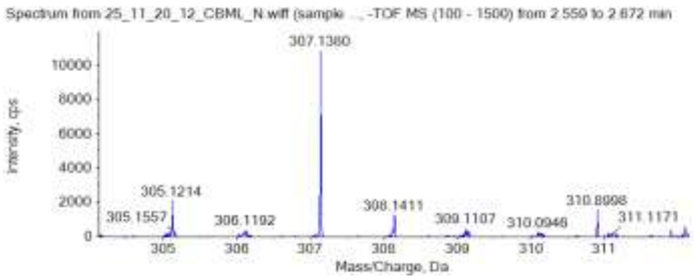

206.0809 / 2.62 (Mass/FragMass/RT/Isotope/Library/Formula/Ion Ratio)

Retention Time: 2.63 minutes
Precursor m/z : 206.0809
Fit (%) 99.5% **RFit (%)** 92.3%

Exp RT: 2.62 minutes
Analyte Name:
206.0809 / 2.62

Collision Energy = 35 ± 15 eV

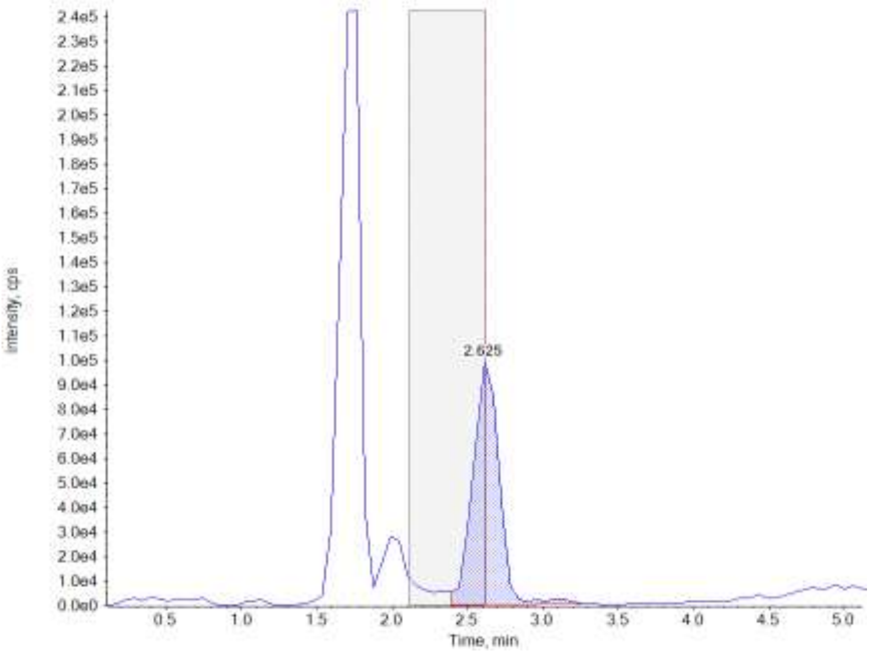

Acquired / Library MSMS

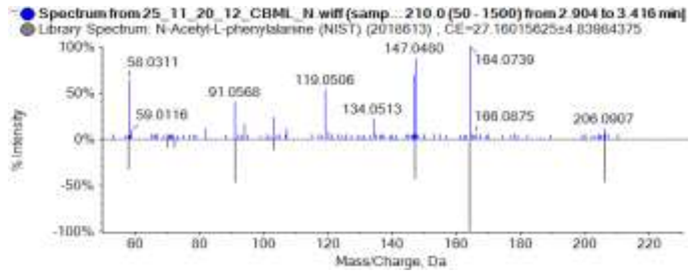

Acquired / Theoretical MS

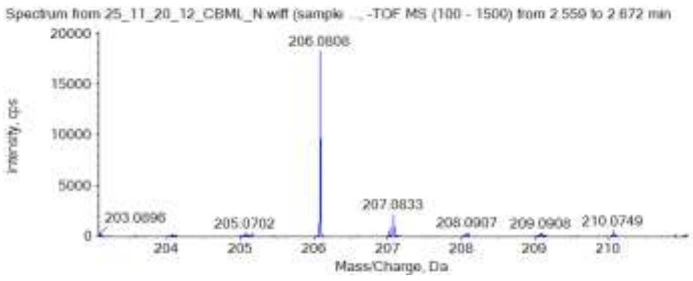

327.1064 / 2.62 (Mass/FragMass/RT/Isotope/Library/Formula/Ion Ratio)

**Retention Time:** 2.64 minutes	**Exp RT:** 2.62 minutes
**Precursor m/z :** 327.1064	**Analyte Name:**
**Fit (%)** 55.3% **RFit (%)** 97.7%	327.1064 / 2.62

Collision Energy = 35 ± 15 eV

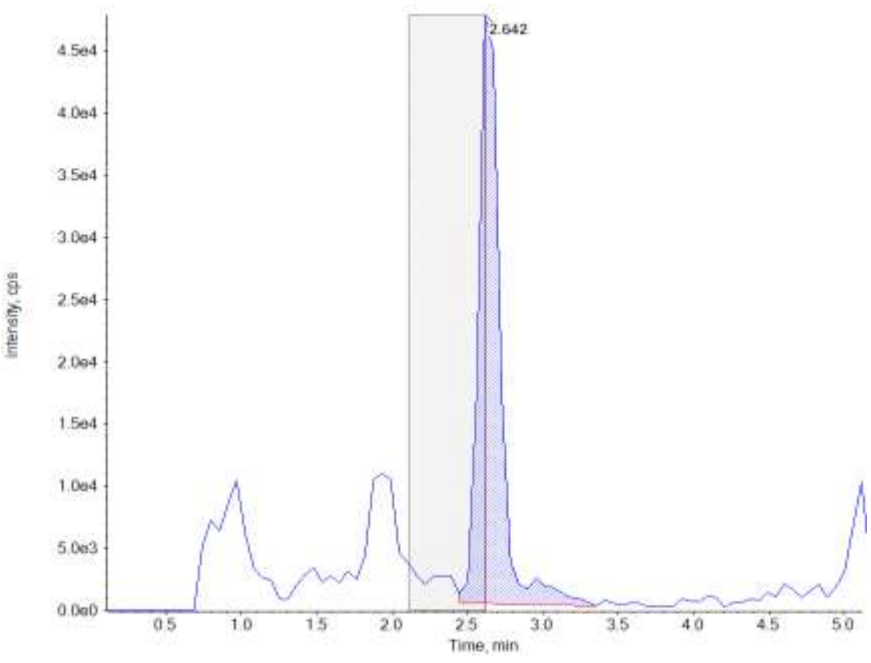

Acquired / Library MSMS

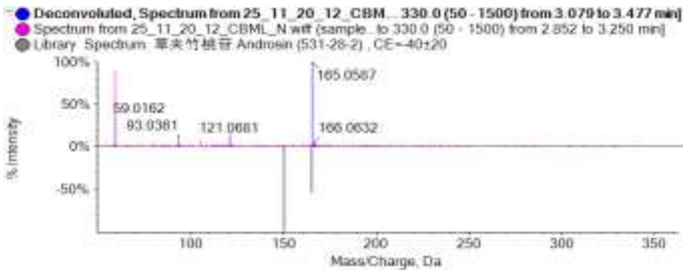

Acquired / Theoretical MS

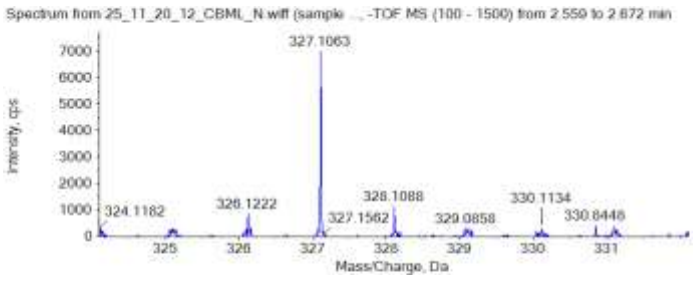

153.0179 / 2.67 (Mass/FragMass/RT/Isotope/Library/Formula/Ion Ratio)

Retention Time: 2.69 minutes
Precursor m/z : 153.0179
Fit (%) 99.4% **RFit (%)** 98.7%

Exp RT: 2.67 minutes
Analyte Name:
153.0179 / 2.67

Collision Energy = 35 ± 15 eV

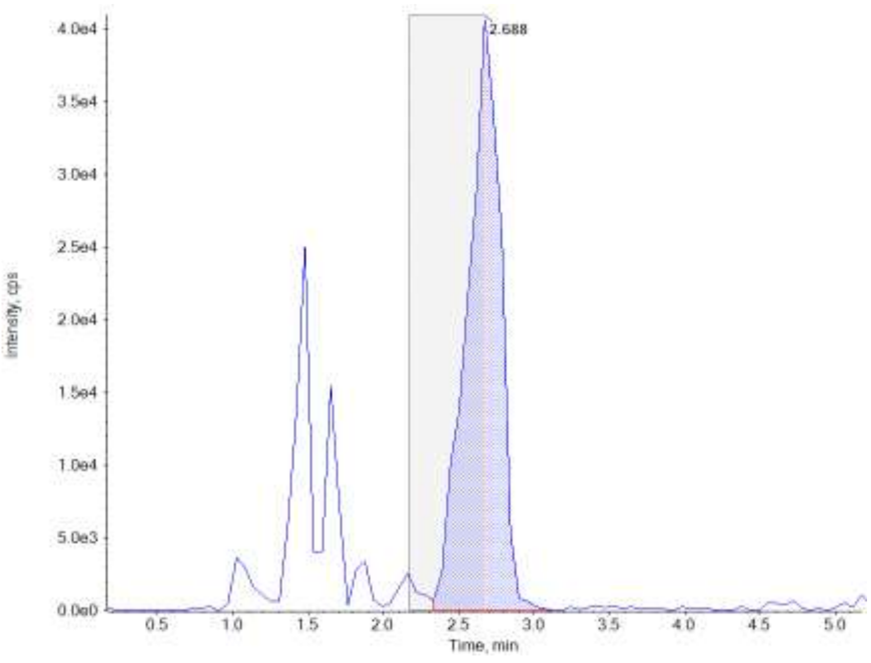

Acquired / Library MSMS

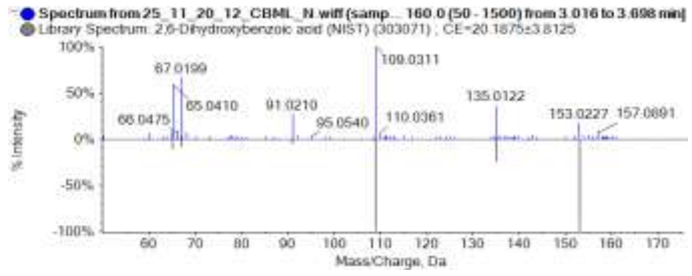

Acquired / Theoretical MS

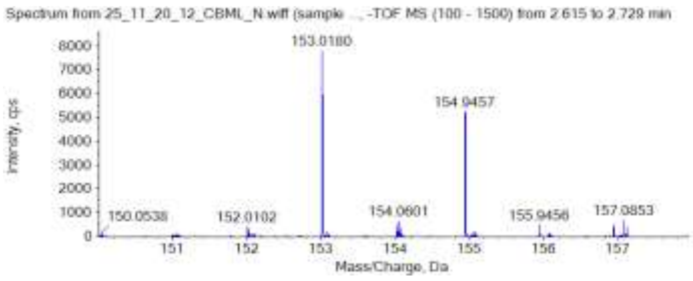

167.0330 / 2.67 (Mass/FragMass/RT/Isotope/Library/Formula/Ion Ratio)

Retention Time: 2.69 minutes **Exp RT:** 2.67 minutes
Precursor m/z : 167.0331 **Analyte Name:**
Fit (%) 98.5% **RFit (%)** 88.4% 167.0330 / 2.67

Collision Energy = 35 ± 15 eV

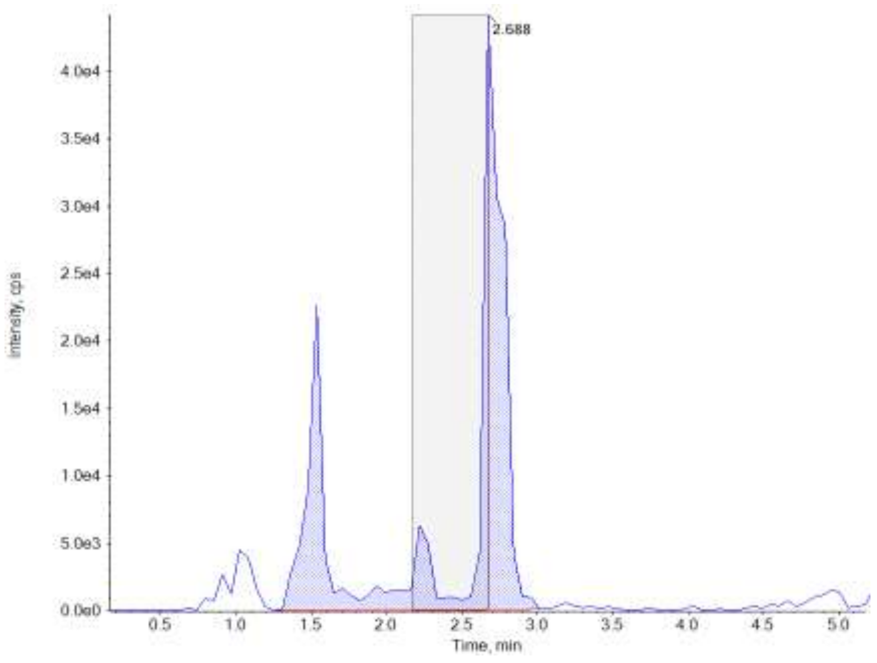

Acquired / Library MSMS

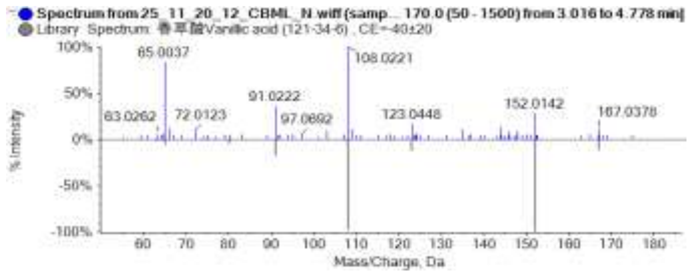

Acquired / Theoretical MS

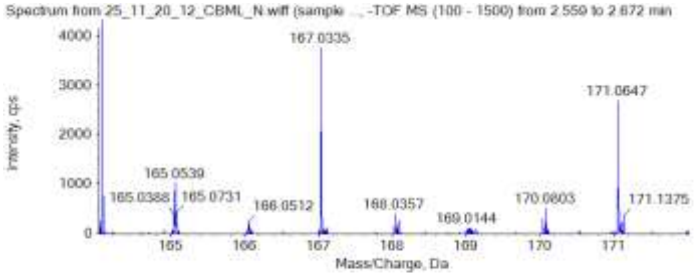

399.1664 / 2.90 (Mass/FragMass/RT/Isotope/Library/Formula/Ion Ratio)

Retention Time: 2.88 minutes
Precursor m/z : 399.1664
Fit (%) 67.4% **RFit (%)** 94.3%

Exp RT: 2.90 minutes
Analyte Name:
399.1664 / 2.90

Collision Energy = 35 ± 15 eV

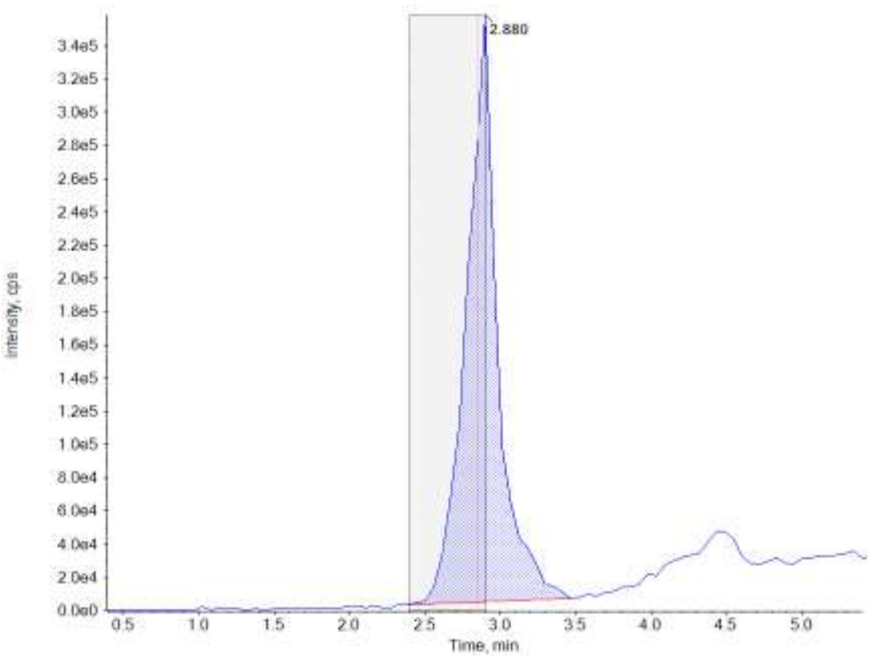

Acquired / Library MSMS

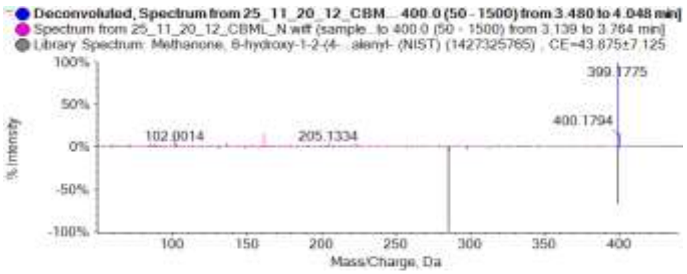

Acquired / Theoretical MS

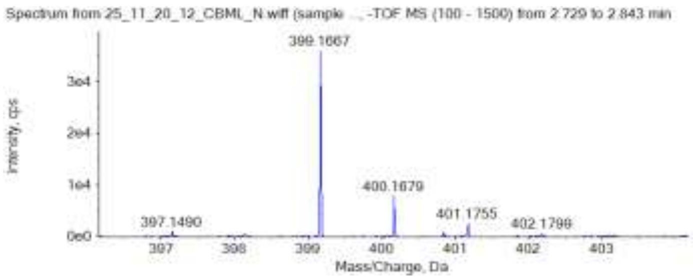

275.1125 / 3.07 (Mass/FragMass/RT/Isotope/Library/Formula/Ion Ratio)

Retention Time: 3.06 minutes
Precursor m/z : 275.1125
Fit (%) 96.8% **RFit (%)** 71.7%

Exp RT: 3.07 minutes
Analyte Name:
275.1125 / 3.07

Collision Energy = 35 ± 15 eV

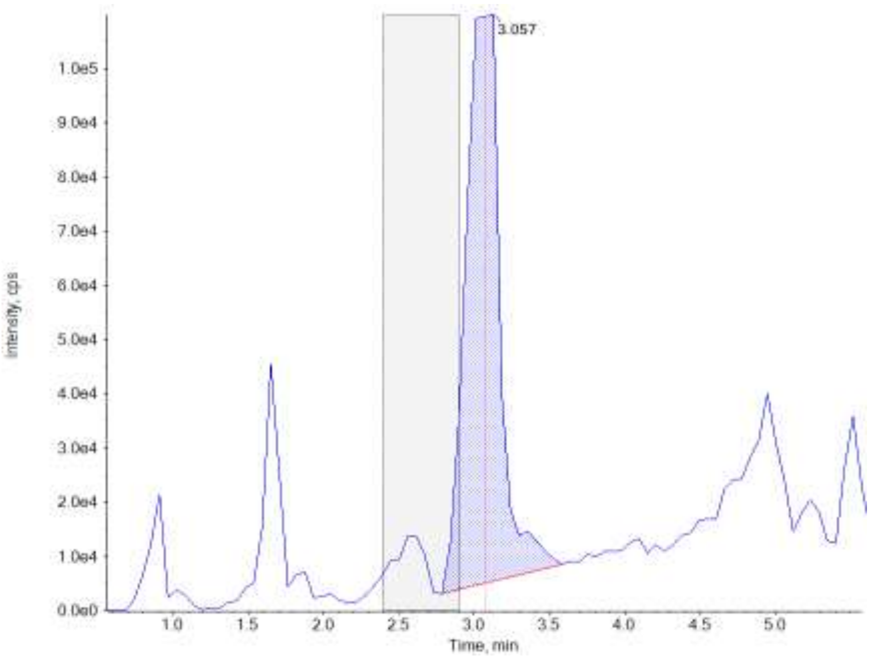

Acquired / Library MSMS

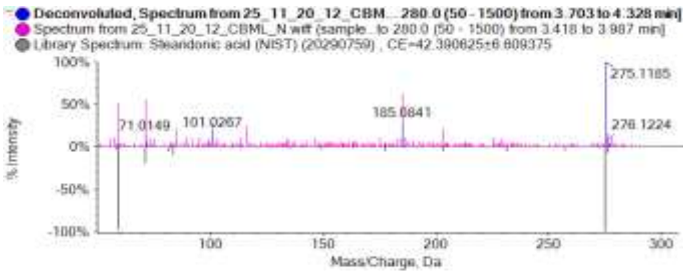

Acquired / Theoretical MS

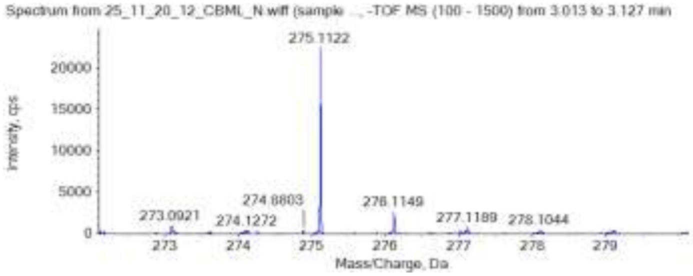

203.0886 / 3.13 (Mass/FragMass/RT/Isotope/Library/Formula/Ion Ratio)

**Retention Time:** 3.13 minutes	**Exp RT:** 3.13 minutes
**Precursor m/z :** 203.0886	**Analyte Name:**
**Fit (%)** 98.6% **RFit (%)** 100.0%	203.0886 / 3.13

Collision Energy = 35 ± 15 eV

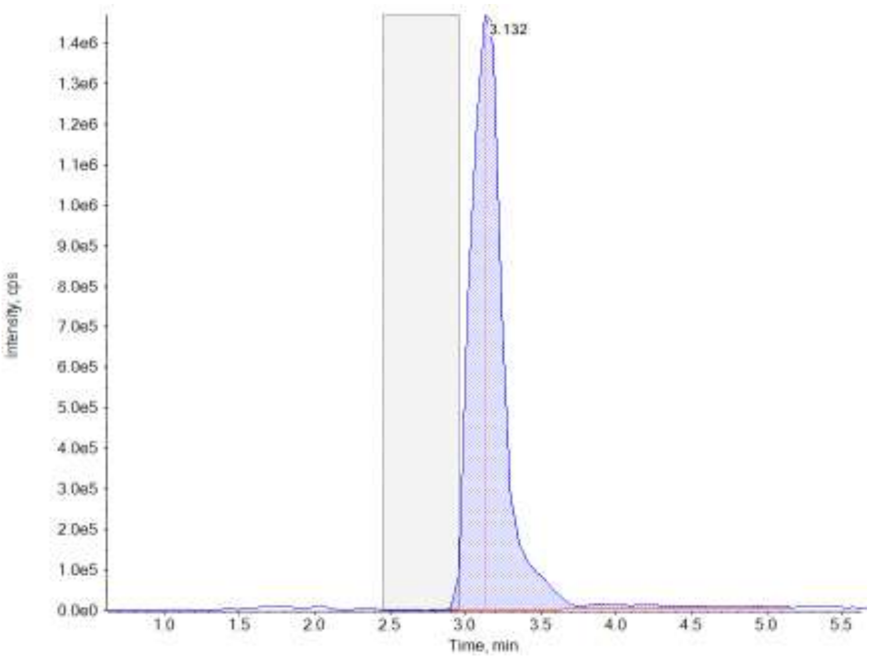

Acquired / Library MSMS

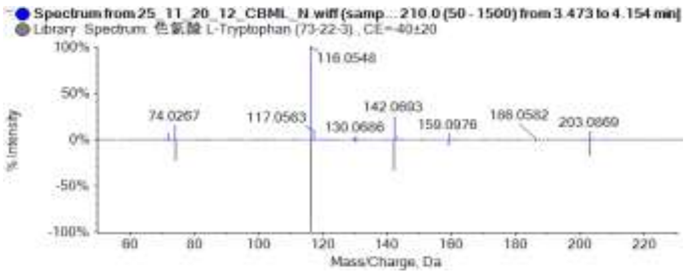

Acquired / Theoretical MS

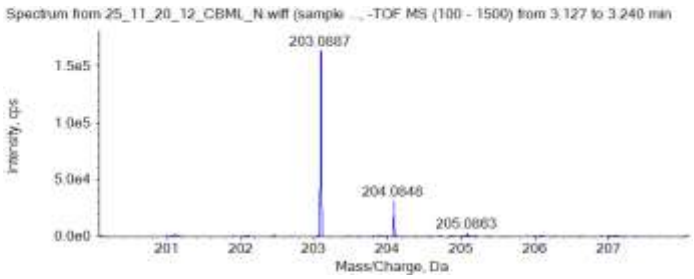

397.1489 / 3.13 (Mass/FragMass/RT/Isotope/Library/Formula/Ion Ratio)

Retention Time: 3.14 minutes
Precursor m/z : 397.1489
Fit (%) 96.9% **RFit (%)** 79.2%

Exp RT: 3.13 minutes
Analyte Name:
397.1489 / 3.13

Collision Energy = 35 ± 15 eV

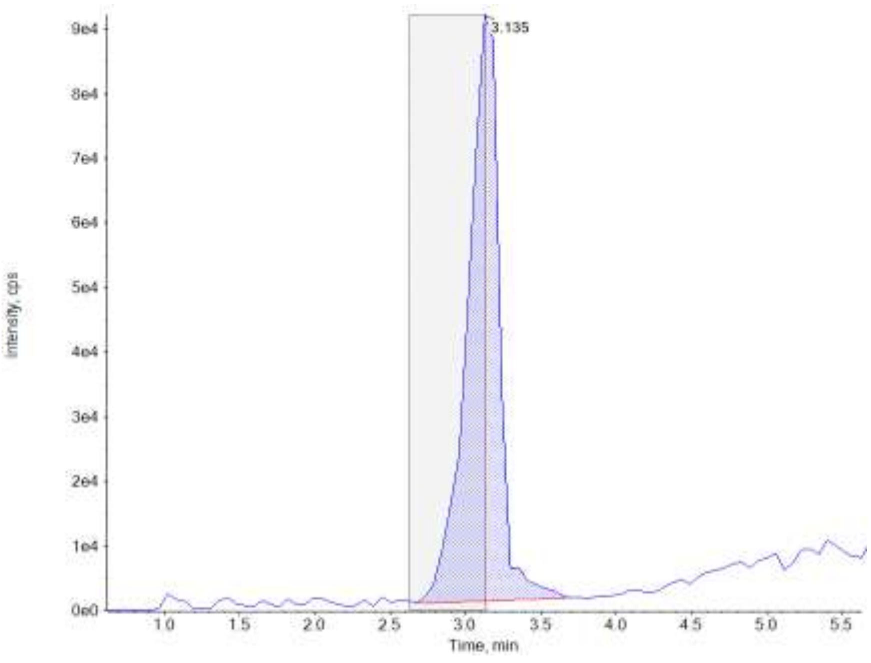

Acquired / Library MSMS

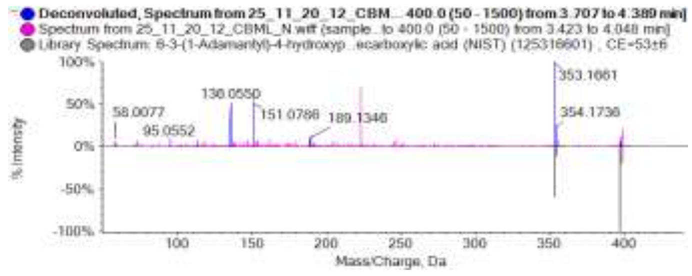

Acquired / Theoretical MS

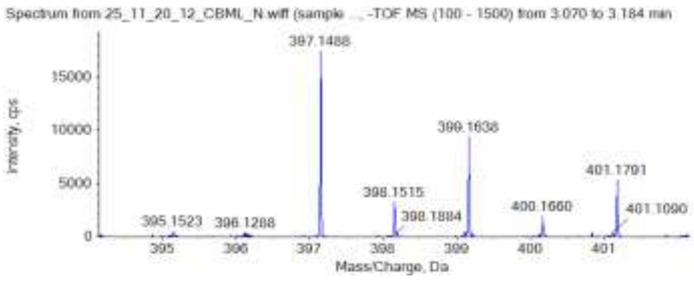

Created with SCIEX OS 3.3.0
Printed:12/9/2025 2:25:51 PM

121.0284 / 3.24 (Mass/FragMass/RT/Isotope/Library/Formula/Ion Ratio)

**Retention Time:** 3.32 minutes	**Exp RT:** 3.24 minutes
**Precursor m/z :** 121.0284	**Analyte Name:**
**Fit (%)** 90.2% **RFit (%)** 95.8%	121.0284 / 3.24

Collision Energy = 35 ± 15 eV

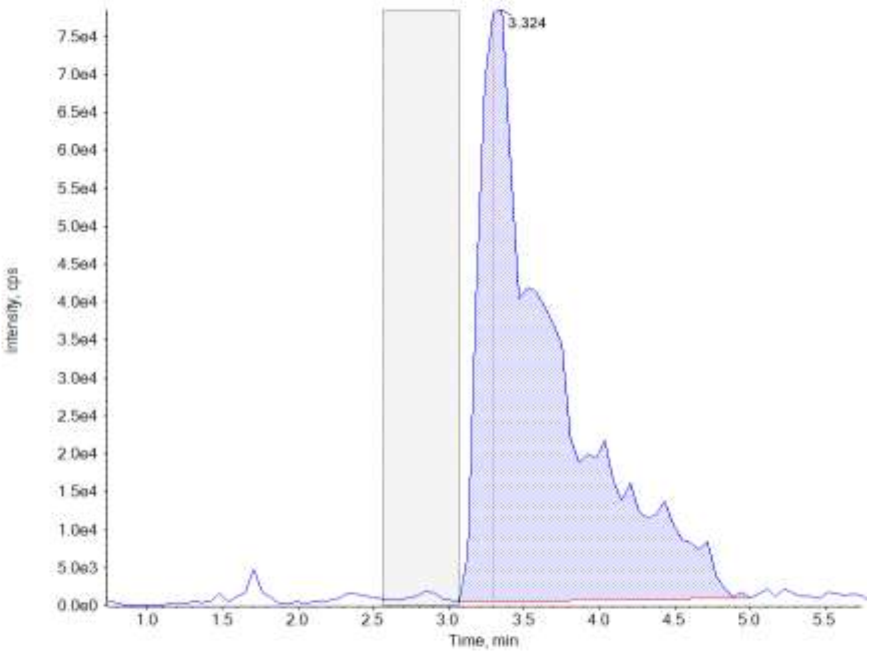

Acquired / Library MSMS

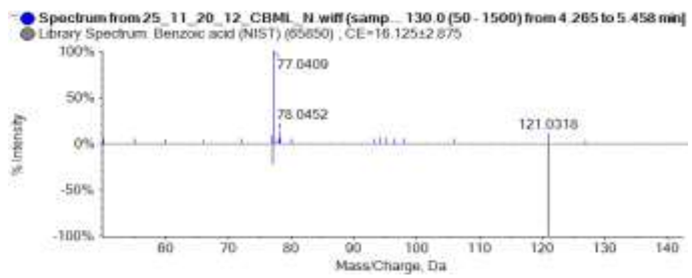

Acquired / Theoretical MS

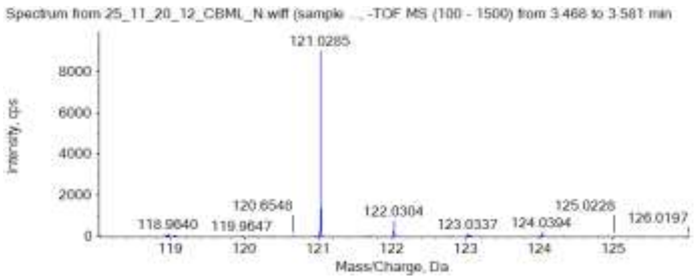

339.0703 / 4.83 (Mass/FragMass/RT/Isotope/Library/Formula/Ion Ratio)

Retention Time: 4.81 minutes
Precursor m/z : 339.0703
Fit (%) 97.2% **RFit (%)** 100.0%

Exp RT: 4.83 minutes
Analyte Name:
339.0703 / 4.83

Collision Energy = 35 ± 15 eV

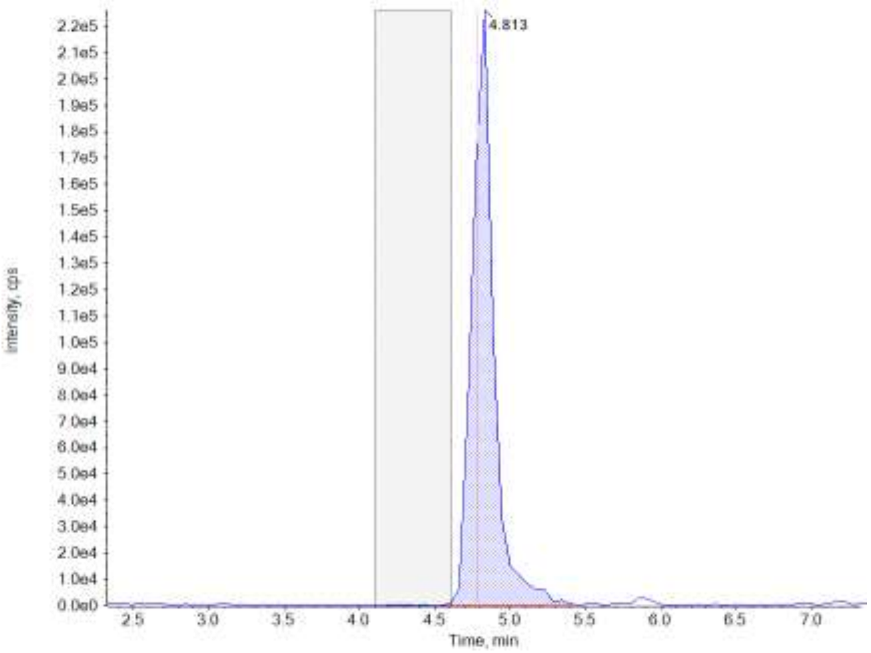

Acquired / Library MSMS

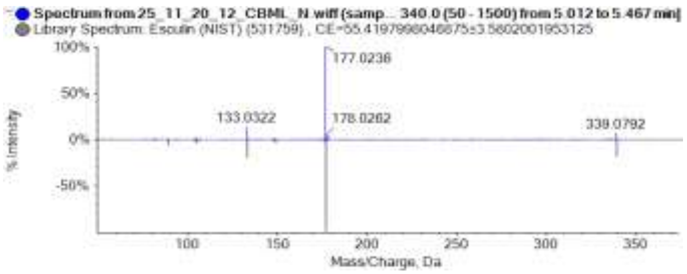

Acquired / Theoretical MS

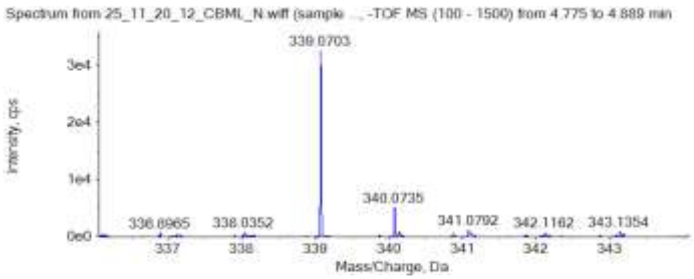

Created with SCIEX OS 3.3.0
Printed:12/9/2025 2:25:51 PM

274.8785 / 5.00 (Mass/FragMass/RT/Isotope/Library/Formula/Ion Ratio)

**Retention Time:** 5.35 minutes	**Exp RT:** 5.00 minutes
**Precursor m/z :** 274.8785	**Analyte Name:**
**Fit (%)** 29.5% **RFit (%)** 100.0%	274.8785 / 5.00

Collision Energy = 35 ± 15 eV

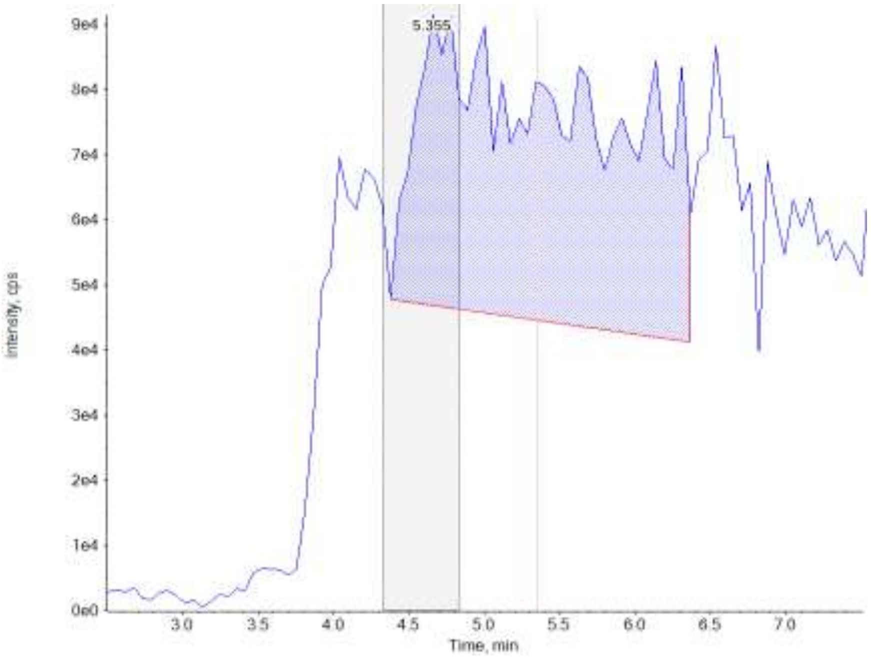

Acquired / Library MSMS

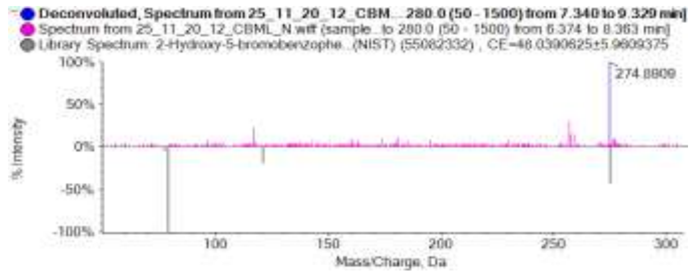

Acquired / Theoretical MS

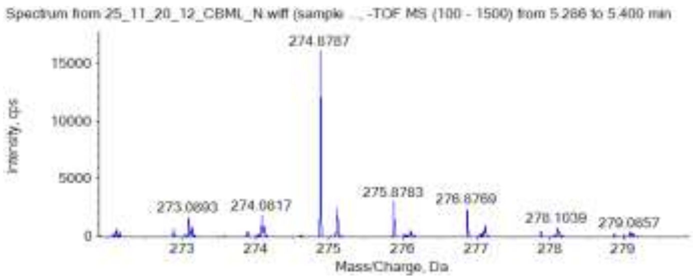

281.1382 / 5.23 (Mass/FragMass/RT/Isotope/Library/Formula/Ion Ratio)

Retention Time: 5.11 minutes
Precursor m/z : 281.1382
Fit (%) 92.0% **RFit (%)** 53.0%

Exp RT: 5.23 minutes
Analyte Name:
281.1382 / 5.23

Collision Energy = 35 ± 15 eV

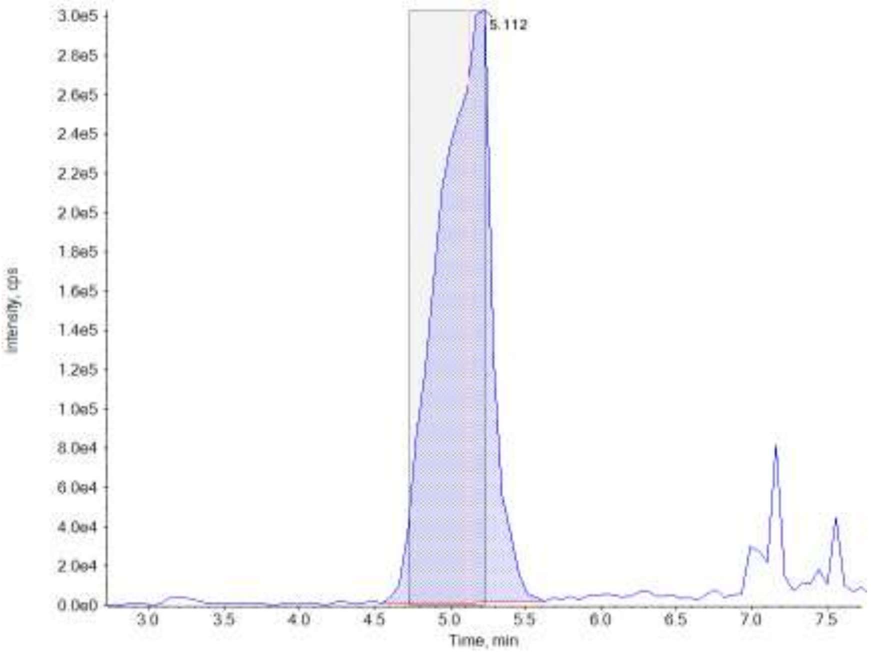

Acquired / Library MSMS

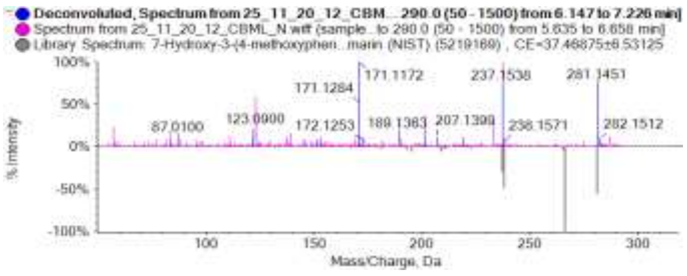

Acquired / Theoretical MS

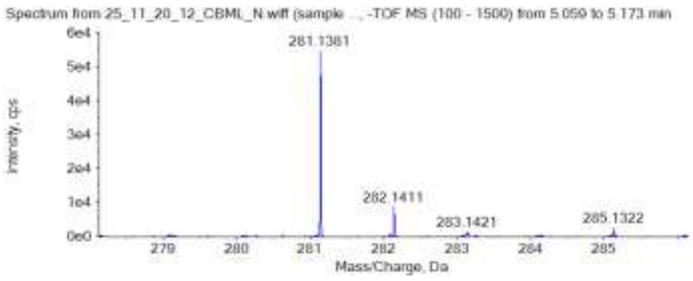

537.1247 / 5.80 (Mass/FragMass/RT/Isotope/Library/Formula/Ion Ratio)

**Retention Time:** 5.80 minutes	**Exp RT:** 5.80 minutes
**Precursor m/z :** 537.1247	**Analyte Name:**
**Fit (%)** 77.7% **RFit (%)** 96.2%	537.1247 / 5.80

Collision Energy = 35 ± 15 eV

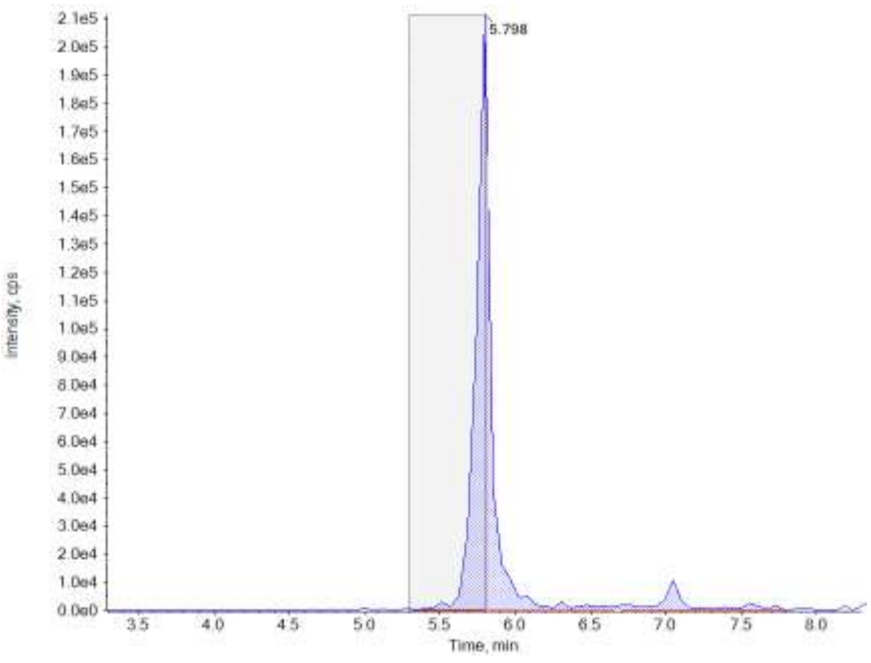

Acquired / Library MSMS

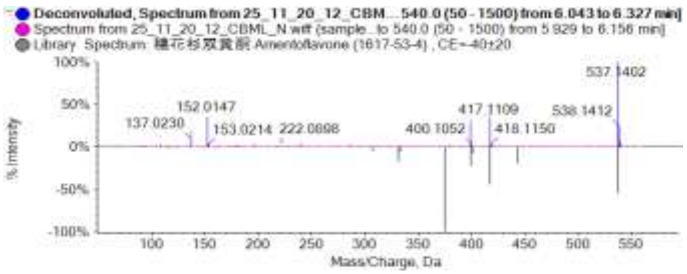

Acquired / Theoretical MS

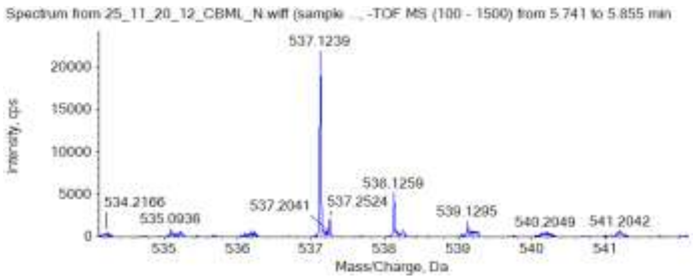

401.1790 / 5.91 (Mass/FragMass/RT/Isotope/Library/Formula/Ion Ratio)

Retention Time: 5.92 minutes
Precursor m/z : 401.1790
Fit (%) 83.6% **RFit (%)** 69.1%

Exp RT: 5.91 minutes
Analyte Name:
401.1790 / 5.91

Collision Energy = 35 ± 15 eV

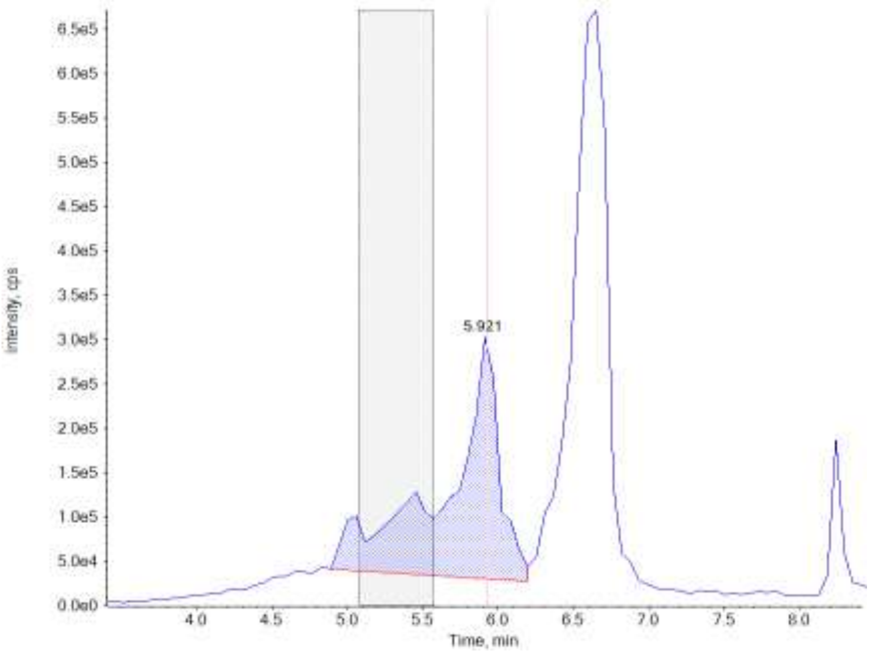

Acquired / Library MSMS

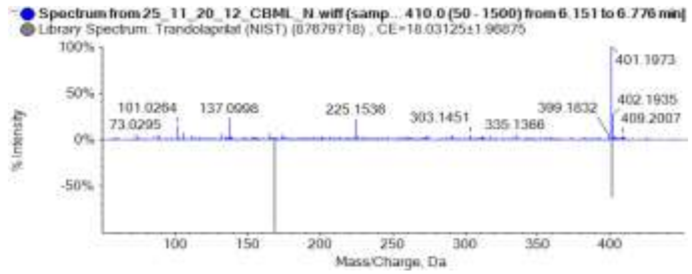

Acquired / Theoretical MS

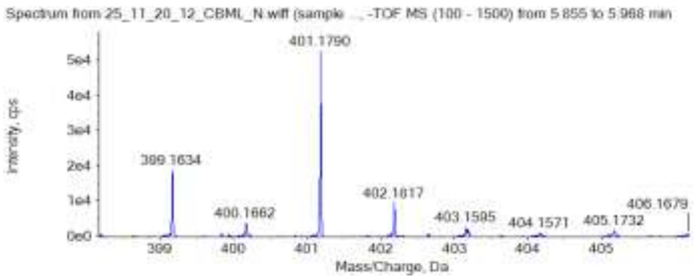

SCIEX

421.1610 / 6.25 (Mass/FragMass/RT/Isotope/Library/Formula/Ion Ratio)

Retention Time: 6.25 minutes **Exp RT:** 6.25 minutes
Precursor m/z : 421.1610 **Analyte Name:**
Fit (%) 90.2% **RFit (%)** 80.5% 421.1610 / 6.25

Collision Energy = 35 ± 15 eV

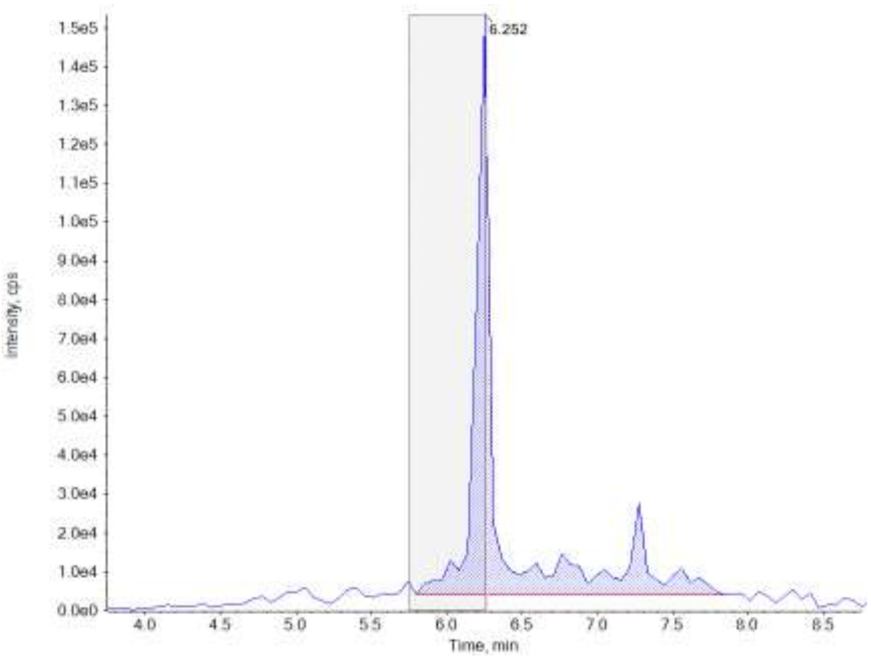

Acquired / Library MSMS

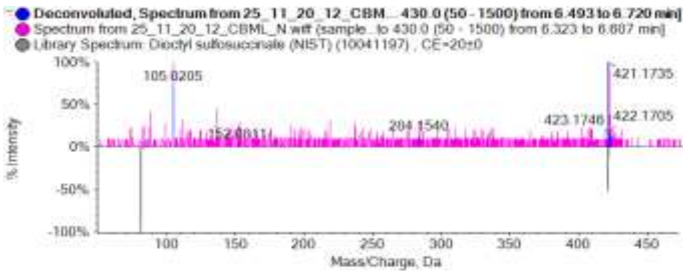

Acquired / Theoretical MS

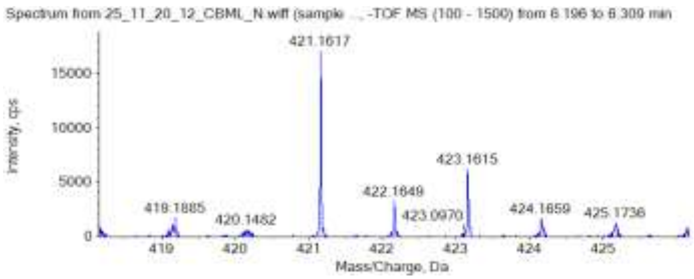

431.1920 / 6.25 (Mass/FragMass/RT/Isotope/Library/Formula/Ion Ratio)

Retention Time: 6.25 minutes
Precursor m/z : 431.1920
Fit (%) 99.6% **RFit (%)** 79.6%

Exp RT: 6.25 minutes
Analyte Name:
431.1920 / 6.25

Collision Energy = 35 ± 15 eV

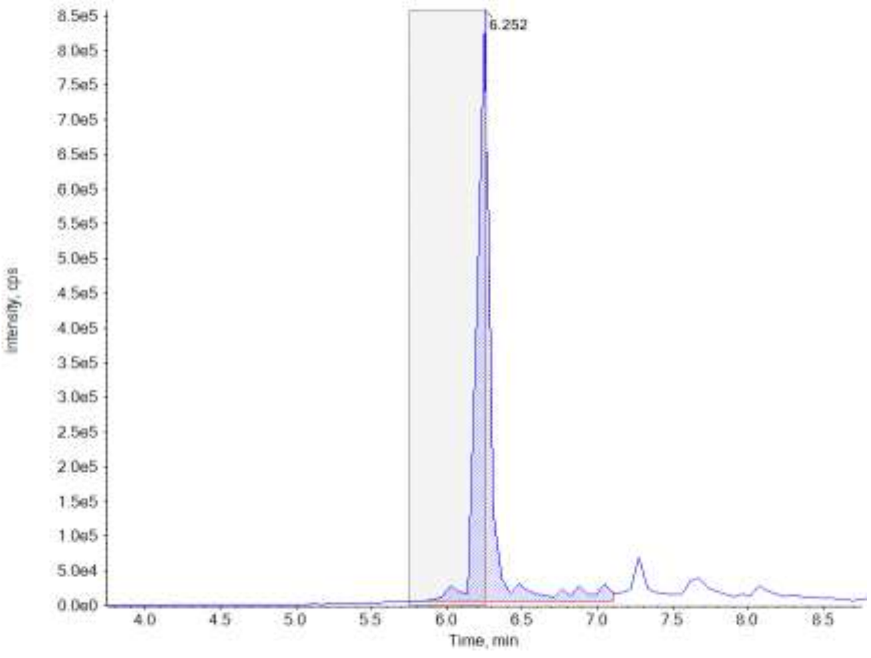

Acquired / Library MSMS

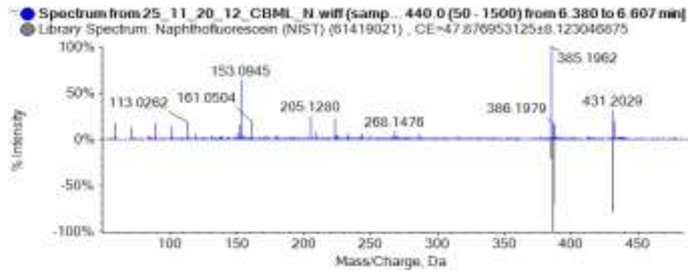

Acquired / Theoretical MS

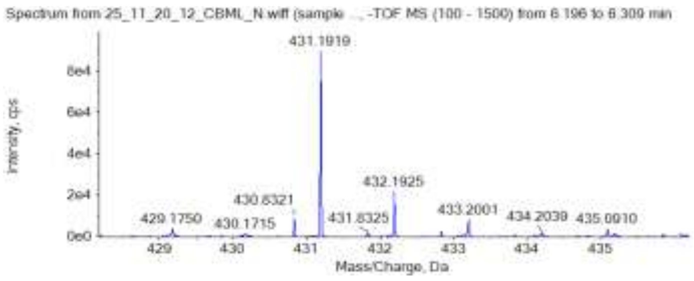

Created with SCIEX OS 3.3.0
Printed:12/9/2025 2:25:51 PM

193.0488 / 6.71 (Mass/FragMass/RT/Isotope/Library/Formula/Ion Ratio)

Retention Time: 6.65 minutes
Precursor m/z : 193.0488
Fit (%) 99.2% **RFit (%)** 98.7%

Exp RT: 6.71 minutes
Analyte Name:
193.0488 / 6.71

Collision Energy = 35 ± 15 eV

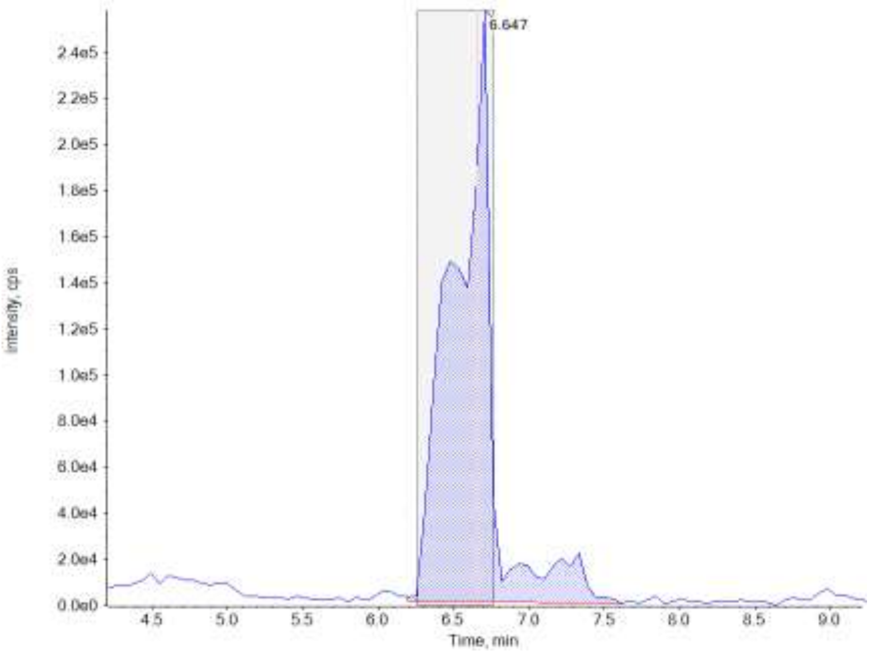

Acquired / Library MSMS

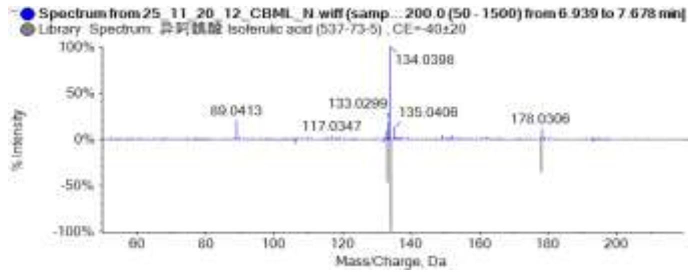

Acquired / Theoretical MS

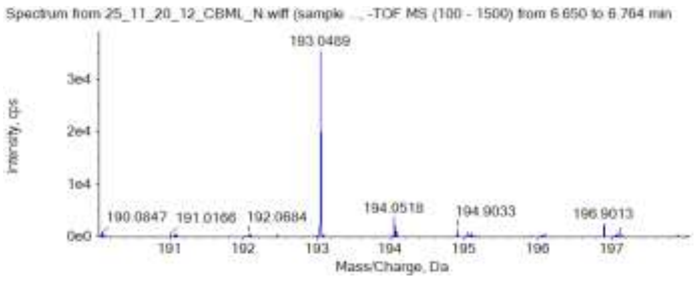

949.3568 / 6.71 (Mass/FragMass/RT/Isotope/Library/Formula/Ion Ratio)

Retention Time: 6.72 minutes
Precursor m/z : 949.3568
Fit (%) 30.5% **RFit (%)** 98.8%

Exp RT: 6.71 minutes
Analyte Name:
949.3568 / 6.71

Collision Energy = 35 ± 15 eV

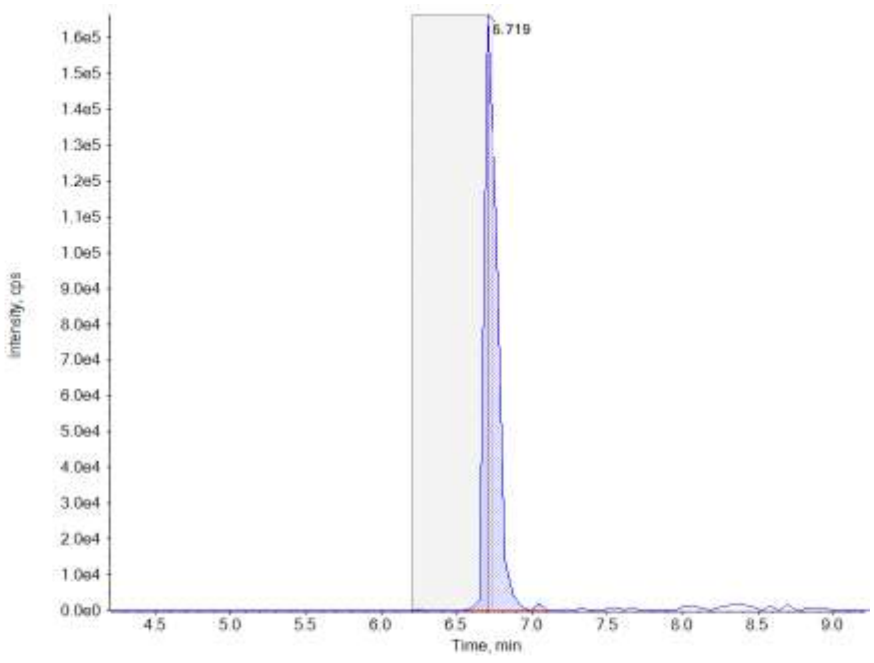

Acquired / Library MSMS

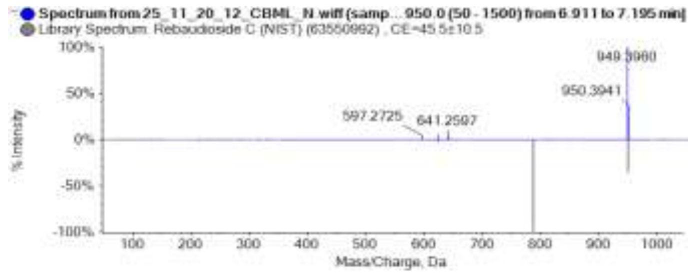

Acquired / Theoretical MS

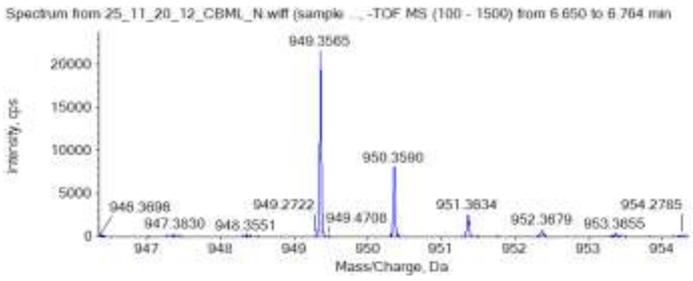

345.1073 / 6.88 (Mass/FragMass/RT/Isotope/Library/Formula/Ion Ratio)

**Retention Time:** 6.88 minutes<br>**Precursor m/z :** 345.1073<br>**Fit (%)** 94.7% **RFit (%)** 60.2%	**Exp RT:** 6.88 minutes<br>**Analyte Name:**<br>345.1073 / 6.88

	**Collision Energy = 35 ± 15 eV**	
	**Acquired / Library MSMS**	
	**Acquired / Theoretical MS**	

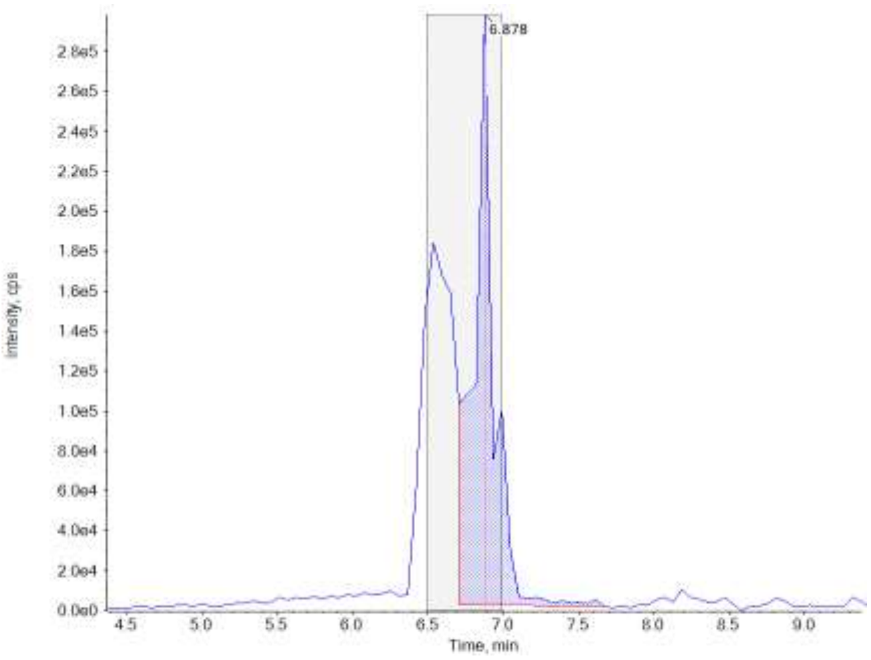

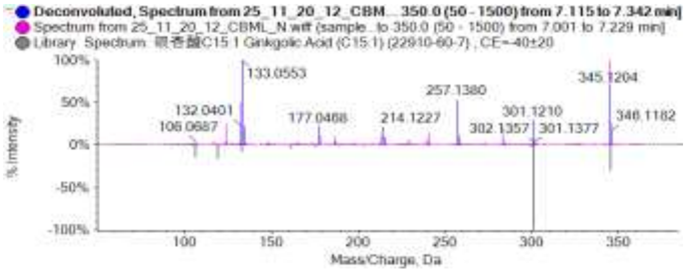

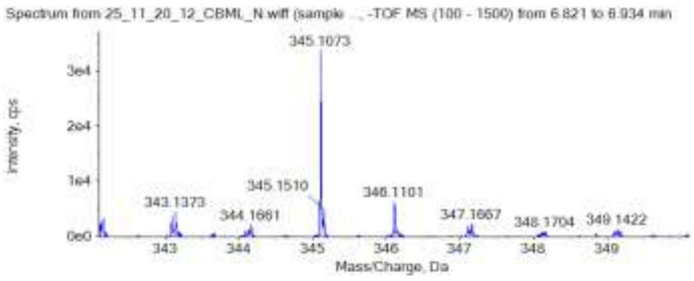

247.1536 / 6.93 (Mass/FragMass/RT/Isotope/Library/Formula/Ion Ratio)

Retention Time: 6.90 minutes
Precursor m/z : 247.1536
Fit (%) 100.0% **RFit (%)** 89.2%

Exp RT: 6.93 minutes
Analyte Name:
247.1536 / 6.93

Collision Energy = 35 ± 15 eV

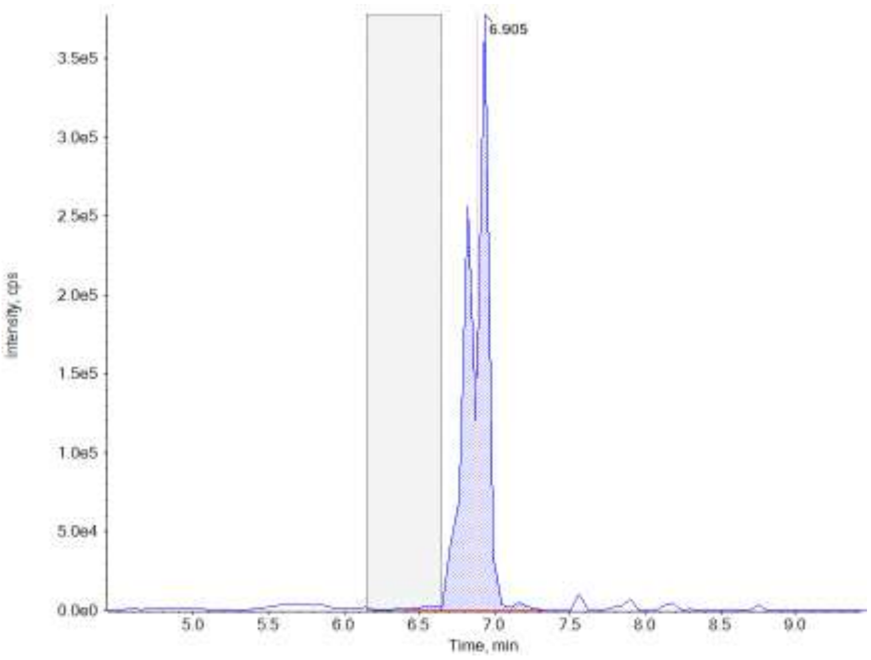

Acquired / Library MSMS

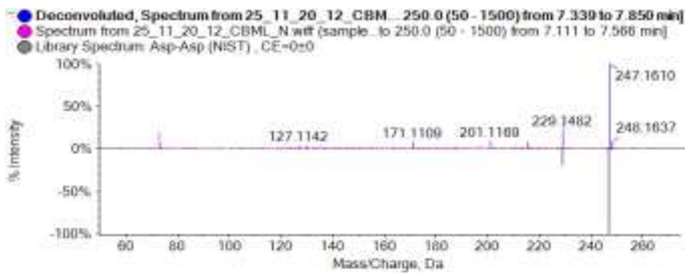

Acquired / Theoretical MS

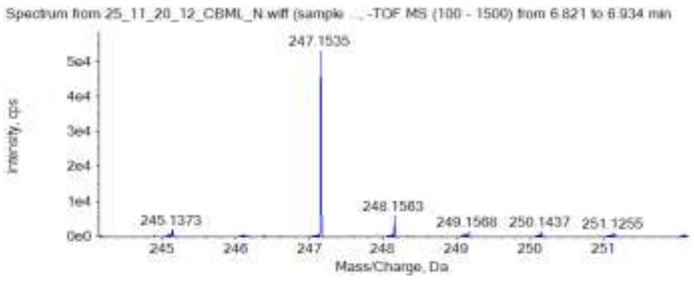

551.2332 / 6.99 (Mass/FragMass/RT/Isotope/Library/Formula/Ion Ratio)

Retention Time: 7.01 minutes
Precursor m/z : 551.2332
Fit (%) 99.0% **RFit (%)** 95.7%

Exp RT: 6.99 minutes
Analyte Name:
551.2332 / 6.99

Collision Energy = 35 ± 15 eV

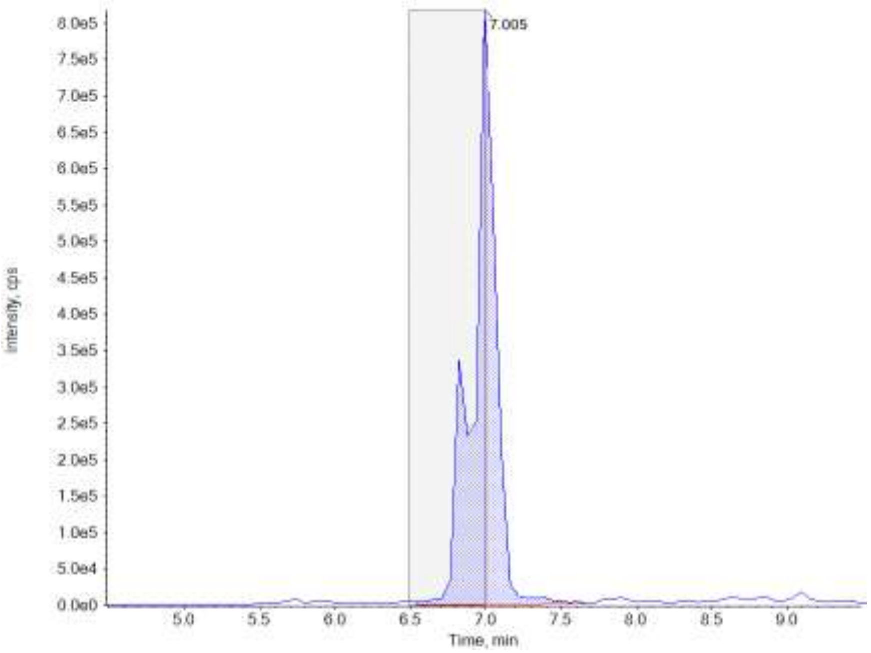

Acquired / Library MSMS

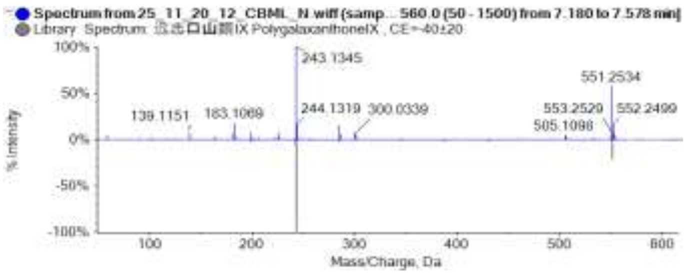

Acquired / Theoretical MS

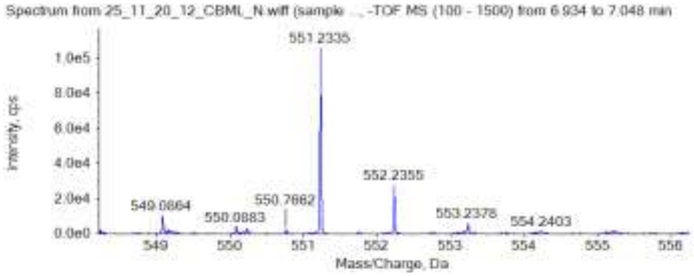

595.1299 / 7.05 (Mass/FragMass/RT/Isotope/Library/Formula/Ion Ratio)

**Retention Time:** 7.05 minutes	**Exp RT:** 7.05 minutes
**Precursor m/z :** 595.1299	**Analyte Name:**
**Fit (%)** 100.0% **RFit (%)** 99.4%	595.1299 / 7.05

Collision Energy = 35 ± 15 eV

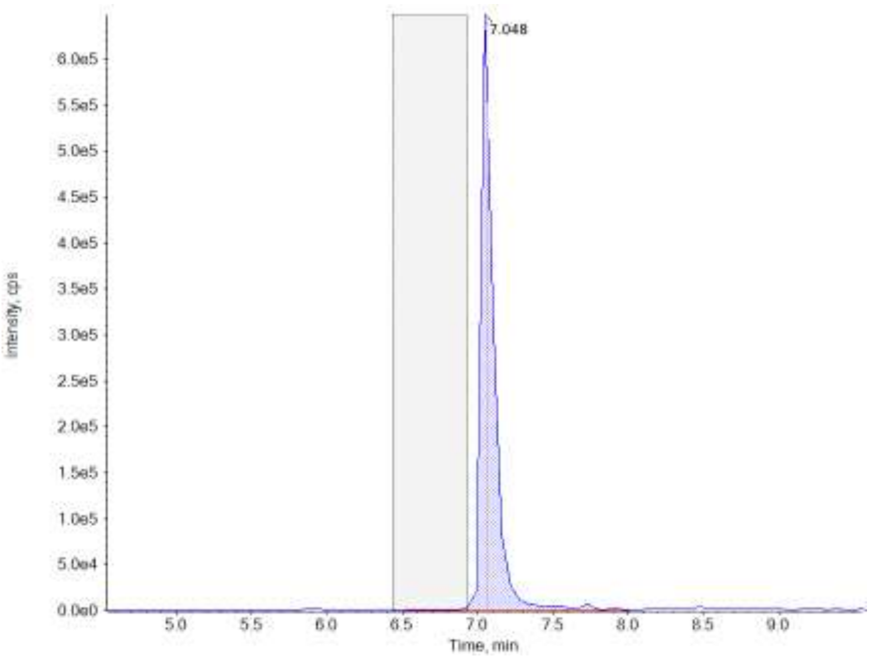

Acquired / Library MSMS

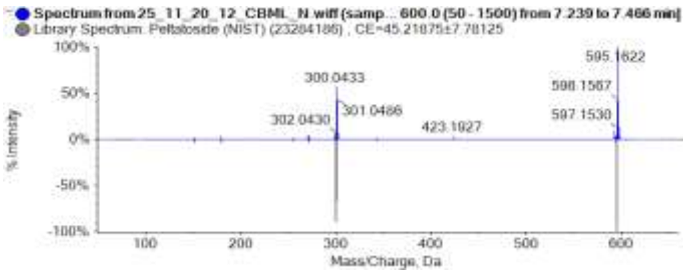

Acquired / Theoretical MS

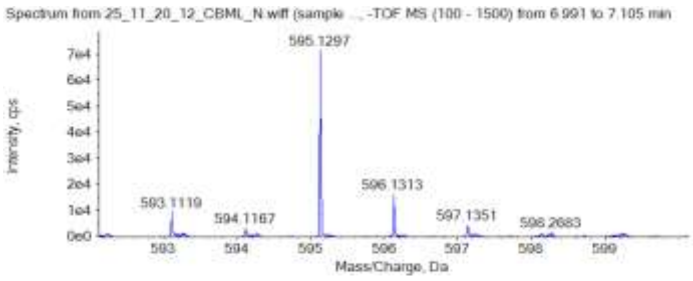

609.1444 / 7.28 (Mass/FragMass/RT/Isotope/Library/Formula/Ion Ratio)

Retention Time: 7.28 minutes **Exp RT:** 7.28 minutes
Precursor m/z : 609.1444 **Analyte Name:** 609.1444 / 7.28
Fit (%) 97.8% **RFit (%)** 100.0%

Collision Energy = 35 ± 15 eV

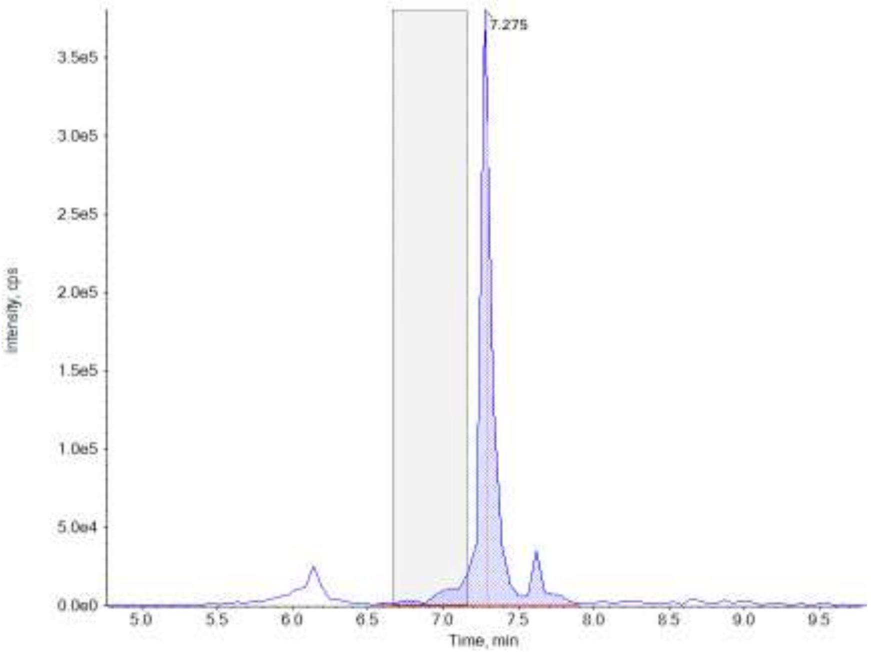

Acquired / Library MSMS

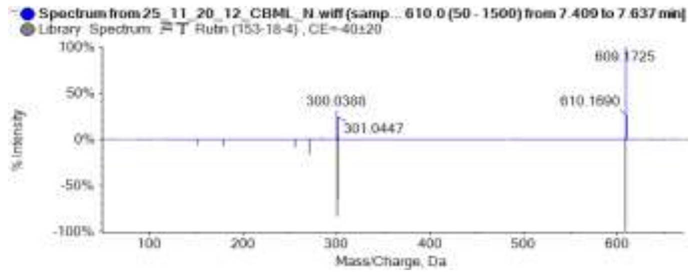

Acquired / Theoretical MS

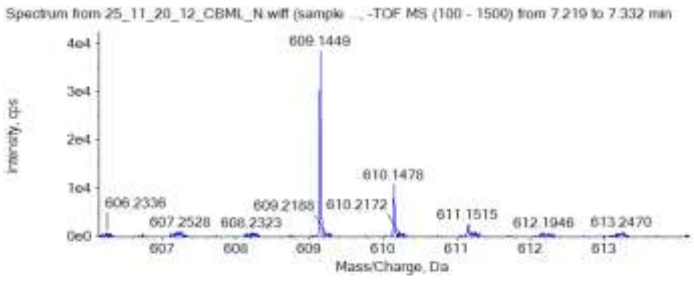

187.0957 / 7.45 (Mass/FragMass/RT/Isotope/Library/Formula/Ion Ratio)

**Retention Time:** 7.46 minutes	**Exp RT:** 7.45 minutes
**Precursor m/z :** 187.0957	**Analyte Name:**
**Fit (%)** 76.9% **RFit (%)** 100.0%	187.0957 / 7.45

Collision Energy = 35 ± 15 eV

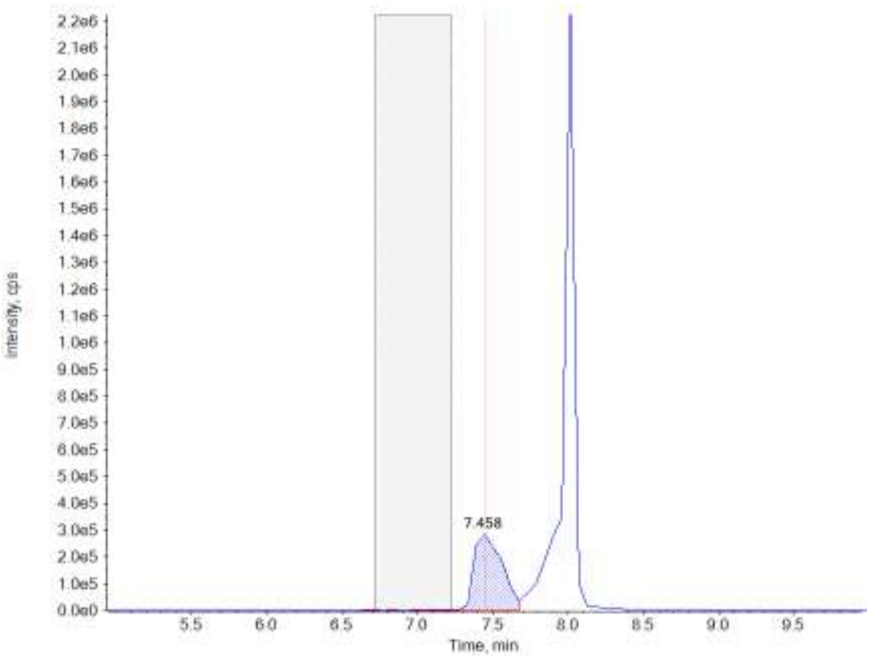

Acquired / Library MSMS

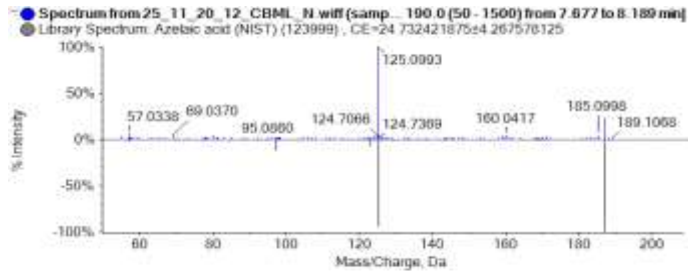

Acquired / Theoretical MS

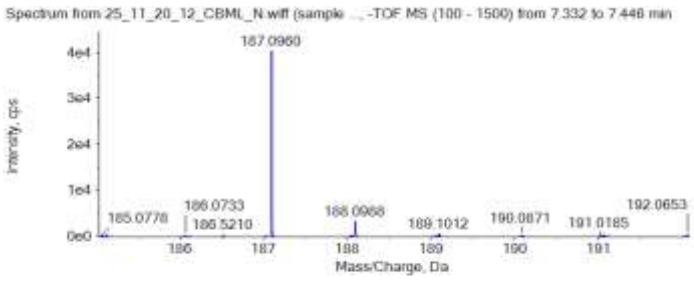

361.2209 / 7.45 (Mass/FragMass/RT/Isotope/Library/Formula/Ion Ratio)

Retention Time: 7.54 minutes
Precursor m/z : 361.2209
Fit (%) 67.8% **RFit (%)** 96.4%

Exp RT: 7.45 minutes
Analyte Name:
361.2209 / 7.45

Collision Energy = 35 ± 15 eV

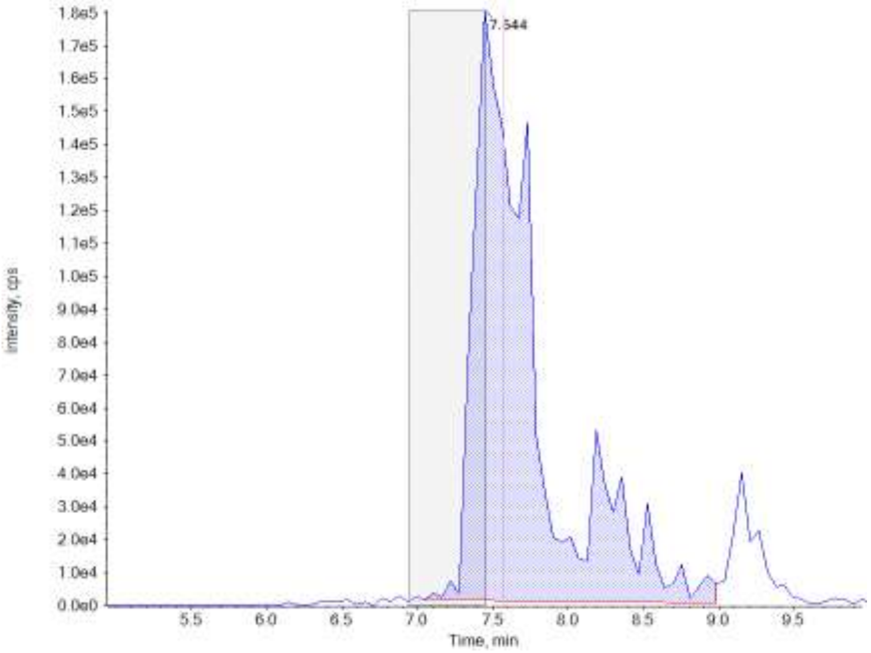

Acquired / Library MSMS

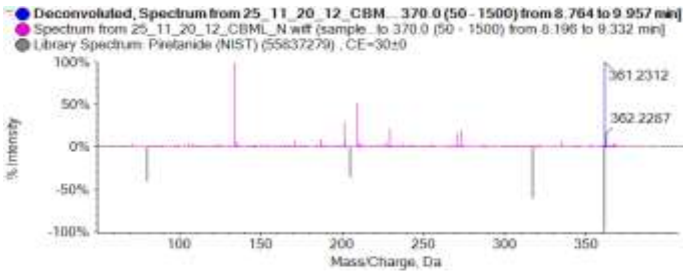

Acquired / Theoretical MS

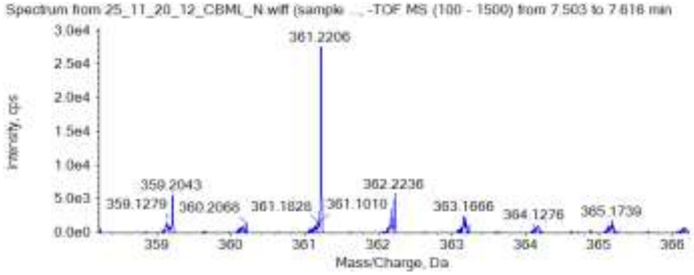

Created with SCIEX OS 3.3.0
Printed:12/9/2025 2:25:51 PM

463.0886 / 7.50 (Mass/FragMass/RT/Isotope/Library/Formula/Ion Ratio)

Retention Time: 7.51 minutes **Exp RT:** 7.50 minutes
Precursor m/z : 463.0886 **Analyte Name:**
Fit (%) 99.7% **RFit (%)** 99.0% 463.0886 / 7.50

Collision Energy = 35 ± 15 eV

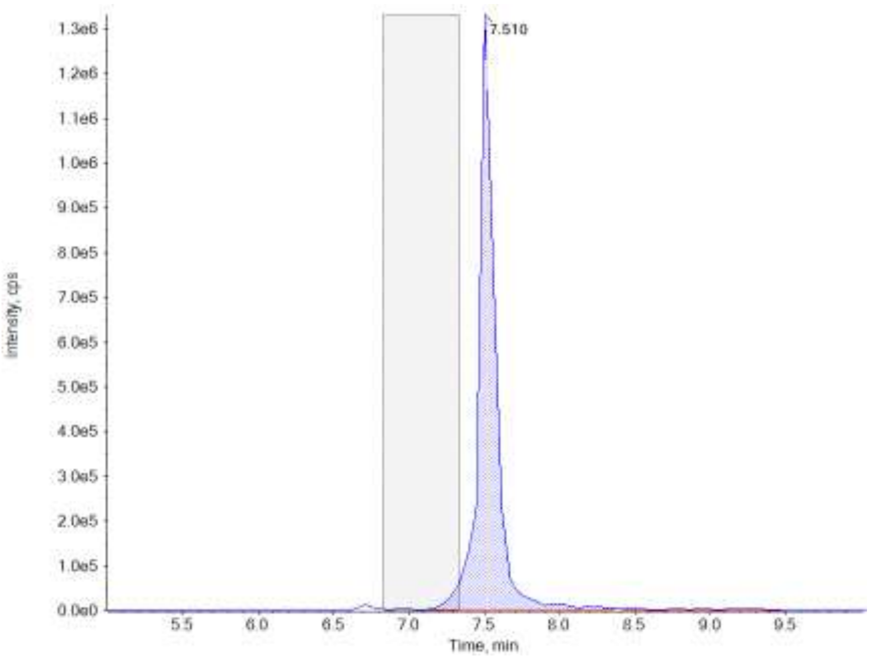

Acquired / Library MSMS

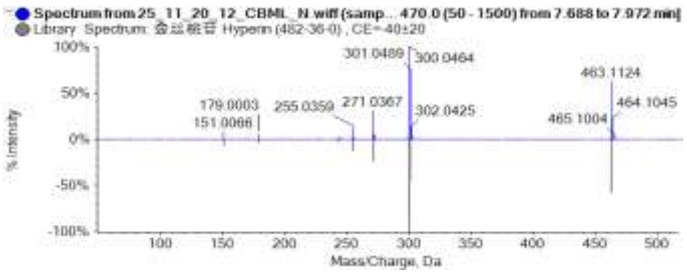

Acquired / Theoretical MS

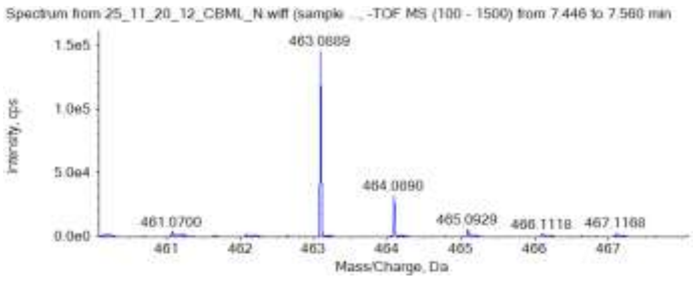

271.1533 / 7.62 (Mass/FragMass/RT/Isotope/Library/Formula/Ion Ratio)

**Retention Time:** 7.67 minutes	**Exp RT:** 7.62 minutes
**Precursor m/z :** 271.1534	**Analyte Name:**
**Fit (%)** 99.3% **RFit (%)** 89.5%	271.1533 / 7.62

Collision Energy = 35 ± 15 eV

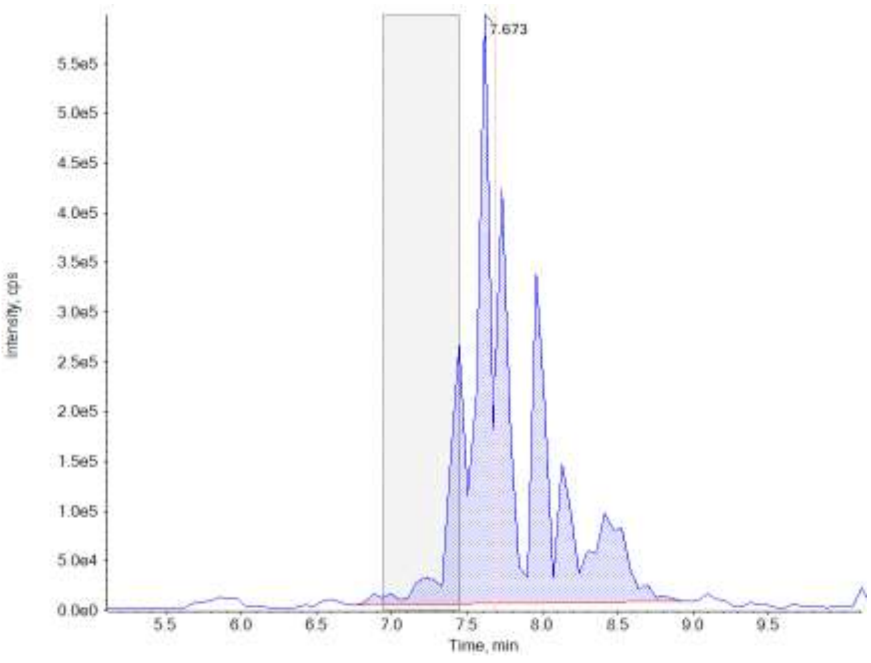

Acquired / Library MSMS

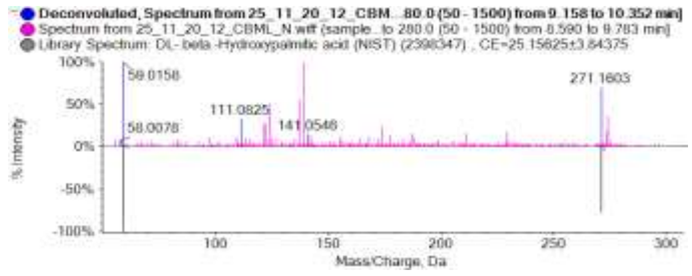

Acquired / Theoretical MS

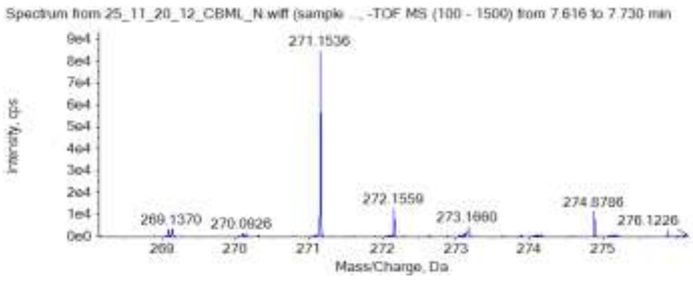

447.0963 / 7.96 (Mass/FragMass/RT/Isotope/Library/Formula/Ion Ratio)

**Retention Time:** 7.97 minutes<br>**Precursor m/z :** 447.0963<br>**Fit (%)** 100.0% **RFit (%)** 99.1%	**Exp RT:** 7.96 minutes<br>**Analyte Name:**<br>447.0963 / 7.96

Collision Energy = 35 ± 15 eV

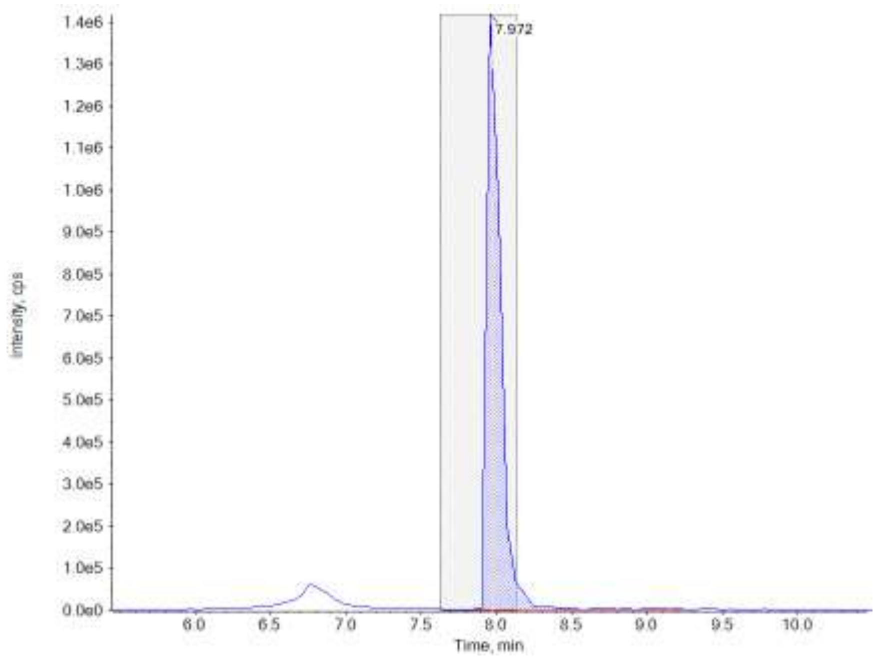

Acquired / Library MSMS

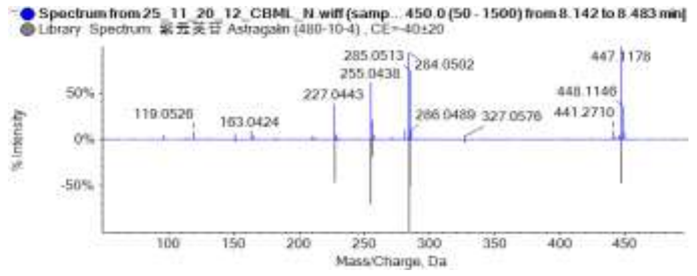

Acquired / Theoretical MS

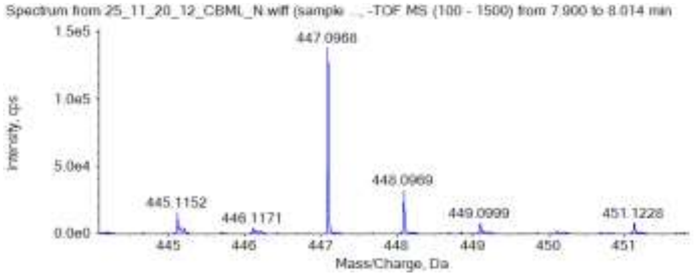

187.1020 / 8.01 (Mass/FragMass/RT/Isotope/Library/Formula/Ion Ratio)

**Retention Time:** 8.01 minutes<br>**Precursor m/z :** 187.1020<br>**Fit (%)** 100.0% **RFit (%)** 98.4%	**Exp RT:** 8.01 minutes<br>**Analyte Name:**<br>187.1020 / 8.01

Collision Energy = 35 ± 15 eV

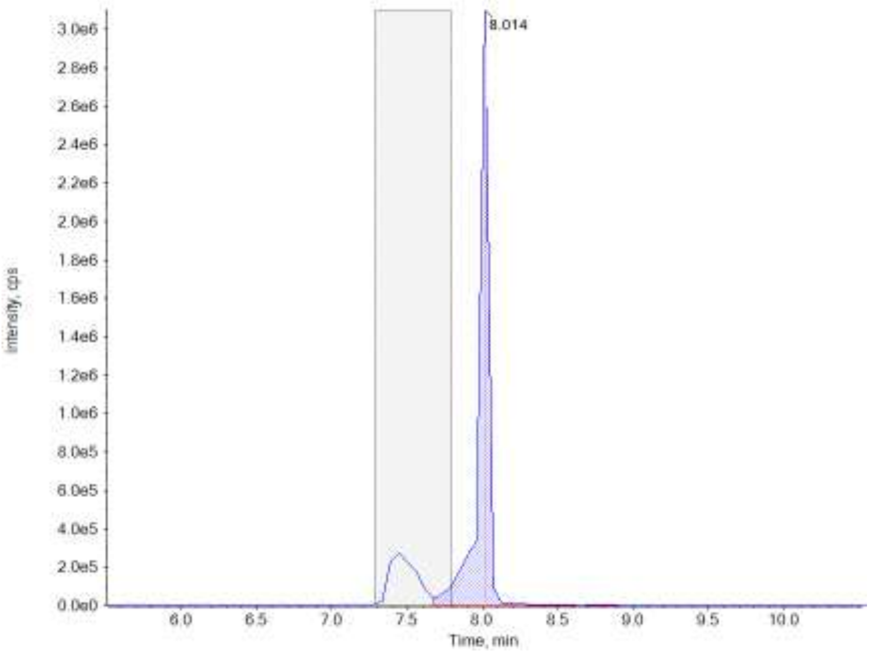

Acquired / Library MSMS

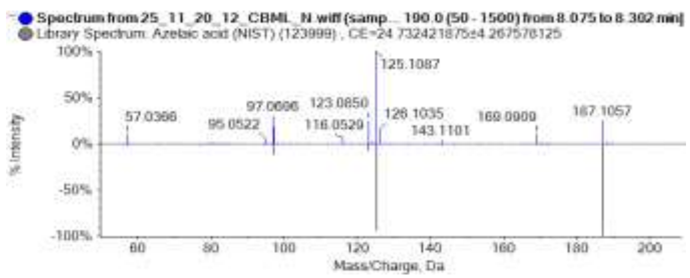

Acquired / Theoretical MS

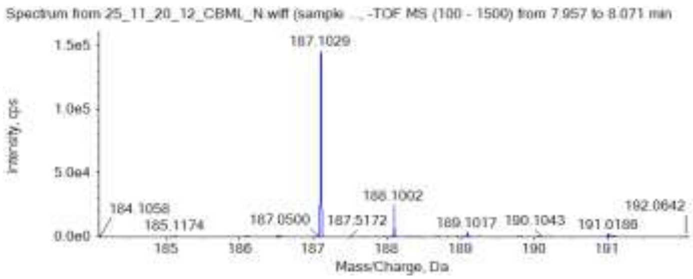

144.0446 / 8.13 (Mass/FragMass/RT/Isotope/Library/Formula/Ion Ratio)

Retention Time: 8.11 minutes
Precursor m/z : 144.0446
Fit (%) 96.9% **RFit (%)** 84.5%

Exp RT: 8.13 minutes
Analyte Name:
144.0446 / 8.13

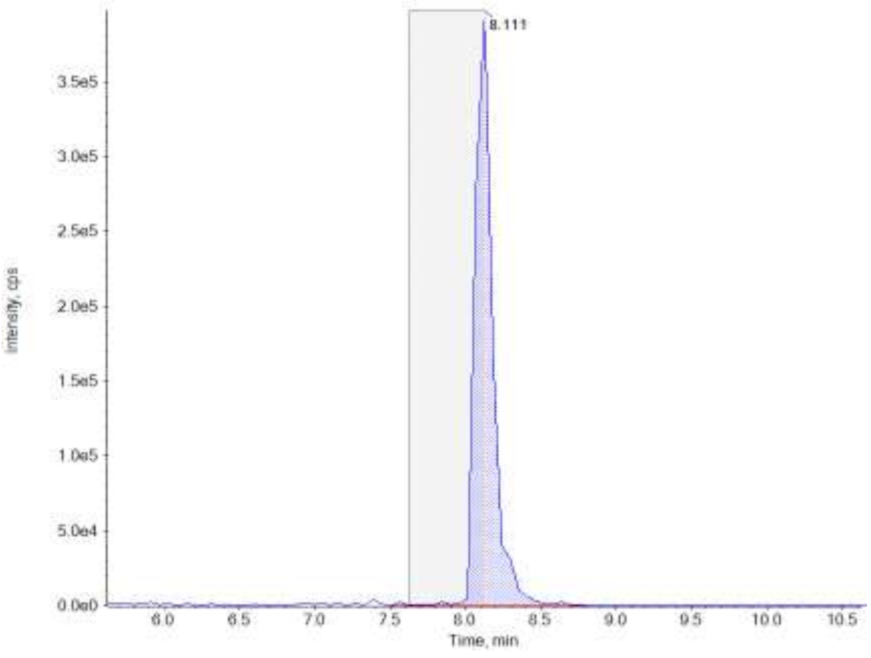

Collision Energy = 35 ± 15 eV

Acquired / Library MSMS

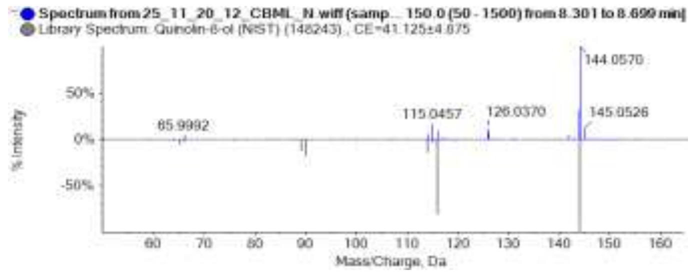

Acquired / Theoretical MS

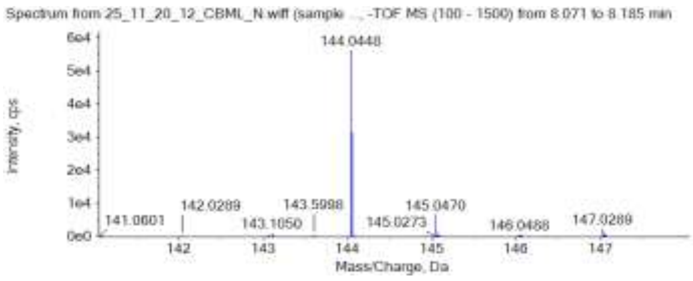

243.1328 / 8.13 (Mass/FragMass/RT/Isotope/Library/Formula/Ion Ratio)

Retention Time: 8.12 minutes
Precursor m/z : 243.1328
Fit (%) 97.3% **RFit (%)** 35.5%

Exp RT: 8.13 minutes
Analyte Name:
243.1328 / 8.13

Collision Energy = 35 ± 15 eV

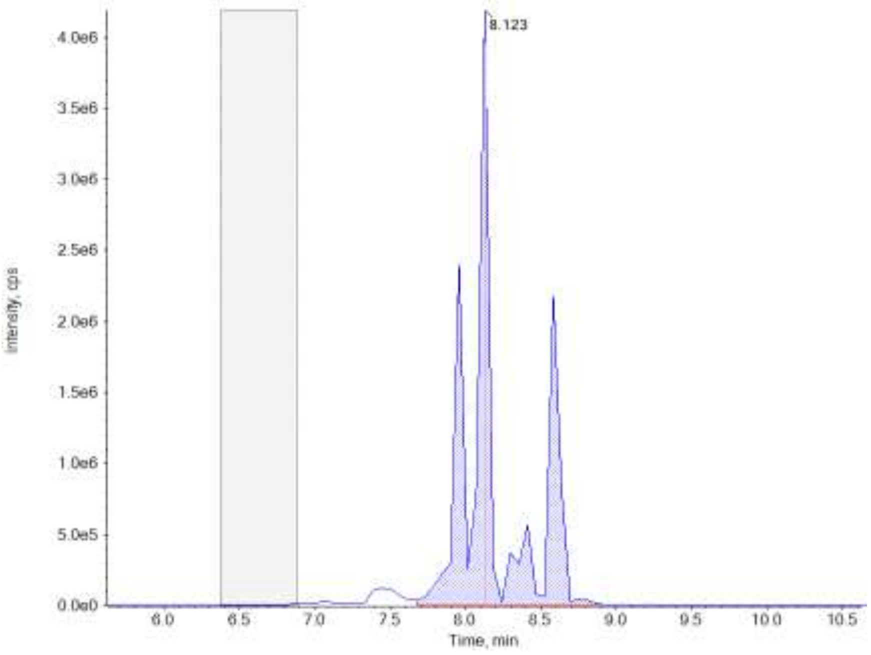

Acquired / Library MSMS

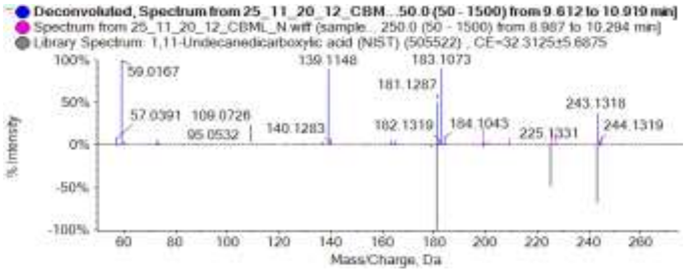

Acquired / Theoretical MS

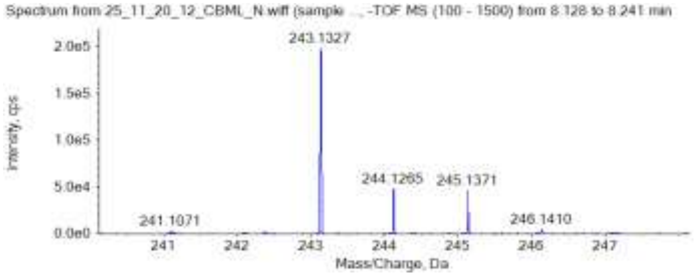

231.1598 / 8.24 (Mass/FragMass/RT/Isotope/Library/Formula/Ion Ratio)

Retention Time: 8.26 minutes | **Exp RT:** 8.24 minutes
Precursor m/z : 231.1598 | **Analyte Name:** 231.1598 / 8.24
Fit (%) 96.4% **RFit (%)** 98.6%

Collision Energy = 35 ± 15 eV

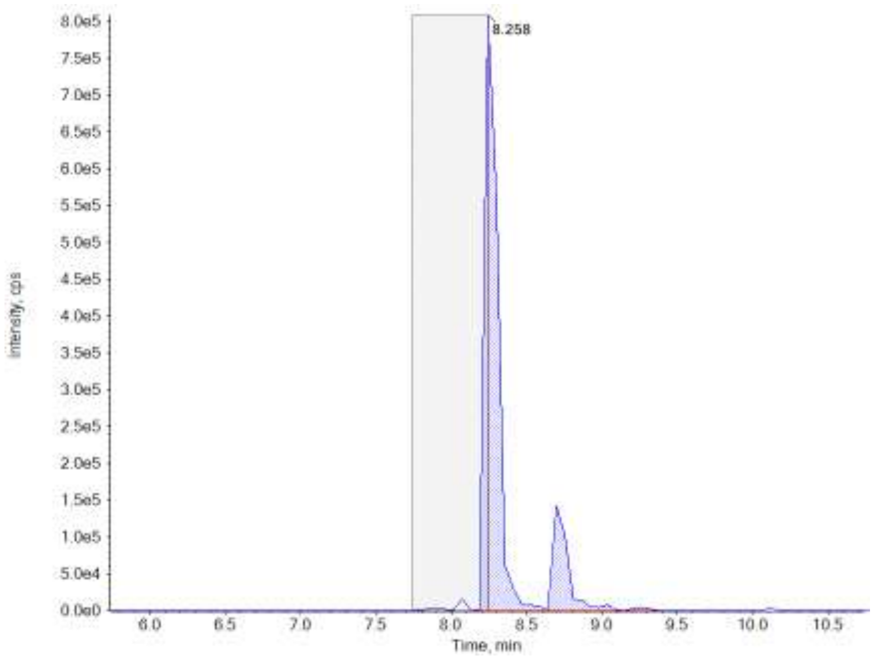

Acquired / Library MSMS

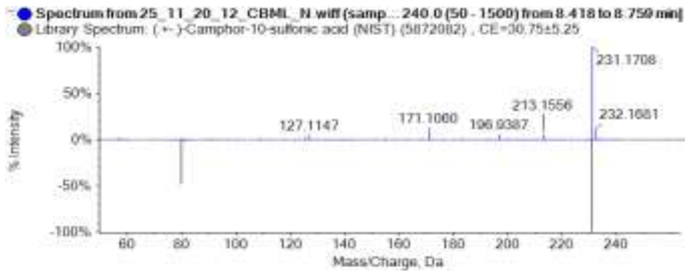

Acquired / Theoretical MS

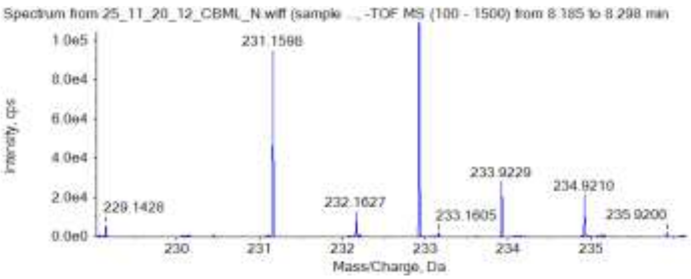

373.1857 / 8.24 (Mass/FragMass/RT/Isotope/Library/Formula/Ion Ratio)

Retention Time: 8.24 minutes
Precursor m/z : 373.1857
Fit (%) 39.9% **RFit (%)** 99.7%

Exp RT: 8.24 minutes
Analyte Name:
373.1857 / 8.24

Collision Energy = 35 ± 15 eV

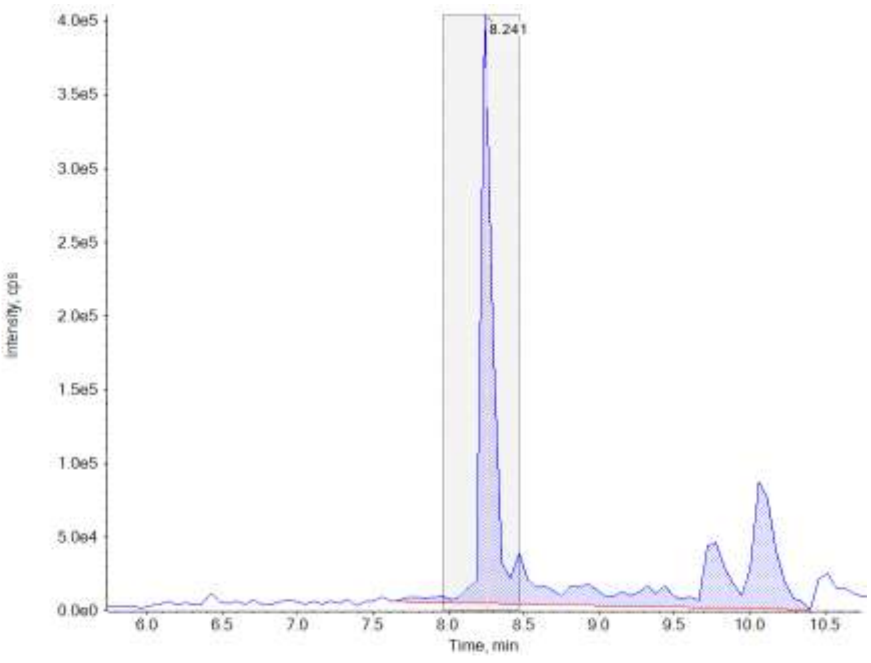

Acquired / Library MSMS

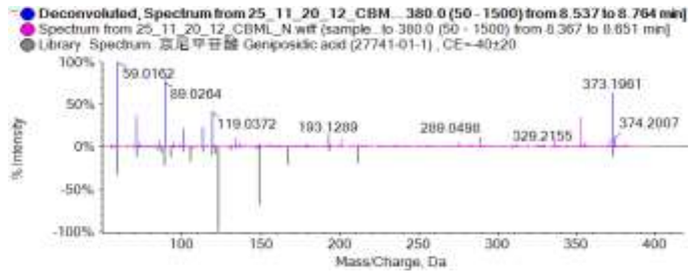

Acquired / Theoretical MS

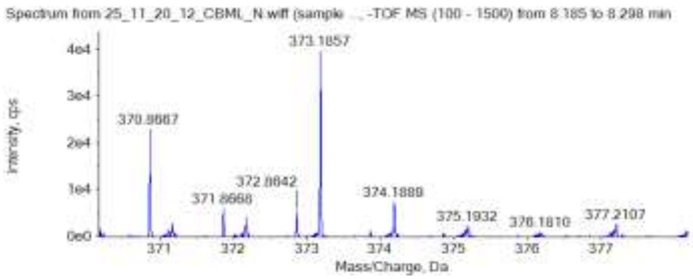

173.1191 / 8.36 (Mass/FragMass/RT/Isotope/Library/Formula/Ion Ratio)

**Retention Time:** 8.36 minutes	**Exp RT:** 8.36 minutes
**Precursor m/z :** 173.1191	**Analyte Name:**
**Fit (%)** 73.4% **RFit (%)** 100.0%	173.1191 / 8.36

Collision Energy = 35 ± 15 eV

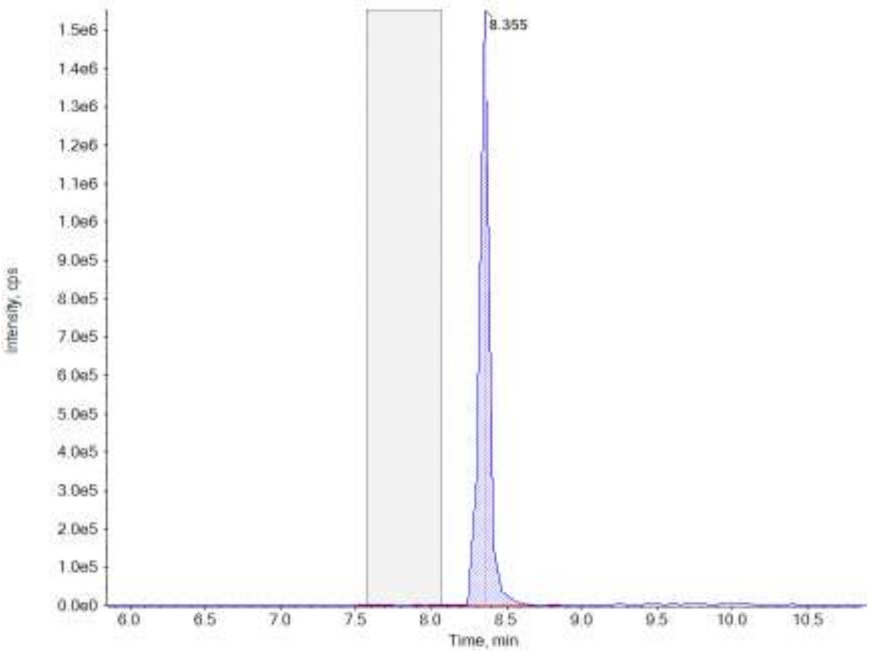

Acquired / Library MSMS

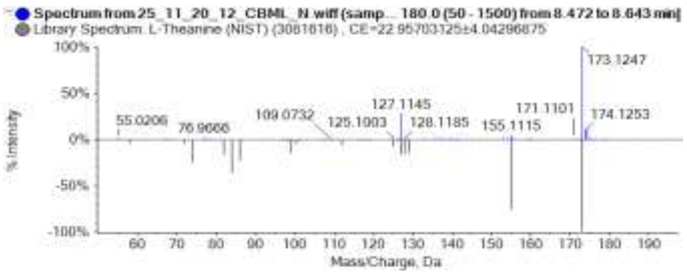

Acquired / Theoretical MS

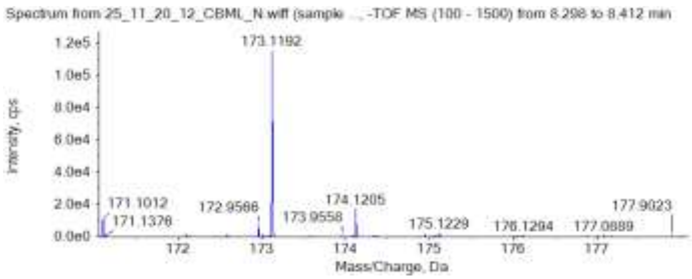

478.1970 / 8.36 (Mass/FragMass/RT/Isotope/Library/Formula/Ion Ratio)

Retention Time: 8.36 minutes
Precursor m/z : 478.1970
Fit (%) 73.8% **RFit (%)** 65.2%

Exp RT: 8.36 minutes
Analyte Name:
478.1970 / 8.36

Collision Energy = 35 ± 15 eV

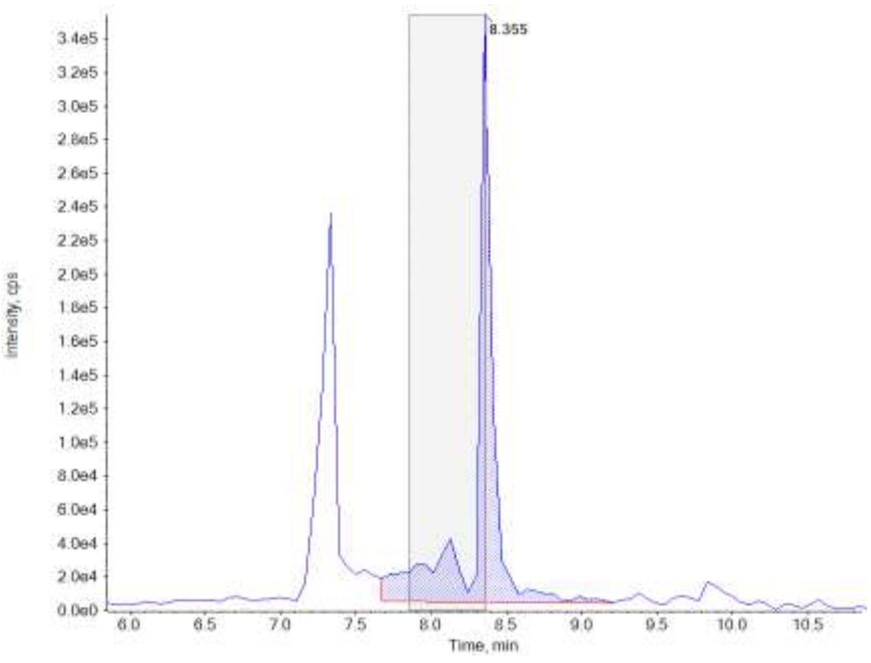

Acquired / Library MSMS

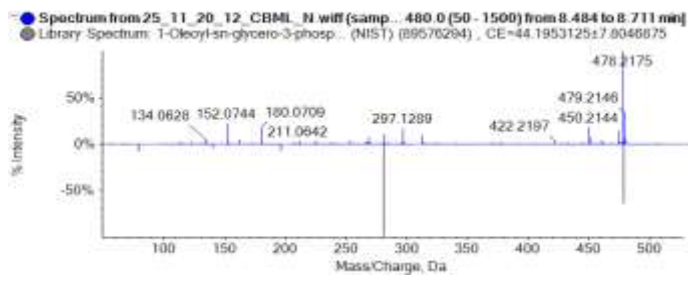

Acquired / Theoretical MS

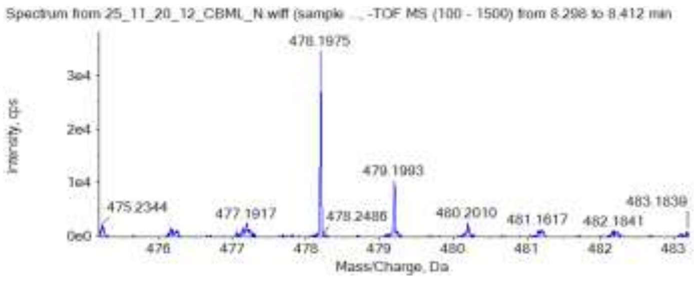

381.1546 / 8.41 [M-H]- (Mass/FragMass/RT/Isotope/Library/Formula/Ion Ratio)

**Retention Time:** 8.41 minutes	**Exp RT:** 8.41 minutes
**Precursor m/z :** 381.1546	**Analyte Name:**
**Fit (%)** 41.2% **RFit (%)** 94.4%	381.1546 / 8.41 [M-H]-

Collision Energy = 35 ± 15 eV

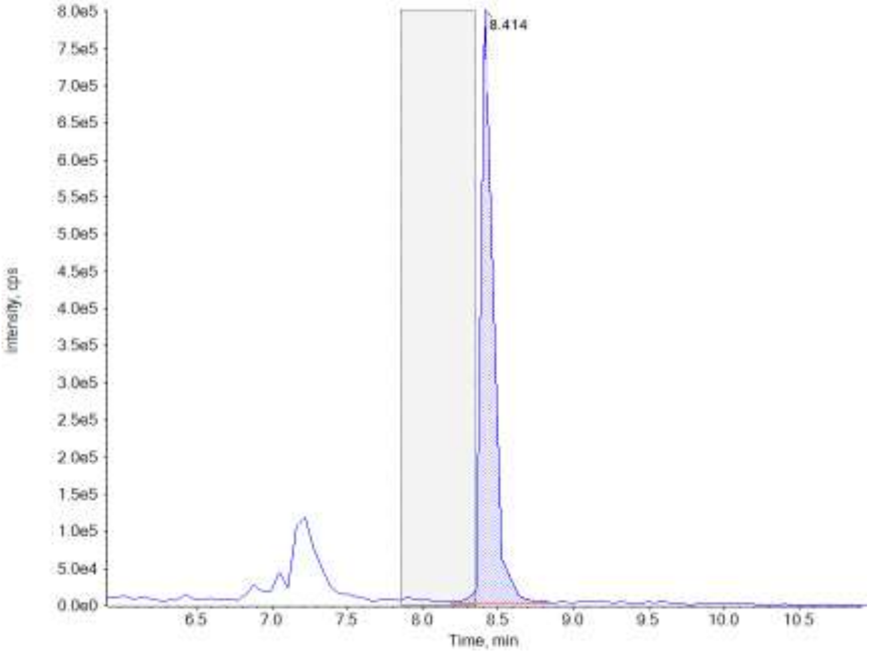

Acquired / Library MSMS

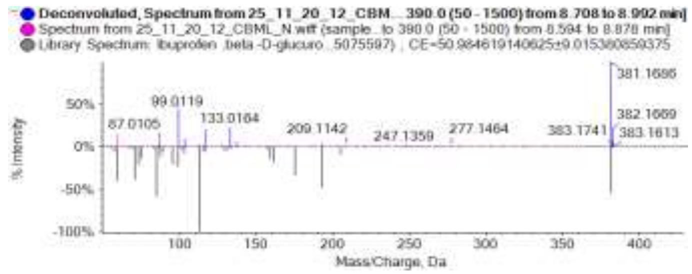

Acquired / Theoretical MS

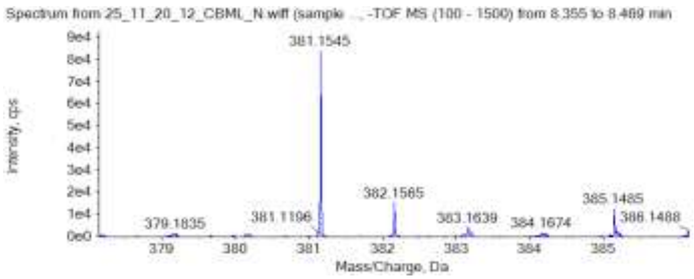

Created with SCIEX OS 3.3.0
Printed:12/9/2025 2:25:51 PM

417.1308 / 8.41 [M+Cl]- (Mass/FragMass/RT/Isotope/Library/Formula/Ion Ratio)

Retention Time: 8.41 minutes
Precursor m/z : 417.1308
Fit (%) 85.8% **RFit (%)** 77.2%

Exp RT: 8.41 minutes
Analyte Name:
417.1308 / 8.41 [M+Cl]-

Collision Energy = 35 ± 15 eV

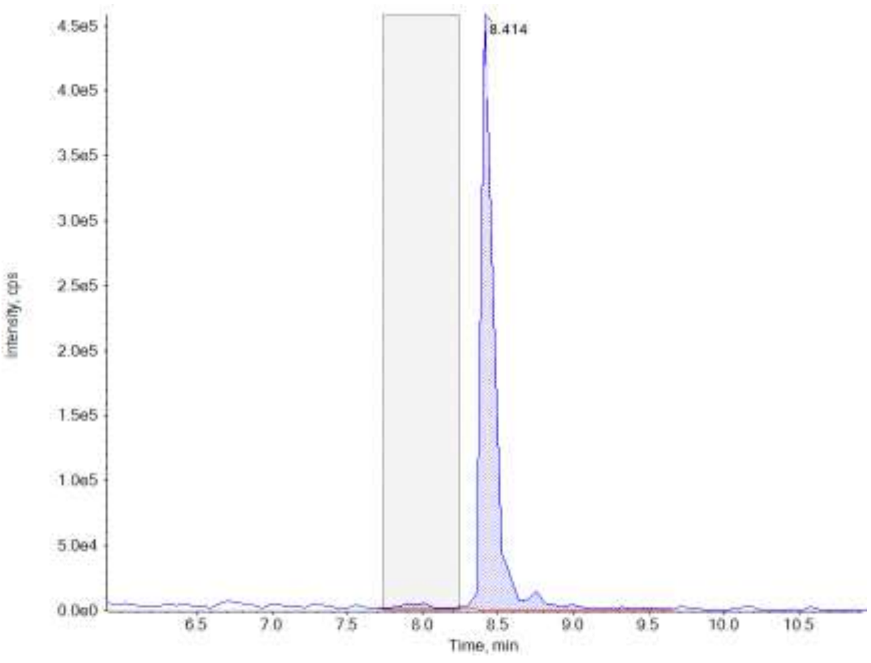

Acquired / Library MSMS

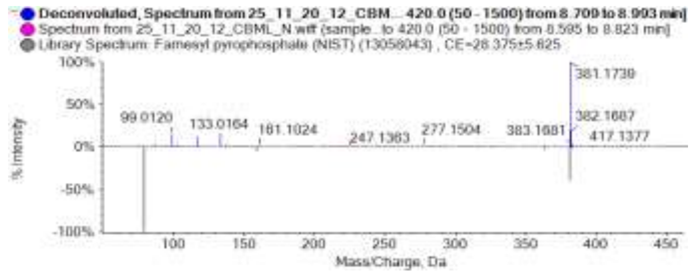

Acquired / Theoretical MS

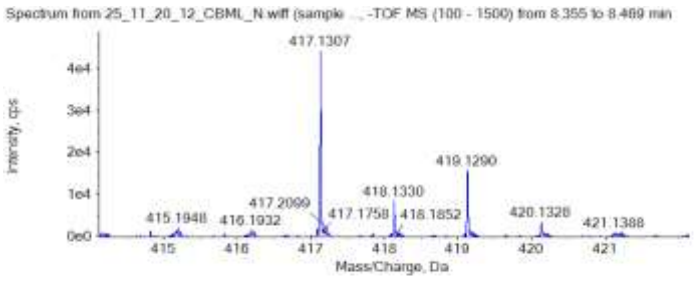

343.2128 / 8.53 (Mass/FragMass/RT/Isotope/Library/Formula/Ion Ratio)

**Retention Time:** 8.56 minutes	**Exp RT:** 8.53 minutes
**Precursor m/z :** 343.2128	**Analyte Name:**
**Fit (%)** 56.4% **RFit (%)** 96.7%	343.2128 / 8.53

Collision Energy = 35 ± 15 eV

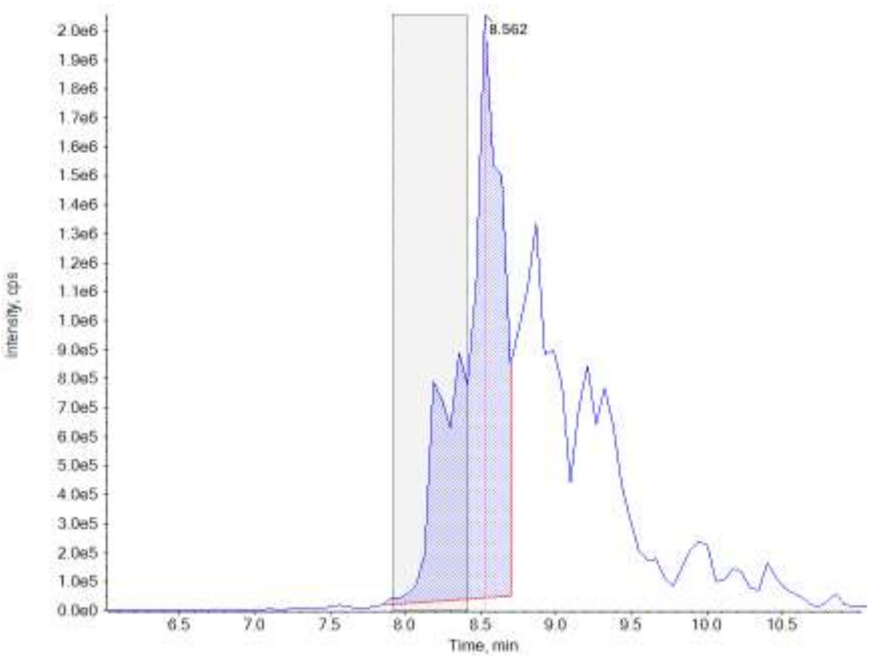

Acquired / Library MSMS

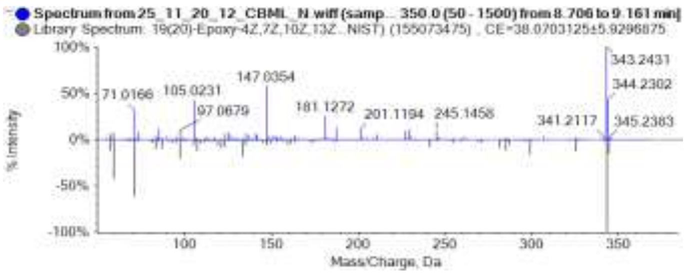

Acquired / Theoretical MS

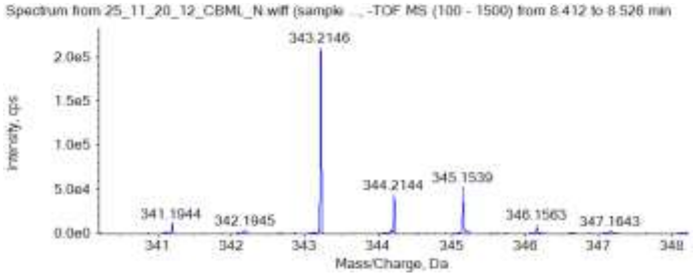

Created with SCIEX OS 3.3.0
Printed:12/9/2025 2:25:51 PM

113.0595 / 8.64 (Mass/FragMass/RT/Isotope/Library/Formula/Ion Ratio)

Retention Time: 8.46 minutes
Precursor m/z : 113.0595
Fit (%) 34.7% **RFit (%)** 100.0%

Exp RT: 8.64 minutes
Analyte Name:
113.0595 / 8.64

Collision Energy = 35 ± 15 eV

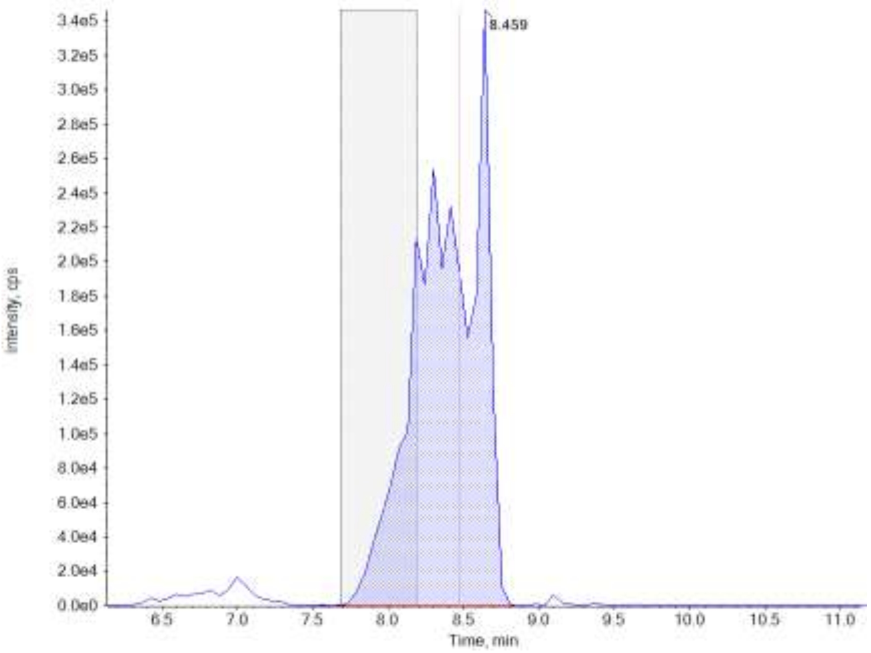

Acquired / Library MSMS

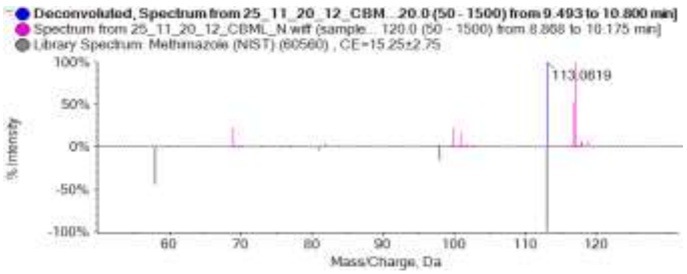

Acquired / Theoretical MS

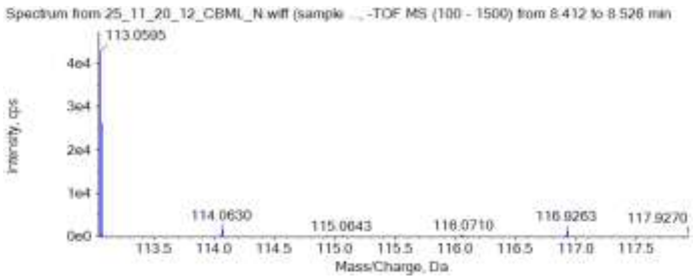

Created with SCIEX OS 3.3.0
Printed:12/9/2025 2:25:51 PM

665.3546 / 8.92 (Mass/FragMass/RT/Isotope/Library/Formula/Ion Ratio)

**Retention Time:** 8.91 minutes<br>**Precursor m/z :** 665.3546<br>**Fit (%)** 87.7% **RFit (%)** 100.0%	**Exp RT:** 8.92 minutes<br>**Analyte Name:**<br>665.3546 / 8.92

		**Collision Energy = 35 ± 15 eV**
	**Acquired / Library MSMS**	
	**Acquired / Theoretical MS**	

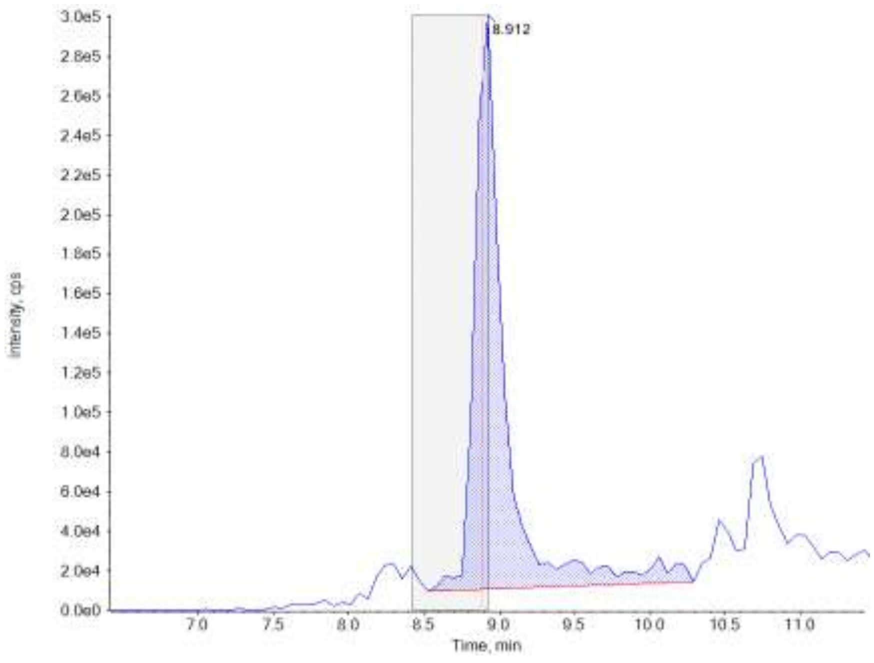

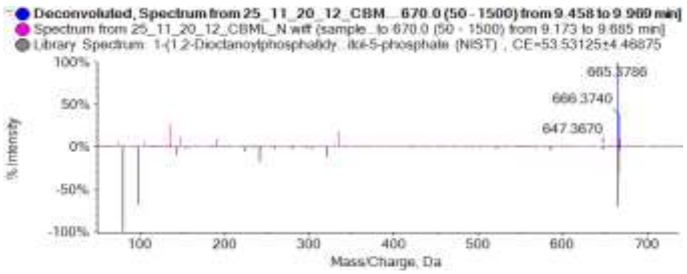

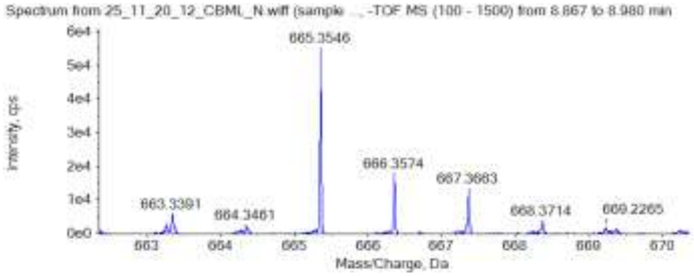

263.1303 / 9.09 (Mass/FragMass/RT/Isotope/Library/Formula/Ion Ratio)

**Retention Time:** 9.09 minutes<br>**Precursor m/z :** 263.1303<br>**Fit (%)** 98.0% **RFit (%)** 99.8%	**Exp RT:** 9.09 minutes<br>**Analyte Name:**<br>263.1303 / 9.09

Collision Energy = 35 ± 15 eV

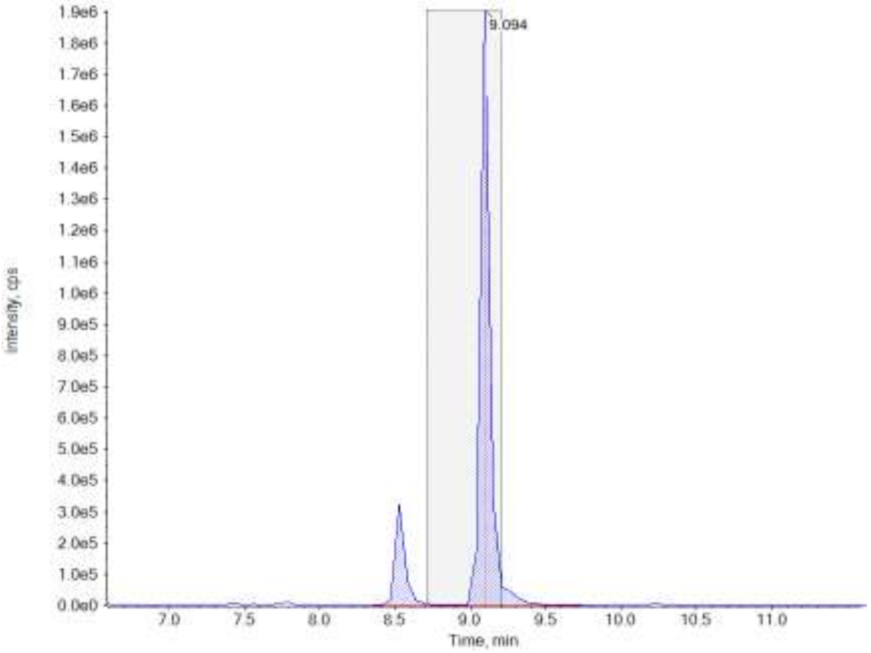

Acquired / Library MSMS

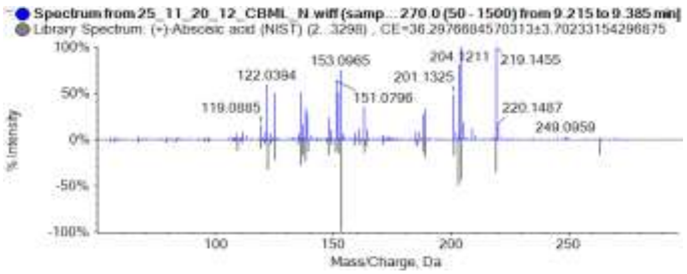

Acquired / Theoretical MS

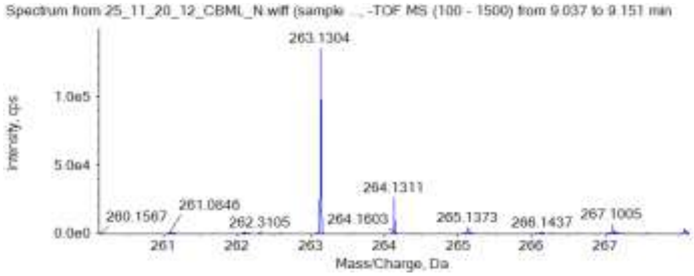

329.1591 / 9.38 (Mass/FragMass/RT/Isotope/Library/Formula/Ion Ratio)

Retention Time: 9.35 minutes
Precursor m/z : 329.1591
Fit (%) 78.5% **RFit (%)** 96.5%

Exp RT: 9.38 minutes
Analyte Name:
329.1591 / 9.38

Collision Energy = 35 ± 15 eV

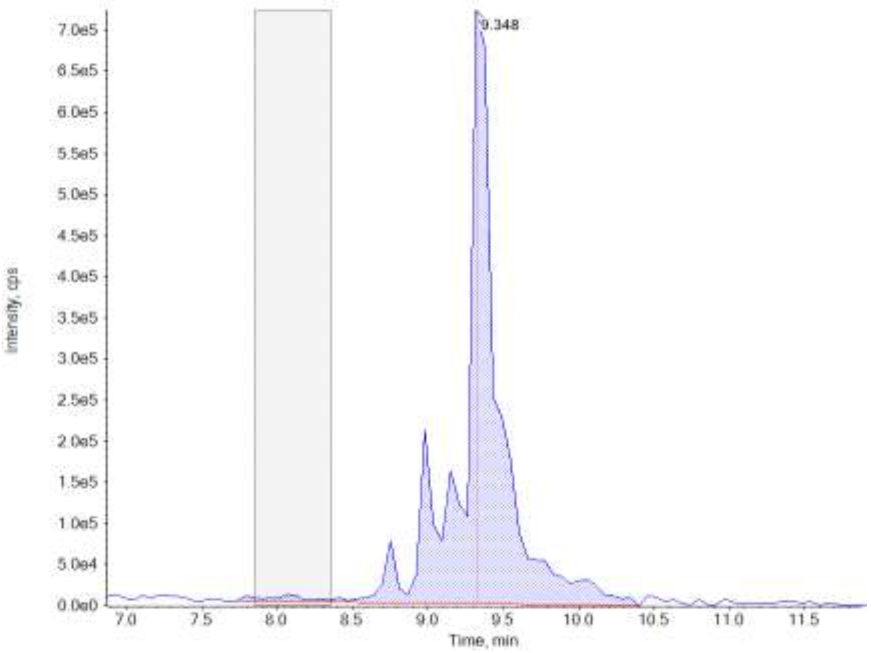

Acquired / Library MSMS

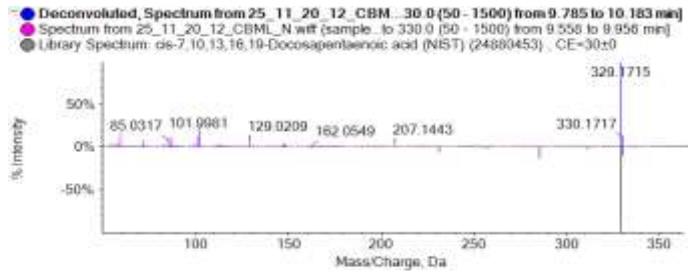

Acquired / Theoretical MS

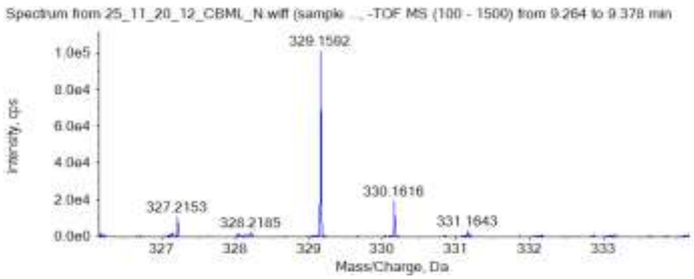

147.0443 / 9.49 (Mass/FragMass/RT/Isotope/Library/Formula/Ion Ratio)

Retention Time: 9.47 minutes
Precursor m/z : 147.0443
Fit (%) 99.5% **RFit (%)** 100.0%

Exp RT: 9.49 minutes
Analyte Name:
147.0443 / 9.49

Collision Energy = 35 ± 15 eV

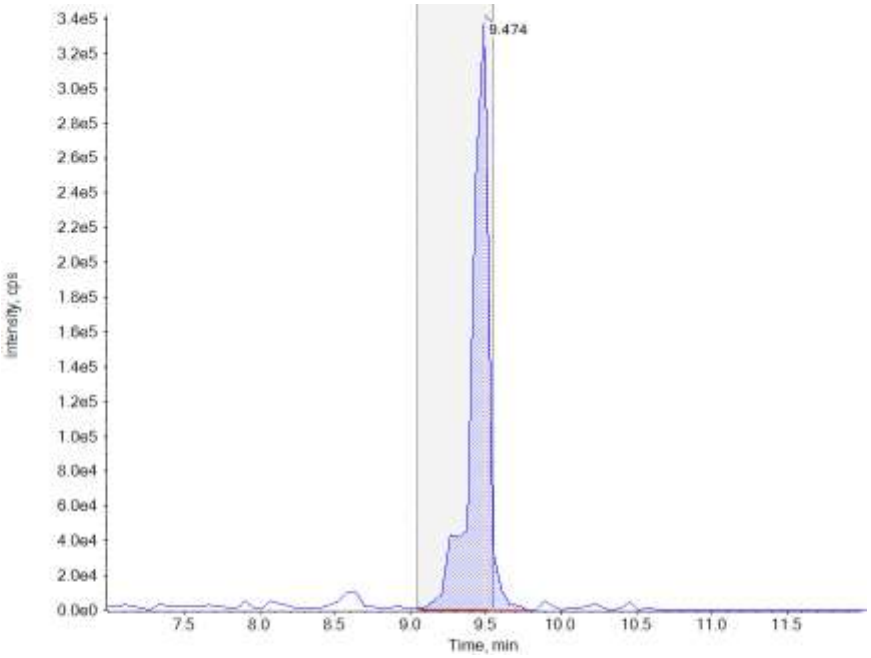

Acquired / Library MSMS

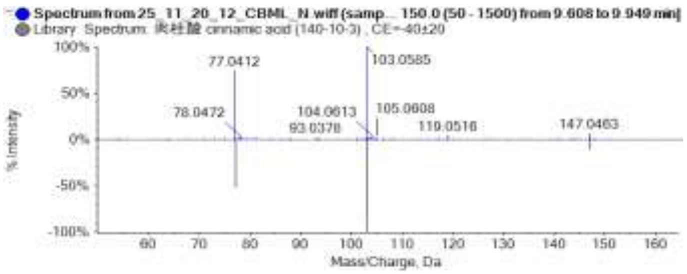

Acquired / Theoretical MS

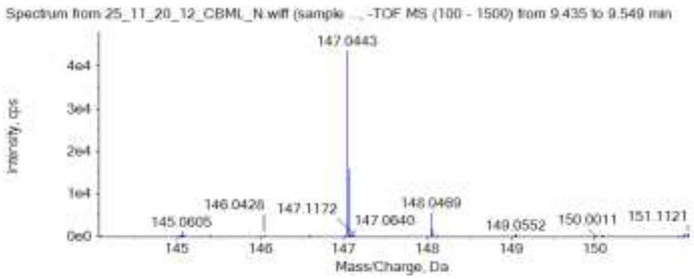

Created with SCIEX OS 3.3.0
Printed:12/9/2025 2:25:51 PM

225.1128 / 9.78 (Mass/FragMass/RT/Isotope/Library/Formula/Ion Ratio)

Retention Time: 9.78 minutes
Precursor m/z : 225.1128
Fit (%) 97.9% **RFit (%)** 56.4%

Exp RT: 9.78 minutes
Analyte Name:
225.1128 / 9.78

Collision Energy = 35 ± 15 eV

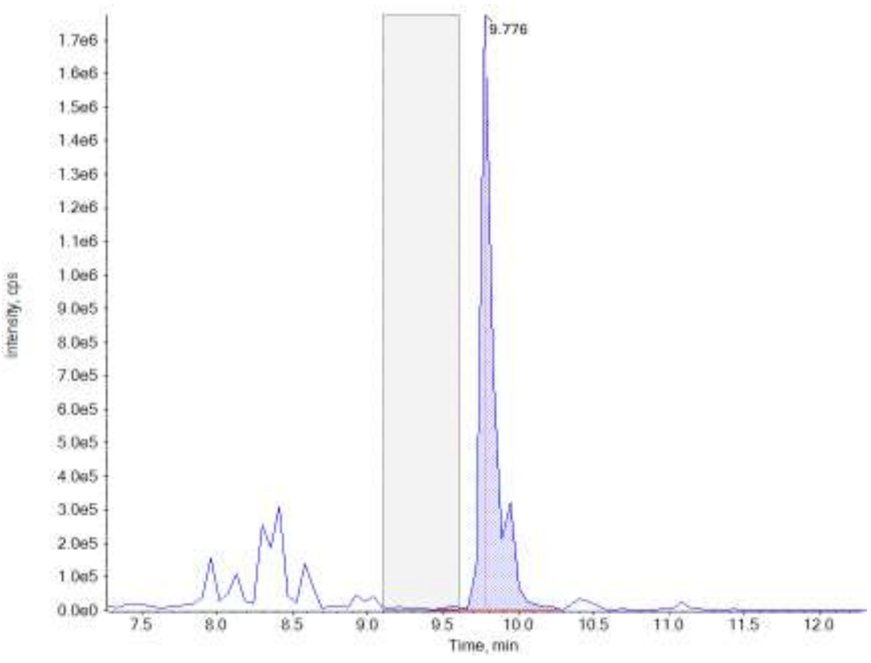

Acquired / Library MSMS

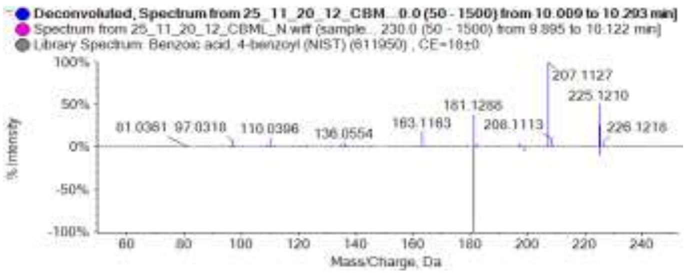

Acquired / Theoretical MS

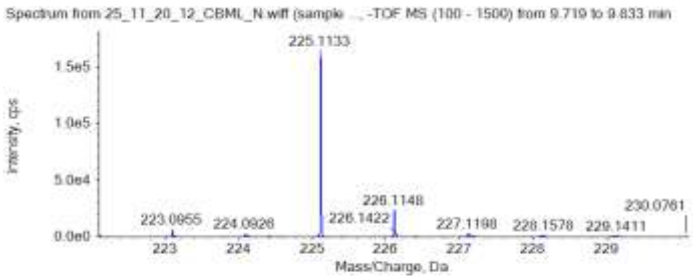

Created with SCIEX OS 3.3.0
Printed:12/9/2025 2:25:51 PM

745.3439 / 9.78 (Mass/FragMass/RT/Isotope/Library/Formula/Ion Ratio)

Retention Time: 9.80 minutes **Exp RT:** 9.78 minutes
Precursor m/z : 745.3439 **Analyte Name:**
Fit (%) 64.6% **RFit (%)** 90.8% 745.3439 / 9.78

Collision Energy = 35 ± 15 eV

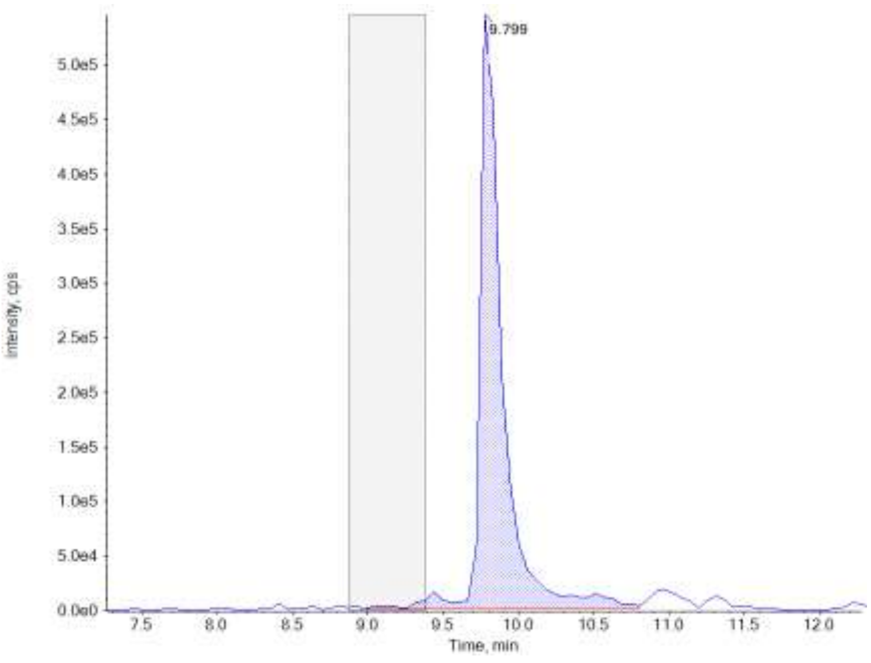

Acquired / Library MSMS

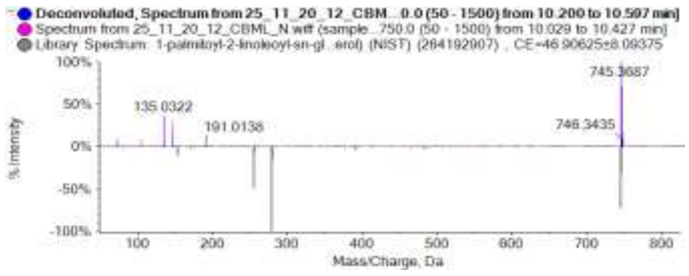

Acquired / Theoretical MS

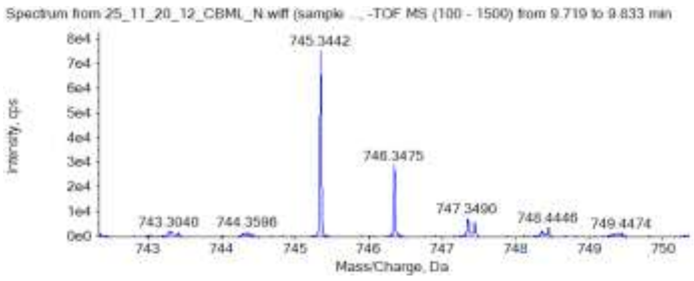

213.1116 / 9.89 (Mass/FragMass/RT/Isotope/Library/Formula/Ion Ratio)

Retention Time: 9.89 minutes
Precursor m/z : 213.1116
Fit (%) 82.9% **RFit (%)** 100.0%

Exp RT: 9.89 minutes
Analyte Name:
213.1116 / 9.89

Collision Energy = 35 ± 15 eV

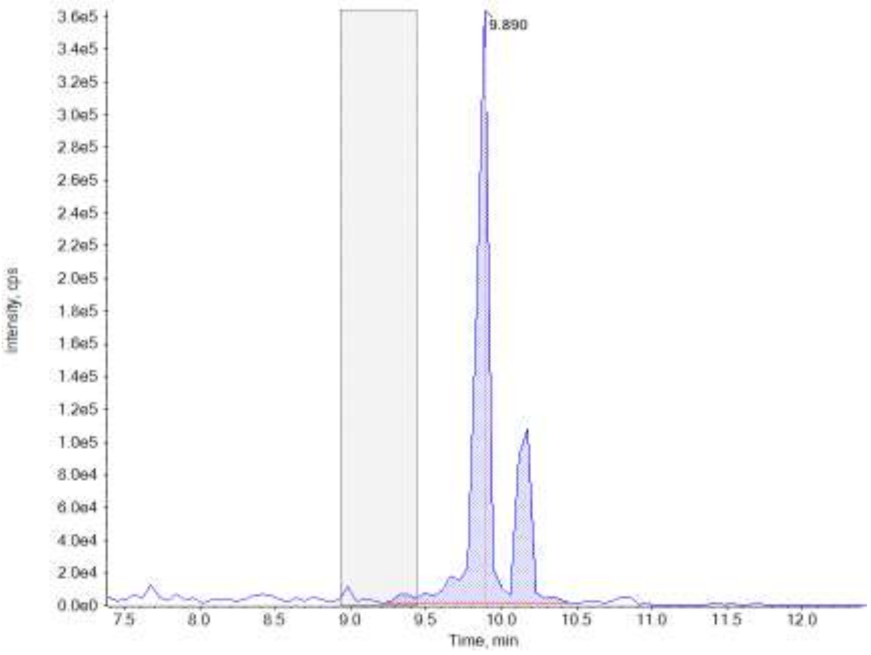

Acquired / Library MSMS

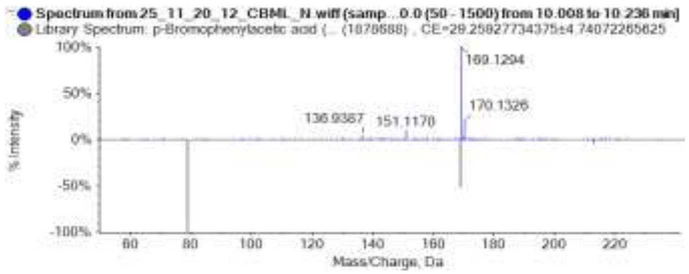

Acquired / Theoretical MS

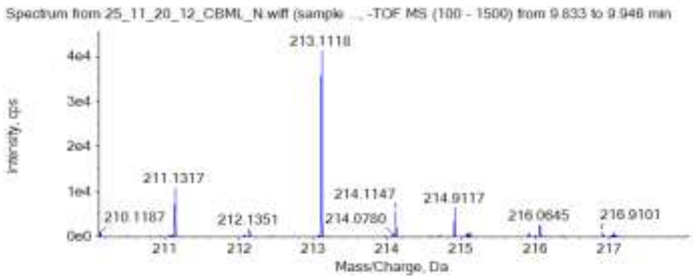

777.4173 / 9.95 [2M-H]- (Mass/FragMass/RT/Isotope/Library/Formula/Ion Ratio)

**Retention Time:** 9.96 minutes	**Exp RT:** 9.95 minutes
**Precursor m/z :** 777.4168	**Analyte Name:**
**Fit (%)** 100.0% **RFit (%)** 20.3%	777.4173 / 9.95 [2M-H]-

Collision Energy = 35 ± 15 eV

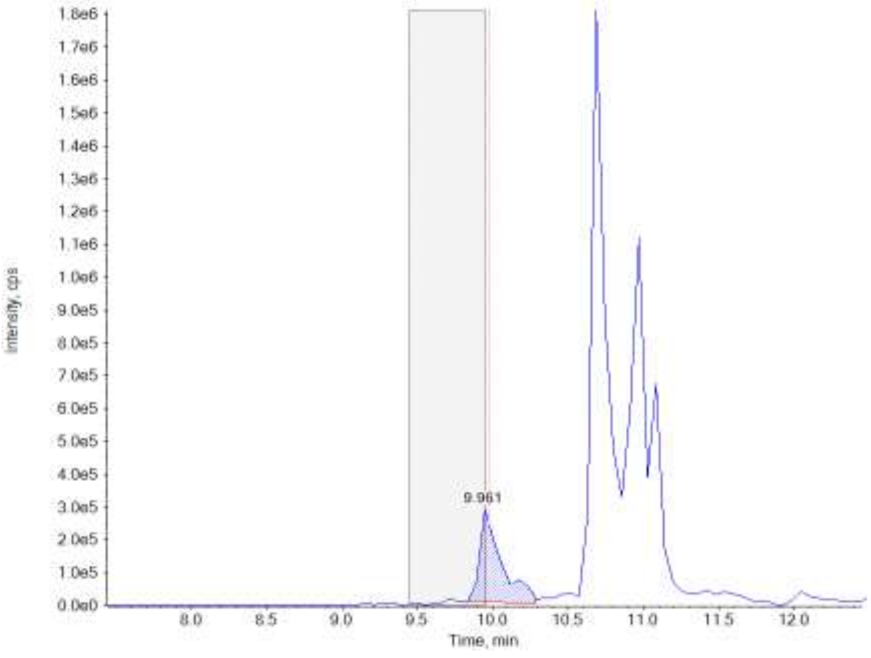

Acquired / Library MSMS

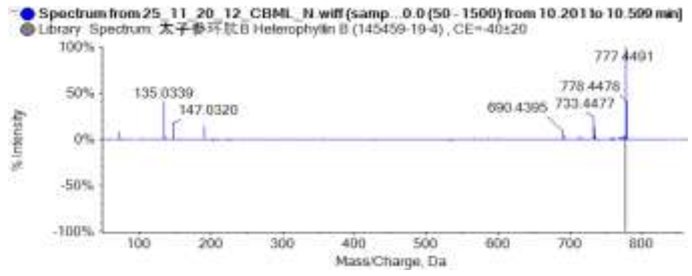

Acquired / Theoretical MS

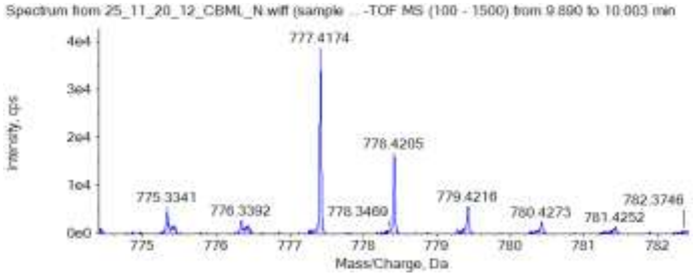

Created with SCIEX OS 3.3.0
Printed:12/9/2025 2:25:51 PM

609.2714 / 10.17 (Mass/FragMass/RT/Isotope/Library/Formula/Ion Ratio)

**Retention Time:** 10.17 minutes	**Exp RT:** 10.17 minutes
**Precursor m/z :** 609.2714	**Analyte Name:**
**Fit (%)** 92.3% **RFit (%)** 89.6%	609.2714 / 10.17

Collision Energy = 35 ± 15 eV

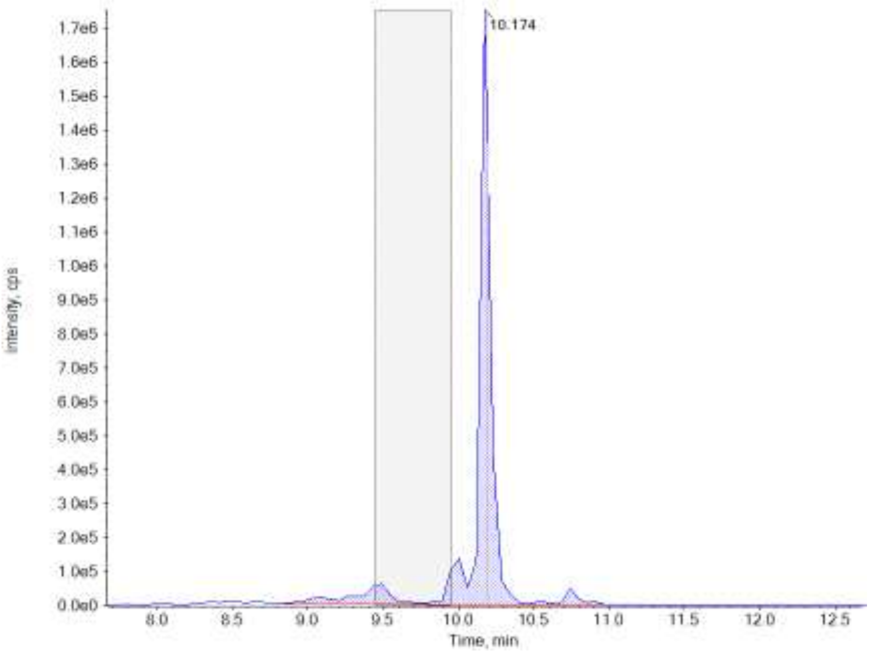

Acquired / Library MSMS

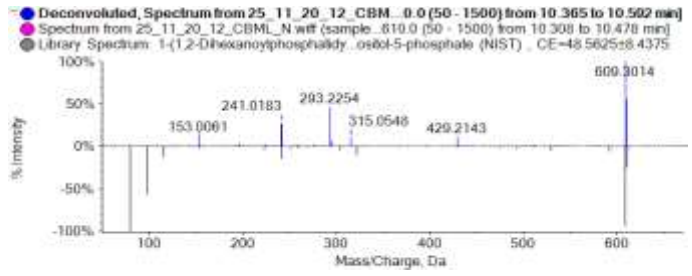

Acquired / Theoretical MS

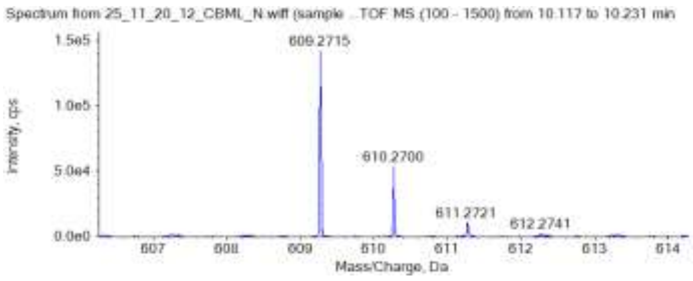

593.2624 / 10.34 (Mass/FragMass/RT/Isotope/Library/Formula/Ion Ratio)

Retention Time: 10.36 minutes **Exp RT:** 10.34 minutes
Precursor m/z : 593.2624 **Analyte Name:**
Fit (%) 20.2% **RFit (%)** 100.0% 593.2624 / 10.34

Collision Energy = 35 ± 15 eV

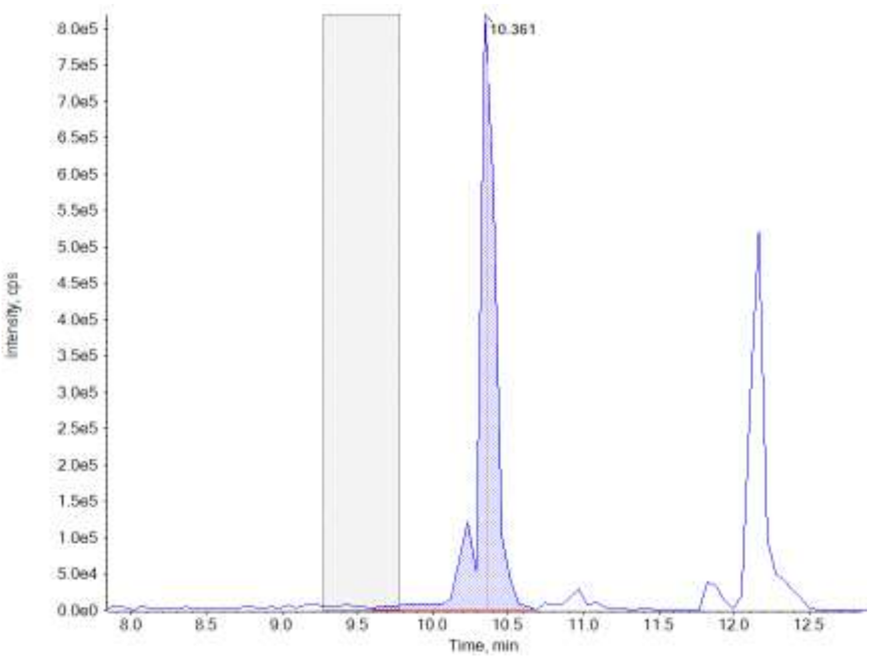

Acquired / Library MSMS

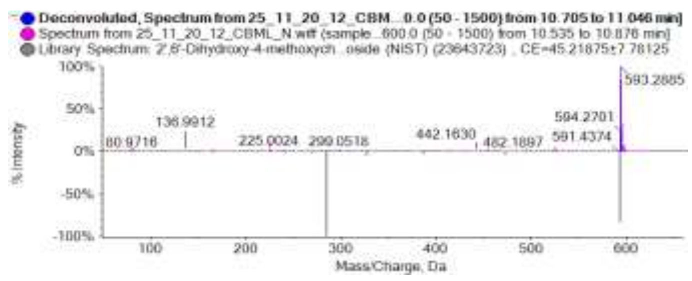

Acquired / Theoretical MS

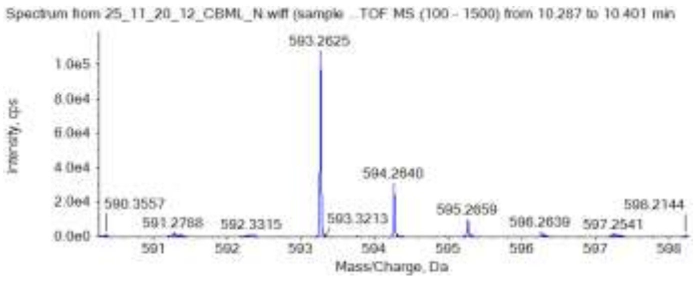

645.3626 / 10.51 (Mass/FragMass/RT/Isotope/Library/Formula/Ion Ratio)

**Retention Time:** 10.55 minutes	**Exp RT:** 10.52 minutes
**Precursor m/z :** 645.3626	**Analyte Name:**
**Fit (%)** 27.6% **RFit (%)** 98.4%	645.3626 / 10.51

Collision Energy = 35 ± 15 eV

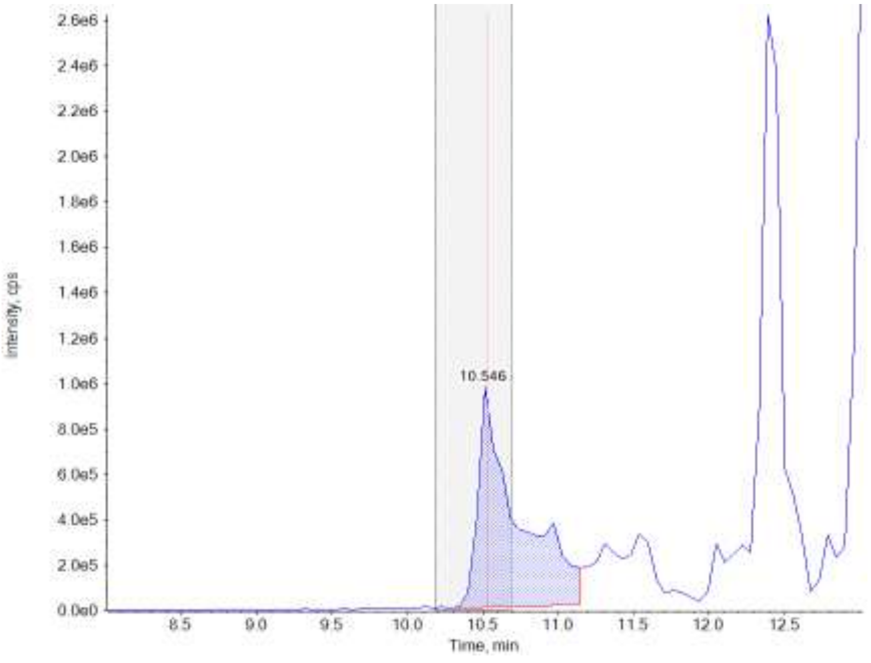

Acquired / Library MSMS

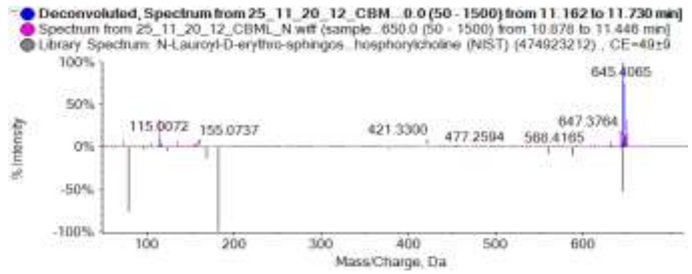

Acquired / Theoretical MS

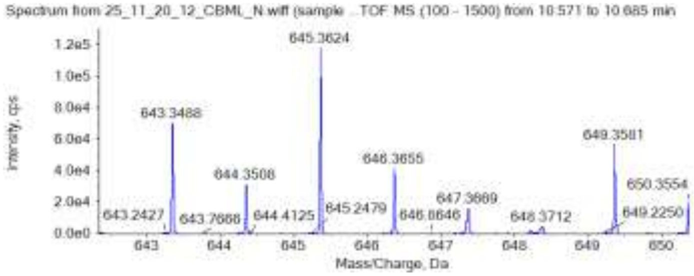

388.2046 / 10.69 (Mass/FragMass/RT/Isotope/Library/Formula/Ion Ratio)

Retention Time: 10.74 minutes
Precursor m/z : 388.2046
Fit (%) 88.8% **RFit (%)** 88.5%

Exp RT: 10.69 minutes
Analyte Name:
388.2046 / 10.69

Collision Energy = 35 ± 15 eV

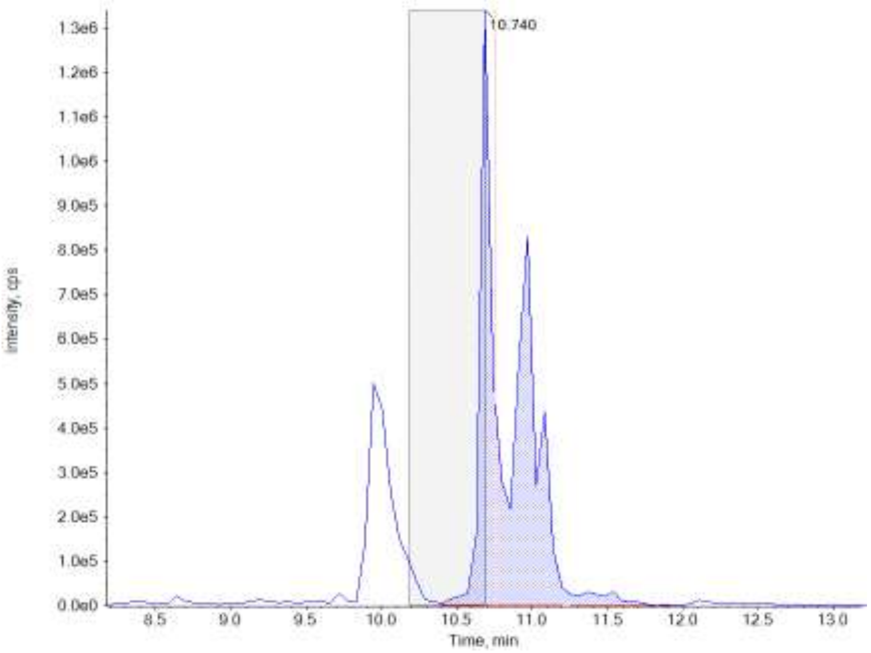

Acquired / Library MSMS

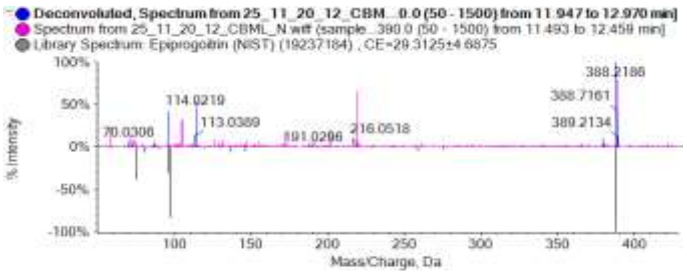

Acquired / Theoretical MS

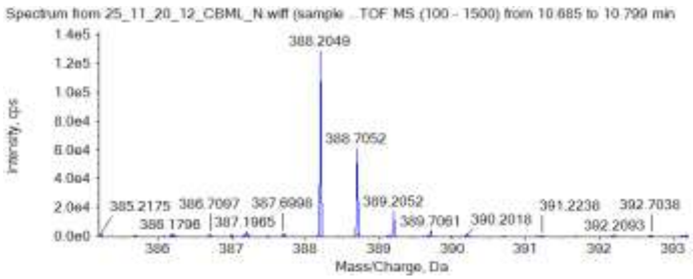

777.4202 / 10.69 (Mass/FragMass/RT/Isotope/Library/Formula/Ion Ratio)

Retention Time: 10.73 minutes **Exp RT:** 10.69 minutes
Precursor m/z : 777.4202 **Analyte Name:**
Fit (%) 100.0% **RFit (%)** 42.3% 777.4202 / 10.69

Collision Energy = 35 ± 15 eV

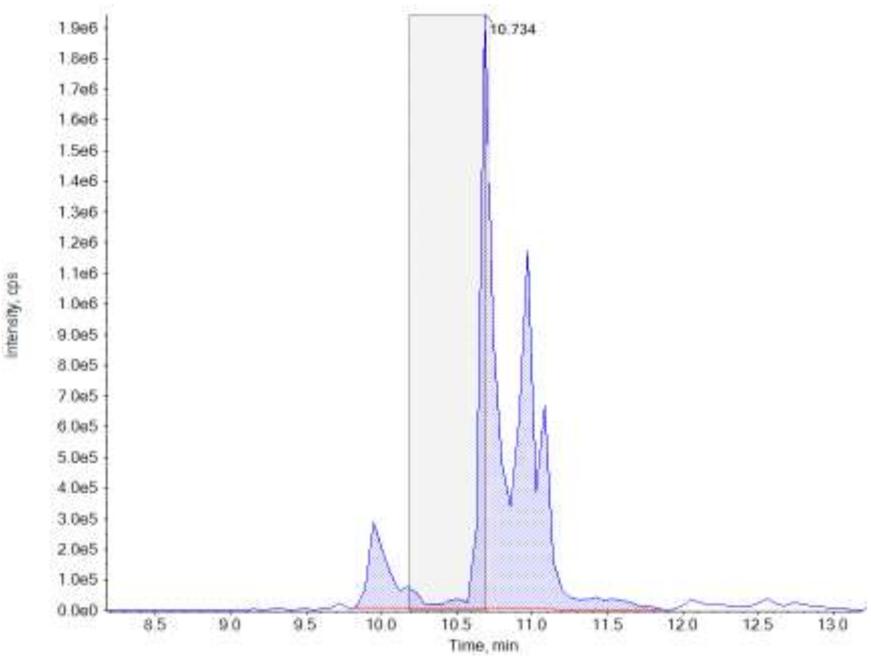

Acquired / Library MSMS

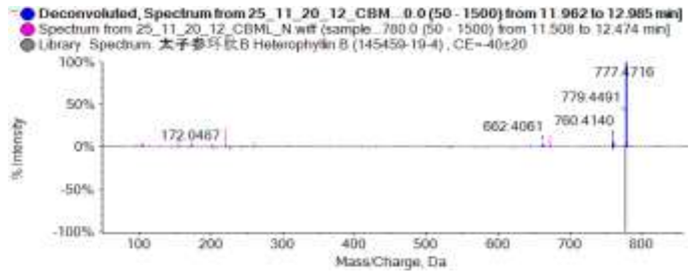

Acquired / Theoretical MS

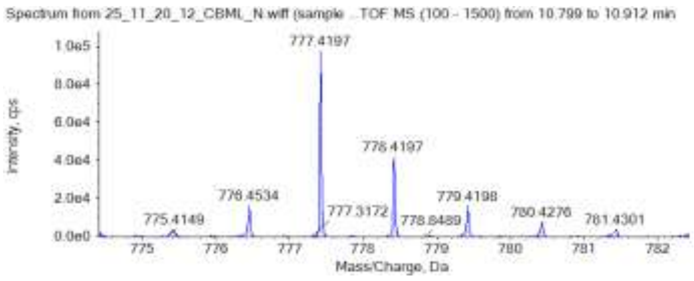

227.1271 / 10.91 (Mass/FragMass/RT/Isotope/Library/Formula/Ion Ratio)

**Retention Time:** 10.79 minutes	**Exp RT:** 10.91 minutes
**Precursor m/z :** 227.1271	**Analyte Name:**
**Fit (%)** 99.1% **RFit (%)** 100.0%	227.1271 / 10.91

Collision Energy = 35 ± 15 eV

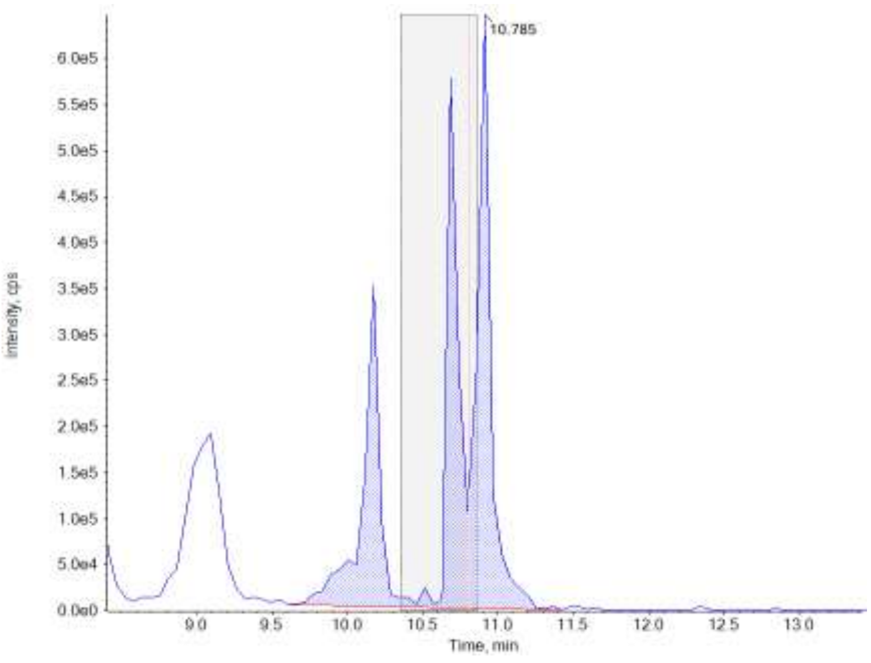

Acquired / Library MSMS

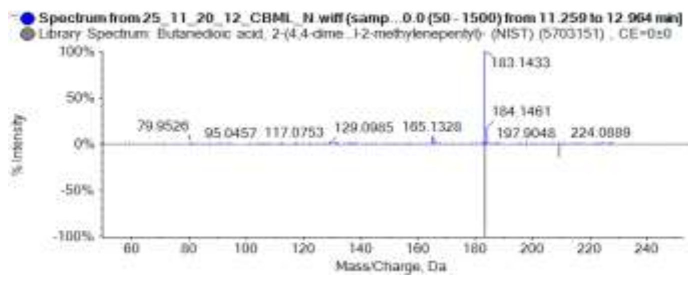

Acquired / Theoretical MS

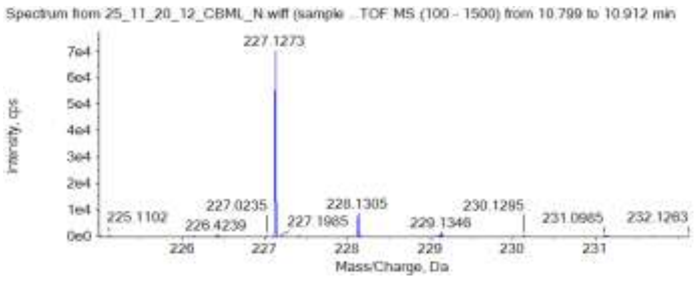

329.2314 / 10.91 (Mass/FragMass/RT/Isotope/Library/Formula/Ion Ratio)

Retention Time: 10.89 minutes
Precursor m/z : 329.2315
Fit (%) 71.0% **RFit (%)** 99.4%

Exp RT: 10.91 minutes
Analyte Name:
329.2314 / 10.91

Collision Energy = 35 ± 15 eV

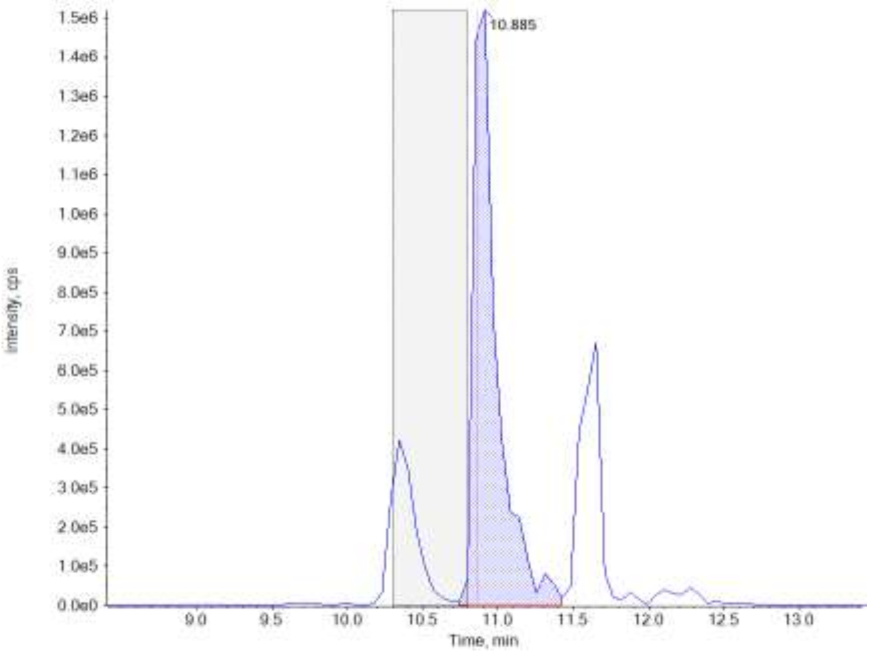

Acquired / Library MSMS

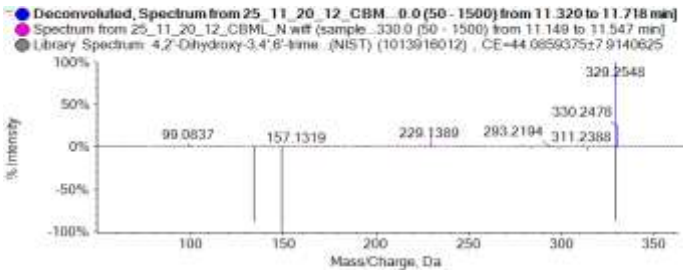

Acquired / Theoretical MS

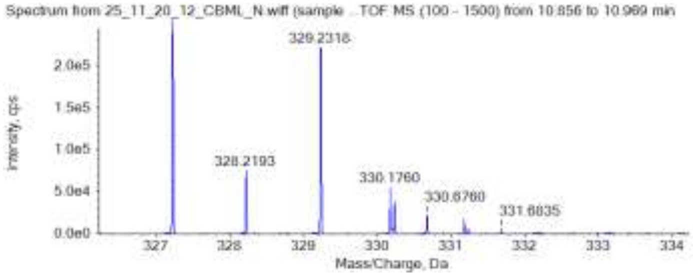

811.4116 / 11.03 (Mass/FragMass/RT/Isotope/Library/Formula/Ion Ratio)

**Retention Time:** 11.03 minutes	**Exp RT:** 11.03 minutes
**Precursor m/z :** 811.4116	**Analyte Name:**
**Fit (%)** 100.0% **RFit (%)** 92.7%	811.4116 / 11.03

Collision Energy = 35 ± 15 eV

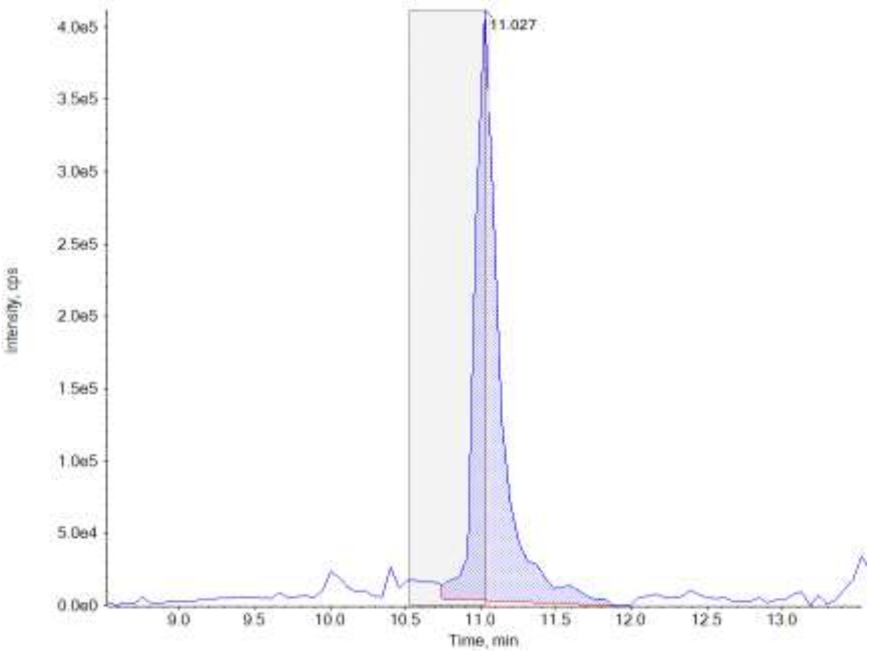

Acquired / Library MSMS

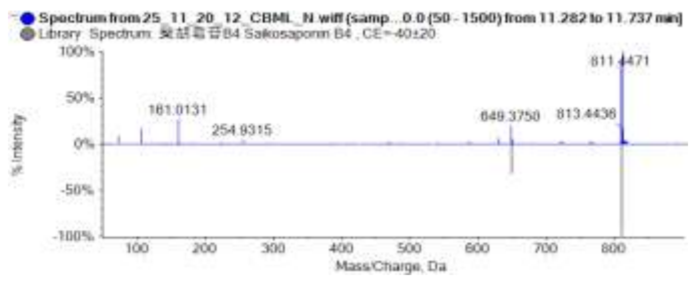

Acquired / Theoretical MS

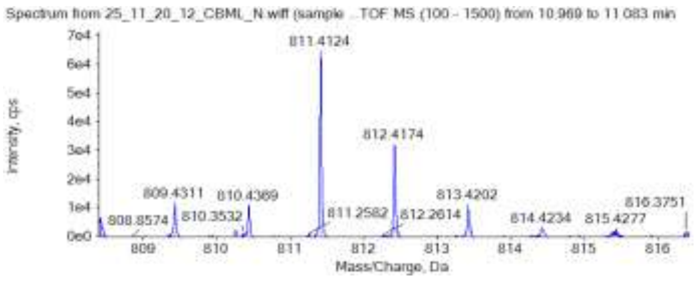

527.3354 / 11.20 (Mass/FragMass/RT/Isotope/Library/Formula/Ion Ratio)

**Retention Time:** 11.20 minutes	**Exp RT:** 11.20 minutes
**Precursor m/z :** 527.3354	**Analyte Name:**
**Fit (%)** 100.0% **RFit (%)** 98.7%	527.3354 / 11.20

Collision Energy = 35 ± 15 eV

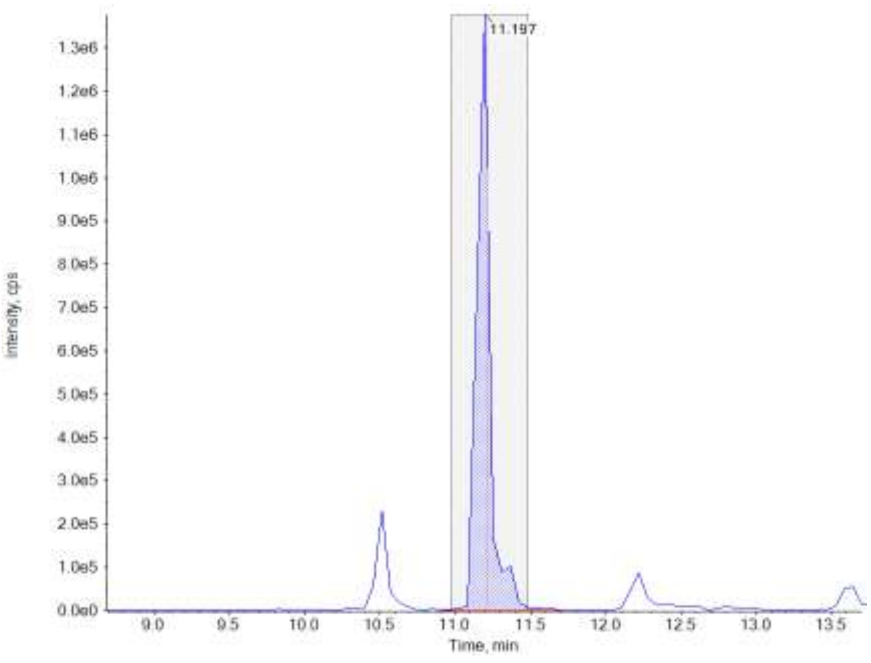

Acquired / Library MSMS

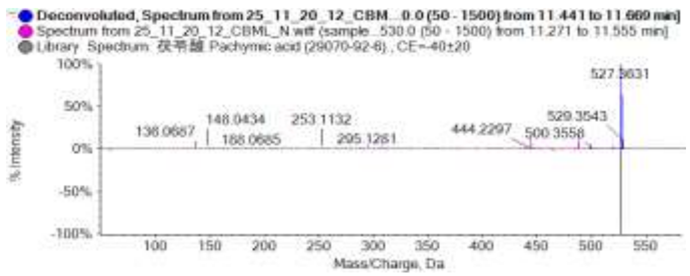

Acquired / Theoretical MS

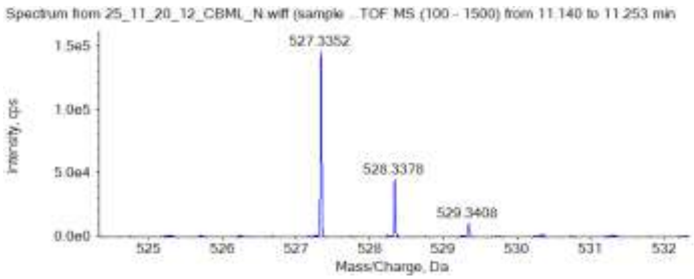

287.2205 / 11.25 (Mass/FragMass/RT/Isotope/Library/Formula/Ion Ratio)

**Retention Time:** 11.28 minutes<br>**Precursor m/z :** 287.2205<br>**Fit (%)** 89.1% **RFit (%)** 97.2%	**Exp RT:** 11.25 minutes<br>**Analyte Name:**<br>287.2205 / 11.25

Collision Energy = 35 ± 15 eV

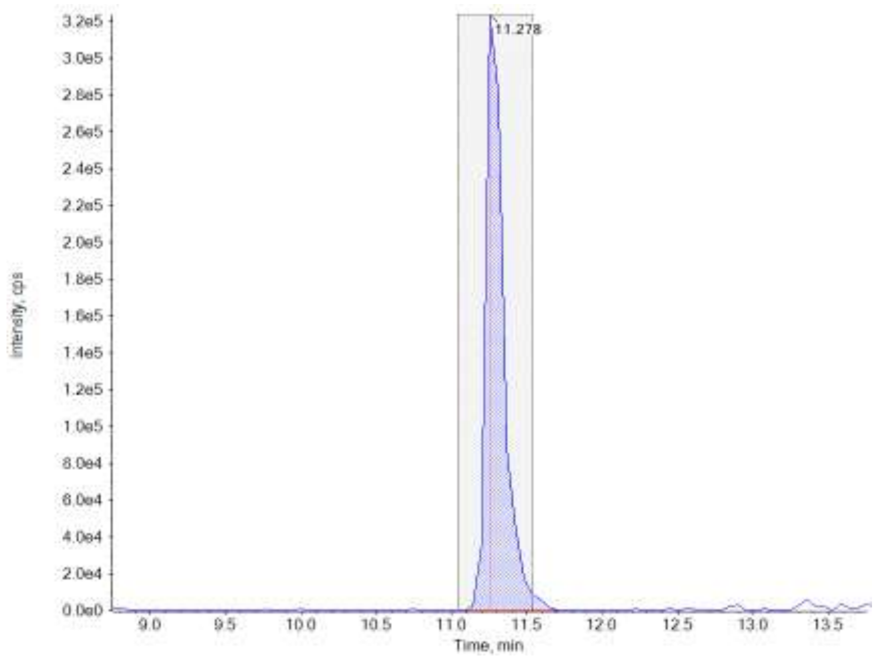

Acquired / Library MSMS

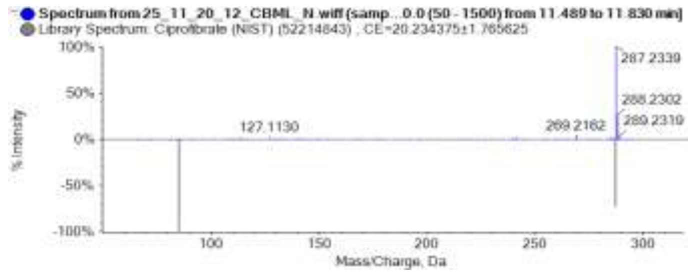

Acquired / Theoretical MS

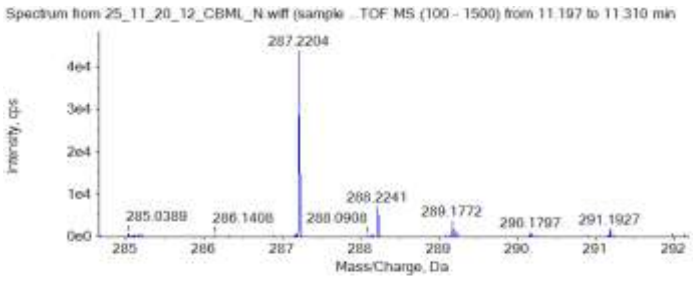

195.1373 / 11.37 (Mass/FragMass/RT/Isotope/Library/Formula/Ion Ratio)

Retention Time: 11.39 minutes **Exp RT:** 11.37 minutes
Precursor m/z : 195.1373 **Analyte Name:**
Fit (%) 82.6% **RFit (%)** 94.6% 195.1373 / 11.37

Collision Energy = 35 ± 15 eV

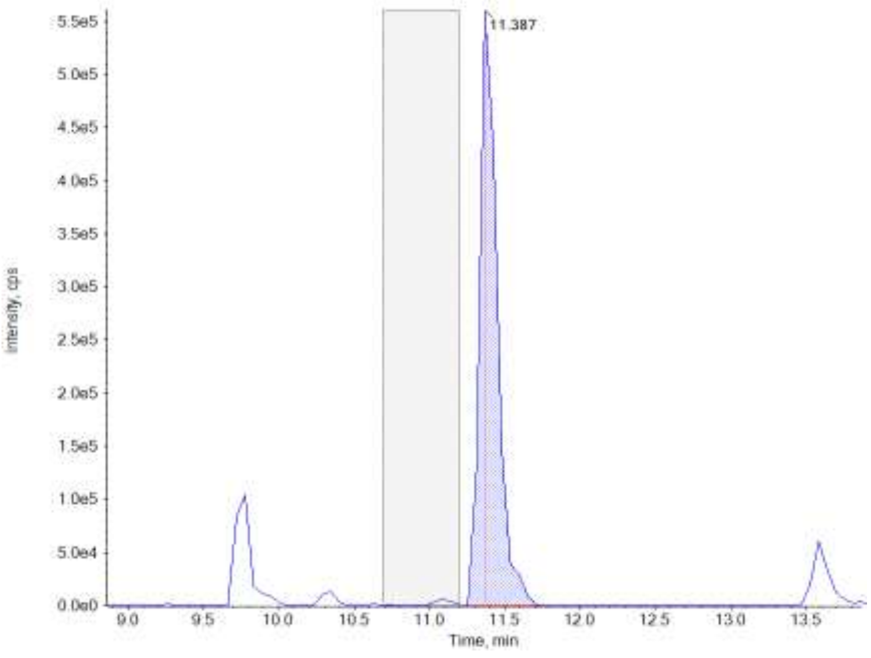

Acquired / Library MSMS

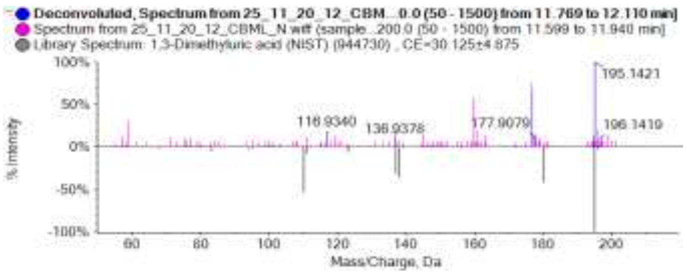

Acquired / Theoretical MS

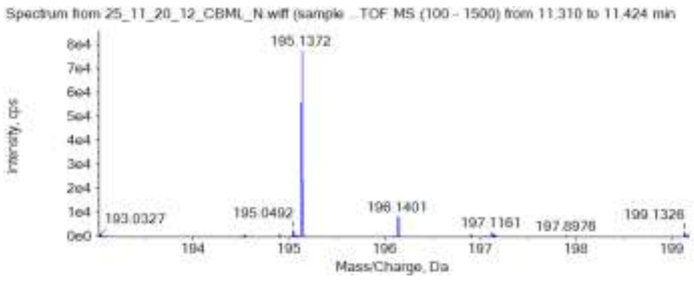

239.1271 / 11.42 (Mass/FragMass/RT/Isotope/Library/Formula/Ion Ratio)

Retention Time: 11.42 minutes
Precursor m/z : 239.1271
Fit (%) 90.8% **RFit (%)** 92.1%

Exp RT: 11.42 minutes
Analyte Name:
239.1271 / 11.42

Collision Energy = 35 ± 15 eV

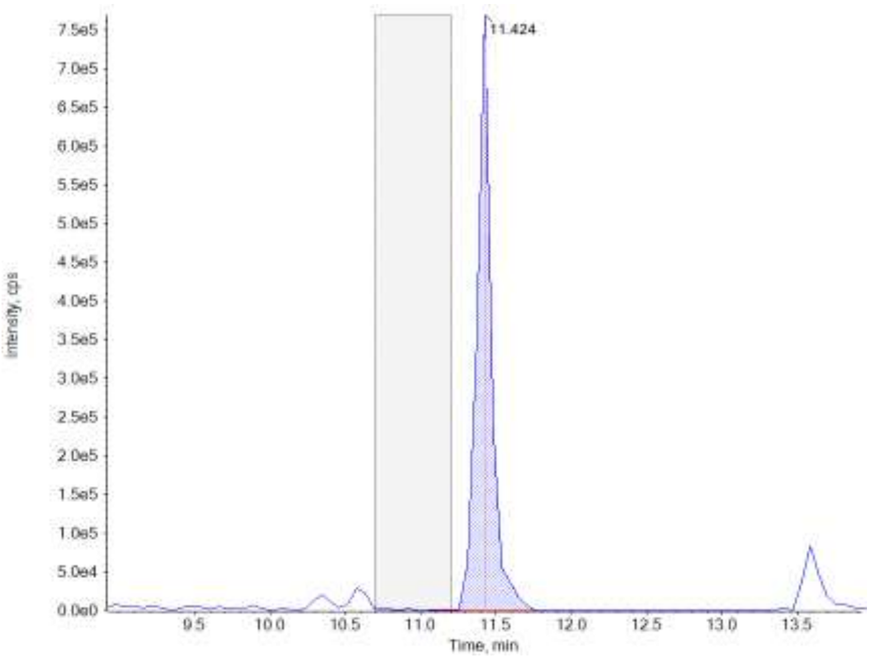

Acquired / Library MSMS

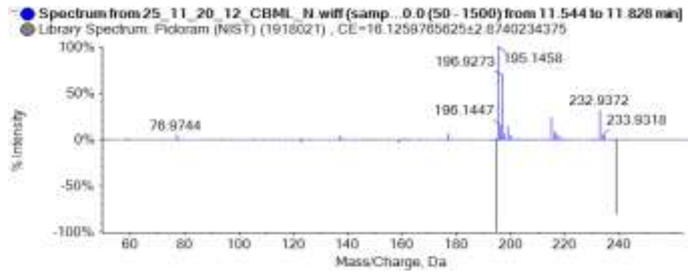

Acquired / Theoretical MS

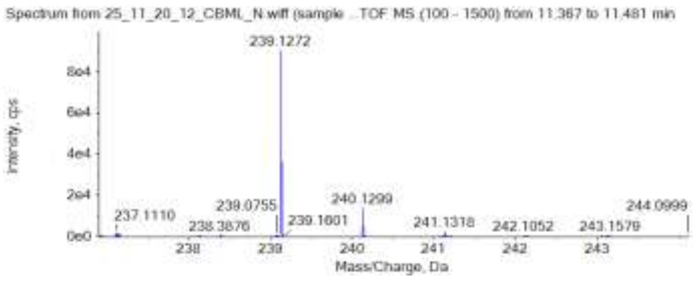

679.3694 / 11.42 (Mass/FragMass/RT/Isotope/Library/Formula/Ion Ratio)

**Retention Time:** 11.42 minutes	**Exp RT:** 11.42 minutes
**Precursor m/z :** 679.3694	**Analyte Name:**
**Fit (%)** 63.6% **RFit (%)** 95.9%	679.3694 / 11.42

	**Collision Energy = 35 ± 15 eV**

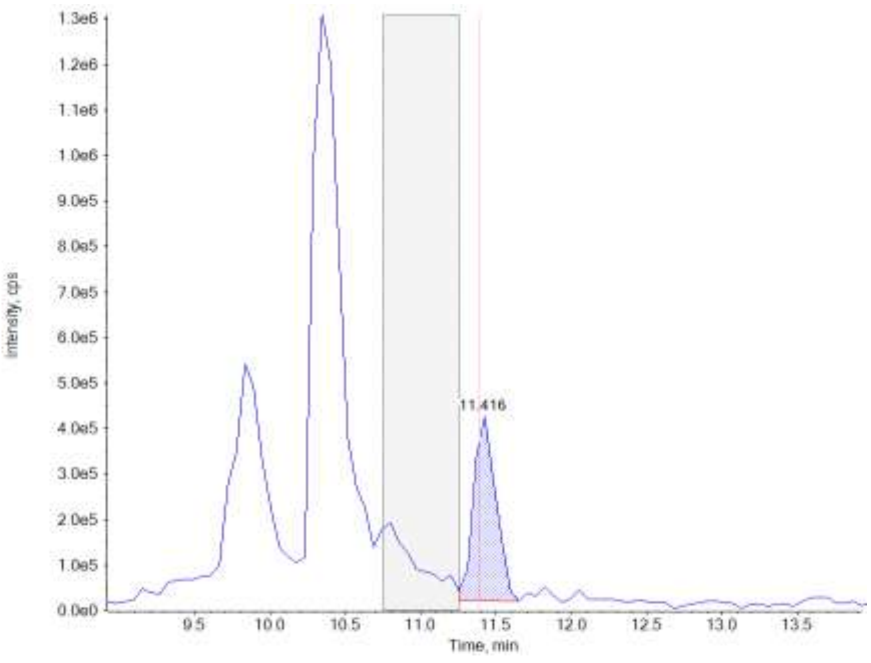

Acquired / Library MSMS

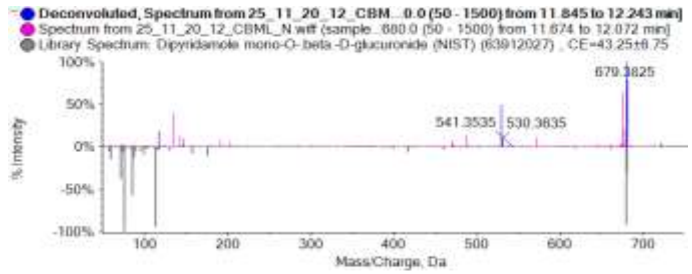

Acquired / Theoretical MS

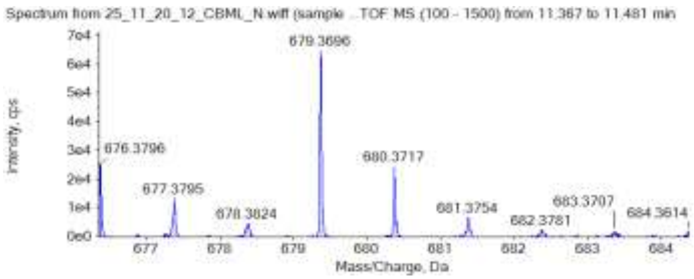

669.3755 / 11.59 (Mass/FragMass/RT/Isotope/Library/Formula/Ion Ratio)

**Retention Time:** 11.60 minutes	**Exp RT:** 11.59 minutes
**Precursor m/z :** 669.3755	**Analyte Name:**
**Fit (%)** 73.8% **RFit (%)** 100.0%	669.3755 / 11.59

Collision Energy = 35 ± 15 eV

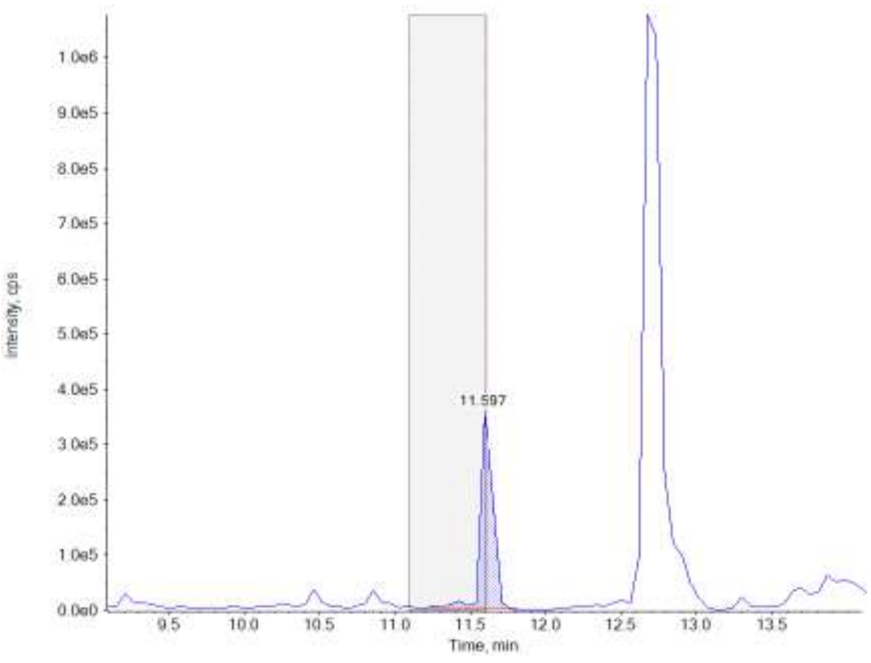

Acquired / Library MSMS

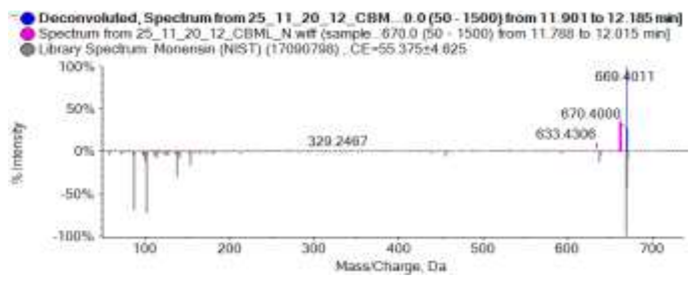

Acquired / Theoretical MS

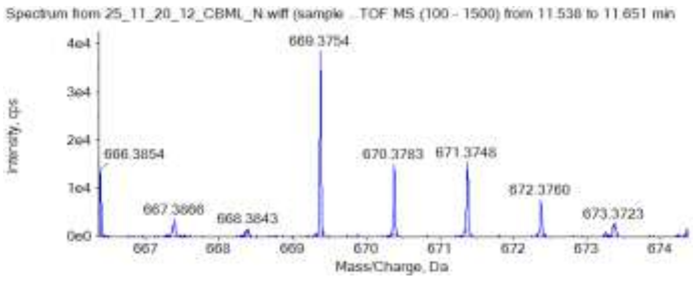

329.2308 / 11.65 (Mass/FragMass/RT/Isotope/Library/Formula/Ion Ratio)

Retention Time: 11.61 minutes **Exp RT:** 11.65 minutes
Precursor m/z : 329.2308 **Analyte Name:**
Fit (%) 75.5% **RFit (%)** 92.2% 329.2308 / 11.65

Collision Energy = 35 ± 15 eV

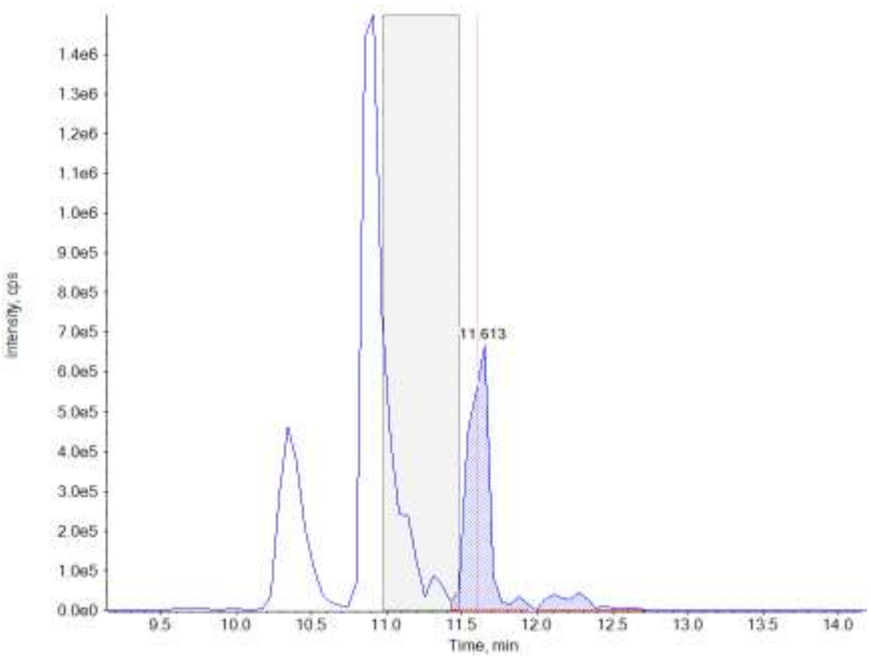

Acquired / Library MSMS

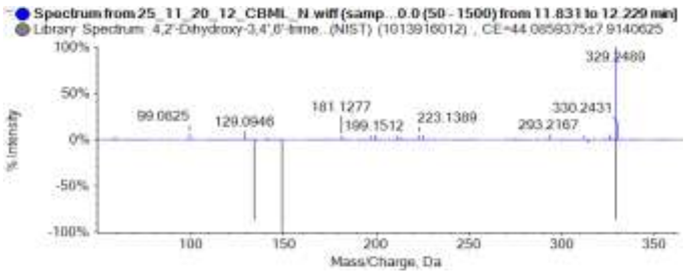

Acquired / Theoretical MS

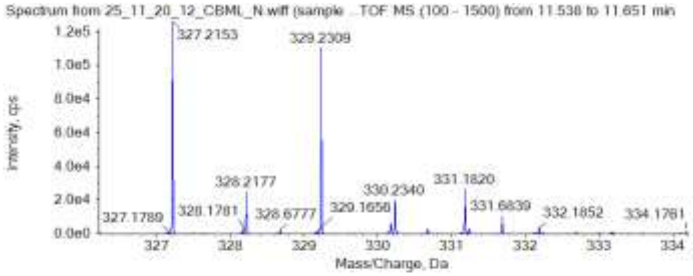

577.2671 / 12.39 (Mass/FragMass/RT/Isotope/Library/Formula/Ion Ratio)

Retention Time: 12.41 minutes
Precursor m/z : 577.2671
Fit (%) 21.1% **RFit (%)** 100.0%

Exp RT: 12.39 minutes
Analyte Name:
577.2671 / 12.39

Collision Energy = 35 ± 15 eV

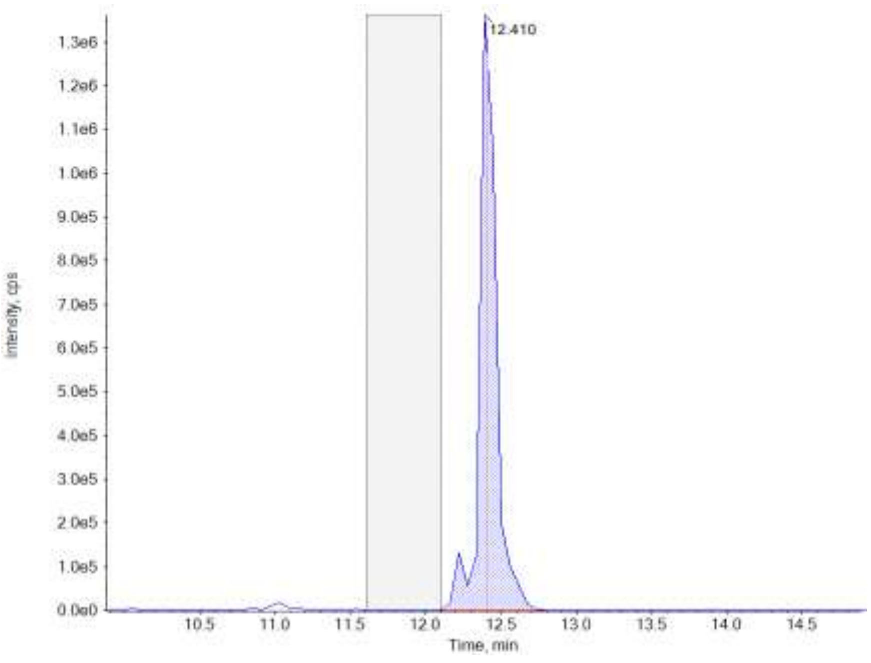

Acquired / Library MSMS

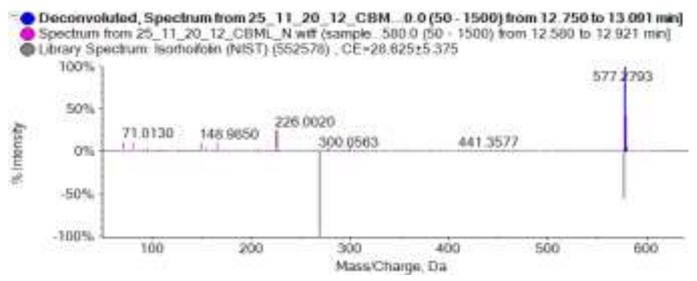

Acquired / Theoretical MS

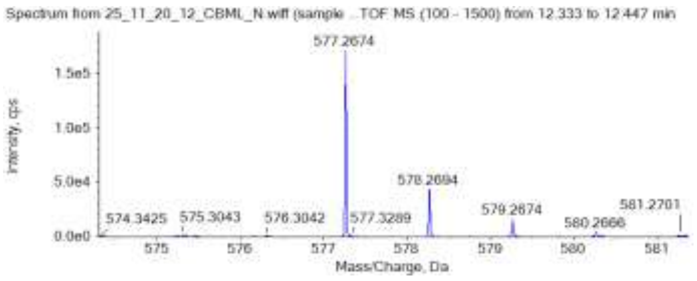

669.3763 / 12.73 (Mass/FragMass/RT/Isotope/Library/Formula/Ion Ratio)

**Retention Time:** 12.70 minutes	**Exp RT:** 12.73 minutes
**Precursor m/z :** 669.3763	**Analyte Name:**
**Fit (%)** 42.5% **RFit (%)** 88.7%	669.3763 / 12.73

Collision Energy = 35 ± 15 eV

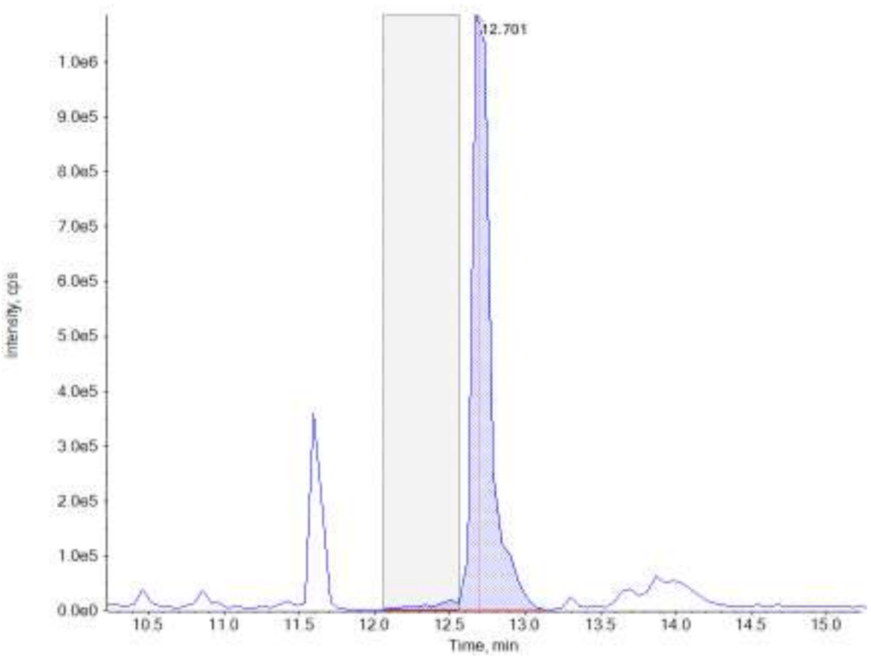

Acquired / Library MSMS

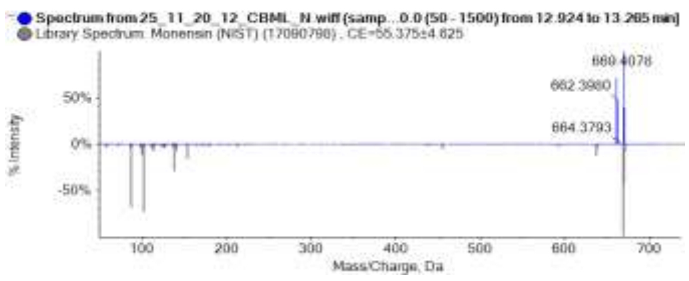

Acquired / Theoretical MS

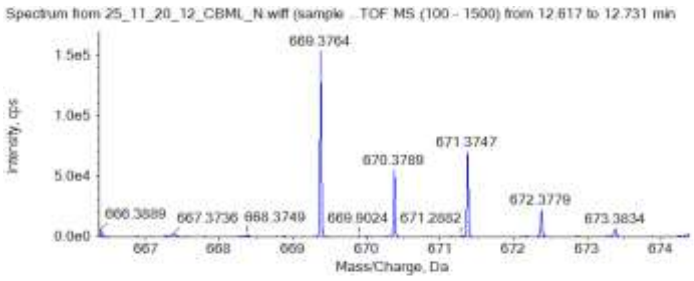

Created with SCIEX OS 3.3.0
Printed:12/9/2025 2:25:51 PM

679.4159 / 12.73 (Mass/FragMass/RT/Isotope/Library/Formula/Ion Ratio)

Retention Time: 12.71 minutes
Precursor m/z : 679.4159
Fit (%) 66.2% **RFit (%)** 86.5%

Exp RT: 12.73 minutes
Analyte Name:
679.4159 / 12.73

Collision Energy = 35 ± 15 eV

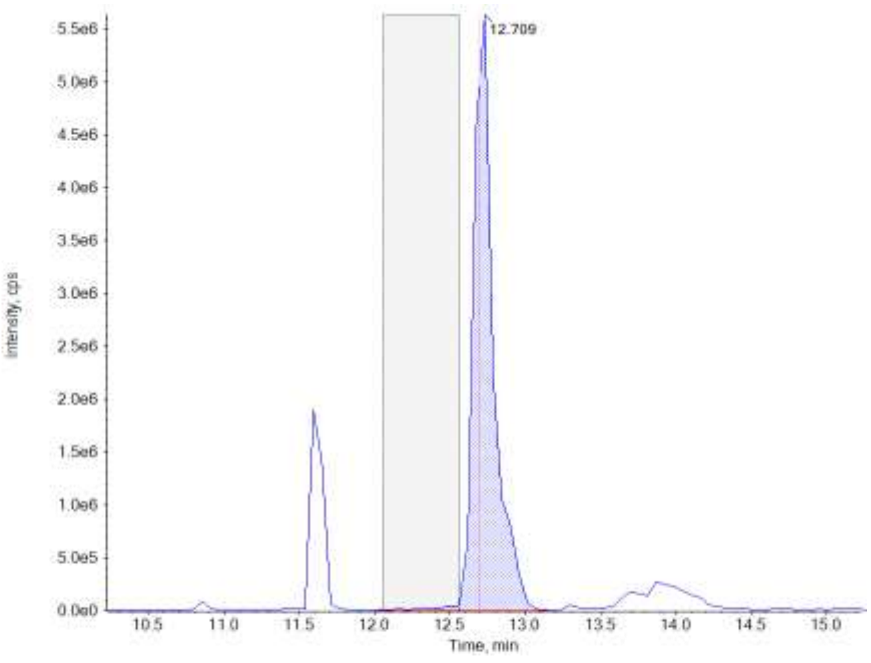

Acquired / Library MSMS

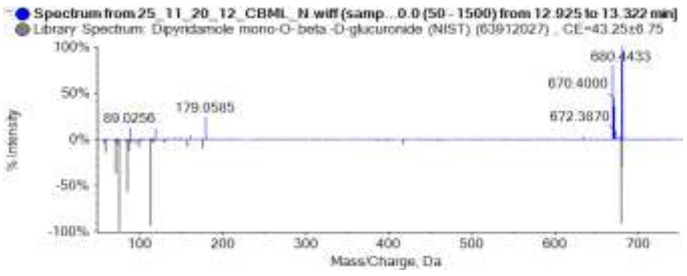

Acquired / Theoretical MS

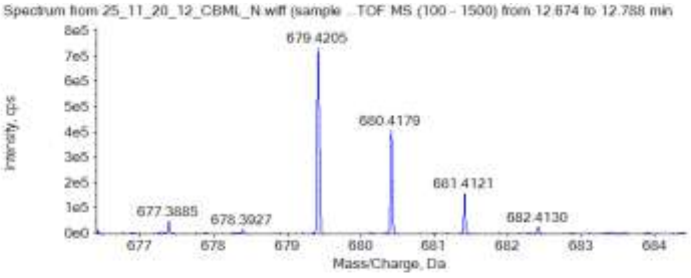

309.2051 / 12.84 (Mass/FragMass/RT/Isotope/Library/Formula/Ion Ratio)

Retention Time: 12.85 minutes
Precursor m/z : 309.2051
Fit (%) 93.9% **RFit (%)** 43.2%

Exp RT: 12.85 minutes
Analyte Name:
309.2051 / 12.84

Collision Energy = 35 ± 15 eV

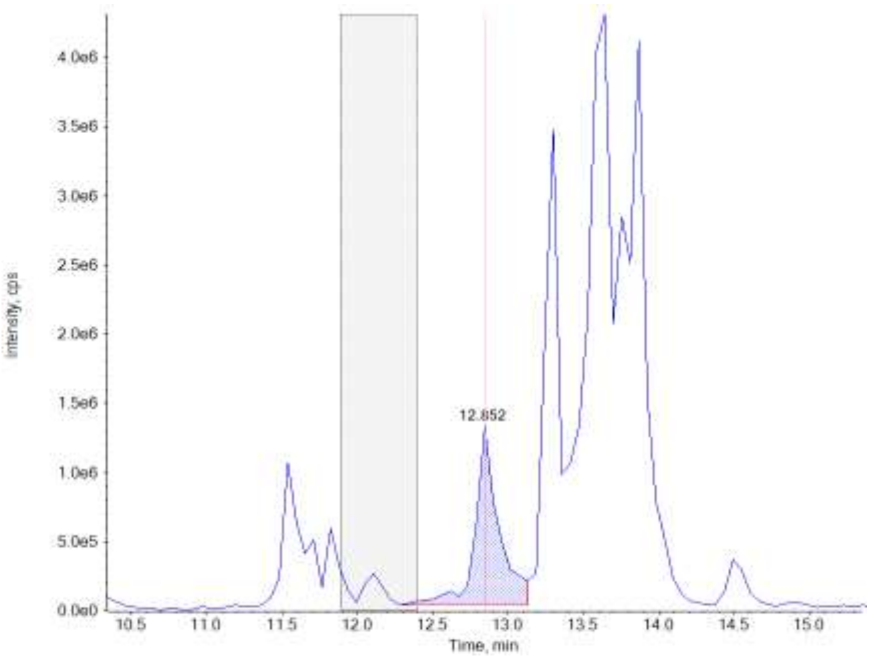

Acquired / Library MSMS

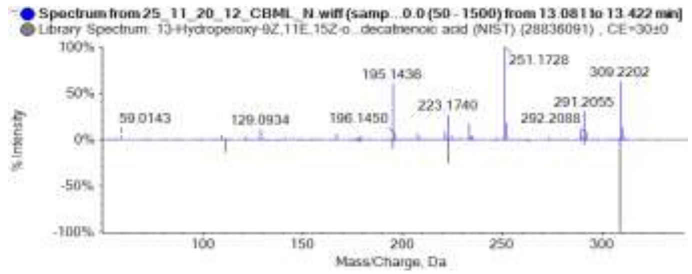

Acquired / Theoretical MS

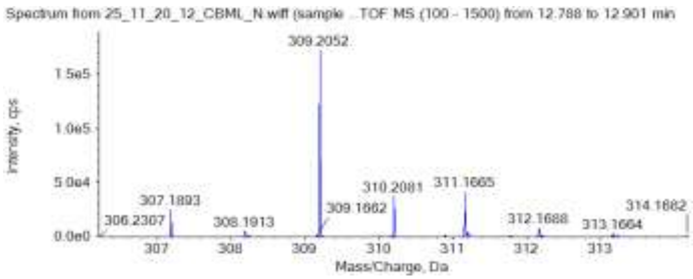

601.3735 / 13.19 (Mass/FragMass/RT/Isotope/Library/Formula/Ion Ratio)

**Retention Time:** 13.17 minutes	**Exp RT:** 13.19 minutes
**Precursor m/z :** 601.3735	**Analyte Name:**
**Fit (%)** 82.6% **RFit (%)** 94.0%	601.3735 / 13.19

Collision Energy = 35 ± 15 eV

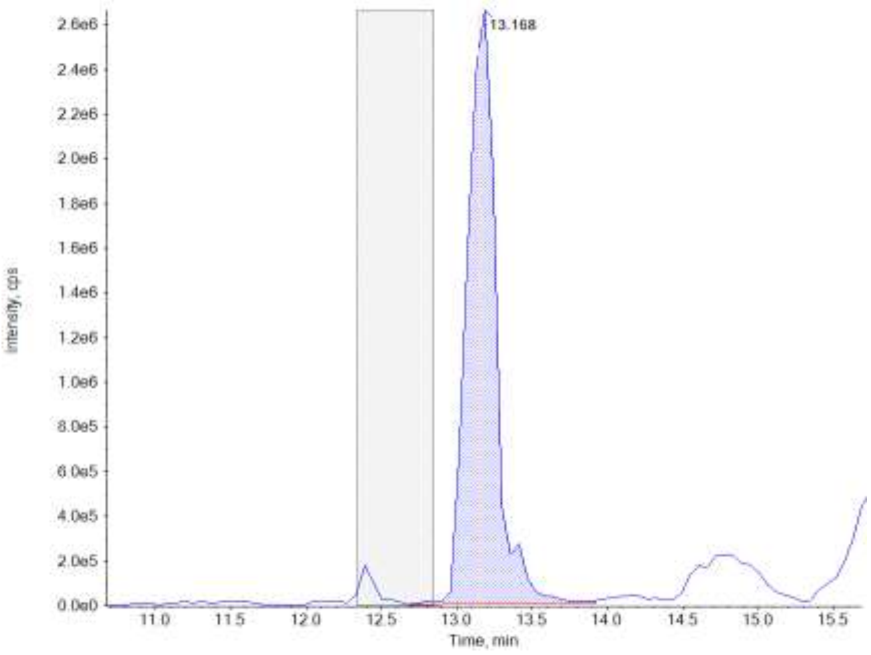

Acquired / Library MSMS

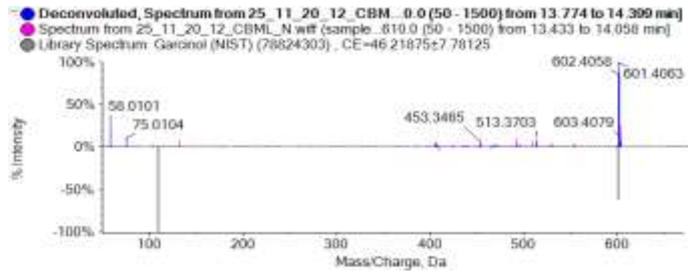

Acquired / Theoretical MS

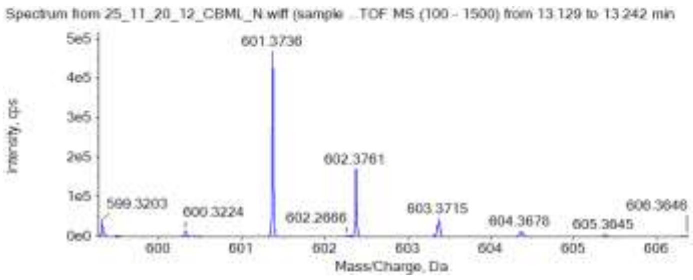

571.2882 / 13.24 (Mass/FragMass/RT/Isotope/Library/Formula/Ion Ratio)

Retention Time: 13.24 minutes **Exp RT:** 13.24 minutes
Precursor m/z : 571.2882 **Analyte Name:**
Fit (%) 97.4% **RFit (%)** 81.9% 571.2882 / 13.24

Collision Energy = 35 ± 15 eV

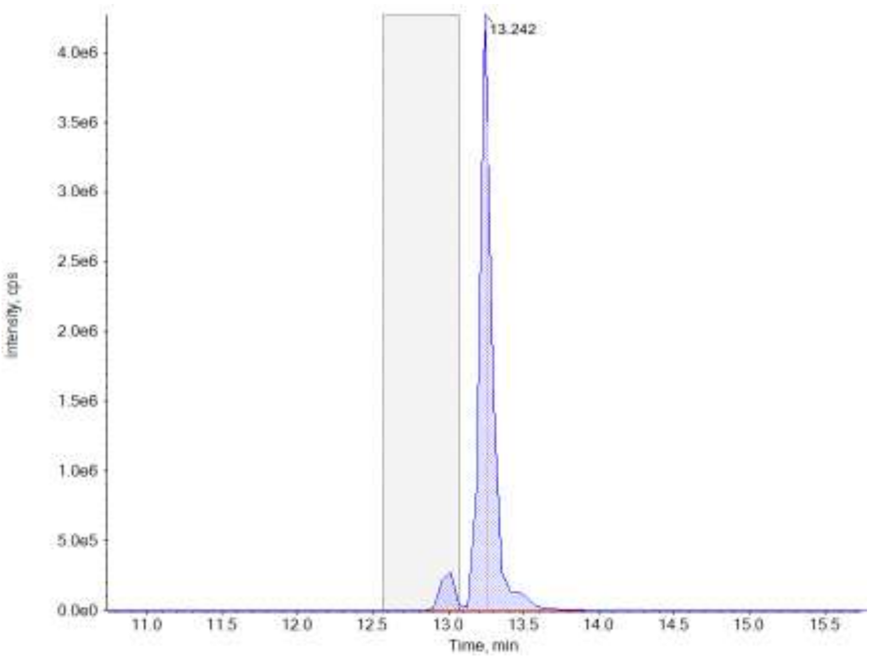

Acquired / Library MSMS

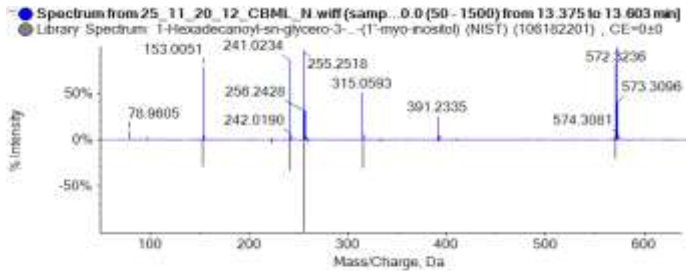

Acquired / Theoretical MS

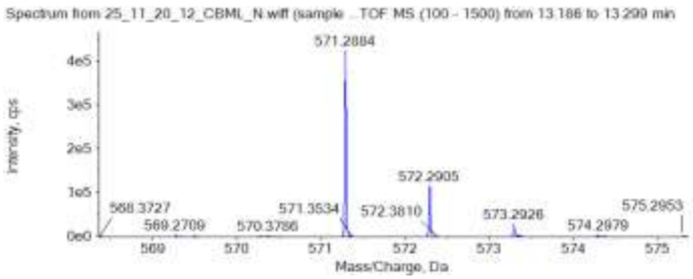

209.1156 / 13.70 (Mass/FragMass/RT/Isotope/Library/Formula/Ion Ratio)

Retention Time: 13.70 minutes
Precursor m/z : 209.1156
Fit (%) 92.2% **RFit (%)** 94.4%

Exp RT: 13.70 minutes
Analyte Name:
209.1156 / 13.70

Collision Energy = 35 ± 15 eV

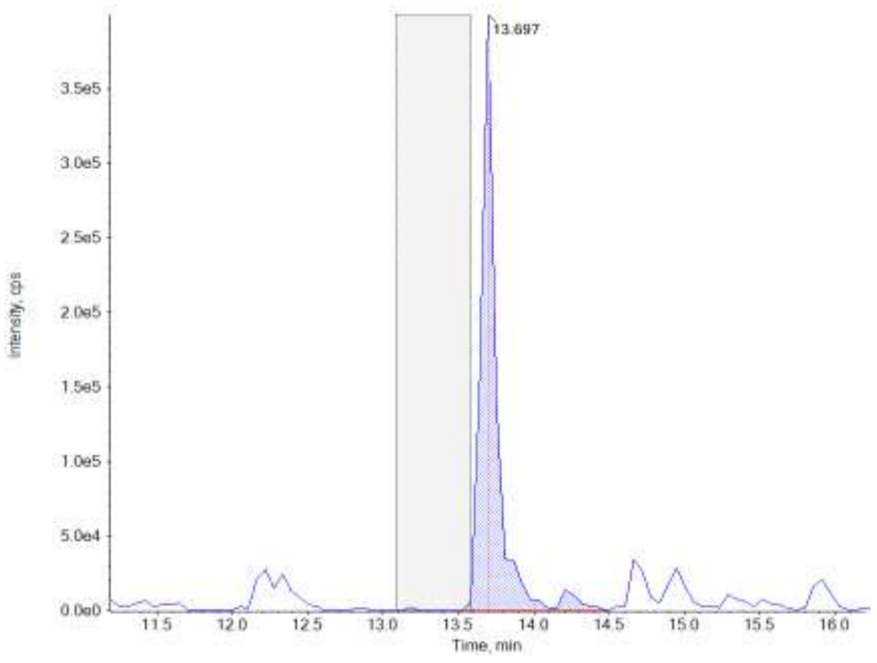

Acquired / Library MSMS

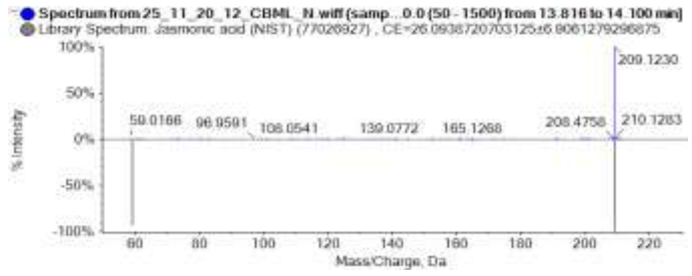

Acquired / Theoretical MS

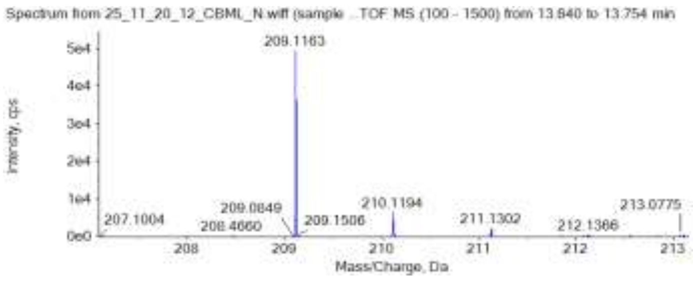

581.2985 / 13.87

(Mass/FragMass/RT/Isotope/Library/Formula/Ion Ratio)

**Retention Time:** 13.87 minutes	**Exp RT:** 13.87 minutes
**Precursor m/z :** 581.2985	**Analyte Name:**
**Fit (%)** 58.2% **RFit (%)** 100.0%	581.2985 / 13.87

Collision Energy = 35 ± 15 eV

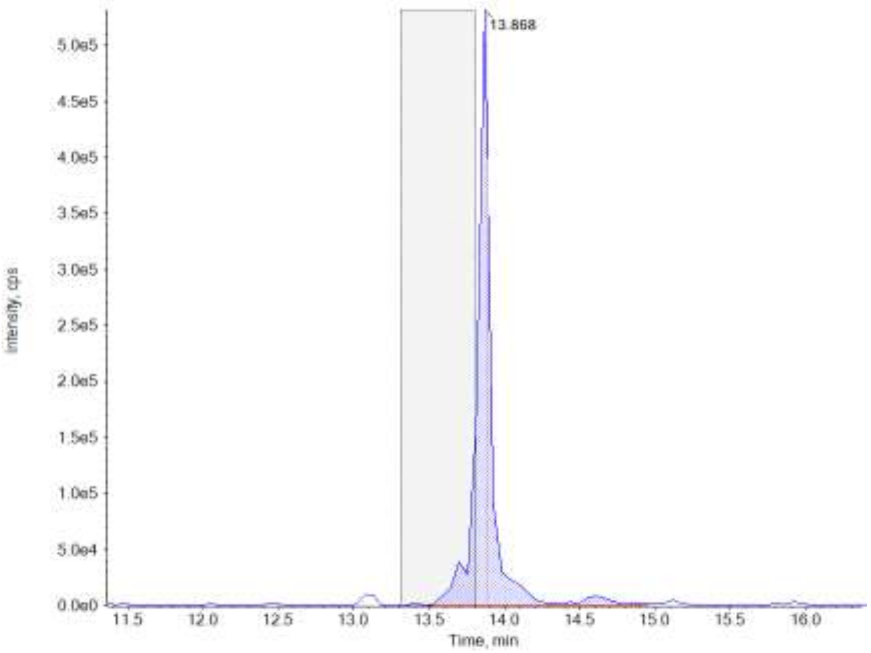

Acquired / Library MSMS

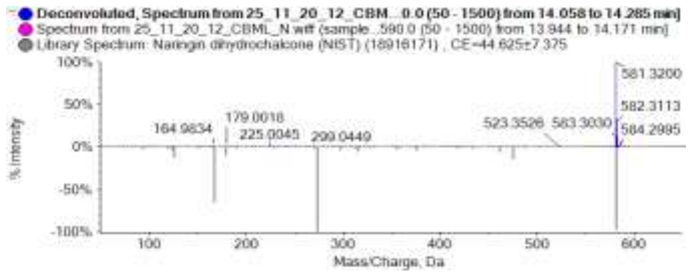

Acquired / Theoretical MS

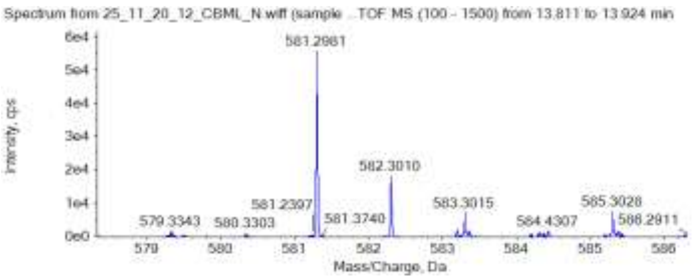

483.2768 / 13.92 (Mass/FragMass/RT/Isotope/Library/Formula/Ion Ratio)

Retention Time: 13.94 minutes
Precursor m/z : 483.2768
Fit (%) 99.8% **RFit (%)** 99.4%

Exp RT: 13.92 minutes
Analyte Name:
483.2768 / 13.92

Collision Energy = 35 ± 15 eV

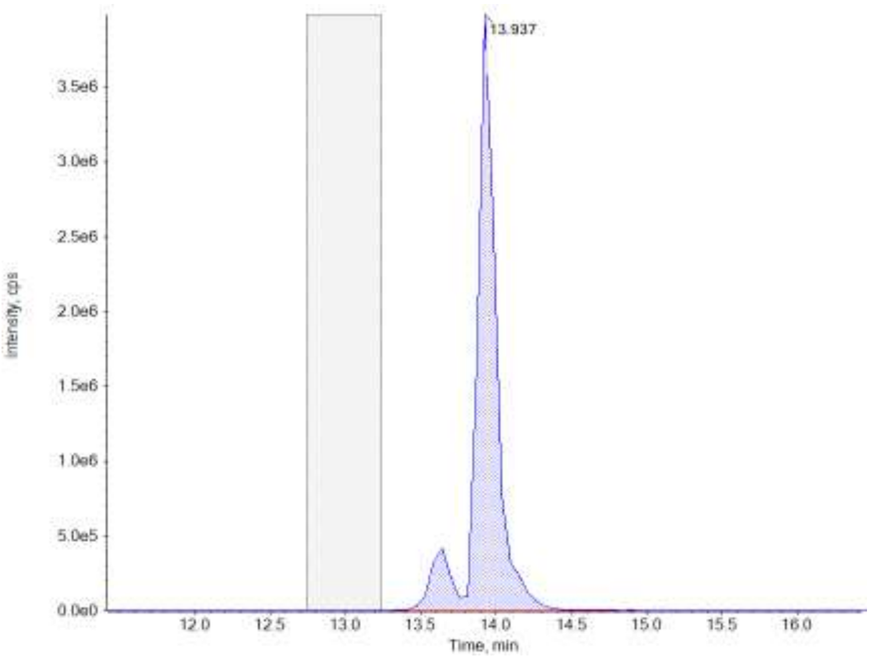

Acquired / Library MSMS

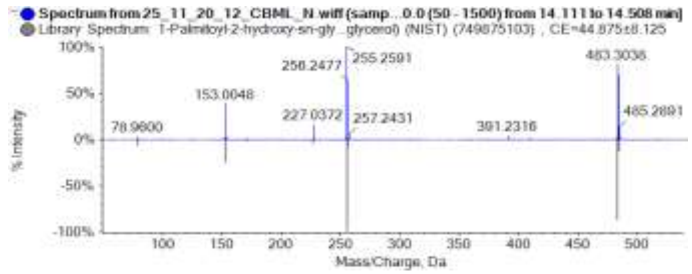

Acquired / Theoretical MS

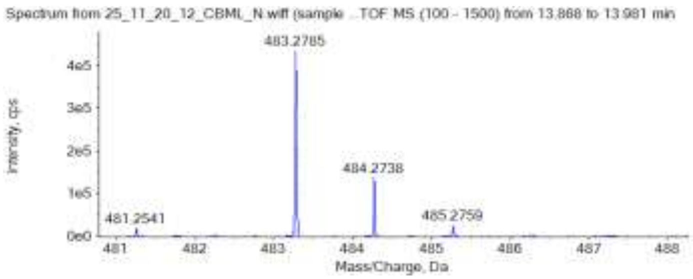

785.4693 / 14.09 (Mass/FragMass/RT/Isotope/Library/Formula/Ion Ratio)

Retention Time: 14.11 minutes **Exp RT:** 14.10 minutes
Precursor m/z : 785.4693 **Analyte Name:**
Fit (%) 50.8% **RFit (%)** 87.8% 785.4693 / 14.09

Collision Energy = 35 ± 15 eV

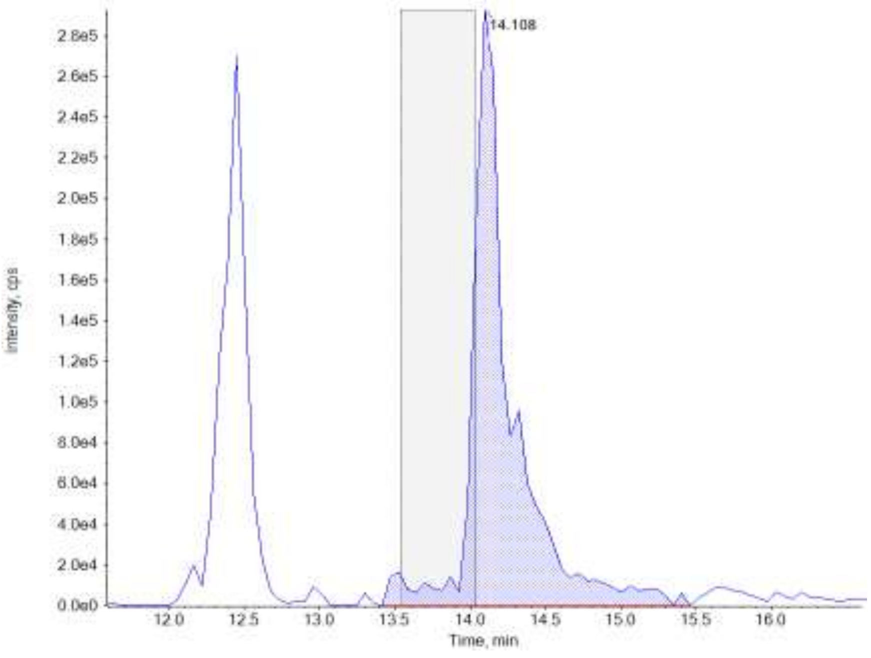

Acquired / Library MSMS

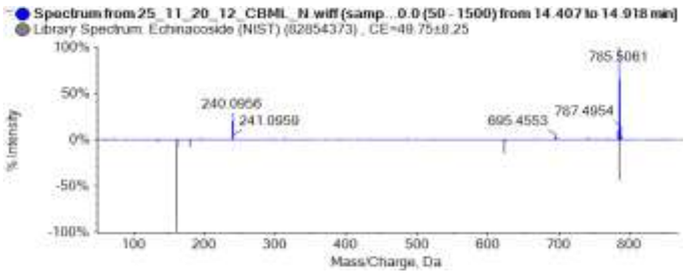

Acquired / Theoretical MS

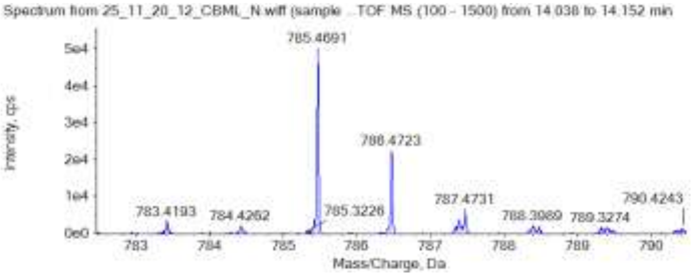

509.2888 / 14.27

(Mass/FragMass/RT/Isotope/Library/Formula/Ion Ratio)

Retention Time: 14.27 minutes
Precursor m/z : 509.2888
Fit (%) 99.0% **RFit (%)** 99.6%

Exp RT: 14.27 minutes
Analyte Name:
509.2888 / 14.27

Collision Energy = 35 ± 15 eV

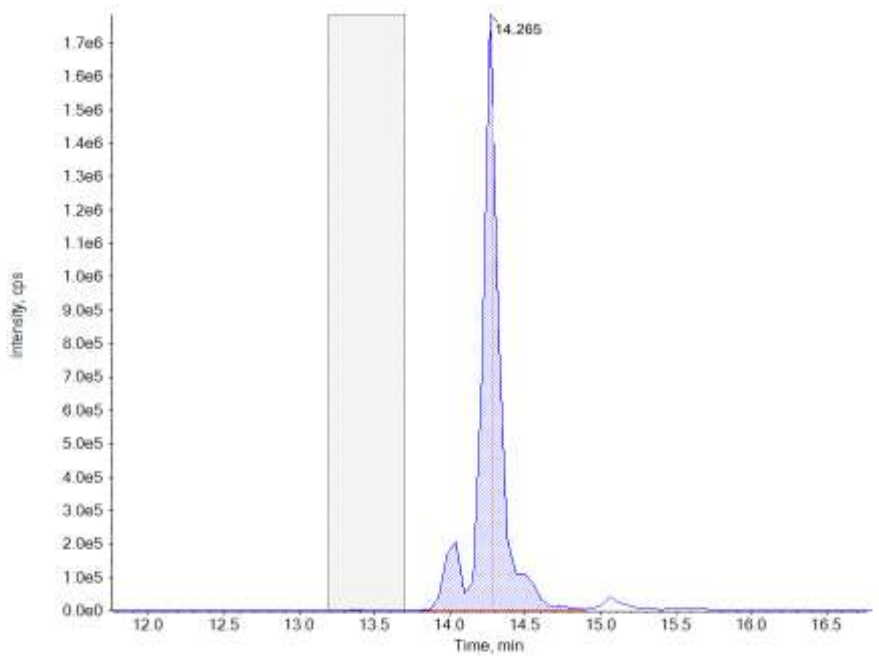

Acquired / Library MSMS

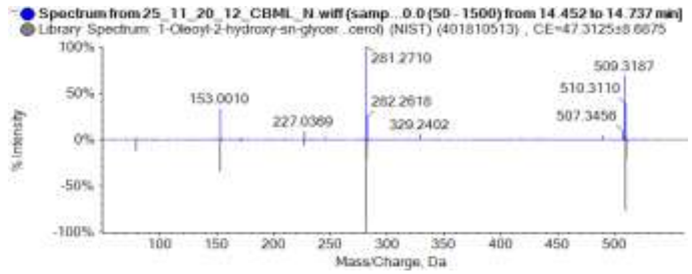

Acquired / Theoretical MS

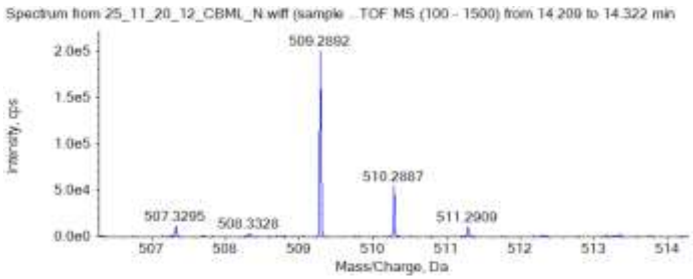

309.2044 / 14.49 (Mass/FragMass/RT/Isotope/Library/Formula/Ion Ratio)

**Retention Time:** 14.51 minutes	**Exp RT:** 14.49 minutes
**Precursor m/z :** 309.2044	**Analyte Name:**
**Fit (%)** 92.6% **RFit (%)** 97.2%	309.2044 / 14.49

Collision Energy = 35 ± 15 eV

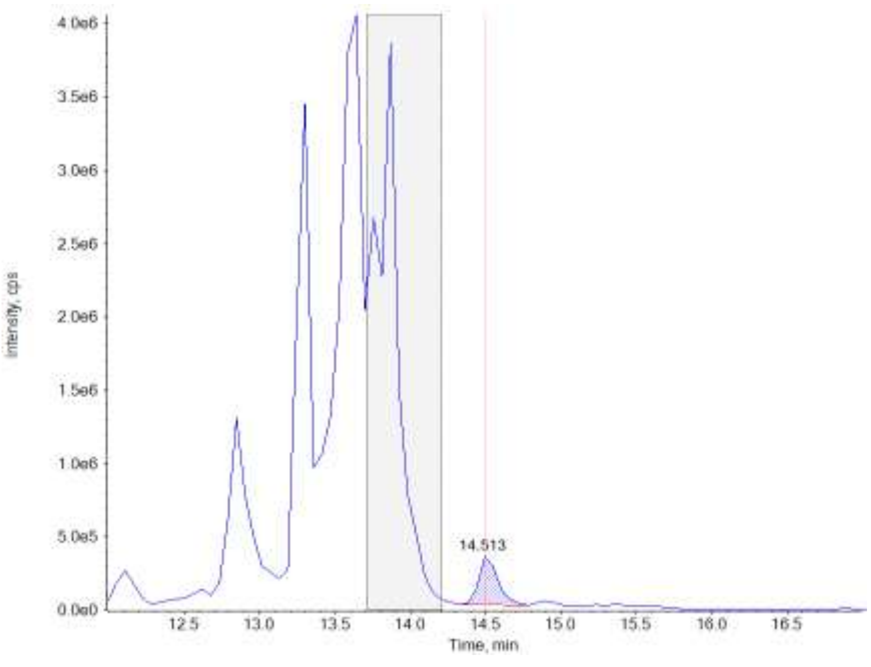

Acquired / Library MSMS

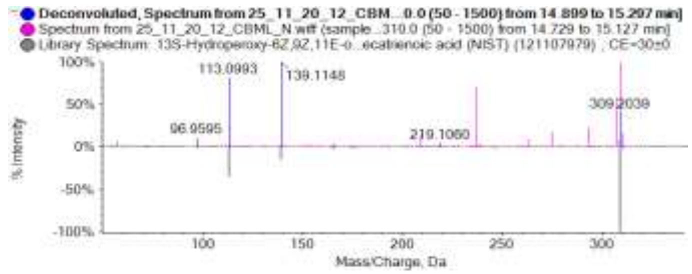

Acquired / Theoretical MS

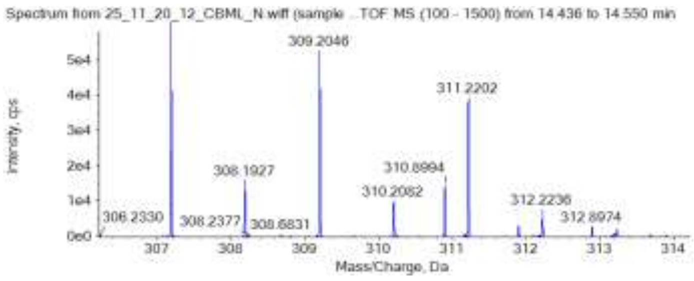

Created with SCIEX OS 3.3.0
Printed:12/9/2025 2:25:51 PM

695.4022 / 14.21 [M+AcO-H]- (Mass/FragMass/RT/Isotope/Library/Formula/Ion Ratio)

Retention Time: 14.18 minutes **Exp RT:** 14.21 minutes
Precursor m/z : 695.4022 **Analyte Name:**
Fit (%) 97.1% **RFit (%)** 97.0% 695.4022 / 14.21 [M+AcO-H]-

Collision Energy = 35 ± 15 eV

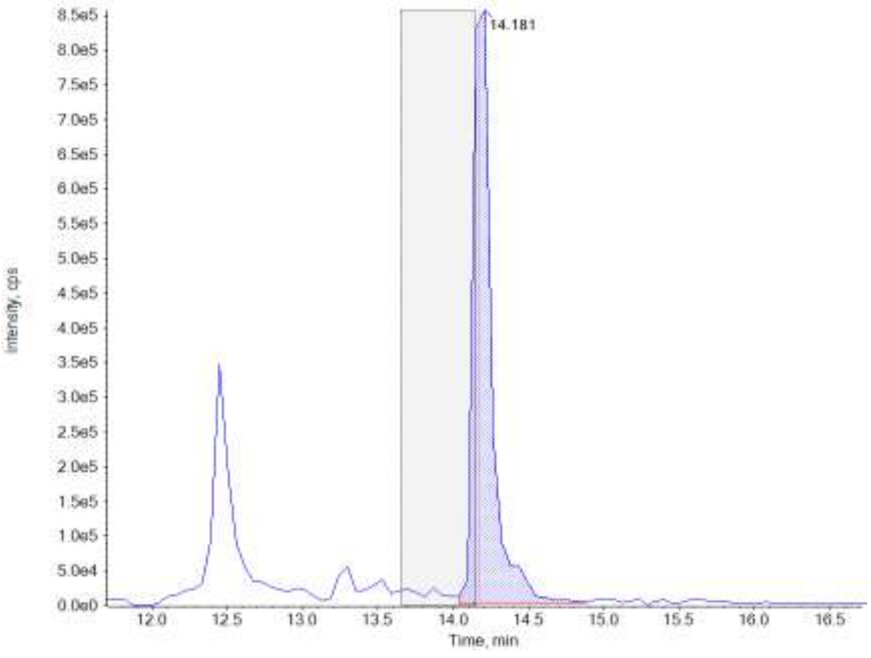

Acquired / Library MSMS

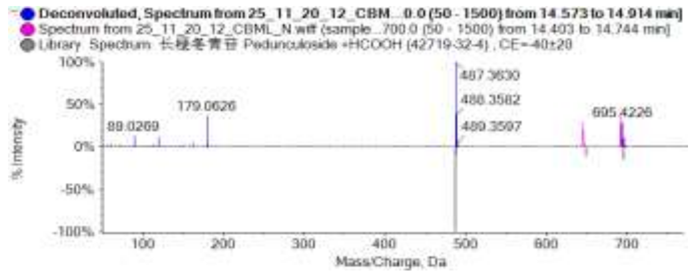

Acquired / Theoretical MS

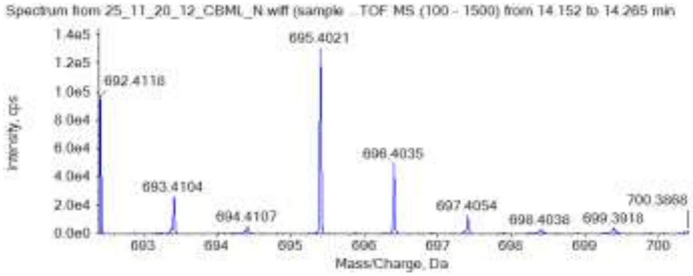

279.1941 / 14.55 (Mass/FragMass/RT/Isotope/Library/Formula/Ion Ratio)

**Retention Time:** 14.48 minutes	**Exp RT:** 14.55 minutes
**Precursor m/z :** 279.1941	**Analyte Name:**
**Fit (%)** 100.0% **RFit (%)** 100.0%	279.1941 / 14.55

Collision Energy = 35 ± 15 eV

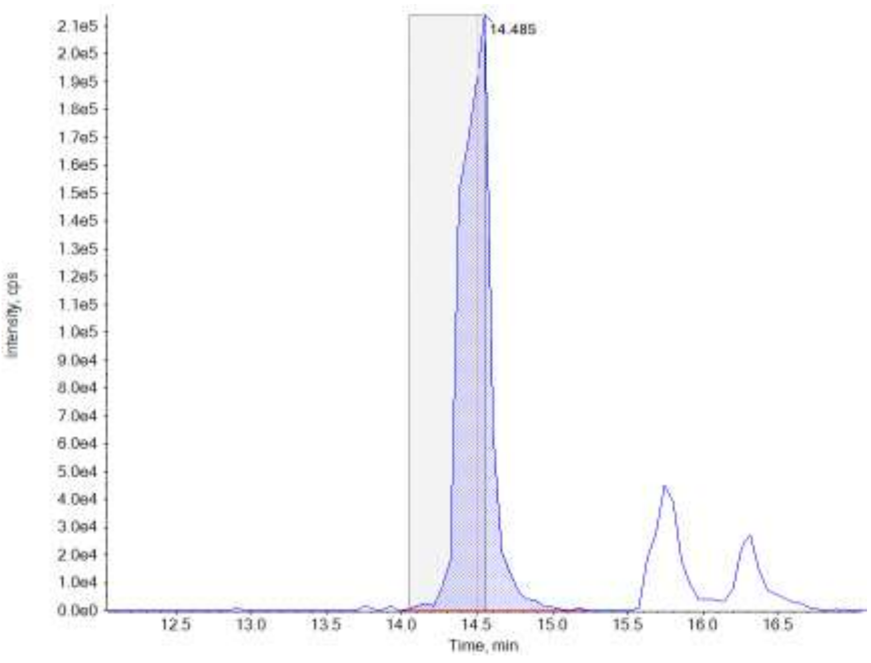

Acquired / Library MSMS

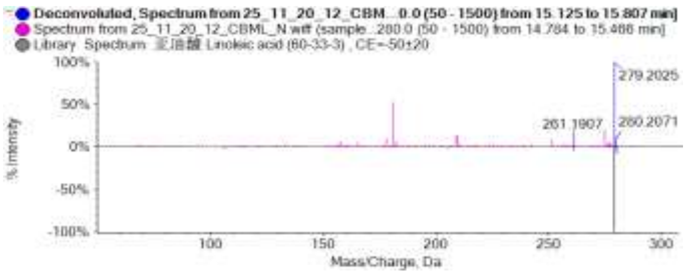

Acquired / Theoretical MS

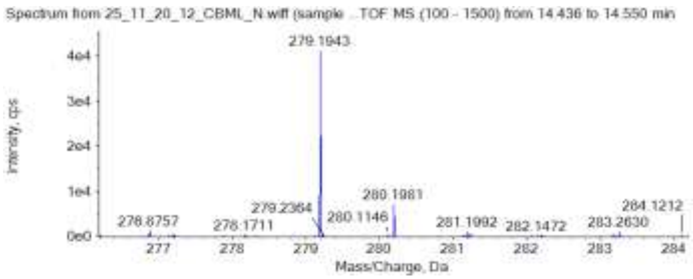

599.3187 / 14.61 (Mass/FragMass/RT/Isotope/Library/Formula/Ion Ratio)

Retention Time: 14.62 minutes
Precursor m/z : 599.3187
Fit (%) 85.6% **RFit (%)** 83.6%

Exp RT: 14.61 minutes
Analyte Name:
599.3187 / 14.61

Collision Energy = 35 ± 15 eV

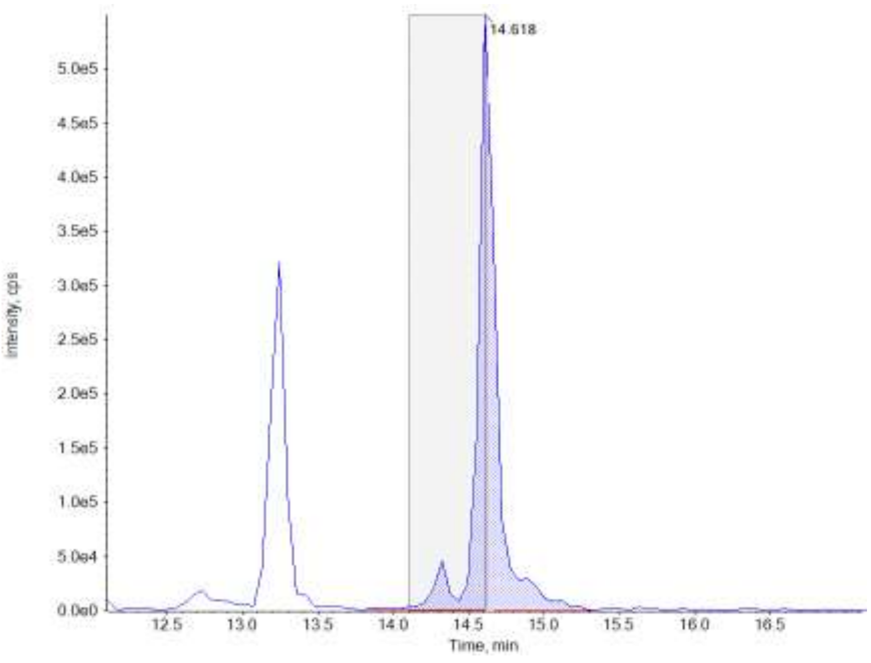

Acquired / Library MSMS

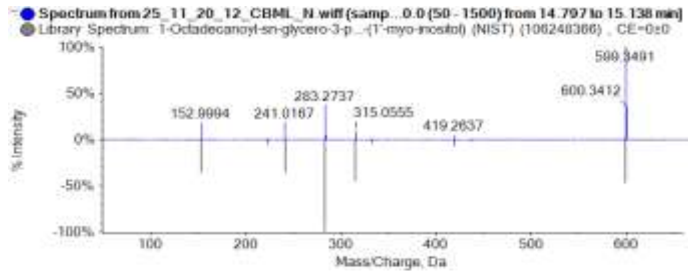

Acquired / Theoretical MS

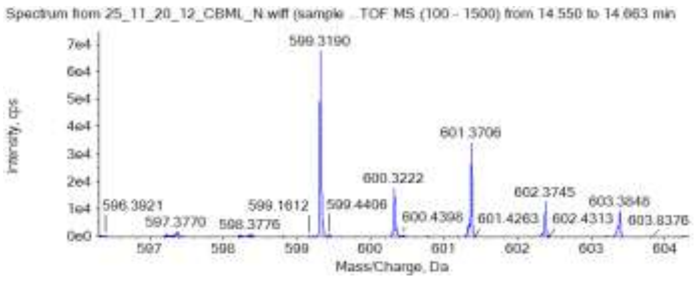

601.3718 / 14.72 (Mass/FragMass/RT/Isotope/Library/Formula/Ion Ratio)

**Retention Time:** 14.78 minutes	**Exp RT:** 14.72 minutes
**Precursor m/z :** 601.3718	**Analyte Name:**
**Fit (%)** 93.8% **RFit (%)** 89.9%	601.3718 / 14.72

Collision Energy = 35 ± 15 eV

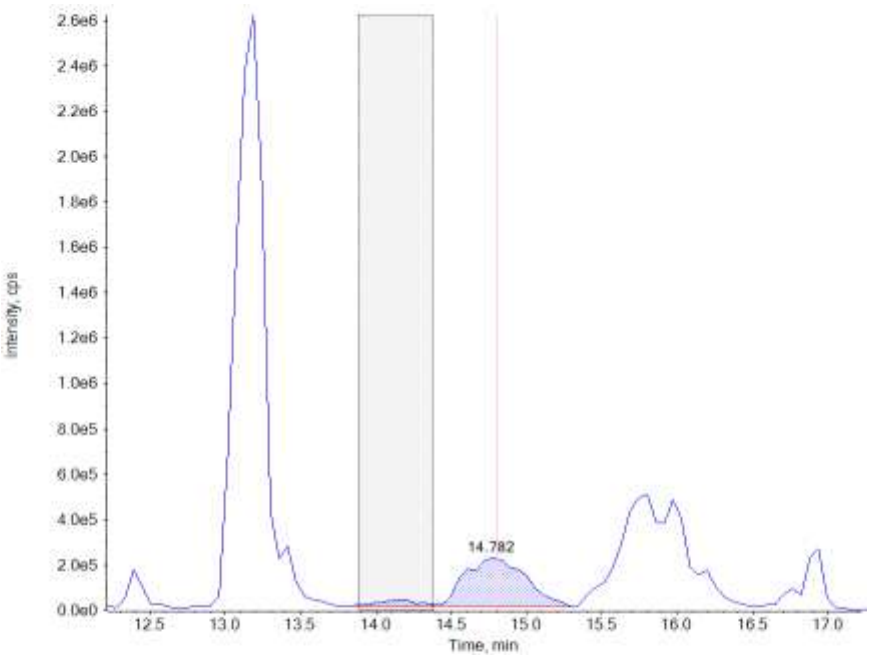

Acquired / Library MSMS

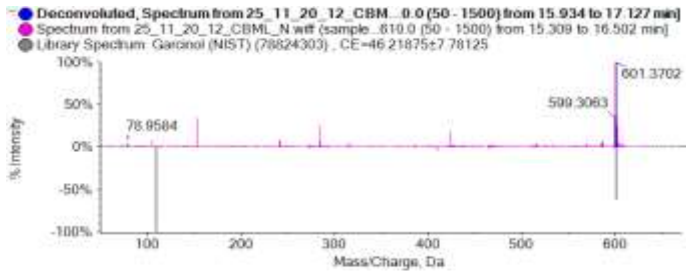

Acquired / Theoretical MS

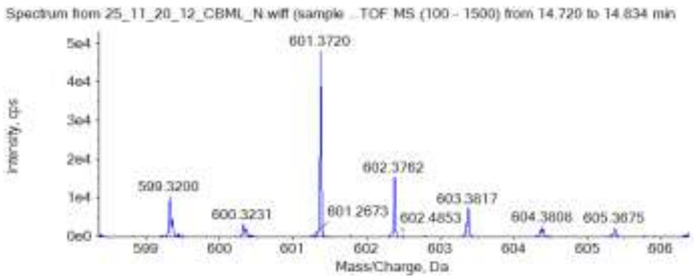

291.1955 / 14.83 (Mass/FragMass/RT/Isotope/Library/Formula/Ion Ratio)

**Retention Time:** 14.84 minutes<br>**Precursor m/z :** 291.1955<br>**Fit (%)** 82.3% **RFit (%)** 78.3%	**Exp RT:** 14.83 minutes<br>**Analyte Name:**<br>291.1955 / 14.83

Collision Energy = 35 ± 15 eV

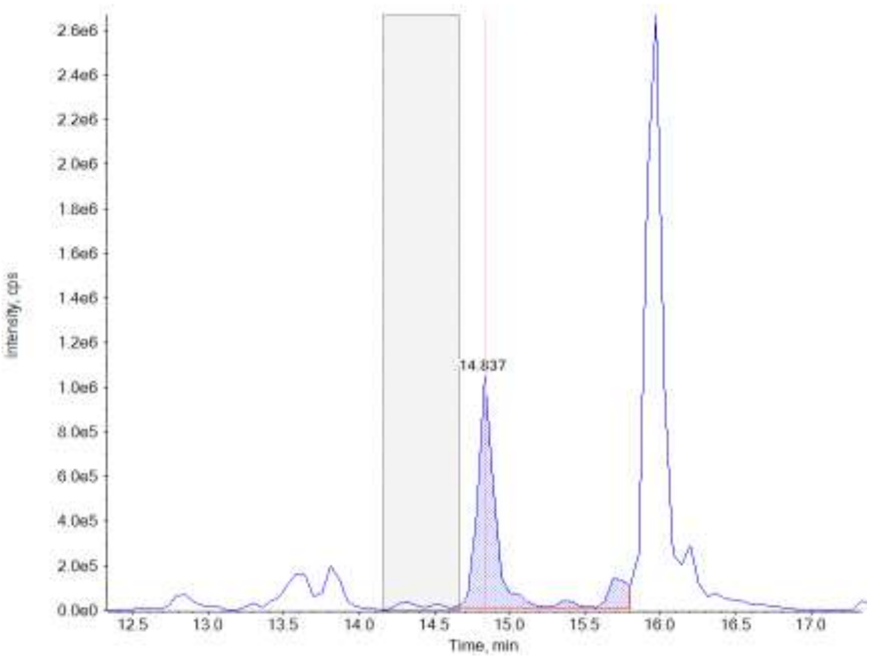

Acquired / Library MSMS

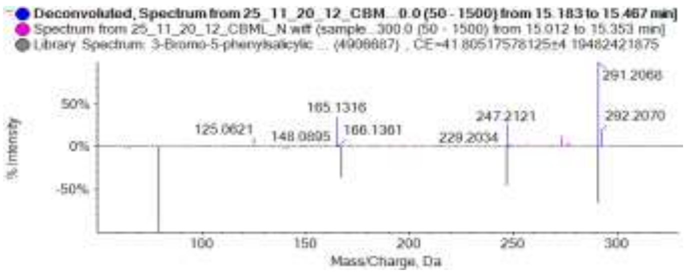

Acquired / Theoretical MS

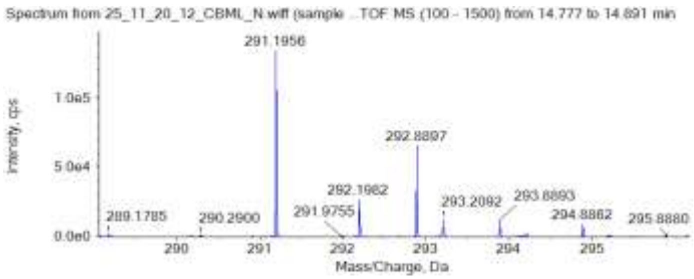

365.2676 / 15.00 (Mass/FragMass/RT/Isotope/Library/Formula/Ion Ratio)

Retention Time: 15.19 minutes
Precursor m/z : 365.2676
Fit (%) 94.3% **RFit (%)** 92.4%

Exp RT: 15.00 minutes
Analyte Name:
365.2676 / 15.00

Collision Energy = 35 ± 15 eV

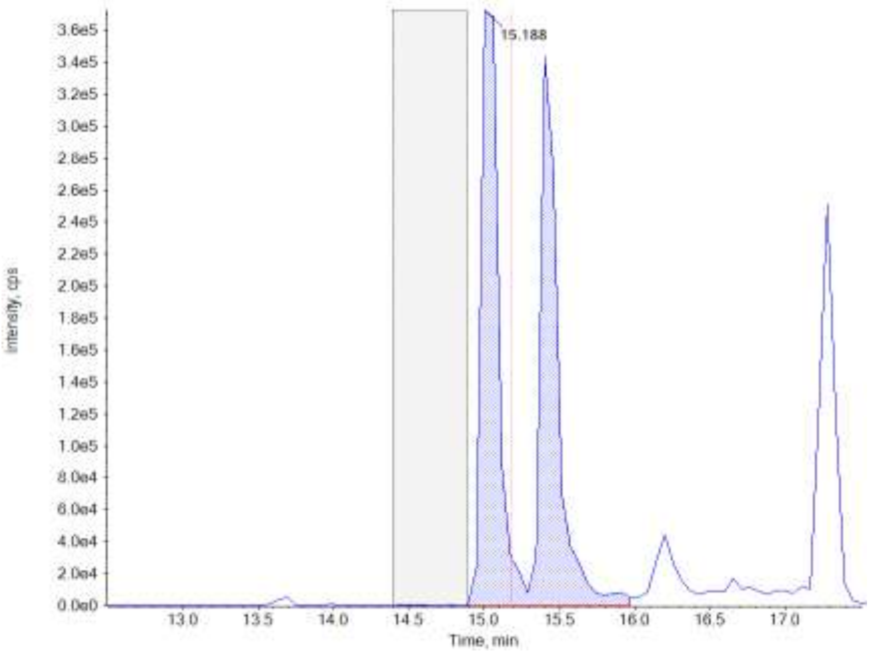

Acquired / Library MSMS

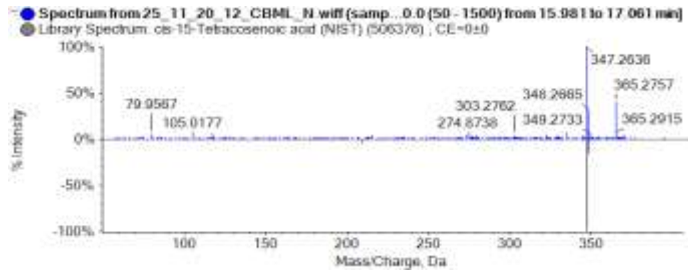

Acquired / Theoretical MS

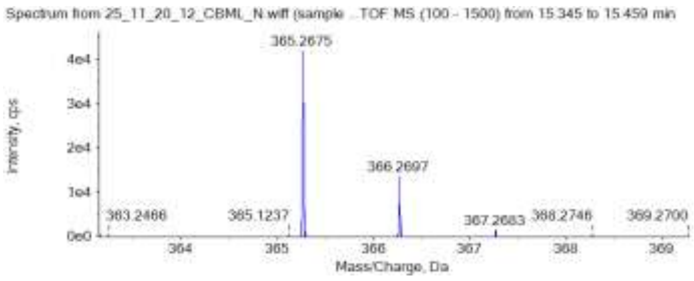

391.2226 / 15.06

(Mass/FragMass/RT/Isotope/Library/Formula/Ion Ratio)

Retention Time: 15.03 minutes
Precursor m/z : 391.2226
Fit (%) 86.5% **RFit (%)** 97.4%

Exp RT: 15.06 minutes
Analyte Name:
391.2226 / 15.06

Collision Energy = 35 ± 15 eV

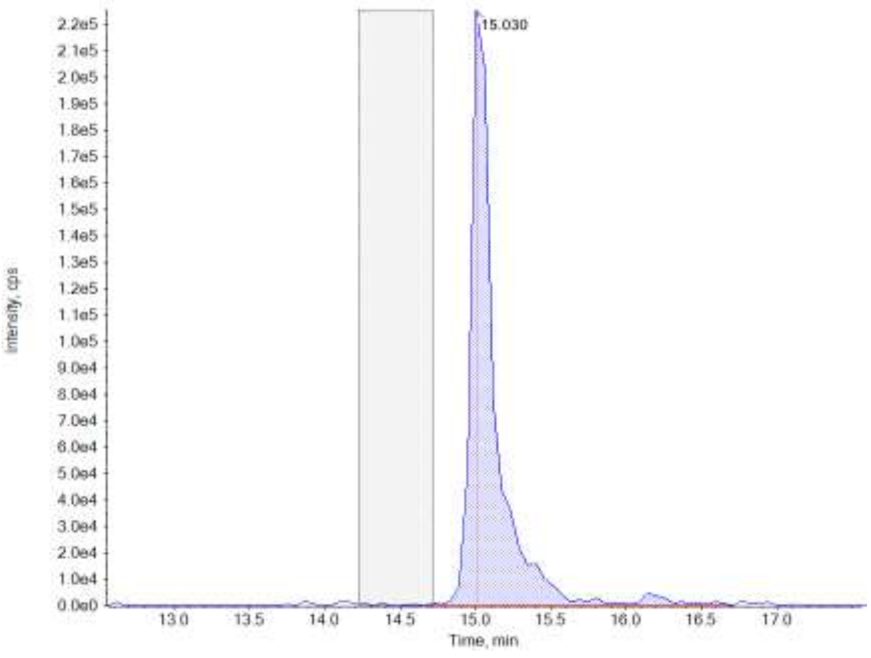

Acquired / Library MSMS

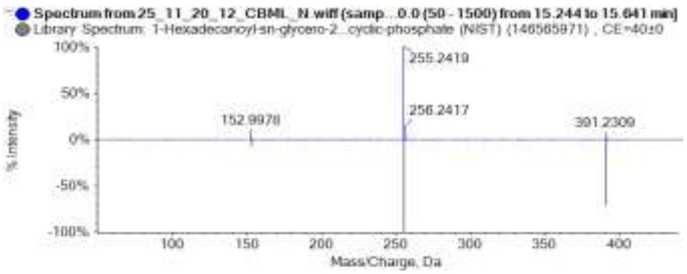

Acquired / Theoretical MS

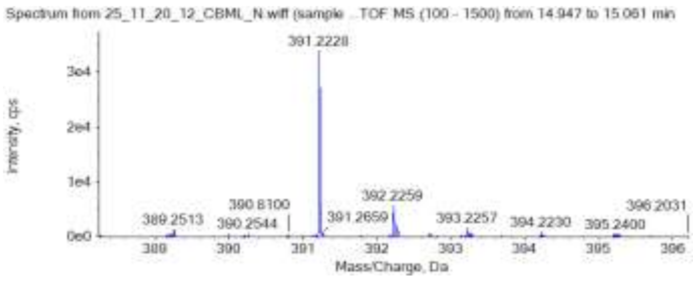

517.3546 / 15.12 (Mass/FragMass/RT/Isotope/Library/Formula/Ion Ratio)

**Retention Time:** 15.12 minutes	**Exp RT:** 15.12 minutes
**Precursor m/z :** 517.3546	**Analyte Name:**
**Fit (%)** 57.0% **RFit (%)** 98.0%	517.3546 / 15.12

Collision Energy = 35 ± 15 eV

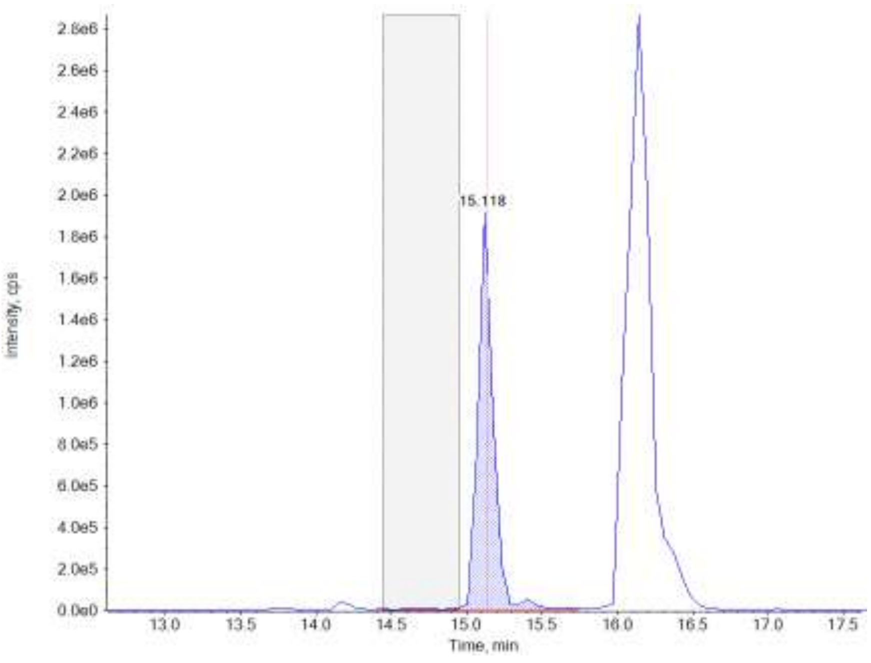

Acquired / Library MSMS

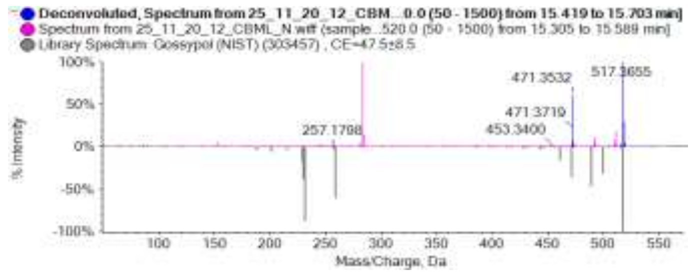

Acquired / Theoretical MS

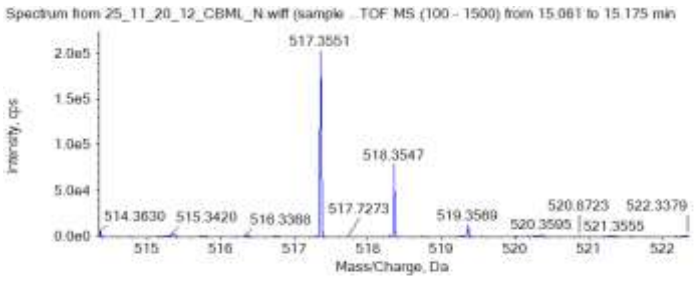

275.2002 / 15.17 [M-H2O-H]- (Mass/FragMass/RT/Isotope/Library/Formula/Ion Ratio)

**Retention Time:** 15.24 minutes	**Exp RT:** 15.18 minutes
**Precursor m/z :** 275.2002	**Analyte Name:**
**Fit (%)** 90.7% **RFit (%)** 92.2%	275.2002 / 15.17 [M-H2O-H]-

Collision Energy = 35 ± 15 eV

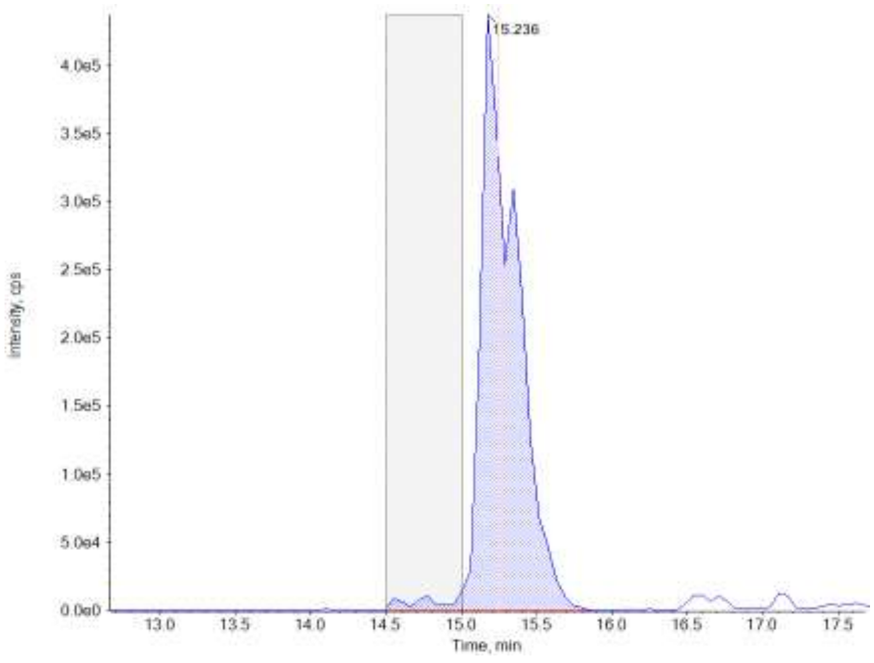

Acquired / Library MSMS

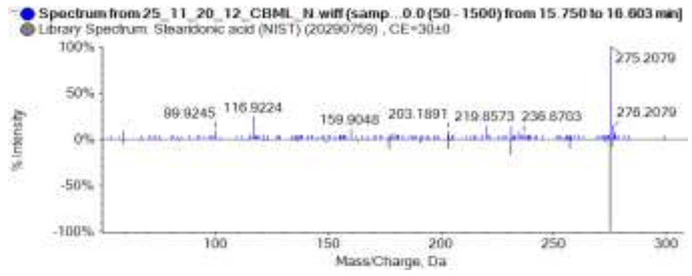

Acquired / Theoretical MS

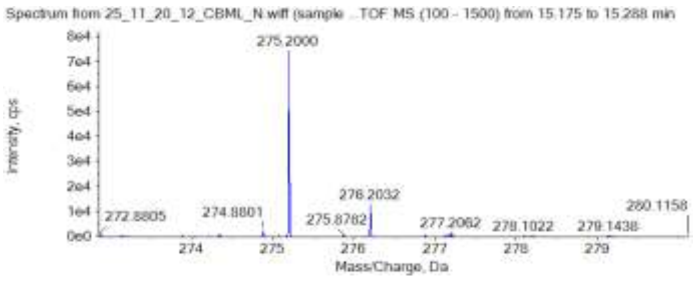

293.2112 / 15.74 [M-H]- (Mass/FragMass/RT/Isotope/Library/Formula/Ion Ratio)

Retention Time: 15.41 minutes
Precursor m/z : 293.2112
Fit (%) 99.5% **RFit (%)** 98.5%

Exp RT: 15.74 minutes
Analyte Name:
293.2112 / 15.74 [M-H]-

Collision Energy = 35 ± 15 eV

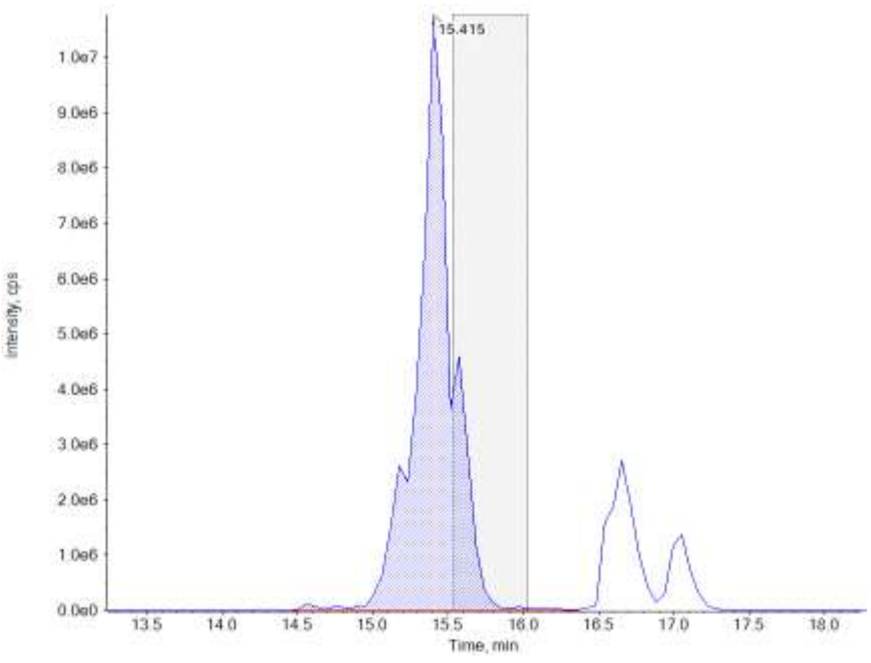

Acquired / Library MSMS

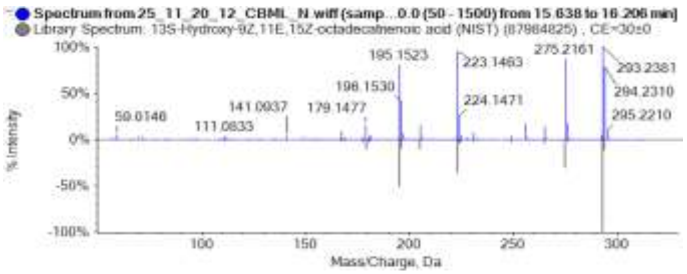

Acquired / Theoretical MS

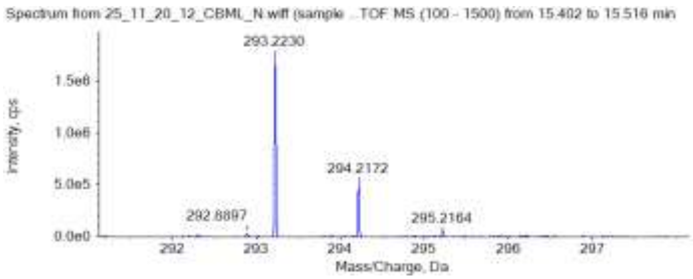

865.5004 / 15.23 (Mass/FragMass/RT/Isotope/Library/Formula/Ion Ratio)

**Retention Time:** 15.25 minutes	**Exp RT:** 15.23 minutes
**Precursor m/z :** 865.5004	**Analyte Name:**
**Fit (%)** 26.9% **RFit (%)** 99.7%	865.5004 / 15.23

Collision Energy = 35 ± 15 eV

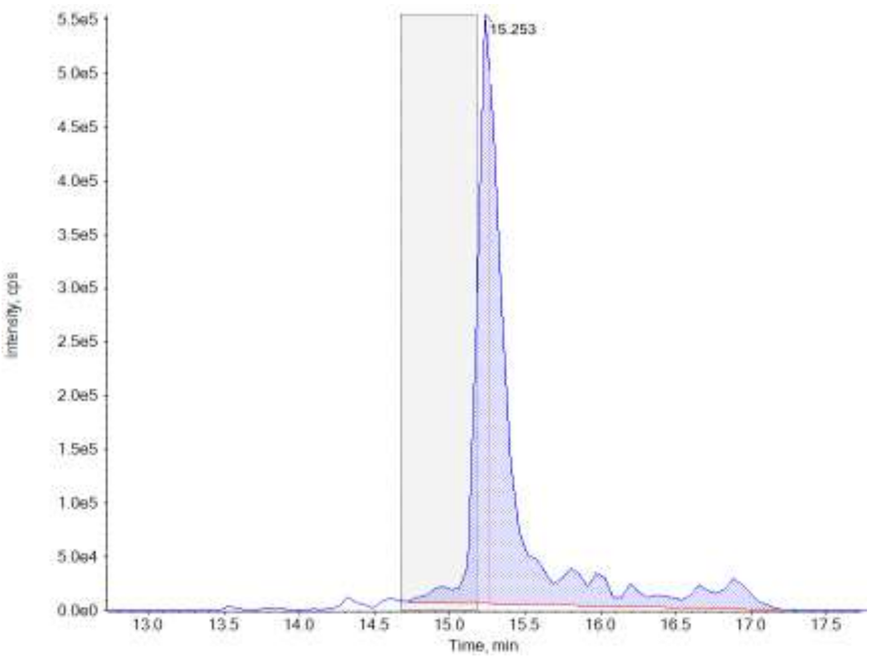

Acquired / Library MSMS

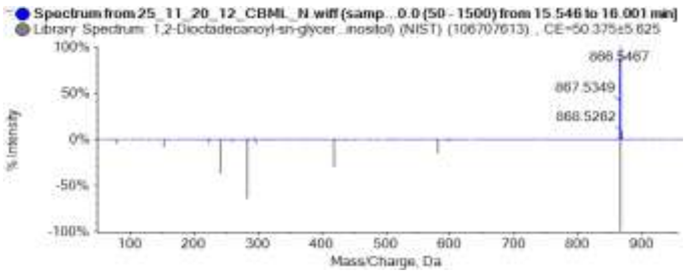

Acquired / Theoretical MS

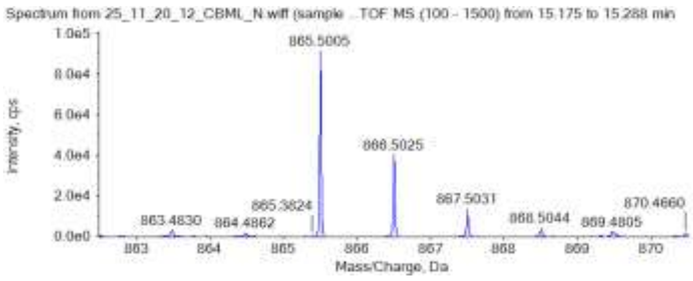

295.2166 / 15.35

(Mass/FragMass/RT/Isotope/Library/Formula/Ion Ratio)

Retention Time: 15.33 minutes
Precursor m/z : 295.2166
Fit (%) 33.3% **RFit (%)** 92.7%

Exp RT: 15.35 minutes
Analyte Name:
295.2166 / 15.35

Collision Energy = 35 ± 15 eV

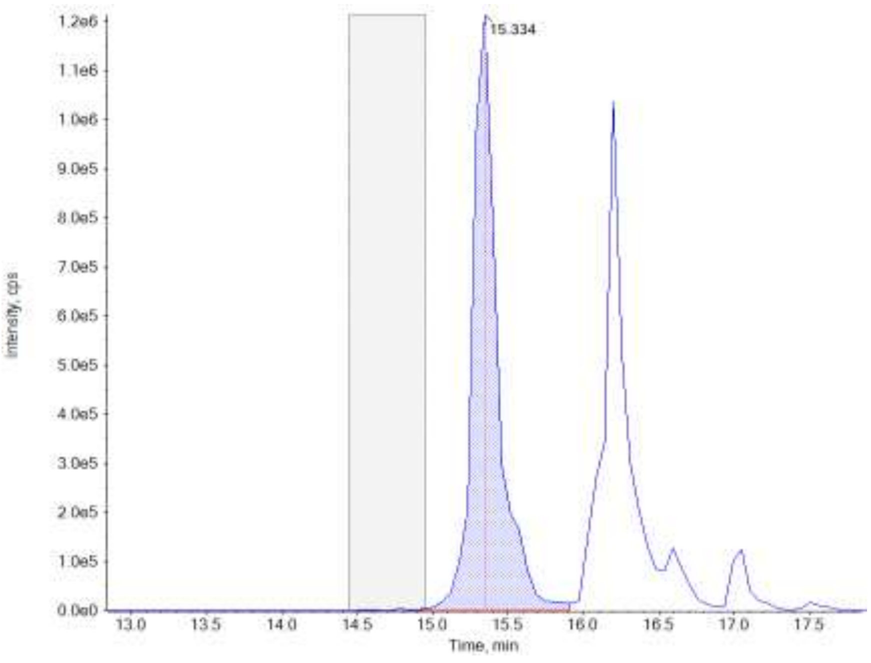

Acquired / Library MSMS

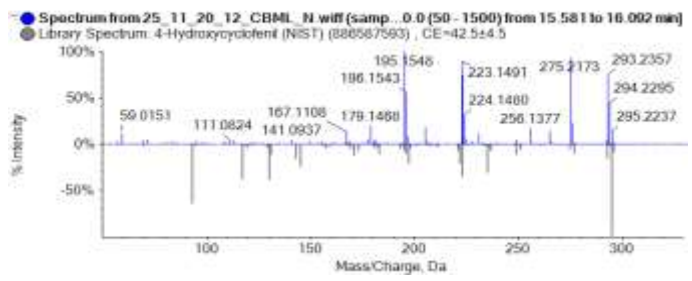

Acquired / Theoretical MS

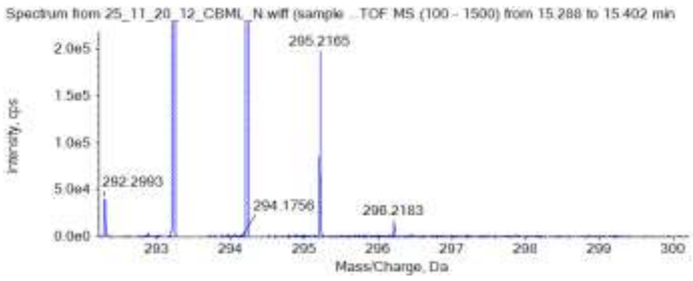

293.2237 / 15.40 (Mass/FragMass/RT/Isotope/Library/Formula/Ion Ratio)

**Retention Time:** 15.39 minutes	**Exp RT:** 15.40 minutes
**Precursor m/z :** 293.2237	**Analyte Name:**
**Fit (%)** 99.5% **RFit (%)** 98.5%	293.2237 / 15.40

Collision Energy = 35 ± 15 eV

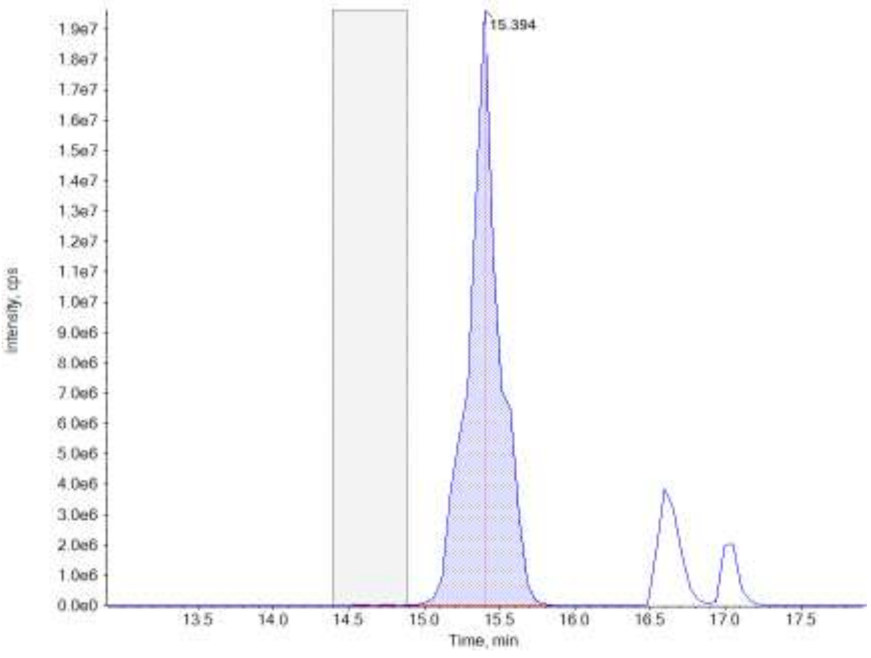

Acquired / Library MSMS

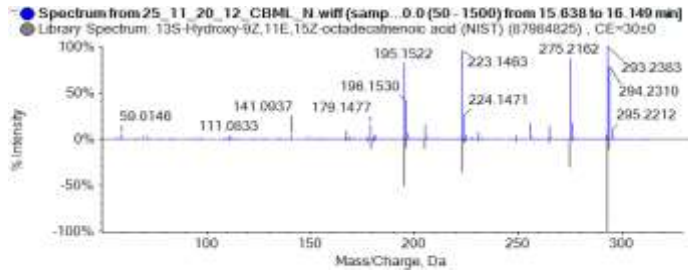

Acquired / Theoretical MS

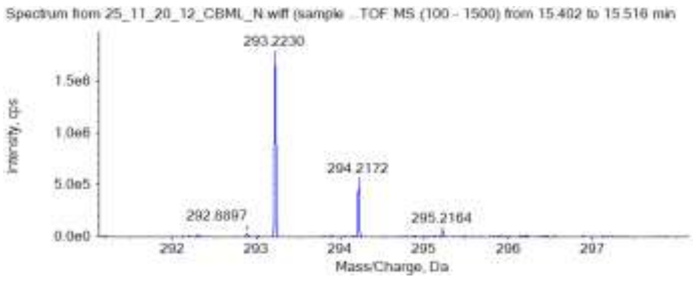

487.3412 / 15.40 (Mass/FragMass/RT/Isotope/Library/Formula/Ion Ratio)

Retention Time: 15.42 minutes
Precursor m/z : 487.3412
Fit (%) 100.0% **RFit (%)** 100.0%

Exp RT: 15.40 minutes
Analyte Name:
487.3412 / 15.40

Collision Energy = 35 ± 15 eV

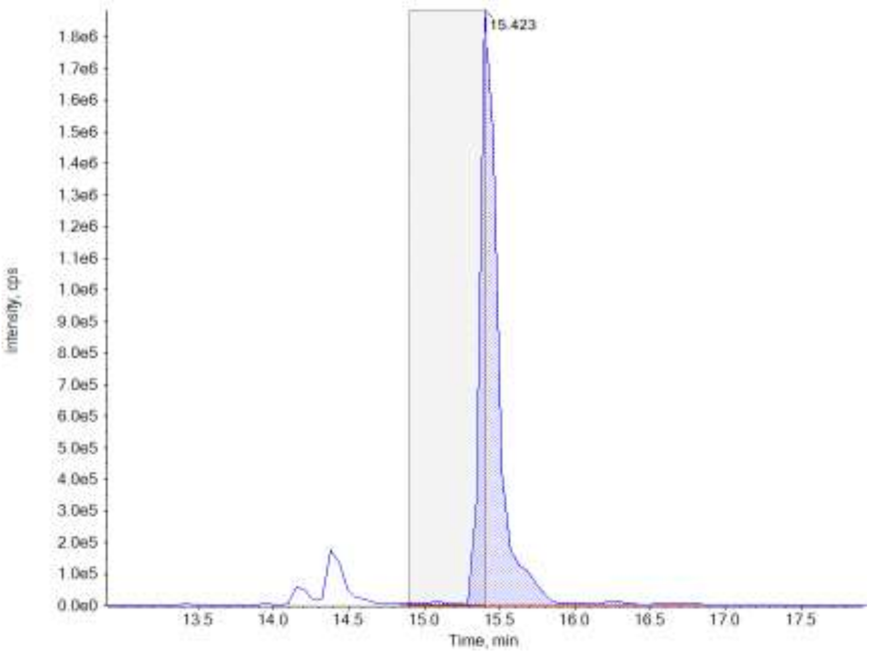

Acquired / Library MSMS

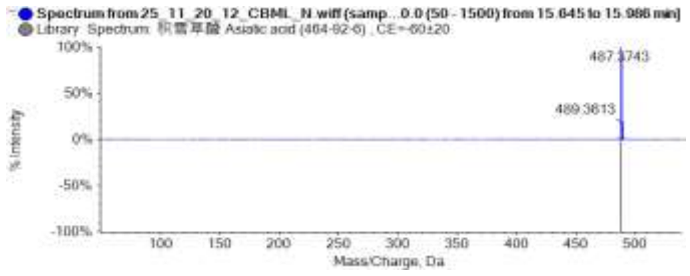

Acquired / Theoretical MS

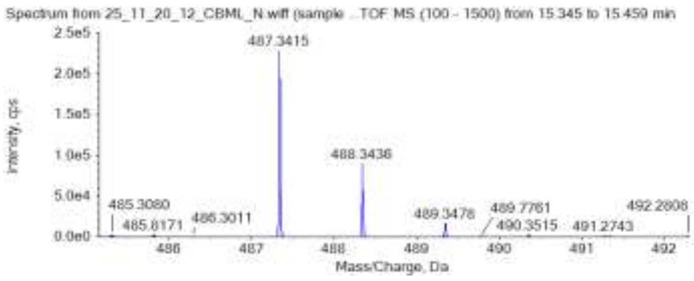

511.3052 / 15.40 (Mass/FragMass/RT/Isotope/Library/Formula/Ion Ratio)

**Retention Time:** 15.43 minutes<br>**Precursor m/z :** 511.3052<br>**Fit (%)** 92.8% **RFit (%)** 92.5%	**Exp RT:** 15.40 minutes<br>**Analyte Name:**<br>511.3052 / 15.40

		Collision Energy = 35 ± 15 eV
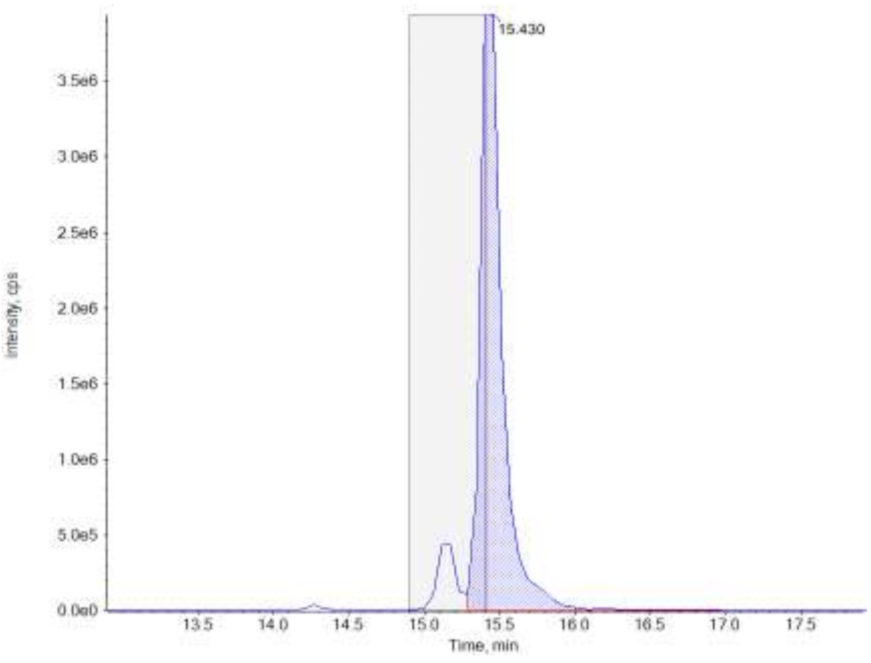	Acquired / Library MSMS	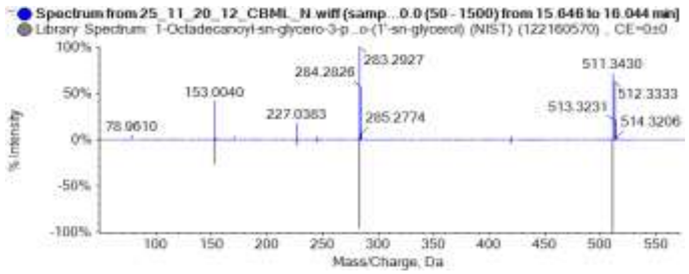
	Acquired / Theoretical MS	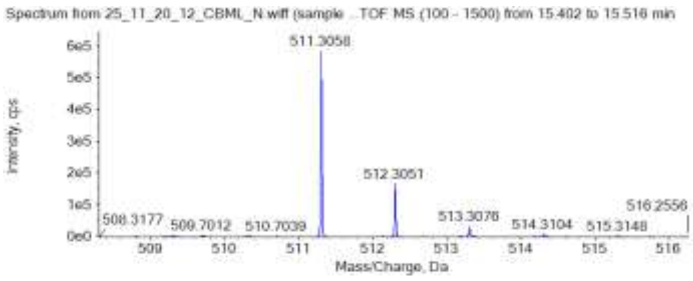

Created with SCIEX OS 3.3.0
Printed:12/9/2025 2:25:51 PM

601.3741 / 15.80 [M-H2O-H]- (Mass/FragMass/RT/Isotope/Library/Formula/Ion Ratio)

**Retention Time:** 15.84 minutes	**Exp RT:** 15.80 minutes
**Precursor m/z :** 601.3741	**Analyte Name:**
**Fit (%)** 90.9% **RFit (%)** 45.9%	601.3741 / 15.80 [M-H2O-H]-

	**Collision Energy = 35 ± 15 eV**

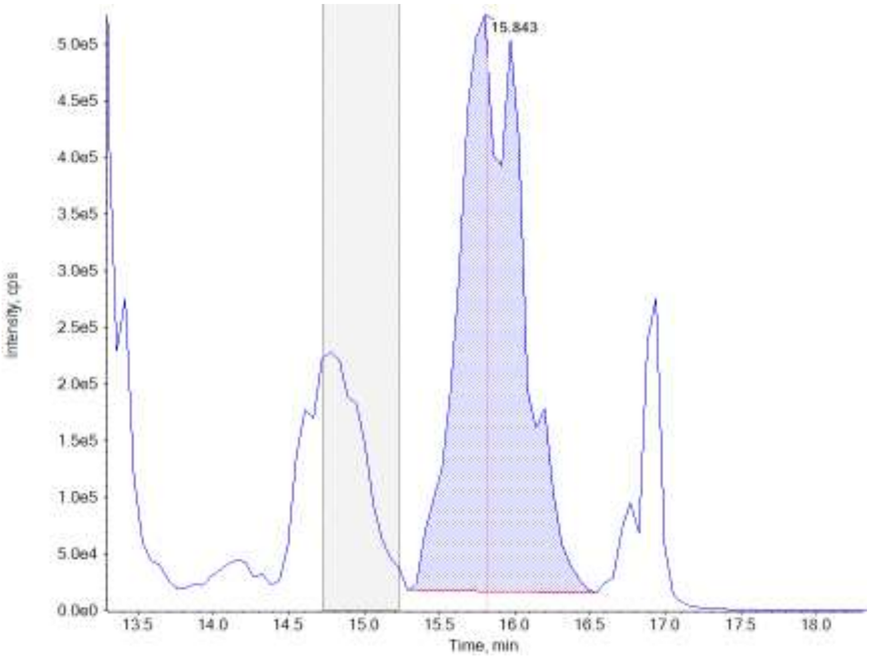

Acquired / Library MSMS

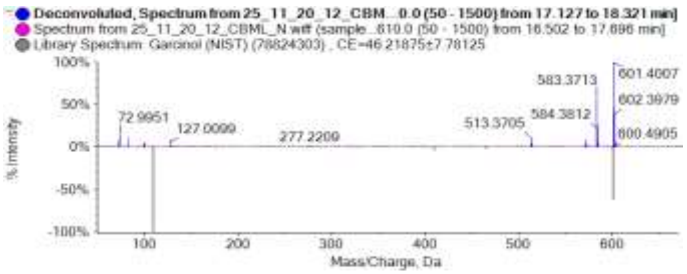

Acquired / Theoretical MS

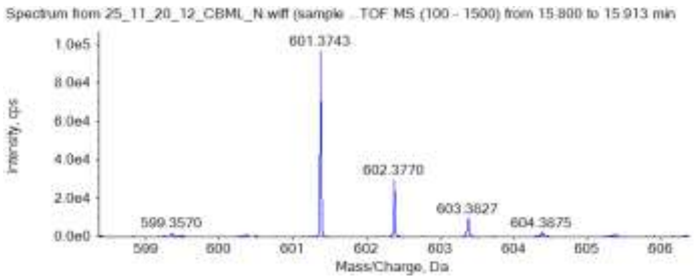

291.1983 / 15.97

(Mass/FragMass/RT/Isotope/Library/Formula/Ion Ratio)

Retention Time: 15.96 minutes
Precursor m/z : 291.1983
Fit (%) 96.5% **RFit (%)** 94.9%

Exp RT: 15.97 minutes
Analyte Name:
291.1983 / 15.97

Collision Energy = 35 ± 15 eV

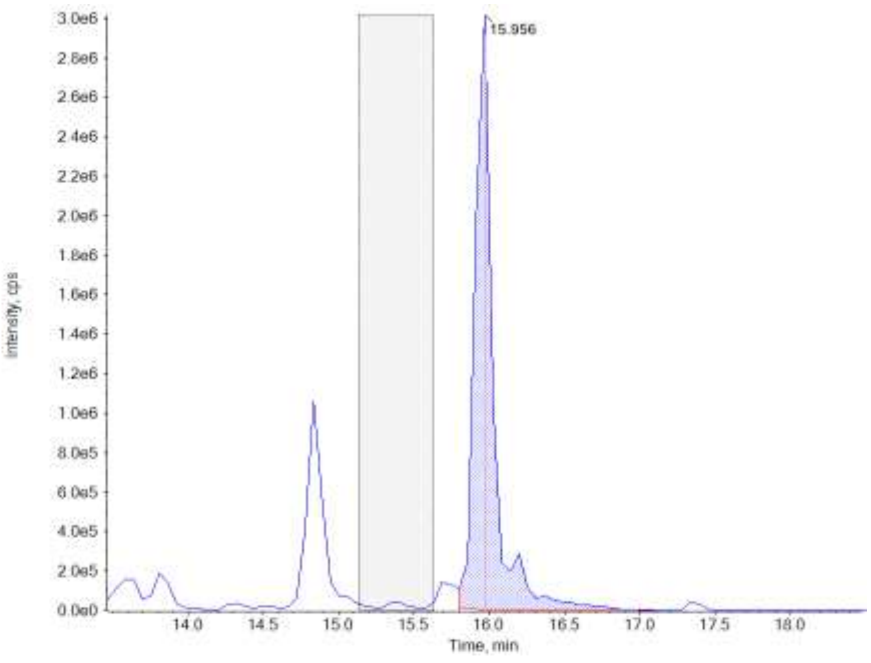

Acquired / Library MSMS

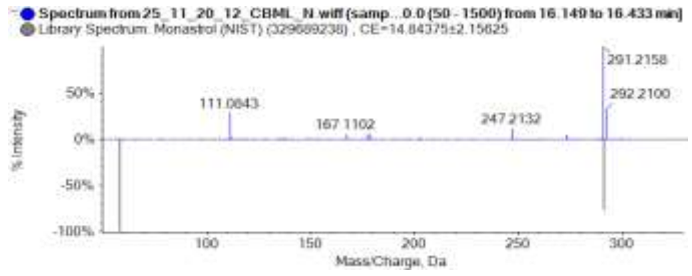

Acquired / Theoretical MS

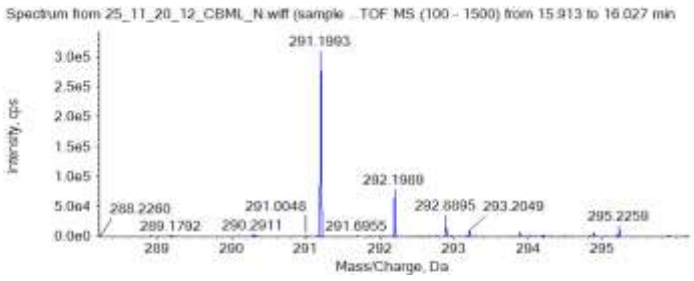

699.3816 / 16.08 (Mass/FragMass/RT/Isotope/Library/Formula/Ion Ratio)

Retention Time: 16.11 minutes
Precursor m/z : 699.3816
Fit (%) 91.3% **RFit (%)** 61.2%

Exp RT: 16.08 minutes
Analyte Name:
699.3816 / 16.08

Collision Energy = 35 ± 15 eV

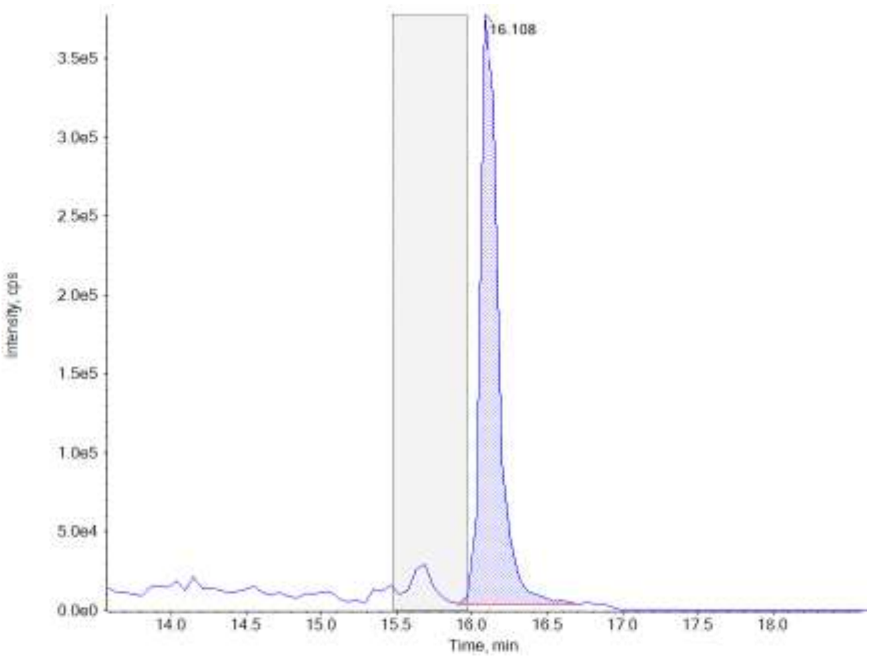

Acquired / Library MSMS

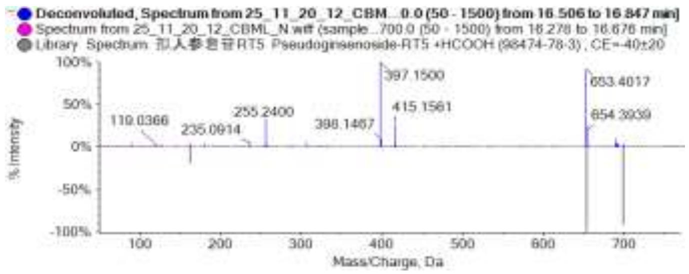

Acquired / Theoretical MS

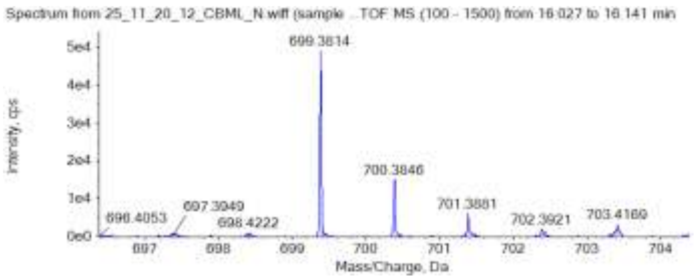

295.2357 / 16.14 (Mass/FragMass/RT/Isotope/Library/Formula/Ion Ratio)

**Retention Time:** 16.14 minutes	**Exp RT:** 16.14 minutes
**Precursor m/z :** 295.2357	**Analyte Name:**
**Fit (%)** 98.7% **RFit (%)** 98.7%	295.2357 / 16.14

Collision Energy = 35 ± 15 eV

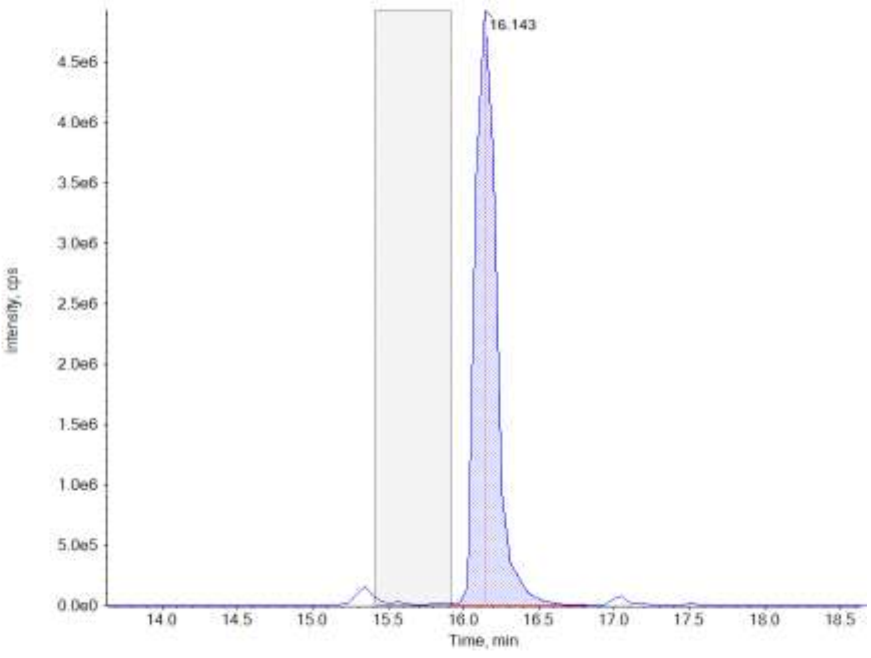

Acquired / Library MSMS

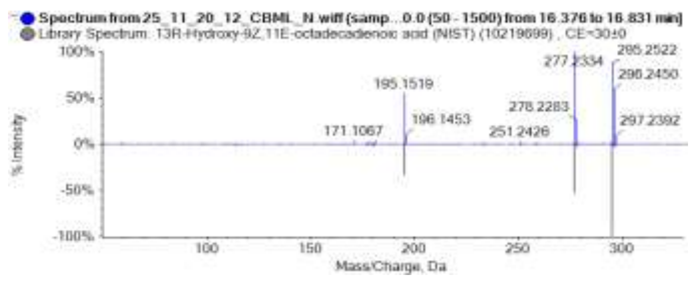

Acquired / Theoretical MS

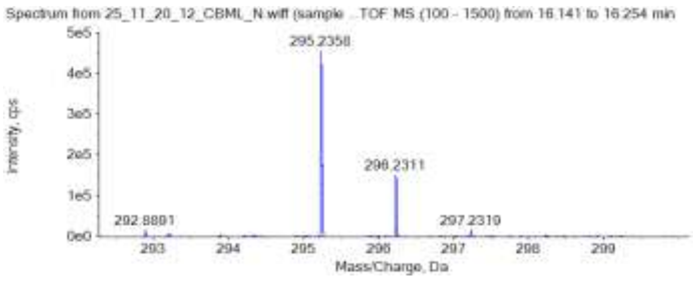

517.3611 / 16.14 (Mass/FragMass/RT/Isotope/Library/Formula/Ion Ratio)

Retention Time: 16.12 minutes
Precursor m/z : 517.3611
Fit (%) 70.9% **RFit (%)** 98.1%

Exp RT: 16.14 minutes
Analyte Name:
517.3611 / 16.14

Collision Energy = 35 ± 15 eV

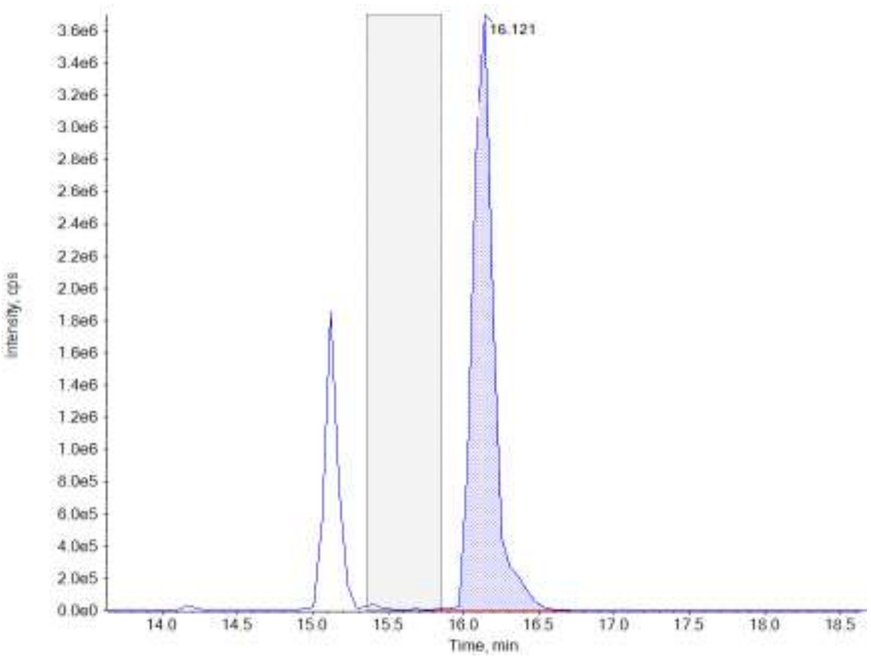

Acquired / Library MSMS

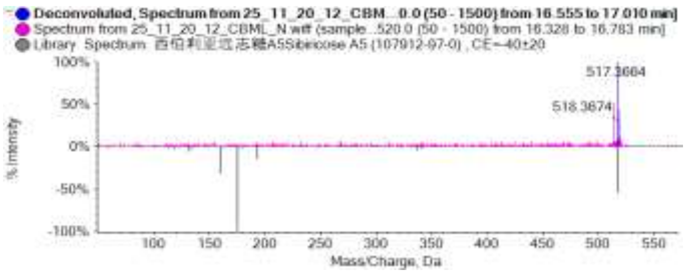

Acquired / Theoretical MS

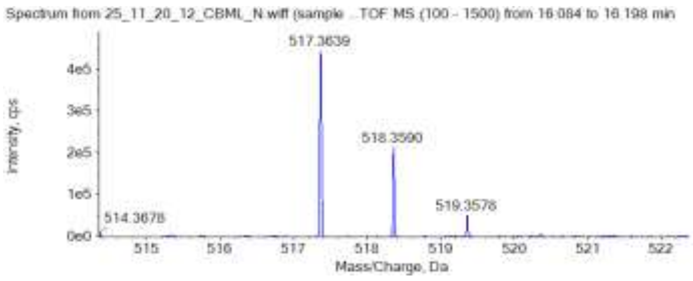

573.3777 / 16.77 [M+AcO-H]- (Mass/FragMass/RT/Isotope/Library/Formula/Ion Ratio)

Retention Time: 16.74 minutes
Precursor m/z : 573.3777
Fit (%) 38.1% **RFit (%)** 99.3%

Exp RT: 16.77 minutes
Analyte Name:
573.3777 / 16.77 [M+AcO-H]-

Collision Energy = 35 ± 15 eV

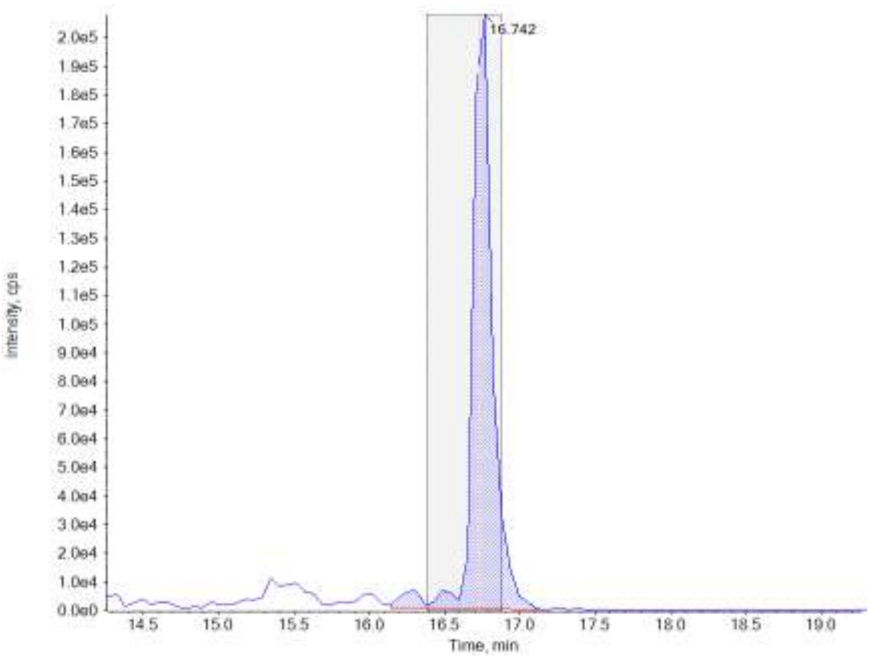

Acquired / Library MSMS

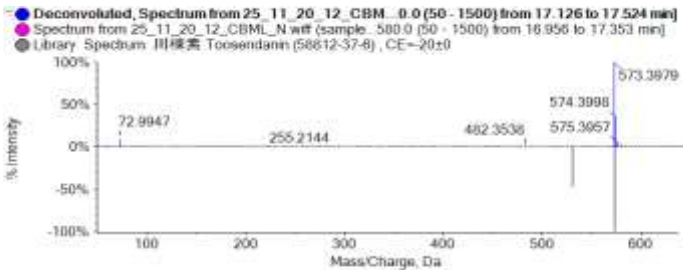

Acquired / Theoretical MS

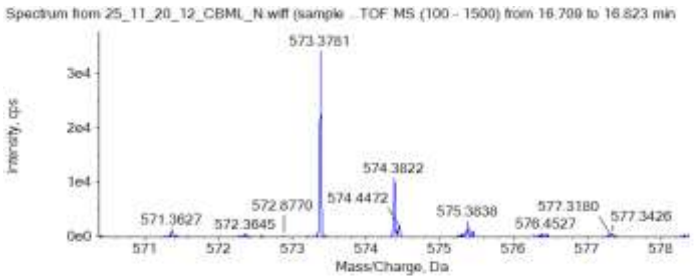

SCIEX

Created with SCIEX OS 3.3.0
Printed:12/9/2025 2:25:51 PM

293.2188 / 16.60 (Mass/FragMass/RT/Isotope/Library/Formula/Ion Ratio)

Retention Time: 16.63 minutes
Precursor m/z : 293.2188
Fit (%) 98.2% **RFit (%)** 100.0%

Exp RT: 16.60 minutes
Analyte Name:
293.2188 / 16.60

Collision Energy = 35 ± 15 eV

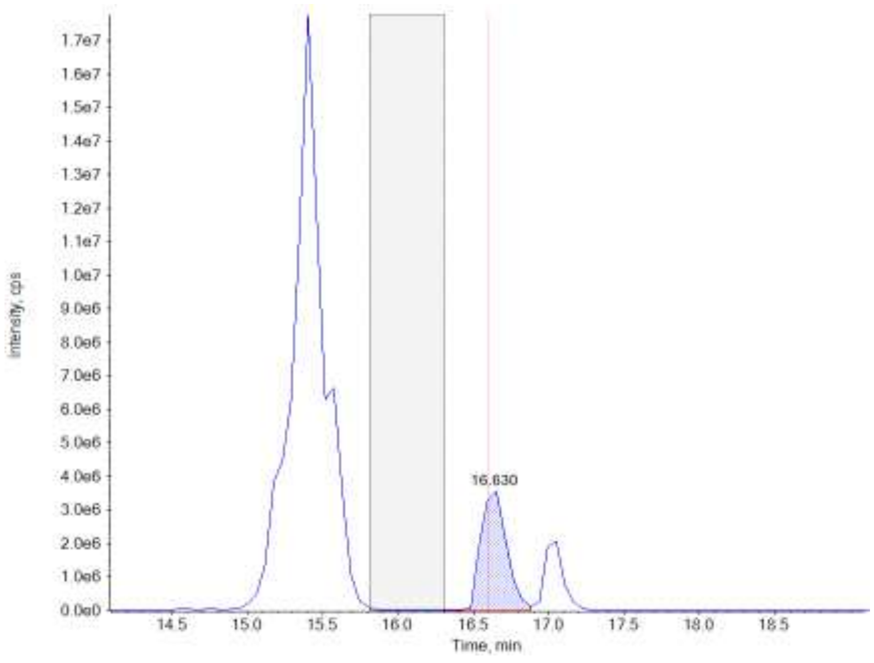

Acquired / Library MSMS

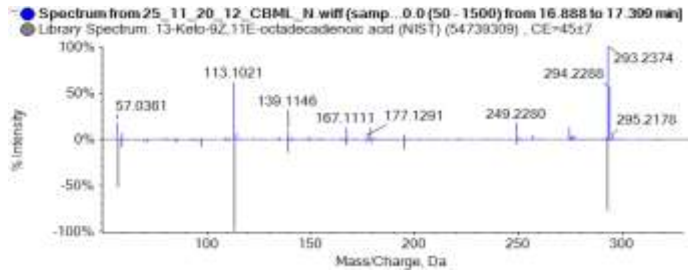

Acquired / Theoretical MS

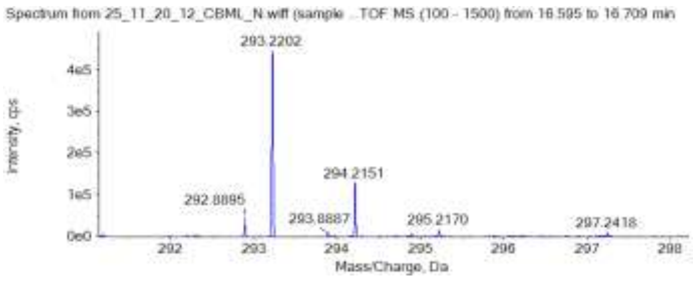

505.2998 / 16.71 (Mass/FragMass/RT/Isotope/Library/Formula/Ion Ratio)

Retention Time: 16.70 minutes
Precursor m/z : 505.2998
Fit (%) 35.1% **RFit (%)** 92.8%

Exp RT: 16.71 minutes
Analyte Name:
505.2998 / 16.71

Collision Energy = 35 ± 15 eV

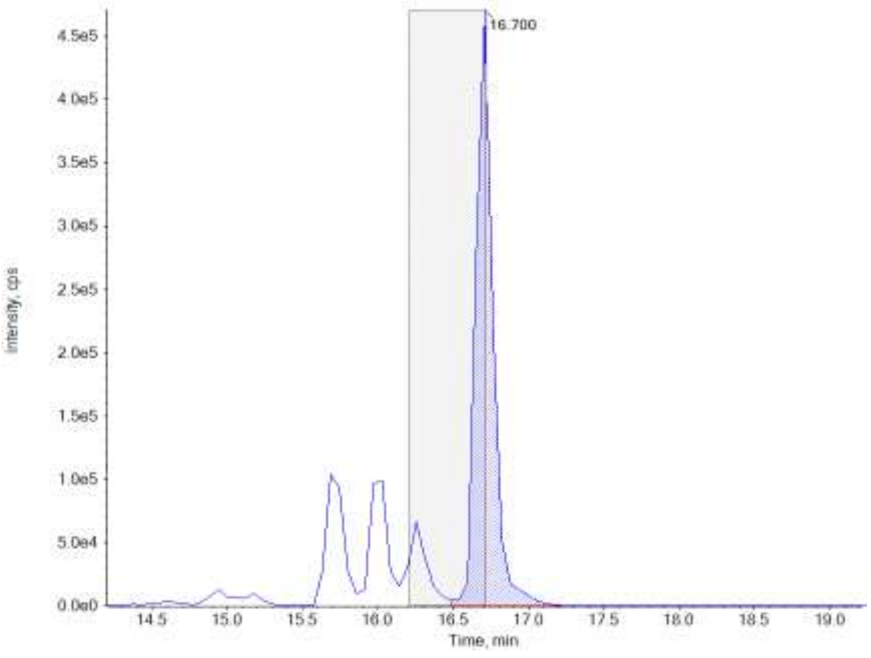

Acquired / Library MSMS

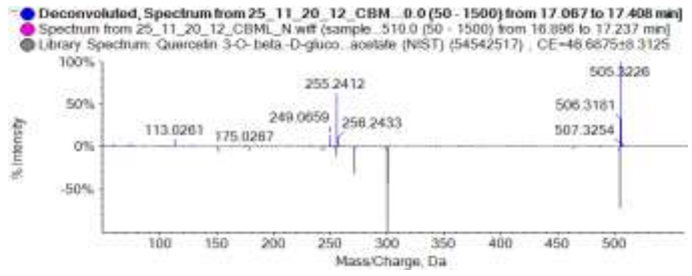

Acquired / Theoretical MS

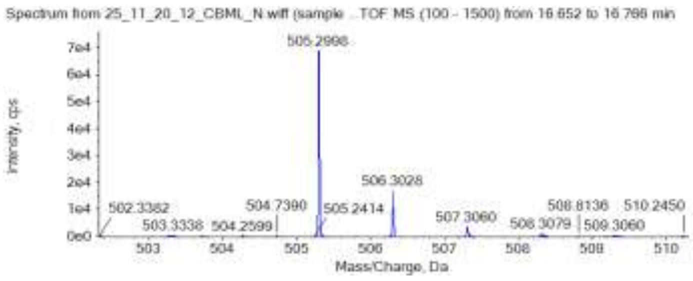

Created with SCIEX OS 3.3.0
Printed:12/9/2025 2:25:51 PM

585.3797 / 16.71 (Mass/FragMass/RT/Isotope/Library/Formula/Ion Ratio)

**Retention Time:** 16.71 minutes	**Exp RT:** 16.71 minutes
**Precursor m/z :** 585.3797	**Analyte Name:**
**Fit (%)** 76.8% **RFit (%)** 99.8%	585.3797 / 16.71

Collision Energy = 35 ± 15 eV

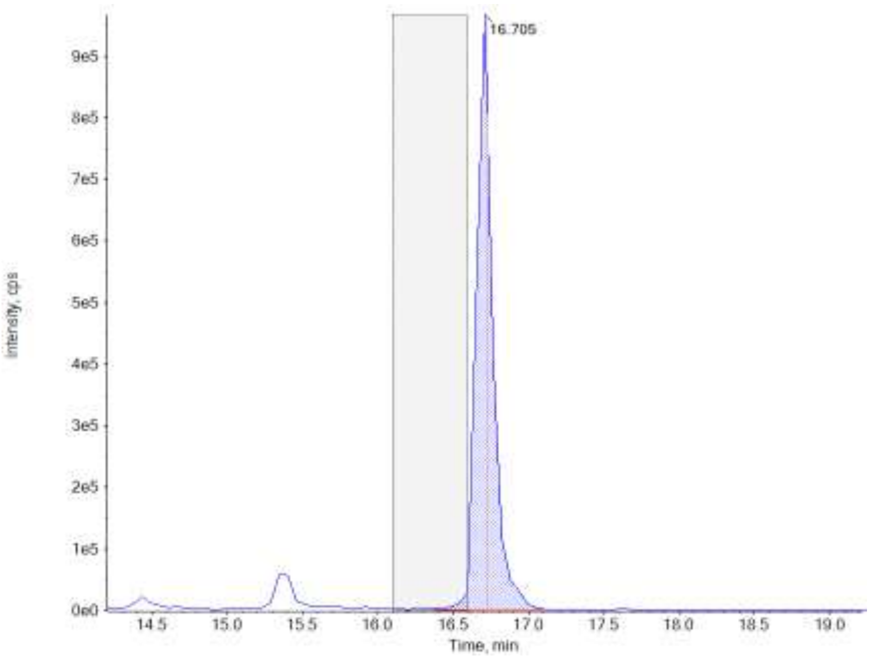

Acquired / Library MSMS

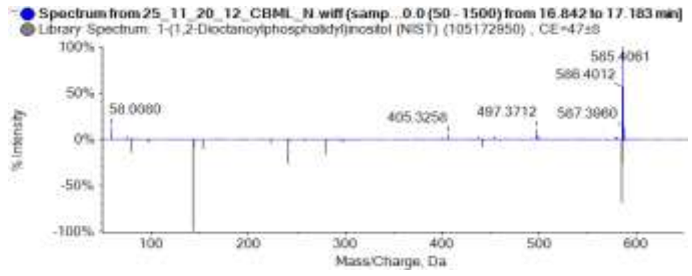

Acquired / Theoretical MS

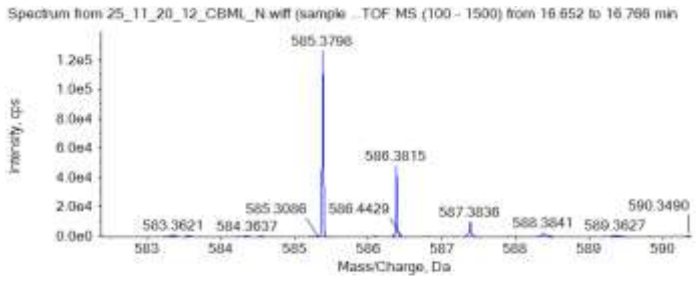

793.5150 / 16.77 (Mass/FragMass/RT/Isotope/Library/Formula/Ion Ratio)

Retention Time: 16.85 minutes
Precursor m/z : 793.5150
Fit (%) 100.0% **RFit (%)** 100.0%

Exp RT: 16.77 minutes
Analyte Name:
793.5150 / 16.77

Collision Energy = 35 ± 15 eV

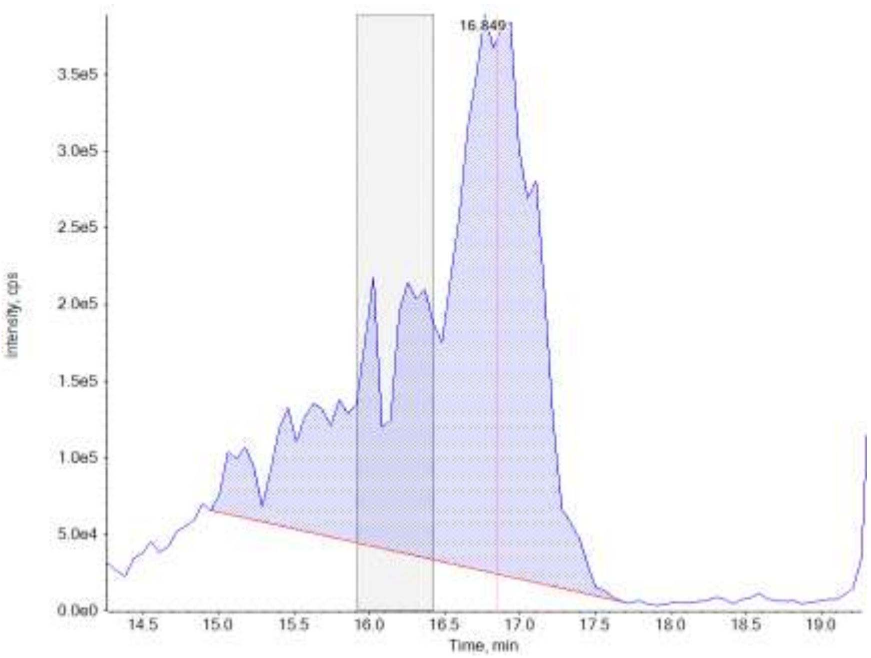

Acquired / Library MSMS

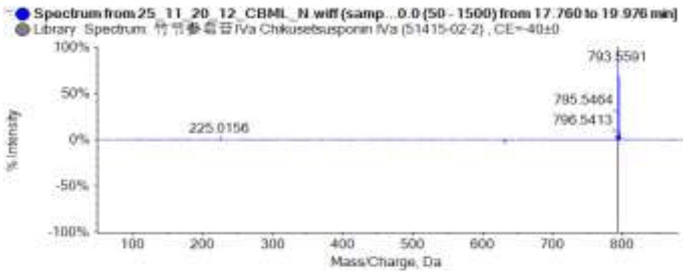

Acquired / Theoretical MS

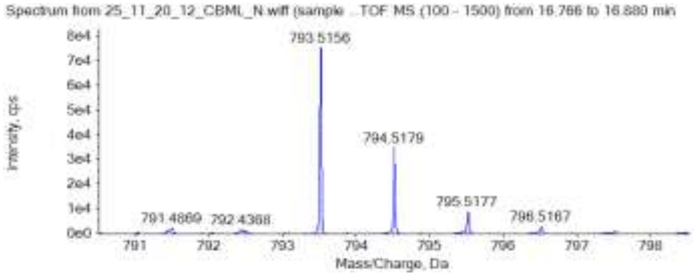

321.2420 / 16.88 [M-H]- (Mass/FragMass/RT/Isotope/Library/Formula/Ion Ratio)

Retention Time: 16.85 minutes
Precursor m/z : 321.2420
Fit (%) 88.9% **RFit (%)** 92.3%

Exp RT: 16.88 minutes
Analyte Name:
321.2420 / 16.88 [M-H]-

Collision Energy = 35 ± 15 eV

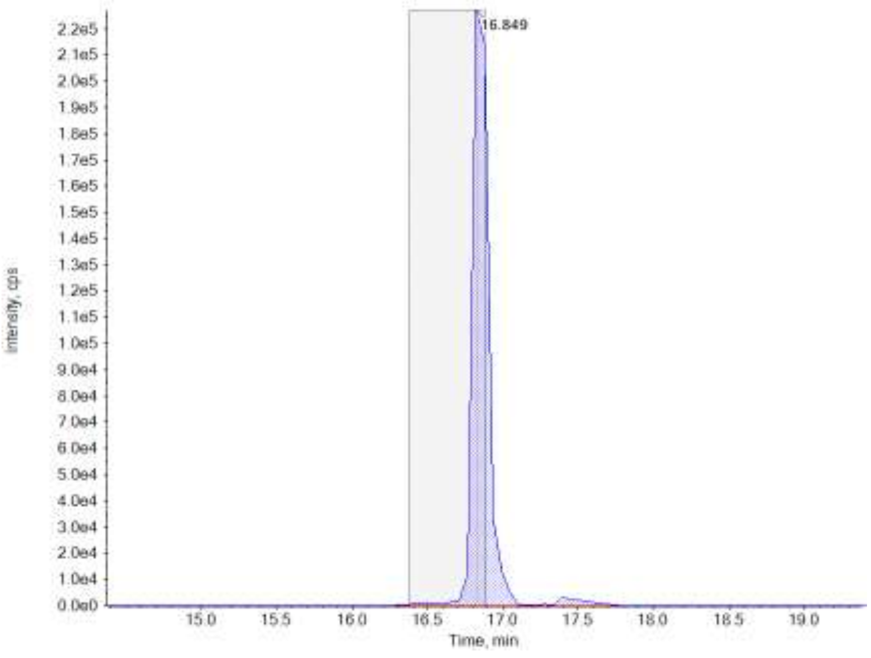

Acquired / Library MSMS

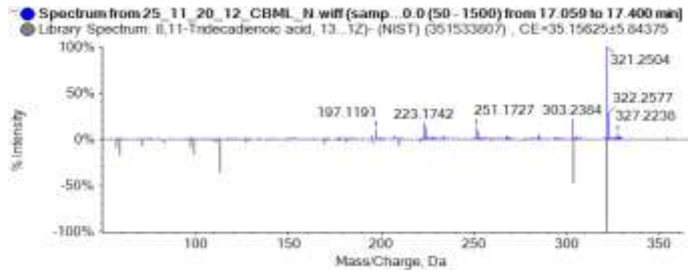

Acquired / Theoretical MS

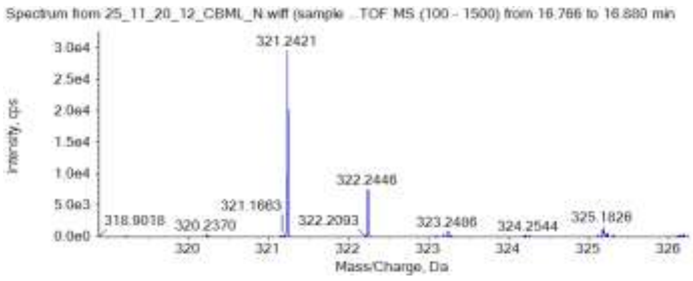

601.3736 / 16.94 (Mass/FragMass/RT/Isotope/Library/Formula/Ion Ratio)

**Retention Time:** 16.91 minutes	**Exp RT:** 16.94 minutes
**Precursor m/z :** 601.3736	**Analyte Name:**
**Fit (%)** 88.8% **RFit (%)** 100.0%	601.3736 / 16.94

Collision Energy = 35 ± 15 eV

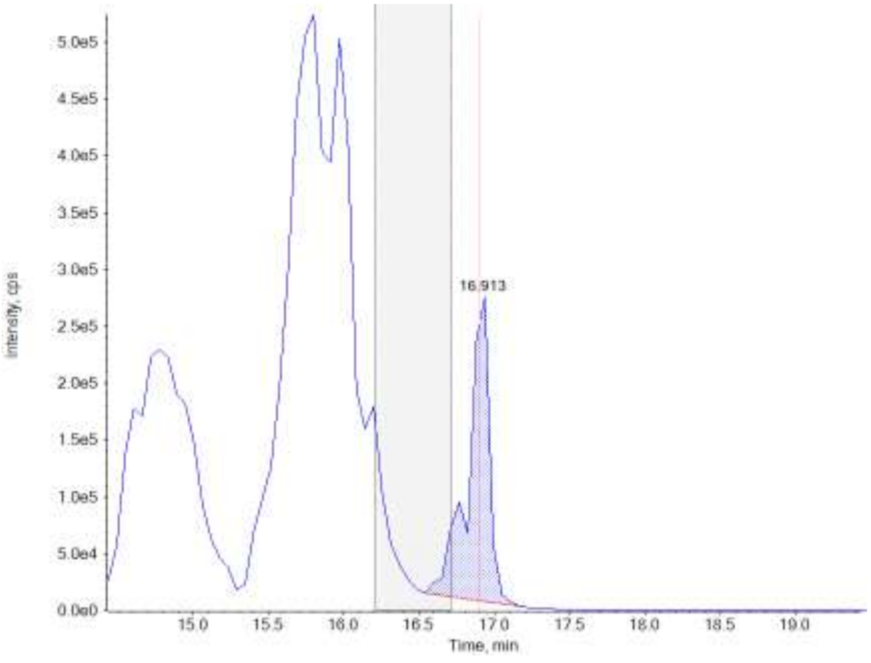

Acquired / Library MSMS

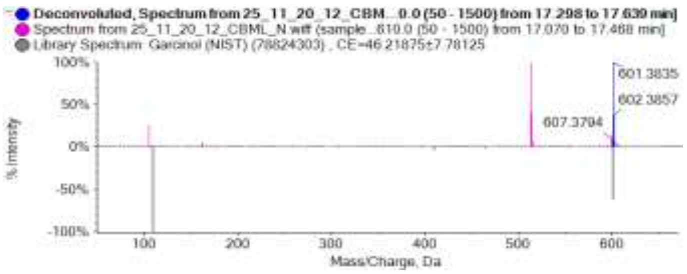

Acquired / Theoretical MS

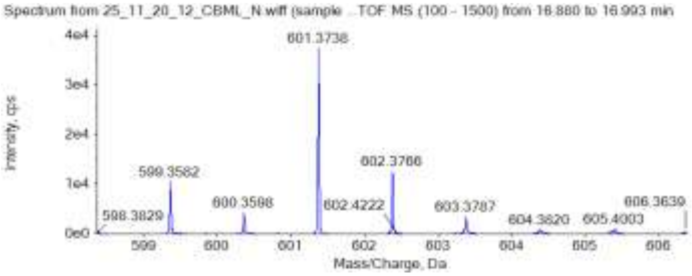

853.4794 / 16.65 [M+Cl]- (Mass/FragMass/RT/Isotope/Library/Formula/Ion Ratio)

**Retention Time:** 16.63 minutes	**Exp RT:** 16.65 minutes
**Precursor m/z :** 853.4794	**Analyte Name:**
**Fit (%)** 32.3% **RFit (%)** 100.0%	853.4794 / 16.65 [M+Cl]-

Collision Energy = 35 ± 15 eV

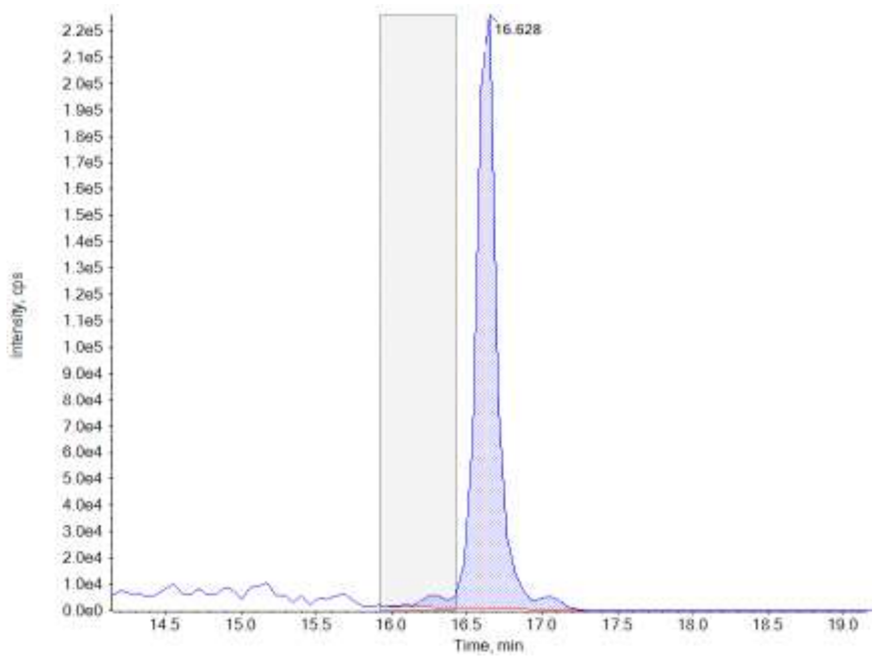

Acquired / Library MSMS

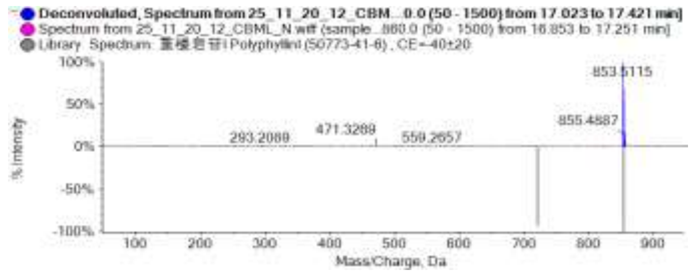

Acquired / Theoretical MS

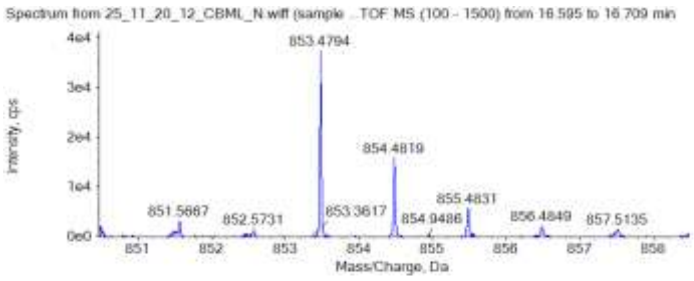

847.4942 / 16.99 (Mass/FragMass/RT/Isotope/Library/Formula/Ion Ratio)

Retention Time: 16.97 minutes **Exp RT:** 16.99 minutes
Precursor m/z : 847.4942 **Analyte Name:**
Fit (%) 49.0% **RFit (%)** 96.1% 847.4942 / 16.99

Collision Energy = 35 ± 15 eV

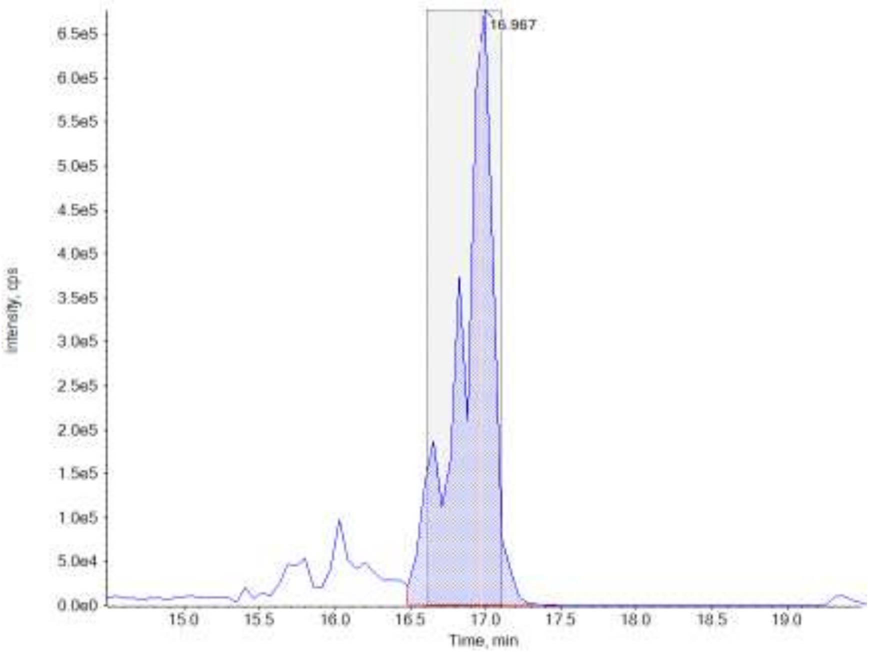

Acquired / Library MSMS

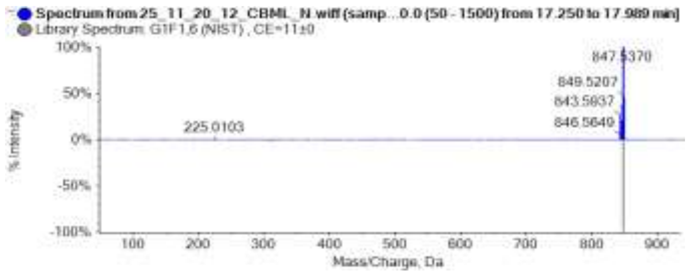

Acquired / Theoretical MS

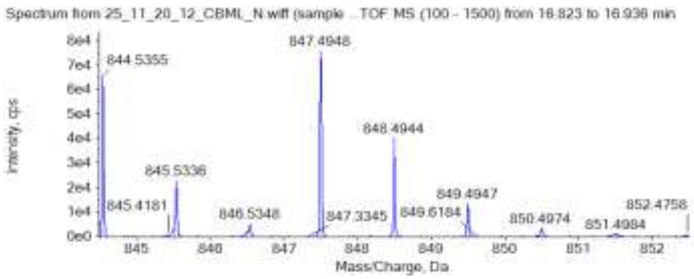

831.5051 / 17.16

(Mass/FragMass/RT/Isotope/Library/Formula/Ion Ratio)

**Retention Time:** 17.61 minutes<br>**Precursor m/z :** 831.5051<br>**Fit (%)** 95.7% **RFit (%)** 95.7%	**Exp RT:** 17.16 minutes<br>**Analyte Name:**<br>831.5051 / 17.16

Collision Energy = 35 ± 15 eV

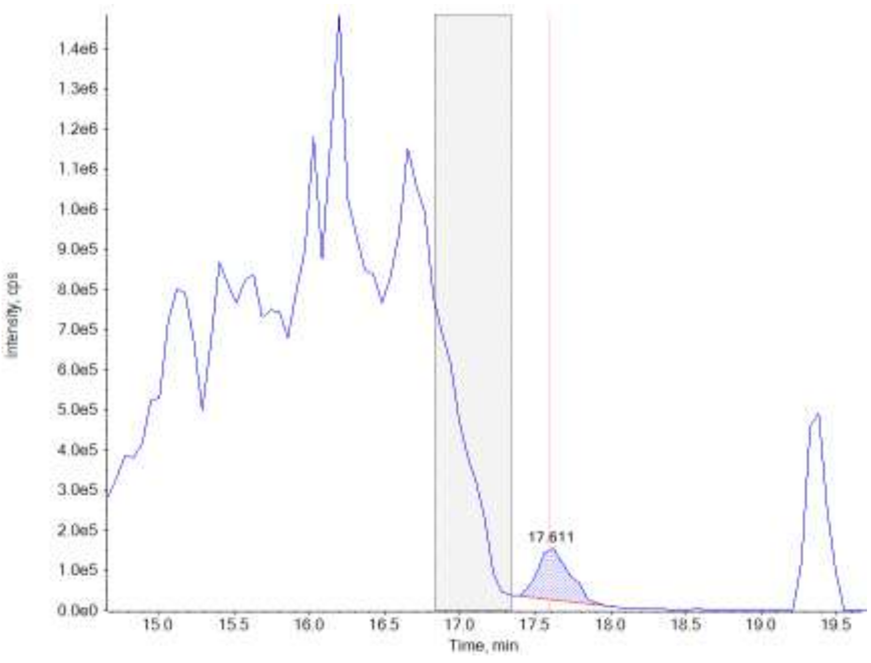

Acquired / Library MSMS

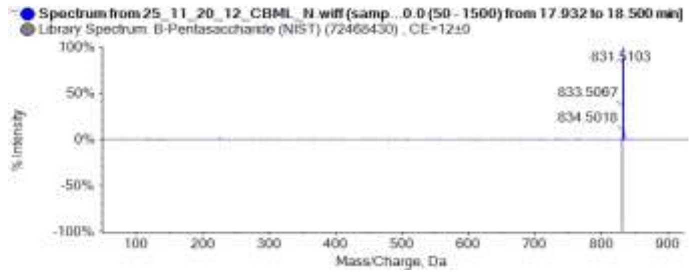

Acquired / Theoretical MS

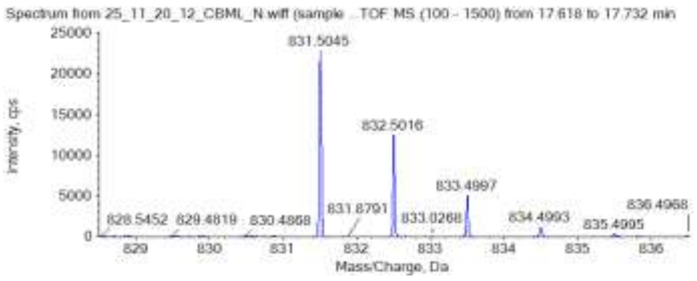

289.1796 / 17.22 (Mass/FragMass/RT/Isotope/Library/Formula/Ion Ratio)

Retention Time: 17.25 minutes
Precursor m/z : 289.1796
Fit (%) 93.9% **RFit (%)** 50.1%

Exp RT: 17.22 minutes
Analyte Name:
289.1796 / 17.22

Collision Energy = 35 ± 15 eV

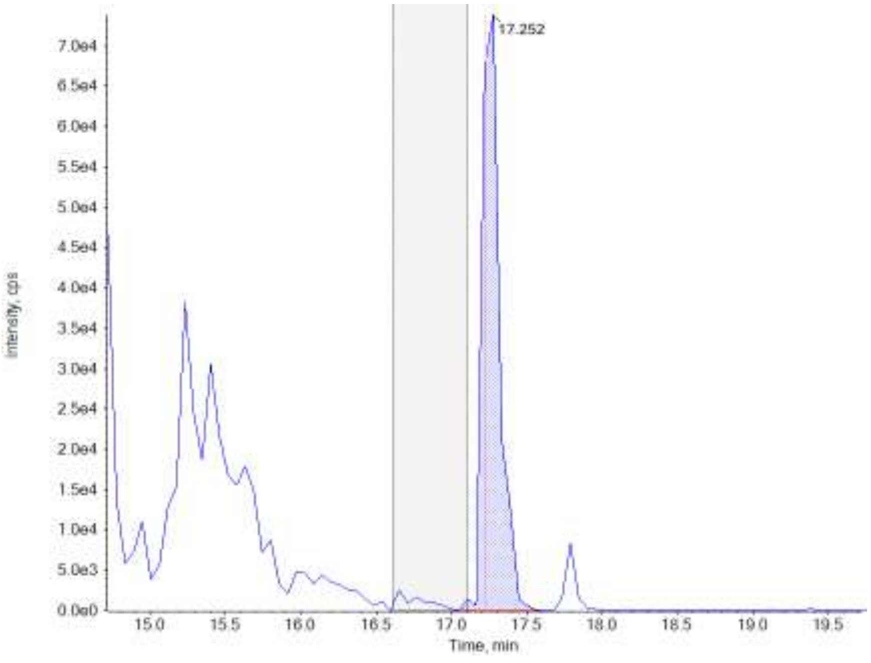

Acquired / Library MSMS

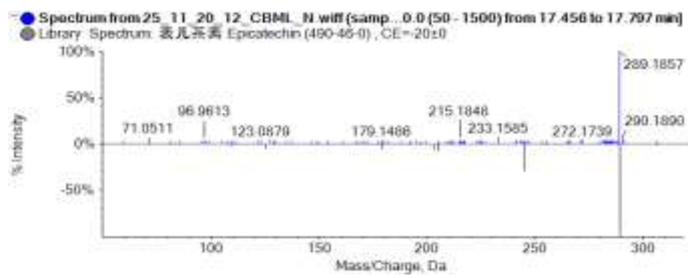

Acquired / Theoretical MS

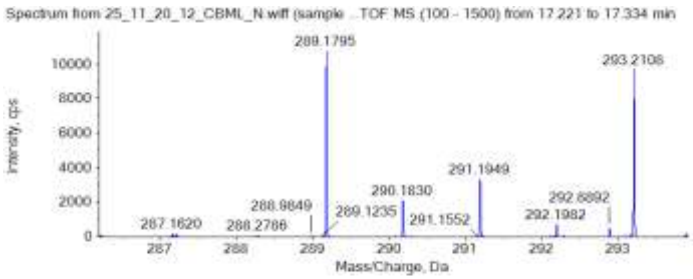

Created with SCIEX OS 3.3.0
Printed:12/9/2025 2:25:51 PM

269.2123 / 17.28 [M-H]- (Mass/FragMass/RT/Isotope/Library/Formula/Ion Ratio)

Retention Time: 17.27 minutes
Precursor m/z : 269.2123
Fit (%) 79.7% **RFit (%)** 100.0%

Exp RT: 17.28 minutes
Analyte Name:
269.2123 / 17.28 [M-H]-

Collision Energy = 35 ± 15 eV

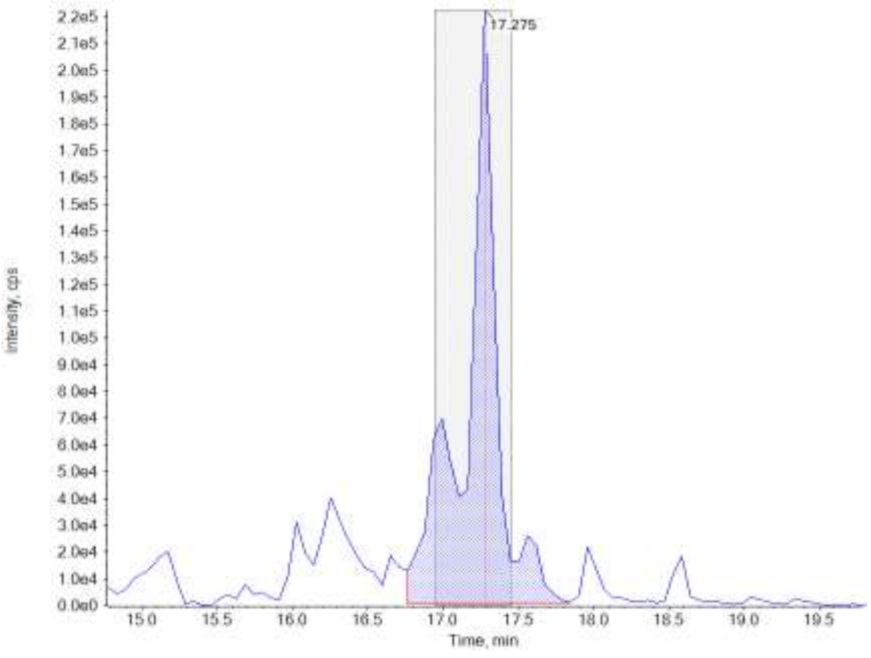

Acquired / Library MSMS

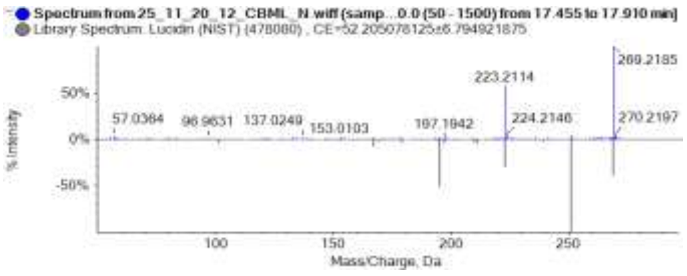

Acquired / Theoretical MS

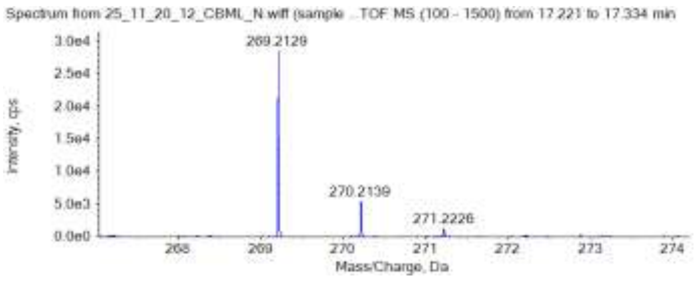

Created with SCIEX OS 3.3.0
Printed:12/9/2025 2:25:51 PM

365.2708 / 17.28 (Mass/FragMass/RT/Isotope/Library/Formula/Ion Ratio)

**Retention Time:** 17.28 minutes	**Exp RT:** 17.28 minutes
**Precursor m/z :** 365.2708	**Analyte Name:**
**Fit (%)** 100.0% **RFit (%)** 88.9%	365.2708 / 17.28

Collision Energy = 35 ± 15 eV

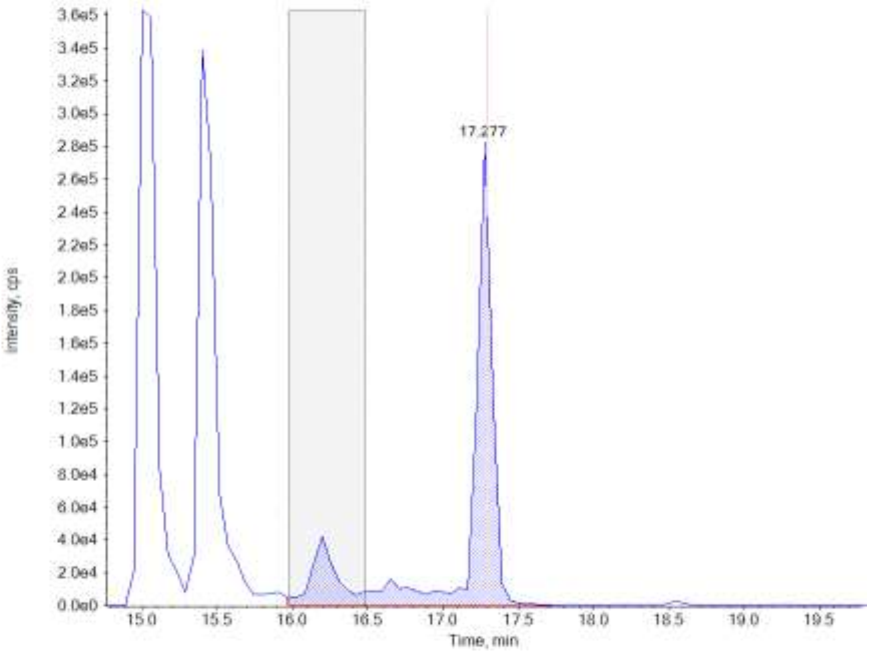

Acquired / Library MSMS

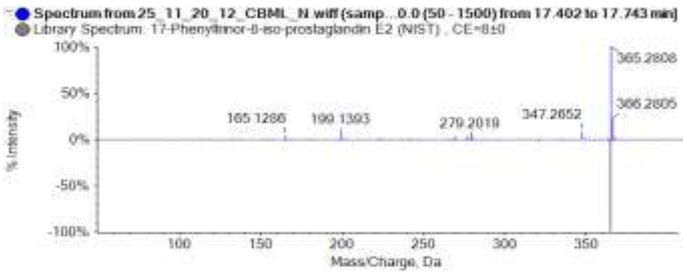

Acquired / Theoretical MS

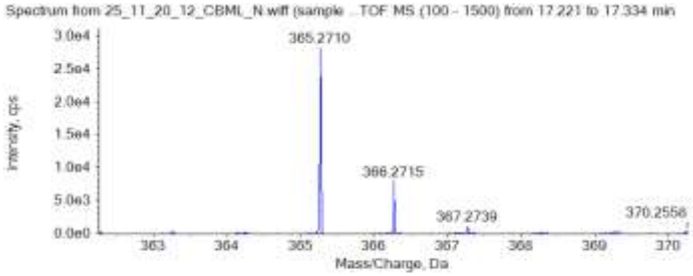

263.1999 / 17.50

(Mass/FragMass/RT/Isotope/Library/Formula/Ion Ratio)

Retention Time: 17.49 minutes
Precursor m/z : 263.1999
Fit (%) 75.2% **RFit (%)** 95.5%

Exp RT: 17.51 minutes
Analyte Name:
263.1999 / 17.50

Collision Energy = 35 ± 15 eV

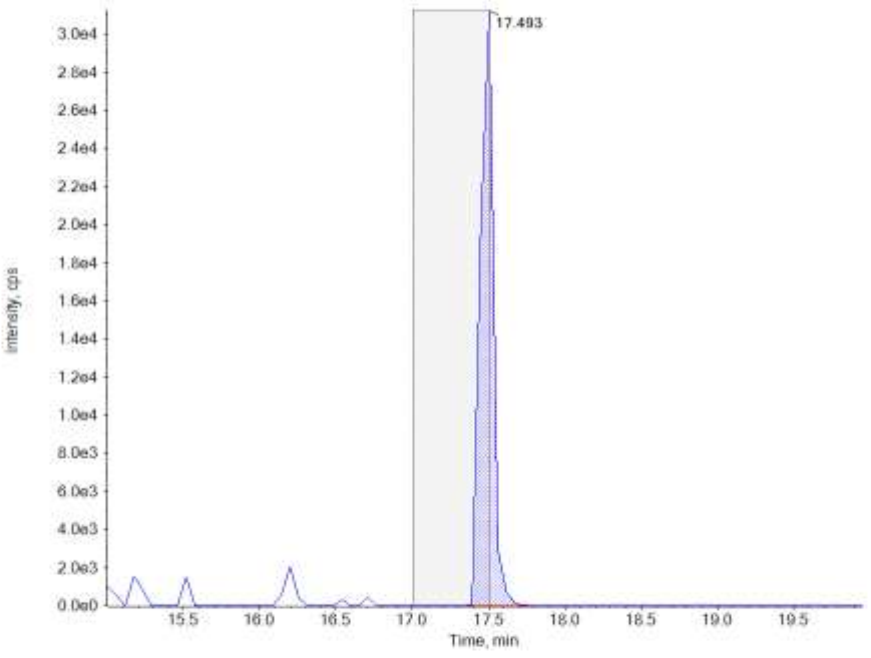

Acquired / Library MSMS

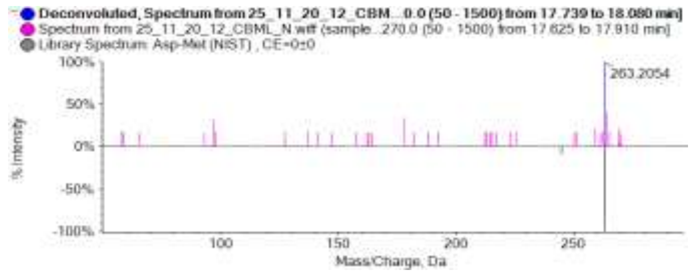

Acquired / Theoretical MS

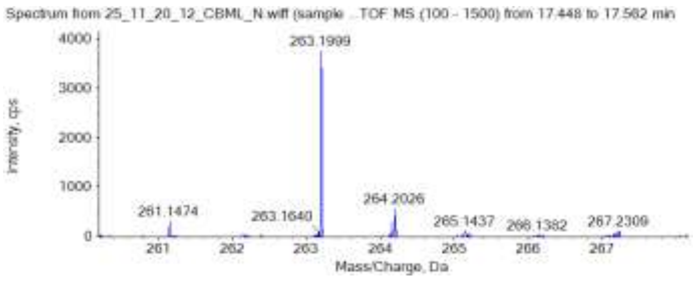

277.2273 / 17.79 (Mass/FragMass/RT/Isotope/Library/Formula/Ion Ratio)

**Retention Time:** 17.79 minutes	**Exp RT:** 17.79 minutes
**Precursor m/z :** 277.2273	**Analyte Name:**
**Fit (%)** 97.1% **RFit (%)** 100.0%	277.2273 / 17.79

Collision Energy = 35 ± 15 eV

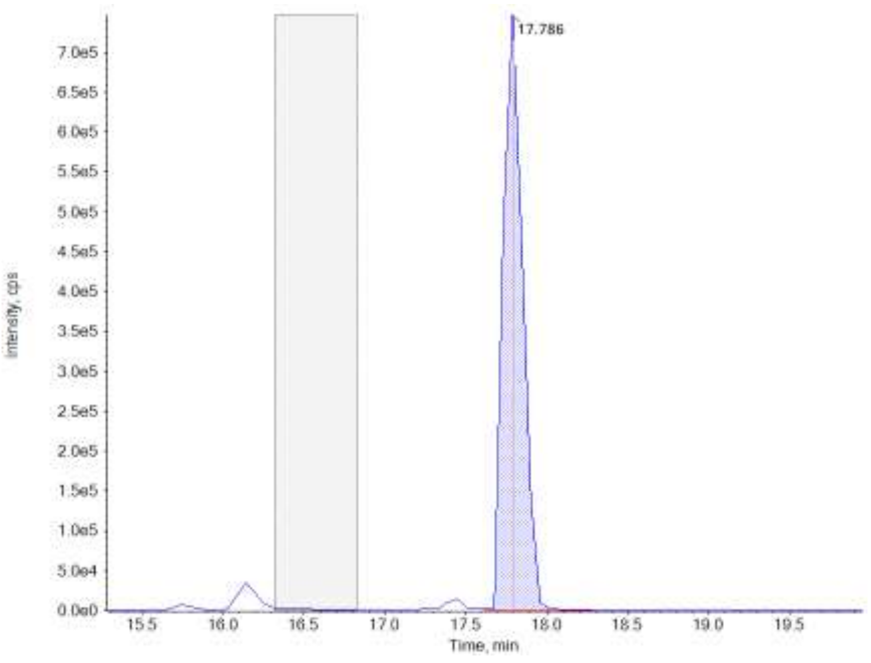

Acquired / Library MSMS

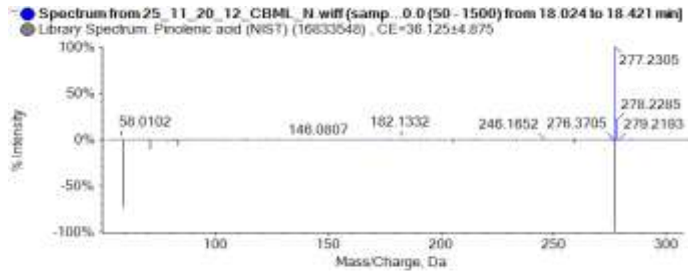

Acquired / Theoretical MS

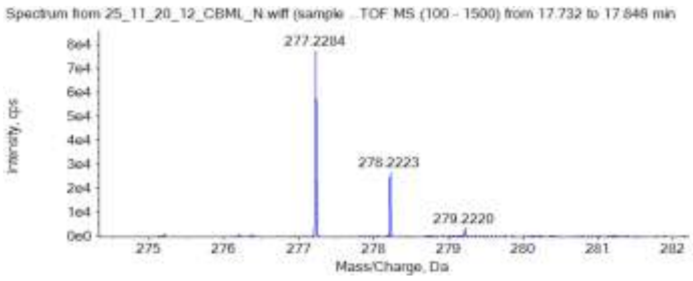

271.2357 / 18.02 (Mass/FragMass/RT/Isotope/Library/Formula/Ion Ratio)

**Retention Time:** 18.01 minutes<br>**Precursor m/z :** 271.2357<br>**Fit (%)** 85.7% **RFit (%)** 100.0%	**Exp RT:** 18.02 minutes<br>**Analyte Name:**<br>271.2357 / 18.02

Collision Energy = 35 ± 15 eV

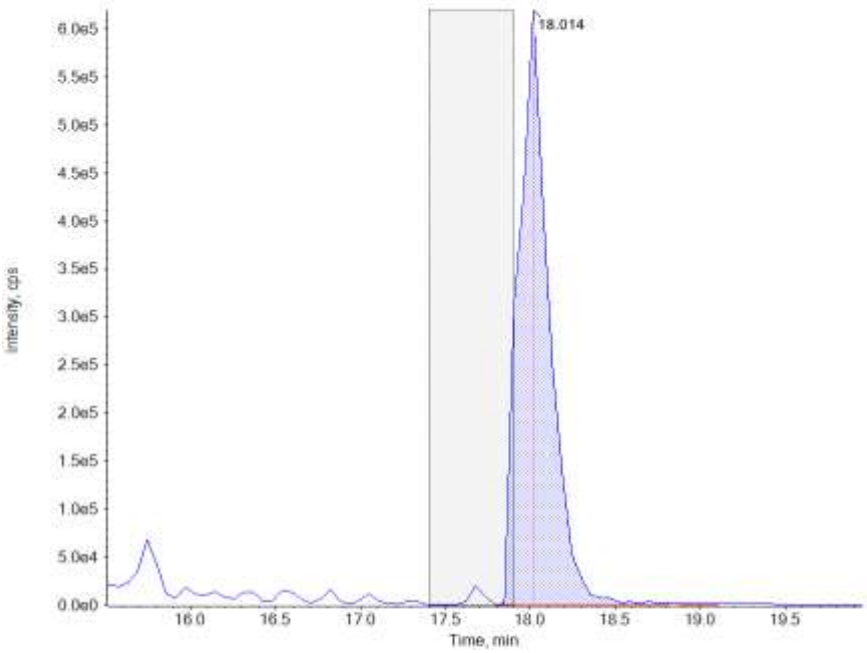

Acquired / Library MSMS

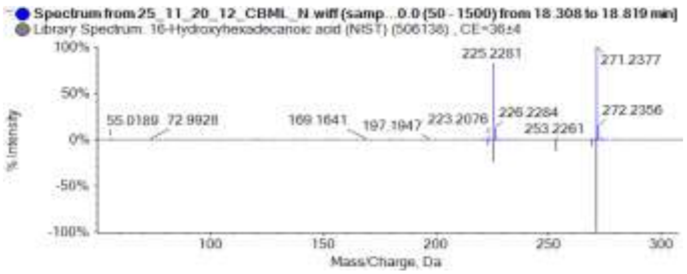

Acquired / Theoretical MS

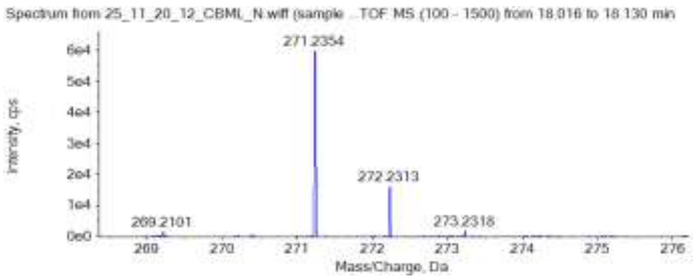

253.2175 / 18.13 (Mass/FragMass/RT/Isotope/Library/Formula/Ion Ratio)

**Retention Time:** 18.14 minutes	**Exp RT:** 18.13 minutes
**Precursor m/z :** 253.2175	**Analyte Name:**
**Fit (%)** 97.3% **RFit (%)** 100.0%	253.2175 / 18.13

Collision Energy = 35 ± 15 eV

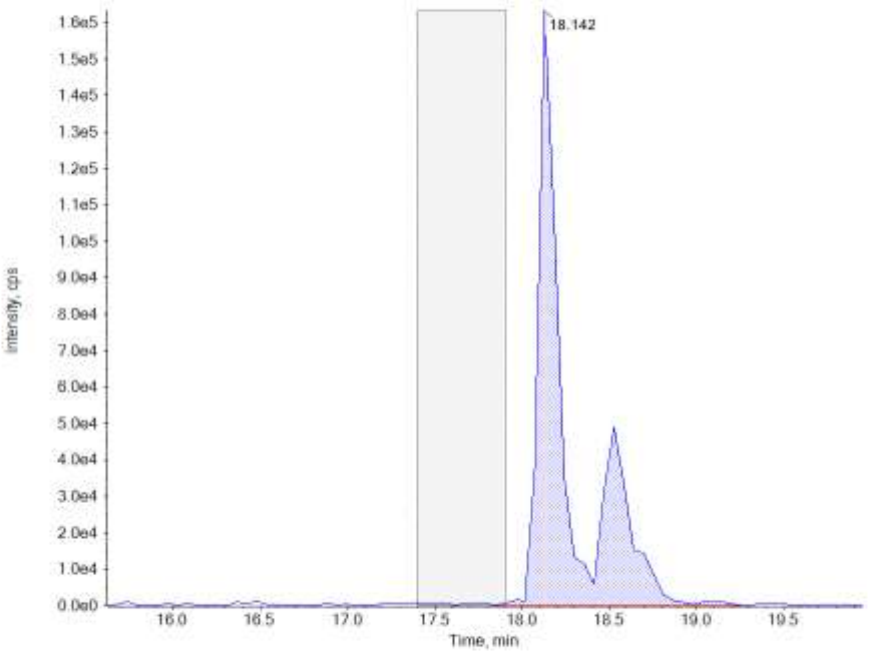

Acquired / Library MSMS

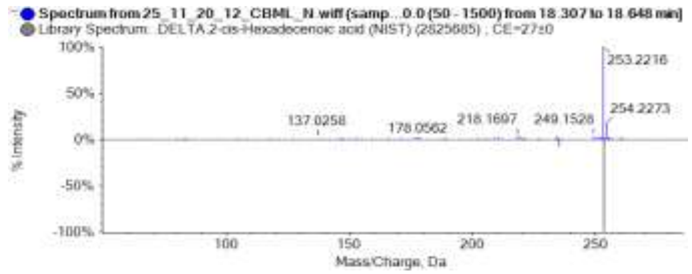

Acquired / Theoretical MS

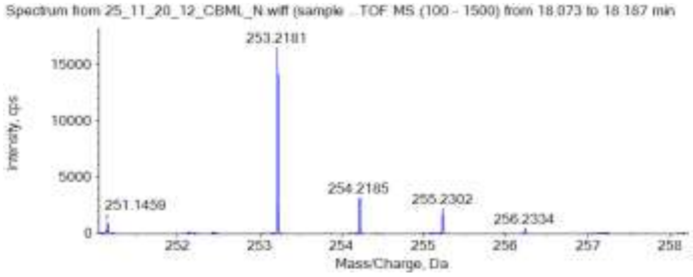

297.2422 / 18.13

(Mass/FragMass/RT/Isotope/Library/Formula/Ion Ratio)

Retention Time: 18.11 minutes
Precursor m/z : 297.2422
Fit (%) 98.7% **RFit (%)** 83.8%

Exp RT: 18.13 minutes
Analyte Name:
297.2422 / 18.13

Collision Energy = 35 ± 15 eV

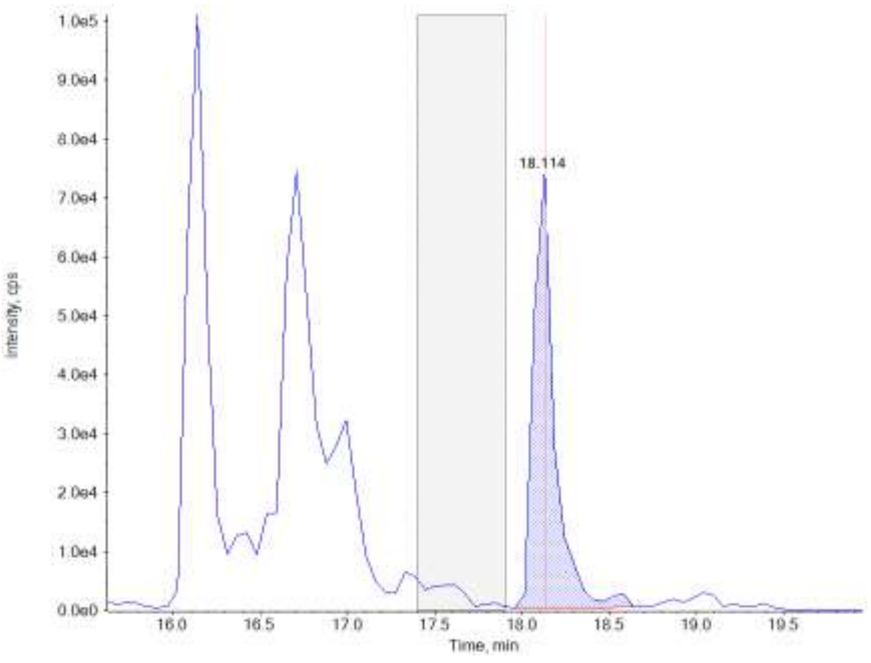

Acquired / Library MSMS

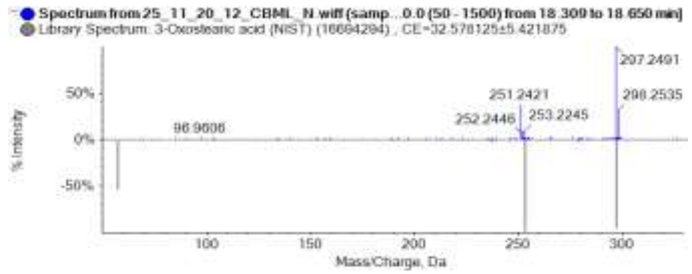

Acquired / Theoretical MS

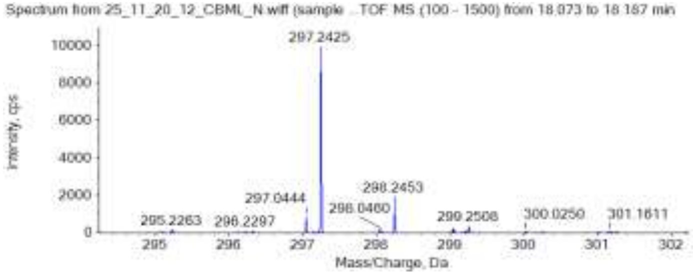

279.2338 / 18.30

(Mass/FragMass/RT/Isotope/Library/Formula/Ion Ratio)

Retention Time: 18.29 minutes
Precursor m/z : 279.2338
Fit (%) 100.0% **RFit (%)** 100.0%

Exp RT: 18.30 minutes
Analyte Name:
279.2338 / 18.30

Collision Energy = 35 ± 15 eV

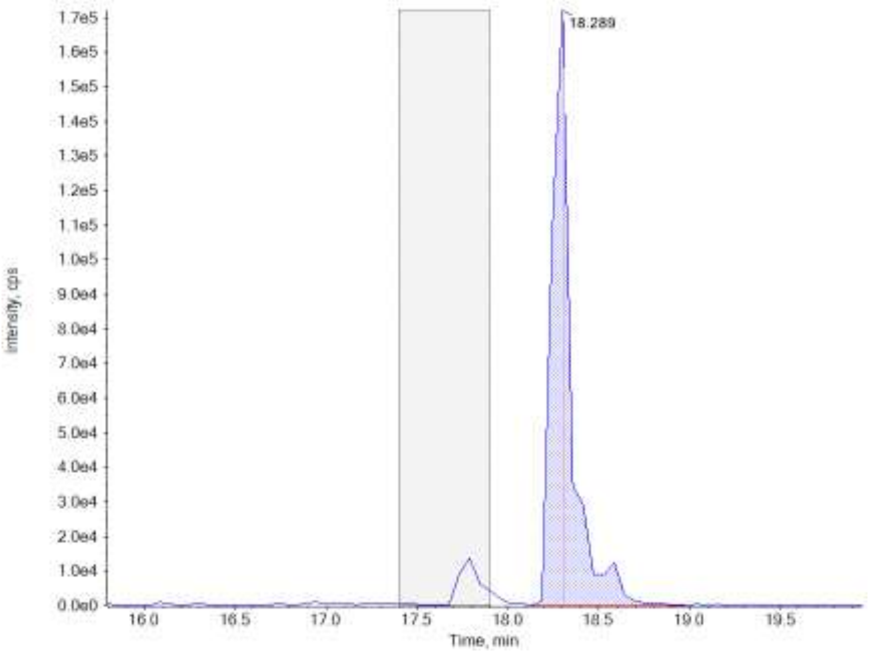

Acquired / Library MSMS

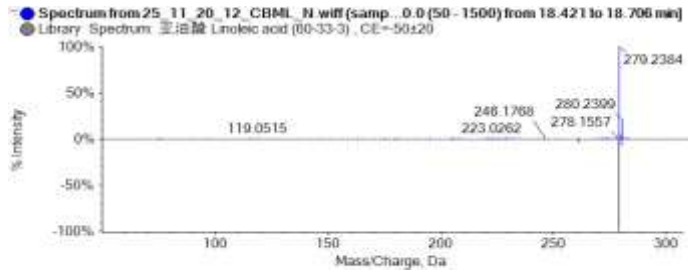

Acquired / Theoretical MS

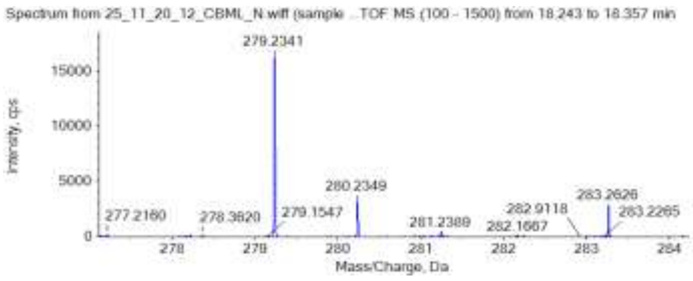

255.2415 / 18.58

(Mass/FragMass/RT/Isotope/Library/Formula/Ion Ratio)

Retention Time: 18.57 minutes
Precursor m/z : 255.2415
Fit (%) 71.7% **RFit (%)** 99.5%

Exp RT: 18.58 minutes
Analyte Name:
255.2415 / 18.58

Collision Energy = 35 ± 15 eV

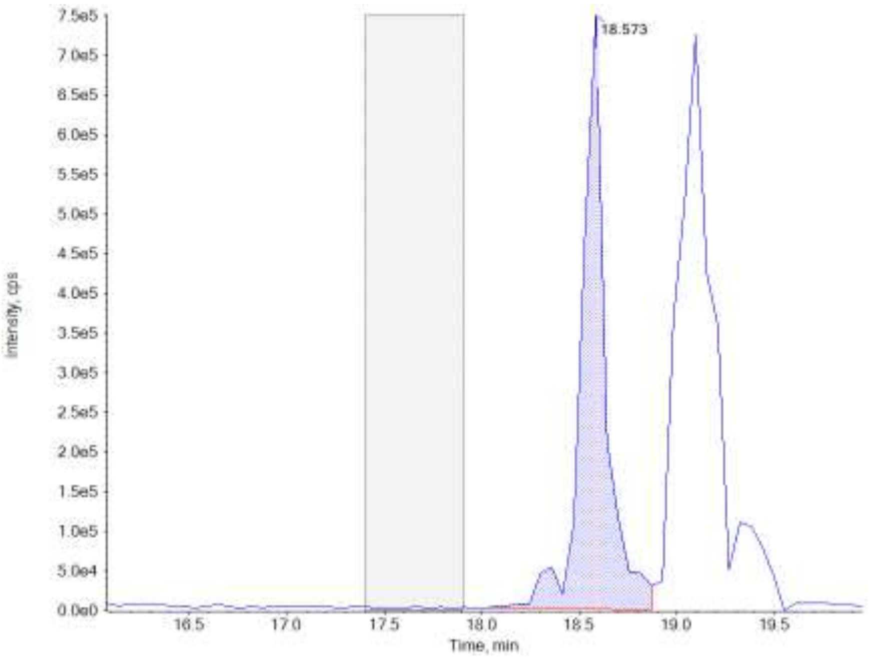

Acquired / Library MSMS

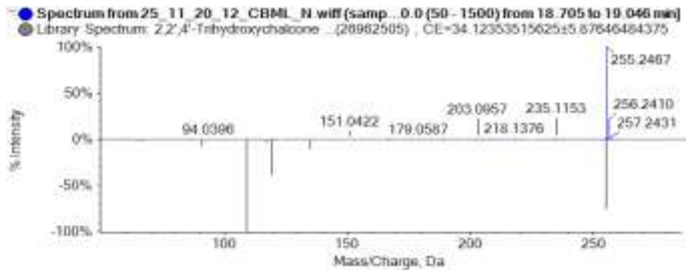

Acquired / Theoretical MS

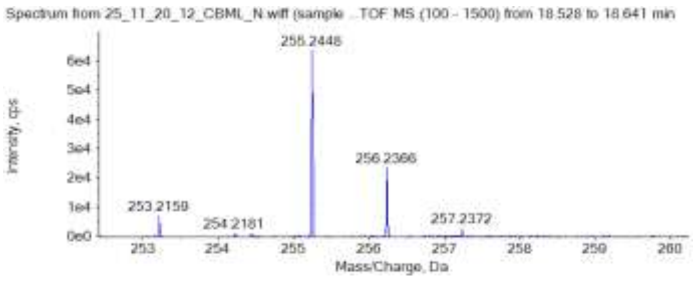

Created with SCIEX OS 3.3.0
Printed:12/9/2025 2:25:51 PM

402.2987 / 18.70 (Mass/FragMass/RT/Isotope/Library/Formula/Ion Ratio)

Retention Time: 18.67 minutes
Precursor m/z : 402.2987
Fit (%) 73.2% **RFit (%)** 68.6%

Exp RT: 18.70 minutes
Analyte Name:
402.2987 / 18.70

Collision Energy = 35 ± 15 eV

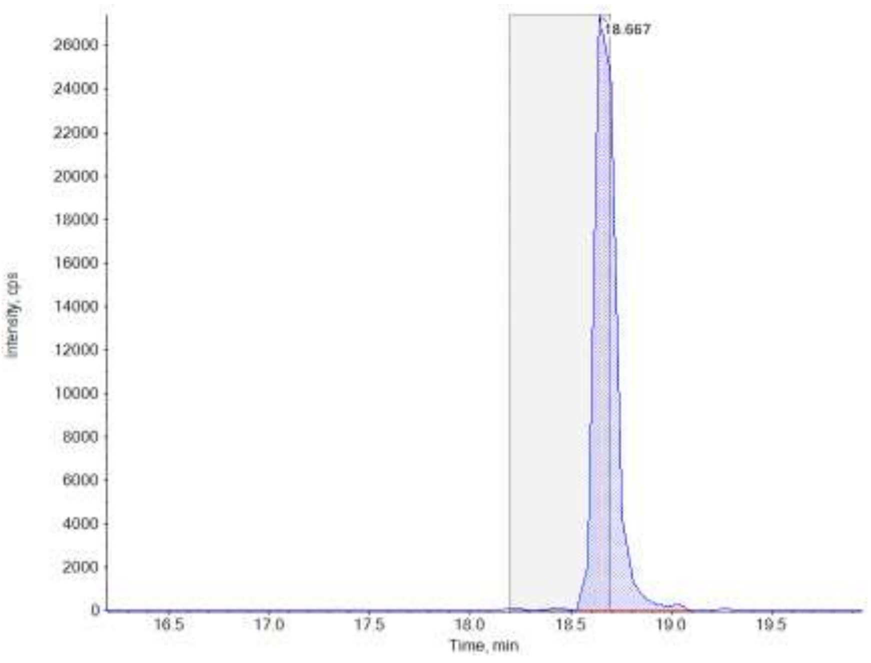

Acquired / Library MSMS

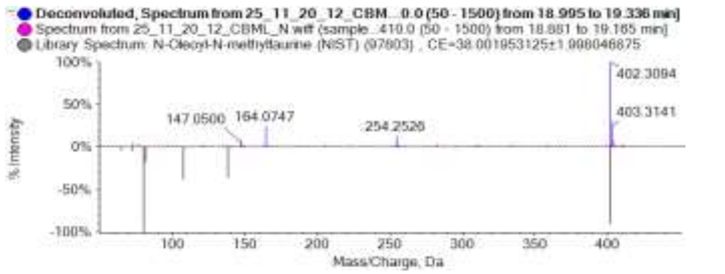

Acquired / Theoretical MS

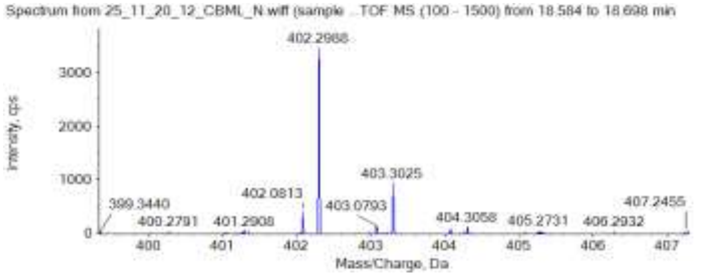

Created with SCIEX OS 3.3.0
Printed:12/9/2025 2:25:51 PM

311.2930 / 18.81 (Mass/FragMass/RT/Isotope/Library/Formula/Ion Ratio)

**Retention Time:** 18.83 minutes	**Exp RT:** 18.81 minutes
**Precursor m/z :** 311.2930	**Analyte Name:**
**Fit (%)** 78.2% **RFit (%)** 96.8%	311.2930 / 18.81

Collision Energy = 35 ± 15 eV

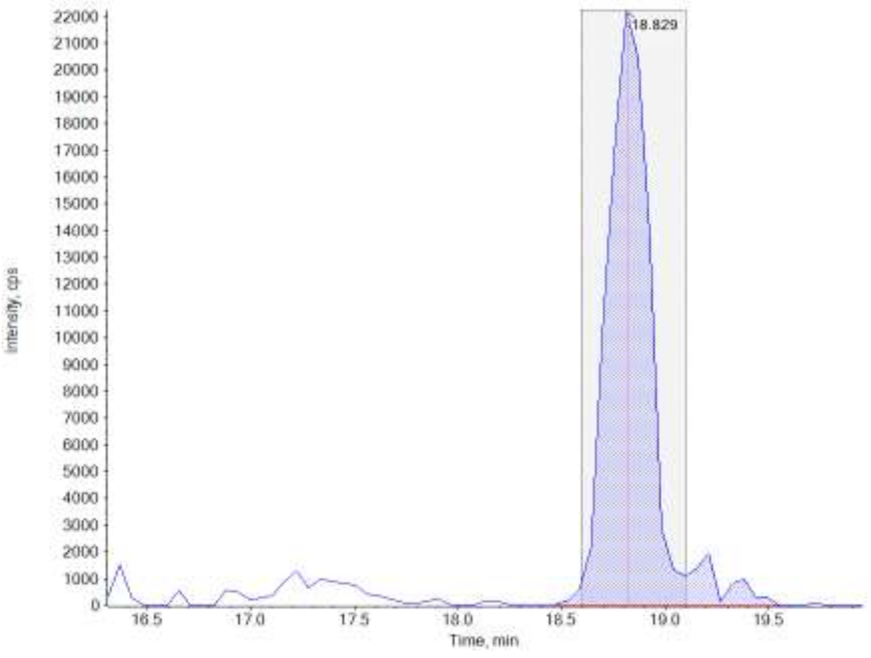

Acquired / Library MSMS

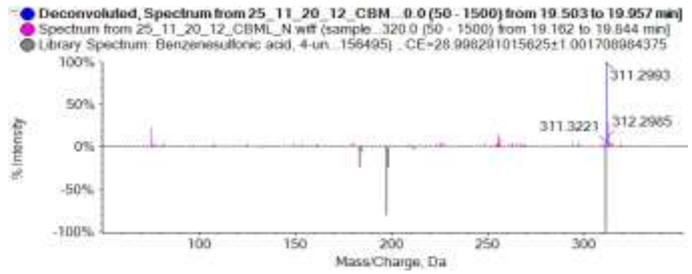

Acquired / Theoretical MS

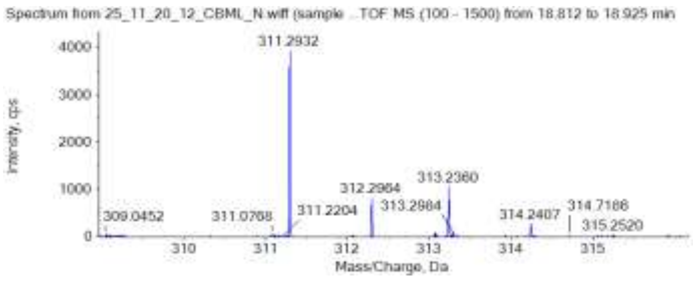

339.3261 / 18.93 (Mass/FragMass/RT/Isotope/Library/Formula/Ion Ratio)

Retention Time: 18.94 minutes
Precursor m/z : 339.3262
Fit (%) 85.3% **RFit (%)** 100.0%

Exp RT: 18.93 minutes
Analyte Name:
339.3261 / 18.93

Collision Energy = 35 ± 15 eV

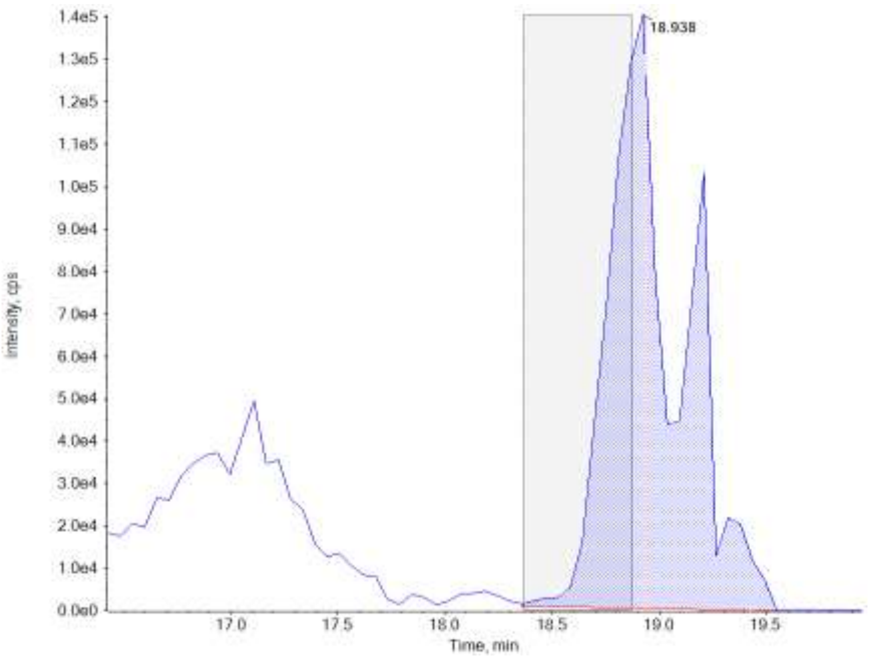

Acquired / Library MSMS

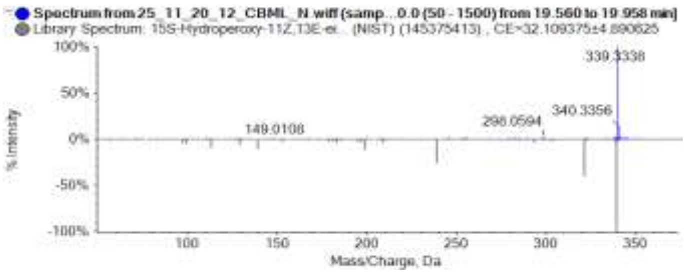

Acquired / Theoretical MS

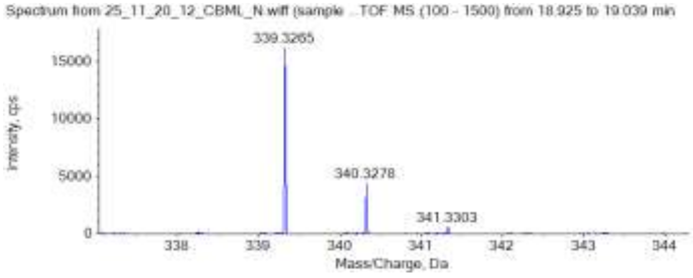

255.2392 / 19.10 (Mass/FragMass/RT/Isotope/Library/Formula/Ion Ratio)

**Retention Time:** 19.09 minutes	**Exp RT:** 19.10 minutes
**Precursor m/z :** 255.2392	**Analyte Name:**
**Fit (%)** 72.8% **RFit (%)** 99.4%	255.2392 / 19.10

Collision Energy = 35 ± 15 eV

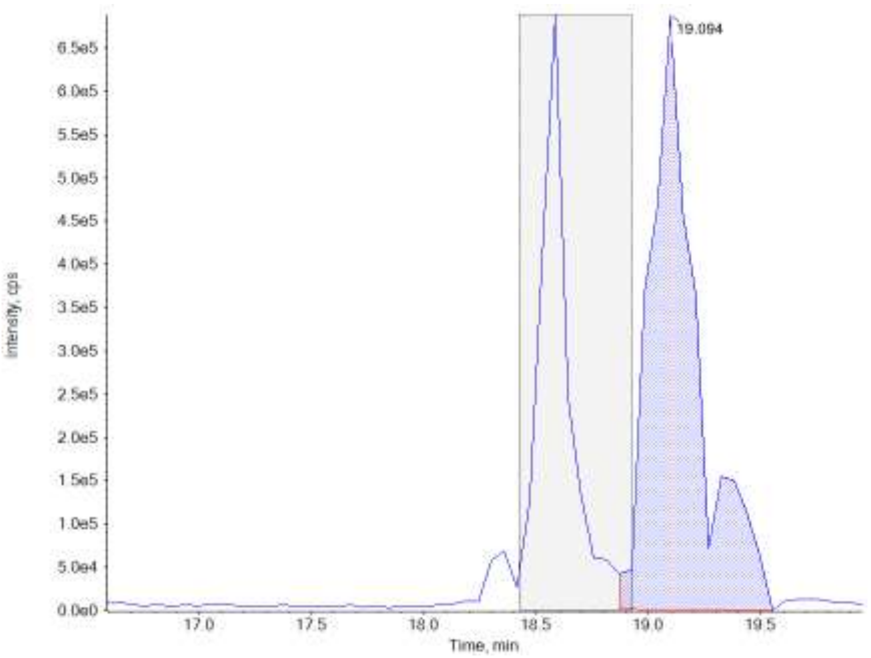

Acquired / Library MSMS

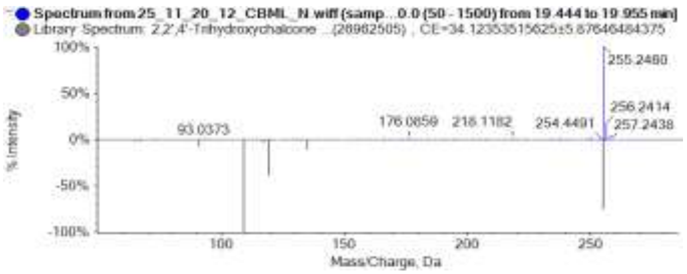

Acquired / Theoretical MS

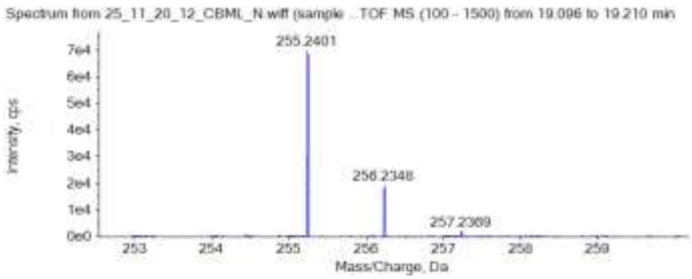

281.2476 / 19.15 [M-H2O-H]- (Mass/FragMass/RT/Isotope/Library/Formula/Ion Ratio)

**Retention Time:** 19.13 minutes	**Exp RT:** 19.15 minutes
**Precursor m/z :** 281.2476	**Analyte Name:**
**Fit (%)** 98.7% **RFit (%)** 76.9%	281.2476 / 19.15 [M-H2O-H]-

Collision Energy = 35 ± 15 eV

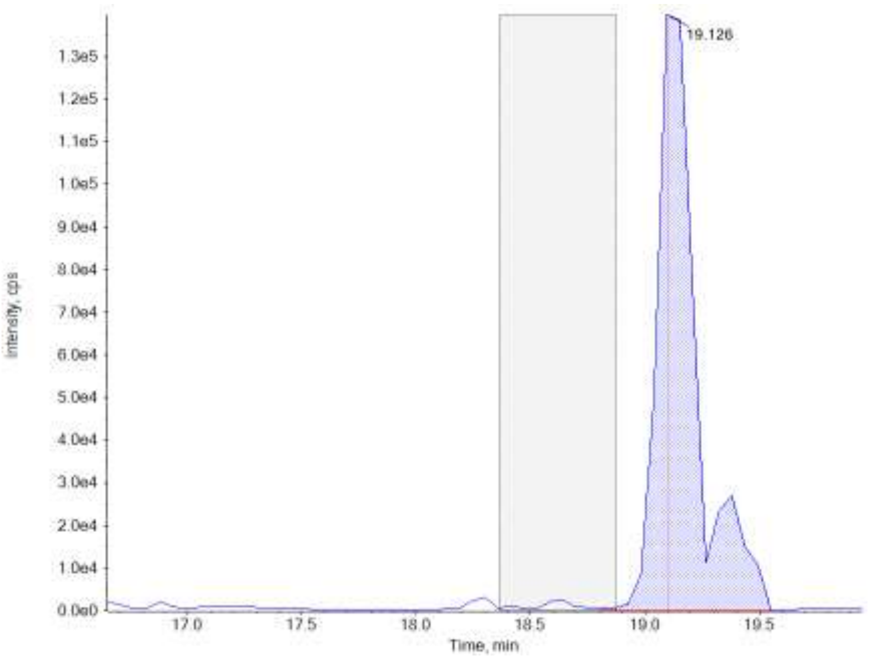

Acquired / Library MSMS

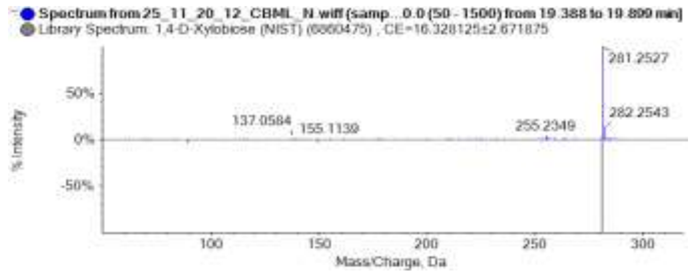

Acquired / Theoretical MS

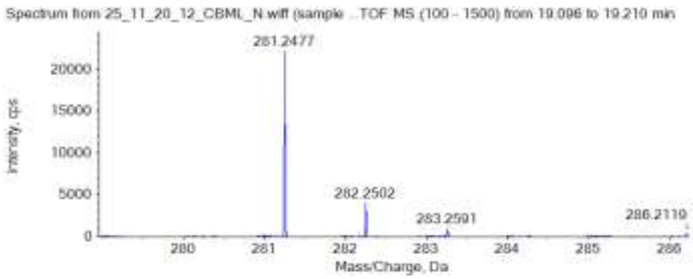

355.3208 / 19.21 (Mass/FragMass/RT/Isotope/Library/Formula/Ion Ratio) ✓

**Retention Time:** 19.21 minutes	**Exp RT:** 19.21 minutes
**Precursor m/z :** 355.3208	**Analyte Name:**
**Fit (%)** 82.8% **RFit (%)** 99.4%	355.3208 / 19.21

Collision Energy = 35 ± 15 eV

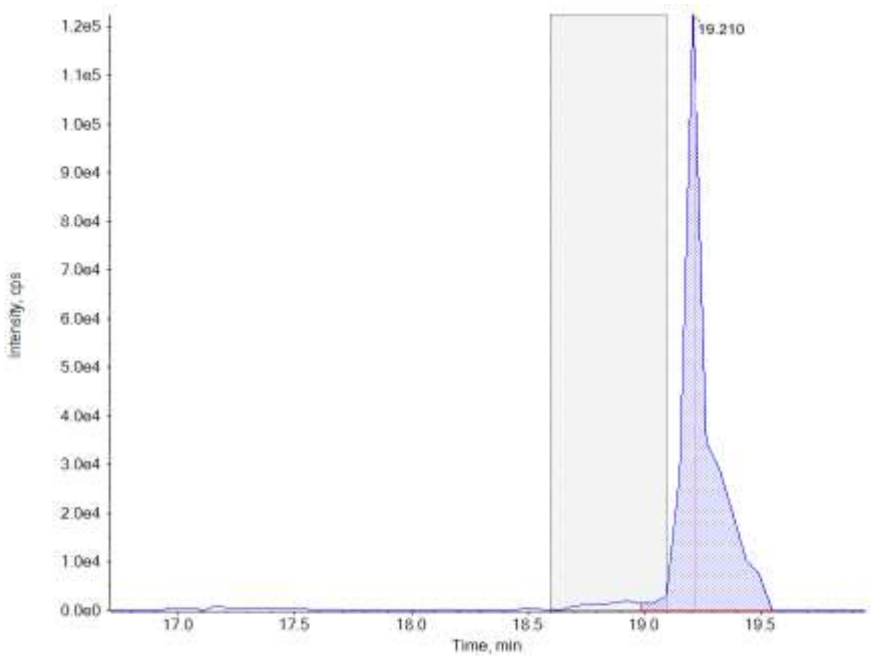

Acquired / Library MSMS

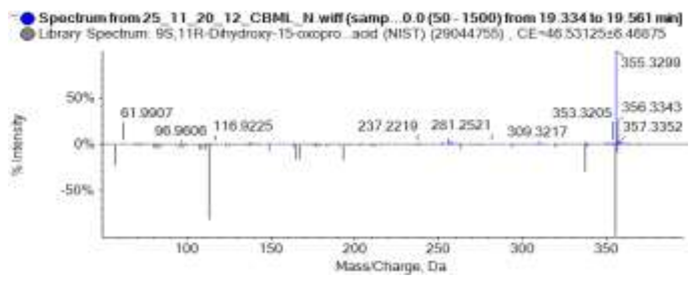

Acquired / Theoretical MS

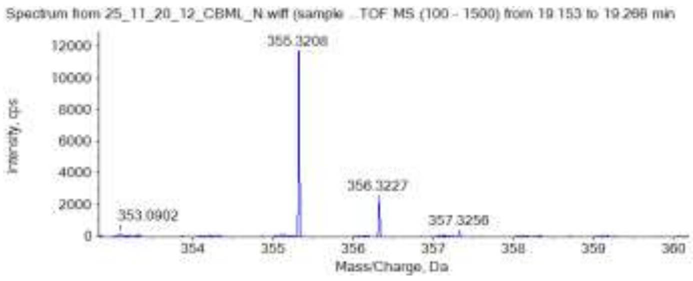

765.5134 / 19.27 (Mass/FragMass/RT/Isotope/Library/Formula/Ion Ratio)

Retention Time: 19.24 minutes
Precursor m/z : 765.5134
Fit (%) 87.8% **RFit (%)** 99.8%

Exp RT: 19.27 minutes
Analyte Name:
765.5134 / 19.27

Collision Energy = 35 ± 15 eV

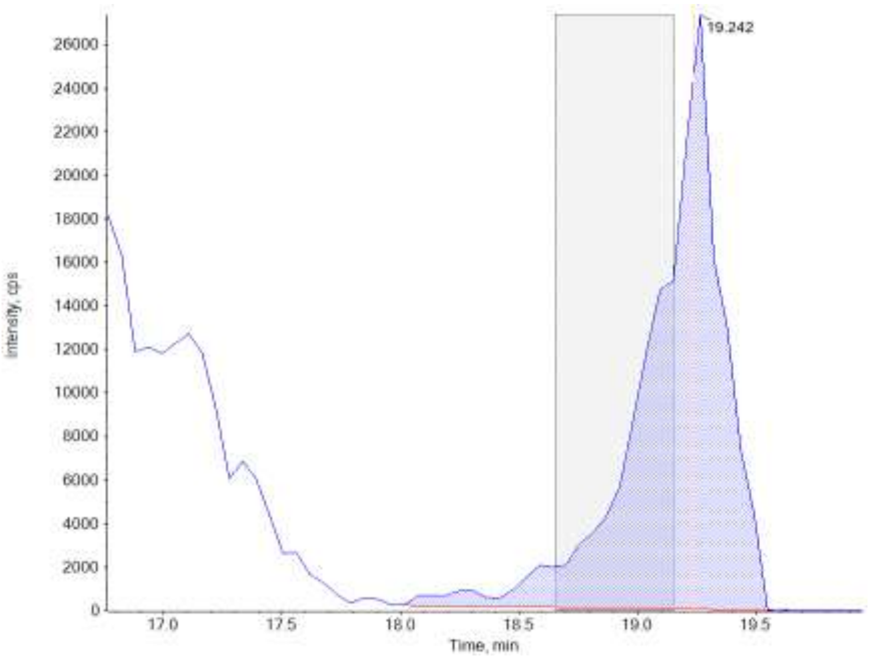

Acquired / Library MSMS

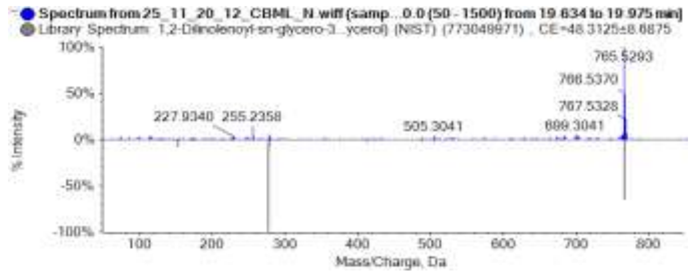

Acquired / Theoretical MS

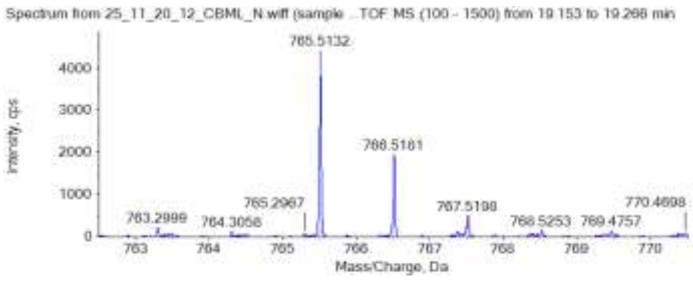

Created with SCIEX OS 3.3.0
Printed:12/9/2025 2:25:51 PM

831.5075 / 19.32 (Mass/FragMass/RT/Isotope/Library/Formula/Ion Ratio)

**Retention Time:** 19.35 minutes	**Exp RT:** 19.32 minutes
**Precursor m/z :** 831.5075	**Analyte Name:**
**Fit (%)** 92.0% **RFit (%)** 36.0%	831.5075 / 19.32

Collision Energy = 35 ± 15 eV

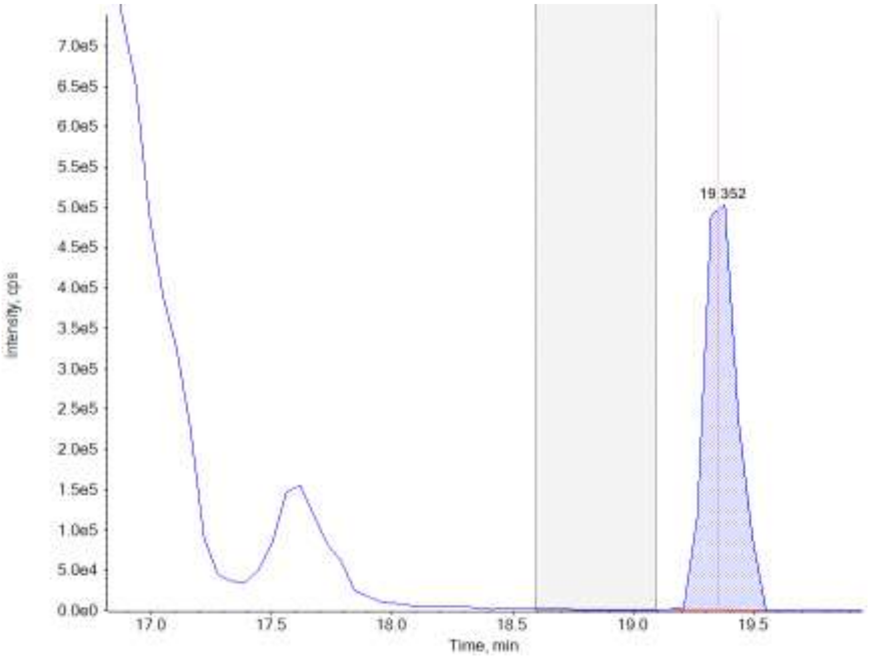

Acquired / Library MSMS

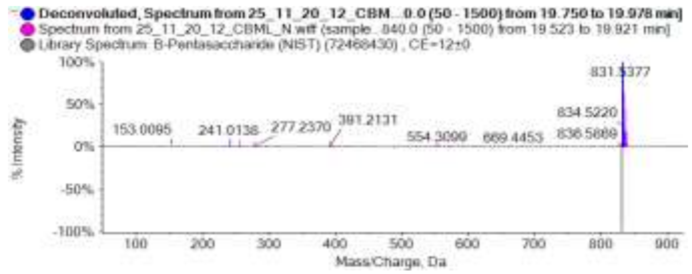

Acquired / Theoretical MS

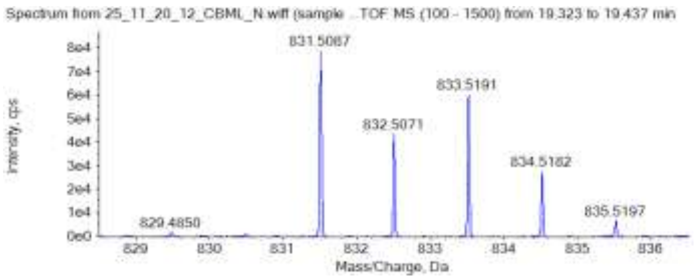

Supplement: Supplementary file 1 [file ijms-27-04945-s001.zip › 2.IJMS-4262115 Supplementary Data BMLE-negative mode.pdf]
