# Supplementary material for: Box–Behnken Design Optimization of High-Pressure Processed Bitter Melon (Momordica charantia) Leaf Extract Enhancing Phytochemicals, Anticancer, and Anti-Inflammatory Activities
Source: Int J Mol Sci. 2026 May 29;27(11):4945. doi: 10.3390/ijms27114945 (PMC13256669; doi:10.3390/ijms27114945)

(BMLE-positive mode)

25_12_08_04_CBMLP.wiff - CBML_P

**Data File**	25_12_08_04_CBMLP.wiff	**Result Table**	CBML-P_CE40
**Acquisition Date**	2025-12-08T13:05:37	**Algorithm Used**	MQ4
**Acquisition Method**	Pos_DIA_20min_251218.dam	**Instrument Name**	TripleTOF 6600
**Project**	Untargeted	**Processing Method**	

Extracted Ion Chromatogram

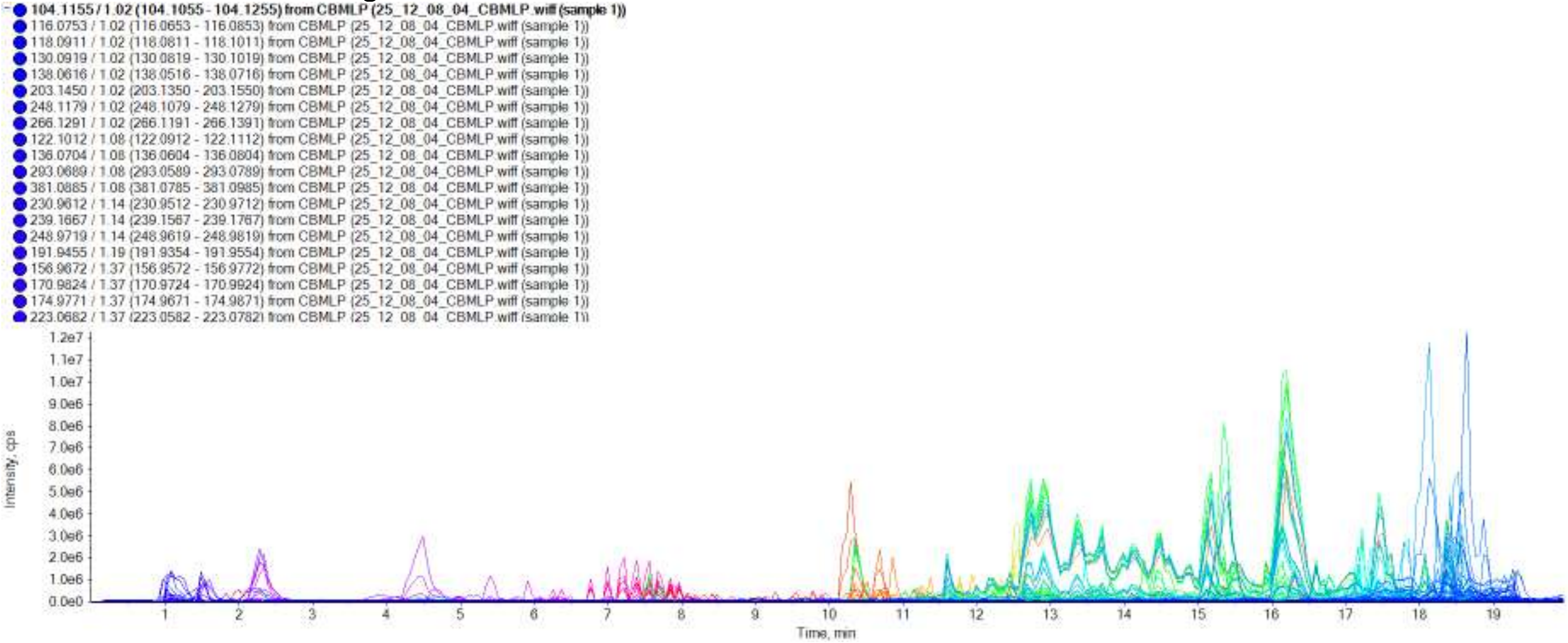

Summary

#	Analyte Peak Name	Mass Error Confidence	Fragment Mass Error Confidence	RT Confidence	Isotope Confidence	Library Confidence	Formula Confidence	Ion Ratio Confidence	Sample Name
1	104.1155 / 1.02	●	●	●	●	✔	●	●	CBML_P
2	116.0753 / 1.02	●	●	●	●	🔴	●	●	CBML_P
3	118.0911 / 1.02	●	●	●	●	▲	●	●	CBML_P
7	248.1179 / 1.02	●	●	●	●	🔴	●	●	CBML_P
10	136.0704 / 1.08	●	●	●	●	▲	●	●	CBML_P
12	381.0885 / 1.08	●	●	●	●	✔	●	●	CBML_P
23	337.0947 / 1.37	●	●	●	●	✔	●	●	CBML_P
28	152.0648 / 1.48	●	●	●	●	✔	●	●	CBML_P
29	182.0868 / 1.48	●	●	●	●	✔	●	●	CBML_P
30	284.1075 / 1.48	●	●	●	●	✔	●	●	CBML_P
32	276.1521 / 1.54	●	●	●	●	▲	●	●	CBML_P
33	132.1101 / 1.59	●	●	●	●	✔	●	●	CBML_P
37	324.1009 / 1.82 [M+K]+	●	●	●	●	✔	●	●	CBML_P
38	282.1271 / 1.82	●	●	●	●	✔	●	●	CBML_P
42	182.0974 / 1.93 [M+H]+	●	●	●	●	✔	●	●	CBML_P
43	204.1281 / 1.93	●	●	●	●	✔	●	●	CBML_P
50	121.0900 / 2.27	●	●	●	●	✔	●	●	CBML_P
51	166.0972 / 2.27	●	●	●	●	✔	●	●	CBML_P
52	328.1478 / 2.27	●	●	●	●	🔴	●	●	CBML_P
53	120.0928 / 2.33	●	●	●	●	▲	●	●	CBML_P

54	220.1231 / 2.44	●	●	●	●	✓	●	●	CBML_P
55	158.0863 / 2.62	●	●	●	●	🔴	●	●	CBML_P
58	186.1285 / 2.67	●	●	●	●	▲	●	●	CBML_P
59	384.1200 / 2.73	●	●	●	●	✓	●	●	CBML_P
64	156.9668 / 3.52	●	●	●	●	🔴	●	●	CBML_P
68	192.1069 / 4.04	●	●	●	●	✓	●	●	CBML_P
71	298.1027 / 4.26	●	●	●	●	✓	●	●	CBML_P
72	118.0697 / 4.43	●	●	●	●	✓	●	●	CBML_P
73	146.0653 / 4.43	●	●	●	●	▲	●	●	CBML_P
74	159.0964 / 4.43	●	●	●	●	✓	●	●	CBML_P
75	205.1058 / 4.43	●	●	●	●	✓	●	●	CBML_P
76	144.0854 / 4.49	●	●	●	●	▲	●	●	CBML_P
77	188.0849 / 4.49	●	●	●	●	✓	●	●	CBML_P
82	139.0448 / 5.00	●	●	●	●	✓	●	●	CBML_P
84	339.0548 / 5.00	●	●	●	●	✓	●	●	CBML_P
86	238.1124 / 5.23	●	●	●	●	🔴	●	●	CBML_P
88	120.0852 / 5.40	●	●	●	●	▲	●	●	CBML_P
90	295.1429 / 5.40	●	●	●	●	▲	●	●	CBML_P
94	174.1546 / 5.74	●	●	●	●	✓	●	●	CBML_P
95	217.1020 / 5.74	●	●	●	●	▲	●	●	CBML_P
101	166.0915 / 6.08	●	●	●	●	🔴	●	●	CBML_P
103	212.0974 / 6.08 [M+H]+	●	●	●	●	🔴	●	●	CBML_P
104	229.1238 / 6.08 [M+NH4]+	●	●	●	●	✓	●	●	CBML_P

114	377.1515 / 6.37								CBML_P
115	265.1488 / 6.42								CBML_P
118	209.1222 / 6.71								CBML_P
132	585.2943 / 7.22 [M+K]+								CBML_P
136	591.3660 / 7.39 [M+H]+								CBML_P
137	629.3216 / 7.39 [M+K]+								CBML_P
144	652.4275 / 7.56 [M+NH4]+								CBML_P
147	347.1287 / 7.56								CBML_P
148	673.3468 / 7.56 [M+K]+								CBML_P
149	635.3900 / 7.56 [M+H]+								CBML_P
165	740.4750 / 7.84								CBML_P
166	768.5018 / 7.84								CBML_P
171	784.4976 / 7.96								CBML_P
177	211.1742 / 8.07								CBML_P
179	414.7690 / 8.07 [M+H+NH4]2+								CBML_P
184	146.0652 / 8.13								CBML_P
198	383.1756 / 8.41								CBML_P
208	213.1540 / 8.70								CBML_P
217	464.2799 / 8.98								CBML_P
222	115.0807 / 9.04								CBML_P
223	309.2108 / 9.04								CBML_P
225	211.1384 / 9.15								CBML_P
231	446.2808 / 9.49								CBML_P

233	448.2860 / 9.55					✓			CBML_P
234	191.1478 / 9.66					▲			CBML_P
235	193.1635 / 9.66					✓			CBML_P
236	211.1746 / 9.66					▲			CBML_P
241	306.2698 / 10.12					●			CBML_P
245	275.2089 / 10.34					▲			CBML_P
246	293.2195 / 10.34					▲			CBML_P
247	311.2273 / 10.34					●			CBML_P
250	195.1429 / 10.40					●			CBML_P
254	325.2329 / 10.51					●			CBML_P
261	731.4532 / 10.74 [M+H]+					●			CBML_P
277	769.4763 / 11.20					✓			CBML_P
285	311.2275 / 11.31					●			CBML_P
290	181.1279 / 11.59					✓			CBML_P
291	391.3413 / 11.59					▲			CBML_P
292	409.3522 / 11.59					●			CBML_P
300	453.3405 / 11.71					▲			CBML_P
301	428.2707 / 11.76					✓			CBML_P
316	291.2022 / 12.22					▲			CBML_P
320	498.3979 / 12.33					✓			CBML_P
325	583.4145 / 12.45					✓			CBML_P
326	540.4124 / 12.50					✓			CBML_P
342	293.2168 / 12.84					▲			CBML_P

346	391.3418 / 12.90	●	●	●	●	🔴	●	●	CBML_P
349	419.3436 / 12.90	●	●	●	●	▲	●	●	CBML_P
362	257.1947 / 13.24	●	●	●	●	🔴	●	●	CBML_P
379	554.4241 / 13.70	●	●	●	●	🔴	●	●	CBML_P
383	275.2068 / 13.81	●	●	●	●	✔	●	●	CBML_P
391	304.2905 / 13.98	●	●	●	●	✔	●	●	CBML_P
396	668.4394 / 14.15	●	●	●	●	🔴	●	●	CBML_P
397	453.3409 / 14.21	●	●	●	●	✔	●	●	CBML_P
409	523.3455 / 14.61	●	●	●	●	🔴	●	●	CBML_P
410	496.3448 / 14.78	●	●	●	●	✔	●	●	CBML_P
411	275.2061 / 14.83	●	●	●	●	✔	●	●	CBML_P
412	293.2166 / 14.83	●	●	●	●	▲	●	●	CBML_P
419	309.2624 / 15.12	●	●	●	●	▲	●	●	CBML_P
420	391.3417 / 15.12	●	●	●	●	▲	●	●	CBML_P
423	419.3406 / 15.12	●	●	●	●	🔴	●	●	CBML_P
424	455.3583 / 15.12	●	●	●	●	▲	●	●	CBML_P
430	279.2279 / 15.29	●	●	●	●	✔	●	●	CBML_P
431	553.4366 / 15.29	●	●	●	●	🔴	●	●	CBML_P
432	277.2312 / 15.35	●	●	●	●	✔	●	●	CBML_P
437	277.2207 / 15.57	●	●	●	●	✔	●	●	CBML_P
439	181.1274 / 15.69	●	●	●	●	🔴	●	●	CBML_P
441	332.3214 / 15.69	●	●	●	●	✔	●	●	CBML_P
444	353.2752 / 15.74	●	●	●	●	🔴	●	●	CBML_P

450	275.2086 / 15.91					●			CBML_P
453	203.1847 / 16.14					●			CBML_P
454	337.2582 / 16.14					●			CBML_P
459	419.3425 / 16.14					●			CBML_P
461	455.3599 / 16.14					▲			CBML_P
468	423.3671 / 16.54					✓			CBML_P
475	277.2252 / 16.60					✓			CBML_P
476	295.2349 / 16.60					●			CBML_P
477	447.2544 / 16.60					✓			CBML_P
481	523.3496 / 16.77					●			CBML_P
483	423.3171 / 16.82					▲			CBML_P
488	355.2892 / 16.94					●			CBML_P
491	154.0545 / 16.99					●			CBML_P
500	599.4141 / 17.11					●			CBML_P
502	453.3430 / 17.16					▲			CBML_P
511	349.2821 / 17.33					▲			CBML_P
514	341.3105 / 17.39					✓			CBML_P
515	359.3216 / 17.39					✓			CBML_P
516	607.2953 / 17.39					▲			CBML_P
518	353.2761 / 17.45					✓			CBML_P
519	391.3408 / 17.45					▲			CBML_P
521	419.3375 / 17.45					●			CBML_P
523	459.3873 / 17.50					●			CBML_P

526	305.2556 / 17.56	●	●	●	●	✓	●	●	CBML_P
531	623.2548 / 17.68	●	●	●	●	●	●	●	CBML_P
534	421.3513 / 17.73	●	●	●	●	▲	●	●	CBML_P
538	149.0282 / 17.79	●	●	●	●	✓	●	●	CBML_P
541	279.2382 / 17.85	●	●	●	●	●	●	●	CBML_P
551	583.4191 / 18.19	●	●	●	●	✓	●	●	CBML_P
558	391.3414 / 18.36	●	●	●	●	✓	●	●	CBML_P
561	419.3390 / 18.36	●	●	●	●	●	●	●	CBML_P
565	609.2838 / 18.41	●	●	●	●	✓	●	●	CBML_P
568	439.3632 / 18.53	●	●	●	●	✓	●	●	CBML_P
573	313.2839 / 18.58	●	●	●	●	▲	●	●	CBML_P
574	331.2919 / 18.58	●	●	●	●	✓	●	●	CBML_P
577	593.2952 / 18.64	●	●	●	●	▲	●	●	CBML_P
589	277.2222 / 19.10	●	●	●	●	✓	●	●	CBML_P
590	309.2850 / 19.10	●	●	●	●	▲	●	●	CBML_P
602	551.4299 / 19.27	●	●	●	●	✓	●	●	CBML_P
604	601.4284 / 19.27	●	●	●	●	✓	●	●	CBML_P
610	535.2757 / 19.32	●	●	●	●	✓	●	●	CBML_P
613	223.0699 / 19.78	●	●	●	●	▲	●	●	CBML_P

#	Analyte Peak Name	Component Name	Component Type	Component Group Name	Expected RT	Area	Retention Time	Retention Time Delta (min)	Formula	Precursor Mass	Found At Mass
1	104.1155 / 1.02	104.1155 / 1.02	Quantifiers	[No data for] Component Group Name	1.02	2.364e+07	1.08	N/A	{103.10881}	104.116	104.1156
2	116.0753 / 1.02	116.0753 / 1.02	Quantifiers	[No data for] Component Group Name	1.02	1.908e+06	1.07	N/A	{115.06854}	116.075	116.0753

3	118.0911 / 1.02	118.0911 / 1.02	Quantifiers	[No data for] Component Group Name	1.02	2.880e+06	1.06	N/A	{117.08433}	118.091	118.0914
7	248.1179 / 1.02	248.1179 / 1.02	Quantifiers	[No data for] Component Group Name	1.02	2.446e+06	1.10	N/A	{247.11121}	248.118	248.1182
10	136.0704 / 1.08	136.0704 / 1.08	Quantifiers	[No data for] Component Group Name	1.08	1.277e+07	1.06	N/A	{135.06366}	136.070	136.0712
12	381.0885 / 1.08	381.0885 / 1.08	Quantifiers	[No data for] Component Group Name	1.08	2.456e+07	1.12	N/A	{380.08178}	381.089	381.0883
23	337.0947 / 1.37	337.0947 / 1.37	Quantifiers	[No data for] Component Group Name	1.37	8.395e+05	1.31	N/A	{336.08796}	337.095	337.0953
28	152.0648 / 1.48	152.0648 / 1.48	Quantifiers	[No data for] Component Group Name	1.48	9.207e+06	1.49	N/A	{151.05812}	152.065	152.0654
29	182.0868 / 1.48	182.0868 / 1.48	Quantifiers	[No data for] Component Group Name	1.48	9.030e+05	1.48	N/A	{181.08003}	182.087	182.0867
30	284.1075 / 1.48	284.1075 / 1.48	Quantifiers	[No data for] Component Group Name	1.48	7.129e+06	1.49	N/A	{283.10079}	284.108	284.1080
32	276.1521 / 1.54	276.1521 / 1.54	Quantifiers	[No data for] Component Group Name	1.54	9.720e+06	1.53	N/A	{275.14540}	276.152	276.1516
33	132.1101 / 1.59	132.1101 / 1.59	Quantifiers	[No data for] Component Group Name	1.59	1.067e+07	1.57	N/A	{131.10342}	132.110	132.1109
37	324.1009 / 1.82 [M+K]+	324.1009 / 1.82 [M+K]+	Qualifiers	286.1454 / 1.76	1.82	8.649e+05	1.78	N/A	{285.13833}	324.101	324.1013
38	282.1271 / 1.82	282.1271 / 1.82	Quantifiers	[No data for] Component Group Name	1.82	3.249e+06	1.82	N/A	{281.12042}	282.127	282.1277
42	182.0974 / 1.93 [M+H]+	182.0974 / 1.93 [M+H]+	Qualifiers	214.1240 / 1.88	1.93	1.183e+06	1.92	N/A	{181.09071}	182.097	182.0976
43	204.1281 / 1.93	204.1281 / 1.93	Quantifiers	[No data for] Component Group Name	1.93	1.126e+06	1.92	N/A	{203.12138}	204.128	204.1283
50	121.0900 / 2.27	121.0900 / 2.27	Quantifiers	[No data for] Component Group Name	2.27	4.407e+06	2.27	N/A	{120.08324}	121.090	121.0900
51	166.0972 / 2.27	166.0972 / 2.27	Quantifiers	[No data for] Component Group Name	2.27	2.912e+07	2.28	N/A	{165.09051}	166.097	166.0964

52	328.1478 / 2.27	328.1478 / 2.27	Quantifiers	[No data for] Component Group Name	2.27	1.553e+07	2.24	N/A	{327.14111}	328.148	328.1470
53	120.0928 / 2.33	120.0928 / 2.33	Quantifiers	[No data for] Component Group Name	2.33	3.067e+07	2.30	N/A	{119.08604}	120.093	120.0929
54	220.1231 / 2.44	220.1231 / 2.44	Quantifiers	[No data for] Component Group Name	2.45	1.381e+06	2.45	N/A	{219.11637}	220.123	220.1233
55	158.0863 / 2.62	158.0863 / 2.62	Quantifiers	[No data for] Component Group Name	2.62	1.449e+06	2.52	N/A	{157.07952}	158.086	158.0863
58	186.1285 / 2.67	186.1285 / 2.67	Quantifiers	[No data for] Component Group Name	2.67	1.258e+06	2.68	N/A	{185.12176}	186.128	186.1288
59	384.1200 / 2.73	384.1200 / 2.73	Quantifiers	[No data for] Component Group Name	2.73	9.860e+05	2.73	N/A	{383.11324}	384.120	384.1200
64	156.9668 / 3.52	156.9668 / 3.52	Quantifiers	[No data for] Component Group Name	3.53	2.571e+06	3.05	N/A	{155.96006}	156.967	156.9669
68	192.1069 / 4.04	192.1069 / 4.04	Quantifiers	[No data for] Component Group Name	4.04	1.008e+06	4.00	N/A	{191.10022}	192.107	192.1071
71	298.1027 / 4.26	298.1027 / 4.26	Quantifiers	[No data for] Component Group Name	4.26	1.681e+06	4.29	N/A	{297.09594}	298.103	298.1028
72	118.0697 / 4.43	118.0697 / 4.43	Quantifiers	[No data for] Component Group Name	4.43	1.695e+06	4.43	N/A	{117.06297}	118.070	118.0697
73	146.0653 / 4.43	146.0653 / 4.43	Quantifiers	[No data for] Component Group Name	4.43	6.210e+06	4.43	N/A	{145.05857}	146.065	146.0655
74	159.0964 / 4.43	159.0964 / 4.43	Quantifiers	[No data for] Component Group Name	4.43	1.465e+06	4.43	N/A	{158.08969}	159.096	159.0966
75	205.1058 / 4.43	205.1058 / 4.43	Quantifiers	[No data for] Component Group Name	4.43	2.150e+07	4.43	N/A	{204.09904}	205.106	205.1059
76	144.0854 / 4.49	144.0854 / 4.49	Quantifiers	[No data for] Component Group Name	4.49	2.012e+06	4.43	N/A	{143.07871}	144.085	144.0855
77	188.0849 / 4.49	188.0849 / 4.49	Quantifiers	[No data for] Component Group Name	4.49	4.805e+07	4.46	N/A	{187.07821}	188.085	188.0848
82	139.0448 / 5.00	139.0448 / 5.00	Quantifiers	[No data for]	5.00	2.700e+06	4.97	N/A	{138.03812}	139.045	139.0450

				Component Group Name							
84	339.0548 / 5.00	339.0548 / 5.00	Quantifiers	[No data for] Component Group Name	5.00	2.762e+06	4.97	N/A	{338.04803}	339.055	339.0550
86	238.1124 / 5.23	238.1124 / 5.23	Quantifiers	[No data for] Component Group Name	5.23	6.398e+05	5.16	N/A	{237.10571}	238.112	238.1124
88	120.0852 / 5.40	120.0852 / 5.40	Quantifiers	[No data for] Component Group Name	5.40	8.584e+05	5.39	N/A	{119.07852}	120.085	120.0856
90	295.1429 / 5.40	295.1429 / 5.40	Quantifiers	[No data for] Component Group Name	5.40	1.064e+07	5.41	N/A	{294.13621}	295.143	295.1436
94	174.1546 / 5.74	174.1546 / 5.74	Quantifiers	[No data for] Component Group Name	5.74	1.633e+06	5.76	N/A	{173.14786}	174.155	174.1548
95	217.1020 / 5.74	217.1020 / 5.74	Quantifiers	[No data for] Component Group Name	5.74	6.583e+05	5.77	N/A	{216.09525}	217.102	217.1025
101	166.0915 / 6.08	166.0915 / 6.08	Quantifiers	[No data for] Component Group Name	6.08	1.258e+06	6.08	N/A	{165.08472}	166.091	166.0916
103	212.0974 / 6.08 [M+H]+	212.0974 / 6.08 [M+H]+	Quantifiers	212.0974 / 6.08	6.08	1.254e+06	6.09	N/A	{211.09063}	212.097	212.0974
104	229.1238 / 6.08 [M+NH4]+	229.1238 / 6.08 [M+NH4]+	Qualifiers	212.0974 / 6.08	6.08	1.075e+06	6.10	N/A	{211.09053}	229.124	229.1239
114	377.1515 / 6.37	377.1515 / 6.37	Quantifiers	[No data for] Component Group Name	6.37	1.396e+06	6.37	N/A	{376.14480}	377.152	377.1514
115	265.1488 / 6.42	265.1488 / 6.42	Quantifiers	[No data for] Component Group Name	6.42	8.719e+05	6.40	N/A	{264.14205}	265.149	265.1489
118	209.1222 / 6.71	209.1222 / 6.71	Quantifiers	[No data for] Component Group Name	6.71	1.070e+06	6.71	N/A	{208.11550}	209.122	209.1225
132	585.2943 / 7.22 [M+K]+	585.2943 / 7.22 [M+K]+	Qualifiers	547.3391 / 7.22	7.22	3.786e+06	7.19	N/A	{546.33170}	585.294	585.2942
136	591.3660 / 7.39 [M+H]+	591.3660 / 7.39 [M+H]+	Quantifiers	591.3660 / 7.39	7.39	3.646e+06	7.39	N/A	{590.35930}	591.366	591.3662
137	629.3216 / 7.39 [M+K]+	629.3216 / 7.39 [M+K]+	Qualifiers	591.3660 / 7.39	7.39	3.277e+06	7.39	N/A	{590.35896}	629.322	629.3218
144	652.4275 / 7.56 [M+NH4]+	652.4275 / 7.56 [M+NH4]+	Quantifiers	652.4275 / 7.56	7.56	1.157e+07	7.55	N/A	{634.39419}	652.427	652.4276
147	347.1287 / 7.56	347.1287 / 7.56	Quantifiers	[No data for] Component Group	7.56	1.253e+06	7.58	N/A	{346.12196}	347.129	347.1293

				Name							
148	673.3468 / 7.56 [M+K]+	673.3468 / 7.56 [M+K]+	Quantifiers	673.3468 / 7.56	7.56	3.161e+06	7.56	N/A	{634.38419}	673.347	673.3464
149	635.3900 / 7.56 [M+H]+	635.3900 / 7.56 [M+H]+	Qualifiers	673.3468 / 7.56	7.56	2.603e+06	7.56	N/A	{634.38328}	635.390	635.3900
165	740.4750 / 7.84	740.4750 / 7.84	Quantifiers	[No data for] Component Group Name	7.84	5.164e+06	7.84	N/A	{739.46827}	740.475	740.4754
166	768.5018 / 7.84	768.5018 / 7.84	Quantifiers	[No data for] Component Group Name	7.84	3.450e+06	7.84	N/A	{767.49508}	768.502	768.5027
171	784.4976 / 7.96	784.4976 / 7.96	Quantifiers	[No data for] Component Group Name	7.96	3.442e+06	7.96	N/A	{783.49084}	784.498	784.4979
177	211.1742 / 8.07	211.1742 / 8.07	Quantifiers	[No data for] Component Group Name	8.07	2.011e+06	8.07	N/A	{210.16751}	211.174	211.1745
179	414.7690 / 8.07 [M+H+NH4]2+	414.7690 / 8.07 [M+H+NH4]2+	Qualifiers	423.2824 / 8.07	8.07	2.283e+06	8.08	N/A	{810.49799}	414.769	414.7689
184	146.0652 / 8.13	146.0652 / 8.13	Quantifiers	[No data for] Component Group Name	8.13	1.186e+06	8.13	N/A	{145.05852}	146.065	146.0652
198	383.1756 / 8.41	383.1756 / 8.41	Quantifiers	[No data for] Component Group Name	8.41	2.523e+06	8.41	N/A	{382.16889}	383.176	383.1757
208	213.1540 / 8.70	213.1540 / 8.70	Quantifiers	[No data for] Component Group Name	8.70	1.985e+06	8.68	N/A	{212.14729}	213.154	213.1541
217	464.2799 / 8.98	464.2799 / 8.98	Quantifiers	[No data for] Component Group Name	8.98	2.546e+06	8.96	N/A	{463.27321}	464.280	464.2797
222	115.0807 / 9.04	115.0807 / 9.04	Quantifiers	[No data for] Component Group Name	9.04	1.493e+06	9.04	N/A	{114.07396}	115.081	115.0806
223	309.2108 / 9.04	309.2108 / 9.04	Quantifiers	[No data for] Component Group Name	9.04	4.295e+06	8.94	N/A	{308.20405}	309.211	309.2110
225	211.1384 / 9.15	211.1384 / 9.15	Quantifiers	[No data for] Component Group Name	9.15	1.834e+06	9.13	N/A	{210.13163}	211.138	211.1383
231	446.2808 / 9.49	446.2808 / 9.49	Quantifiers	[No data for] Component Group Name	9.49	5.606e+06	9.59	N/A	{445.27402}	446.281	446.2809
233	448.2860 / 9.55	448.2860 / 9.55	Quantifiers	[No data for] Component Group Name	9.55	3.031e+06	9.55	N/A	{447.27928}	448.286	448.2861

234	191.1478 / 9.66	191.1478 / 9.66	Quantifiers	[No data for] Component Group Name	9.66	1.301e+06	9.69	N/A	{190.14108}	191.148	191.1482
235	193.1635 / 9.66	193.1635 / 9.66	Quantifiers	[No data for] Component Group Name	9.66	1.855e+06	9.63	N/A	{192.15678}	193.164	193.1637
236	211.1746 / 9.66	211.1746 / 9.66	Quantifiers	[No data for] Component Group Name	9.66	1.302e+06	9.64	N/A	{210.16785}	211.175	211.1746
241	306.2698 / 10.12	306.2698 / 10.12	Quantifiers	[No data for] Component Group Name	10.12	8.456e+03	10.05	N/A	{305.26310}	306.270	306.2733
245	275.2089 / 10.34	275.2089 / 10.34	Quantifiers	[No data for] Component Group Name	10.34	3.771e+07	10.36	N/A	{274.20218}	275.209	275.2097
246	293.2195 / 10.34	293.2195 / 10.34	Quantifiers	[No data for] Component Group Name	10.34	3.351e+07	10.36	N/A	{292.21279}	293.220	293.2203
247	311.2273 / 10.34	311.2273 / 10.34	Quantifiers	[No data for] Component Group Name	10.34	6.392e+06	10.35	N/A	{310.22060}	311.227	311.2275
250	195.1429 / 10.40	195.1429 / 10.40	Quantifiers	[No data for] Component Group Name	10.40	3.024e+06	10.38	N/A	{194.13617}	195.143	195.1430
254	325.2329 / 10.51	325.2329 / 10.51	Quantifiers	[No data for] Component Group Name	10.52	4.082e+07	10.33	N/A	{324.22621}	325.233	325.2382
261	731.4532 / 10.74 [M+H]+	731.4532 / 10.74 [M+H]+	Qualifiers	763.4792 / 10.69	10.74	3.027e+06	10.73	N/A	{730.44649}	731.453	731.4528
277	769.4763 / 11.20	769.4763 / 11.20	Quantifiers	[No data for] Component Group Name	11.20	2.071e+06	11.23	N/A	{768.46961}	769.476	769.4771
285	311.2275 / 11.31	311.2275 / 11.31	Quantifiers	[No data for] Component Group Name	11.31	6.023e+06	11.34	N/A	{310.22080}	311.228	311.2279
290	181.1279 / 11.59	181.1279 / 11.59	Quantifiers	[No data for] Component Group Name	11.59	4.909e+06	11.59	N/A	{180.12117}	181.128	181.1280
291	391.3413 / 11.59	391.3413 / 11.59	Quantifiers	[No data for] Component Group Name	11.59	3.921e+06	11.59	N/A	{390.33462}	391.341	391.3421
292	409.3522 / 11.59	409.3522 / 11.59	Quantifiers	[No data for] Component Group Name	11.59	6.348e+06	11.59	N/A	{408.34549}	409.352	409.3537
300	453.3405 / 11.71	453.3405 / 11.71	Quantifiers	[No data for] Component Group	11.71	7.318e+06	11.76	N/A	{452.33382}	453.341	453.3410

				Name							
301	428.2707 / 11.76	428.2707 / 11.76	Quantifiers	[No data for] Component Group Name	11.77	2.434e+06	11.76	N/A	{427.26396}	428.271	428.2708
316	291.2022 / 12.22	291.2022 / 12.22	Quantifiers	[No data for] Component Group Name	12.22	1.523e+07	12.22	N/A	{290.19545}	291.202	291.2025
320	498.3979 / 12.33	498.3979 / 12.33	Quantifiers	[No data for] Component Group Name	12.33	3.169e+06	12.33	N/A	{497.39122}	498.398	498.3987
325	583.4145 / 12.45	583.4145 / 12.45	Quantifiers	[No data for] Component Group Name	12.45	3.202e+06	12.46	N/A	{582.40773}	583.414	583.4147
326	540.4124 / 12.50	540.4124 / 12.50	Quantifiers	[No data for] Component Group Name	12.50	1.065e+07	12.53	N/A	{539.40569}	540.412	540.4127
342	293.2168 / 12.84	293.2168 / 12.84	Quantifiers	[No data for] Component Group Name	12.85	6.317e+06	12.84	N/A	{292.21011}	293.217	293.2167
346	391.3418 / 12.90	391.3418 / 12.90	Quantifiers	[No data for] Component Group Name	12.90	2.950e+07	12.83	N/A	{390.33505}	391.342	391.3422
349	419.3436 / 12.90	419.3436 / 12.90	Quantifiers	[No data for] Component Group Name	12.90	1.359e+08	12.88	N/A	{418.33689}	419.344	419.3435
362	257.1947 / 13.24	257.1947 / 13.24	Quantifiers	[No data for] Component Group Name	13.24	3.152e+06	13.26	N/A	{256.18802}	257.195	257.1951
379	554.4241 / 13.70	554.4241 / 13.70	Quantifiers	[No data for] Component Group Name	13.70	4.958e+06	13.69	N/A	{553.41739}	554.424	554.4240
383	275.2068 / 13.81	275.2068 / 13.81	Quantifiers	[No data for] Component Group Name	13.81	1.835e+07	13.79	N/A	{274.20010}	275.207	275.2065
391	304.2905 / 13.98	304.2905 / 13.98	Quantifiers	[No data for] Component Group Name	13.98	1.021e+07	13.91	N/A	{303.28380}	304.291	304.2905
396	668.4394 / 14.15	668.4394 / 14.15	Quantifiers	[No data for] Component Group Name	14.15	3.831e+06	14.17	N/A	{667.43268}	668.439	668.4399
397	453.3409 / 14.21	453.3409 / 14.21	Quantifiers	[No data for] Component Group Name	14.21	1.594e+07	13.97	N/A	{452.33417}	453.341	453.3411
409	523.3455 / 14.61	523.3455 / 14.61	Quantifiers	[No data for] Component Group Name	14.61	5.120e+06	14.63	N/A	{522.33880}	523.346	523.3453

410	496.3448 / 14.78	496.3448 / 14.78	Quantifiers	[No data for] Component Group Name	14.78	1.853e+07	14.76	N/A	{495.33811}	496.345	496.3453
411	275.2061 / 14.83	275.2061 / 14.83	Quantifiers	[No data for] Component Group Name	14.83	1.833e+06	14.83	N/A	{274.19940}	275.206	275.2062
412	293.2166 / 14.83	293.2166 / 14.83	Quantifiers	[No data for] Component Group Name	14.83	2.844e+06	14.83	N/A	{292.20990}	293.217	293.2167
419	309.2624 / 15.12	309.2624 / 15.12	Quantifiers	[No data for] Component Group Name	15.12	2.973e+06	15.12	N/A	{308.25567}	309.262	309.2624
420	391.3417 / 15.12	391.3417 / 15.12	Quantifiers	[No data for] Component Group Name	15.12	1.343e+07	15.12	N/A	{390.33496}	391.342	391.3425
423	419.3406 / 15.12	419.3406 / 15.12	Quantifiers	[No data for] Component Group Name	15.12	2.467e+07	15.13	N/A	{418.33391}	419.341	419.3422
424	455.3583 / 15.12	455.3583 / 15.12	Quantifiers	[No data for] Component Group Name	15.12	1.883e+07	15.12	N/A	{454.35153}	455.358	455.3585
430	279.2279 / 15.29	279.2279 / 15.29	Quantifiers	[No data for] Component Group Name	15.29	3.540e+06	15.32	N/A	{278.22114}	279.228	279.2281
431	553.4366 / 15.29	553.4366 / 15.29	Quantifiers	[No data for] Component Group Name	15.29	2.049e+07	15.31	N/A	{552.42984}	553.437	553.4371
432	277.2312 / 15.35	277.2312 / 15.35	Quantifiers	[No data for] Component Group Name	15.35	9.695e+07	15.36	N/A	{276.22444}	277.231	277.2313
437	277.2207 / 15.57	277.2207 / 15.57	Quantifiers	[No data for] Component Group Name	15.57	6.647e+07	15.38	N/A	{276.21401}	277.221	277.2280
439	181.1274 / 15.69	181.1274 / 15.69	Quantifiers	[No data for] Component Group Name	15.69	1.956e+06	15.66	N/A	{180.12070}	181.127	181.1276
441	332.3214 / 15.69	332.3214 / 15.69	Quantifiers	[No data for] Component Group Name	15.69	4.060e+06	15.77	N/A	{331.31471}	332.321	332.3213
444	353.2752 / 15.74	353.2752 / 15.74	Quantifiers	[No data for] Component Group Name	15.74	6.141e+06	15.74	N/A	{352.26845}	353.275	353.2752
450	275.2086 / 15.91	275.2086 / 15.91	Quantifiers	[No data for] Component Group Name	15.91	8.428e+06	15.92	N/A	{274.20188}	275.209	275.2087
453	203.1847 / 16.14	203.1847 / 16.14	Quantifiers	[No data for]	16.14	6.694e+06	16.14	N/A	{202.17801}	203.185	203.1847

				Component Group Name							
454	337.2582 / 16.14	337.2582 / 16.14	Quantifiers	[No data for] Component Group Name	16.14	1.363e+07	16.14	N/A	{336.25150}	337.258	337.2585
459	419.3425 / 16.14	419.3425 / 16.14	Quantifiers	[No data for] Component Group Name	16.14	1.037e+08	16.16	N/A	{418.33573}	419.342	419.3418
461	455.3599 / 16.14	455.3599 / 16.14	Quantifiers	[No data for] Component Group Name	16.14	6.086e+07	16.14	N/A	{454.35320}	455.360	455.3598
468	423.3671 / 16.54	423.3671 / 16.54	Quantifiers	[No data for] Component Group Name	16.54	2.390e+06	16.55	N/A	{422.36038}	423.367	423.3670
475	277.2252 / 16.60	277.2252 / 16.60	Quantifiers	[No data for] Component Group Name	16.60	1.886e+07	16.60	N/A	{276.21849}	277.225	277.2257
476	295.2349 / 16.60	295.2349 / 16.60	Quantifiers	[No data for] Component Group Name	16.60	1.491e+07	16.64	N/A	{294.22813}	295.235	295.2345
477	447.2544 / 16.60	447.2544 / 16.60	Quantifiers	[No data for] Component Group Name	16.60	2.055e+06	16.59	N/A	{446.24767}	447.254	447.2552
481	523.3496 / 16.77	523.3496 / 16.77	Quantifiers	[No data for] Component Group Name	16.77	9.693e+06	16.78	N/A	{522.34288}	523.350	523.3504
483	423.3171 / 16.82	423.3171 / 16.82	Quantifiers	[No data for] Component Group Name	16.82	6.729e+06	16.82	N/A	{422.31040}	423.317	423.3173
488	355.2892 / 16.94	355.2892 / 16.94	Quantifiers	[No data for] Component Group Name	16.94	1.222e+07	17.10	N/A	{354.28251}	355.289	355.2889
491	154.0545 / 16.99	154.0545 / 16.99	Quantifiers	[No data for] Component Group Name	16.99	1.307e+06	16.99	N/A	{153.04782}	154.055	154.0549
500	599.4141 / 17.11	599.4141 / 17.11	Quantifiers	[No data for] Component Group Name	17.11	1.396e+07	17.31	N/A	{598.40741}	599.414	599.4137
502	453.3430 / 17.16	453.3430 / 17.16	Quantifiers	[No data for] Component Group Name	17.16	1.822e+07	16.97	N/A	{452.33624}	453.343	453.3434
511	349.2821 / 17.33	349.2821 / 17.33	Quantifiers	[No data for] Component Group Name	17.33	1.633e+07	17.33	N/A	{348.27536}	349.282	349.2824
514	341.3105 / 17.39	341.3105 / 17.39	Quantifiers	[No data for] Component Group	17.39	1.450e+07	17.38	N/A	{340.30377}	341.310	341.3108

				Name							
515	359.3216 / 17.39	359.3216 / 17.39	Quantifiers	[No data for] Component Group Name	17.39	1.301e+07	17.38	N/A	{358.31491}	359.322	359.3217
516	607.2953 / 17.39	607.2953 / 17.39	Quantifiers	[No data for] Component Group Name	17.39	6.371e+06	17.40	N/A	{606.28860}	607.295	607.2954
518	353.2761 / 17.45	353.2761 / 17.45	Quantifiers	[No data for] Component Group Name	17.45	1.726e+07	17.47	N/A	{352.26940}	353.276	353.2766
519	391.3408 / 17.45	391.3408 / 17.45	Quantifiers	[No data for] Component Group Name	17.45	1.869e+07	17.46	N/A	{390.33410}	391.341	391.3409
521	419.3375 / 17.45	419.3375 / 17.45	Quantifiers	[No data for] Component Group Name	17.45	4.093e+07	17.46	N/A	{418.33082}	419.338	419.3390
523	459.3873 / 17.50	459.3873 / 17.50	Quantifiers	[No data for] Component Group Name	17.51	4.165e+06	17.51	N/A	{458.38061}	459.387	459.3875
526	305.2556 / 17.56	305.2556 / 17.56	Quantifiers	[No data for] Component Group Name	17.56	1.065e+07	17.56	N/A	{304.24886}	305.256	305.2558
531	623.2548 / 17.68	623.2548 / 17.68	Quantifiers	[No data for] Component Group Name	17.68	9.690e+06	17.59	N/A	{622.24804}	623.255	623.2545
534	421.3513 / 17.73	421.3513 / 17.73	Quantifiers	[No data for] Component Group Name	17.73	6.326e+06	17.73	N/A	{420.34452}	421.351	421.3510
538	149.0282 / 17.79	149.0282 / 17.79	Quantifiers	[No data for] Component Group Name	17.79	7.570e+06	17.80	N/A	{148.02152}	149.028	149.0284
541	279.2382 / 17.85	279.2382 / 17.85	Quantifiers	[No data for] Component Group Name	17.85	8.661e+06	17.82	N/A	{278.23150}	279.238	279.2382
551	583.4191 / 18.19	583.4191 / 18.19	Quantifiers	[No data for] Component Group Name	18.19	1.582e+07	18.17	N/A	{582.41235}	583.419	583.4191
558	391.3414 / 18.36	391.3414 / 18.36	Quantifiers	[No data for] Component Group Name	18.36	1.667e+07	18.37	N/A	{390.33470}	391.341	391.3418
561	419.3390 / 18.36	419.3390 / 18.36	Quantifiers	[No data for] Component Group Name	18.36	3.185e+07	18.38	N/A	{418.33224}	419.339	419.3428
565	609.2838 / 18.41	609.2838 / 18.41	Quantifiers	[No data for] Component Group Name	18.41	4.767e+07	18.36	N/A	{608.27709}	609.284	609.2844

568	439.3632 / 18.53	439.3632 / 18.53	Quantifiers	[No data for] Component Group Name	18.53	1.729e+07	18.53	N/A	{438.35647}	439.363	439.3627
573	313.2839 / 18.58	313.2839 / 18.58	Quantifiers	[No data for] Component Group Name	18.58	6.833e+07	18.54	N/A	{312.27718}	313.284	313.2839
574	331.2919 / 18.58	331.2919 / 18.58	Quantifiers	[No data for] Component Group Name	18.58	3.669e+07	18.57	N/A	{330.28521}	331.292	331.2927
577	593.2952 / 18.64	593.2952 / 18.64	Quantifiers	[No data for] Component Group Name	18.64	1.254e+08	18.63	N/A	{592.28844}	593.295	593.2975
589	277.2222 / 19.10	277.2222 / 19.10	Quantifiers	[No data for] Component Group Name	19.10	3.106e+06	19.08	N/A	{276.21545}	277.222	277.2223
590	309.2850 / 19.10	309.2850 / 19.10	Quantifiers	[No data for] Component Group Name	19.10	3.933e+06	19.12	N/A	{308.27828}	309.285	309.2849
602	551.4299 / 19.27	551.4299 / 19.27	Quantifiers	[No data for] Component Group Name	19.27	1.211e+07	19.23	N/A	{550.42319}	551.430	551.4304
604	601.4284 / 19.27	601.4284 / 19.27	Quantifiers	[No data for] Component Group Name	19.27	4.285e+06	19.22	N/A	{600.42164}	601.428	601.4285
610	535.2757 / 19.32	535.2757 / 19.32	Quantifiers	[No data for] Component Group Name	19.32	2.315e+06	19.32	N/A	{534.26902}	535.276	535.2756
613	223.0699 / 19.78	223.0699 / 19.78	Quantifiers	[No data for] Component Group Name	19.78	6.304e+05	19.62	N/A	{222.06321}	223.070	223.0699

#	Analyte Peak Name	Library Hit	Library Score
1	104.1155 / 1.02	Choline cation (NIST) [Smart Confirmation]	95.2
2	116.0753 / 1.02	脯氨酸 Proline [Smart Confirmation]	49.7
3	118.0911 / 1.02	N-Methyl-.alpha.-aminoisobutyric acid (NIST) [Smart Confirmation]	60.8
7	248.1179 / 1.02	Lys-Thr (NIST) [Smart Confirmation]	42.0
10	136.0704 / 1.08	Adenine (NIST) [Smart Confirmation]	56.1
12	381.0885 / 1.08	Lactobionic acid (NIST) [Smart Confirmation]	95.0
23	337.0947 / 1.37	Isoprostaglandin-F2.alpha.-IV (NIST) [Smart Confirmation]	100.0
28	152.0648 / 1.48	O-Methyltyramine (NIST) [Smart Confirmation]	82.1
29	182.0868 / 1.48	L-Tyrosine (NIST) [Smart Confirmation]	97.8
30	284.1075 / 1.48	Guanosine (NIST) [Smart Confirmation]	100.0
32	276.1521 / 1.54	Gln-Phe (NIST) [Smart Confirmation]	58.7
33	132.1101 / 1.59	异亮氨酸Isoleucine [Smart Confirmation]	100.0
37	324.1009 / 1.82 [M+K]+	Cyamemazine (NIST) [Smart Confirmation]	98.4

38	282.1271 / 1.82	2'-O-Methyladenosine (NIST) [Smart Confirmation]	96.8
42	182.0974 / 1.93 [M+H]+	DL-m-Tyrosine (NIST) [Smart Confirmation]	87.3
43	204.1281 / 1.93	Indole-3-pyruvic acid (NIST) [Smart Confirmation]	99.1
50	121.0900 / 2.27	2-(3-Hydroxyphenyl)ethanol (NIST) [Smart Confirmation]	95.3
51	166.0972 / 2.27	苯丙氨酸Phenylalanine [Smart Confirmation]	99.8
52	328.1478 / 2.27	10-Nitrooleic acid (NIST) [Smart Confirmation]	46.1
53	120.0928 / 2.33	S-(2-Aminoethyl)isothiourea (NIST) [Smart Confirmation]	69.1
54	220.1231 / 2.44	Pantothenic acid (NIST) [Smart Confirmation]	87.1
55	158.0863 / 2.62	Ser-Ala-Arg (NIST) [Smart Confirmation]	23.1
58	186.1285 / 2.67	Phosphoserine (NIST) [Smart Confirmation]	55.8
59	384.1200 / 2.73	Degrasyn (NIST) [Smart Confirmation]	73.9
64	156.9668 / 3.52	4-Hydroxynonenal (NIST) [Smart Confirmation]	30.1
68	192.1069 / 4.04	(Z)-1-N-(3-Aminopropyl)-N-(3-ammoniopropyl)aminodiazen-1-ium-1,2-diolate (NIST) [Smart Confirmation]	80.4
71	298.1027 / 4.26	5'-S-Methyl-5'-thioadenosine (NIST) [Smart Confirmation]	100.0
72	118.0697 / 4.43	Indole (NIST) [Smart Confirmation]	95.2
73	146.0653 / 4.43	Indole-6-carboxaldehyde (NIST) [Smart Confirmation]	56.7
74	159.0964 / 4.43	1,5-Diaminonaphthalene (NIST) [Smart Confirmation]	85.1
75	205.1058 / 4.43	L-Tryptophan (NIST) [Smart Confirmation]	98.0
76	144.0854 / 4.49	1-Naphthalenamine (NIST) [Smart Confirmation]	64.8
77	188.0849 / 4.49	3-Indoleacrylic acid (NIST) [Smart Confirmation]	96.8
82	139.0448 / 5.00	Salicylic acid (NIST) [Smart Confirmation]	95.5
84	339.0548 / 5.00	Tetraphenylphosphonium cation (NIST) [Smart Confirmation]	95.7
86	238.1124 / 5.23	6-Biopterin (NIST) [Smart Confirmation]	28.2
88	120.0852 / 5.40	Benzoxazole (NIST) [Smart Confirmation]	53.9
90	295.1429 / 5.40	Glu-Phe (NIST) [Smart Confirmation]	66.6
94	174.1546 / 5.74	Swainsonine (NIST) [Smart Confirmation]	77.6
95	217.1020 / 5.74	N,N-Diethyltryptamine (NIST) [Smart Confirmation]	54.3
101	166.0915 / 6.08	(-)-Pseudoephedrine (NIST) [Smart Confirmation]	42.8
103	212.0974 / 6.08 [M+H]+	Methyldopa (NIST) [Smart Confirmation]	22.2
104	229.1238 / 6.08 [M+NH4]+	Zebularine (NIST) [Smart Confirmation]	76.1
114	377.1515 / 6.37	维生素B2 Vitamin B2 [Smart Confirmation]	60.1
115	265.1488 / 6.42	Dinor-12-oxophytodienoic acid (NIST) [Smart Confirmation]	49.9
118	209.1222 / 6.71	(E)-Chalcone (NIST) [Smart Confirmation]	29.8
132	585.2943 / 7.22 [M+K]+	Hemslecin A (NIST) [Smart Confirmation]	56.6
136	591.3660 / 7.39 [M+H]+	Urobilin (NIST) [Smart Confirmation]	42.7
137	629.3216 / 7.39 [M+K]+	Octadecamethyloctasiloxane (NIST) [Smart Confirmation]	100.0
144	652.4275 / 7.56 [M+NH4]+	1-Hexadecyl-2-azelaoyl-sn-glycero-3-phosphocholine (NIST) [Smart Confirmation]	35.5
147	347.1287 / 7.56	.beta.-Estradiol 17-propionate (NIST) [Smart Confirmation]	70.6
148	673.3468 / 7.56 [M+K]+	Thapsigargin (NIST) [Smart Confirmation]	87.3
149	635.3900 / 7.56 [M+H]+	Rescinnamine (NIST) [Smart Confirmation]	22.5
165	740.4750 / 7.84	1,2-Dilinoleoyl-sn-glycero-3-phosphoethanolamine (NIST) [Smart Confirmation]	21.2
166	768.5018 / 7.84	1-Hexadecyl-2-(5Z,8Z,11Z,14Z-eicosatetraenoyl)-sn-glycero-3-phosphocholine (NIST) [Smart Confirmation]	37.3
171	784.4976 / 7.96	Arachidonoylthiophosphorylcholine (NIST) [Smart Confirmation]	74.0
177	211.1742 / 8.07	Jasmonic acid (NIST) [Smart Confirmation]	25.4

179	414.7690 / 8.07 [M+H+NH4]2+	Nonaethylene glycol (NIST) [Smart Confirmation]	75.3
184	146.0652 / 8.13	Indole-6-carboxaldehyde (NIST) [Smart Confirmation]	98.0
198	383.1756 / 8.41	7.alpha.,25-Dihydroxycholesterol (NIST) [Smart Confirmation]	96.3
208	213.1540 / 8.70	Tetradec-11-en-1-ol (NIST) [Smart Confirmation]	28.2
217	464.2799 / 8.98	Benzamide, N-5-2-(3,5-dimethoxyphenyl)ethyl-1H-pyrazol-3-yl-4-(3R,5S)-3,5-dimethyl-1-piperazinyl-, rel- (NIST) [Smart Confirmation]	22.9
222	115.0807 / 9.04	Dihydrouracil (NIST) [Smart Confirmation]	49.4
223	309.2108 / 9.04	Gestrinone (NIST) [Smart Confirmation]	30.8
225	211.1384 / 9.15	Jasmonic acid (NIST) [Smart Confirmation]	20.3
231	446.2808 / 9.49	L-Serine, N-(1-oxohexadecyl)-O-phosphono- (NIST) [Smart Confirmation]	78.8
233	448.2860 / 9.55	Sulprostone (NIST) [Smart Confirmation]	94.2
234	191.1478 / 9.66	Z-Ligustilide (NIST) [Smart Confirmation]	54.6
235	193.1635 / 9.66	.alpha.-Ionone (NIST) [Smart Confirmation]	79.1
236	211.1746 / 9.66	Jasmonic acid (NIST) [Smart Confirmation]	64.2
241	306.2698 / 10.12	Ethanone, 1-(1-pentyl-1H-indol-3-yl)-2-phenyl- (NIST) [Smart Confirmation]	23.8
245	275.2089 / 10.34	17.alpha.-Nandrolone (NIST) [Smart Confirmation]	65.5
246	293.2195 / 10.34	9-Oxo-10E,12Z,15Z-octadecatrienoic acid (NIST) [Smart Confirmation]	68.7
247	311.2273 / 10.34	cis-11-Eicosenoic acid (NIST) [Smart Confirmation]	28.6
250	195.1429 / 10.40	Sedanolide (NIST) [Smart Confirmation]	46.9
254	325.2329 / 10.51	2,5-Pyrrolidinedione, 1-2-2,5-dimethyl-1-(phenylmethyl)-1H-pyrrol-3-yl-2-oxoethyl- (NIST) [Smart Confirmation]	28.2
261	731.4532 / 10.74 [M+H]+	N-(Octadecanoyl)sphing-4-enine-1-phosphocholine (NIST) [Smart Confirmation]	26.2
277	769.4763 / 11.20	1-Palmitoyl-2-stearoyl-sn-glycero-3-phospho-rac-1-glycerol (NIST) [Smart Confirmation]	90.8
285	311.2275 / 11.31	cis-13-Eicosenoic acid (NIST) [Smart Confirmation]	45.9
290	181.1279 / 11.59	4-Hydroxyphenylpyruvic acid (NIST) [Smart Confirmation]	86.2
291	391.3413 / 11.59	.gamma.-Muricholic acid (NIST) [Smart Confirmation]	63.1
292	409.3522 / 11.59	Beclomethasone (NIST) [Smart Confirmation]	42.3
300	453.3405 / 11.71	Met(O)-Met-Arg (NIST) [Smart Confirmation]	50.1
301	428.2707 / 11.76	Adenosine 5'-diphosphate (NIST) [Smart Confirmation]	88.7
316	291.2022 / 12.22	trans-Androsterone (NIST) [Smart Confirmation]	54.6
320	498.3979 / 12.33	Microcystin LR (NIST) [Smart Confirmation]	80.5
325	583.4145 / 12.45	Geldanamycin (NIST) [Smart Confirmation]	87.1
326	540.4124 / 12.50	Nintedanib (NIST) [Smart Confirmation]	71.3
342	293.2168 / 12.84	13S-Hydroxy-9Z,11E-octadecadienoic acid, methyl ester (NIST) [Smart Confirmation]	60.5
346	391.3418 / 12.90	.gamma.-Muricholic acid (NIST) [Smart Confirmation]	46.0
349	419.3436 / 12.90	5.alpha.-Hydroxy-6-ketocholesterol (NIST) [Smart Confirmation]	52.1
362	257.1947 / 13.24	补骨脂酚Bakuchiol [Smart Confirmation]	23.2
379	554.4241 / 13.70	3,5,9-Trioxa-4-phosphatetracosan-1-aminium, 7-(acetyloxy)-24-carboxy-4-hydroxy-N,N,N-trimethyl-, inner salt, 4-oxide, (R)- (NIST) [Smart Confirmation]	25.5
383	275.2068 / 13.81	Nandrolone (NIST) [Smart Confirmation]	73.8
391	304.2905 / 13.98	Glu-Arg (NIST) [Smart Confirmation]	100.0
396	668.4394 / 14.15	长梗冬青苷 Pedunculoside +NH3 [Smart Confirmation]	48.8
397	453.3409 / 14.21	17-Trifluoromethylphenyltrinorprostaglandin F2.alpha. methyl ester (NIST) [Smart Confirmation]	84.7
409	523.3455 / 14.61	Ticagrelor (NIST) [Smart Confirmation]	24.2
410	496.3448 / 14.78	1-Palmitoyl-sn-glycero-3-phosphocholine (NIST) [Smart Confirmation]	97.2

411	275.2061 / 14.83	5.beta.-Androstane-3.beta.,17.beta.-diol (NIST) [Smart Confirmation]	97.8
412	293.2166 / 14.83	Cyclopentanehexanoic acid, 2-(2Z)-4-carboxy-2-buten-1-yl-3,5-dihydroxy-.gamma.-oxo-, (1R,2R,3S,5S)- (NIST) [Smart Confirmation]	63.8
419	309.2624 / 15.12	2,2,4-Trimethyl-1,3-pentanediol diisobutyrate (NIST) [Smart Confirmation]	52.5
420	391.3417 / 15.12	(R)-Butaprost (NIST) [Smart Confirmation]	63.1
423	419.3406 / 15.12	5.alpha.-Hydroxy-6-ketocholesterol (NIST) [Smart Confirmation]	37.4
424	455.3583 / 15.12	路路通酸 Betulonicacid [Smart Confirmation]	50.9
430	279.2279 / 15.29	Oxymetazoline impurity A (NIST) [Smart Confirmation]	77.4
431	553.4366 / 15.29	1-Stearoyl-2-hydroxy-sn-glycero-3-phosphate (NIST) [Smart Confirmation]	31.1
432	277.2312 / 15.35	Stearidonic acid (NIST) [Smart Confirmation]	97.5
437	277.2207 / 15.57	9,12-Octadecadiynoic acid (NIST) [Smart Confirmation]	96.1
439	181.1274 / 15.69	Butylated hydroxyanisole (NIST) [Smart Confirmation]	34.5
441	332.3214 / 15.69	Benzyldimethyltetradecylammonium cation (NIST) [Smart Confirmation]	91.5
444	353.2752 / 15.74	Monolinolenin (9c,12c,15c) (NIST) [Smart Confirmation]	34.4
450	275.2086 / 15.91	Phloretin (NIST) [Smart Confirmation]	43.4
453	203.1847 / 16.14	Amylcinnamic aldehyde (NIST) [Smart Confirmation]	34.8
454	337.2582 / 16.14	Fluoxymesterone (NIST) [Smart Confirmation]	30.2
459	419.3425 / 16.14	5.alpha.-Hydroxy-6-ketocholesterol (NIST) [Smart Confirmation]	40.0
461	455.3599 / 16.14	路路通酸 Betulonicacid [Smart Confirmation]	59.7
468	423.3671 / 16.54	1,2-Didecanoyl-sn-glycerol (NIST) [Smart Confirmation]	73.4
475	277.2252 / 16.60	9,12-Octadecadiynoic acid (NIST) [Smart Confirmation]	79.9
476	295.2349 / 16.60	13-Keto-9Z,11E-octadecadienoic acid (NIST) [Smart Confirmation]	29.5
477	447.2544 / 16.60	Cefuroxime (NIST) [Smart Confirmation]	77.5
481	523.3496 / 16.77	Ticagrelor (NIST) [Smart Confirmation]	23.5
483	423.3171 / 16.82	1,2-Didecanoyl-sn-glycerol (NIST) [Smart Confirmation]	54.2
488	355.2892 / 16.94	Tetrahydrocurcumin (NIST) [Smart Confirmation]	31.2
491	154.0545 / 16.99	3-Aminosalicylic acid (NIST) [Smart Confirmation]	41.2
500	599.4141 / 17.11	Phe-Met-Arg-Phe-amide (NIST) [Smart Confirmation]	21.8
502	453.3430 / 17.16	Met(O)-Met-Arg (NIST) [Smart Confirmation]	58.5
511	349.2821 / 17.33	Serpentine cation (NIST) [Smart Confirmation]	50.5
514	341.3105 / 17.39	(+)-Pinoresinol (NIST) [Smart Confirmation]	92.8
515	359.3216 / 17.39	1-Stearoyl-rac-glycerol (NIST) [Smart Confirmation]	83.2
516	607.2953 / 17.39	1,2-Dioctadecanoyl-sn-glycerol (NIST) [Smart Confirmation]	53.3
518	353.2761 / 17.45	Monolinolenin (9c,12c,15c) (NIST) [Smart Confirmation]	96.4
519	391.3408 / 17.45	(R)-Butaprost (NIST) [Smart Confirmation]	67.2
521	419.3375 / 17.45	5.alpha.-Hydroxy-6-ketocholesterol (NIST) [Smart Confirmation]	29.1
523	459.3873 / 17.50	Protopanaxatriol (NIST) [Smart Confirmation]	29.1
526	305.2556 / 17.56	Stearidonic acid ethyl ester (NIST) [Smart Confirmation]	97.4
531	623.2548 / 17.68	6,10,11-Triethylcarbonate-1-demethyl daunomycinone (NIST) [Smart Confirmation]	21.8
534	421.3513 / 17.73	4,6-Pyrimidinediamine, 2-1-(2-fluorophenyl)methyl-1H-pyrazolo3,4-bpyridin-3-yl-5-(4-morpholinyl)- (NIST) [Smart Confirmation]	52.2
538	149.0282 / 17.79	Phthalic anhydride (NIST) [Smart Confirmation]	95.0
541	279.2382 / 17.85	Pentoxifylline (NIST) [Smart Confirmation]	21.6
551	583.4191 / 18.19	Geldanamycin (NIST) [Smart Confirmation]	91.9
558	391.3414 / 18.36	.gamma.-Muricholic acid (NIST) [Smart Confirmation]	100.0
561	419.3390 / 18.36	5.alpha.-Hydroxy-6-ketocholesterol (NIST) [Smart Confirmation]	30.6

565	609.2838 / 18.41	1-(1,2-Dioctanoylphosphatidyl)inositol (NIST) [Smart Confirmation]	99.1
568	439.3632 / 18.53	.alpha.,.alpha.'-Dilaurin (NIST) [Smart Confirmation]	95.2
573	313.2839 / 18.58	18-Methylnonadecanoic acid (NIST) [Smart Confirmation]	54.9
574	331.2919 / 18.58	1-Palmitoylglycerol (NIST) [Smart Confirmation]	94.6
577	593.2952 / 18.64	Pheophorbide a (NIST) [Smart Confirmation]	50.8
589	277.2222 / 19.10	Stearidonic acid (NIST) [Smart Confirmation]	92.1
590	309.2850 / 19.10	8Z,14Z-Eicosadienoic acid (NIST) [Smart Confirmation]	64.8
602	551.4299 / 19.27	Pluripotin (NIST) [Smart Confirmation]	76.9
604	601.4284 / 19.27	1-Oleoyl-2-linoleoyl-rac-glycerol (NIST) [Smart Confirmation]	79.4
610	535.2757 / 19.32	Kahweol palmitate (NIST) [Smart Confirmation]	71.2
613	223.0699 / 19.78	Hexamethylcyclotrisiloxane (NIST) [Smart Confirmation]	54.5

End of Table

104.1155 / 1.02 (Mass/FragMass/RT/Isotope/Library/Formula/Ion Ratio)

Retention Time: 1.08 minutes **Exp RT:** 1.02 minutes
Precursor m/z : 104.1155 **Analyte Name:** 104.1155 / 1.02
Fit (%) 97.0% **RFit (%)** 98.9%

Collision Energy = 35 ± 15 eV

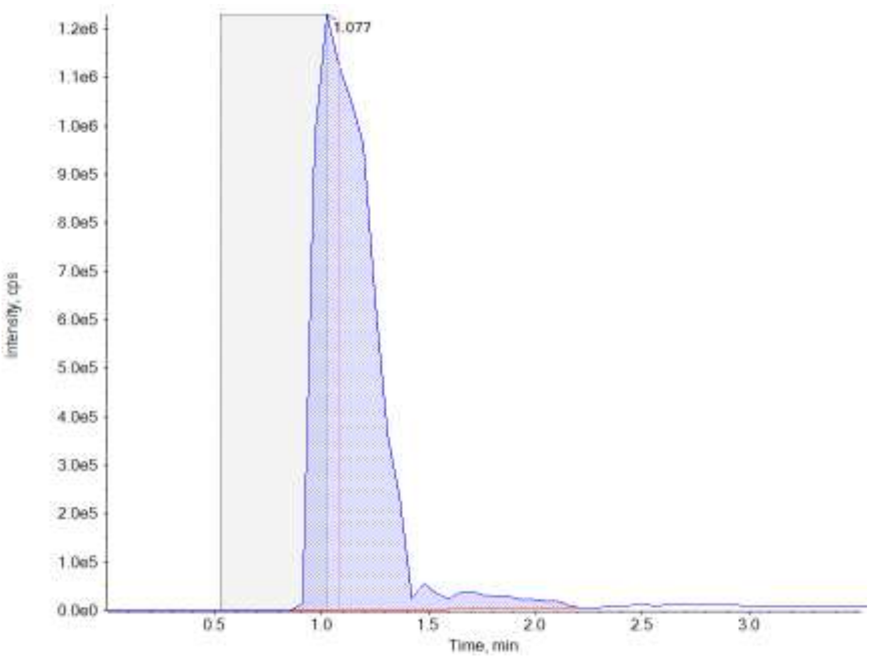

Acquired / Library MSMS

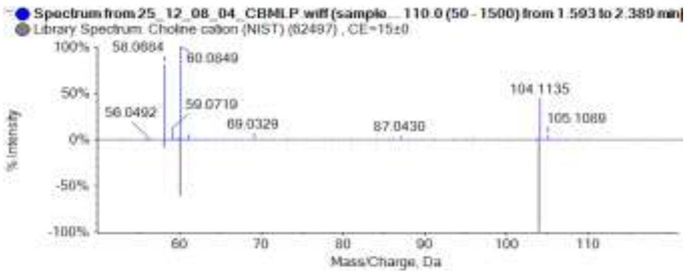

Acquired / Theoretical MS

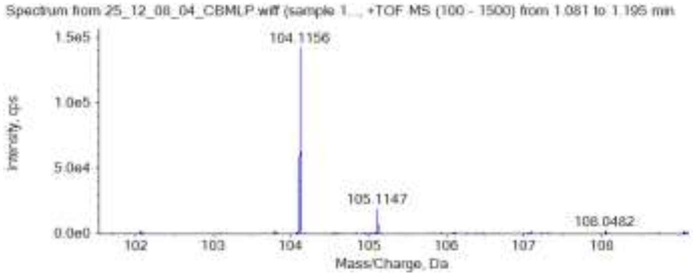

116.0753 / 1.02

(Mass/FragMass/RT/Isotope/Library/Formula/Ion Ratio)

Retention Time: 1.07 minutes
Precursor m/z : 116.0753
Fit (%) 98.5% **RFit (%)** 57.3%

Exp RT: 1.02 minutes
Analyte Name:
116.0753 / 1.02

Collision Energy = 35 ± 15 eV

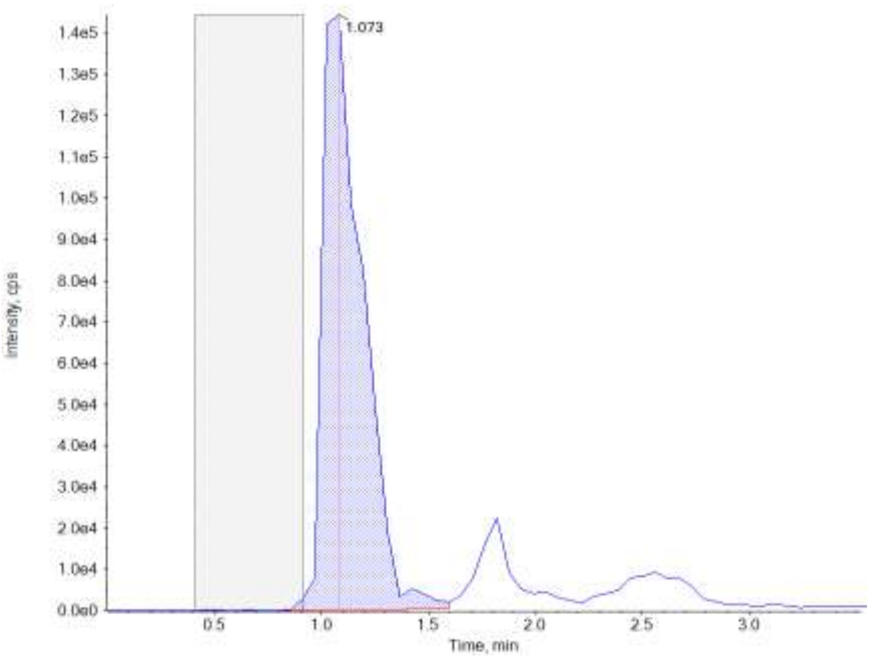

Acquired / Library MSMS

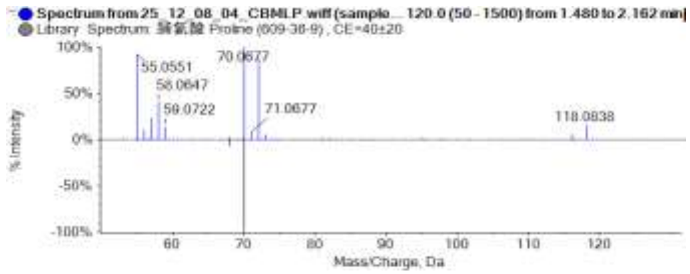

Acquired / Theoretical MS

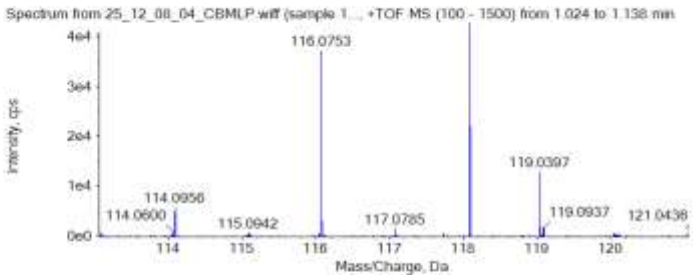

118.0911 / 1.02 (Mass/FragMass/RT/Isotope/Library/Formula/Ion Ratio)

**Retention Time:** 1.06 minutes	**Exp RT:** 1.02 minutes
**Precursor m/z :** 118.0911	**Analyte Name:**
**Fit (%)** 89.6% **RFit (%)** 87.7%	118.0911 / 1.02

Collision Energy = 35 ± 15 eV

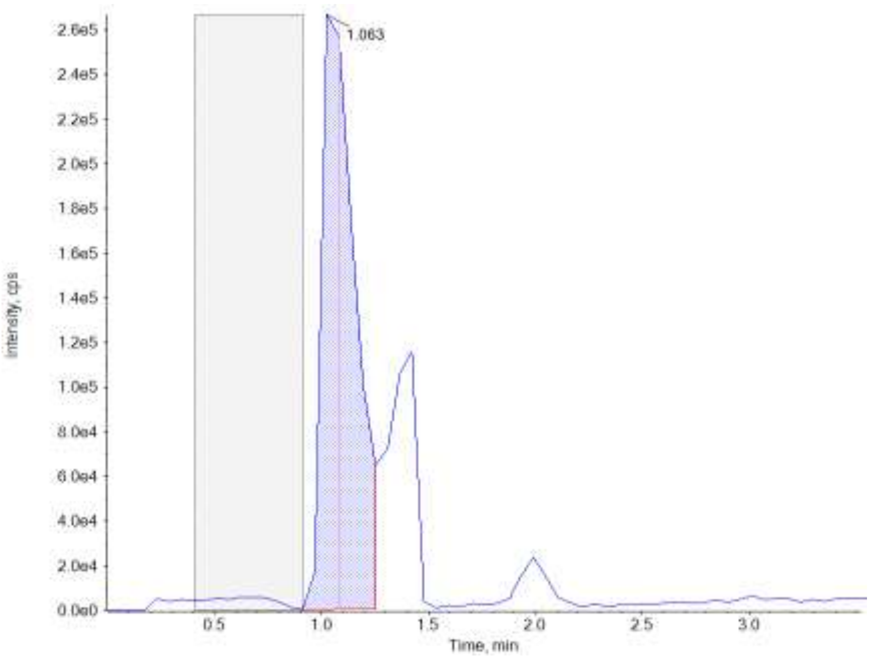

Acquired / Library MSMS

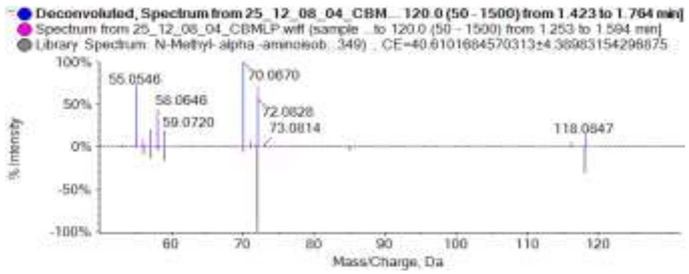

Acquired / Theoretical MS

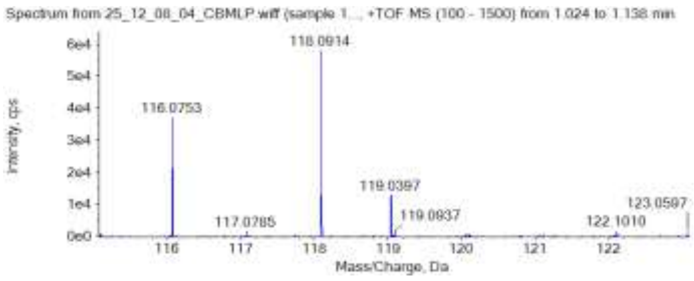

248.1179 / 1.02 (Mass/FragMass/RT/Isotope/Library/Formula/Ion Ratio)

Retention Time: 1.10 minutes
Precursor m/z : 248.1179
Fit (%) 75.1% **RFit (%)** 88.8%

Exp RT: 1.02 minutes
Analyte Name:
248.1179 / 1.02

Collision Energy = 35 ± 15 eV

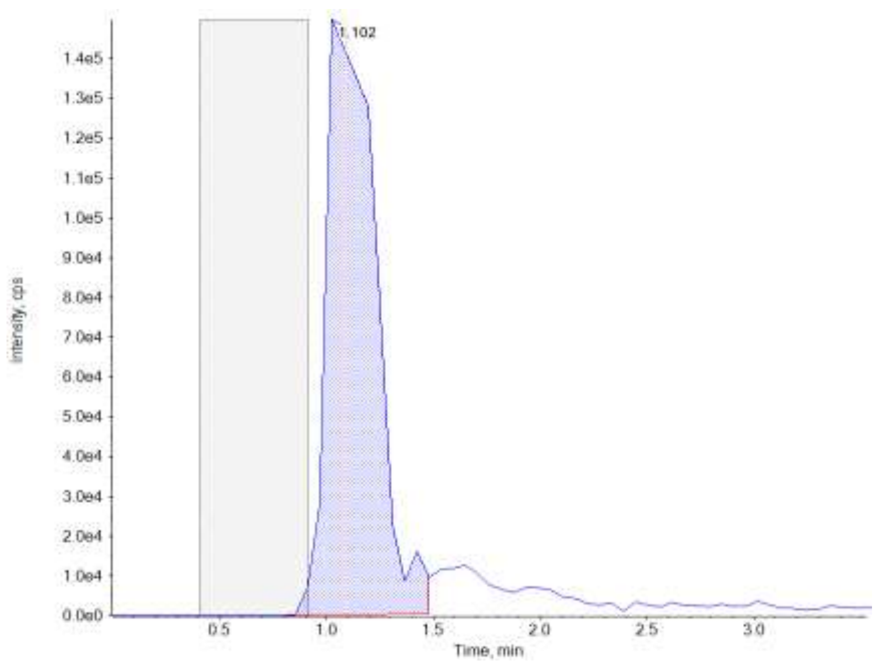

Acquired / Library MSMS

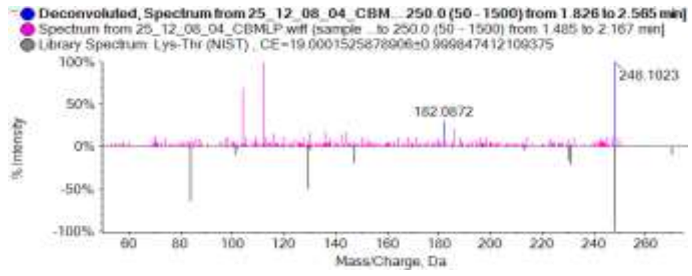

Acquired / Theoretical MS

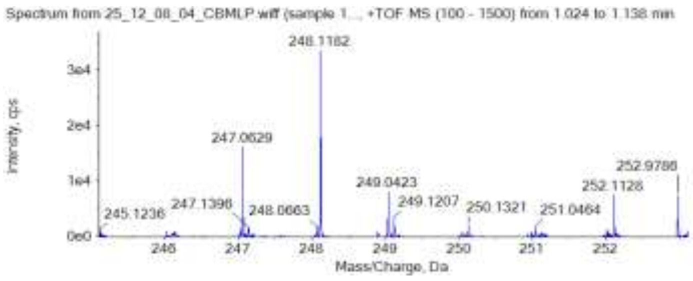

136.0704 / 1.08 (Mass/FragMass/RT/Isotope/Library/Formula/Ion Ratio)

**Retention Time:** 1.06 minutes	**Exp RT:** 1.08 minutes
**Precursor m/z :** 136.0704	**Analyte Name:**
**Fit (%)** 99.4% **RFit (%)** 59.3%	136.0704 / 1.08

Collision Energy = 35 ± 15 eV

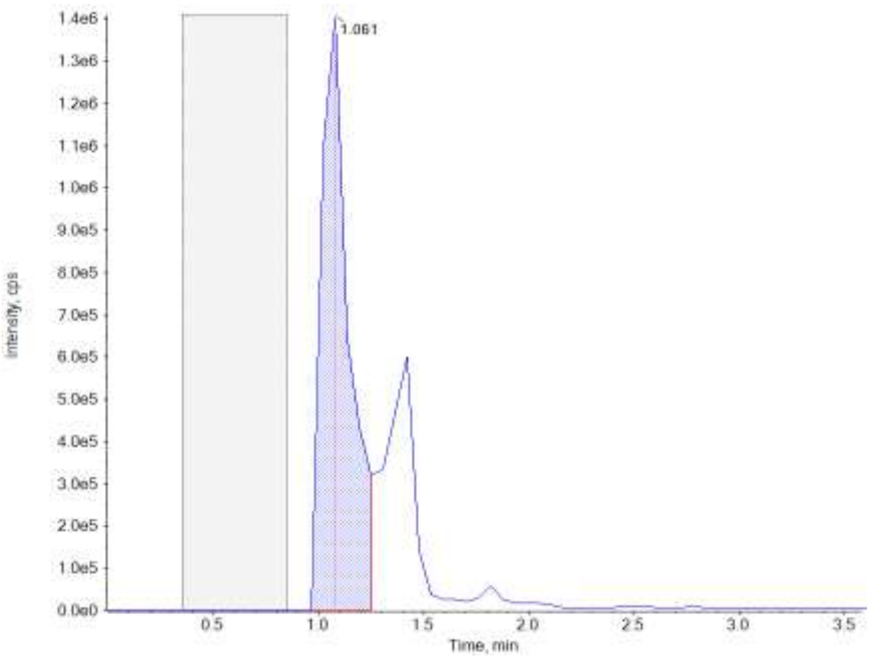

Acquired / Library MSMS

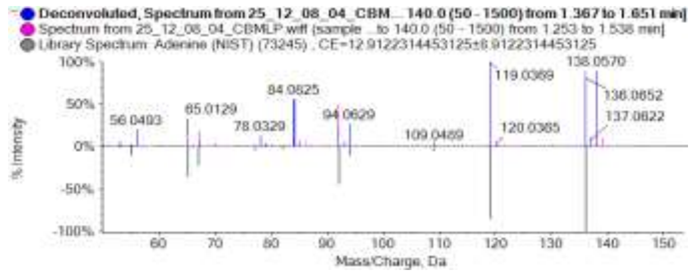

Acquired / Theoretical MS

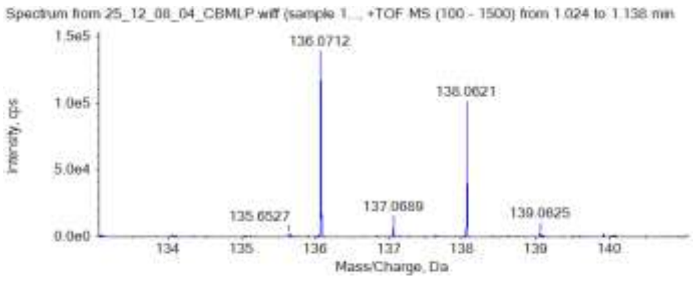

Created with SCIEX OS 3.3.0
Printed:12/9/2025 2:04:37 PM

381.0885 / 1.08 (Mass/FragMass/RT/Isotope/Library/Formula/Ion Ratio)

Retention Time: 1.12 minutes
Precursor m/z : 381.0885
Fit (%) 95.0% **RFit (%)** 100.0%

Exp RT: 1.08 minutes
Analyte Name:
381.0885 / 1.08

Collision Energy = 35 ± 15 eV

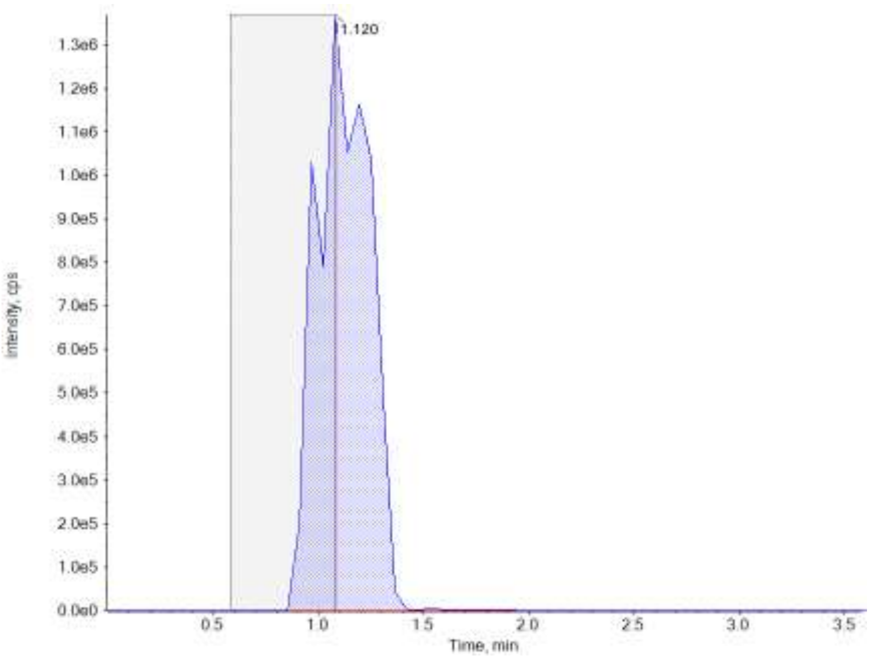

Acquired / Library MSMS

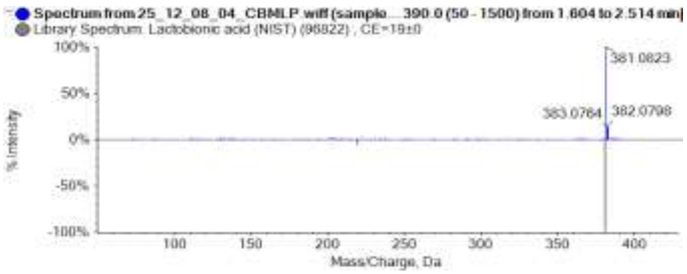

Acquired / Theoretical MS

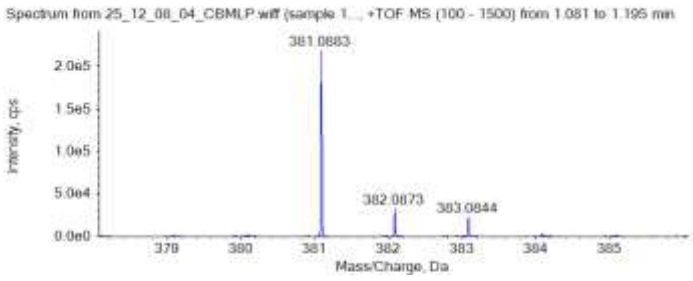

337.0947 / 1.37 (Mass/FragMass/RT/Isotope/Library/Formula/Ion Ratio)

**Retention Time:** 1.31 minutes	**Exp RT:** 1.37 minutes
**Precursor m/z :** 337.0947	**Analyte Name:**
**Fit (%)** 100.0% **RFit (%)** 100.0%	337.0947 / 1.37

Collision Energy = 35 ± 15 eV

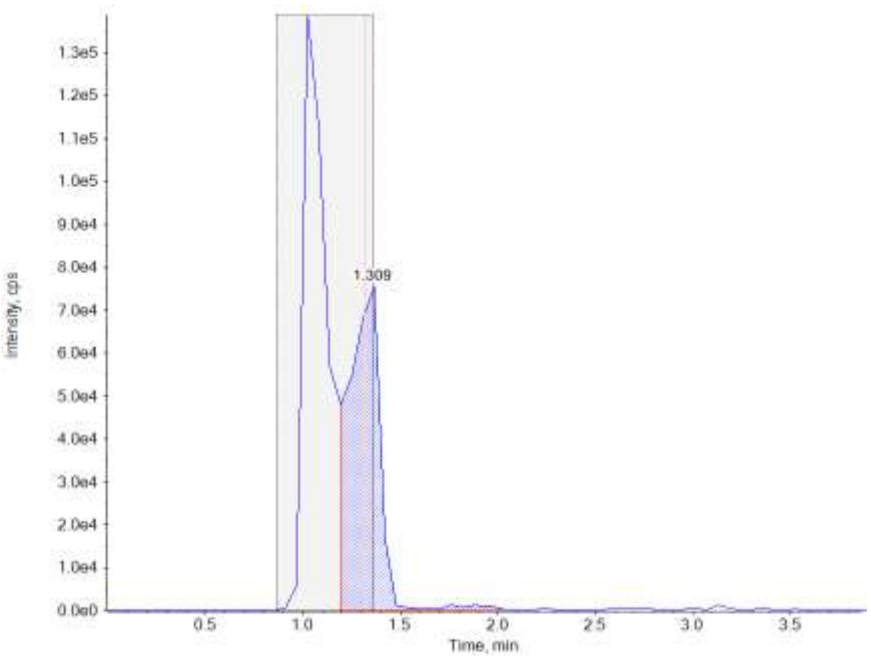

Acquired / Library MSMS

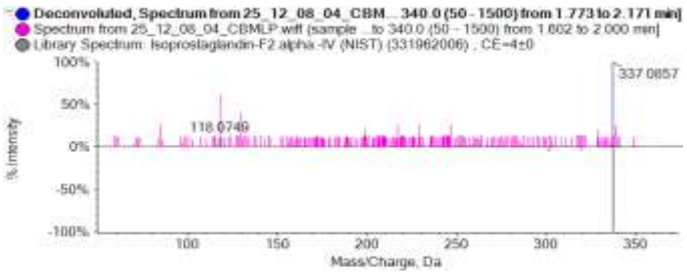

Acquired / Theoretical MS

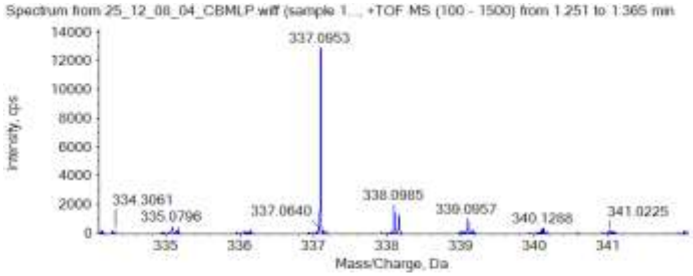

152.0648 / 1.48 (Mass/FragMass/RT/Isotope/Library/Formula/Ion Ratio)

Retention Time: 1.49 minutes **Exp RT:** 1.48 minutes
Precursor m/z : 152.0648 **Analyte Name:**
Fit (%) 100.0% **RFit (%)** 82.1% 152.0648 / 1.48

Collision Energy = 35 ± 15 eV

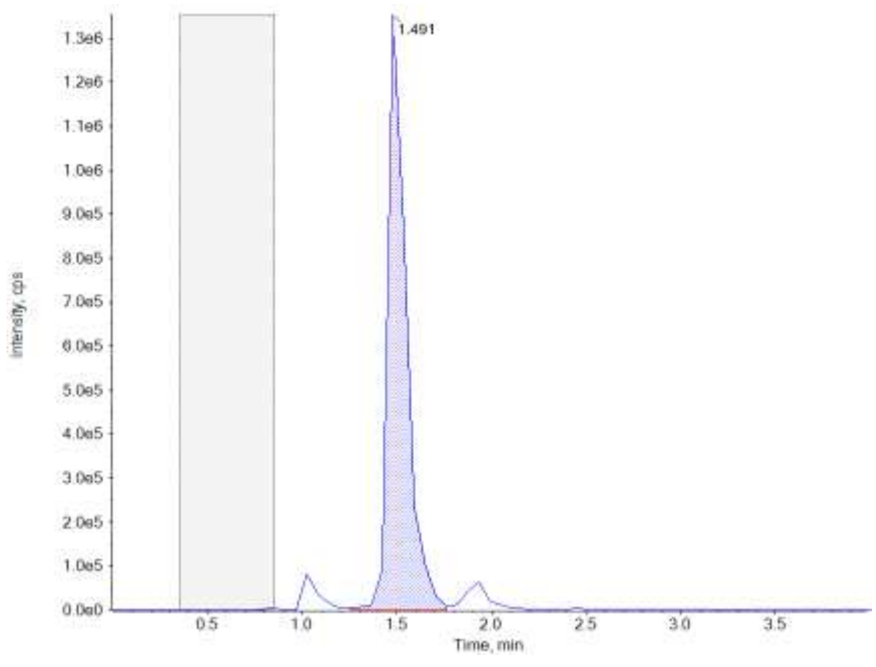

Acquired / Library MSMS

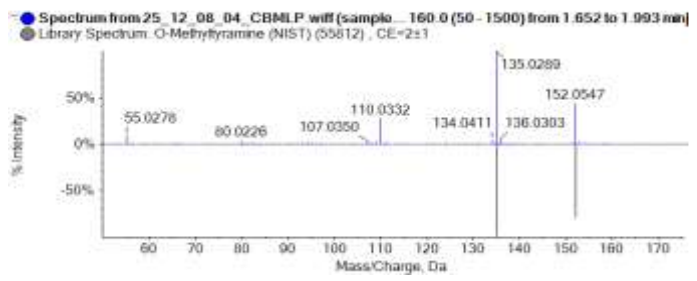

Acquired / Theoretical MS

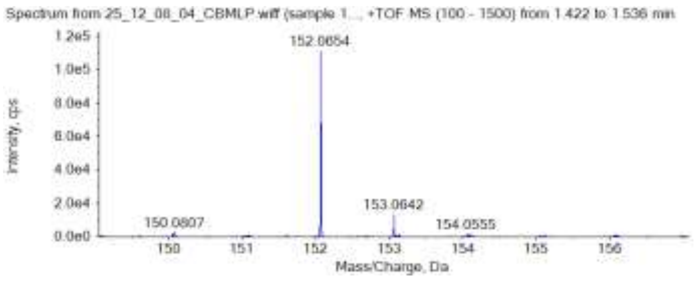

182.0868 / 1.48 (Mass/FragMass/RT/Isotope/Library/Formula/Ion Ratio)

Retention Time: 1.48 minutes
Precursor m/z : 182.0868
Fit (%) 98.5% **RFit (%)** 99.5%

Exp RT: 1.48 minutes
Analyte Name:
182.0868 / 1.48

Collision Energy = 35 ± 15 eV

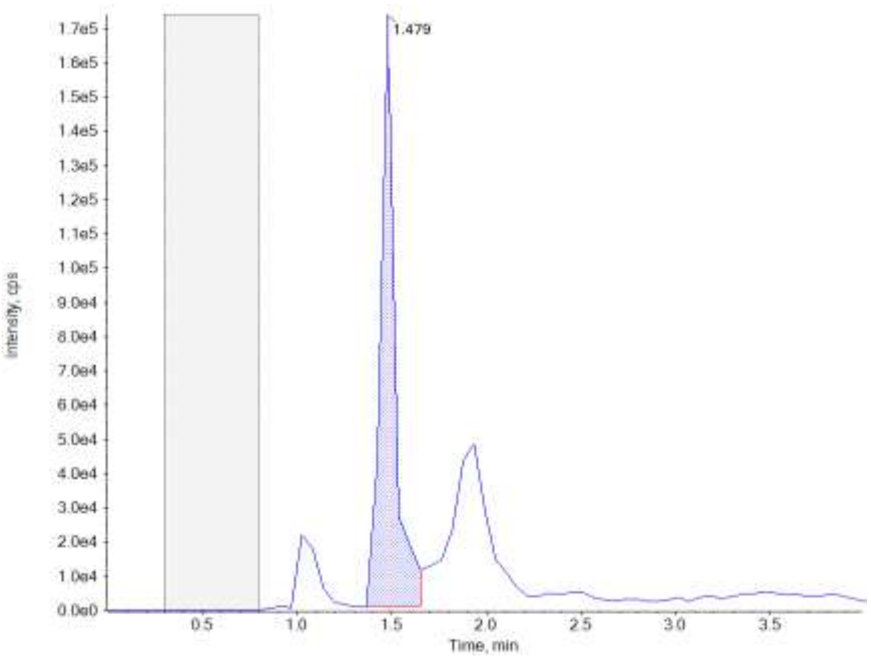

Acquired / Library MSMS

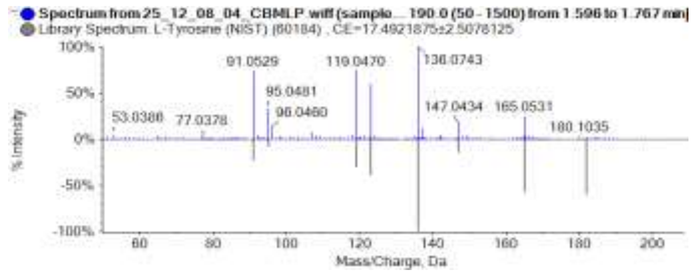

Acquired / Theoretical MS

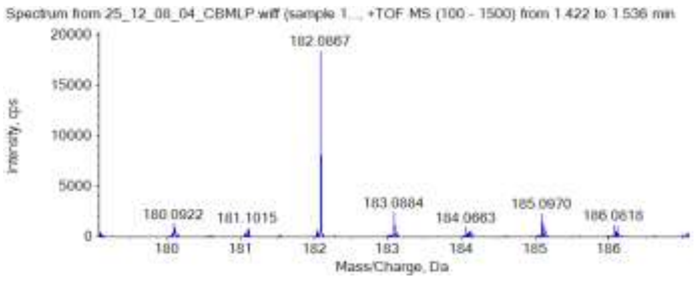

284.1075 / 1.48 (Mass/FragMass/RT/Isotope/Library/Formula/Ion Ratio)

**Retention Time:** 1.49 minutes	**Exp RT:** 1.48 minutes
**Precursor m/z :** 284.1075	**Analyte Name:**
**Fit (%)** 100.0% **RFit (%)** 100.0%	284.1075 / 1.48

Collision Energy = 35 ± 15 eV

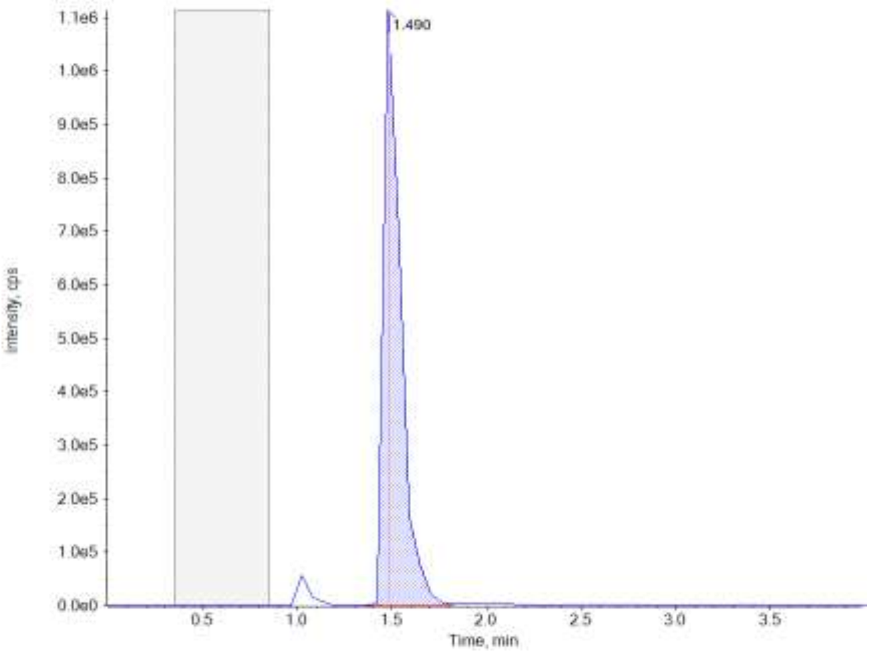

Acquired / Library MSMS

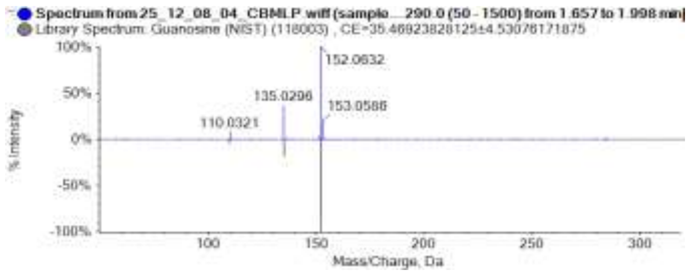

Acquired / Theoretical MS

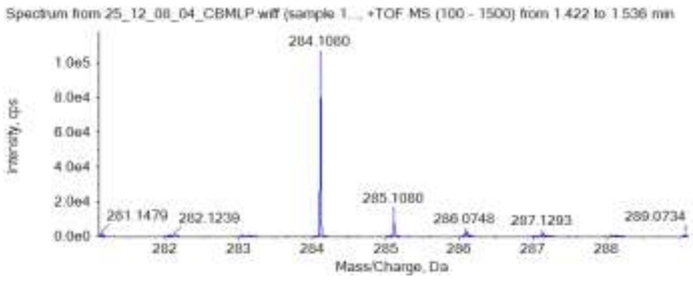

276.1521 / 1.54 (Mass/FragMass/RT/Isotope/Library/Formula/Ion Ratio)

**Retention Time:** 1.53 minutes	**Exp RT:** 1.54 minutes
**Precursor m/z :** 276.1521	**Analyte Name:**
**Fit (%)** 97.0% **RFit (%)** 72.0%	276.1521 / 1.54

Collision Energy = 35 ± 15 eV

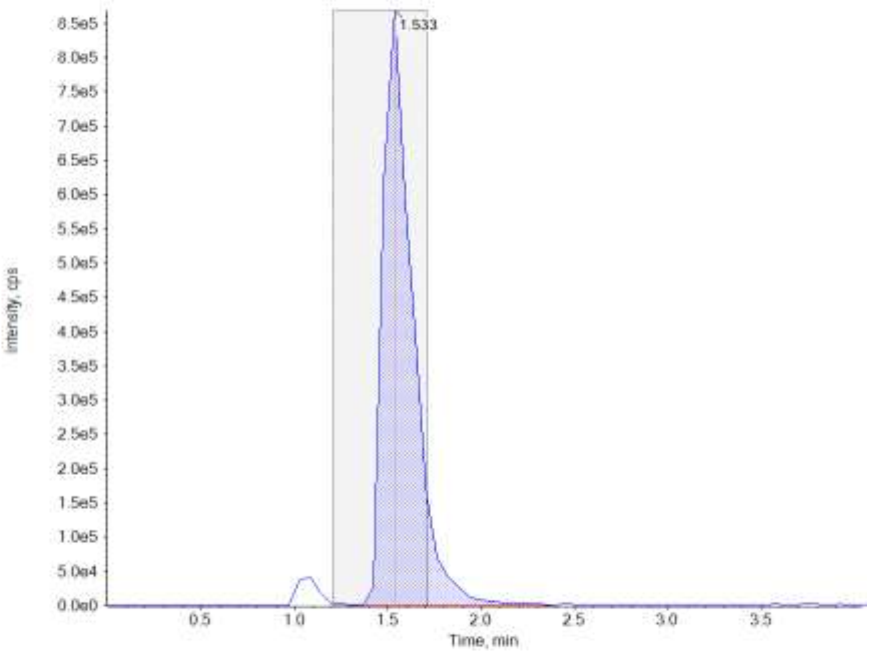

Acquired / Library MSMS

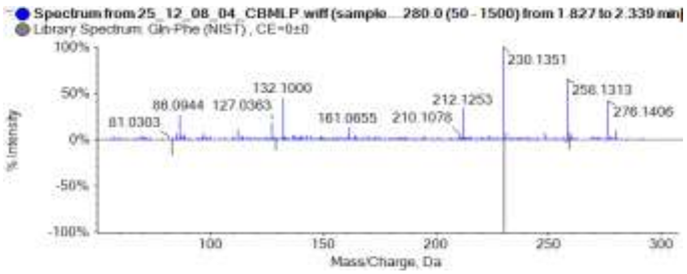

Acquired / Theoretical MS

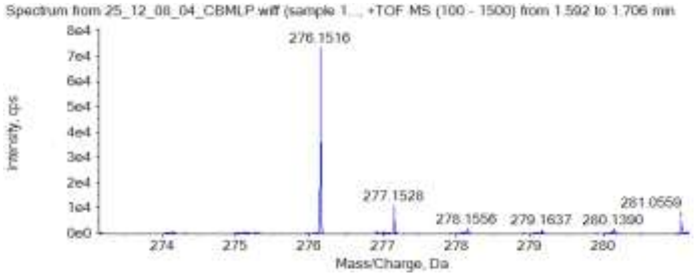

132.1101 / 1.59 (Mass/FragMass/RT/Isotope/Library/Formula/Ion Ratio)

Retention Time: 1.57 minutes
Precursor m/z : 132.1101
Fit (%) 100.0% **RFit (%)** 100.0%

Exp RT: 1.59 minutes
Analyte Name:
132.1101 / 1.59

Collision Energy = 35 ± 15 eV

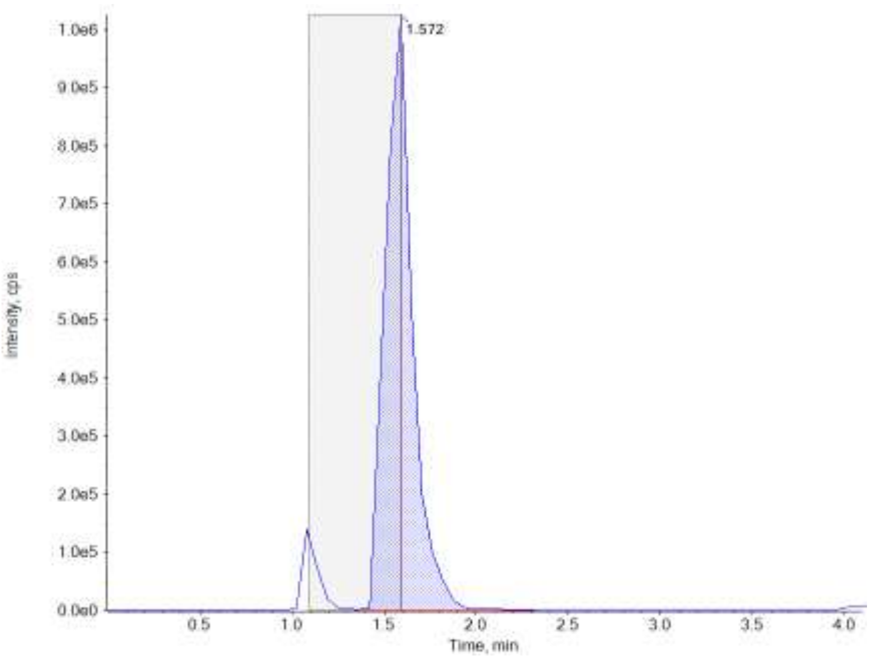

Acquired / Library MSMS

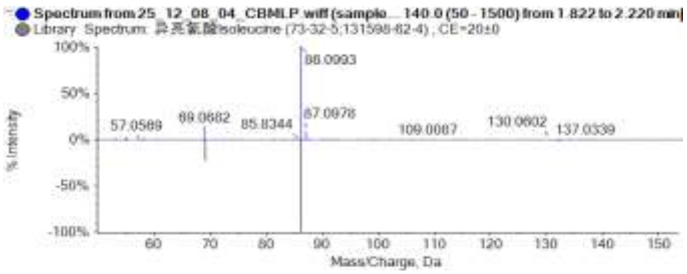

Acquired / Theoretical MS

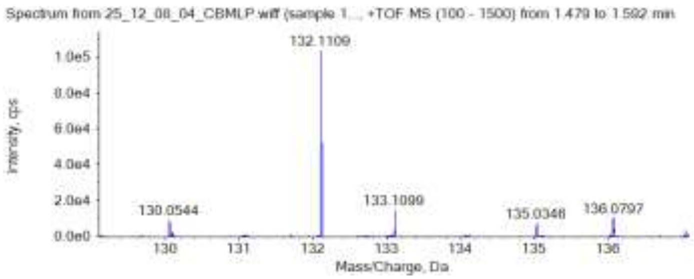

324.1009 / 1.82 [M+K]+

(Mass/FragMass/RT/Isotope/Library/Formula/Ion Ratio)

Retention Time: 1.78 minutes
Precursor m/z : 324.1009
Fit (%) 98.9% **RFit (%)** 99.7%

Exp RT: 1.82 minutes
Analyte Name:
324.1009 / 1.82 [M+K]+

Collision Energy = 35 ± 15 eV

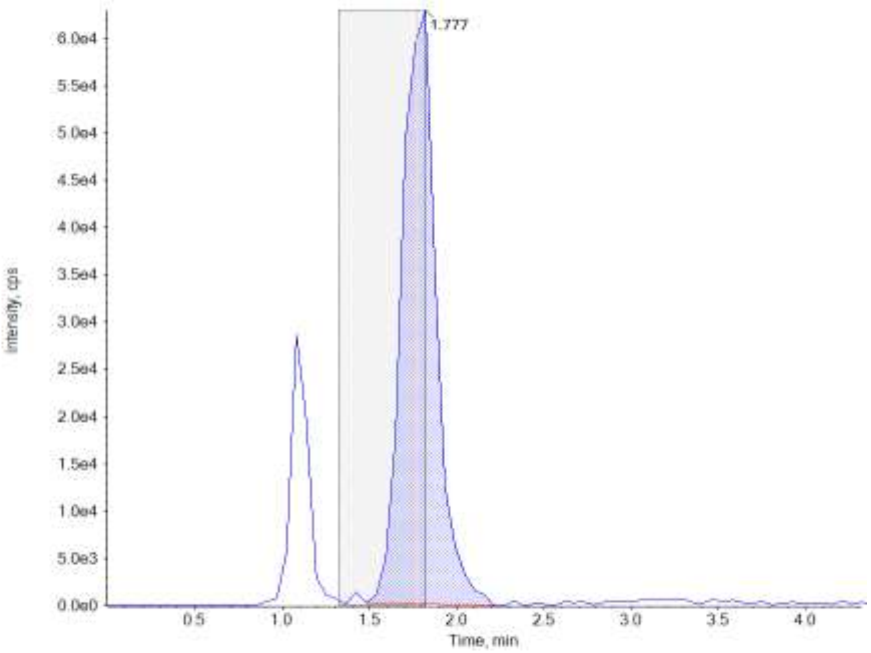

Acquired / Library MSMS

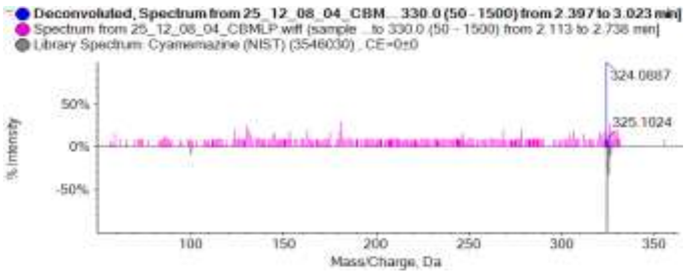

Acquired / Theoretical MS

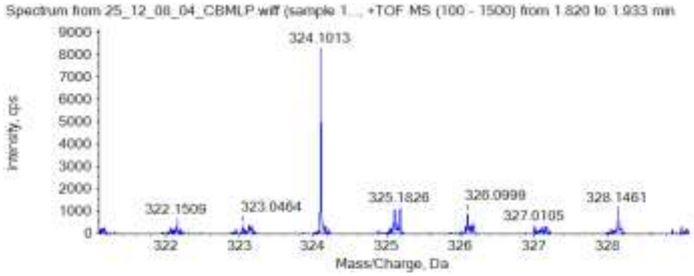

282.1271 / 1.82 (Mass/FragMass/RT/Isotope/Library/Formula/Ion Ratio)

**Retention Time:** 1.82 minutes	**Exp RT:** 1.82 minutes
**Precursor m/z :** 282.1271	**Analyte Name:**
**Fit (%)** 96.8% **RFit (%)** 100.0%	282.1271 / 1.82

Collision Energy = 35 ± 15 eV

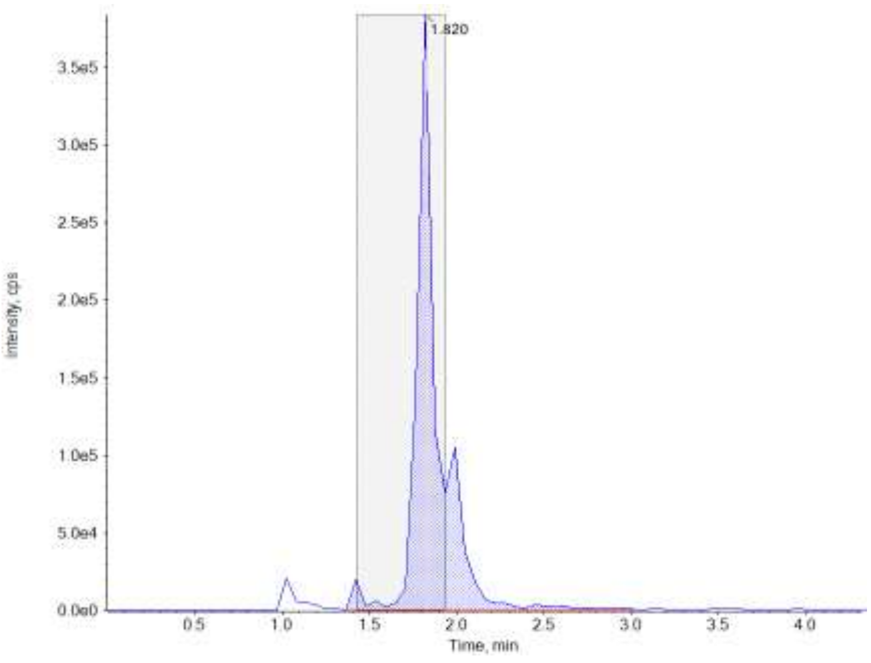

Acquired / Library MSMS

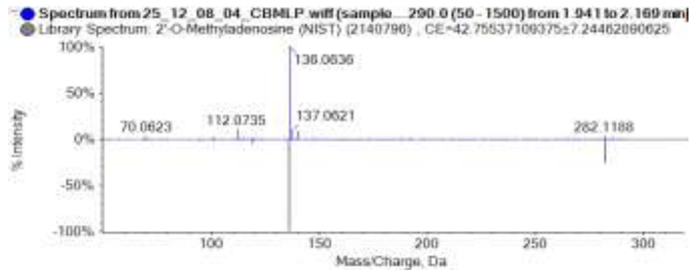

Acquired / Theoretical MS

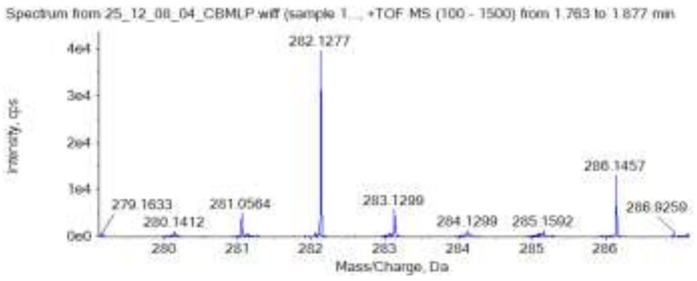

182.0974 / 1.93 [M+H]+ (Mass/FragMass/RT/Isotope/Library/Formula/Ion Ratio)

**Retention Time:** 1.92 minutes	**Exp RT:** 1.93 minutes
**Precursor m/z :** 182.0974	**Analyte Name:**
**Fit (%)** 100.0% **RFit (%)** 87.3%	182.0974 / 1.93 [M+H]+

	**Collision Energy = 35 ± 15 eV**

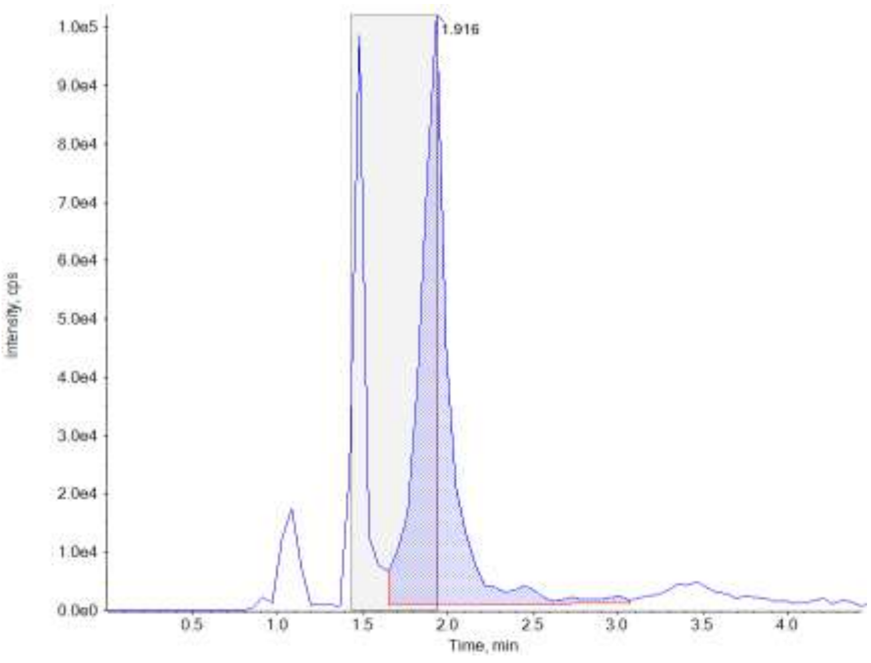

Acquired / Library MSMS

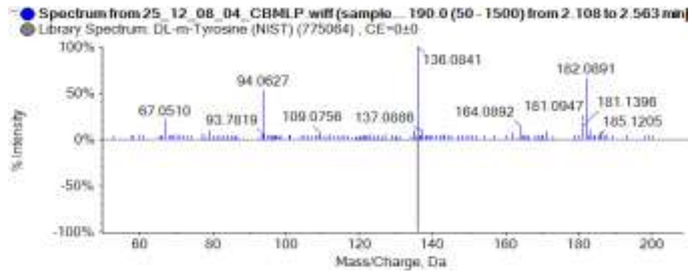

Acquired / Theoretical MS

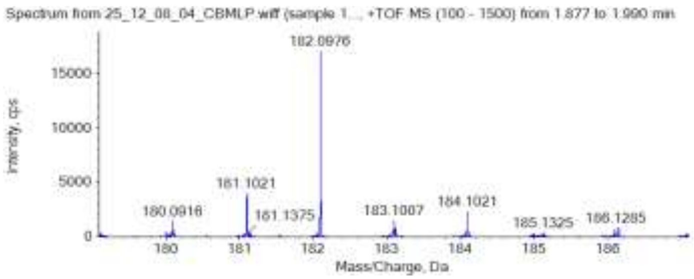

204.1281 / 1.93 (Mass/FragMass/RT/Isotope/Library/Formula/Ion Ratio)

Retention Time: 1.92 minutes **Exp RT:** 1.93 minutes
Precursor m/z : 204.1281 **Analyte Name:**
Fit (%) 100.0% **RFit (%)** 99.1% 204.1281 / 1.93

Collision Energy = 35 ± 15 eV

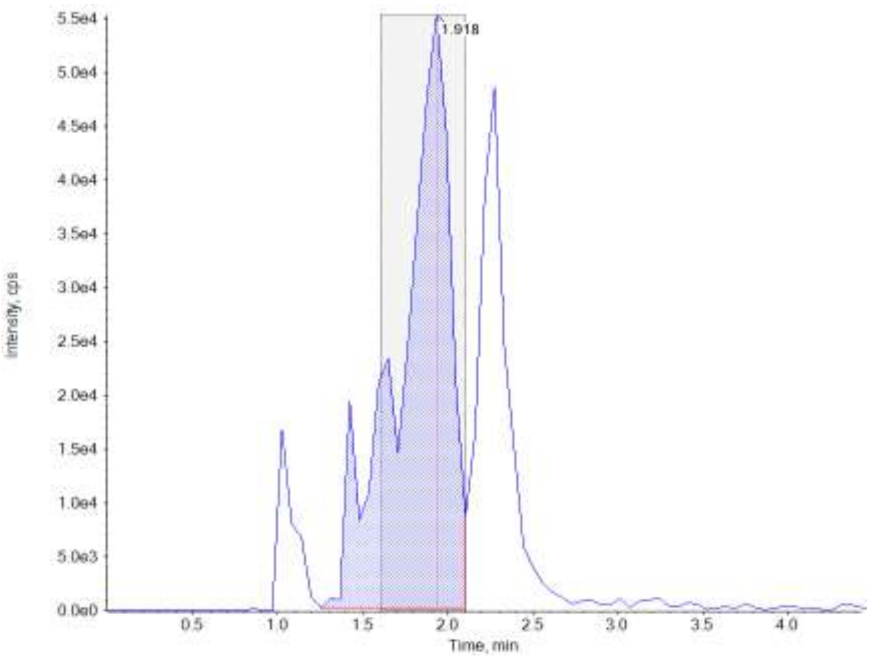

Acquired / Library MSMS

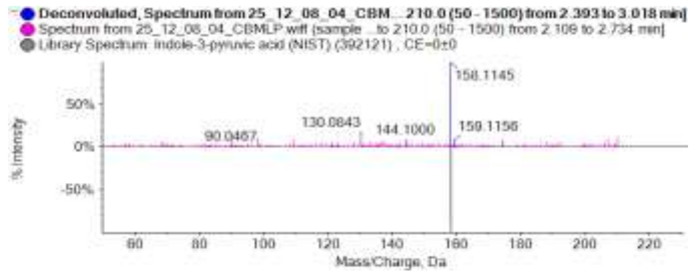

Acquired / Theoretical MS

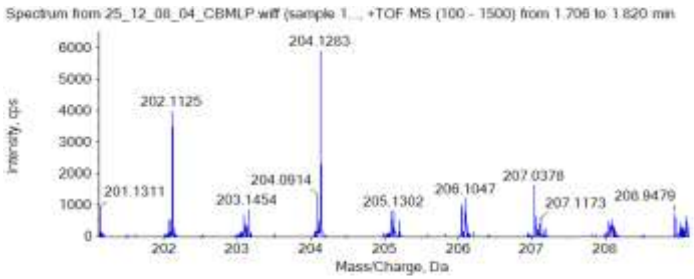

121.0900 / 2.27 (Mass/FragMass/RT/Isotope/Library/Formula/Ion Ratio)

Retention Time: 2.27 minutes
Precursor m/z : 121.0900
Fit (%) 100.0% **RFit (%)** 95.3%

Exp RT: 2.27 minutes
Analyte Name:
121.0900 / 2.27

Collision Energy = 35 ± 15 eV

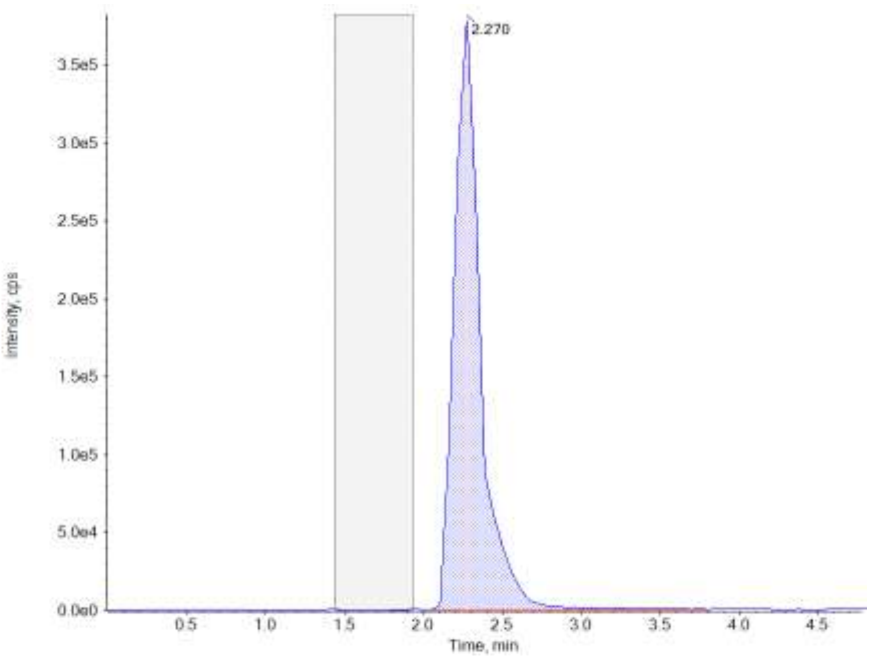

Acquired / Library MSMS

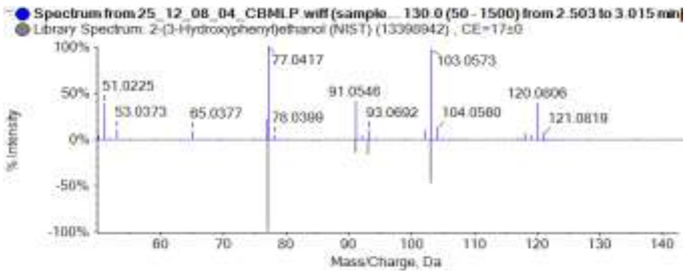

Acquired / Theoretical MS

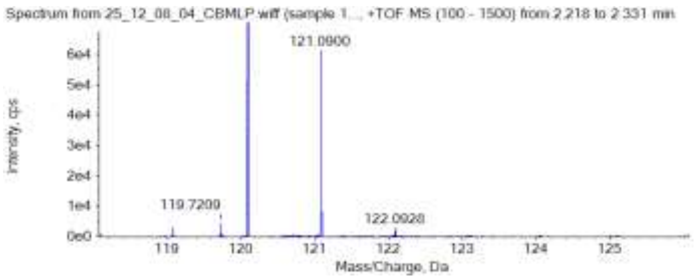

166.0972 / 2.27 (Mass/FragMass/RT/Isotope/Library/Formula/Ion Ratio)

Retention Time: 2.28 minutes
Precursor m/z : 166.0972
Fit (%) 99.8% **RFit (%)** 100.0%

Exp RT: 2.27 minutes
Analyte Name:
166.0972 / 2.27

Collision Energy = 35 ± 15 eV

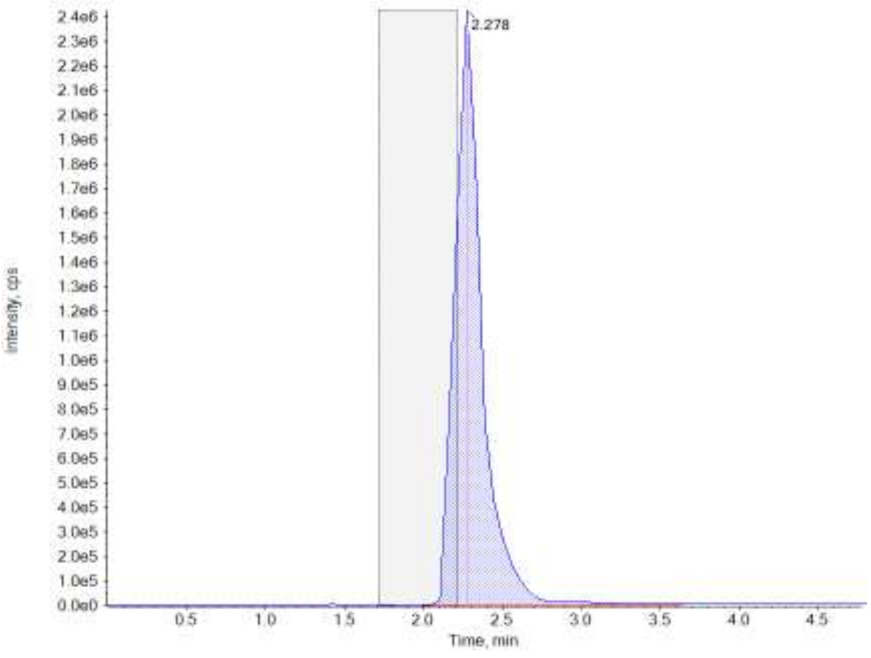

Acquired / Library MSMS

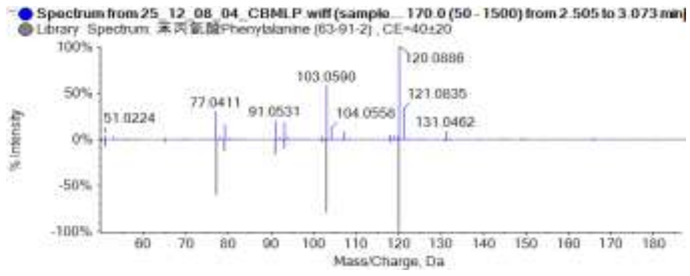

Acquired / Theoretical MS

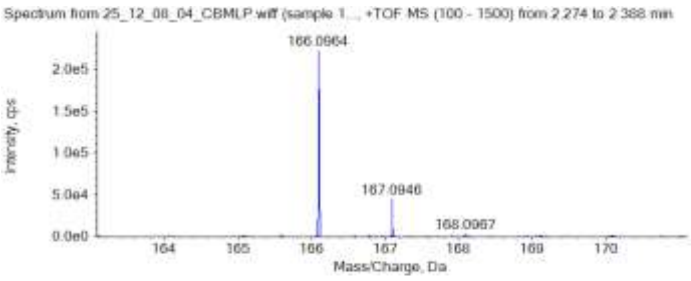

328.1478 / 2.27 (Mass/FragMass/RT/Isotope/Library/Formula/Ion Ratio)

Retention Time: 2.24 minutes **Exp RT:** 2.27 minutes
Precursor m/z : 328.1478 **Analyte Name:**
Fit (%) 100.0% **RFit (%)** 46.1% 328.1478 / 2.27

Collision Energy = 35 ± 15 eV

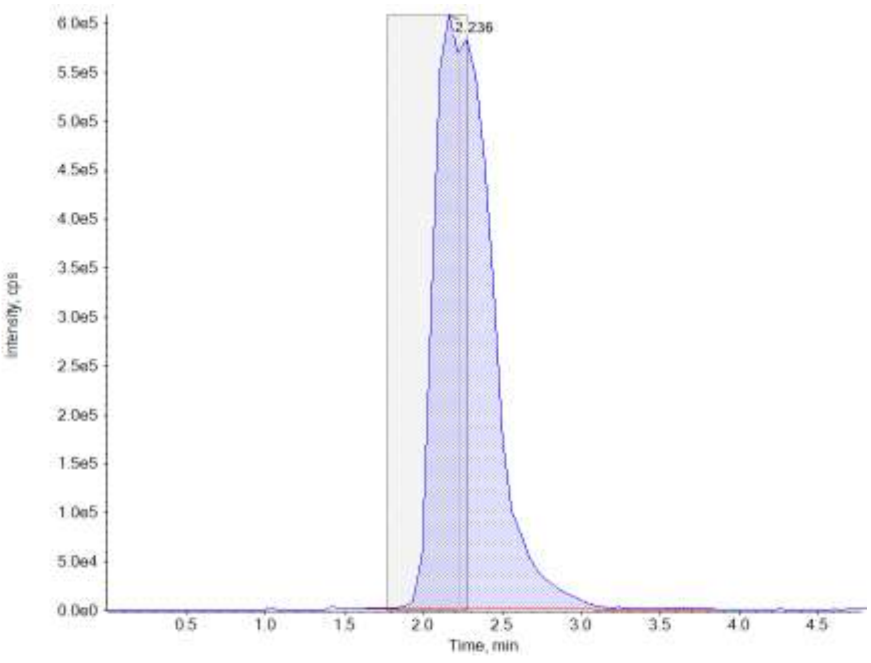

Acquired / Library MSMS

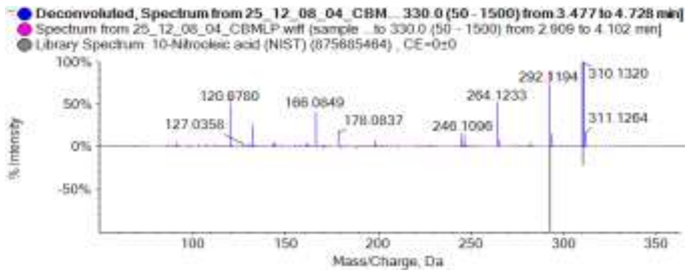

Acquired / Theoretical MS

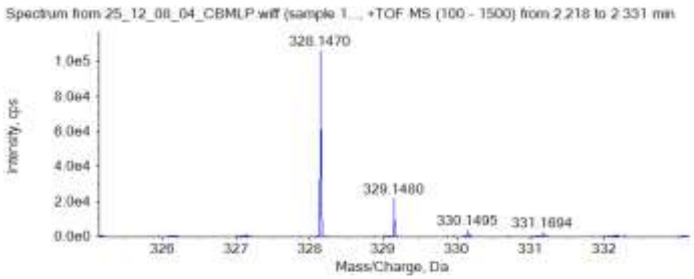

120.0928 / 2.33 (Mass/FragMass/RT/Isotope/Library/Formula/Ion Ratio)

Retention Time: 2.30 minutes
Precursor m/z : 120.0928
Fit (%) 96.2% **RFit (%)** 82.8%

Exp RT: 2.33 minutes
Analyte Name:
120.0928 / 2.33

Collision Energy = 35 ± 15 eV

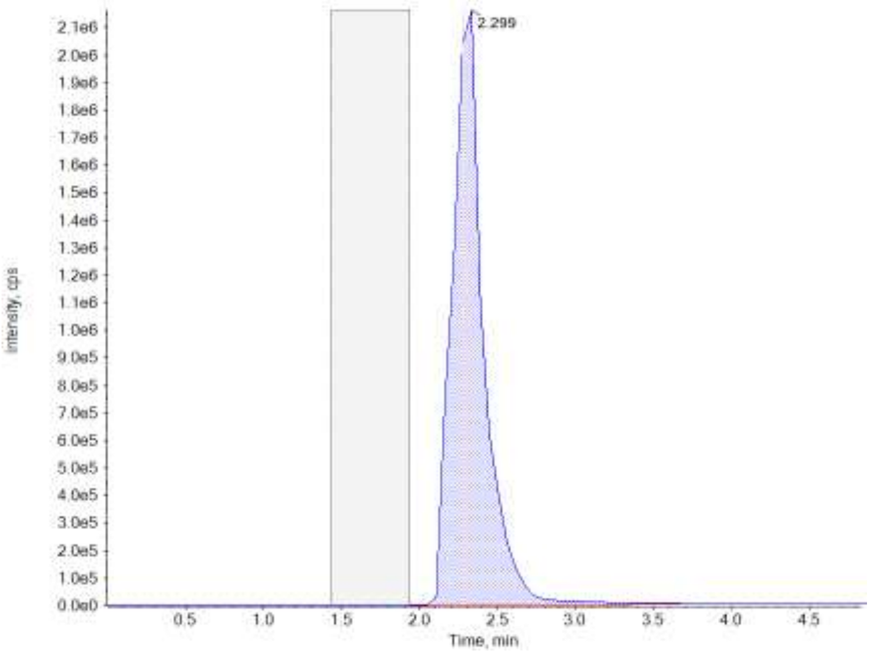

Acquired / Library MSMS

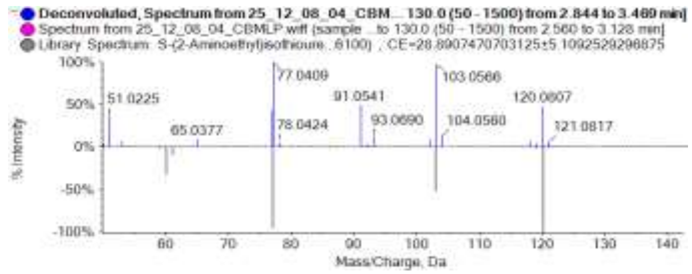

Acquired / Theoretical MS

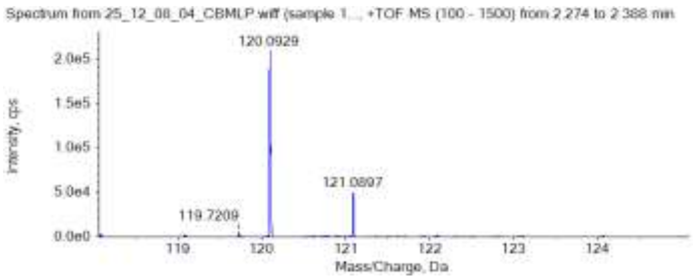

220.1231 / 2.44 (Mass/FragMass/RT/Isotope/Library/Formula/Ion Ratio)

Retention Time: 2.45 minutes
Precursor m/z : 220.1231
Fit (%) 99.5% **RFit (%)** 88.7%

Exp RT: 2.45 minutes
Analyte Name:
220.1231 / 2.44

Collision Energy = 35 ± 15 eV

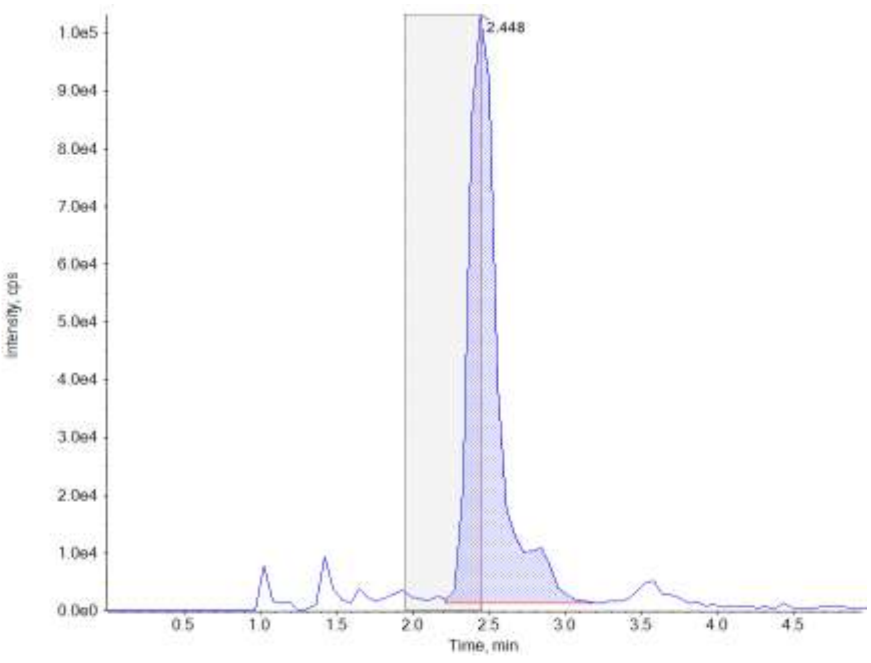

Acquired / Library MSMS

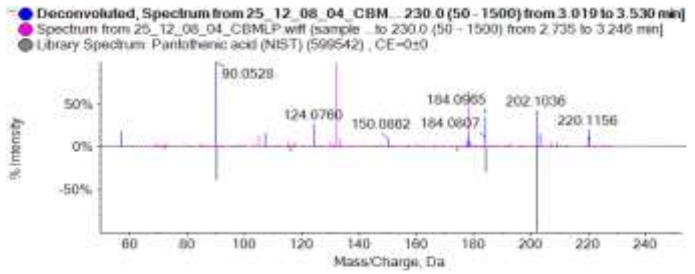

Acquired / Theoretical MS

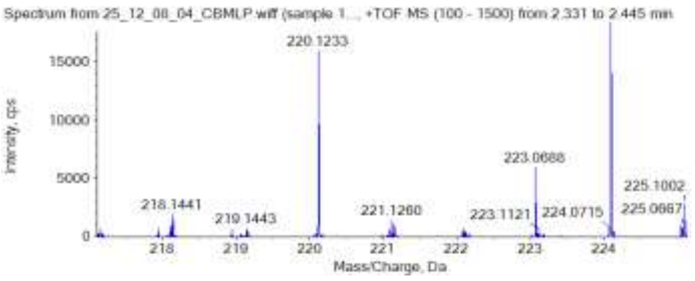

Created with SCIEX OS 3.3.0
Printed:12/9/2025 2:04:37 PM

158.0863 / 2.62 (Mass/FragMass/RT/Isotope/Library/Formula/Ion Ratio)

Retention Time: 2.52 minutes **Exp RT:** 2.62 minutes
Precursor m/z : 158.0862 **Analyte Name:**
Fit (%) 24.8% **RFit (%)** 97.4% 158.0863 / 2.62

Collision Energy = 35 ± 15 eV

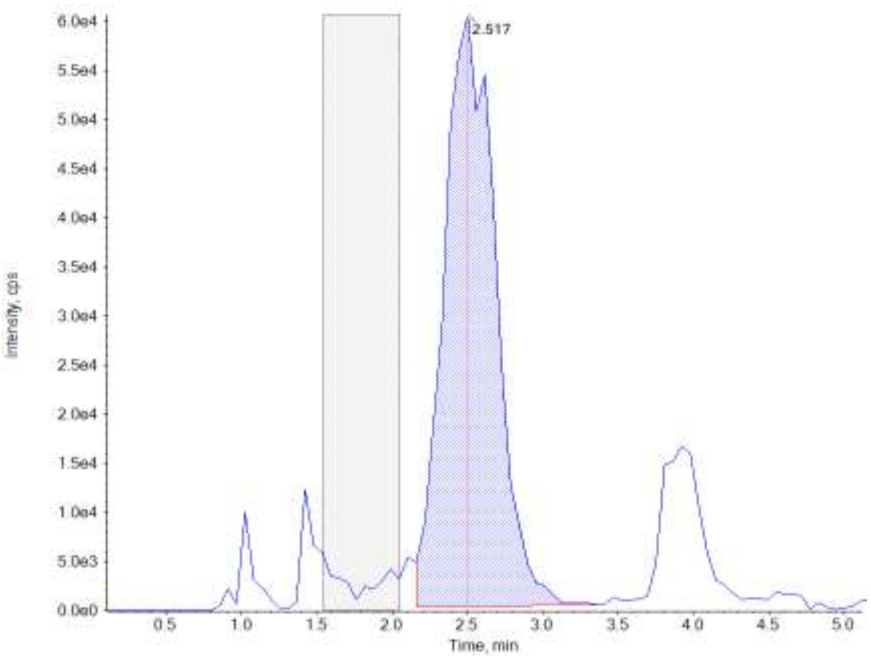

Acquired / Library MSMS

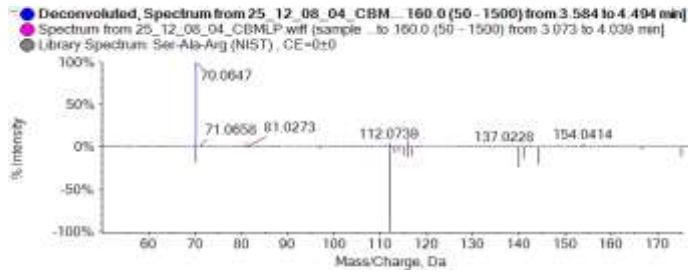

Acquired / Theoretical MS

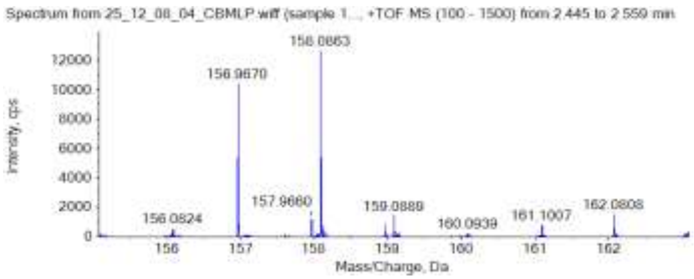

186.1285 / 2.67 (Mass/FragMass/RT/Isotope/Library/Formula/Ion Ratio)

Retention Time: 2.68 minutes
Precursor m/z : 186.1285
Fit (%) 57.5% **RFit (%)** 99.7%

Exp RT: 2.67 minutes
Analyte Name:
186.1285 / 2.67

Collision Energy = 35 ± 15 eV

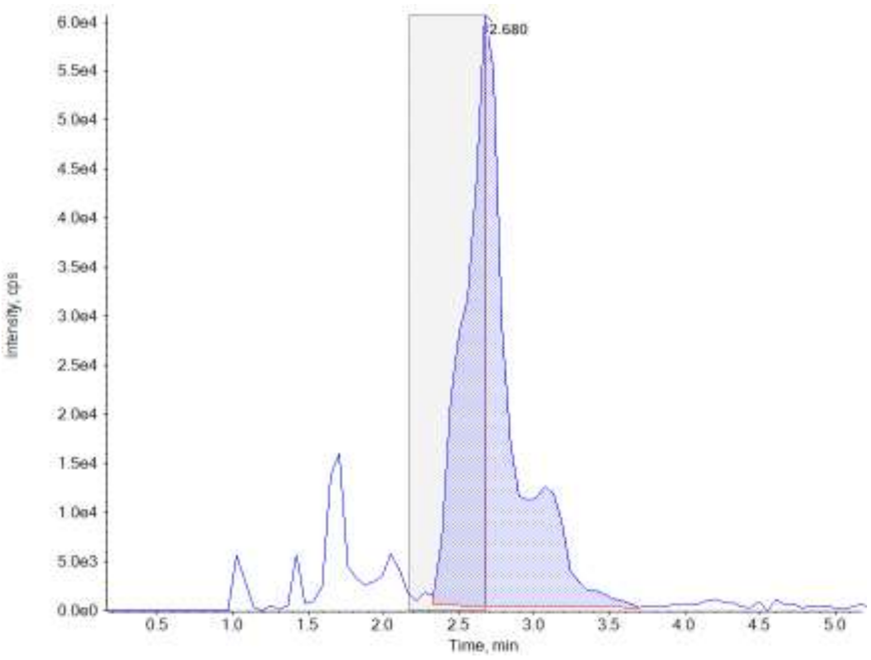

Acquired / Library MSMS

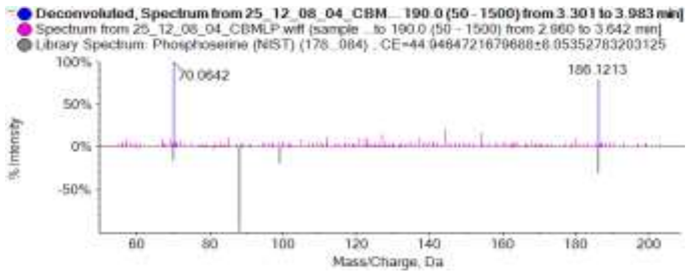

Acquired / Theoretical MS

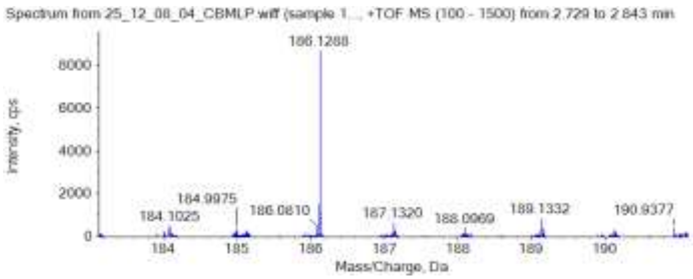

384.1200 / 2.73 (Mass/FragMass/RT/Isotope/Library/Formula/Ion Ratio)

Retention Time: 2.73 minutes
Precursor m/z : 384.1200
Fit (%) 77.2% **RFit (%)** 99.3%

Exp RT: 2.73 minutes
Analyte Name:
384.1200 / 2.73

Collision Energy = 35 ± 15 eV

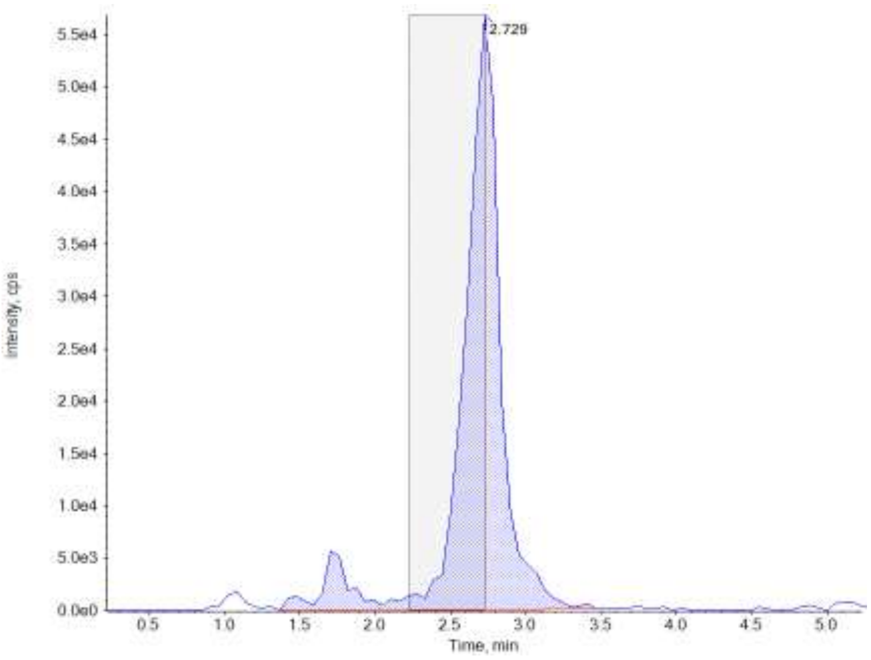

Acquired / Library MSMS

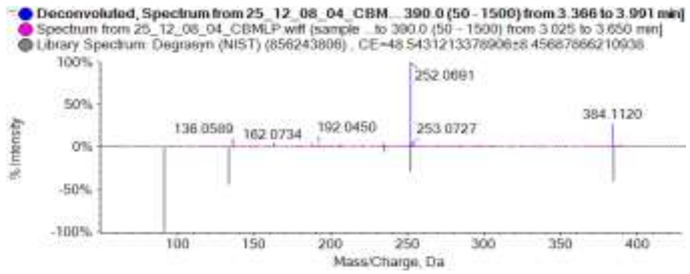

Acquired / Theoretical MS

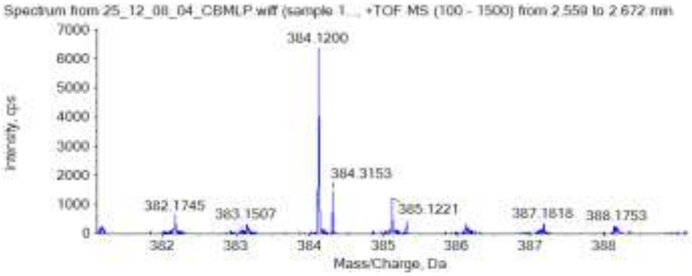

156.9668 / 3.52 (Mass/FragMass/RT/Isotope/Library/Formula/Ion Ratio)

Retention Time: 3.05 minutes **Exp RT:** 3.53 minutes
Precursor m/z : 156.9668 **Analyte Name:**
Fit (%) 96.3% **RFit (%)** 42.4% 156.9668 / 3.52

Collision Energy = 35 ± 15 eV

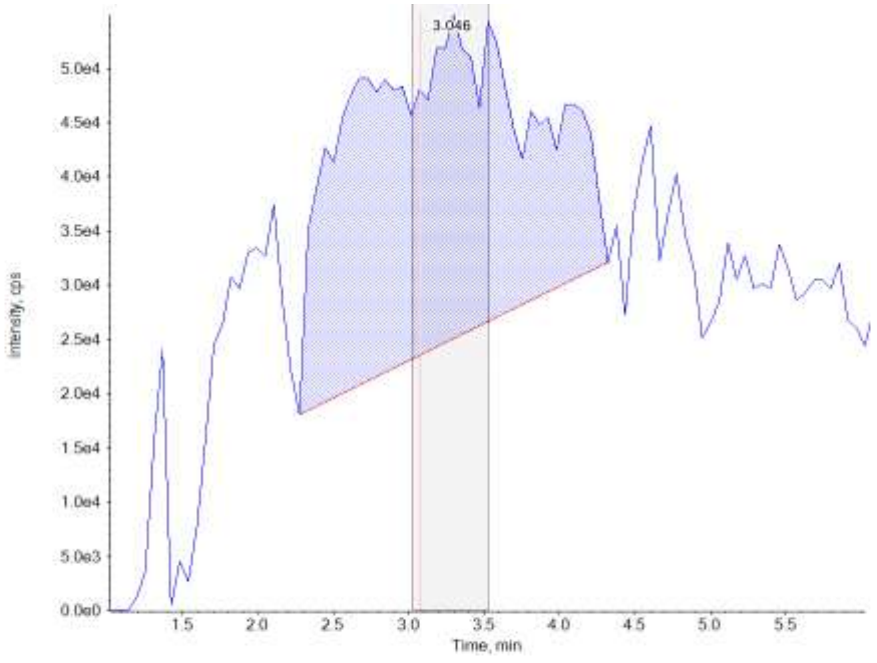

Acquired / Library MSMS

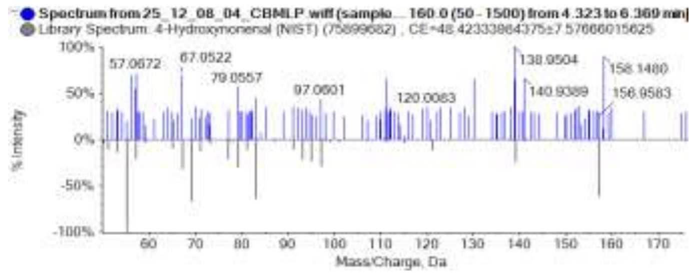

Acquired / Theoretical MS

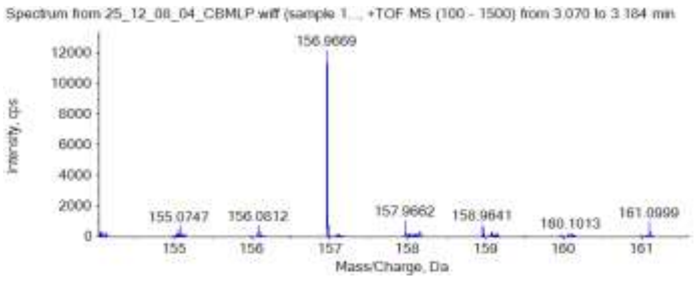

192.1069 / 4.04 (Mass/FragMass/RT/Isotope/Library/Formula/Ion Ratio)

Retention Time: 4.00 minutes
Precursor m/z : 192.1069
Fit (%) 92.4% **RFit (%)** 94.8%

Exp RT: 4.04 minutes
Analyte Name:
192.1069 / 4.04

Collision Energy = 35 ± 15 eV

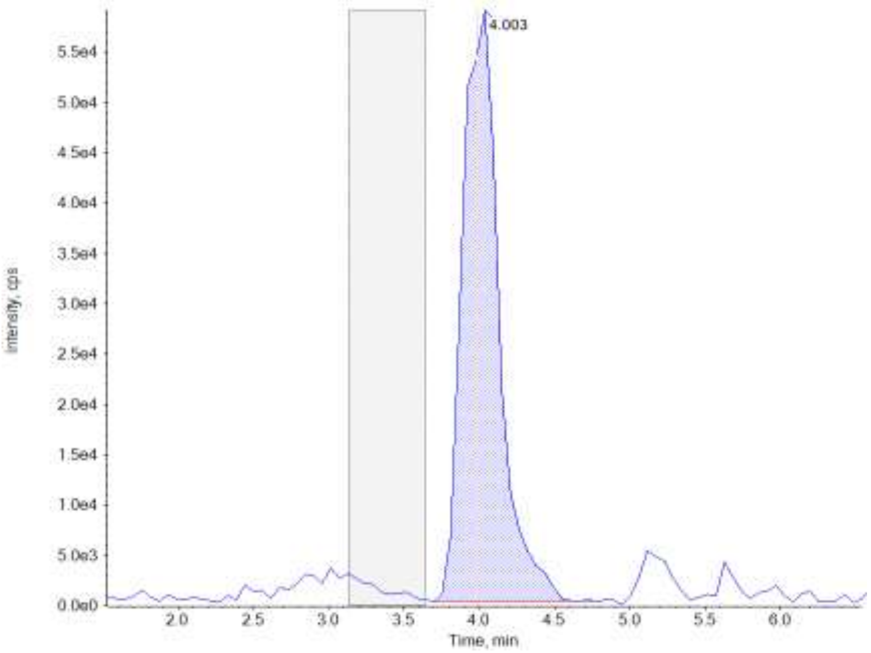

Acquired / Library MSMS

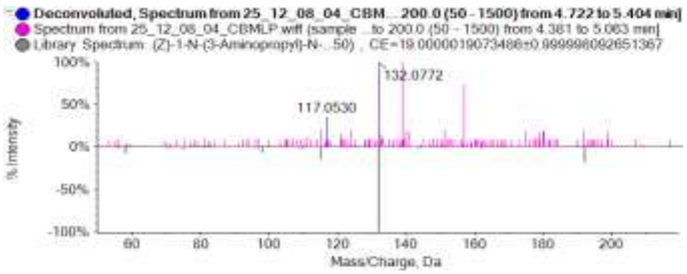

Acquired / Theoretical MS

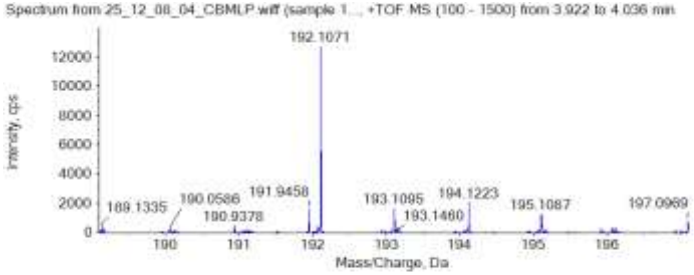

298.1027 / 4.26 (Mass/FragMass/RT/Isotope/Library/Formula/Ion Ratio)

Retention Time: 4.29 minutes **Exp RT:** 4.26 minutes
Precursor m/z : 298.1027 **Analyte Name:**
Fit (%) 100.0% **RFit (%)** 100.0% 298.1027 / 4.26

Collision Energy = 35 ± 15 eV

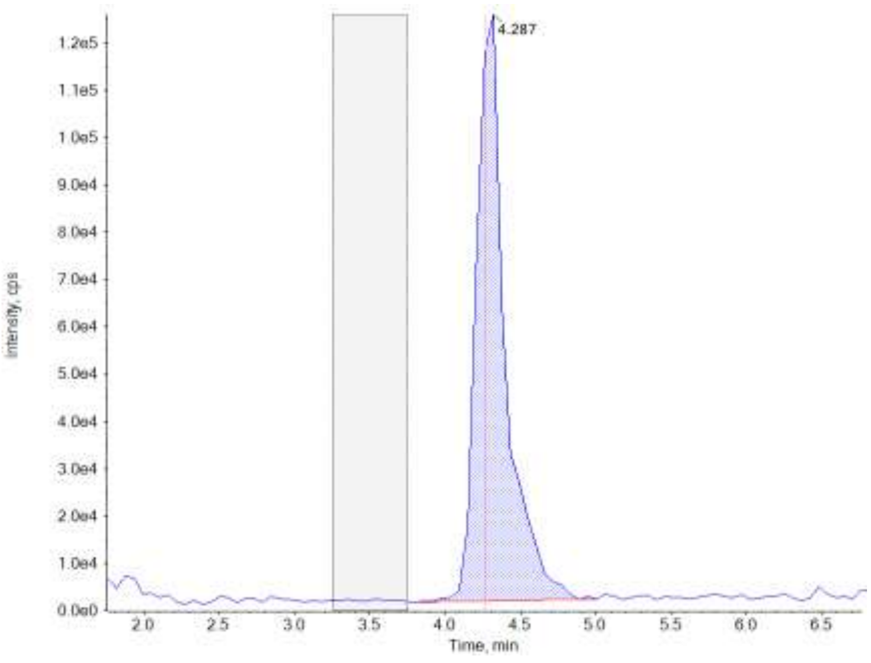

Acquired / Library MSMS

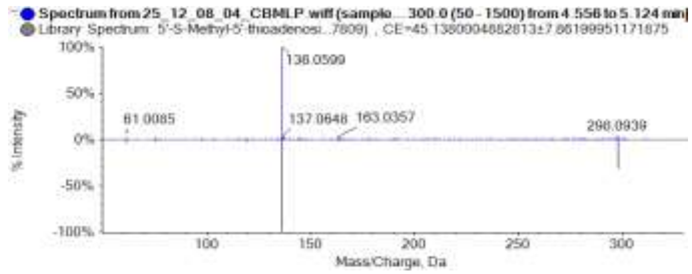

Acquired / Theoretical MS

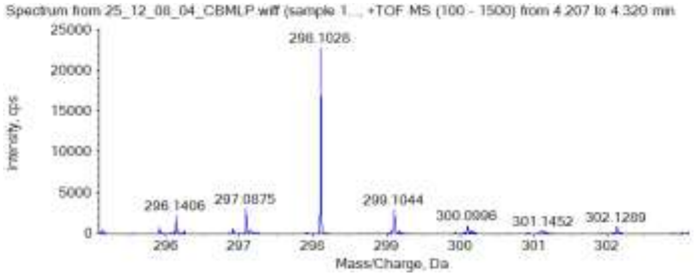

118.0697 / 4.43 (Mass/FragMass/RT/Isotope/Library/Formula/Ion Ratio)

Retention Time: 4.43 minutes
Precursor m/z : 118.0697
Fit (%) 99.5% **RFit (%)** 96.1%

Exp RT: 4.43 minutes
Analyte Name:
118.0697 / 4.43

Collision Energy = 35 ± 15 eV

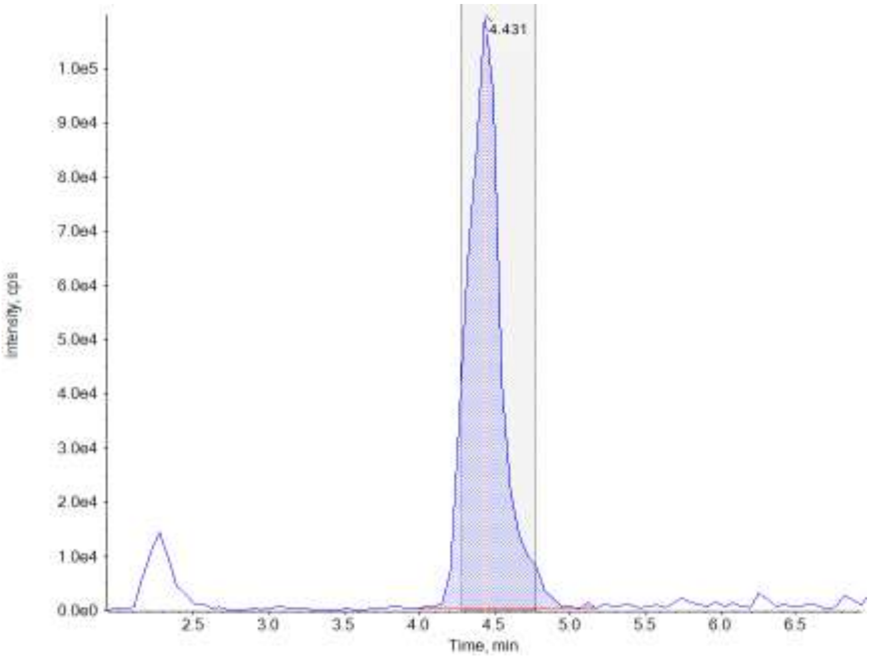

Acquired / Library MSMS

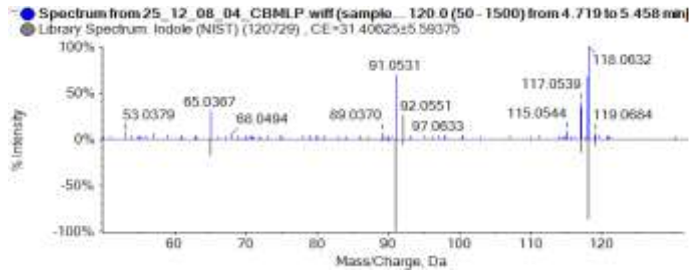

Acquired / Theoretical MS

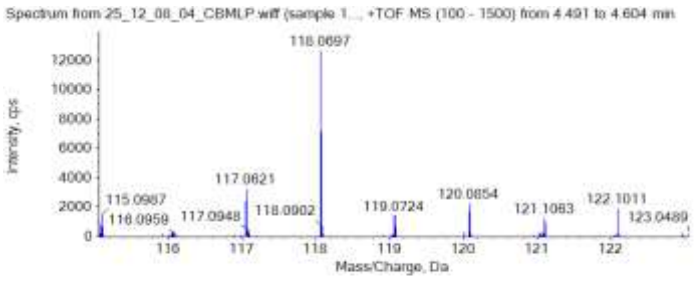

146.0653 / 4.43 (Mass/FragMass/RT/Isotope/Library/Formula/Ion Ratio)

**Retention Time:** 4.43 minutes	**Exp RT:** 4.43 minutes
**Precursor m/z :** 146.0653	**Analyte Name:**
**Fit (%)** 96.1% **RFit (%)** 73.2%	146.0653 / 4.43

Collision Energy = 35 ± 15 eV

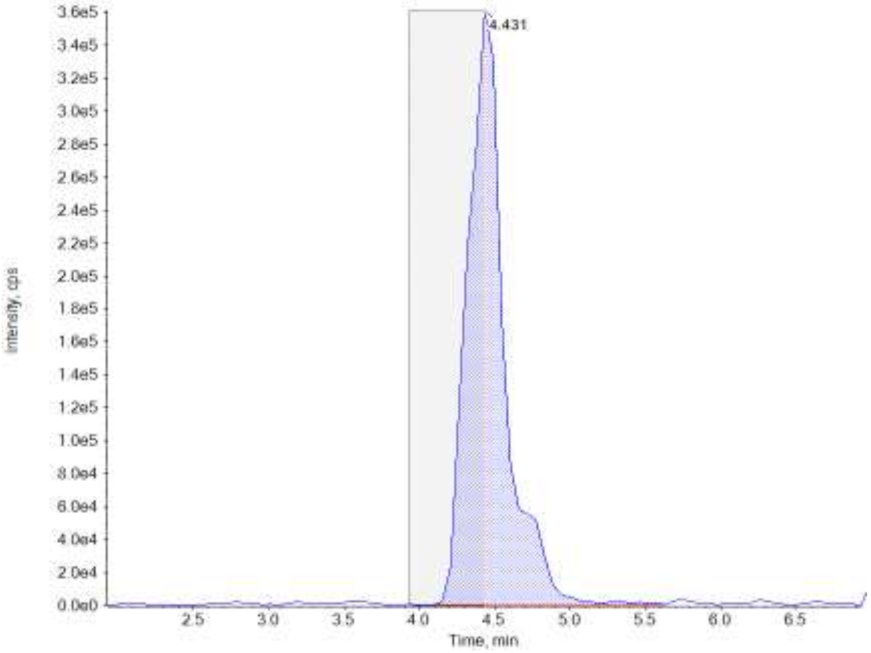

Acquired / Library MSMS

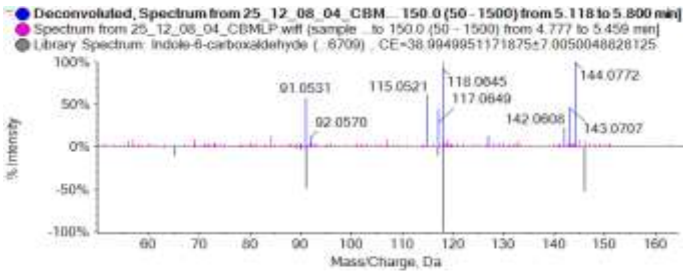

Acquired / Theoretical MS

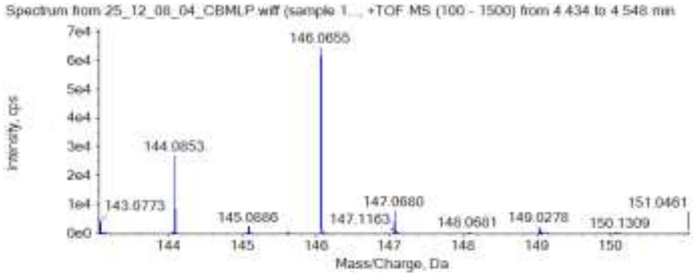

159.0964 / 4.43 (Mass/FragMass/RT/Isotope/Library/Formula/Ion Ratio)

**Retention Time:** 4.43 minutes	**Exp RT:** 4.43 minutes
**Precursor m/z :** 159.0964	**Analyte Name:**
**Fit (%)** 90.1% **RFit (%)** 98.2%	159.0964 / 4.43

Collision Energy = 35 ± 15 eV

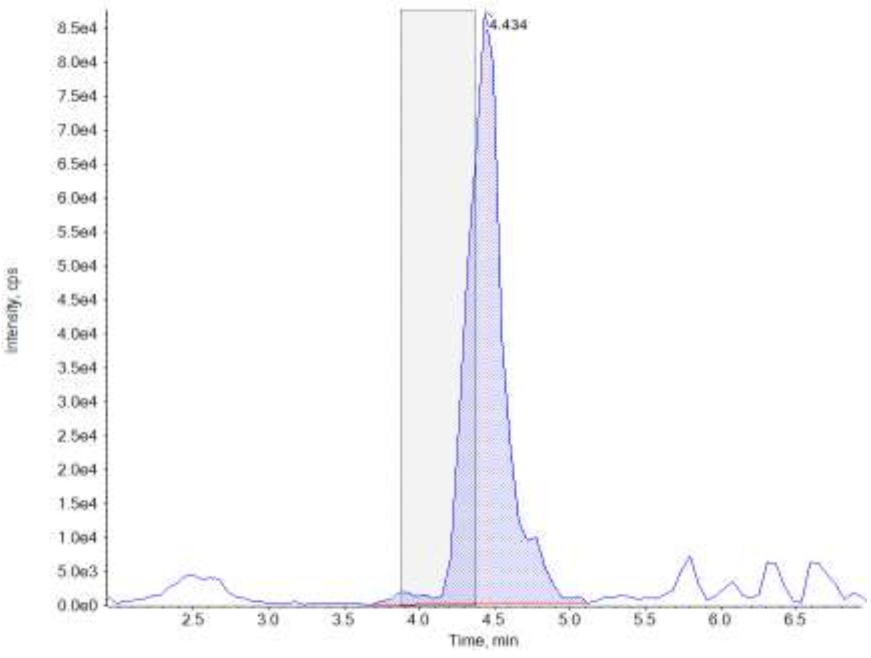

Acquired / Library MSMS

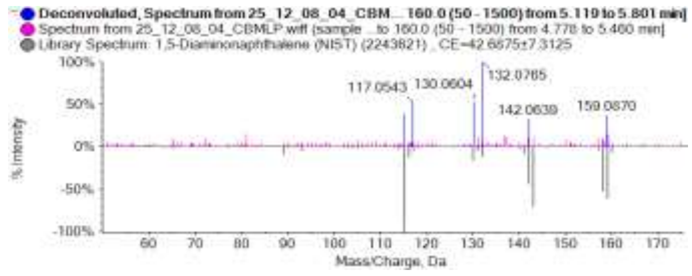

Acquired / Theoretical MS

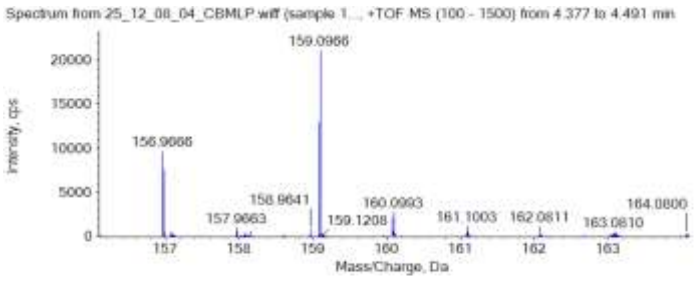

205.1058 / 4.43 (Mass/FragMass/RT/Isotope/Library/Formula/Ion Ratio)

**Retention Time:** 4.43 minutes	**Exp RT:** 4.43 minutes
**Precursor m/z :** 205.1058	**Analyte Name:**
**Fit (%)** 98.0% **RFit (%)** 100.0%	205.1058 / 4.43

Collision Energy = 35 ± 15 eV

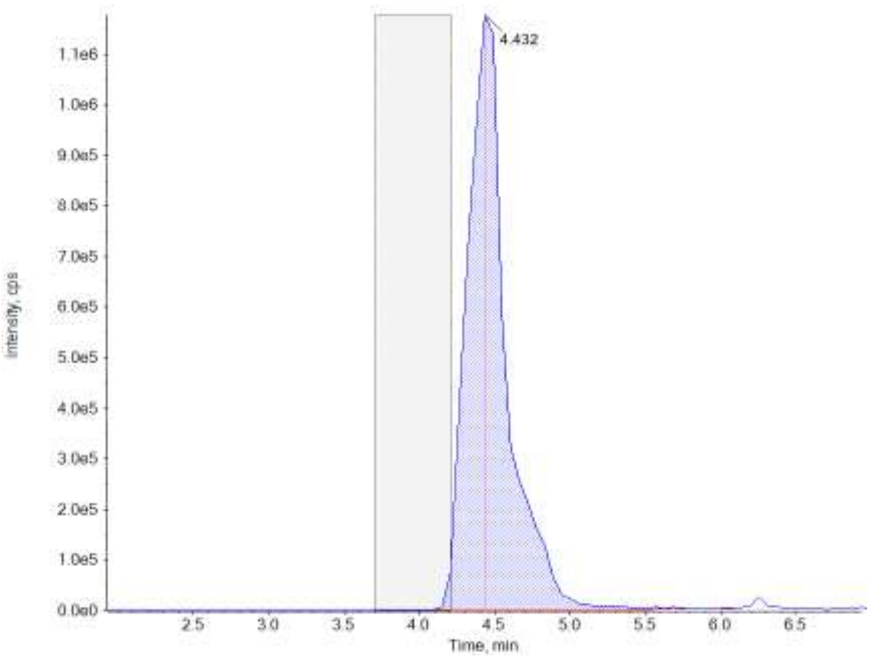

Acquired / Library MSMS

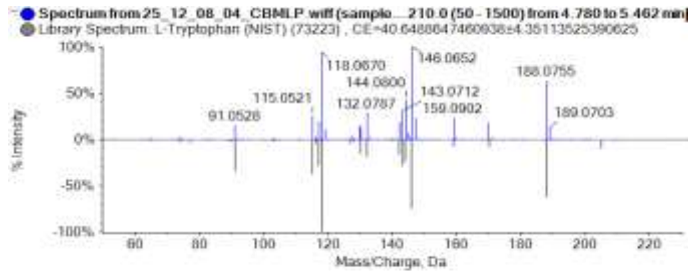

Acquired / Theoretical MS

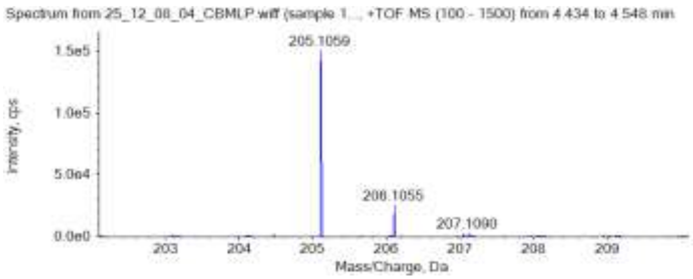

144.0854 / 4.49

(Mass/FragMass/RT/Isotope/Library/Formula/Ion Ratio)

Retention Time: 4.43 minutes
Precursor m/z : 144.0854
Fit (%) 93.0% **RFit (%)** 85.9%

Exp RT: 4.49 minutes
Analyte Name:
144.0854 / 4.49

Collision Energy = 35 ± 15 eV

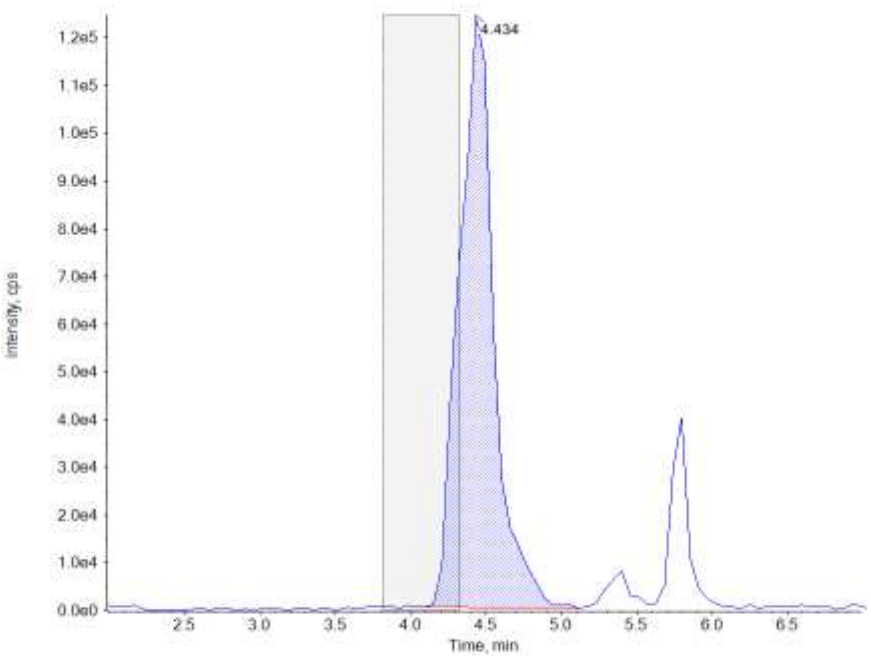

Acquired / Library MSMS

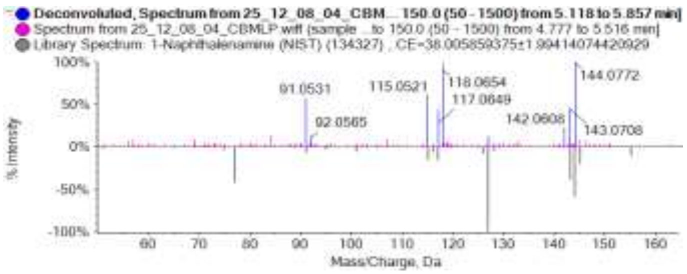

Acquired / Theoretical MS

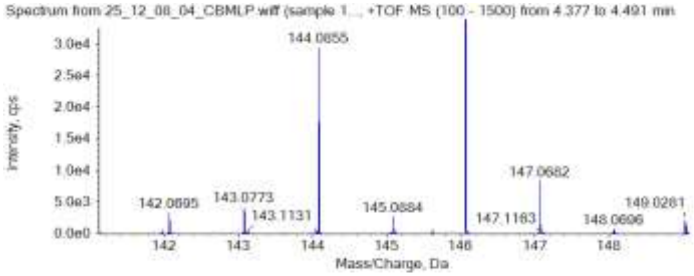

188.0849 / 4.49 (Mass/FragMass/RT/Isotope/Library/Formula/Ion Ratio)

Retention Time: 4.46 minutes
Precursor m/z : 188.0849
Fit (%) 96.8% **RFit (%)** 100.0%

Exp RT: 4.49 minutes
Analyte Name:
188.0849 / 4.49

Collision Energy = 35 ± 15 eV

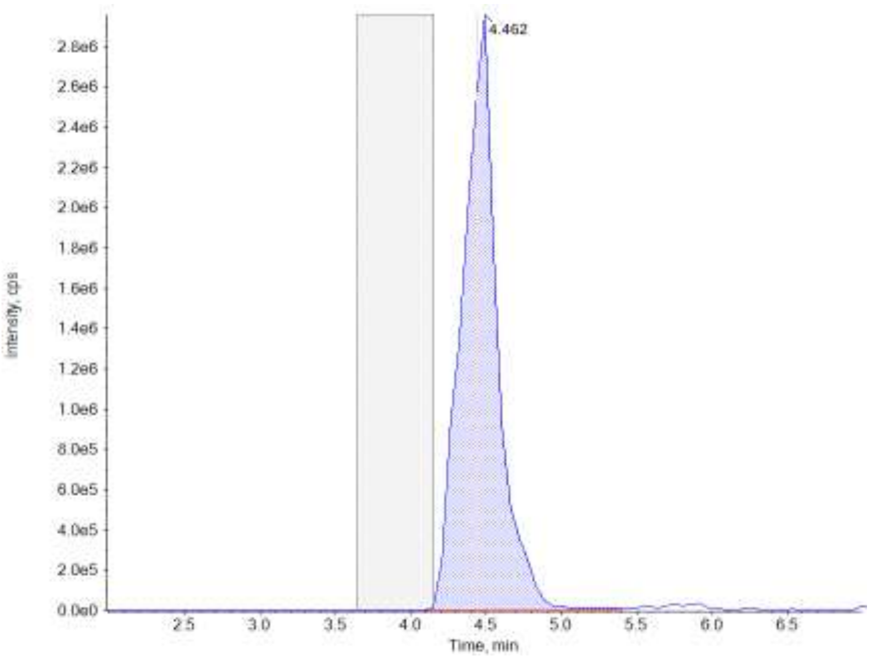

Acquired / Library MSMS

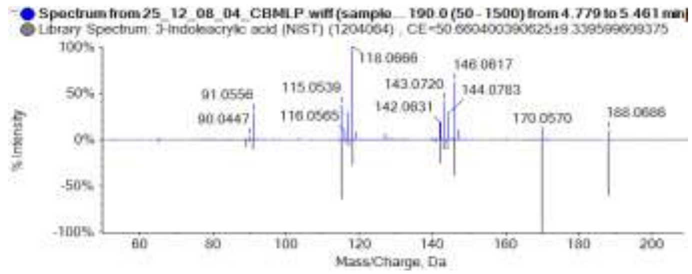

Acquired / Theoretical MS

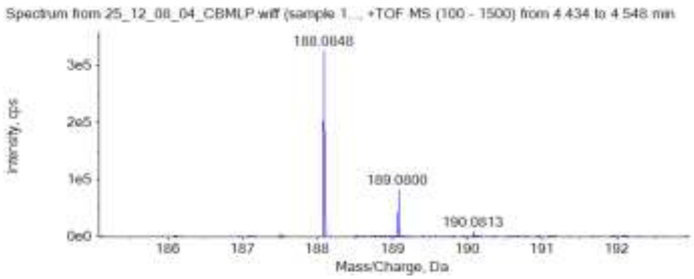

139.0448 / 5.00 (Mass/FragMass/RT/Isotope/Library/Formula/Ion Ratio)

Retention Time: 4.97 minutes
Precursor m/z : 139.0448
Fit (%) 95.5% **RFit (%)** 100.0%

Exp RT: 5.00 minutes
Analyte Name:
139.0448 / 5.00

Collision Energy = 35 ± 15 eV

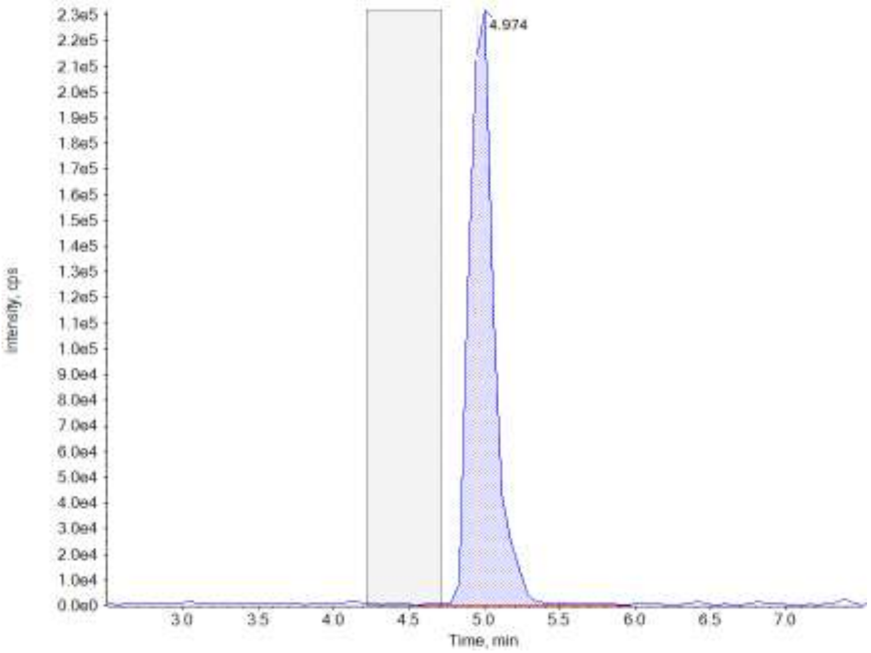

Acquired / Library MSMS

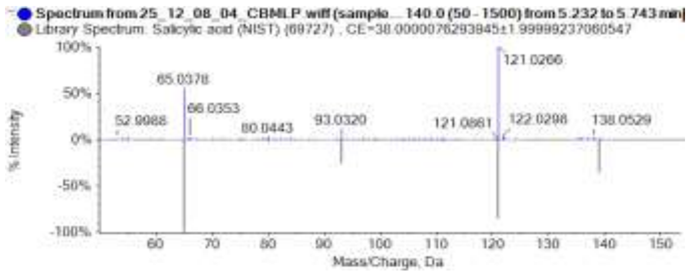

Acquired / Theoretical MS

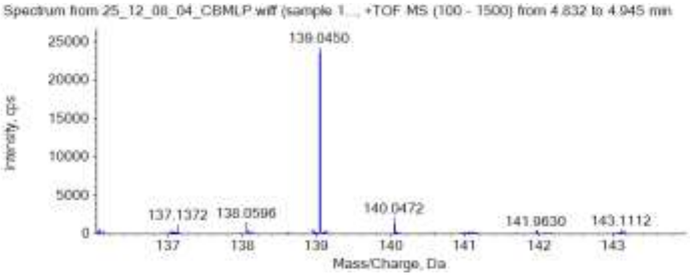

339.0548 / 5.00 (Mass/FragMass/RT/Isotope/Library/Formula/Ion Ratio)

Retention Time: 4.97 minutes
Precursor m/z : 339.0548
Fit (%) 99.0% **RFit (%)** 97.3%

Exp RT: 5.00 minutes
Analyte Name:
339.0548 / 5.00

Collision Energy = 35 ± 15 eV

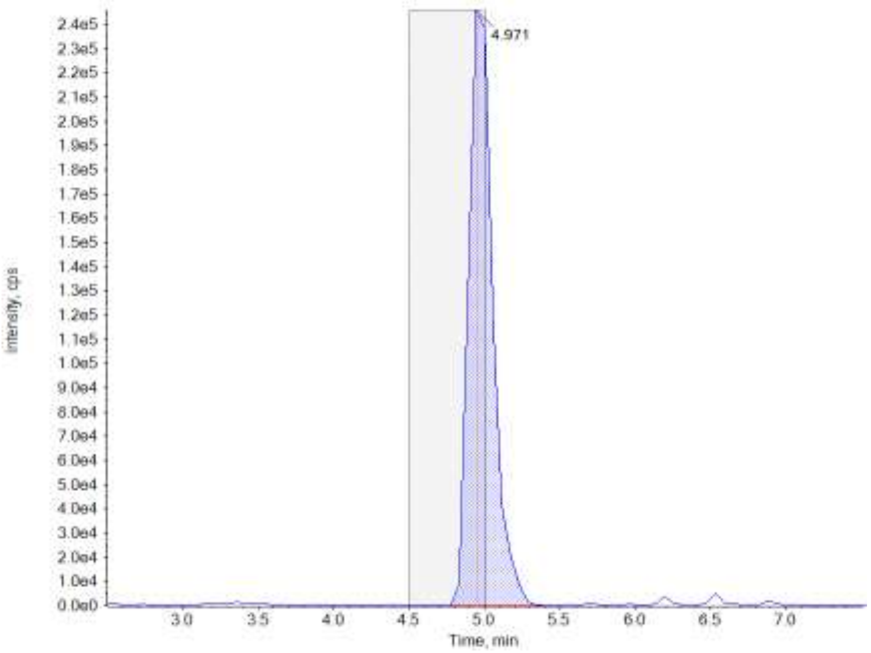

Acquired / Library MSMS

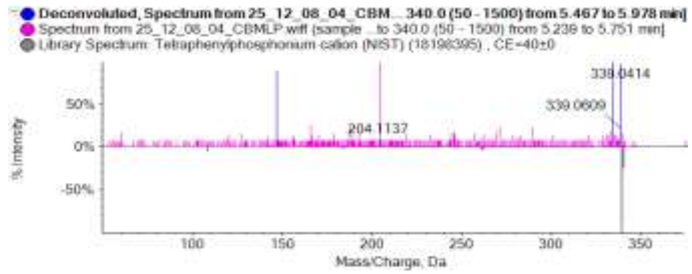

Acquired / Theoretical MS

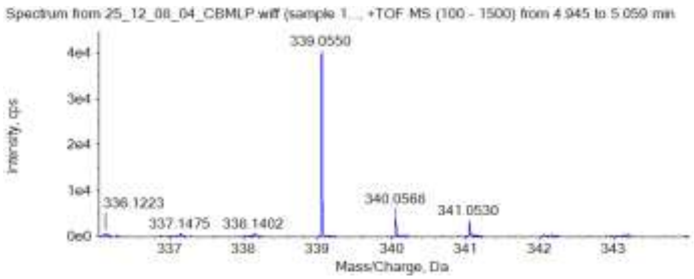

238.1124 / 5.23 (Mass/FragMass/RT/Isotope/Library/Formula/Ion Ratio)

Retention Time: 5.16 minutes
Precursor m/z : 238.1124
Fit (%) 86.0% **RFit (%)** 61.8%

Exp RT: 5.23 minutes
Analyte Name:
238.1124 / 5.23

Collision Energy = 35 ± 15 eV

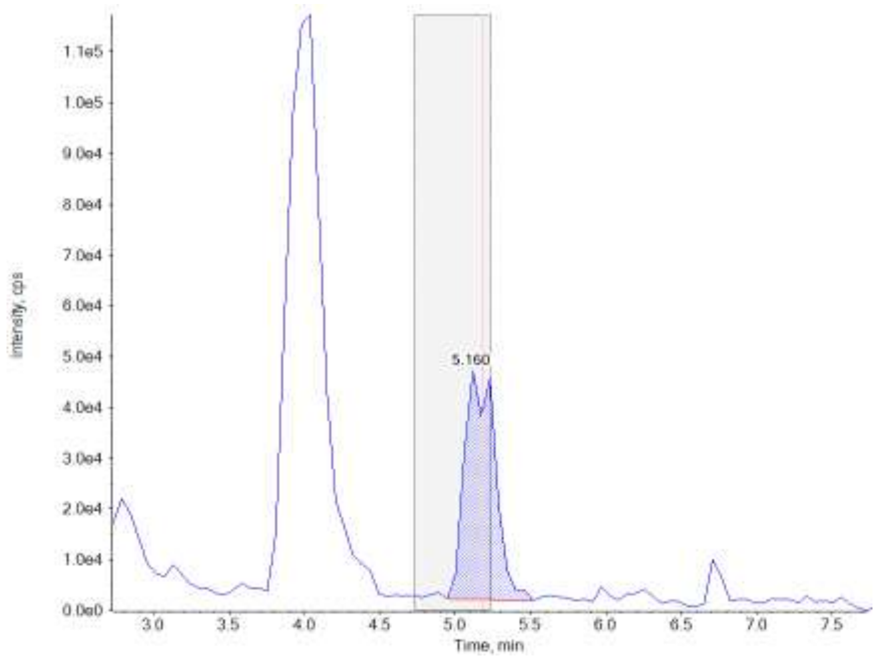

Acquired / Library MSMS

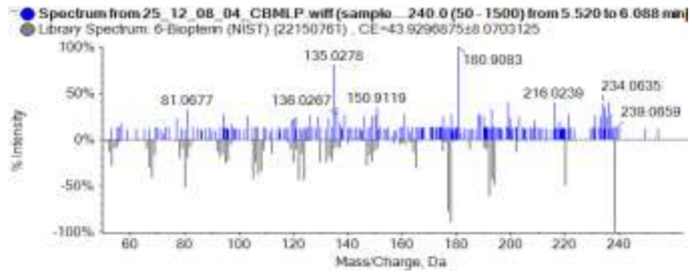

Acquired / Theoretical MS

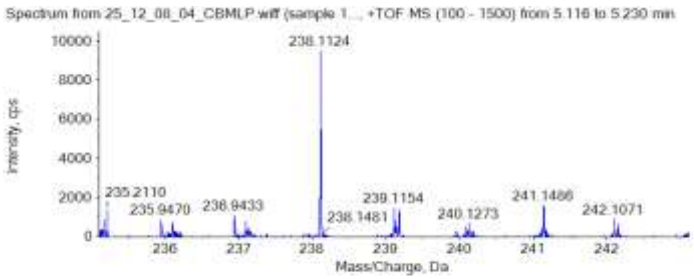

120.0852 / 5.40 (Mass/FragMass/RT/Isotope/Library/Formula/Ion Ratio)

**Retention Time:** 5.39 minutes	**Exp RT:** 5.40 minutes
**Precursor m/z :** 120.0852	**Analyte Name:**
**Fit (%)** 74.3% **RFit (%)** 94.7%	120.0852 / 5.40

Collision Energy = 35 ± 15 eV

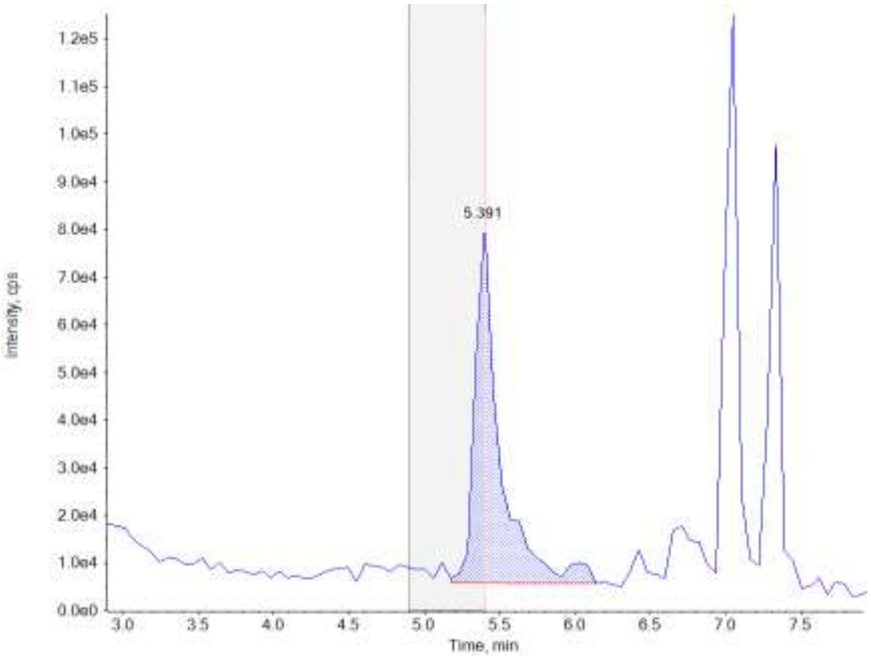

Acquired / Library MSMS

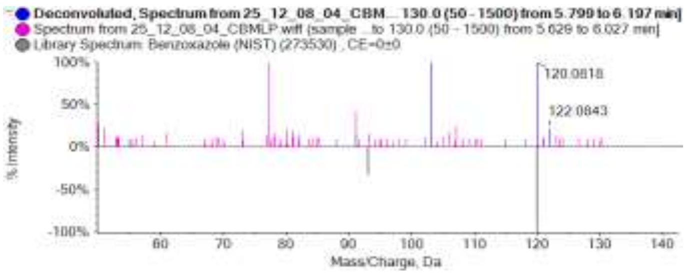

Acquired / Theoretical MS

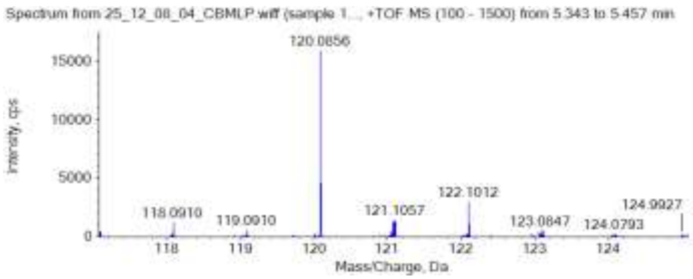

Created with SCIEX OS 3.3.0
Printed:12/9/2025 2:04:37 PM

295.1429 / 5.40 (Mass/FragMass/RT/Isotope/Library/Formula/Ion Ratio)

Retention Time: 5.41 minutes **Exp RT:** 5.40 minutes
Precursor m/z : 295.1429 **Analyte Name:**
Fit (%) 84.4% **RFit (%)** 94.6% 295.1429 / 5.40

Collision Energy = 35 ± 15 eV

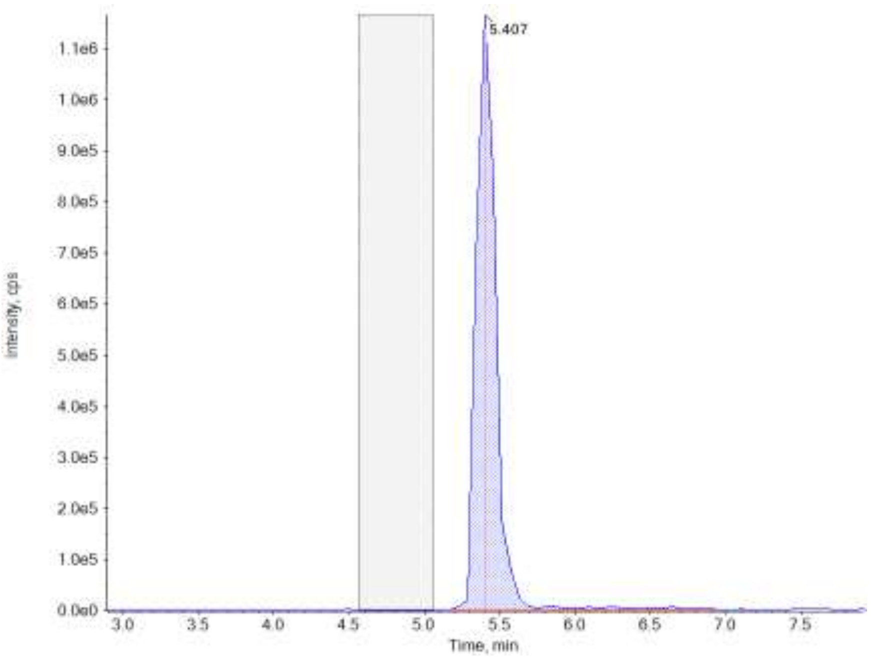

Acquired / Library MSMS

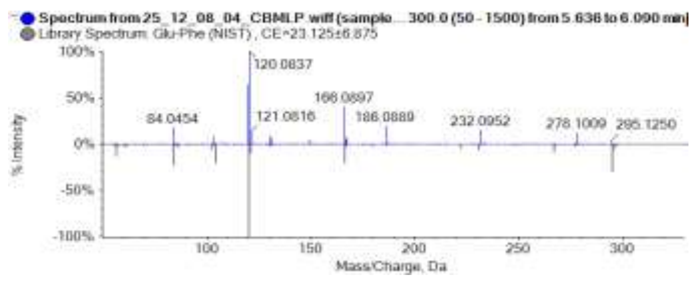

Acquired / Theoretical MS

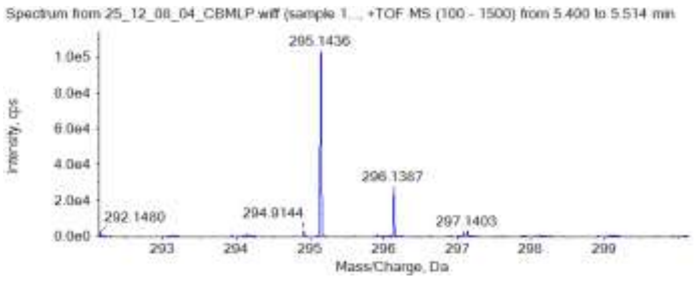

Created with SCIEX OS 3.3.0
Printed:12/9/2025 2:04:37 PM

174.1546 / 5.74 (Mass/FragMass/RT/Isotope/Library/Formula/Ion Ratio)

Retention Time: 5.76 minutes
Precursor m/z : 174.1546
Fit (%) 100.0% **RFit (%)** 77.6%

Exp RT: 5.74 minutes
Analyte Name:
174.1546 / 5.74

Collision Energy = 35 ± 15 eV

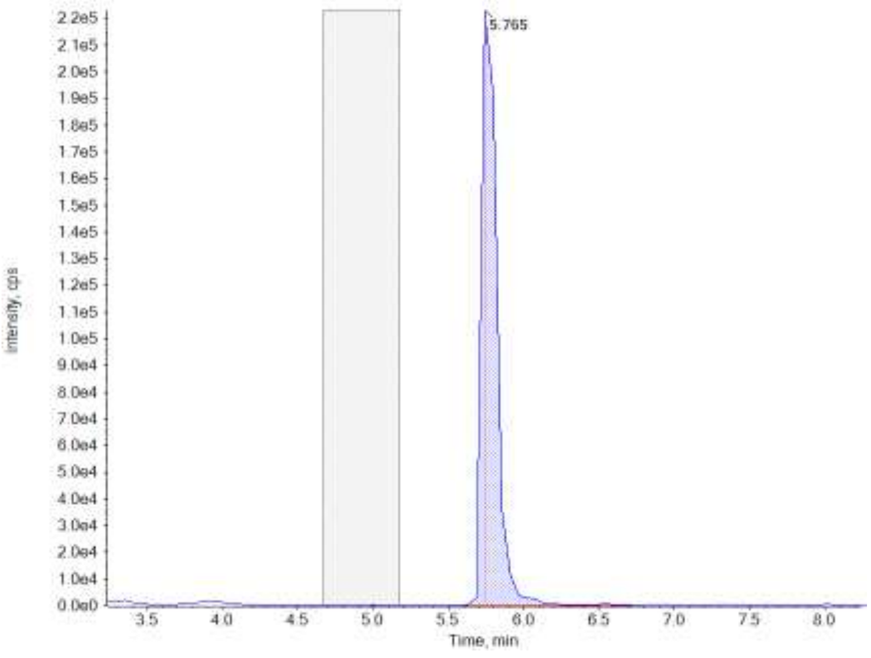

Acquired / Library MSMS

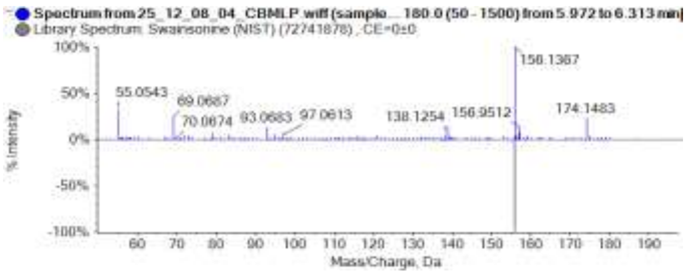

Acquired / Theoretical MS

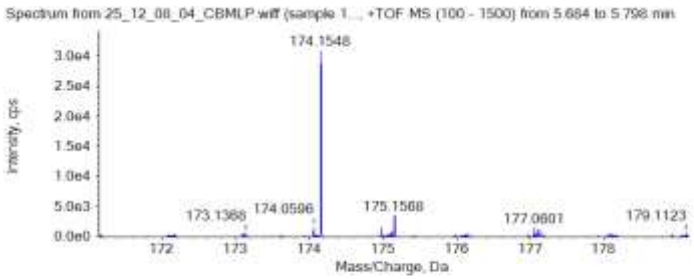

Created with SCIEX OS 3.3.0
Printed:12/9/2025 2:04:37 PM

217.1020 / 5.74 (Mass/FragMass/RT/Isotope/Library/Formula/Ion Ratio)

Retention Time: 5.77 minutes **Exp RT:** 5.74 minutes
Precursor m/z : 217.1020 **Analyte Name:**
Fit (%) 63.6% **RFit (%)** 98.1% 217.1020 / 5.74

Collision Energy = 35 ± 15 eV

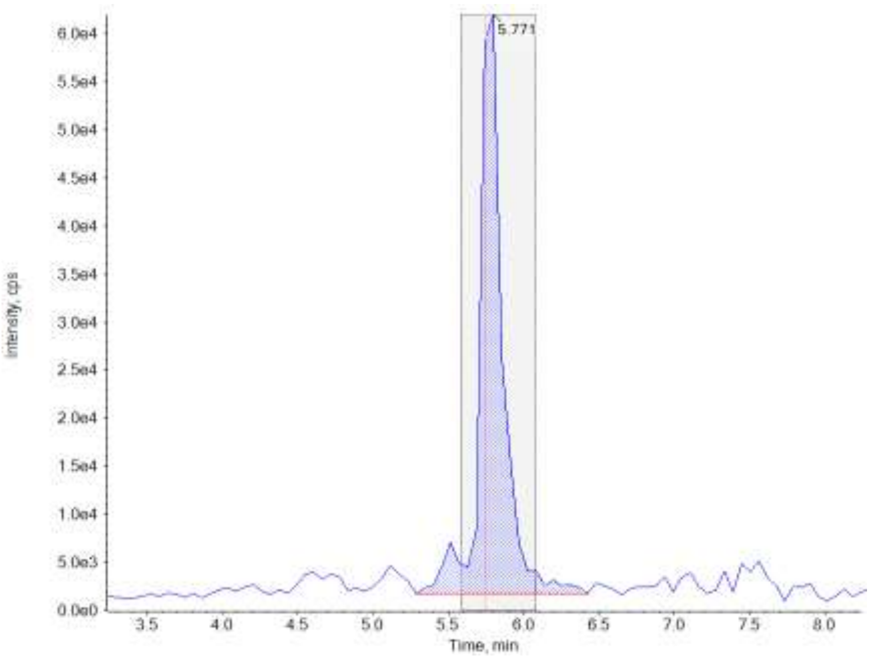

Acquired / Library MSMS

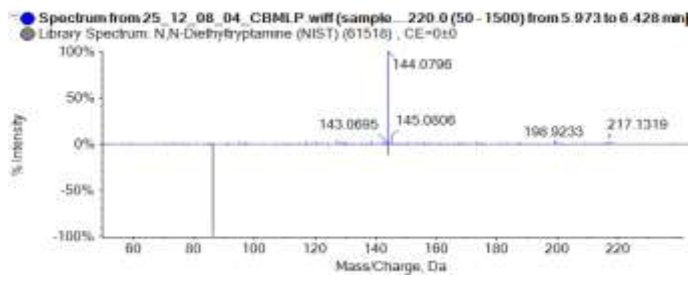

Acquired / Theoretical MS

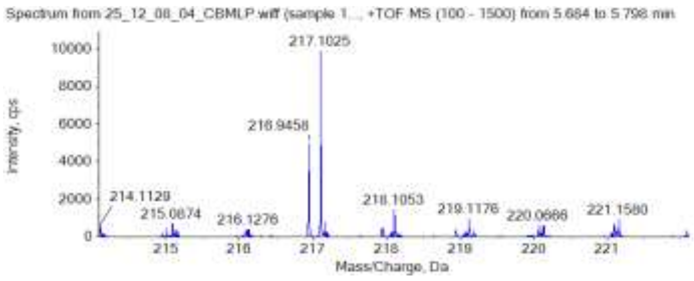

166.0915 / 6.08 (Mass/FragMass/RT/Isotope/Library/Formula/Ion Ratio)

Retention Time: 6.08 minutes
Precursor m/z : 166.0914
Fit (%) 100.0% **RFit (%)** 42.8%

Exp RT: 6.08 minutes
Analyte Name:
166.0915 / 6.08

Collision Energy = 35 ± 15 eV

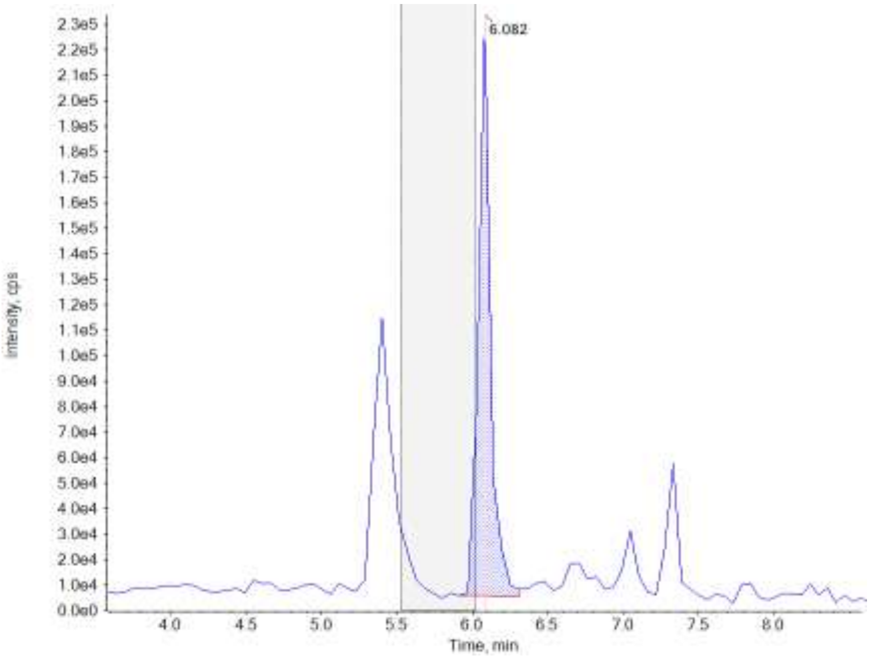

Acquired / Library MSMS

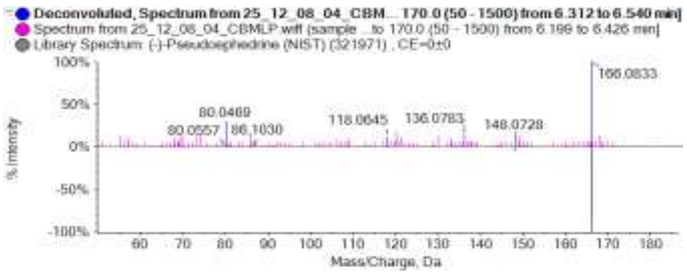

Acquired / Theoretical MS

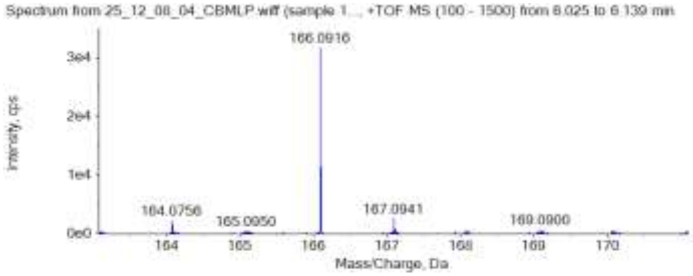

212.0974 / 6.08 [M+H]+ (Mass/FragMass/RT/Isotope/Library/Formula/Ion Ratio)

Retention Time: 6.09 minutes
Precursor m/z : 212.0974
Fit (%) 69.5% **RFit (%)** 70.9%

Exp RT: 6.08 minutes
Analyte Name:
212.0974 / 6.08 [M+H]+

Collision Energy = 35 ± 15 eV

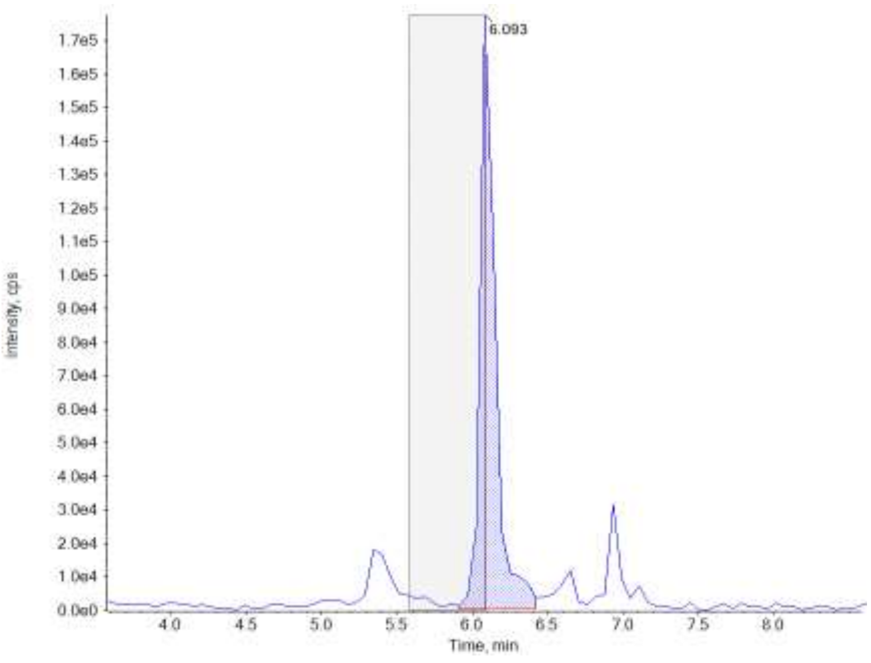

Acquired / Library MSMS

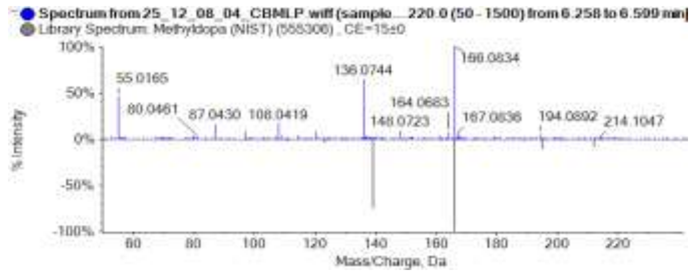

Acquired / Theoretical MS

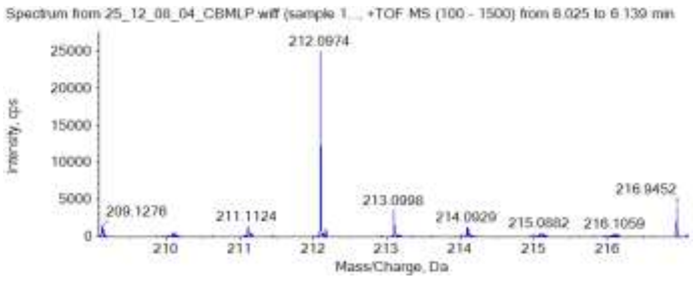

229.1238 / 6.08 [M+NH4]+

(Mass/FragMass/RT/Isotope/Library/Formula/Ion Ratio)

Retention Time: 6.10 minutes
Precursor m/z : 229.1238
Fit (%) 97.8% **RFit (%)** 84.2%

Exp RT: 6.08 minutes
Analyte Name:
229.1238 / 6.08 [M+NH4]+

Collision Energy = 35 ± 15 eV

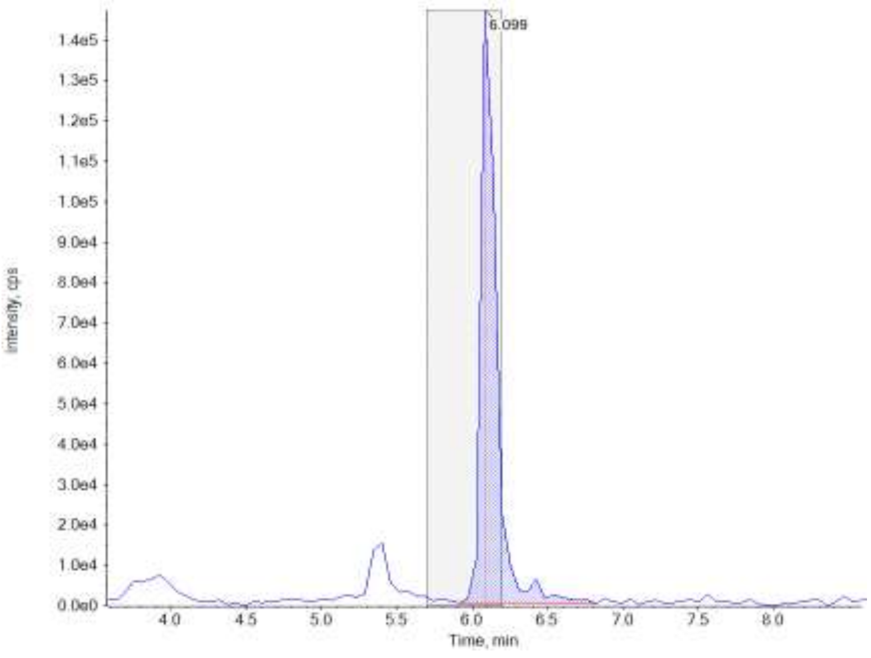

Acquired / Library MSMS

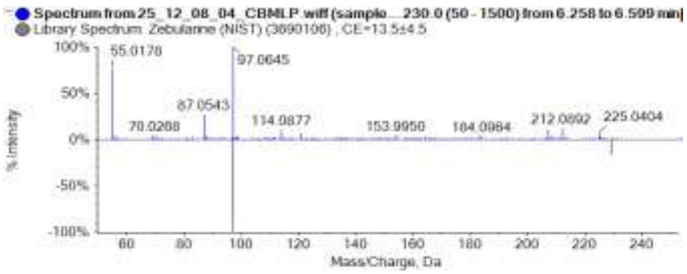

Acquired / Theoretical MS

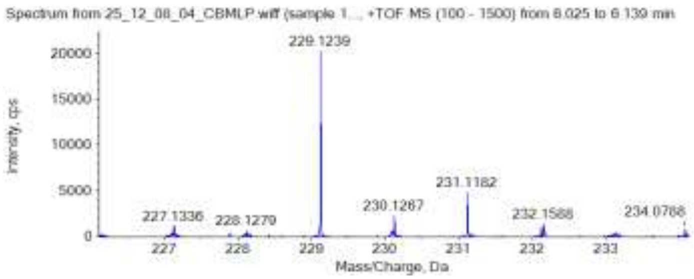

377.1515 / 6.37 (Mass/FragMass/RT/Isotope/Library/Formula/Ion Ratio)

Retention Time: 6.37 minutes
Precursor m/z : 377.1515
Fit (%) 87.7% **RFit (%)** 89.5%

Exp RT: 6.37 minutes
Analyte Name:
377.1515 / 6.37

Collision Energy = 35 ± 15 eV

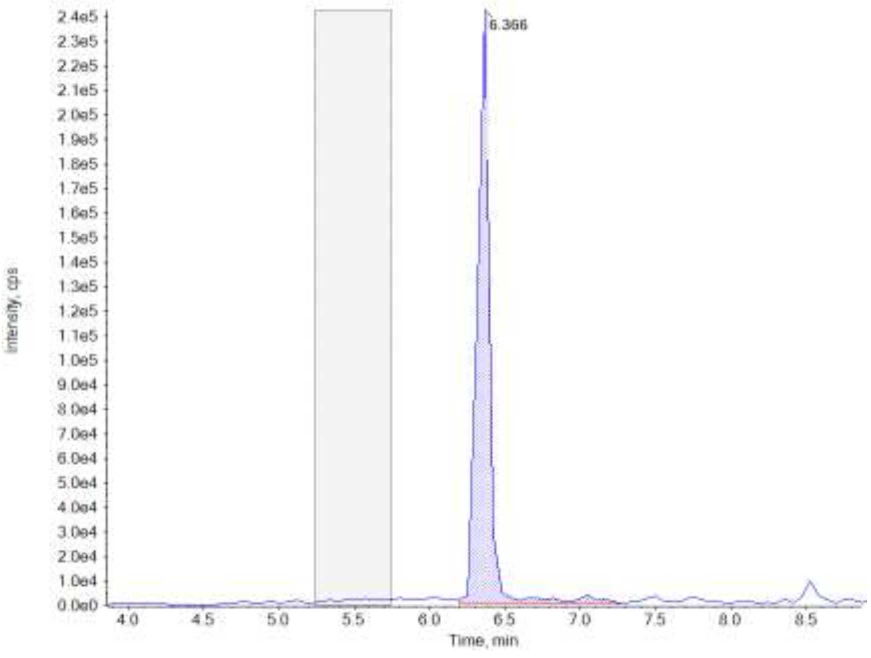

Acquired / Library MSMS

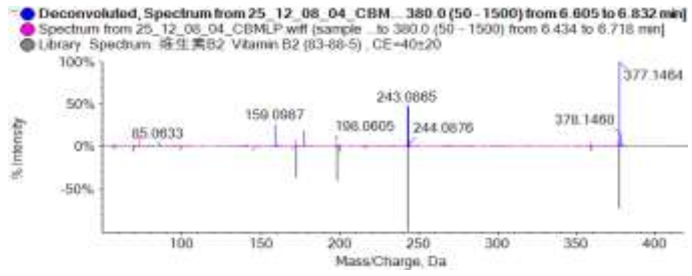

Acquired / Theoretical MS

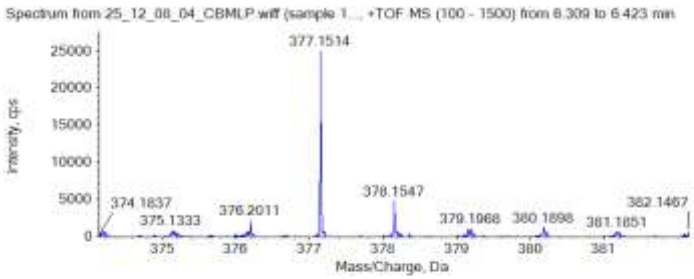

265.1488 / 6.42 (Mass/FragMass/RT/Isotope/Library/Formula/Ion Ratio)

**Retention Time:** 6.40 minutes	**Exp RT:** 6.42 minutes
**Precursor m/z :** 265.1488	**Analyte Name:**
**Fit (%)** 99.5% **RFit (%)** 52.0%	265.1488 / 6.42

Collision Energy = 35 ± 15 eV

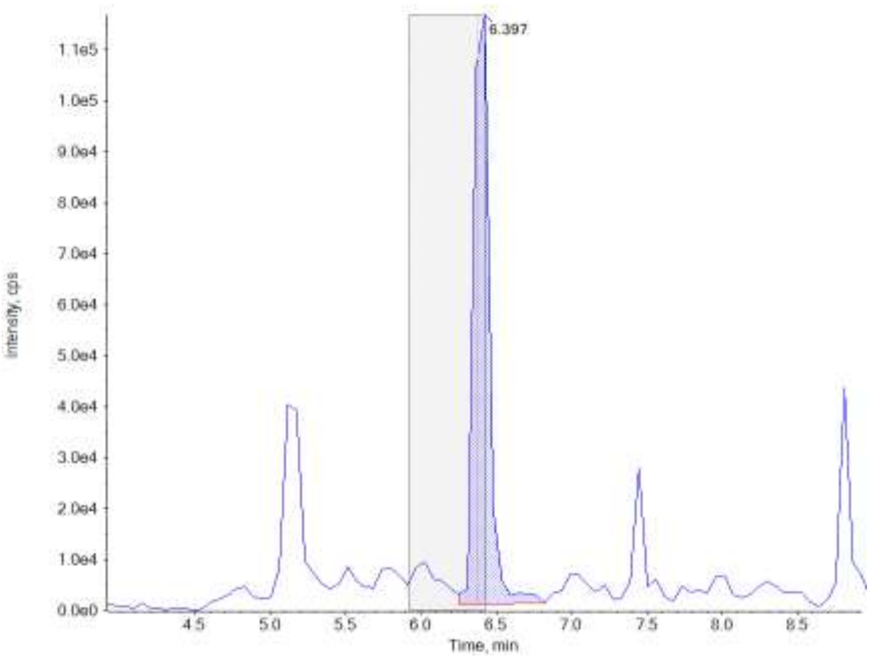

Acquired / Library MSMS

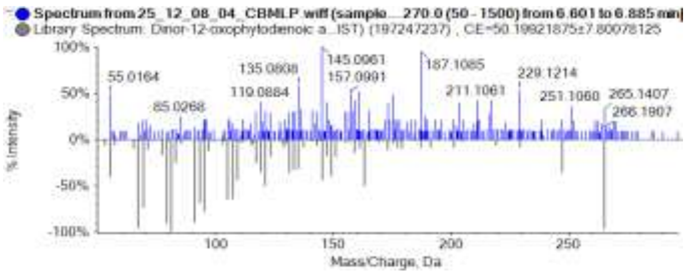

Acquired / Theoretical MS

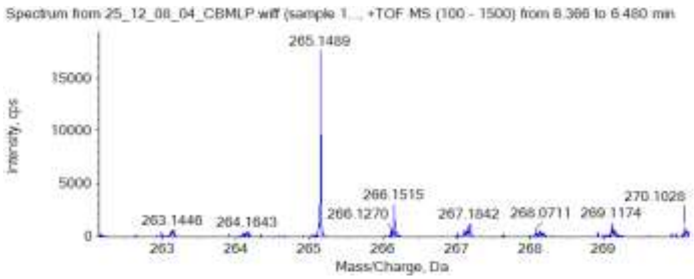

209.1222 / 6.71 (Mass/FragMass/RT/Isotope/Library/Formula/Ion Ratio)

Retention Time: 6.71 minutes
Precursor m/z : 209.1222
Fit (%) 41.8% **RFit (%)** 96.3%

Exp RT: 6.71 minutes
Analyte Name:
209.1222 / 6.71

Collision Energy = 35 ± 15 eV

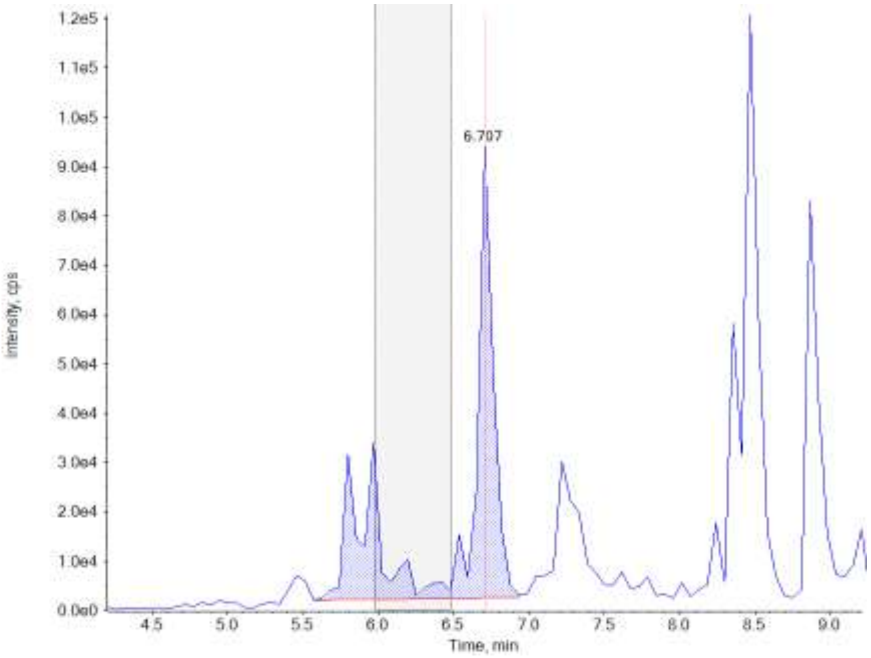

Acquired / Library MSMS

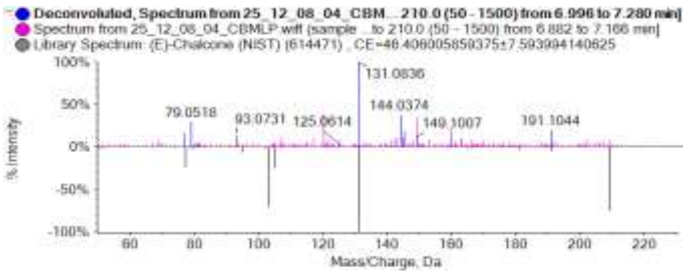

Acquired / Theoretical MS

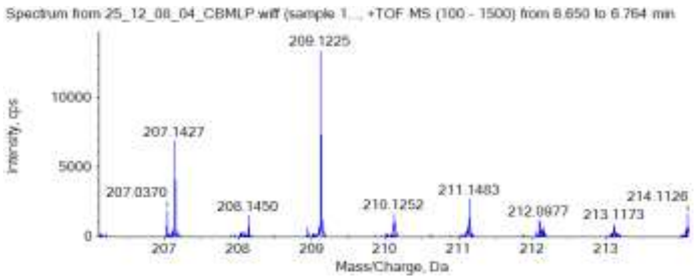

585.2943 / 7.22 [M+K]+ (Mass/FragMass/RT/Isotope/Library/Formula/Ion Ratio)

Retention Time: 7.19 minutes
Precursor m/z : 585.2943
Fit (%) 56.6% **RFit (%)** 100.0%

Exp RT: 7.22 minutes
Analyte Name:
585.2943 / 7.22 [M+K]+

Collision Energy = 35 ± 15 eV

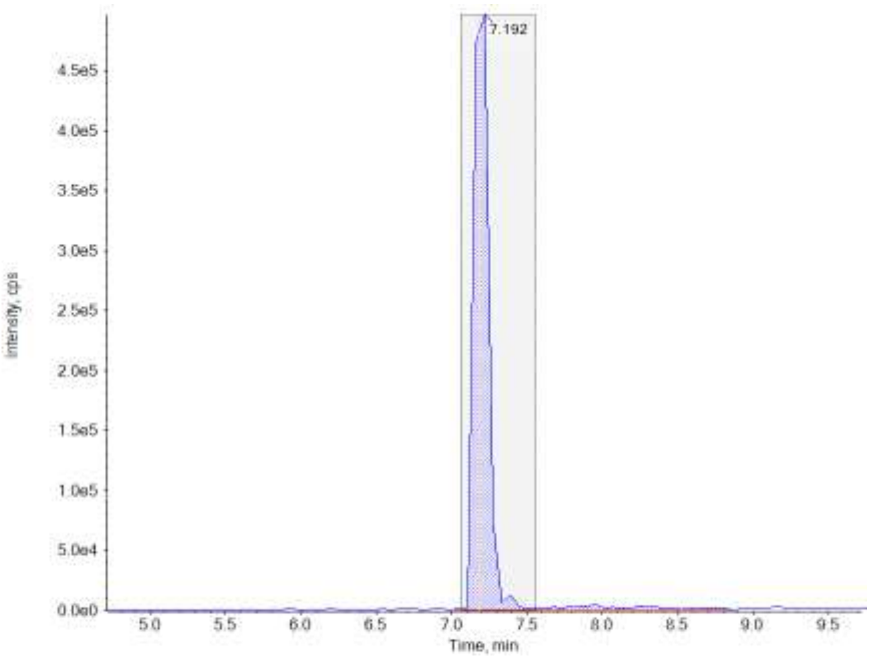

Acquired / Library MSMS

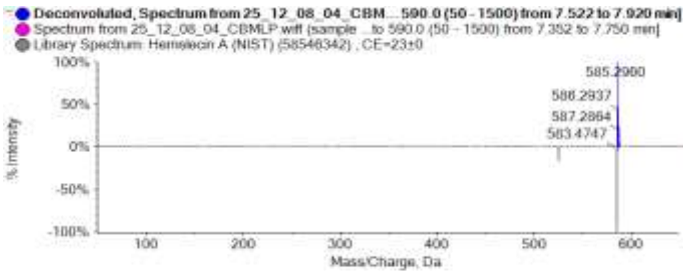

Acquired / Theoretical MS

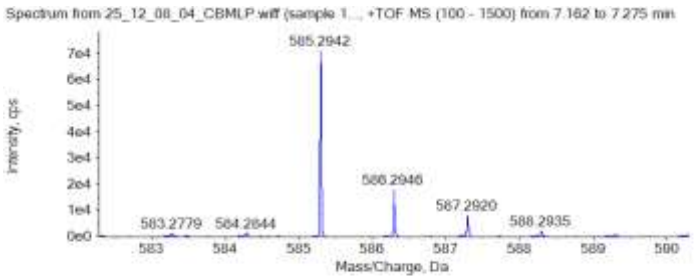

591.3660 / 7.39 [M+H]+ (Mass/FragMass/RT/Isotope/Library/Formula/Ion Ratio)

**Retention Time:** 7.39 minutes<br>**Precursor m/z :** 591.3660<br>**Fit (%)** 67.9% **RFit (%)** 92.9%	**Exp RT:** 7.39 minutes<br>**Analyte Name:**<br>591.3660 / 7.39 [M+H]+

	**Collision Energy = 35 ± 15 eV**	
	**Acquired / Library MSMS**	
	**Acquired / Theoretical MS**	

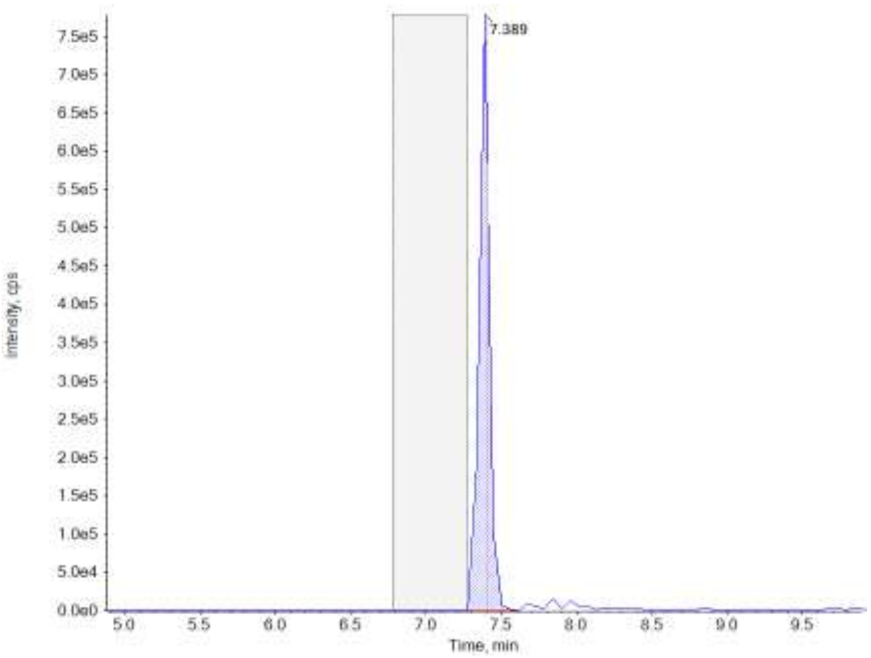

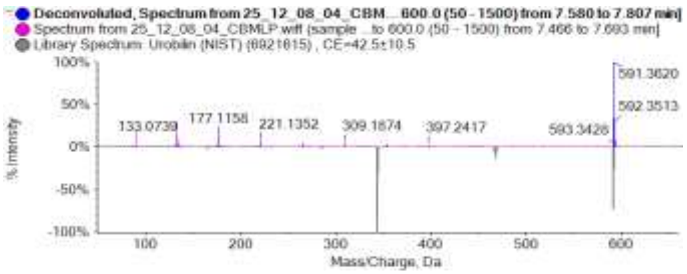

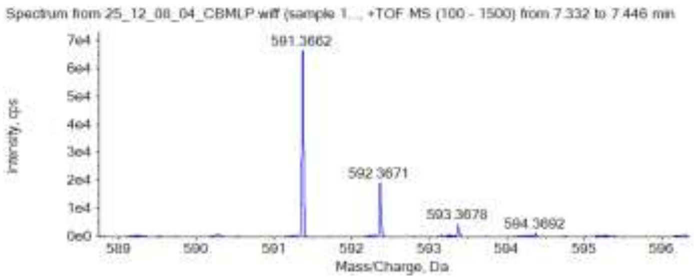

629.3216 / 7.39 [M+K]+ (Mass/FragMass/RT/Isotope/Library/Formula/Ion Ratio)

**Retention Time:** 7.39 minutes<br>**Precursor m/z :** 629.3216<br>**Fit (%)** 100.0% **RFit (%)** 100.0%	**Exp RT:** 7.39 minutes<br>**Analyte Name:**<br>629.3216 / 7.39 [M+K]+

		**Collision Energy = 35 ± 15 eV**
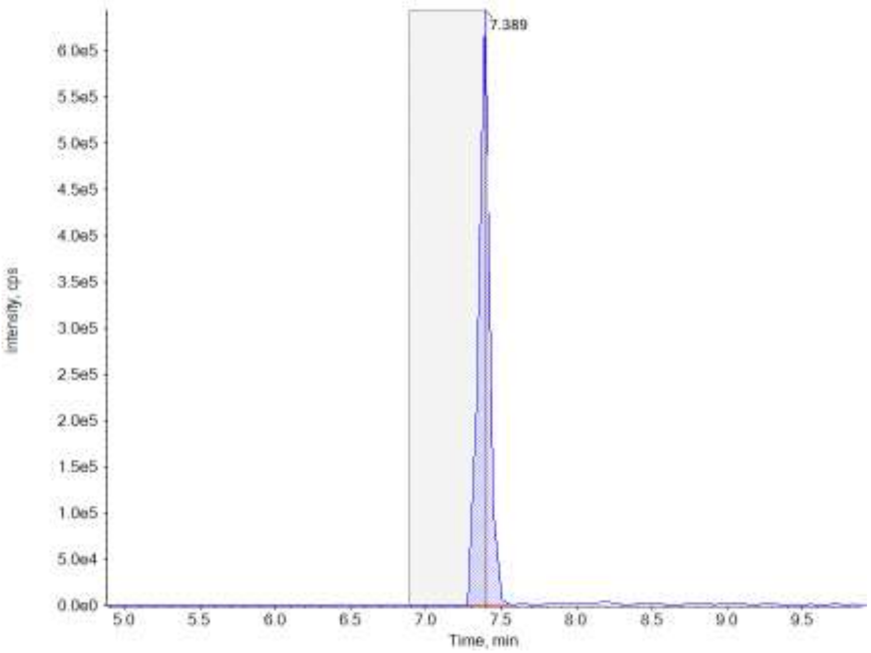	**Acquired / Library MSMS**	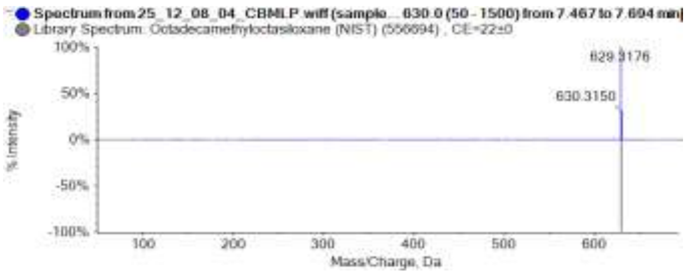
	**Acquired / Theoretical MS**	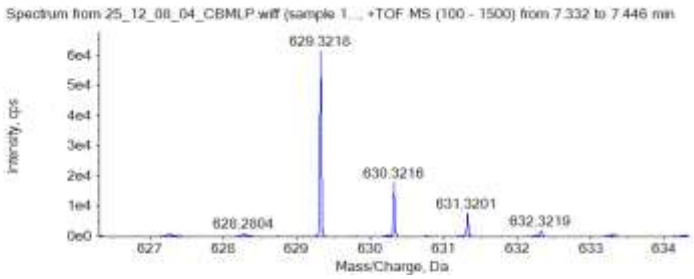

652.4275 / 7.56 [M+NH4]+

(Mass/FragMass/RT/Isotope/Library/Formula/Ion Ratio)

Retention Time: 7.55 minutes
Precursor m/z : 652.4275
Fit (%) 78.0% **RFit (%)** 81.6%

Exp RT: 7.56 minutes
Analyte Name:
652.4275 / 7.56 [M+NH4]+

Collision Energy = 35 ± 15 eV

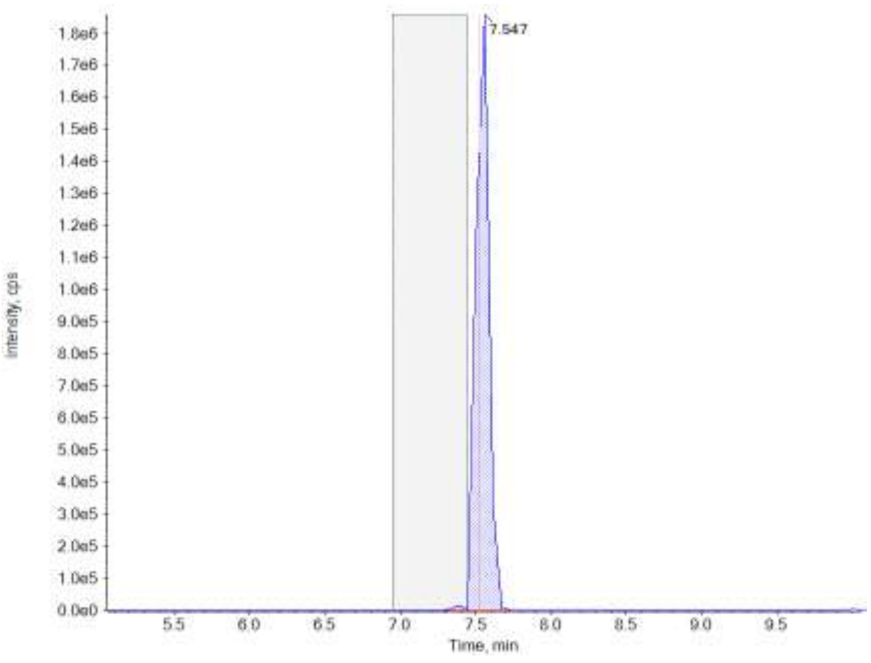

Acquired / Library MSMS

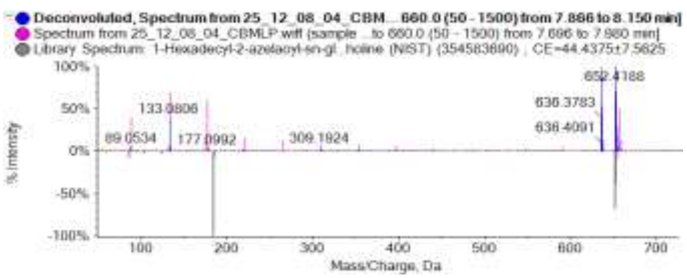

Acquired / Theoretical MS

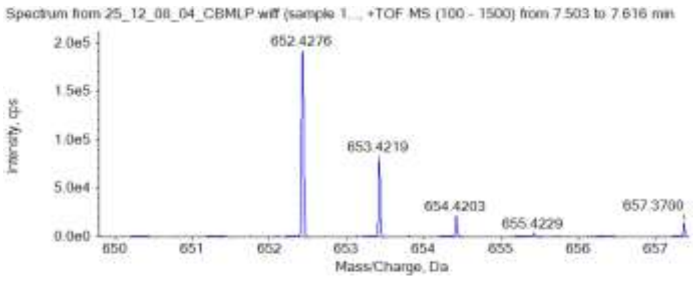

Created with SCIEX OS 3.3.0
Printed:12/9/2025 2:04:37 PM

347.1287 / 7.56 (Mass/FragMass/RT/Isotope/Library/Formula/Ion Ratio)

Retention Time: 7.58 minutes
Precursor m/z : 347.1287
Fit (%) 90.4% **RFit (%)** 92.1%

Exp RT: 7.56 minutes
Analyte Name:
347.1287 / 7.56

Collision Energy = 35 ± 15 eV

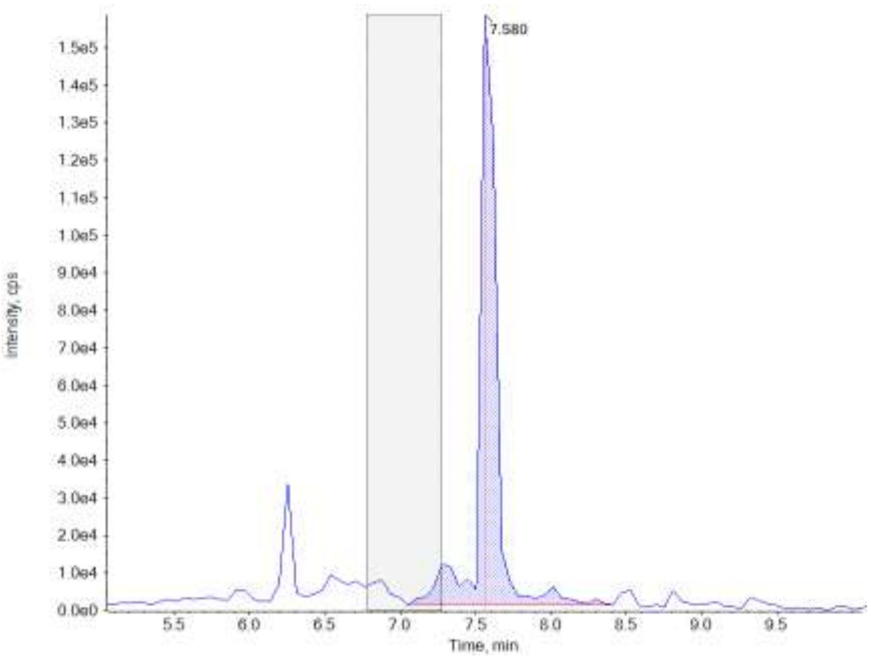

Acquired / Library MSMS

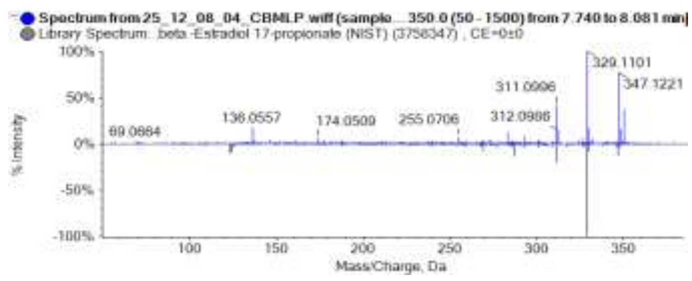

Acquired / Theoretical MS

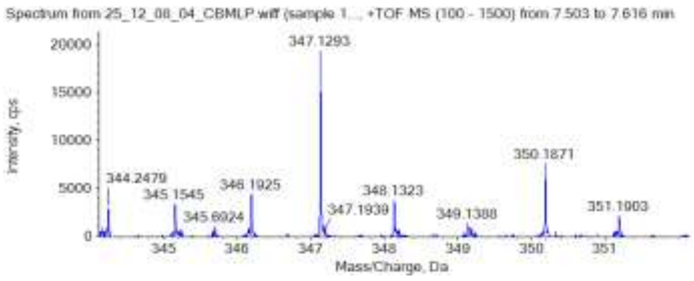

673.3468 / 7.56 [M+K]+ (Mass/FragMass/RT/Isotope/Library/Formula/Ion Ratio)

Retention Time: 7.56 minutes
Precursor m/z : 673.3468
Fit (%) 100.0% **RFit (%)** 87.3%

Exp RT: 7.56 minutes
Analyte Name:
673.3468 / 7.56 [M+K]+

Collision Energy = 35 ± 15 eV

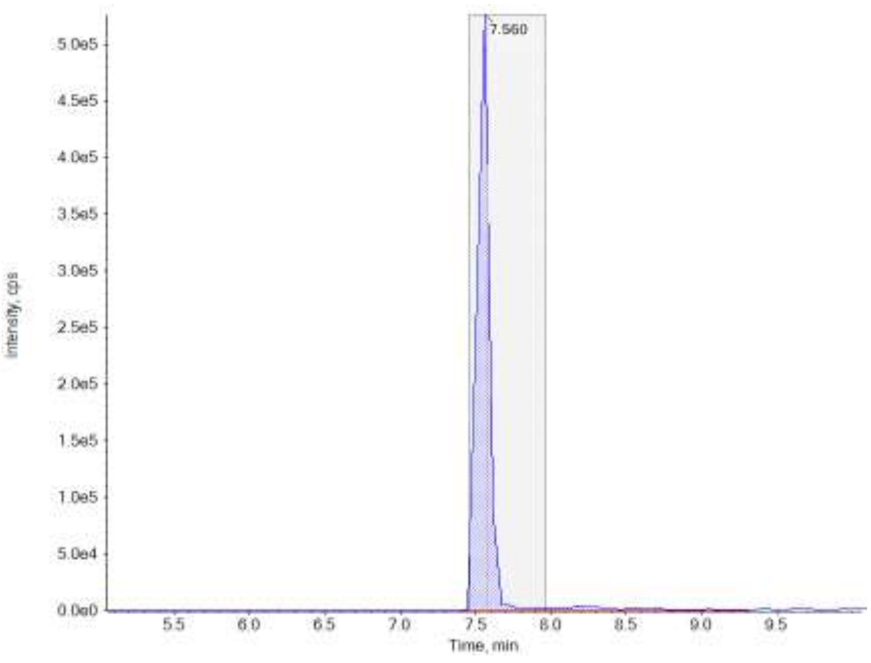

Acquired / Library MSMS

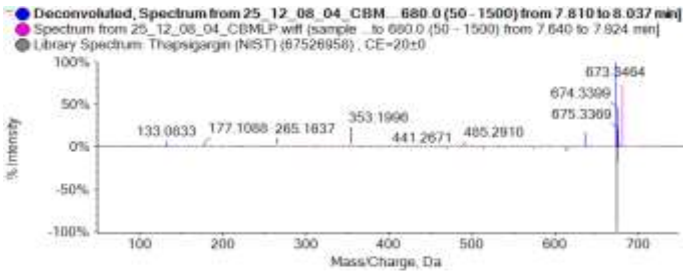

Acquired / Theoretical MS

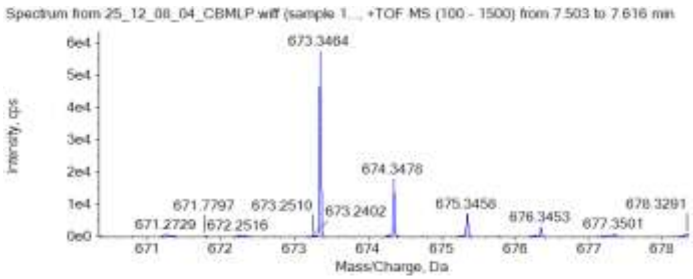

635.3900 / 7.56 [M+H]+

(Mass/FragMass/RT/Isotope/Library/Formula/Ion Ratio)

**Retention Time:** 7.56 minutes	**Exp RT:** 7.56 minutes
**Precursor m/z :** 635.3900	**Analyte Name:**
**Fit (%)** 50.9% **RFit (%)** 86.1%	635.3900 / 7.56 [M+H]+

Collision Energy = 35 ± 15 eV

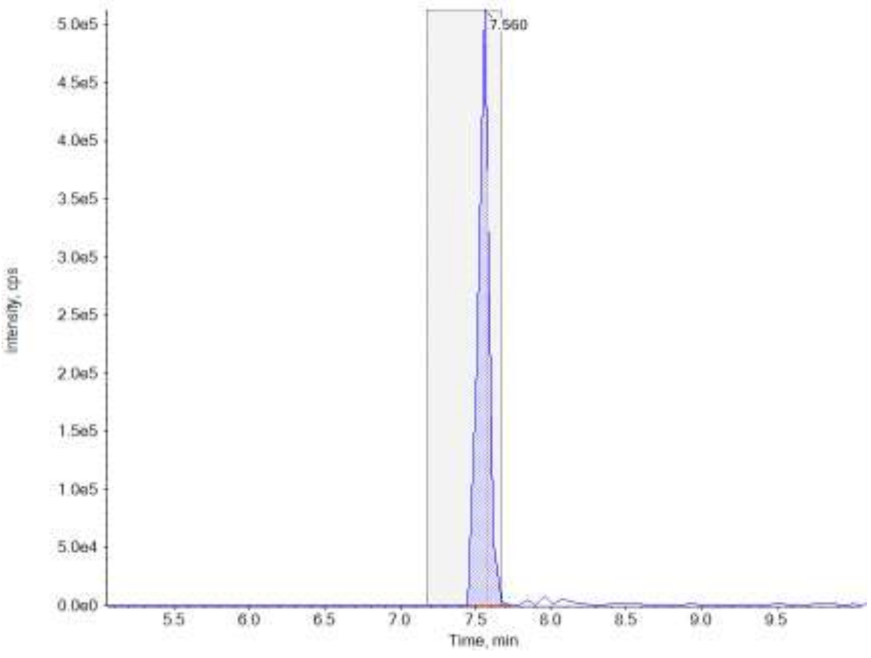

Acquired / Library MSMS

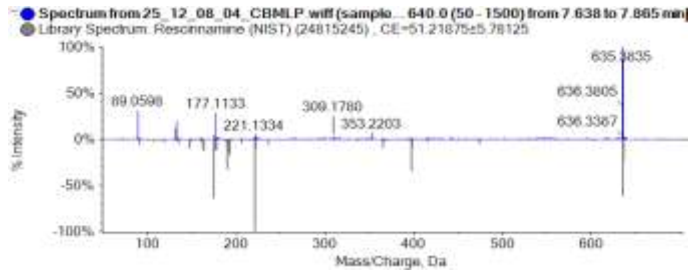

Acquired / Theoretical MS

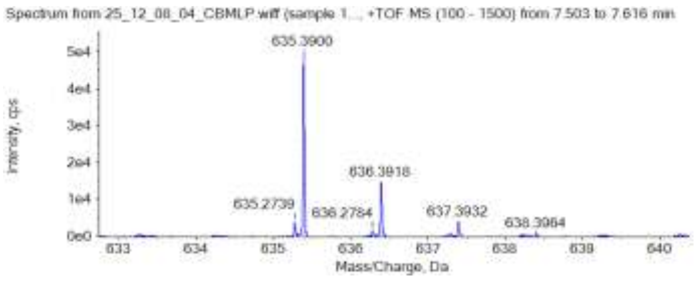

740.4750 / 7.84 (Mass/FragMass/RT/Isotope/Library/Formula/Ion Ratio)

Retention Time: 7.84 minutes
Precursor m/z : 740.4750
Fit (%) 38.3% **RFit (%)** 91.9%

Exp RT: 7.84 minutes
Analyte Name:
740.4750 / 7.84

Collision Energy = 35 ± 15 eV

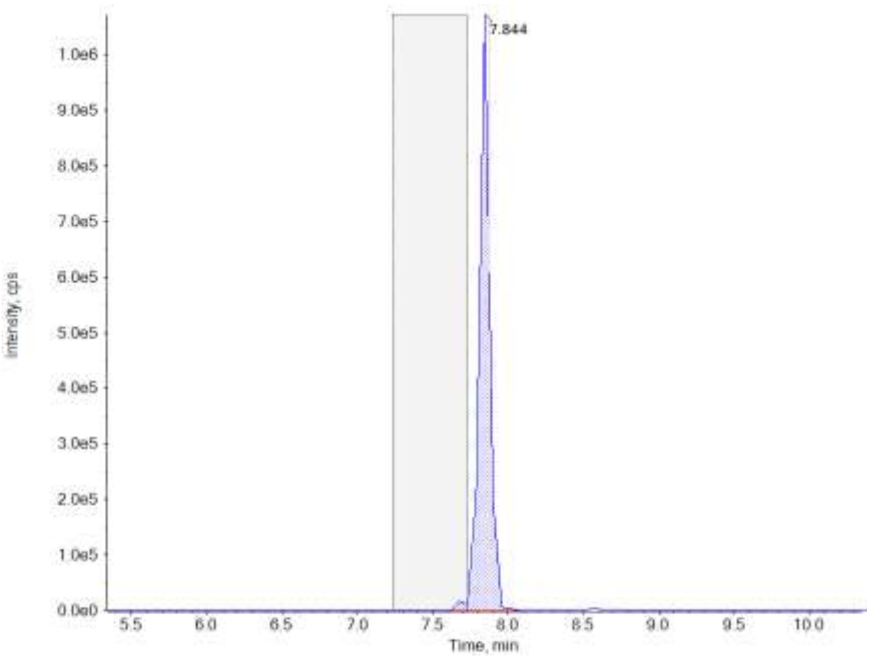

Acquired / Library MSMS

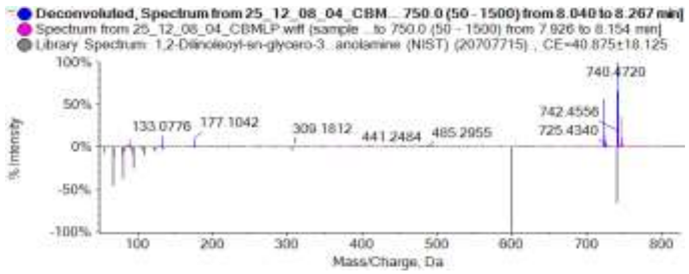

Acquired / Theoretical MS

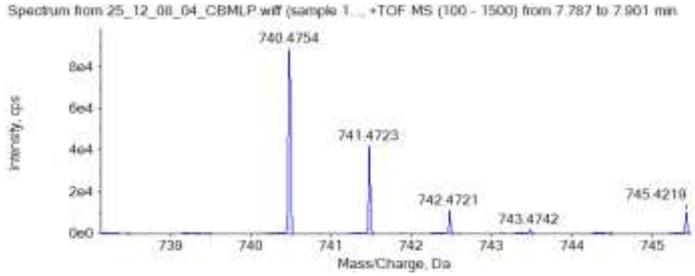

768.5018 / 7.84 (Mass/FragMass/RT/Isotope/Library/Formula/Ion Ratio)

**Retention Time:** 7.84 minutes	**Exp RT:** 7.84 minutes
**Precursor m/z :** 768.5018	**Analyte Name:**
**Fit (%)** 82.2% **RFit (%)** 79.2%	768.5018 / 7.84

	Collision Energy = 35 ± 15 eV

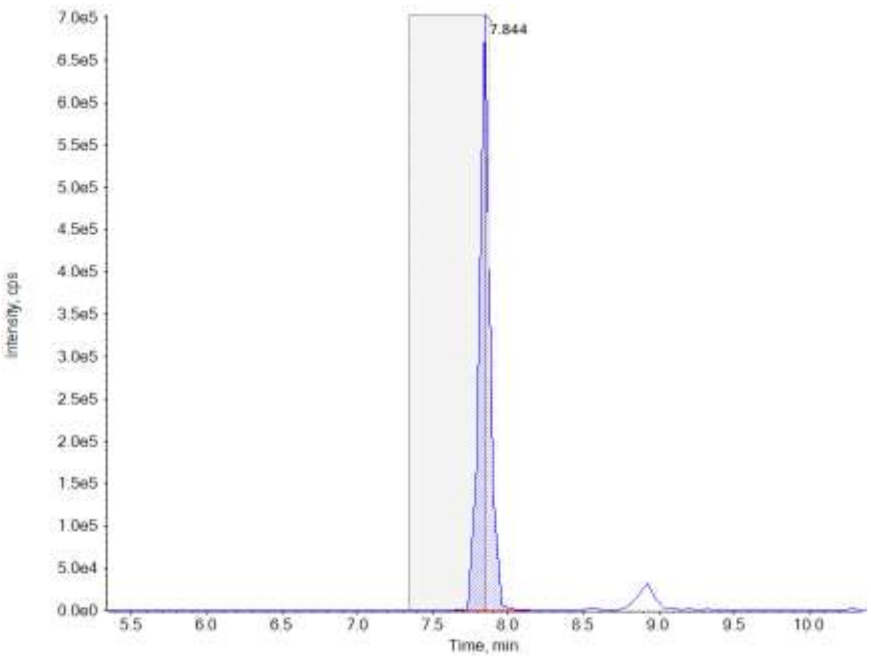

Acquired / Library MSMS

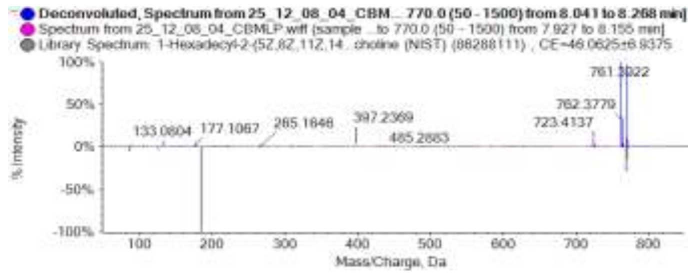

Acquired / Theoretical MS

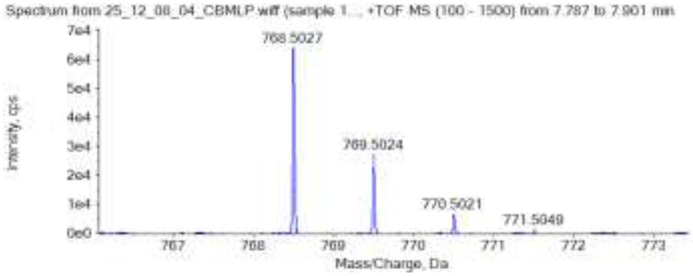

784.4976 / 7.96 (Mass/FragMass/RT/Isotope/Library/Formula/Ion Ratio)

Retention Time: 7.96 minutes
Precursor m/z : 784.4976
Fit (%) 91.8% **RFit (%)** 92.5%

Exp RT: 7.96 minutes
Analyte Name:
784.4976 / 7.96

Collision Energy = 35 ± 15 eV

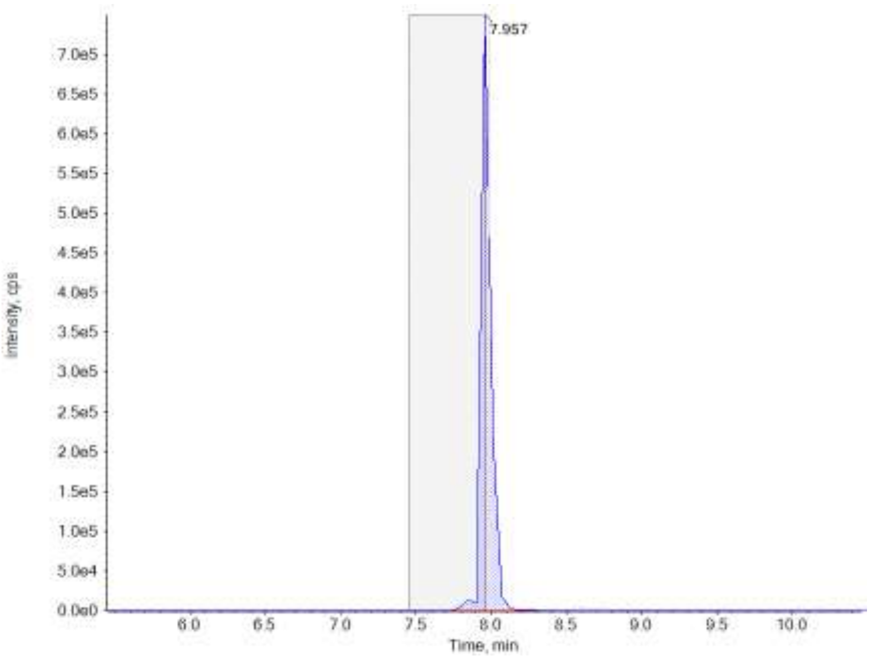

Acquired / Library MSMS

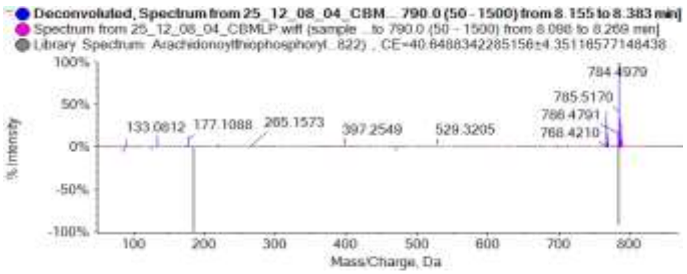

Acquired / Theoretical MS

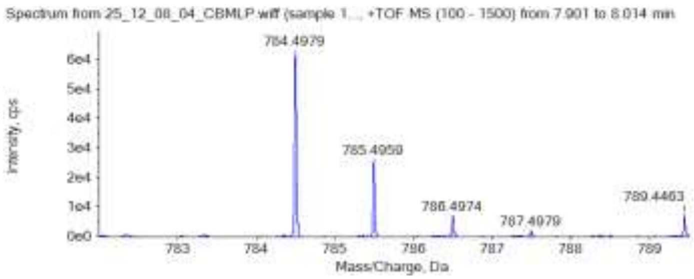

211.1742 / 8.07 (Mass/FragMass/RT/Isotope/Library/Formula/Ion Ratio)

**Retention Time:** 8.07 minutes<br>**Precursor m/z :** 211.1742<br>**Fit (%)** 97.6% **RFit (%)** 32.5%	**Exp RT:** 8.07 minutes<br>**Analyte Name:**<br>211.1742 / 8.07

Collision Energy = 35 ± 15 eV

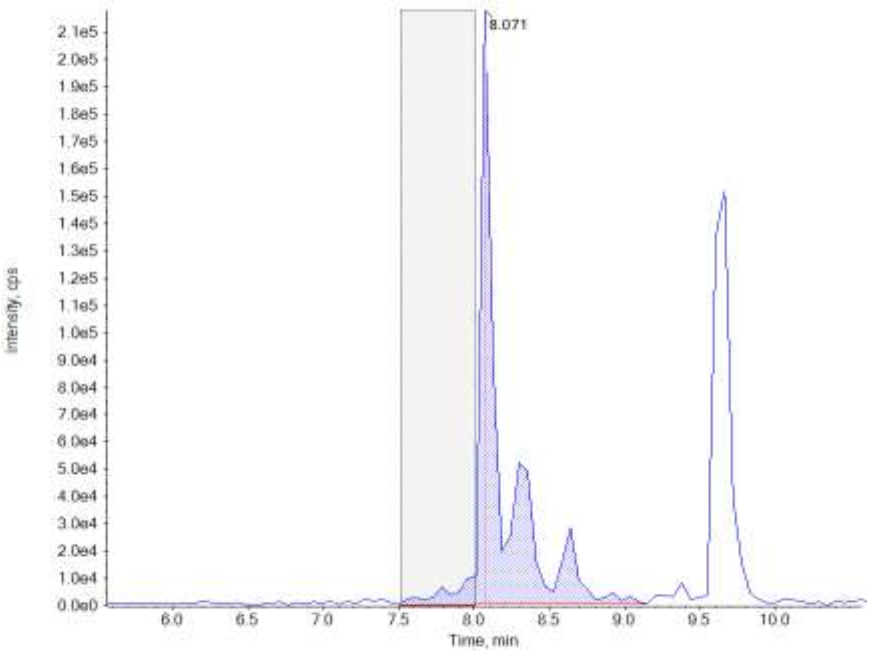

Acquired / Library MSMS

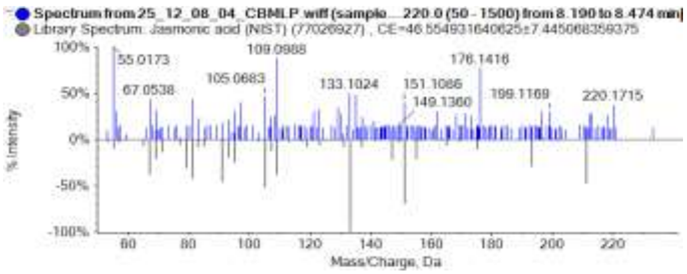

Acquired / Theoretical MS

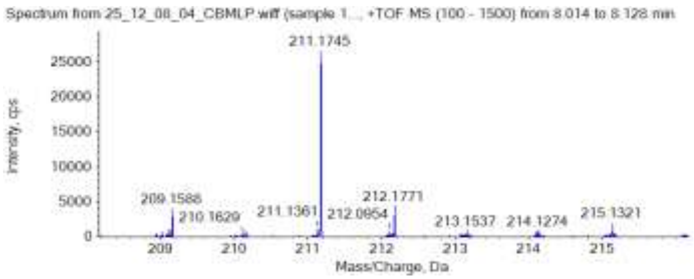

414.7690 / 8.07 [M+H+NH4]2+ (Mass/FragMass/RT/Isotope/Library/Formula/Ion Ratio)

Retention Time: 8.08 minutes
Precursor m/z : 414.7690
Fit (%) 93.7% **RFit (%)** 91.1%

Exp RT: 8.07 minutes
Analyte Name:
414.7690 / 8.07 [M+H+NH4]2+

Collision Energy = 35 ± 15 eV

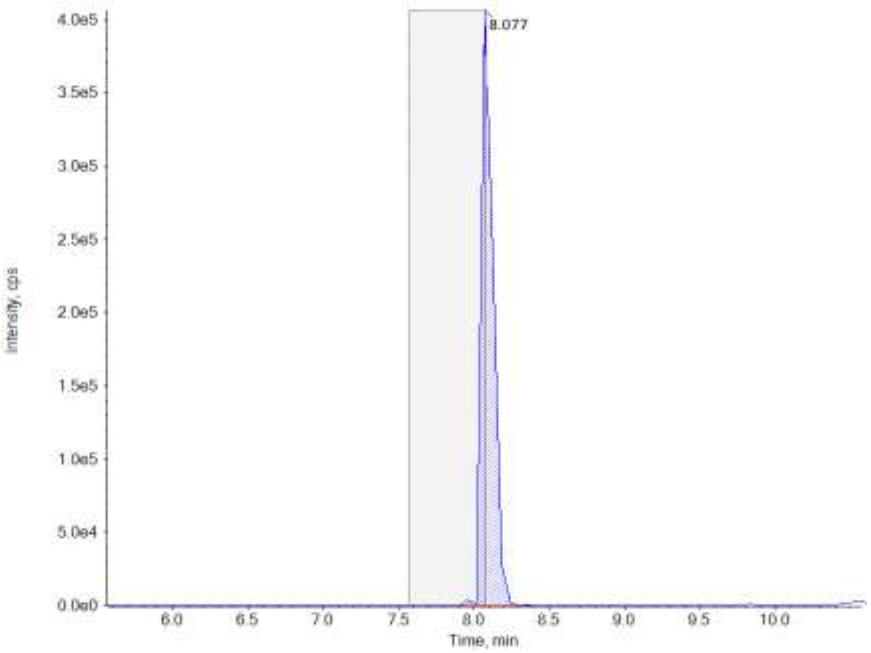

Acquired / Library MSMS

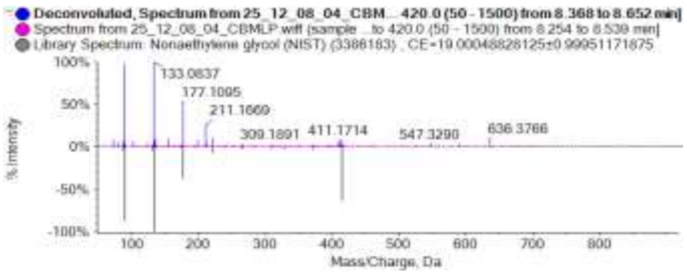

Acquired / Theoretical MS

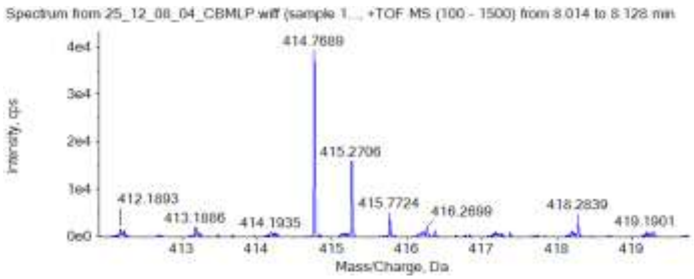

146.0652 / 8.13 (Mass/FragMass/RT/Isotope/Library/Formula/Ion Ratio)

Retention Time: 8.13 minutes
Precursor m/z : 146.0652
Fit (%) 98.0% **RFit (%)** 100.0%

Exp RT: 8.13 minutes
Analyte Name:
146.0652 / 8.13

Collision Energy = 35 ± 15 eV

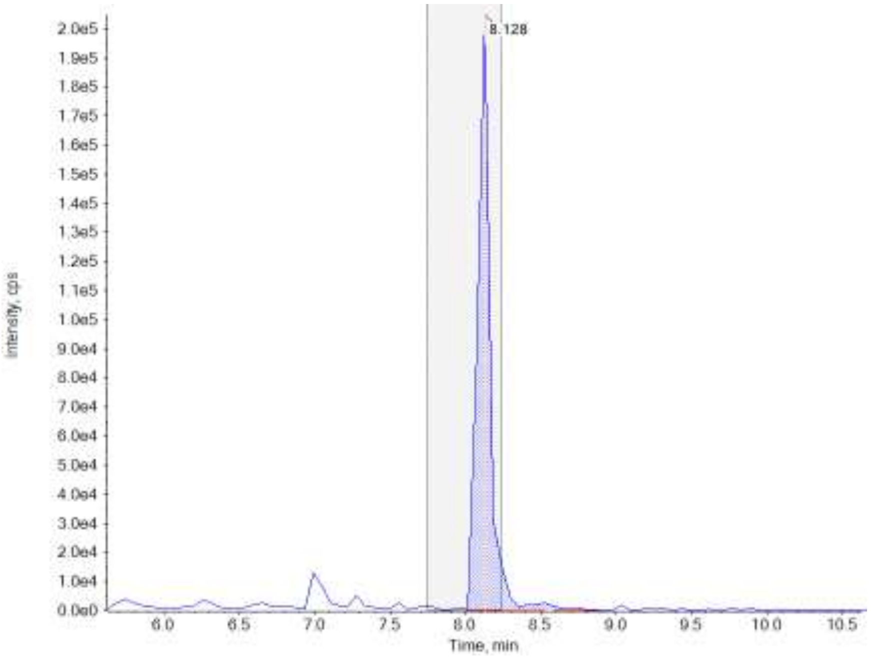

Acquired / Library MSMS

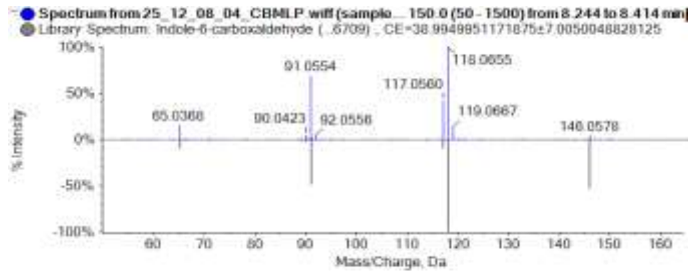

Acquired / Theoretical MS

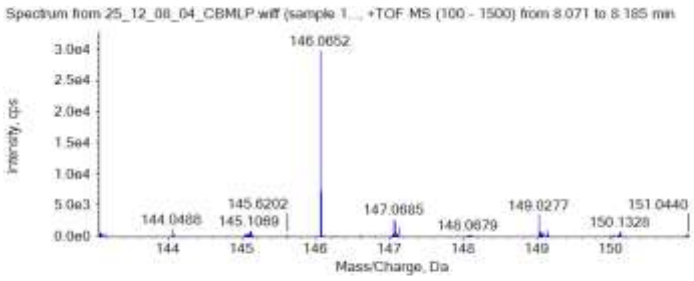

383.1756 / 8.41 (Mass/FragMass/RT/Isotope/Library/Formula/Ion Ratio)

Retention Time: 8.41 minutes
Precursor m/z : 383.1756
Fit (%) 100.0% **RFit (%)** 96.3%

Exp RT: 8.41 minutes
Analyte Name:
383.1756 / 8.41

Collision Energy = 35 ± 15 eV

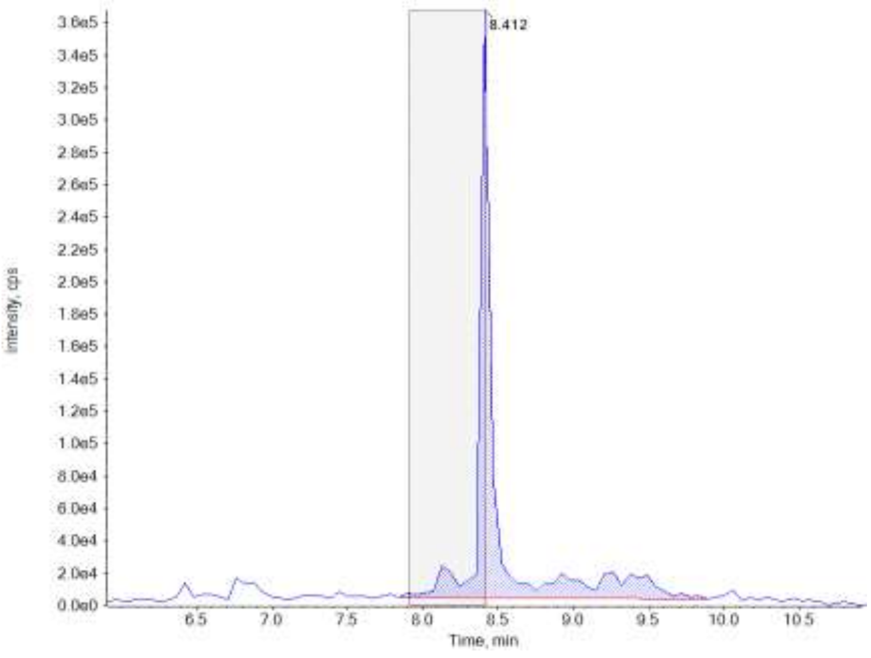

Acquired / Library MSMS

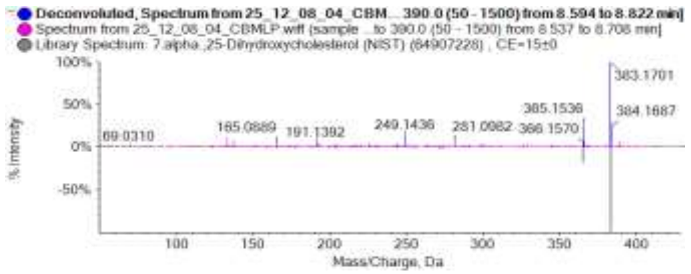

Acquired / Theoretical MS

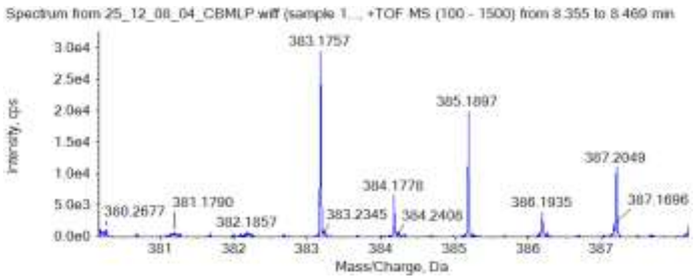

213.1540 / 8.70 (Mass/FragMass/RT/Isotope/Library/Formula/Ion Ratio)

**Retention Time:** 8.68 minutes	**Exp RT:** 8.70 minutes
**Precursor m/z :** 213.1540	**Analyte Name:**
**Fit (%)** 59.4% **RFit (%)** 87.5%	213.1540 / 8.70

Collision Energy = 35 ± 15 eV

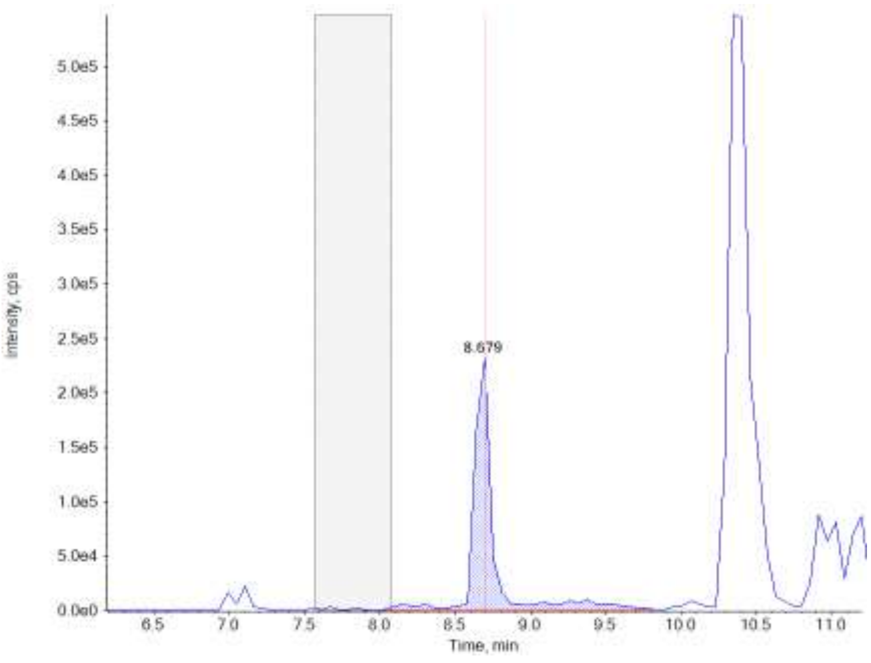

Acquired / Library MSMS

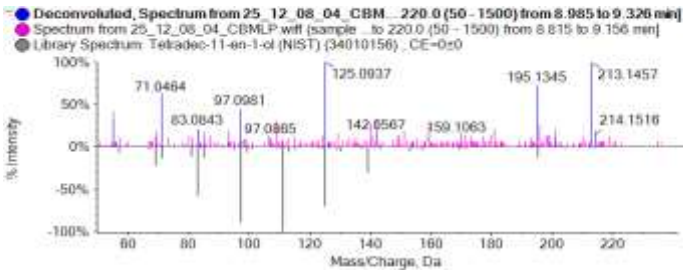

Acquired / Theoretical MS

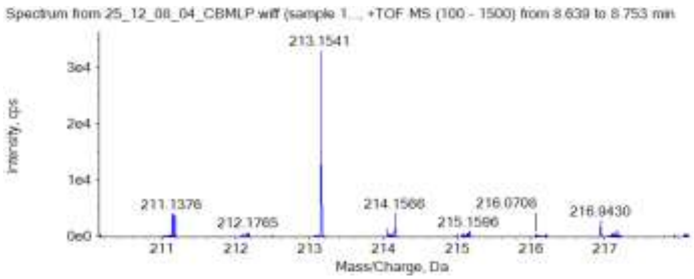

464.2799 / 8.98 (Mass/FragMass/RT/Isotope/Library/Formula/Ion Ratio)

Retention Time: 8.96 minutes
Precursor m/z : 464.2799
Fit (%) 39.1% **RFit (%)** 93.1%

Exp RT: 8.98 minutes
Analyte Name:
464.2799 / 8.98

Collision Energy = 35 ± 15 eV

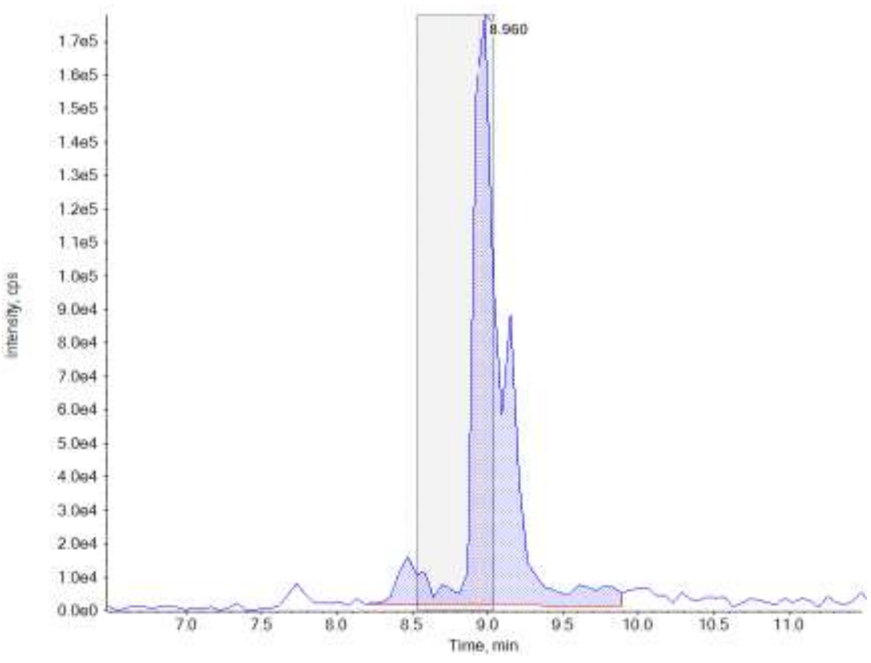

Acquired / Library MSMS

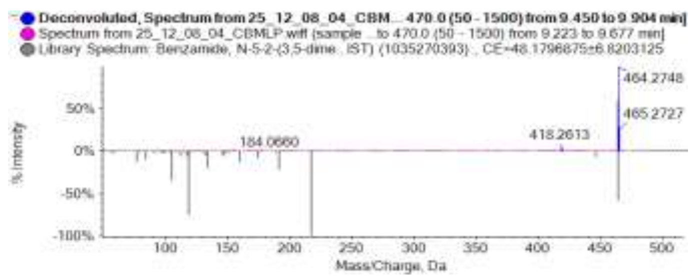

Acquired / Theoretical MS

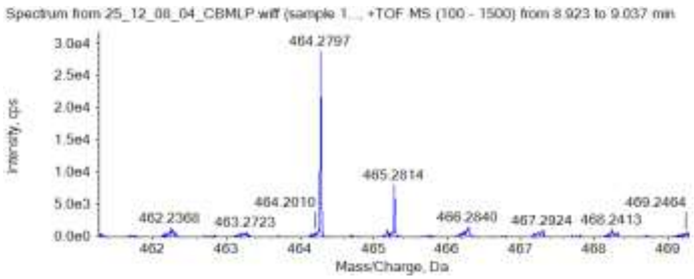

115.0807 / 9.04 (Mass/FragMass/RT/Isotope/Library/Formula/Ion Ratio)

**Retention Time:** 9.04 minutes	**Exp RT:** 9.04 minutes
**Precursor m/z :** 115.0807	**Analyte Name:**
**Fit (%)** 49.4% **RFit (%)** 100.0%	115.0807 / 9.04

Collision Energy = 35 ± 15 eV

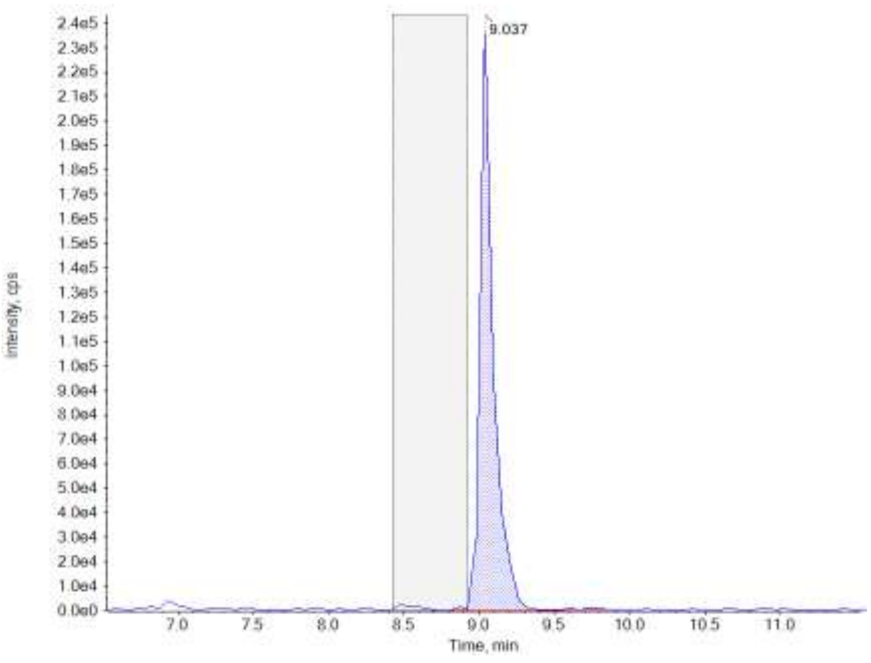

Acquired / Library MSMS

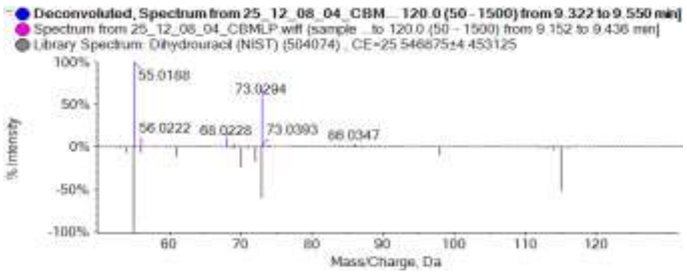

Acquired / Theoretical MS

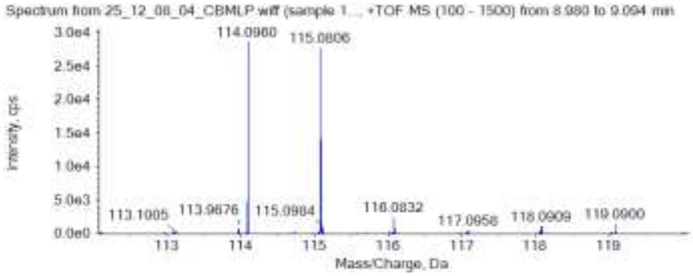

309.2108 / 9.04

(Mass/FragMass/RT/Isotope/Library/Formula/Ion Ratio)

Retention Time: 8.94 minutes
Precursor m/z : 309.2108
Fit (%) 96.8% **RFit (%)** 41.6%

Exp RT: 9.04 minutes
Analyte Name:
309.2108 / 9.04

Collision Energy = 35 ± 15 eV

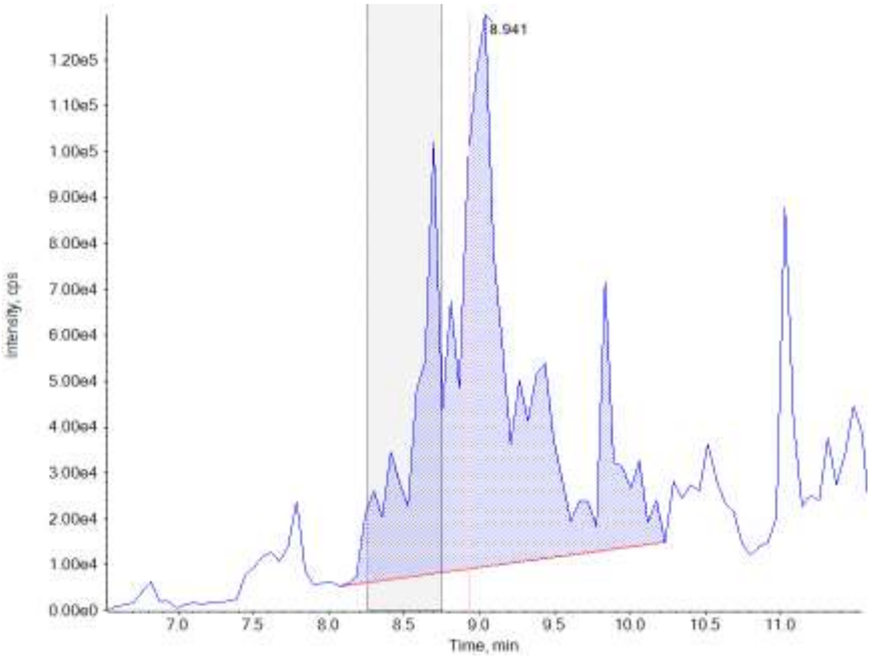

Acquired / Library MSMS

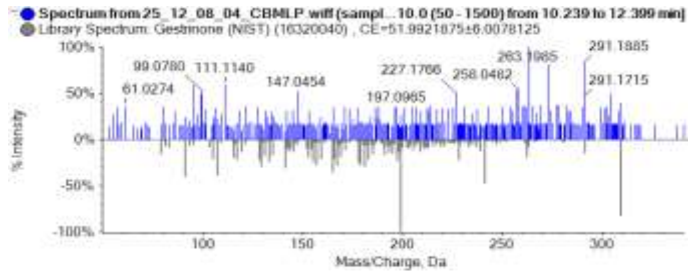

Acquired / Theoretical MS

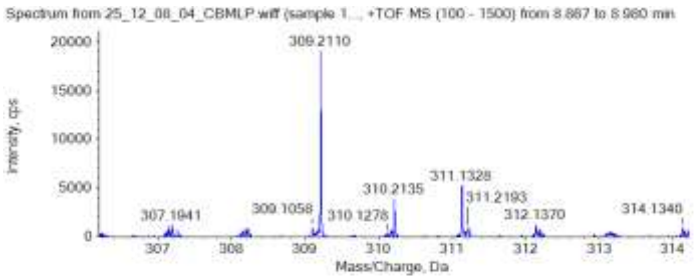

211.1384 / 9.15 (Mass/FragMass/RT/Isotope/Library/Formula/Ion Ratio)

Retention Time: 9.13 minutes
Precursor m/z : 211.1384
Fit (%) 94.3% **RFit (%)** 32.0%

Exp RT: 9.15 minutes
Analyte Name:
211.1384 / 9.15

Collision Energy = 35 ± 15 eV

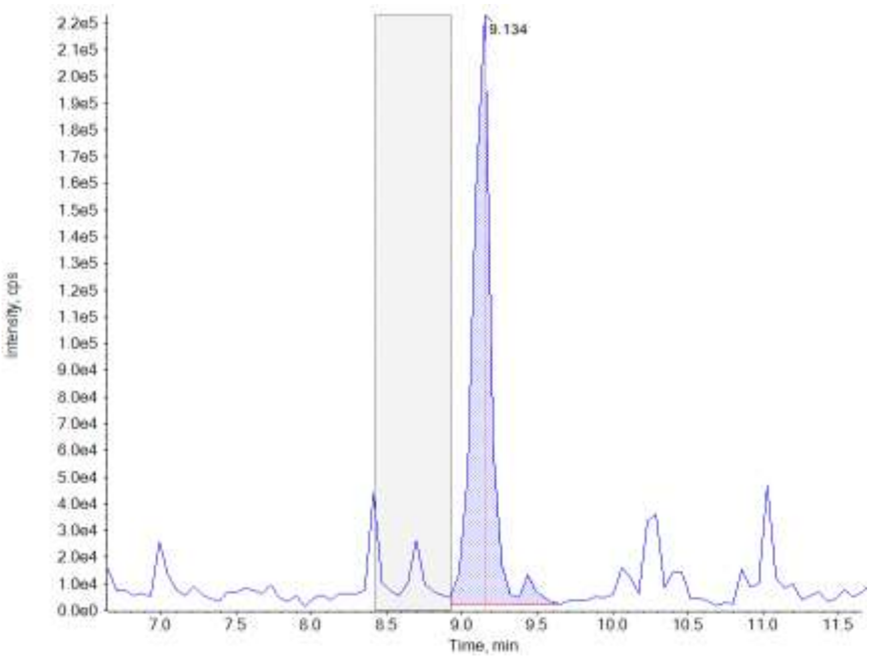

Acquired / Library MSMS

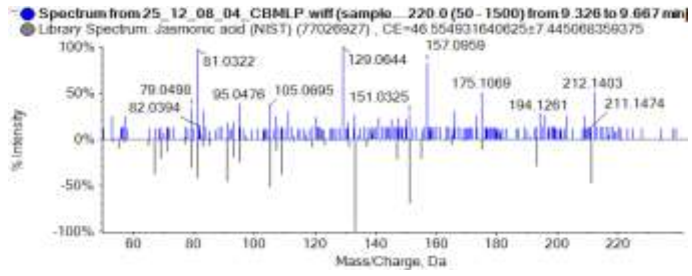

Acquired / Theoretical MS

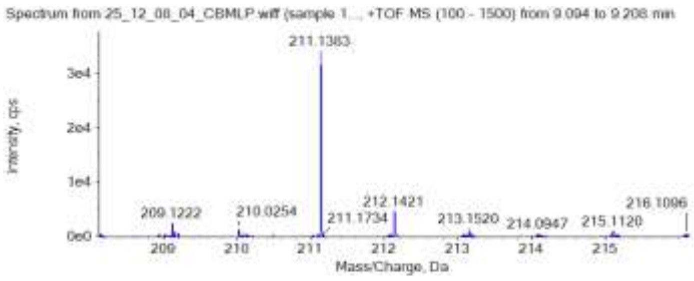

446.2808 / 9.49 (Mass/FragMass/RT/Isotope/Library/Formula/Ion Ratio)

Retention Time: 9.59 minutes **Exp RT:** 9.49 minutes
Precursor m/z : 446.2807 **Analyte Name:**
Fit (%) 78.8% **RFit (%)** 100.0% 446.2808 / 9.49

Collision Energy = 35 ± 15 eV

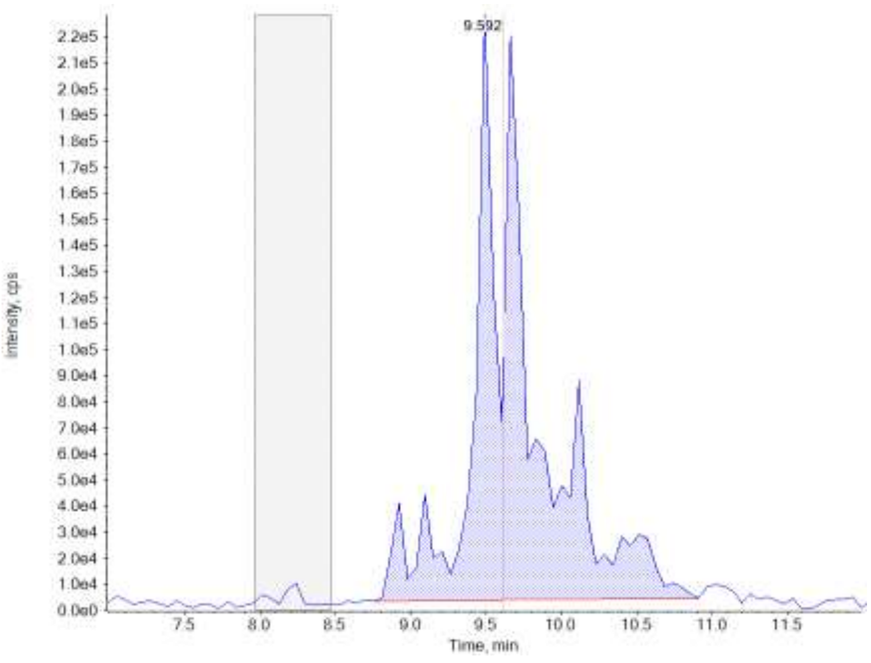

Acquired / Library MSMS

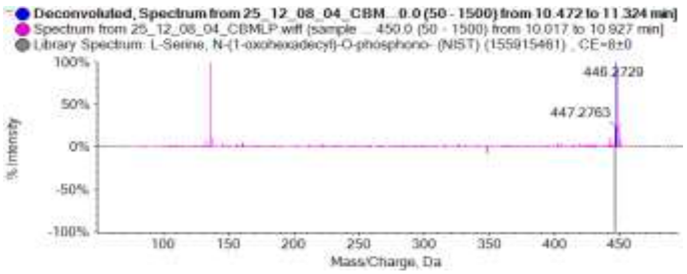

Acquired / Theoretical MS

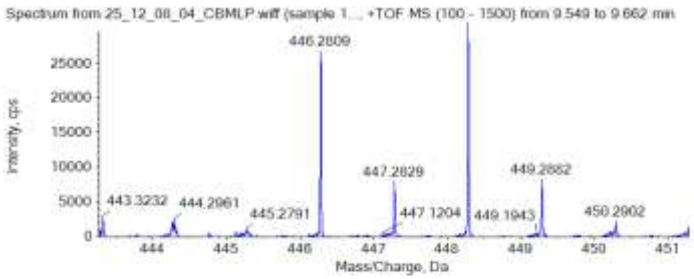

448.2860 / 9.55 (Mass/FragMass/RT/Isotope/Library/Formula/Ion Ratio)

**Retention Time:** 9.55 minutes<br>**Precursor m/z :** 448.2860<br>**Fit (%)** 99.5% **RFit (%)** 95.1%	**Exp RT:** 9.55 minutes<br>**Analyte Name:**<br>448.2860 / 9.55

Collision Energy = 35 ± 15 eV

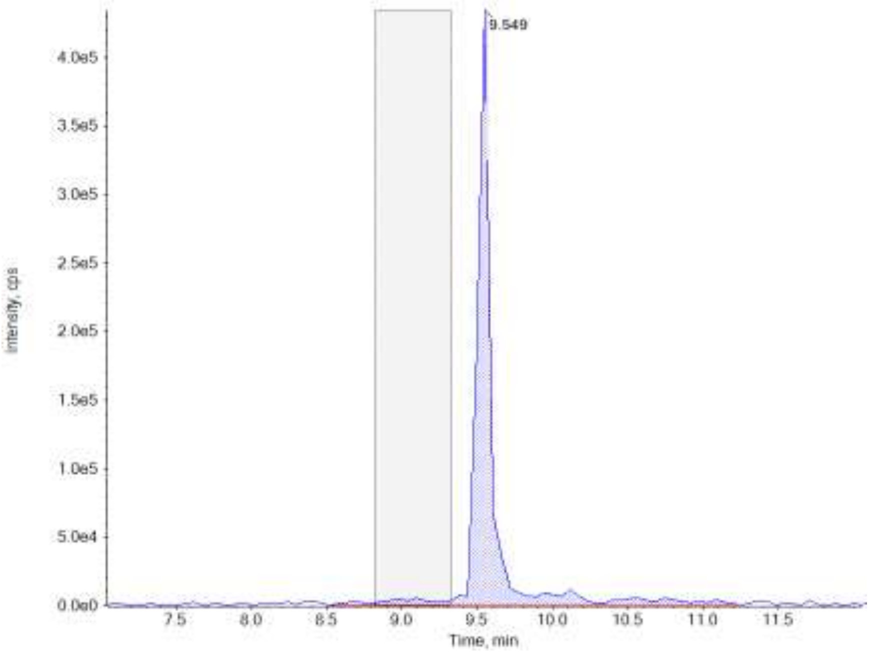

Acquired / Library MSMS

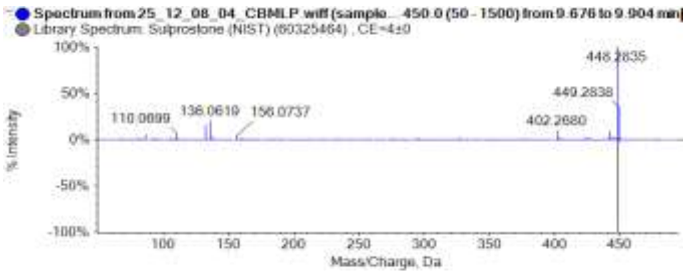

Acquired / Theoretical MS

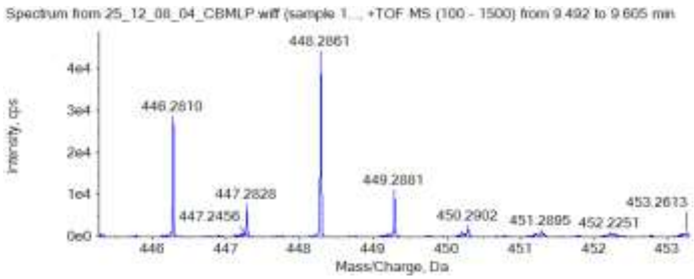

Created with SCIEX OS 3.3.0
Printed:12/9/2025 2:04:37 PM

191.1478 / 9.66 (Mass/FragMass/RT/Isotope/Library/Formula/Ion Ratio)

Retention Time: 9.69 minutes
Precursor m/z : 191.1478
Fit (%) 98.0% **RFit (%)** 64.5%

Exp RT: 9.66 minutes
Analyte Name:
191.1478 / 9.66

Collision Energy = 35 ± 15 eV

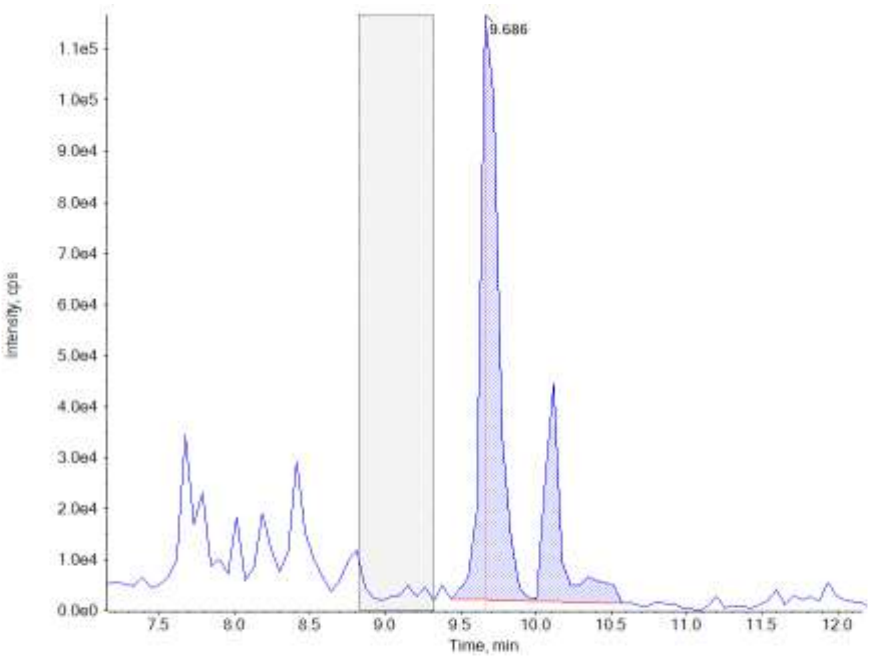

Acquired / Library MSMS

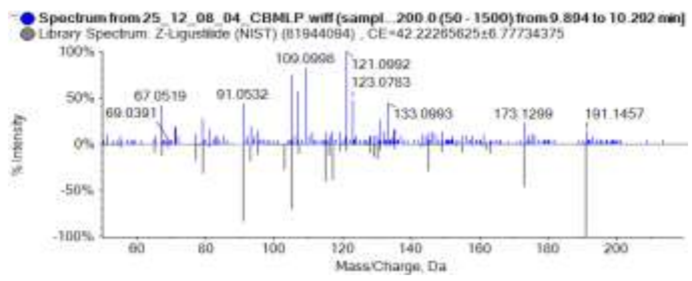

Acquired / Theoretical MS

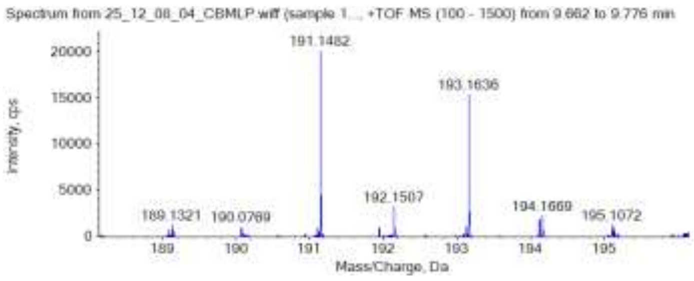

Created with SCIEX OS 3.3.0
Printed:12/9/2025 2:04:37 PM

193.1635 / 9.66 (Mass/FragMass/RT/Isotope/Library/Formula/Ion Ratio)

Retention Time: 9.63 minutes
Precursor m/z : 193.1635
Fit (%) 98.8% **RFit (%)** 83.4%

Exp RT: 9.66 minutes
Analyte Name:
193.1635 / 9.66

Collision Energy = 35 ± 15 eV

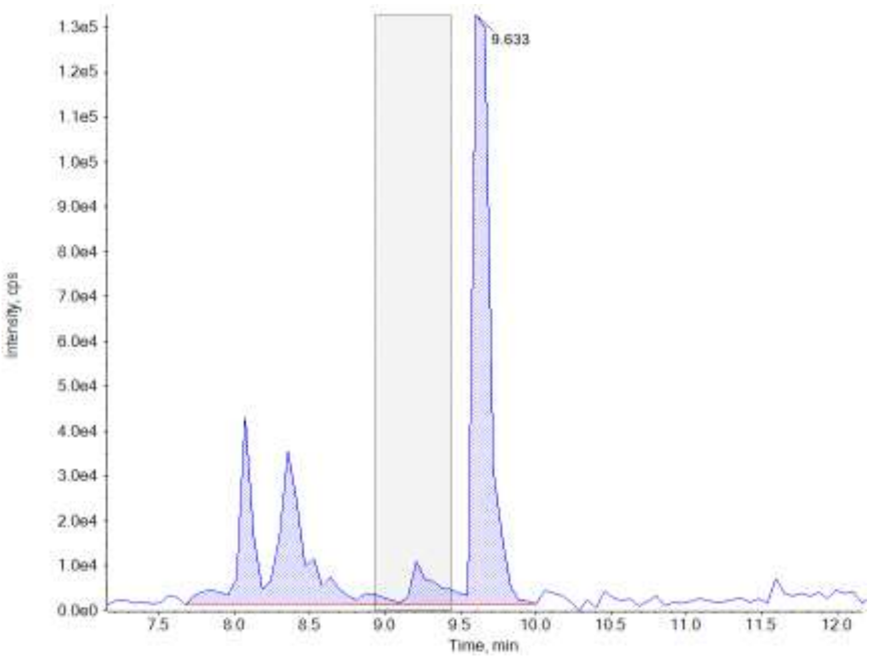

Acquired / Library MSMS

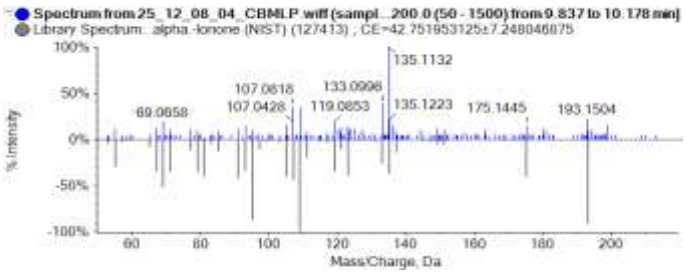

Acquired / Theoretical MS

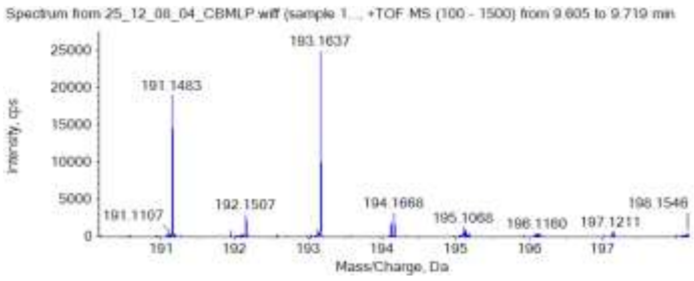

211.1746 / 9.66 (Mass/FragMass/RT/Isotope/Library/Formula/Ion Ratio)

**Retention Time:** 9.64 minutes	**Exp RT:** 9.66 minutes
**Precursor m/z :** 211.1746	**Analyte Name:**
**Fit (%)** 87.9% **RFit (%)** 91.3%	211.1746 / 9.66

Collision Energy = 35 ± 15 eV

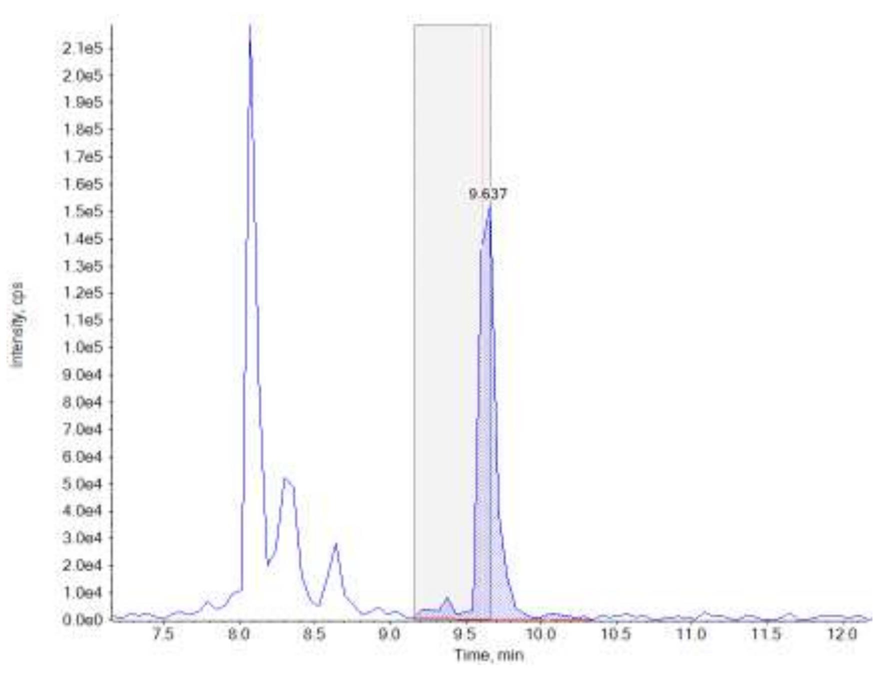

Acquired / Library MSMS

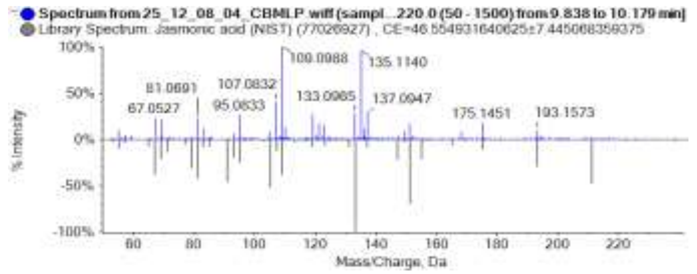

Acquired / Theoretical MS

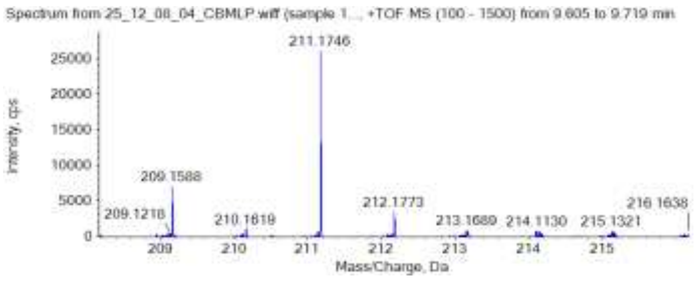

Created with SCIEX OS 3.3.0
Printed:12/9/2025 2:04:37 PM

306.2698 / 10.12 (Mass/FragMass/RT/Isotope/Library/Formula/Ion Ratio)

Retention Time: 10.05 minutes
Precursor m/z : 306.2698
Fit (%) 25.2% **RFit (%)** 99.5%

Exp RT: 10.12 minutes
Analyte Name:
306.2698 / 10.12

Collision Energy = 35 ± 15 eV

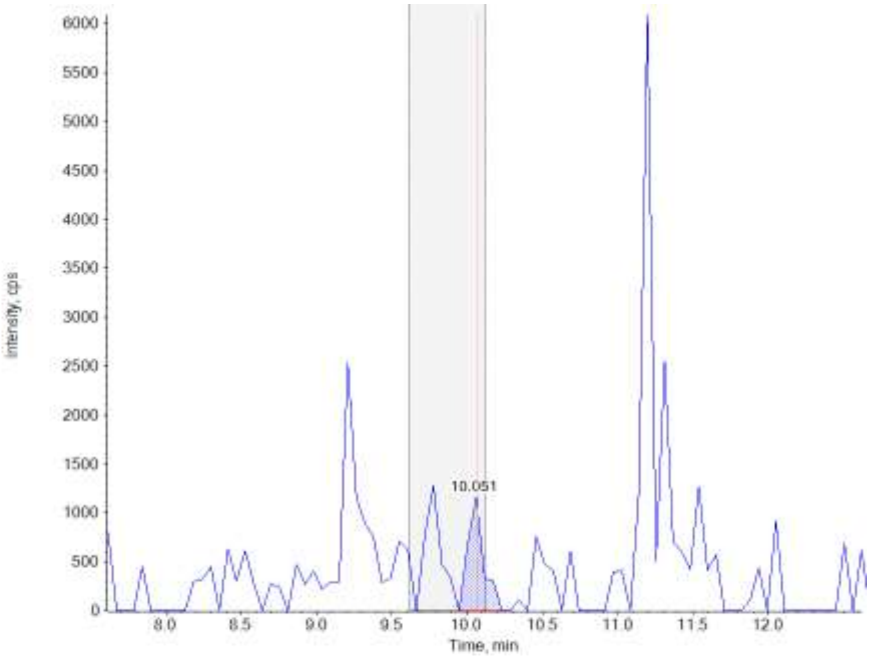

Acquired / Library MSMS

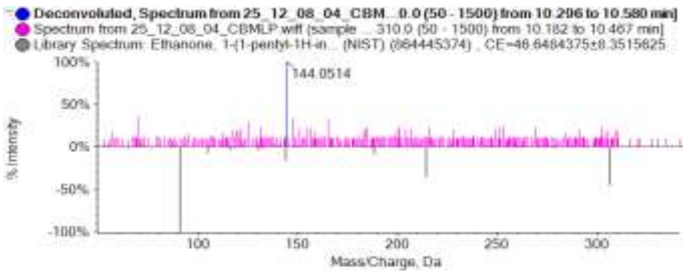

Acquired / Theoretical MS

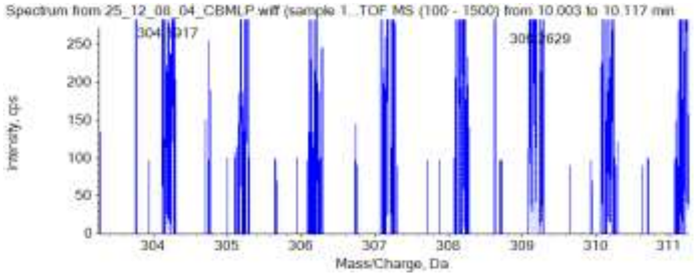

275.2089 / 10.34 (Mass/FragMass/RT/Isotope/Library/Formula/Ion Ratio)

**Retention Time:** 10.36 minutes	**Exp RT:** 10.34 minutes
**Precursor m/z :** 275.2089	**Analyte Name:**
**Fit (%)** 68.3% **RFit (%)** 99.5%	275.2089 / 10.34

Collision Energy = 35 ± 15 eV

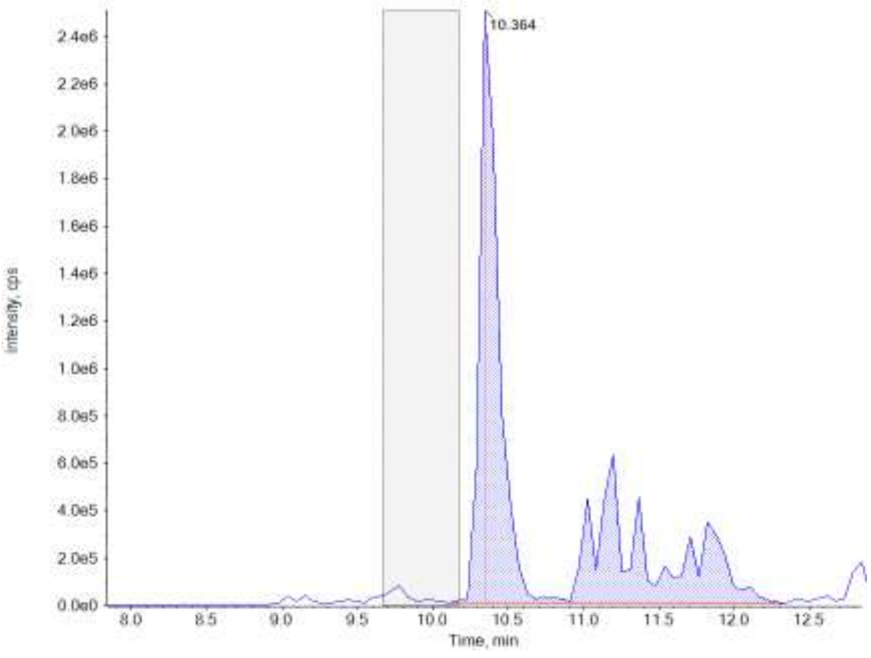

Acquired / Library MSMS

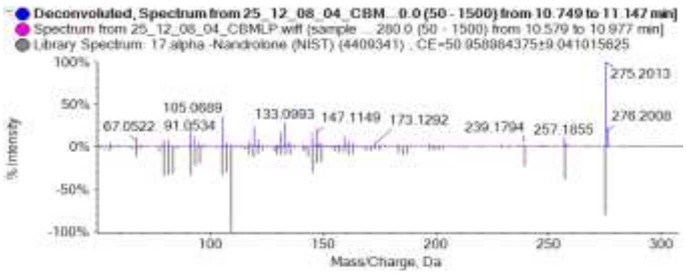

Acquired / Theoretical MS

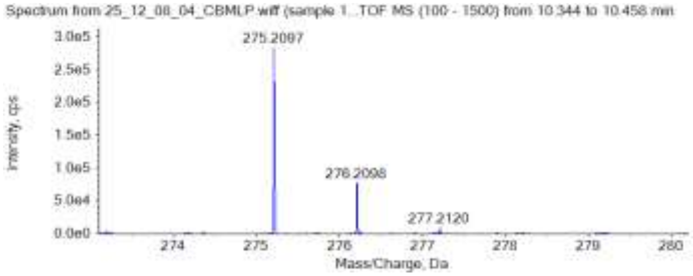

293.2195 / 10.34

(Mass/FragMass/RT/Isotope/Library/Formula/Ion Ratio)

**Retention Time:** 10.36 minutes	**Exp RT:** 10.34 minutes
**Precursor m/z :** 293.2195	**Analyte Name:**
**Fit (%)** 87.6% **RFit (%)** 92.9%	293.2195 / 10.34

Collision Energy = 35 ± 15 eV

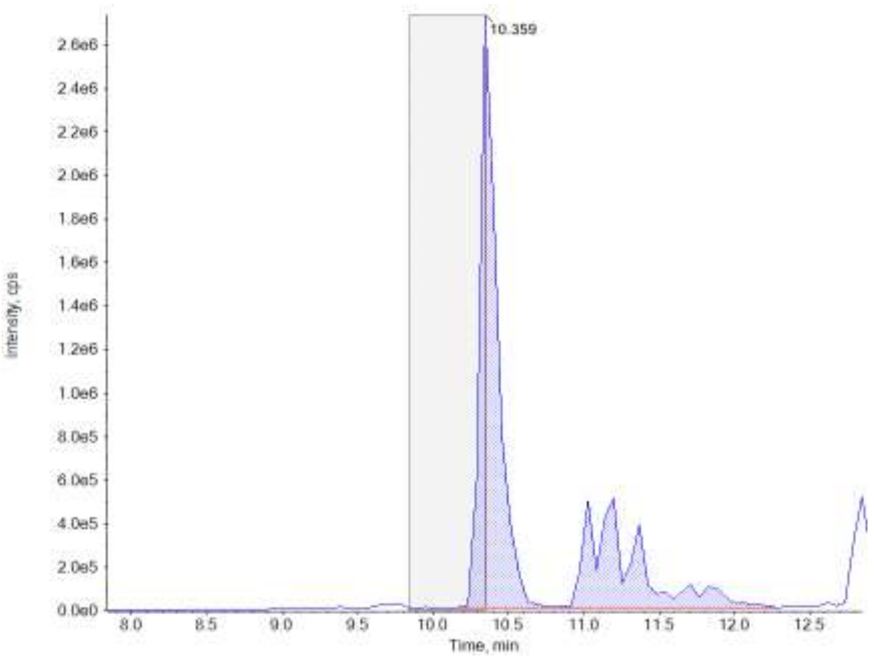

Acquired / Library MSMS

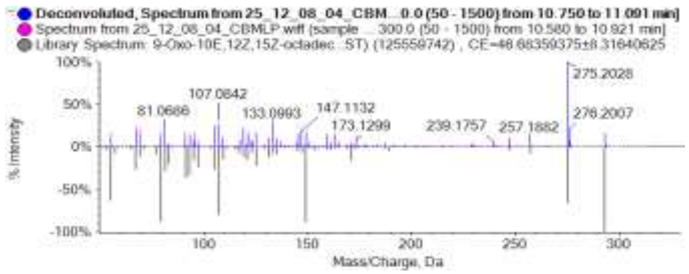

Acquired / Theoretical MS

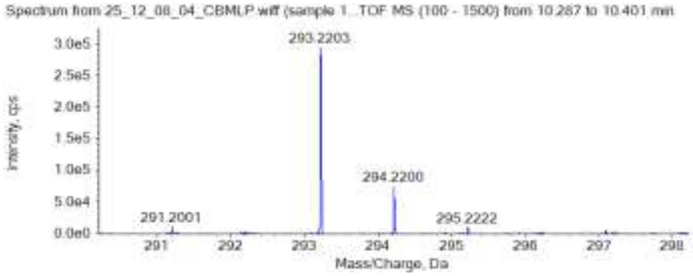

311.2273 / 10.34 (Mass/FragMass/RT/Isotope/Library/Formula/Ion Ratio)

Retention Time: 10.35 minutes
Precursor m/z : 311.2273
Fit (%) 80.9% **RFit (%)** 68.2%

Exp RT: 10.34 minutes
Analyte Name:
311.2273 / 10.34

Collision Energy = 35 ± 15 eV

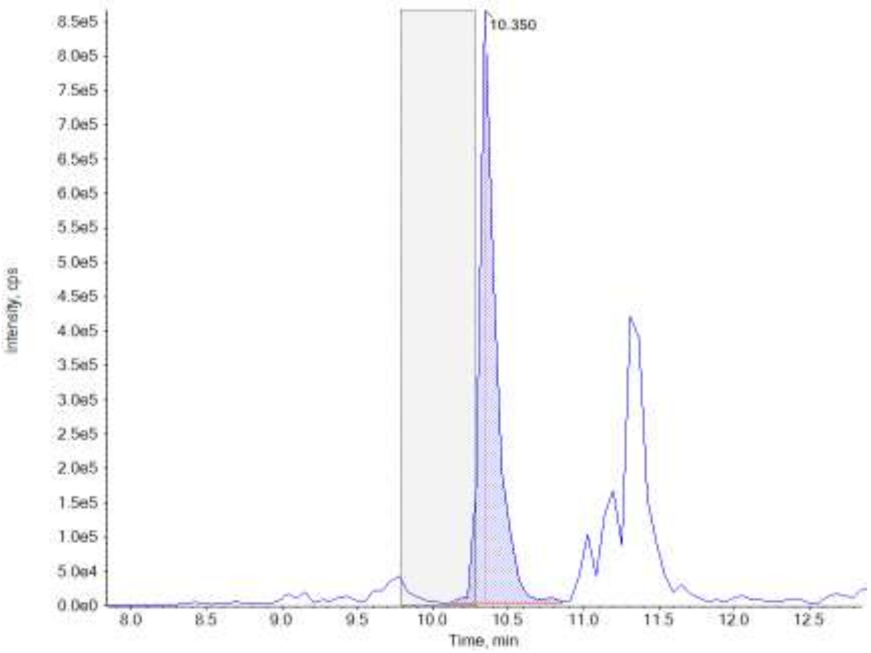

Acquired / Library MSMS

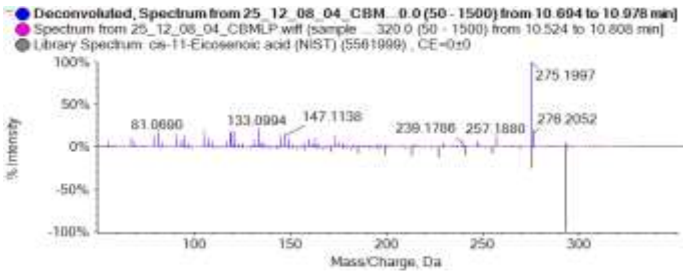

Acquired / Theoretical MS

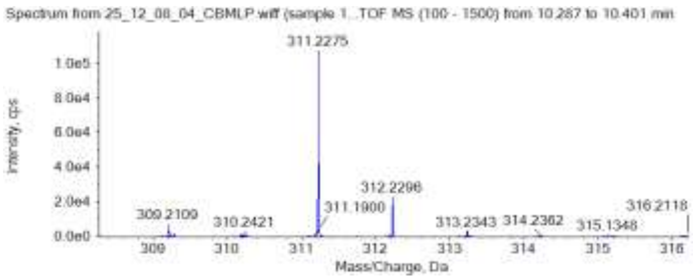

195.1429 / 10.40

(Mass/FragMass/RT/Isotope/Library/Formula/Ion Ratio)

Retention Time: 10.38 minutes
Precursor m/z : 195.1429
Fit (%) 75.1% **RFit (%)** 91.2%

Exp RT: 10.40 minutes
Analyte Name:
195.1429 / 10.40

Collision Energy = 35 ± 15 eV

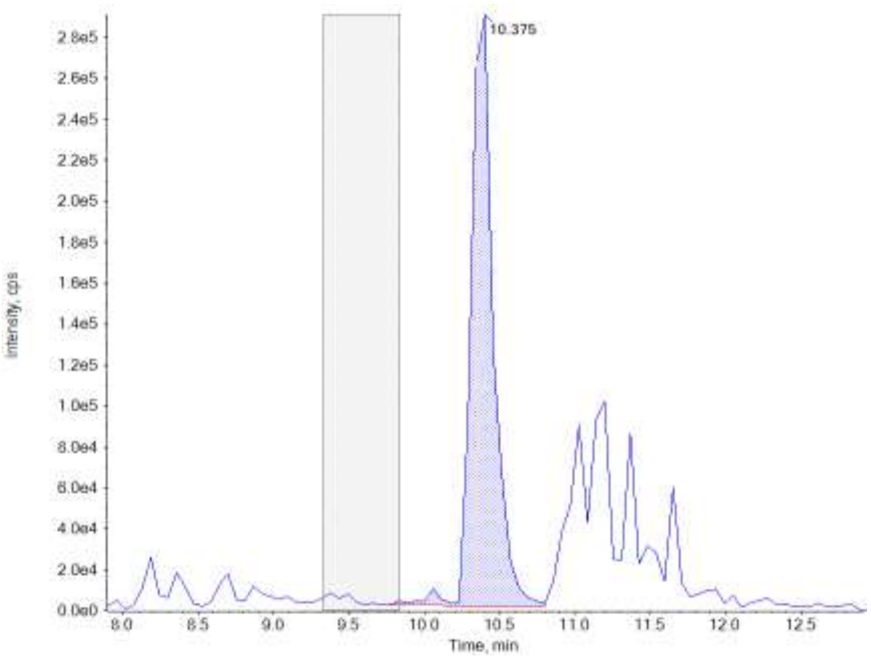

Acquired / Library MSMS

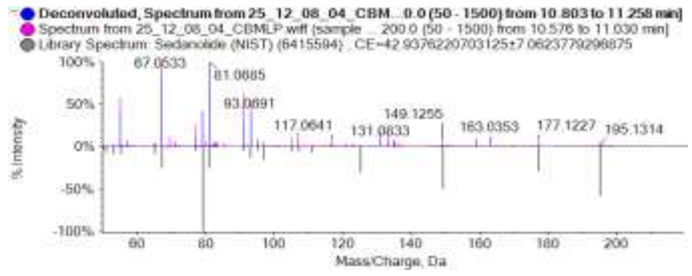

Acquired / Theoretical MS

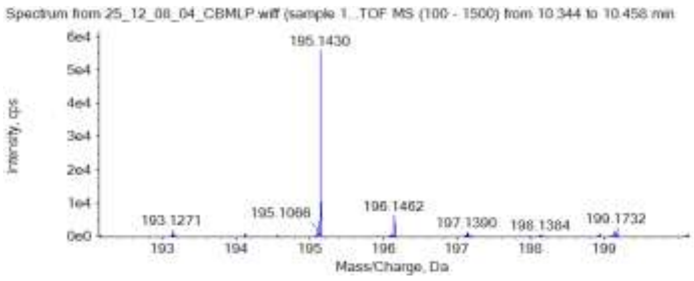

325.2329 / 10.51 (Mass/FragMass/RT/Isotope/Library/Formula/Ion Ratio)

**Retention Time:** 10.33 minutes	**Exp RT:** 10.52 minutes
**Precursor m/z :** 325.2329	**Analyte Name:**
**Fit (%)** 73.9% **RFit (%)** 76.9%	325.2329 / 10.51

Collision Energy = 35 ± 15 eV

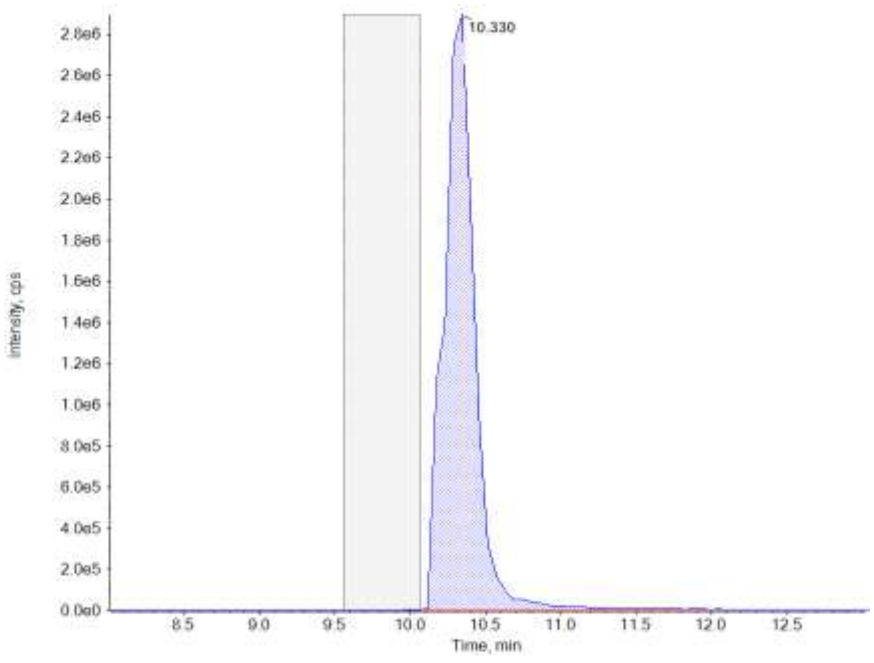

Acquired / Library MSMS

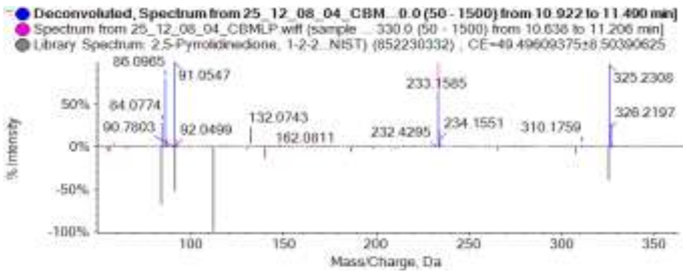

Acquired / Theoretical MS

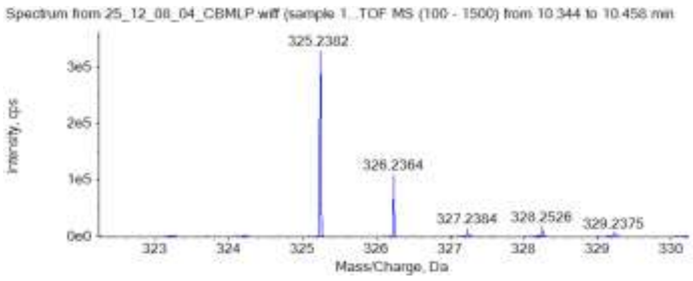

731.4532 / 10.74 [M+H]+ (Mass/FragMass/RT/Isotope/Library/Formula/Ion Ratio)

Retention Time: 10.73 minutes
Precursor m/z : 731.4532
Fit (%) 73.3% **RFit (%)** 74.5%

Exp RT: 10.74 minutes
Analyte Name:
731.4532 / 10.74 [M+H]+

Collision Energy = 35 ± 15 eV

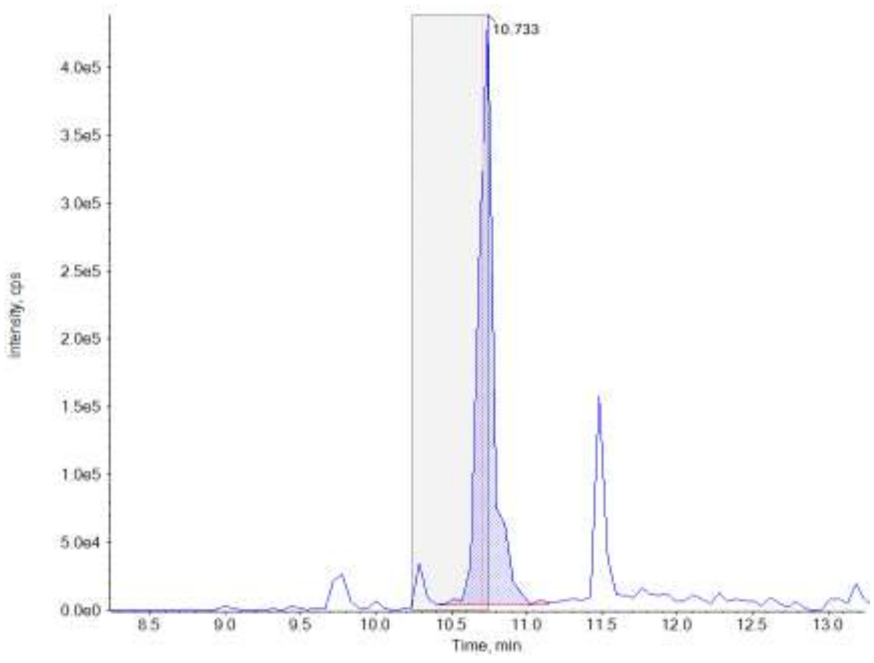

Acquired / Library MSMS

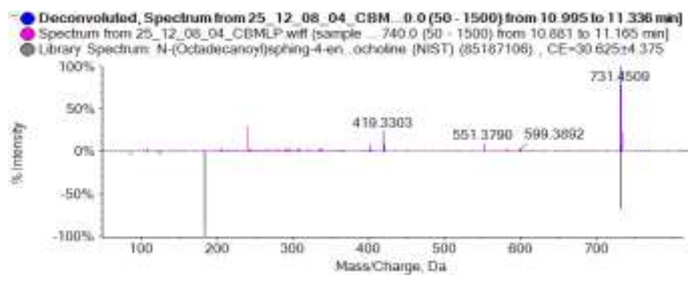

Acquired / Theoretical MS

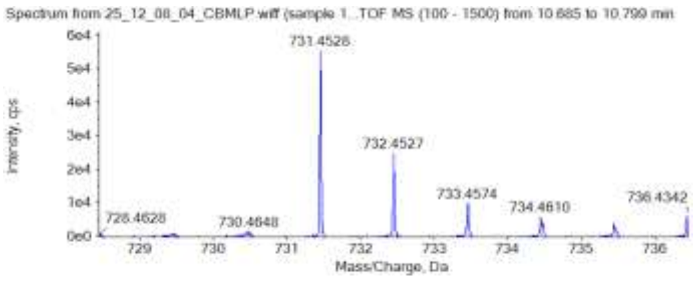

769.4763 / 11.20 (Mass/FragMass/RT/Isotope/Library/Formula/Ion Ratio)

Retention Time: 11.23 minutes
Precursor m/z : 769.4763
Fit (%) 91.4% **RFit (%)** 99.8%

Exp RT: 11.20 minutes
Analyte Name:
769.4763 / 11.20

Collision Energy = 35 ± 15 eV

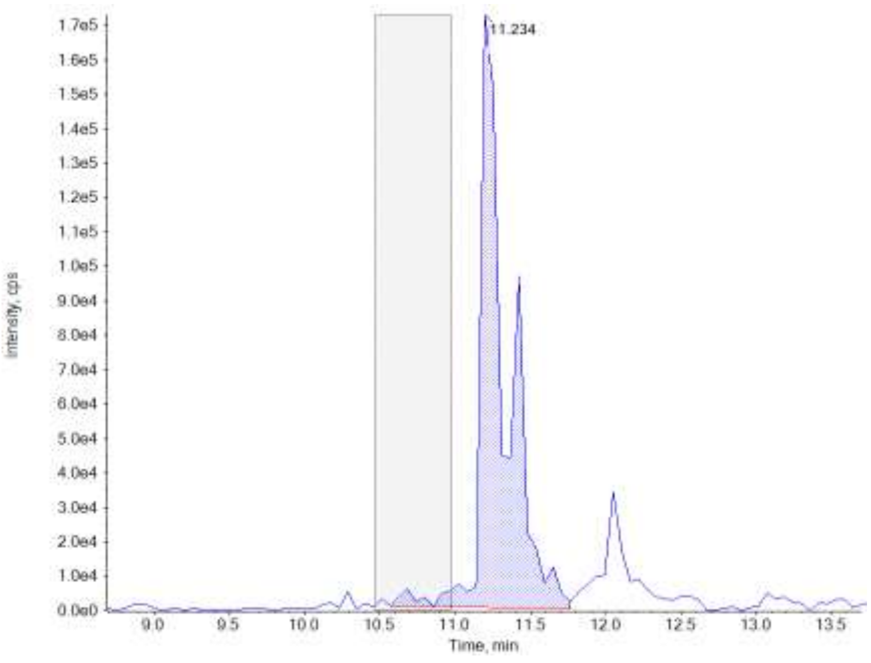

Acquired / Library MSMS

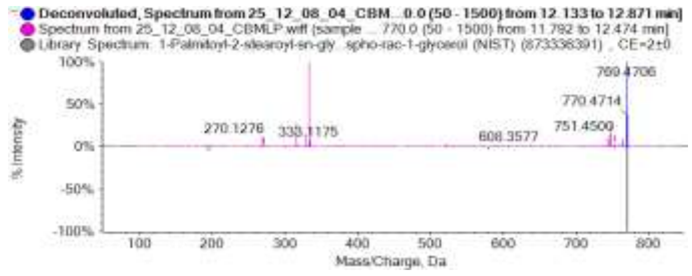

Acquired / Theoretical MS

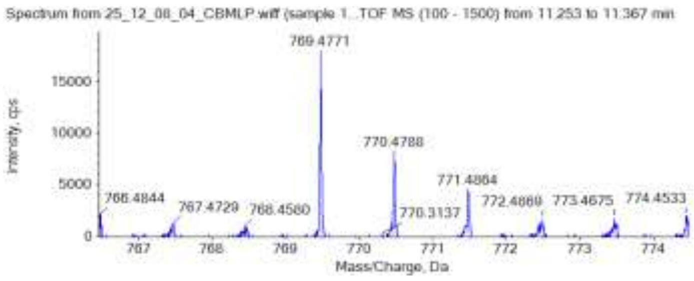

311.2275 / 11.31 (Mass/FragMass/RT/Isotope/Library/Formula/Ion Ratio)

Retention Time: 11.34 minutes
Precursor m/z : 311.2275
Fit (%) 72.0% **RFit (%)** 92.4%

Exp RT: 11.31 minutes
Analyte Name:
311.2275 / 11.31

Collision Energy = 35 ± 15 eV

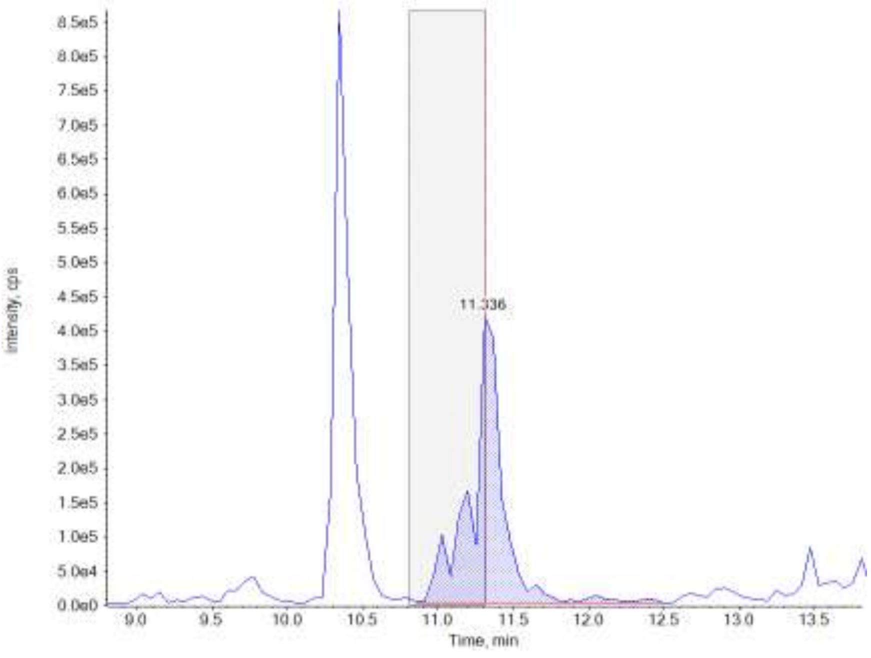

Acquired / Library MSMS

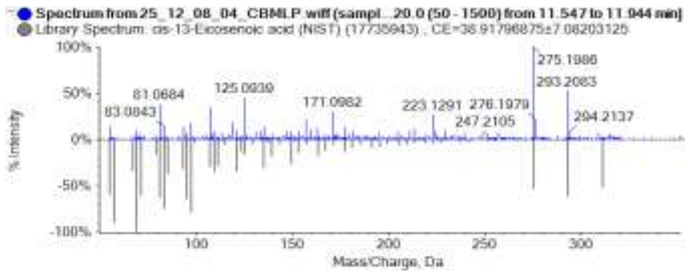

Acquired / Theoretical MS

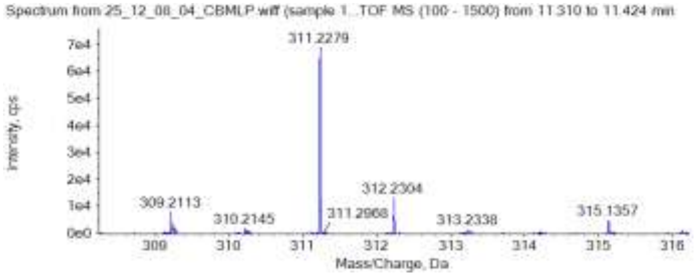

181.1279 / 11.59 (Mass/FragMass/RT/Isotope/Library/Formula/Ion Ratio)

Retention Time: 11.59 minutes
Precursor m/z : 181.1279
Fit (%) 95.8% **RFit (%)** 94.6%

Exp RT: 11.59 minutes
Analyte Name:
181.1279 / 11.59

Collision Energy = 35 ± 15 eV

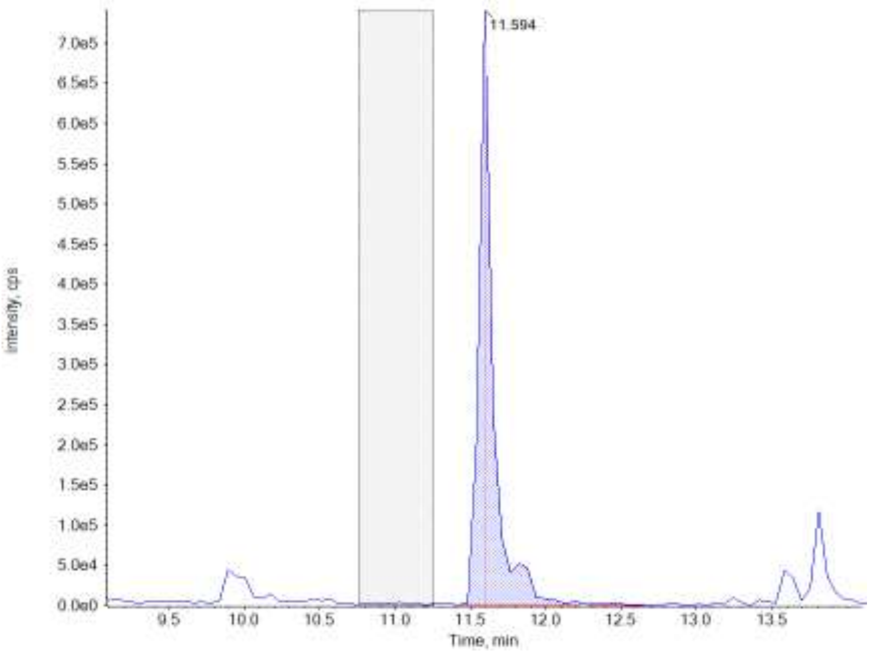

Acquired / Library MSMS

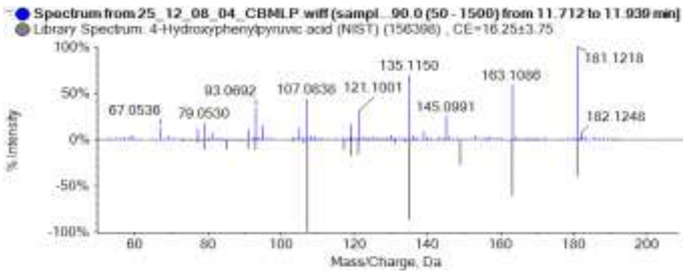

Acquired / Theoretical MS

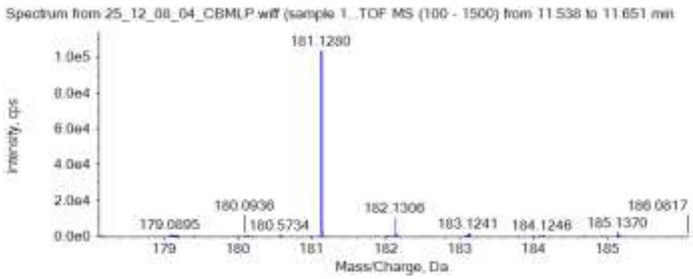

Created with SCIEX OS 3.3.0
Printed:12/9/2025 2:04:37 PM

391.3413 / 11.59 (Mass/FragMass/RT/Isotope/Library/Formula/Ion Ratio)

Retention Time: 11.59 minutes
Precursor m/z : 391.3413
Fit (%) 100.0% **RFit (%)** 63.1%

Exp RT: 11.59 minutes
Analyte Name:
391.3413 / 11.59

Collision Energy = 35 ± 15 eV

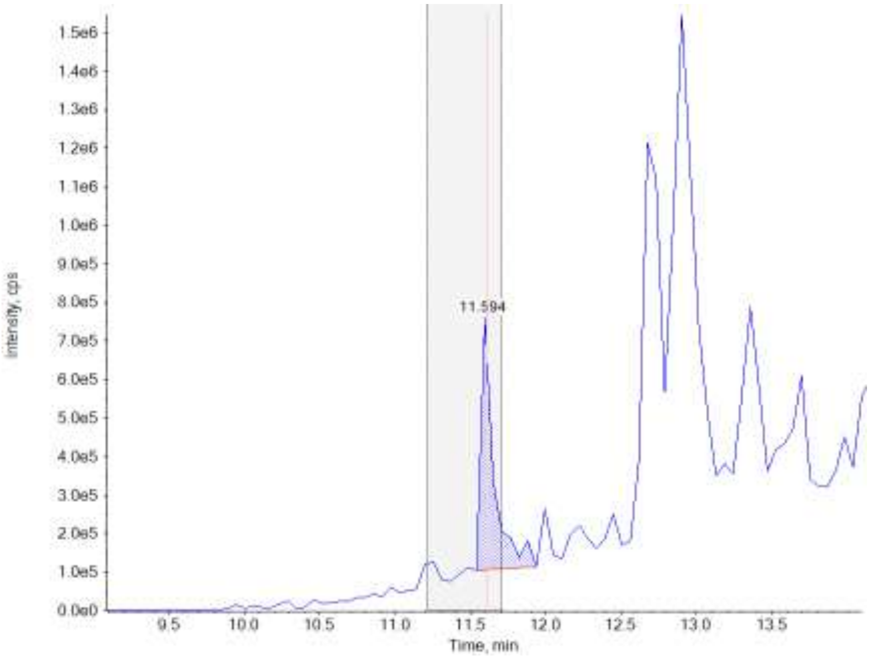

Acquired / Library MSMS

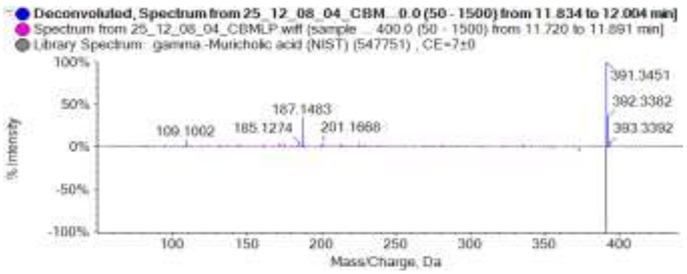

Acquired / Theoretical MS

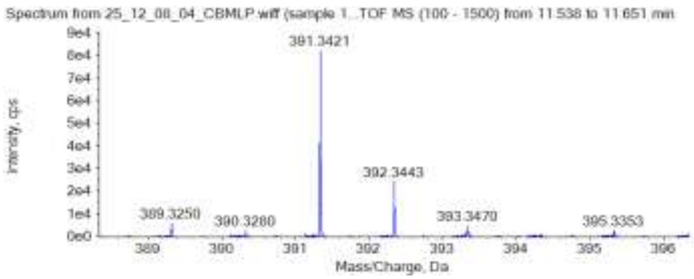

Created with SCIEX OS 3.3.0
Printed:12/9/2025 2:04:37 PM

409.3522 / 11.59

(Mass/FragMass/RT/Isotope/Library/Formula/Ion Ratio)

Retention Time: 11.59 minutes
Precursor m/z : 409.3522
Fit (%) 92.1% **RFit (%)** 69.3%

Exp RT: 11.59 minutes
Analyte Name:
409.3522 / 11.59

Collision Energy = 35 ± 15 eV

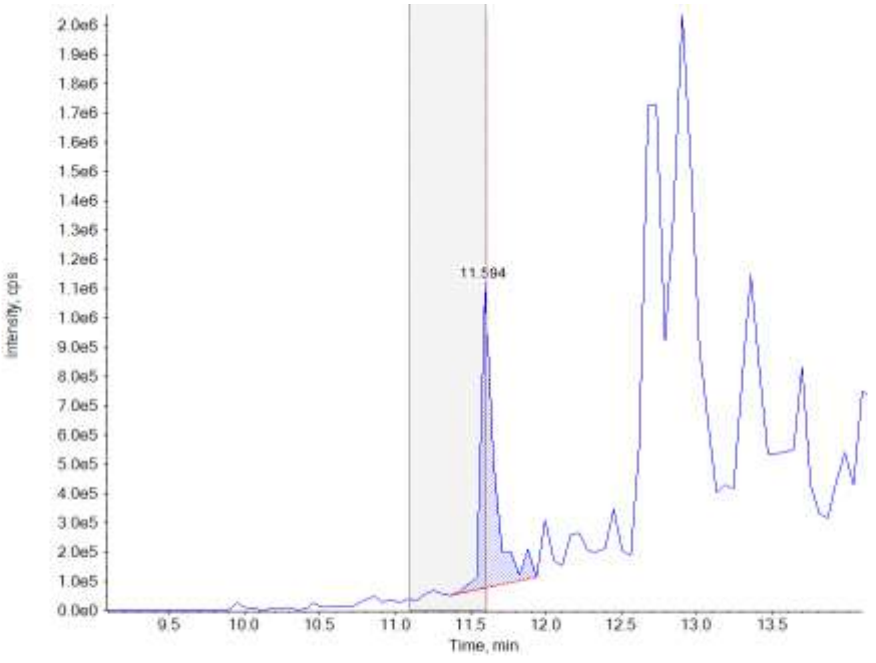

Acquired / Library MSMS

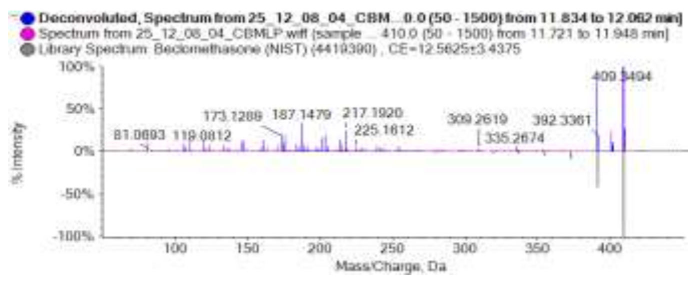

Acquired / Theoretical MS

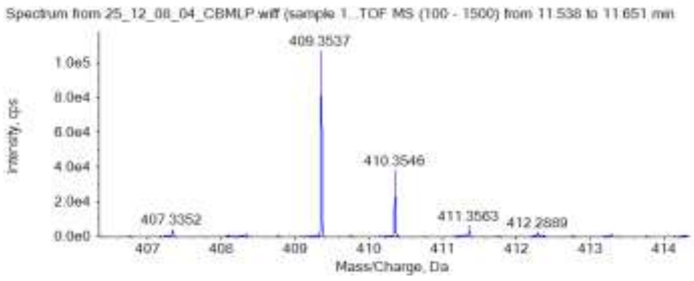

453.3405 / 11.71

(Mass/FragMass/RT/Isotope/Library/Formula/Ion Ratio)

Retention Time: 11.76 minutes
Precursor m/z : 453.3405
Fit (%) 100.0% **RFit (%)** 50.1%

Exp RT: 11.71 minutes
Analyte Name:
453.3405 / 11.71

Collision Energy = 35 ± 15 eV

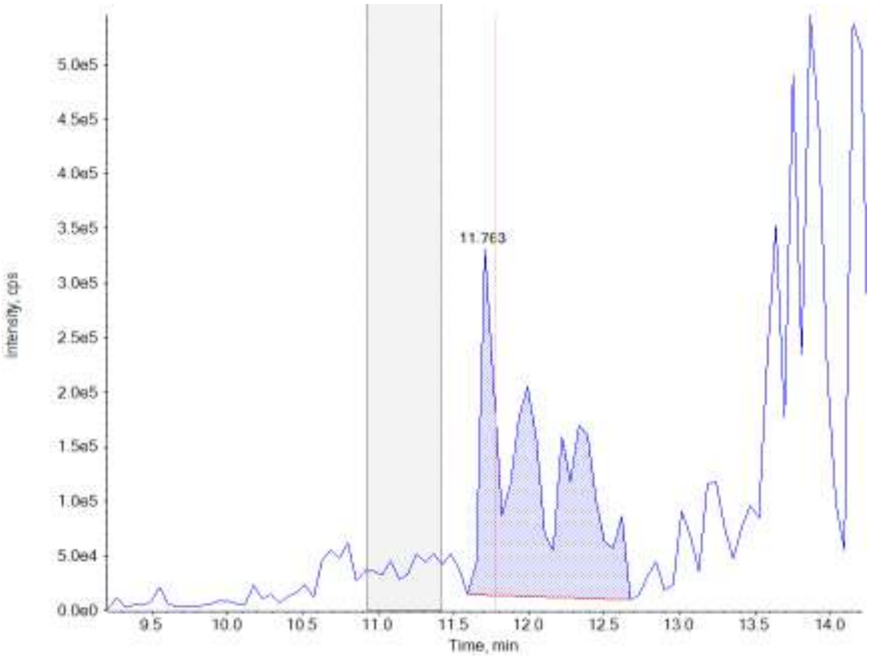

Acquired / Library MSMS

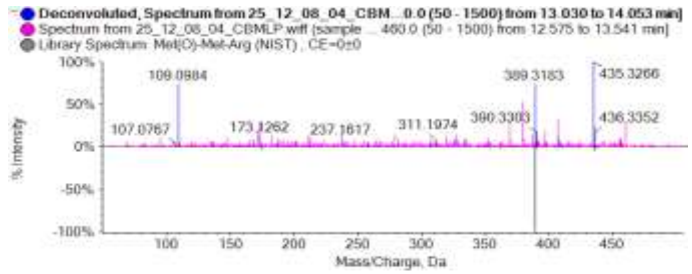

Acquired / Theoretical MS

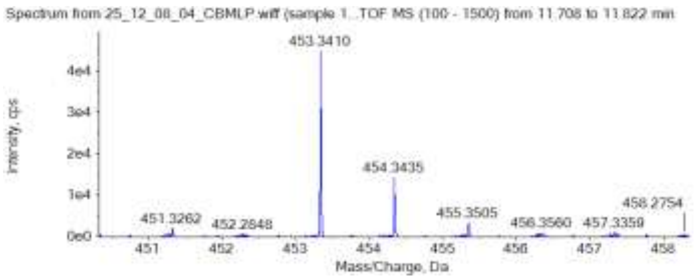

Created with SCIEX OS 3.3.0
Printed:12/9/2025 2:04:37 PM

428.2707 / 11.76 (Mass/FragMass/RT/Isotope/Library/Formula/Ion Ratio)

Retention Time: 11.76 minutes
Precursor m/z : 428.2707
Fit (%) 95.6% **RFit (%)** 96.3%

Exp RT: 11.77 minutes
Analyte Name:
428.2707 / 11.76

Collision Energy = 35 ± 15 eV

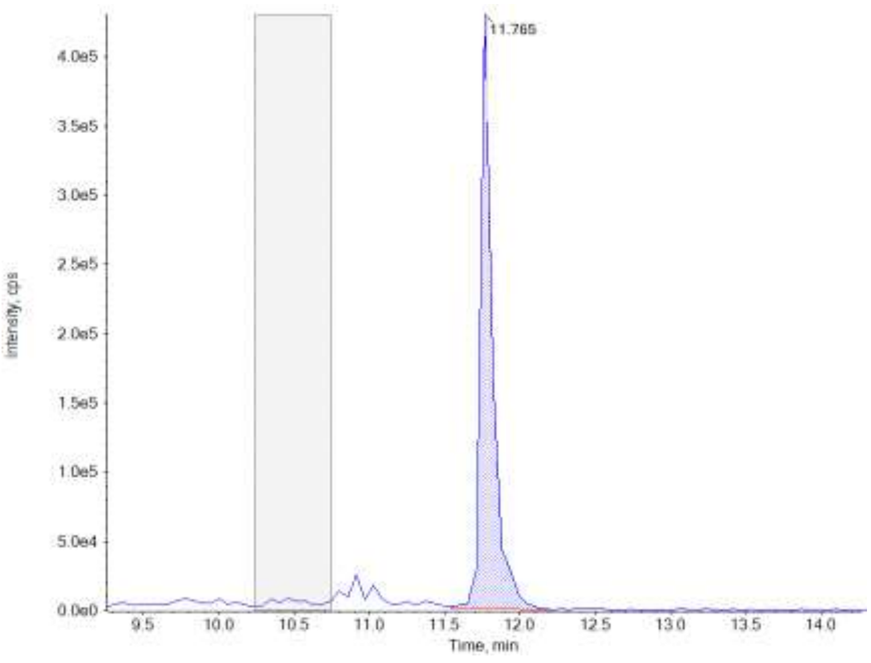

Acquired / Library MSMS

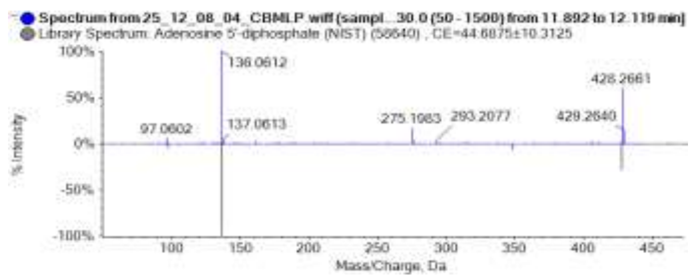

Acquired / Theoretical MS

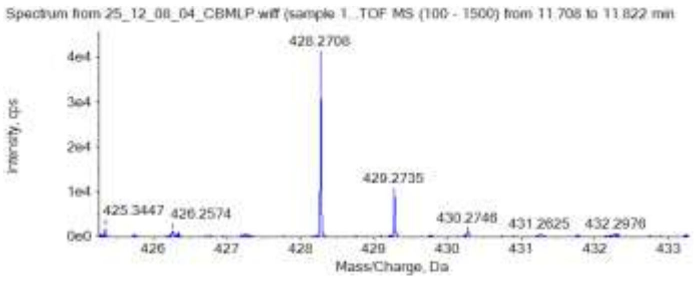

291.2022 / 12.22 (Mass/FragMass/RT/Isotope/Library/Formula/Ion Ratio)

Retention Time: 12.22 minutes
Precursor m/z : 291.2022
Fit (%) 87.4% **RFit (%)** 86.5%

Exp RT: 12.22 minutes
Analyte Name:
291.2022 / 12.22

Collision Energy = 35 ± 15 eV

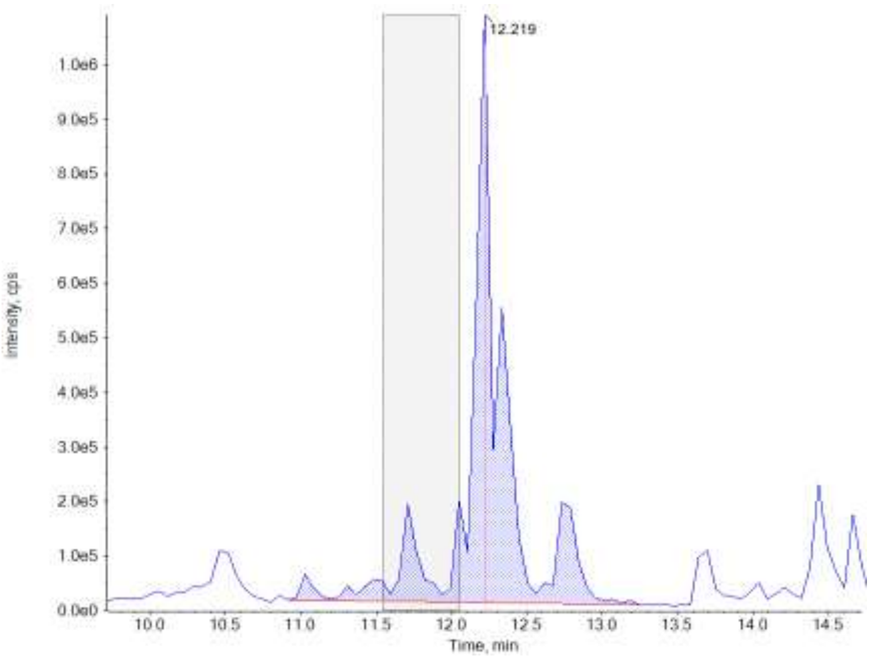

Acquired / Library MSMS

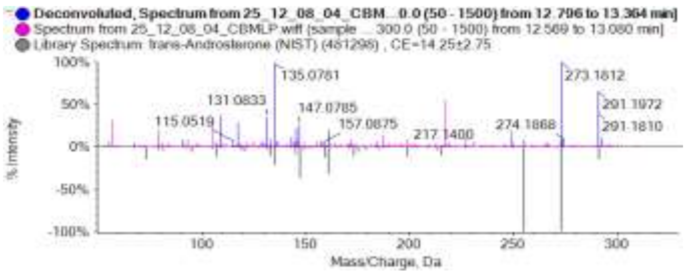

Acquired / Theoretical MS

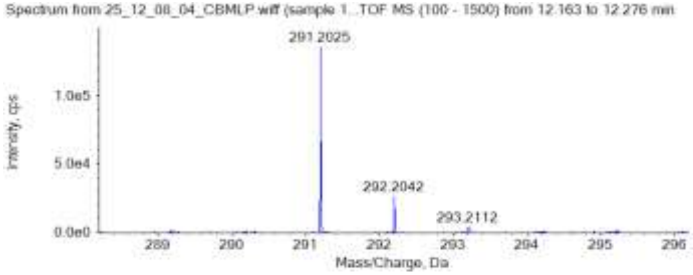

498.3979 / 12.33

(Mass/FragMass/RT/Isotope/Library/Formula/Ion Ratio)

Retention Time: 12.33 minutes
Precursor m/z : 498.3979
Fit (%) 80.5% **RFit (%)** 100.0%

Exp RT: 12.33 minutes
Analyte Name:
498.3979 / 12.33

Collision Energy = 35 ± 15 eV

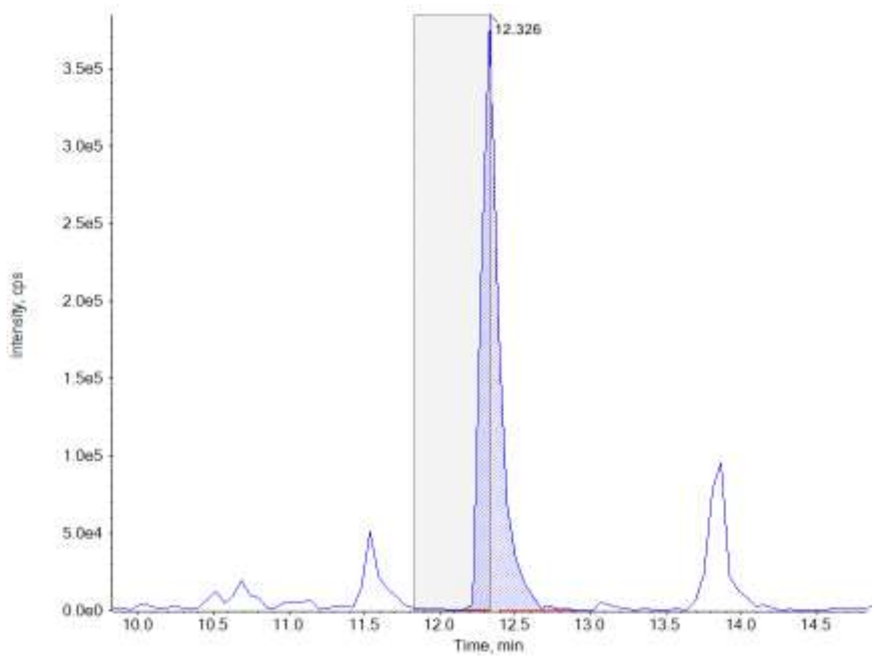

Acquired / Library MSMS

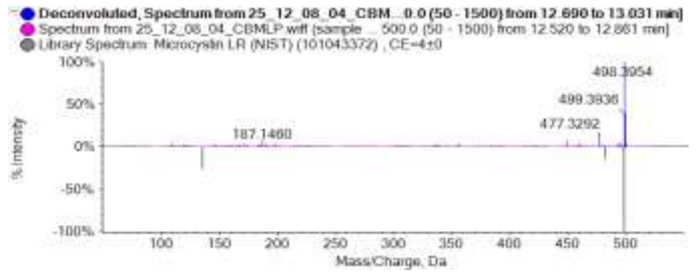

Acquired / Theoretical MS

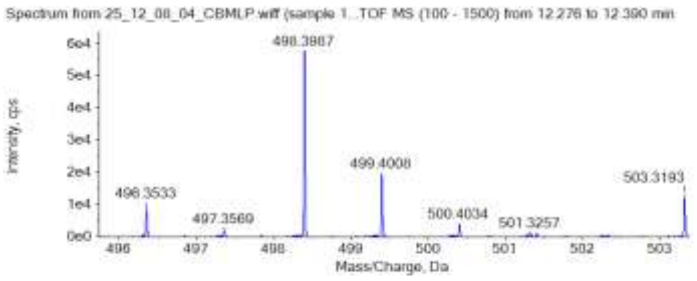

583.4145 / 12.45

(Mass/FragMass/RT/Isotope/Library/Formula/Ion Ratio)

Retention Time: 12.46 minutes
Precursor m/z : 583.4145
Fit (%) 100.0% **RFit (%)** 87.1%

Exp RT: 12.45 minutes
Analyte Name:
583.4145 / 12.45

Collision Energy = 35 ± 15 eV

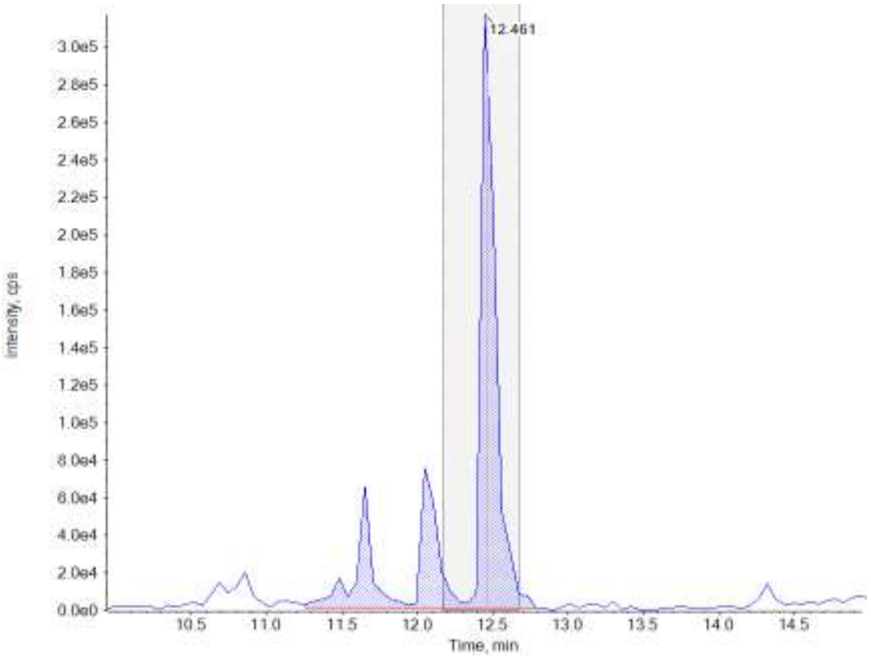

Acquired / Library MSMS

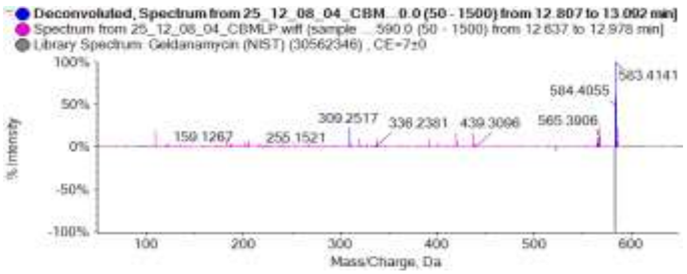

Acquired / Theoretical MS

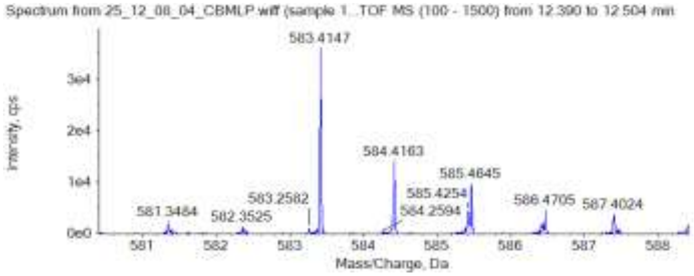

540.4124 / 12.50

(Mass/FragMass/RT/Isotope/Library/Formula/Ion Ratio)

Retention Time: 12.53 minutes
Precursor m/z : 540.4124
Fit (%) 72.1% **RFit (%)** 99.8%

Exp RT: 12.50 minutes
Analyte Name:
540.4124 / 12.50

Collision Energy = 35 ± 15 eV

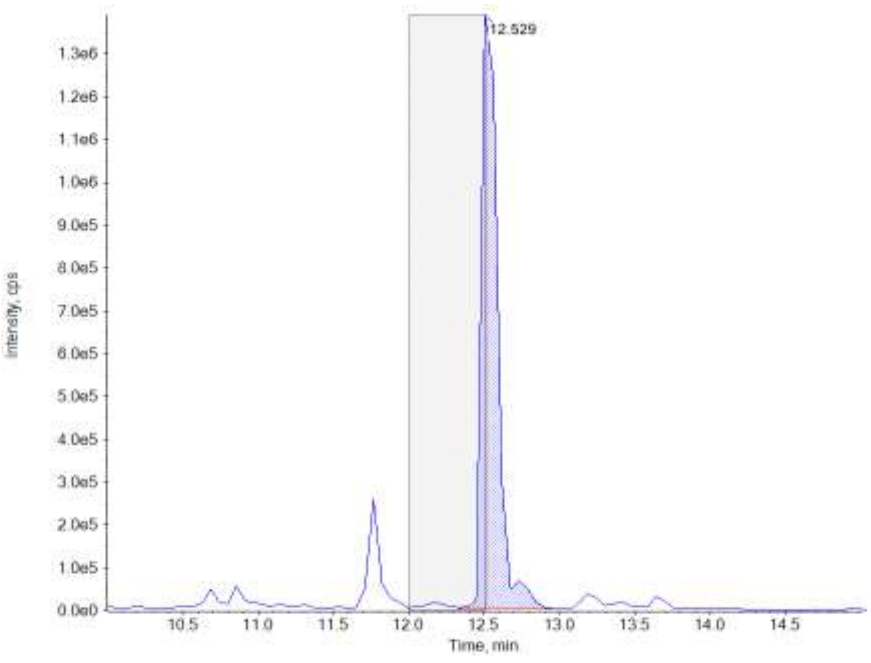

Acquired / Library MSMS

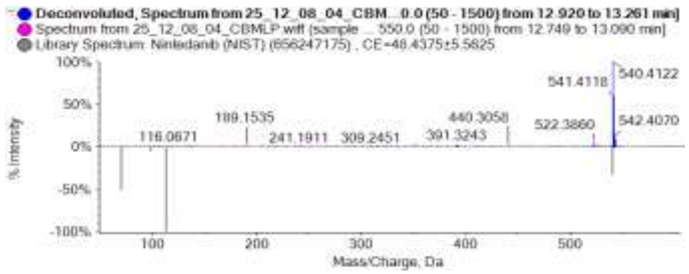

Acquired / Theoretical MS

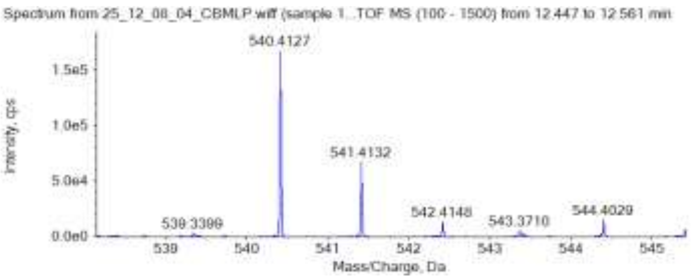

293.2168 / 12.84 (Mass/FragMass/RT/Isotope/Library/Formula/Ion Ratio)

Retention Time: 12.84 minutes
Precursor m/z : 293.2168
Fit (%) 100.0% **RFit (%)** 60.5%

Exp RT: 12.85 minutes
Analyte Name:
293.2168 / 12.84

Collision Energy = 35 ± 15 eV

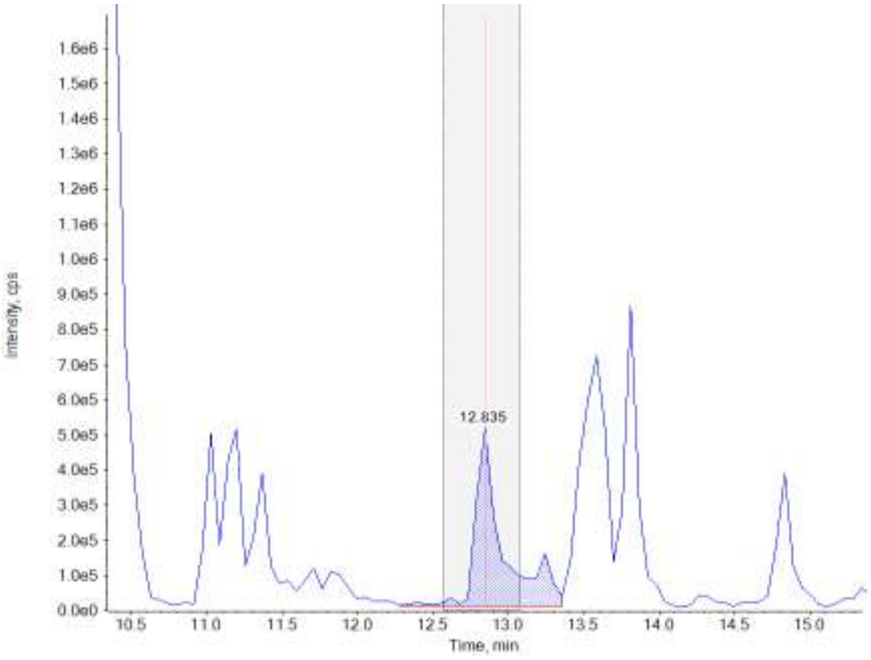

Acquired / Library MSMS

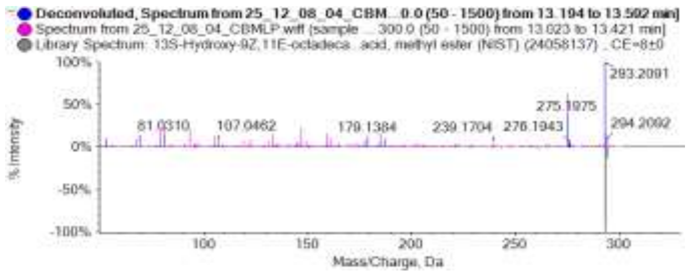

Acquired / Theoretical MS

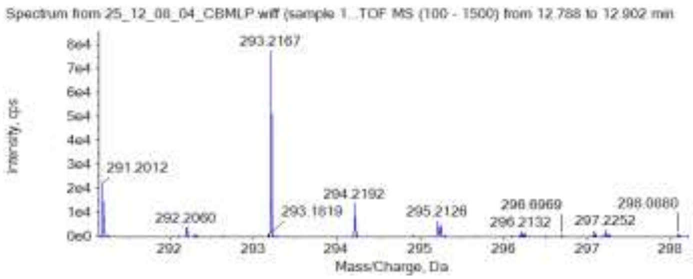

Created with SCIEX OS 3.3.0
Printed:12/9/2025 2:04:37 PM

391.3418 / 12.90 (Mass/FragMass/RT/Isotope/Library/Formula/Ion Ratio)

Retention Time: 12.83 minutes
Precursor m/z : 391.3418
Fit (%) 100.0% **RFit (%)** 46.0%

Exp RT: 12.90 minutes
Analyte Name:
391.3418 / 12.90

Collision Energy = 35 ± 15 eV

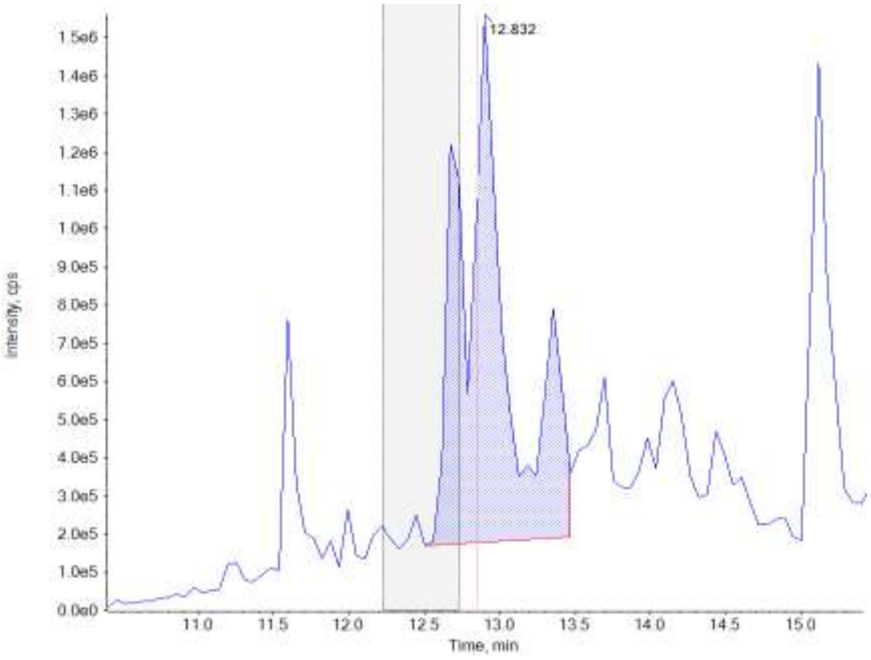

Acquired / Library MSMS

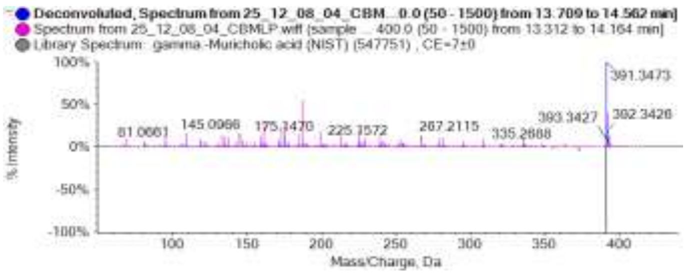

Acquired / Theoretical MS

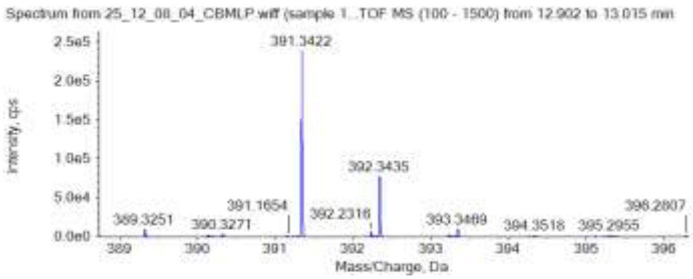

Created with SCIEX OS 3.3.0
Printed:12/9/2025 2:04:37 PM

419.3436 / 12.90 (Mass/FragMass/RT/Isotope/Library/Formula/Ion Ratio)

Retention Time: 12.88 minutes
Precursor m/z : 419.3436
Fit (%) 89.2% **RFit (%)** 83.0%

Exp RT: 12.90 minutes
Analyte Name:
419.3436 / 12.90

Collision Energy = 35 ± 15 eV

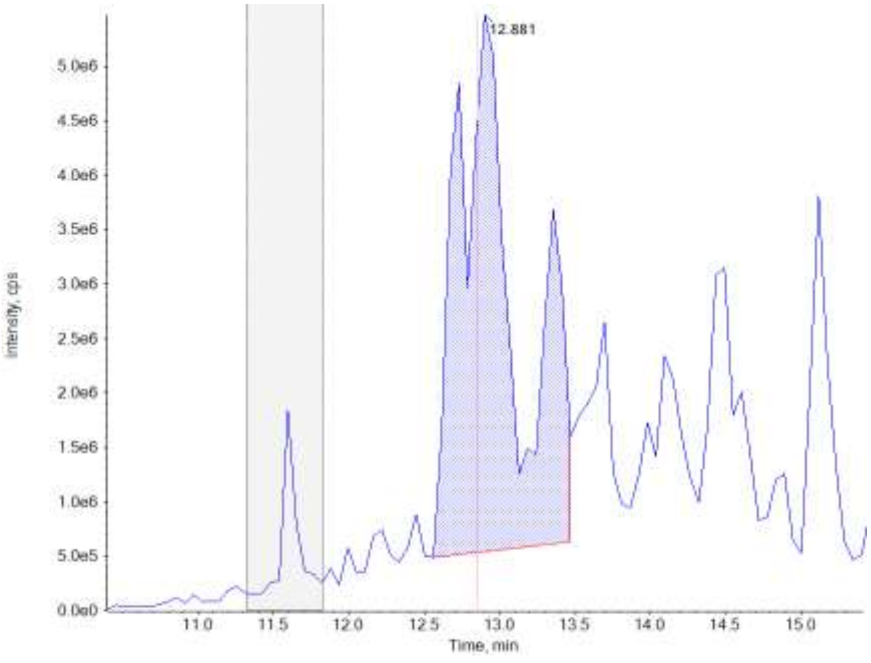

Acquired / Library MSMS

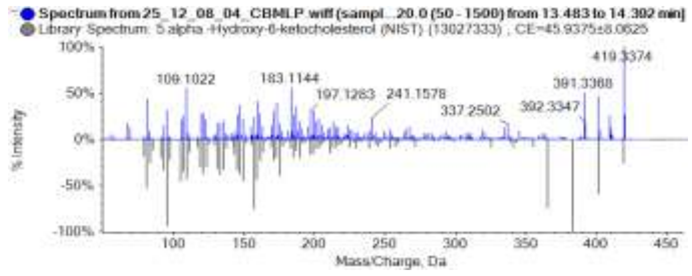

Acquired / Theoretical MS

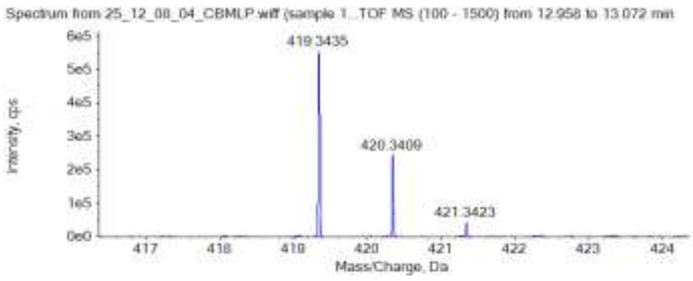

257.1947 / 13.24 (Mass/FragMass/RT/Isotope/Library/Formula/Ion Ratio)

Retention Time: 13.26 minutes **Exp RT:** 13.24 minutes
Precursor m/z : 257.1947 **Analyte Name:**
Fit (%) 94.2% **RFit (%)** 36.7% 257.1947 / 13.24

Collision Energy = 35 ± 15 eV

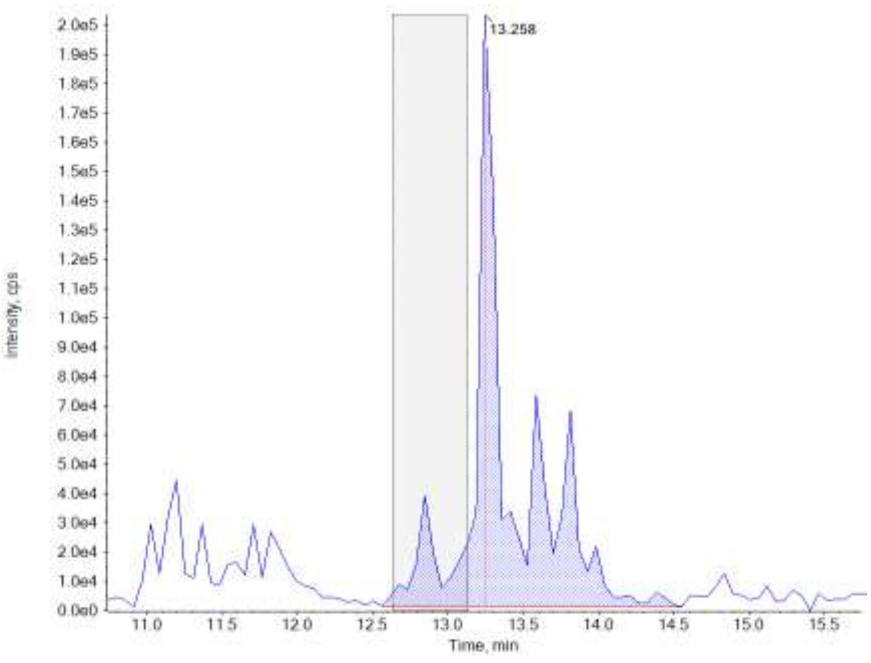

Acquired / Library MSMS

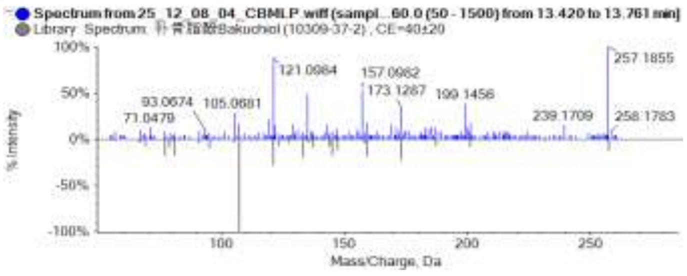

Acquired / Theoretical MS

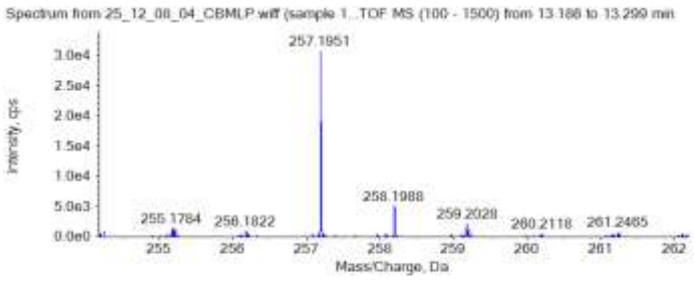

554.4241 / 13.70

(Mass/FragMass/RT/Isotope/Library/Formula/Ion Ratio)

**Retention Time:** 13.69 minutes	**Exp RT:** 13.70 minutes
**Precursor m/z :** 554.4241	**Analyte Name:**
**Fit (%)** 56.5% **RFit (%)** 86.4%	554.4241 / 13.70

Collision Energy = 35 ± 15 eV

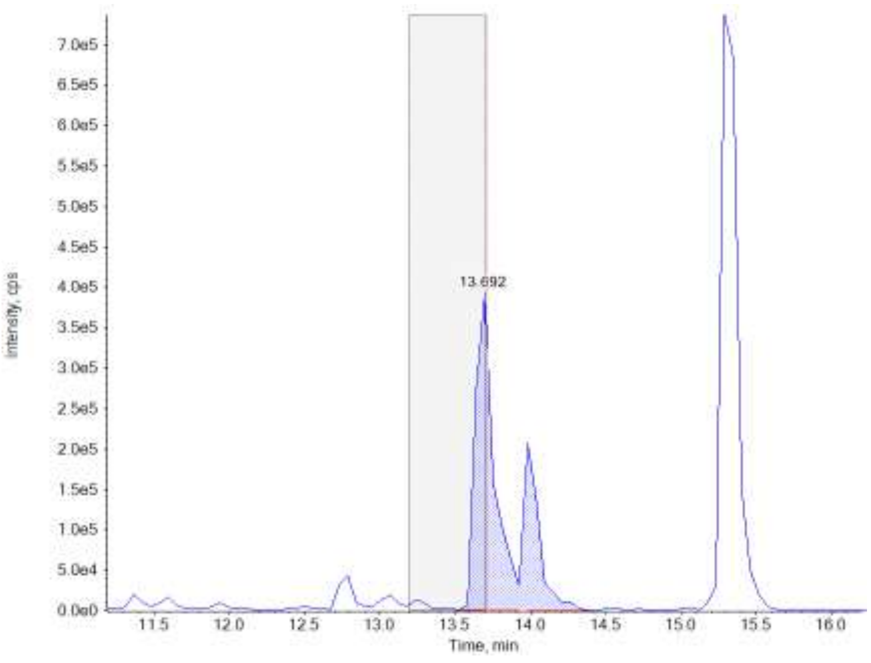

Acquired / Library MSMS

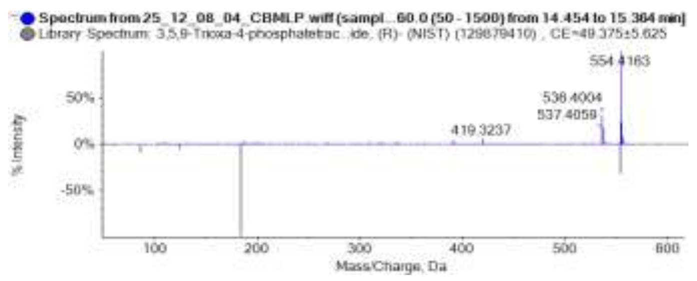

Acquired / Theoretical MS

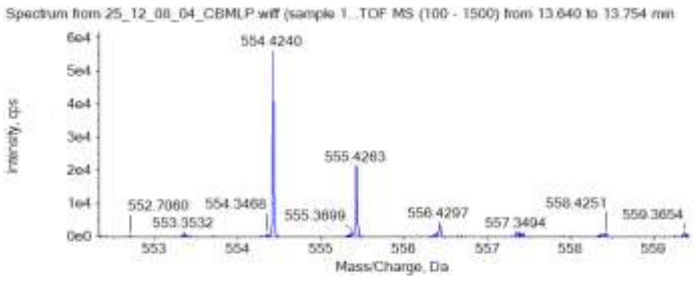

275.2068 / 13.81 (Mass/FragMass/RT/Isotope/Library/Formula/Ion Ratio)

Retention Time: 13.79 minutes
Precursor m/z : 275.2068
Fit (%) 84.0% **RFit (%)** 97.2%

Exp RT: 13.81 minutes
Analyte Name:
275.2068 / 13.81

Collision Energy = 35 ± 15 eV

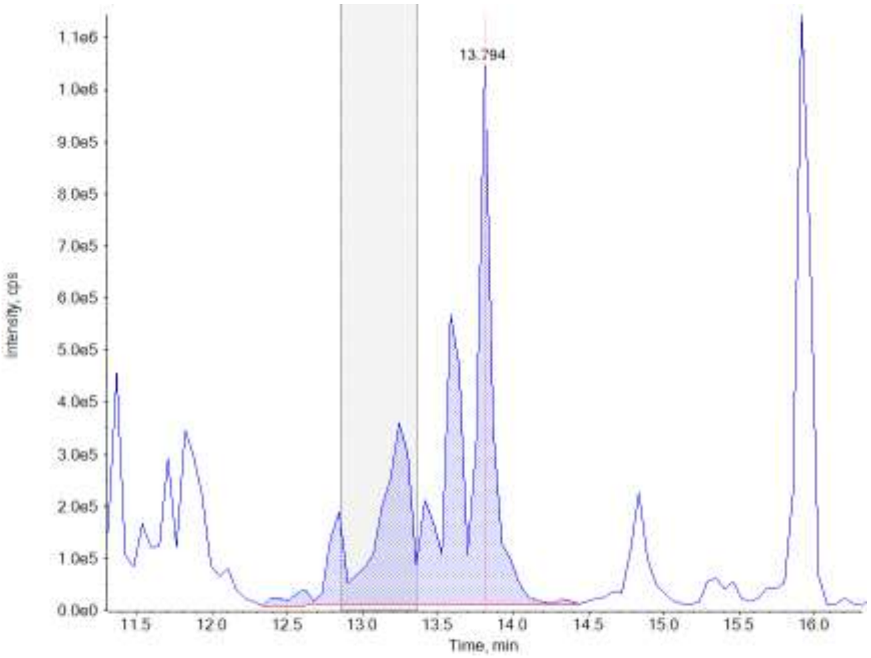

Acquired / Library MSMS

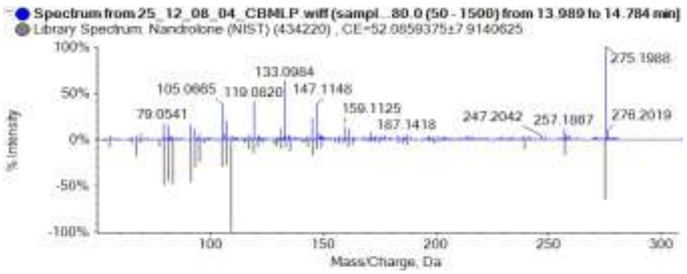

Acquired / Theoretical MS

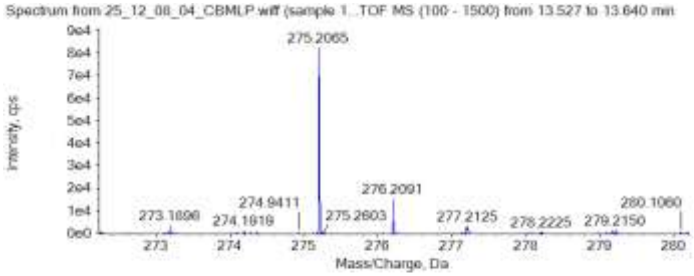

304.2905 / 13.98 (Mass/FragMass/RT/Isotope/Library/Formula/Ion Ratio)

Retention Time: 13.91 minutes
Precursor m/z : 304.2905
Fit (%) 100.0% **RFit (%)** 100.0%

Exp RT: 13.98 minutes
Analyte Name:
304.2905 / 13.98

Collision Energy = 35 ± 15 eV

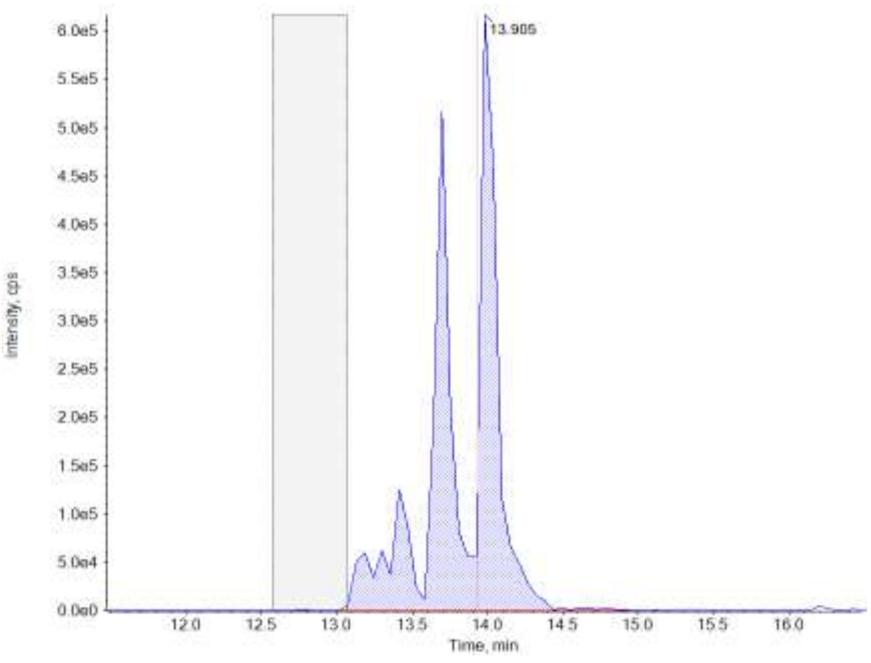

Acquired / Library MSMS

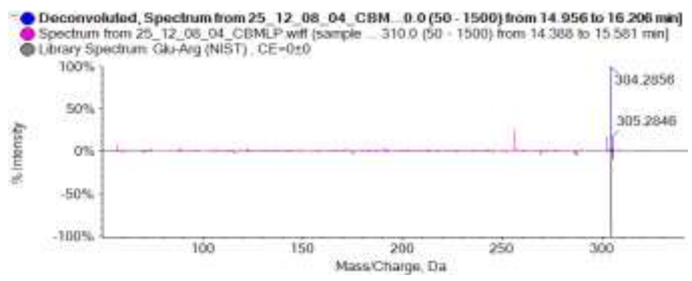

Acquired / Theoretical MS

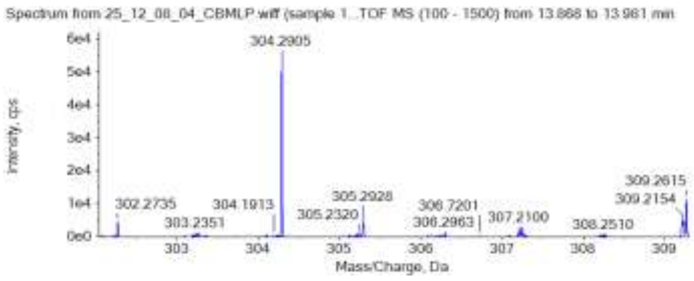

668.4394 / 14.15 (Mass/FragMass/RT/Isotope/Library/Formula/Ion Ratio)

**Retention Time:** 14.17 minutes<br>**Precursor m/z :** 668.4394<br>**Fit (%)** 71.5% **RFit (%)** 94.0%	**Exp RT:** 14.15 minutes<br>**Analyte Name:**<br>668.4394 / 14.15

Collision Energy = 35 ± 15 eV

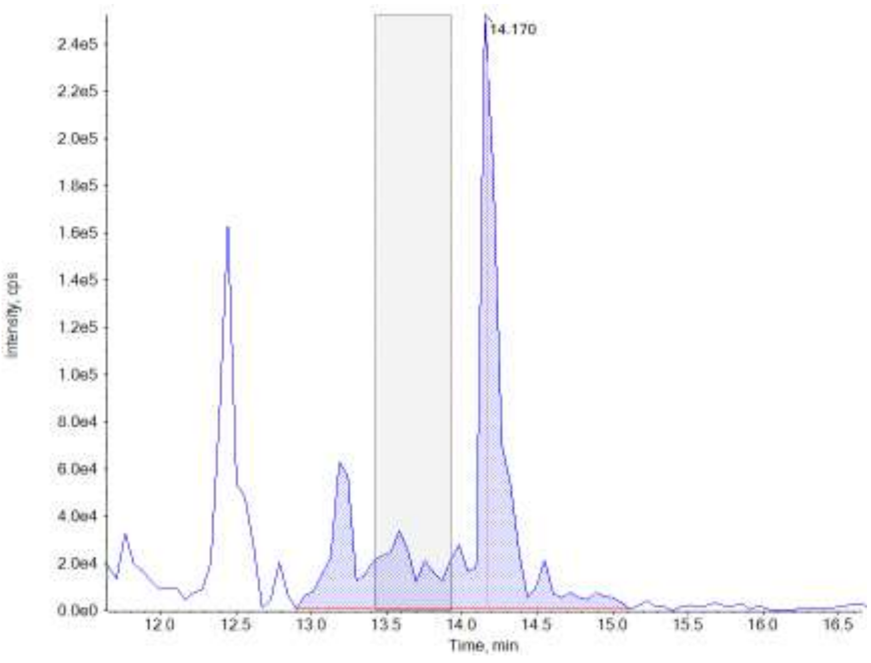

Acquired / Library MSMS

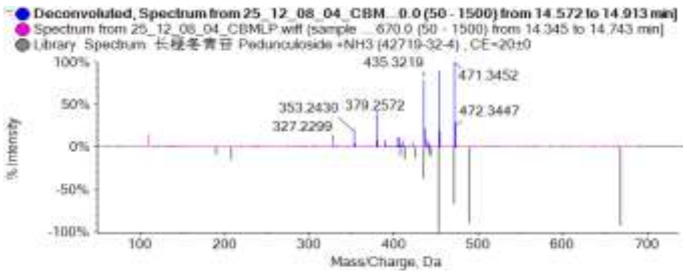

Acquired / Theoretical MS

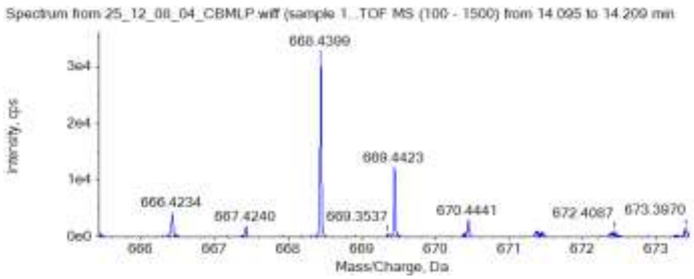

453.3409 / 14.21 (Mass/FragMass/RT/Isotope/Library/Formula/Ion Ratio)

Retention Time: 13.97 minutes
Precursor m/z : 453.3409
Fit (%) 92.8% **RFit (%)** 96.5%

Exp RT: 14.21 minutes
Analyte Name:
453.3409 / 14.21

Collision Energy = 35 ± 15 eV

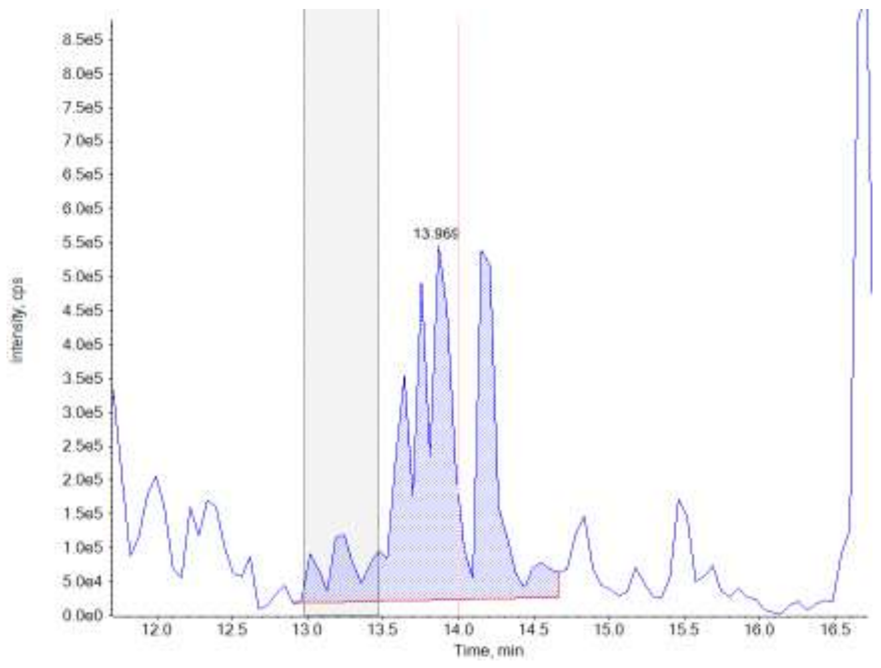

Acquired / Library MSMS

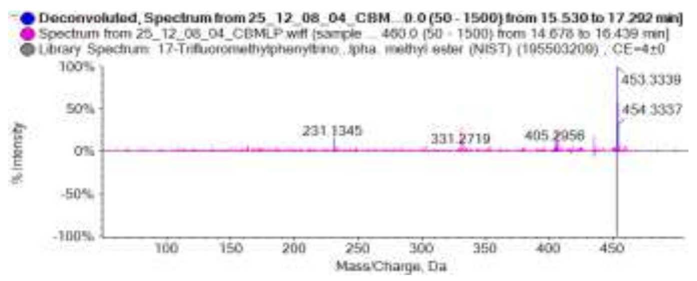

Acquired / Theoretical MS

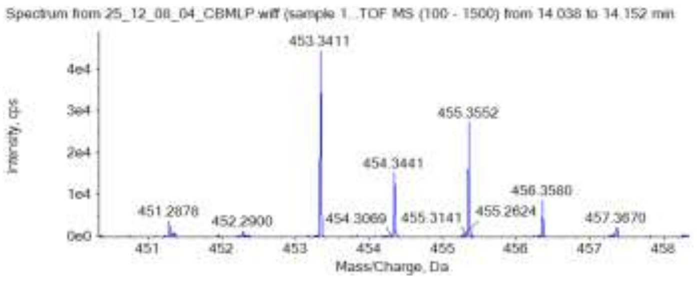

523.3455 / 14.61

(Mass/FragMass/RT/Isotope/Library/Formula/Ion Ratio)

Retention Time: 14.63 minutes
Precursor m/z : 523.3455
Fit (%) 72.3% **RFit (%)** 72.1%

Exp RT: 14.61 minutes
Analyte Name:
523.3455 / 14.61

Collision Energy = 35 ± 15 eV

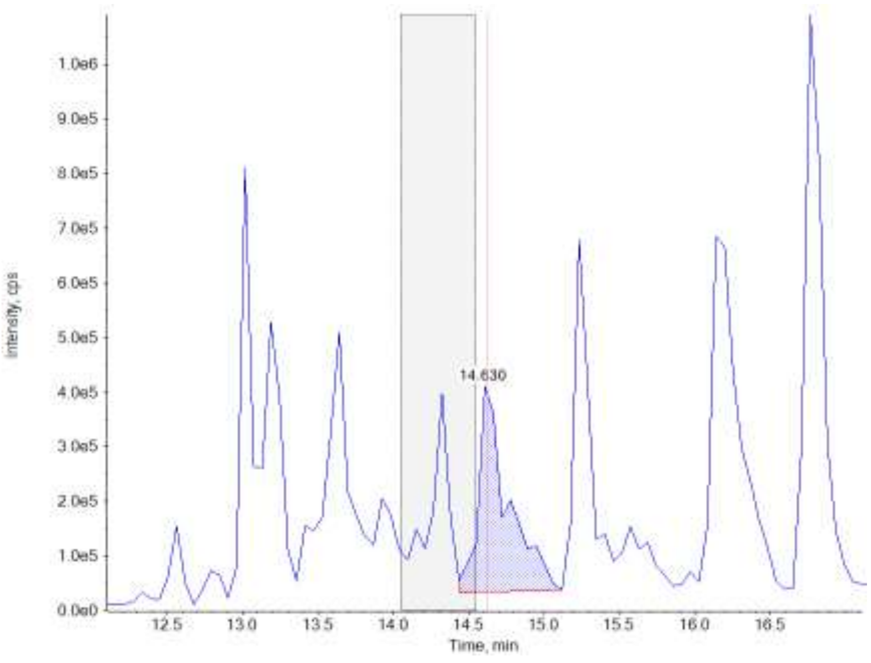

Acquired / Library MSMS

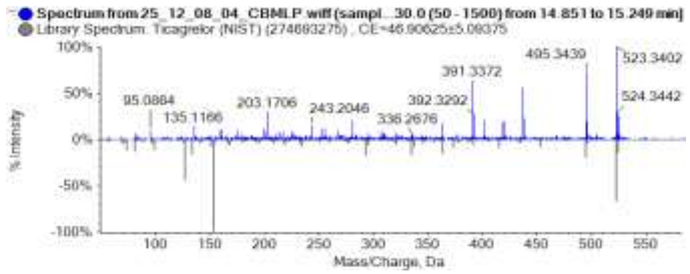

Acquired / Theoretical MS

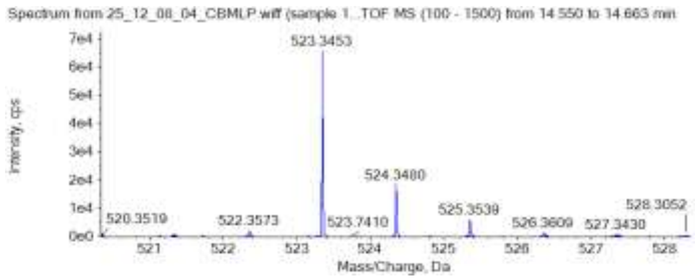

496.3448 / 14.78 (Mass/FragMass/RT/Isotope/Library/Formula/Ion Ratio)

Retention Time: 14.76 minutes
Precursor m/z : 496.3448
Fit (%) 97.2% **RFit (%)** 100.0%

Exp RT: 14.78 minutes
Analyte Name:
496.3448 / 14.78

Collision Energy = 35 ± 15 eV

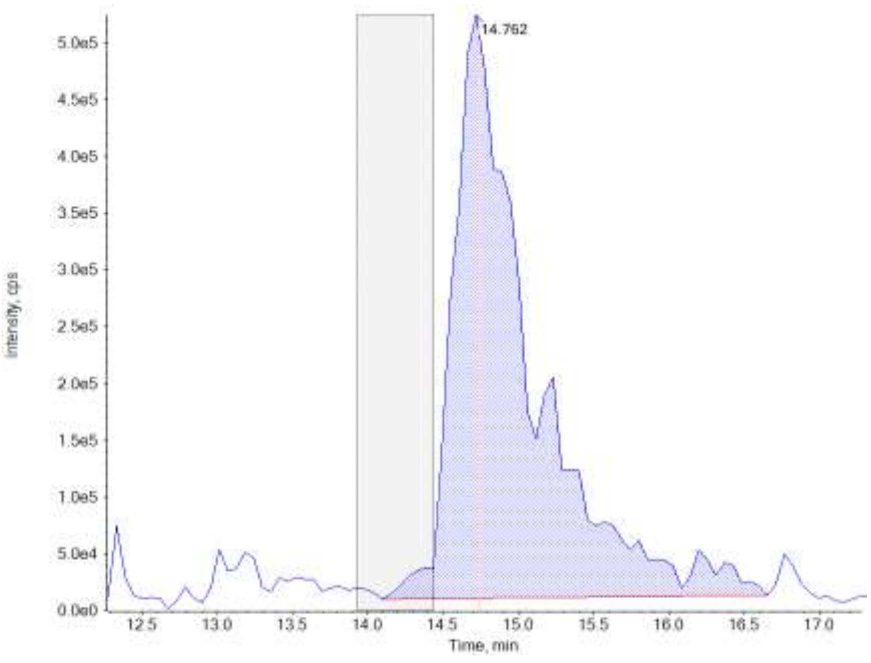

Acquired / Library MSMS

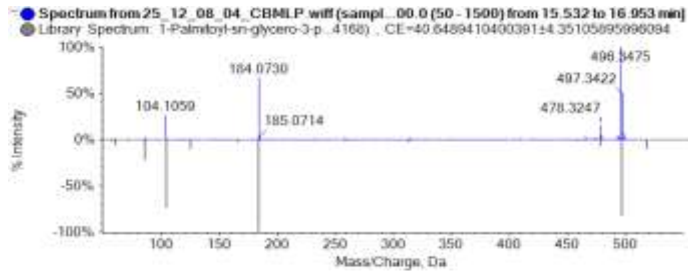

Acquired / Theoretical MS

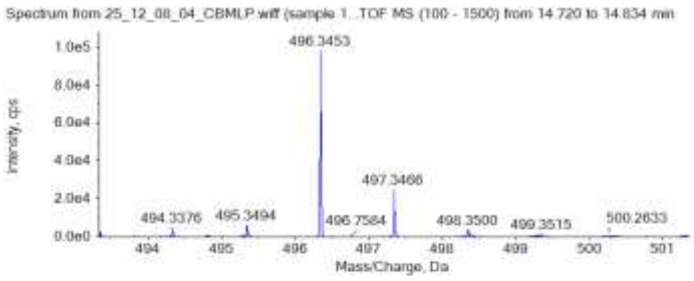

Created with SCIEX OS 3.3.0
Printed:12/9/2025 2:04:37 PM

275.2061 / 14.83 (Mass/FragMass/RT/Isotope/Library/Formula/Ion Ratio)

Retention Time: 14.83 minutes
Precursor m/z : 275.2061
Fit (%) 97.9% **RFit (%)** 100.0%

Exp RT: 14.83 minutes
Analyte Name:
275.2061 / 14.83

Collision Energy = 35 ± 15 eV

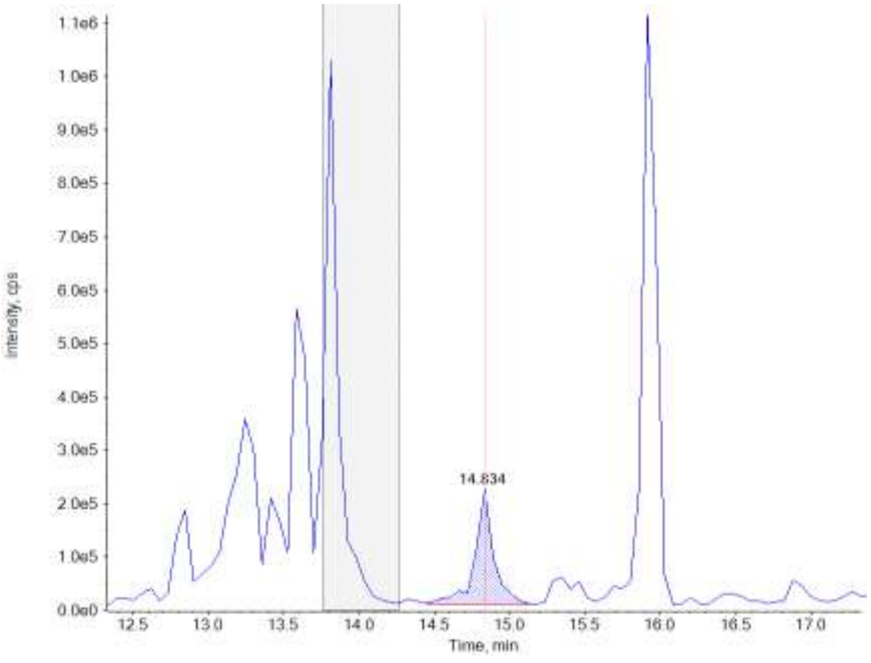

Acquired / Library MSMS

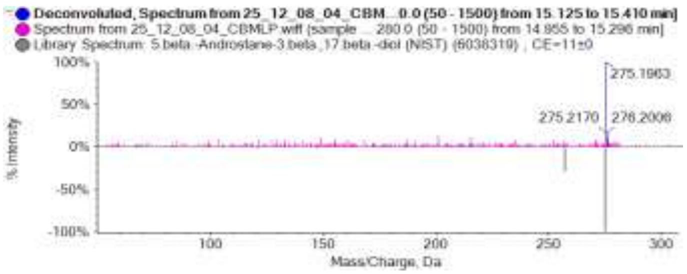

Acquired / Theoretical MS

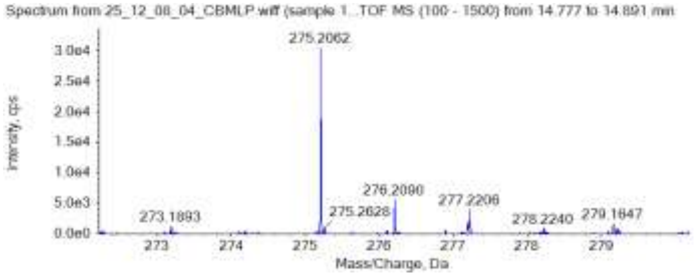

293.2166 / 14.83 (Mass/FragMass/RT/Isotope/Library/Formula/Ion Ratio)

**Retention Time:** 14.83 minutes	**Exp RT:** 14.83 minutes
**Precursor m/z :** 293.2166	**Analyte Name:**
**Fit (%)** 80.9% **RFit (%)** 95.3%	293.2166 / 14.83

Collision Energy = 35 ± 15 eV

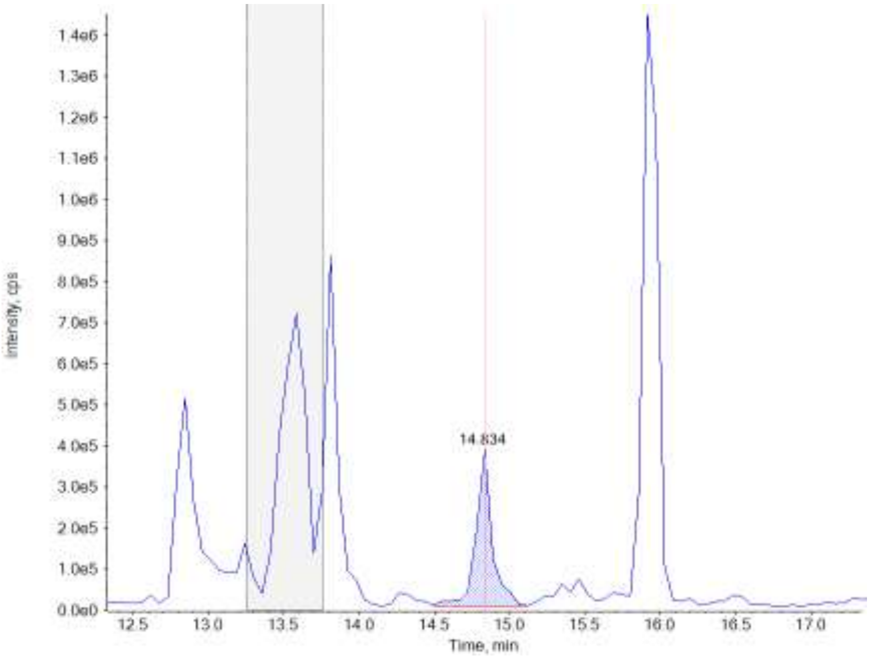

Acquired / Library MSMS

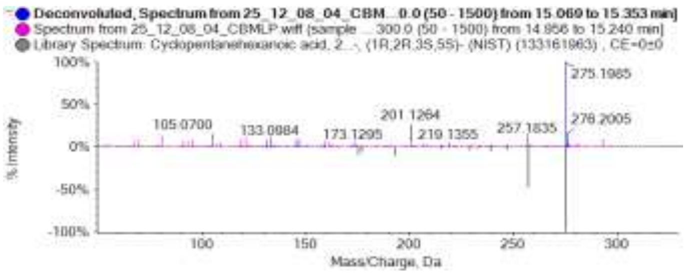

Acquired / Theoretical MS

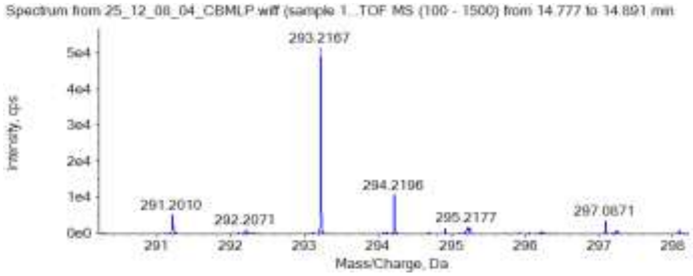

309.2624 / 15.12 (Mass/FragMass/RT/Isotope/Library/Formula/Ion Ratio)

Retention Time: 15.12 minutes
Precursor m/z : 309.2624
Fit (%) 100.0% **RFit (%)** 52.5%

Exp RT: 15.12 minutes
Analyte Name:
309.2624 / 15.12

Collision Energy = 35 ± 15 eV

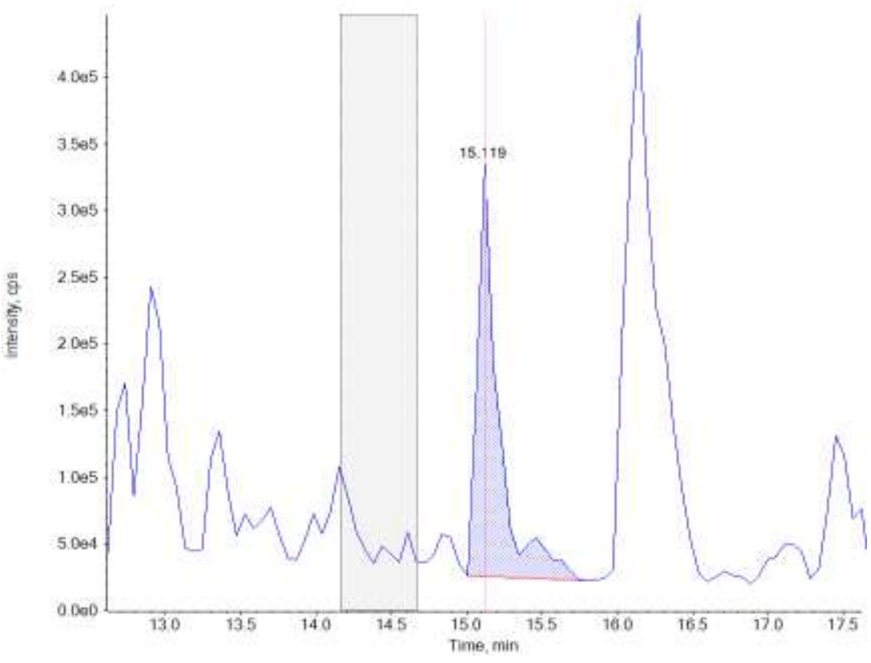

Acquired / Library MSMS

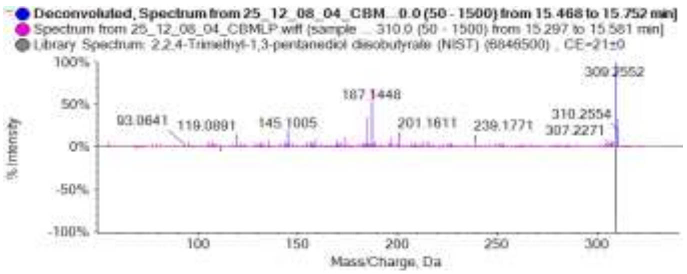

Acquired / Theoretical MS

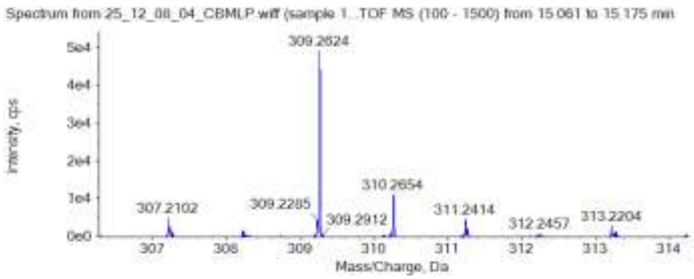

391.3417 / 15.12 (Mass/FragMass/RT/Isotope/Library/Formula/Ion Ratio)

**Retention Time:** 15.12 minutes	**Exp RT:** 15.12 minutes
**Precursor m/z :** 391.3417	**Analyte Name:**
**Fit (%)** 100.0% **RFit (%)** 63.1%	391.3417 / 15.12

Collision Energy = 35 ± 15 eV

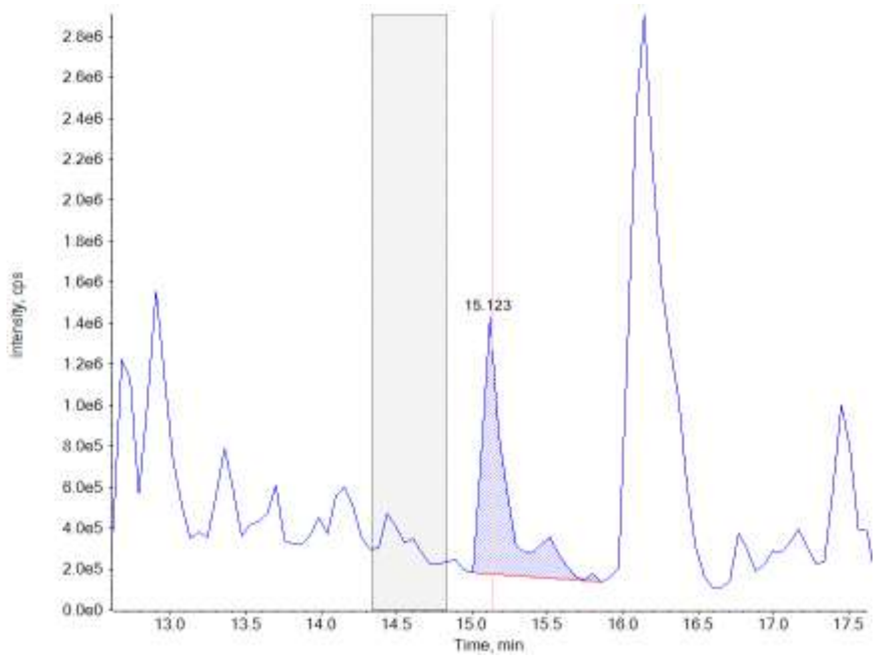

Acquired / Library MSMS

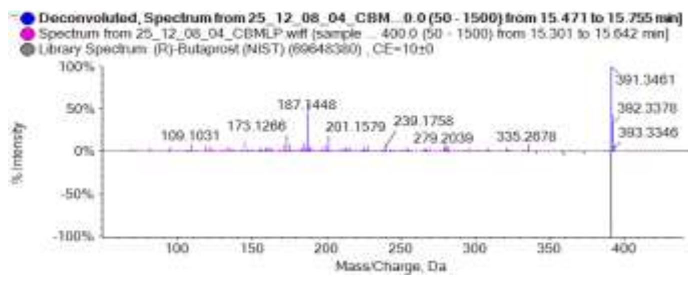

Acquired / Theoretical MS

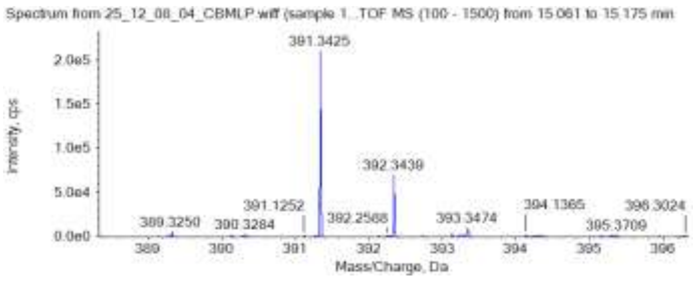

419.3406 / 15.12 (Mass/FragMass/RT/Isotope/Library/Formula/Ion Ratio)

Retention Time: 15.13 minutes
Precursor m/z : 419.3406
Fit (%) 82.2% **RFit (%)** 79.3%

Exp RT: 15.12 minutes
Analyte Name:
419.3406 / 15.12

Collision Energy = 35 ± 15 eV

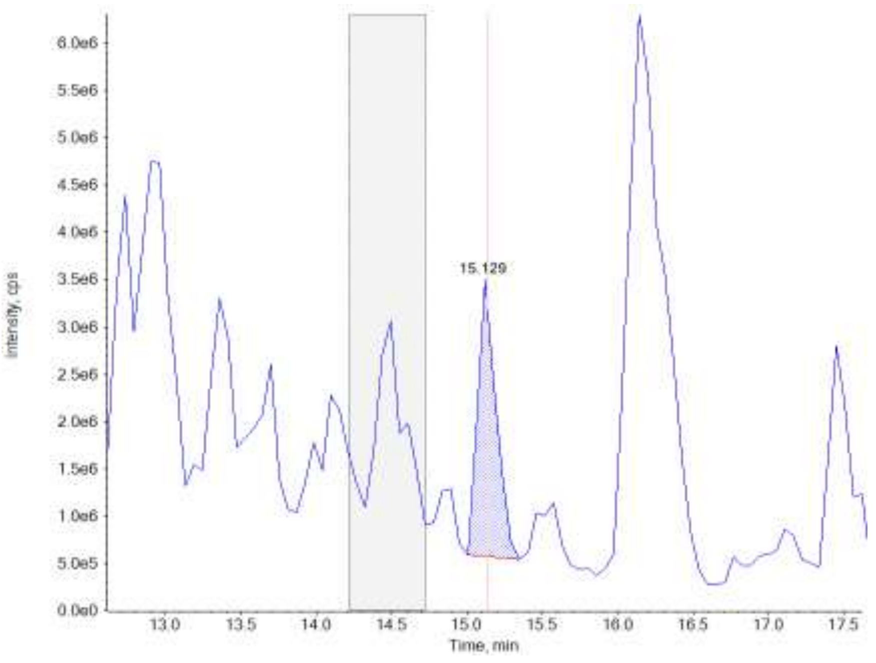

Acquired / Library MSMS

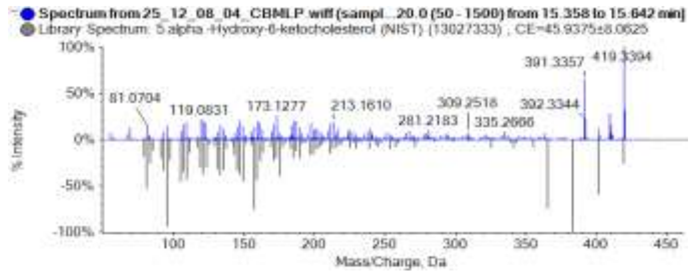

Acquired / Theoretical MS

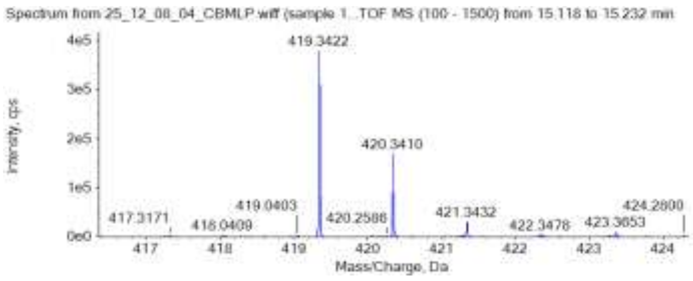

455.3583 / 15.12 (Mass/FragMass/RT/Isotope/Library/Formula/Ion Ratio)

Retention Time: 15.12 minutes
Precursor m/z : 455.3583
Fit (%) 90.6% **RFit (%)** 80.1%

Exp RT: 15.12 minutes
Analyte Name:
455.3583 / 15.12

Collision Energy = 35 ± 15 eV

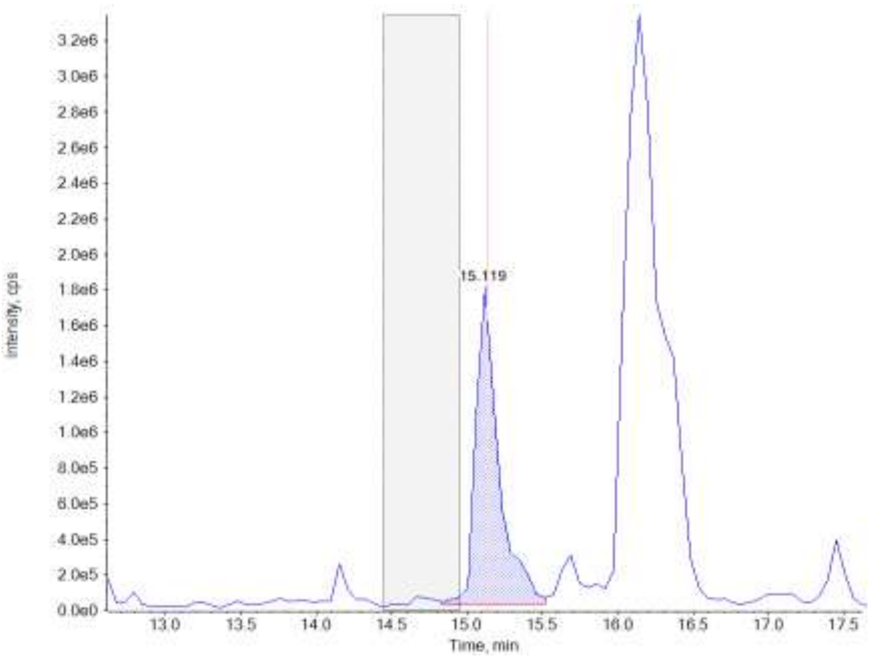

Acquired / Library MSMS

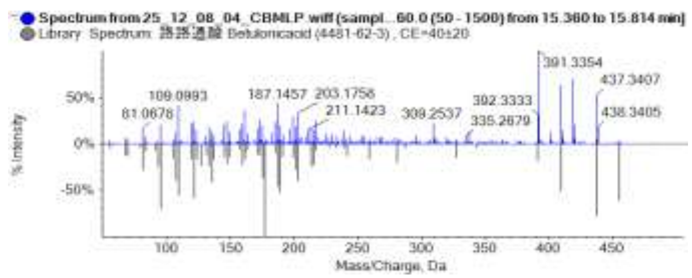

Acquired / Theoretical MS

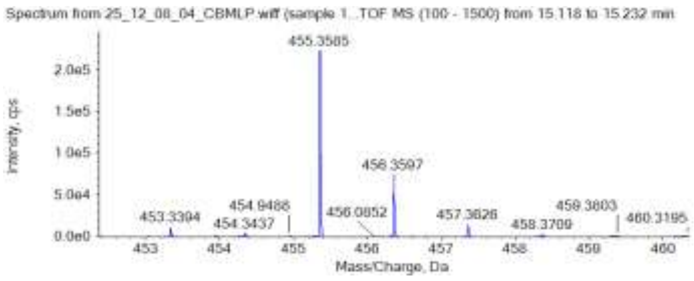

279.2279 / 15.29 (Mass/FragMass/RT/Isotope/Library/Formula/Ion Ratio)

Retention Time: 15.32 minutes
Precursor m/z : 279.2279
Fit (%) 97.1% **RFit (%)** 86.8%

Exp RT: 15.29 minutes
Analyte Name:
279.2279 / 15.29

Collision Energy = 35 ± 15 eV

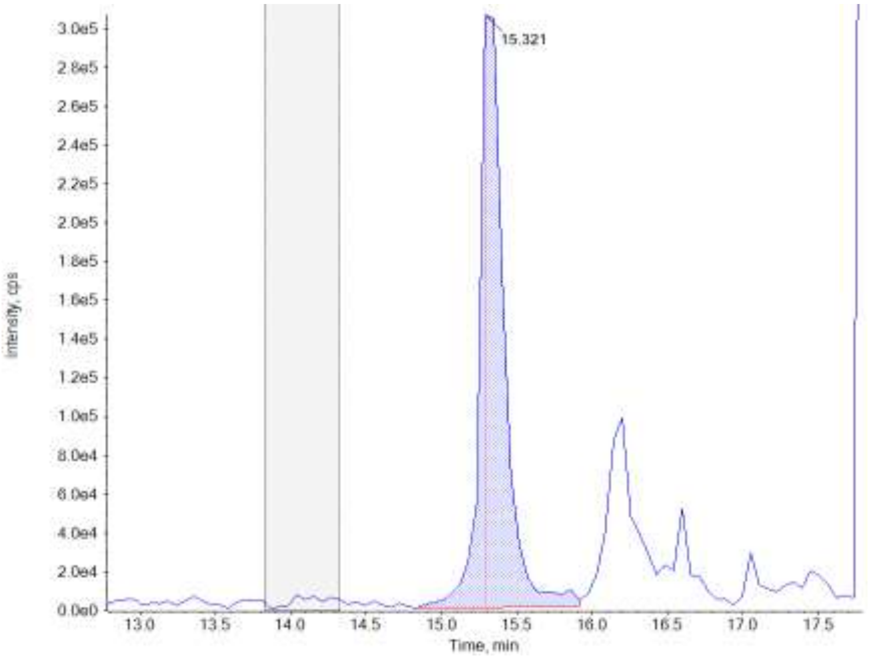

Acquired / Library MSMS

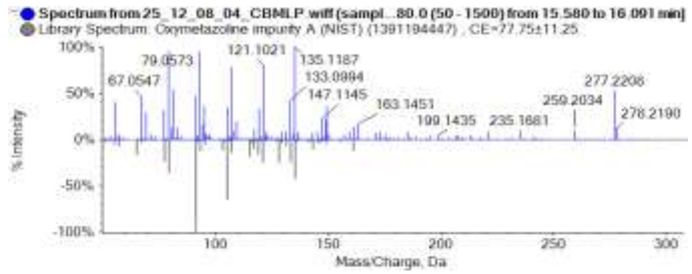

Acquired / Theoretical MS

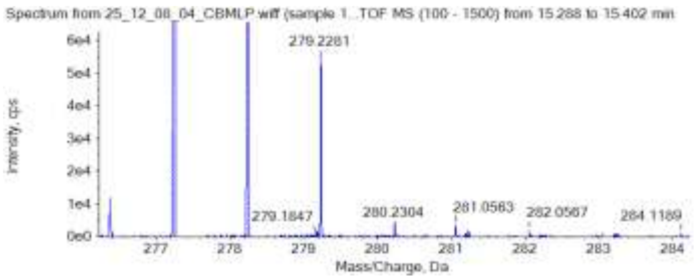

553.4366 / 15.29 (Mass/FragMass/RT/Isotope/Library/Formula/Ion Ratio)

Retention Time: 15.31 minutes
Precursor m/z : 553.4366
Fit (%) 92.5% **RFit (%)** 54.0%

Exp RT: 15.29 minutes
Analyte Name:
553.4366 / 15.29

Collision Energy = 35 ± 15 eV

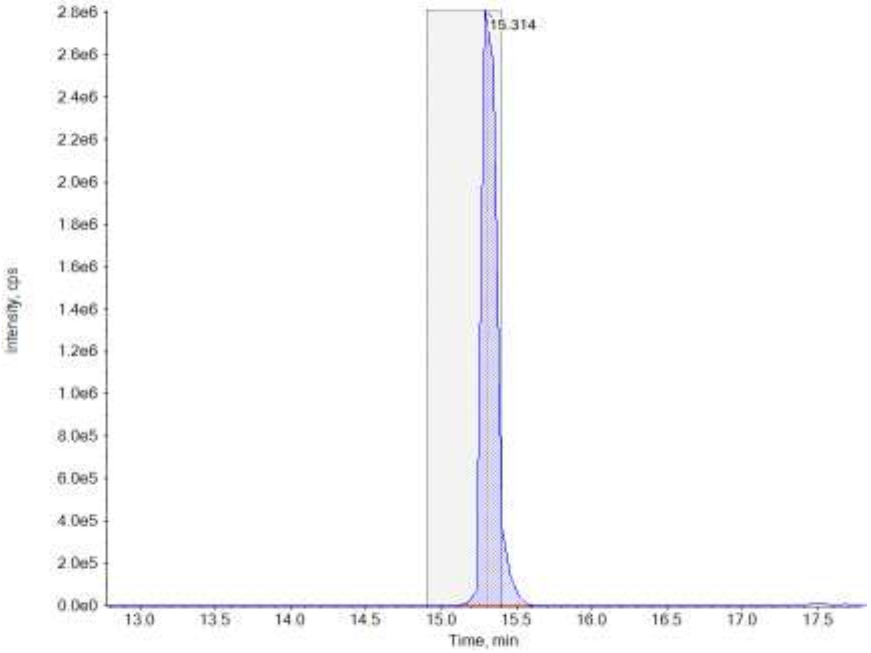

Acquired / Library MSMS

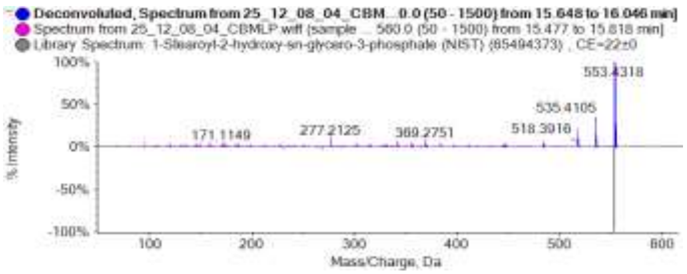

Acquired / Theoretical MS

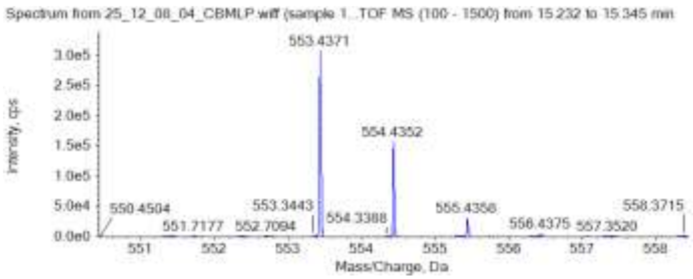

277.2312 / 15.35 (Mass/FragMass/RT/Isotope/Library/Formula/Ion Ratio)

Retention Time: 15.36 minutes
Precursor m/z : 277.2312
Fit (%) 99.2% **RFit (%)** 98.5%

Exp RT: 15.35 minutes
Analyte Name:
277.2312 / 15.35

Collision Energy = 35 ± 15 eV

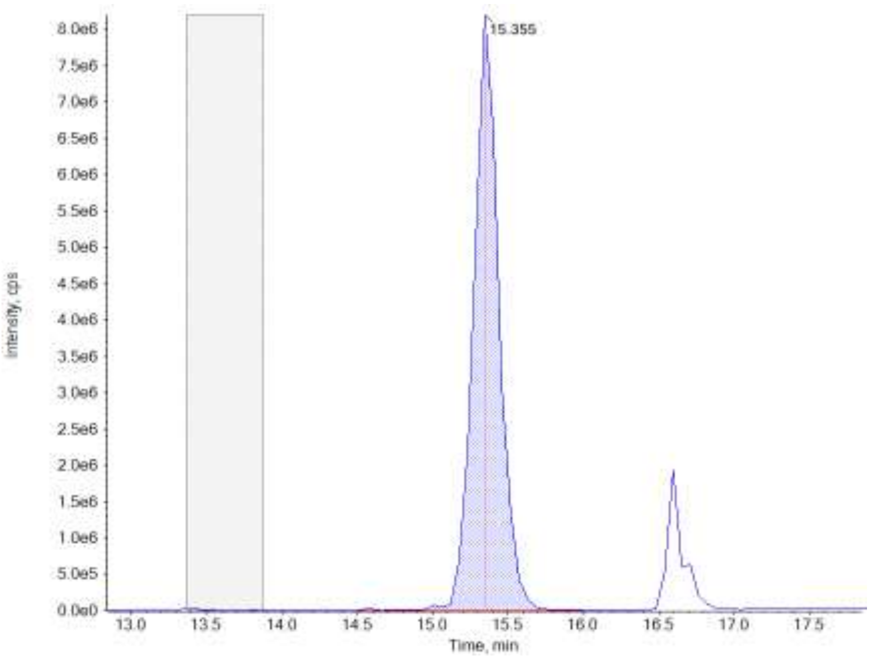

Acquired / Library MSMS

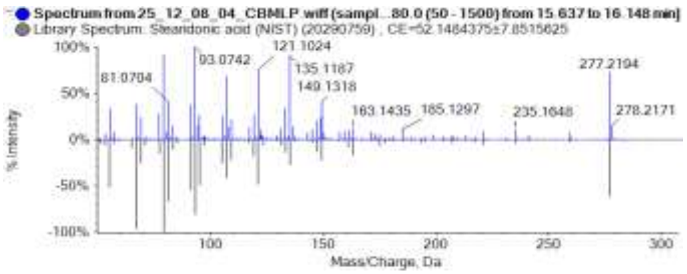

Acquired / Theoretical MS

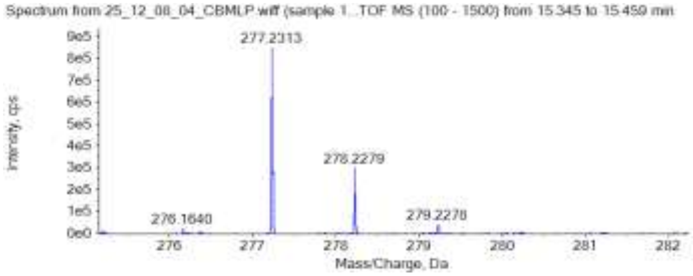

277.2207 / 15.57 (Mass/FragMass/RT/Isotope/Library/Formula/Ion Ratio)

**Retention Time:** 15.38 minutes	**Exp RT:** 15.57 minutes
**Precursor m/z :** 277.2207	**Analyte Name:**
**Fit (%)** 97.1% **RFit (%)** 99.4%	277.2207 / 15.57

Collision Energy = 35 ± 15 eV

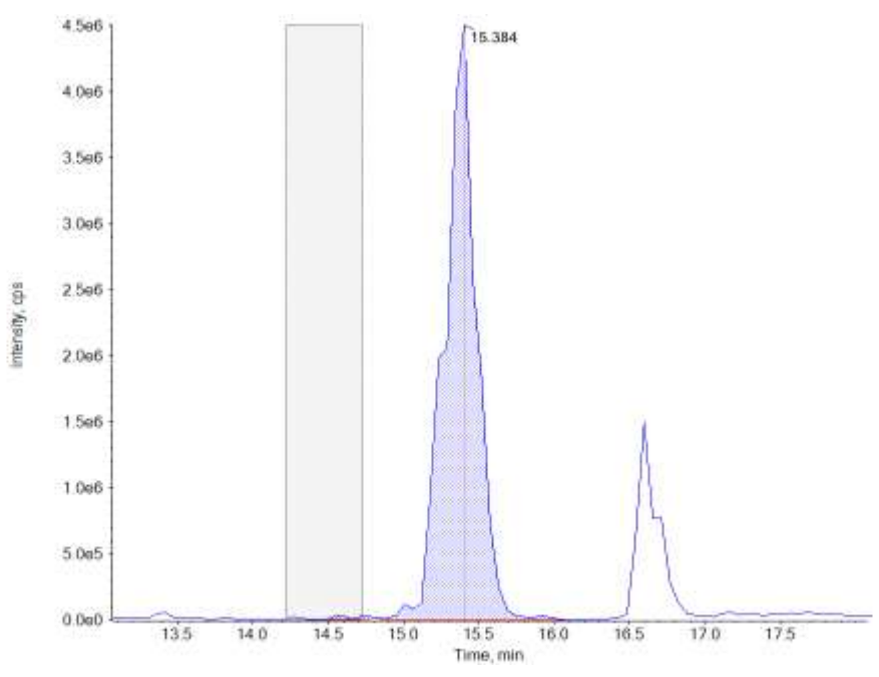

Acquired / Library MSMS

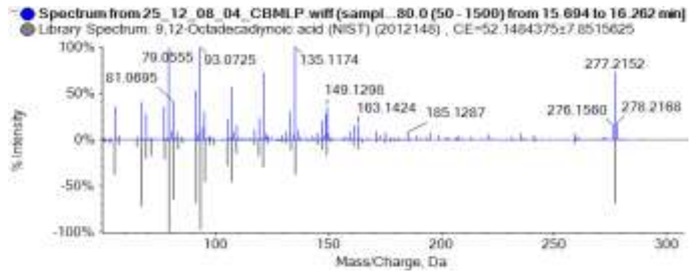

Acquired / Theoretical MS

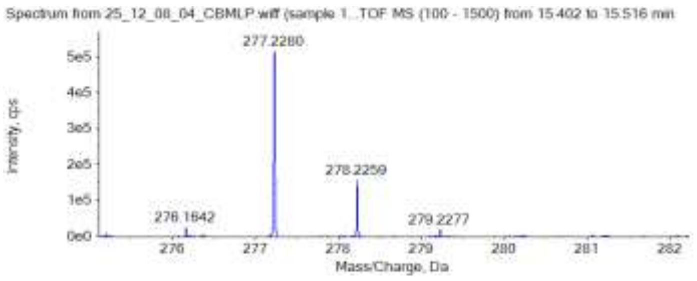

Created with SCIEX OS 3.3.0
Printed:12/9/2025 2:04:37 PM

181.1274 / 15.69 (Mass/FragMass/RT/Isotope/Library/Formula/Ion Ratio)

Retention Time: 15.66 minutes
Precursor m/z : 181.1274
Fit (%) 87.7% **RFit (%)** 68.4%

Exp RT: 15.69 minutes
Analyte Name:
181.1274 / 15.69

Collision Energy = 35 ± 15 eV

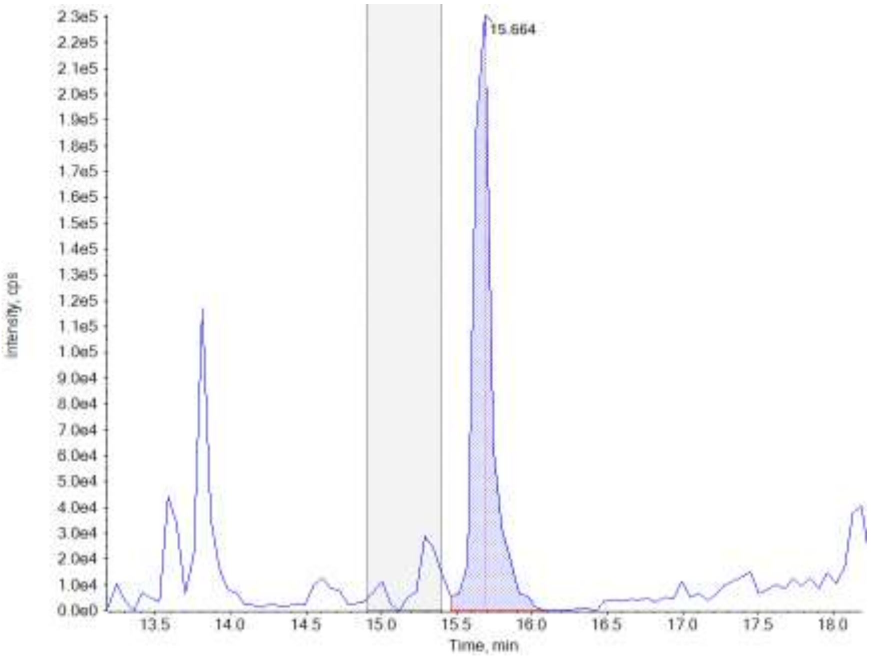

Acquired / Library MSMS

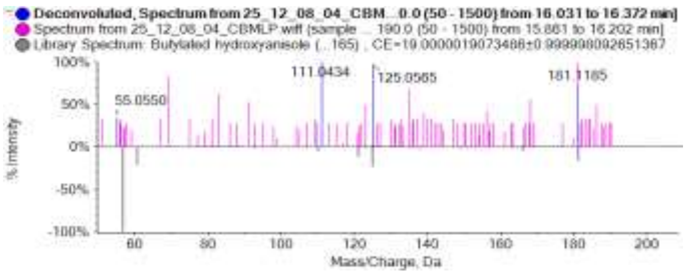

Acquired / Theoretical MS

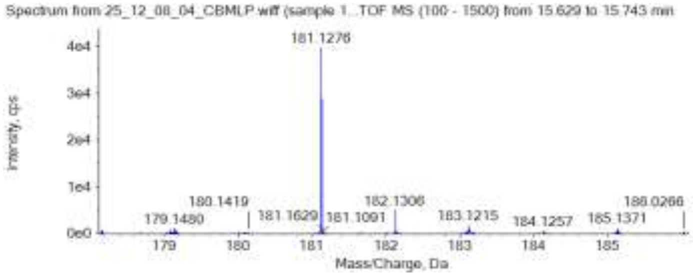

332.3214 / 15.69 (Mass/FragMass/RT/Isotope/Library/Formula/Ion Ratio)

**Retention Time:** 15.77 minutes	**Exp RT:** 15.69 minutes
**Precursor m/z :** 332.3214	**Analyte Name:**
**Fit (%)** 99.5% **RFit (%)** 92.8%	332.3214 / 15.69

Collision Energy = 35 ± 15 eV

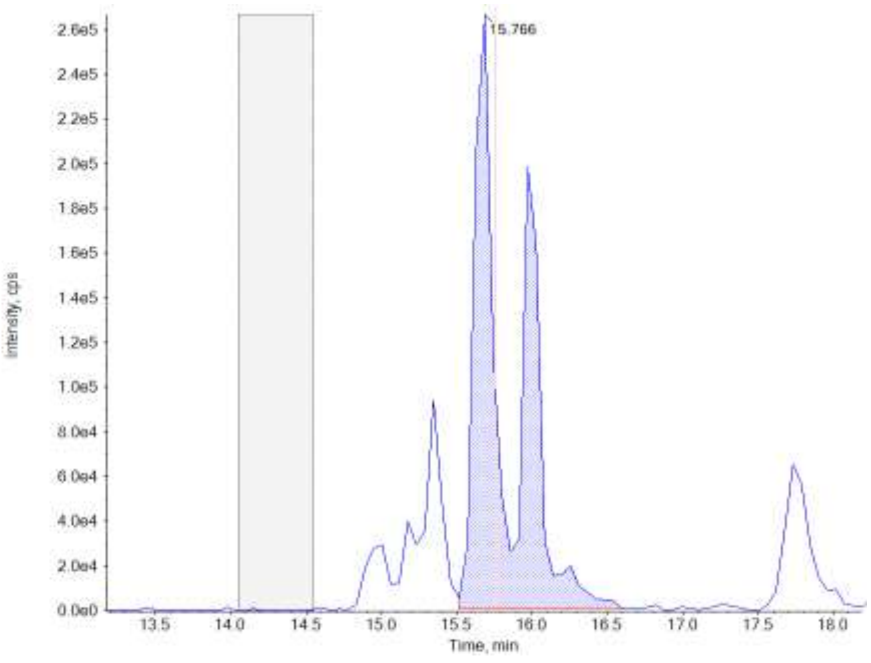

Acquired / Library MSMS

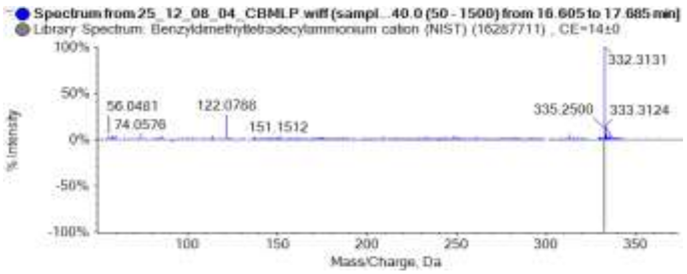

Acquired / Theoretical MS

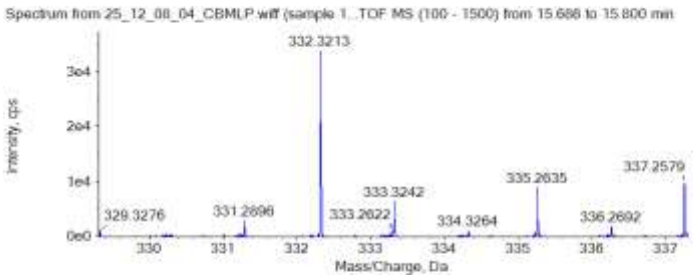

353.2752 / 15.74 (Mass/FragMass/RT/Isotope/Library/Formula/Ion Ratio)

Retention Time: 15.74 minutes
Precursor m/z : 353.2752
Fit (%) 36.2% **RFit (%)** 99.1%

Exp RT: 15.74 minutes
Analyte Name:
353.2752 / 15.74

Collision Energy = 35 ± 15 eV

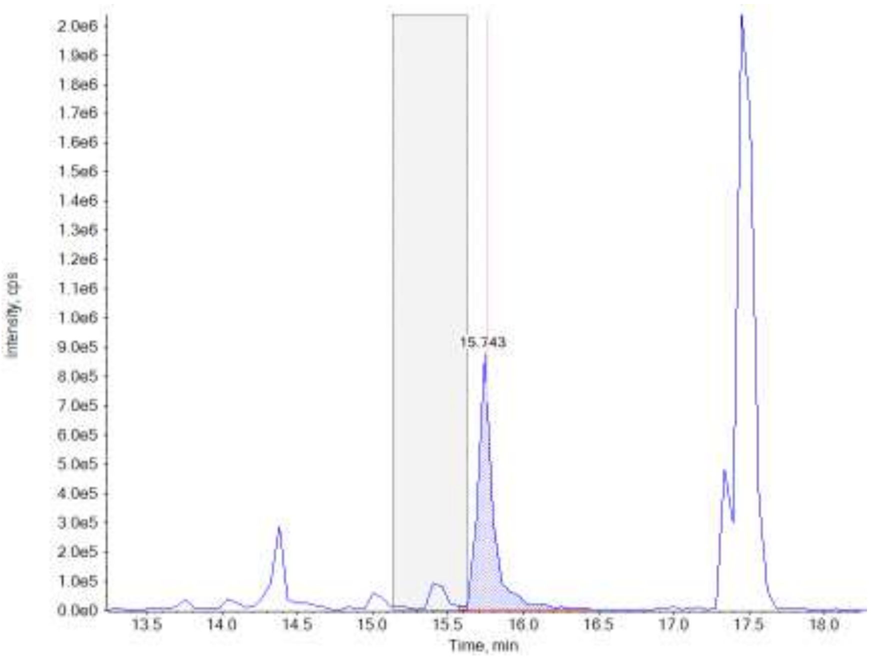

Acquired / Library MSMS

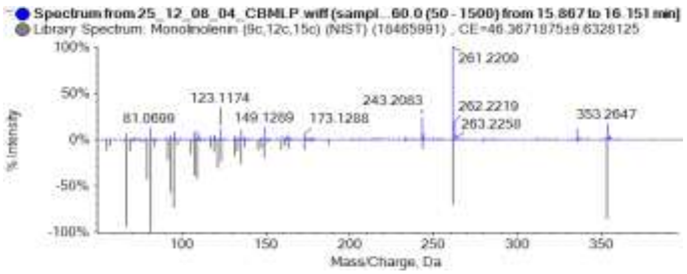

Acquired / Theoretical MS

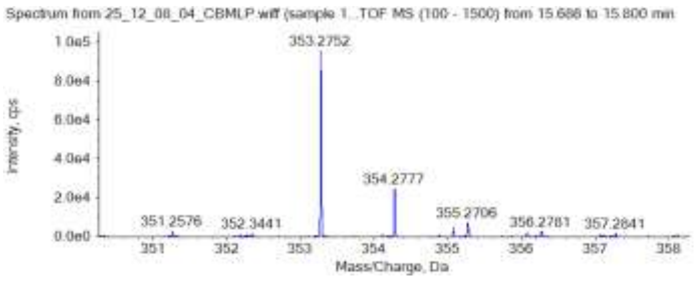

275.2086 / 15.91 (Mass/FragMass/RT/Isotope/Library/Formula/Ion Ratio)

**Retention Time:** 15.92 minutes	**Exp RT:** 15.91 minutes
**Precursor m/z :** 275.2086	**Analyte Name:**
**Fit (%)** 93.3% **RFit (%)** 67.2%	275.2086 / 15.91

Collision Energy = 35 ± 15 eV

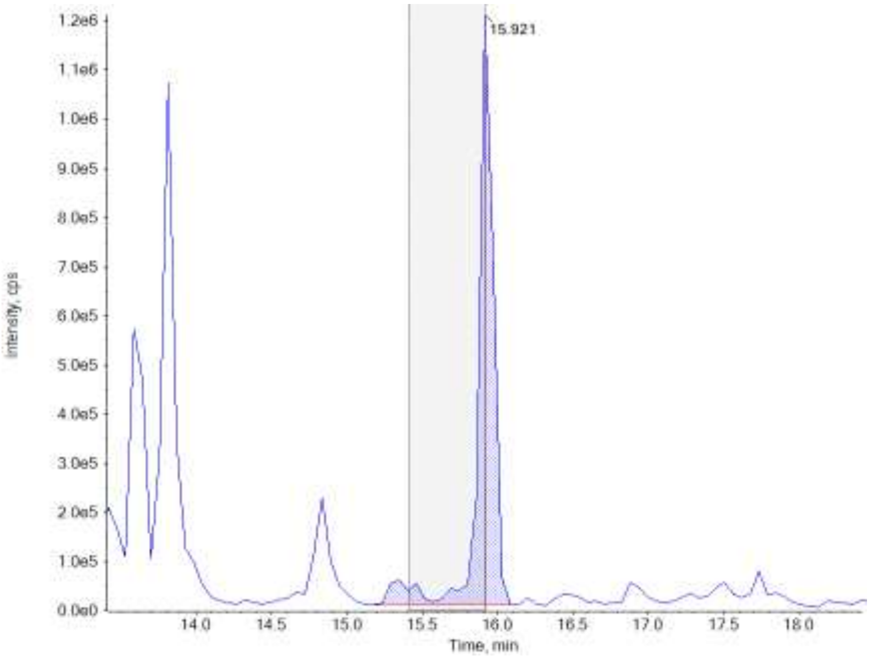

Acquired / Library MSMS

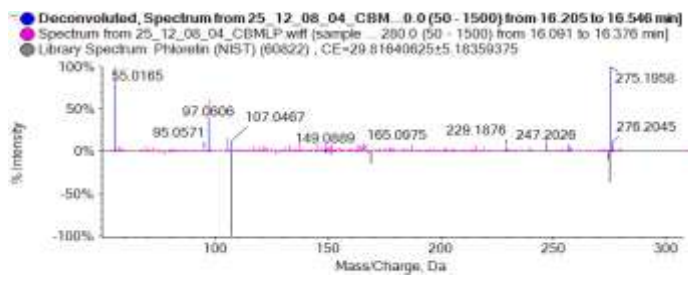

Acquired / Theoretical MS

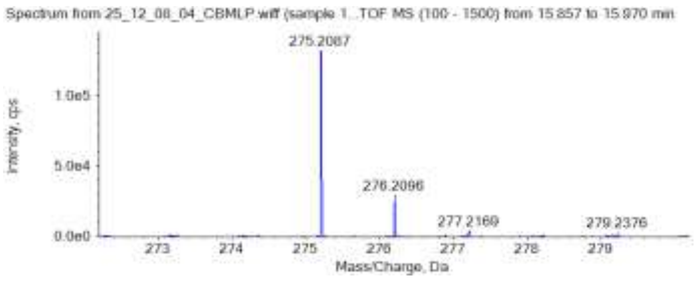

Created with SCIEX OS 3.3.0
Printed:12/9/2025 2:04:37 PM

203.1847 / 16.14 (Mass/FragMass/RT/Isotope/Library/Formula/Ion Ratio)

**Retention Time:** 16.14 minutes	**Exp RT:** 16.14 minutes
**Precursor m/z :** 203.1847	**Analyte Name:**
**Fit (%)** 98.3% **RFit (%)** 40.3%	203.1847 / 16.14

Collision Energy = 35 ± 15 eV

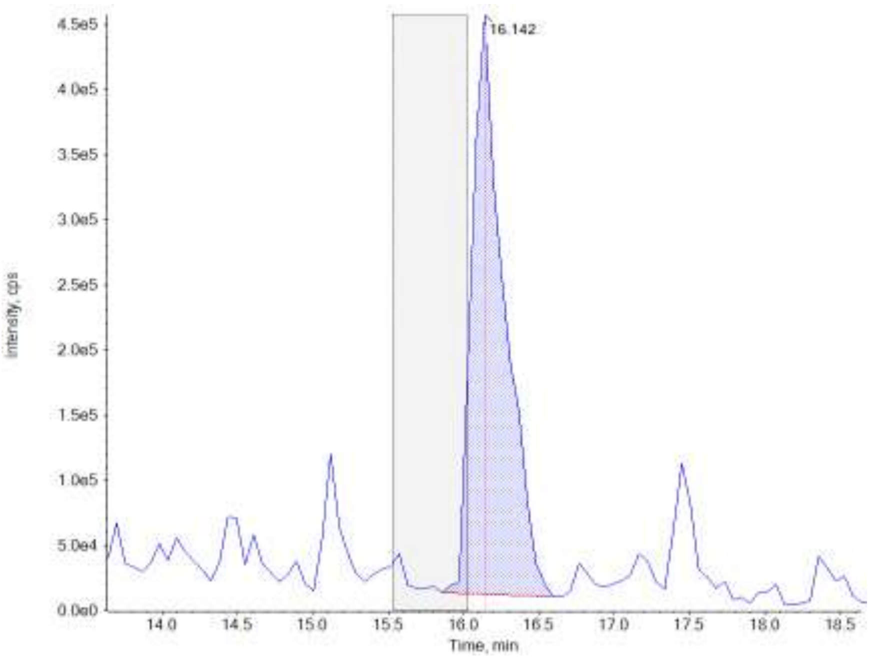

Acquired / Library MSMS

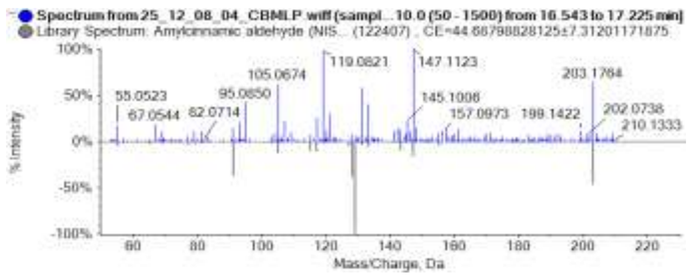

Acquired / Theoretical MS

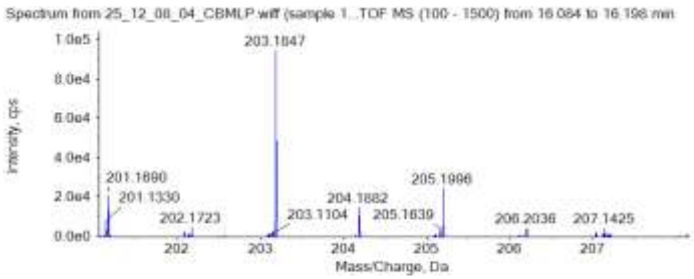

337.2582 / 16.14 (Mass/FragMass/RT/Isotope/Library/Formula/Ion Ratio)

Retention Time: 16.14 minutes
Precursor m/z : 337.2582
Fit (%) 56.0% **RFit (%)** 90.9%

Exp RT: 16.14 minutes
Analyte Name:
337.2582 / 16.14

Collision Energy = 35 ± 15 eV

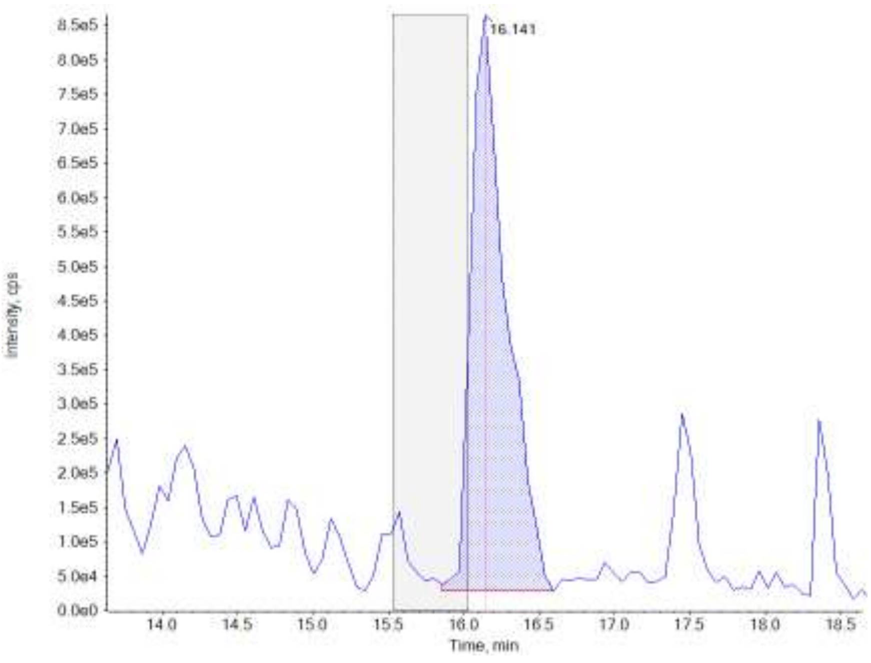

Acquired / Library MSMS

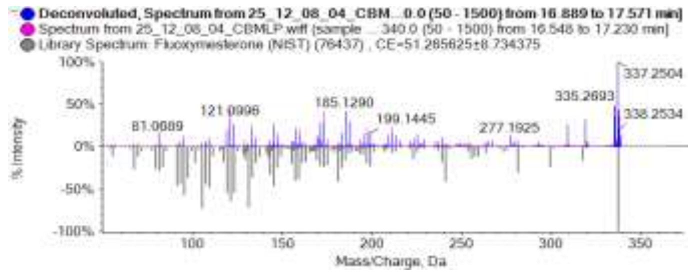

Acquired / Theoretical MS

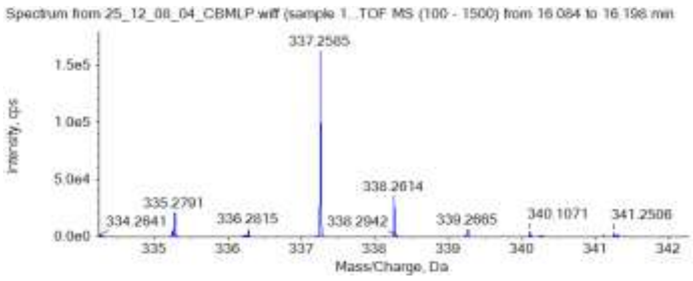

419.3425 / 16.14 (Mass/FragMass/RT/Isotope/Library/Formula/Ion Ratio)

**Retention Time:** 16.16 minutes	**Exp RT:** 16.14 minutes
**Precursor m/z :** 419.3425	**Analyte Name:**
**Fit (%)** 73.6% **RFit (%)** 88.5%	419.3425 / 16.14

Collision Energy = 35 ± 15 eV

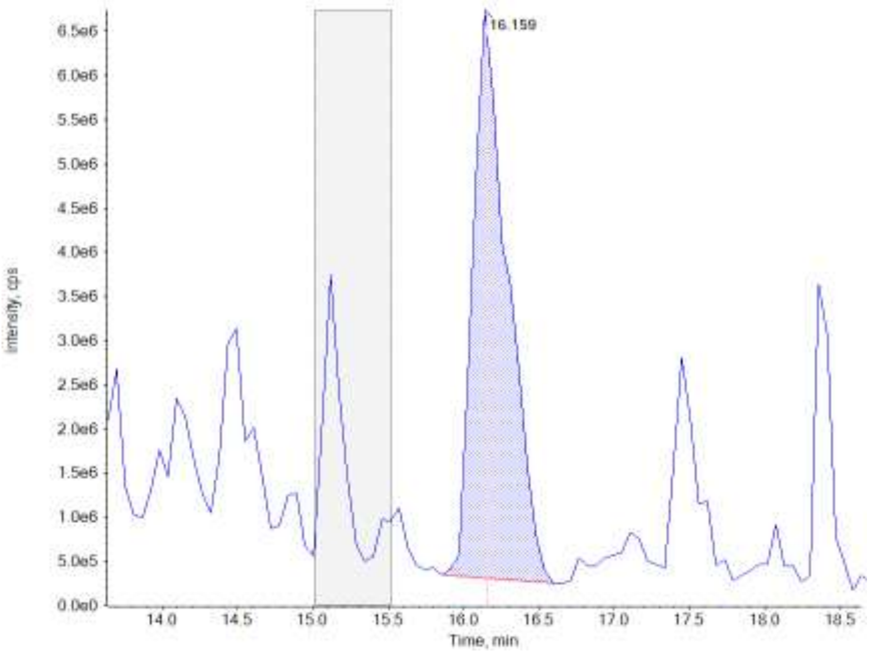

Acquired / Library MSMS

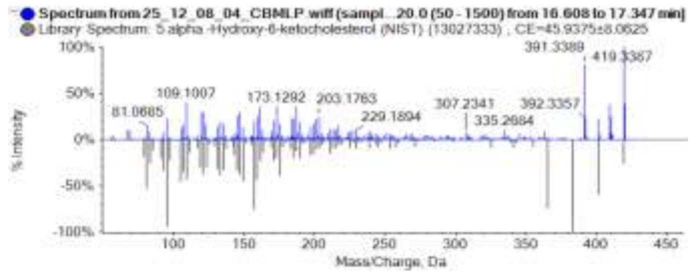

Acquired / Theoretical MS

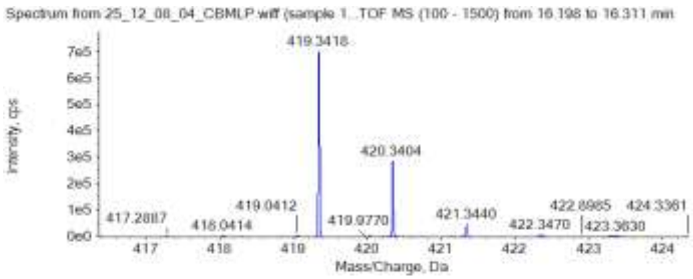

Created with SCIEX OS 3.3.0
Printed:12/9/2025 2:04:37 PM

455.3599 / 16.14

(Mass/FragMass/RT/Isotope/Library/Formula/Ion Ratio)

Retention Time: 16.14 minutes
Precursor m/z : 455.3599
Fit (%) 94.6% **RFit (%)** 79.4%

Exp RT: 16.14 minutes
Analyte Name:
455.3599 / 16.14

Collision Energy = 35 ± 15 eV

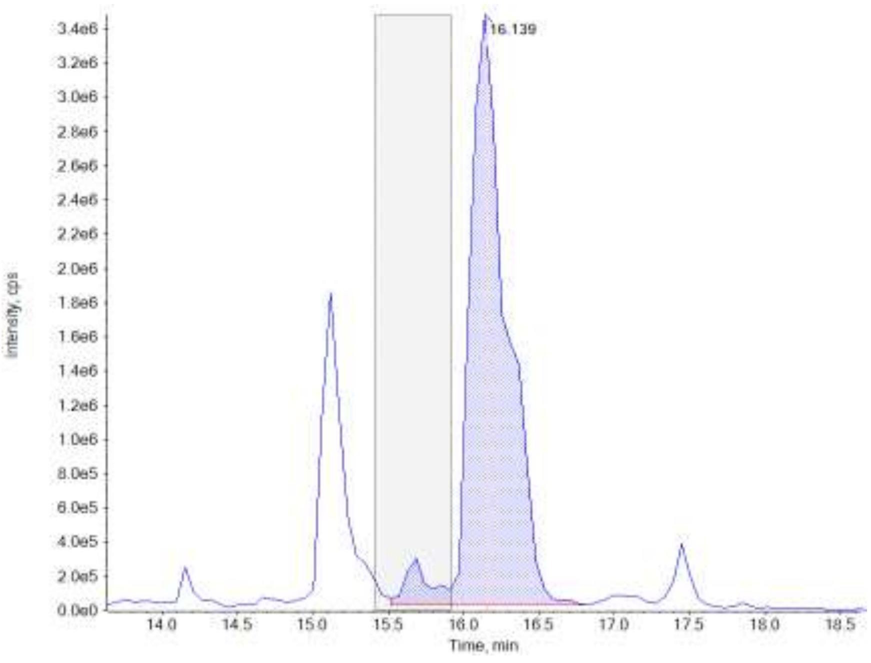

Acquired / Library MSMS

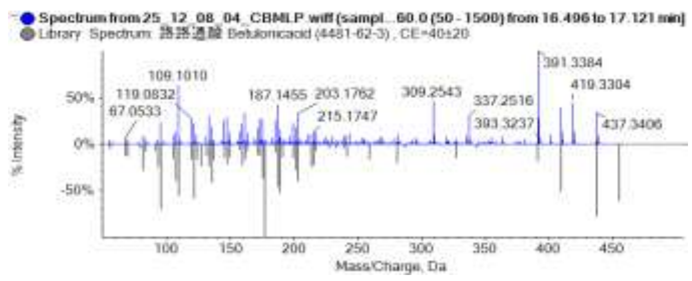

Acquired / Theoretical MS

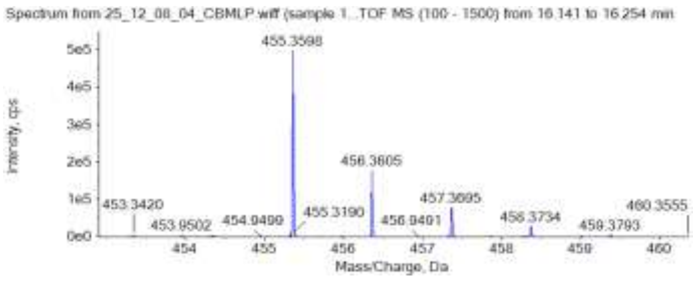

423.3671 / 16.54 (Mass/FragMass/RT/Isotope/Library/Formula/Ion Ratio)

**Retention Time:** 16.55 minutes<br>**Precursor m/z :** 423.3671<br>**Fit (%)** 99.9% **RFit (%)** 74.0%	**Exp RT:** 16.54 minutes<br>**Analyte Name:**<br>423.3671 / 16.54

Collision Energy = 35 ± 15 eV

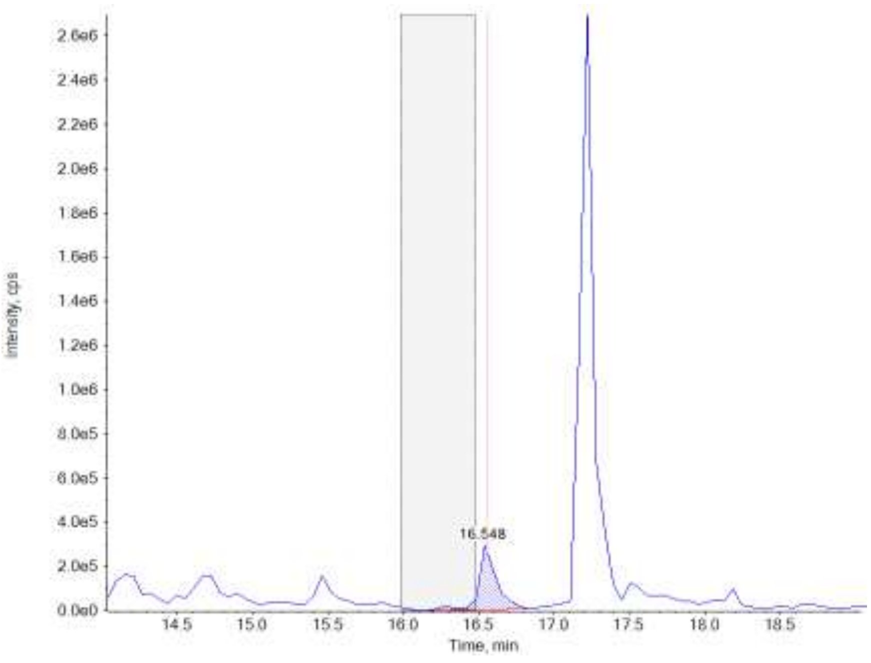

Acquired / Library MSMS

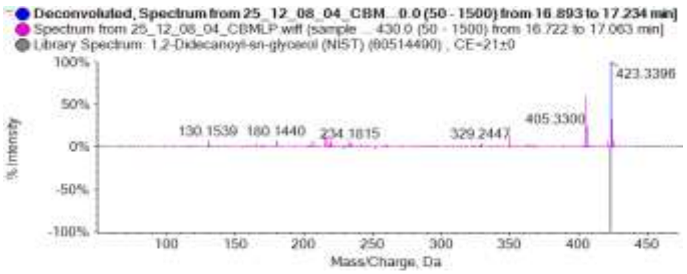

Acquired / Theoretical MS

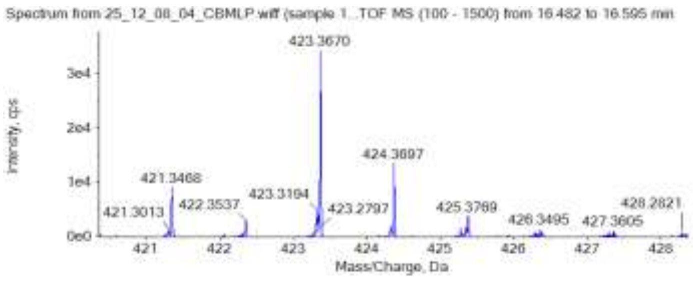

277.2252 / 16.60

(Mass/FragMass/RT/Isotope/Library/Formula/Ion Ratio)

Retention Time: 16.60 minutes
Precursor m/z : 277.2252
Fit (%) 93.5% **RFit (%)** 93.6%

Exp RT: 16.60 minutes
Analyte Name:
277.2252 / 16.60

Collision Energy = 35 ± 15 eV

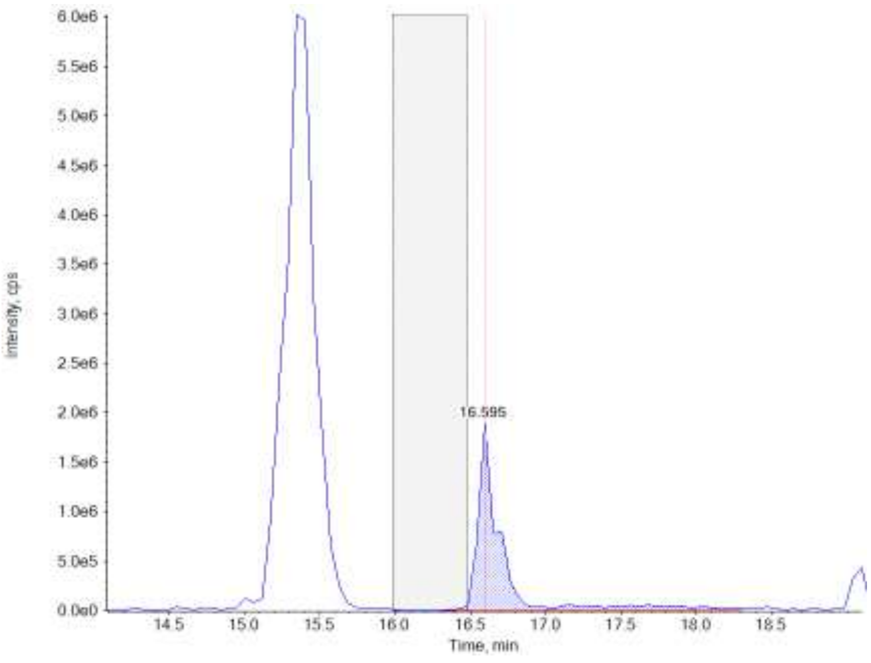

Acquired / Library MSMS

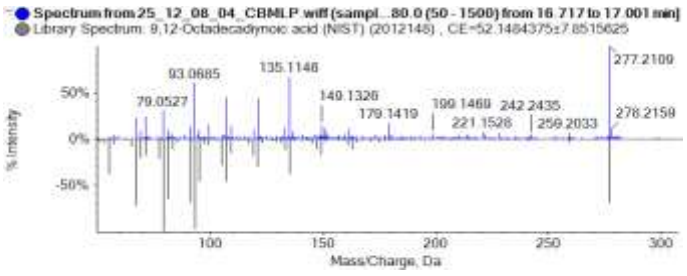

Acquired / Theoretical MS

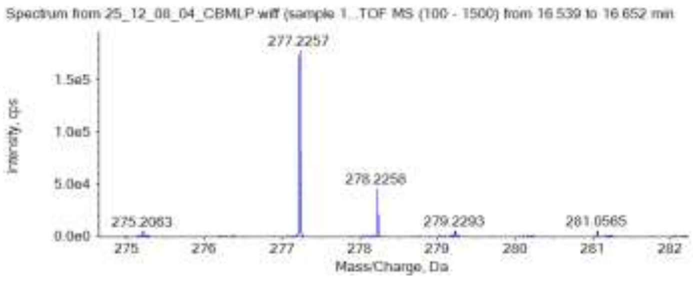

Created with SCIEX OS 3.3.0
Printed:12/9/2025 2:04:37 PM

295.2349 / 16.60

(Mass/FragMass/RT/Isotope/Library/Formula/Ion Ratio)

Retention Time: 16.64 minutes
Precursor m/z : 295.2349
Fit (%) 30.2% **RFit (%)** 99.1%

Exp RT: 16.60 minutes
Analyte Name:
295.2349 / 16.60

Collision Energy = 35 ± 15 eV

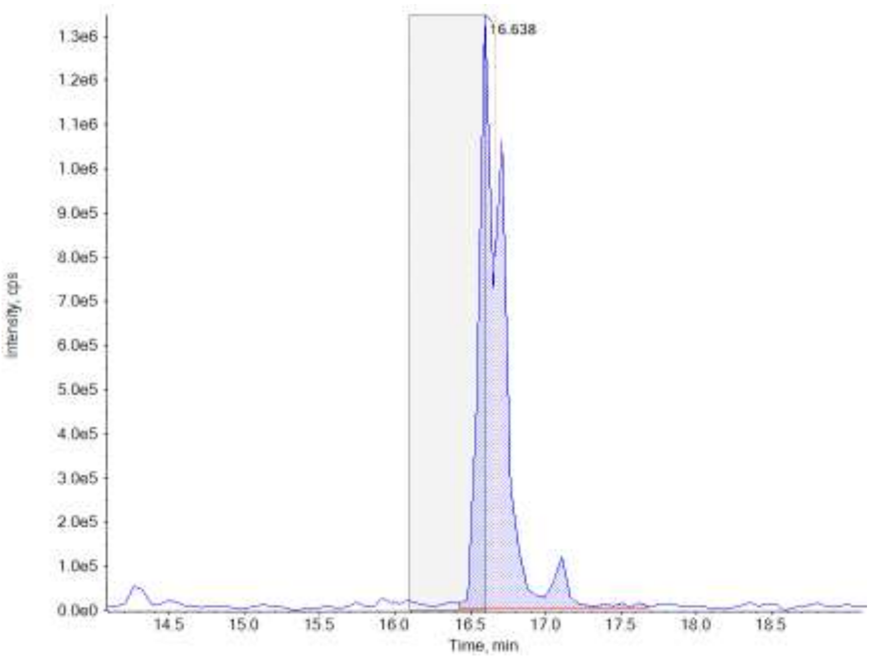

Acquired / Library MSMS

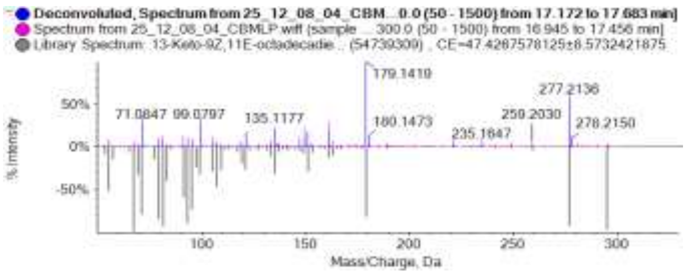

Acquired / Theoretical MS

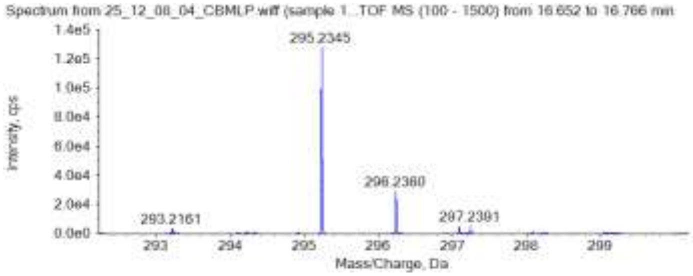

447.2544 / 16.60 (Mass/FragMass/RT/Isotope/Library/Formula/Ion Ratio)

**Retention Time:** 16.59 minutes	**Exp RT:** 16.60 minutes
**Precursor m/z :** 447.2544	**Analyte Name:**
**Fit (%)** 98.0% **RFit (%)** 84.6%	447.2544 / 16.60

Collision Energy = 35 ± 15 eV

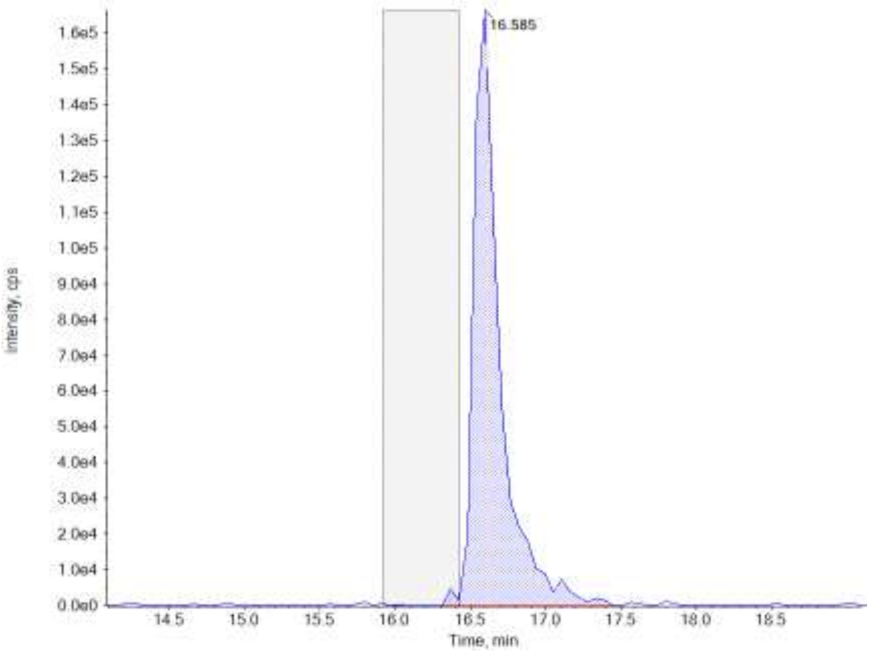

Acquired / Library MSMS

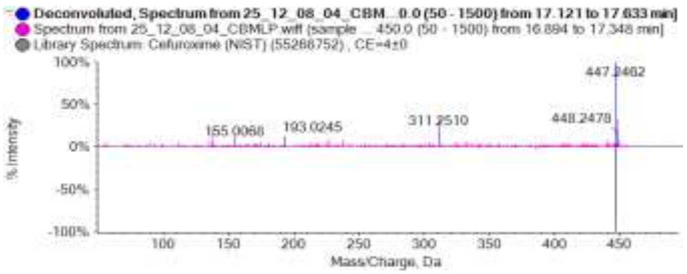

Acquired / Theoretical MS

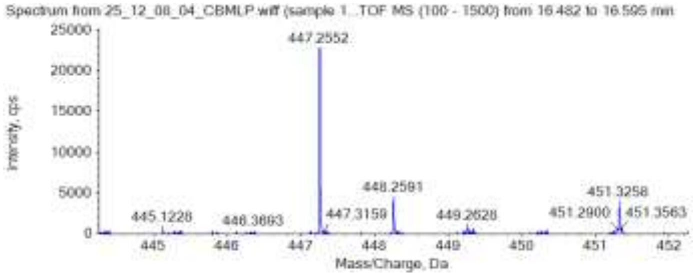

523.3496 / 16.77 (Mass/FragMass/RT/Isotope/Library/Formula/Ion Ratio)

Retention Time: 16.78 minutes
Precursor m/z : 523.3496
Fit (%) 58.1% **RFit (%)** 82.5%

Exp RT: 16.77 minutes
Analyte Name:
523.3496 / 16.77

Collision Energy = 35 ± 15 eV

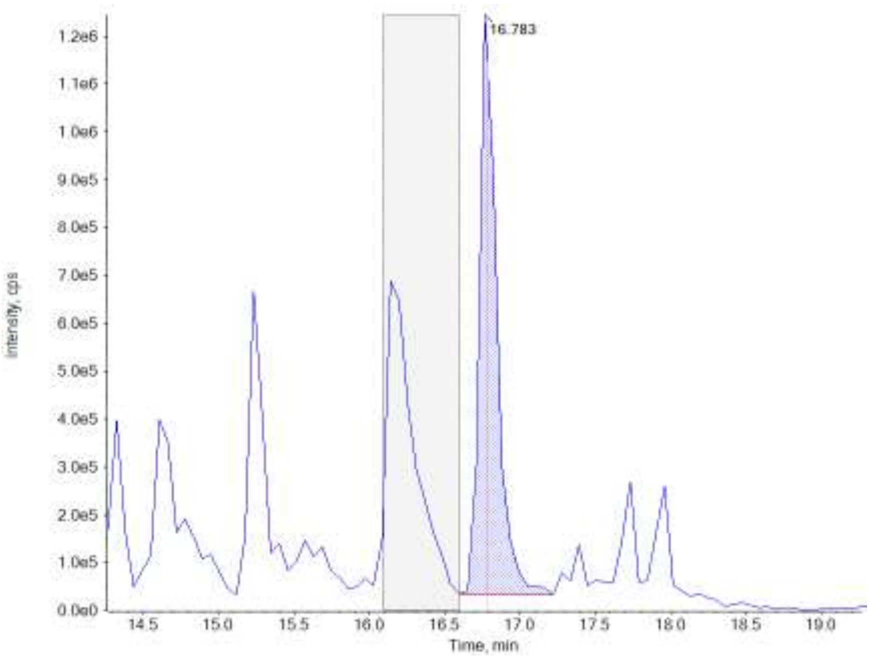

Acquired / Library MSMS

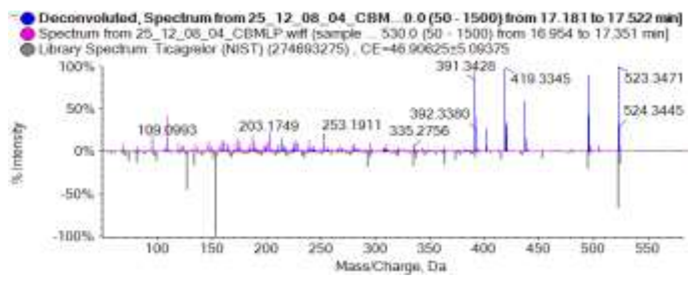

Acquired / Theoretical MS

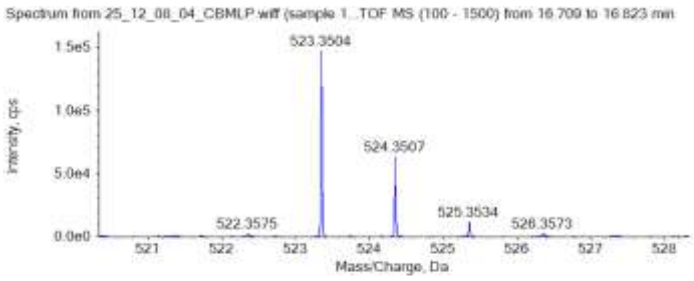

423.3171 / 16.82 (Mass/FragMass/RT/Isotope/Library/Formula/Ion Ratio)

Retention Time: 16.82 minutes
Precursor m/z : 423.3171
Fit (%) 100.0% **RFit (%)** 54.2%

Exp RT: 16.82 minutes
Analyte Name:
423.3171 / 16.82

Collision Energy = 35 ± 15 eV

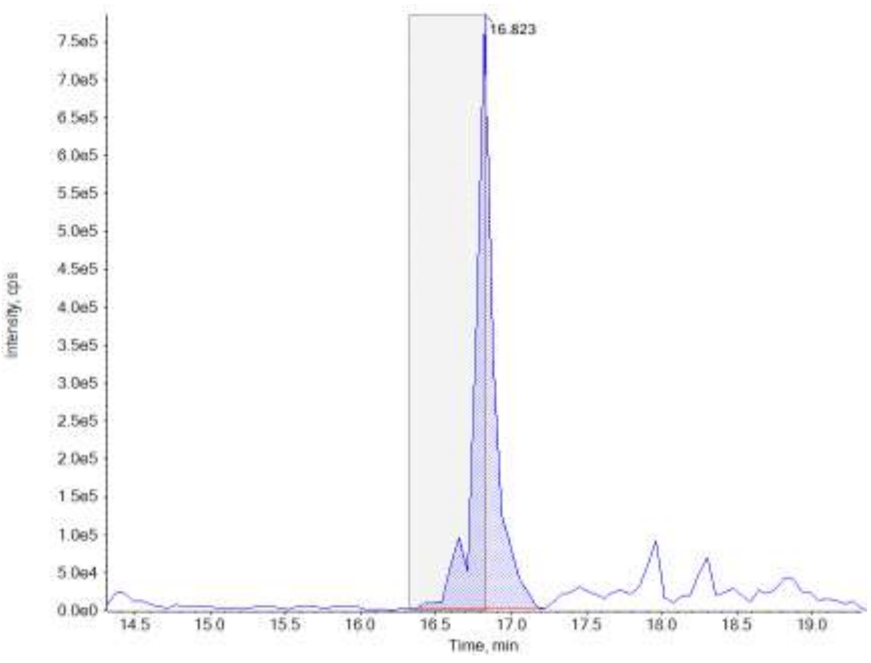

Acquired / Library MSMS

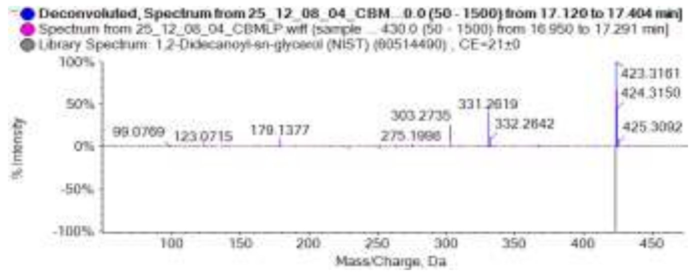

Acquired / Theoretical MS

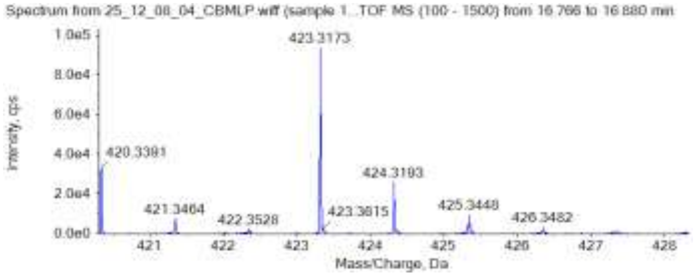

355.2892 / 16.94

(Mass/FragMass/RT/Isotope/Library/Formula/Ion Ratio)

**Retention Time:** 17.10 minutes	**Exp RT:** 16.94 minutes
**Precursor m/z :** 355.2892	**Analyte Name:**
**Fit (%)** 54.0% **RFit (%)** 92.5%	355.2892 / 16.94

Collision Energy = 35 ± 15 eV

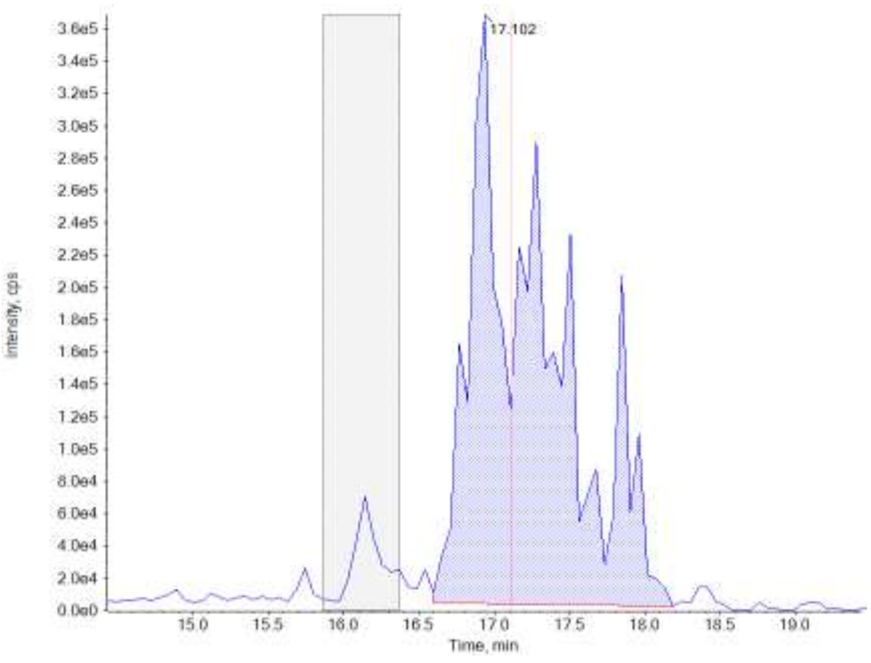

Acquired / Library MSMS

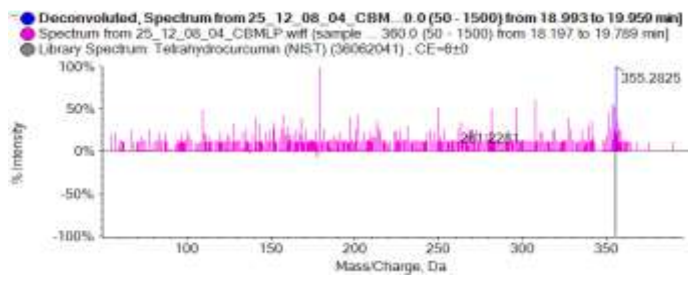

Acquired / Theoretical MS

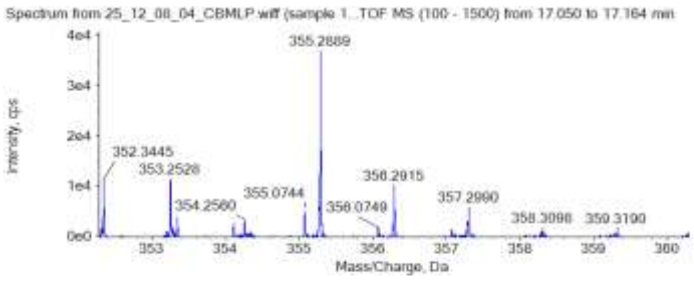

154.0545 / 16.99 (Mass/FragMass/RT/Isotope/Library/Formula/Ion Ratio)

Retention Time: 16.99 minutes
Precursor m/z : 154.0545
Fit (%) 94.3% **RFit (%)** 62.6%

Exp RT: 16.99 minutes
Analyte Name:
154.0545 / 16.99

Collision Energy = 35 ± 15 eV

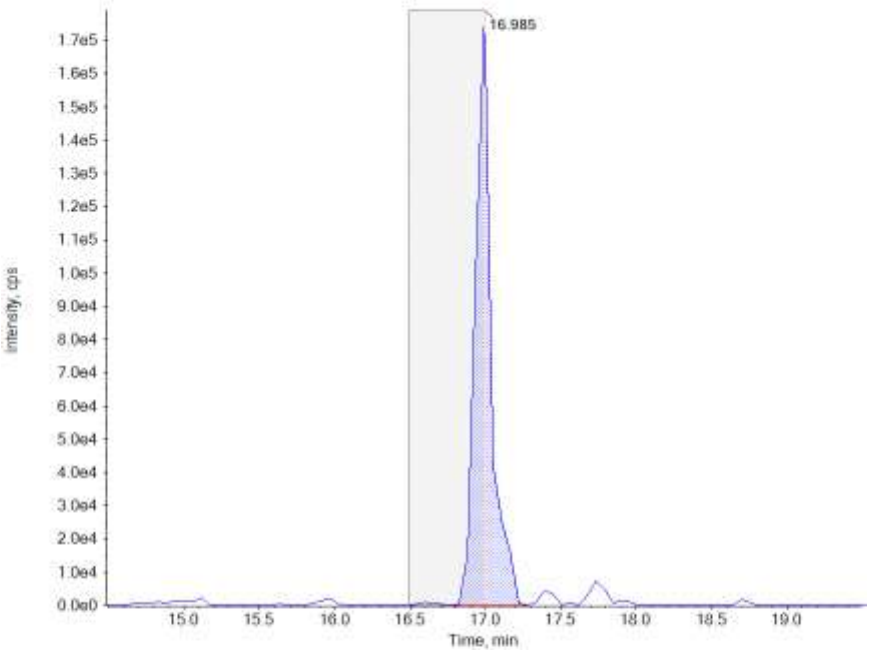

Acquired / Library MSMS

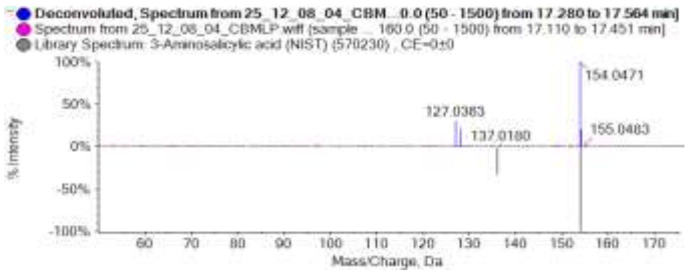

Acquired / Theoretical MS

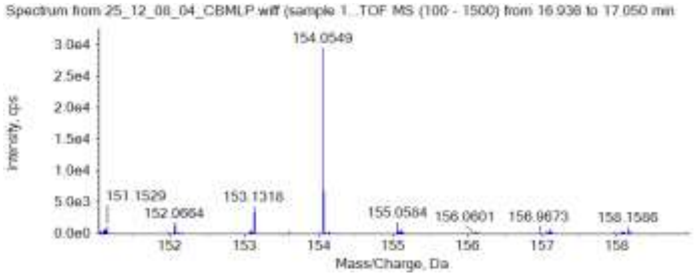

599.4141 / 17.11 (Mass/FragMass/RT/Isotope/Library/Formula/Ion Ratio)

**Retention Time:** 17.31 minutes	**Exp RT:** 17.11 minutes
**Precursor m/z :** 599.4141	**Analyte Name:**
**Fit (%)** 35.3% **RFit (%)** 94.0%	599.4141 / 17.11

Collision Energy = 35 ± 15 eV

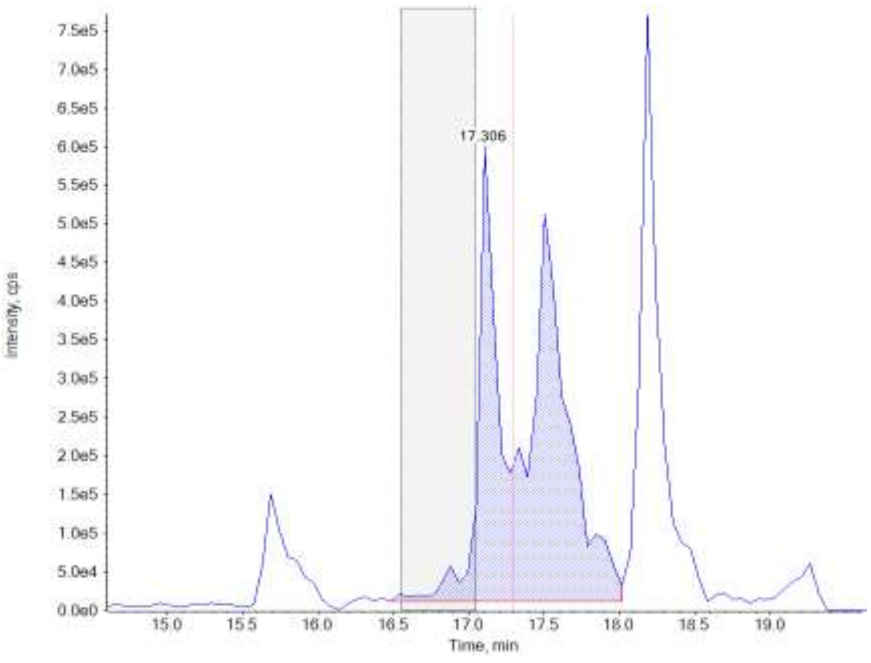

Acquired / Library MSMS

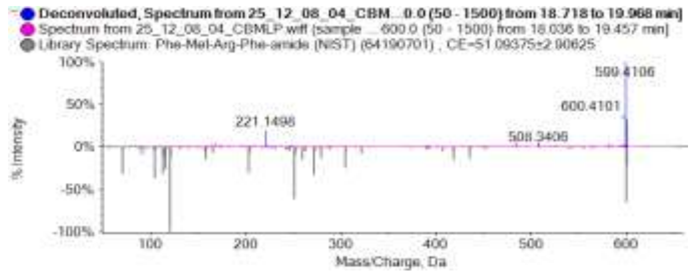

Acquired / Theoretical MS

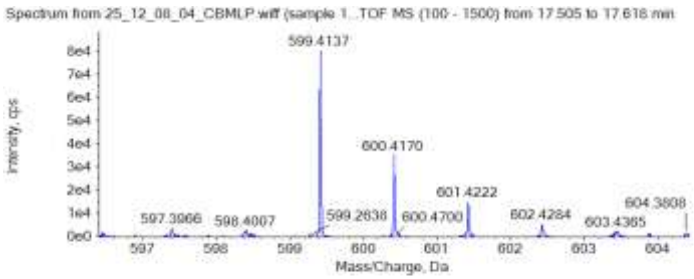

Created with SCIEX OS 3.3.0
Printed:12/9/2025 2:04:37 PM

453.3430 / 17.16 (Mass/FragMass/RT/Isotope/Library/Formula/Ion Ratio)

Retention Time: 16.97 minutes
Precursor m/z : 453.3430
Fit (%) 100.0% **RFit (%)** 58.5%

Exp RT: 17.16 minutes
Analyte Name:
453.3430 / 17.16

Collision Energy = 35 ± 15 eV

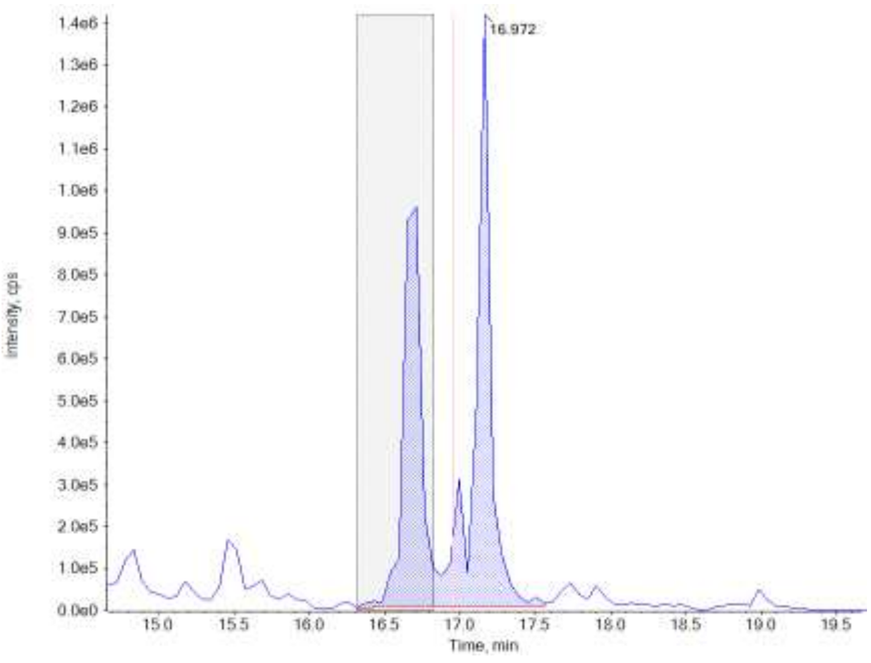

Acquired / Library MSMS

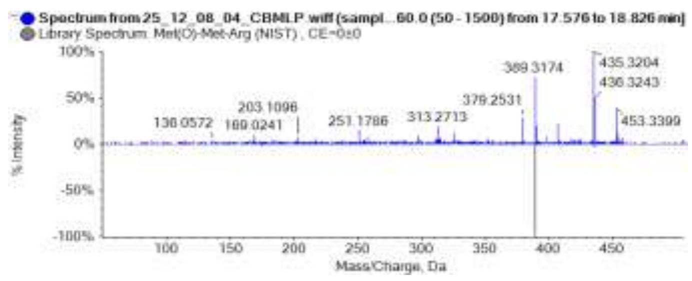

Acquired / Theoretical MS

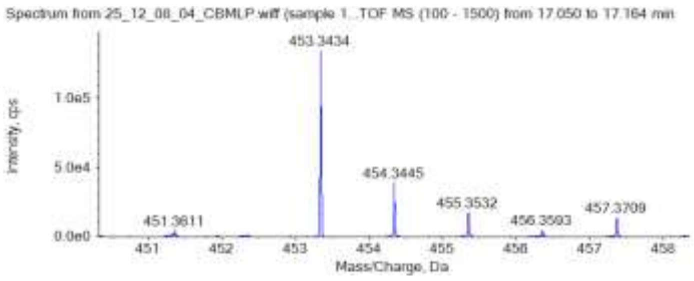

349.2821 / 17.33 (Mass/FragMass/RT/Isotope/Library/Formula/Ion Ratio)

Retention Time: 17.33 minutes
Precursor m/z : 349.2821
Fit (%) 100.0% **RFit (%)** 50.5%

Exp RT: 17.33 minutes
Analyte Name:
349.2821 / 17.33

Collision Energy = 35 ± 15 eV

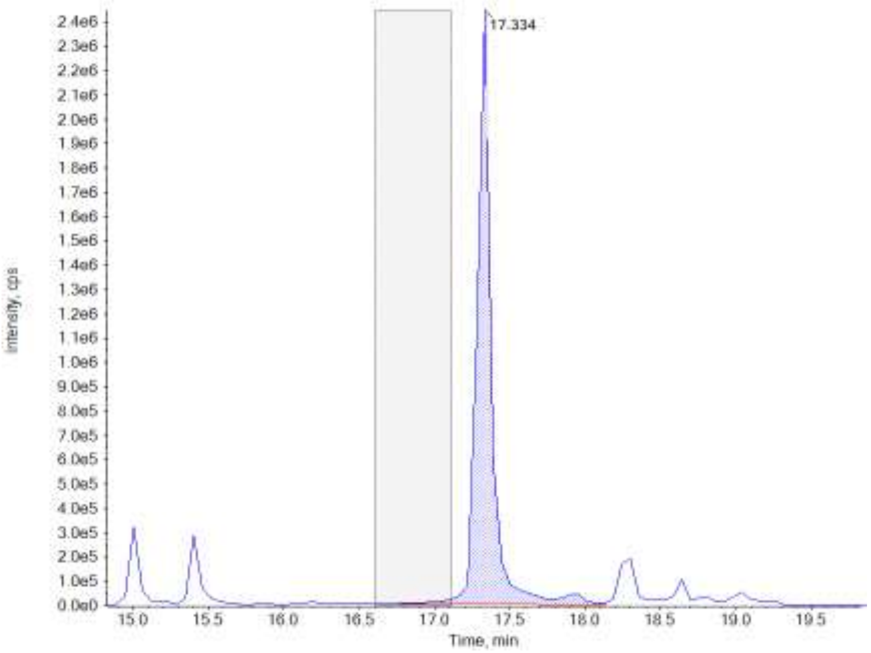

Acquired / Library MSMS

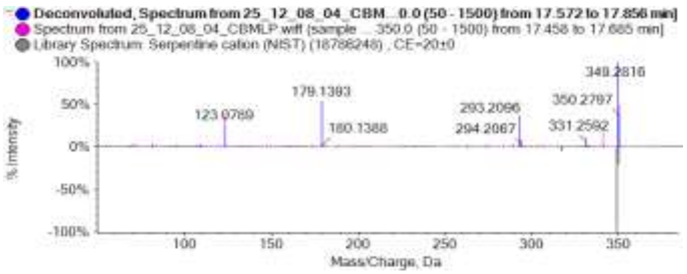

Acquired / Theoretical MS

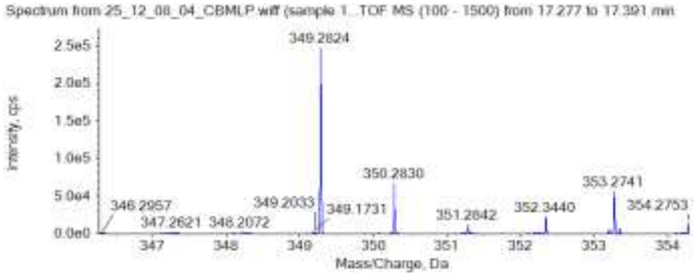

341.3105 / 17.39 (Mass/FragMass/RT/Isotope/Library/Formula/Ion Ratio)

Retention Time: 17.38 minutes
Precursor m/z : 341.3105
Fit (%) 95.5% **RFit (%)** 98.5%

Exp RT: 17.39 minutes
Analyte Name:
341.3105 / 17.39

Collision Energy = 35 ± 15 eV

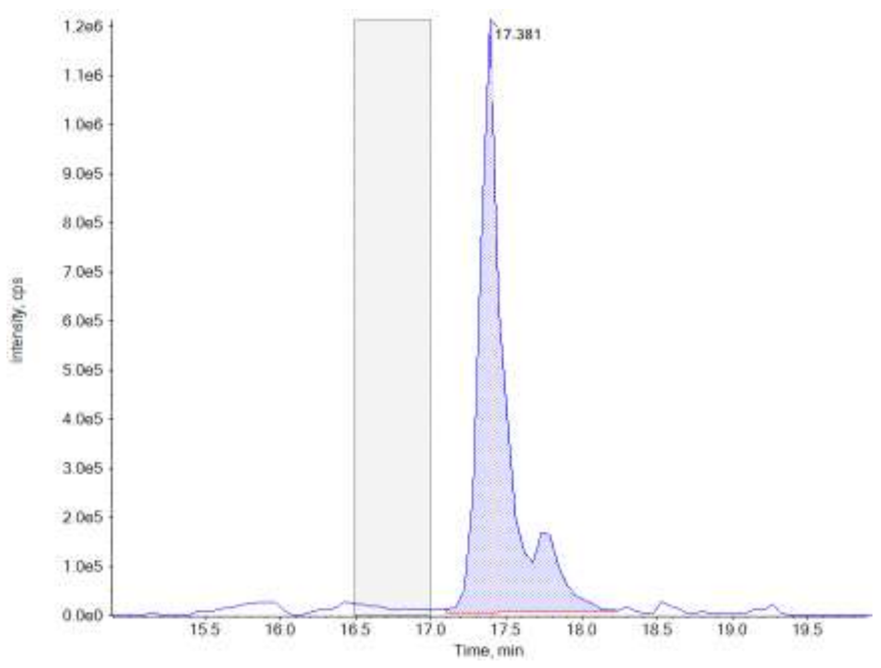

Acquired / Library MSMS

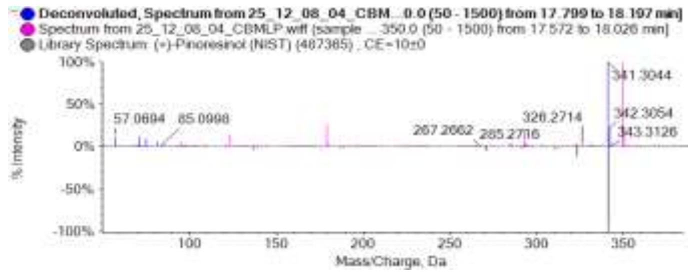

Acquired / Theoretical MS

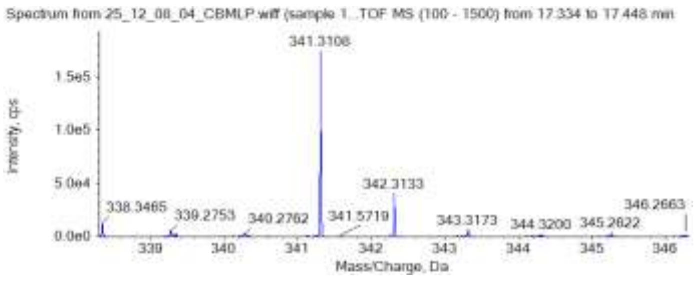

359.3216 / 17.39 (Mass/FragMass/RT/Isotope/Library/Formula/Ion Ratio)

**Retention Time:** 17.38 minutes	**Exp RT:** 17.39 minutes
**Precursor m/z :** 359.3216	**Analyte Name:**
**Fit (%)** 92.6% **RFit (%)** 96.0%	359.3216 / 17.39

Collision Energy = 35 ± 15 eV

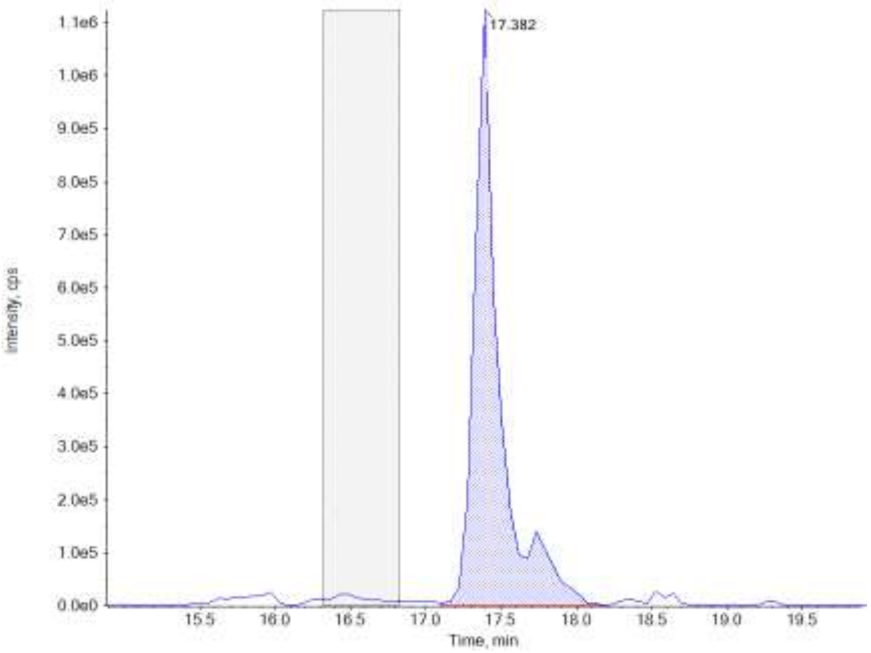

Acquired / Library MSMS

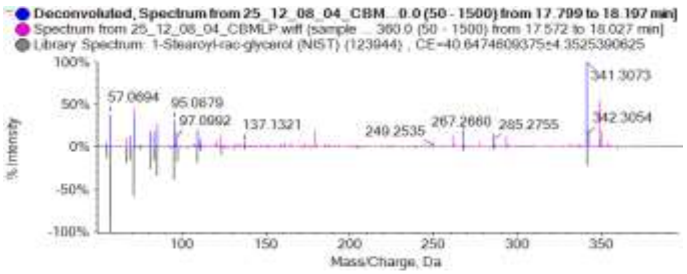

Acquired / Theoretical MS

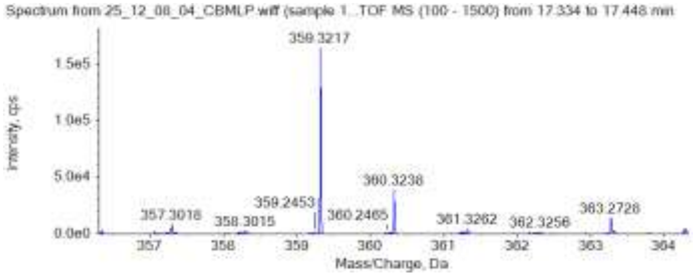

Created with SCIEX OS 3.3.0
Printed:12/9/2025 2:04:37 PM

607.2953 / 17.39 (Mass/FragMass/RT/Isotope/Library/Formula/Ion Ratio)

Retention Time: 17.40 minutes
Precursor m/z : 607.2953
Fit (%) 74.4% **RFit (%)** 94.5%

Exp RT: 17.39 minutes
Analyte Name:
607.2953 / 17.39

Collision Energy = 35 ± 15 eV

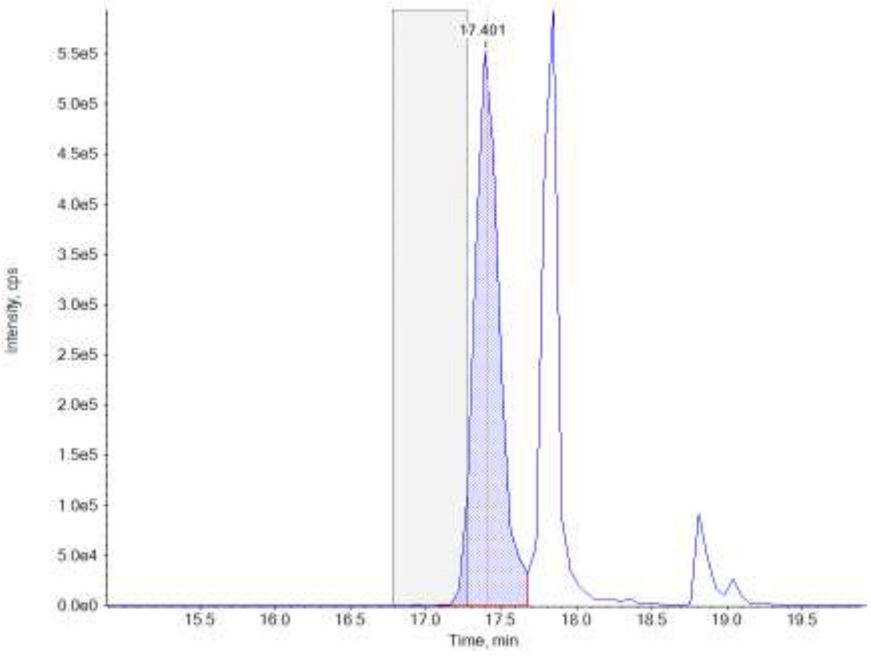

Acquired / Library MSMS

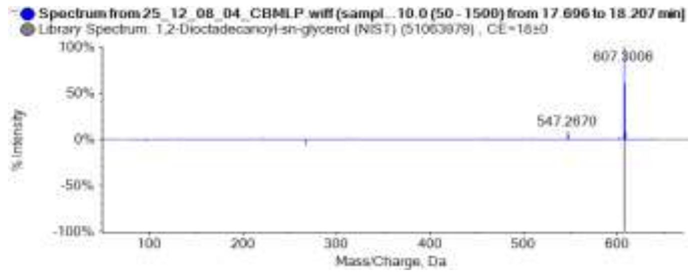

Acquired / Theoretical MS

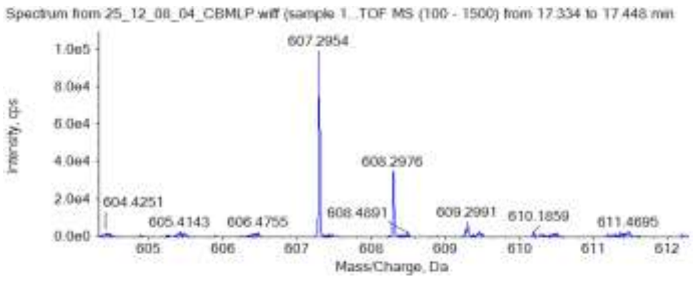

Created with SCIEX OS 3.3.0
Printed:12/9/2025 2:04:37 PM

353.2761 / 17.45

(Mass/FragMass/RT/Isotope/Library/Formula/Ion Ratio)

Retention Time: 17.47 minutes
Precursor m/z : 353.2761
Fit (%) 97.6% **RFit (%)** 99.2%

Exp RT: 17.45 minutes
Analyte Name:
353.2761 / 17.45

Collision Energy = 35 ± 15 eV

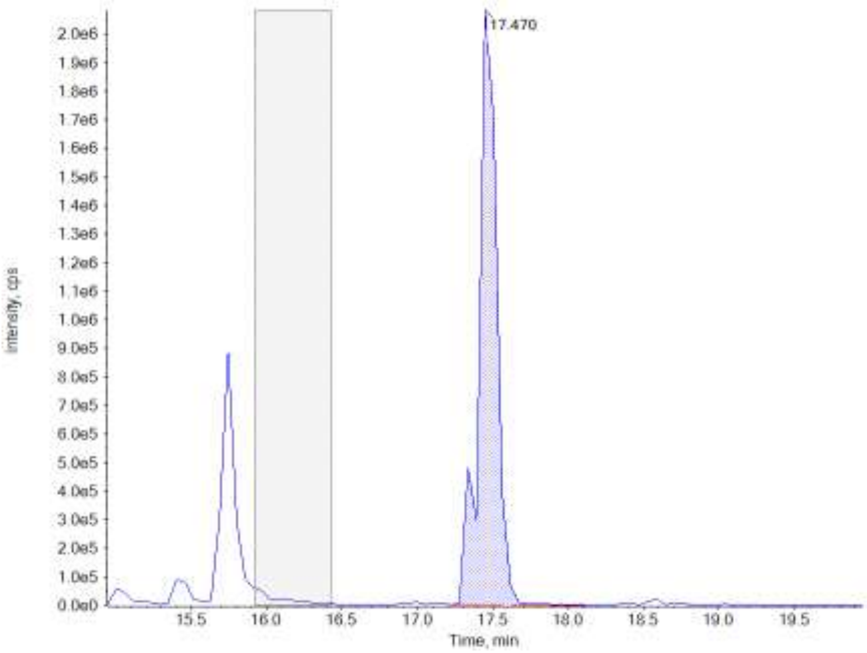

Acquired / Library MSMS

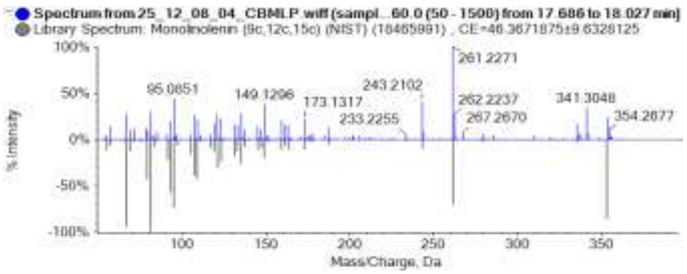

Acquired / Theoretical MS

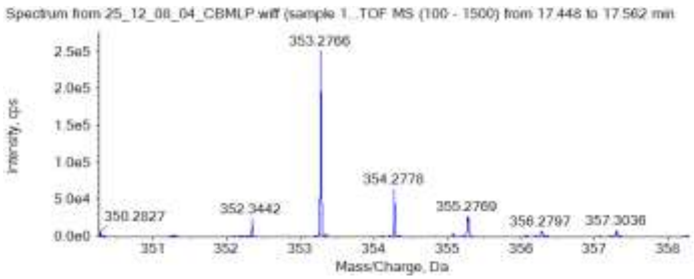

391.3408 / 17.45 (Mass/FragMass/RT/Isotope/Library/Formula/Ion Ratio)

**Retention Time:** 17.46 minutes	**Exp RT:** 17.45 minutes
**Precursor m/z :** 391.3408	**Analyte Name:**
**Fit (%)** 100.0% **RFit (%)** 67.2%	391.3408 / 17.45

Collision Energy = 35 ± 15 eV

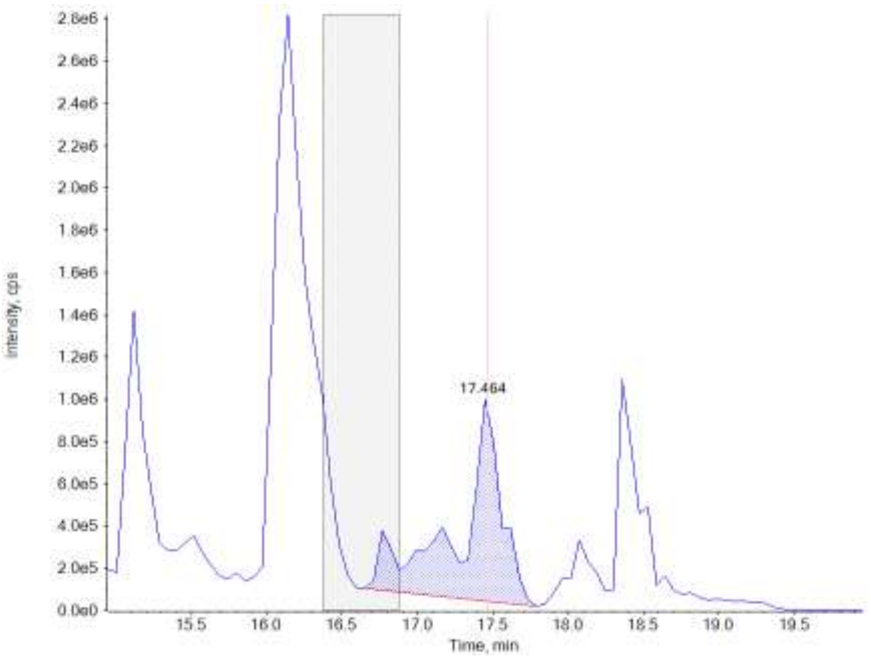

Acquired / Library MSMS

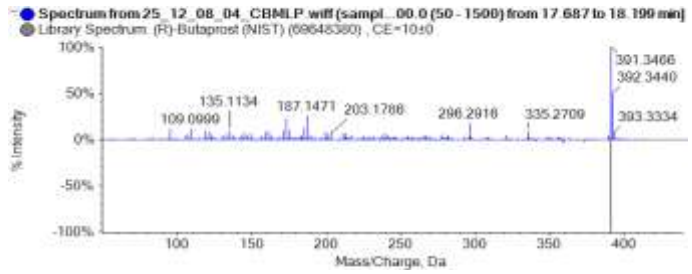

Acquired / Theoretical MS

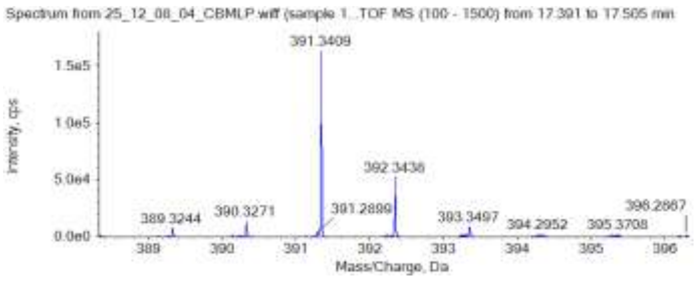

419.3375 / 17.45 (Mass/FragMass/RT/Isotope/Library/Formula/Ion Ratio)

Retention Time: 17.46 minutes
Precursor m/z : 419.3375
Fit (%) 59.7% **RFit (%)** 88.1%

Exp RT: 17.45 minutes
Analyte Name:
419.3375 / 17.45

Collision Energy = 35 ± 15 eV

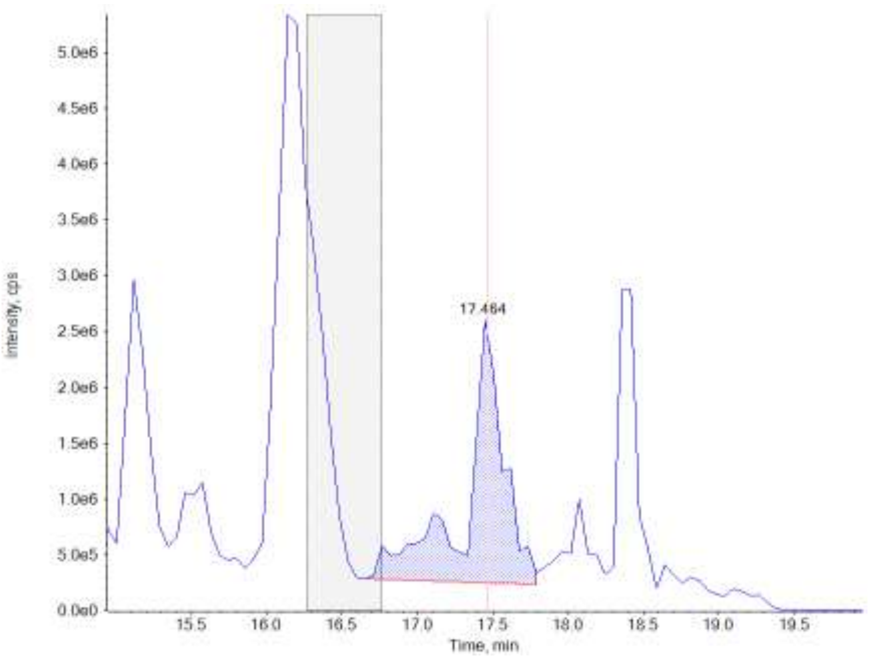

Acquired / Library MSMS

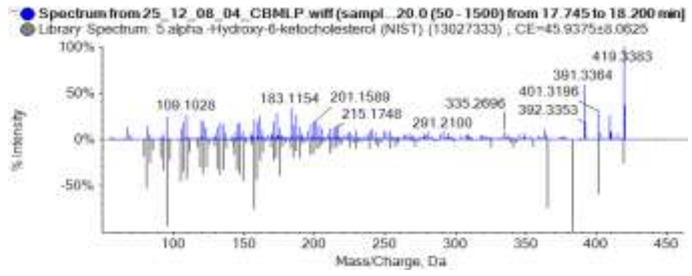

Acquired / Theoretical MS

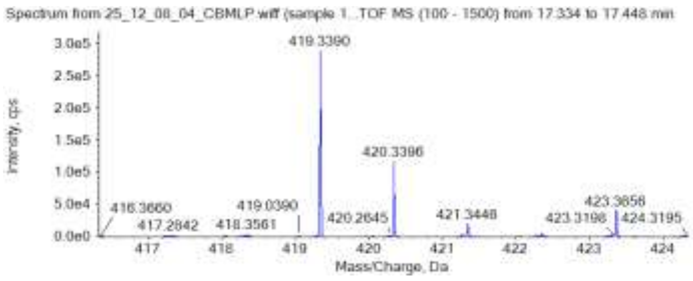

459.3873 / 17.50 (Mass/FragMass/RT/Isotope/Library/Formula/Ion Ratio)

Retention Time: 17.51 minutes
Precursor m/z : 459.3873
Fit (%) 93.5% **RFit (%)** 48.2%

Exp RT: 17.51 minutes
Analyte Name:
459.3873 / 17.50

Collision Energy = 35 ± 15 eV

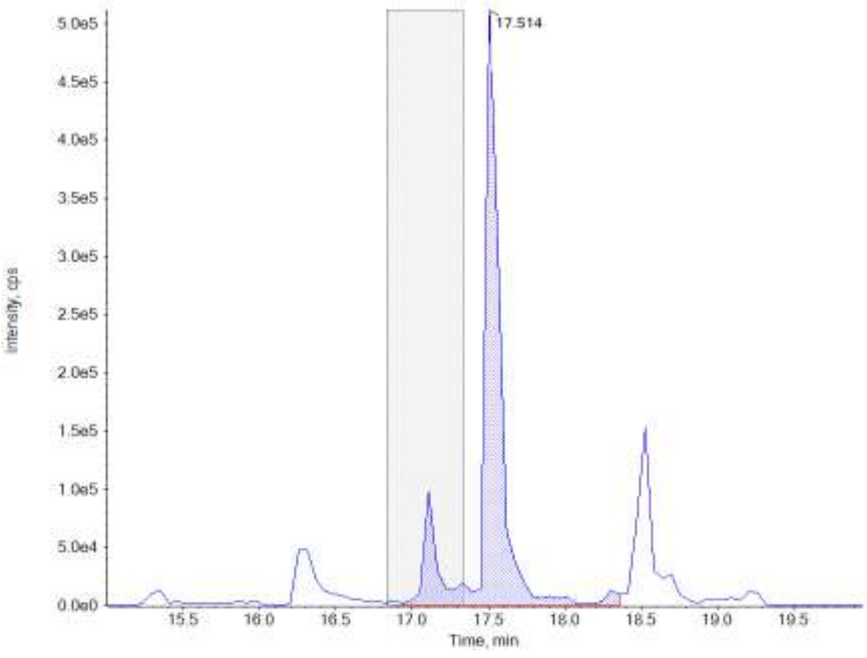

Acquired / Library MSMS

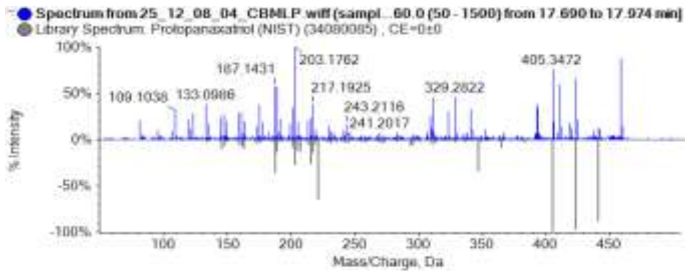

Acquired / Theoretical MS

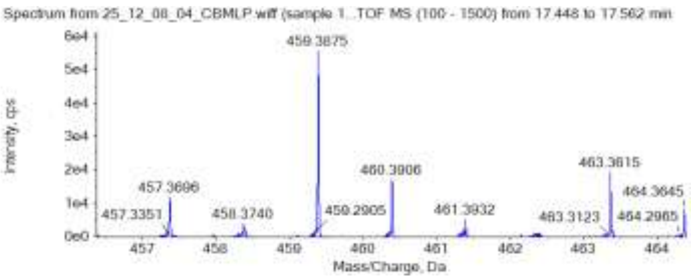

305.2556 / 17.56 (Mass/FragMass/RT/Isotope/Library/Formula/Ion Ratio)

**Retention Time:** 17.56 minutes<br>**Precursor m/z :** 305.2556<br>**Fit (%)** 98.2% **RFit (%)** 99.4%	**Exp RT:** 17.56 minutes<br>**Analyte Name:**<br>305.2556 / 17.56

		**Collision Energy = 35 ± 15 eV**
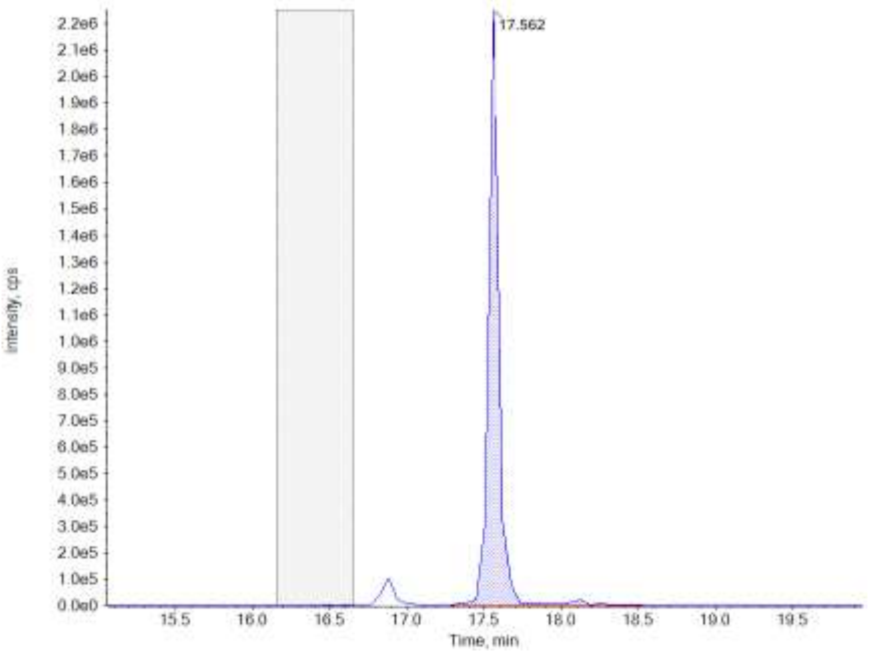	**Acquired / Library MSMS**	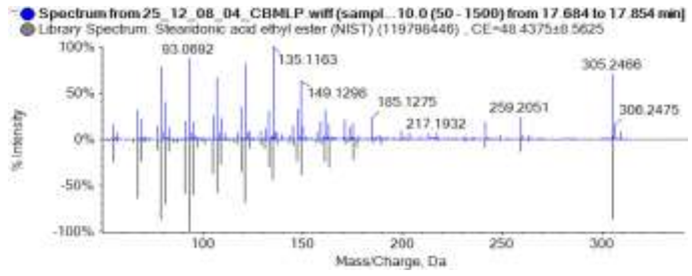
	**Acquired / Theoretical MS**	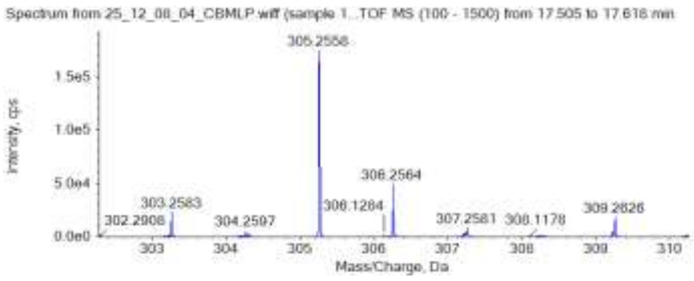

623.2548 / 17.68

(Mass/FragMass/RT/Isotope/Library/Formula/Ion Ratio)

**Retention Time:** 17.59 minutes	**Exp RT:** 17.68 minutes
**Precursor m/z :** 623.2548	**Analyte Name:**
**Fit (%)** 68.1% **RFit (%)** 71.5%	623.2548 / 17.68

Collision Energy = 35 ± 15 eV

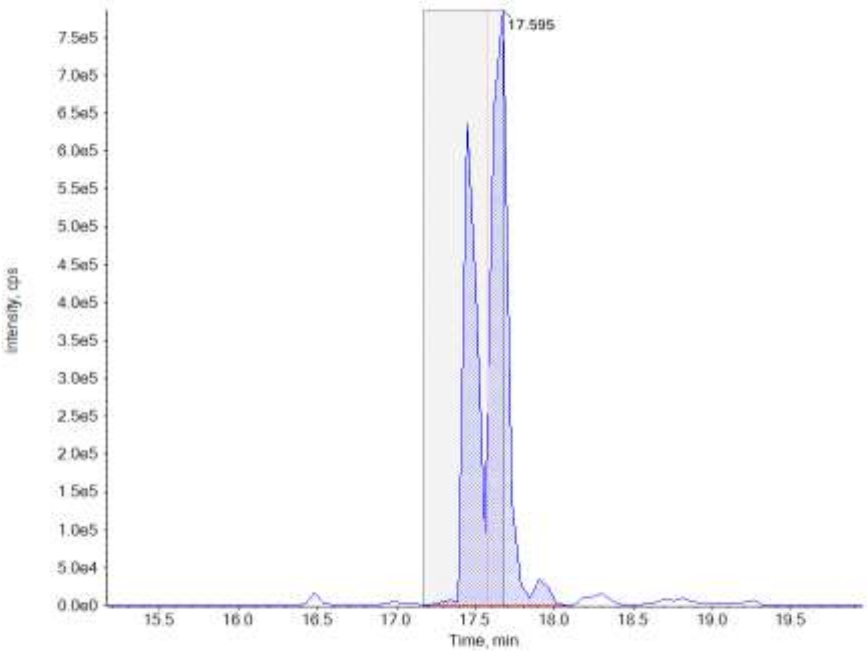

Acquired / Library MSMS

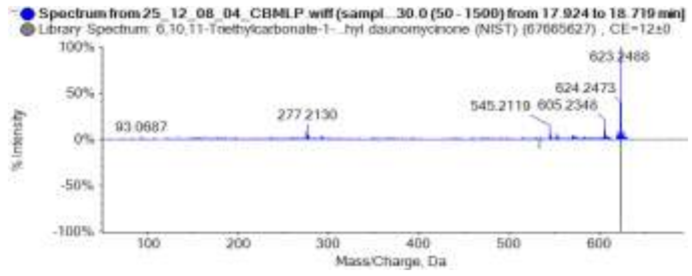

Acquired / Theoretical MS

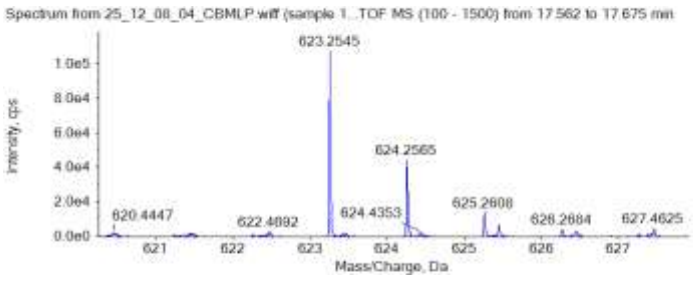

421.3513 / 17.73 (Mass/FragMass/RT/Isotope/Library/Formula/Ion Ratio)

Retention Time: 17.73 minutes
Precursor m/z : 421.3512
Fit (%) 92.3% **RFit (%)** 78.2%

Exp RT: 17.73 minutes
Analyte Name:
421.3513 / 17.73

Collision Energy = 35 ± 15 eV

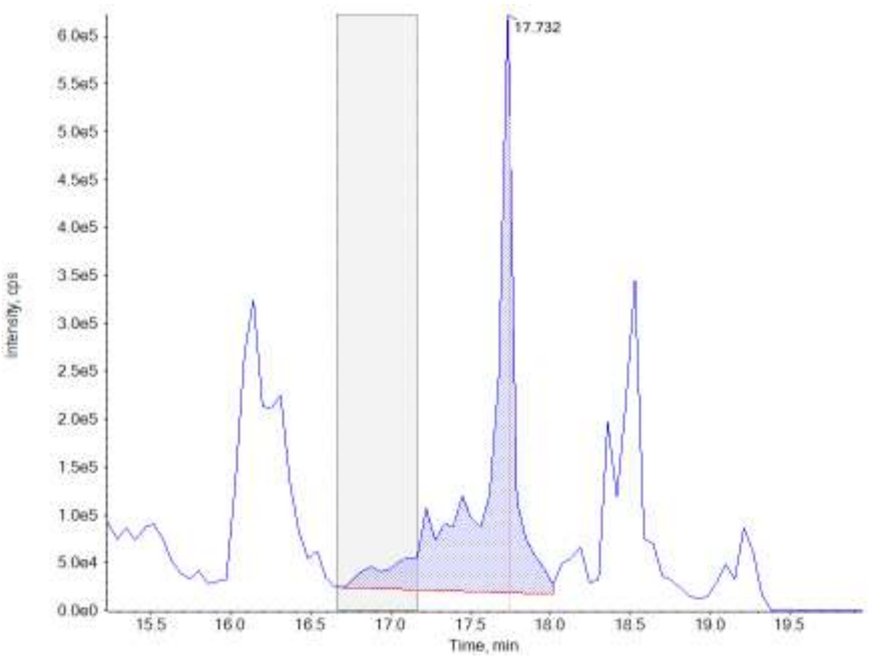

Acquired / Library MSMS

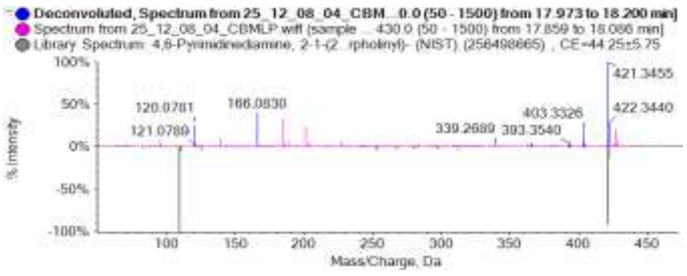

Acquired / Theoretical MS

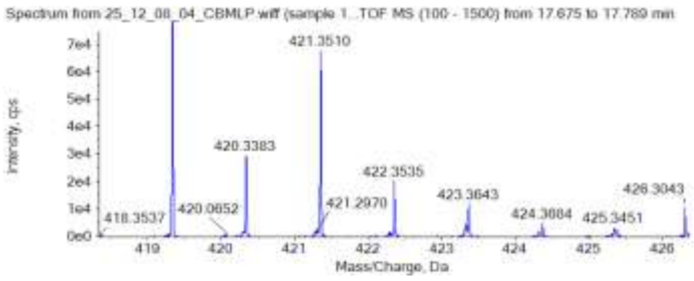

149.0282 / 17.79 (Mass/FragMass/RT/Isotope/Library/Formula/Ion Ratio)

**Retention Time:** 17.80 minutes	**Exp RT:** 17.79 minutes
**Precursor m/z :** 149.0282	**Analyte Name:**
**Fit (%)** 100.0% **RFit (%)** 95.0%	149.0282 / 17.79

Collision Energy = 35 ± 15 eV

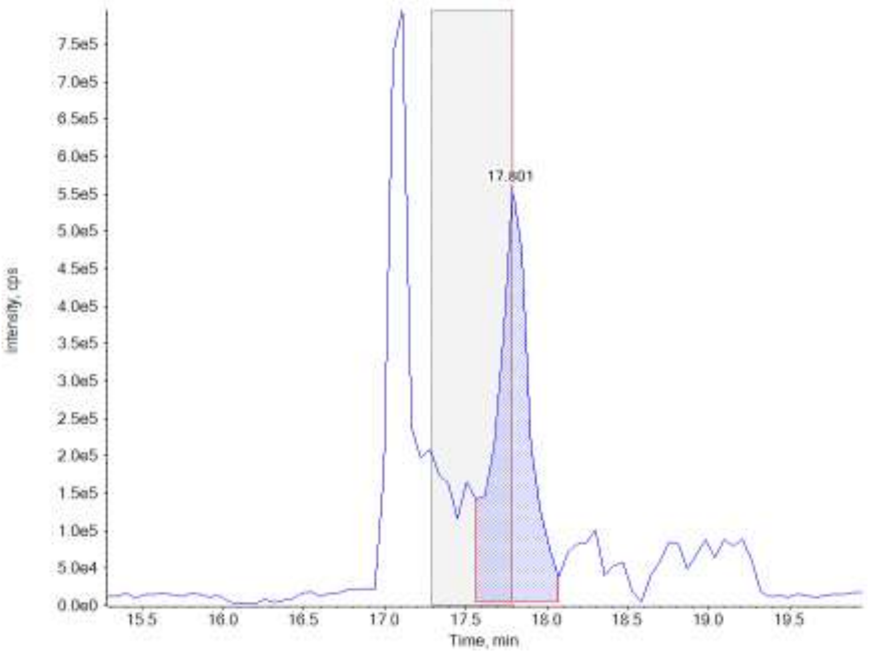

Acquired / Library MSMS

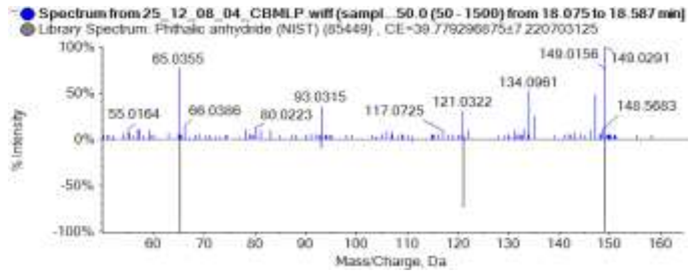

Acquired / Theoretical MS

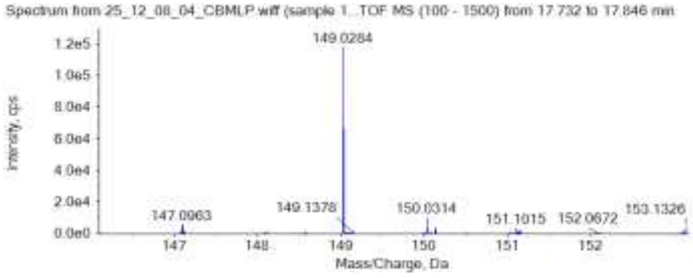

279.2382 / 17.85 (Mass/FragMass/RT/Isotope/Library/Formula/Ion Ratio)

**Retention Time:** 17.82 minutes	**Exp RT:** 17.85 minutes
**Precursor m/z :** 279.2382	**Analyte Name:**
**Fit (%)** 76.9% **RFit (%)** 61.5%	279.2382 / 17.85

Collision Energy = 35 ± 15 eV

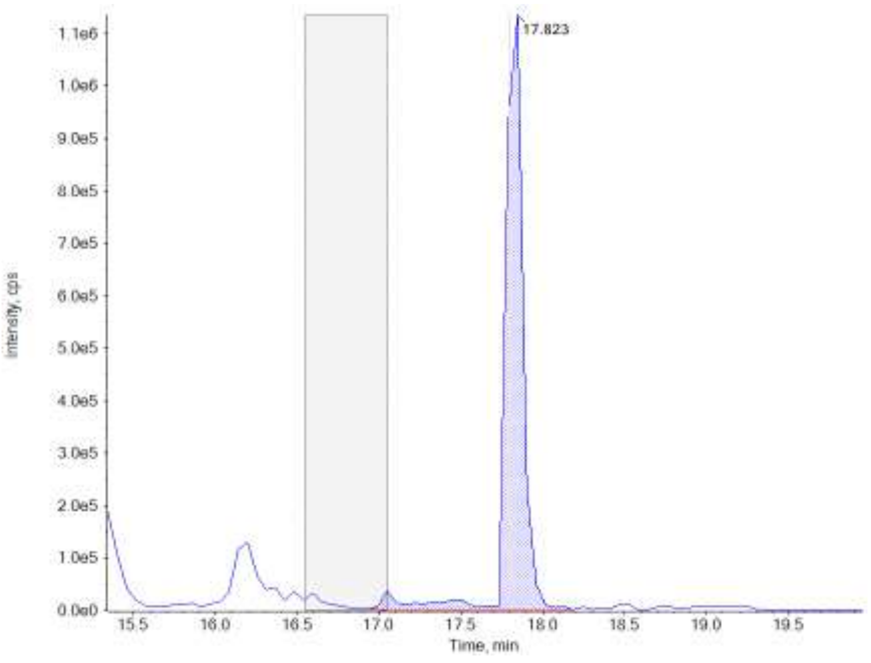

Acquired / Library MSMS

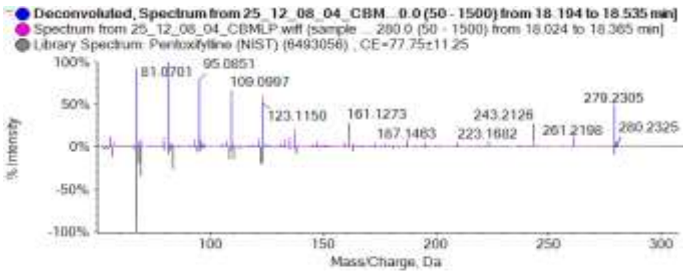

Acquired / Theoretical MS

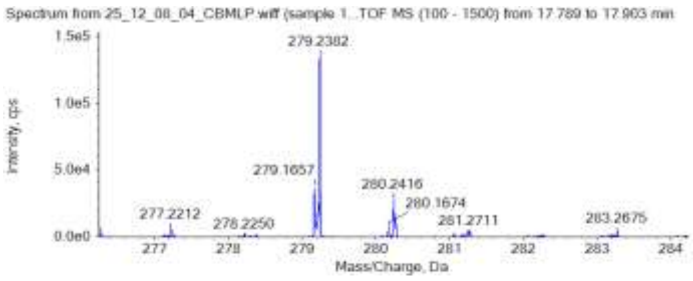

583.4191 / 18.19 (Mass/FragMass/RT/Isotope/Library/Formula/Ion Ratio)

Retention Time: 18.17 minutes
Precursor m/z : 583.4191
Fit (%) 99.1% **RFit (%)** 93.9%

Exp RT: 18.19 minutes
Analyte Name:
583.4191 / 18.19

Collision Energy = 35 ± 15 eV

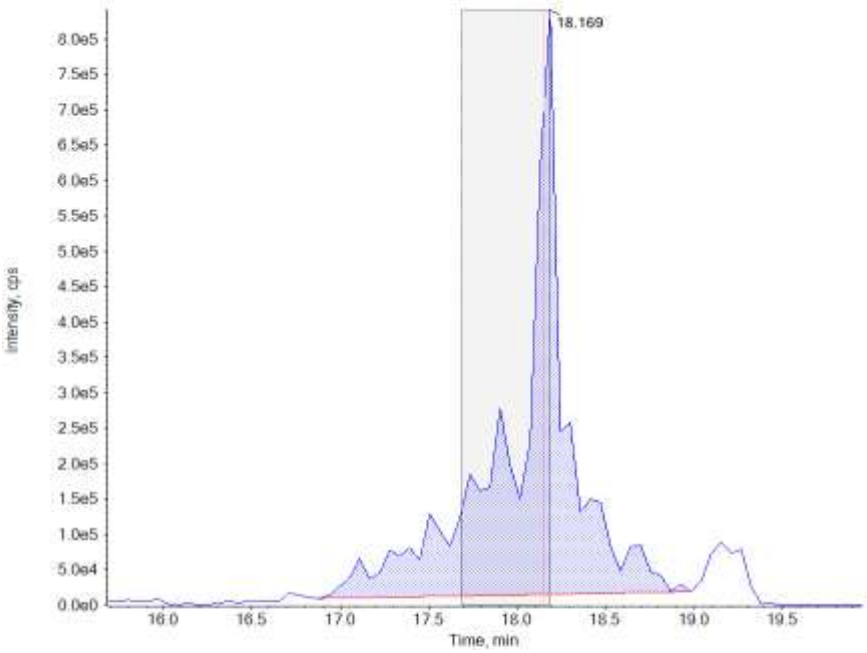

Acquired / Library MSMS

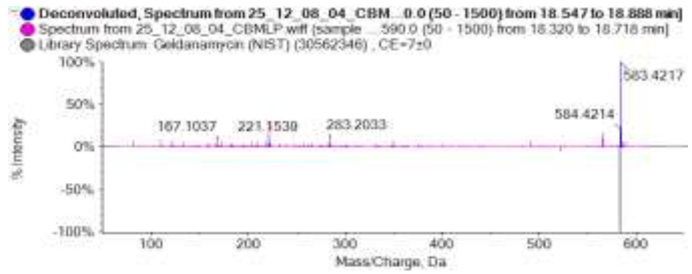

Acquired / Theoretical MS

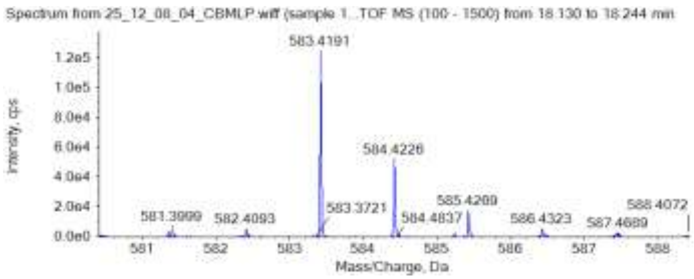

391.3414 / 18.36 (Mass/FragMass/RT/Isotope/Library/Formula/Ion Ratio)

Retention Time: 18.37 minutes
Precursor m/z : 391.3414
Fit (%) 100.0% **RFit (%)** 100.0%

Exp RT: 18.36 minutes
Analyte Name:
391.3414 / 18.36

Collision Energy = 35 ± 15 eV

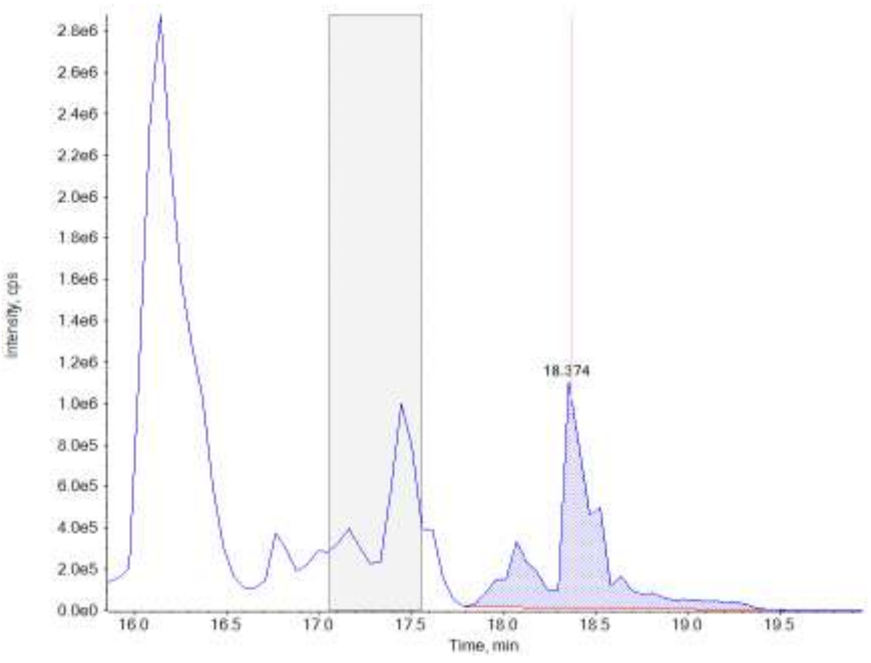

Acquired / Library MSMS

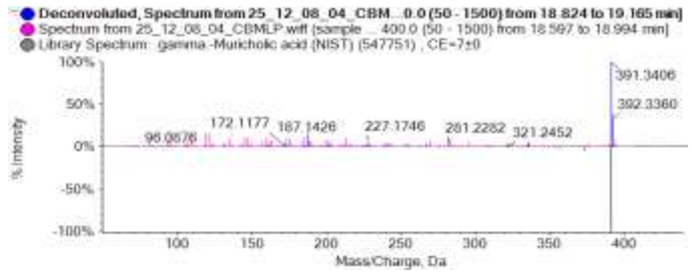

Acquired / Theoretical MS

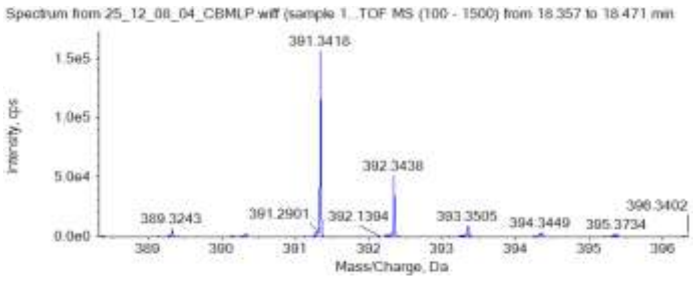

419.3390 / 18.36 (Mass/FragMass/RT/Isotope/Library/Formula/Ion Ratio)

Retention Time: 18.38 minutes
Precursor m/z : 419.3390
Fit (%) 68.3% **RFit (%)** 84.3%

Exp RT: 18.36 minutes
Analyte Name:
419.3390 / 18.36

Collision Energy = 35 ± 15 eV

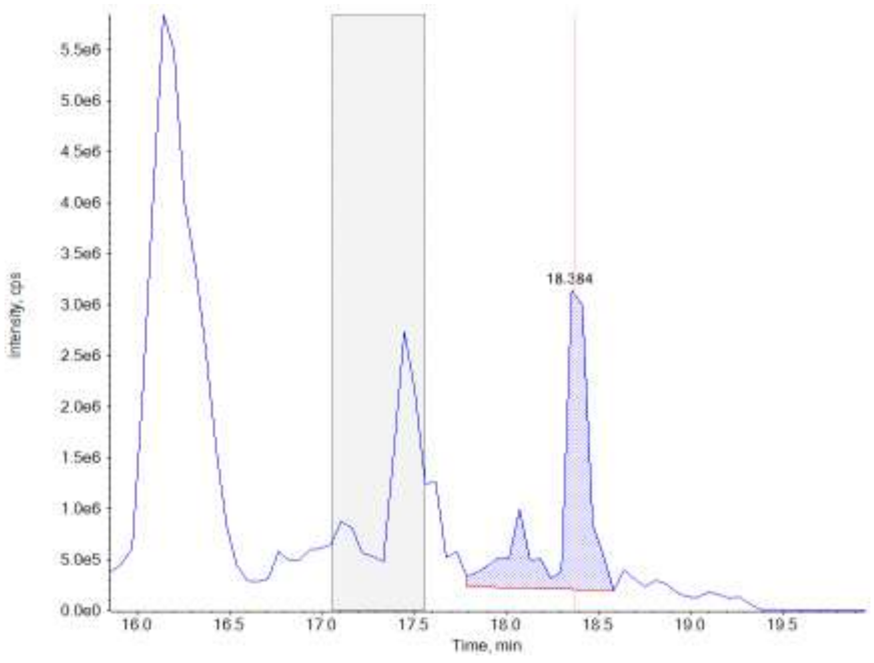

Acquired / Library MSMS

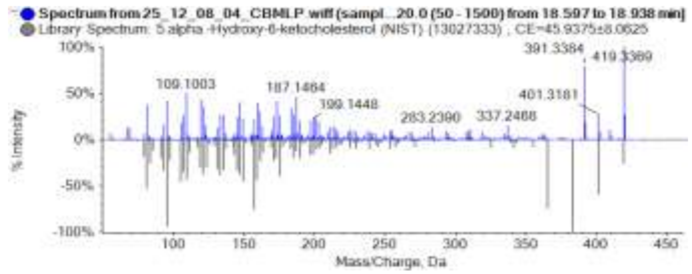

Acquired / Theoretical MS

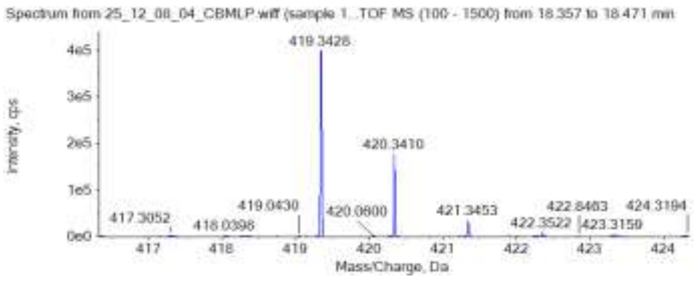

609.2838 / 18.41 (Mass/FragMass/RT/Isotope/Library/Formula/Ion Ratio)

Retention Time: 18.36 minutes
Precursor m/z : 609.2838
Fit (%) 99.1% **RFit (%)** 100.0%

Exp RT: 18.41 minutes
Analyte Name:
609.2838 / 18.41

Collision Energy = 35 ± 15 eV

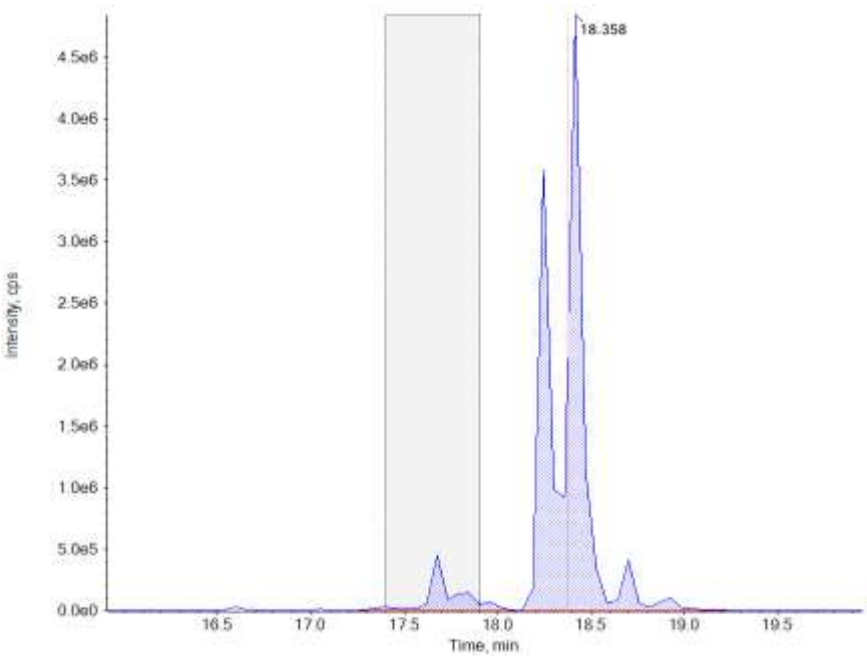

Acquired / Library MSMS

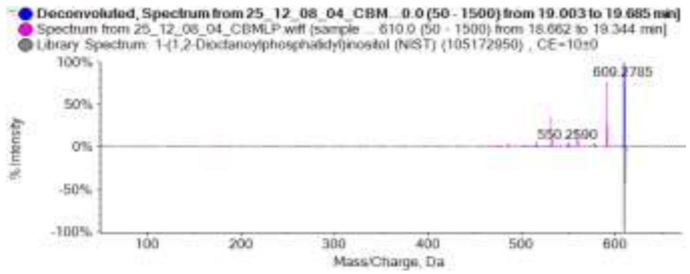

Acquired / Theoretical MS

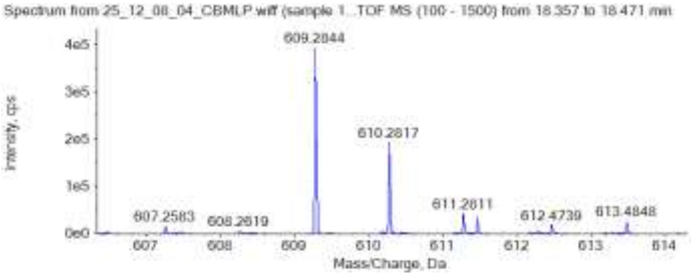

439.3632 / 18.53 (Mass/FragMass/RT/Isotope/Library/Formula/Ion Ratio)

Retention Time: 18.53 minutes
Precursor m/z : 439.3632
Fit (%) 97.7% **RFit (%)** 98.2%

Exp RT: 18.53 minutes
Analyte Name:
439.3632 / 18.53

Collision Energy = 35 ± 15 eV

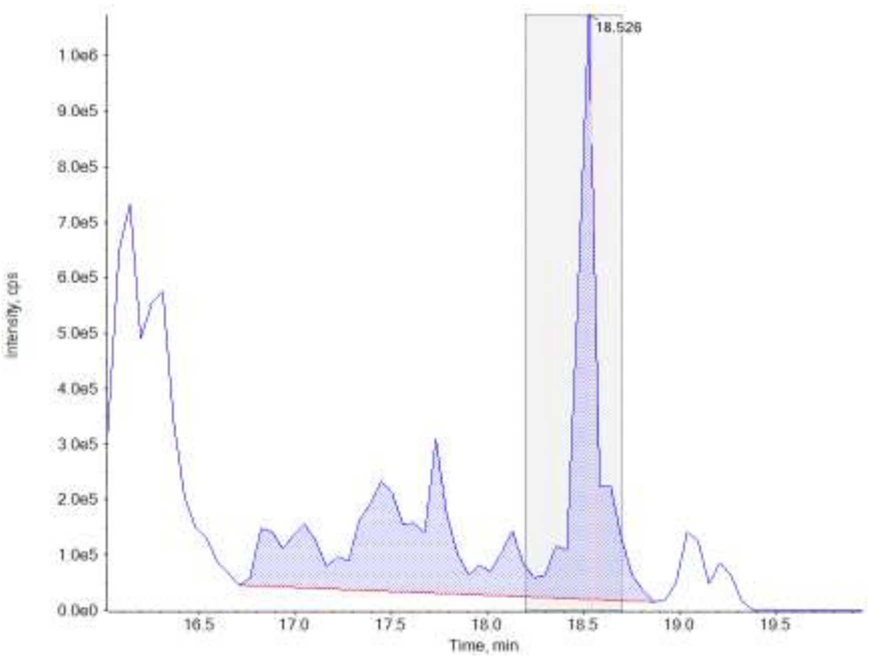

Acquired / Library MSMS

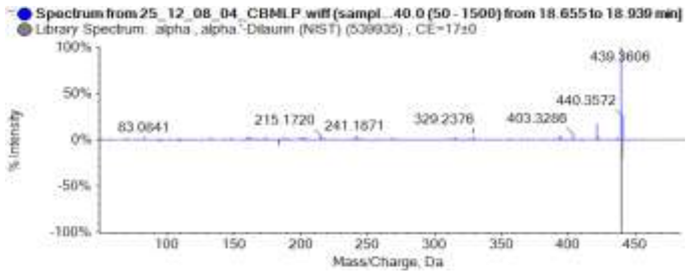

Acquired / Theoretical MS

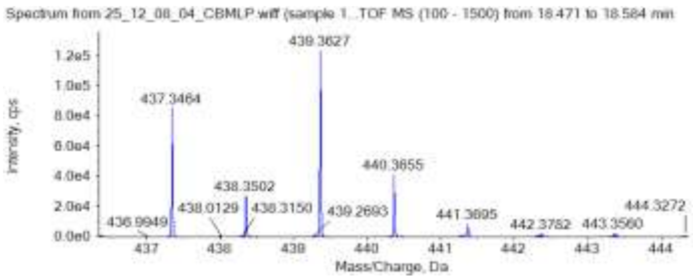

313.2839 / 18.58 (Mass/FragMass/RT/Isotope/Library/Formula/Ion Ratio)

Retention Time: 18.54 minutes
Precursor m/z : 313.2839
Fit (%) 70.7% **RFit (%)** 96.3%

Exp RT: 18.58 minutes
Analyte Name:
313.2839 / 18.58

Collision Energy = 35 ± 15 eV

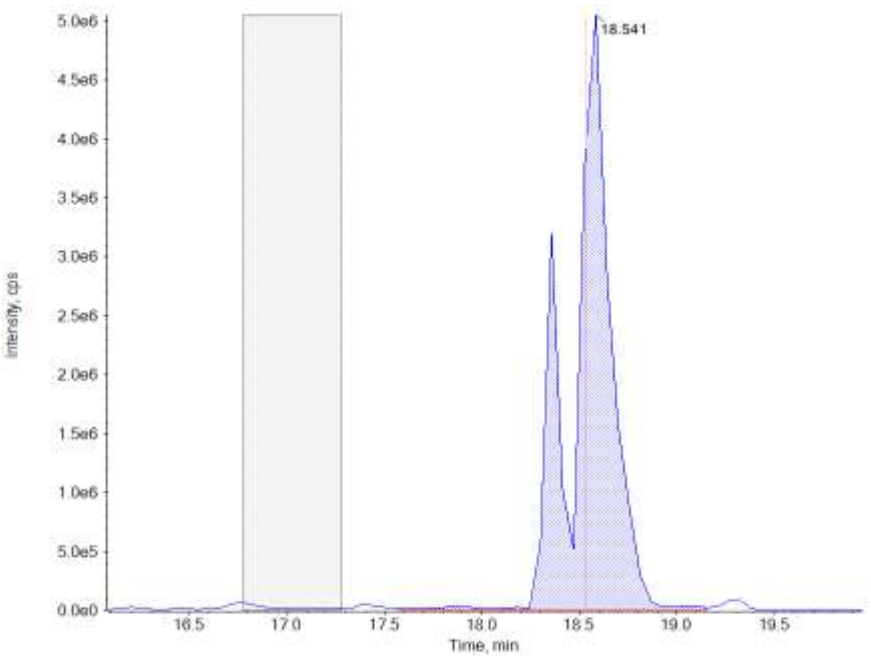

Acquired / Library MSMS

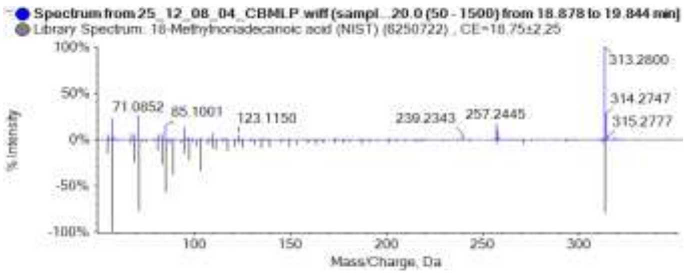

Acquired / Theoretical MS

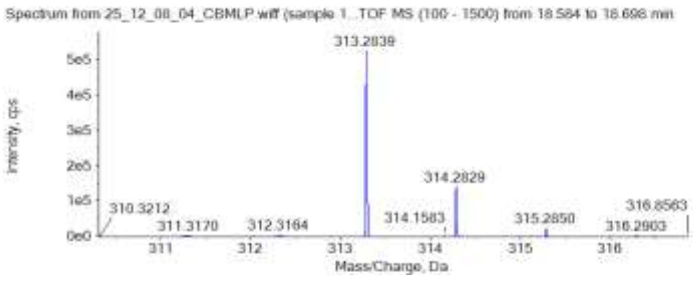

331.2919 / 18.58 (Mass/FragMass/RT/Isotope/Library/Formula/Ion Ratio)

Retention Time: 18.57 minutes
Precursor m/z : 331.2919
Fit (%) 98.9% **RFit (%)** 96.4%

Exp RT: 18.58 minutes
Analyte Name:
331.2919 / 18.58

Collision Energy = 35 ± 15 eV

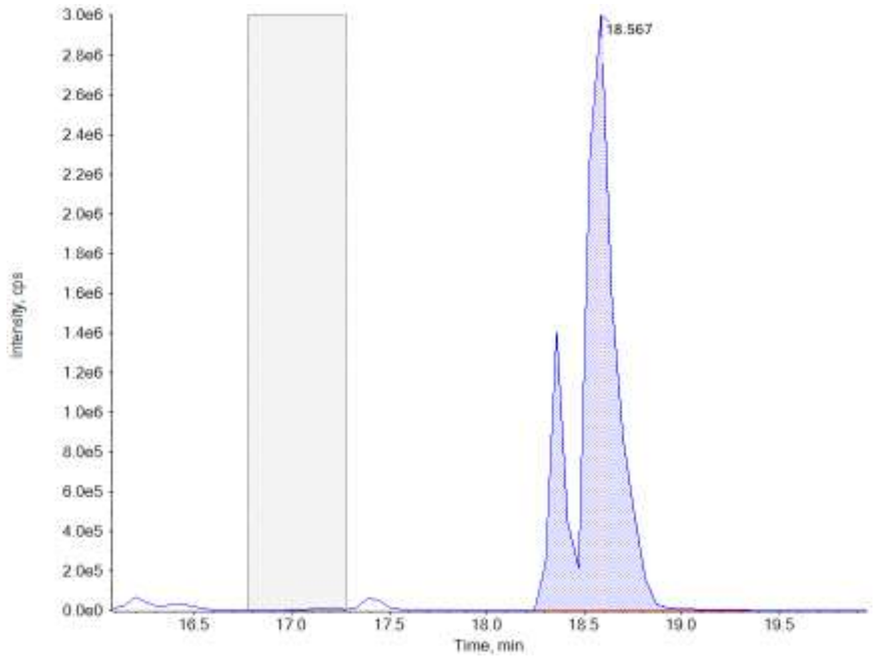

Acquired / Library MSMS

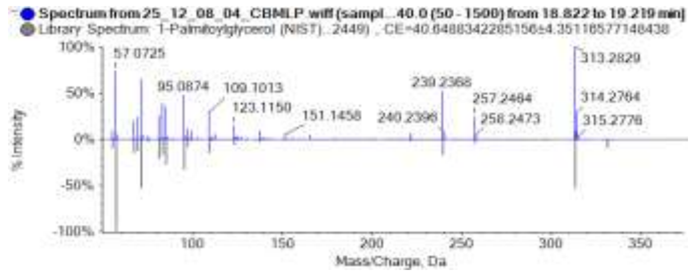

Acquired / Theoretical MS

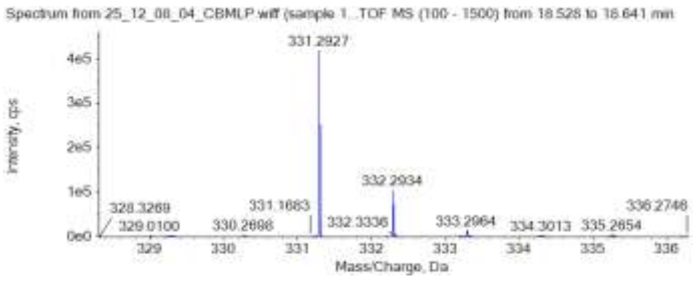

593.2952 / 18.64 (Mass/FragMass/RT/Isotope/Library/Formula/Ion Ratio)

**Retention Time:** 18.63 minutes	**Exp RT:** 18.64 minutes
**Precursor m/z :** 593.2952	**Analyte Name:**
**Fit (%)** 71.5% **RFit (%)** 94.7%	593.2952 / 18.64

Collision Energy = 35 ± 15 eV

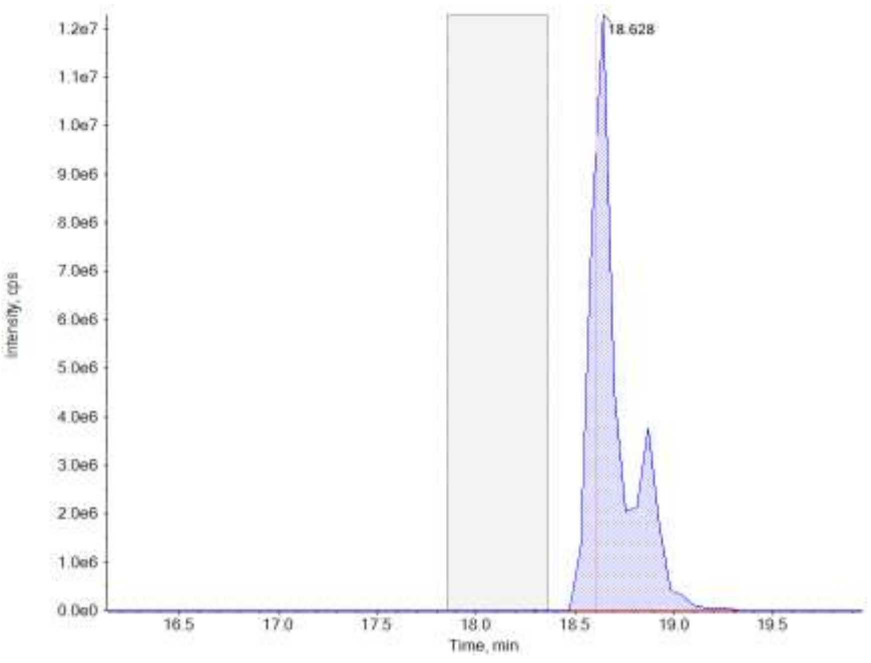

Acquired / Library MSMS

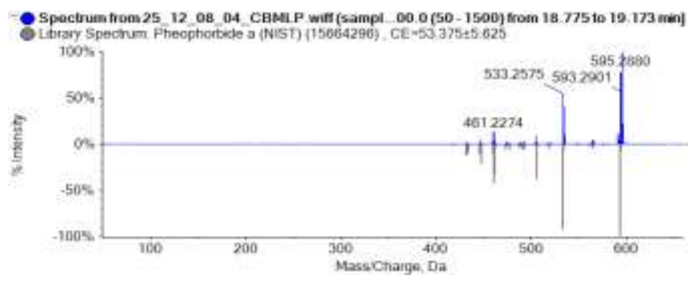

Acquired / Theoretical MS

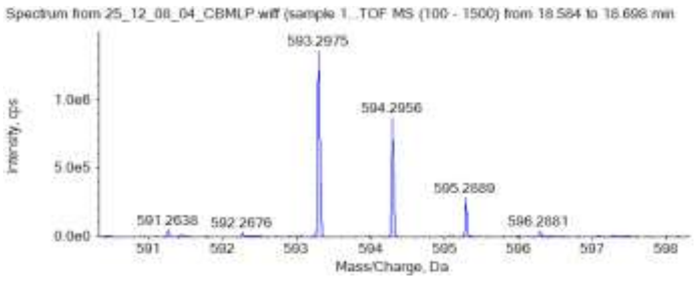

277.2222 / 19.10 (Mass/FragMass/RT/Isotope/Library/Formula/Ion Ratio)

**Retention Time:** 19.08 minutes	**Exp RT:** 19.10 minutes
**Precursor m/z :** 277.2222	**Analyte Name:**
**Fit (%)** 97.1% **RFit (%)** 96.8%	277.2222 / 19.10

	**Collision Energy = 35 ± 15 eV**

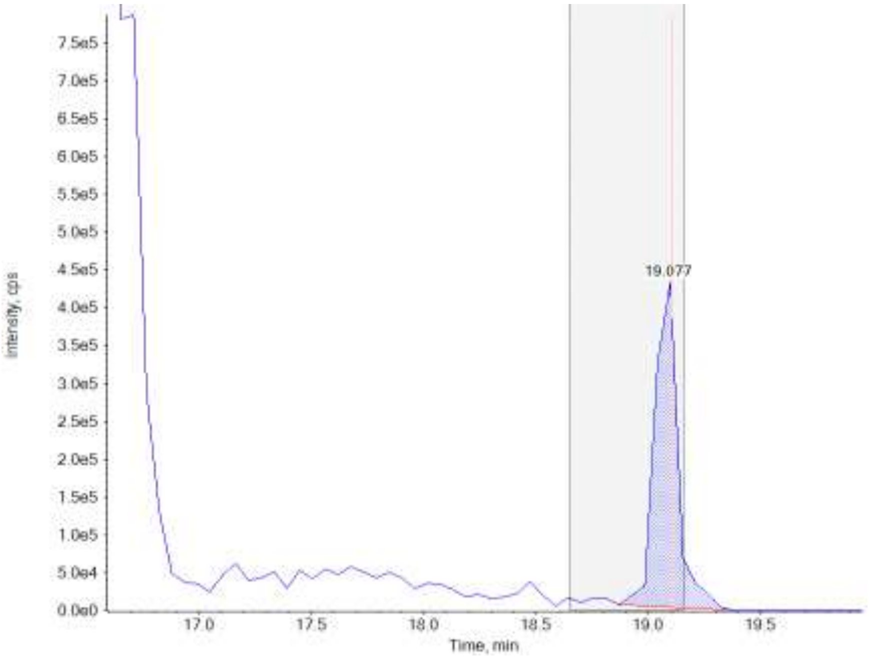

Acquired / Library MSMS

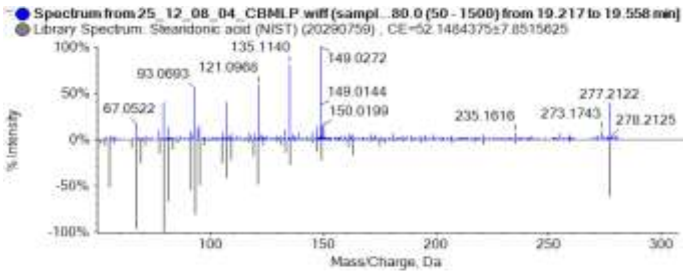

Acquired / Theoretical MS

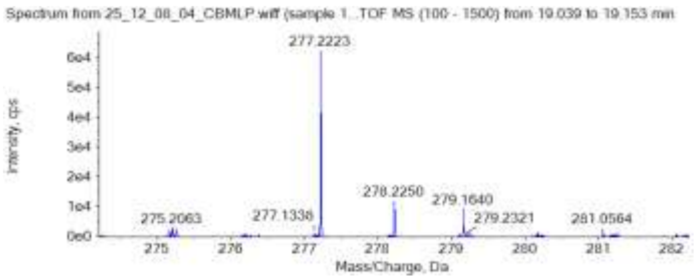

309.2850 / 19.10 (Mass/FragMass/RT/Isotope/Library/Formula/Ion Ratio)

Retention Time: 19.12 minutes
Precursor m/z : 309.2850
Fit (%) 87.8% **RFit (%)** 91.6%

Exp RT: 19.10 minutes
Analyte Name:
309.2850 / 19.10

Collision Energy = 35 ± 15 eV

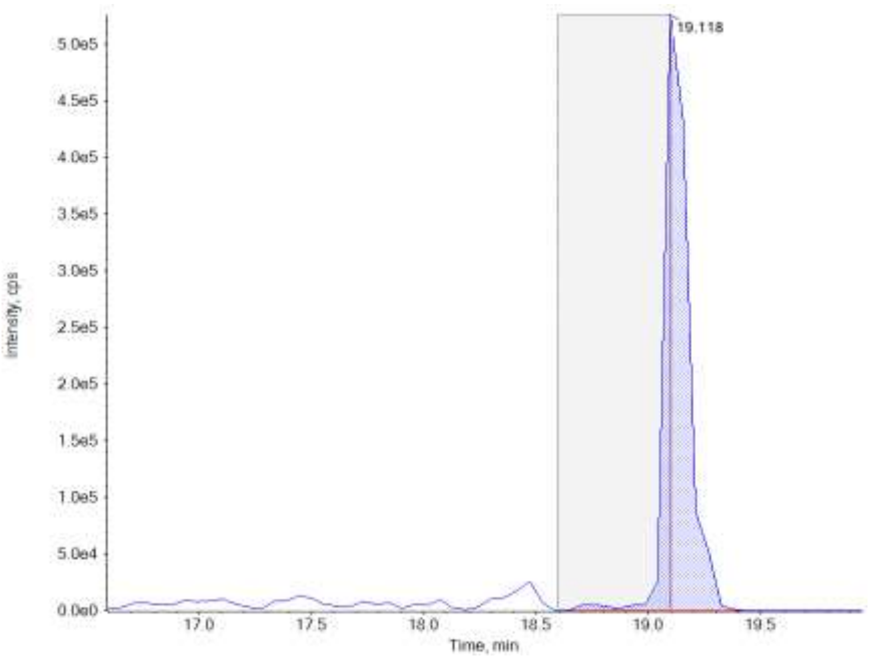

Acquired / Library MSMS

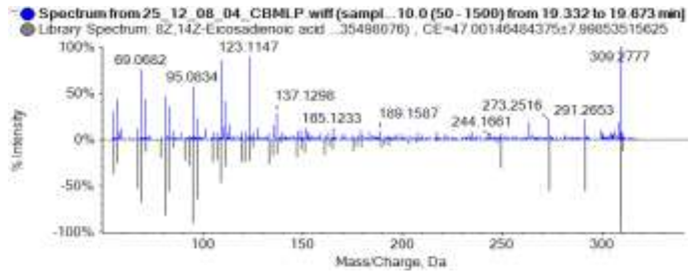

Acquired / Theoretical MS

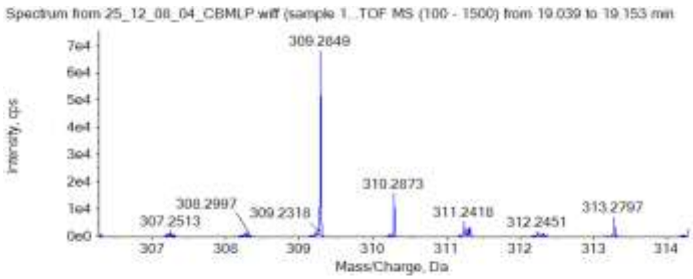

551.4299 / 19.27 (Mass/FragMass/RT/Isotope/Library/Formula/Ion Ratio)

Retention Time: 19.23 minutes
Precursor m/z : 551.4299
Fit (%) 78.2% **RFit (%)** 99.7%

Exp RT: 19.27 minutes
Analyte Name:
551.4299 / 19.27

Collision Energy = 35 ± 15 eV

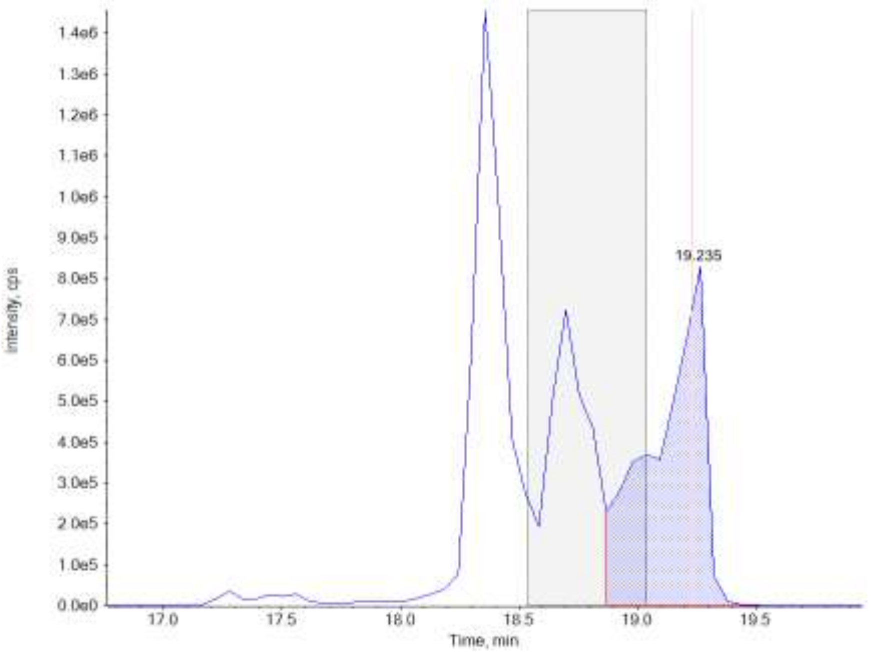

Acquired / Library MSMS

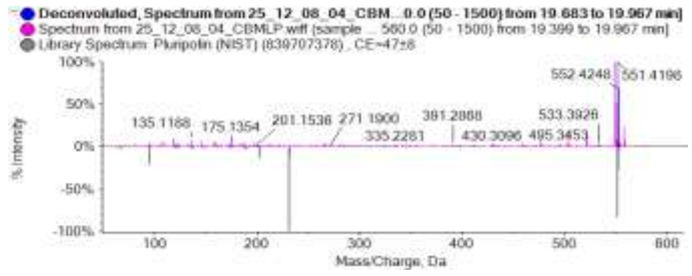

Acquired / Theoretical MS

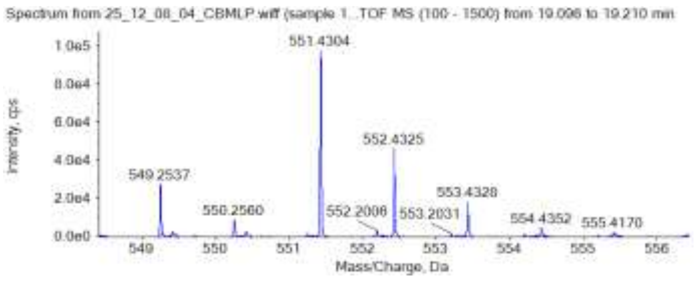

Created with SCIEX OS 3.3.0
Printed:12/9/2025 2:04:37 PM

601.4284 / 19.27 (Mass/FragMass/RT/Isotope/Library/Formula/Ion Ratio)

Retention Time: 19.22 minutes
Precursor m/z : 601.4284
Fit (%) 99.0% **RFit (%)** 83.2%

Exp RT: 19.27 minutes
Analyte Name:
601.4284 / 19.27

Collision Energy = 35 ± 15 eV

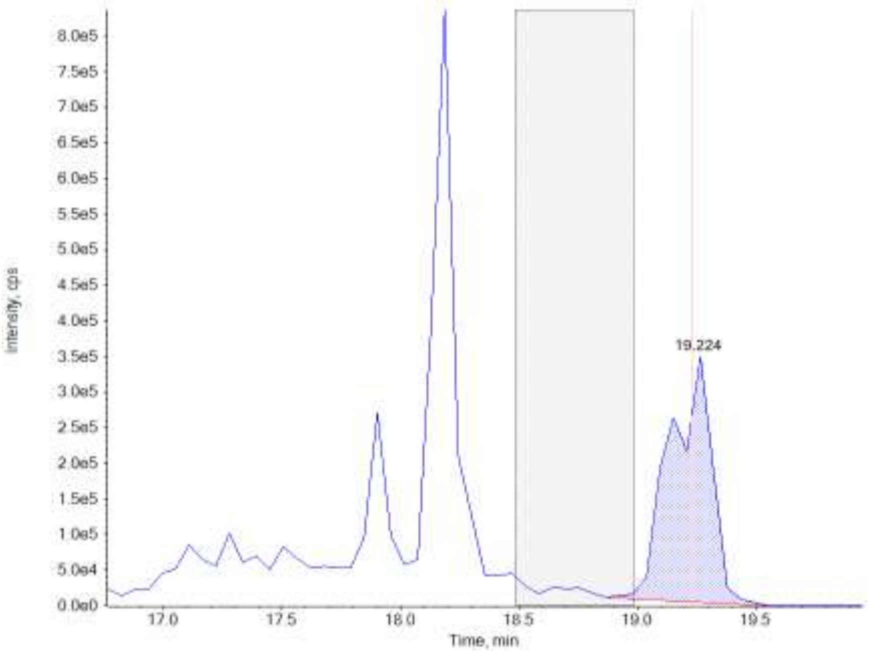

Acquired / Library MSMS

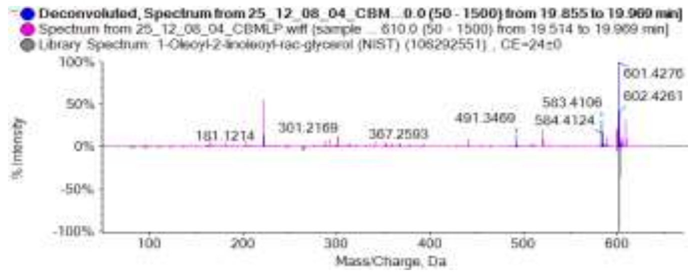

Acquired / Theoretical MS

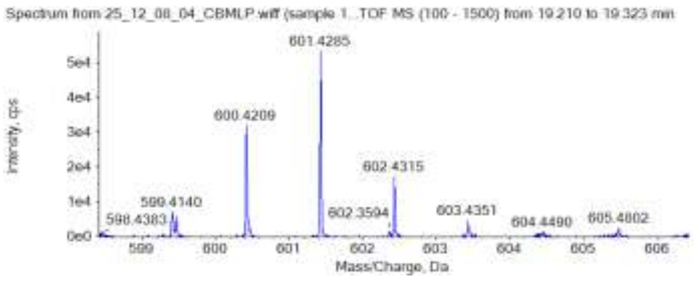

535.2757 / 19.32 (Mass/FragMass/RT/Isotope/Library/Formula/Ion Ratio)

**Retention Time:** 19.32 minutes	**Exp RT:** 19.32 minutes
**Precursor m/z :** 535.2757	**Analyte Name:**
**Fit (%)** 71.2% **RFit (%)** 100.0%	535.2757 / 19.32

Collision Energy = 35 ± 15 eV

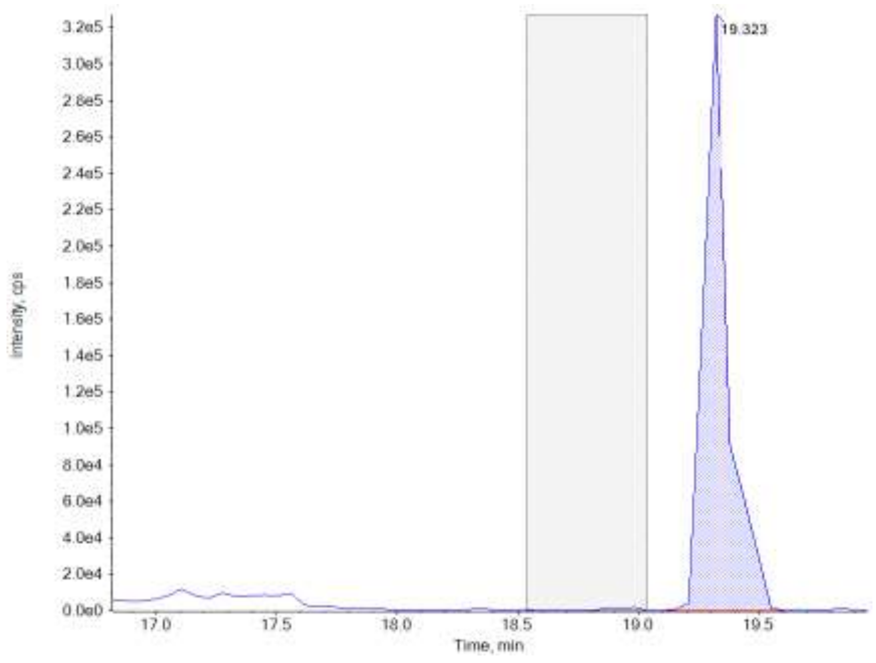

Acquired / Library MSMS

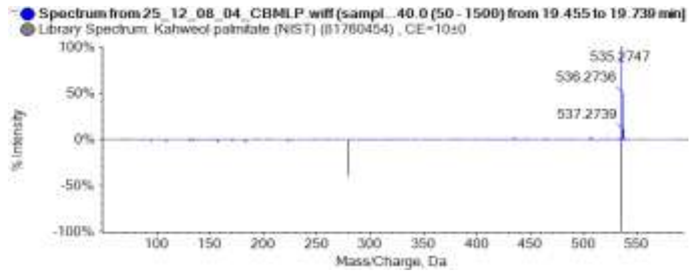

Acquired / Theoretical MS

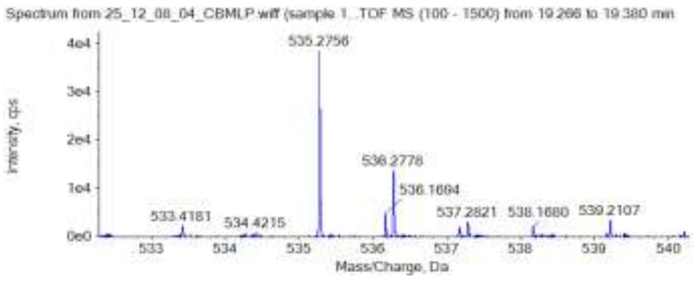

223.0699 / 19.78

(Mass/FragMass/RT/Isotope/Library/Formula/Ion Ratio)

Retention Time: 19.62 minutes
Precursor m/z : 223.0699
Fit (%) 93.6% **RFit (%)** 77.5%

Exp RT: 19.78 minutes
Analyte Name:
223.0699 / 19.78

Collision Energy = 35 ± 15 eV

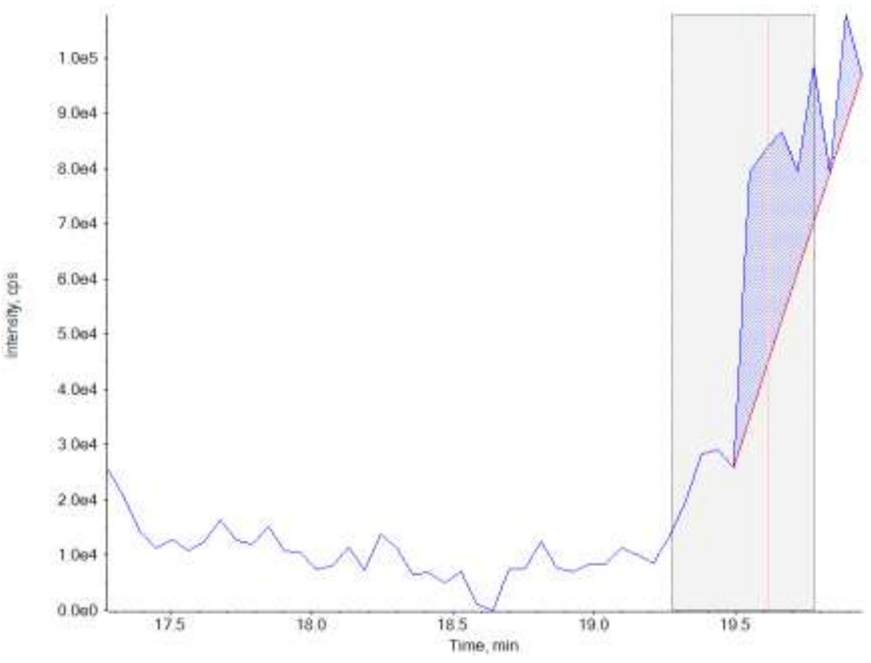

Acquired / Library MSMS

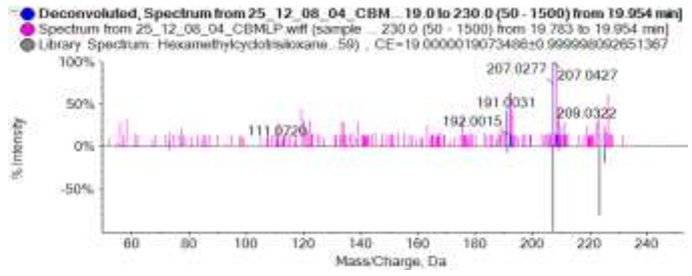

Acquired / Theoretical MS

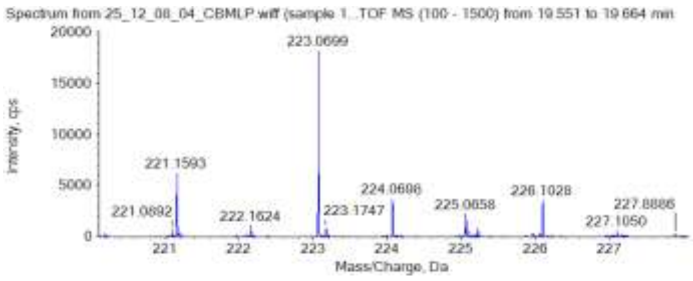

Supplement: Supplementary file 1 [file ijms-27-04945-s001.zip › 2.IJMS-4262115 Supplementary Data BMLE-positive mode.pdf]
