# Supplementary material for: Box–Behnken Design Optimization of High-Pressure Processed Bitter Melon (Momordica charantia) Leaf Extract Enhancing Phytochemicals, Anticancer, and Anti-Inflammatory Activities
Source: Int J Mol Sci. 2026 May 29;27(11):4945. doi: 10.3390/ijms27114945 (PMC13256669; doi:10.3390/ijms27114945)

# O-BMLE (negative mode)

## 25_11_20_08_OBML_N.wiff - OBML_N

| Data File | 25_11_20_08_OBML_N.wiff | Result Table | OBML-N-CE40 |
|---|---|---|---|
| Acquisition Date | 2025-11-20T15:30:12 | Algorithm Used | MQ4 |
| Acquisition Method | Neg_DIA_20min_250723_001.dam | Instrument Name | TripleTOF 6600 |
| Project | | Processing Method | |

## Extracted Ion Chromatogram

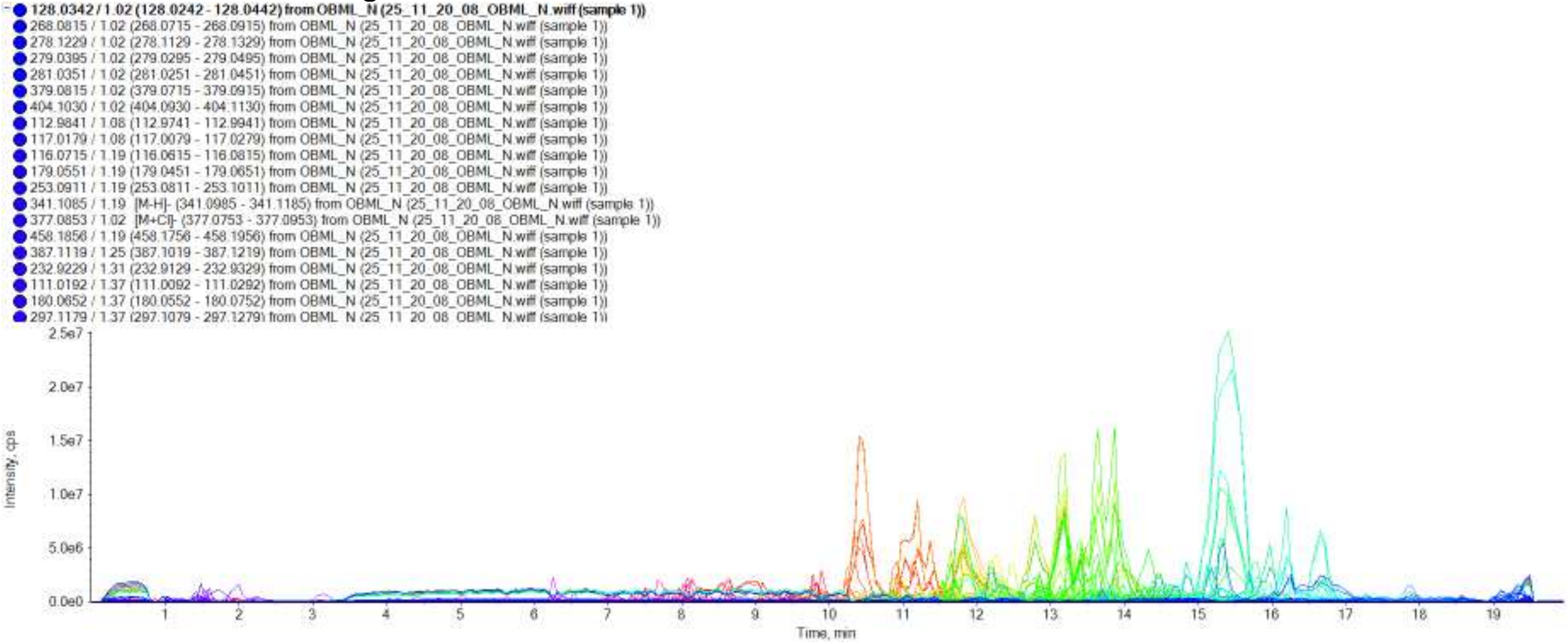

# Summary

| # | Analyte Peak Name | Mass Error Confidence | Fragment Mass Error Confidence | RT Confidence | Isotope Confidence | Library Confidence | Formula Confidence | Ion Ratio Confidence | Sample Name |
|---|---|---|---|---|---|---|---|---|---|
| 1 | 128.0342 / 1.02 | ● | ● | ● | ● | ✓ | ● | ● | OBML_N |
| 10 | 116.0715 / 1.19 | ● | ● | ● | ● | 🔴 | ● | ● | OBML_N |
| 11 | 179.0551 / 1.19 | ● | ● | ● | ● | ✓ | ● | ● | OBML_N |
| 13 | 341.1085 / 1.19 [M-H]- | ● | ● | ● | ● | ▲ | ● | ● | OBML_N |
| 18 | 111.0192 / 1.37 | ● | ● | ● | ● | 🔴 | ● | ● | OBML_N |
| 19 | 180.0652 / 1.37 | ● | ● | ● | ● | ✓ | ● | ● | OBML_N |
| 24 | 243.0683 / 1.42 | ● | ● | ● | ● | ✓ | ● | ● | OBML_N |
| 27 | 130.0881 / 1.48 | ● | ● | ● | ● | ✓ | ● | ● | OBML_N |
| 29 | 202.1070 / 1.48 | ● | ● | ● | ● | 🔴 | ● | ● | OBML_N |
| 30 | 247.1173 / 1.48 [M-H]- | ● | ● | ● | ● | ✓ | ● | ● | OBML_N |
| 35 | 282.0858 / 1.54 | ● | ● | ● | ● | ✓ | ● | ● | OBML_N |
| 36 | 137.0240 / 1.59 | ● | ● | ● | ● | ✓ | ● | ● | OBML_N |
| 38 | 299.0821 / 1.59 | ● | ● | ● | ● | 🔴 | ● | ● | OBML_N |
| 42 | 134.0519 / 1.71 | ● | ● | ● | ● | ✓ | ● | ● | OBML_N |
| 43 | 337.1493 / 1.71 | ● | ● | ● | ● | 🔴 | ● | ● | OBML_N |
| 44 | 175.0981 / 1.82 | ● | ● | ● | ● | ▲ | ● | ● | OBML_N |
| 45 | 257.0773 / 1.82 | ● | ● | ● | ● | ▲ | ● | ● | OBML_N |
| 49 | 164.0765 / 1.99 | ● | ● | ● | ● | ✓ | ● | ● | OBML_N |
| 52 | 137.0280 / 2.27 | ● | ● | ● | ● | ✓ | ● | ● | OBML_N |
| 53 | 192.0656 / 2.33 | ● | ● | ● | ● | ✓ | ● | ● | OBML_N |

| | | | | | | | | | |
|---|---|---|---|---|---|---|---|---|---|
| 55 | 172.0968 / 2.39 | | | | | ✓ | | | OBML_N |
| 57 | 138.0552 / 2.44 | | | | | ✓ | | | OBML_N |
| 62 | 307.1381 / 2.67 | | | | | 🔴 | | | OBML_N |
| 64 | 206.0801 / 2.73 | | | | | ▲ | | | OBML_N |
| 65 | 327.1067 / 2.73 | | | | | ▲ | | | OBML_N |
| 66 | 153.0178 / 2.79 | | | | | ✓ | | | OBML_N |
| 69 | 167.0335 / 2.84 | | | | | ✓ | | | OBML_N |
| 70 | 245.0654 / 2.84 | | | | | 🔴 | | | OBML_N |
| 71 | 443.1905 / 2.96 | | | | | 🔴 | | | OBML_N |
| 73 | 203.0858 / 3.07 | | | | | ✓ | | | OBML_N |
| 74 | 399.1642 / 3.13 | | | | | ▲ | | | OBML_N |
| 77 | 387.1636 / 3.35 | | | | | 🔴 | | | OBML_N |
| 84 | 339.0710 / 4.83 | | | | | ✓ | | | OBML_N |
| 88 | 461.1476 / 5.00 | | | | | 🔴 | | | OBML_N |
| 89 | 387.1644 / 5.34 | | | | | ▲ | | | OBML_N |
| 93 | 433.2074 / 5.63 | | | | | 🔴 | | | OBML_N |
| 106 | 121.0287 / 6.20 | | | | | ✓ | | | OBML_N |
| 107 | 421.1642 / 6.25 [M+Cl]- | | | | | ▲ | | | OBML_N |
| 109 | 431.2011 / 6.25 | | | | | ✓ | | | OBML_N |
| 114 | 353.1259 / 6.37 | | | | | ✓ | | | OBML_N |
| 122 | 585.2392 / 6.65 | | | | | ▲ | | | OBML_N |
| 126 | 475.1597 / 6.71 | | | | | 🔴 | | | OBML_N |
| 127 | 193.0495 / 6.93 | | | | | ✓ | | | OBML_N |

| | | | | | | | | | |
|---|---|---|---|---|---|---|---|---|---|
| 128 | 247.1540 / 6.93 | | | | | ✓ | | | OBML_N |
| 133 | 551.2346 / 6.99 | | | | | ✓ | | | OBML_N |
| 134 | 159.1023 / 7.05 | | | | | ✓ | | | OBML_N |
| 136 | 433.2066 / 7.10 | | | | | ✓ | | | OBML_N |
| 144 | 361.2209 / 7.45 | | | | | ▲ | | | OBML_N |
| 149 | 463.0867 / 7.50 | | | | | ✓ | | | OBML_N |
| 151 | 271.1539 / 7.67 | | | | | ✓ | | | OBML_N |
| 157 | 187.0992 / 8.01 | | | | | ✓ | | | OBML_N |
| 161 | 447.0934 / 8.01 | | | | | ✓ | | | OBML_N |
| 162 | 229.1455 / 8.07 | | | | | ✓ | | | OBML_N |
| 167 | 431.1732 / 8.07 | | | | | ● | | | OBML_N |
| 169 | 144.0446 / 8.13 | | | | | ✓ | | | OBML_N |
| 173 | 231.1604 / 8.30 | | | | | ✓ | | | OBML_N |
| 176 | 173.1181 / 8.36 | | | | | ▲ | | | OBML_N |
| 177 | 171.1015 / 8.41 | | | | | ● | | | OBML_N |
| 178 | 381.1544 / 8.41 | | | | | ● | | | OBML_N |
| 181 | 229.1454 / 8.58 | | | | | ✓ | | | OBML_N |
| 183 | 281.0808 / 9.04 [M+K-2H]- | | | | | ✓ | | | OBML_N |
| 184 | 343.2146 / 8.64 | | | | | ● | | | OBML_N |
| 185 | 113.0603 / 8.70 [M-H]- | | | | | ▲ | | | OBML_N |
| 189 | 299.1844 / 8.81 | | | | | ● | | | OBML_N |
| 193 | 153.0902 / 9.09 | | | | | ▲ | | | OBML_N |
| 195 | 263.1306 / 9.09 | | | | | ✓ | | | OBML_N |

| | | | | | | | | | |
|---|---|---|---|---|---|---|---|---|---|
| 198 | 493.2285 / 9.26 [M+FA-H]- | | | | | | | | OBML_N |
| 199 | 447.2230 / 9.26 [M-H]- | | | | | | | | OBML_N |
| 202 | 329.1601 / 9.38 | | | | | | | | OBML_N |
| 204 | 383.1340 / 9.43 | | | | | | | | OBML_N |
| 206 | 147.0454 / 9.49 | | | | | | | | OBML_N |
| 207 | 185.1171 / 9.49 | | | | | | | | OBML_N |
| 209 | 449.2736 / 9.61 | | | | | | | | OBML_N |
| 210 | 683.4034 / 9.61 [M+FA-H]- | | | | | | | | OBML_N |
| 211 | 637.3950 / 9.61 [M-H]- | | | | | | | | OBML_N |
| 216 | 225.1144 / 9.83 | | | | | | | | OBML_N |
| 223 | 375.1480 / 9.89 | | | | | | | | OBML_N |
| 225 | 199.1336 / 9.95 | | | | | | | | OBML_N |
| 226 | 341.1949 / 10.00 | | | | | | | | OBML_N |
| 227 | 593.2997 / 10.06 | | | | | | | | OBML_N |
| 235 | 609.2699 / 10.23 | | | | | | | | OBML_N |
| 243 | 645.3644 / 10.57 [M-H2O-H]- | | | | | | | | OBML_N |
| 245 | 201.1476 / 10.69 | | | | | | | | OBML_N |
| 252 | 679.4110 / 10.86 | | | | | | | | OBML_N |
| 253 | 227.1280 / 10.91 | | | | | | | | OBML_N |
| 255 | 329.2406 / 10.91 | | | | | | | | OBML_N |
| 259 | 707.4025 / 11.03 | | | | | | | | OBML_N |
| 266 | 695.4015 / 11.20 | | | | | | | | OBML_N |
| 267 | 287.2215 / 11.25 | | | | | | | | OBML_N |

| | | | | | | | | | |
|---|---|---|---|---|---|---|---|---|---|
| 272 | 195.1383 / 11.42  [M-H2O-H]- | | | | | | | | OBML_N |
| 273 | 213.1487 / 11.08  [M-H]- | | | | | | | | OBML_N |
| 274 | 239.1280 / 11.42 | | | | | | | | OBML_N |
| 278 | 795.4549 / 11.54 | | | | | | | | OBML_N |
| 284 | 669.3803 / 11.65 | | | | | | | | OBML_N |
| 289 | 309.2073 / 11.82 | | | | | | | | OBML_N |
| 319 | 718.4561 / 12.56 | | | | | | | | OBML_N |
| 320 | 237.1495 / 12.62 | | | | | | | | OBML_N |
| 323 | 669.3805 / 12.73 | | | | | | | | OBML_N |
| 326 | 679.4268 / 12.79 | | | | | | | | OBML_N |
| 346 | 601.3746 / 13.13 | | | | | | | | OBML_N |
| 349 | 571.2930 / 13.30 | | | | | | | | OBML_N |
| 351 | 253.1793 / 13.36 | | | | | | | | OBML_N |
| 359 | 665.4288 / 13.47 | | | | | | | | OBML_N |
| 367 | 667.3590 / 13.64 | | | | | | | | OBML_N |
| 368 | 941.6015 / 13.64 | | | | | | | | OBML_N |
| 370 | 209.1173 / 13.70 | | | | | | | | OBML_N |
| 371 | 289.1787 / 13.70 | | | | | | | | OBML_N |
| 373 | 197.1163 / 13.81 | | | | | | | | OBML_N |
| 377 | 665.4274 / 13.98  [M+FA-H]- | | | | | | | | OBML_N |
| 380 | 483.2779 / 13.92 | | | | | | | | OBML_N |
| 381 | 679.4117 / 13.92 | | | | | | | | OBML_N |
| 389 | 305.1736 / 14.15 | | | | | | | | OBML_N |

| | | | | | | | | | |
|---|---|---|---|---|---|---|---|---|---|
| 390 | 695.4024 / 14.21 | | | | | | | | OBML_N |
| 394 | 509.2960 / 14.32 | | | | | | | | OBML_N |
| 396 | 279.1946 / 14.38 | | | | | | | | OBML_N |
| 405 | 617.3669 / 14.49 | | | | | | | | OBML_N |
| 408 | 311.2227 / 14.55 | | | | | | | | OBML_N |
| 409 | 831.4924 / 14.55 | | | | | | | | OBML_N |
| 412 | 681.3411 / 14.66 [M+Cl]- | | | | | | | | OBML_N |
| 415 | 289.1787 / 14.72 | | | | | | | | OBML_N |
| 420 | 291.2008 / 14.83 | | | | | | | | OBML_N |
| 427 | 583.3172 / 15.00 | | | | | | | | OBML_N |
| 430 | 365.2686 / 15.06 | | | | | | | | OBML_N |
| 432 | 517.3580 / 15.12 | | | | | | | | OBML_N |
| 435 | 235.1686 / 15.17 | | | | | | | | OBML_N |
| 436 | 275.2017 / 15.17 | | | | | | | | OBML_N |
| 438 | 911.6336 / 15.29 | | | | | | | | OBML_N |
| 439 | 293.2260 / 15.35 | | | | | | | | OBML_N |
| 443 | 511.3075 / 15.40 | | | | | | | | OBML_N |
| 447 | 271.2257 / 15.74 | | | | | | | | OBML_N |
| 453 | 295.2253 / 15.86 | | | | | | | | OBML_N |
| 456 | 291.2014 / 15.97 | | | | | | | | OBML_N |
| 464 | 517.3580 / 16.14 | | | | | | | | OBML_N |
| 465 | 699.3827 / 16.14 | | | | | | | | OBML_N |
| 467 | 295.2390 / 16.20 | | | | | | | | OBML_N |

| | | | | | | | | | |
|---|---|---|---|---|---|---|---|---|---|
| 470 | 869.4814 / 16.42 [M+Na-2H]- | | | | | | | | OBML_N |
| 471 | 337.2362 / 16.31 | | | | | | | | OBML_N |
| 472 | 519.3654 / 16.31 | | | | | | | | OBML_N |
| 474 | 295.2261 / 16.37 | | | | | | | | OBML_N |
| 476 | 849.5047 / 16.37 | | | | | | | | OBML_N |
| 479 | 243.1947 / 16.54 | | | | | | | | OBML_N |
| 480 | 573.3767 / 16.54 | | | | | | | | OBML_N |
| 482 | 293.2205 / 16.65 | | | | | | | | OBML_N |
| 486 | 585.3782 / 16.71 | | | | | | | | OBML_N |
| 497 | 321.2448 / 16.88 | | | | | | | | OBML_N |
| 501 | 295.2262 / 17.05 | | | | | | | | OBML_N |
| 502 | 362.2682 / 17.05 | | | | | | | | OBML_N |
| 507 | 335.2210 / 17.16 [M+AcO-H]- | | | | | | | | OBML_N |
| 511 | 249.1858 / 17.16 | | | | | | | | OBML_N |
| 516 | 289.1808 / 17.28 | | | | | | | | OBML_N |
| 517 | 365.2725 / 17.28 | | | | | | | | OBML_N |
| 518 | 787.4677 / 17.28 | | | | | | | | OBML_N |
| 522 | 269.2462 / 17.33 | | | | | | | | OBML_N |
| 525 | 813.4836 / 17.33 | | | | | | | | OBML_N |
| 531 | 277.2156 / 17.45 | | | | | | | | OBML_N |
| 532 | 283.2638 / 17.45 | | | | | | | | OBML_N |
| 535 | 263.2003 / 17.50 | | | | | | | | OBML_N |
| 537 | 831.5072 / 17.50 | | | | | | | | OBML_N |

| | | | | | | | | | |
|---|---|---|---|---|---|---|---|---|---|
| 539 | 251.2000 / 17.62 | | | | | | | | OBML_N |
| 545 | 307.1899 / 17.79 [M-H]- | | | | | | | | OBML_N |
| 548 | 277.2279 / 17.85 | | | | | | | | OBML_N |
| 550 | 590.4449 / 17.85 | | | | | | | | OBML_N |
| 554 | 831.4934 / 18.02 | | | | | | | | OBML_N |
| 556 | 253.2232 / 18.13 [M-H2O-H]- | | | | | | | | OBML_N |
| 557 | 271.2331 / 17.96 [M-H]- | | | | | | | | OBML_N |
| 560 | 279.2401 / 18.30 | | | | | | | | OBML_N |
| 565 | 255.2390 / 18.58 | | | | | | | | OBML_N |
| 572 | 311.2933 / 18.81 | | | | | | | | OBML_N |
| 575 | 281.2487 / 19.15 | | | | | | | | OBML_N |
| 576 | 349.2733 / 19.15 | | | | | | | | OBML_N |
| 580 | 609.3920 / 19.27 | | | | | | | | OBML_N |
| 590 | 255.2372 / 19.38 | | | | | | | | OBML_N |

| # | Analyte Peak Name | Sample Type | Component Name | Component Type | Component Group Name | Expected RT | Area | Retention Time | Retention Time Delta (min) | Formula | Precursor Mass |
|---|---|---|---|---|---|---|---|---|---|---|---|
| 1 | 128.0342 / 1.02 | Unknown | 128.0342 / 1.02 | Quantifiers | [No data for] Component Group Name | 1.02 | 1.190e+06 | 1.02 | N/A | {129.04094} | 128.034 |
| 10 | 116.0715 / 1.19 | Unknown | 116.0715 / 1.19 | Quantifiers | [No data for] Component Group Name | 1.20 | 2.588e+06 | 1.24 | N/A | {117.07823} | 116.072 |
| 11 | 179.0551 / 1.19 | Unknown | 179.0551 / 1.19 | Quantifiers | [No data for] Component Group Name | 1.20 | 1.972e+06 | 1.17 | N/A | {180.06181} | 179.055 |
| 13 | 341.1085 / 1.19 [M-H]- | Unknown | 341.1085 / 1.19 [M-H]- | Quantifiers | 341.1085 / 1.19 | 1.20 | 3.315e+06 | 1.20 | N/A | {342.11522} | 341.108 |
| 18 | 111.0192 / 1.37 | Unknown | 111.0192 / 1.37 | Quantifiers | [No data for] Component Group Name | 1.37 | 7.004e+05 | 1.37 | N/A | {112.02588} | 111.019 |
| 19 | 180.0652 / 1.37 | Unknown | 180.0652 / 1.37 | Quantifiers | [No data for] Component Group | 1.37 | 9.805e+05 | 1.39 | N/A | {181.07188} | 180.065 |

| | | | | | | | | | | | |
|---|---|---|---|---|---|---|---|---|---|---|---|
| | | | | | Name | | | | | | |
| 24 | 243.0683 / 1.42 | Unknown | 243.0683 / 1.42 | Quantifiers | [No data for] Component Group Name | 1.42 | 1.015e+07 | 1.41 | N/A | {244.07508} | 243.068 |
| 27 | 130.0881 / 1.48 | Unknown | 130.0881 / 1.48 | Quantifiers | [No data for] Component Group Name | 1.48 | 8.096e+06 | 1.45 | N/A | {131.09487} | 130.088 |
| 29 | 202.1070 / 1.48 | Unknown | 202.1070 / 1.48 | Quantifiers | [No data for] Component Group Name | 1.48 | 1.816e+06 | 1.45 | N/A | {203.11368} | 202.107 |
| 30 | 247.1173 / 1.48 [M-H]- | Unknown | 247.1173 / 1.48 [M-H]- | Quantifiers | 247.1173 / 1.48 | 1.48 | 1.876e+06 | 1.48 | N/A | {248.12406} | 247.117 |
| 35 | 282.0858 / 1.54 | Unknown | 282.0858 / 1.54 | Quantifiers | [No data for] Component Group Name | 1.54 | 7.005e+06 | 1.54 | N/A | {283.09254} | 282.086 |
| 36 | 137.0240 / 1.59 | Unknown | 137.0240 / 1.59 | Quantifiers | [No data for] Component Group Name | 1.59 | 1.796e+06 | 1.59 | N/A | {138.03072} | 137.024 |
| 38 | 299.0821 / 1.59 | Unknown | 299.0821 / 1.59 | Quantifiers | [No data for] Component Group Name | 1.59 | 6.187e+06 | 1.59 | N/A | {300.08879} | 299.082 |
| 42 | 134.0519 / 1.71 | Unknown | 134.0519 / 1.71 | Quantifiers | [No data for] Component Group Name | 1.71 | 1.940e+07 | 1.71 | N/A | {135.05867} | 134.052 |
| 43 | 337.1493 / 1.71 | Unknown | 337.1493 / 1.71 | Quantifiers | [No data for] Component Group Name | 1.71 | 9.870e+05 | 1.69 | N/A | {338.15606} | 337.149 |
| 44 | 175.0981 / 1.82 | Unknown | 175.0981 / 1.82 | Quantifiers | [No data for] Component Group Name | 1.82 | 3.893e+06 | 1.83 | N/A | {176.10483} | 175.098 |
| 45 | 257.0773 / 1.82 | Unknown | 257.0773 / 1.82 | Quantifiers | [No data for] Component Group Name | 1.82 | 1.432e+06 | 1.82 | N/A | {258.08407} | 257.077 |
| 49 | 164.0765 / 1.99 | Unknown | 164.0765 / 1.99 | Quantifiers | [No data for] Component Group Name | 1.99 | 1.740e+07 | 1.96 | N/A | {165.08327} | 164.077 |
| 52 | 137.0280 / 2.27 | Unknown | 137.0280 / 2.27 | Quantifiers | [No data for] Component Group Name | 2.27 | 5.992e+06 | 2.23 | N/A | {138.03471} | 137.028 |
| 53 | 192.0656 / 2.33 | Unknown | 192.0656 / 2.33 | Quantifiers | [No data for] Component Group Name | 2.33 | 1.169e+06 | 2.32 | N/A | {193.07232} | 192.066 |
| 55 | 172.0968 / 2.39 | Unknown | 172.0968 / 2.39 | Quantifiers | [No data for] Component Group Name | 2.39 | 1.094e+06 | 2.44 | N/A | {173.10353} | 172.097 |
| 57 | 138.0552 / 2.44 | Unknown | 138.0552 / 2.44 | Quantifiers | [No data for] | 2.45 | 3.830e+05 | 2.44 | N/A | {139.06195} | 138.055 |

| | | | | | | | | | | | |
|---|---|---|---|---|---|---|---|---|---|---|---|
| | | | | | Component Group Name | | | | | | |
| 62 | 307.1381 / 2.67 | Unknown | 307.1381 / 2.67 | Quantifiers | [No data for] Component Group Name | 2.67 | 8.332e+05 | 2.69 | N/A | {308.14484} | 307.138 |
| 64 | 206.0801 / 2.73 | Unknown | 206.0801 / 2.73 | Quantifiers | [No data for] Component Group Name | 2.73 | 6.245e+05 | 2.72 | N/A | {207.08683} | 206.080 |
| 65 | 327.1067 / 2.73 | Unknown | 327.1067 / 2.73 | Quantifiers | [No data for] Component Group Name | 2.73 | 3.374e+05 | 2.73 | N/A | {328.11343} | 327.107 |
| 66 | 153.0178 / 2.79 | Unknown | 153.0178 / 2.79 | Quantifiers | [No data for] Component Group Name | 2.79 | 6.631e+05 | 2.80 | N/A | {154.02455} | 153.018 |
| 69 | 167.0335 / 2.84 | Unknown | 167.0335 / 2.84 | Quantifiers | [No data for] Component Group Name | 2.84 | 3.077e+05 | 2.81 | N/A | {168.04024} | 167.034 |
| 70 | 245.0654 / 2.84 | Unknown | 245.0654 / 2.84 | Quantifiers | [No data for] Component Group Name | 2.84 | 1.157e+06 | 2.85 | N/A | {246.07213} | 245.065 |
| 71 | 443.1905 / 2.96 | Unknown | 443.1905 / 2.96 | Quantifiers | [No data for] Component Group Name | 2.96 | 1.233e+06 | 2.93 | N/A | {444.19726} | 443.191 |
| 73 | 203.0858 / 3.07 | Unknown | 203.0858 / 3.07 | Quantifiers | [No data for] Component Group Name | 3.07 | 1.069e+07 | 3.13 | N/A | {204.09250} | 203.086 |
| 74 | 399.1642 / 3.13 | Unknown | 399.1642 / 3.13 | Quantifiers | [No data for] Component Group Name | 3.13 | 2.162e+06 | 3.10 | N/A | {400.17096} | 399.164 |
| 77 | 387.1636 / 3.35 | Unknown | 387.1636 / 3.35 | Quantifiers | [No data for] Component Group Name | 3.35 | 5.945e+05 | 3.36 | N/A | {388.17029} | 387.164 |
| 84 | 339.0710 / 4.83 | Unknown | 339.0710 / 4.83 | Quantifiers | [No data for] Component Group Name | 4.83 | 2.602e+06 | 4.84 | N/A | {340.07772} | 339.071 |
| 88 | 461.1476 / 5.00 | Unknown | 461.1476 / 5.00 | Quantifiers | [No data for] Component Group Name | 5.00 | 2.908e+06 | 5.03 | N/A | {462.15430} | 461.148 |
| 89 | 387.1644 / 5.34 | Unknown | 387.1644 / 5.34 | Quantifiers | [No data for] Component Group Name | 5.34 | 2.242e+06 | 5.33 | N/A | {388.17115} | 387.164 |
| 93 | 433.2074 / 5.63 | Unknown | 433.2074 / 5.63 | Quantifiers | [No data for] Component Group Name | 5.63 | 3.018e+06 | 5.63 | N/A | {434.21417} | 433.207 |
| 106 | 121.0287 / 6.20 | Unknown | 121.0287 / 6.20 | Quantifiers | [No data for] Component Group | 6.20 | 2.104e+06 | 6.22 | N/A | {122.03543} | 121.029 |

| | | | | | | | | | | | |
|---|---|---|---|---|---|---|---|---|---|---|---|
| | | | | | Name | | | | | | |
| 107 | 421.1642 / 6.25 [M+Cl]- | Unknown | 421.1642 / 6.25 [M+Cl]- | Quantifiers | 421.1642 / 6.25 | 6.25 | 4.626e+06 | 6.25 | N/A | {386.19423} | 421.164 |
| 109 | 431.2011 / 6.25 | Unknown | 431.2011 / 6.25 | Quantifiers | [No data for] Component Group Name | 6.25 | 1.519e+07 | 6.25 | N/A | {432.20787} | 431.201 |
| 114 | 353.1259 / 6.37 | Unknown | 353.1259 / 6.37 | Quantifiers | [No data for] Component Group Name | 6.37 | 1.374e+06 | 6.37 | N/A | {354.13263} | 353.126 |
| 122 | 585.2392 / 6.65 | Unknown | 585.2392 / 6.65 | Quantifiers | [No data for] Component Group Name | 6.65 | 1.143e+06 | 6.63 | N/A | {586.24595} | 585.239 |
| 126 | 475.1597 / 6.71 | Unknown | 475.1597 / 6.71 | Quantifiers | [No data for] Component Group Name | 6.71 | 2.888e+06 | 6.61 | N/A | {476.16641} | 475.160 |
| 127 | 193.0495 / 6.93 | Unknown | 193.0495 / 6.93 | Quantifiers | [No data for] Component Group Name | 6.93 | 2.491e+06 | 6.93 | N/A | {194.05625} | 193.050 |
| 128 | 247.1540 / 6.93 | Unknown | 247.1540 / 6.93 | Quantifiers | [No data for] Component Group Name | 6.93 | 3.231e+06 | 6.93 | N/A | {248.16076} | 247.154 |
| 133 | 551.2346 / 6.99 | Unknown | 551.2346 / 6.99 | Quantifiers | [No data for] Component Group Name | 6.99 | 4.762e+06 | 6.99 | N/A | {552.24128} | 551.235 |
| 134 | 159.1023 / 7.05 | Unknown | 159.1023 / 7.05 | Quantifiers | [No data for] Component Group Name | 7.05 | 4.411e+06 | 7.06 | N/A | {160.10907} | 159.102 |
| 136 | 433.2066 / 7.10 | Unknown | 433.2066 / 7.10 | Quantifiers | [No data for] Component Group Name | 7.11 | 8.813e+06 | 7.12 | N/A | {434.21329} | 433.207 |
| 144 | 361.2209 / 7.45 | Unknown | 361.2209 / 7.45 | Quantifiers | [No data for] Component Group Name | 7.45 | 4.108e+06 | 7.54 | N/A | {362.22761} | 361.221 |
| 149 | 463.0867 / 7.50 | Unknown | 463.0867 / 7.50 | Quantifiers | [No data for] Component Group Name | 7.50 | 2.810e+06 | 7.51 | N/A | {464.09338} | 463.087 |
| 151 | 271.1539 / 7.67 | Unknown | 271.1539 / 7.67 | Quantifiers | [No data for] Component Group Name | 7.67 | 9.443e+06 | 7.71 | N/A | {272.16059} | 271.154 |
| 157 | 187.0992 / 8.01 | Unknown | 187.0992 / 8.01 | Quantifiers | [No data for] Component Group Name | 8.01 | 1.007e+07 | 8.02 | N/A | {188.10589} | 187.099 |
| 161 | 447.0934 / 8.01 | Unknown | 447.0934 / 8.01 | Quantifiers | [No data for] Component Group Name | 8.01 | 4.698e+06 | 7.99 | N/A | {448.10009} | 447.093 |
| 162 | 229.1455 / 8.07 | Unknown | 229.1455 / 8.07 | Quantifiers | [No data for] | 8.07 | 1.717e+07 | 8.30 | N/A | {230.15221} | 229.145 |

| | | | | | | | | | | | |
|---|---|---|---|---|---|---|---|---|---|---|---|
| | | | | | Component Group Name | | | | | | |
| 167 | 431.1732 / 8.07 | Unknown | 431.1732 / 8.07 | Quantifiers | [No data for] Component Group Name | 8.07 | 2.195e+06 | 8.10 | N/A | {432.17992} | 431.173 |
| 169 | 144.0446 / 8.13 | Unknown | 144.0446 / 8.13 | Quantifiers | [No data for] Component Group Name | 8.13 | 3.642e+06 | 8.13 | N/A | {145.05138} | 144.045 |
| 173 | 231.1604 / 8.30 | Unknown | 231.1604 / 8.30 | Quantifiers | [No data for] Component Group Name | 8.30 | 6.988e+06 | 8.30 | N/A | {232.16717} | 231.160 |
| 176 | 173.1181 / 8.36 | Unknown | 173.1181 / 8.36 | Quantifiers | [No data for] Component Group Name | 8.36 | 4.267e+06 | 8.36 | N/A | {174.12484} | 173.118 |
| 177 | 171.1015 / 8.41 | Unknown | 171.1015 / 8.41 | Quantifiers | [No data for] Component Group Name | 8.41 | 2.238e+06 | 8.41 | N/A | {172.10825} | 171.102 |
| 178 | 381.1544 / 8.41 | Unknown | 381.1544 / 8.41 | Quantifiers | [No data for] Component Group Name | 8.41 | 2.611e+06 | 8.41 | N/A | {382.16113} | 381.154 |
| 181 | 229.1454 / 8.58 | Unknown | 229.1454 / 8.58 | Quantifiers | [No data for] Component Group Name | 8.58 | 1.717e+07 | 8.30 | N/A | {230.15215} | 229.145 |
| 183 | 281.0808 / 9.04 [M+K-2H]- | Unknown | 281.0808 / 9.04 [M+K-2H]- | Qualifiers | 243.1243 / 8.64 | 9.04 | 2.187e+06 | 9.04 | N/A | {244.13161} | 281.081 |
| 184 | 343.2146 / 8.64 | Unknown | 343.2146 / 8.64 | Quantifiers | [No data for] Component Group Name | 8.64 | 1.050e+08 | 8.76 | N/A | {344.22134} | 343.215 |
| 185 | 113.0603 / 8.70 [M-H]- | Unknown | 113.0603 / 8.70 [M-H]- | Quantifiers | 113.0603 / 8.70 | 8.70 | 6.660e+06 | 8.69 | N/A | {114.06703} | 113.060 |
| 189 | 299.1844 / 8.81 | Unknown | 299.1844 / 8.81 | Quantifiers | [No data for] Component Group Name | 8.81 | 2.304e+06 | 8.84 | N/A | {300.19112} | 299.184 |
| 193 | 153.0902 / 9.09 | Unknown | 153.0902 / 9.09 | Quantifiers | [No data for] Component Group Name | 9.09 | 1.039e+06 | 9.09 | N/A | {154.09692} | 153.090 |
| 195 | 263.1306 / 9.09 | Unknown | 263.1306 / 9.09 | Quantifiers | [No data for] Component Group Name | 9.09 | 1.050e+07 | 9.10 | N/A | {264.13734} | 263.131 |
| 198 | 493.2285 / 9.26 [M+FA-H]- | Unknown | 493.2285 / 9.26 [M+FA-H]- | Quantifiers | 493.2285 / 9.26 | 9.26 | 6.299e+06 | 9.28 | N/A | {448.22977} | 493.229 |
| 199 | 447.2230 / 9.26 [M-H]- | Unknown | 447.2230 / 9.26 [M-H]- | Qualifiers | 493.2285 / 9.26 | 9.26 | 4.858e+06 | 9.29 | N/A | {448.22974} | 447.223 |
| 202 | 329.1601 / 9.38 | Unknown | 329.1601 / 9.38 | Quantifiers | [No data for] Component Group Name | 9.38 | 1.123e+07 | 9.38 | N/A | {330.16685} | 329.160 |

| | | | | | | | | | | | |
|---|---|---|---|---|---|---|---|---|---|---|---|
| 204 | 383.1340 / 9.43 | Unknown | 383.1340 / 9.43 | Quantifiers | [No data for] Component Group Name | 9.44 | 3.940e+06 | 9.43 | N/A | {384.14077} | 383.134 |
| 206 | 147.0454 / 9.49 | Unknown | 147.0454 / 9.49 | Quantifiers | [No data for] Component Group Name | 9.49 | 4.702e+06 | 9.49 | N/A | {148.05212} | 147.045 |
| 207 | 185.1171 / 9.49 | Unknown | 185.1171 / 9.49 | Quantifiers | [No data for] Component Group Name | 9.49 | 3.034e+06 | 9.49 | N/A | {186.12388} | 185.117 |
| 209 | 449.2736 / 9.61 | Unknown | 449.2736 / 9.61 | Quantifiers | [No data for] Component Group Name | 9.61 | 2.436e+06 | 9.62 | N/A | {450.28032} | 449.274 |
| 210 | 683.4034 / 9.61 [M+FA-H]- | Unknown | 683.4034 / 9.61 [M+FA-H]- | Quantifiers | 683.4034 / 9.61 | 9.61 | 3.757e+06 | 9.61 | N/A | {638.40463} | 683.403 |
| 211 | 637.3950 / 9.61 [M-H]- | Unknown | 637.3950 / 9.61 [M-H]- | Qualifiers | 683.4034 / 9.61 | 9.61 | 2.472e+06 | 9.61 | N/A | {638.40173} | 637.395 |
| 216 | 225.1144 / 9.83 | Unknown | 225.1144 / 9.83 | Quantifiers | [No data for] Component Group Name | 9.83 | 1.435e+07 | 9.81 | N/A | {226.12114} | 225.114 |
| 223 | 375.1480 / 9.89 | Unknown | 375.1480 / 9.89 | Quantifiers | [No data for] Component Group Name | 9.89 | 5.444e+06 | 9.89 | N/A | {376.15471} | 375.148 |
| 225 | 199.1336 / 9.95 | Unknown | 199.1336 / 9.95 | Quantifiers | [No data for] Component Group Name | 9.95 | 1.055e+07 | 9.97 | N/A | {200.14030} | 199.134 |
| 226 | 341.1949 / 10.00 | Unknown | 341.1949 / 10.00 | Quantifiers | [No data for] Component Group Name | 10.00 | 6.781e+06 | 9.91 | N/A | {342.20158} | 341.195 |
| 227 | 593.2997 / 10.06 | Unknown | 593.2997 / 10.06 | Quantifiers | [No data for] Component Group Name | 10.06 | 4.046e+06 | 10.06 | N/A | {594.30643} | 593.300 |
| 235 | 609.2699 / 10.23 | Unknown | 609.2699 / 10.23 | Quantifiers | [No data for] Component Group Name | 10.23 | 6.721e+06 | 10.23 | N/A | {610.27662} | 609.270 |
| 243 | 645.3644 / 10.57 [M-H2O-H]- | Unknown | 645.3644 / 10.57 [M-H2O-H]- | Quantifiers | 645.3644 / 10.57 | 10.57 | 1.725e+07 | 10.58 | N/A | {664.38174} | 645.364 |
| 245 | 201.1476 / 10.69 | Unknown | 201.1476 / 10.69 | Quantifiers | [No data for] Component Group Name | 10.69 | 1.330e+06 | 10.66 | N/A | {202.15433} | 201.148 |
| 252 | 679.4110 / 10.86 | Unknown | 679.4110 / 10.86 | Quantifiers | [No data for] Component Group Name | 10.86 | 5.562e+06 | 10.86 | N/A | {680.41772} | 679.411 |
| 253 | 227.1280 / 10.91 | Unknown | 227.1280 / 10.91 | Quantifiers | [No data for] Component Group Name | 10.91 | 8.146e+06 | 10.92 | N/A | {228.13478} | 227.128 |
| 255 | 329.2406 / 10.91 | Unknown | 329.2406 / 10.91 | Quantifiers | [No data for] | 10.91 | 5.277e+07 | 10.89 | N/A | {330.24731} | 329.241 |

| | | | | | | | | | | | |
|---|---|---|---|---|---|---|---|---|---|---|---|
| | | | | | Component Group Name | | | | | | |
| 259 | 707.4025 / 11.03 | Unknown | 707.4025 / 11.03 | Quantifiers | [No data for] Component Group Name | 11.03 | 3.441e+06 | 11.03 | N/A | {708.40919} | 707.402 |
| 266 | 695.4015 / 11.20 | Unknown | 695.4015 / 11.20 | Quantifiers | [No data for] Component Group Name | 11.20 | 1.766e+07 | 10.92 | N/A | {696.40822} | 695.401 |
| 267 | 287.2215 / 11.25 | Unknown | 287.2215 / 11.25 | Quantifiers | [No data for] Component Group Name | 11.25 | 5.209e+06 | 11.26 | N/A | {288.22827} | 287.222 |
| 272 | 195.1383 / 11.42 [M-H2O-H]- | Unknown | 195.1383 / 11.42 [M-H2O-H]- | Quantifiers | 195.1383 / 11.42 | 11.42 | 6.824e+06 | 11.40 | N/A | {214.15564} | 195.138 |
| 273 | 213.1487 / 11.08 [M-H]- | Unknown | 213.1487 / 11.08 [M-H]- | Qualifiers | 195.1383 / 11.42 | 11.08 | 6.001e+06 | 11.07 | N/A | {214.15543} | 213.149 |
| 274 | 239.1280 / 11.42 | Unknown | 239.1280 / 11.42 | Quantifiers | [No data for] Component Group Name | 11.42 | 6.513e+06 | 11.43 | N/A | {240.13476} | 239.128 |
| 278 | 795.4549 / 11.54 | Unknown | 795.4549 / 11.54 | Quantifiers | [No data for] Component Group Name | 11.54 | 1.693e+06 | 11.52 | N/A | {796.46163} | 795.455 |
| 284 | 669.3803 / 11.65 | Unknown | 669.3803 / 11.65 | Quantifiers | [No data for] Component Group Name | 11.65 | 9.216e+06 | 11.62 | N/A | {670.38701} | 669.380 |
| 289 | 309.2073 / 11.82 | Unknown | 309.2073 / 11.82 | Quantifiers | [No data for] Component Group Name | 11.82 | 6.238e+07 | 11.80 | N/A | {310.21400} | 309.207 |
| 319 | 718.4561 / 12.56 | Unknown | 718.4561 / 12.56 | Quantifiers | [No data for] Component Group Name | 12.56 | 6.681e+06 | 12.56 | N/A | {719.46284} | 718.456 |
| 320 | 237.1495 / 12.62 | Unknown | 237.1495 / 12.62 | Quantifiers | [No data for] Component Group Name | 12.62 | 4.372e+06 | 12.62 | N/A | {238.15622} | 237.149 |
| 323 | 669.3805 / 12.73 | Unknown | 669.3805 / 12.73 | Quantifiers | [No data for] Component Group Name | 12.73 | 2.957e+07 | 12.75 | N/A | {670.38725} | 669.381 |
| 326 | 679.4268 / 12.79 | Unknown | 679.4268 / 12.79 | Quantifiers | [No data for] Component Group Name | 12.79 | 1.087e+08 | 12.79 | N/A | {680.43348} | 679.427 |
| 346 | 601.3746 / 13.13 | Unknown | 601.3746 / 13.13 | Quantifiers | [No data for] Component Group Name | 13.13 | 3.106e+07 | 13.12 | N/A | {602.38130} | 601.375 |
| 349 | 571.2930 / 13.30 | Unknown | 571.2930 / 13.30 | Quantifiers | [No data for] Component Group Name | 13.30 | 1.464e+07 | 13.30 | N/A | {572.29970} | 571.293 |
| 351 | 253.1793 / 13.36 | Unknown | 253.1793 / 13.36 | Quantifiers | [No data for] | 13.36 | 5.181e+06 | 13.38 | N/A | {254.18605} | 253.179 |

| | | | | | | | | | | | |
|---|---|---|---|---|---|---|---|---|---|---|---|
| | | | | | Component Group Name | | | | | | |
| 359 | 665.4288 / 13.47 | Unknown | 665.4288 / 13.47 | Quantifiers | [No data for] Component Group Name | 13.47 | 1.678e+07 | 13.47 | N/A | {666.43549} | 665.429 |
| 367 | 667.3590 / 13.64 | Unknown | 667.3590 / 13.64 | Quantifiers | [No data for] Component Group Name | 13.64 | 7.358e+06 | 13.65 | N/A | {668.36568} | 667.359 |
| 368 | 941.6015 / 13.64 | Unknown | 941.6015 / 13.64 | Quantifiers | [No data for] Component Group Name | 13.64 | 2.403e+07 | 13.64 | N/A | {942.60821} | 941.601 |
| 370 | 209.1173 / 13.70 | Unknown | 209.1173 / 13.70 | Quantifiers | [No data for] Component Group Name | 13.70 | 1.268e+07 | 13.70 | N/A | {210.12398} | 209.117 |
| 371 | 289.1787 / 13.70 | Unknown | 289.1787 / 13.70 | Quantifiers | [No data for] Component Group Name | 13.70 | 7.655e+06 | 13.70 | N/A | {290.18540} | 289.179 |
| 373 | 197.1163 / 13.81 | Unknown | 197.1163 / 13.81 | Quantifiers | [No data for] Component Group Name | 13.81 | 1.450e+07 | 13.73 | N/A | {198.12308} | 197.116 |
| 377 | 665.4274 / 13.98 [M+FA-H]- | Unknown | 665.4274 / 13.98 [M+FA-H]- | Quantifiers | 665.4274 / 13.98 | 13.98 | 1.414e+07 | 13.98 | N/A | {620.42863} | 665.427 |
| 380 | 483.2779 / 13.92 | Unknown | 483.2779 / 13.92 | Quantifiers | [No data for] Component Group Name | 13.92 | 9.909e+07 | 13.94 | N/A | {484.28462} | 483.278 |
| 381 | 679.4117 / 13.92 | Unknown | 679.4117 / 13.92 | Quantifiers | [No data for] Component Group Name | 13.92 | 7.255e+07 | 13.97 | N/A | {680.41840} | 679.412 |
| 389 | 305.1736 / 14.15 | Unknown | 305.1736 / 14.15 | Quantifiers | [No data for] Component Group Name | 14.15 | 5.081e+06 | 14.17 | N/A | {306.18028} | 305.174 |
| 390 | 695.4024 / 14.21 | Unknown | 695.4024 / 14.21 | Quantifiers | [No data for] Component Group Name | 14.21 | 8.926e+06 | 14.19 | N/A | {696.40914} | 695.402 |
| 394 | 509.2960 / 14.32 | Unknown | 509.2960 / 14.32 | Quantifiers | [No data for] Component Group Name | 14.32 | 6.939e+07 | 14.32 | N/A | {510.30274} | 509.296 |
| 396 | 279.1946 / 14.38 | Unknown | 279.1946 / 14.38 | Quantifiers | [No data for] Component Group Name | 14.38 | 8.189e+06 | 14.41 | N/A | {280.20138} | 279.195 |
| 405 | 617.3669 / 14.49 | Unknown | 617.3669 / 14.49 | Quantifiers | [No data for] Component Group Name | 14.49 | 1.009e+07 | 14.55 | N/A | {618.37361} | 617.367 |
| 408 | 311.2227 / 14.55 | Unknown | 311.2227 / 14.55 | Quantifiers | [No data for] Component Group Name | 14.55 | 3.871e+07 | 14.47 | N/A | {312.22947} | 311.223 |

| | | | | | | | | | | | |
|---|---|---|---|---|---|---|---|---|---|---|---|
| 409 | 831.4924 / 14.55 | Unknown | 831.4924 / 14.55 | Quantifiers | [No data for] Component Group Name | 14.55 | 1.392e+07 | 14.59 | N/A | {832.49913} | 831.492 |
| 412 | 681.3411 / 14.66 [M+Cl]- | Unknown | 681.3411 / 14.66 [M+Cl]- | Qualifiers | 645.3646 / 14.61 | 14.66 | 5.390e+06 | 14.63 | N/A | {646.37112} | 681.341 |
| 415 | 289.1787 / 14.72 | Unknown | 289.1787 / 14.72 | Quantifiers | [No data for] Component Group Name | 14.72 | 3.974e+06 | 14.69 | N/A | {290.18546} | 289.179 |
| 420 | 291.2008 / 14.83 | Unknown | 291.2008 / 14.83 | Quantifiers | [No data for] Component Group Name | 14.83 | 2.944e+07 | 14.85 | N/A | {292.20748} | 291.201 |
| 427 | 583.3172 / 15.00 | Unknown | 583.3172 / 15.00 | Quantifiers | [No data for] Component Group Name | 15.00 | 1.432e+07 | 14.99 | N/A | {584.32394} | 583.317 |
| 430 | 365.2686 / 15.06 | Unknown | 365.2686 / 15.06 | Quantifiers | [No data for] Component Group Name | 15.06 | 6.779e+06 | 15.04 | N/A | {366.27529} | 365.269 |
| 432 | 517.3580 / 15.12 | Unknown | 517.3580 / 15.12 | Quantifiers | [No data for] Component Group Name | 15.12 | 2.287e+07 | 15.12 | N/A | {518.36471} | 517.358 |
| 435 | 235.1686 / 15.17 | Unknown | 235.1686 / 15.17 | Quantifiers | [No data for] Component Group Name | 15.18 | 4.811e+06 | 15.15 | N/A | {236.17532} | 235.169 |
| 436 | 275.2017 / 15.17 | Unknown | 275.2017 / 15.17 | Quantifiers | [No data for] Component Group Name | 15.18 | 3.453e+07 | 15.18 | N/A | {276.20840} | 275.202 |
| 438 | 911.6336 / 15.29 | Unknown | 911.6336 / 15.29 | Quantifiers | [No data for] Component Group Name | 15.29 | 5.604e+07 | 15.32 | N/A | {912.64034} | 911.634 |
| 439 | 293.2260 / 15.35 | Unknown | 293.2260 / 15.35 | Quantifiers | [No data for] Component Group Name | 15.35 | 6.636e+08 | 15.39 | N/A | {294.23276} | 293.226 |
| 443 | 511.3075 / 15.40 | Unknown | 511.3075 / 15.40 | Quantifiers | [No data for] Component Group Name | 15.40 | 1.742e+08 | 15.45 | N/A | {512.31420} | 511.307 |
| 447 | 271.2257 / 15.74 | Unknown | 271.2257 / 15.74 | Quantifiers | [No data for] Component Group Name | 15.74 | 7.360e+06 | 15.76 | N/A | {272.23239} | 271.226 |
| 453 | 295.2253 / 15.86 | Unknown | 295.2253 / 15.86 | Quantifiers | [No data for] Component Group Name | 15.86 | 5.277e+07 | 16.22 | N/A | {296.23205} | 295.225 |
| 456 | 291.2014 / 15.97 | Unknown | 291.2014 / 15.97 | Quantifiers | [No data for] Component Group Name | 15.97 | 7.894e+07 | 15.95 | N/A | {292.20811} | 291.201 |
| 464 | 517.3580 / 16.14 | Unknown | 517.3580 / 16.14 | Quantifiers | [No data for] Component Group | 16.14 | 3.353e+07 | 16.16 | N/A | {518.36470} | 517.358 |

| | | | | | | | | | | | |
|---|---|---|---|---|---|---|---|---|---|---|---|
| | | | | | Name | | | | | | |
| 465 | 699.3827 / 16.14 | Unknown | 699.3827 / 16.14 | Quantifiers | [No data for] Component Group Name | 16.14 | 9.307e+06 | 16.11 | N/A | {700.38947} | 699.383 |
| 467 | 295.2390 / 16.20 | Unknown | 295.2390 / 16.20 | Quantifiers | [No data for] Component Group Name | 16.20 | 7.644e+07 | 16.19 | N/A | {296.24574} | 295.239 |
| 470 | 869.4814 / 16.42 [M+Na-2H]- | Unknown | 869.4814 / 16.42 [M+Na-2H]- | Qualifiers | 847.5013 / 16.25 | 16.43 | 3.760e+06 | 16.43 | N/A | {848.50616} | 869.481 |
| 471 | 337.2362 / 16.31 | Unknown | 337.2362 / 16.31 | Quantifiers | [No data for] Component Group Name | 16.31 | 2.390e+06 | 16.33 | N/A | {338.24297} | 337.236 |
| 472 | 519.3654 / 16.31 | Unknown | 519.3654 / 16.31 | Quantifiers | [No data for] Component Group Name | 16.31 | 4.010e+06 | 16.27 | N/A | {520.37209} | 519.365 |
| 474 | 295.2261 / 16.37 | Unknown | 295.2261 / 16.37 | Quantifiers | [No data for] Component Group Name | 16.37 | 5.573e+07 | 16.22 | N/A | {296.23286} | 295.226 |
| 476 | 849.5047 / 16.37 | Unknown | 849.5047 / 16.37 | Quantifiers | [No data for] Component Group Name | 16.37 | 7.288e+06 | 16.31 | N/A | {850.51144} | 849.505 |
| 479 | 243.1947 / 16.54 | Unknown | 243.1947 / 16.54 | Quantifiers | [No data for] Component Group Name | 16.54 | 3.307e+06 | 16.57 | N/A | {244.20143} | 243.195 |
| 480 | 573.3767 / 16.54 | Unknown | 573.3767 / 16.54 | Quantifiers | [No data for] Component Group Name | 16.54 | 2.123e+06 | 16.53 | N/A | {574.38347} | 573.377 |
| 482 | 293.2205 / 16.65 | Unknown | 293.2205 / 16.65 | Quantifiers | [No data for] Component Group Name | 16.65 | 9.385e+07 | 16.66 | N/A | {294.22721} | 293.220 |
| 486 | 585.3782 / 16.71 | Unknown | 585.3782 / 16.71 | Quantifiers | [No data for] Component Group Name | 16.71 | 5.689e+06 | 16.71 | N/A | {586.38493} | 585.378 |
| 497 | 321.2448 / 16.88 | Unknown | 321.2448 / 16.88 | Quantifiers | [No data for] Component Group Name | 16.88 | 1.051e+07 | 16.86 | N/A | {322.25157} | 321.245 |
| 501 | 295.2262 / 17.05 | Unknown | 295.2262 / 17.05 | Quantifiers | [No data for] Component Group Name | 17.05 | 2.884e+06 | 17.03 | N/A | {296.23288} | 295.226 |
| 502 | 362.2682 / 17.05 | Unknown | 362.2682 / 17.05 | Quantifiers | [No data for] Component Group Name | 17.05 | 2.146e+06 | 17.03 | N/A | {363.27492} | 362.268 |
| 507 | 335.2210 / 17.16 [M+AcO-H]- | Unknown | 335.2210 / 17.16 [M+AcO-H]- | Quantifiers | 335.2210 / 17.16 | 17.16 | 8.267e+05 | 17.15 | N/A | {276.20664} | 335.221 |
| 511 | 249.1858 / 17.16 | Unknown | 249.1858 / 17.16 | Quantifiers | [No data for] Component Group | 17.16 | 2.622e+06 | 17.19 | N/A | {250.19257} | 249.186 |

| | | | | | | | | | | | |
|---|---|---|---|---|---|---|---|---|---|---|---|
| | | | | | Name | | | | | | |
| 516 | 289.1808 / 17.28 | Unknown | 289.1808 / 17.28 | Quantifiers | [No data for] Component Group Name | 17.28 | 3.361e+06 | 17.25 | N/A | {290.18752} | 289.181 |
| 517 | 365.2725 / 17.28 | Unknown | 365.2725 / 17.28 | Quantifiers | [No data for] Component Group Name | 17.28 | 7.796e+06 | 17.28 | N/A | {366.27928} | 365.273 |
| 518 | 787.4677 / 17.28 | Unknown | 787.4677 / 17.28 | Quantifiers | [No data for] Component Group Name | 17.28 | 1.890e+06 | 17.25 | N/A | {788.47442} | 787.468 |
| 522 | 269.2462 / 17.33 | Unknown | 269.2462 / 17.33 | Quantifiers | [No data for] Component Group Name | 17.33 | 3.424e+06 | 17.34 | N/A | {270.25292} | 269.246 |
| 525 | 813.4836 / 17.33 | Unknown | 813.4836 / 17.33 | Quantifiers | [No data for] Component Group Name | 17.33 | 2.571e+06 | 17.35 | N/A | {814.49035} | 813.484 |
| 531 | 277.2156 / 17.45 | Unknown | 277.2156 / 17.45 | Quantifiers | [No data for] Component Group Name | 17.45 | 1.109e+07 | 17.88 | N/A | {278.22234} | 277.216 |
| 532 | 283.2638 / 17.45 | Unknown | 283.2638 / 17.45 | Quantifiers | [No data for] Component Group Name | 17.45 | 4.076e+06 | 17.45 | N/A | {284.27049} | 283.264 |
| 535 | 263.2003 / 17.50 | Unknown | 263.2003 / 17.50 | Quantifiers | [No data for] Component Group Name | 17.51 | 7.966e+05 | 17.50 | N/A | {264.20704} | 263.200 |
| 537 | 831.5072 / 17.50 | Unknown | 831.5072 / 17.50 | Quantifiers | [No data for] Component Group Name | 17.51 | 1.122e+07 | 17.54 | N/A | {832.51393} | 831.507 |
| 539 | 251.2000 / 17.62 | Unknown | 251.2000 / 17.62 | Quantifiers | [No data for] Component Group Name | 17.62 | 4.880e+05 | 17.61 | N/A | {252.20674} | 251.200 |
| 545 | 307.1899 / 17.79 [M-H]- | Unknown | 307.1899 / 17.79 [M-H]- | Quantifiers | 307.1899 / 17.79 | 17.79 | 6.882e+05 | 17.79 | N/A | {308.19664} | 307.190 |
| 548 | 277.2279 / 17.85 | Unknown | 277.2279 / 17.85 | Quantifiers | [No data for] Component Group Name | 17.85 | 1.801e+07 | 17.87 | N/A | {278.23464} | 277.228 |
| 550 | 590.4449 / 17.85 | Unknown | 590.4449 / 17.85 | Quantifiers | [No data for] Component Group Name | 17.85 | 2.706e+06 | 17.87 | N/A | {591.45159} | 590.445 |
| 554 | 831.4934 / 18.02 | Unknown | 831.4934 / 18.02 | Quantifiers | [No data for] Component Group Name | 18.02 | 8.843e+06 | 17.57 | N/A | {832.50016} | 831.493 |
| 556 | 253.2232 / 18.13 [M-H2O-H]- | Unknown | 253.2232 / 18.13 [M-H2O-H]- | Quantifiers | 253.2232 / 18.13 | 18.13 | 5.362e+06 | 18.19 | N/A | {272.24046} | 253.223 |
| 557 | 271.2331 / 17.96 [M-H]- | Unknown | 271.2331 / 17.96 [M-H]- | Qualifiers | 253.2232 / 18.13 | 17.96 | 4.745e+06 | 17.97 | N/A | {272.23979} | 271.233 |

| | | | | | | | | | | | |
|---|---|---|---|---|---|---|---|---|---|---|---|
| 560 | 279.2401 / 18.30 | Unknown | 279.2401 / 18.30 | Quantifiers | [No data for] Component Group Name | 18.30 | 3.381e+06 | 18.28 | N/A | {280.24680} | 279.240 |
| 565 | 255.2390 / 18.58 | Unknown | 255.2390 / 18.58 | Quantifiers | [No data for] Component Group Name | 18.58 | 5.384e+06 | 18.57 | N/A | {256.24571} | 255.239 |
| 572 | 311.2933 / 18.81 | Unknown | 311.2933 / 18.81 | Quantifiers | [No data for] Component Group Name | 18.81 | 2.879e+05 | 18.83 | N/A | {312.30000} | 311.293 |
| 575 | 281.2487 / 19.15 | Unknown | 281.2487 / 19.15 | Quantifiers | [No data for] Component Group Name | 19.15 | 6.012e+06 | 19.15 | N/A | {282.25540} | 281.249 |
| 576 | 349.2733 / 19.15 | Unknown | 349.2733 / 19.15 | Quantifiers | [No data for] Component Group Name | 19.15 | 4.711e+05 | 19.16 | N/A | {350.28006} | 349.273 |
| 580 | 609.3920 / 19.27 | Unknown | 609.3920 / 19.27 | Quantifiers | [No data for] Component Group Name | 19.27 | 2.845e+06 | 19.27 | N/A | {610.39871} | 609.392 |
| 590 | 255.2372 / 19.38 | Unknown | 255.2372 / 19.38 | Quantifiers | [No data for] Component Group Name | 19.38 | 2.054e+07 | 19.22 | N/A | {256.24396} | 255.237 |

| # | Analyte Peak Name | Found At Mass | Library Hit | Library Score |
|---|---|---|---|---|
| 1 | 128.0342 / 1.02 | 128.0343 | D-Pyroglutamic acid (NIST) [Smart Confirmation] | 100.0 |
| 10 | 116.0715 / 1.19 | 116.0713 | 3-Aminopentanoic acid (NIST) [Smart Confirmation] | 50.0 |
| 11 | 179.0551 / 1.19 | 179.0551 | D-甘露糖 D-(+)-Mannose [Smart Confirmation] | 97.5 |
| 13 | 341.1085 / 1.19 [M-H]- | 341.1083 | D-(+)-Trehalose (NIST) [Smart Confirmation] | 67.7 |
| 18 | 111.0192 / 1.37 | 111.0191 | 2-Furancarboxylic acid (NIST) [Smart Confirmation] | 39.0 |
| 19 | 180.0652 / 1.37 | 180.0652 | L-Tyrosine (NIST) [Smart Confirmation] | 91.7 |
| 24 | 243.0683 / 1.42 | 243.0695 | 尿苷 Uridine [Smart Confirmation] | 91.0 |
| 27 | 130.0881 / 1.48 | 130.0884 | 异亮氨酸Isoleucine [Smart Confirmation] | 100.0 |
| 29 | 202.1070 / 1.48 | 202.1069 | 1-(2,8-Dihydroxyquinolin-5-yl)ethan-1-one (NIST) [Smart Confirmation] | 45.0 |
| 30 | 247.1173 / 1.48 [M-H]- | 247.1174 | Asp-Asp (NIST) [Smart Confirmation] | 96.4 |
| 35 | 282.0858 / 1.54 | 282.0863 | 鸟苷guanosine [Smart Confirmation] | 100.0 |
| 36 | 137.0240 / 1.59 | 137.0240 | 3-Hydroxybenzoic acid (NIST) [Smart Confirmation] | 99.2 |
| 38 | 299.0821 / 1.59 | 299.0823 | 13,14-Dihydro-15-ketotetranorprostaglandin F1.alpha. (NIST) [Smart Confirmation] | 25.9 |
| 42 | 134.0519 / 1.71 | 134.0521 | 腺嘌呤Adenine [Smart Confirmation] | 99.2 |
| 43 | 337.1493 / 1.71 | 337.1492 | Bavachinin (NIST) [Smart Confirmation] | 25.5 |
| 44 | 175.0981 / 1.82 | 175.0979 | 2-Isopropylmalic acid (NIST) [Smart Confirmation] | 60.6 |
| 45 | 257.0773 / 1.82 | 257.0771 | 5-Methyluridine (NIST) [Smart Confirmation] | 60.5 |
| 49 | 164.0765 / 1.99 | 164.0778 | 苯丙氨酸Phenprobamate [Smart Confirmation] | 98.6 |
| 52 | 137.0280 / 2.27 | 137.0279 | 对羟基苯甲酸 4-Hydroxybenzoic acid [Smart Confirmation] | 100.0 |
| 53 | 192.0656 / 2.33 | 192.0657 | 4-(Butylamino)benzoic acid (NIST) [Smart Confirmation] | 80.9 |

| | | | | |
|---|---|---|---|---|
| 55 | 172.0968 / 2.39 | 172.0969 | N-Acetyl-D-norleucine (NIST) [Smart Confirmation] | 95.7 |
| 57 | 138.0552 / 2.44 | 138.0551 | 5-Amino-2-methoxyphenol (NIST) [Smart Confirmation] | 97.1 |
| 62 | 307.1381 / 2.67 | 307.1382 | Bisdemethoxycurcumin (NIST) [Smart Confirmation] | 43.2 |
| 64 | 206.0801 / 2.73 | 206.0800 | Propachlor OA (NIST) [Smart Confirmation] | 54.2 |
| 65 | 327.1067 / 2.73 | 327.1068 | 草夹竹桃苷 Androsin [Smart Confirmation] | 53.9 |
| 66 | 153.0178 / 2.79 | 153.0180 | 2,6-Dihydroxybenzoic acid (NIST) [Smart Confirmation] | 94.1 |
| 69 | 167.0335 / 2.84 | 167.0335 | 香草酸Vanillic acid [Smart Confirmation] | 91.8 |
| 70 | 245.0654 / 2.84 | 245.0655 | Met-Pro (NIST) [Smart Confirmation] | 23.1 |
| 71 | 443.1905 / 2.96 | 443.1906 | O-Desmethylastemizole (NIST) [Smart Confirmation] | 24.3 |
| 73 | 203.0858 / 3.07 | 203.0861 | 色氨酸 L-Tryptophan [Smart Confirmation] | 77.3 |
| 74 | 399.1642 / 3.13 | 399.1646 | Methanone, 6-hydroxy-1-2-(4-morpholinyl)ethyl-1H-indol-3-yl-1-naphthalenyl- (NIST) [Smart Confirmation] | 60.3 |
| 77 | 387.1636 / 3.35 | 387.1634 | 15-Ketolatanoprost (free acid) (NIST) [Smart Confirmation] | 31.4 |
| 84 | 339.0710 / 4.83 | 339.0711 | Esculin (NIST) [Smart Confirmation] | 92.1 |
| 88 | 461.1476 / 5.00 | 461.1478 | 野黄芩苷 Scutellarin [Smart Confirmation] | 21.7 |
| 89 | 387.1644 / 5.34 | 387.1647 | 京尼平苷 Geniposide [Smart Confirmation] | 58.6 |
| 93 | 433.2074 / 5.63 | 433.2077 | Engeletin (NIST) [Smart Confirmation] | 30.1 |
| 106 | 121.0287 / 6.20 | 121.0288 | 3-Hydroxybenzaldehyde (NIST) [Smart Confirmation] | 95.0 |
| 107 | 421.1642 / 6.25 [M+Cl]- | 421.1641 | Dioctyl sulfosuccinate (NIST) [Smart Confirmation] | 56.7 |
| 109 | 431.2011 / 6.25 | 431.2020 | Naphthofluorescein (NIST) [Smart Confirmation] | 75.0 |
| 114 | 353.1259 / 6.37 | 353.1262 | 绿原酸 Chlorogenic acid [Smart Confirmation] | 77.2 |
| 122 | 585.2392 / 6.65 | 585.2394 | 1-(1,2-Dioctanoylphosphatidyl)inositol (NIST) [Smart Confirmation] | 62.4 |
| 126 | 475.1597 / 6.71 | 475.1593 | Glycine, N,N'-1,2-ethanediylbis(oxy-2,1-phenylene)bisN-(carboxymethyl)- (NIST) [Smart Confirmation] | 36.2 |
| 127 | 193.0495 / 6.93 | 193.0494 | 异阿魏酸 Isoferulic acid [Smart Confirmation] | 94.1 |
| 128 | 247.1540 / 6.93 | 247.1541 | Asp-Asp (NIST) [Smart Confirmation] | 81.8 |
| 133 | 551.2346 / 6.99 | 551.2351 | 远志口山酮IX PolygalaxanthoneⅨ [Smart Confirmation] | 85.8 |
| 134 | 159.1023 / 7.05 | 159.1024 | 2-Hydroxyoctanoic acid (NIST) [Smart Confirmation] | 90.3 |
| 136 | 433.2066 / 7.10 | 433.2070 | 栀子苷 Geniposide + HCOOH [Smart Confirmation] | 95.1 |
| 144 | 361.2209 / 7.45 | 361.2208 | Pyrasulfotole (NIST) [Smart Confirmation] | 57.3 |
| 149 | 463.0867 / 7.50 | 463.0866 | 金丝桃苷 Hyperin [Smart Confirmation] | 98.7 |
| 151 | 271.1539 / 7.67 | 271.1539 | DL-.beta.-Hydroxypalmitic acid (NIST) [Smart Confirmation] | 99.1 |
| 157 | 187.0992 / 8.01 | 187.0992 | Azelaic acid (NIST) [Smart Confirmation] | 98.5 |
| 161 | 447.0934 / 8.01 | 447.0931 | 紫云英苷 Astragalin [Smart Confirmation] | 100.0 |
| 162 | 229.1455 / 8.07 | 229.1456 | 2,3,4,6-Tetrachlorophenol (NIST) [Smart Confirmation] | 100.0 |
| 167 | 431.1732 / 8.07 | 431.1729 | 3.beta.,7.alpha.-Dihydroxy-5-cholestenoic acid (NIST) [Smart Confirmation] | 35.8 |
| 169 | 144.0446 / 8.13 | 144.0449 | Quinolin-2-ol (NIST) [Smart Confirmation] | 78.9 |
| 173 | 231.1604 / 8.30 | 231.1605 | (.+-.)-Camphor-10-sulfonic acid (NIST) [Smart Confirmation] | 86.1 |
| 176 | 173.1181 / 8.36 | 173.1181 | L-Theanine (NIST) [Smart Confirmation] | 65.6 |
| 177 | 171.1015 / 8.41 | 171.1016 | trans-1,4-Cyclohexanedicarboxylic acid (NIST) [Smart Confirmation] | 24.8 |
| 178 | 381.1544 / 8.41 | 381.1545 | Ibuprofen .beta.-D-glucuronide (NIST) [Smart Confirmation] | 22.5 |
| 181 | 229.1454 / 8.58 | 229.1456 | 2,3,4,6-Tetrachlorophenol (NIST) [Smart Confirmation] | 100.0 |
| 183 | 281.0808 / 9.04 [M+K-2H]- | 281.0805 | Niflumic acid (NIST) [Smart Confirmation] | 78.1 |

| | | | | |
|---|---|---|---|---|
| 184 | 343.2146 / 8.64 | 343.2143 | Lactitol (NIST) [Smart Confirmation] | 22.7 |
| 185 | 113.0603 / 8.70 [M-H]- | 113.0607 | Trifluoroacetic acid (NIST) [Smart Confirmation] | 50.7 |
| 189 | 299.1844 / 8.81 | 299.1843 | Cinnabarinic acid (NIST) [Smart Confirmation] | 29.3 |
| 193 | 153.0902 / 9.09 | 153.0904 | O,O-Diethyl phosphate (NIST) [Smart Confirmation] | 69.6 |
| 195 | 263.1306 / 9.09 | 263.1308 | (+)-Abscisic acid (NIST) [Smart Confirmation] | 98.1 |
| 198 | 493.2285 / 9.26 [M+FA-H]- | 493.2287 | Glucobrassicin (NIST) [Smart Confirmation] | 21.9 |
| 199 | 447.2230 / 9.26 [M-H]- | 447.2229 | 1,3,5(10)-Estratrien-3,17.beta.-diol 17-glucosiduronate (NIST) [Smart Confirmation] | 22.1 |
| 202 | 329.1601 / 9.38 | 329.1605 | Furosemide (NIST) [Smart Confirmation] | 51.6 |
| 204 | 383.1340 / 9.43 | 383.1342 | 17,20-Dimethylprostaglandin F1.alpha. (NIST) [Smart Confirmation] | 25.5 |
| 206 | 147.0454 / 9.49 | 147.0454 | 肉桂酸 cinnamic acid [Smart Confirmation] | 99.4 |
| 207 | 185.1171 / 9.49 | 185.1174 | Gabapentin related compound E (NIST) [Smart Confirmation] | 83.9 |
| 209 | 449.2736 / 9.61 | 449.2740 | Eriodictyol-7-O-glucoside (NIST) [Smart Confirmation] | 76.9 |
| 210 | 683.4034 / 9.61 [M+FA-H]- | 683.4030 | 20(R)-人参皂苷Rh1 20(R)-Ginsenoside Rh1 +HCOOH [Smart Confirmation] | 85.5 |
| 211 | 637.3950 / 9.61 [M-H]- | 637.3951 | 异肉苁蓉苷Ciso-Cistanoside C [Smart Confirmation] | 64.7 |
| 216 | 225.1144 / 9.83 | 225.1147 | Benzoic acid, 4-benzoyl (NIST) [Smart Confirmation] | 35.9 |
| 223 | 375.1480 / 9.89 | 375.1478 | Resolvin D2 (NIST) [Smart Confirmation] | 26.7 |
| 225 | 199.1336 / 9.95 | 199.1338 | Dodecanoic acid (NIST) [Smart Confirmation] | 95.0 |
| 226 | 341.1949 / 10.00 | 341.1951 | Hexadecanedioic acid, 3,3,14,14-tetramethyl- (NIST) [Smart Confirmation] | 67.4 |
| 227 | 593.2997 / 10.06 | 593.2994 | 2',6'-Dihydroxy-4-methoxychalcone-4'-O-neohesperidoside (NIST) [Smart Confirmation] | 60.8 |
| 235 | 609.2699 / 10.23 | 609.2694 | 1-(1,2-Dihexanoylphosphatidyl)inositol-5-phosphate (NIST) [Smart Confirmation] | 76.5 |
| 243 | 645.3644 / 10.57 [M-H2O-H]- | 645.3645 | N-Lauroyl-D-erythro-sphingosylphosphorylcholine (NIST) [Smart Confirmation] | 23.9 |
| 245 | 201.1476 / 10.69 | 201.1481 | Bergaptol (NIST) [Smart Confirmation] | 65.7 |
| 252 | 679.4110 / 10.86 | 679.4114 | Dipyridamole mono-O-.beta.-D-glucuronide (NIST) [Smart Confirmation] | 57.6 |
| 253 | 227.1280 / 10.91 | 227.1281 | Butanedioic acid, 2-(4,4-dimethyl-2-methylenepentyl)- (NIST) [Smart Confirmation] | 95.5 |
| 255 | 329.2406 / 10.91 | 329.2423 | 4,2'-Dihydroxy-3,4',6'-trimethoxychalcone (NIST) [Smart Confirmation] | 66.2 |
| 259 | 707.4025 / 11.03 | 707.4027 | Ionomycin (NIST) [Smart Confirmation] | 31.7 |
| 266 | 695.4015 / 11.20 | 695.4009 | 杠柳毒苷 Periplocoside [Smart Confirmation] | 29.3 |
| 267 | 287.2215 / 11.25 | 287.2217 | Ciprofibrate (NIST) [Smart Confirmation] | 81.2 |
| 272 | 195.1383 / 11.42 [M-H2O-H]- | 195.1383 | 2,4,6-Trichlorophenol (NIST) [Smart Confirmation] | 100.0 |
| 273 | 213.1487 / 11.08 [M-H]- | 213.1487 | Tridecanoic acid (NIST) [Smart Confirmation] | 22.7 |
| 274 | 239.1280 / 11.42 | 239.1280 | Picloram (NIST) [Smart Confirmation] | 87.5 |
| 278 | 795.4549 / 11.54 | 795.4544 | α-常春藤皂苷 α-hederin +HCOOH [Smart Confirmation] | 33.2 |
| 284 | 669.3803 / 11.65 | 669.3800 | Monensin (NIST) [Smart Confirmation] | 41.2 |
| 289 | 309.2073 / 11.82 | 309.2075 | 9-Hydroperoxy-10E,12Z,15Z-octadecatrienoic acid (NIST) [Smart Confirmation] | 20.0 |
| 319 | 718.4561 / 12.56 | 718.4562 | 1-Palmitoyl-2-(4-ketododec-3-enedioyl)phosphatidylcholine (NIST) [Smart Confirmation] | 24.5 |
| 320 | 237.1495 / 12.62 | 237.1495 | 4-(2-Hydroxyethyl)piperazine-1-ethanesulfonic acid (NIST) [Smart Confirmation] | 62.2 |
| 323 | 669.3805 / 12.73 | 669.3804 | Monensin (NIST) [Smart Confirmation] | 36.8 |
| 326 | 679.4268 / 12.79 | 679.4270 | Dipyridamole mono-O-.beta.-D-glucuronide (NIST) [Smart Confirmation] | 20.8 |
| 346 | 601.3746 / 13.13 | 601.3747 | Garcinol (NIST) [Smart Confirmation] | 69.9 |
| 349 | 571.2930 / 13.30 | 571.2936 | 1-Hexadecanoyl-sn-glycero-3-phospho-(1'-myo-inositol) (NIST) [Smart Confirmation] | 87.9 |

| | | | | |
|---|---|---|---|---|
| 351 | 253.1793 / 13.36 | 253.1792 | Palmitelaidic acid (NIST) [Smart Confirmation] | 83.8 |
| 359 | 665.4288 / 13.47 | 665.4290 | 1-(1,2-Dioctanoylphosphatidyl)inositol-5-phosphate (NIST) [Smart Confirmation] | 68.1 |
| 367 | 667.3590 / 13.64 | 667.3588 | 20(R)-人参皂苷Rh2 20(R)-Ginsenoside Rh2 +HCOOH [Smart Confirmation] | 35.9 |
| 368 | 941.6015 / 13.64 | 941.6021 | Soyasaponin I (NIST) [Smart Confirmation] | 74.8 |
| 370 | 209.1173 / 13.70 | 209.1172 | Jasmonic acid (NIST) [Smart Confirmation] | 77.2 |
| 371 | 289.1787 / 13.70 | 289.1788 | 表儿茶素 Epicatechin [Smart Confirmation] | 89.5 |
| 373 | 197.1163 / 13.81 | 197.1165 | cis-5-Dodecenoic acid (NIST) [Smart Confirmation] | 50.9 |
| 377 | 665.4274 / 13.98 [M+FA-H]- | 665.4276 | 1-(1,2-Dioctanoylphosphatidyl)inositol-5-phosphate (NIST) [Smart Confirmation] | 55.7 |
| 380 | 483.2779 / 13.92 | 483.2812 | 1-Palmitoyl-2-hydroxy-sn-glycero-3-phospho-(1'-rac-glycerol) (NIST) [Smart Confirmation] | 98.5 |
| 381 | 679.4117 / 13.92 | 679.4121 | Dipyridamole mono-O-.beta.-D-glucuronide (NIST) [Smart Confirmation] | 64.4 |
| 389 | 305.1736 / 14.15 | 305.1737 | 5Z,8Z,14Z-Eicosatrienoic acid (NIST) [Smart Confirmation] | 30.2 |
| 390 | 695.4024 / 14.21 | 695.4024 | 长梗冬青苷 Pedunculoside +HCOOH [Smart Confirmation] | 91.0 |
| 394 | 509.2960 / 14.32 | 509.2981 | 1-Oleoyl-2-hydroxy-sn-glycero-3-phospho-(1'-rac-glycerol) (NIST) [Smart Confirmation] | 99.7 |
| 396 | 279.1946 / 14.38 | 279.1947 | 10E,12Z-octadecadienoic acid (NIST) [Smart Confirmation] | 84.9 |
| 405 | 617.3669 / 14.49 | 617.3673 | 7-Ethyl-10-(4-N-aminopentanoic acid)-1-piperidino)carbonyloxycamptothecin (NIST) [Smart Confirmation] | 27.7 |
| 408 | 311.2227 / 14.55 | 311.2228 | 9-Hydroperoxy-10E,12Z-octadecadienoic acid (NIST) [Smart Confirmation] | 29.3 |
| 409 | 831.4924 / 14.55 | 831.4917 | B-Pentasaccharide (NIST) [Smart Confirmation] | 78.6 |
| 412 | 681.3411 / 14.66 [M+Cl]- | 681.3411 | Deacylgymnemic acid (NIST) [Smart Confirmation] | 44.5 |
| 415 | 289.1787 / 14.72 | 289.1788 | 表儿茶素 Epicatechin [Smart Confirmation] | 25.0 |
| 420 | 291.2008 / 14.83 | 291.2019 | 3-Bromo-5-phenylsalicylic acid (NIST) [Smart Confirmation] | 22.6 |
| 427 | 583.3172 / 15.00 | 583.3184 | 胆红素 Bilirubin [Smart Confirmation] | 23.3 |
| 430 | 365.2686 / 15.06 | 365.2684 | cis-15-Tetracosenoic acid (NIST) [Smart Confirmation] | 84.4 |
| 432 | 517.3580 / 15.12 | 517.3583 | 西伯利亚远志糖A5Sibiricose A5 [Smart Confirmation] | 61.8 |
| 435 | 235.1686 / 15.17 | 235.1686 | Asp-Cys (NIST) [Smart Confirmation] | 87.3 |
| 436 | 275.2017 / 15.17 | 275.2018 | Stearidonic acid (NIST) [Smart Confirmation] | 92.0 |
| 438 | 911.6336 / 15.29 | 911.6349 | Soyasaponin II (NIST) [Smart Confirmation] | 61.4 |
| 439 | 293.2260 / 15.35 | 293.2264 | 13S-Hydroxy-9Z,11E,15Z-octadecatrienoic acid (NIST) [Smart Confirmation] | 96.5 |
| 443 | 511.3075 / 15.40 | 511.3081 | 1-Octadecanoyl-sn-glycero-3-phospho-(1'-sn-glycerol) (NIST) [Smart Confirmation] | 76.1 |
| 447 | 271.2257 / 15.74 | 271.2255 | DL-.beta.-Hydroxypalmitic acid (NIST) [Smart Confirmation] | 87.0 |
| 453 | 295.2253 / 15.86 | 295.2360 | 13R-Hydroxy-9Z,11E-octadecadienoic acid (NIST) [Smart Confirmation] | 93.9 |
| 456 | 291.2014 / 15.97 | 291.1977 | Monastrol (NIST) [Smart Confirmation] | 62.2 |
| 464 | 517.3580 / 16.14 | 517.3572 | Gossypol (NIST) [Smart Confirmation] | 52.4 |
| 465 | 699.3827 / 16.14 | 699.3830 | 拟人参皂苷RT5 Pseudoginsenoside-RT5 +HCOOH [Smart Confirmation] | 41.8 |
| 467 | 295.2390 / 16.20 | 295.2392 | 13R-Hydroxy-9Z,11E-octadecadienoic acid (NIST) [Smart Confirmation] | 94.4 |
| 470 | 869.4814 / 16.42 [M+Na-2H]- | 869.4815 | 短葶山麦冬皂苷 C Liriope muscari baily saponins C [Smart Confirmation] | 32.5 |
| 471 | 337.2362 / 16.31 | 337.2364 | Ethylene glycol tetradecyl ether sulfate (NIST) [Smart Confirmation] | 74.2 |
| 472 | 519.3654 / 16.31 | 519.3647 | Beclomethasone dipropionate (NIST) [Smart Confirmation] | 56.0 |
| 474 | 295.2261 / 16.37 | 295.2360 | 13R-Hydroxy-9Z,11E-octadecadienoic acid (NIST) [Smart Confirmation] | 93.9 |
| 476 | 849.5047 / 16.37 | 849.5041 | Man7-2AA (NIST) [Smart Confirmation] | 30.2 |
| 479 | 243.1947 / 16.54 | 243.1947 | 3-Hydroxymyristic acid (NIST) [Smart Confirmation] | 37.6 |

| | | | | |
|---|---|---|---|---|
| 480 | 573.3767 / 16.54 | 573.3763 | 川楝素 Toosendanin [Smart Confirmation] | 40.1 |
| 482 | 293.2205 / 16.65 | 293.2200 | 13-Keto-9Z,11E-octadecadienoic acid (NIST) [Smart Confirmation] | 93.9 |
| 486 | 585.3782 / 16.71 | 585.3783 | 1-(1,2-Dioctanoylphosphatidyl)inositol (NIST) [Smart Confirmation] | 54.6 |
| 497 | 321.2448 / 16.88 | 321.2449 | Neobavaisoflavone (NIST) [Smart Confirmation] | 35.5 |
| 501 | 295.2262 / 17.05 | 295.2263 | 12(13)-Epoxy-9Z-octadecenoic acid (NIST) [Smart Confirmation] | 77.5 |
| 502 | 362.2682 / 17.05 | 362.2682 | N-Palmitoyltaurine (NIST) [Smart Confirmation] | 37.2 |
| 507 | 335.2210 / 17.16 [M+AcO-H]- | 335.2206 | Stearidonic acid (NIST) [Smart Confirmation] | 84.7 |
| 511 | 249.1858 / 17.16 | 249.1857 | Gemfibrozil (NIST) [Smart Confirmation] | 30.0 |
| 516 | 289.1808 / 17.28 | 289.1809 | 表儿茶素 Epicatechin [Smart Confirmation] | 84.9 |
| 517 | 365.2725 / 17.28 | 365.2734 | 17-Phenyltrinor-8-iso-prostaglandin E2 (NIST) [Smart Confirmation] | 91.7 |
| 518 | 787.4677 / 17.28 | 787.4677 | Phomopsin A (NIST) [Smart Confirmation] | 28.0 |
| 522 | 269.2462 / 17.33 | 269.2461 | Heptadecanoic acid (NIST) [Smart Confirmation] | 98.7 |
| 525 | 813.4836 / 17.33 | 813.4841 | Crocin II (NIST) [Smart Confirmation] | 21.7 |
| 531 | 277.2156 / 17.45 | 277.2250 | Pinolenic acid (NIST) [Smart Confirmation] | 92.3 |
| 532 | 283.2638 / 17.45 | 283.2642 | 2-Bromo-4,6-di(tert-butyl)phenol (NIST) [Smart Confirmation] | 72.5 |
| 535 | 263.2003 / 17.50 | 263.2001 | Asp-Met (NIST) [Smart Confirmation] | 92.2 |
| 537 | 831.5072 / 17.50 | 831.5090 | B-Pentasaccharide (NIST) [Smart Confirmation] | 73.3 |
| 539 | 251.2000 / 17.62 | 251.2002 | N-2-Hydroxyethylpiperazine-N-3-propanesulfonic acid (NIST) [Smart Confirmation] | 74.3 |
| 545 | 307.1899 / 17.79 [M-H]- | 307.1899 | 2,2-Bis(3-allyl-4-hydroxyphenyl)propane (NIST) [Smart Confirmation] | 35.8 |
| 548 | 277.2279 / 17.85 | 277.2288 | Pinolenic acid (NIST) [Smart Confirmation] | 92.3 |
| 550 | 590.4449 / 17.85 | 590.4452 | Propanoic acid, 2-1-3-4-(1,1'-biphenyl-4-ylcarbonyl)-2-propylphenoxy propyl-1,2,3,4-tetrahydro-5-quinolinyloxy-2-methyl- (NIST) [Smart Confirmation] | 34.3 |
| 554 | 831.4934 / 18.02 | 831.4939 | B-Pentasaccharide (NIST) [Smart Confirmation] | 79.6 |
| 556 | 253.2232 / 18.13 [M-H2O-H]- | 253.2239 | cis-7-Hexadecenoic acid (NIST) [Smart Confirmation] | 97.5 |
| 557 | 271.2331 / 17.96 [M-H]- | 271.2342 | 16-Hydroxyhexadecanoic acid (NIST) [Smart Confirmation] | 81.4 |
| 560 | 279.2401 / 18.30 | 279.2408 | 亚油酸 Linoleic acid [Smart Confirmation] | 100.0 |
| 565 | 255.2390 / 18.58 | 255.2414 | 2,2',4'-Trihydroxychalcone (NIST) [Smart Confirmation] | 64.2 |
| 572 | 311.2933 / 18.81 | 311.2939 | Benzenesulfonic acid, 4-undecyl- (NIST) [Smart Confirmation] | 71.7 |
| 575 | 281.2487 / 19.15 | 281.2504 | 1,4-D-Xylobiose (NIST) [Smart Confirmation] | 96.6 |
| 576 | 349.2733 / 19.15 | 349.2732 | 9,11-Dideoxy-9.alpha.,11.alpha.-epoxymethanoprosta-5E,13E-dien-1-oic acid (NIST) [Smart Confirmation] | 70.7 |
| 580 | 609.3920 / 19.27 | 609.3925 | 1,2-Dilauroyl-sn-glycero-3-phospho-sn-glycerol (NIST) [Smart Confirmation] | 83.6 |
| 590 | 255.2372 / 19.38 | 255.2355 | 2,2',4'-Trihydroxychalcone (NIST) [Smart Confirmation] | 69.1 |

End of Table

## 128.0342 / 1.02 (Mass/FragMass/RT/Isotope/Library/Formula/Ion Ratio)

**Retention Time:** 1.02 minutes
**Precursor m/z :** 128.0342
**Fit (%)** 100.0% **RFit (%)** 100.0%

**Exp RT:** 1.02 minutes
**Analyte Name:**
128.0342 / 1.02

**Collision Energy = 35 ± 15 eV**

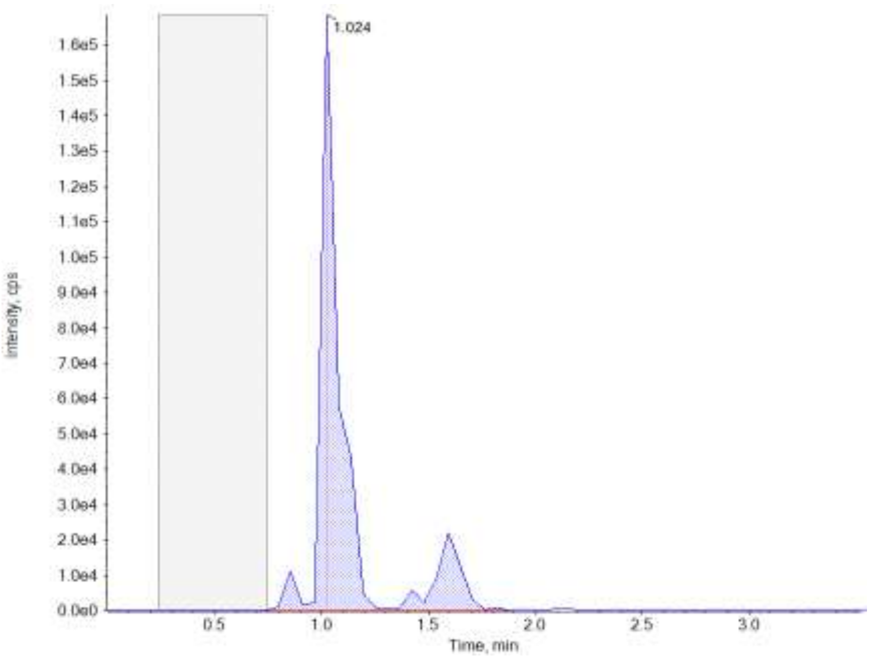


Acquired / Library MSMS

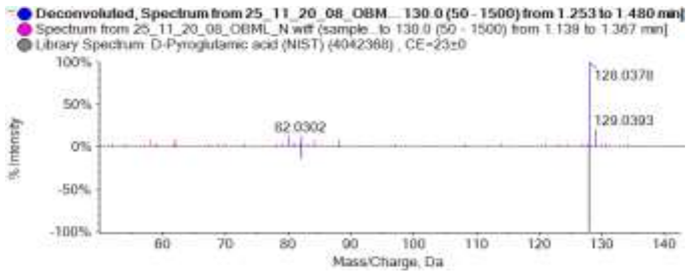


Acquired / Theoretical MS

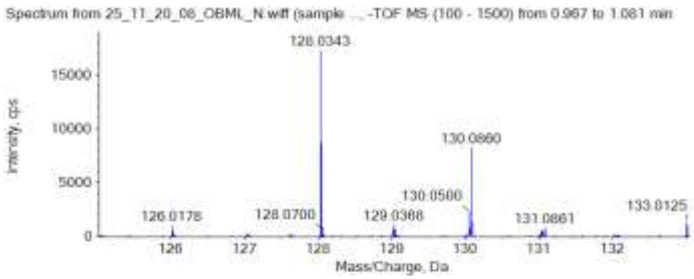

**116.0715 / 1.19** (Mass/FragMass/RT/Isotope/Library/Formula/Ion Ratio)

**Retention Time:** 1.24 minutes
**Precursor m/z :** 116.0715
**Fit (%)** 52.8% **RFit (%)** 99.5%

**Exp RT:** 1.20 minutes
**Analyte Name:**
116.0715 / 1.19

**Collision Energy = 35 ± 15 eV**

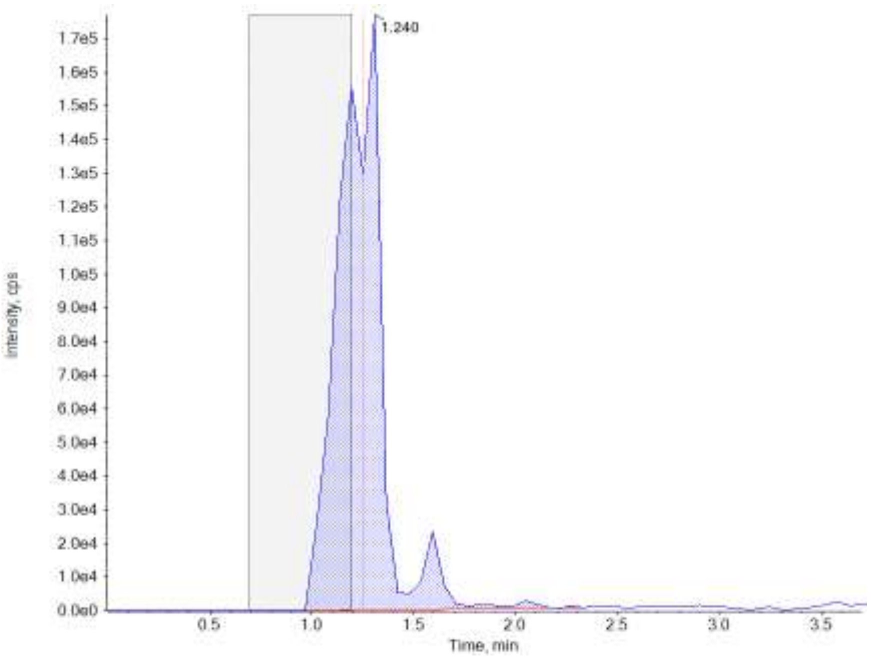


**Acquired / Library MSMS**

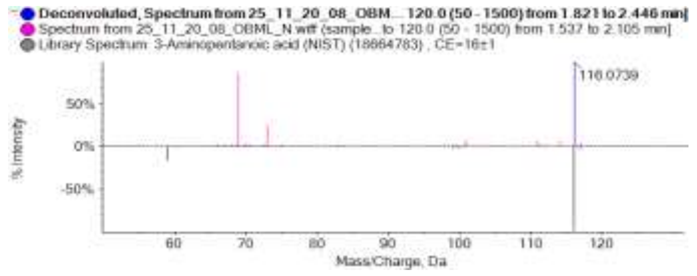


**Acquired / Theoretical MS**

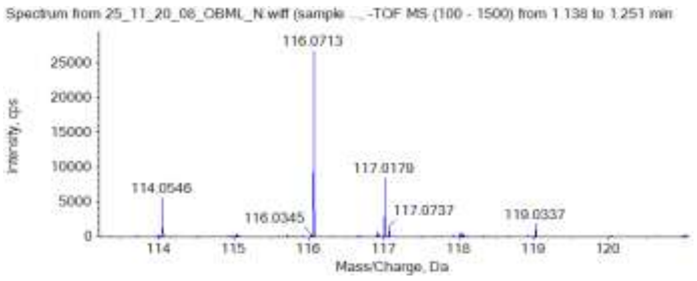

## 179.0551 / 1.19 (Mass/FragMass/RT/Isotope/Library/Formula/Ion Ratio)

**Retention Time:** 1.17 minutes
**Precursor m/z :** 179.0551
**Fit (%)** 98.3% **RFit (%)** 99.4%

**Exp RT:** 1.20 minutes
**Analyte Name:**
179.0551 / 1.19

**Collision Energy = 35 ± 15 eV**

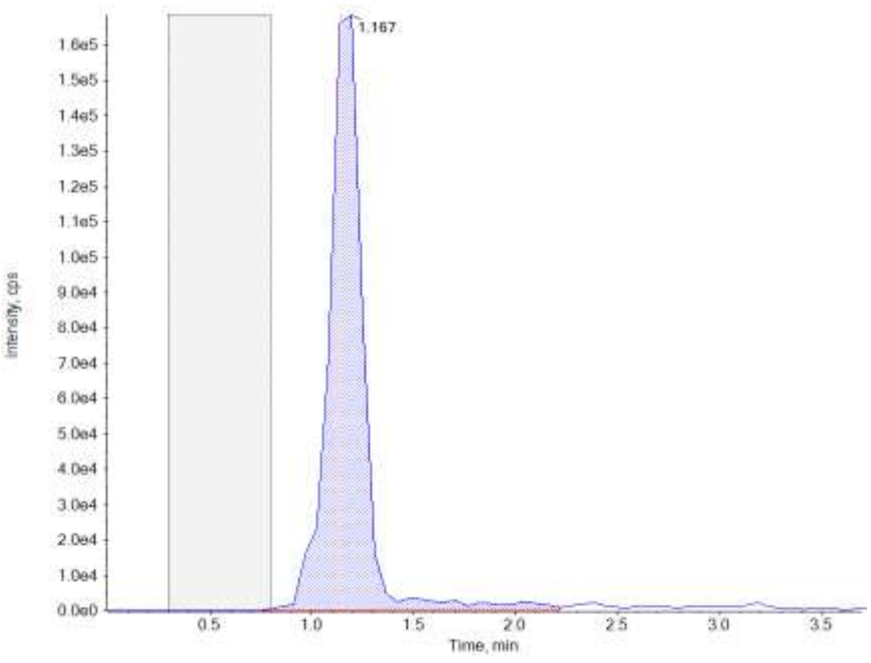


**Acquired / Library MSMS**

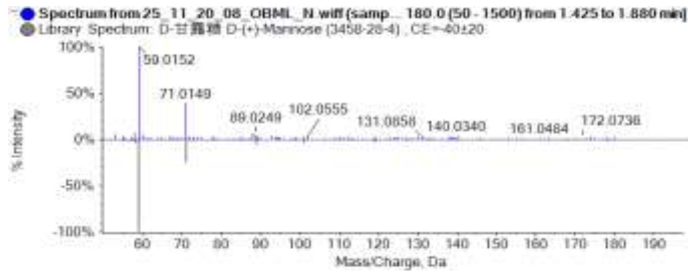


**Acquired / Theoretical MS**

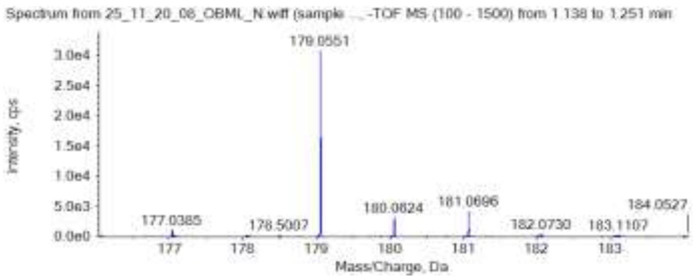

## 341.1085 / 1.19 [M-H]- (Mass/FragMass/RT/Isotope/Library/Formula/Ion Ratio)

| | |
|---|---|
| **Retention Time:** 1.20 minutes<br>**Precursor m/z :** 341.1085<br>**Fit (%)** 96.2% **RFit (%)** 81.8% | **Exp RT:** 1.20 minutes<br>**Analyte Name:**<br>341.1085 / 1.19 [M-H]- |

| | | **Collision Energy = 35 ± 15 eV** |
|---|---|---|
| 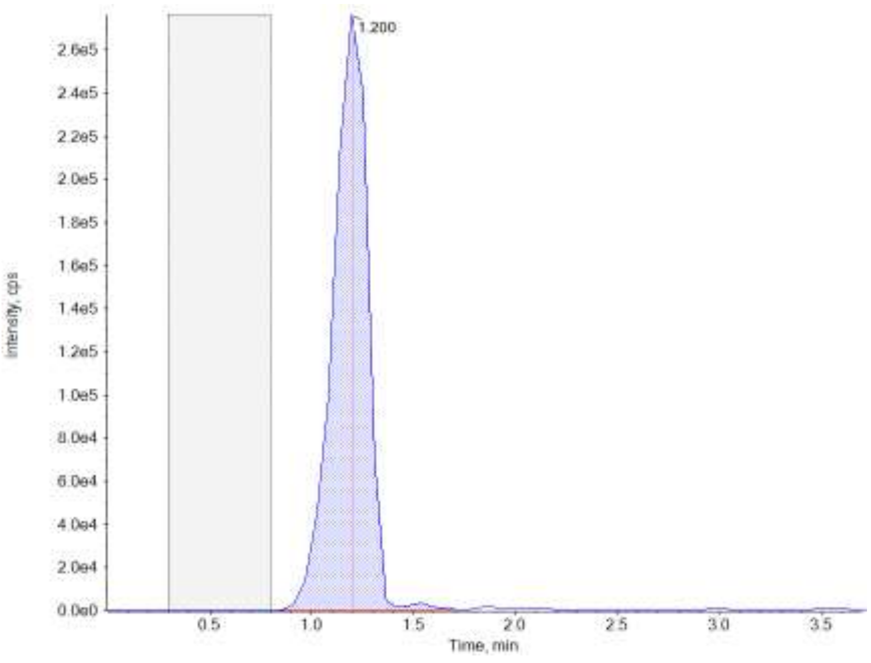 | **Acquired / Library MSMS** | 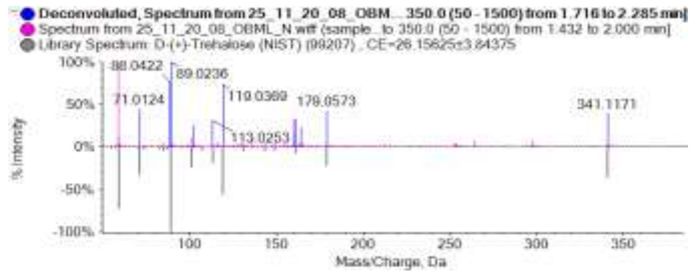 |
| | **Acquired / Theoretical MS** | 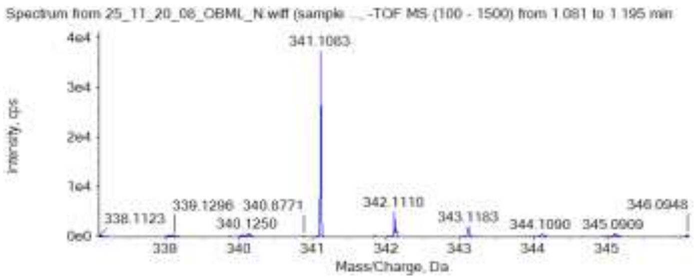 |

## 111.0192 / 1.37 (Mass/FragMass/RT/Isotope/Library/Formula/Ion Ratio)

**Retention Time:** 1.37 minutes
**Precursor m/z :** 111.0192
**Fit (%)** 100.0% **RFit (%)** 39.0%

**Exp RT:** 1.37 minutes
**Analyte Name:**
111.0192 / 1.37

**Collision Energy = 35 ± 15 eV**

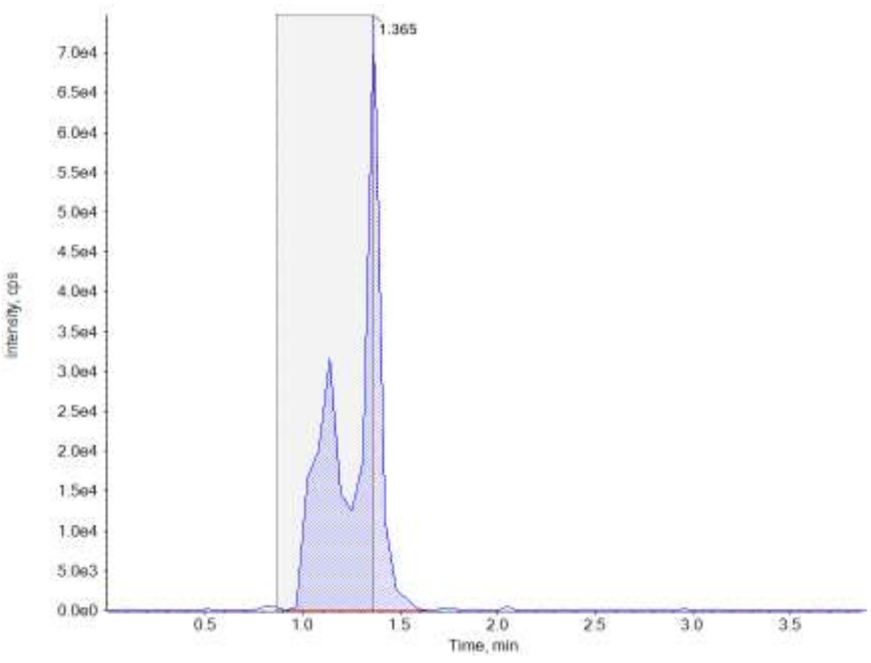


**Acquired / Library MSMS**

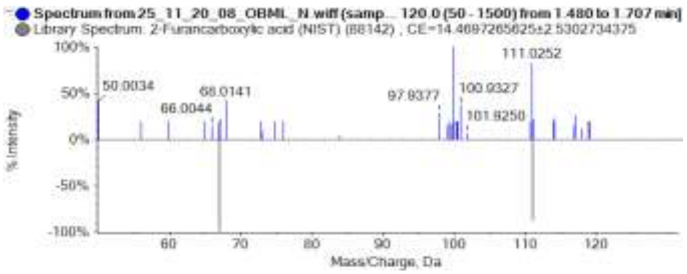


**Acquired / Theoretical MS**

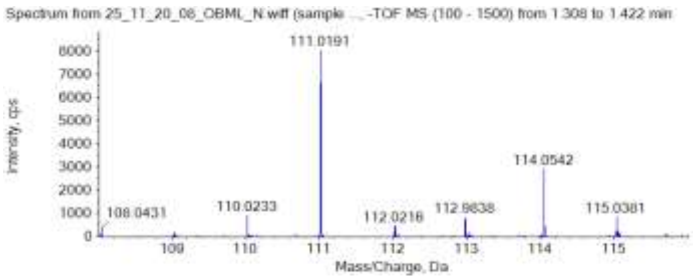

## 180.0652 / 1.37 (Mass/FragMass/RT/Isotope/Library/Formula/Ion Ratio)

| | |
|---|---|
| **Retention Time:** 1.39 minutes | **Exp RT:** 1.37 minutes |
| **Precursor m/z :** 180.0652 | **Analyte Name:** |
| **Fit (%)** 91.7% **RFit (%)** 100.0% | 180.0652 / 1.37 |

**Collision Energy = 35 ± 15 eV**

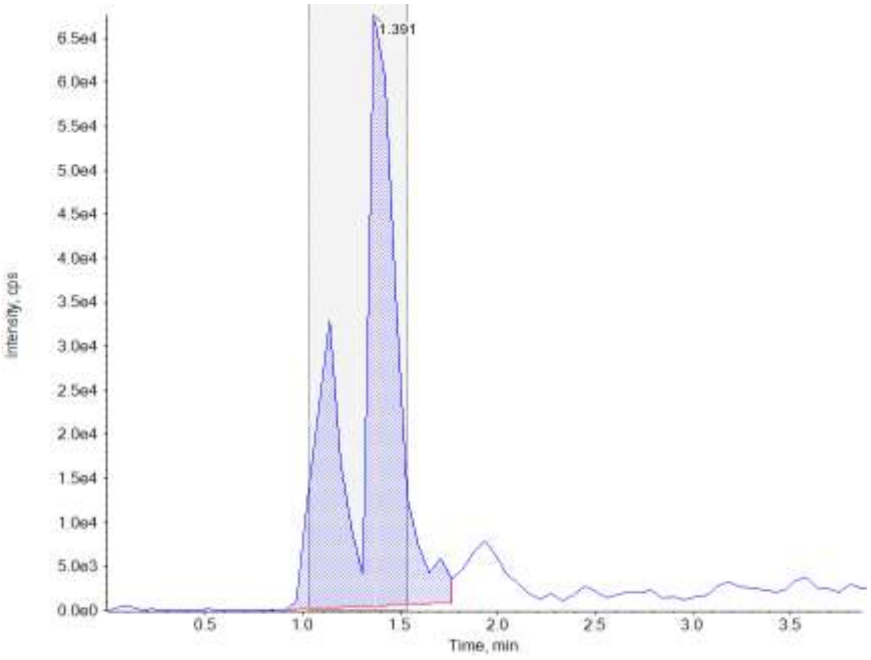


**Acquired / Library MSMS**

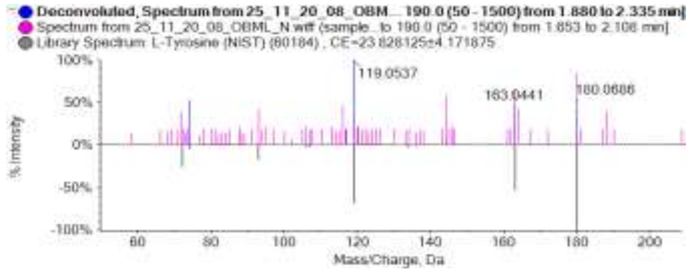


**Acquired / Theoretical MS**

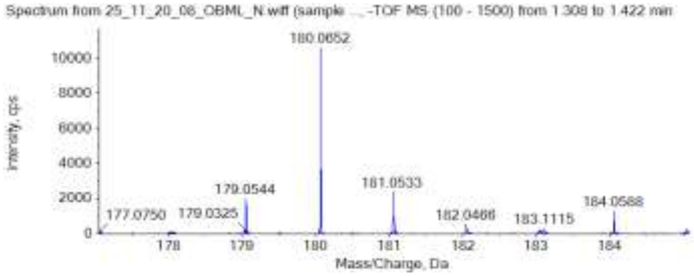

**243.0683 / 1.42** (Mass/FragMass/RT/Isotope/Library/Formula/Ion Ratio)

**Retention Time:** 1.41 minutes **Exp RT:** 1.42 minutes
**Precursor m/z :** 243.0684 **Analyte Name:**
**Fit (%)** 95.1% **RFit (%)** 97.9% 243.0683 / 1.42

**Collision Energy = 35 ± 15 eV**

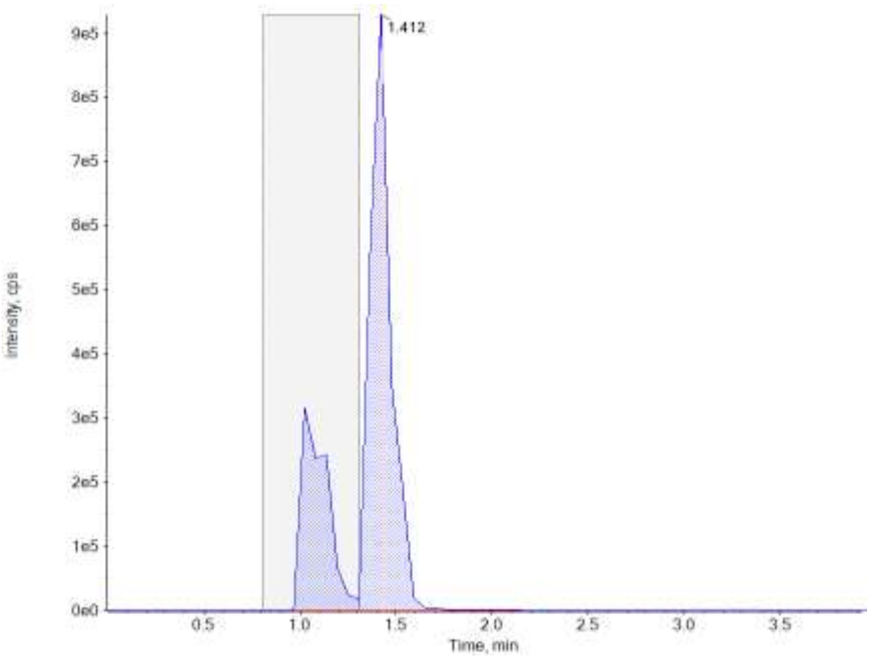


**Acquired / Library MSMS**

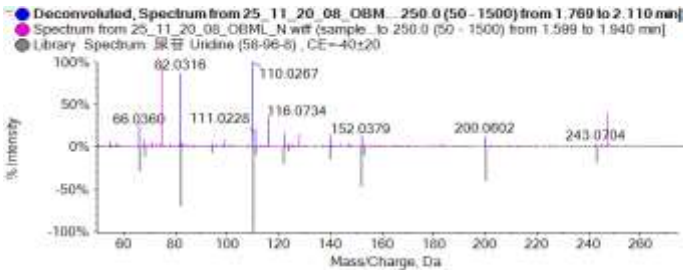


**Acquired / Theoretical MS**

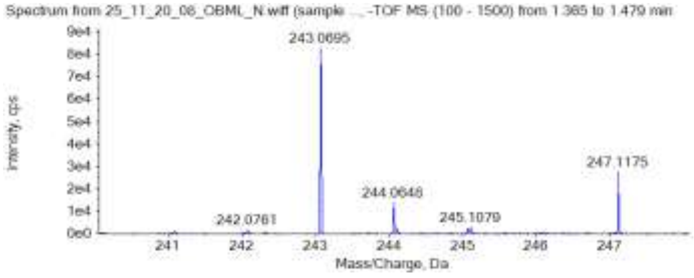

**130.0881 / 1.48** (Mass/FragMass/RT/Isotope/Library/Formula/Ion Ratio)

| | |
|---|---|
| **Retention Time:** 1.45 minutes<br>**Precursor m/z :** 130.0881<br>**Fit (%)** 100.0% **RFit (%)** 100.0% | **Exp RT:** 1.48 minutes<br>**Analyte Name:**<br>130.0881 / 1.48 |

| | | **Collision Energy = 35 ± 15 eV** |
|---|---|---|
| 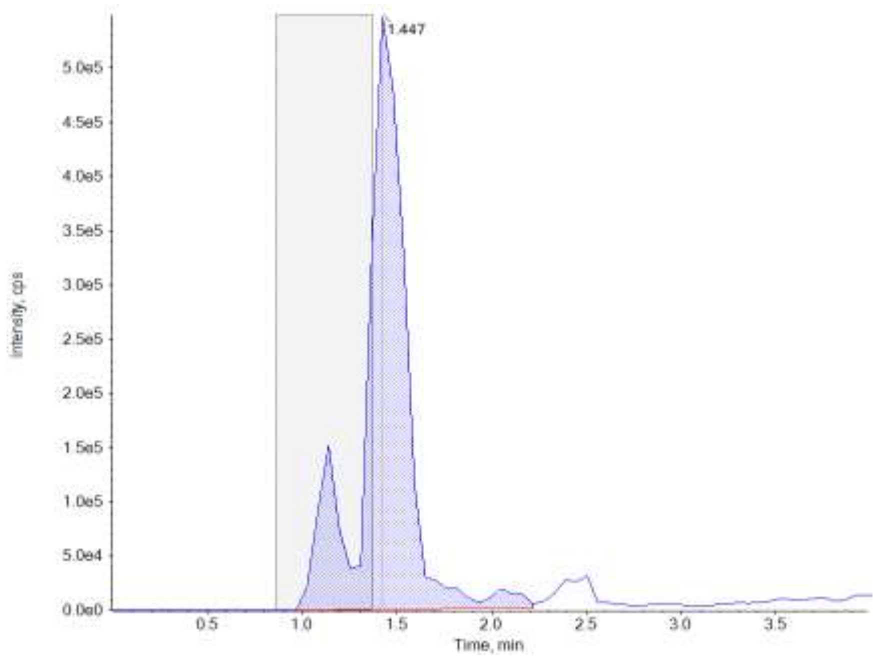 | **Acquired / Library MSMS** | 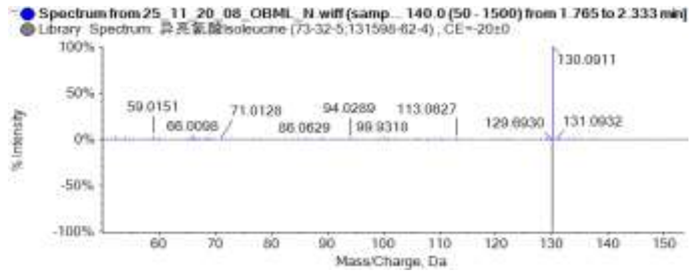 |
| | **Acquired / Theoretical MS** | 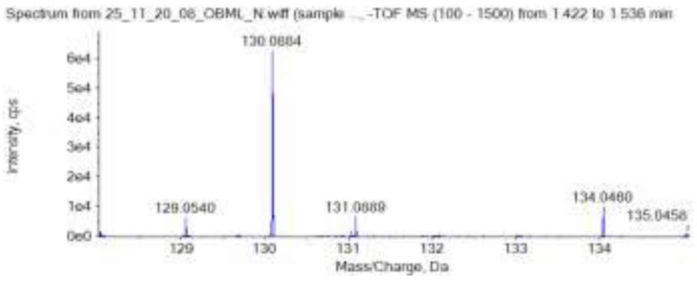 |

## 202.1070 / 1.48

(Mass/FragMass/RT/Isotope/Library/Formula/Ion Ratio)

**Retention Time:** 1.45 minutes
**Precursor m/z :** 202.1070
**Fit (%)** 54.8%   **RFit (%)** 95.7%

**Exp RT:** 1.48 minutes
**Analyte Name:**
202.1070 / 1.48

**Collision Energy = 35 ± 15 eV**

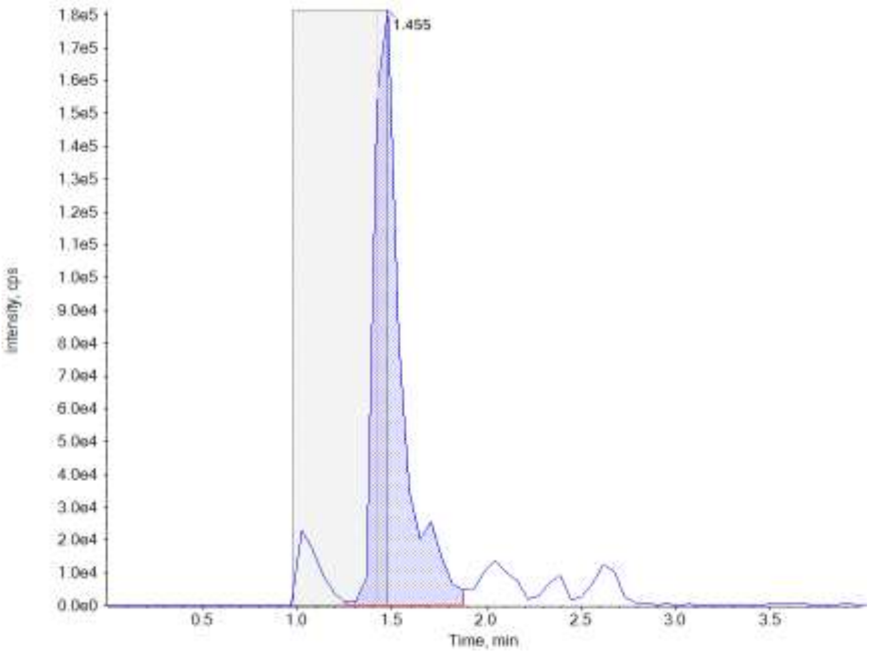


**Acquired / Library MSMS**

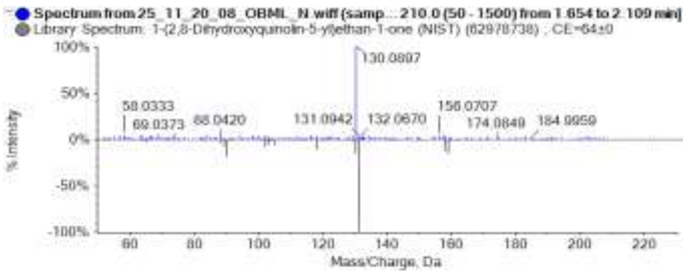


**Acquired / Theoretical MS**

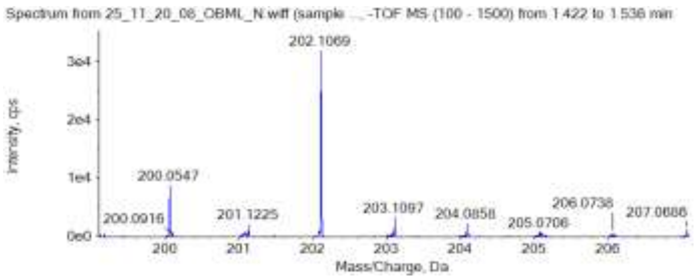

## 247.1173 / 1.48 [M-H]- (Mass/FragMass/RT/Isotope/Library/Formula/Ion Ratio)

| **Retention Time:** 1.48 minutes | **Exp RT:** 1.48 minutes |
|---|---|
| **Precursor m/z :** 247.1173 | **Analyte Name:** |
| **Fit (%)** 98.1% **RFit (%)** 98.7% | 247.1173 / 1.48 [M-H]- |

**Collision Energy = 35 ± 15 eV**

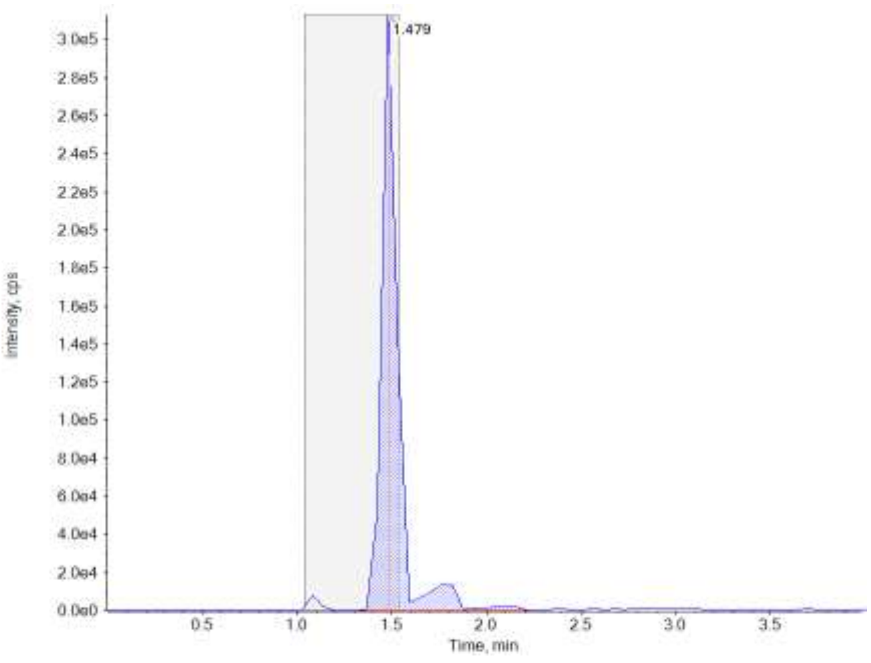


**Acquired / Library MSMS**

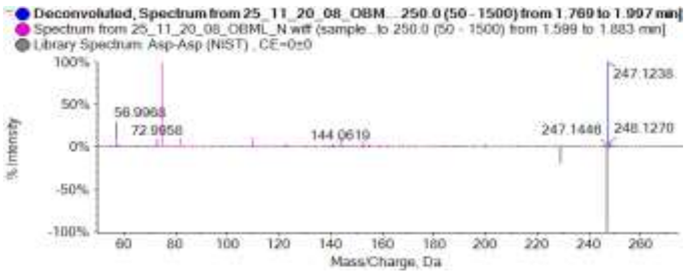


**Acquired / Theoretical MS**

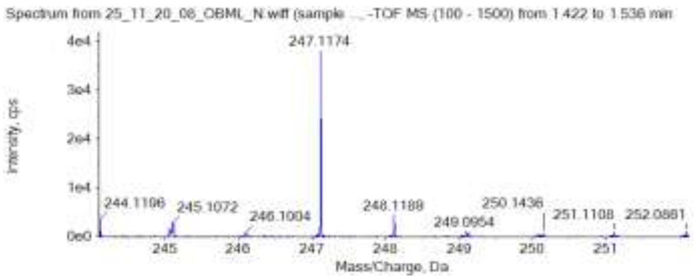

**282.0858 / 1.54** (Mass/FragMass/RT/Isotope/Library/Formula/Ion Ratio)

**Retention Time:** 1.54 minutes
**Precursor m/z :** 282.0858
**Fit (%)** 100.0% **RFit (%)** 100.0%

**Exp RT:** 1.54 minutes
**Analyte Name:**
282.0858 / 1.54

**Collision Energy = 35 ± 15 eV**

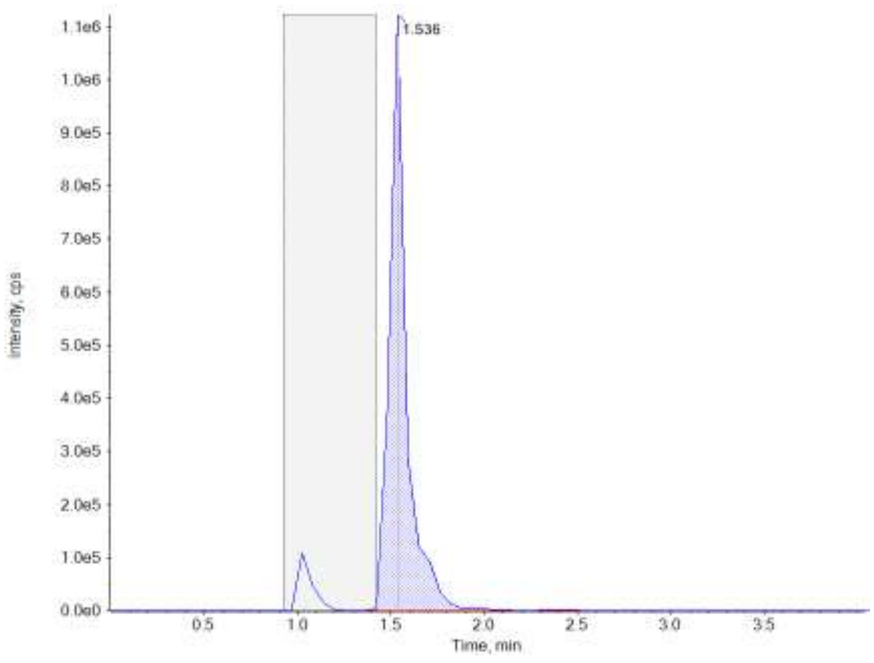


**Acquired / Library MSMS**

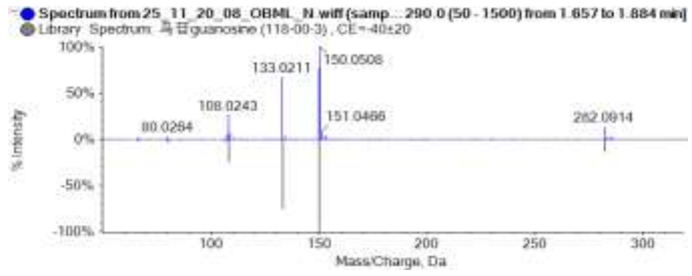


**Acquired / Theoretical MS**

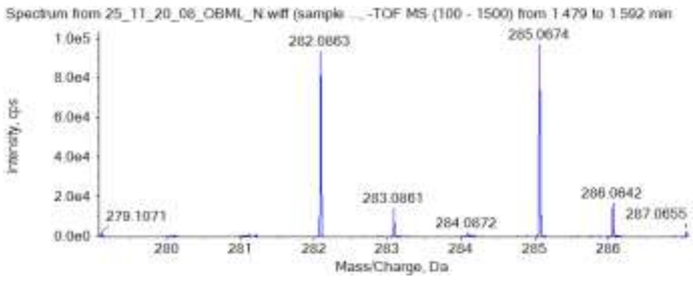

## 137.0240 / 1.59 (Mass/FragMass/RT/Isotope/Library/Formula/Ion Ratio)

| | |
|---|---|
| **Retention Time:** 1.59 minutes | **Exp RT:** 1.59 minutes |
| **Precursor m/z :** 137.0240 | **Analyte Name:** |
| **Fit (%)** 99.2% **RFit (%)** 100.0% | 137.0240 / 1.59 |

**Collision Energy = 35 ± 15 eV**

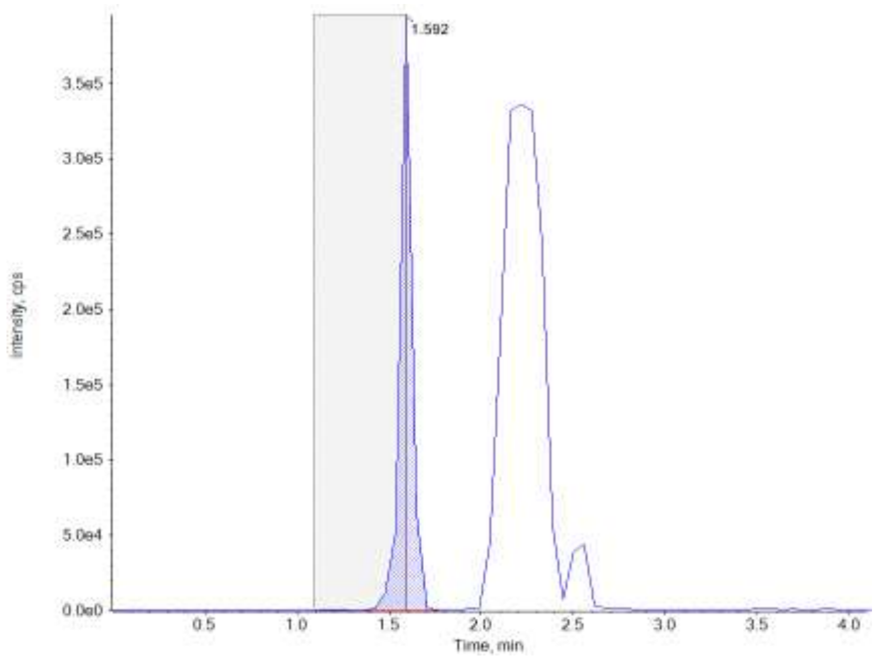


**Acquired / Library MSMS**

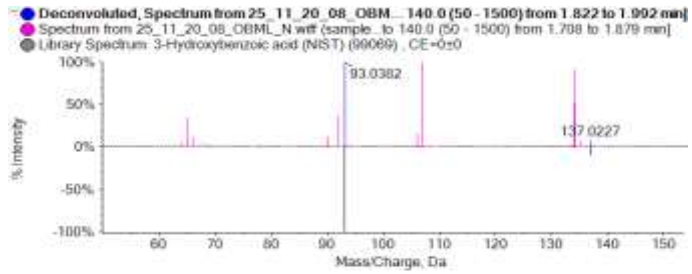


**Acquired / Theoretical MS**

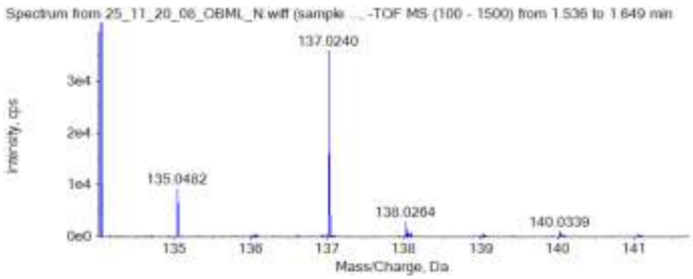

## 299.0821 / 1.59 (Mass/FragMass/RT/Isotope/Library/Formula/Ion Ratio)

**Retention Time:** 1.59 minutes
**Precursor m/z :** 299.0821
**Fit (%)** 25.9% **RFit (%)** 100.0%

**Exp RT:** 1.59 minutes
**Analyte Name:**
299.0821 / 1.59

**Collision Energy = 35 ± 15 eV**

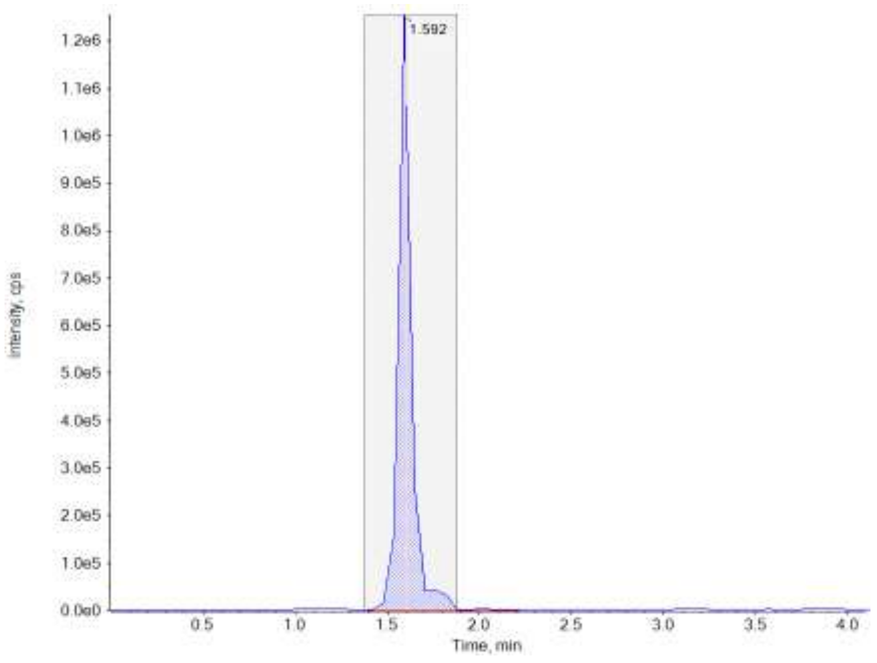


Acquired / Library MSMS

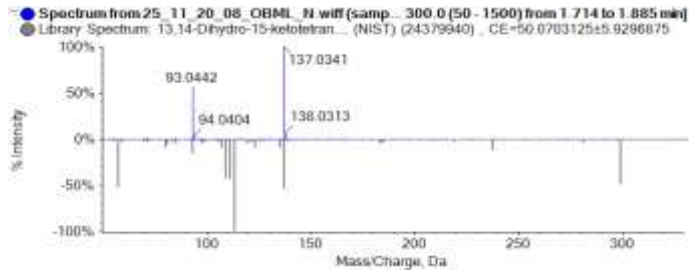


Acquired / Theoretical MS

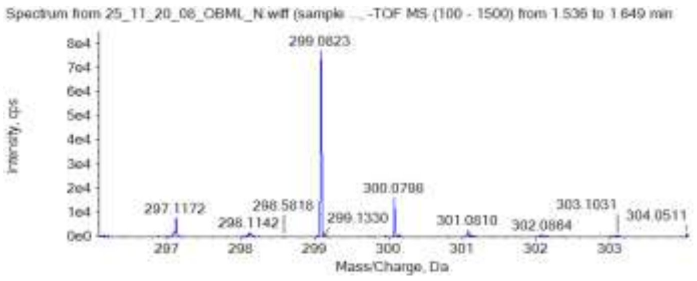

## 134.0519 / 1.71 (Mass/FragMass/RT/Isotope/Library/Formula/Ion Ratio)

| | |
|---|---|
| **Retention Time:** 1.71 minutes | **Exp RT:** 1.71 minutes |
| **Precursor m/z :** 134.0519 | **Analyte Name:** |
| **Fit (%)** 99.2% **RFit (%)** 100.0% | 134.0519 / 1.71 |

**Collision Energy = 35 ± 15 eV**

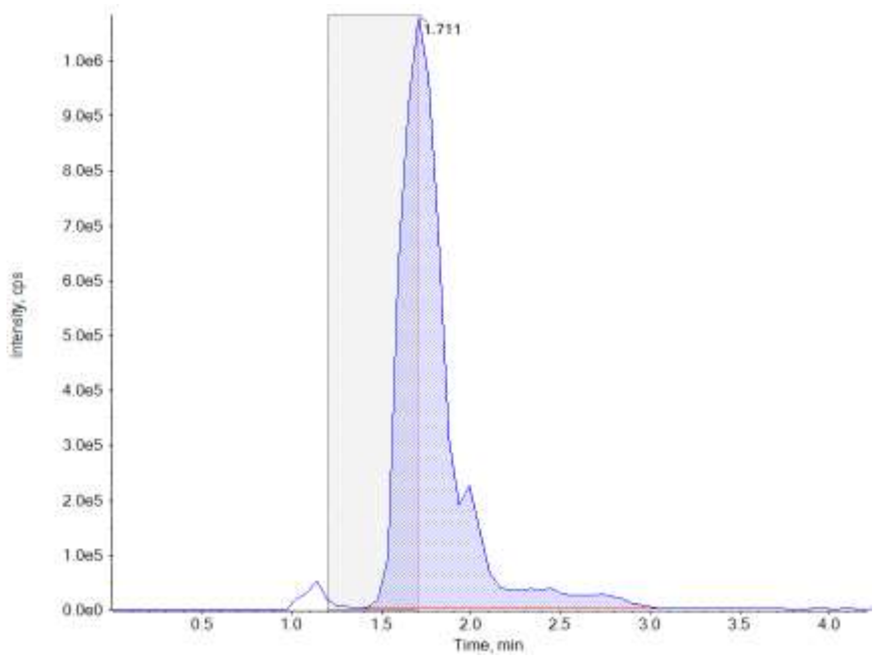


**Acquired / Library MSMS**

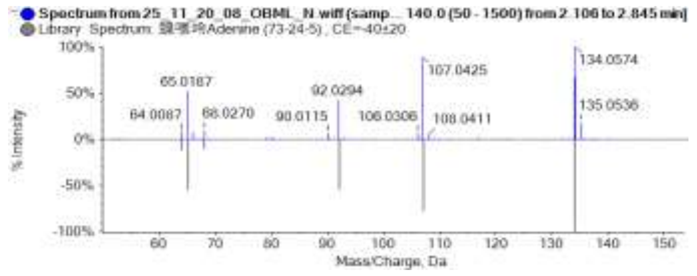


**Acquired / Theoretical MS**

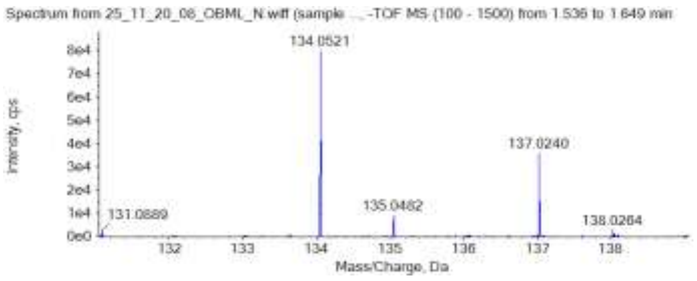

**337.1493 / 1.71** (Mass/FragMass/RT/Isotope/Library/Formula/Ion Ratio)

| | |
|---|---|
| **Retention Time:** 1.69 minutes | **Exp RT:** 1.71 minutes |
| **Precursor m/z :** 337.1493 | **Analyte Name:** |
| **Fit (%)** 99.8% **RFit (%)** 26.0% | 337.1493 / 1.71 |

**Collision Energy = 35 ± 15 eV**

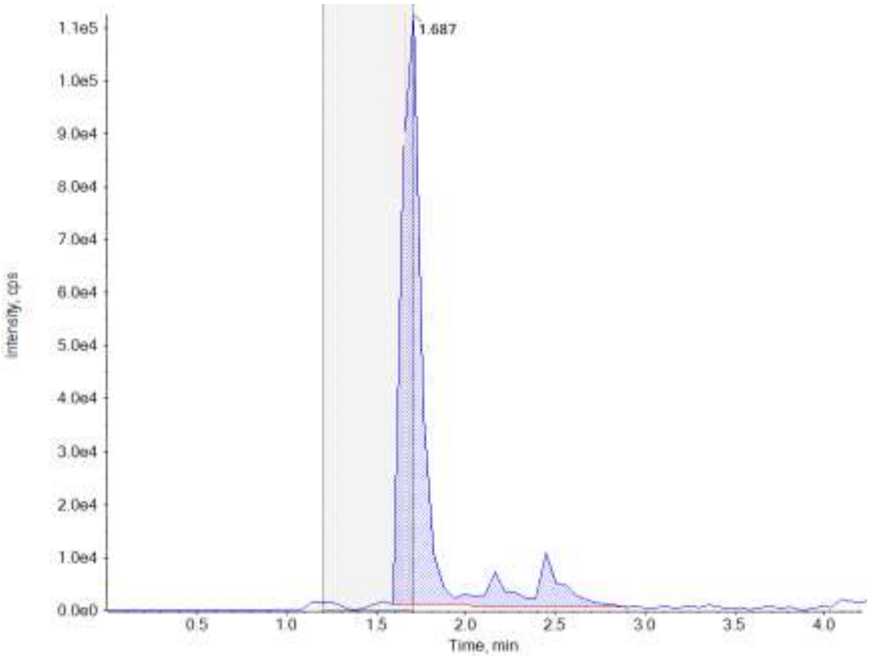


**Acquired / Library MSMS**

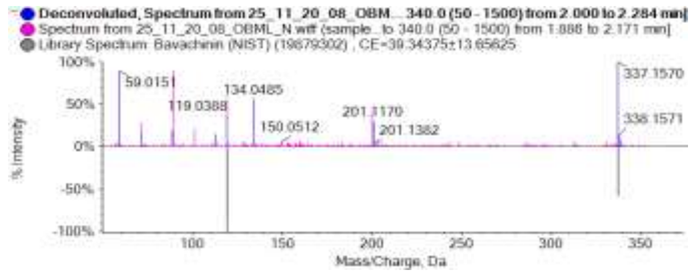


**Acquired / Theoretical MS**

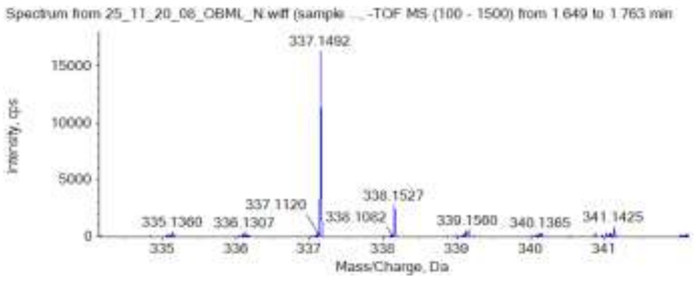

## 175.0981 / 1.82 (Mass/FragMass/RT/Isotope/Library/Formula/Ion Ratio)

| | |
|---|---|
| **Retention Time:** 1.83 minutes | **Exp RT:** 1.82 minutes |
| **Precursor m/z :** 175.0981 | **Analyte Name:** |
| **Fit (%)** 82.6% **RFit (%)** 93.3% | 175.0981 / 1.82 |

**Collision Energy = 35 ± 15 eV**

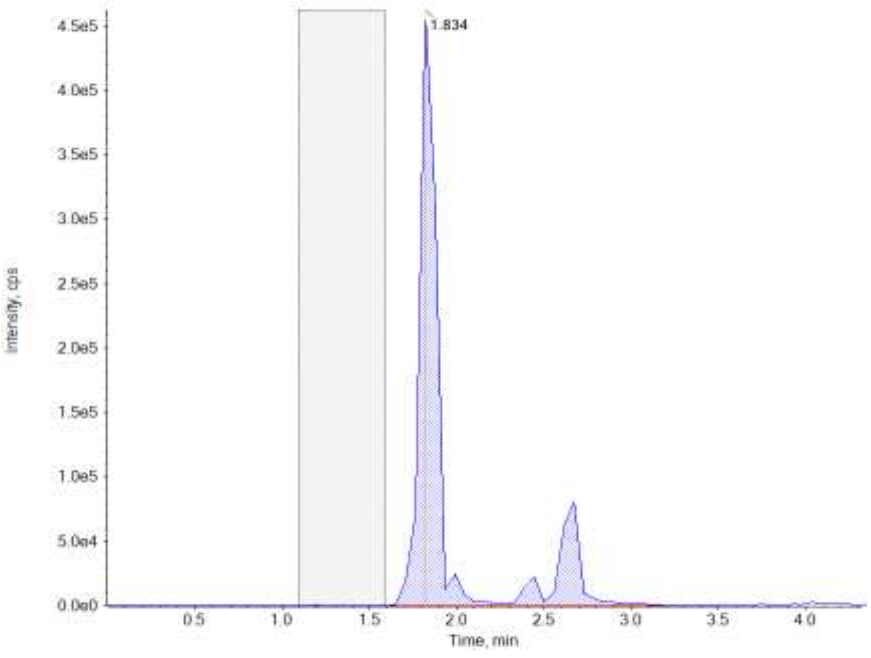


**Acquired / Library MSMS**

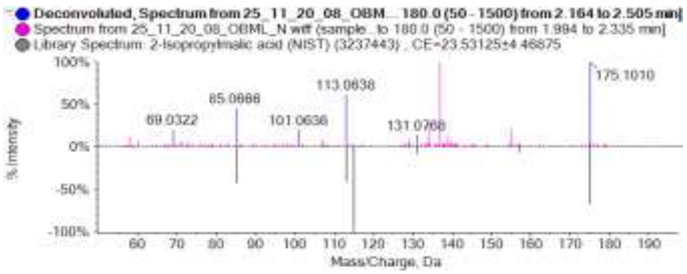


**Acquired / Theoretical MS**

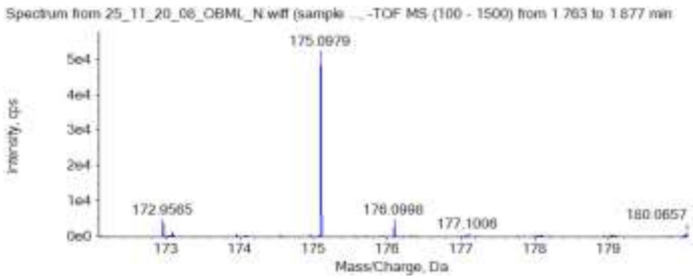

**257.0773 / 1.82** (Mass/FragMass/RT/Isotope/Library/Formula/Ion Ratio)

**Retention Time:** 1.82 minutes
**Precursor m/z :** 257.0773
**Fit (%)** 66.5% **RFit (%)** 98.8%

**Exp RT:** 1.82 minutes
**Analyte Name:**
257.0773 / 1.82

**Collision Energy = 35 ± 15 eV**

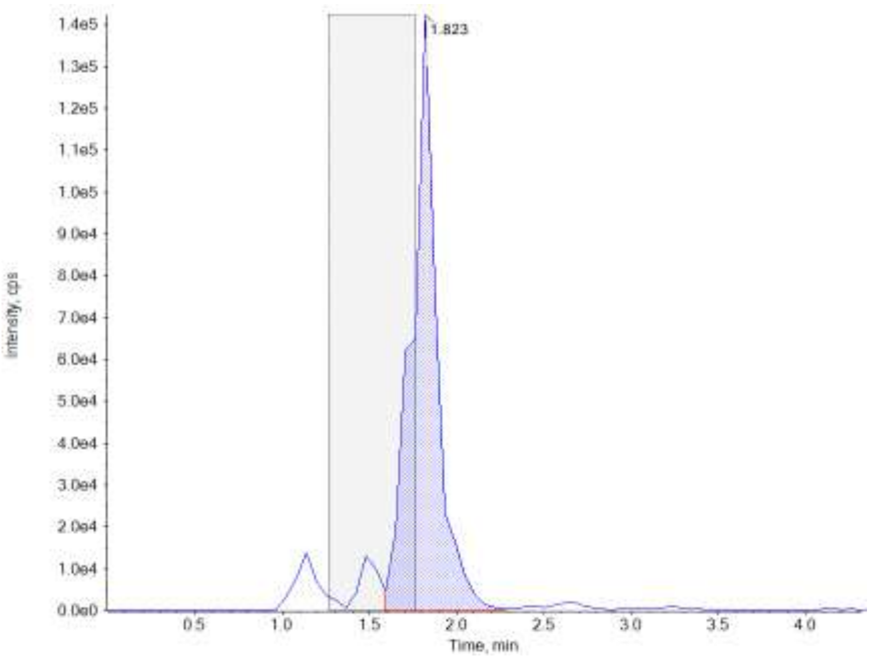


**Acquired / Library MSMS**

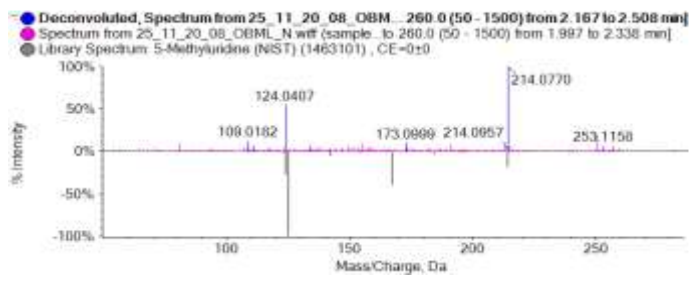


**Acquired / Theoretical MS**

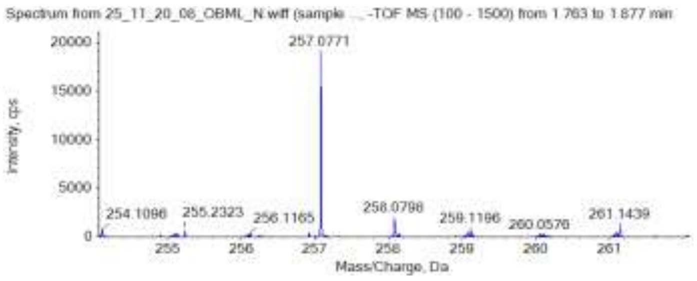

**164.0765 / 1.99** (Mass/FragMass/RT/Isotope/Library/Formula/Ion Ratio)

| | |
|---|---|
| **Retention Time:** 1.96 minutes | **Exp RT:** 1.99 minutes |
| **Precursor m/z :** 164.0765 | **Analyte Name:** |
| **Fit (%)** 100.0% **RFit (%)** 98.6% | 164.0765 / 1.99 |

**Collision Energy = 35 ± 15 eV**

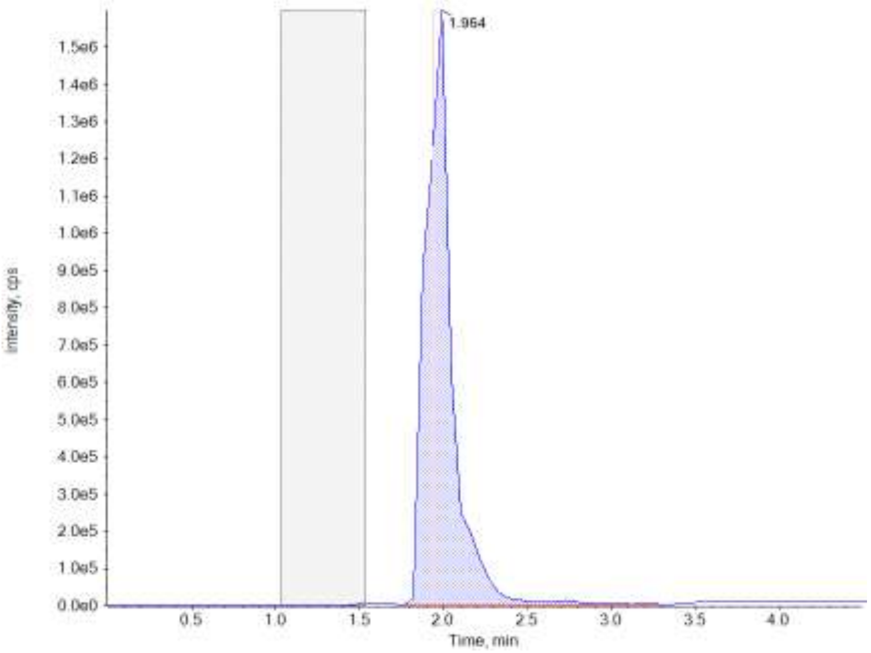


**Acquired / Library MSMS**

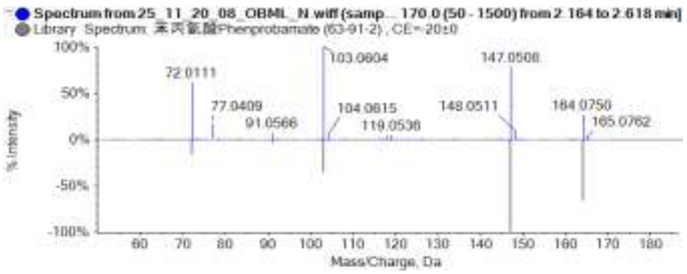


**Acquired / Theoretical MS**

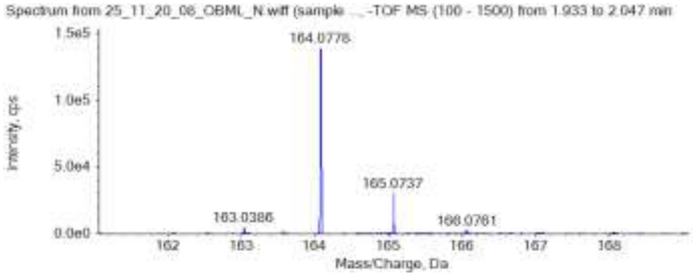

**137.0280 / 2.27** (Mass/FragMass/RT/Isotope/Library/Formula/Ion Ratio)

**Retention Time:** 2.23 minutes
**Precursor m/z :** 137.0280
**Fit (%)** 100.0% **RFit (%)** 100.0%

**Exp RT:** 2.27 minutes
**Analyte Name:**
137.0280 / 2.27

**Collision Energy = 35 ± 15 eV**

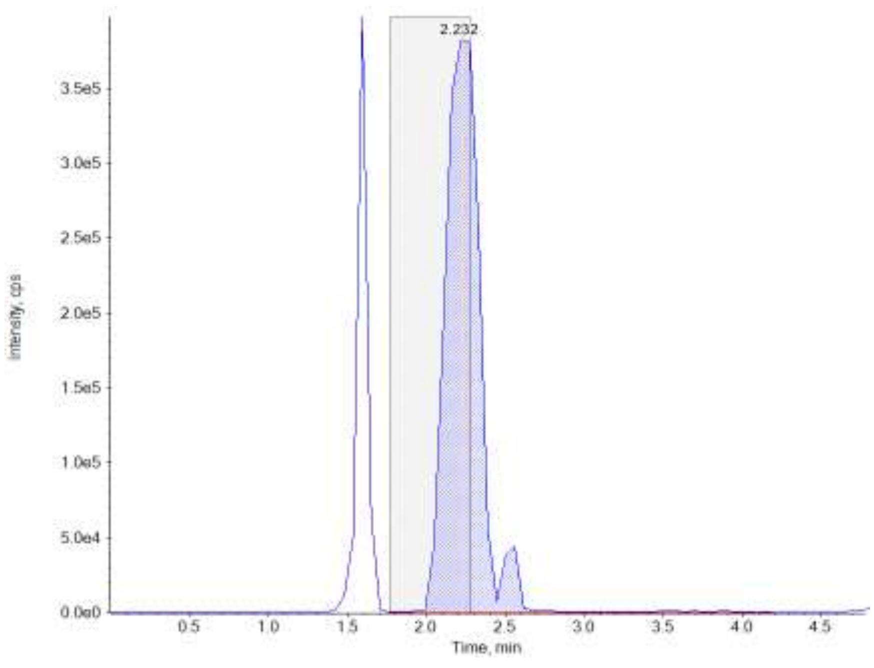


**Acquired / Library MSMS**

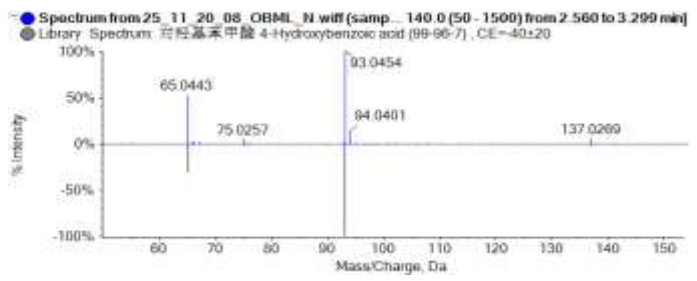


**Acquired / Theoretical MS**

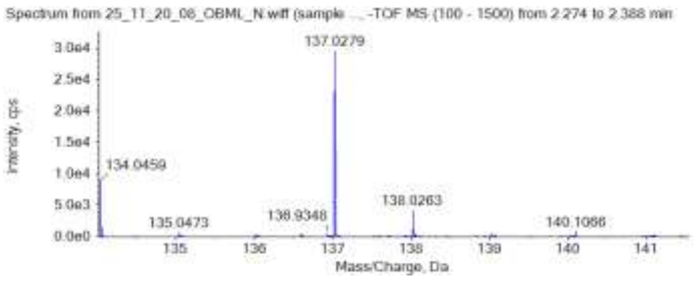

## 192.0656 / 2.33 (Mass/FragMass/RT/Isotope/Library/Formula/Ion Ratio)

**Retention Time:** 2.32 minutes
**Precursor m/z :** 192.0656
**Fit (%)** 97.0% **RFit (%)** 89.5%

**Exp RT:** 2.33 minutes
**Analyte Name:**
192.0656 / 2.33

**Collision Energy = 35 ± 15 eV**

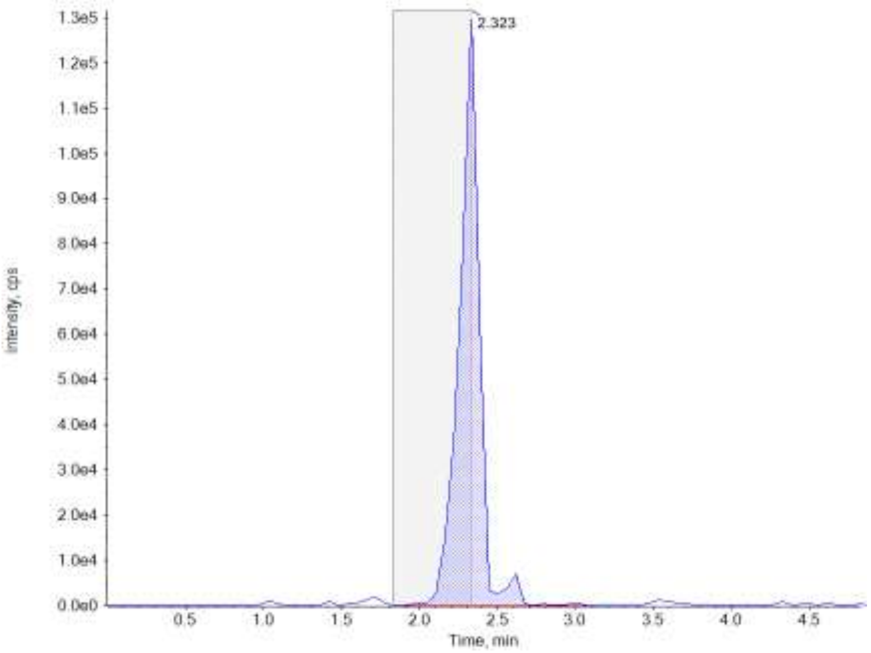


Acquired / Library MSMS

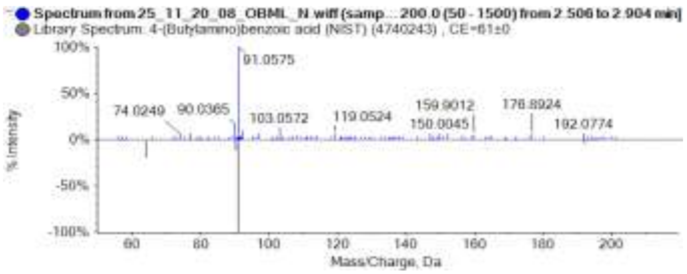


Acquired / Theoretical MS

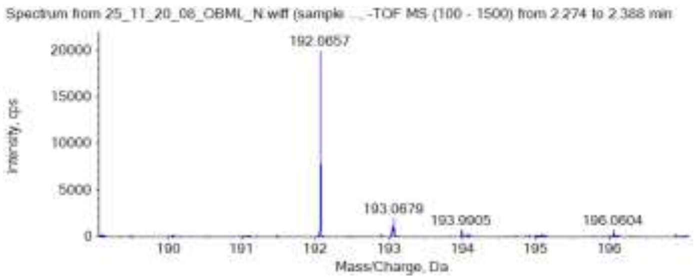

## 172.0968 / 2.39 (Mass/FragMass/RT/Isotope/Library/Formula/Ion Ratio)

**Retention Time:** 2.44 minutes
**Precursor m/z :** 172.0968
**Fit (%)** 100.0% **RFit (%)** 95.7%

**Exp RT:** 2.39 minutes
**Analyte Name:**
172.0968 / 2.39

**Collision Energy = 35 ± 15 eV**

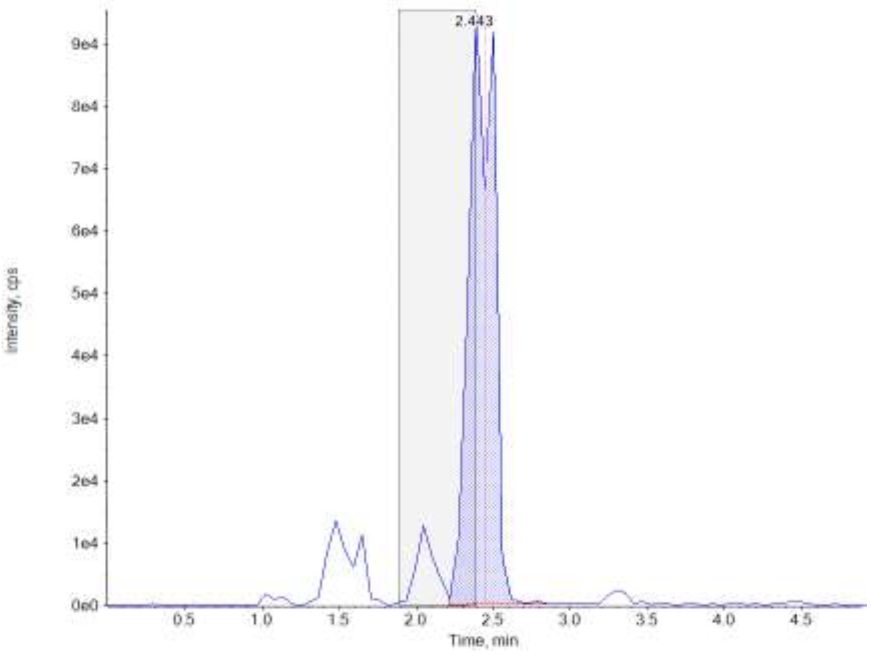


**Acquired / Library MSMS**

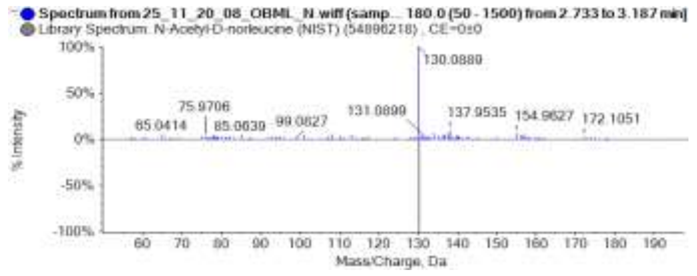


**Acquired / Theoretical MS**

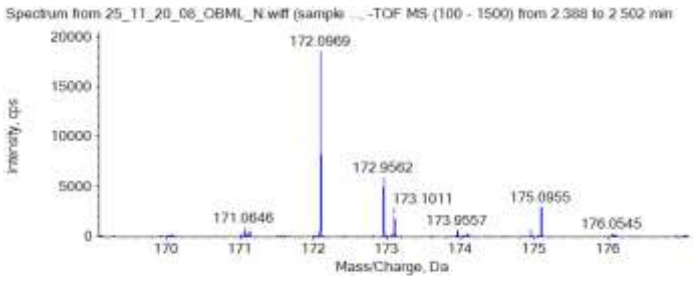

## 138.0552 / 2.44

(Mass/FragMass/RT/Isotope/Library/Formula/Ion Ratio)

**Retention Time:** 2.44 minutes
**Precursor m/z :** 138.0552
**Fit (%)** 100.0% **RFit (%)** 97.1%

**Exp RT:** 2.45 minutes
**Analyte Name:**
138.0552 / 2.44

**Collision Energy = 35 ± 15 eV**

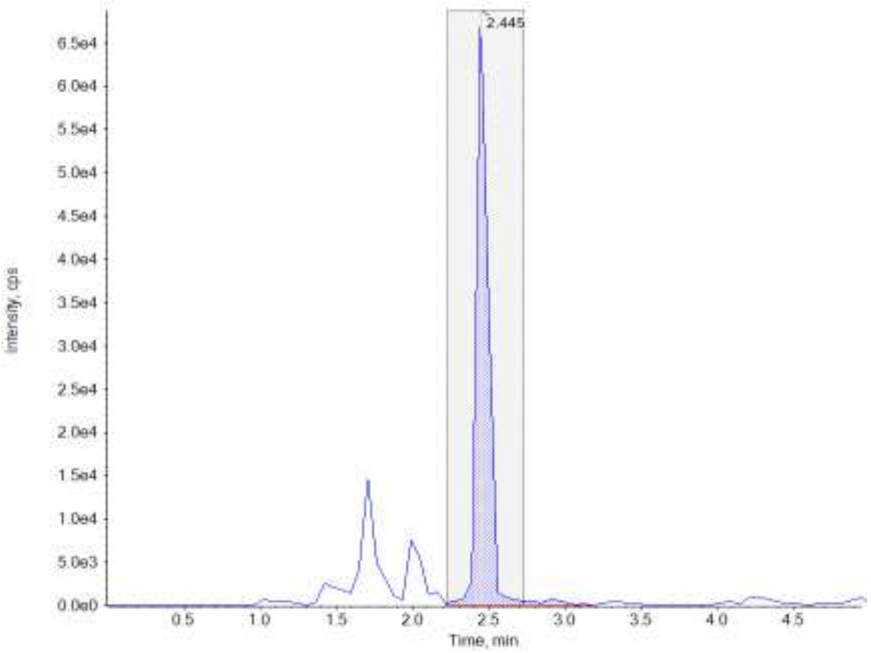


**Acquired / Library MSMS**

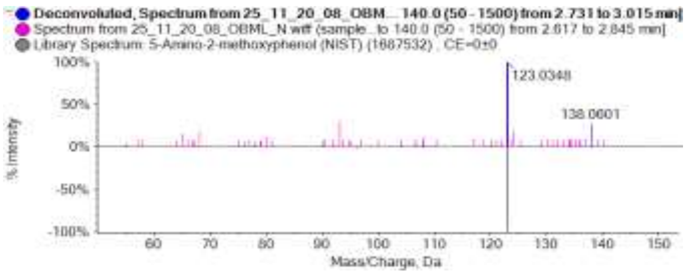


**Acquired / Theoretical MS**

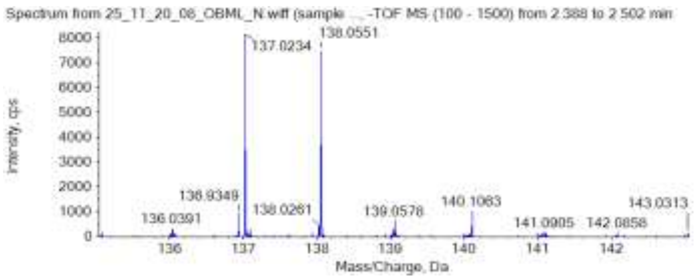

**307.1381 / 2.67** (Mass/FragMass/RT/Isotope/Library/Formula/Ion Ratio)

| | |
|---|---|
| **Retention Time:** 2.69 minutes | **Exp RT:** 2.67 minutes |
| **Precursor m/z :** 307.1381 | **Analyte Name:** |
| **Fit (%)** 54.6% **RFit (%)** 97.5% | 307.1381 / 2.67 |

**Collision Energy = 35 ± 15 eV**

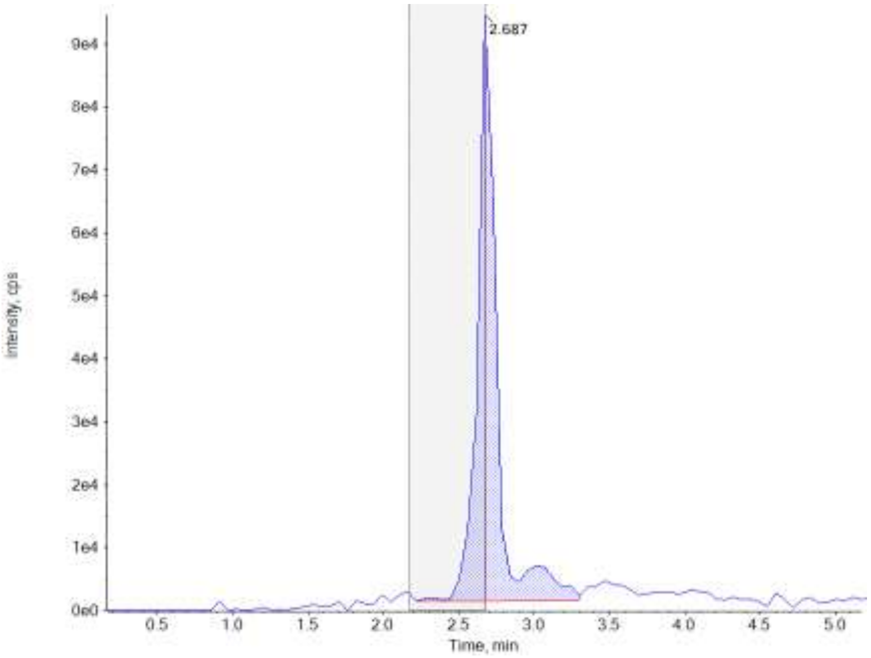


**Acquired / Library MSMS**

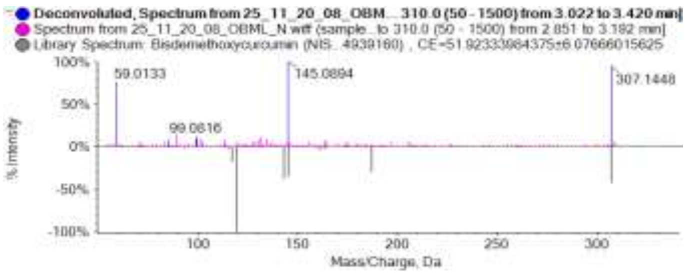


**Acquired / Theoretical MS**

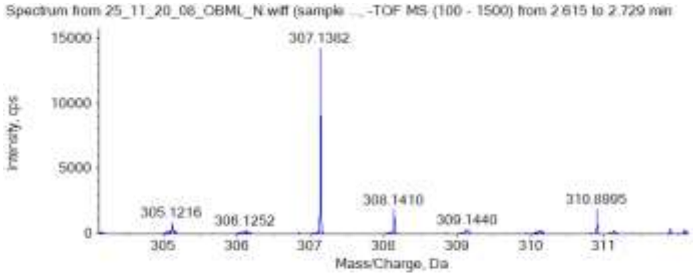

**206.0801 / 2.73** (Mass/FragMass/RT/Isotope/Library/Formula/Ion Ratio)

| | | |
|---|---|---|
| **Retention Time:** 2.72 minutes | **Exp RT:** 2.73 minutes | |
| **Precursor m/z :** 206.0801 | **Analyte Name:** | |
| **Fit (%)** 89.2% **RFit (%)** 84.5% | 206.0801 / 2.73 | |

**Collision Energy = 35 ± 15 eV**

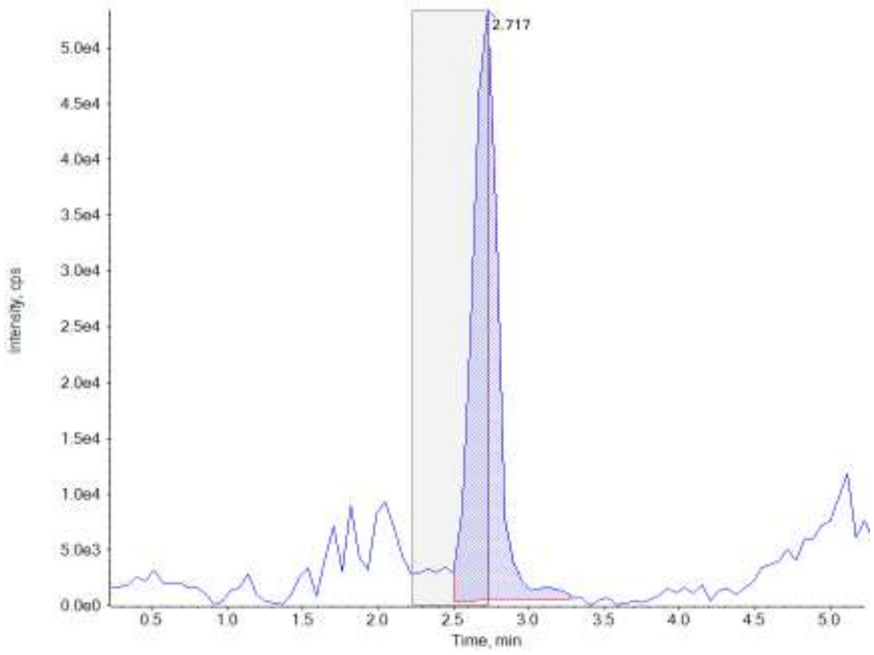


Acquired / Library MSMS

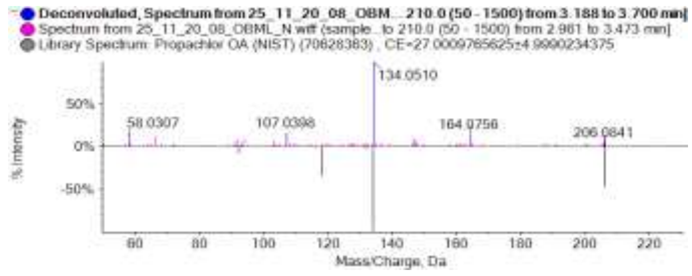


Acquired / Theoretical MS

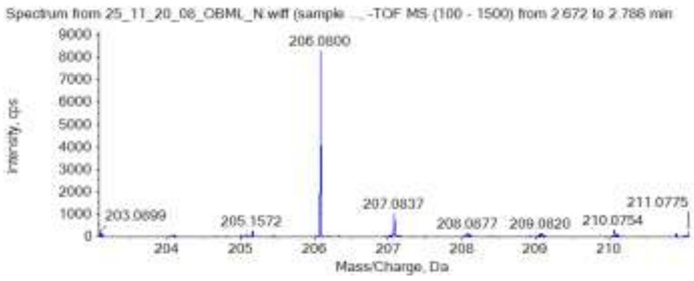

## 327.1067 / 2.73 (Mass/FragMass/RT/Isotope/Library/Formula/Ion Ratio)

| | |
|---|---|
| **Retention Time:** 2.73 minutes | **Exp RT:** 2.73 minutes |
| **Precursor m/z :** 327.1067 | **Analyte Name:** |
| **Fit (%)** 55.3% **RFit (%)** 99.7% | 327.1067 / 2.73 |

**Collision Energy = 35 ± 15 eV**

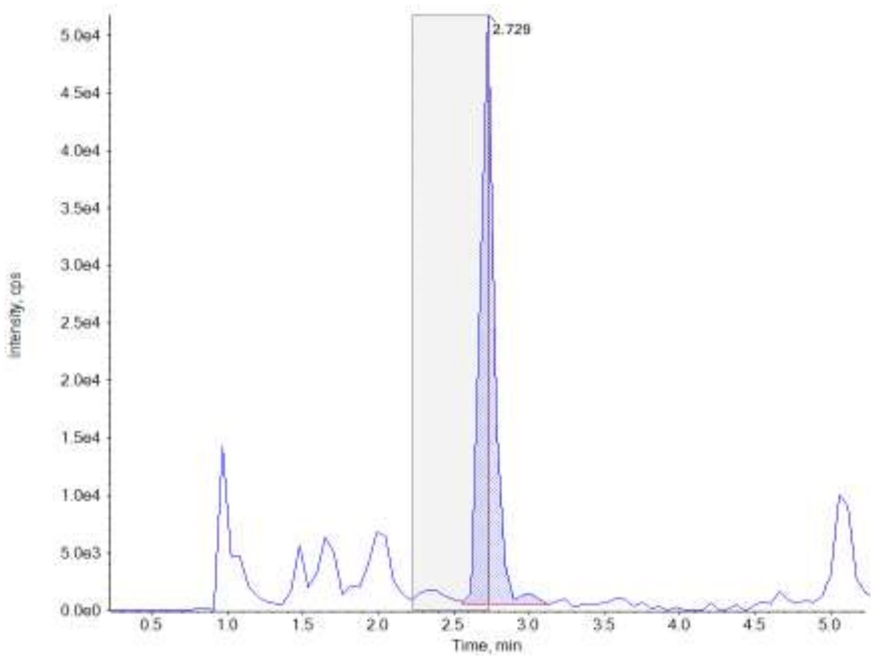


**Acquired / Library MSMS**

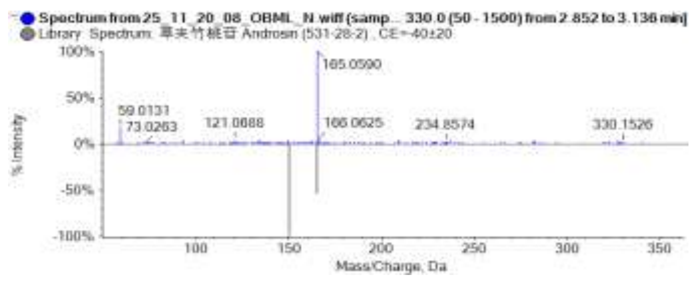


**Acquired / Theoretical MS**

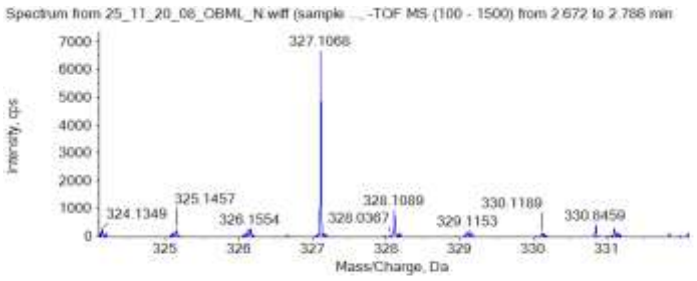

**153.0178 / 2.79** (Mass/FragMass/RT/Isotope/Library/Formula/Ion Ratio)

**Retention Time:** 2.80 minutes
**Precursor m/z :** 153.0178
**Fit (%)** 99.6% **RFit (%)** 94.8%

**Exp RT:** 2.79 minutes
**Analyte Name:**
153.0178 / 2.79

**Collision Energy = 35 ± 15 eV**

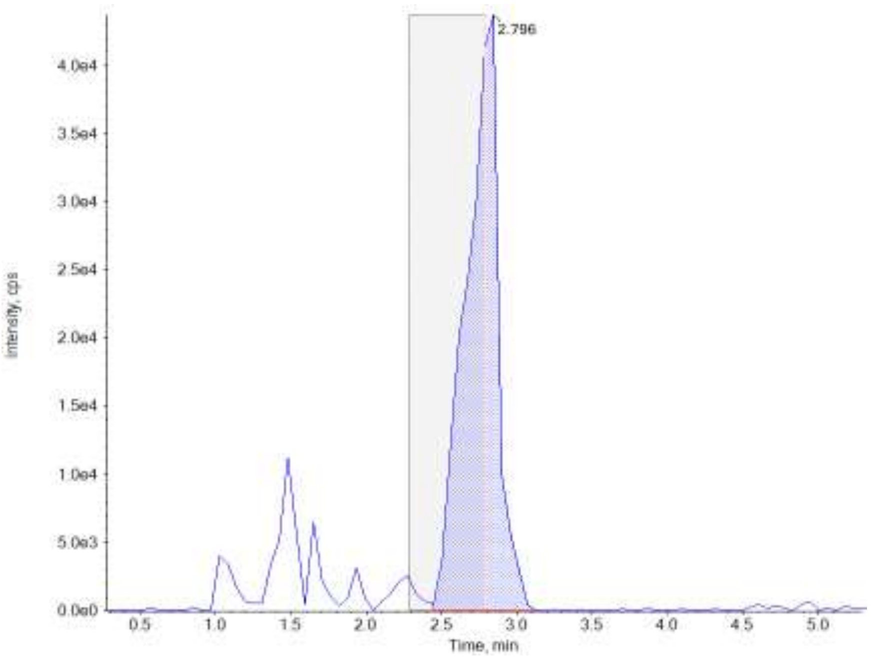


**Acquired / Library MSMS**

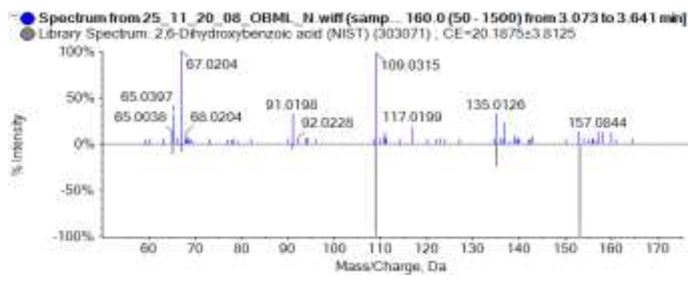


**Acquired / Theoretical MS**

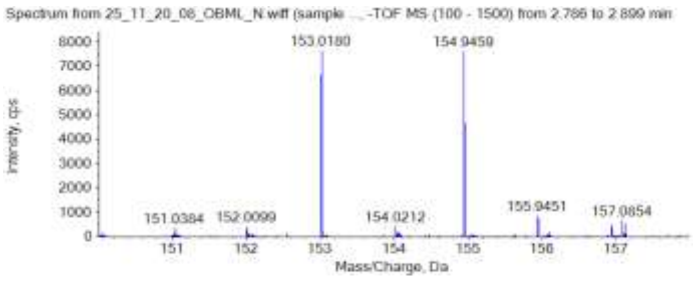

## 167.0335 / 2.84 (Mass/FragMass/RT/Isotope/Library/Formula/Ion Ratio)

**Retention Time:** 2.81 minutes
**Precursor m/z :** 167.0335
**Fit (%)** 99.3% **RFit (%)** 93.3%

**Exp RT:** 2.84 minutes
**Analyte Name:**
167.0335 / 2.84

**Collision Energy = 35 ± 15 eV**

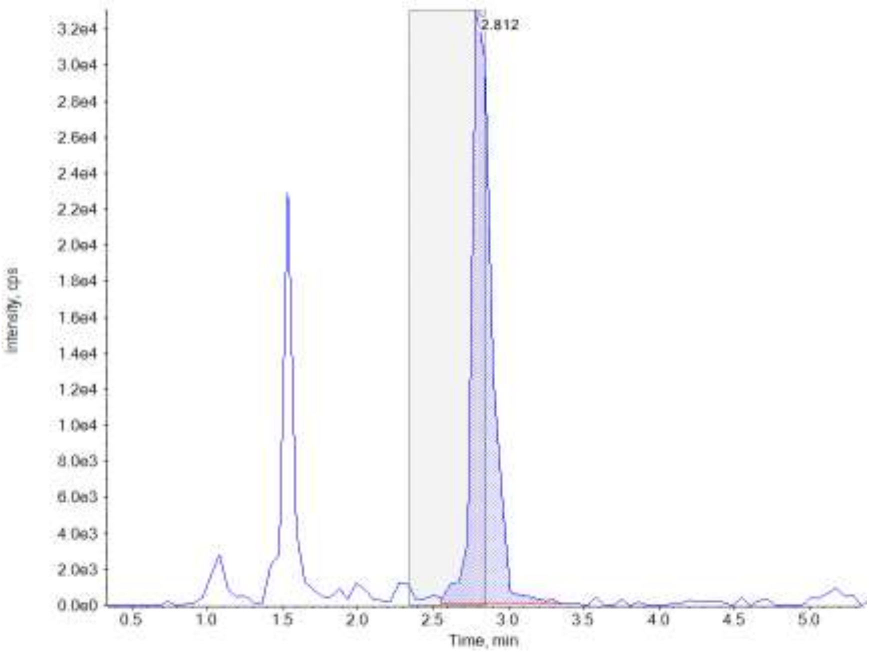


**Acquired / Library MSMS**

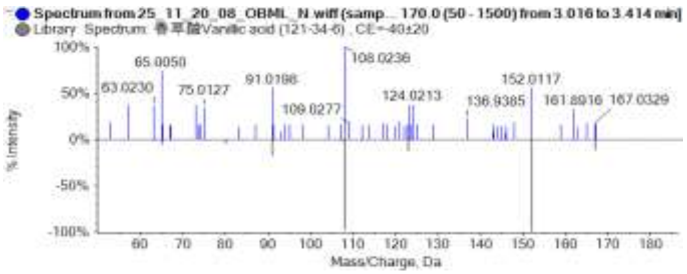


**Acquired / Theoretical MS**

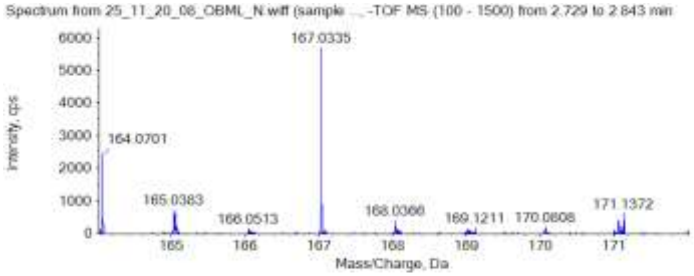

**245.0654 / 2.84** (Mass/FragMass/RT/Isotope/Library/Formula/Ion Ratio)

**Retention Time:** 2.85 minutes
**Precursor m/z :** 245.0654
**Fit (%)** 100.0% **RFit (%)** 23.1%

**Exp RT:** 2.84 minutes
**Analyte Name:**
245.0654 / 2.84

**Collision Energy = 35 ± 15 eV**

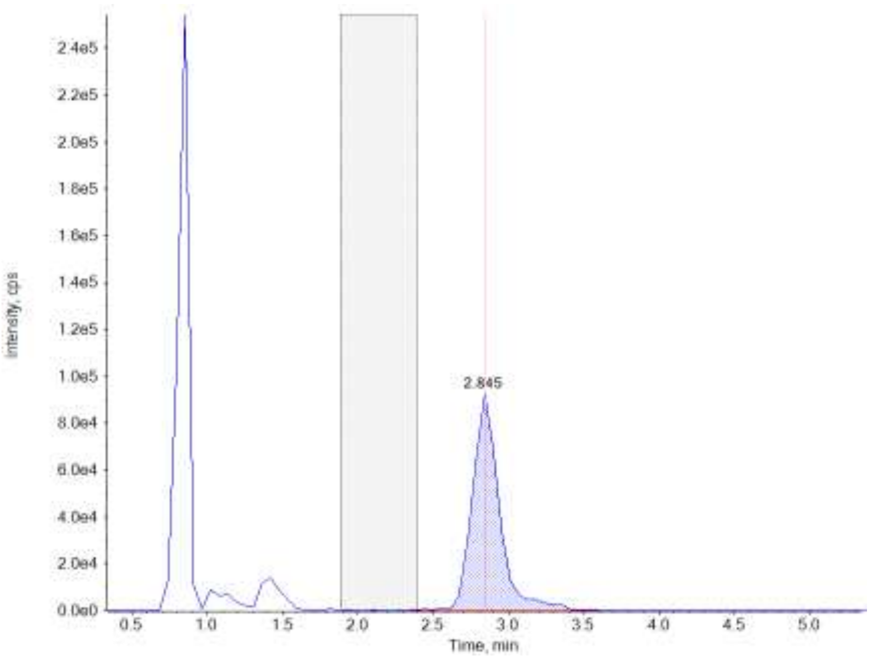


**Acquired / Library MSMS**

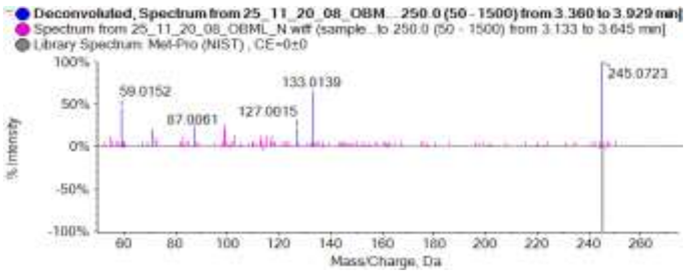


**Acquired / Theoretical MS**

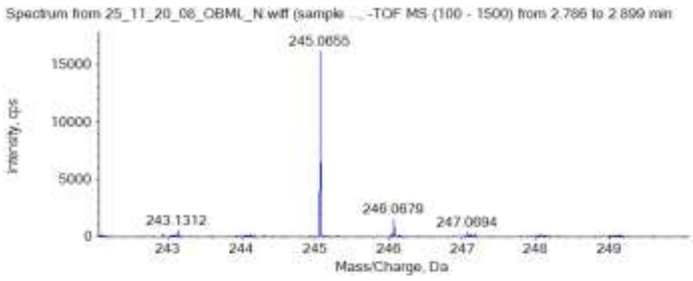

## 443.1905 / 2.96 (Mass/FragMass/RT/Isotope/Library/Formula/Ion Ratio)

**Retention Time:** 2.93 minutes **Exp RT:** 2.96 minutes
**Precursor m/z :** 443.1905 **Analyte Name:**
**Fit (%)** 31.1% **RFit (%)** 97.5% 443.1905 / 2.96

**Collision Energy = 35 ± 15 eV**

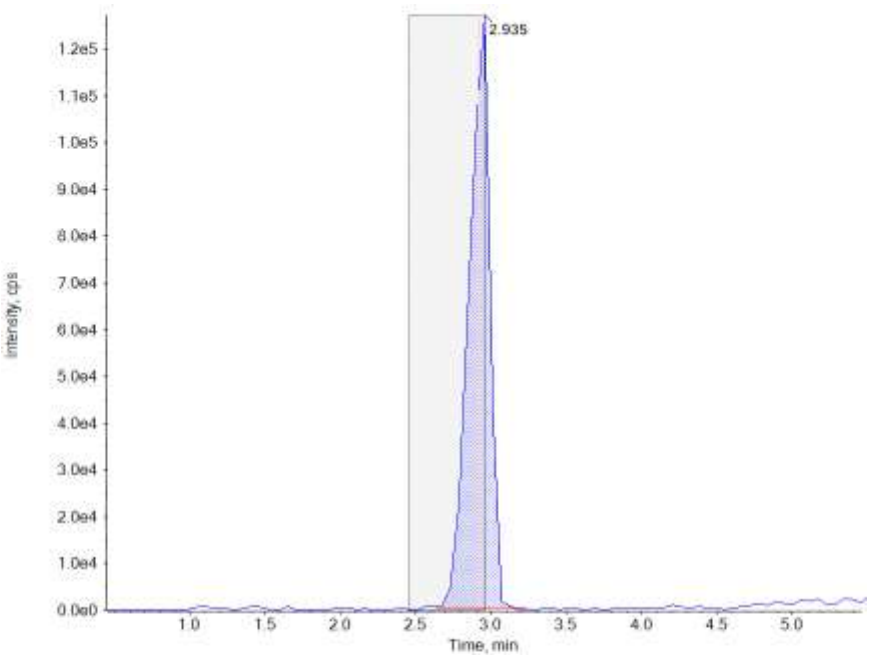


**Acquired / Library MSMS**

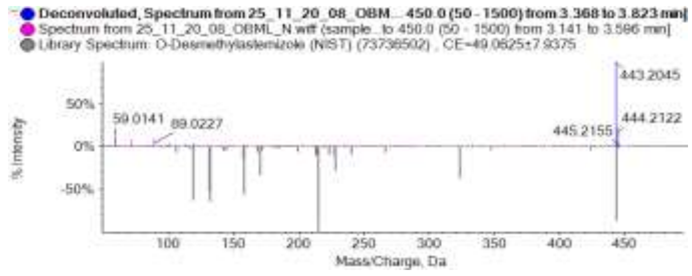


**Acquired / Theoretical MS**

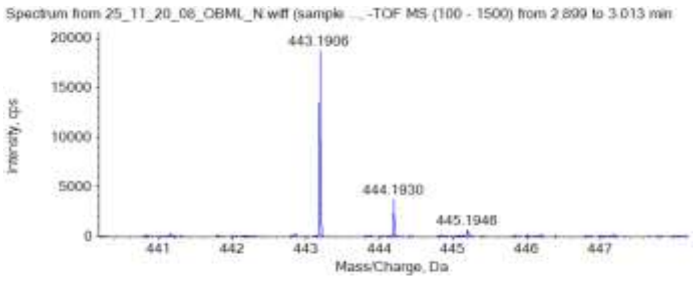

## 203.0858 / 3.07 (Mass/FragMass/RT/Isotope/Library/Formula/Ion Ratio)

**Retention Time:** 3.13 minutes
**Precursor m/z :** 203.0858
**Fit (%)** 98.5% **RFit (%)** 83.0%

**Exp RT:** 3.07 minutes
**Analyte Name:**
203.0858 / 3.07

**Collision Energy = 35 ± 15 eV**

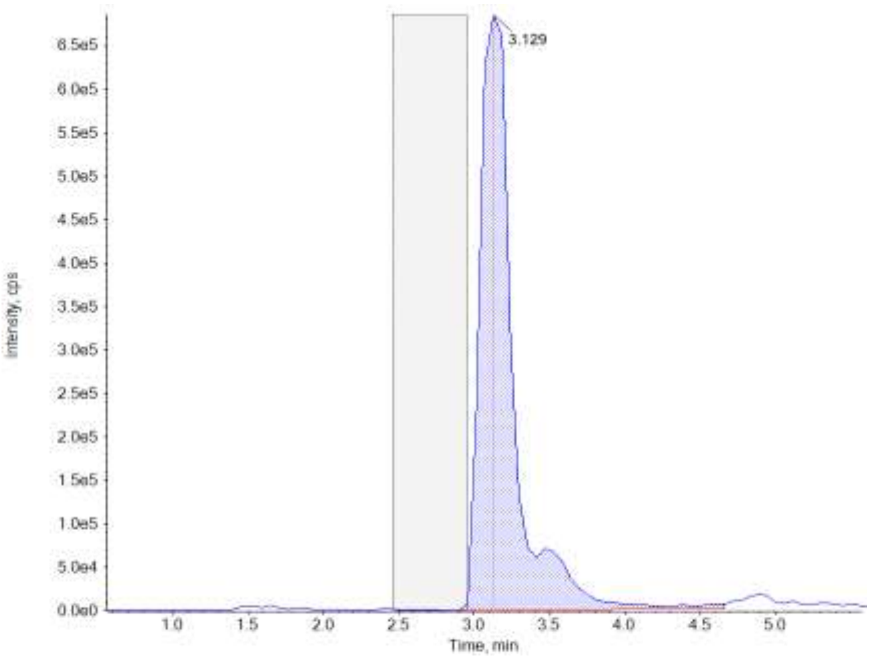


**Acquired / Library MSMS**

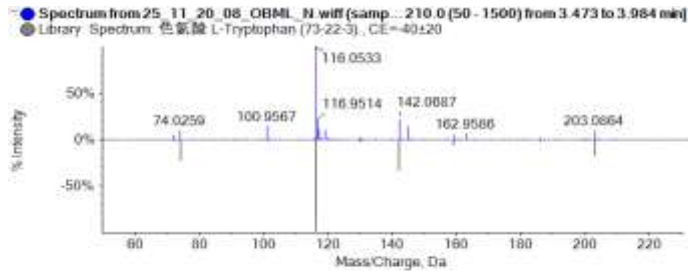


**Acquired / Theoretical MS**

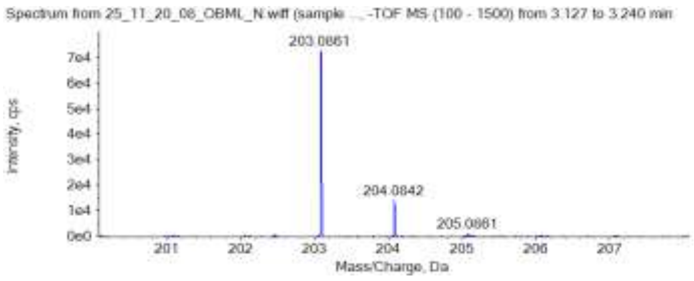

## 399.1642 / 3.13 (Mass/FragMass/RT/Isotope/Library/Formula/Ion Ratio)

| | |
|---|---|
| **Retention Time:** 3.10 minutes | **Exp RT:** 3.13 minutes |
| **Precursor m/z :** 399.1642 | **Analyte Name:** |
| **Fit (%)** 66.0% **RFit (%)** 98.9% | 399.1642 / 3.13 |

**Collision Energy = 35 ± 15 eV**

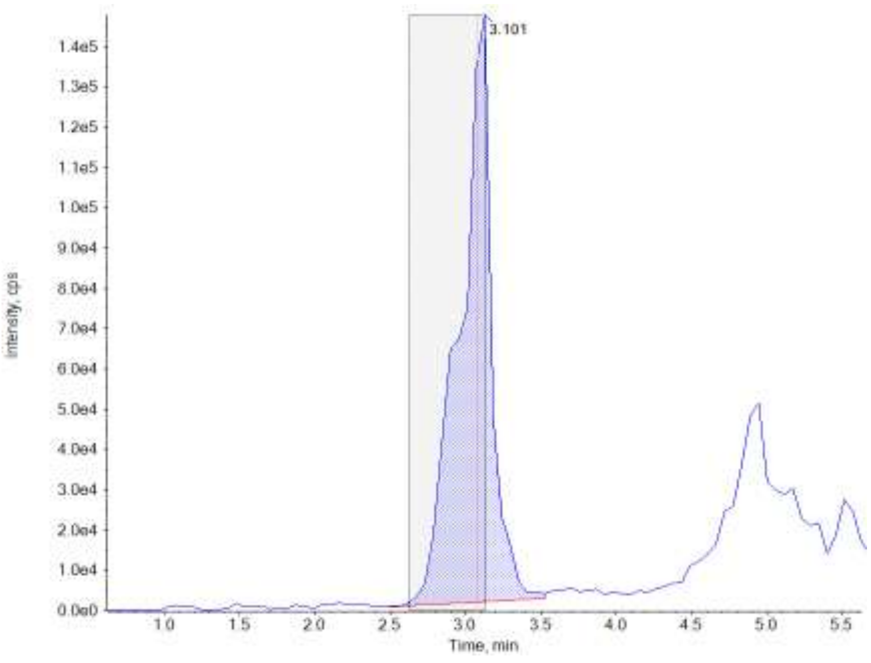


**Acquired / Library MSMS**

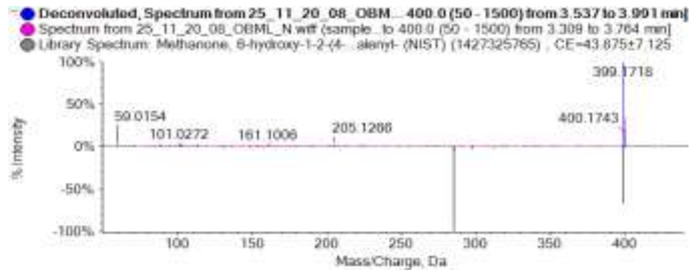


**Acquired / Theoretical MS**

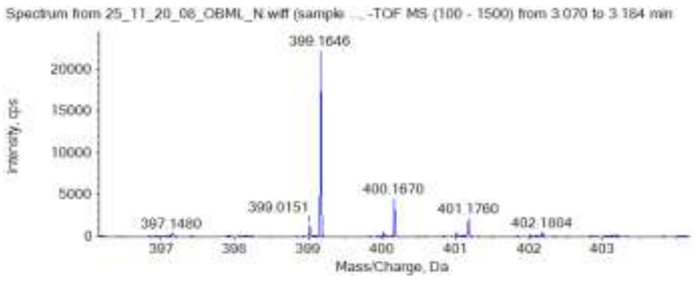

## 387.1636 / 3.35 (Mass/FragMass/RT/Isotope/Library/Formula/Ion Ratio)

**Retention Time:** 3.36 minutes
**Precursor m/z :** 387.1636
**Fit (%)** 34.9% **RFit (%)** 99.0%

**Exp RT:** 3.35 minutes
**Analyte Name:**
387.1636 / 3.35

**Collision Energy = 35 ± 15 eV**

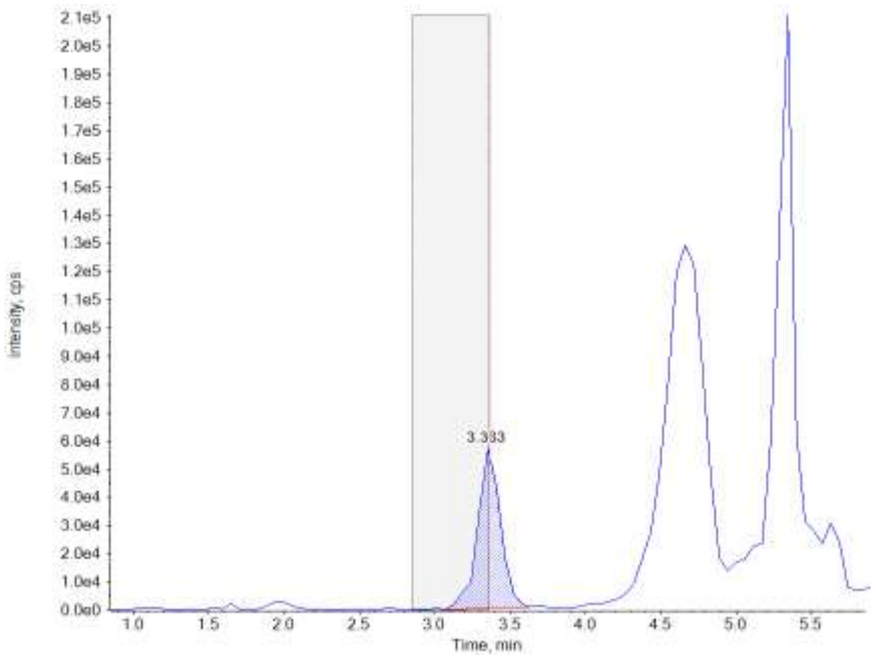


**Acquired / Library MSMS**

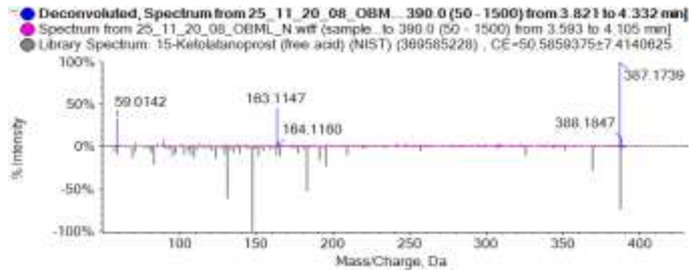


**Acquired / Theoretical MS**

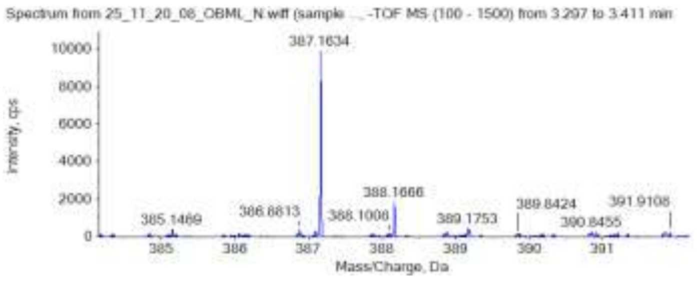

## 339.0710 / 4.83

(Mass/FragMass/RT/Isotope/Library/Formula/Ion Ratio)

**Retention Time:** 4.84 minutes
**Precursor m/z :** 339.0710
**Fit (%)** 92.1% **RFit (%)** 100.0%

**Exp RT:** 4.83 minutes
**Analyte Name:**
339.0710 / 4.83

**Collision Energy = 35 ± 15 eV**

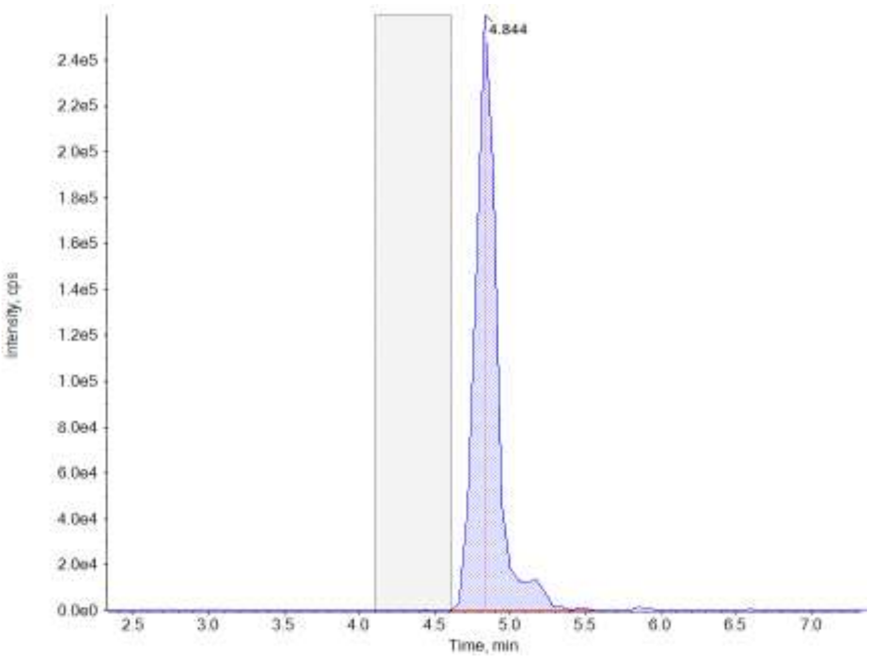


**Acquired / Library MSMS**

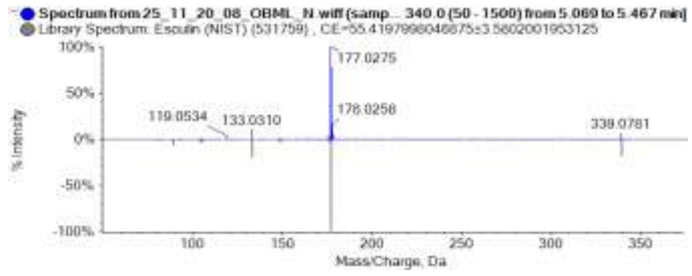


**Acquired / Theoretical MS**

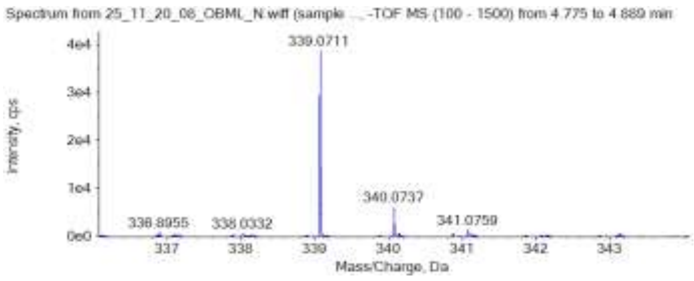

## 461.1476 / 5.00

(Mass/FragMass/RT/Isotope/Library/Formula/Ion Ratio)

| | |
|---|---|
| **Retention Time:** 5.03 minutes | **Exp RT:** 5.00 minutes |
| **Precursor m/z :** 461.1476 | **Analyte Name:** |
| **Fit (%)** 68.1% **RFit (%)** 71.3% | 461.1476 / 5.00 |

**Collision Energy = 35 ± 15 eV**

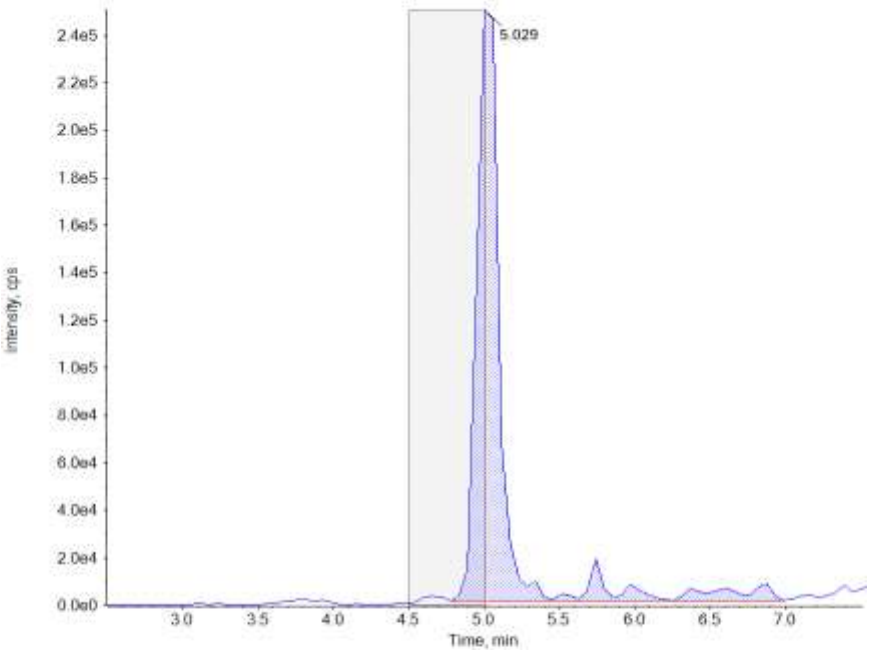


**Acquired / Library MSMS**

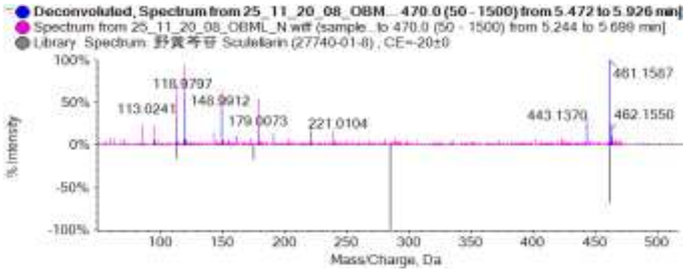


**Acquired / Theoretical MS**

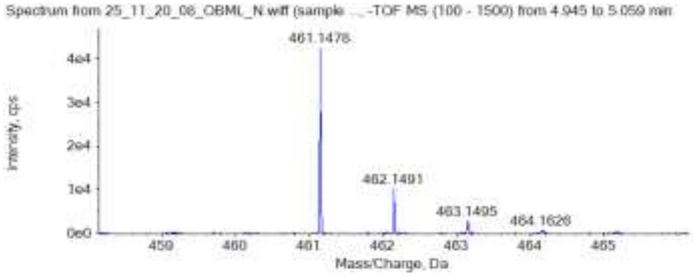

**387.1644 / 5.34** (Mass/FragMass/RT/Isotope/Library/Formula/Ion Ratio)

| | |
|---|---|
| **Retention Time:** 5.33 minutes<br>**Precursor m/z :** 387.1644<br>**Fit (%)** 58.8% **RFit (%)** 100.0% | **Exp RT:** 5.34 minutes<br>**Analyte Name:**<br>387.1644 / 5.34 |

| | | **Collision Energy = 35 ± 15 eV** |
|---|---|---|
| 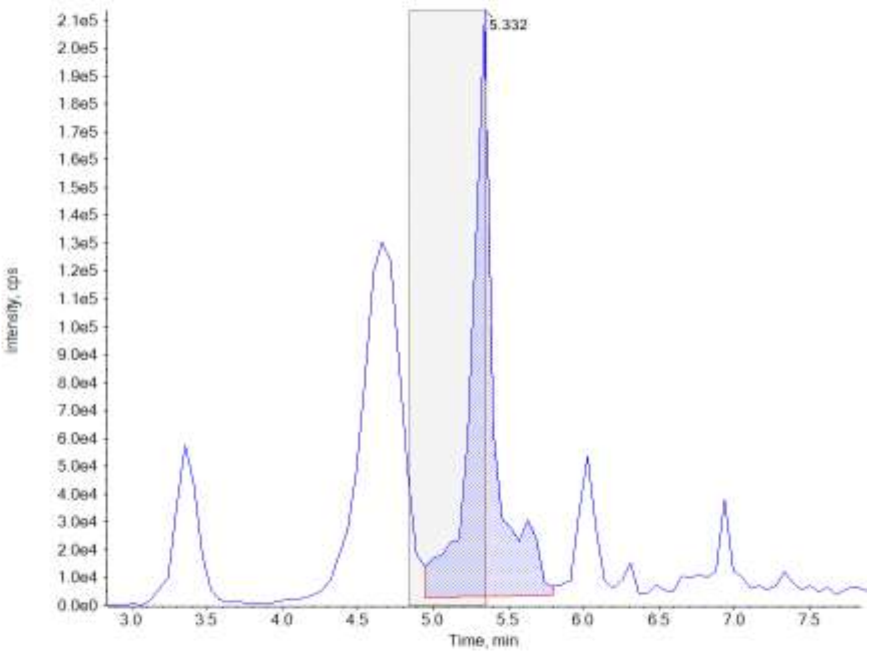 | **Acquired / Library MSMS** | 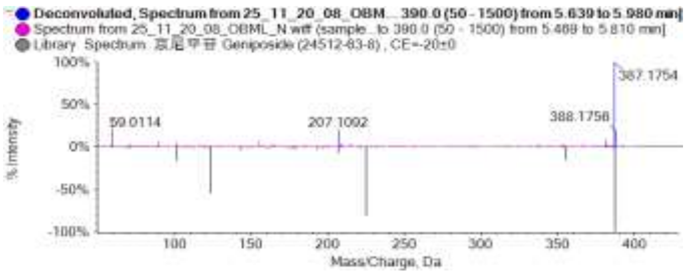 |
| | **Acquired / Theoretical MS** | 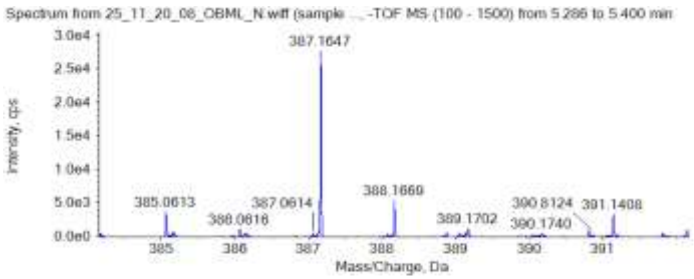 |

## 433.2074 / 5.63 (Mass/FragMass/RT/Isotope/Library/Formula/Ion Ratio)

**Retention Time:** 5.63 minutes
**Precursor m/z :** 433.2074
**Fit (%)** 30.1% **RFit (%)** 100.0%

**Exp RT:** 5.63 minutes
**Analyte Name:**
433.2074 / 5.63

**Collision Energy = 35 ± 15 eV**

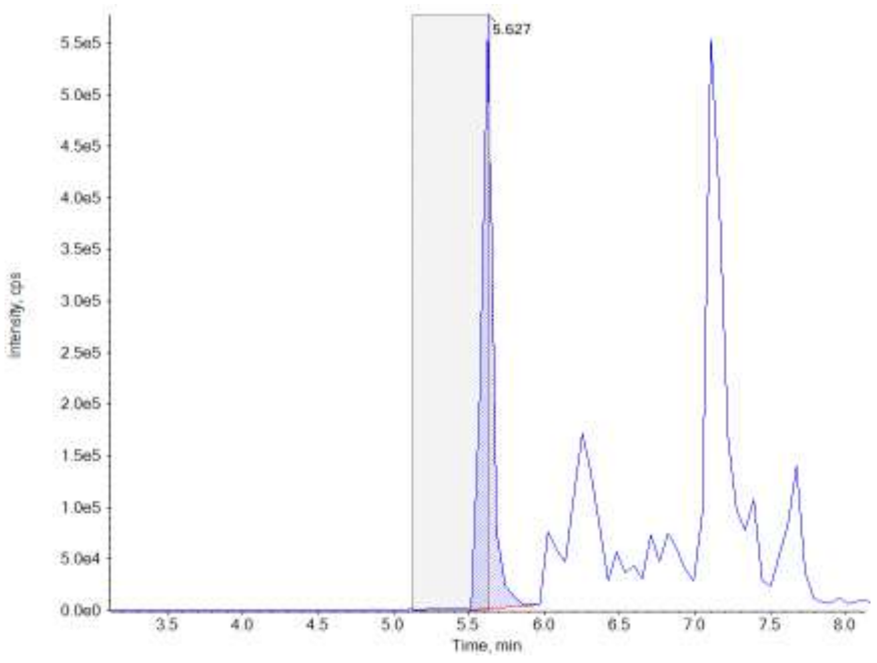


**Acquired / Library MSMS**

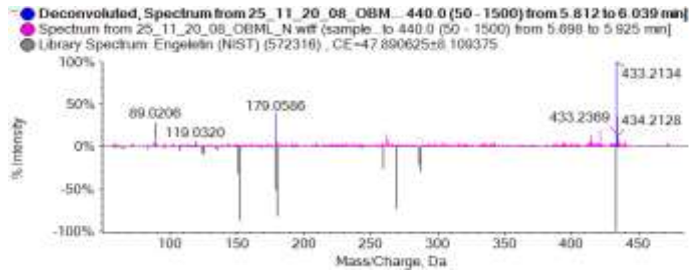


**Acquired / Theoretical MS**

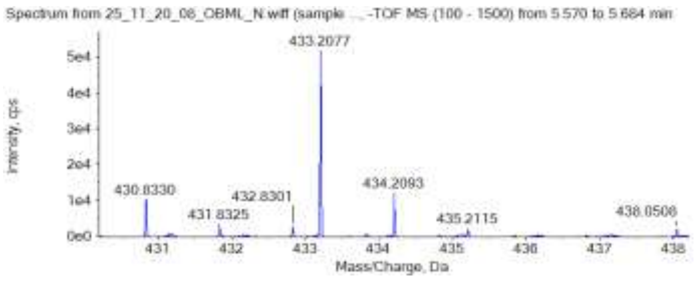

## 121.0287 / 6.20 (Mass/FragMass/RT/Isotope/Library/Formula/Ion Ratio)

**Retention Time:** 6.22 minutes
**Precursor m/z :** 121.0287
**Fit (%)** 97.6% **RFit (%)** 98.2%

**Exp RT:** 6.20 minutes
**Analyte Name:**
121.0287 / 6.20

**Collision Energy = 35 ± 15 eV**

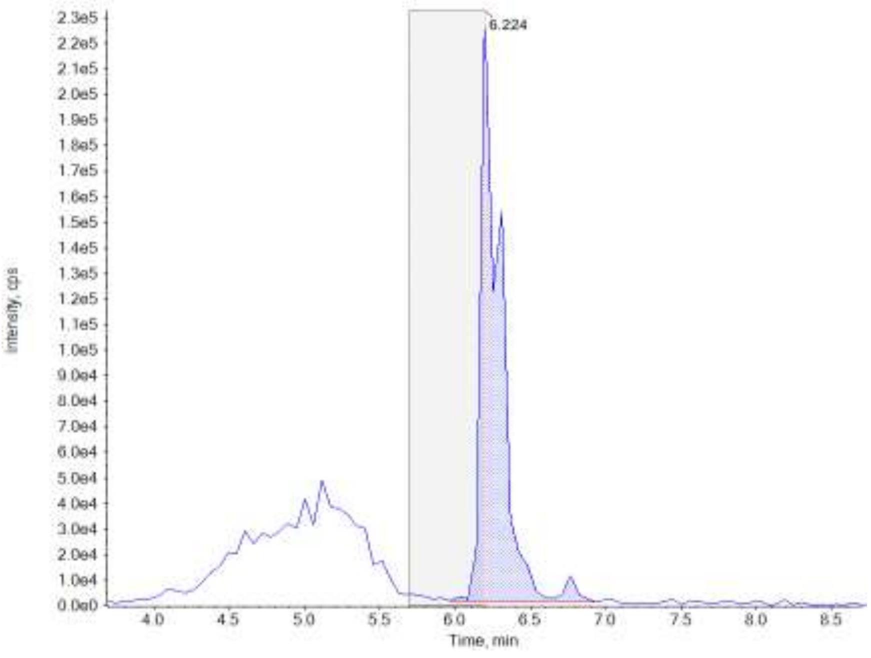


**Acquired / Library MSMS**

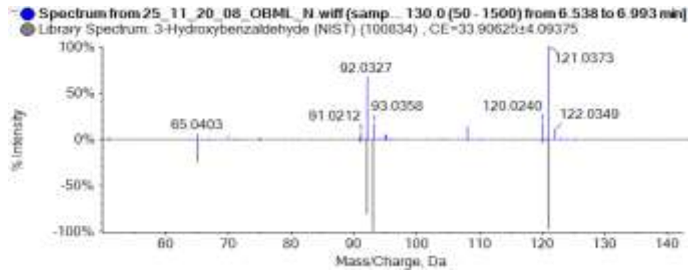


**Acquired / Theoretical MS**

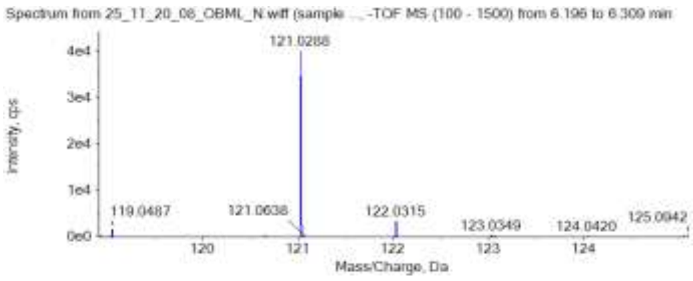

Created with SCIEX OS 3.3.0
Printed:12/9/2025 2:26:41 PM

## 421.1642 / 6.25 [M+Cl]- (Mass/FragMass/RT/Isotope/Library/Formula/Ion Ratio)

**Retention Time:** 6.25 minutes
**Precursor m/z :** 421.1642
**Fit (%)** 93.3% **RFit (%)** 79.9%

**Exp RT:** 6.25 minutes
**Analyte Name:**
421.1642 / 6.25 [M+Cl]-

**Collision Energy = 35 ± 15 eV**

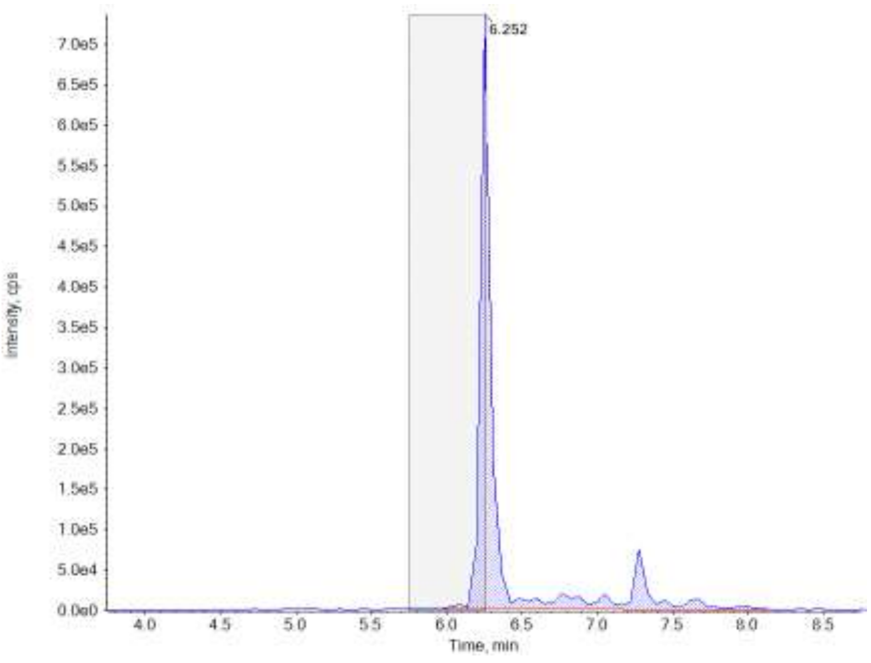


**Acquired / Library MSMS**

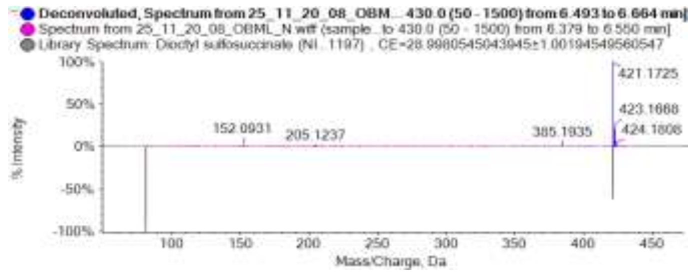


**Acquired / Theoretical MS**

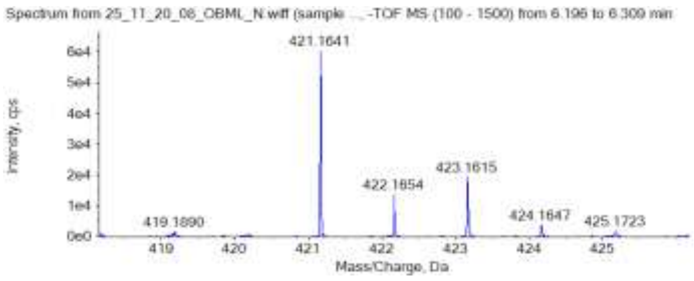

## 431.2011 / 6.25 (Mass/FragMass/RT/Isotope/Library/Formula/Ion Ratio)

**Retention Time:** 6.25 minutes
**Precursor m/z :** 431.2011
**Fit (%)** 99.8% **RFit (%)** 75.7%

**Exp RT:** 6.25 minutes
**Analyte Name:**
431.2011 / 6.25

**Collision Energy = 35 ± 15 eV**

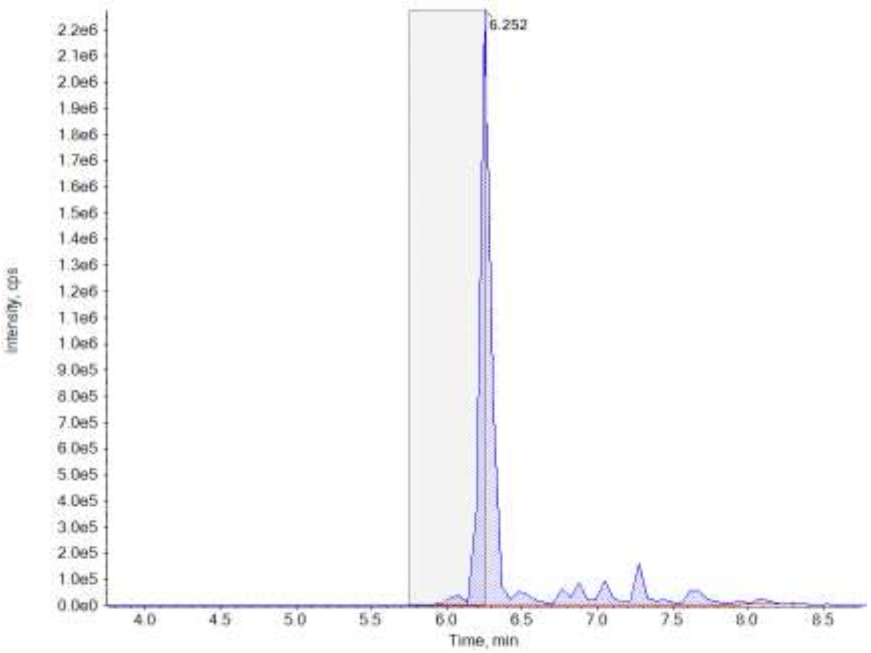


**Acquired / Library MSMS**

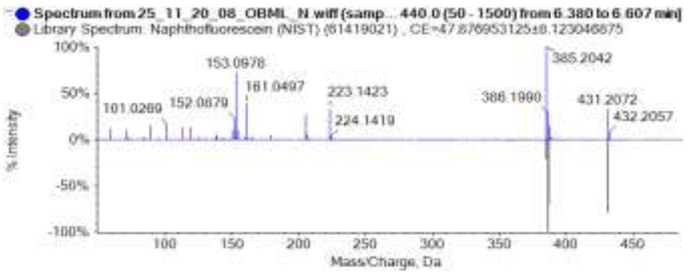


**Acquired / Theoretical MS**

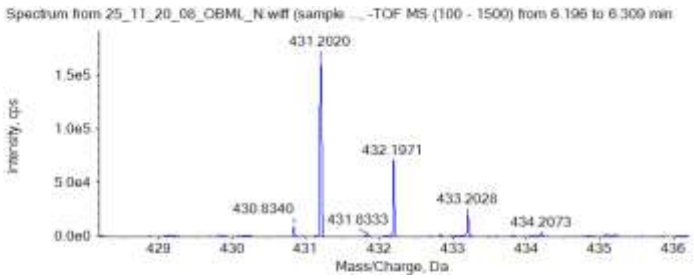

## 353.1259 / 6.37 (Mass/FragMass/RT/Isotope/Library/Formula/Ion Ratio)

**Retention Time:** 6.37 minutes
**Precursor m/z :** 353.1259
**Fit (%)** 100.0% **RFit (%)** 77.2%

**Exp RT:** 6.37 minutes
**Analyte Name:**
353.1259 / 6.37

**Collision Energy = 35 ± 15 eV**

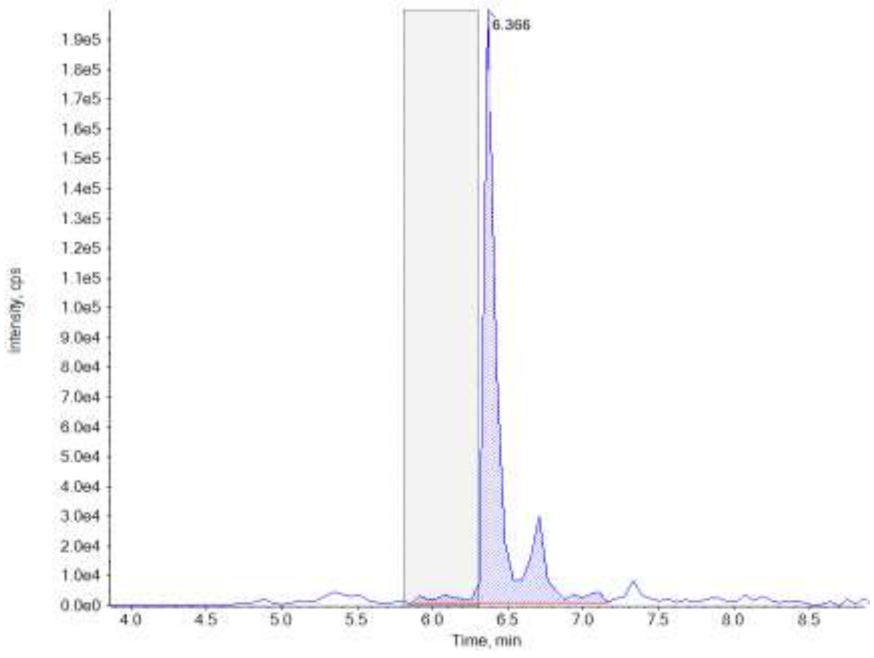


Acquired / Library MSMS

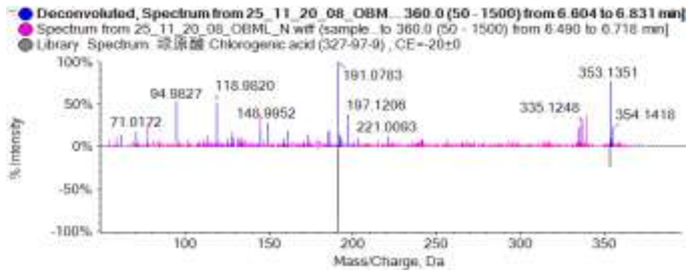


Acquired / Theoretical MS

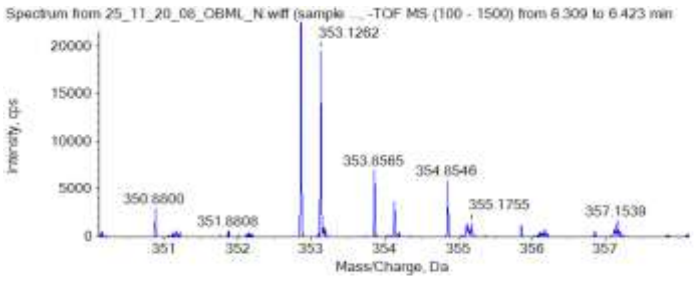

**585.2392 / 6.65** (Mass/FragMass/RT/Isotope/Library/Formula/Ion Ratio)

| | |
|---|---|
| **Retention Time:** 6.63 minutes | **Exp RT:** 6.65 minutes |
| **Precursor m/z :** 585.2392 | **Analyte Name:** |
| **Fit (%)** 81.2% **RFit (%)** 94.7% | 585.2392 / 6.65 |

**Collision Energy = 35 ± 15 eV**

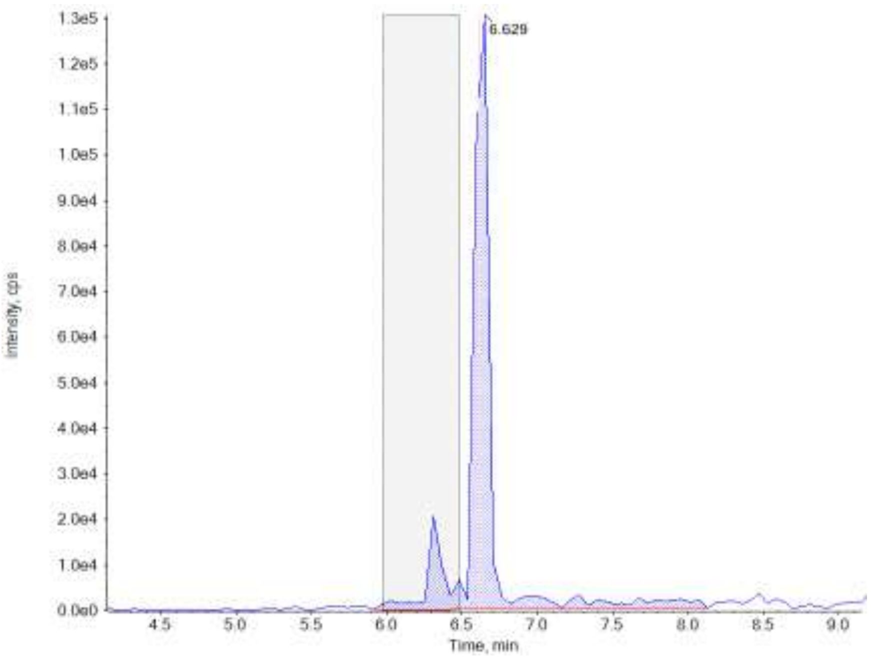


**Acquired / Library MSMS**

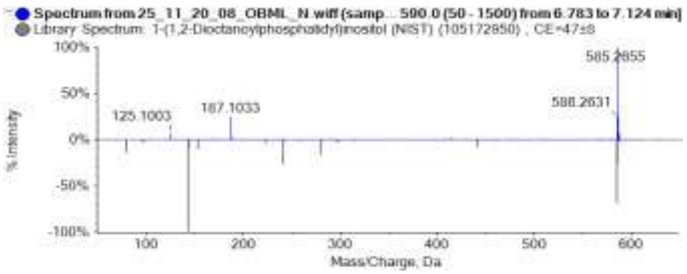


**Acquired / Theoretical MS**

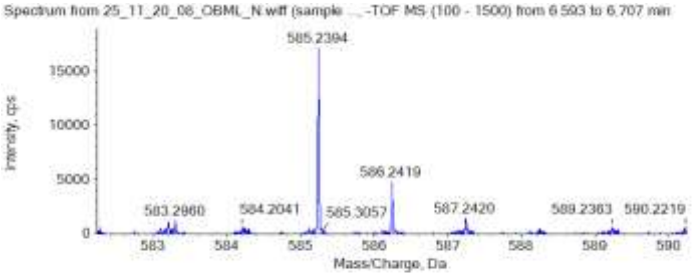

**475.1597 / 6.71** (Mass/FragMass/RT/Isotope/Library/Formula/Ion Ratio)

| | |
|---|---|
| **Retention Time:** 6.61 minutes<br>**Precursor m/z :** 475.1597<br>**Fit (%)** 36.7% **RFit (%)** 99.9% | **Exp RT:** 6.71 minutes<br>**Analyte Name:**<br>475.1597 / 6.71 |

**Collision Energy = 35 ± 15 eV**

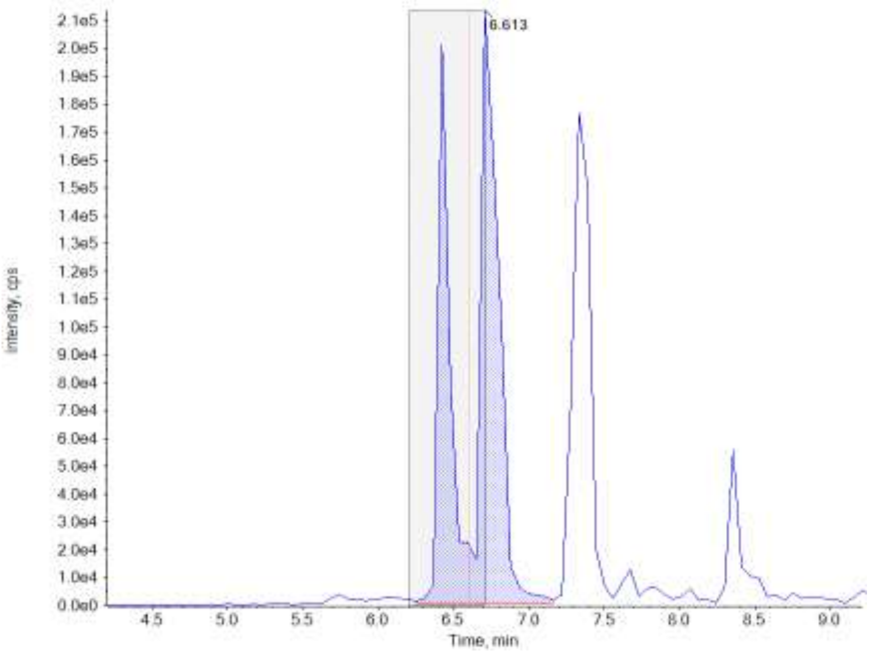


**Acquired / Library MSMS**

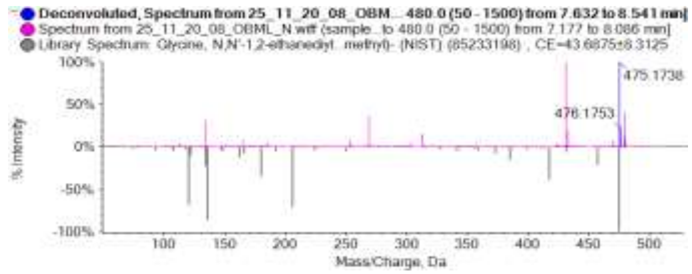


**Acquired / Theoretical MS**

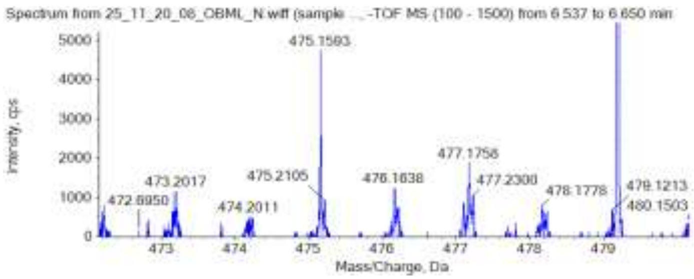

## 193.0495 / 6.93 (Mass/FragMass/RT/Isotope/Library/Formula/Ion Ratio)

| | |
|---|---|
| **Retention Time:** 6.93 minutes | **Exp RT:** 6.93 minutes |
| **Precursor m/z :** 193.0495 | **Analyte Name:** |
| **Fit (%)** 96.6% **RFit (%)** 98.5% | 193.0495 / 6.93 |

**Collision Energy = 35 ± 15 eV**

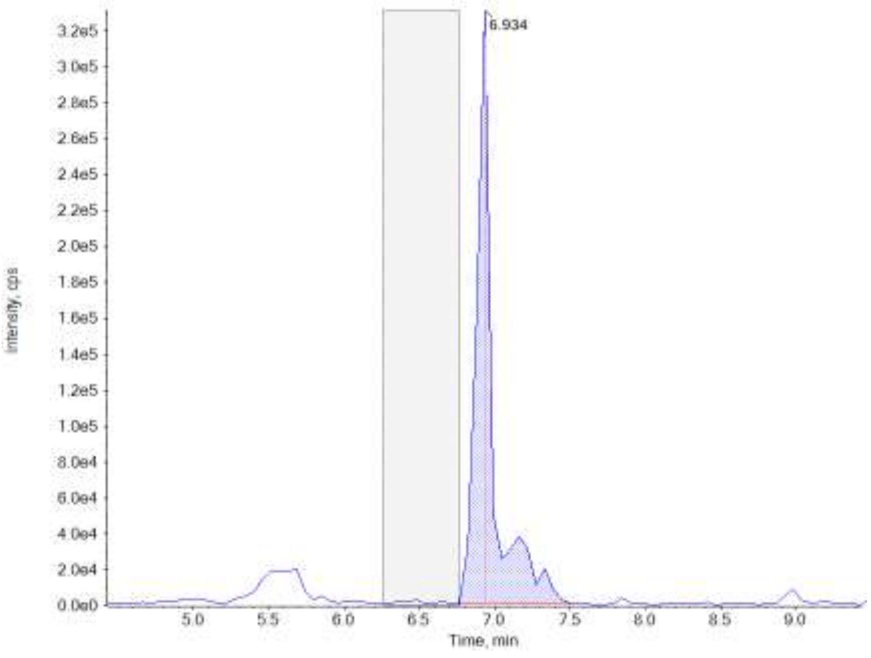


**Acquired / Library MSMS**

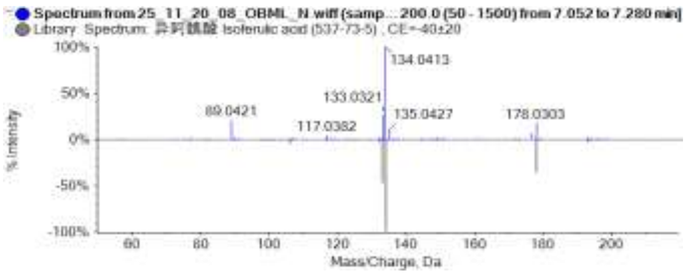


**Acquired / Theoretical MS**

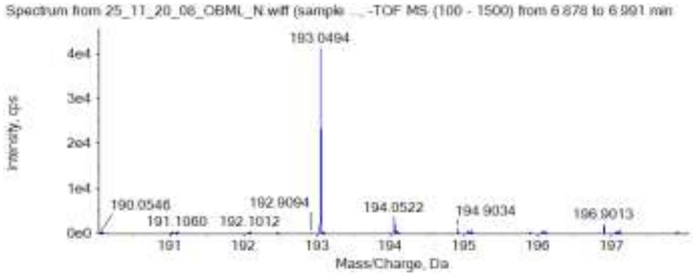

## 247.1540 / 6.93 (Mass/FragMass/RT/Isotope/Library/Formula/Ion Ratio)

**Retention Time:** 6.93 minutes
**Precursor m/z :** 247.1540
**Fit (%)** 100.0% **RFit (%)** 81.8%

**Exp RT:** 6.93 minutes
**Analyte Name:**
247.1540 / 6.93

**Collision Energy = 35 ± 15 eV**

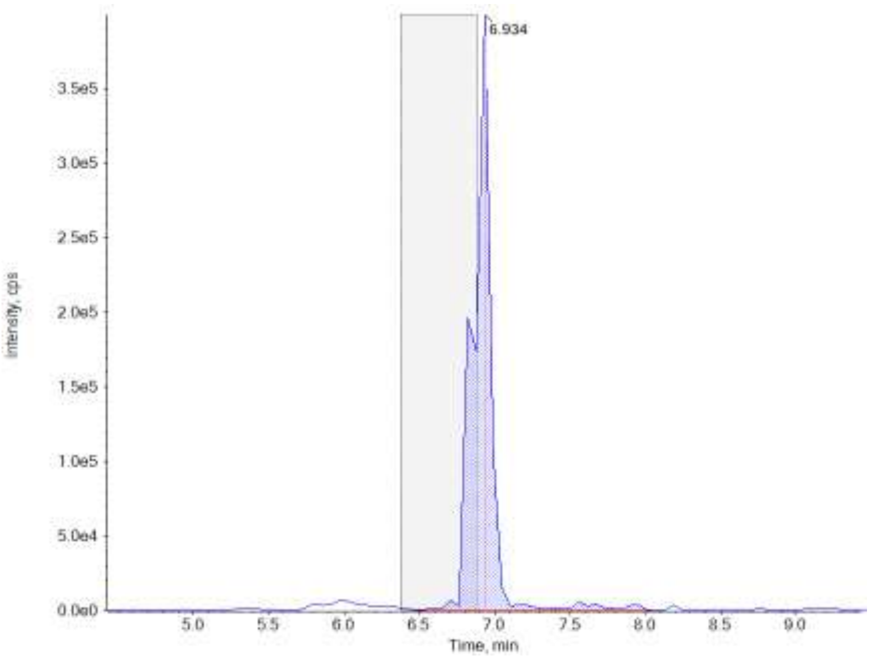


**Acquired / Library MSMS**

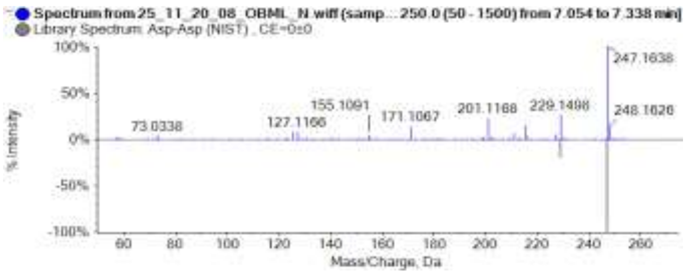


**Acquired / Theoretical MS**

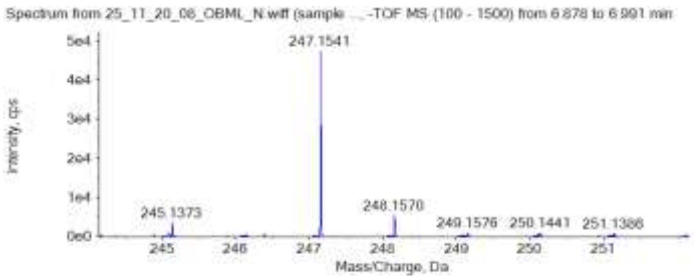

## 551.2346 / 6.99

(Mass/FragMass/RT/Isotope/Library/Formula/Ion Ratio)

**Retention Time:** 6.99 minutes
**Precursor m/z :** 551.2346
**Fit (%)** 99.1% **RFit (%)** 88.5%

**Exp RT:** 6.99 minutes
**Analyte Name:**
551.2346 / 6.99

**Collision Energy = 35 ± 15 eV**

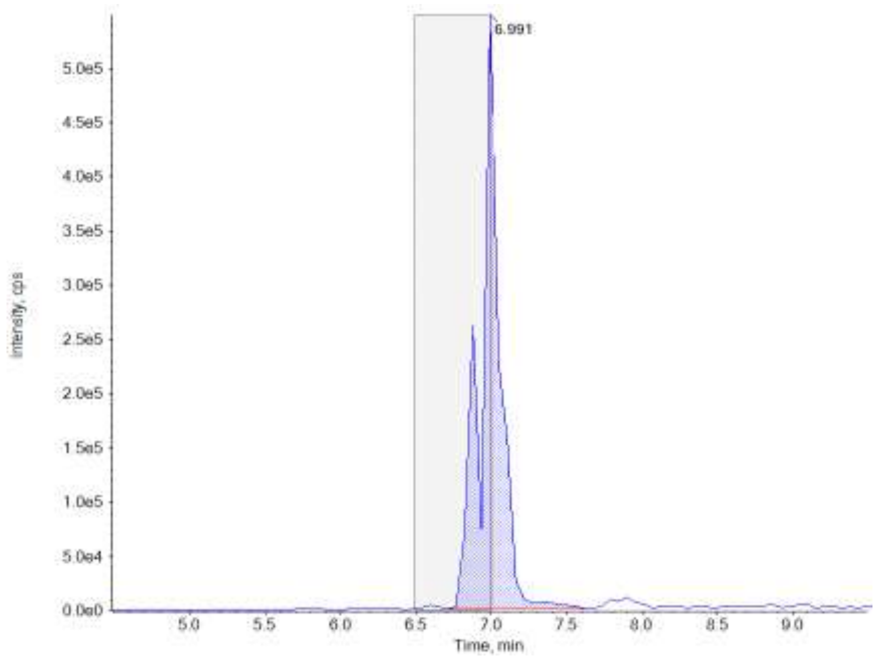


**Acquired / Library MSMS**

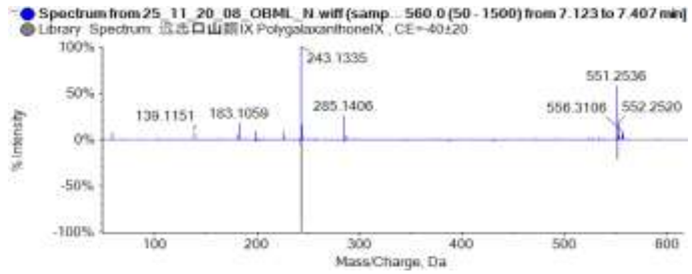


**Acquired / Theoretical MS**

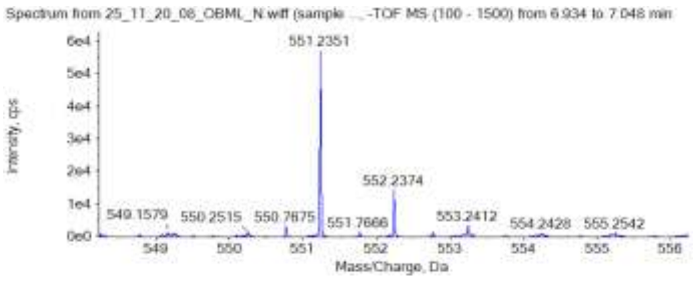

## 159.1023 / 7.05 (Mass/FragMass/RT/Isotope/Library/Formula/Ion Ratio)

**Retention Time:** 7.06 minutes
**Precursor m/z :** 159.1023
**Fit (%)** 100.0% **RFit (%)** 90.3%

**Exp RT:** 7.05 minutes
**Analyte Name:**
159.1023 / 7.05

**Collision Energy = 35 ± 15 eV**

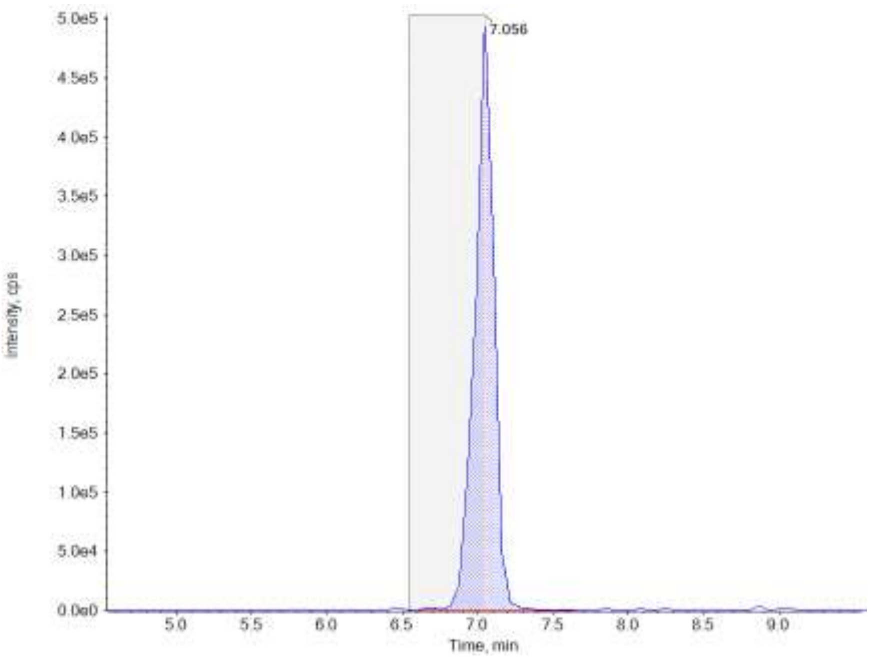


**Acquired / Library MSMS**

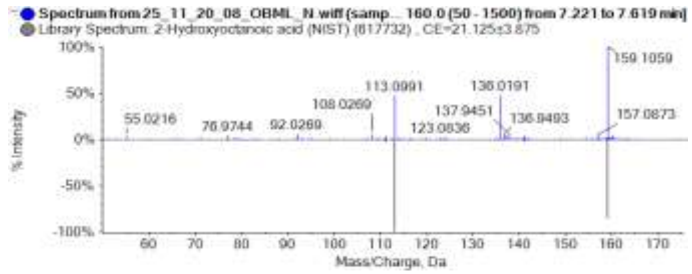


**Acquired / Theoretical MS**

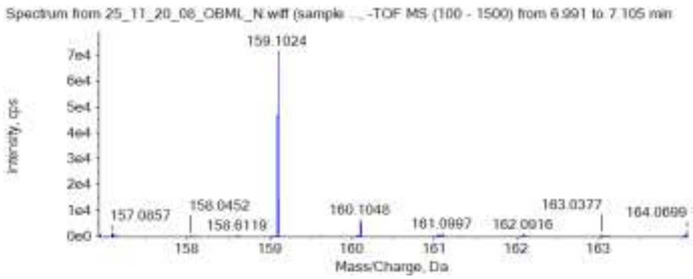

Created with SCIEX OS 3.3.0
Printed:12/9/2025 2:26:41 PM

## 433.2066 / 7.10 (Mass/FragMass/RT/Isotope/Library/Formula/Ion Ratio)

**Retention Time:** 7.12 minutes
**Precursor m/z :** 433.2066
**Fit (%)** 98.6% **RFit (%)** 96.9%

**Exp RT:** 7.11 minutes
**Analyte Name:**
433.2066 / 7.10

**Collision Energy = 35 ± 15 eV**

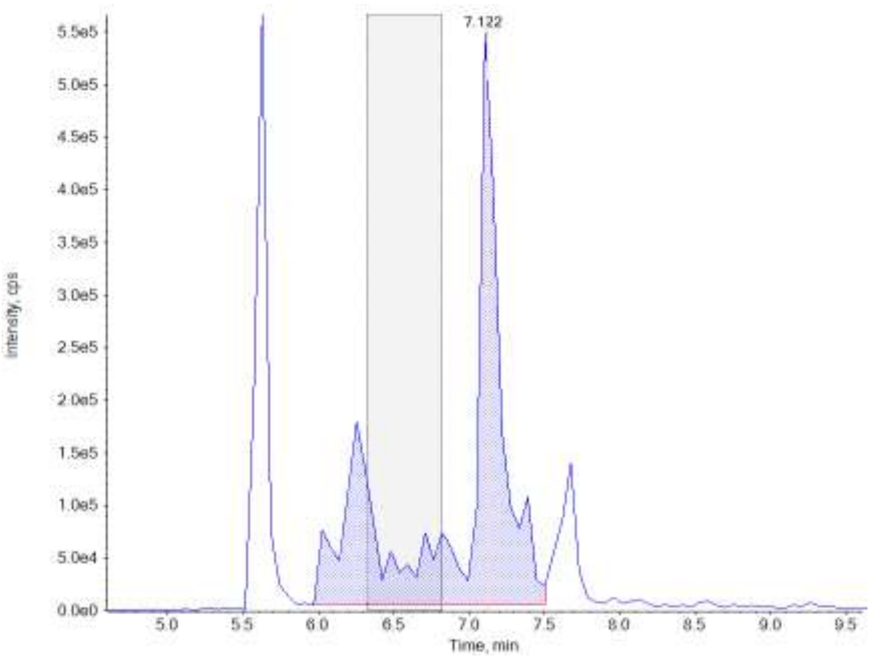


**Acquired / Library MSMS**

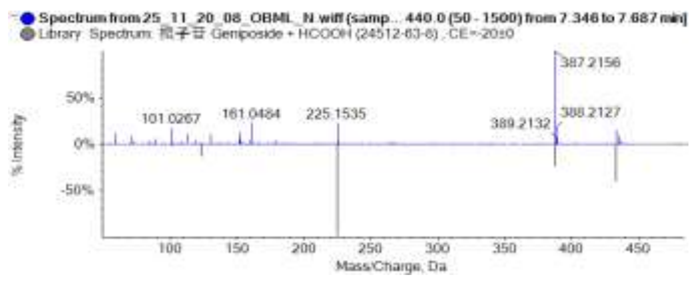


**Acquired / Theoretical MS**

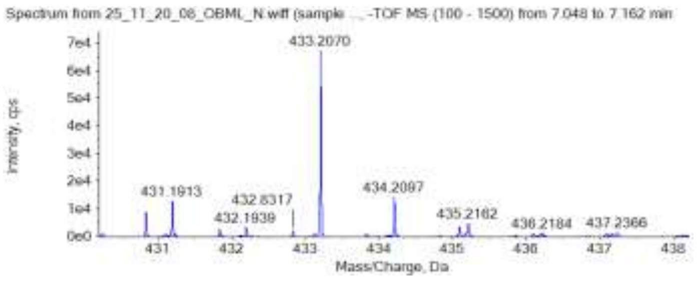

Created with SCIEX OS 3.3.0
Printed:12/9/2025 2:26:41 PM

## 361.2209 / 7.45 (Mass/FragMass/RT/Isotope/Library/Formula/Ion Ratio)

**Retention Time:** 7.54 minutes
**Precursor m/z :** 361.2209
**Fit (%)** 92.1% **RFit (%)** 82.4%

**Exp RT:** 7.45 minutes
**Analyte Name:**
361.2209 / 7.45

**Collision Energy = 35 ± 15 eV**

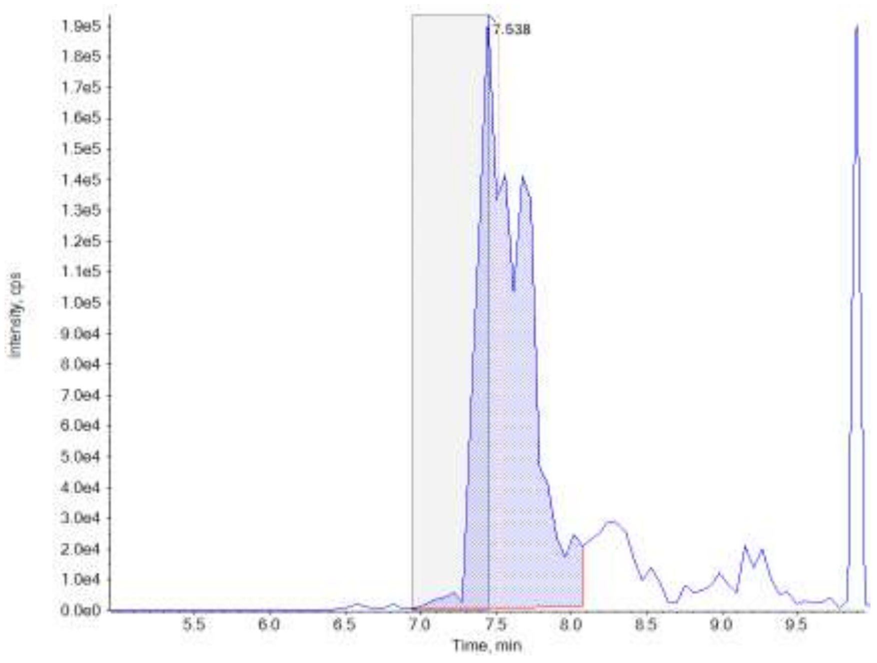


Acquired / Library MSMS

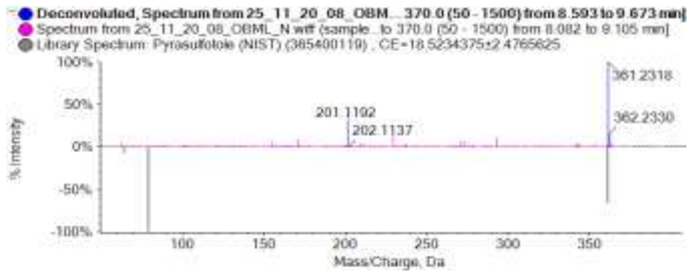


Acquired / Theoretical MS

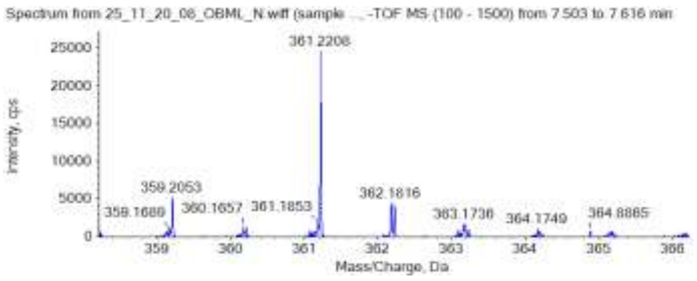

## 463.0867 / 7.50 (Mass/FragMass/RT/Isotope/Library/Formula/Ion Ratio)

**Retention Time:** 7.51 minutes
**Precursor m/z :** 463.0867
**Fit (%)** 99.6% **RFit (%)** 99.2%

**Exp RT:** 7.50 minutes
**Analyte Name:**
463.0867 / 7.50

**Collision Energy = 35 ± 15 eV**

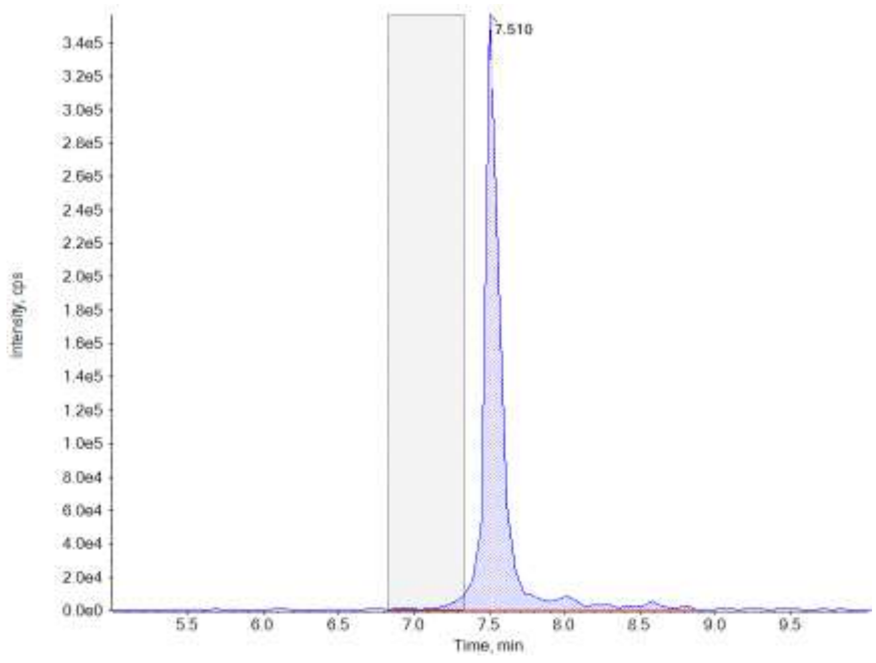


Acquired / Library MSMS

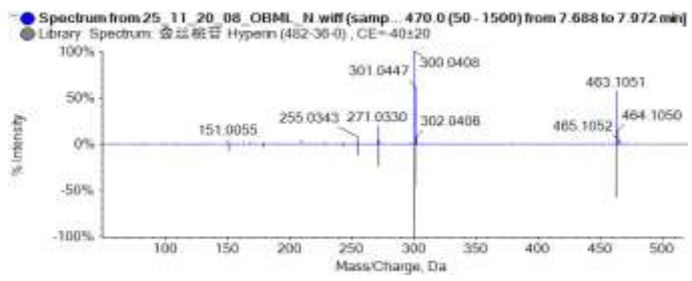


Acquired / Theoretical MS

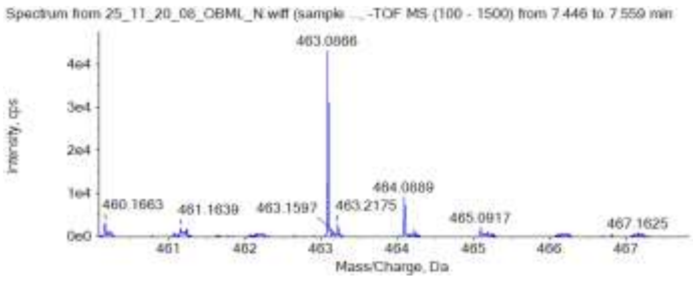

## 271.1539 / 7.67 (Mass/FragMass/RT/Isotope/Library/Formula/Ion Ratio)

**Retention Time:** 7.71 minutes **Exp RT:** 7.67 minutes
**Precursor m/z :** 271.1539 **Analyte Name:**
**Fit (%)** 99.1% **RFit (%)** 100.0% 271.1539 / 7.67

**Collision Energy = 35 ± 15 eV**

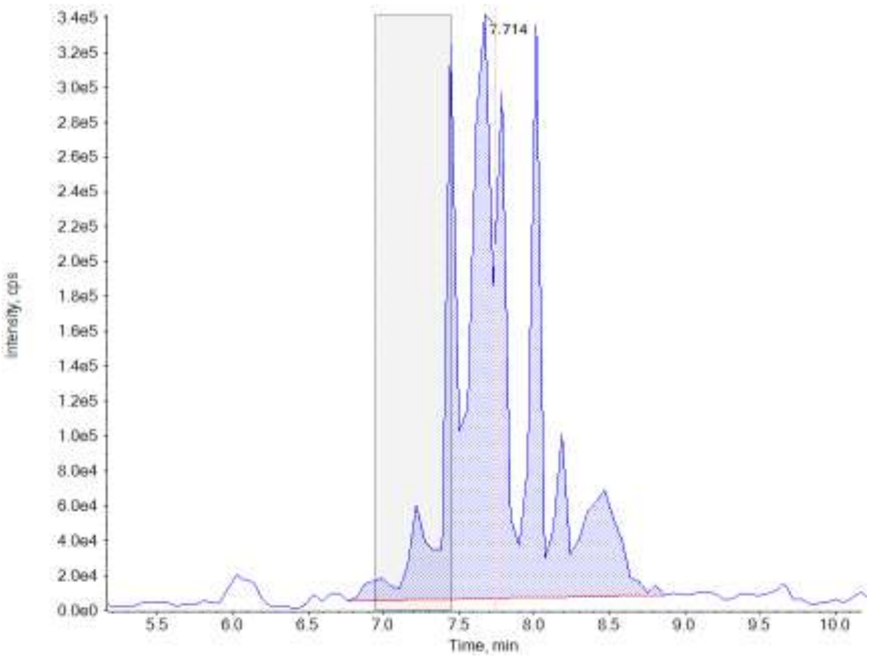


**Acquired / Library MSMS**

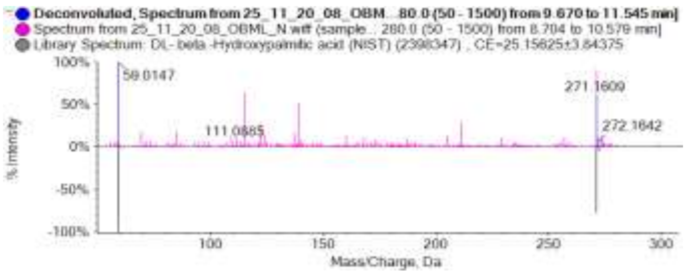


**Acquired / Theoretical MS**

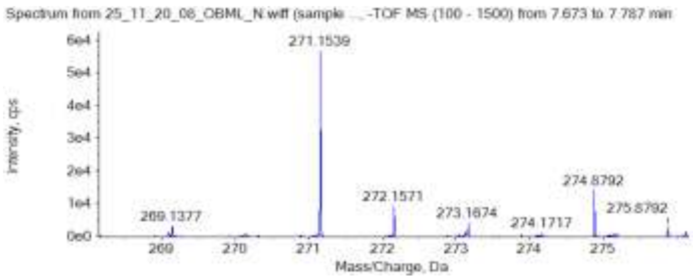

## 187.0992 / 8.01 (Mass/FragMass/RT/Isotope/Library/Formula/Ion Ratio)

**Retention Time:** 8.02 minutes
**Precursor m/z :** 187.0992
**Fit (%)** 100.0% **RFit (%)** 98.5%

**Exp RT:** 8.01 minutes
**Analyte Name:**
187.0992 / 8.01

**Collision Energy = 35 ± 15 eV**

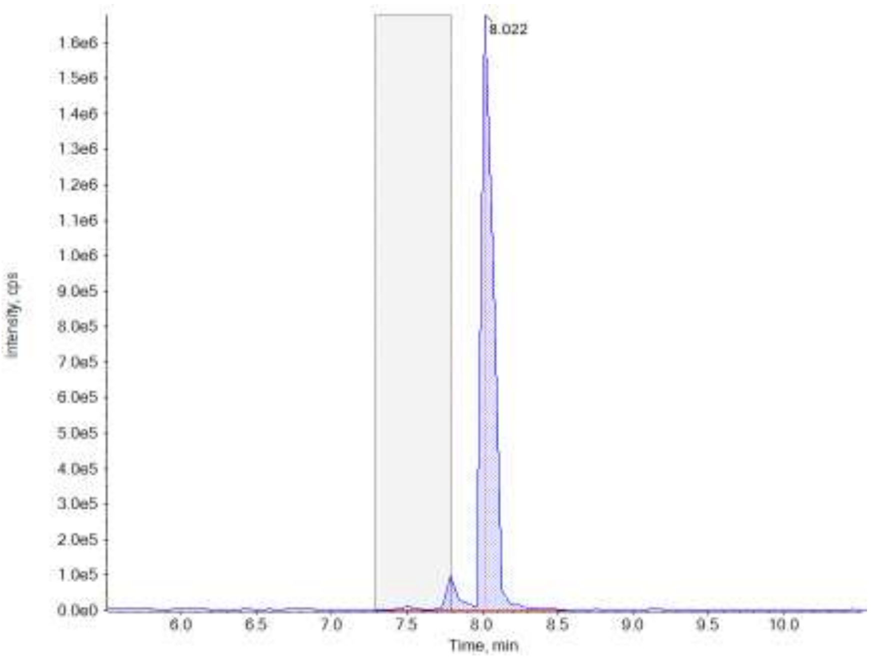


**Acquired / Library MSMS**

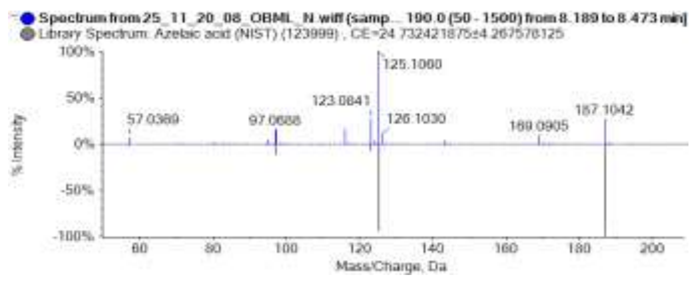


**Acquired / Theoretical MS**

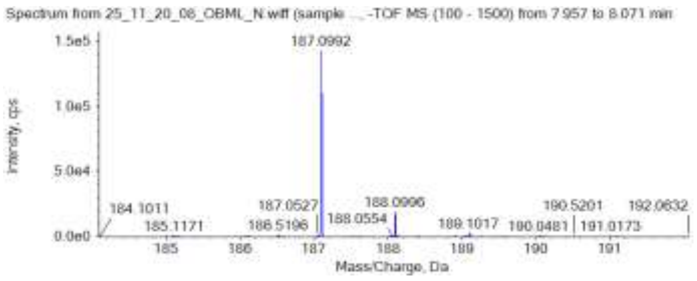

**447.0934 / 8.01** (Mass/FragMass/RT/Isotope/Library/Formula/Ion Ratio)

| | |
|---|---|
| **Retention Time:** 7.99 minutes | **Exp RT:** 8.01 minutes |
| **Precursor m/z :** 447.0934 | **Analyte Name:** |
| **Fit (%)** 100.0% **RFit (%)** 100.0% | 447.0934 / 8.01 |

**Collision Energy = 35 ± 15 eV**

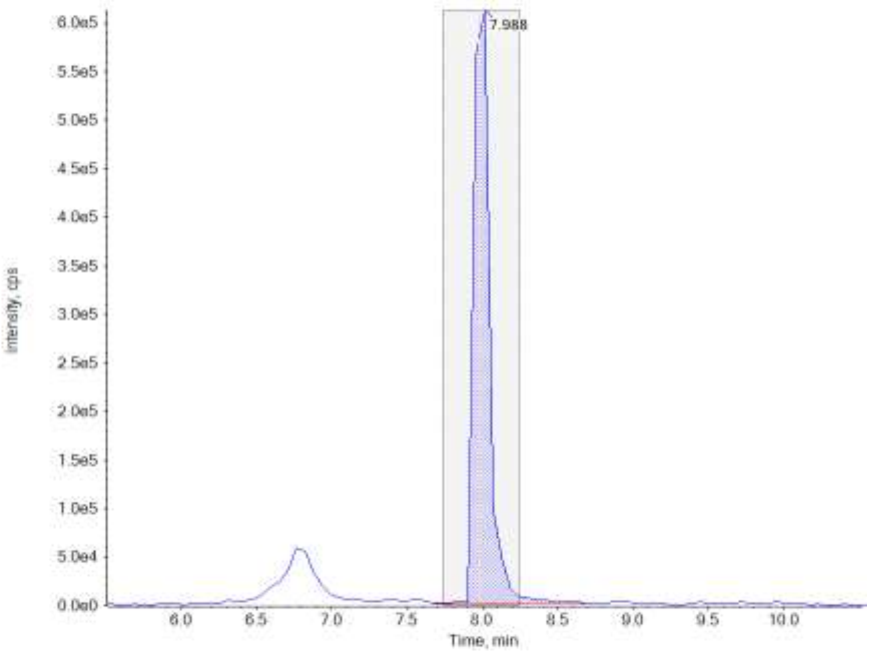


**Acquired / Library MSMS**

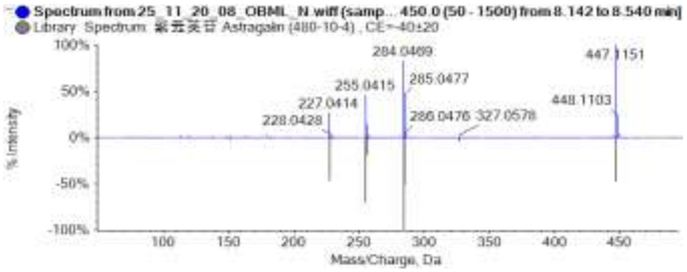


**Acquired / Theoretical MS**

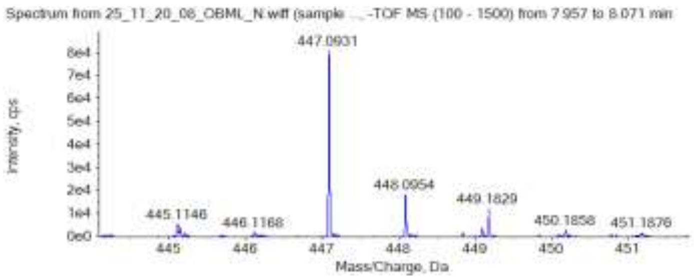

**229.1455 / 8.07** (Mass/FragMass/RT/Isotope/Library/Formula/Ion Ratio)

**Retention Time:** 8.30 minutes
**Precursor m/z :** 229.1455
**Fit (%)** 100.0% **RFit (%)** 100.0%

**Exp RT:** 8.07 minutes
**Analyte Name:**
229.1455 / 8.07

**Collision Energy = 35 ± 15 eV**

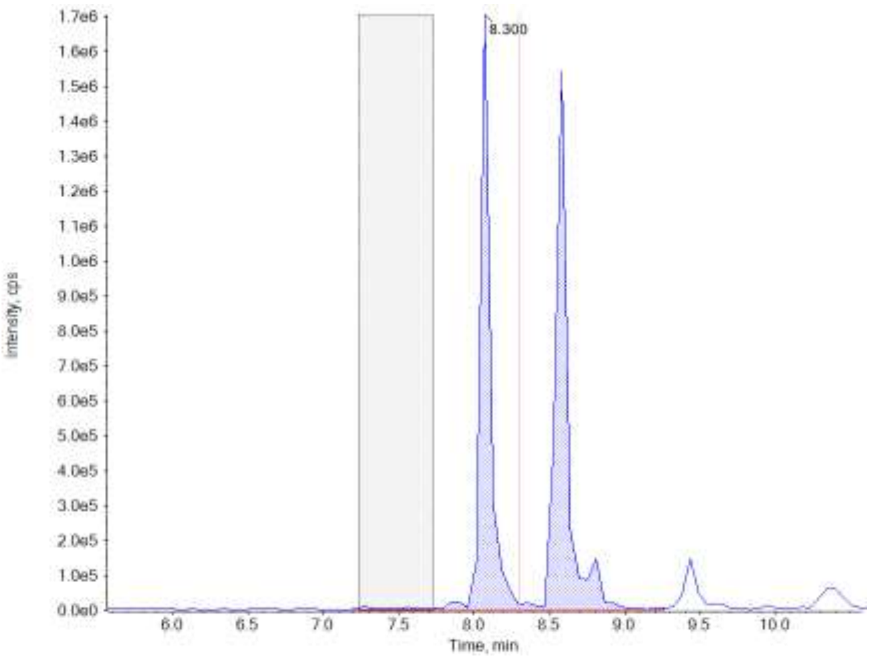


**Acquired / Library MSMS**

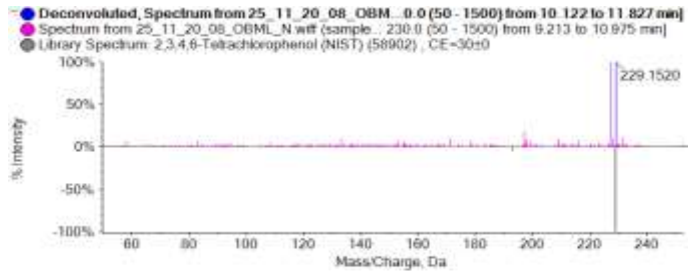


**Acquired / Theoretical MS**

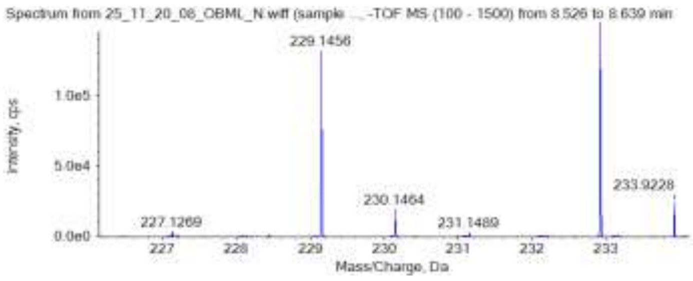

SCIEX



**431.1732 / 8.07** (Mass/FragMass/RT/Isotope/Library/Formula/Ion Ratio)

**Retention Time:** 8.10 minutes
**Precursor m/z :** 431.1732
**Fit (%)** 100.0% **RFit (%)** 35.8%

**Exp RT:** 8.07 minutes
**Analyte Name:**
431.1732 / 8.07

**Collision Energy = 35 ± 15 eV**

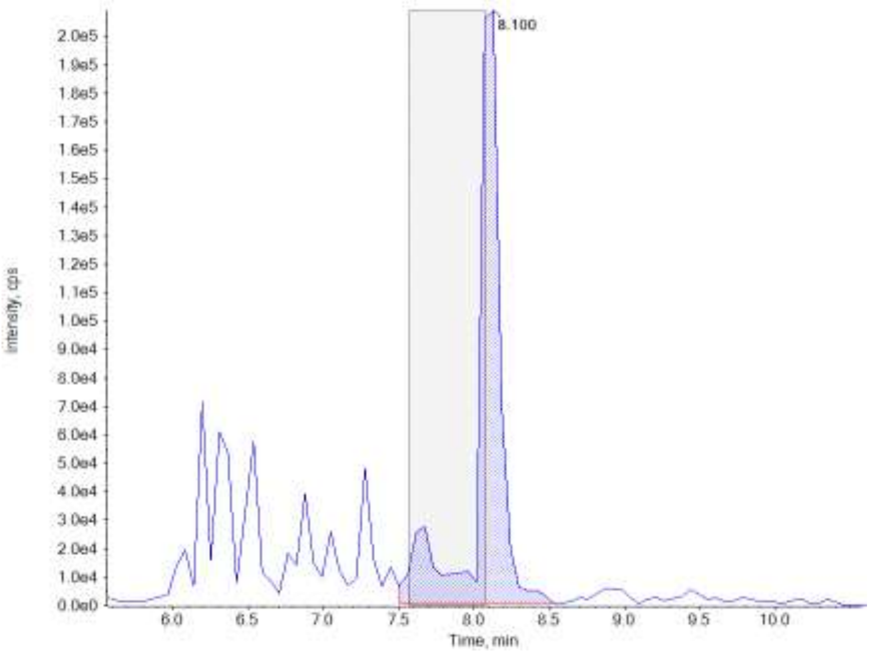


**Acquired / Library MSMS**

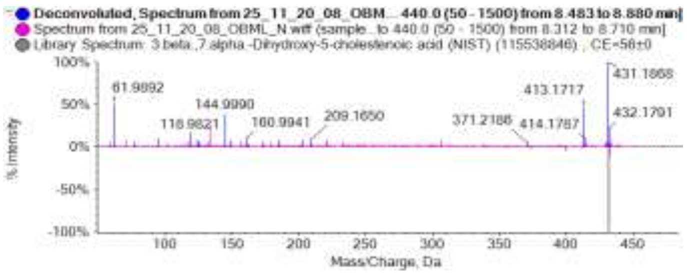


**Acquired / Theoretical MS**

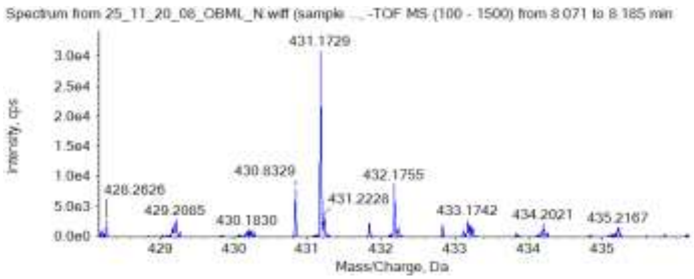

**144.0446 / 8.13** (Mass/FragMass/RT/Isotope/Library/Formula/Ion Ratio)

| | |
|---|---|
| **Retention Time:** 8.13 minutes | **Exp RT:** 8.13 minutes |
| **Precursor m/z :** 144.0447 | **Analyte Name:** |
| **Fit (%)** 100.0% **RFit (%)** 78.9% | 144.0446 / 8.13 |

**Collision Energy = 35 ± 15 eV**

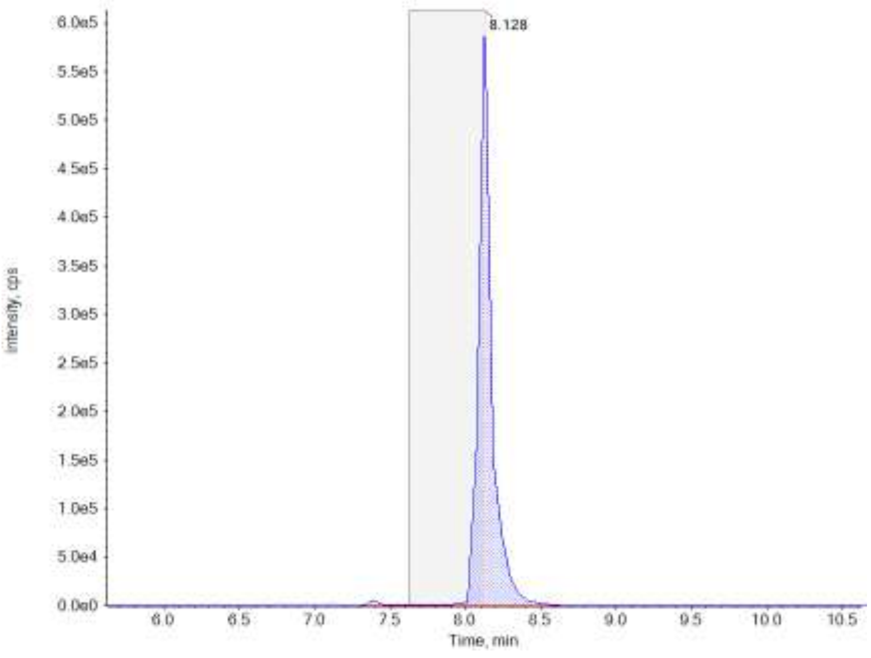


**Acquired / Library MSMS**

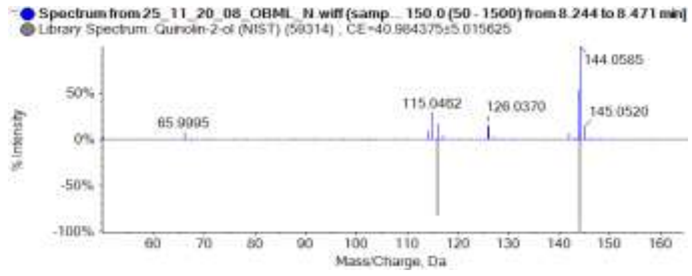


**Acquired / Theoretical MS**

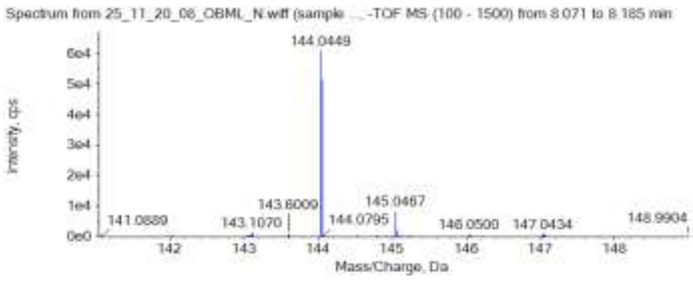

**231.1604 / 8.30** (Mass/FragMass/RT/Isotope/Library/Formula/Ion Ratio)

**Retention Time:** 8.30 minutes
**Precursor m/z :** 231.1604
**Fit (%)** 96.6% **RFit (%)** 93.5%

**Exp RT:** 8.30 minutes
**Analyte Name:**
231.1604 / 8.30

**Collision Energy = 35 ± 15 eV**

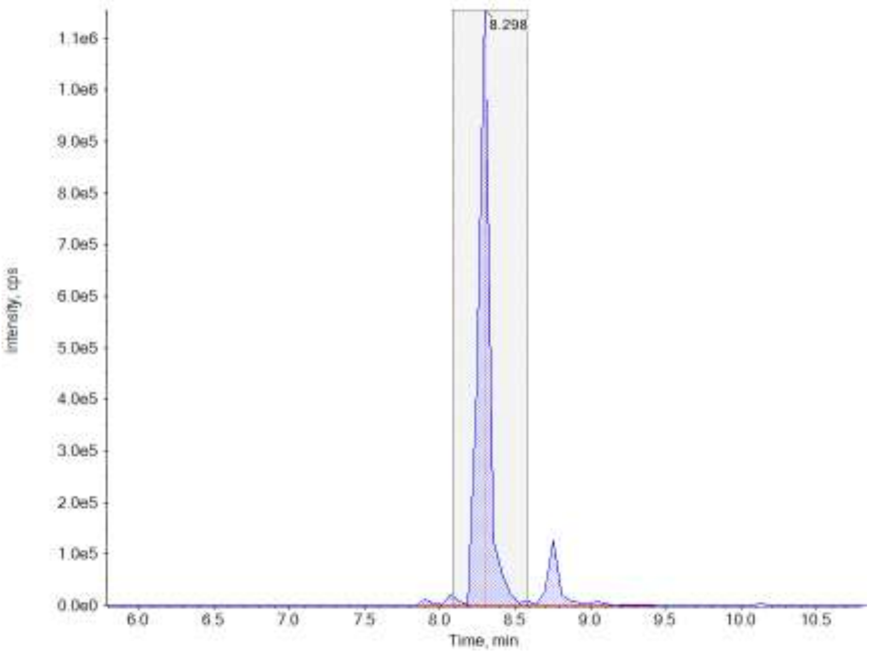


Acquired / Library MSMS

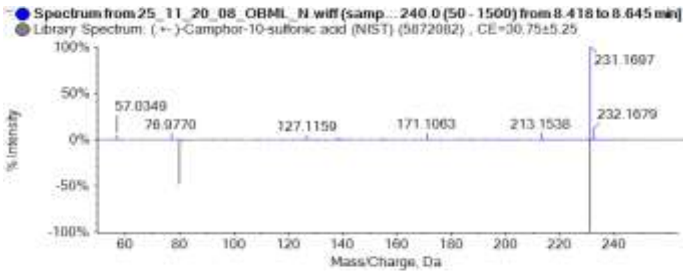


Acquired / Theoretical MS

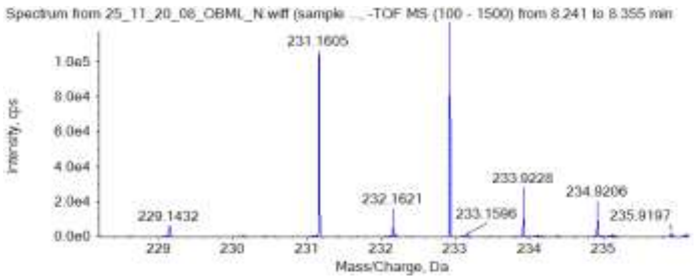

## 173.1181 / 8.36 (Mass/FragMass/RT/Isotope/Library/Formula/Ion Ratio)

**Retention Time:** 8.36 minutes
**Precursor m/z :** 173.1181
**Fit (%)** 65.6% **RFit (%)** 100.0%

**Exp RT:** 8.36 minutes
**Analyte Name:**
173.1181 / 8.36

**Collision Energy = 35 ± 15 eV**

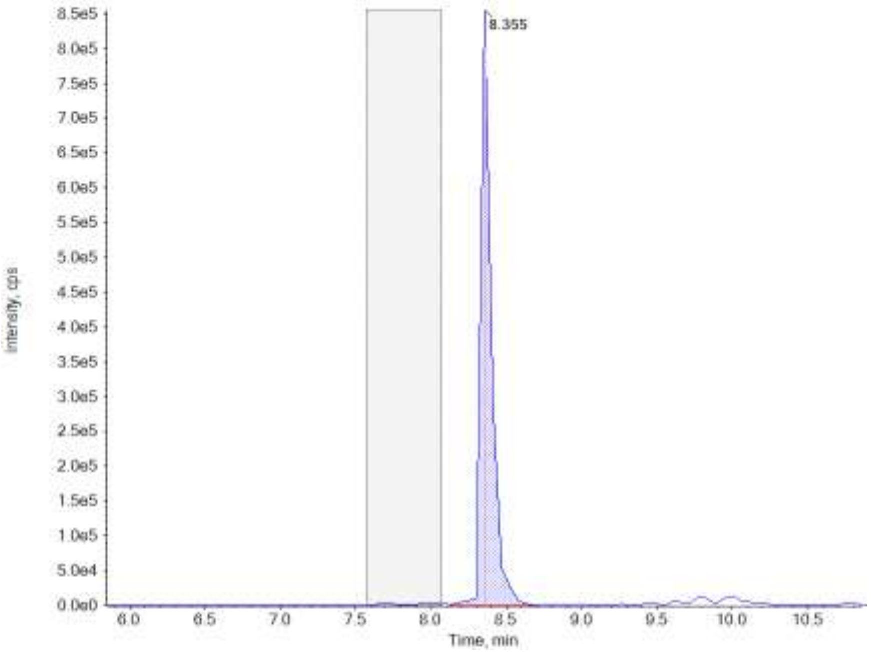


**Acquired / Library MSMS**

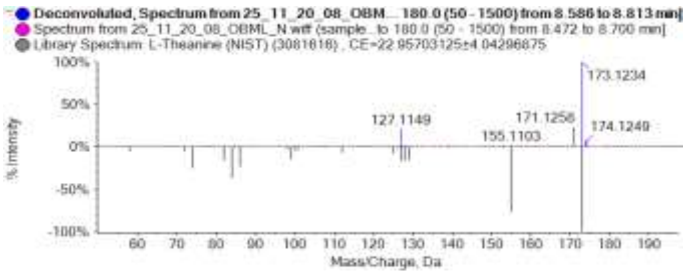


**Acquired / Theoretical MS**

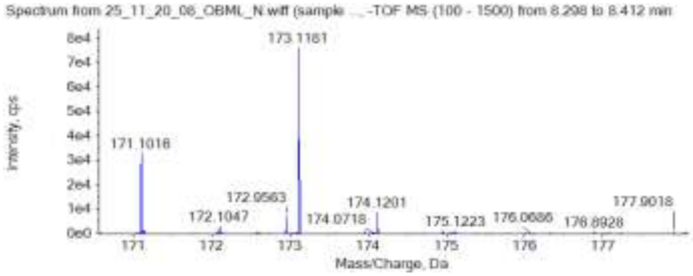

**171.1015 / 8.41** (Mass/FragMass/RT/Isotope/Library/Formula/Ion Ratio)

| | |
|---|---|
| **Retention Time:** 8.41 minutes | **Exp RT:** 8.41 minutes |
| **Precursor m/z :** 171.1015 | **Analyte Name:** |
| **Fit (%)** 100.0% **RFit (%)** 24.8% | 171.1015 / 8.41 |

**Collision Energy = 35 ± 15 eV**

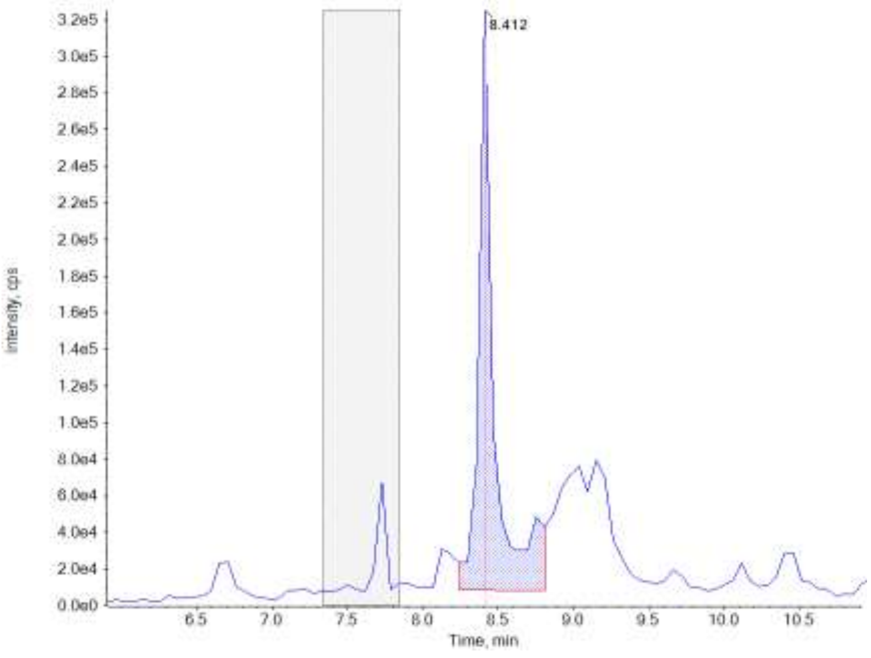


**Acquired / Library MSMS**

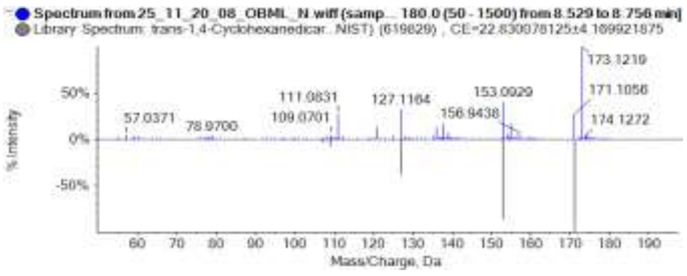


**Acquired / Theoretical MS**

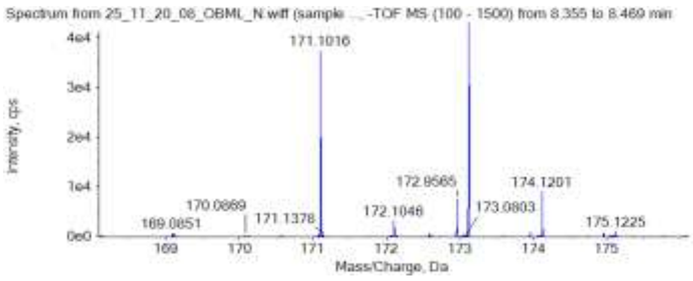

## 381.1544 / 8.41 (Mass/FragMass/RT/Isotope/Library/Formula/Ion Ratio)

**Retention Time:** 8.41 minutes
**Precursor m/z :** 381.1544
**Fit (%)** 47.5% **RFit (%)** 88.2%

**Exp RT:** 8.41 minutes
**Analyte Name:**
381.1544 / 8.41

**Collision Energy = 35 ± 15 eV**

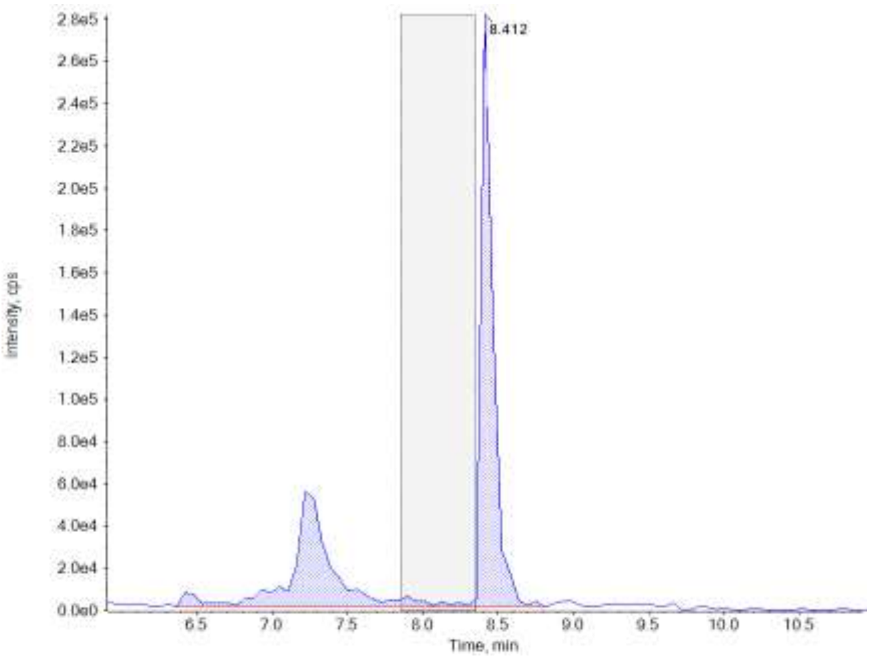


**Acquired / Library MSMS**

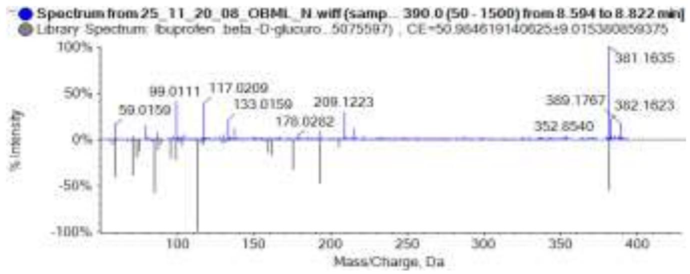


**Acquired / Theoretical MS**

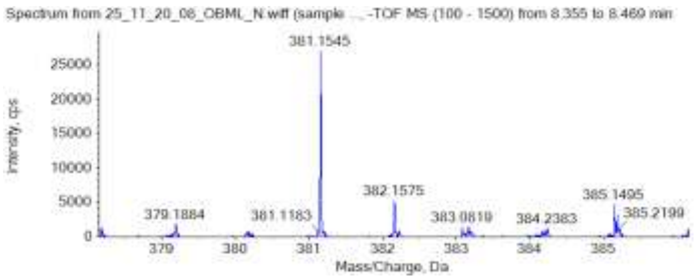

**229.1454 / 8.58** (Mass/FragMass/RT/Isotope/Library/Formula/Ion Ratio)

**Retention Time:** 8.30 minutes
**Precursor m/z :** 229.1454
**Fit (%)** 100.0% **RFit (%)** 100.0%

**Exp RT:** 8.58 minutes
**Analyte Name:**
229.1454 / 8.58

**Collision Energy = 35 ± 15 eV**

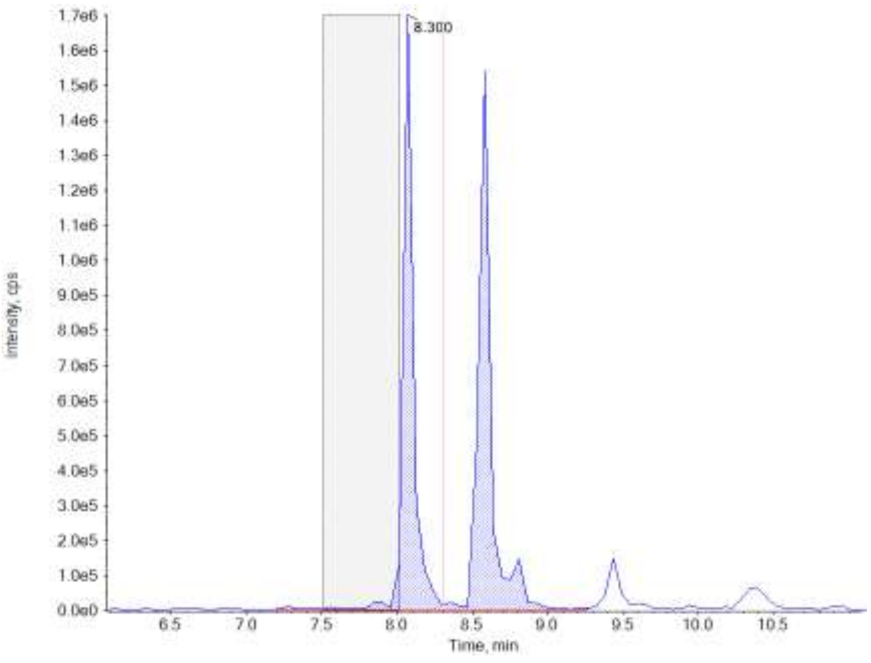


**Acquired / Library MSMS**

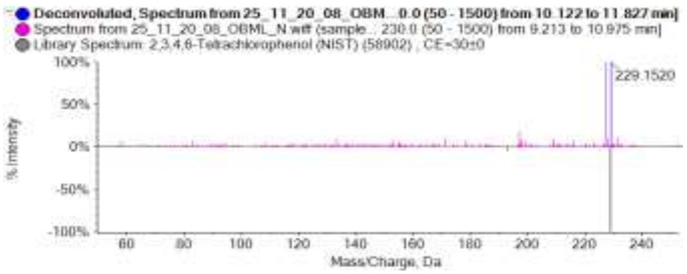


**Acquired / Theoretical MS**

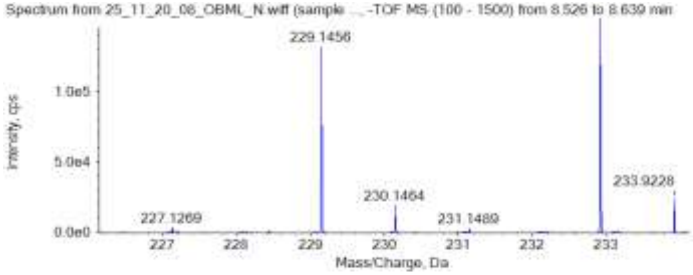

**281.0808 / 9.04 [M+K-2H]-** (Mass/FragMass/RT/Isotope/Library/Formula/Ion Ratio)

| | |
|---|---|
| **Retention Time:** 9.04 minutes | **Exp RT:** 9.04 minutes |
| **Precursor m/z :** 281.0808 | **Analyte Name:** |
| **Fit (%)** 85.5% **RFit (%)** 97.9% | 281.0808 / 9.04 [M+K-2H]- |

**Collision Energy = 35 ± 15 eV**

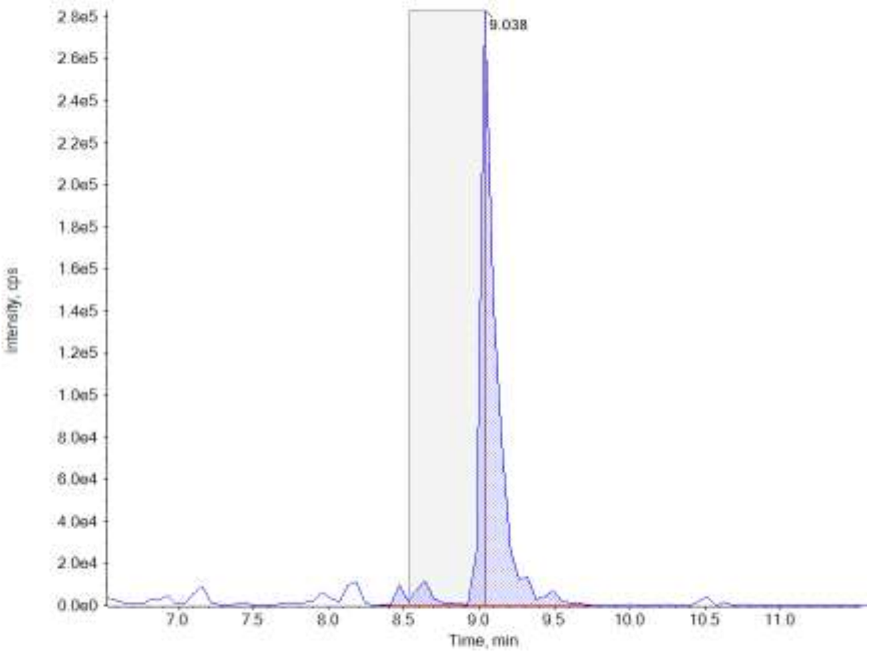


**Acquired / Library MSMS**

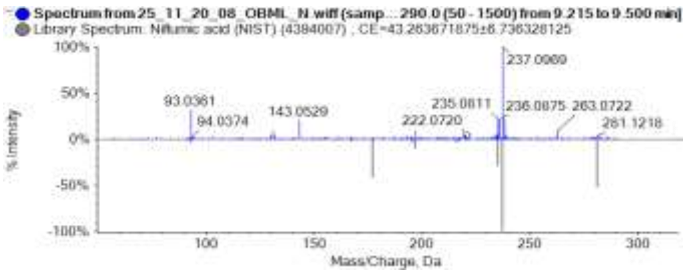


**Acquired / Theoretical MS**

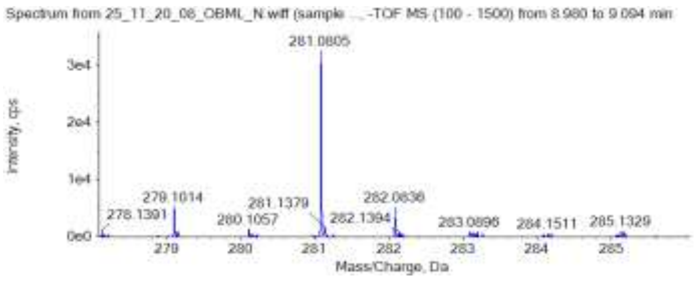

**343.2146 / 8.64** (Mass/FragMass/RT/Isotope/Library/Formula/Ion Ratio)

**Retention Time:** 8.76 minutes
**Precursor m/z :** 343.2146
**Fit (%)** 99.0% **RFit (%)** 25.5%

**Exp RT:** 8.64 minutes
**Analyte Name:**
343.2146 / 8.64

**Collision Energy = 35 ± 15 eV**

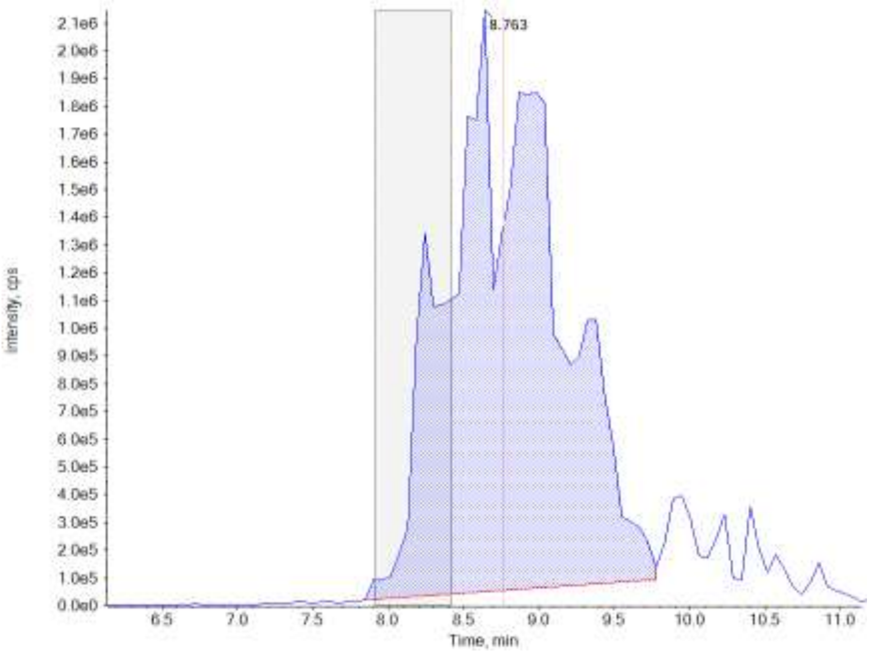


**Acquired / Library MSMS**

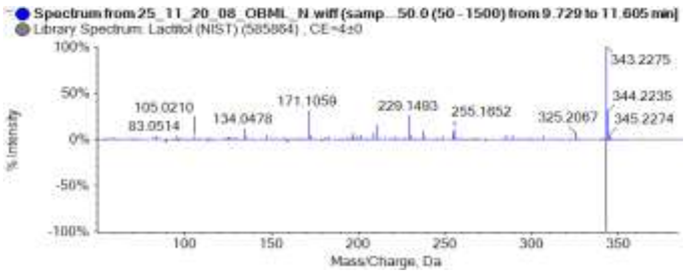


**Acquired / Theoretical MS**

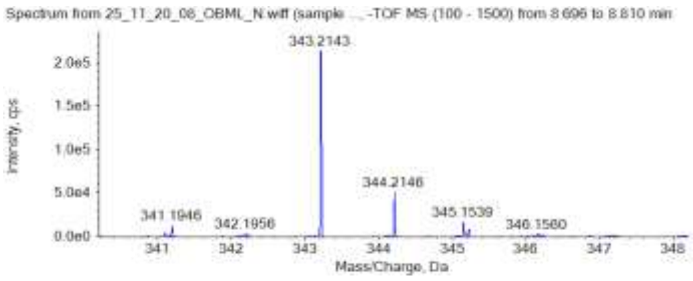

## 113.0603 / 8.70 [M-H]- (Mass/FragMass/RT/Isotope/Library/Formula/Ion Ratio)

**Retention Time:** 8.69 minutes
**Precursor m/z :** 113.0603
**Fit (%)** 97.4% **RFit (%)** 60.3%

**Exp RT:** 8.70 minutes
**Analyte Name:**
113.0603 / 8.70 [M-H]-

**Collision Energy = 35 ± 15 eV**

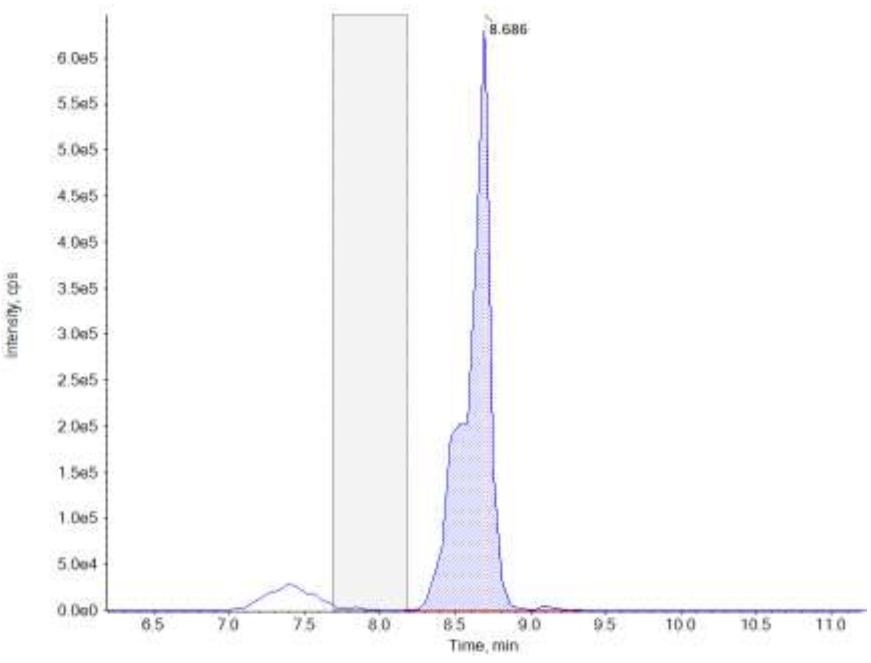


**Acquired / Library MSMS**

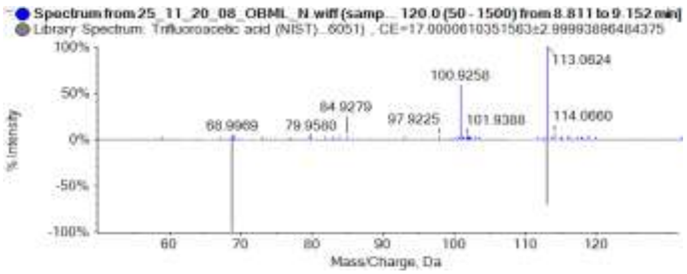


**Acquired / Theoretical MS**

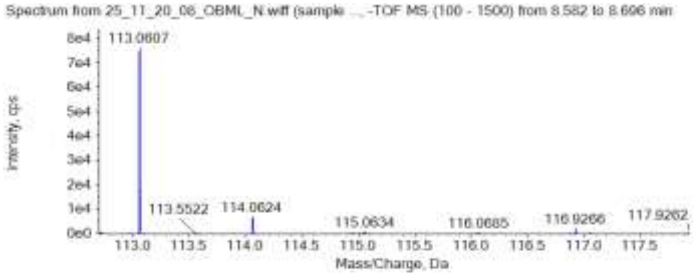

**299.1844 / 8.81** (Mass/FragMass/RT/Isotope/Library/Formula/Ion Ratio)

| | |
|---|---|
| **Retention Time:** 8.84 minutes | **Exp RT:** 8.81 minutes |
| **Precursor m/z :** 299.1844 | **Analyte Name:** |
| **Fit (%)** 71.5% **RFit (%)** 80.5% | 299.1844 / 8.81 |

**Collision Energy = 35 ± 15 eV**

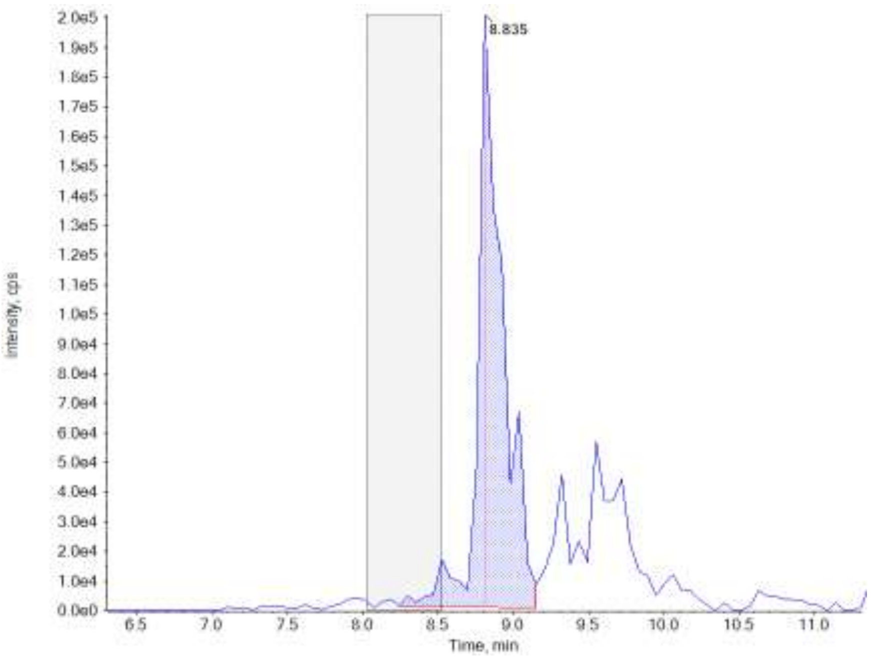


**Acquired / Library MSMS**

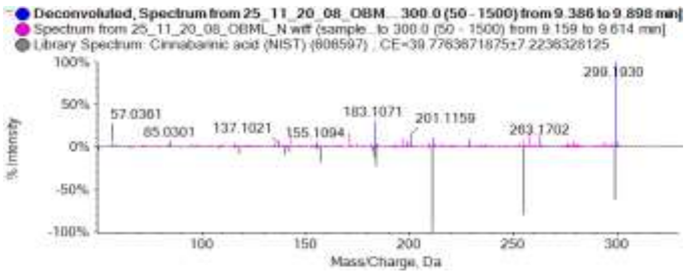


**Acquired / Theoretical MS**

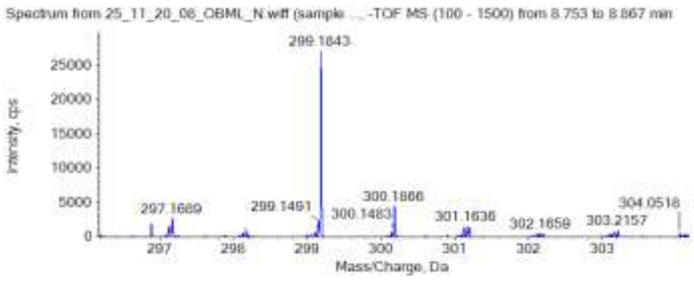

## 153.0902 / 9.09 (Mass/FragMass/RT/Isotope/Library/Formula/Ion Ratio)

**Retention Time:** 9.09 minutes
**Precursor m/z :** 153.0902
**Fit (%)** 69.6% **RFit (%)** 100.0%

**Exp RT:** 9.09 minutes
**Analyte Name:**
153.0902 / 9.09

**Collision Energy = 35 ± 15 eV**

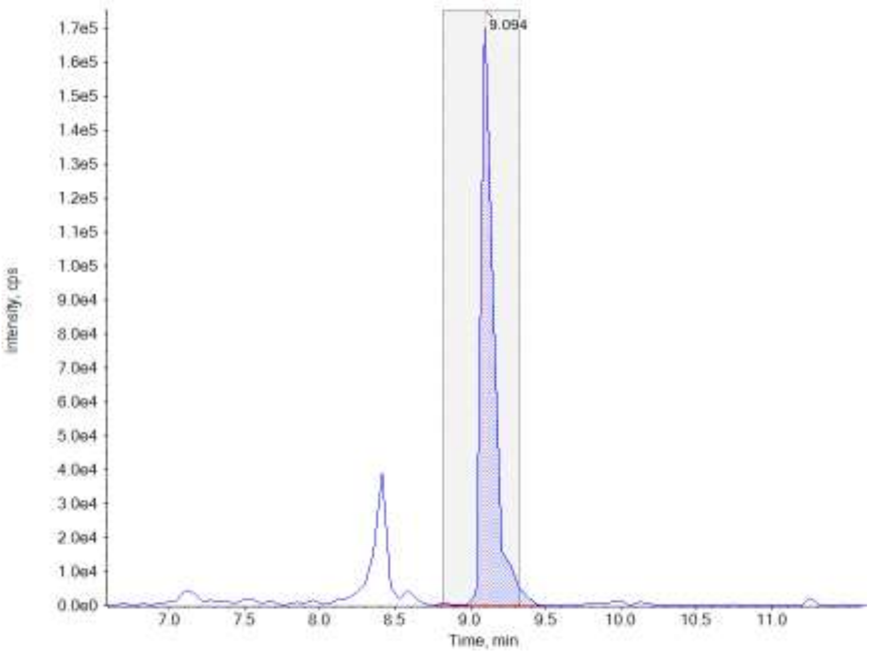


**Acquired / Library MSMS**

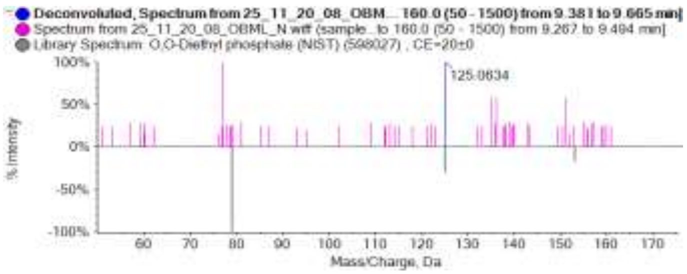


**Acquired / Theoretical MS**

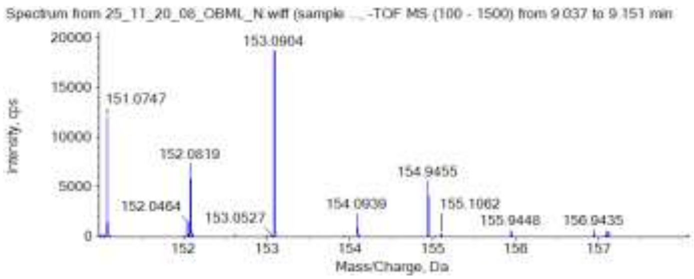

## 263.1306 / 9.09 (Mass/FragMass/RT/Isotope/Library/Formula/Ion Ratio)

| | |
|---|---|
| **Retention Time:** 9.10 minutes<br>**Precursor m/z :** 263.1306<br>**Fit (%)** 98.4% **RFit (%)** 99.8% | **Exp RT:** 9.09 minutes<br>**Analyte Name:**<br>263.1306 / 9.09 |

**Collision Energy = 35 ± 15 eV**

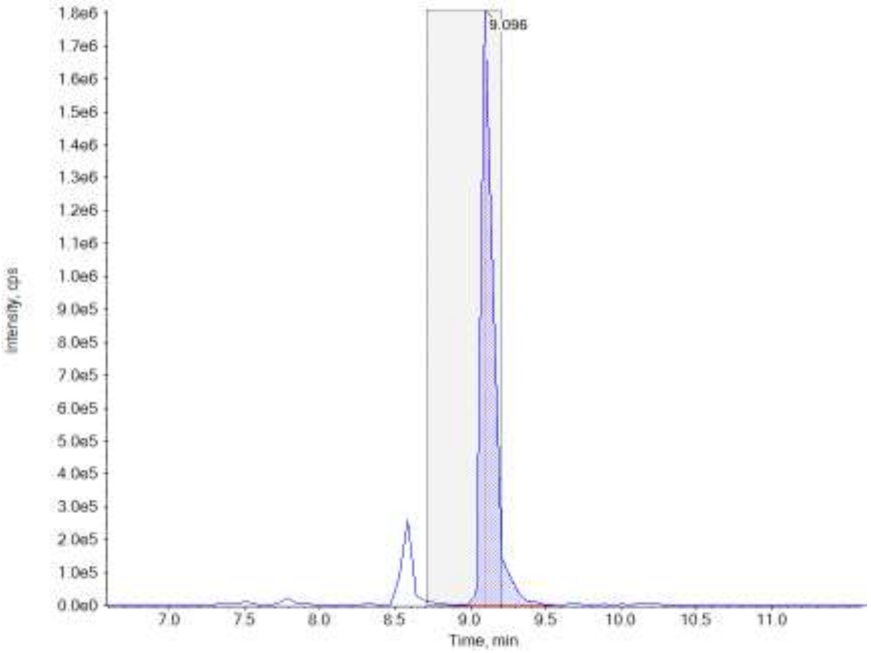

**Acquired / Library MSMS**

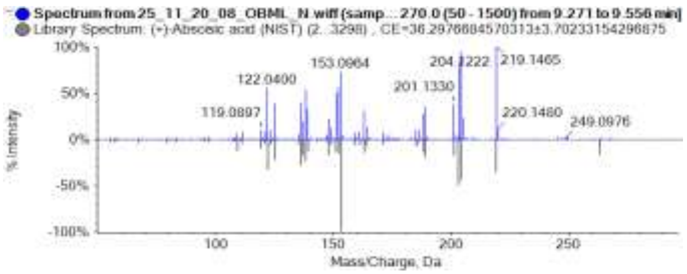

**Acquired / Theoretical MS**

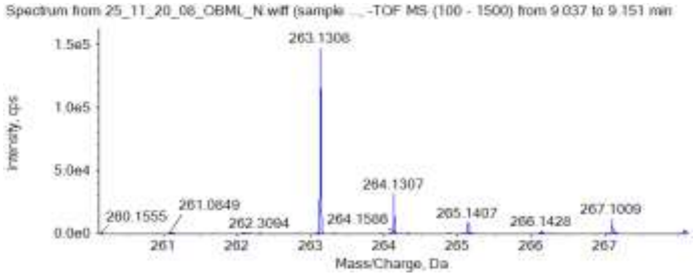

## 493.2285 / 9.26  [M+FA-H]-

(Mass/FragMass/RT/Isotope/Library/Formula/Ion Ratio)

| | |
|---|---|
| **Retention Time:** 9.28 minutes | **Exp RT:** 9.26 minutes |
| **Precursor m/z :** 493.2285 | **Analyte Name:** |
| **Fit (%)** 76.6%   **RFit (%)** 62.8% | 493.2285 / 9.26  [M+FA-H]- |

**Collision Energy = 35 ± 15 eV**

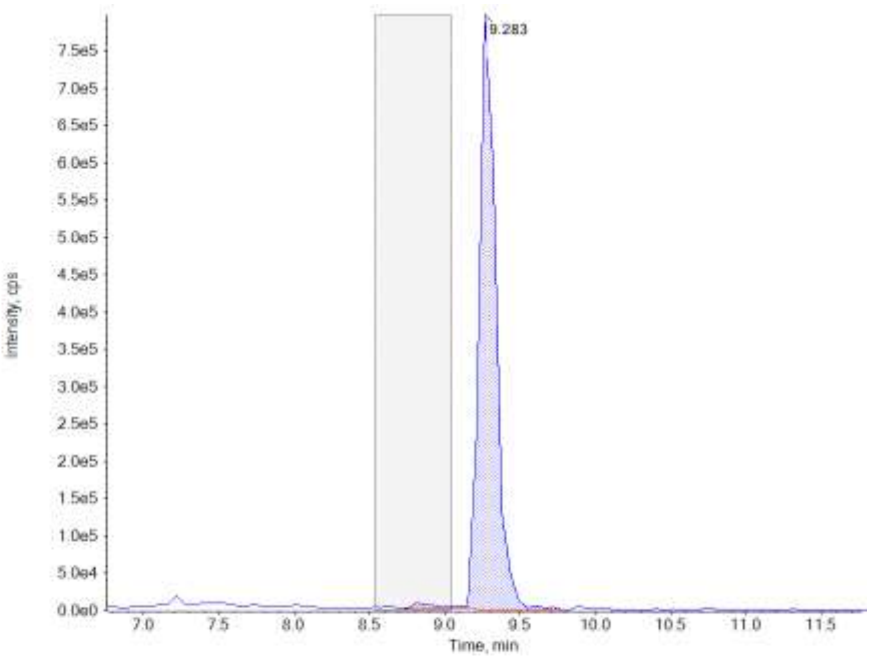


**Acquired / Library MSMS**

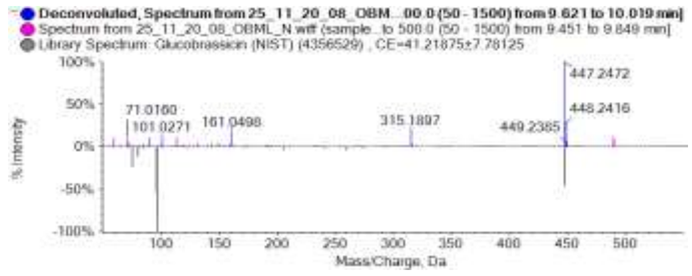


**Acquired / Theoretical MS**

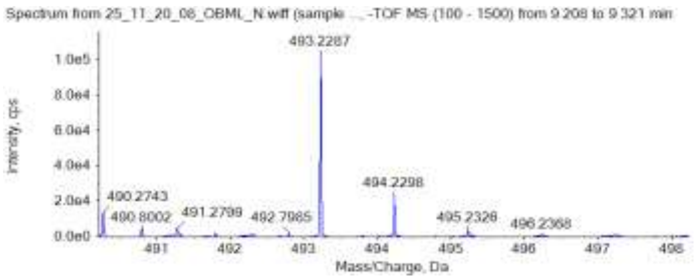

## 447.2230 / 9.26 [M-H]- (Mass/FragMass/RT/Isotope/Library/Formula/Ion Ratio)

| | |
|---|---|
| **Retention Time:** 9.29 minutes | **Exp RT:** 9.26 minutes |
| **Precursor m/z :** 447.2230 | **Analyte Name:** |
| **Fit (%)** 77.4% **RFit (%)** 62.2% | 447.2230 / 9.26 [M-H]- |

**Collision Energy = 35 ± 15 eV**

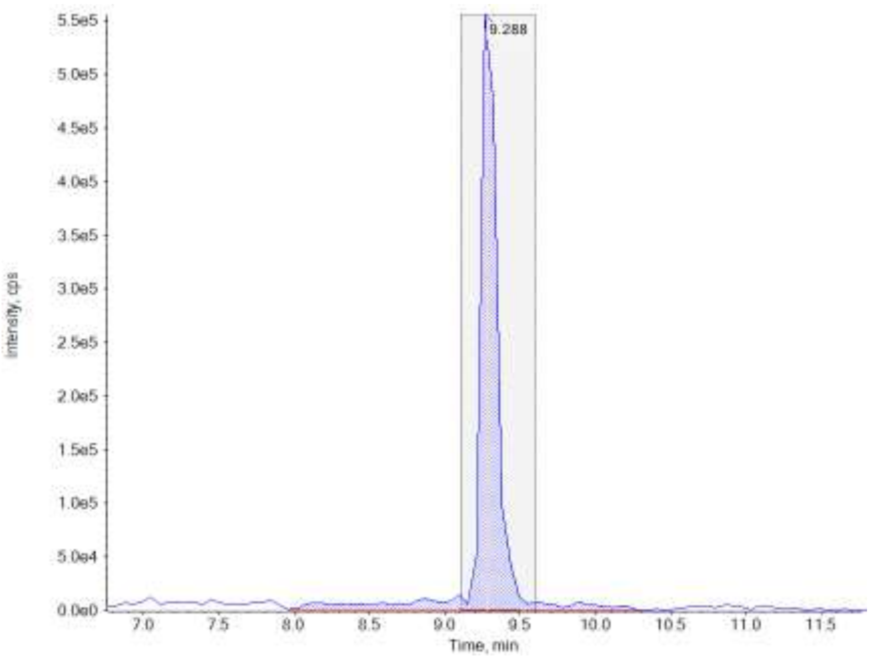


**Acquired / Library MSMS**

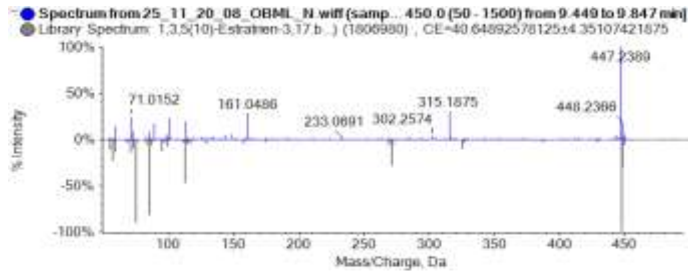


**Acquired / Theoretical MS**

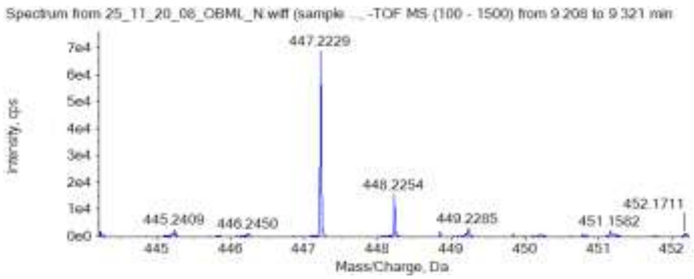

## 329.1601 / 9.38 (Mass/FragMass/RT/Isotope/Library/Formula/Ion Ratio)

**Retention Time:** 9.38 minutes
**Precursor m/z :** 329.1601
**Fit (%)** 78.4% **RFit (%)** 91.9%

**Exp RT:** 9.38 minutes
**Analyte Name:**
329.1601 / 9.38

**Collision Energy = 35 ± 15 eV**

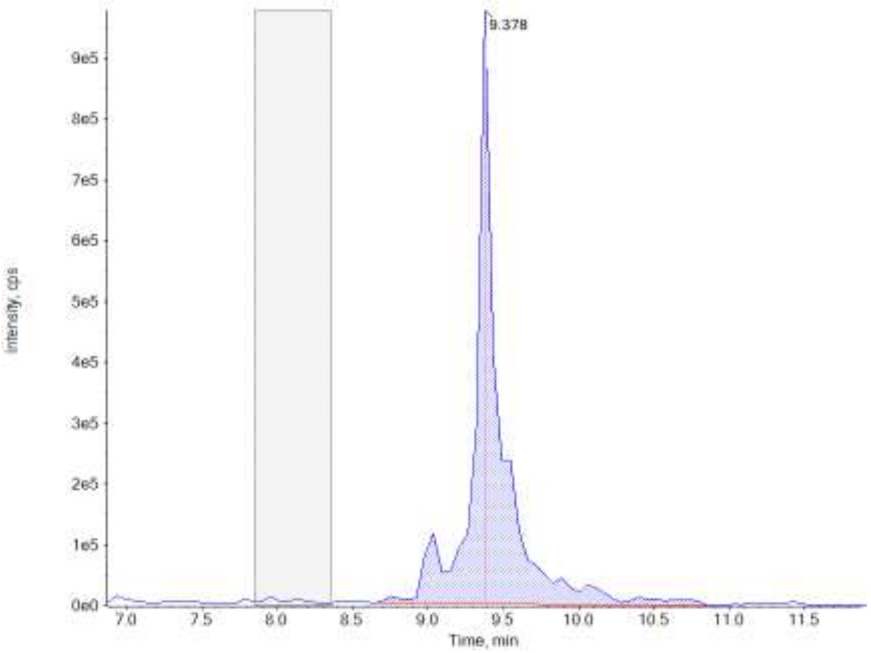


**Acquired / Library MSMS**

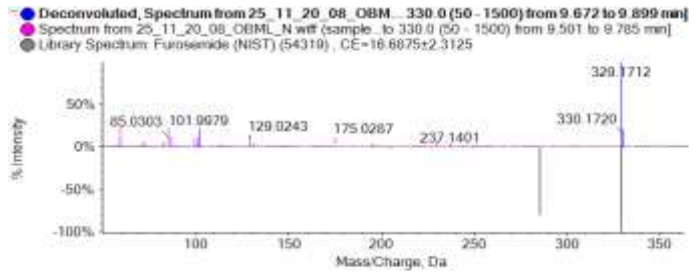


**Acquired / Theoretical MS**

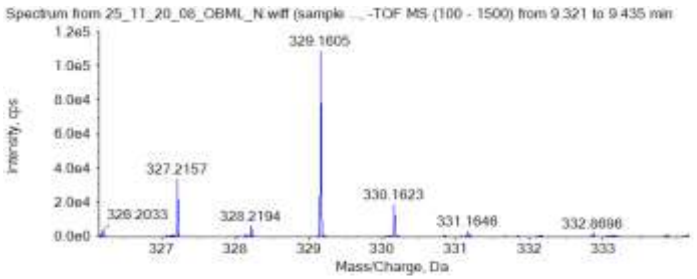

**383.1340 / 9.43** (Mass/FragMass/RT/Isotope/Library/Formula/Ion Ratio)

| | |
|---|---|
| **Retention Time:** 9.43 minutes | **Exp RT:** 9.44 minutes |
| **Precursor m/z :** 383.1340 | **Analyte Name:** |
| **Fit (%)** 25.5% **RFit (%)** 100.0% | 383.1340 / 9.43 |

**Collision Energy = 35 ± 15 eV**

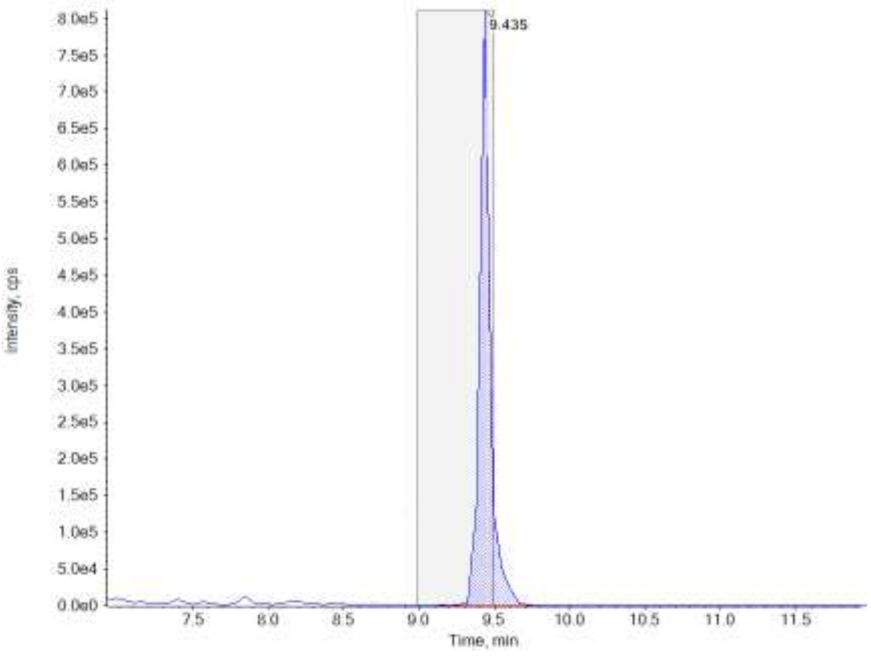

**Acquired / Library MSMS**

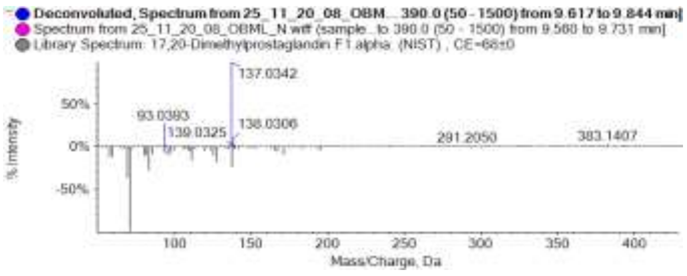

**Acquired / Theoretical MS**

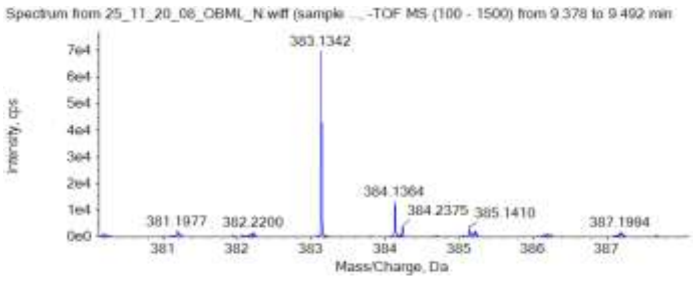

**147.0454 / 9.49** (Mass/FragMass/RT/Isotope/Library/Formula/Ion Ratio)

**Retention Time:** 9.49 minutes
**Precursor m/z :** 147.0454
**Fit (%)** 99.4% **RFit (%)** 100.0%

**Exp RT:** 9.49 minutes
**Analyte Name:**
147.0454 / 9.49

**Collision Energy = 35 ± 15 eV**

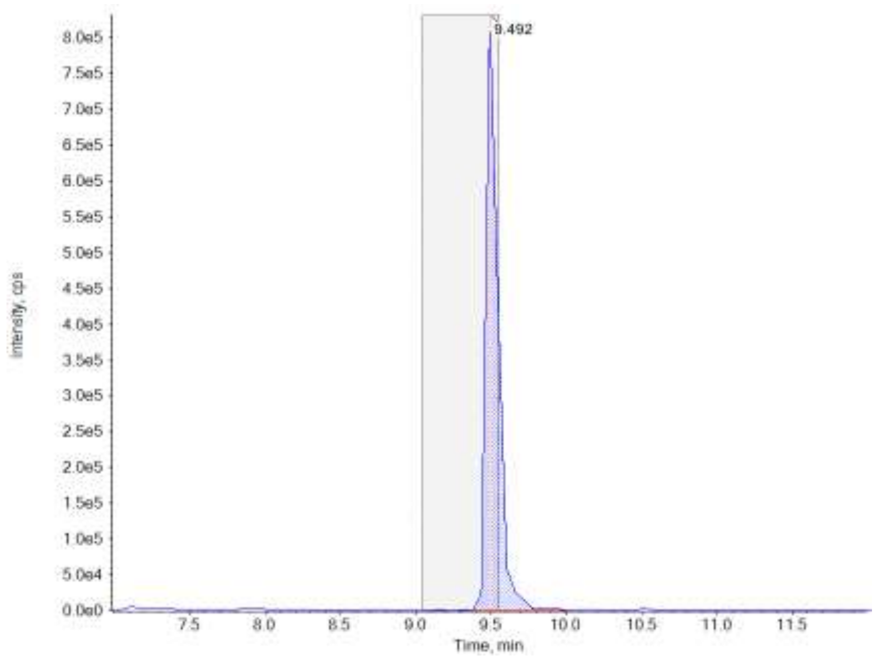


**Acquired / Library MSMS**

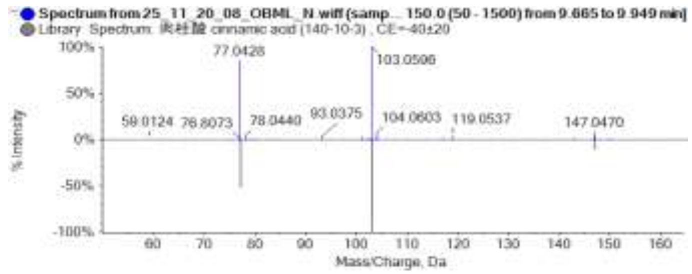


**Acquired / Theoretical MS**

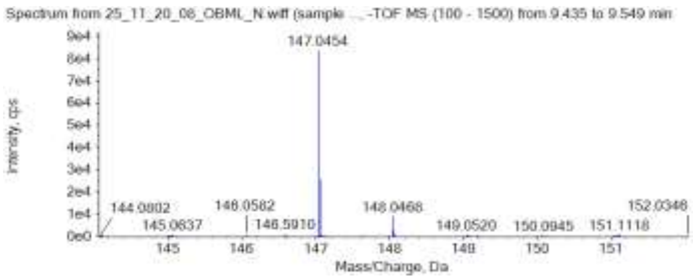

## 185.1171 / 9.49 (Mass/FragMass/RT/Isotope/Library/Formula/Ion Ratio)

**Retention Time:** 9.49 minutes
**Precursor m/z :** 185.1172
**Fit (%)** 95.4% **RFit (%)** 93.6%

**Exp RT:** 9.49 minutes
**Analyte Name:**
185.1171 / 9.49

**Collision Energy = 35 ± 15 eV**

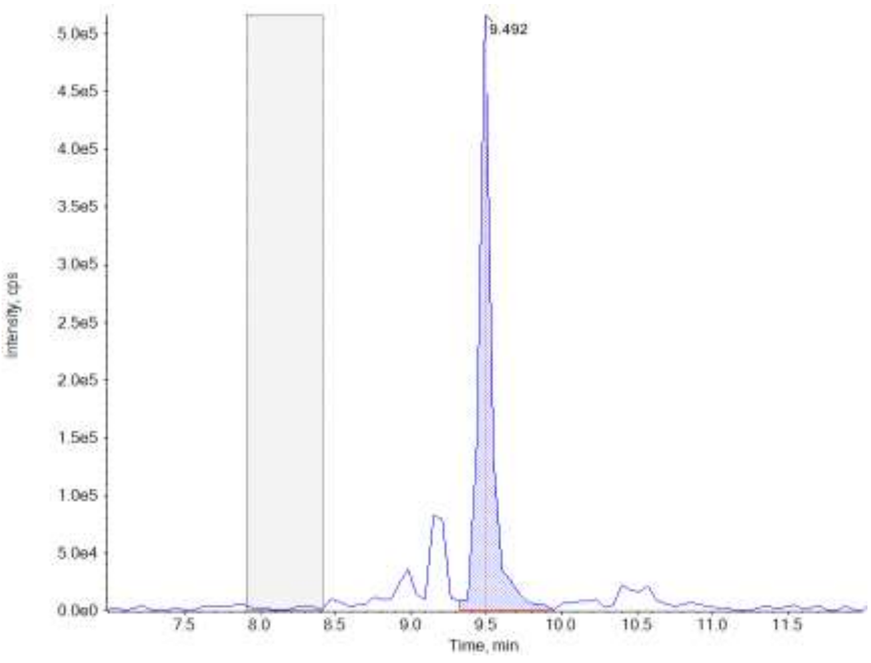


**Acquired / Library MSMS**

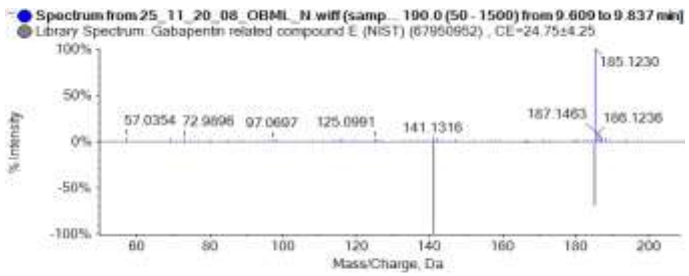


**Acquired / Theoretical MS**

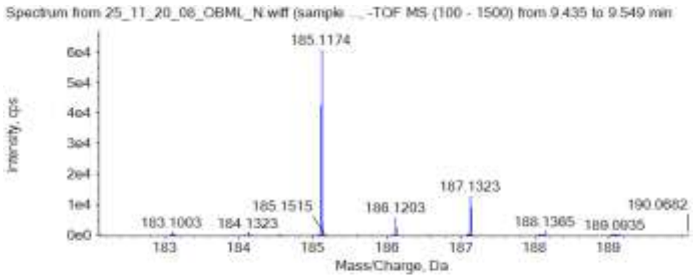

## 449.2736 / 9.61 (Mass/FragMass/RT/Isotope/Library/Formula/Ion Ratio)

**Retention Time:** 9.62 minutes **Exp RT:** 9.61 minutes
**Precursor m/z :** 449.2736 **Analyte Name:**
**Fit (%)** 92.6% **RFit (%)** 93.1% 449.2736 / 9.61

**Collision Energy = 35 ± 15 eV**

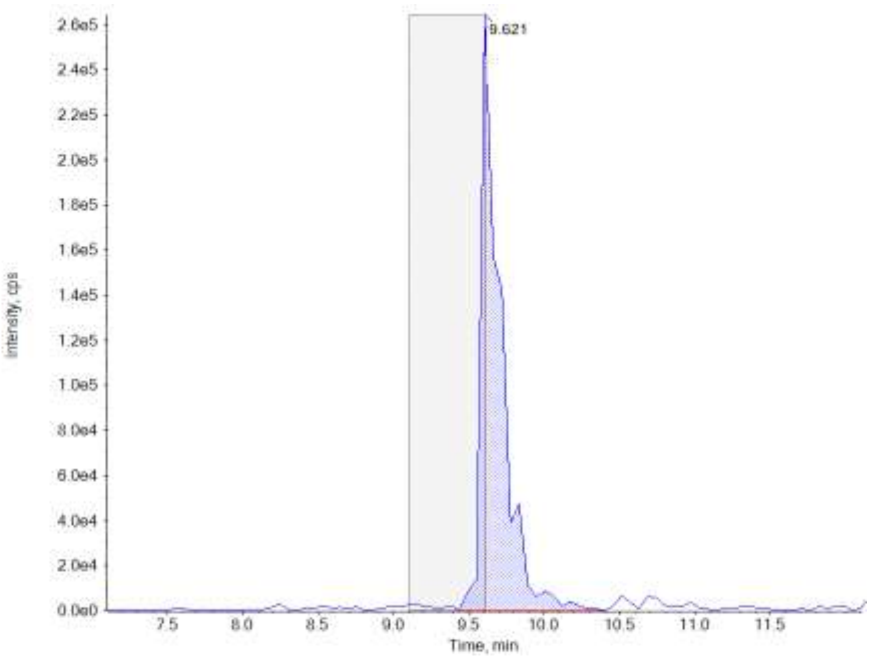


Acquired / Library MSMS

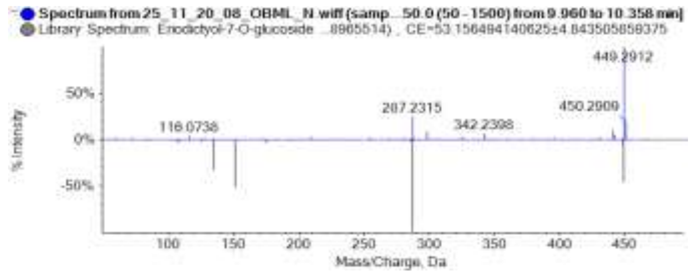


Acquired / Theoretical MS

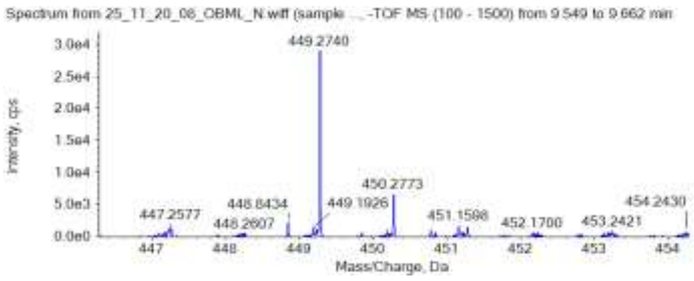

## 683.4034 / 9.61 [M+FA-H]- (Mass/FragMass/RT/Isotope/Library/Formula/Ion Ratio)

**Retention Time:** 9.61 minutes
**Precursor m/z :** 683.4034
**Fit (%)** 86.3% **RFit (%)** 99.8%

**Exp RT:** 9.61 minutes
**Analyte Name:**
683.4034 / 9.61 [M+FA-H]-

**Collision Energy = 35 ± 15 eV**

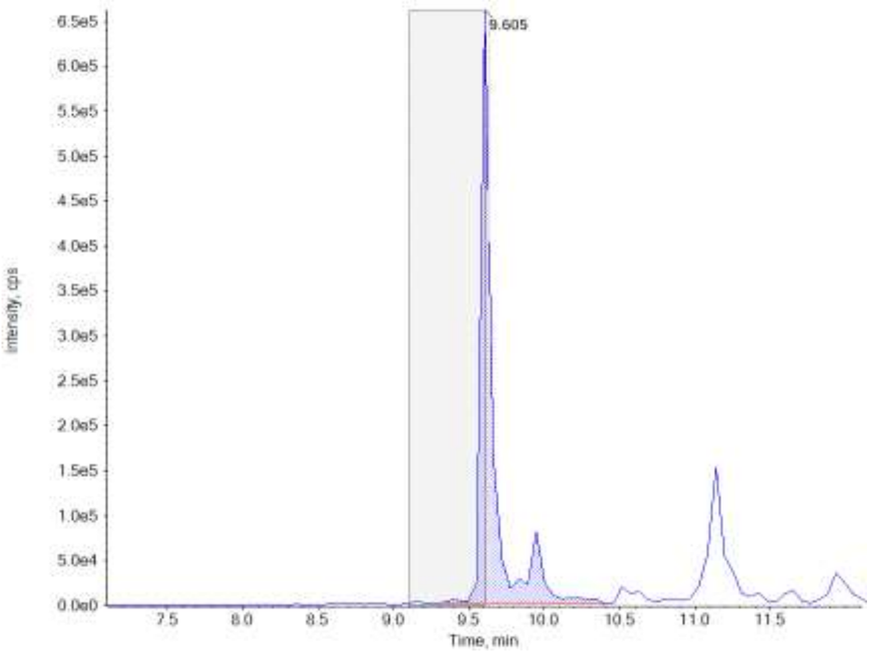


**Acquired / Library MSMS**

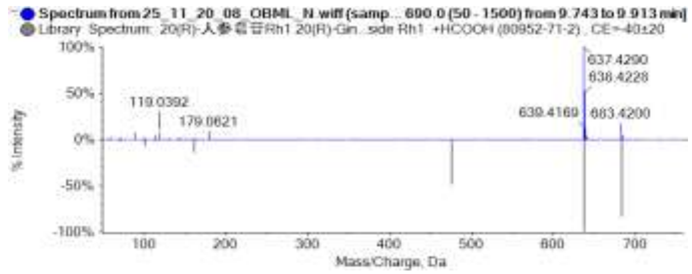


**Acquired / Theoretical MS**

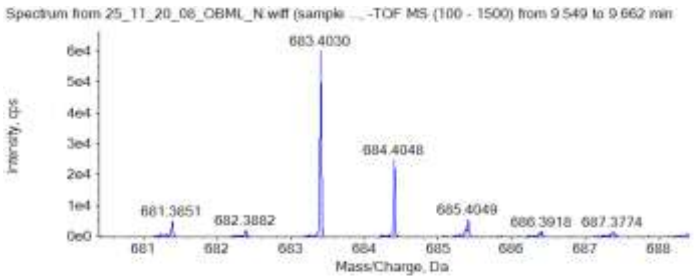

## 637.3950 / 9.61 [M-H]- (Mass/FragMass/RT/Isotope/Library/Formula/Ion Ratio)

| | |
|---|---|
| **Retention Time:** 9.61 minutes | **Exp RT:** 9.61 minutes |
| **Precursor m/z :** 637.3950 | **Analyte Name:** |
| **Fit (%)** 66.5% **RFit (%)** 99.7% | 637.3950 / 9.61 [M-H]- |

**Collision Energy = 35 ± 15 eV**

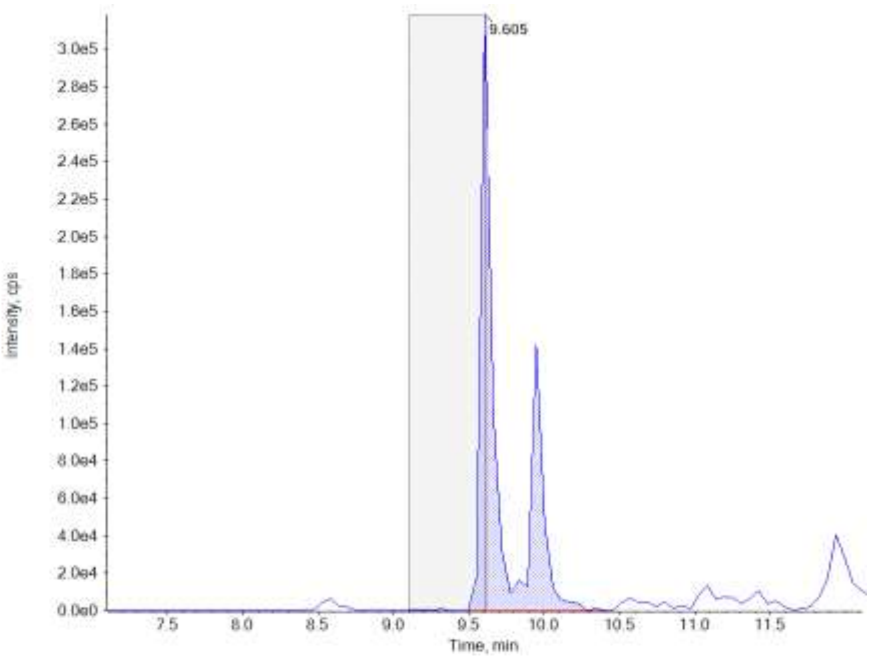


**Acquired / Library MSMS**

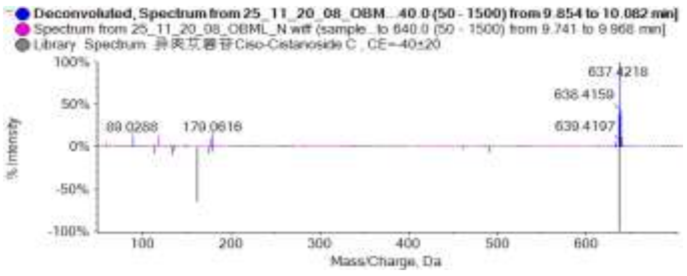


**Acquired / Theoretical MS**

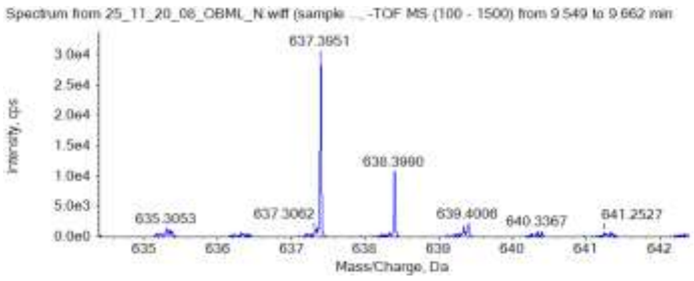

**225.1144 / 9.83** (Mass/FragMass/RT/Isotope/Library/Formula/Ion Ratio)

**Retention Time:** 9.81 minutes
**Precursor m/z :** 225.1144
**Fit (%)** 97.0% **RFit (%)** 47.6%

**Exp RT:** 9.83 minutes
**Analyte Name:**
225.1144 / 9.83

**Collision Energy = 35 ± 15 eV**

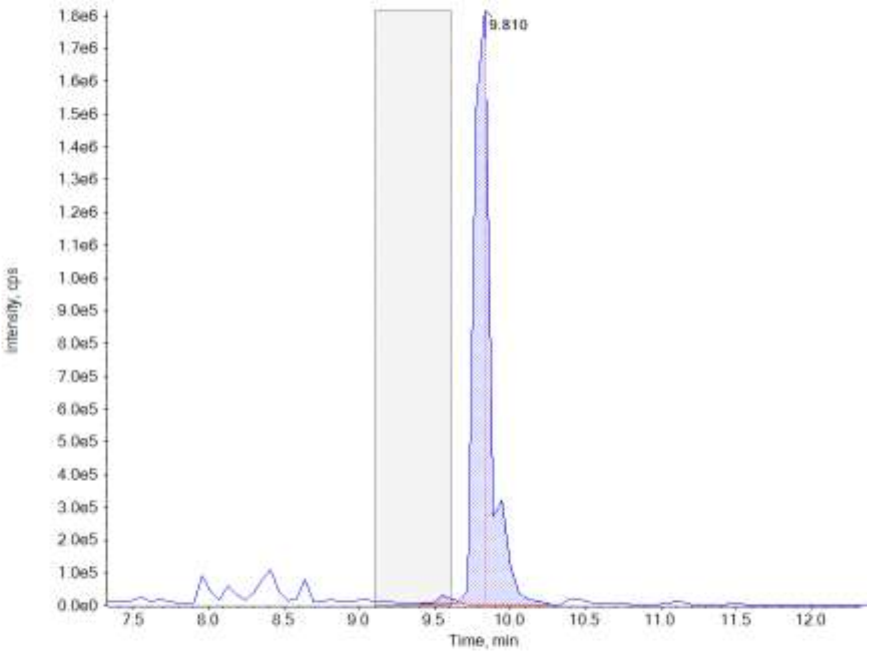


**Acquired / Library MSMS**

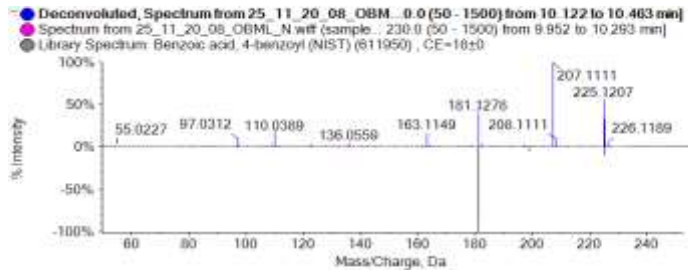


**Acquired / Theoretical MS**

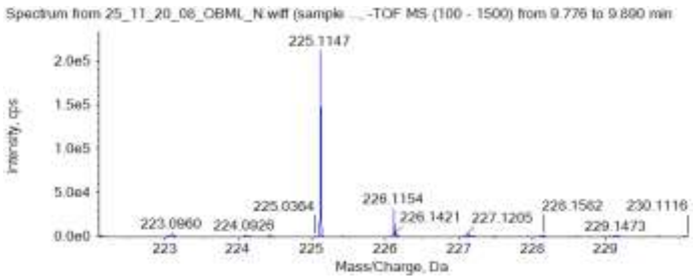

**375.1480 / 9.89** (Mass/FragMass/RT/Isotope/Library/Formula/Ion Ratio)

| | |
|---|---|
| **Retention Time:** 9.89 minutes<br>**Precursor m/z :** 375.1480<br>**Fit (%)** 49.6% **RFit (%)** 91.3% | **Exp RT:** 9.89 minutes<br>**Analyte Name:**<br>375.1480 / 9.89 |

| | | |
|---|---|---|
| | | **Collision Energy = 35 ± 15 eV** |
| 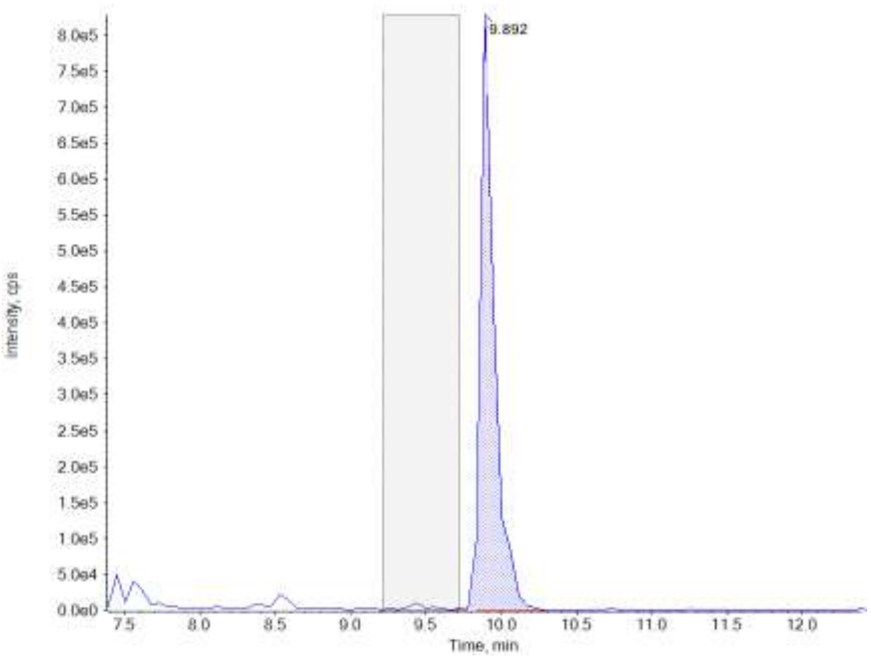 | **Acquired / Library MSMS** | 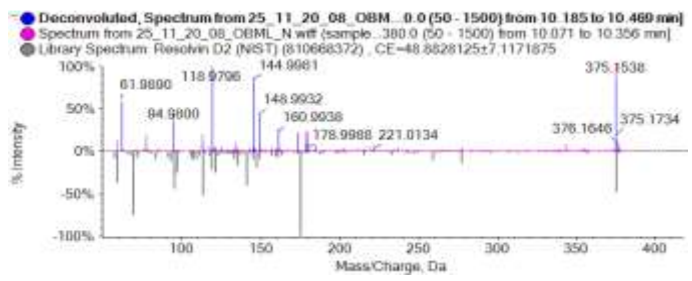 |
| | **Acquired / Theoretical MS** | 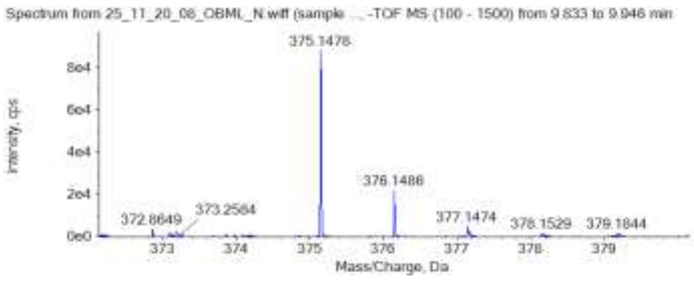 |

## 199.1336 / 9.95 (Mass/FragMass/RT/Isotope/Library/Formula/Ion Ratio)

**Retention Time:** 9.97 minutes
**Precursor m/z :** 199.1336
**Fit (%)** 99.0% **RFit (%)** 96.7%

**Exp RT:** 9.95 minutes
**Analyte Name:**
199.1336 / 9.95

**Collision Energy = 35 ± 15 eV**

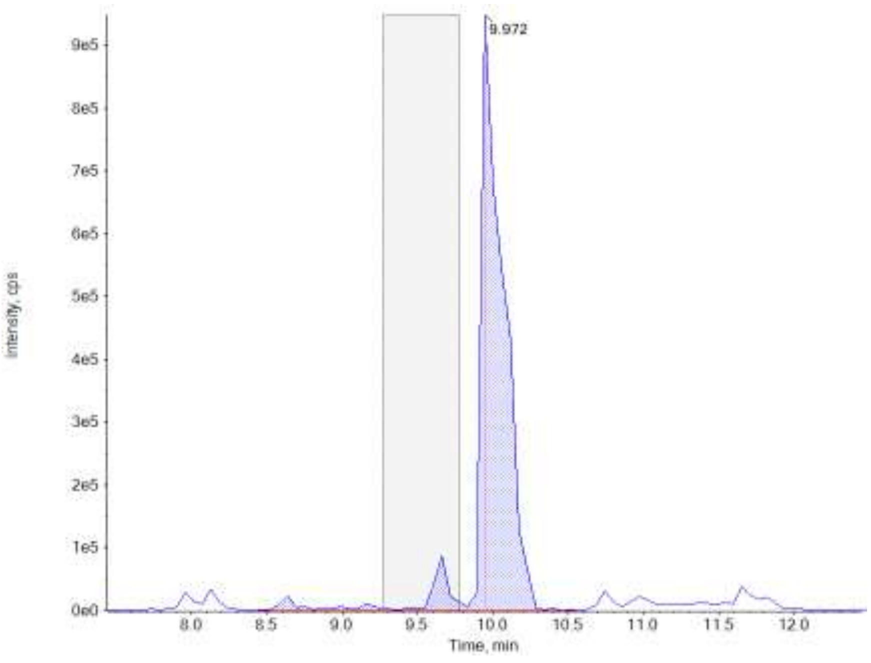


**Acquired / Library MSMS**

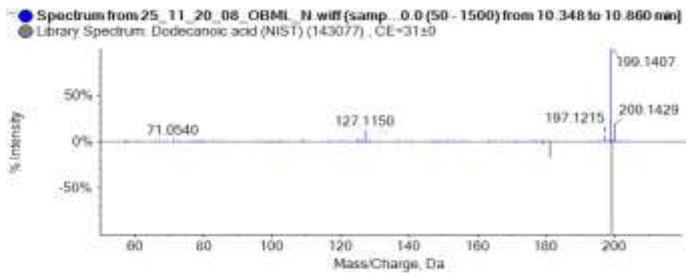


**Acquired / Theoretical MS**

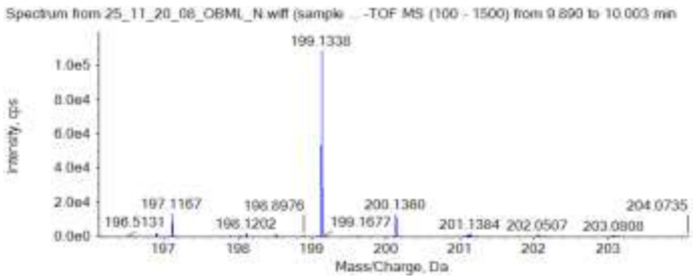

## 341.1949 / 10.00

(Mass/FragMass/RT/Isotope/Library/Formula/Ion Ratio)

**Retention Time:** 9.91 minutes
**Precursor m/z :** 341.1949
**Fit (%)** 78.0% **RFit (%)** 97.5%

**Exp RT:** 10.00 minutes
**Analyte Name:**
341.1949 / 10.00

**Collision Energy = 35 ± 15 eV**

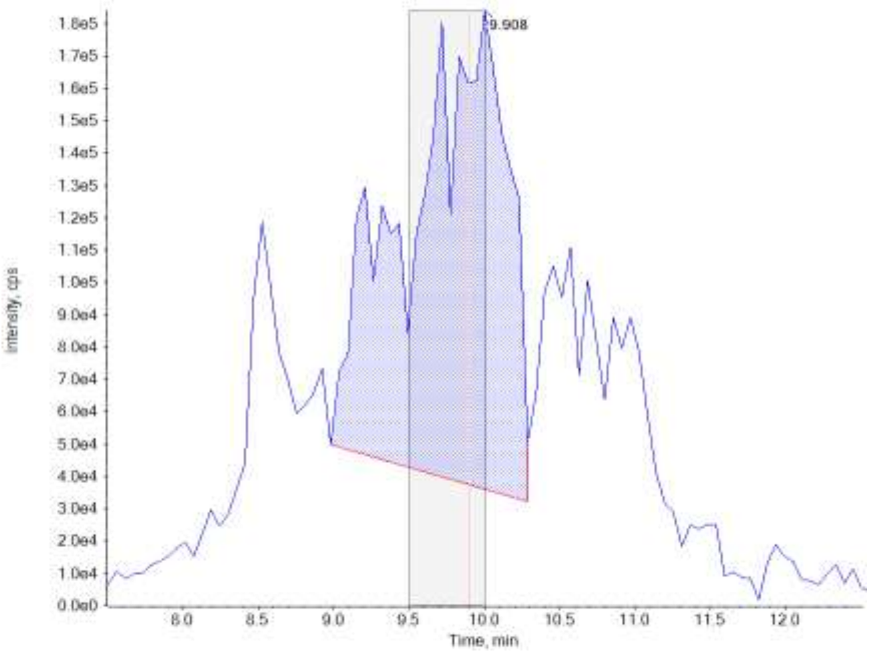


**Acquired / Library MSMS**

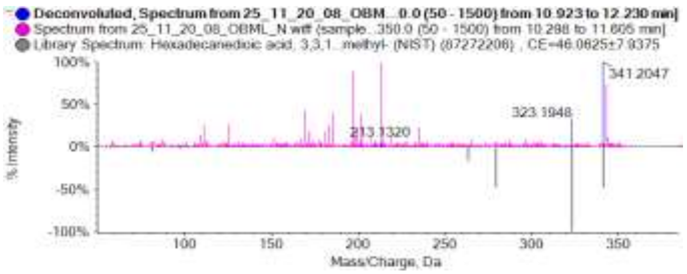


**Acquired / Theoretical MS**

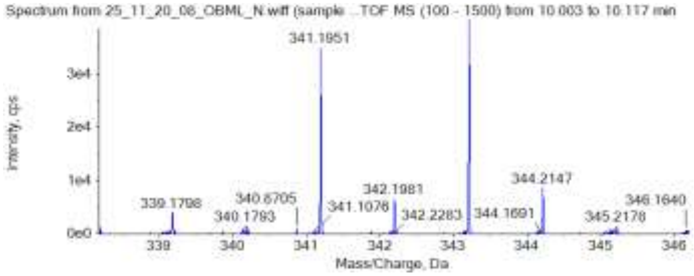

**593.2997 / 10.06** (Mass/FragMass/RT/Isotope/Library/Formula/Ion Ratio)

| | |
|---|---|
| **Retention Time:** 10.06 minutes | **Exp RT:** 10.06 minutes |
| **Precursor m/z :** 593.2997 | **Analyte Name:** |
| **Fit (%)** 86.1% **RFit (%)** 91.2% | 593.2997 / 10.06 |

**Collision Energy = 35 ± 15 eV**

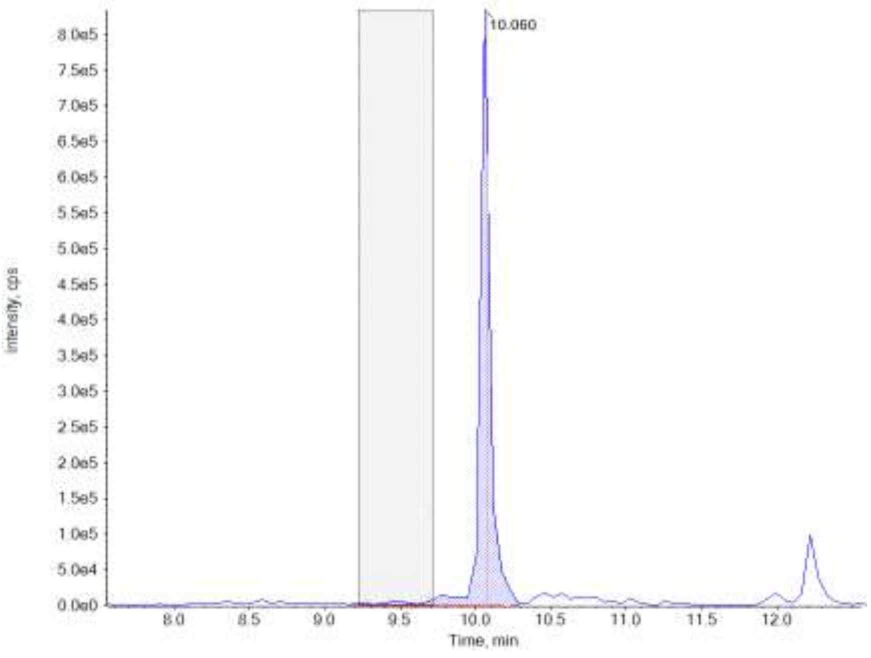


**Acquired / Library MSMS**

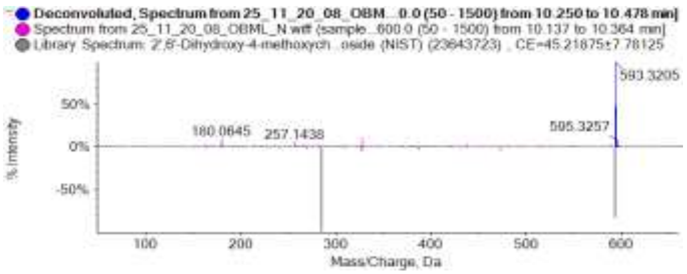


**Acquired / Theoretical MS**

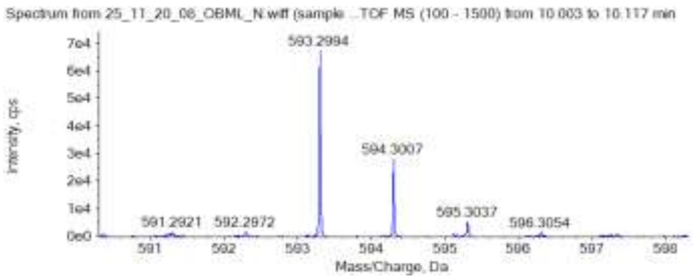

**609.2699 / 10.23** (Mass/FragMass/RT/Isotope/Library/Formula/Ion Ratio)

**Retention Time:** 10.23 minutes
**Precursor m/z :** 609.2699
**Fit (%)** 93.9% **RFit (%)** 91.5%

**Exp RT:** 10.23 minutes
**Analyte Name:**
609.2699 / 10.23

**Collision Energy = 35 ± 15 eV**

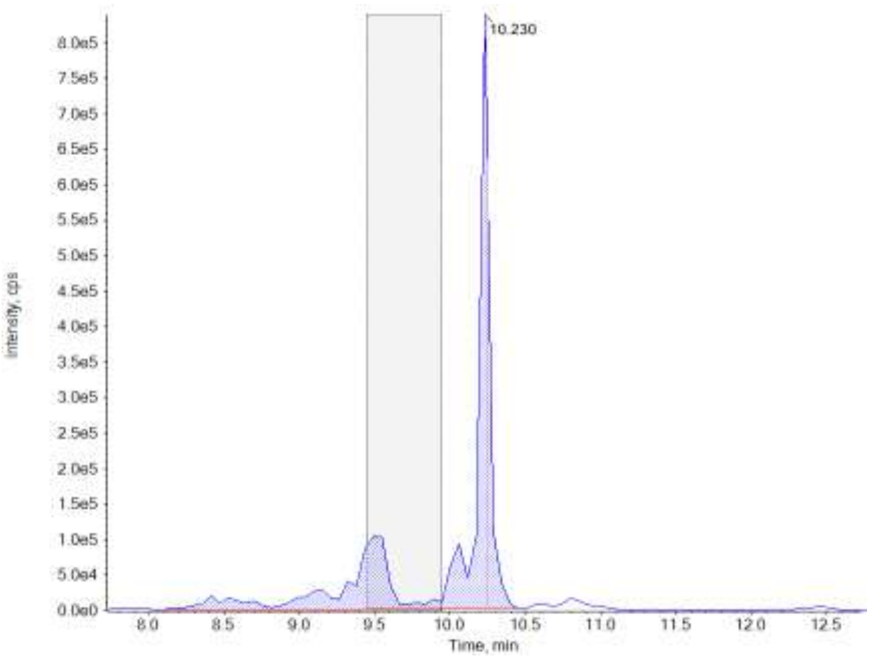


**Acquired / Library MSMS**

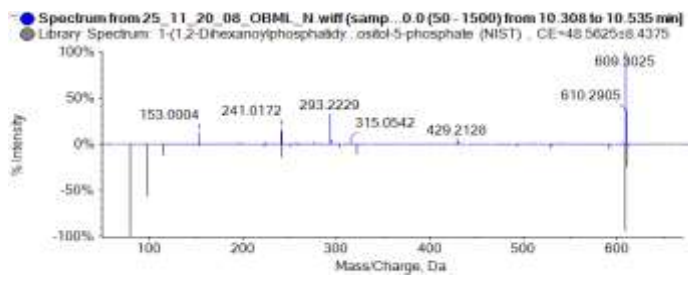


**Acquired / Theoretical MS**

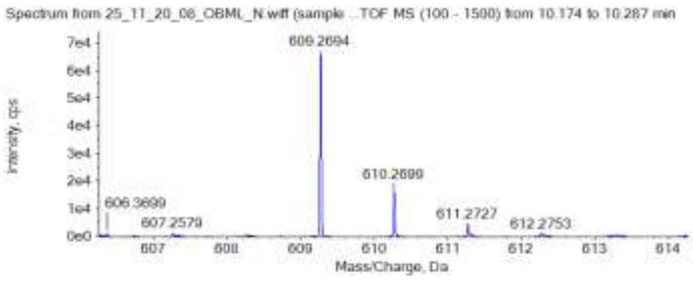

## 645.3644 / 10.57 [M-H2O-H]- (Mass/FragMass/RT/Isotope/Library/Formula/Ion Ratio)

| | |
|---|---|
| **Retention Time:** 10.58 minutes | **Exp RT:** 10.57 minutes |
| **Precursor m/z :** 645.3644 | **Analyte Name:** |
| **Fit (%)** 23.9% **RFit (%)** 100.0% | 645.3644 / 10.57 [M-H2O-H]- |

**Collision Energy = 35 ± 15 eV**

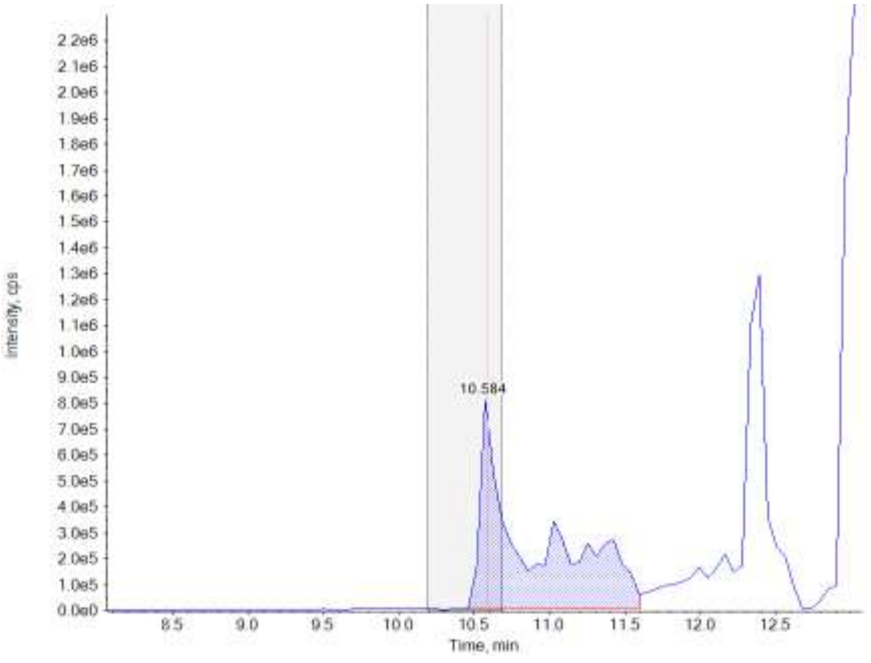


**Acquired / Library MSMS**

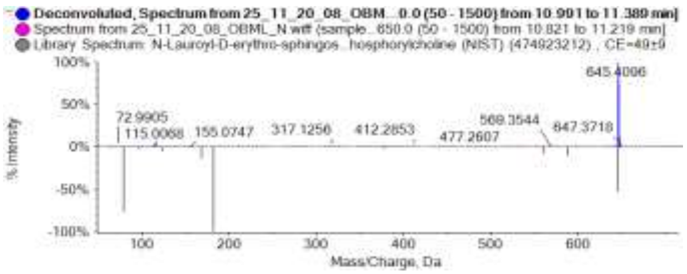


**Acquired / Theoretical MS**

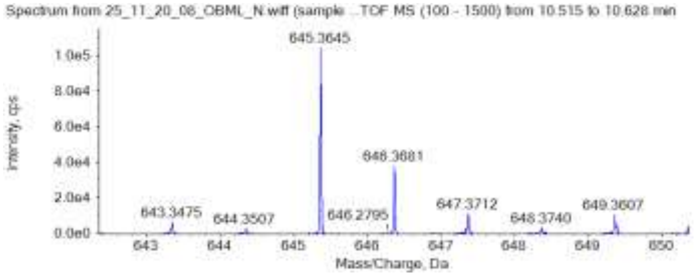

## 201.1476 / 10.69 (Mass/FragMass/RT/Isotope/Library/Formula/Ion Ratio)

**Retention Time:** 10.66 minutes
**Precursor m/z :** 201.1476
**Fit (%)** 73.2% **RFit (%)** 98.4%

**Exp RT:** 10.69 minutes
**Analyte Name:**
201.1476 / 10.69

**Collision Energy = 35 ± 15 eV**

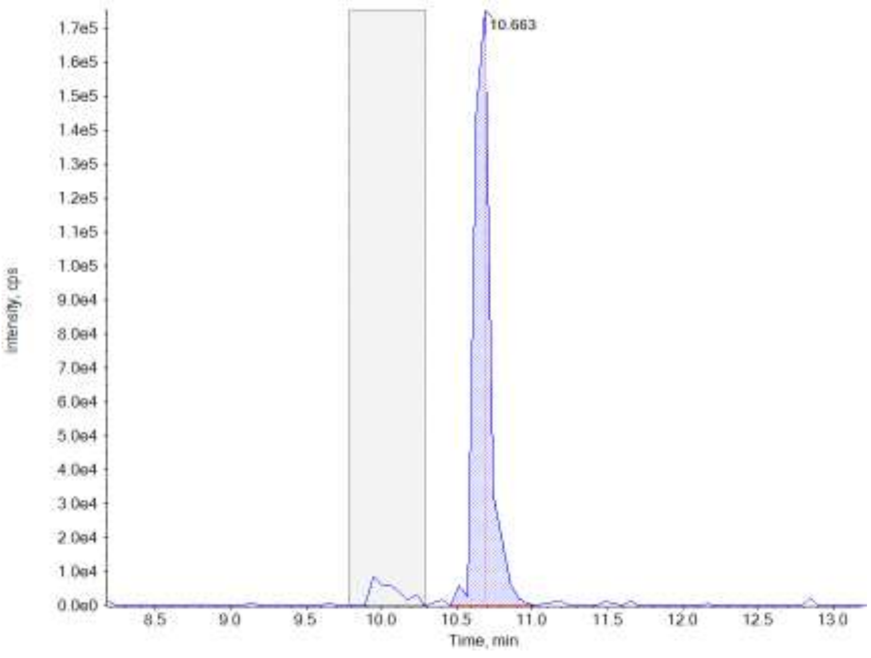


**Acquired / Library MSMS**

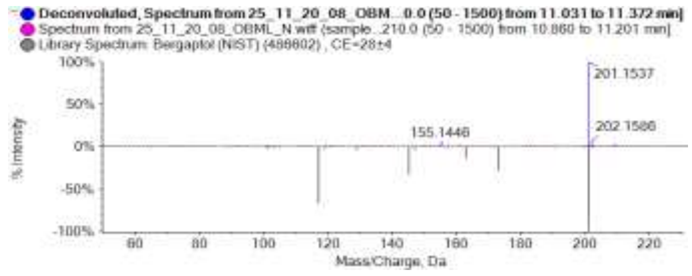


**Acquired / Theoretical MS**

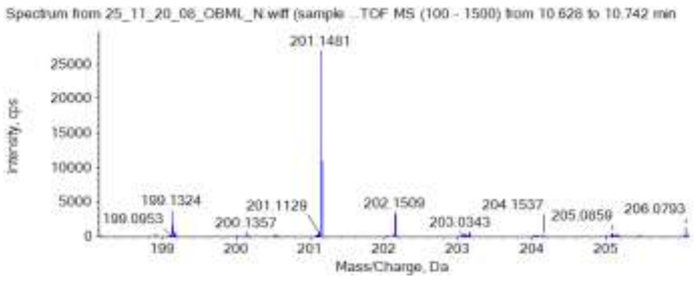

**679.4110 / 10.86** (Mass/FragMass/RT/Isotope/Library/Formula/Ion Ratio)

**Retention Time:** 10.86 minutes
**Precursor m/z :** 679.4110
**Fit (%)** 79.1% **RFit (%)** 94.0%

**Exp RT:** 10.86 minutes
**Analyte Name:**
679.4110 / 10.86

**Collision Energy = 35 ± 15 eV**

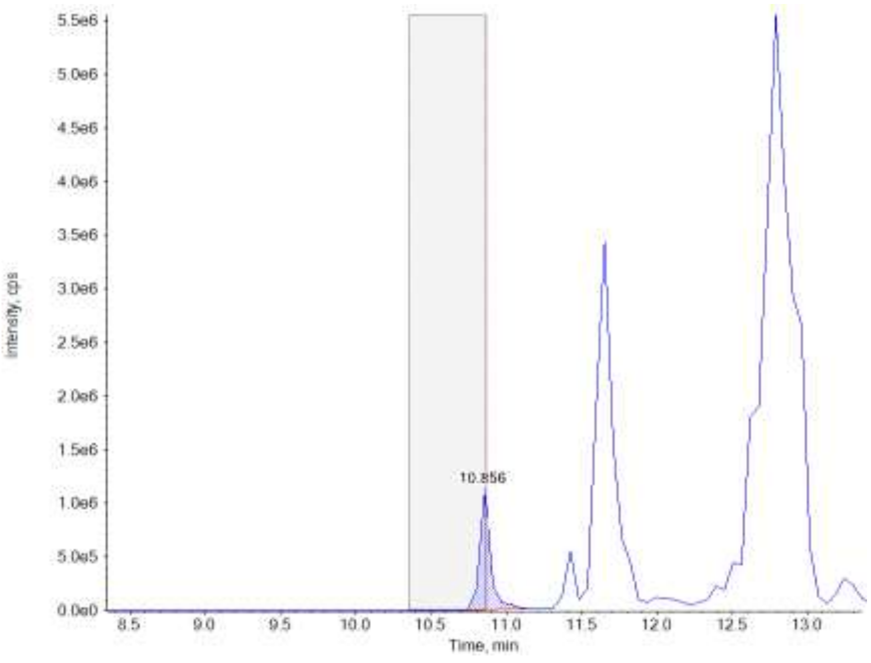


**Acquired / Library MSMS**

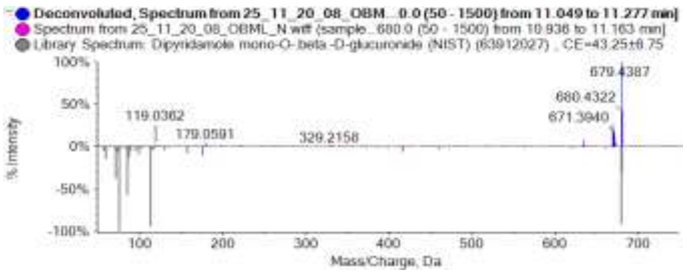


**Acquired / Theoretical MS**

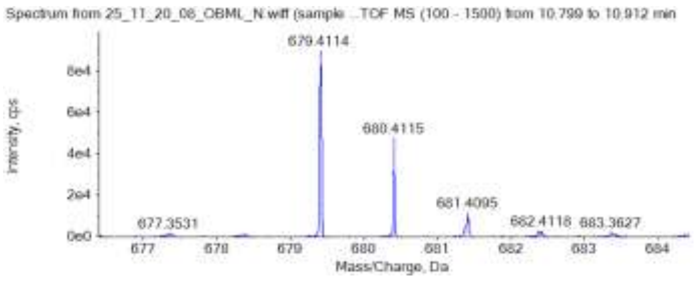

## 227.1280 / 10.91 (Mass/FragMass/RT/Isotope/Library/Formula/Ion Ratio)

**Retention Time:** 10.92 minutes
**Precursor m/z :** 227.1281
**Fit (%)** 99.1% **RFit (%)** 96.8%

**Exp RT:** 10.91 minutes
**Analyte Name:**
227.1280 / 10.91

**Collision Energy = 35 ± 15 eV**

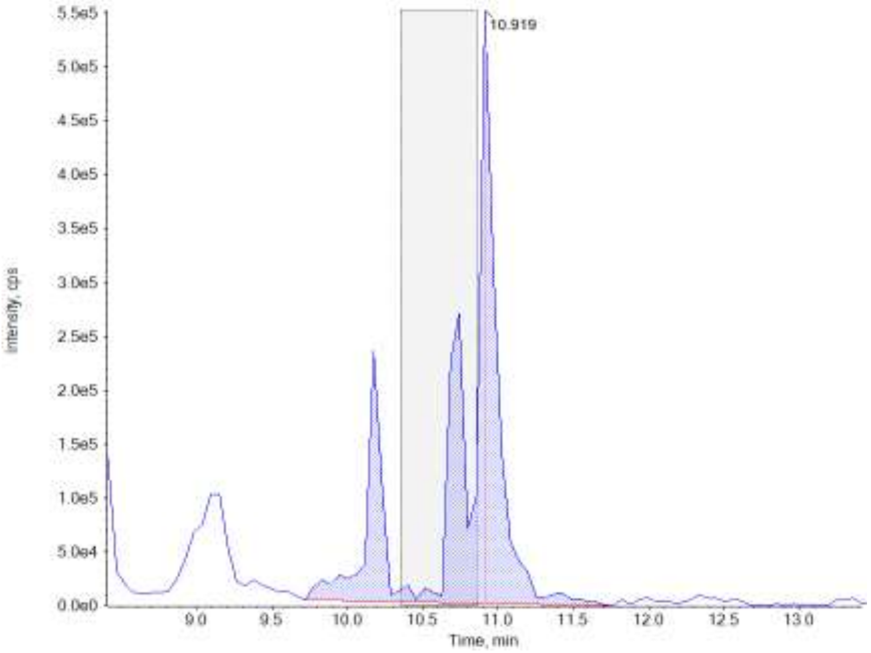


**Acquired / Library MSMS**

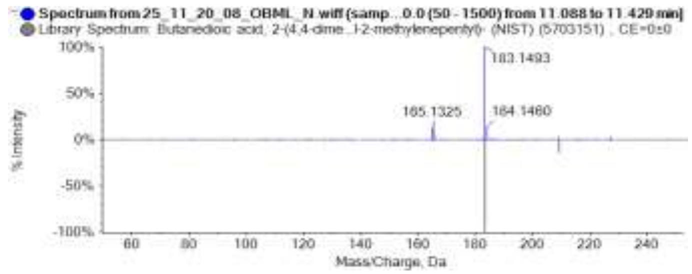


**Acquired / Theoretical MS**

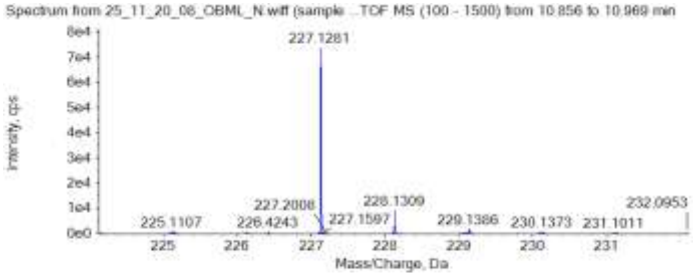

**329.2406 / 10.91** (Mass/FragMass/RT/Isotope/Library/Formula/Ion Ratio)

| | |
|---|---|
| **Retention Time:** 10.89 minutes | **Exp RT:** 10.91 minutes |
| **Precursor m/z :** 329.2406 | **Analyte Name:** |
| **Fit (%)** 66.6% **RFit (%)** 99.9% | 329.2406 / 10.91 |

**Collision Energy = 35 ± 15 eV**

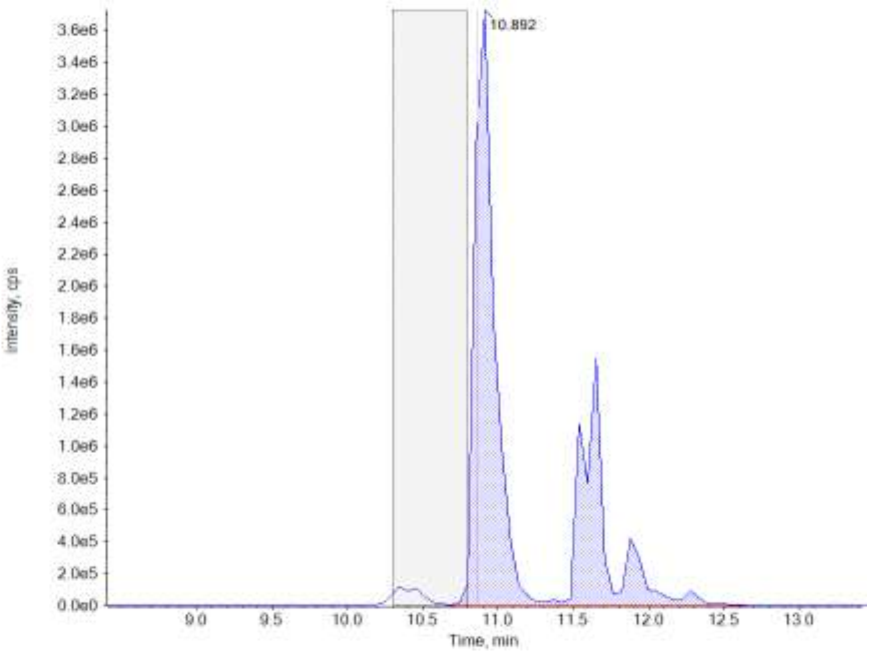


**Acquired / Library MSMS**

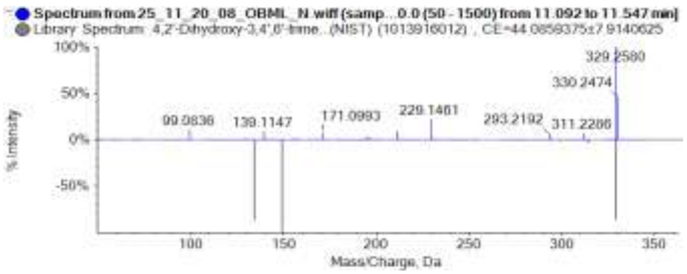


**Acquired / Theoretical MS**

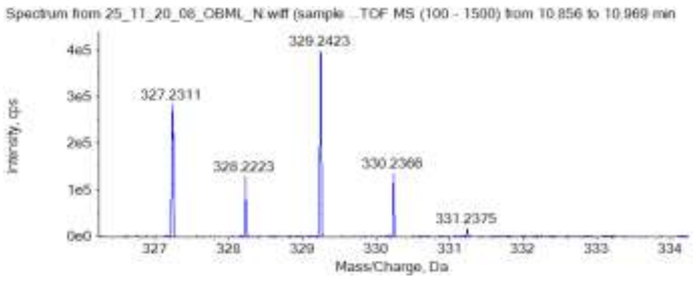

## 707.4025 / 11.03 (Mass/FragMass/RT/Isotope/Library/Formula/Ion Ratio)

**Retention Time:** 11.03 minutes
**Precursor m/z :** 707.4025
**Fit (%)** 93.8% **RFit (%)** 51.5%

**Exp RT:** 11.03 minutes
**Analyte Name:**
707.4025 / 11.03

**Collision Energy = 35 ± 15 eV**

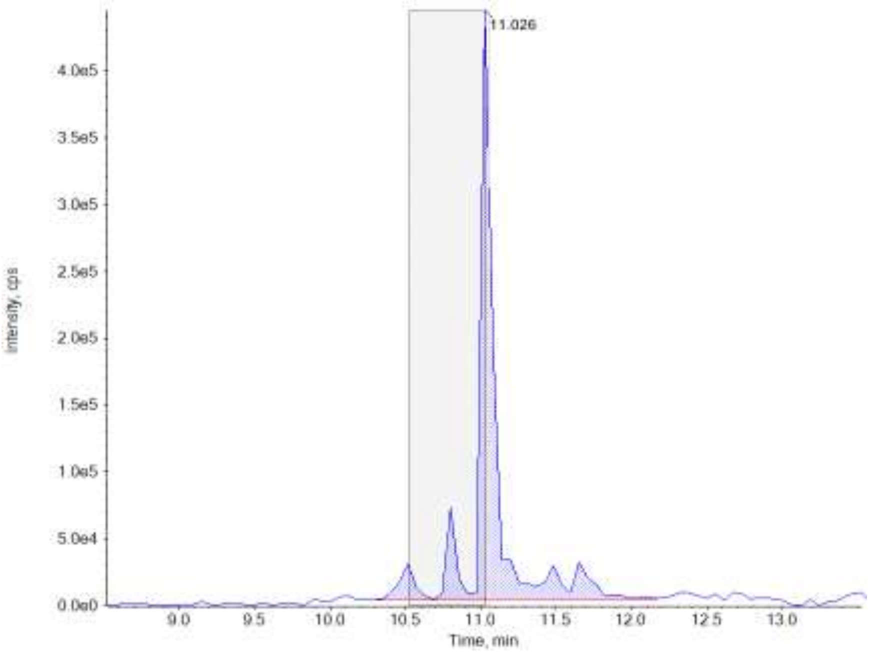


**Acquired / Library MSMS**

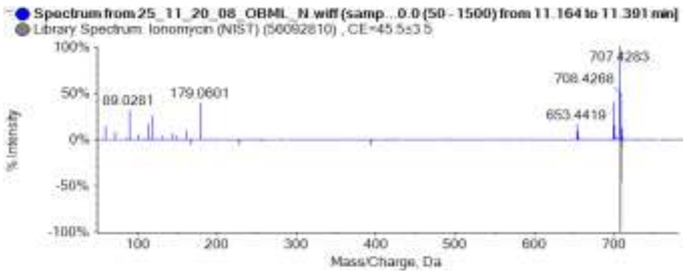


**Acquired / Theoretical MS**

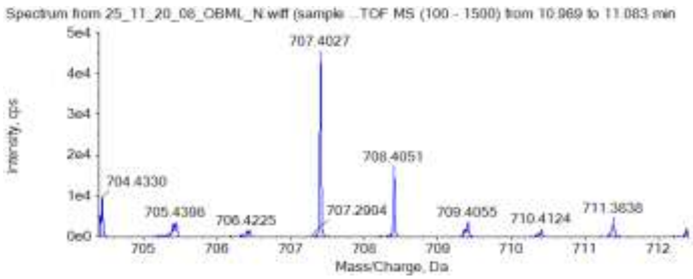

## 695.4015 / 11.20 (Mass/FragMass/RT/Isotope/Library/Formula/Ion Ratio)

| | |
|---|---|
| **Retention Time:** 10.92 minutes | **Exp RT:** 11.20 minutes |
| **Precursor m/z :** 695.4015 | **Analyte Name:** |
| **Fit (%)** 29.3% **RFit (%)** 100.0% | 695.4015 / 11.20 |

**Collision Energy = 35 ± 15 eV**

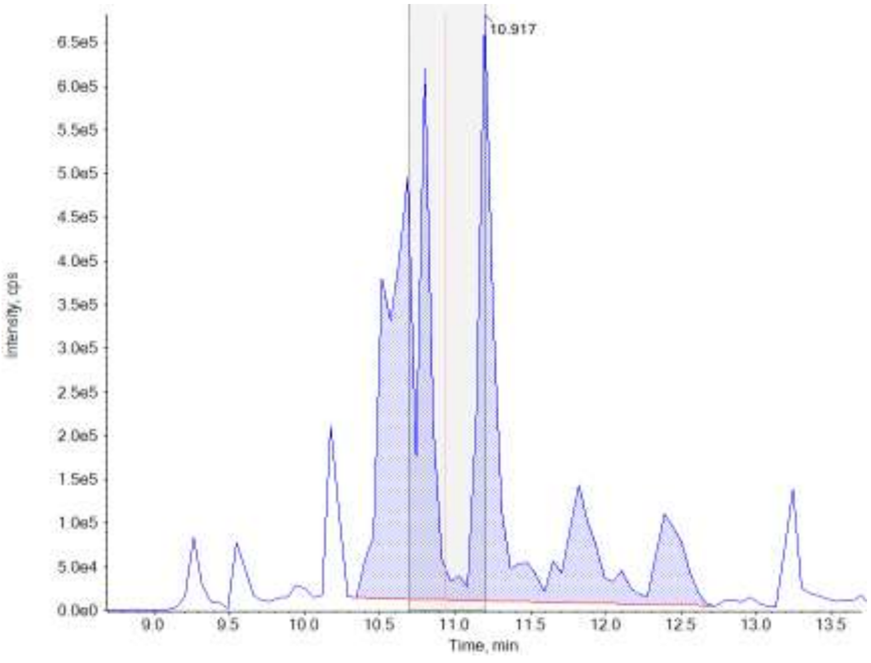


**Acquired / Library MSMS**

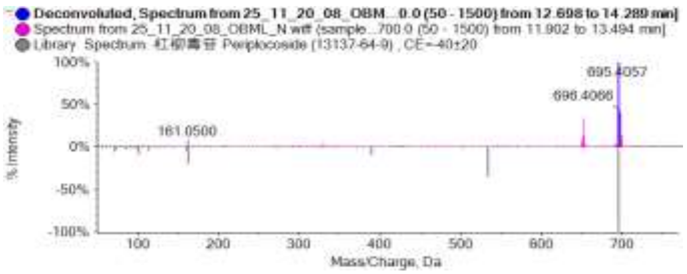


**Acquired / Theoretical MS**

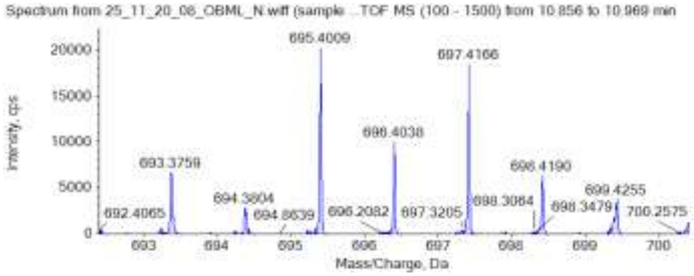

## 287.2215 / 11.25 (Mass/FragMass/RT/Isotope/Library/Formula/Ion Ratio)

**Retention Time:** 11.26 minutes
**Precursor m/z :** 287.2215
**Fit (%)** 88.8% **RFit (%)** 97.5%

**Exp RT:** 11.25 minutes
**Analyte Name:**
287.2215 / 11.25

**Collision Energy = 35 ± 15 eV**

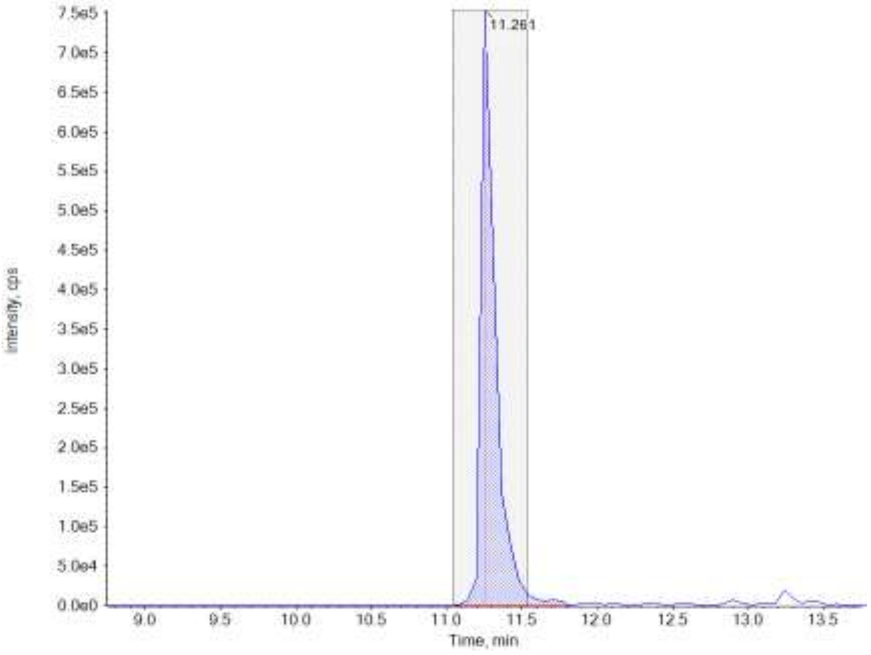


**Acquired / Library MSMS**

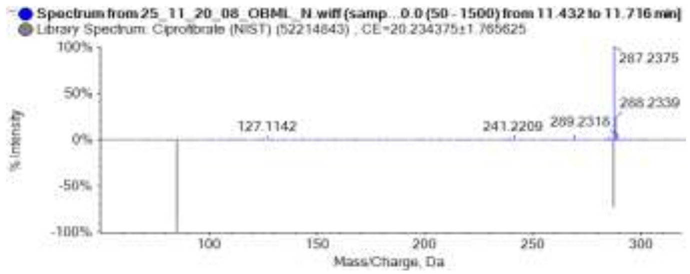


**Acquired / Theoretical MS**

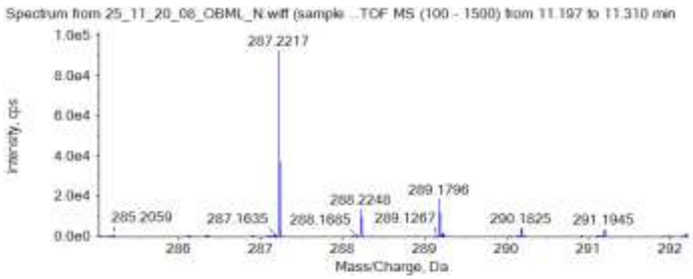

## 195.1383 / 11.42 [M-H2O-H]- (Mass/FragMass/RT/Isotope/Library/Formula/Ion Ratio)

**Retention Time:** 11.40 minutes
**Precursor m/z :** 195.1383
**Fit (%)** 100.0% **RFit (%)** 100.0%

**Exp RT:** 11.42 minutes
**Analyte Name:**
195.1383 / 11.42 [M-H2O-H]-

**Collision Energy = 35 ± 15 eV**

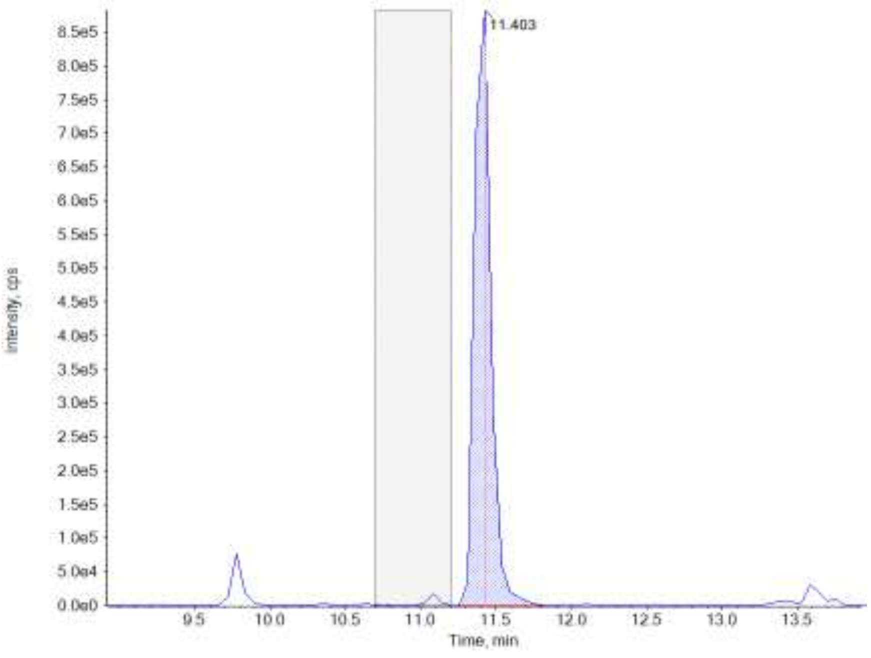


**Acquired / Library MSMS**

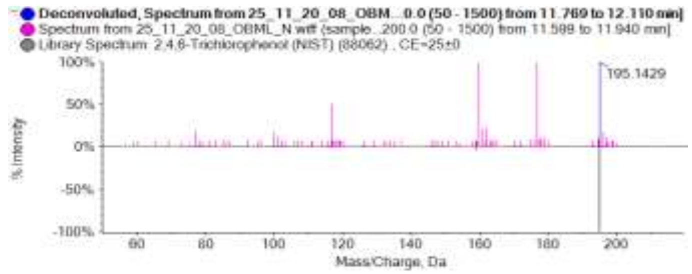


**Acquired / Theoretical MS**

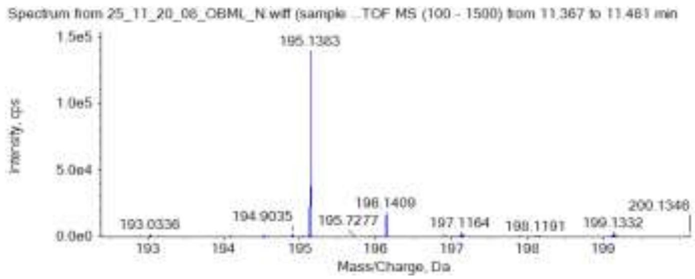

## 213.1487 / 11.08 [M-H]- (Mass/FragMass/RT/Isotope/Library/Formula/Ion Ratio)

| | |
|---|---|
| **Retention Time:** 11.07 minutes | **Exp RT:** 11.08 minutes |
| **Precursor m/z :** 213.1487 | **Analyte Name:** |
| **Fit (%)** 96.5% **RFit (%)** 29.1% | 213.1487 / 11.08 [M-H]- |

**Collision Energy = 35 ± 15 eV**

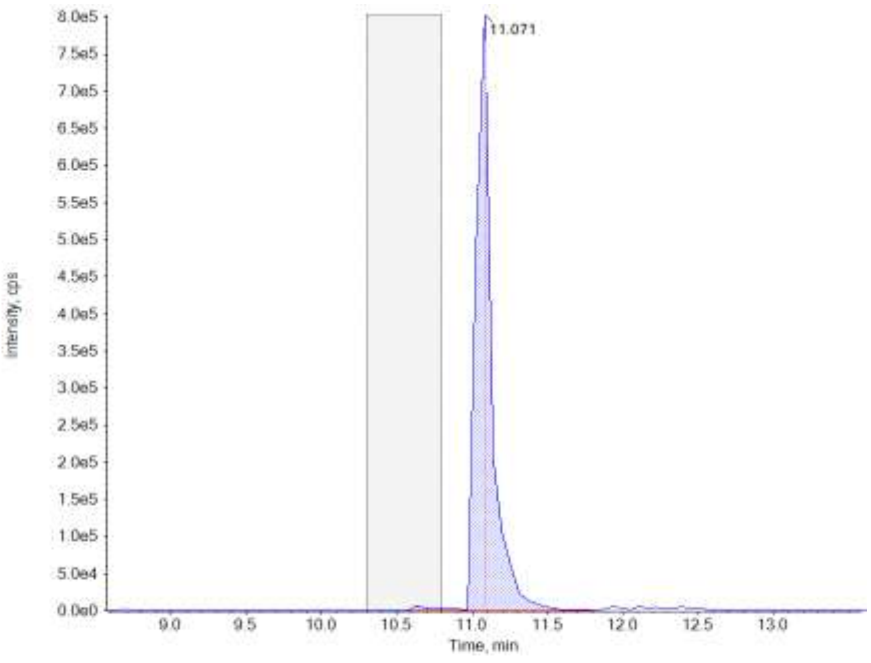


**Acquired / Library MSMS**

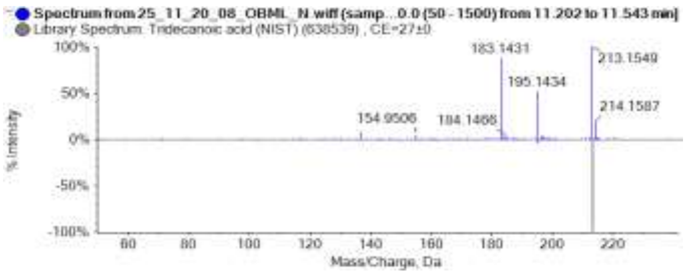


**Acquired / Theoretical MS**

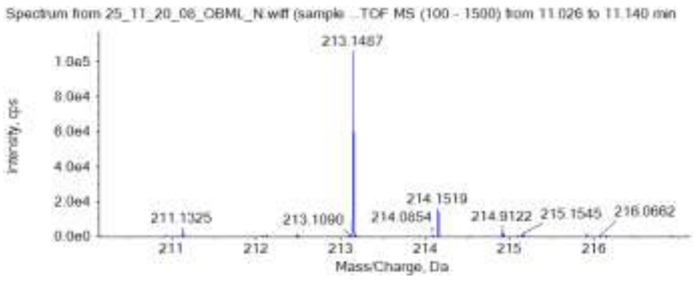

## 239.1280 / 11.42 (Mass/FragMass/RT/Isotope/Library/Formula/Ion Ratio)

| | |
|---|---|
| **Retention Time:** 11.43 minutes<br>**Precursor m/z :** 239.1280<br>**Fit (%)** 90.5% **RFit (%)** 98.9% | **Exp RT:** 11.42 minutes<br>**Analyte Name:**<br>239.1280 / 11.42 |

| | | **Collision Energy = 35 ± 15 eV** |
|---|---|---|
| 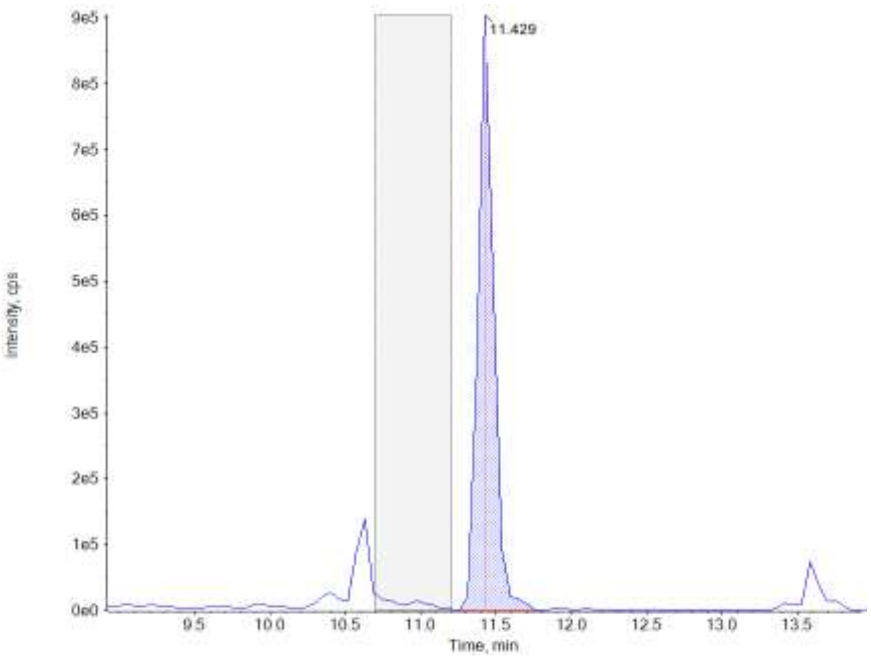 | **Acquired / Library MSMS** | 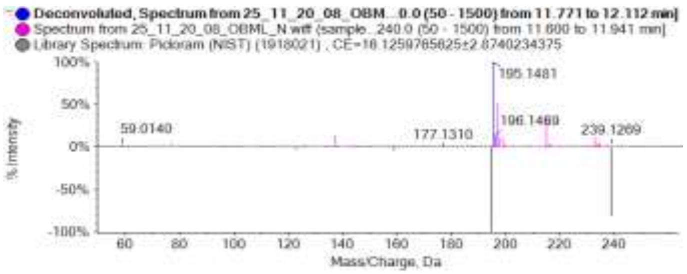 |
| | **Acquired / Theoretical MS** | 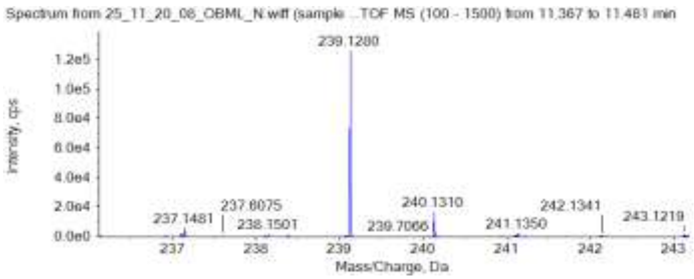 |

## 795.4549 / 11.54 (Mass/FragMass/RT/Isotope/Library/Formula/Ion Ratio)

| | |
|---|---|
| **Retention Time:** 11.52 minutes | **Exp RT:** 11.54 minutes |
| **Precursor m/z :** 795.4549 | **Analyte Name:** |
| **Fit (%)** 33.2% **RFit (%)** 100.0% | 795.4549 / 11.54 |

**Collision Energy = 35 ± 15 eV**

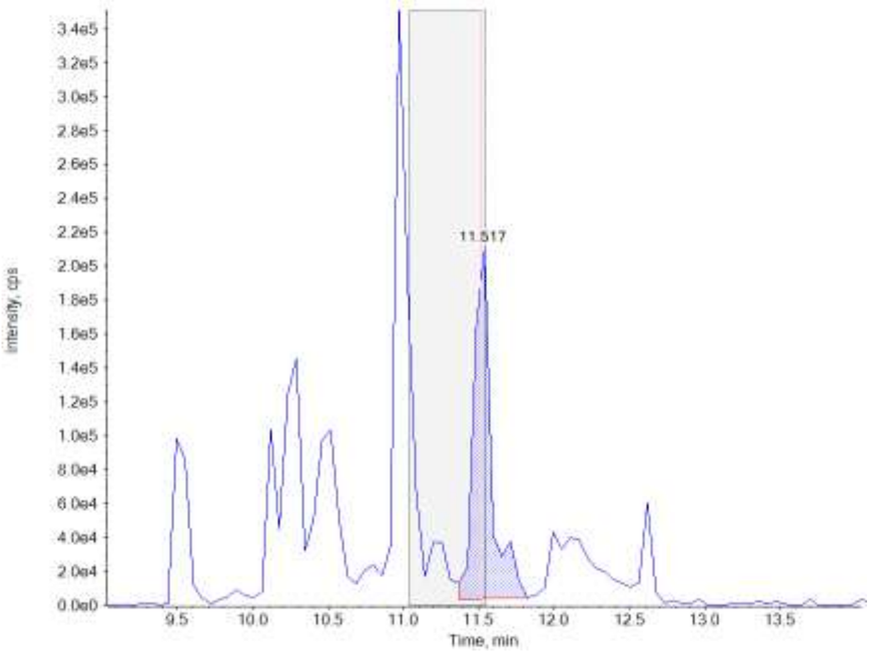


**Acquired / Library MSMS**

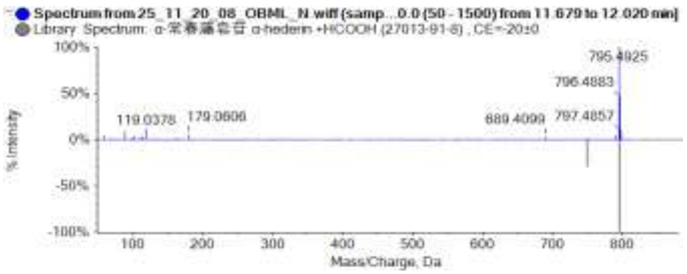


**Acquired / Theoretical MS**

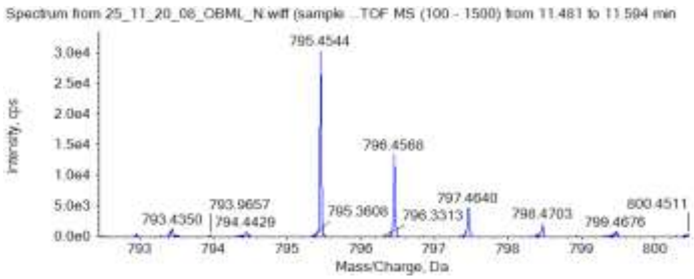

## 669.3803 / 11.65 (Mass/FragMass/RT/Isotope/Library/Formula/Ion Ratio)

**Retention Time:** 11.62 minutes **Exp RT:** 11.65 minutes
**Precursor m/z :** 669.3803 **Analyte Name:**
**Fit (%)** 41.2% **RFit (%)** 100.0% 669.3803 / 11.65

**Collision Energy = 35 ± 15 eV**

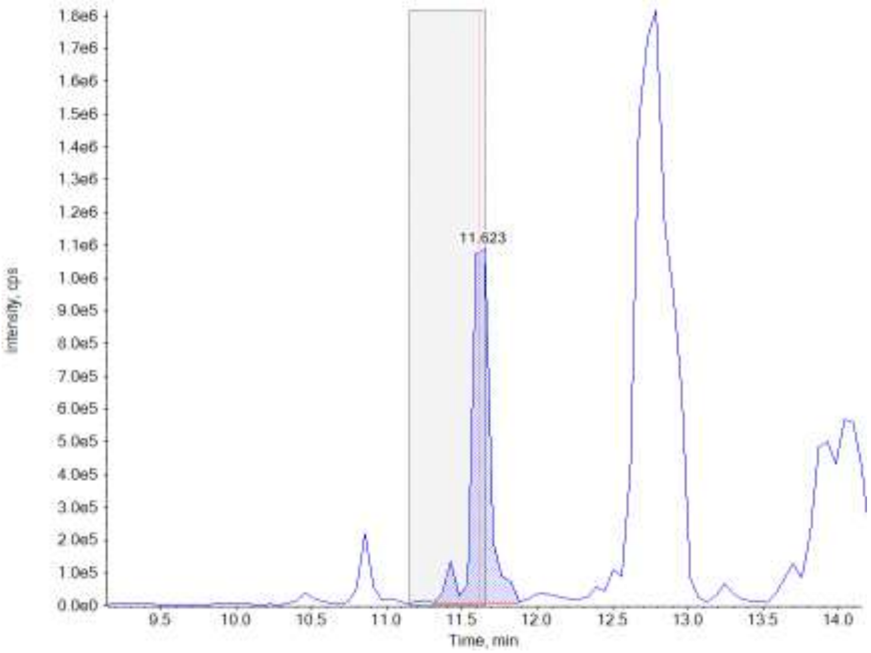


**Acquired / Library MSMS**

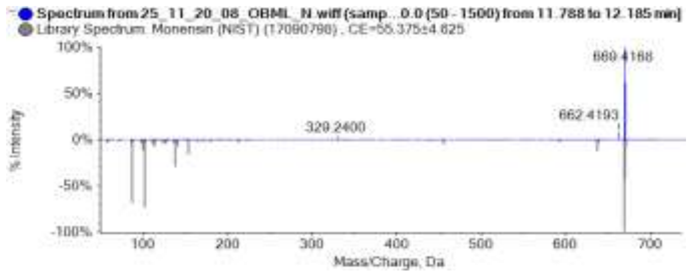


**Acquired / Theoretical MS**

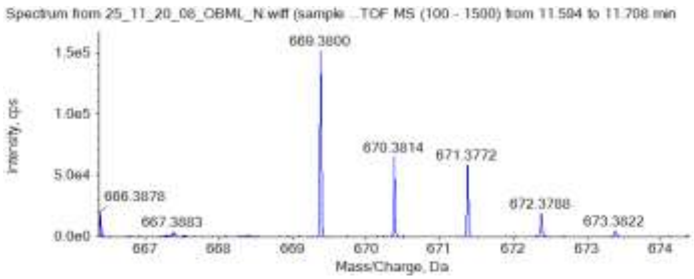

## 309.2073 / 11.82

(Mass/FragMass/RT/Isotope/Library/Formula/Ion Ratio)

**Retention Time:** 11.80 minutes
**Precursor m/z :** 309.2073
**Fit (%)** 54.4% **RFit (%)** 79.5%

**Exp RT:** 11.82 minutes
**Analyte Name:**
309.2073 / 11.82

**Collision Energy = 35 ± 15 eV**

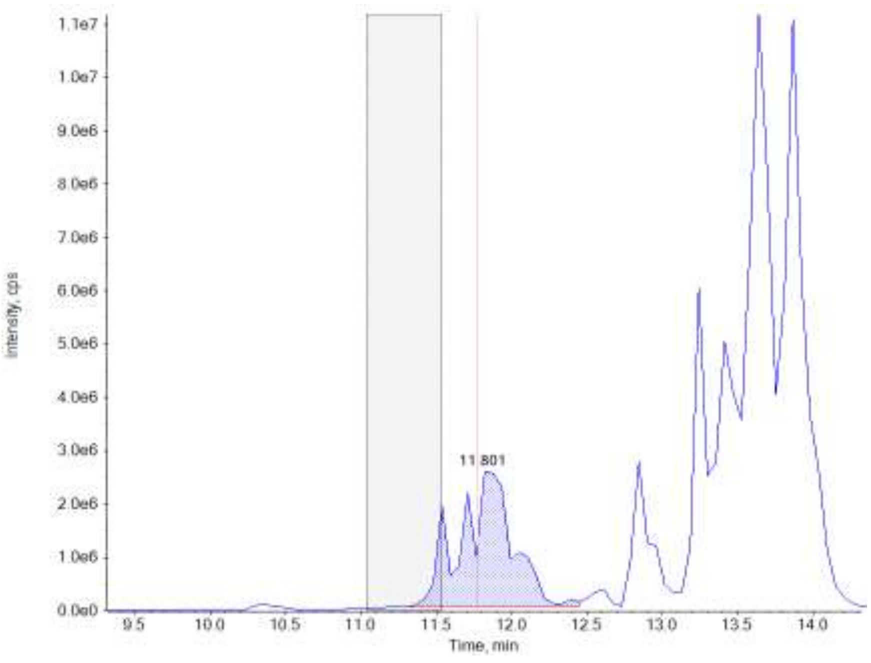


**Acquired / Library MSMS**

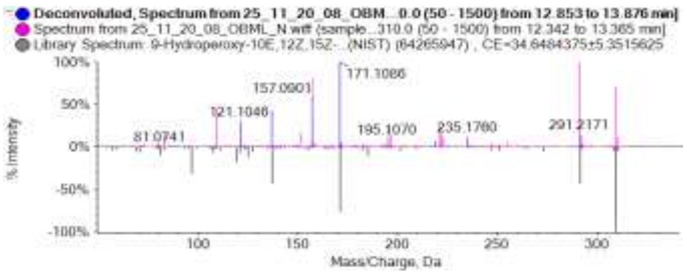


**Acquired / Theoretical MS**

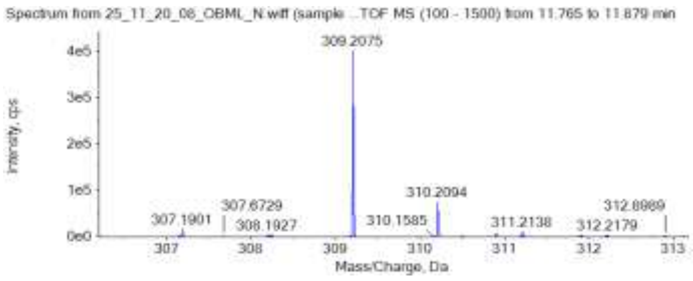

## 718.4561 / 12.56

(Mass/FragMass/RT/Isotope/Library/Formula/Ion Ratio)

**Retention Time:** 12.56 minutes
**Precursor m/z :** 718.4561
**Fit (%)** 24.5% **RFit (%)** 100.0%

**Exp RT:** 12.56 minutes
**Analyte Name:**
718.4561 / 12.56

**Collision Energy = 35 ± 15 eV**

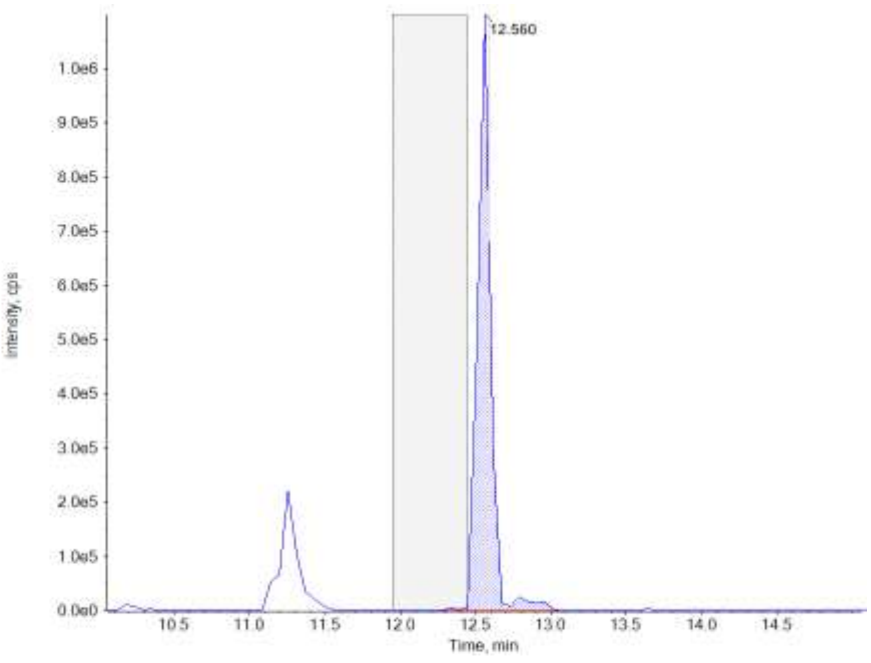


**Acquired / Library MSMS**

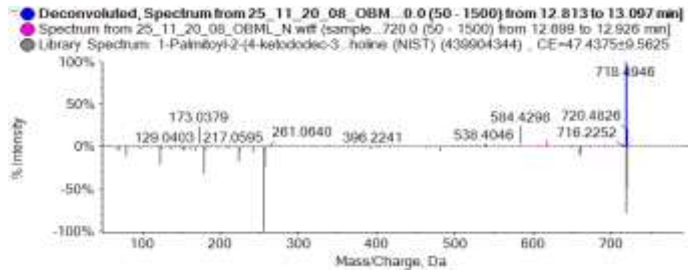


**Acquired / Theoretical MS**

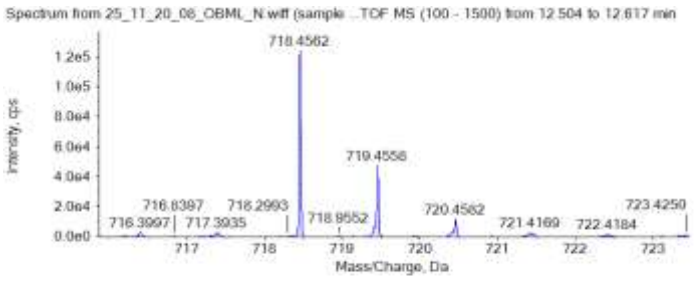

## 237.1495 / 12.62 (Mass/FragMass/RT/Isotope/Library/Formula/Ion Ratio)

| | |
|---|---|
| **Retention Time:** 12.62 minutes | **Exp RT:** 12.62 minutes |
| **Precursor m/z :** 237.1495 | **Analyte Name:** |
| **Fit (%)** 92.7% **RFit (%)** 84.8% | 237.1495 / 12.62 |

**Collision Energy = 35 ± 15 eV**

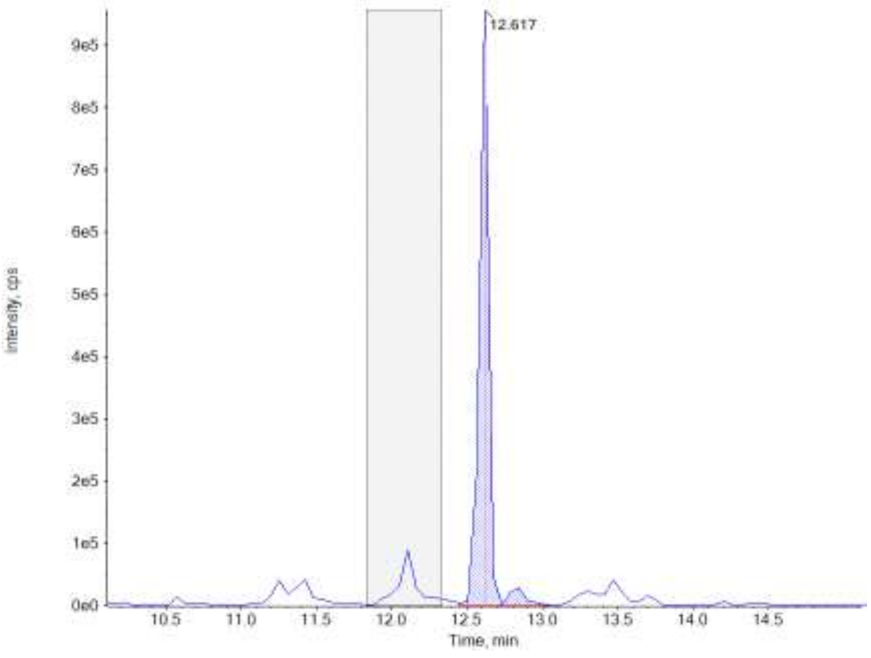


**Acquired / Library MSMS**

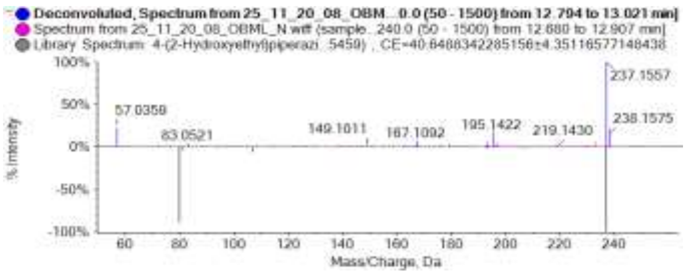


**Acquired / Theoretical MS**

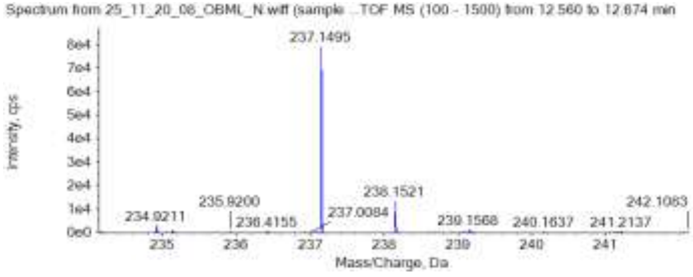

## 669.3805 / 12.73

(Mass/FragMass/RT/Isotope/Library/Formula/Ion Ratio)

**Retention Time:** 12.75 minutes
**Precursor m/z :** 669.3805
**Fit (%)** 36.8% **RFit (%)** 100.0%

**Exp RT:** 12.73 minutes
**Analyte Name:**
669.3805 / 12.73

**Collision Energy = 35 ± 15 eV**

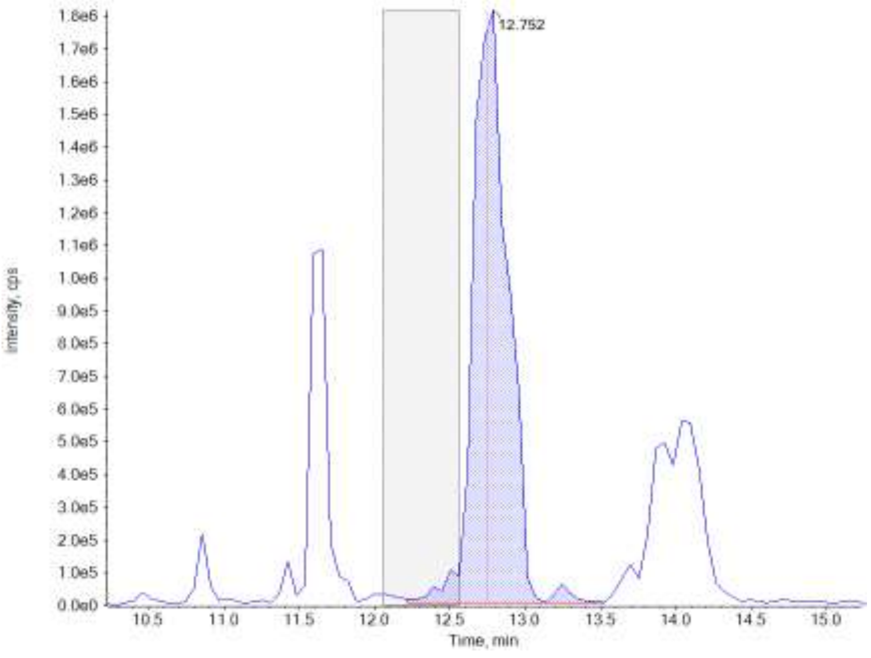


**Acquired / Library MSMS**

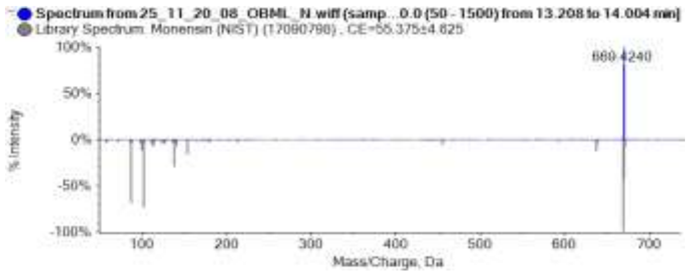


**Acquired / Theoretical MS**

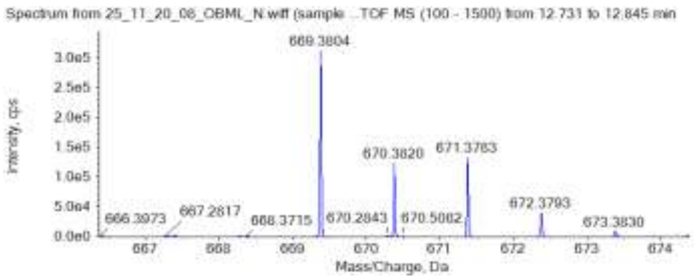

## 679.4268 / 12.79 (Mass/FragMass/RT/Isotope/Library/Formula/Ion Ratio)

**Retention Time:** 12.79 minutes
**Precursor m/z :** 679.4268
**Fit (%)** 48.0% **RFit (%)** 85.5%

**Exp RT:** 12.79 minutes
**Analyte Name:**
679.4268 / 12.79

**Collision Energy = 35 ± 15 eV**

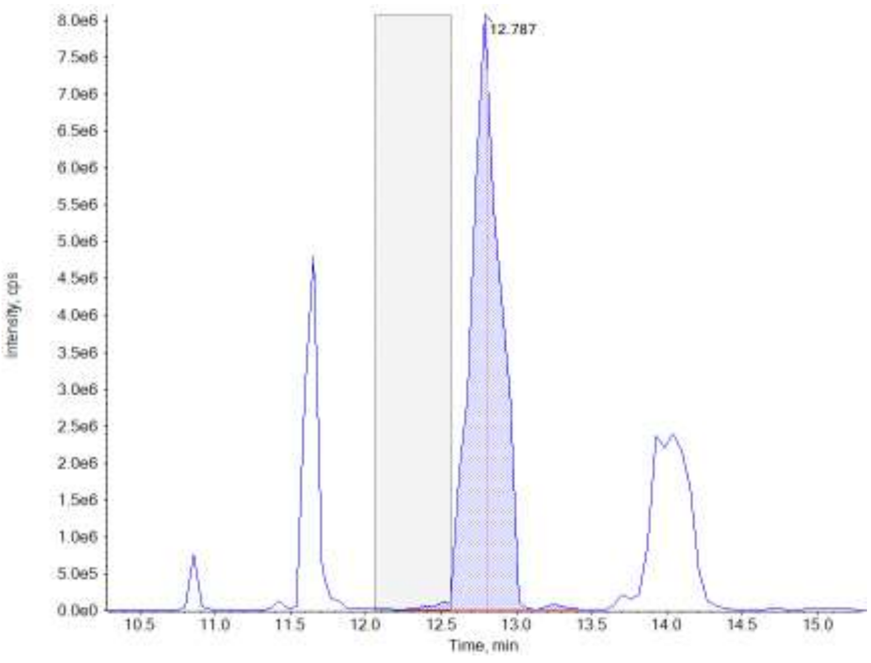


**Acquired / Library MSMS**

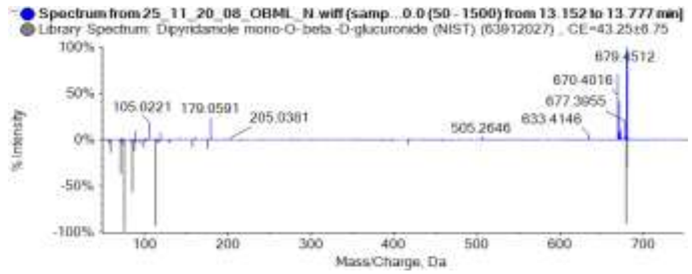


**Acquired / Theoretical MS**

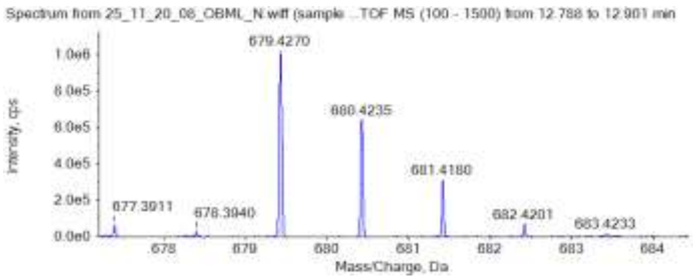

## 601.3746 / 13.13 (Mass/FragMass/RT/Isotope/Library/Formula/Ion Ratio)

| | |
|---|---|
| **Retention Time:** 13.12 minutes | **Exp RT:** 13.13 minutes |
| **Precursor m/z :** 601.3746 | **Analyte Name:** |
| **Fit (%)** 87.0% **RFit (%)** 94.4% | 601.3746 / 13.13 |

**Collision Energy = 35 ± 15 eV**

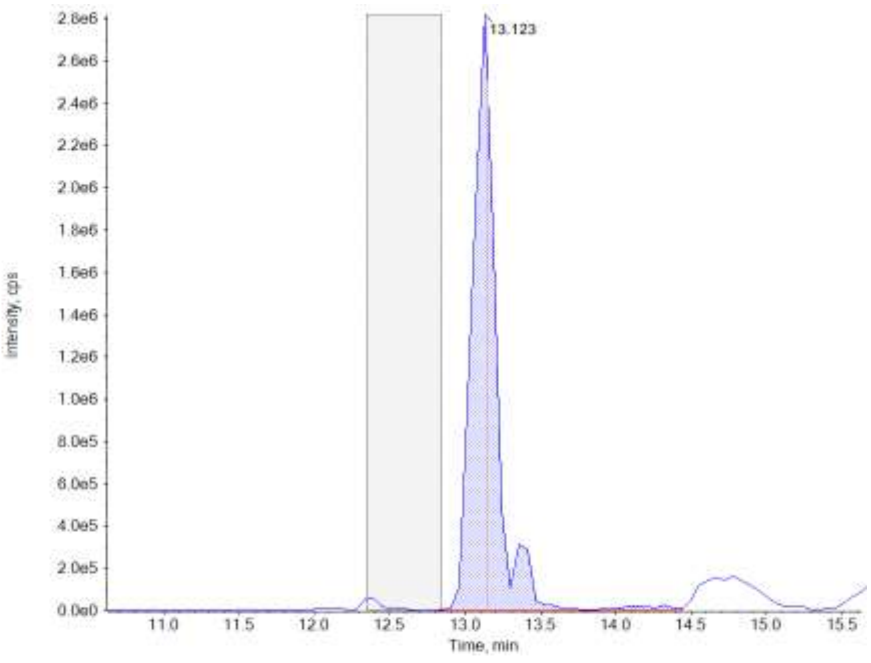


**Acquired / Library MSMS**

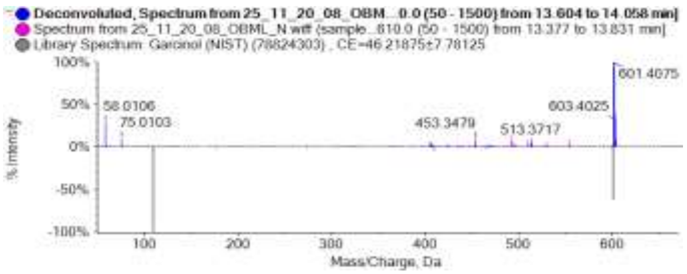


**Acquired / Theoretical MS**

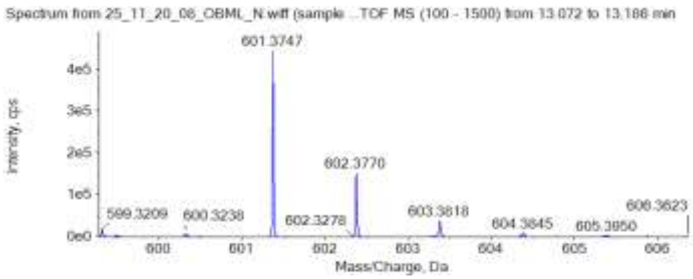

## 571.2930 / 13.30 (Mass/FragMass/RT/Isotope/Library/Formula/Ion Ratio)

**Retention Time:** 13.30 minutes
**Precursor m/z :** 571.2930
**Fit (%)** 97.0% **RFit (%)** 93.9%

**Exp RT:** 13.30 minutes
**Analyte Name:**
571.2930 / 13.30

**Collision Energy = 35 ± 15 eV**

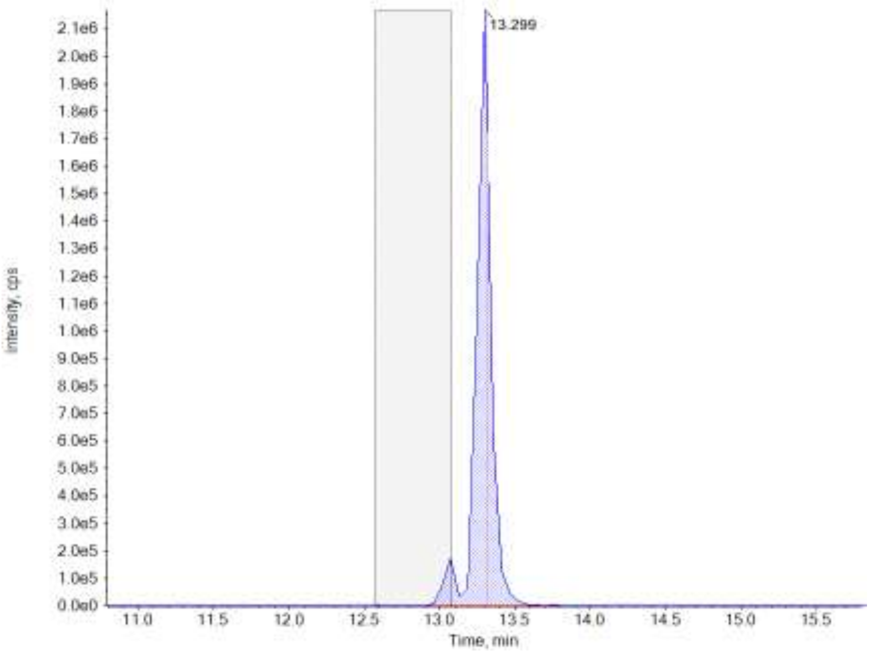


**Acquired / Library MSMS**

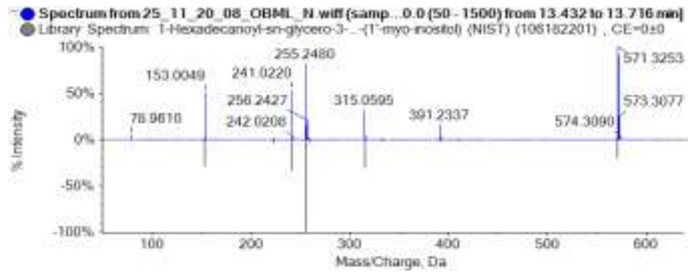


**Acquired / Theoretical MS**

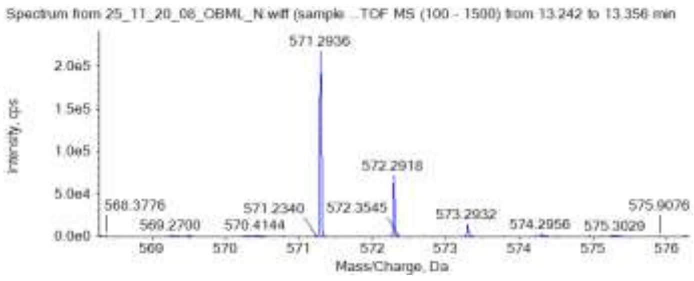

**253.1793 / 13.36** (Mass/FragMass/RT/Isotope/Library/Formula/Ion Ratio)

**Retention Time:** 13.38 minutes  **Exp RT:** 13.36 minutes
**Precursor m/z :** 253.1793  **Analyte Name:** 253.1793 / 13.36
**Fit (%)** 99.5% **RFit (%)** 85.4%

**Collision Energy = 35 ± 15 eV**

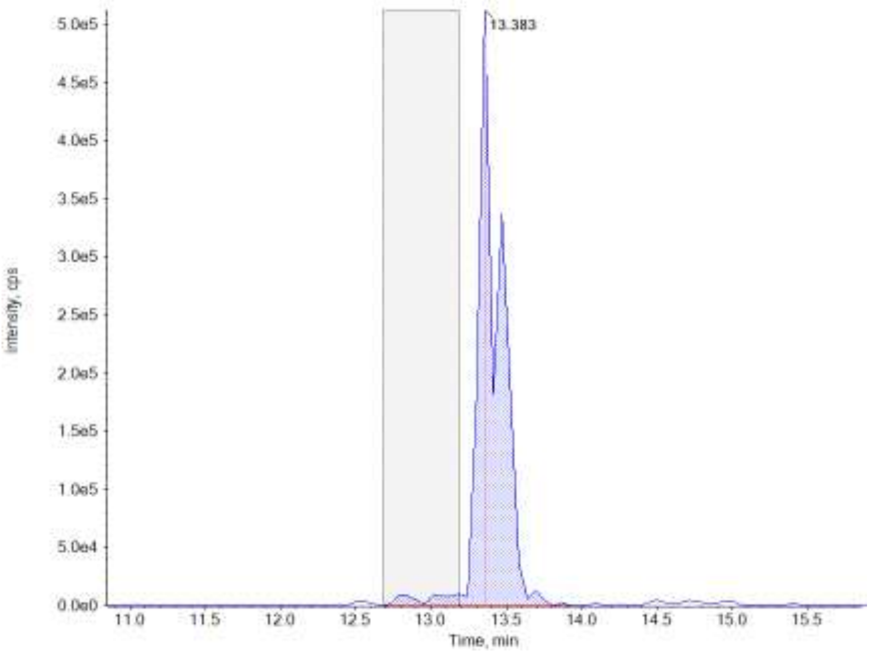


**Acquired / Library MSMS**

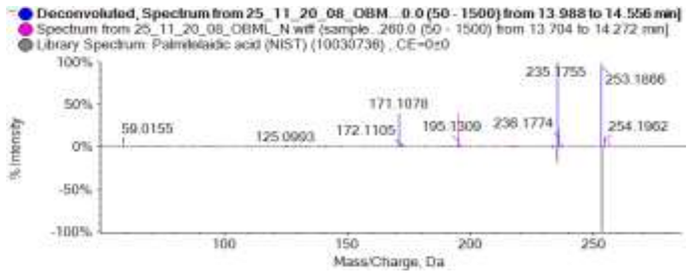


**Acquired / Theoretical MS**

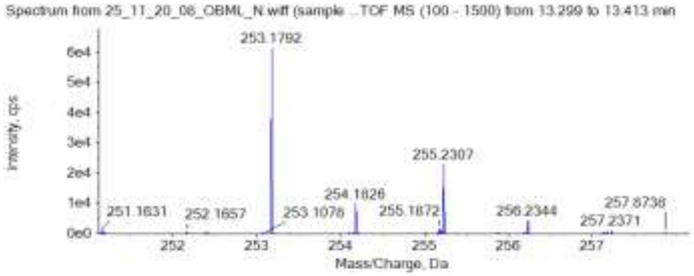

## 665.4288 / 13.47 (Mass/FragMass/RT/Isotope/Library/Formula/Ion Ratio)

**Retention Time:** 13.47 minutes
**Precursor m/z :** 665.4288
**Fit (%)** 74.8% **RFit (%)** 98.6%

**Exp RT:** 13.47 minutes
**Analyte Name:**
665.4288 / 13.47

**Collision Energy = 35 ± 15 eV**

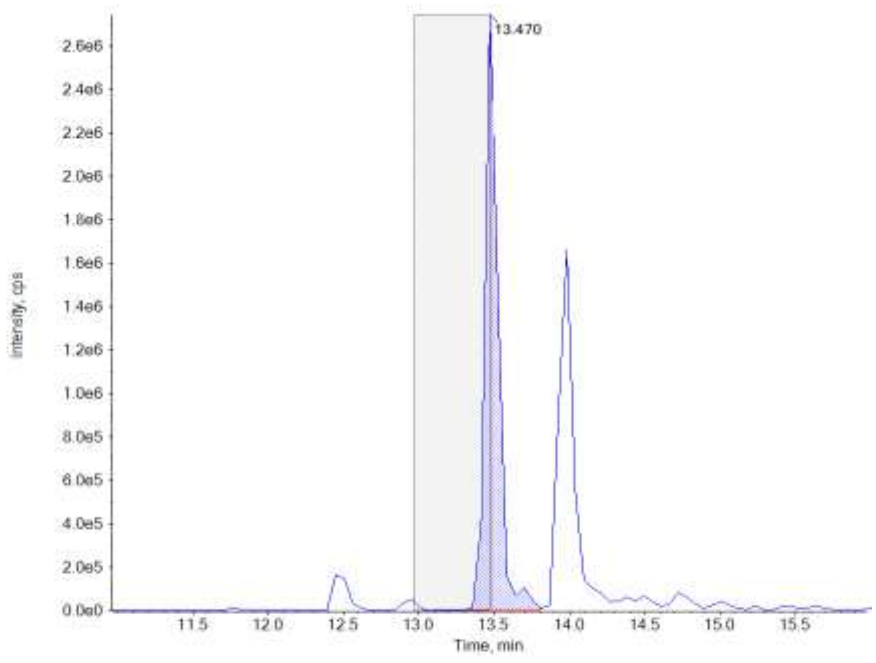


**Acquired / Library MSMS**

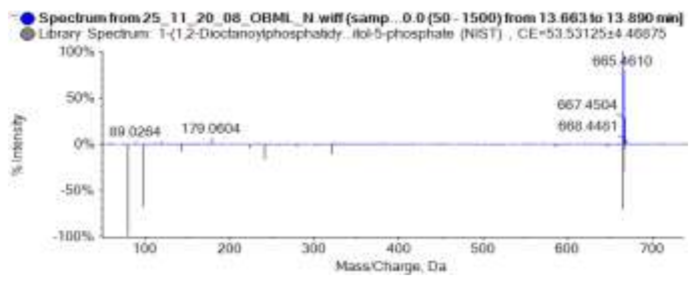


**Acquired / Theoretical MS**

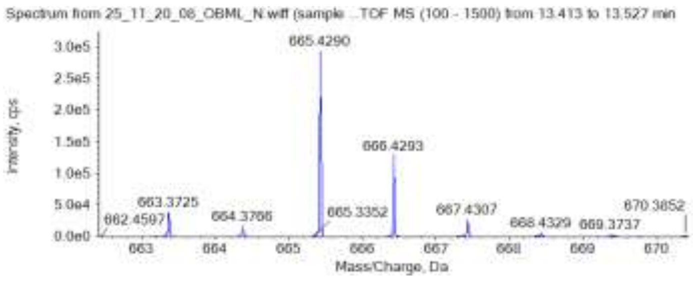

## 667.3590 / 13.64

(Mass/FragMass/RT/Isotope/Library/Formula/Ion Ratio)

| | |
|---|---|
| **Retention Time:** 13.65 minutes | **Exp RT:** 13.64 minutes |
| **Precursor m/z :** 667.3590 | **Analyte Name:** |
| **Fit (%)** 36.3% **RFit (%)** 99.9% | 667.3590 / 13.64 |

**Collision Energy = 35 ± 15 eV**

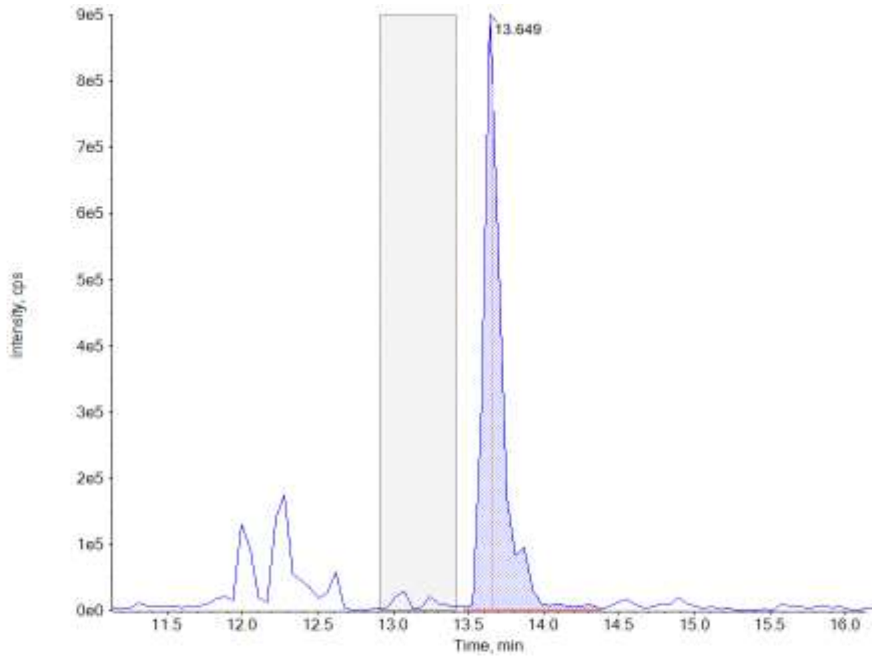


**Acquired / Library MSMS**

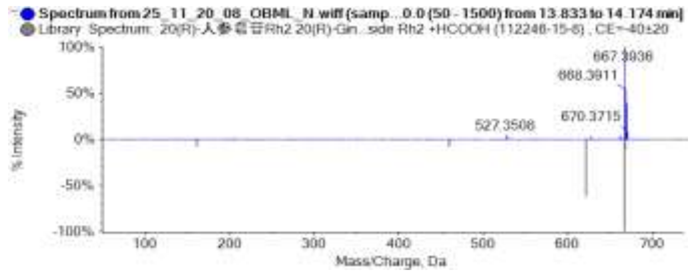


**Acquired / Theoretical MS**

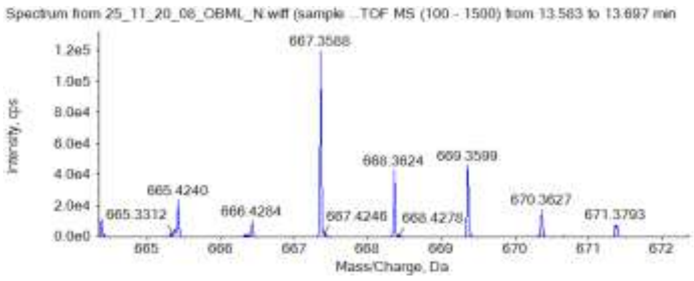

## 941.6015 / 13.64

(Mass/FragMass/RT/Isotope/Library/Formula/Ion Ratio)

| | |
|---|---|
| **Retention Time:** 13.64 minutes | **Exp RT:** 13.64 minutes |
| **Precursor m/z :** 941.6015 | **Analyte Name:** |
| **Fit (%)** 86.7% **RFit (%)** 96.3% | 941.6015 / 13.64 |

**Collision Energy = 35 ± 15 eV**

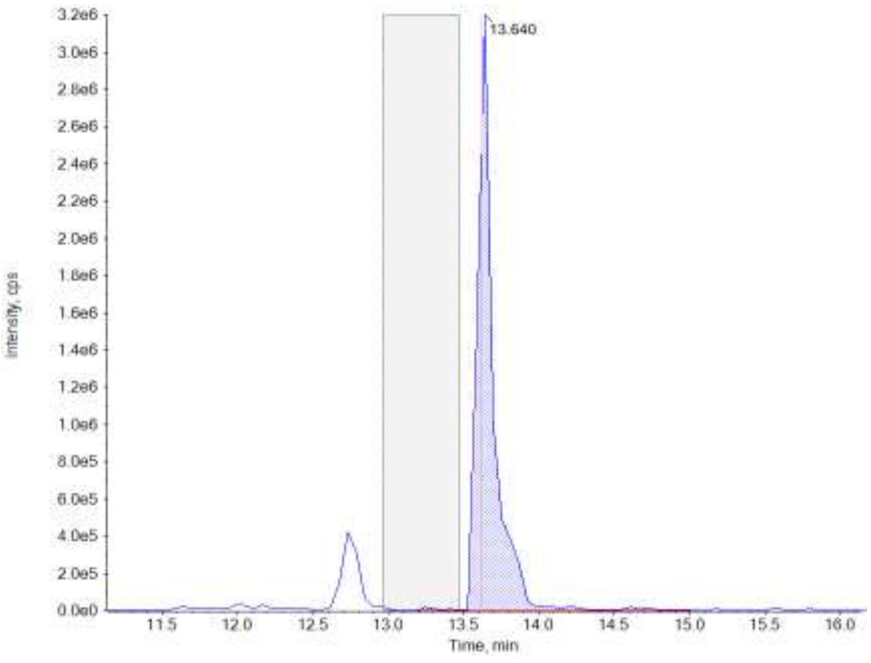


**Acquired / Library MSMS**

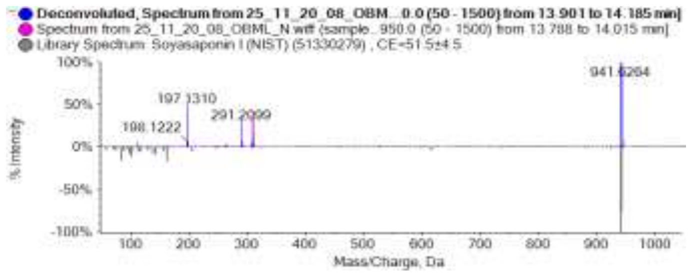


**Acquired / Theoretical MS**

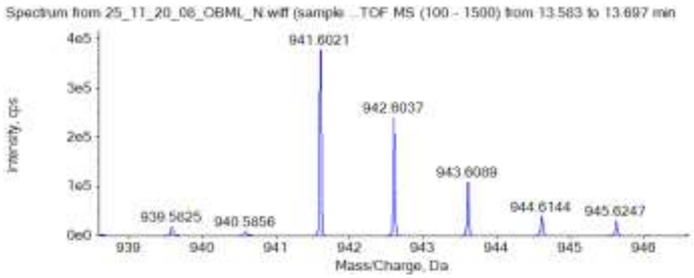

**209.1173 / 13.70** (Mass/FragMass/RT/Isotope/Library/Formula/Ion Ratio)

| | |
|---|---|
| **Retention Time:** 13.70 minutes | **Exp RT:** 13.70 minutes |
| **Precursor m/z :** 209.1173 | **Analyte Name:** |
| **Fit (%)** 77.2% **RFit (%)** 100.0% | 209.1173 / 13.70 |

**Collision Energy = 35 ± 15 eV**

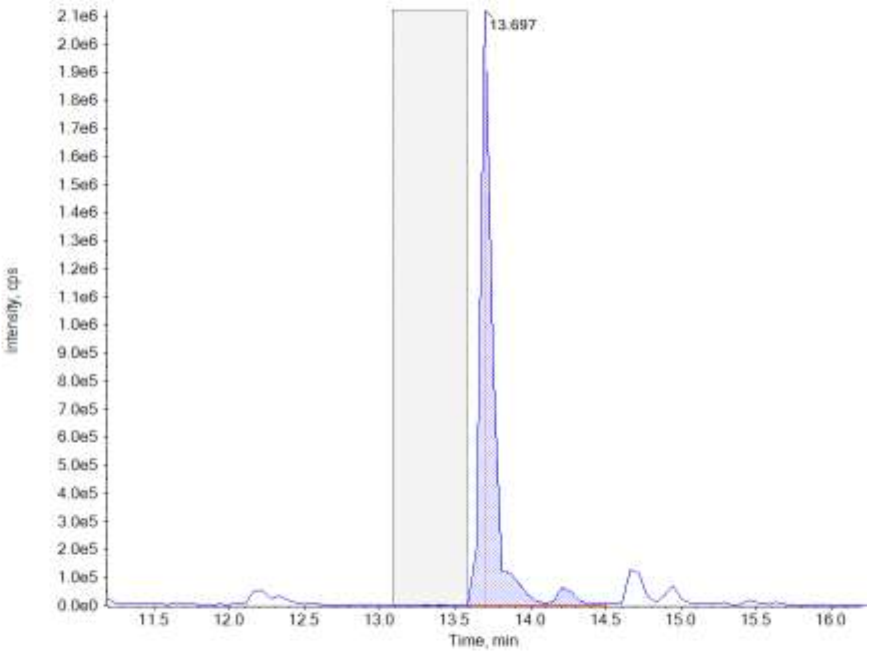


**Acquired / Library MSMS**

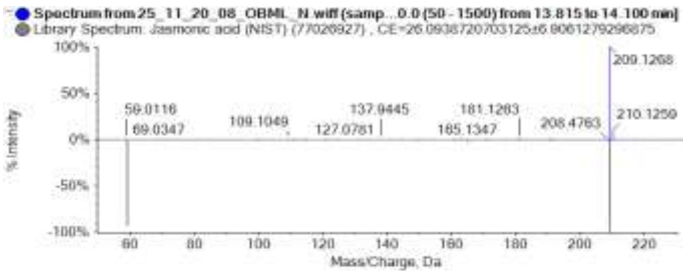


**Acquired / Theoretical MS**

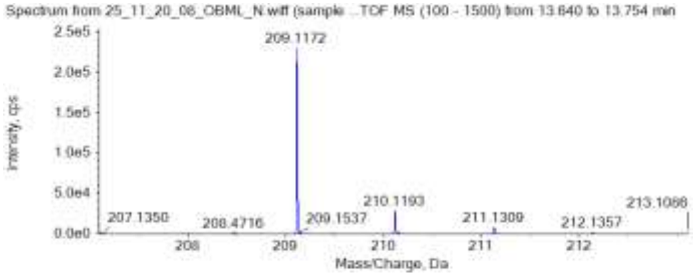

## 289.1787 / 13.70 (Mass/FragMass/RT/Isotope/Library/Formula/Ion Ratio)

**Retention Time:** 13.70 minutes  **Exp RT:** 13.70 minutes
**Precursor m/z :** 289.1787  **Analyte Name:**
**Fit (%)** 89.5% **RFit (%)** 100.0%  289.1787 / 13.70

**Collision Energy = 35 ± 15 eV**

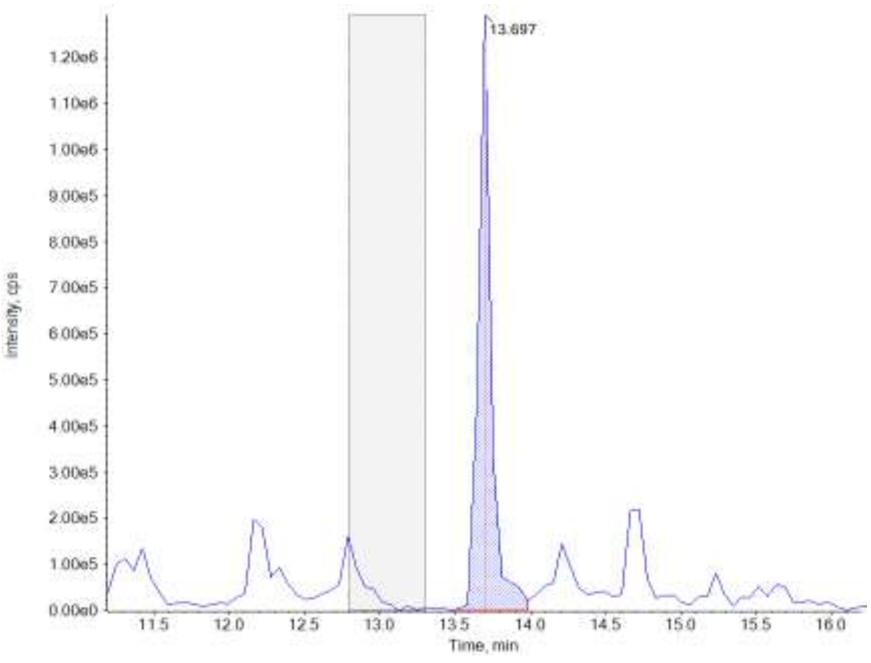


**Acquired / Library MSMS**

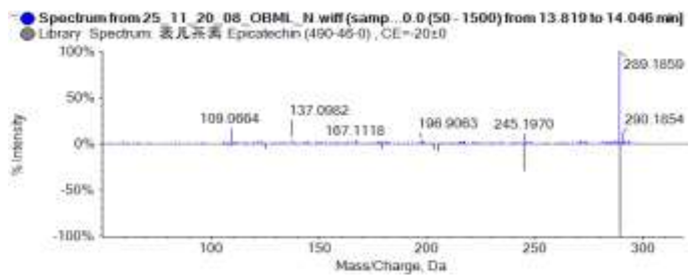


**Acquired / Theoretical MS**

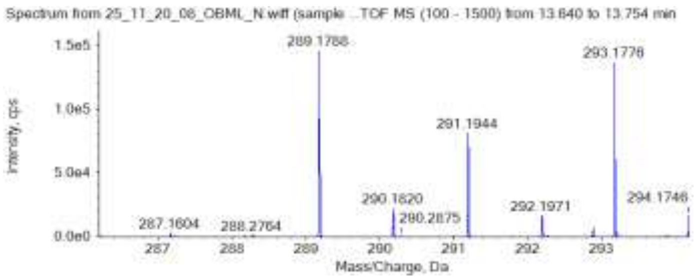

## 197.1163 / 13.81

(Mass/FragMass/RT/Isotope/Library/Formula/Ion Ratio)

**Retention Time:** 13.73 minutes
**Precursor m/z :** 197.1164
**Fit (%)** 50.9% **RFit (%)** 100.0%

**Exp RT:** 13.81 minutes
**Analyte Name:**
197.1163 / 13.81

**Collision Energy = 35 ± 15 eV**

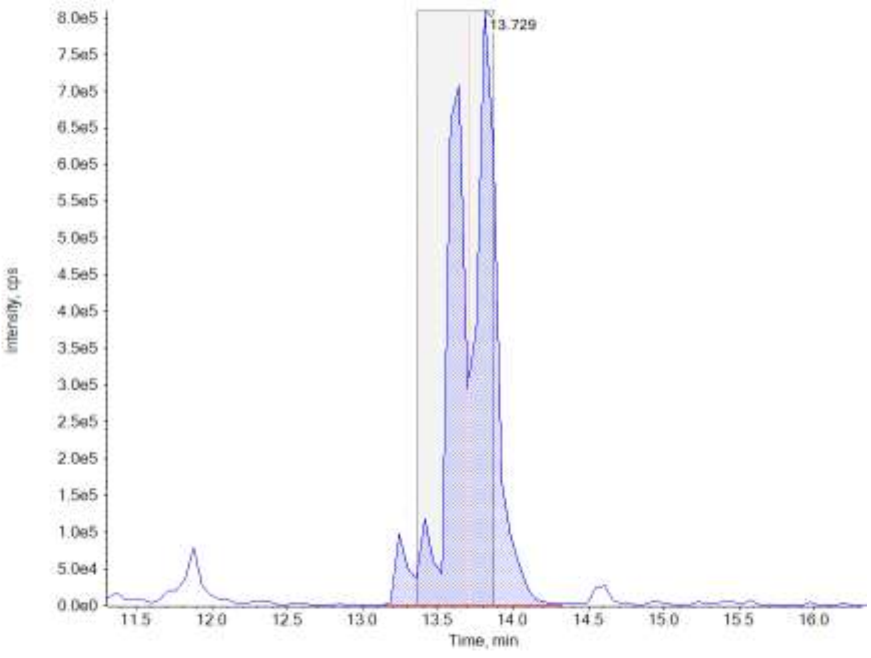


**Acquired / Library MSMS**

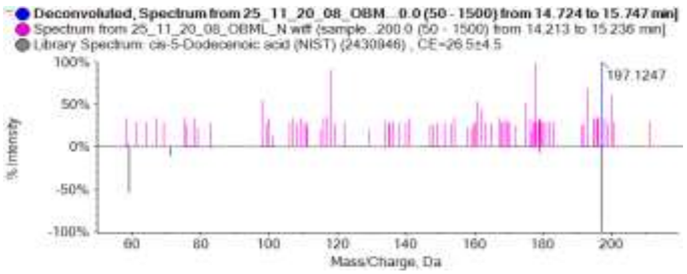


**Acquired / Theoretical MS**

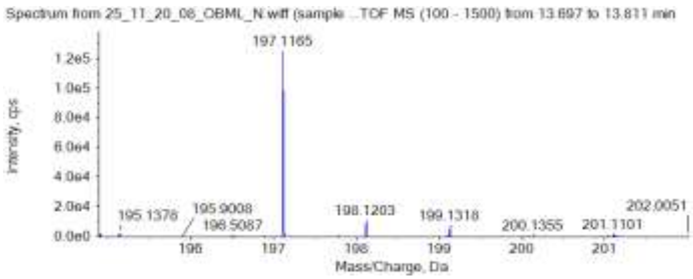

## 665.4274 / 13.98 [M+FA-H]- (Mass/FragMass/RT/Isotope/Library/Formula/Ion Ratio)

| | |
|---|---|
| **Retention Time:** 13.98 minutes | **Exp RT:** 13.98 minutes |
| **Precursor m/z :** 665.4274 | **Analyte Name:** |
| **Fit (%)** 85.3% **RFit (%)** 89.3% | 665.4274 / 13.98 [M+FA-H]- |

**Collision Energy = 35 ± 15 eV**

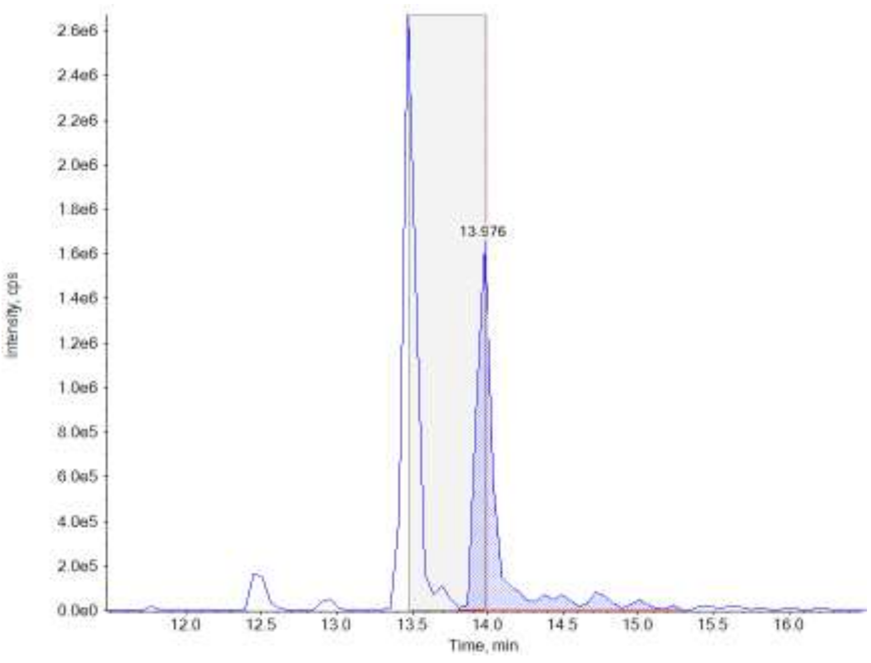


**Acquired / Library MSMS**

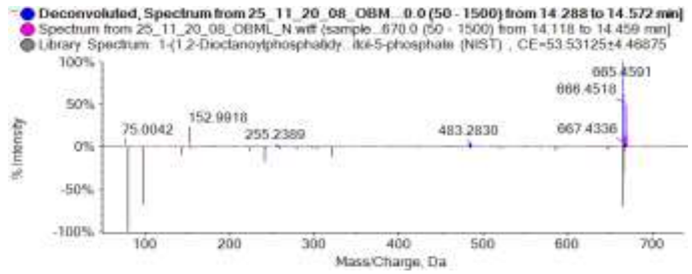


**Acquired / Theoretical MS**

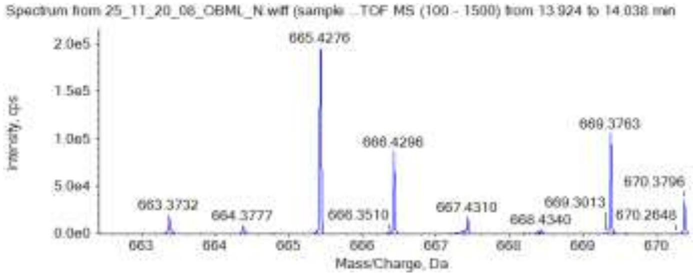

## 483.2779 / 13.92 (Mass/FragMass/RT/Isotope/Library/Formula/Ion Ratio)

**Retention Time:** 13.94 minutes
**Precursor m/z :** 483.2779
**Fit (%)** 99.6% **RFit (%)** 99.0%

**Exp RT:** 13.92 minutes
**Analyte Name:**
483.2779 / 13.92

**Collision Energy = 35 ± 15 eV**

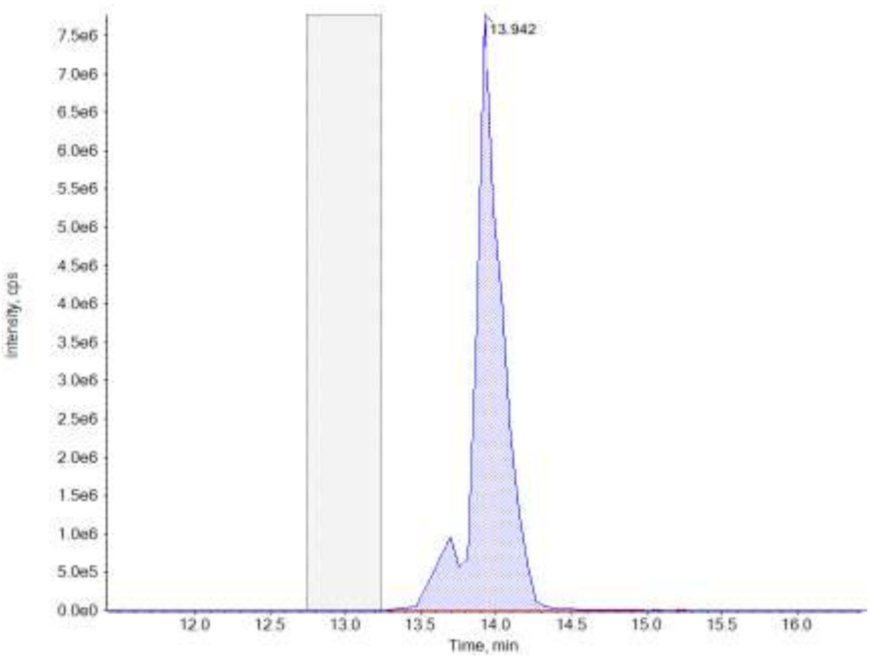


**Acquired / Library MSMS**

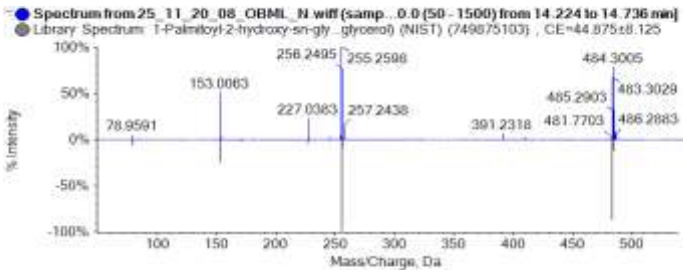


**Acquired / Theoretical MS**

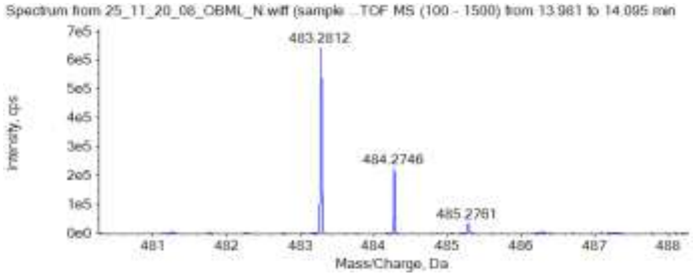

**679.4117 / 13.92** (Mass/FragMass/RT/Isotope/Library/Formula/Ion Ratio)

**Retention Time:** 13.97 minutes **Exp RT:** 13.92 minutes
**Precursor m/z :** 679.4117 **Analyte Name:**
**Fit (%)** 72.4% **RFit (%)** 98.3% 679.4117 / 13.92

**Collision Energy = 35 ± 15 eV**

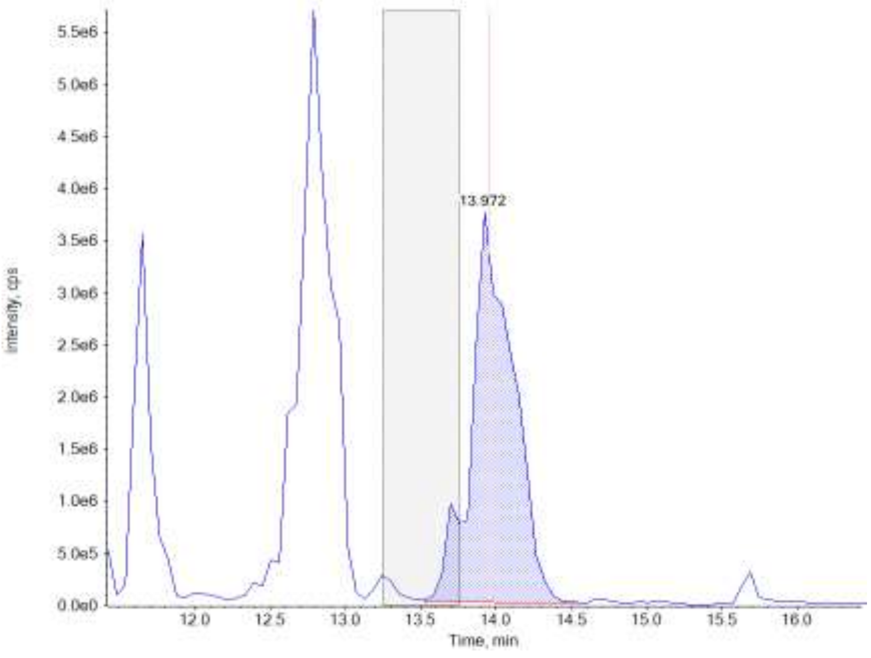


**Acquired / Library MSMS**

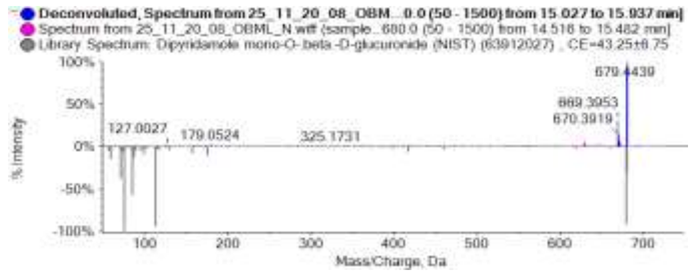


**Acquired / Theoretical MS**

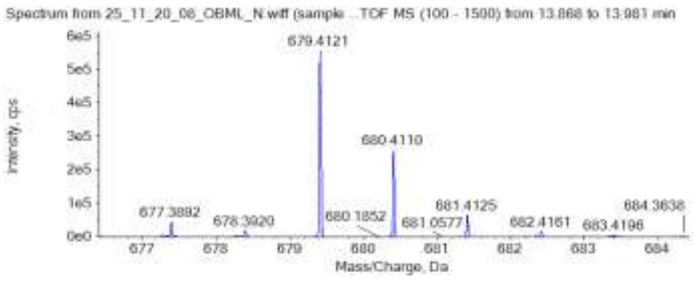

**305.1736 / 14.15** (Mass/FragMass/RT/Isotope/Library/Formula/Ion Ratio)

| | |
|---|---|
| **Retention Time:** 14.17 minutes | **Exp RT:** 14.15 minutes |
| **Precursor m/z :** 305.1736 | **Analyte Name:** |
| **Fit (%)** 78.7% **RFit (%)** 74.8% | 305.1736 / 14.15 |

**Collision Energy = 35 ± 15 eV**

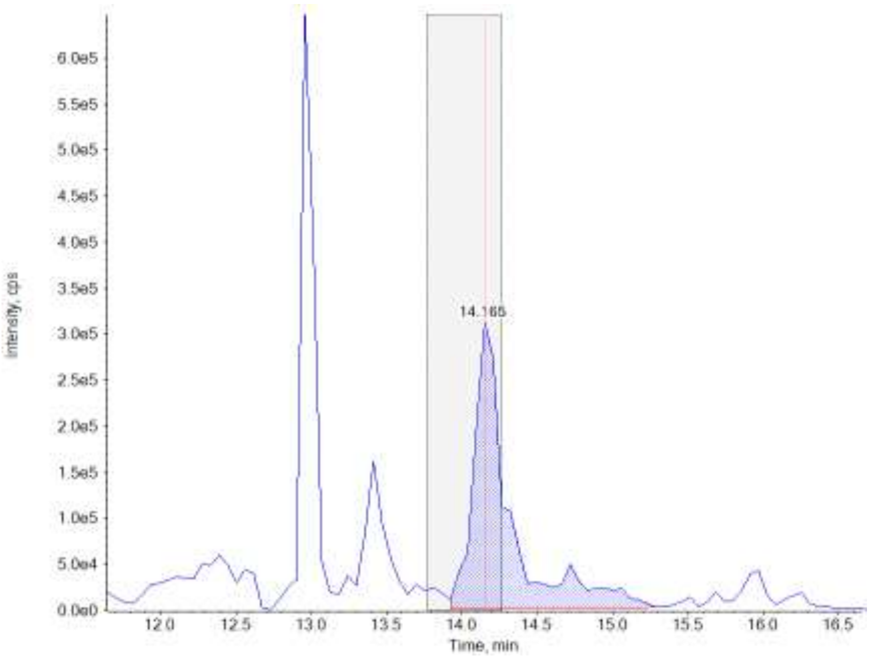


**Acquired / Library MSMS**

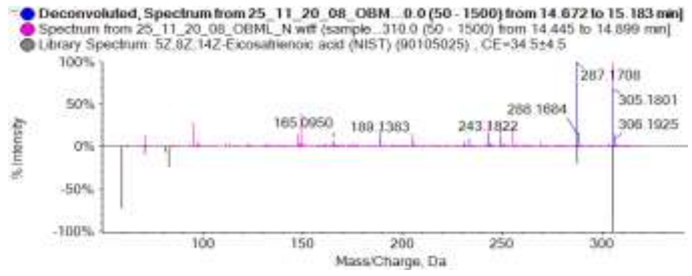


**Acquired / Theoretical MS**

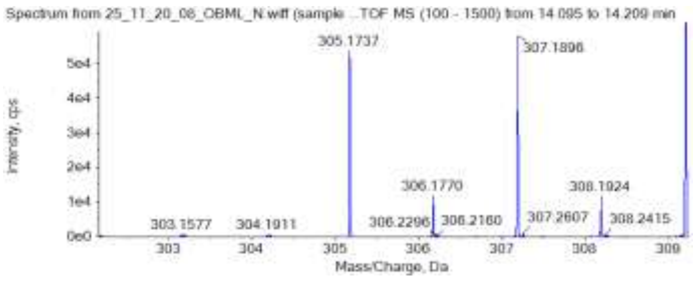

## 695.4024 / 14.21 (Mass/FragMass/RT/Isotope/Library/Formula/Ion Ratio)

| | |
|---|---|
| **Retention Time:** 14.19 minutes | **Exp RT:** 14.21 minutes |
| **Precursor m/z :** 695.4024 | **Analyte Name:** |
| **Fit (%)** 97.0% **RFit (%)** 96.1% | 695.4024 / 14.21 |

**Collision Energy = 35 ± 15 eV**

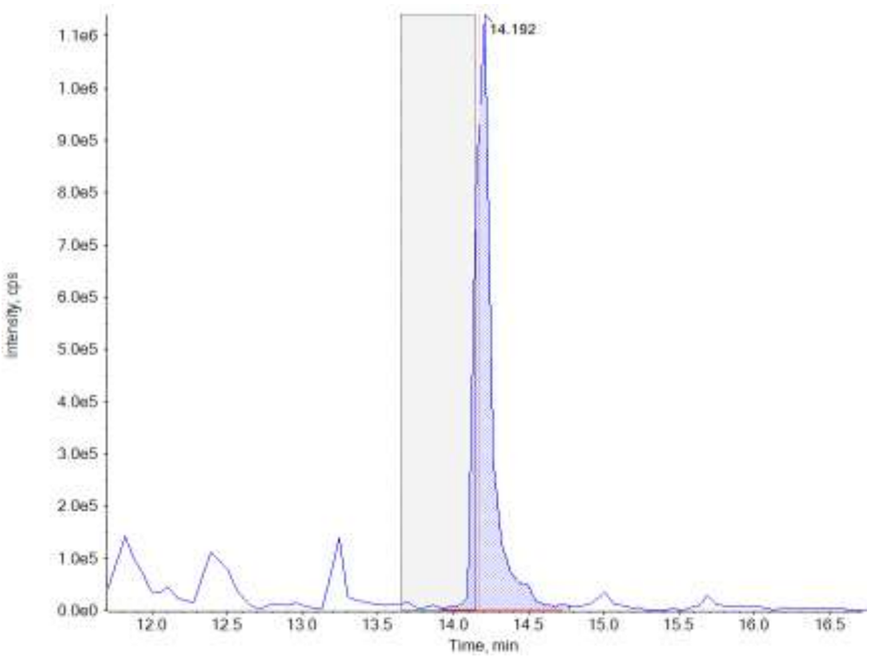


**Acquired / Library MSMS**

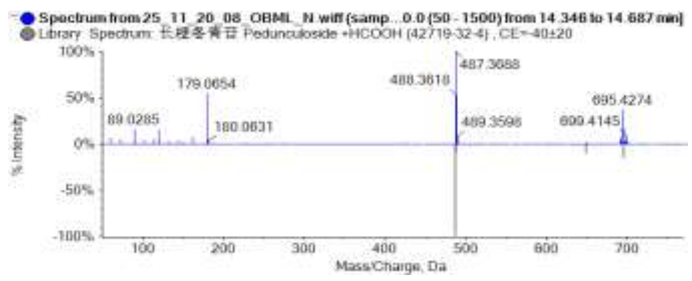


**Acquired / Theoretical MS**

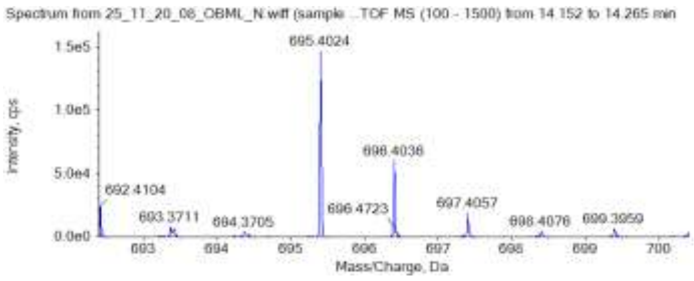

## 509.2960 / 14.32

(Mass/FragMass/RT/Isotope/Library/Formula/Ion Ratio)

| | |
|---|---|
| **Retention Time:** 14.32 minutes | **Exp RT:** 14.32 minutes |
| **Precursor m/z :** 509.2960 | **Analyte Name:** |
| **Fit (%)** 99.7% **RFit (%)** 100.0% | 509.2960 / 14.32 |

**Collision Energy = 35 ± 15 eV**

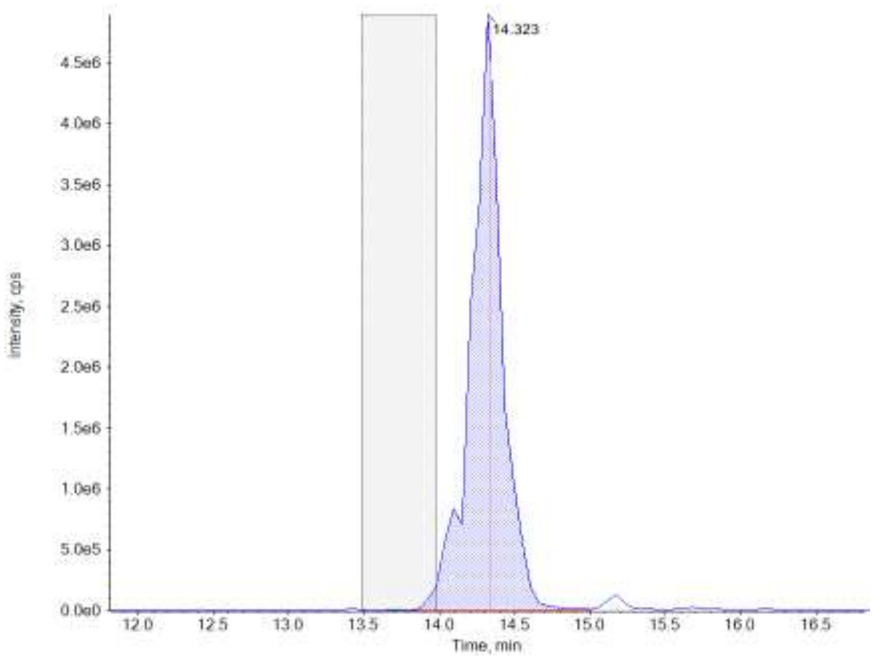


**Acquired / Library MSMS**

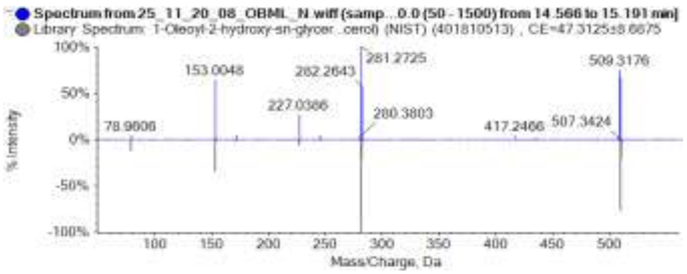


**Acquired / Theoretical MS**

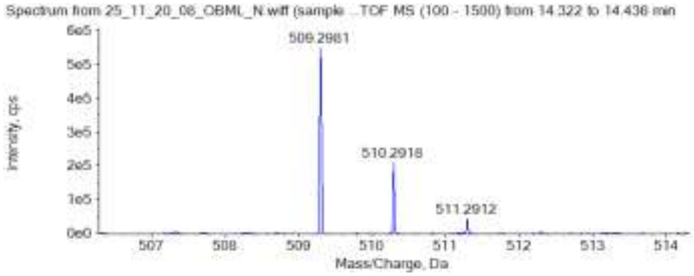

## 279.1946 / 14.38

(Mass/FragMass/RT/Isotope/Library/Formula/Ion Ratio)

**Retention Time:** 14.41 minutes
**Precursor m/z :** 279.1947
**Fit (%)** 99.3% **RFit (%)** 87.2%

**Exp RT:** 14.38 minutes
**Analyte Name:**
279.1946 / 14.38

**Collision Energy = 35 ± 15 eV**

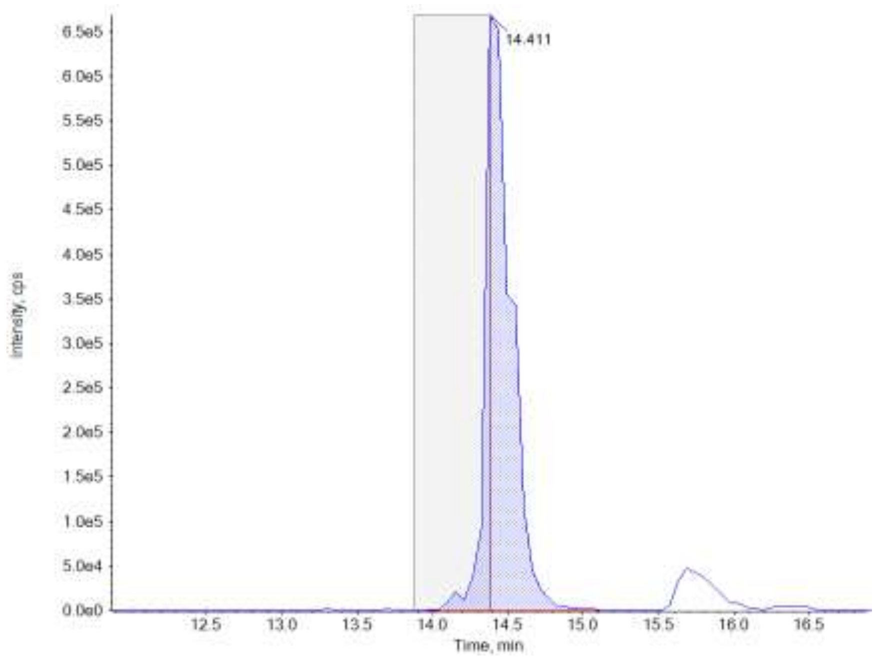


**Acquired / Library MSMS**

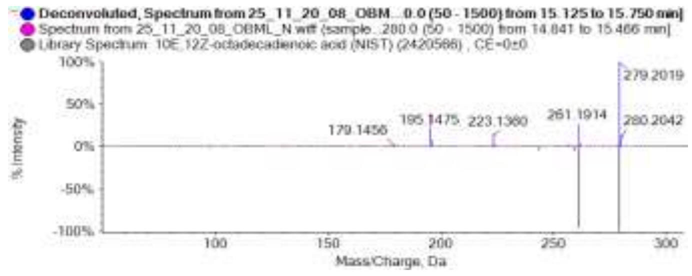


**Acquired / Theoretical MS**

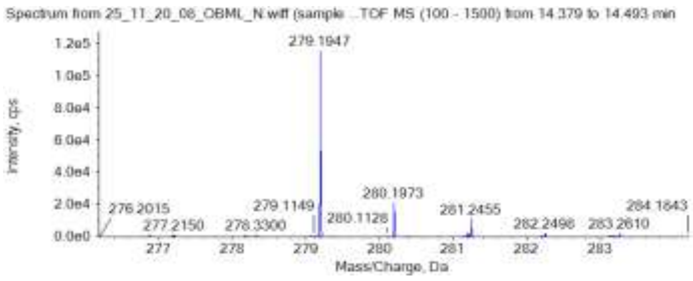

Created with SCIEX OS 3.3.0
Printed:12/9/2025 2:26:41 PM

## 617.3669 / 14.49 (Mass/FragMass/RT/Isotope/Library/Formula/Ion Ratio)

| | |
|---|---|
| **Retention Time:** 14.55 minutes | **Exp RT:** 14.49 minutes |
| **Precursor m/z :** 617.3669 | **Analyte Name:** |
| **Fit (%)** 46.0% **RFit (%)** 93.6% | 617.3669 / 14.49 |

**Collision Energy = 35 ± 15 eV**

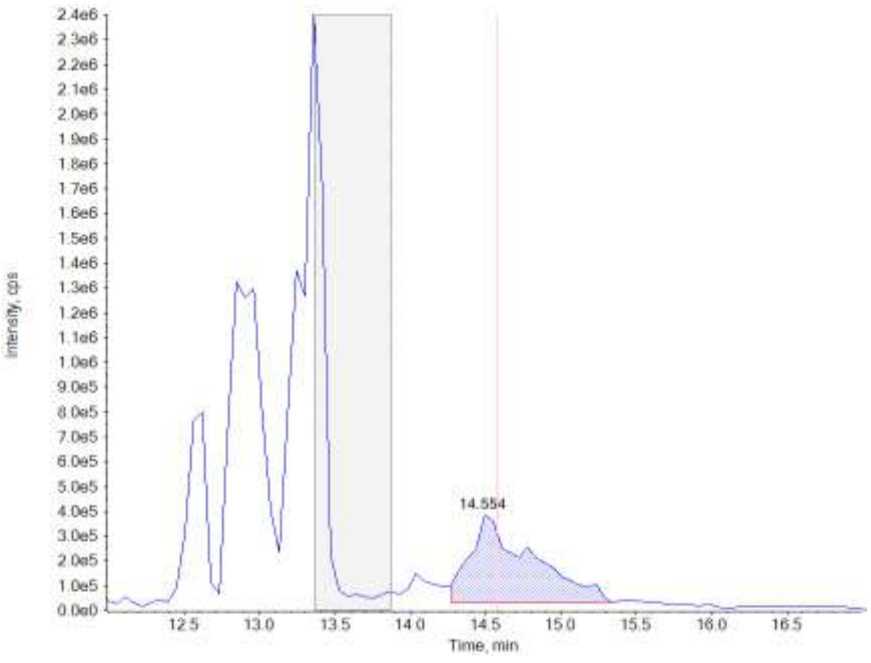


**Acquired / Library MSMS**

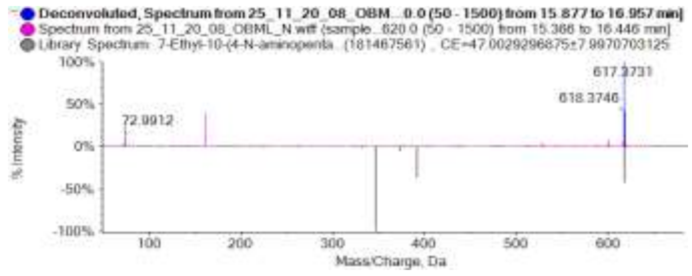


**Acquired / Theoretical MS**

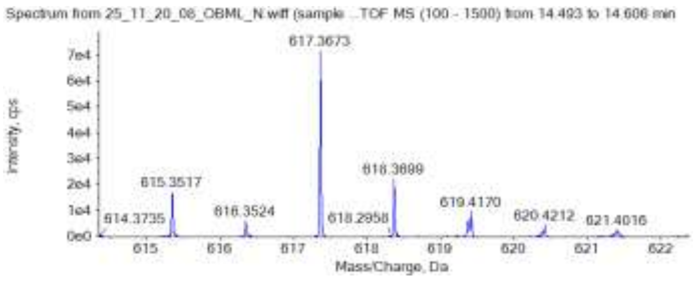

**311.2227 / 14.55** (Mass/FragMass/RT/Isotope/Library/Formula/Ion Ratio)

| | |
|---|---|
| **Retention Time:** 14.47 minutes | **Exp RT:** 14.55 minutes |
| **Precursor m/z :** 311.2227 | **Analyte Name:** |
| **Fit (%)** 40.9% **RFit (%)** 96.4% | 311.2227 / 14.55 |

**Collision Energy = 35 ± 15 eV**

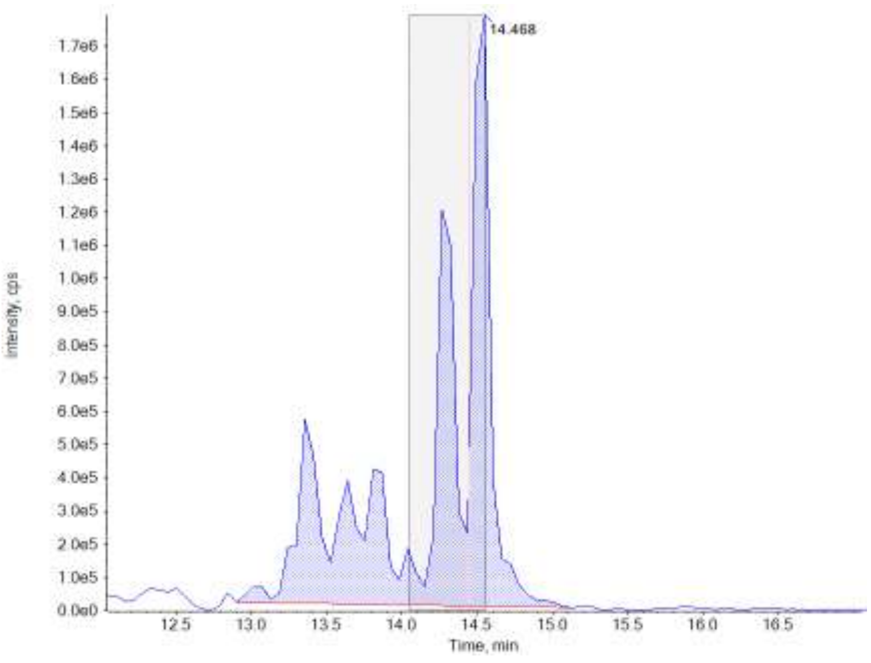


**Acquired / Library MSMS**

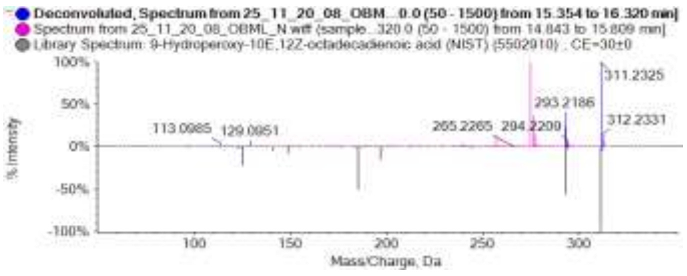


**Acquired / Theoretical MS**

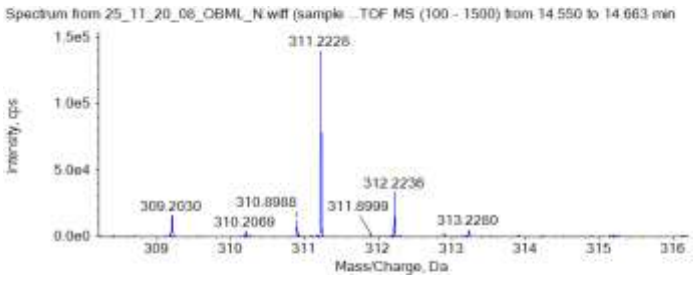

## 831.4924 / 14.55 (Mass/FragMass/RT/Isotope/Library/Formula/Ion Ratio)

**Retention Time:** 14.59 minutes
**Precursor m/z :** 831.4924
**Fit (%)** 93.3% **RFit (%)** 93.3%

**Exp RT:** 14.55 minutes
**Analyte Name:**
831.4924 / 14.55

**Collision Energy = 35 ± 15 eV**

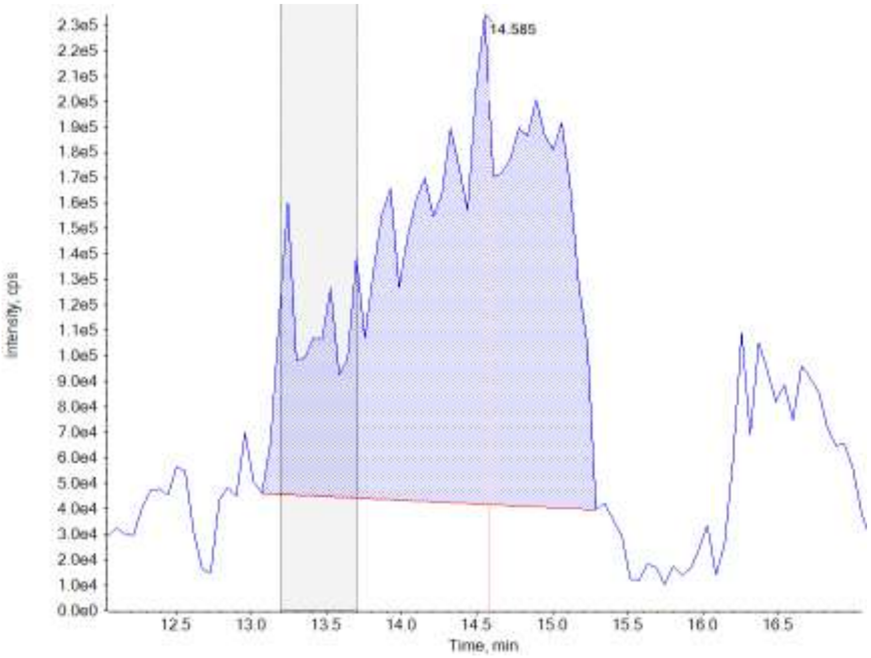


**Acquired / Library MSMS**

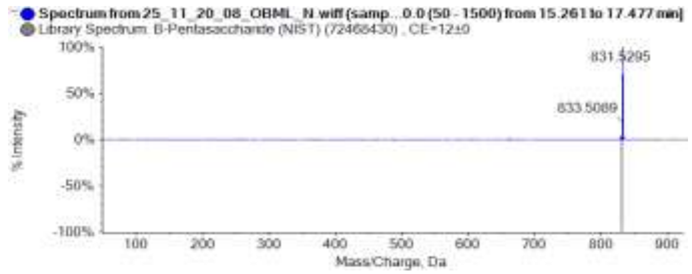


**Acquired / Theoretical MS**

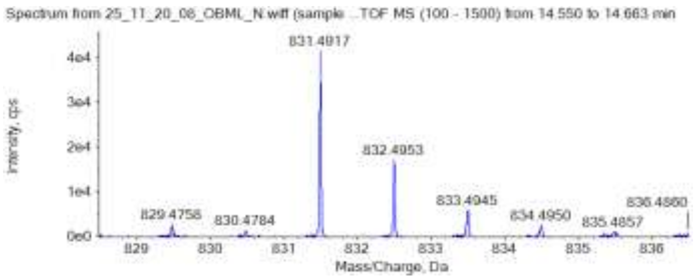

Created with SCIEX OS 3.3.0
Printed:12/9/2025 2:26:41 PM

## 681.3411 / 14.66 [M+Cl]- (Mass/FragMass/RT/Isotope/Library/Formula/Ion Ratio)

**Retention Time:** 14.63 minutes
**Precursor m/z :** 681.3411
**Fit (%)** 84.7% **RFit (%)** 82.8%

**Exp RT:** 14.66 minutes
**Analyte Name:**
681.3411 / 14.66 [M+Cl]-

**Collision Energy = 35 ± 15 eV**

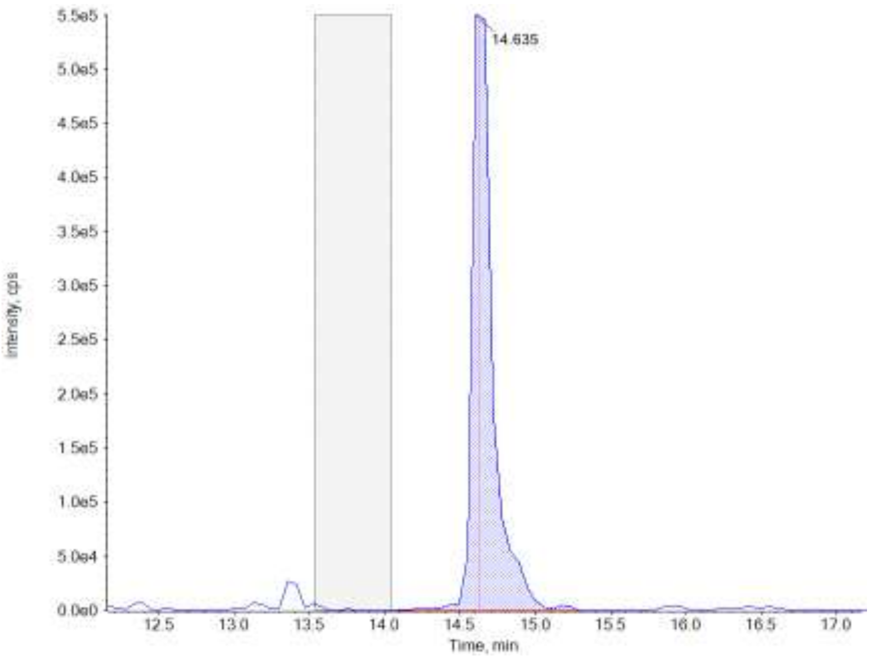


Acquired / Library MSMS

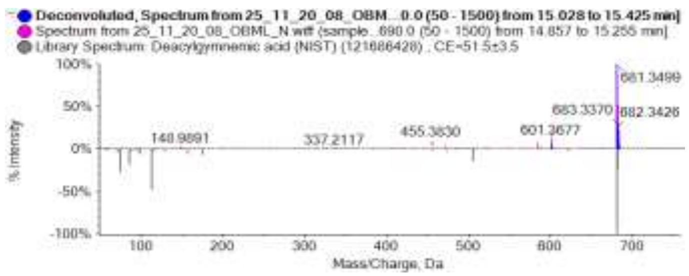


Acquired / Theoretical MS

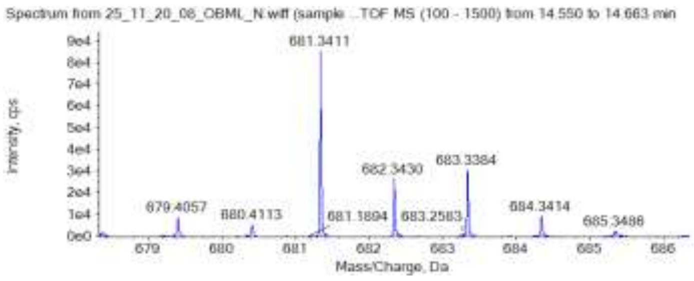

**289.1787 / 14.72** (Mass/FragMass/RT/Isotope/Library/Formula/Ion Ratio)

| | |
|---|---|
| **Retention Time:** 14.69 minutes | **Exp RT:** 14.72 minutes |
| **Precursor m/z :** 289.1787 | **Analyte Name:** |
| **Fit (%)** 25.0% **RFit (%)** 100.0% | 289.1787 / 14.72 |

**Collision Energy = 35 ± 15 eV**

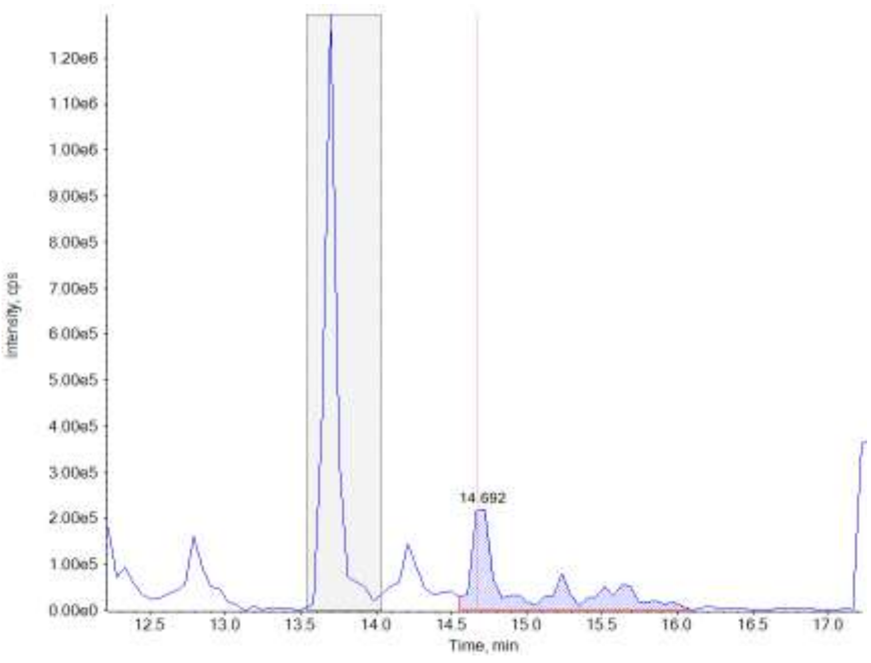


**Acquired / Library MSMS**

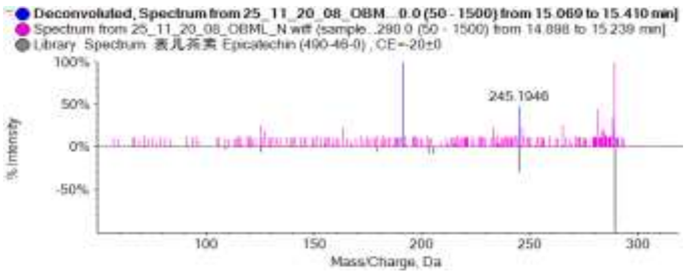


**Acquired / Theoretical MS**

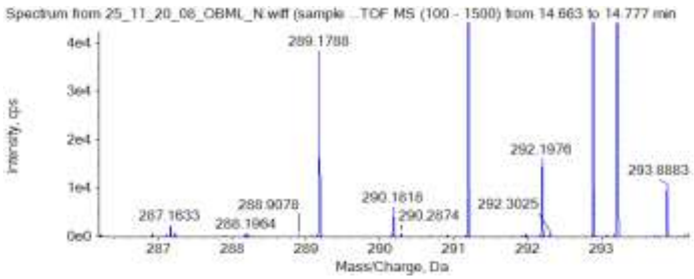

**291.2008 / 14.83** (Mass/FragMass/RT/Isotope/Library/Formula/Ion Ratio)

| | |
|---|---|
| **Retention Time:** 14.85 minutes | **Exp RT:** 14.83 minutes |
| **Precursor m/z :** 291.2008 | **Analyte Name:** |
| **Fit (%)** 77.7% **RFit (%)** 62.8% | 291.2008 / 14.83 |

**Collision Energy = 35 ± 15 eV**

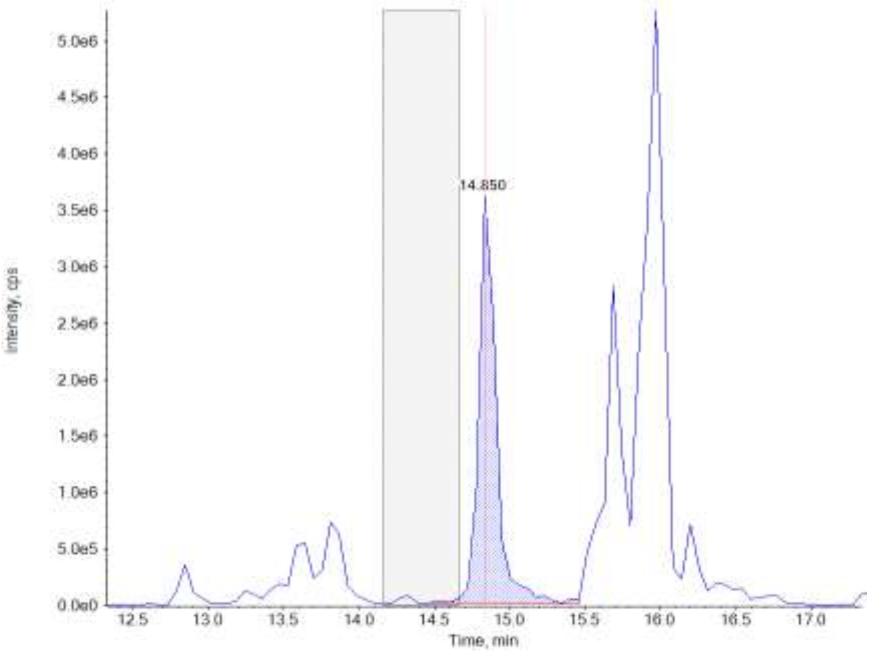


**Acquired / Library MSMS**

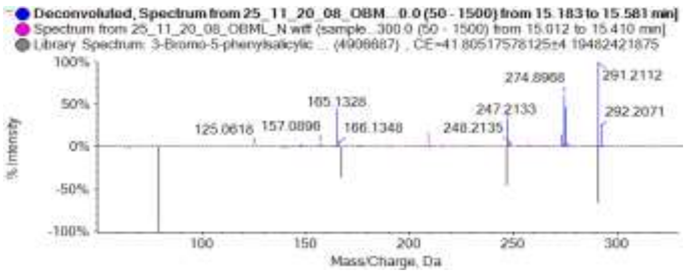


**Acquired / Theoretical MS**

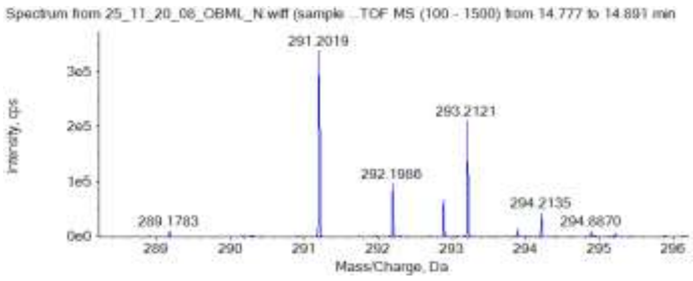

Created with SCIEX OS 3.3.0
Printed:12/9/2025 2:26:41 PM

**583.3172 / 15.00** (Mass/FragMass/RT/Isotope/Library/Formula/Ion Ratio)

| **Retention Time:** 14.99 minutes | **Exp RT:** 15.00 minutes |
|---|---|
| **Precursor m/z :** 583.3172 | **Analyte Name:** |
| **Fit (%)** 35.4% **RFit (%)** 95.1% | 583.3172 / 15.00 |

**Collision Energy = 35 ± 15 eV**

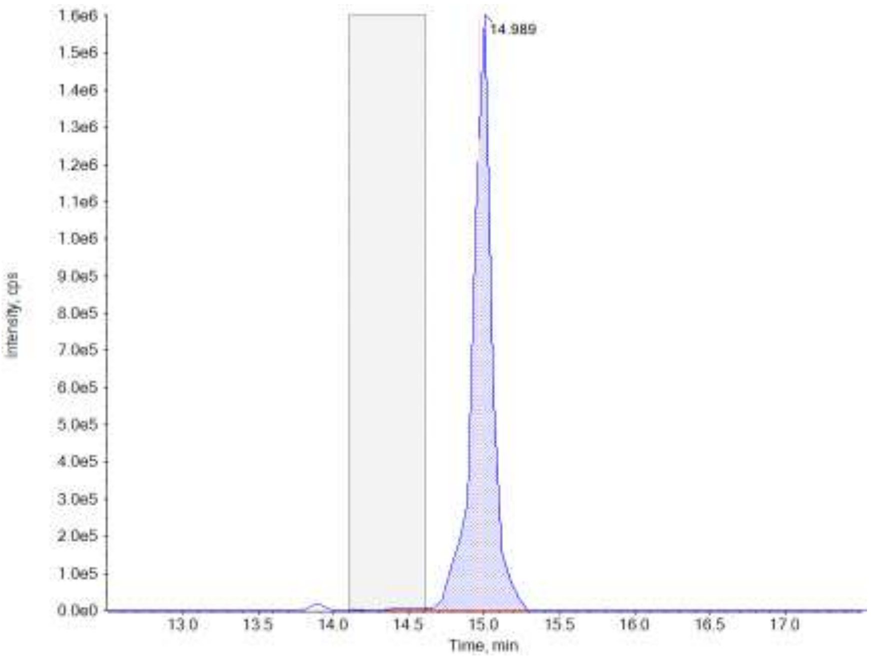


**Acquired / Library MSMS**

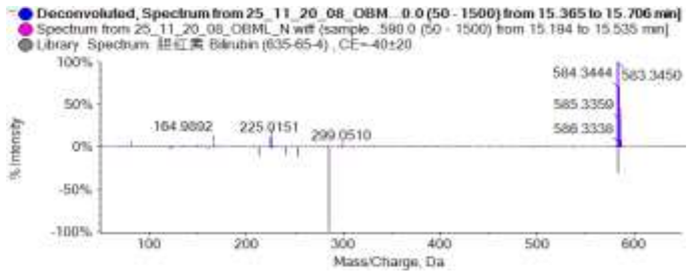


**Acquired / Theoretical MS**

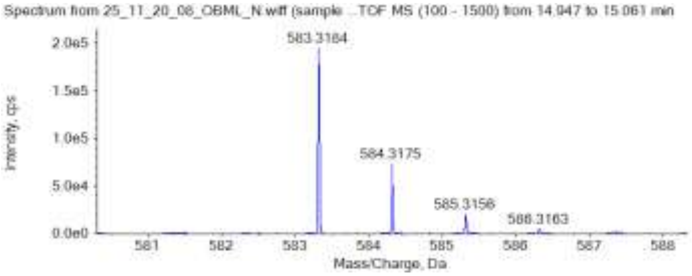

**365.2686 / 15.06** (Mass/FragMass/RT/Isotope/Library/Formula/Ion Ratio)

**Retention Time:** 15.04 minutes
**Precursor m/z :** 365.2686
**Fit (%)** 96.2% **RFit (%)** 93.0%

**Exp RT:** 15.06 minutes
**Analyte Name:**
365.2686 / 15.06

**Collision Energy = 35 ± 15 eV**

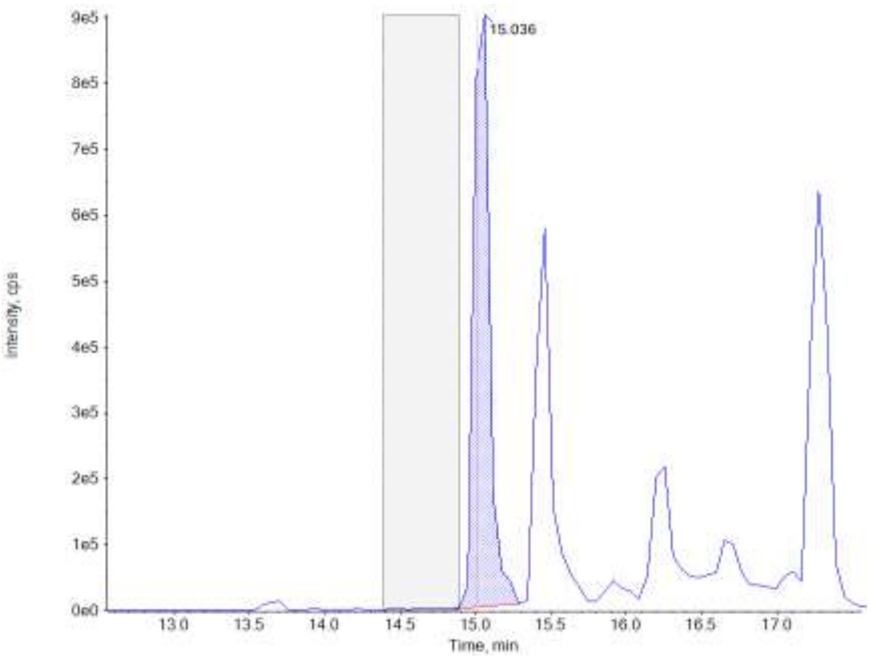


**Acquired / Library MSMS**

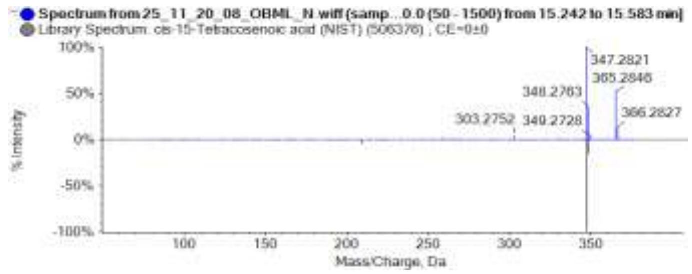


**Acquired / Theoretical MS**

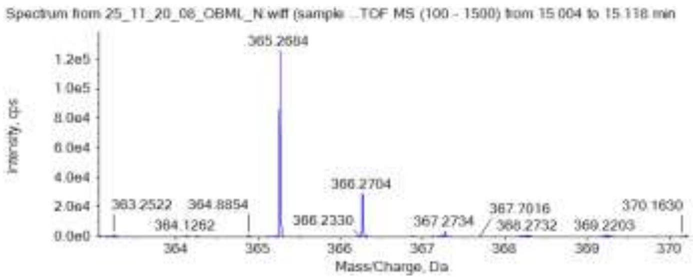

## 517.3580 / 15.12 (Mass/FragMass/RT/Isotope/Library/Formula/Ion Ratio)

**Retention Time:** 15.12 minutes
**Precursor m/z :** 517.3580
**Fit (%)** 65.5% **RFit (%)** 99.3%

**Exp RT:** 15.12 minutes
**Analyte Name:**
517.3580 / 15.12

**Collision Energy = 35 ± 15 eV**

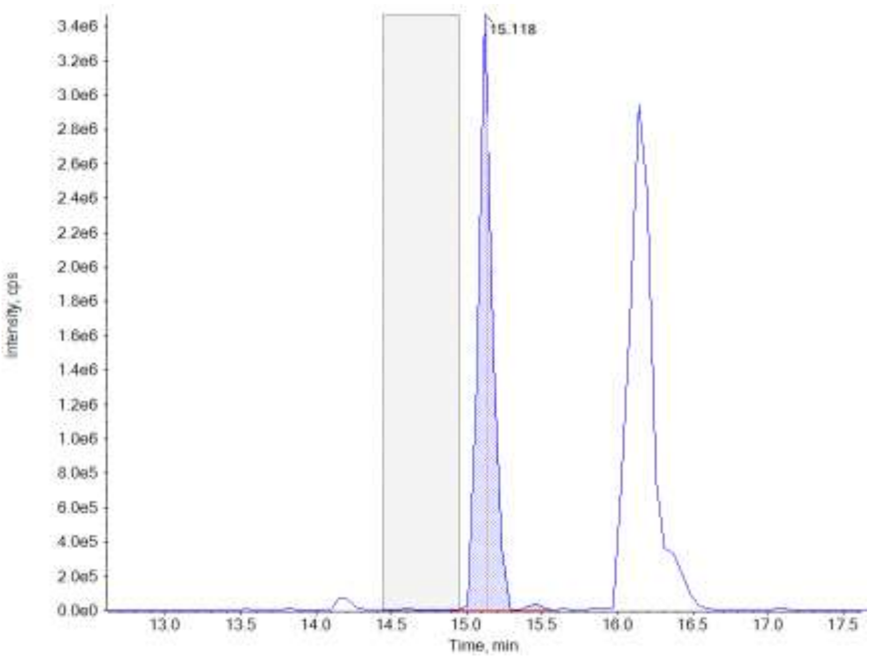


**Acquired / Library MSMS**

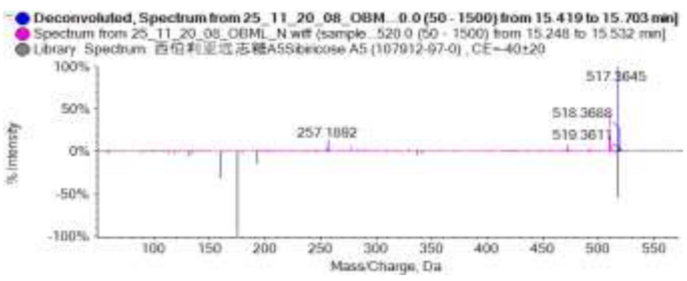


**Acquired / Theoretical MS**

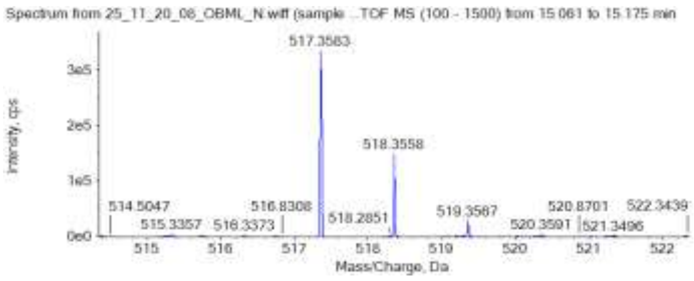

## 235.1686 / 15.17 (Mass/FragMass/RT/Isotope/Library/Formula/Ion Ratio)

| | |
|---|---|
| **Retention Time:** 15.15 minutes | **Exp RT:** 15.18 minutes |
| **Precursor m/z :** 235.1686 | **Analyte Name:** |
| **Fit (%)** 97.7% **RFit (%)** 92.8% | 235.1686 / 15.17 |

**Collision Energy = 35 ± 15 eV**

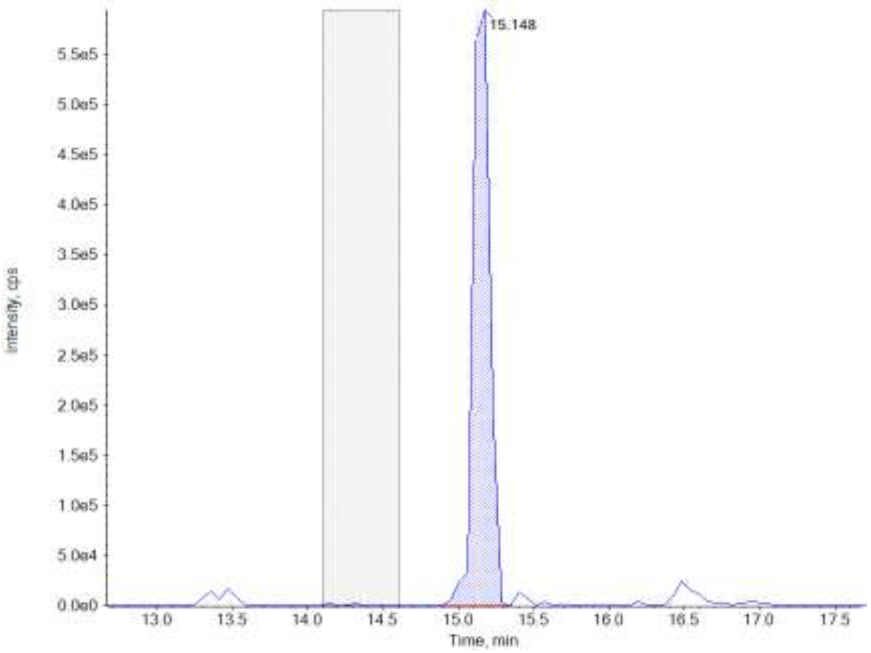


**Acquired / Library MSMS**

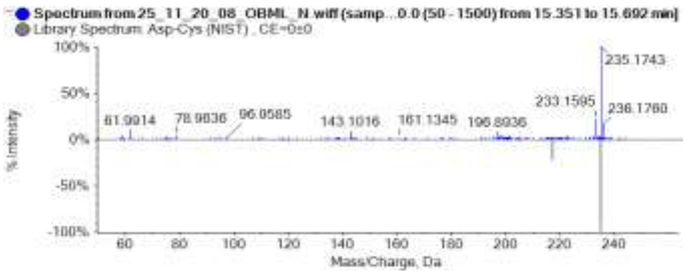


**Acquired / Theoretical MS**

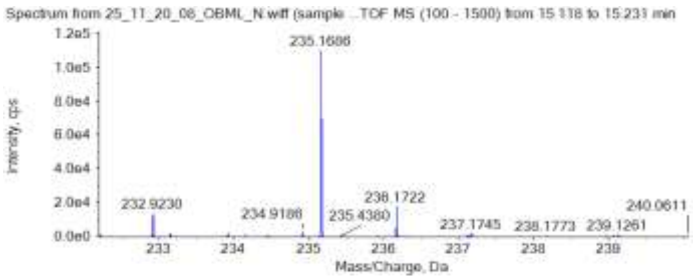

**275.2017 / 15.17** (Mass/FragMass/RT/Isotope/Library/Formula/Ion Ratio)

**Retention Time:** 15.18 minutes **Exp RT:** 15.18 minutes
**Precursor m/z :** 275.2017 **Analyte Name:**
**Fit (%)** 95.1% **RFit (%)** 98.4% 275.2017 / 15.17

**Collision Energy = 35 ± 15 eV**

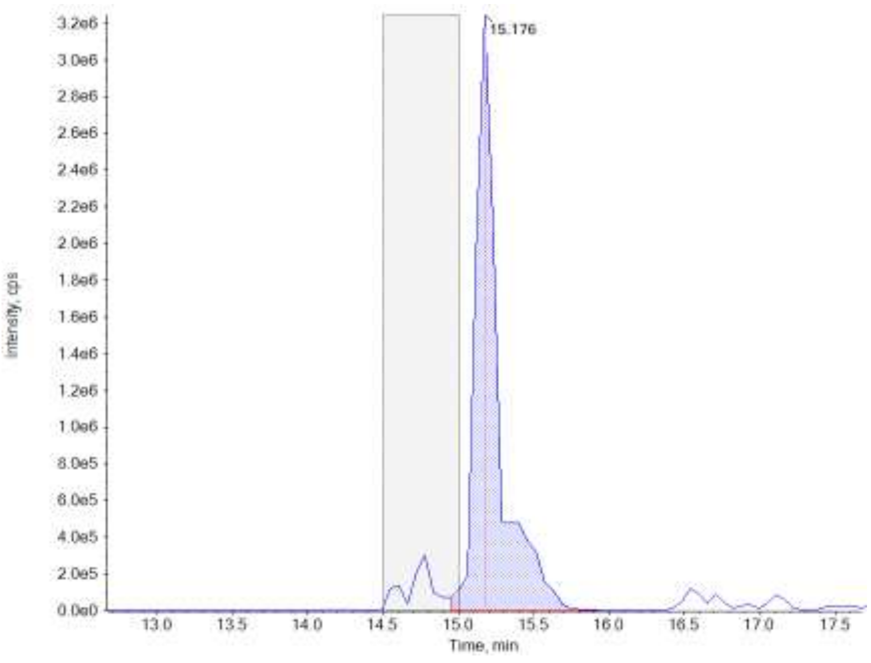


**Acquired / Library MSMS**

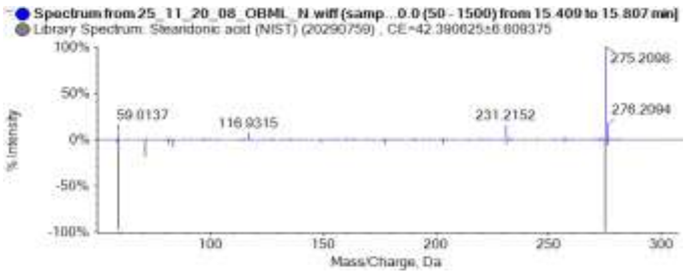


**Acquired / Theoretical MS**

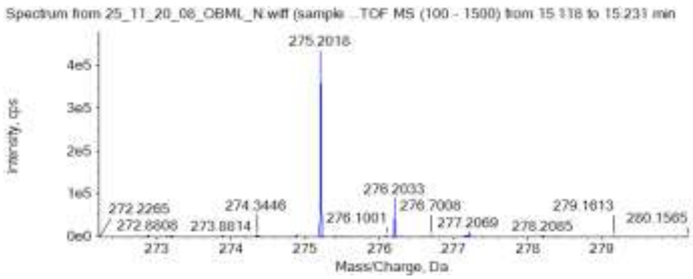

## 911.6336 / 15.29 (Mass/FragMass/RT/Isotope/Library/Formula/Ion Ratio)

**Retention Time:** 15.32 minutes | **Exp RT:** 15.29 minutes
**Precursor m/z :** 911.6336 | **Analyte Name:**
**Fit (%)** 92.1% **RFit (%)** 85.1% | 911.6336 / 15.29

**Collision Energy = 35 ± 15 eV**

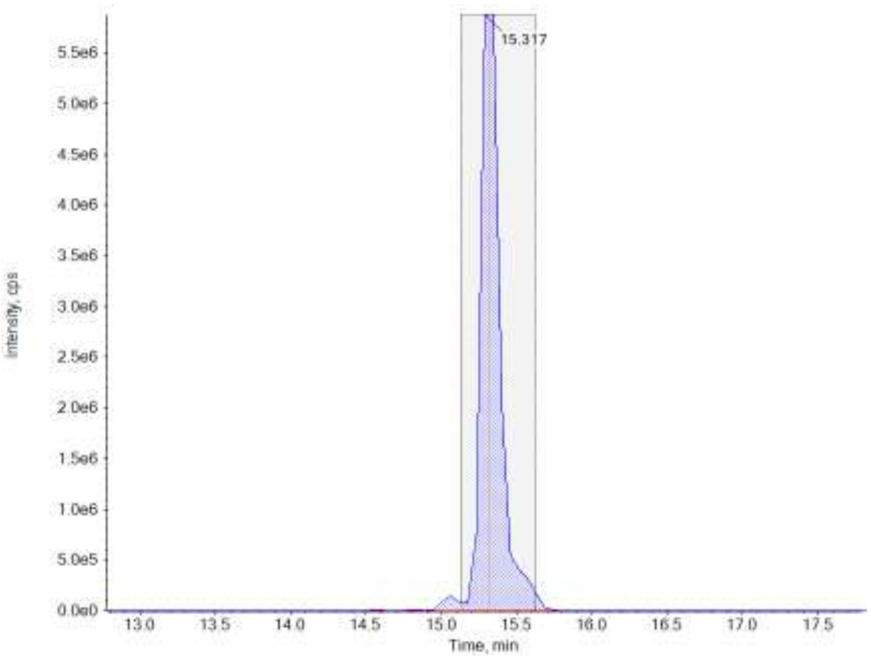


Acquired / Library MSMS

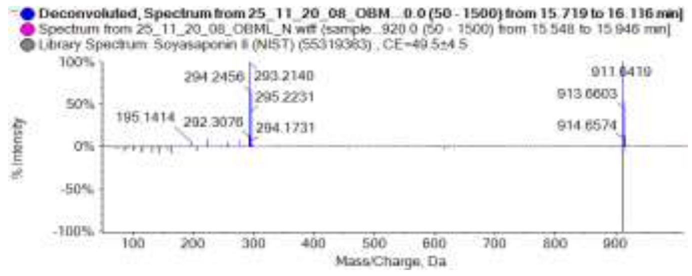


Acquired / Theoretical MS

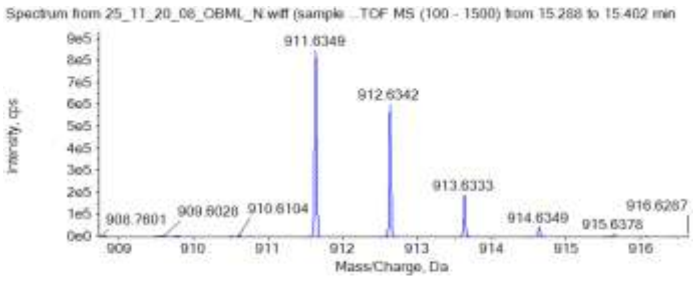

## 293.2260 / 15.35

(Mass/FragMass/RT/Isotope/Library/Formula/Ion Ratio)

**Retention Time:** 15.39 minutes
**Precursor m/z :** 293.2260
**Fit (%)** 99.7% **RFit (%)** 97.0%

**Exp RT:** 15.35 minutes
**Analyte Name:**
293.2260 / 15.35

**Collision Energy = 35 ± 15 eV**

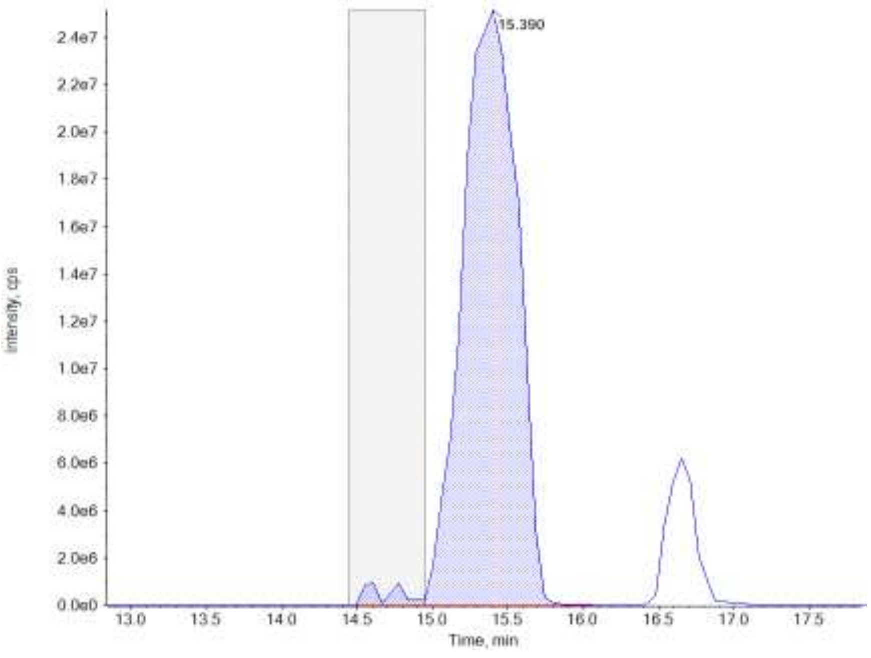


**Acquired / Library MSMS**

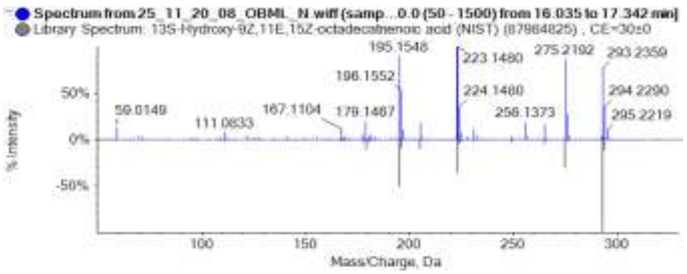


**Acquired / Theoretical MS**

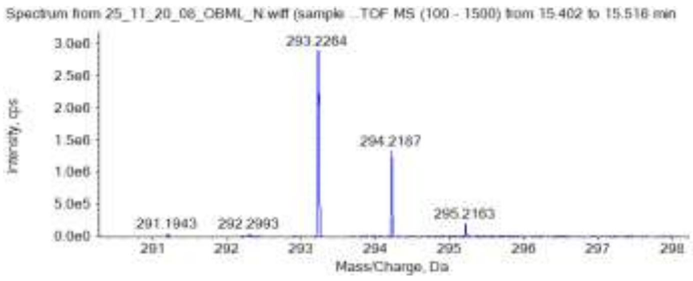

**511.3075 / 15.40** (Mass/FragMass/RT/Isotope/Library/Formula/Ion Ratio)

| | |
|---|---|
| **Retention Time:** 15.45 minutes | **Exp RT:** 15.40 minutes |
| **Precursor m/z :** 511.3075 | **Analyte Name:** |
| **Fit (%)** 93.4% **RFit (%)** 91.8% | 511.3075 / 15.40 |

**Collision Energy = 35 ± 15 eV**

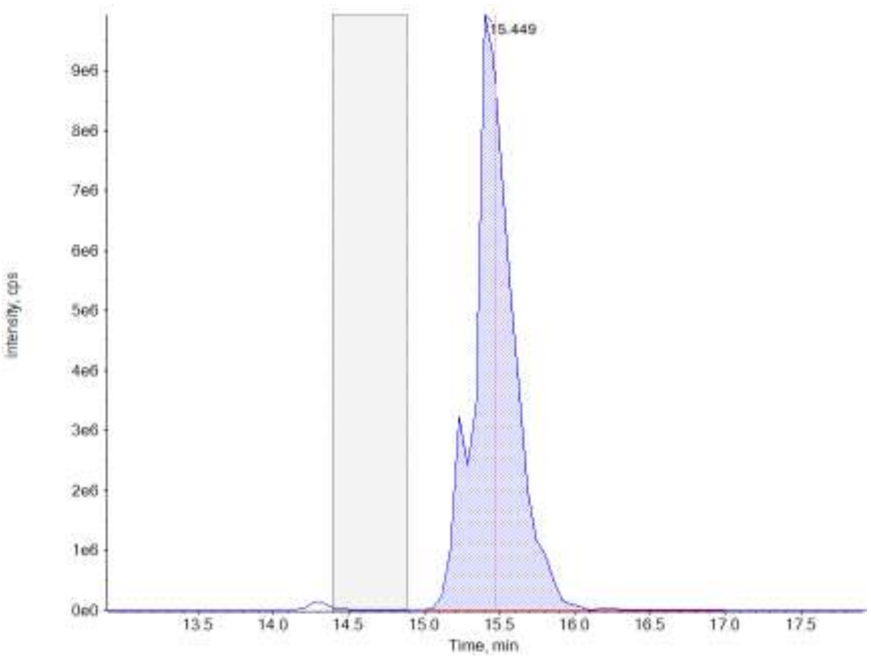


**Acquired / Library MSMS**

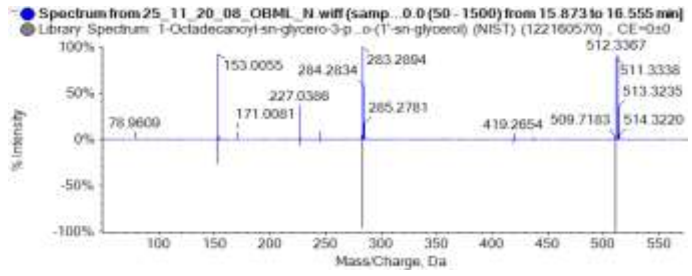


**Acquired / Theoretical MS**

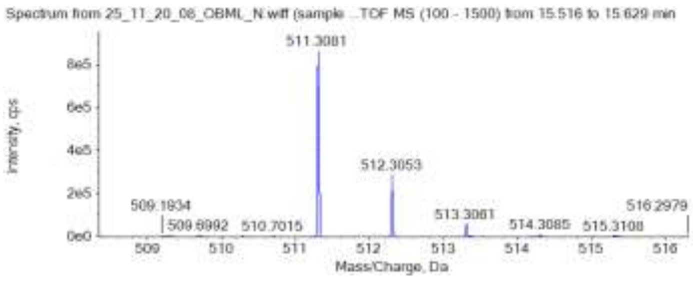

## 271.2257 / 15.74

(Mass/FragMass/RT/Isotope/Library/Formula/Ion Ratio)

| | |
|---|---|
| **Retention Time:** 15.76 minutes | **Exp RT:** 15.74 minutes |
| **Precursor m/z :** 271.2257 | **Analyte Name:** |
| **Fit (%)** 94.4% **RFit (%)** 96.3% | 271.2257 / 15.74 |

**Collision Energy = 35 ± 15 eV**

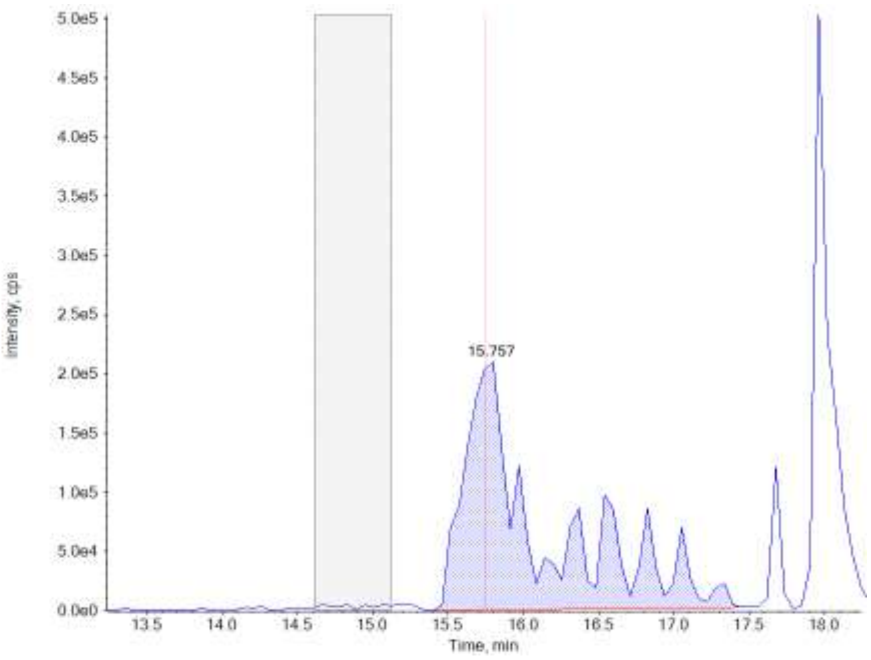


**Acquired / Library MSMS**

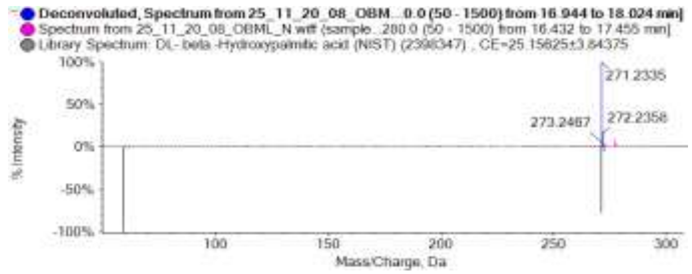


**Acquired / Theoretical MS**

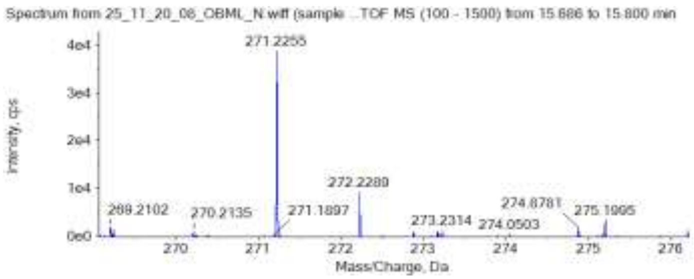

## 295.2253 / 15.86 (Mass/FragMass/RT/Isotope/Library/Formula/Ion Ratio)

**Retention Time:** 16.22 minutes **Exp RT:** 15.86 minutes
**Precursor m/z :** 295.2253 **Analyte Name:**
**Fit (%)** 98.3% **RFit (%)** 96.7% 295.2253 / 15.86

**Collision Energy = 35 ± 15 eV**

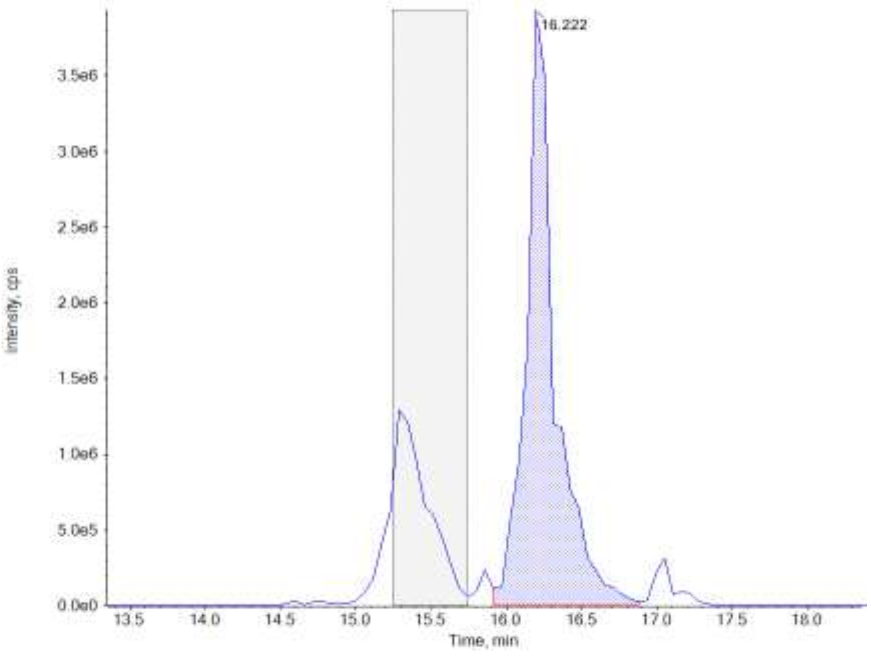


**Acquired / Library MSMS**

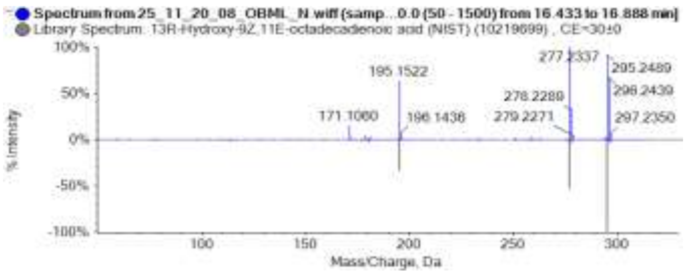


**Acquired / Theoretical MS**

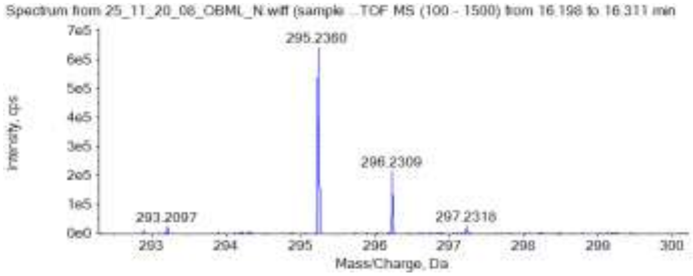

**291.2014 / 15.97** (Mass/FragMass/RT/Isotope/Library/Formula/Ion Ratio)

**Retention Time:** 15.95 minutes
**Precursor m/z :** 291.2014
**Fit (%)** 95.7% **RFit (%)** 78.6%

**Exp RT:** 15.97 minutes
**Analyte Name:**
291.2014 / 15.97

**Collision Energy = 35 ± 15 eV**

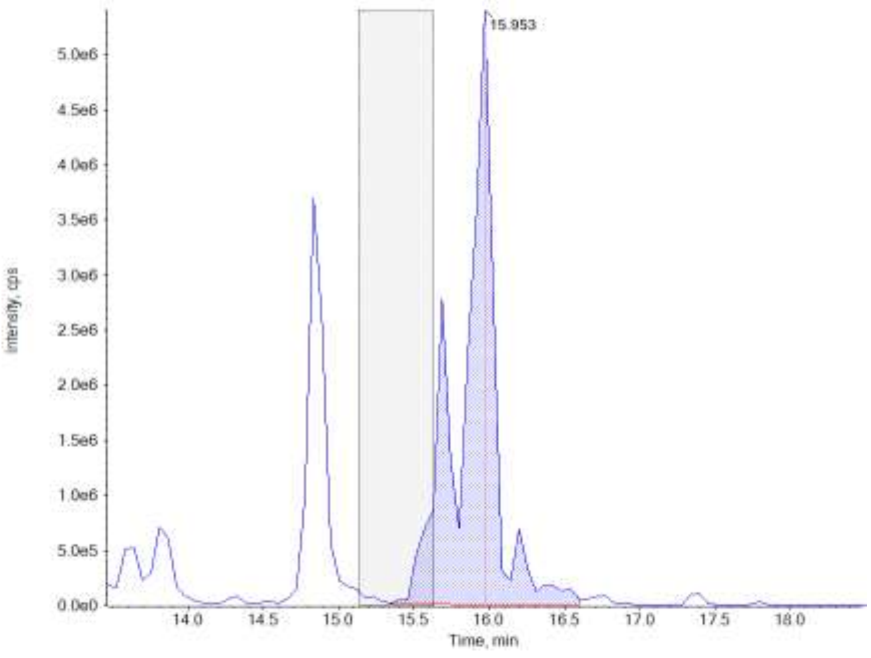


Acquired / Library MSMS

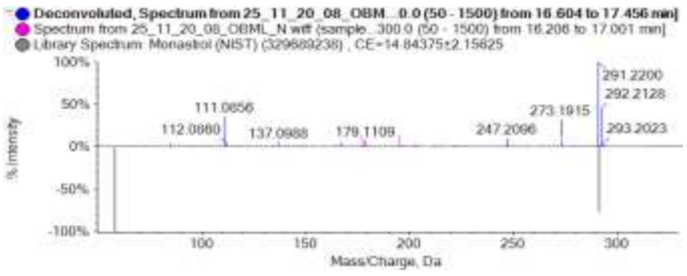


Acquired / Theoretical MS

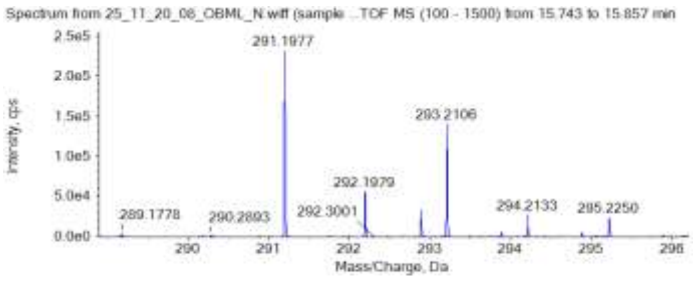

**517.3580 / 16.14** (Mass/FragMass/RT/Isotope/Library/Formula/Ion Ratio)

**Retention Time:** 16.16 minutes
**Precursor m/z :** 517.3580
**Fit (%)** 62.9% **RFit (%)** 97.8%

**Exp RT:** 16.14 minutes
**Analyte Name:**
517.3580 / 16.14

**Collision Energy = 35 ± 15 eV**

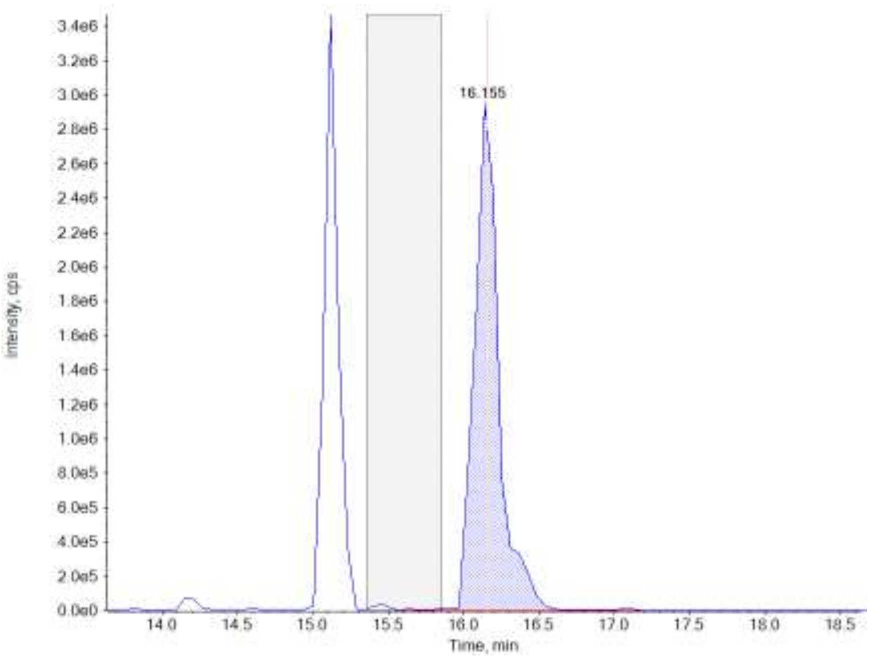


**Acquired / Library MSMS**

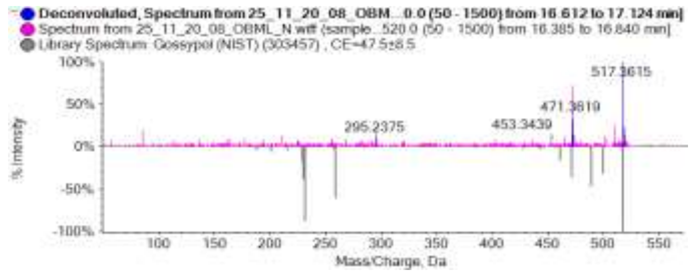


**Acquired / Theoretical MS**

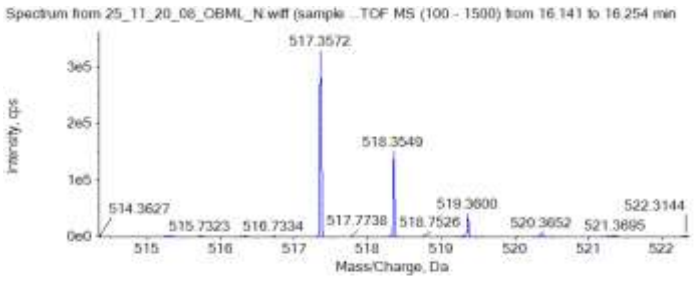

## 699.3827 / 16.14 (Mass/FragMass/RT/Isotope/Library/Formula/Ion Ratio)

**Retention Time:** 16.11 minutes **Exp RT:** 16.14 minutes
**Precursor m/z :** 699.3827 **Analyte Name:**
**Fit (%)** 98.6% **RFit (%)** 47.3% 699.3827 / 16.14

**Collision Energy = 35 ± 15 eV**

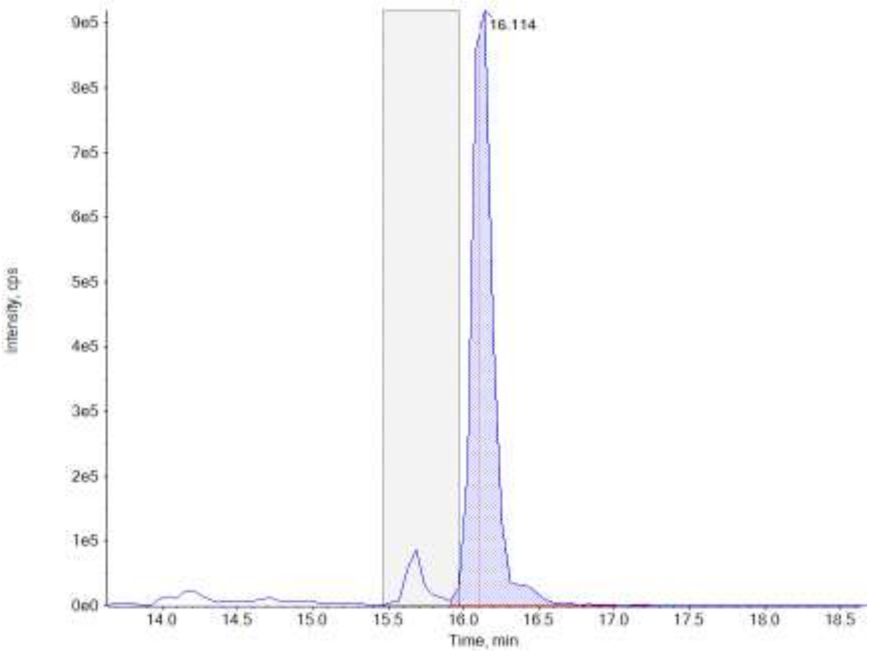

**Acquired / Library MSMS**

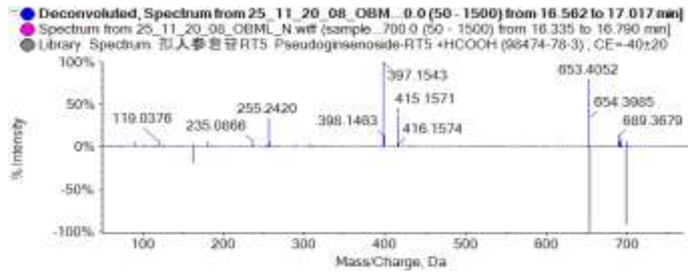

**Acquired / Theoretical MS**

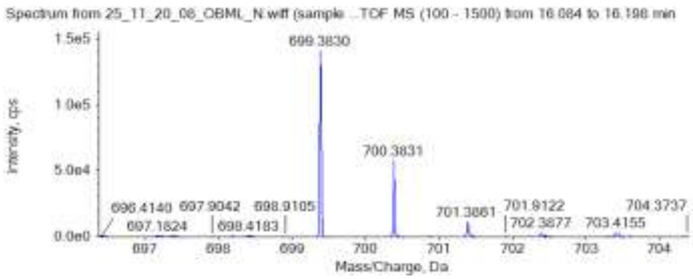

Created with SCIEX OS 3.3.0
Printed:12/9/2025 2:26:41 PM

## 295.2390 / 16.20 (Mass/FragMass/RT/Isotope/Library/Formula/Ion Ratio)

| | |
|---|---|
| **Retention Time:** 16.19 minutes | **Exp RT:** 16.20 minutes |
| **Precursor m/z :** 295.2390 | **Analyte Name:** |
| **Fit (%)** 98.4% **RFit (%)** 96.9% | 295.2390 / 16.20 |

**Collision Energy = 35 ± 15 eV**

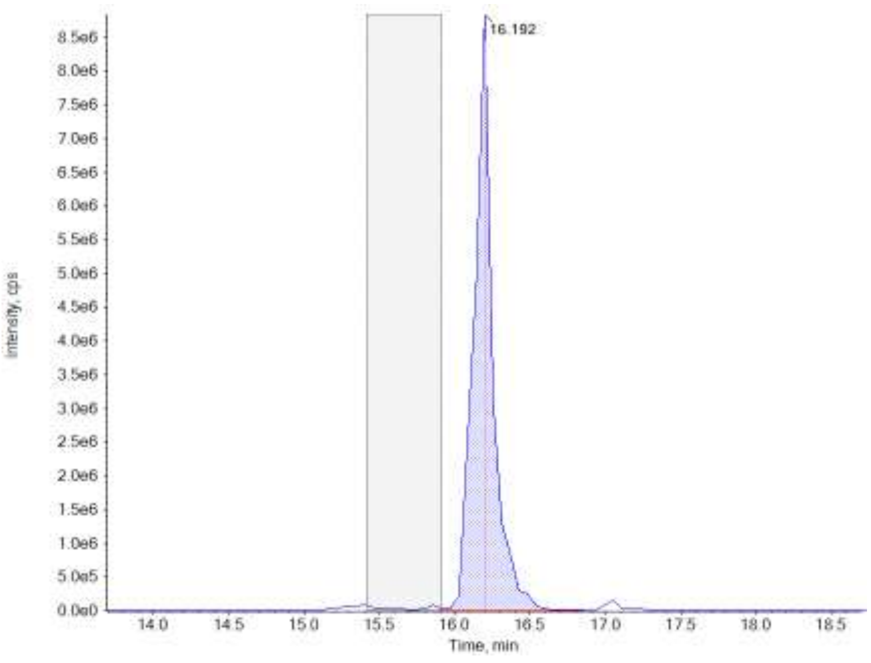


**Acquired / Library MSMS**

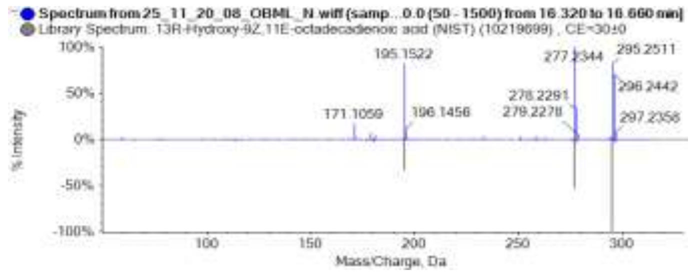


**Acquired / Theoretical MS**

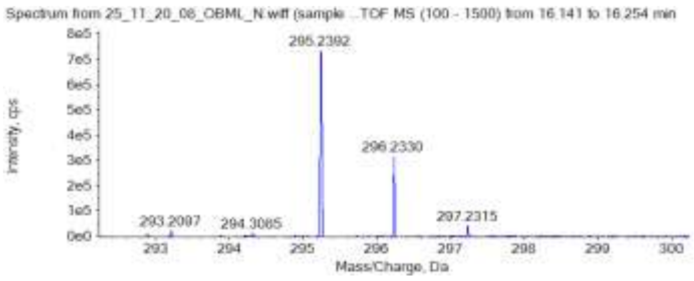

## 869.4814 / 16.42 [M+Na-2H]- (Mass/FragMass/RT/Isotope/Library/Formula/Ion Ratio)

| | |
|---|---|
| **Retention Time:** 16.43 minutes | **Exp RT:** 16.43 minutes |
| **Precursor m/z :** 869.4814 | **Analyte Name:** |
| **Fit (%)** 32.5% **RFit (%)** 100.0% | 869.4814 / 16.42 [M+Na-2H]- |

**Collision Energy = 35 ± 15 eV**

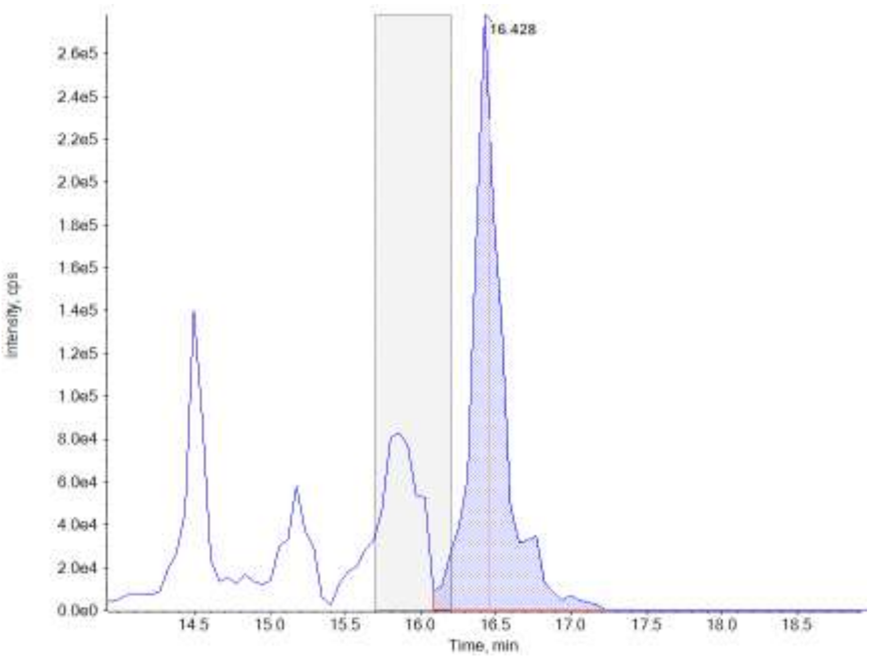


**Acquired / Library MSMS**

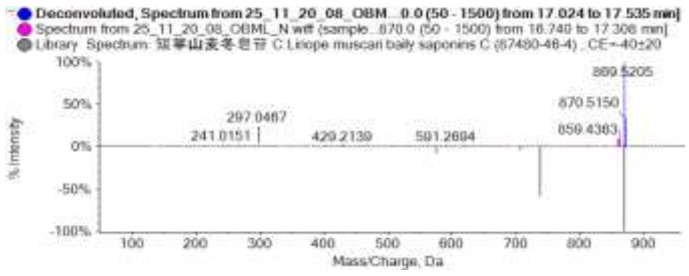


**Acquired / Theoretical MS**

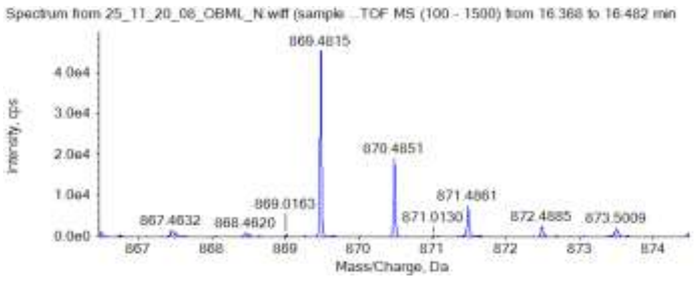

**337.2362 / 16.31** (Mass/FragMass/RT/Isotope/Library/Formula/Ion Ratio)

| | |
|---|---|
| **Retention Time:** 16.33 minutes | **Exp RT:** 16.31 minutes |
| **Precursor m/z :** 337.2362 | **Analyte Name:** |
| **Fit (%)** 93.9% **RFit (%)** 90.2% | 337.2362 / 16.31 |

**Collision Energy = 35 ± 15 eV**

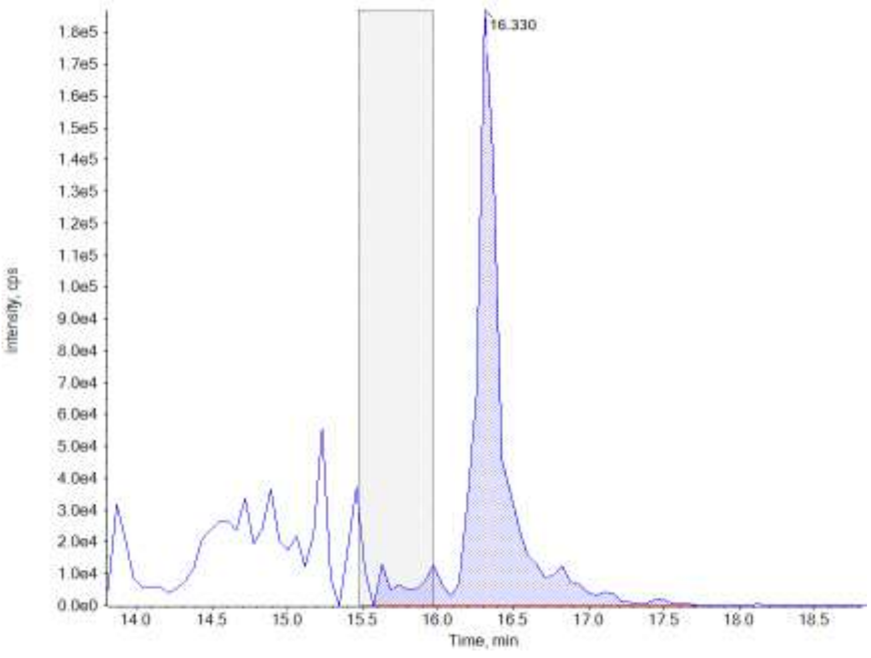


**Acquired / Library MSMS**

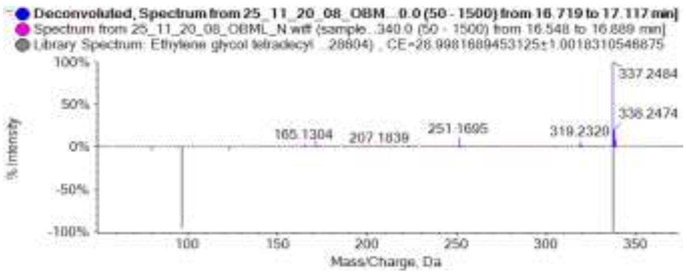


**Acquired / Theoretical MS**

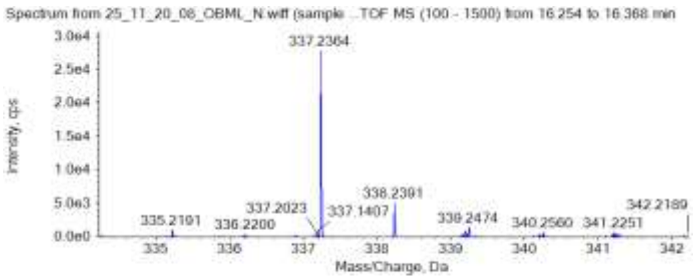

## 519.3654 / 16.31 (Mass/FragMass/RT/Isotope/Library/Formula/Ion Ratio)

| | |
|---|---|
| **Retention Time:** 16.27 minutes<br>**Precursor m/z :** 519.3654<br>**Fit (%)** 87.9% **RFit (%)** 85.5% | **Exp RT:** 16.31 minutes<br>**Analyte Name:**<br>519.3654 / 16.31 |

| | Collision Energy = 35 ± 15 eV |
|---|---|

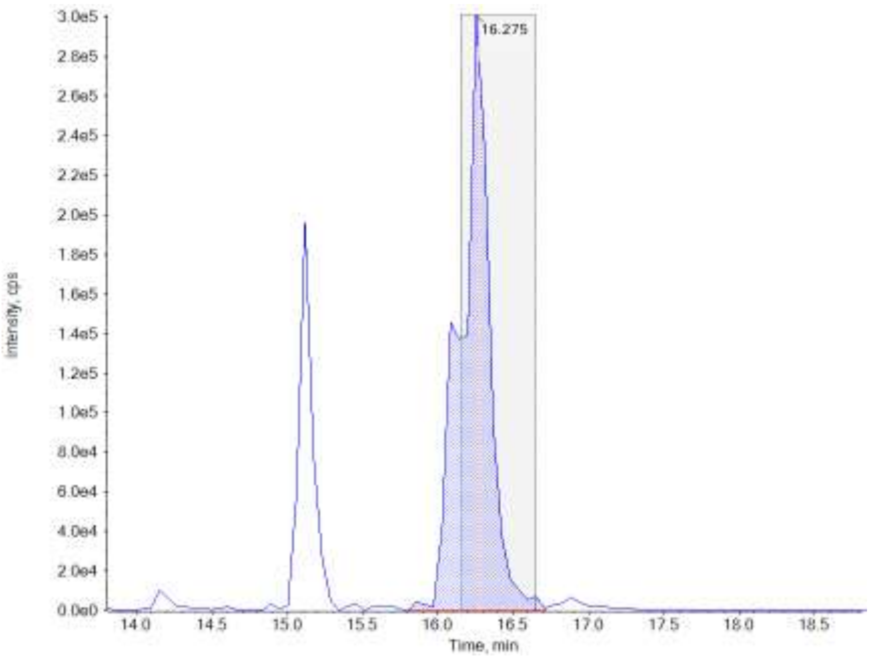


**Acquired / Library MSMS**

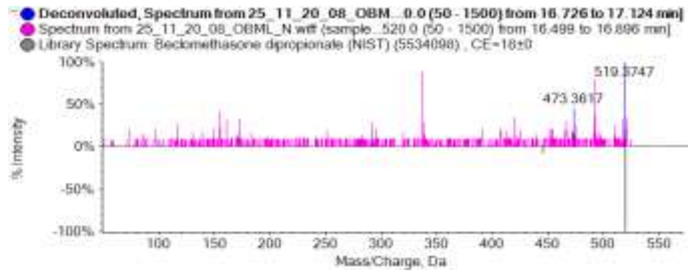


**Acquired / Theoretical MS**

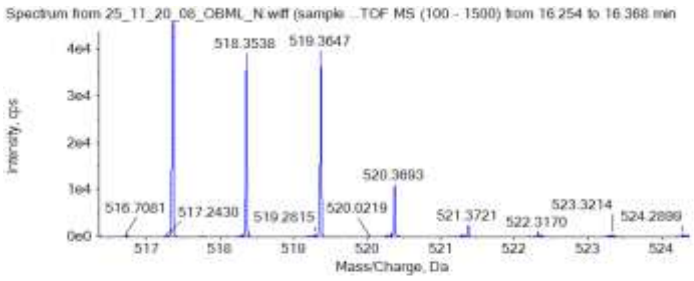

## 295.2261 / 16.37 (Mass/FragMass/RT/Isotope/Library/Formula/Ion Ratio)

**Retention Time:** 16.22 minutes
**Precursor m/z :** 295.2261
**Fit (%)** 98.3% **RFit (%)** 96.7%

**Exp RT:** 16.37 minutes
**Analyte Name:**
295.2261 / 16.37

**Collision Energy = 35 ± 15 eV**

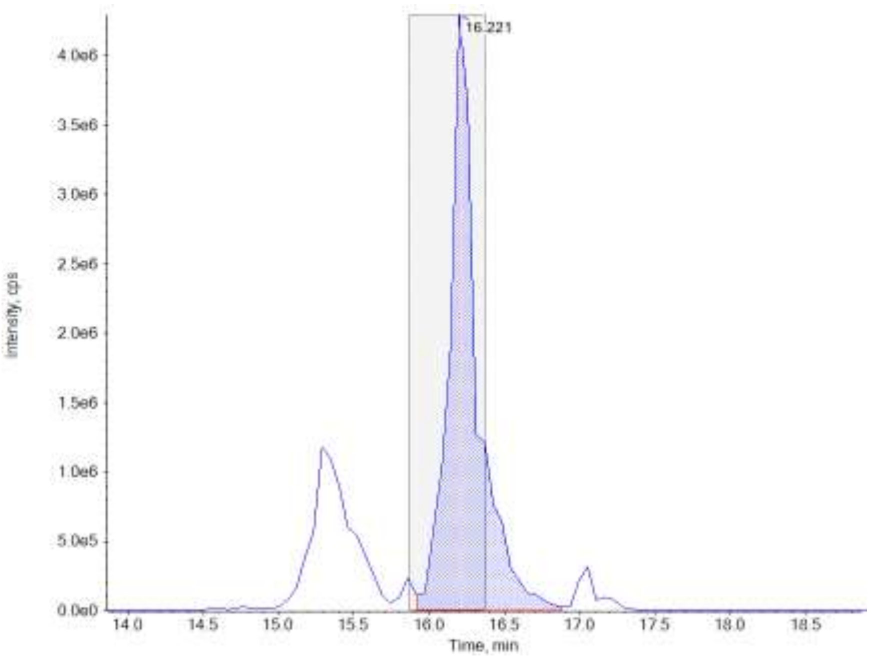


Acquired / Library MSMS

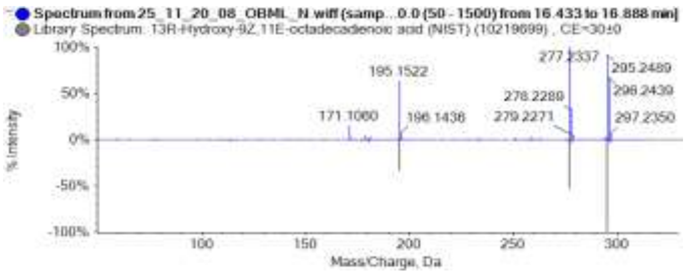


Acquired / Theoretical MS

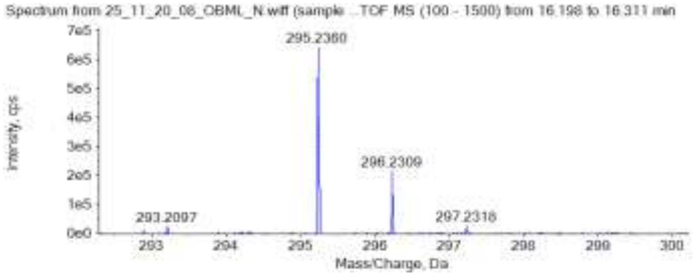

## 849.5047 / 16.37 (Mass/FragMass/RT/Isotope/Library/Formula/Ion Ratio)

**Retention Time:** 16.31 minutes
**Precursor m/z :** 849.5047
**Fit (%)** 64.2% **RFit (%)** 81.3%

**Exp RT:** 16.37 minutes
**Analyte Name:**
849.5047 / 16.37

**Collision Energy = 35 ± 15 eV**

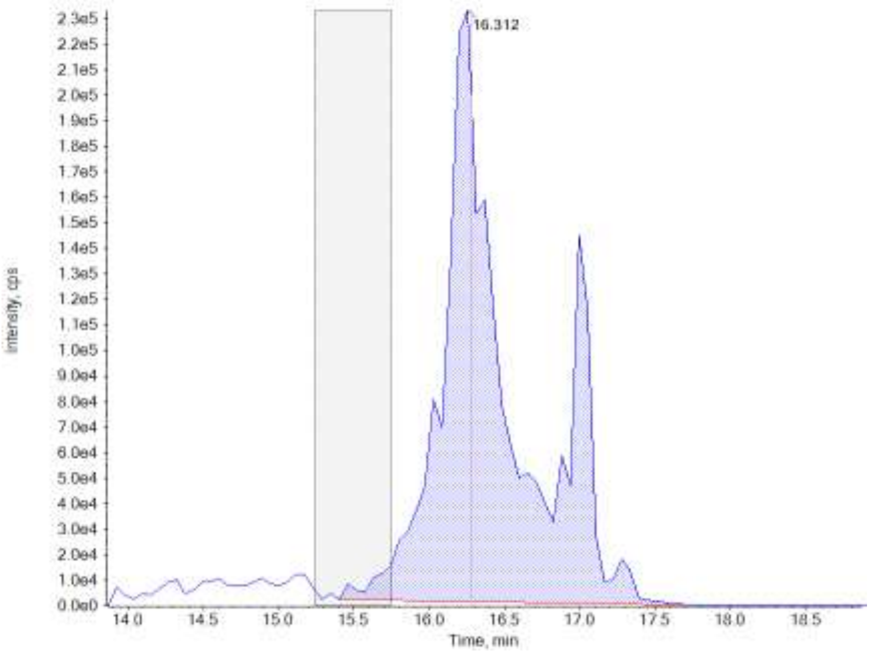


**Acquired / Library MSMS**

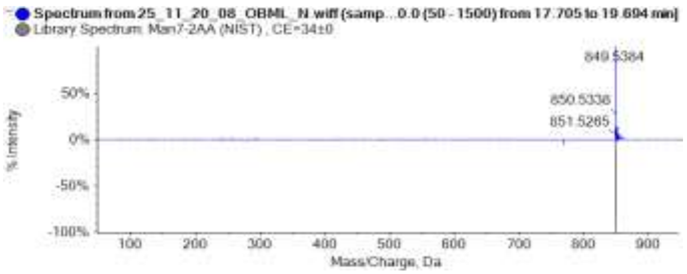


**Acquired / Theoretical MS**

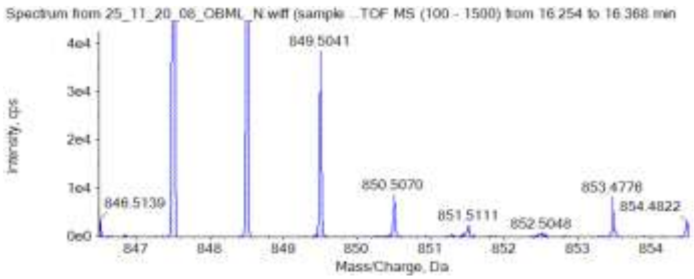

## 243.1947 / 16.54 (Mass/FragMass/RT/Isotope/Library/Formula/Ion Ratio)

**Retention Time:** 16.57 minutes
**Precursor m/z :** 243.1947
**Fit (%)** 37.6% **RFit (%)** 100.0%

**Exp RT:** 16.54 minutes
**Analyte Name:**
243.1947 / 16.54

**Collision Energy = 35 ± 15 eV**

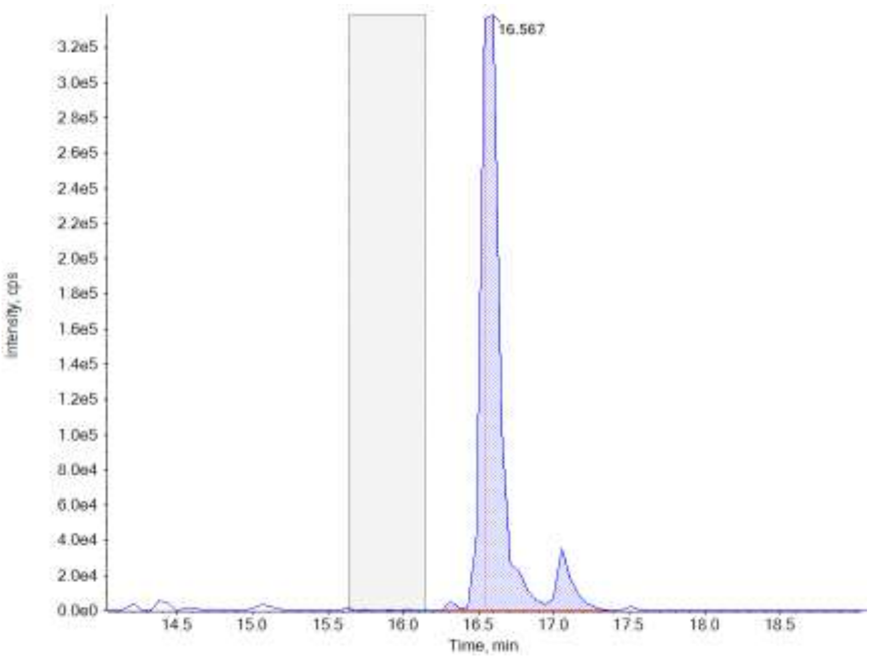


**Acquired / Library MSMS**

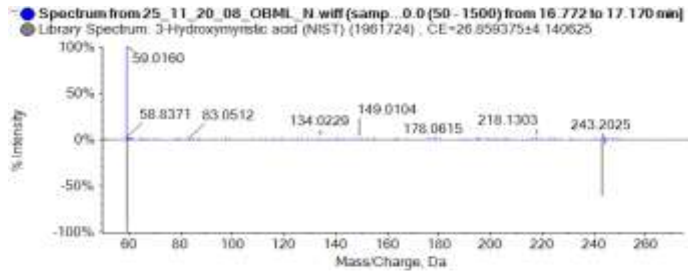


**Acquired / Theoretical MS**

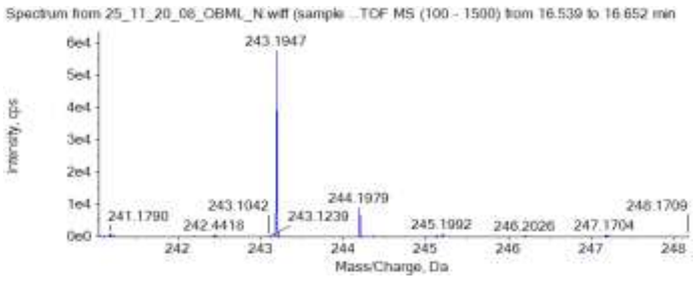

## 573.3767 / 16.54

(Mass/FragMass/RT/Isotope/Library/Formula/Ion Ratio)

| | |
|---|---|
| **Retention Time:** 16.53 minutes | **Exp RT:** 16.54 minutes |
| **Precursor m/z :** 573.3767 | **Analyte Name:** |
| **Fit (%)** 40.1% **RFit (%)** 100.0% | 573.3767 / 16.54 |

**Collision Energy = 35 ± 15 eV**

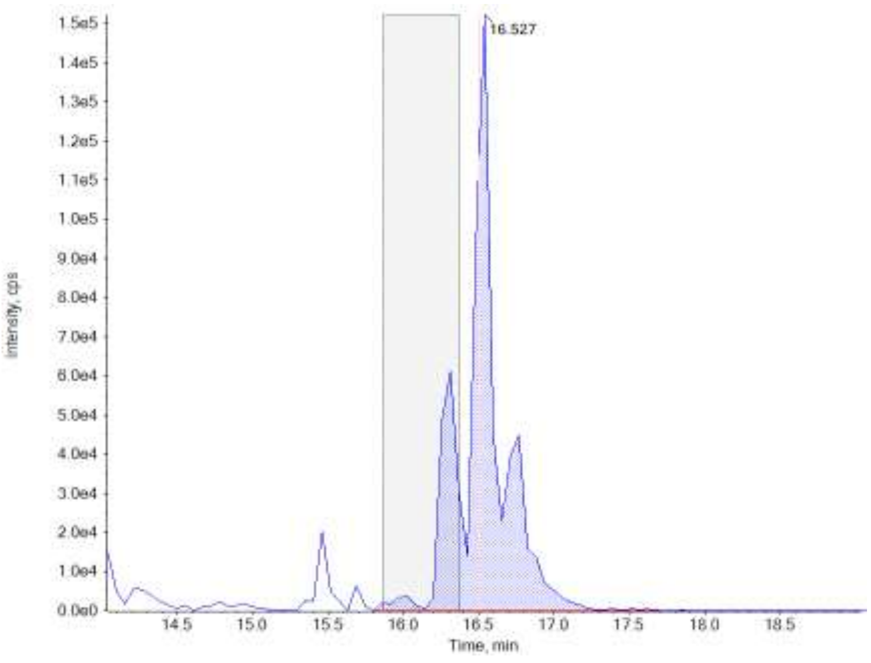


**Acquired / Library MSMS**

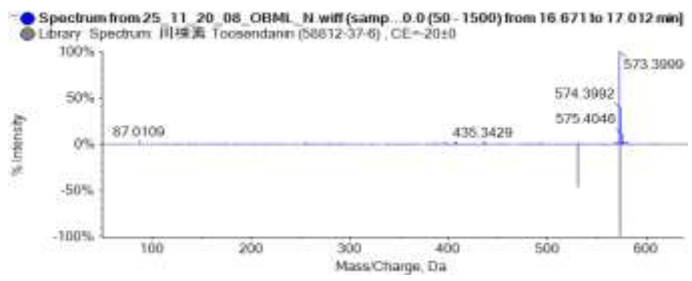


**Acquired / Theoretical MS**

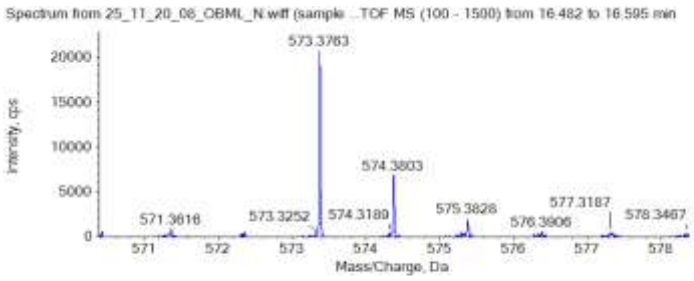

## 293.2205 / 16.65 (Mass/FragMass/RT/Isotope/Library/Formula/Ion Ratio)

| | |
|---|---|
| **Retention Time:** 16.66 minutes | **Exp RT:** 16.65 minutes |
| **Precursor m/z :** 293.2205 | **Analyte Name:** |
| **Fit (%)** 97.1% **RFit (%)** 97.9% | 293.2205 / 16.65 |

**Collision Energy = 35 ± 15 eV**

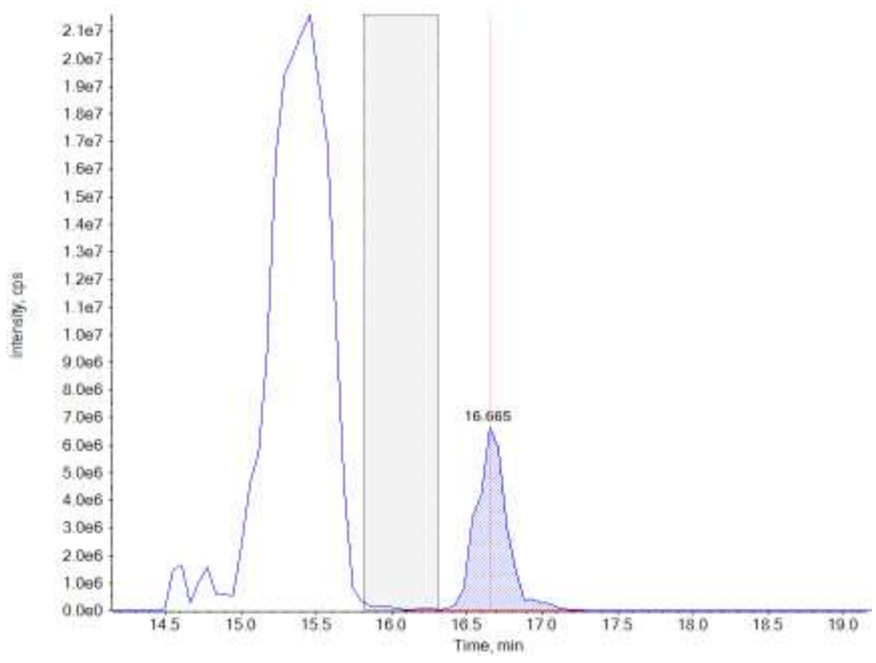


**Acquired / Library MSMS**

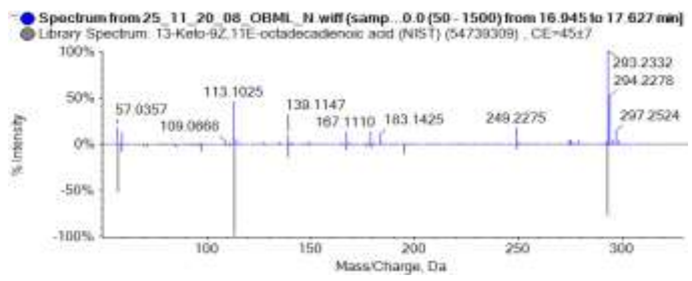


**Acquired / Theoretical MS**

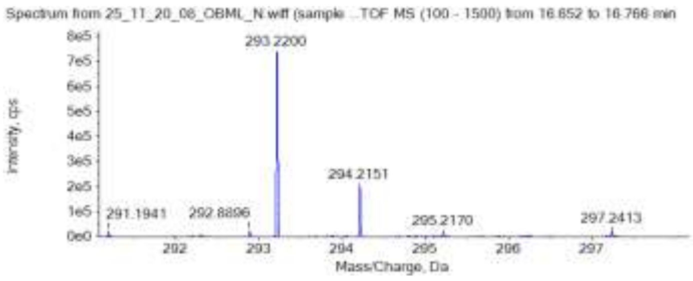

**585.3782 / 16.71** (Mass/FragMass/RT/Isotope/Library/Formula/Ion Ratio)

**Retention Time:** 16.71 minutes
**Precursor m/z :** 585.3782
**Fit (%)** 72.6% **RFit (%)** 95.6%

**Exp RT:** 16.71 minutes
**Analyte Name:**
585.3782 / 16.71

**Collision Energy = 35 ± 15 eV**

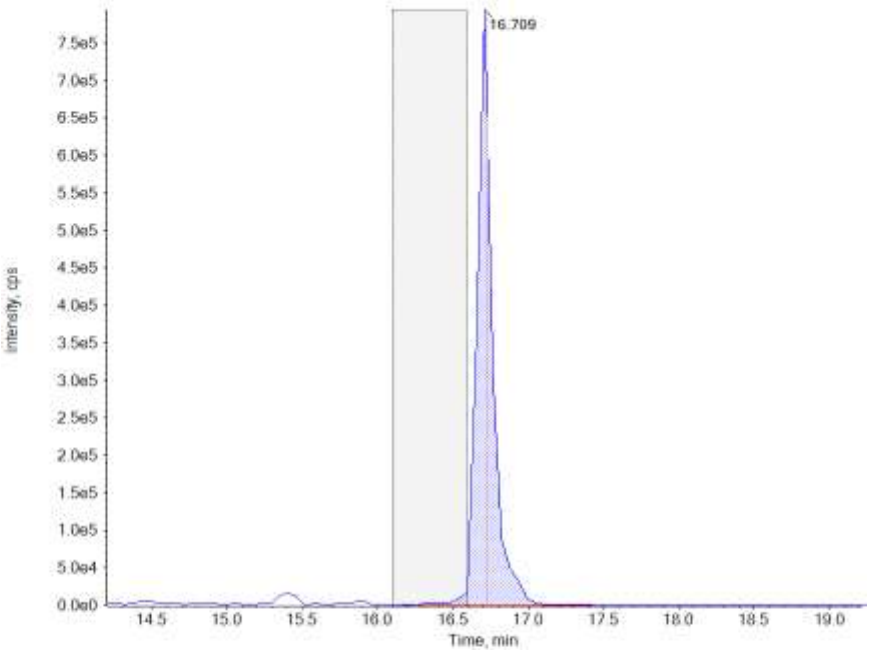


**Acquired / Library MSMS**

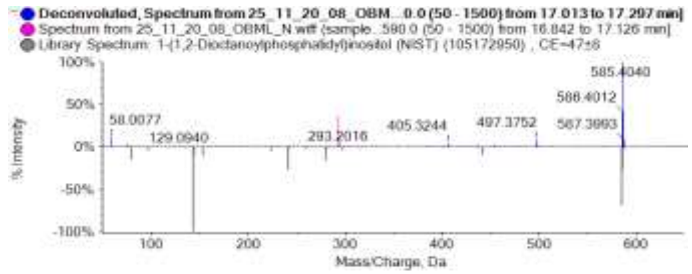


**Acquired / Theoretical MS**

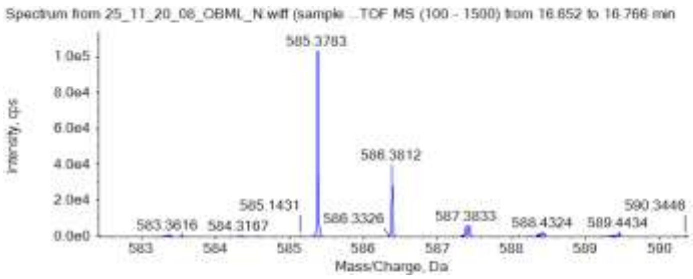

**321.2448 / 16.88** (Mass/FragMass/RT/Isotope/Library/Formula/Ion Ratio)

| | |
|---|---|
| **Retention Time:** 16.86 minutes | **Exp RT:** 16.88 minutes |
| **Precursor m/z :** 321.2448 | **Analyte Name:** |
| **Fit (%)** 49.4% **RFit (%)** 96.3% | 321.2448 / 16.88 |

**Collision Energy = 35 ± 15 eV**

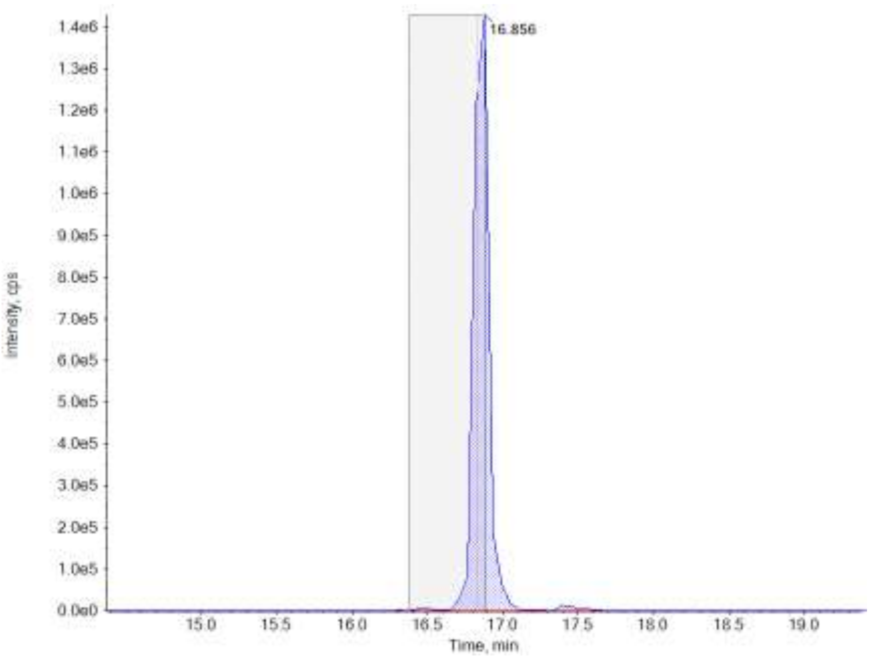


**Acquired / Library MSMS**

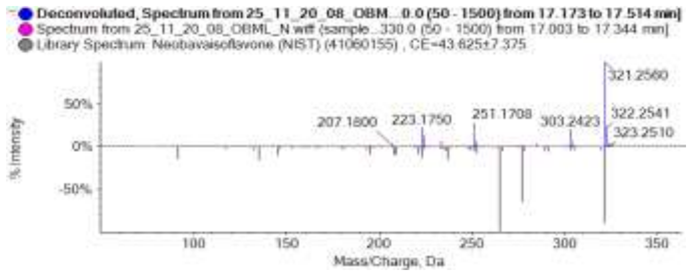


**Acquired / Theoretical MS**

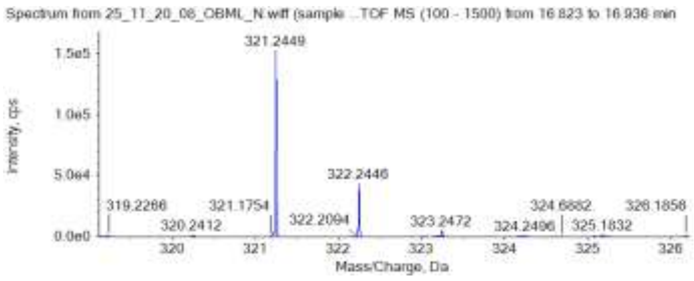

## 295.2262 / 17.05

(Mass/FragMass/RT/Isotope/Library/Formula/Ion Ratio)

**Retention Time:** 17.03 minutes
**Precursor m/z :** 295.2262
**Fit (%)** 77.5% **RFit (%)** 100.0%

**Exp RT:** 17.05 minutes
**Analyte Name:**
295.2262 / 17.05

**Collision Energy = 35 ± 15 eV**

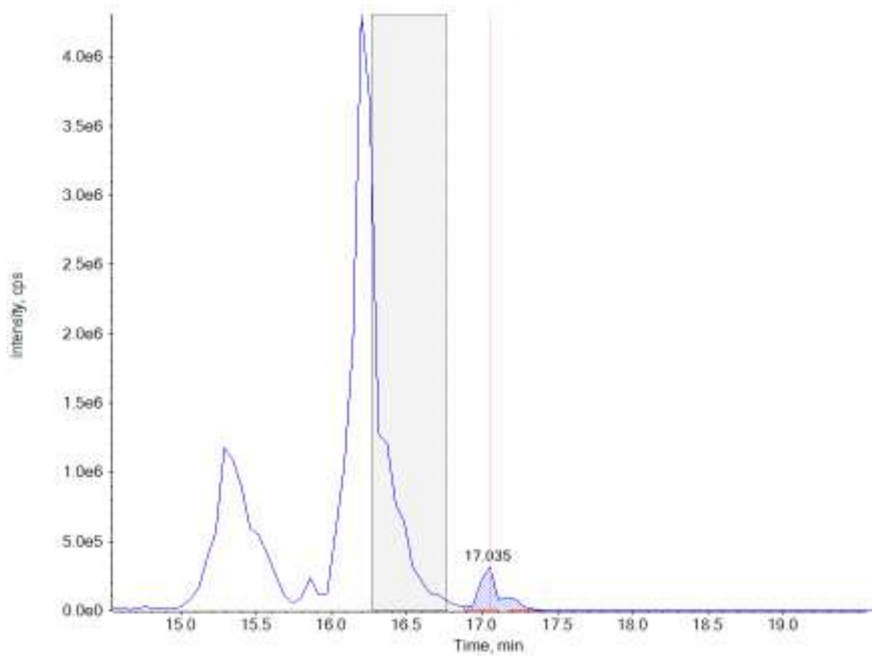


**Acquired / Library MSMS**

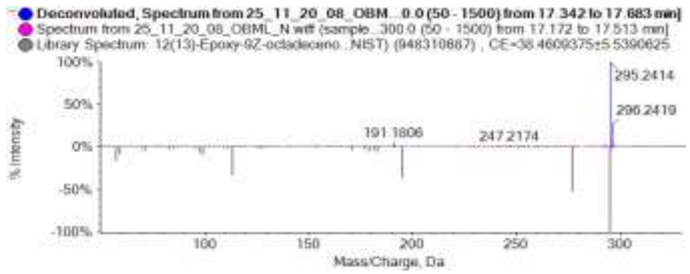


**Acquired / Theoretical MS**

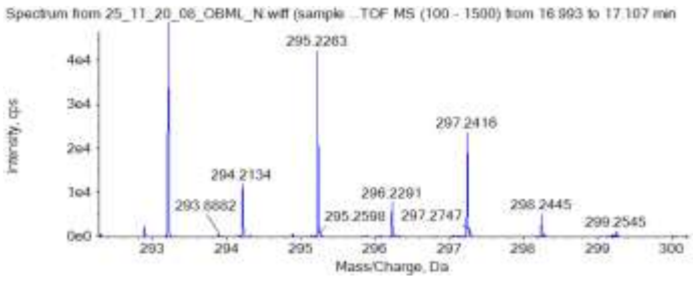

**362.2682 / 17.05** (Mass/FragMass/RT/Isotope/Library/Formula/Ion Ratio)

**Retention Time:** 17.03 minutes **Exp RT:** 17.05 minutes
**Precursor m/z :** 362.2682 **Analyte Name:**
**Fit (%)** 75.8% **RFit (%)** 84.9% 362.2682 / 17.05

**Collision Energy = 35 ± 15 eV**

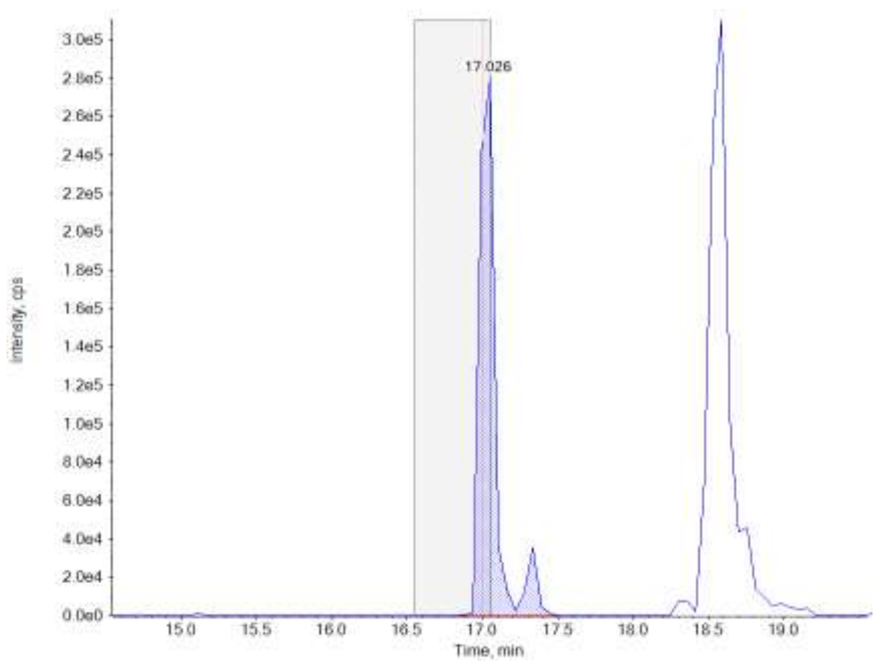


**Acquired / Library MSMS**

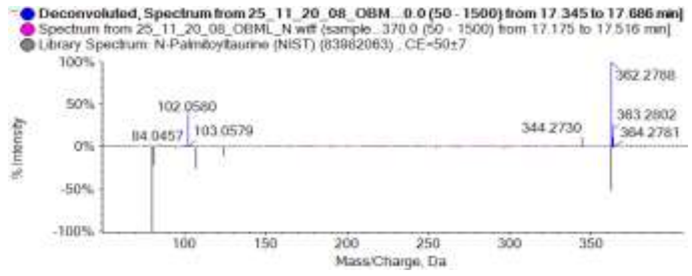


**Acquired / Theoretical MS**

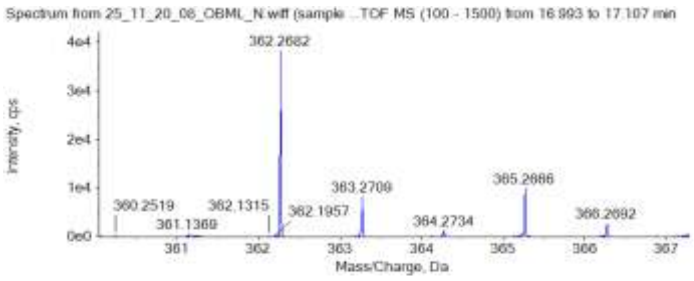

## 335.2210 / 17.16 [M+AcO-H]- (Mass/FragMass/RT/Isotope/Library/Formula/Ion Ratio)

**Retention Time:** 17.15 minutes
**Precursor m/z :** 335.2210
**Fit (%)** 84.7% **RFit (%)** 100.0%

**Exp RT:** 17.16 minutes
**Analyte Name:**
335.2210 / 17.16 [M+AcO-H]-

**Collision Energy = 35 ± 15 eV**

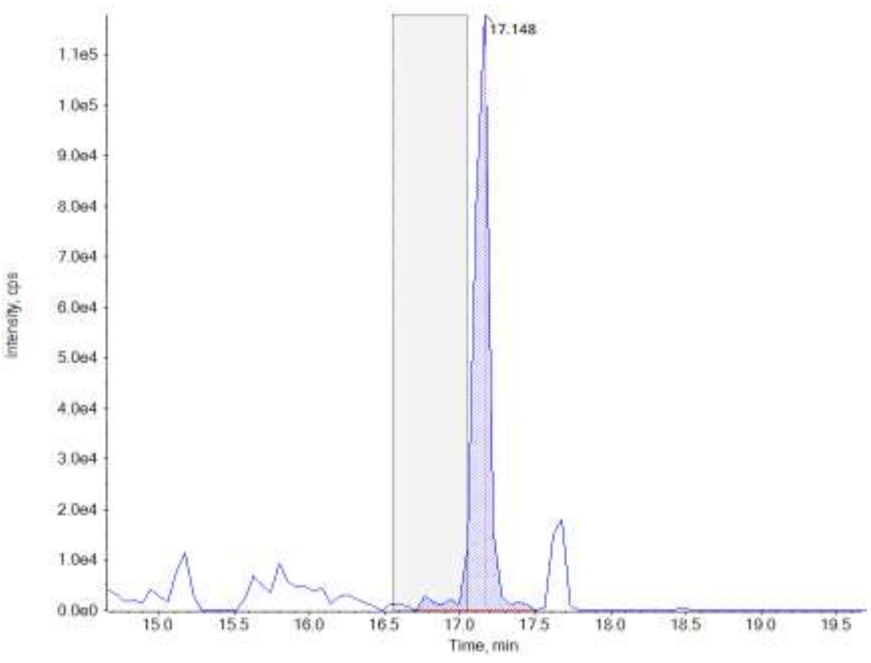

**Acquired / Library MSMS**

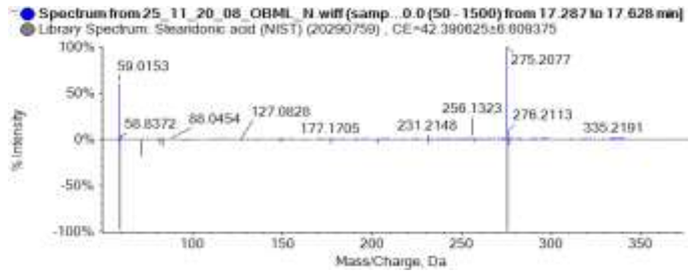

**Acquired / Theoretical MS**

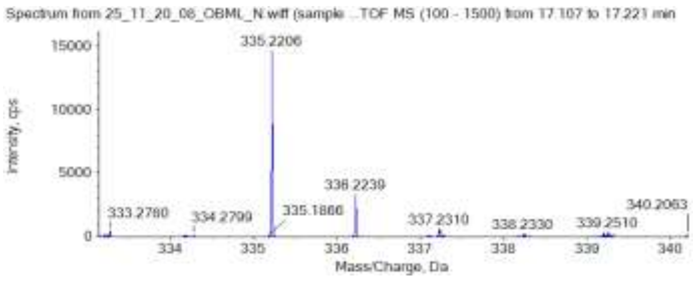

## 249.1858 / 17.16 (Mass/FragMass/RT/Isotope/Library/Formula/Ion Ratio)

| | |
|---|---|
| **Retention Time:** 17.19 minutes | **Exp RT:** 17.16 minutes |
| **Precursor m/z :** 249.1858 | **Analyte Name:** |
| **Fit (%)** 45.4% **RFit (%)** 95.2% | 249.1858 / 17.16 |

**Collision Energy = 35 ± 15 eV**

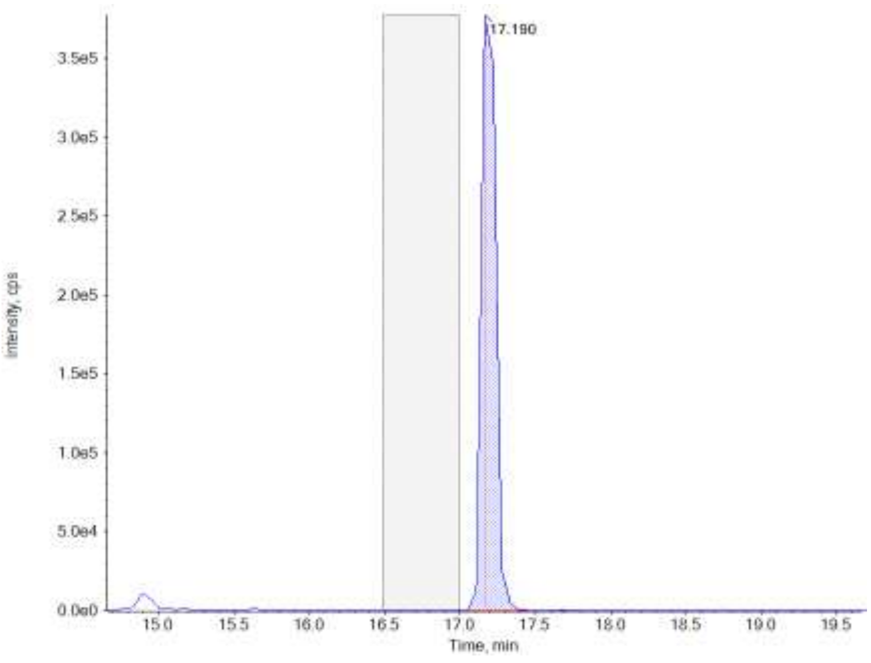


**Acquired / Library MSMS**

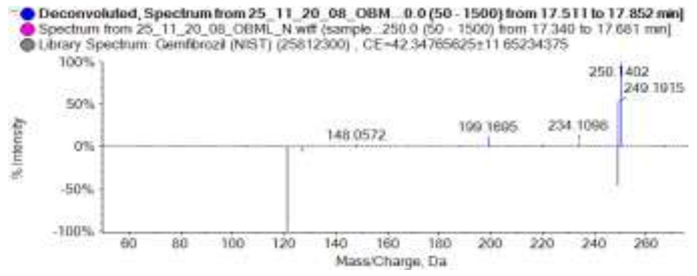


**Acquired / Theoretical MS**

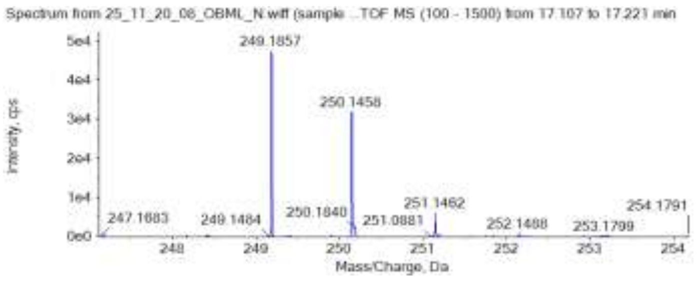

## 289.1808 / 17.28 (Mass/FragMass/RT/Isotope/Library/Formula/Ion Ratio)

| | |
|---|---|
| **Retention Time:** 17.25 minutes | **Exp RT:** 17.28 minutes |
| **Precursor m/z :** 289.1808 | **Analyte Name:** |
| **Fit (%)** 84.9% **RFit (%)** 100.0% | 289.1808 / 17.28 |

**Collision Energy = 35 ± 15 eV**

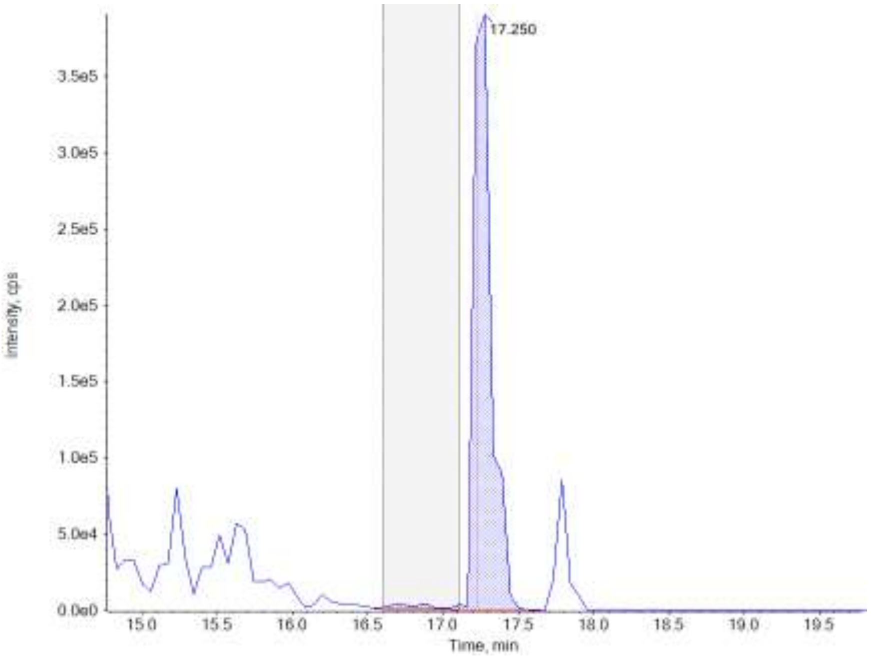


**Acquired / Library MSMS**

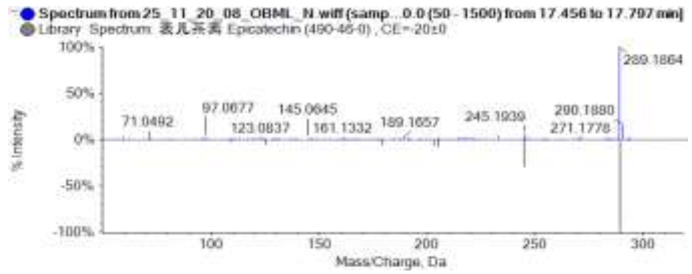


**Acquired / Theoretical MS**

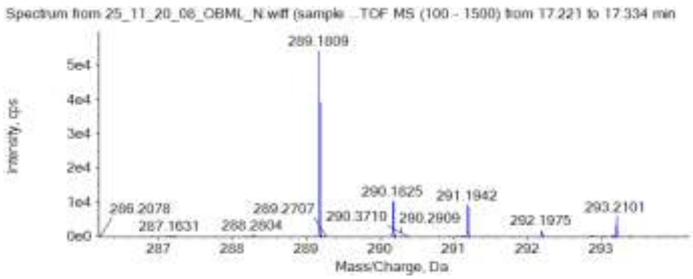

## 365.2725 / 17.28 (Mass/FragMass/RT/Isotope/Library/Formula/Ion Ratio)

| | |
|---|---|
| **Retention Time:** 17.28 minutes | **Exp RT:** 17.28 minutes |
| **Precursor m/z :** 365.2726 | **Analyte Name:** |
| **Fit (%)** 100.0% **RFit (%)** 91.7% | 365.2725 / 17.28 |

**Collision Energy = 35 ± 15 eV**

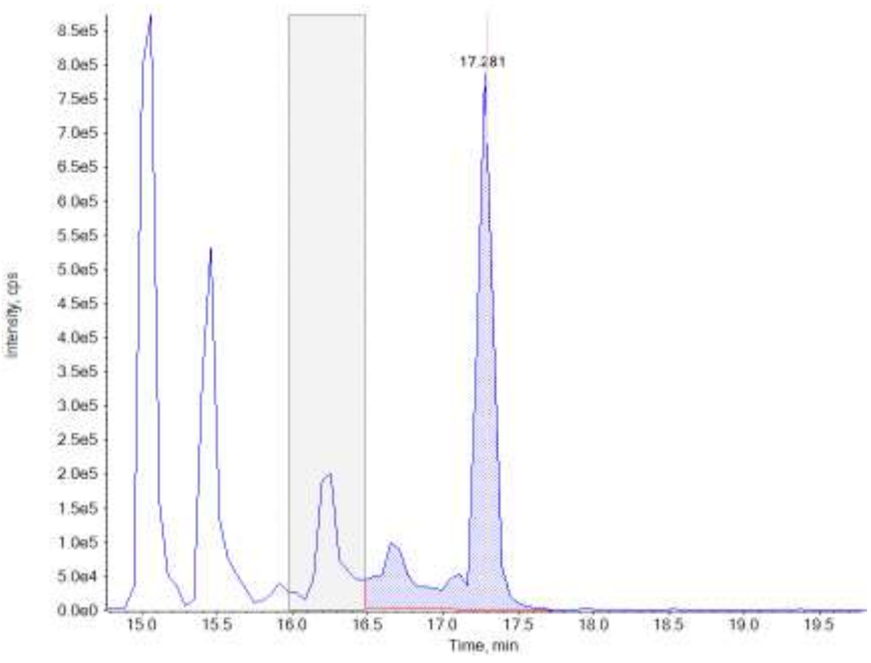


**Acquired / Library MSMS**

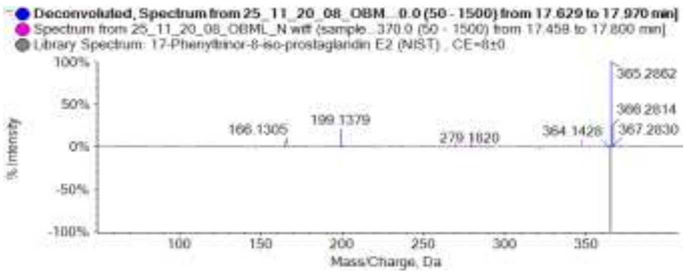


**Acquired / Theoretical MS**

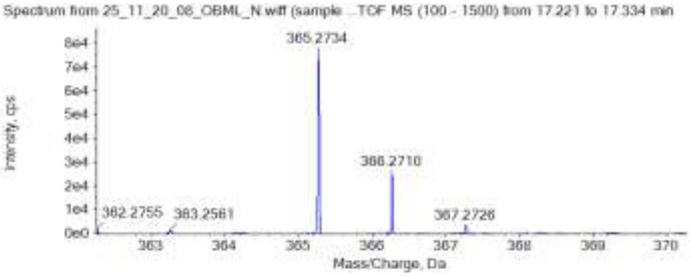

## 787.4677 / 17.28 (Mass/FragMass/RT/Isotope/Library/Formula/Ion Ratio)

| | |
|---|---|
| **Retention Time:** 17.25 minutes | **Exp RT:** 17.28 minutes |
| **Precursor m/z :** 787.4677 | **Analyte Name:** |
| **Fit (%)** 32.6% **RFit (%)** 98.6% | 787.4677 / 17.28 |

**Collision Energy = 35 ± 15 eV**

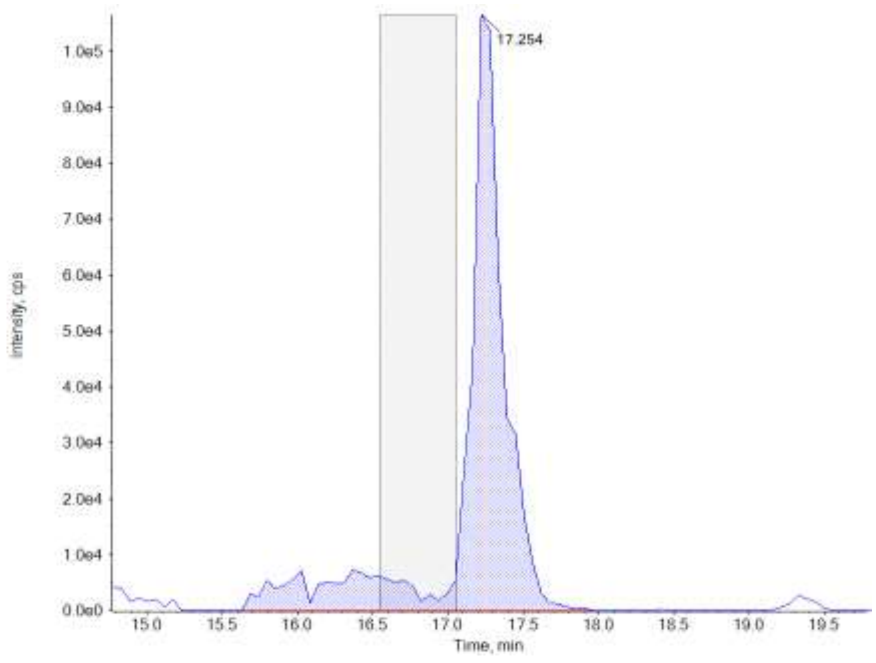


**Acquired / Library MSMS**

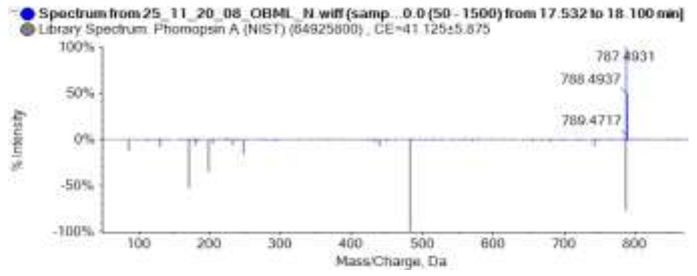


**Acquired / Theoretical MS**

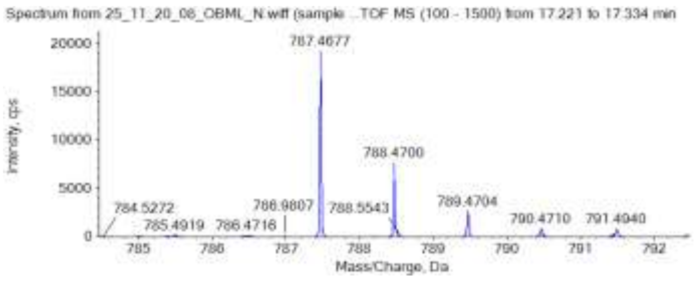

## 269.2462 / 17.33 (Mass/FragMass/RT/Isotope/Library/Formula/Ion Ratio)

| | |
|---|---|
| **Retention Time:** 17.34 minutes | **Exp RT:** 17.33 minutes |
| **Precursor m/z :** 269.2462 | **Analyte Name:** |
| **Fit (%)** 98.7% **RFit (%)** 100.0% | 269.2462 / 17.33 |

**Collision Energy = 35 ± 15 eV**

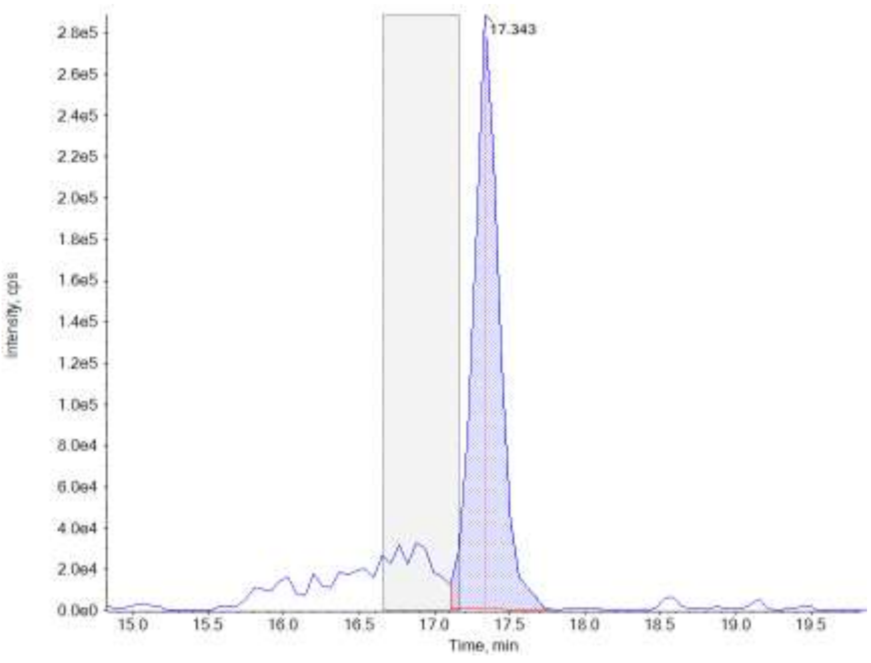


**Acquired / Library MSMS**

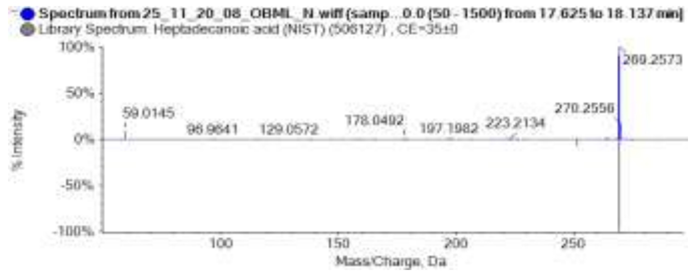


**Acquired / Theoretical MS**

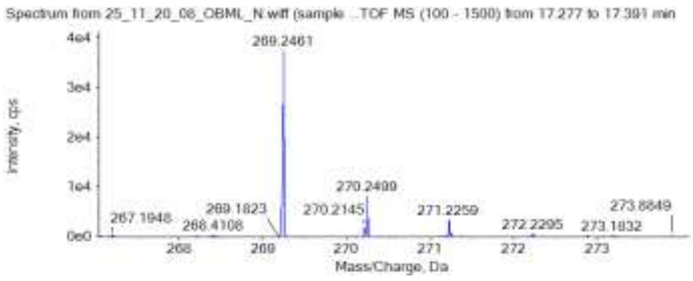

## 813.4836 / 17.33 (Mass/FragMass/RT/Isotope/Library/Formula/Ion Ratio)

**Retention Time:** 17.35 minutes
**Precursor m/z :** 813.4836
**Fit (%)** 21.7% **RFit (%)** 100.0%

**Exp RT:** 17.33 minutes
**Analyte Name:**
813.4836 / 17.33

**Collision Energy = 35 ± 15 eV**

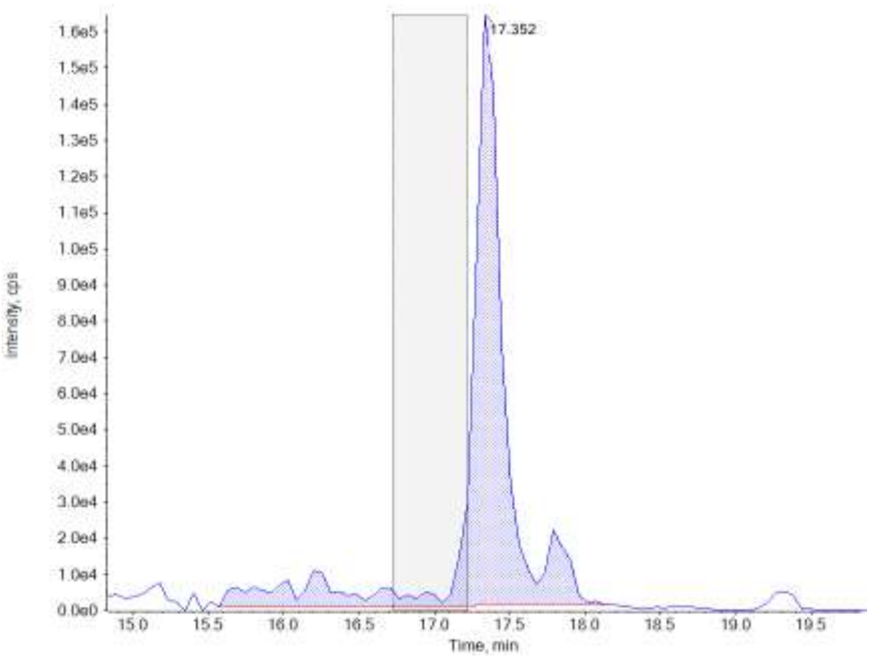


**Acquired / Library MSMS**

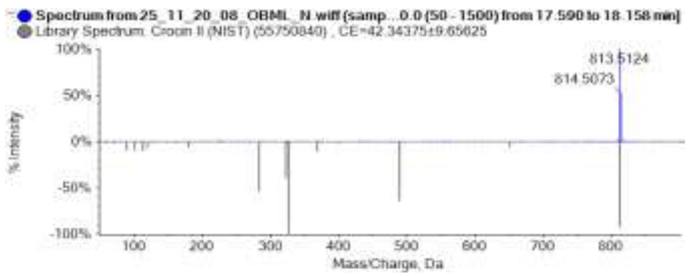


**Acquired / Theoretical MS**

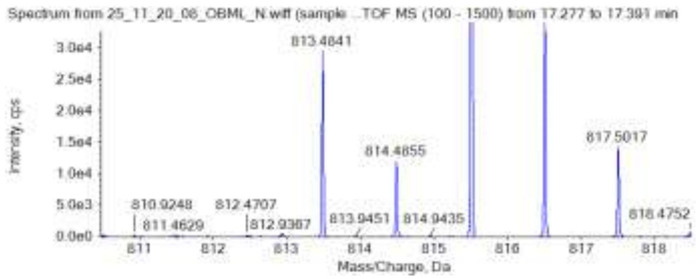

## 277.2156 / 17.45 (Mass/FragMass/RT/Isotope/Library/Formula/Ion Ratio)

**Retention Time:** 17.88 minutes
**Precursor m/z :** 277.2156
**Fit (%)** 92.3% **RFit (%)** 100.0%

**Exp RT:** 17.45 minutes
**Analyte Name:**
277.2156 / 17.45

**Collision Energy = 35 ± 15 eV**

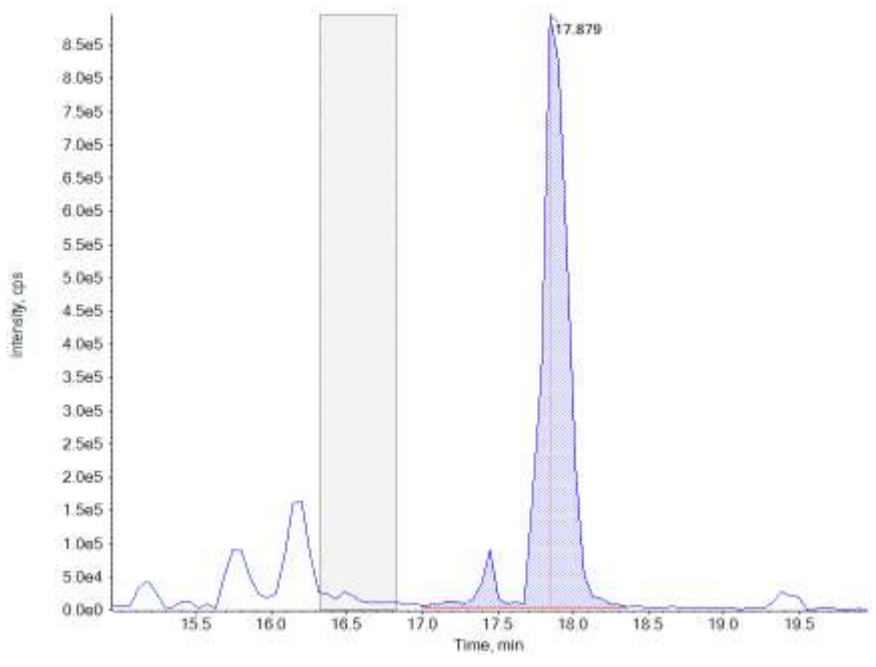


**Acquired / Library MSMS**

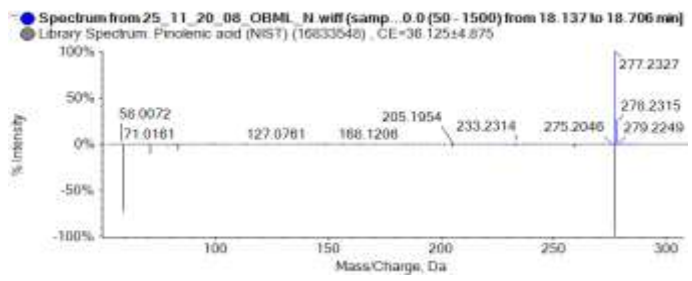


**Acquired / Theoretical MS**

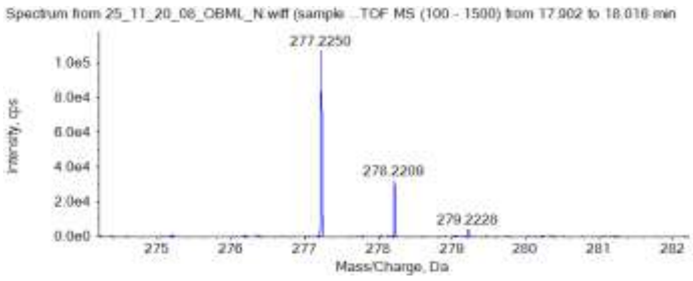

**283.2638 / 17.45** (Mass/FragMass/RT/Isotope/Library/Formula/Ion Ratio)

**Retention Time:** 17.45 minutes
**Precursor m/z :** 283.2638
**Fit (%)** 87.6% **RFit (%)** 95.0%

**Exp RT:** 17.45 minutes
**Analyte Name:**
283.2638 / 17.45

**Collision Energy = 35 ± 15 eV**

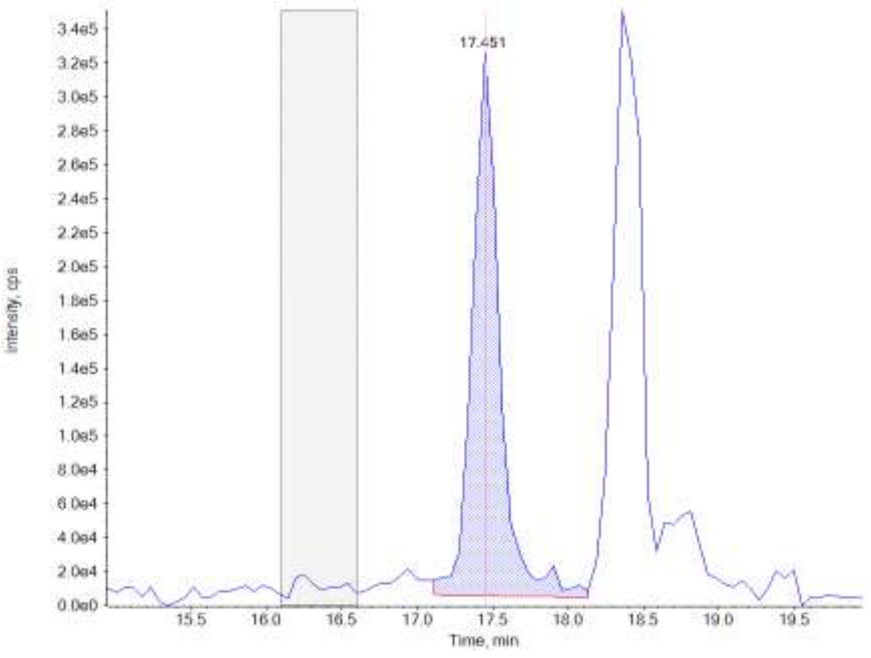


**Acquired / Library MSMS**

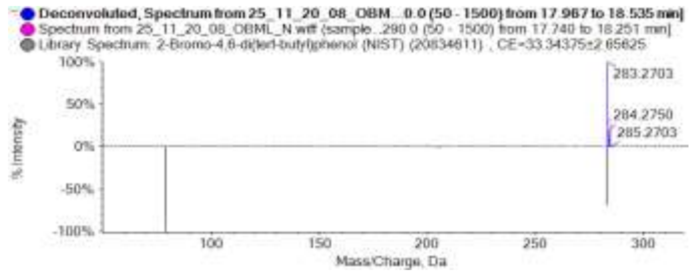


**Acquired / Theoretical MS**

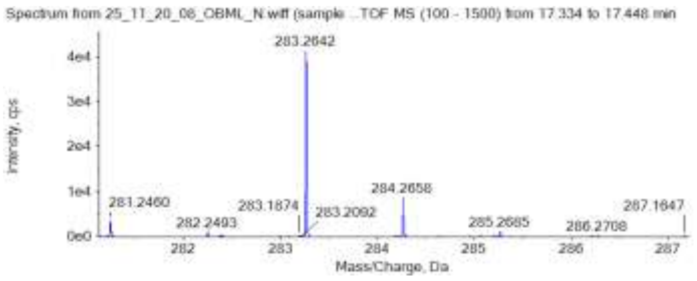

## 263.2003 / 17.50 (Mass/FragMass/RT/Isotope/Library/Formula/Ion Ratio)

| | |
|---|---|
| **Retention Time:** 17.50 minutes | **Exp RT:** 17.51 minutes |
| **Precursor m/z :** 263.2003 | **Analyte Name:** |
| **Fit (%)** 97.1% **RFit (%)** 96.9% | 263.2003 / 17.50 |

**Collision Energy = 35 ± 15 eV**

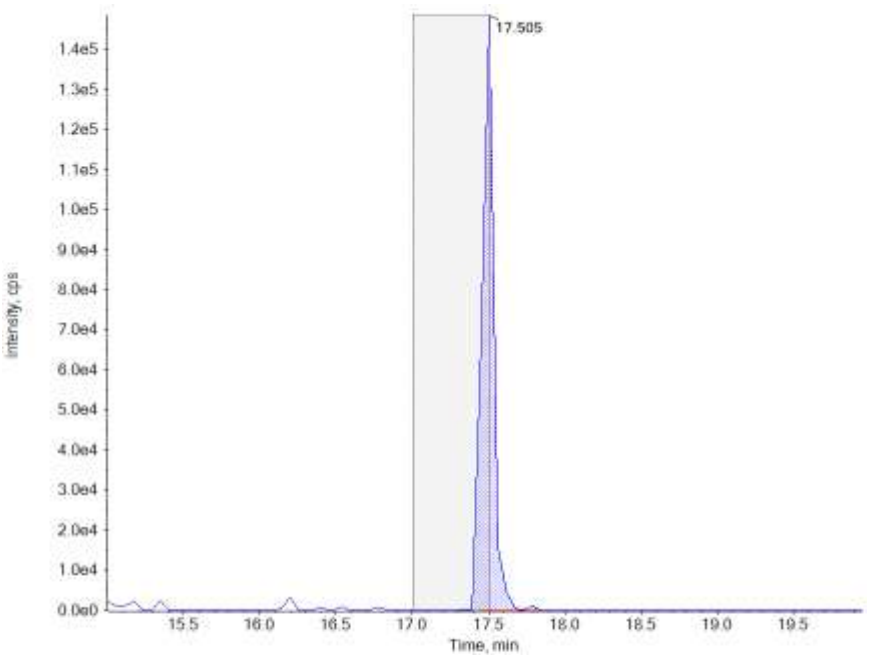


**Acquired / Library MSMS**

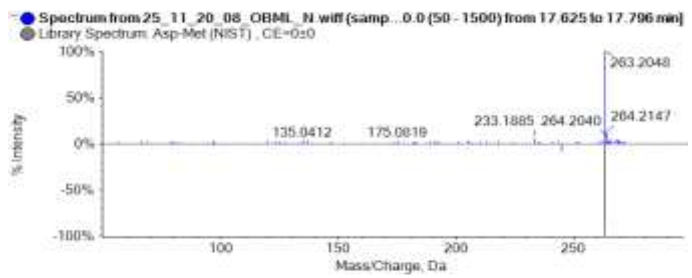


**Acquired / Theoretical MS**

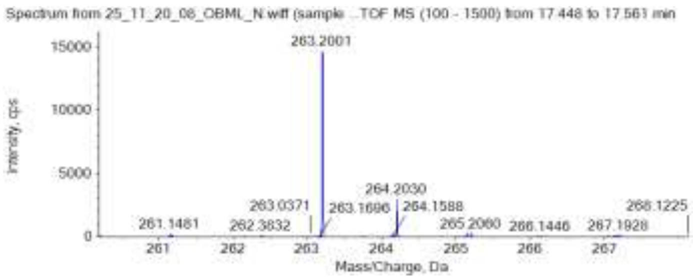

## 831.5072 / 17.50 (Mass/FragMass/RT/Isotope/Library/Formula/Ion Ratio)

| | |
|---|---|
| **Retention Time:** 17.54 minutes | **Exp RT:** 17.51 minutes |
| **Precursor m/z :** 831.5072 | **Analyte Name:** |
| **Fit (%)** 92.0% **RFit (%)** 92.0% | 831.5072 / 17.50 |

**Collision Energy = 35 ± 15 eV**

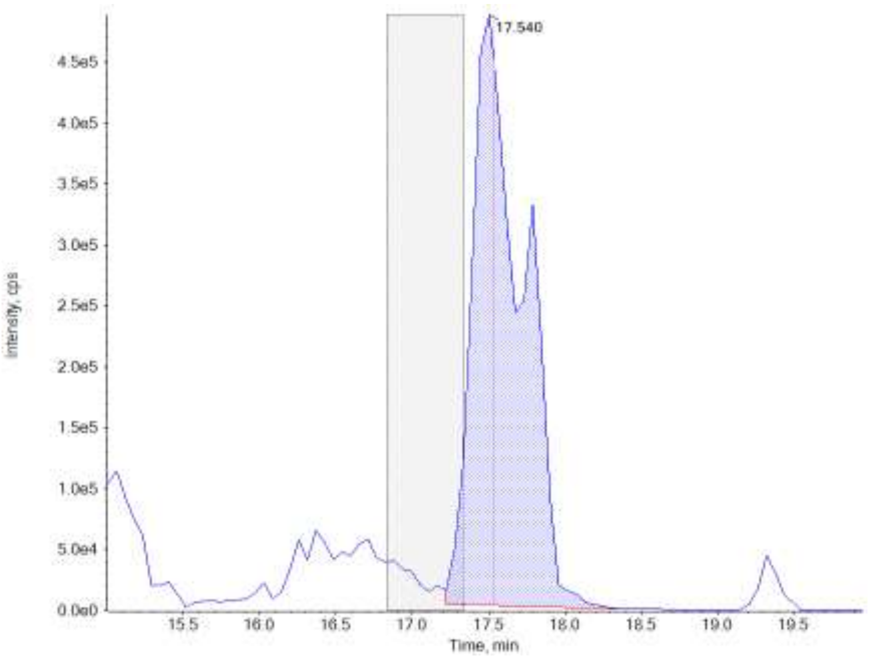


**Acquired / Library MSMS**

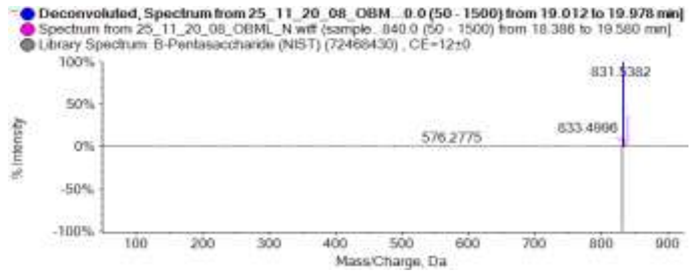


**Acquired / Theoretical MS**

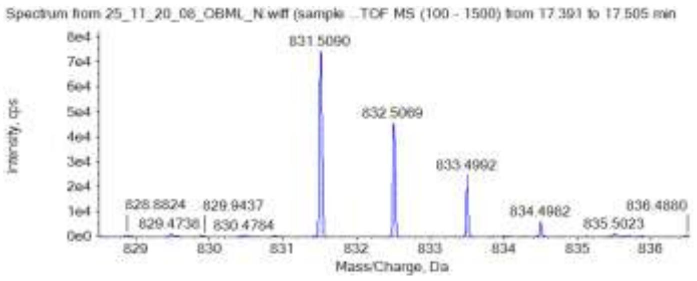

**251.2000 / 17.62** (Mass/FragMass/RT/Isotope/Library/Formula/Ion Ratio)

| | |
|---|---|
| **Retention Time:** 17.61 minutes<br>**Precursor m/z :** 251.2000<br>**Fit (%)** 91.3% **RFit (%)** 93.1% | **Exp RT:** 17.62 minutes<br>**Analyte Name:**<br>251.2000 / 17.62 |

**Collision Energy = 35 ± 15 eV**

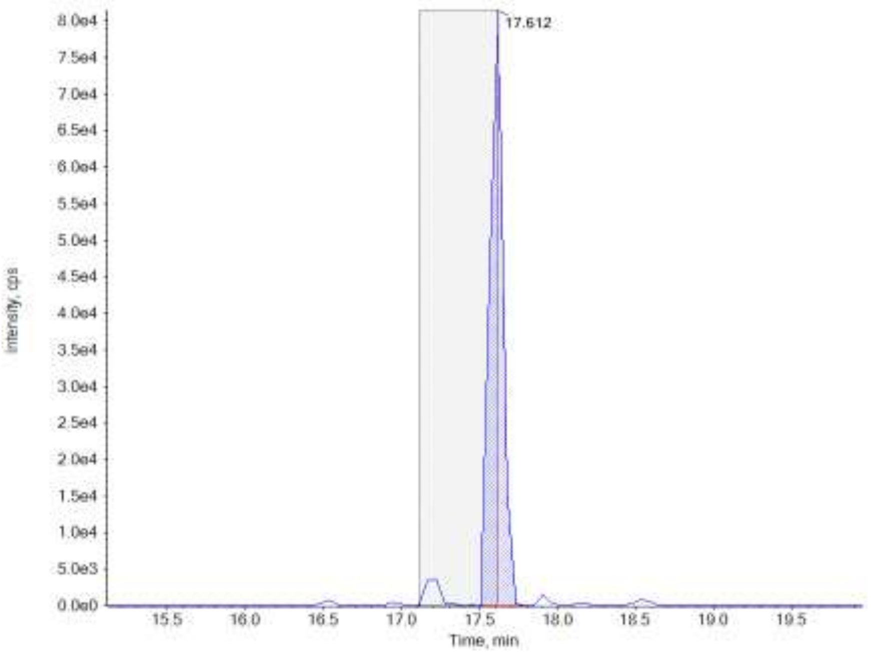

**Acquired / Library MSMS**

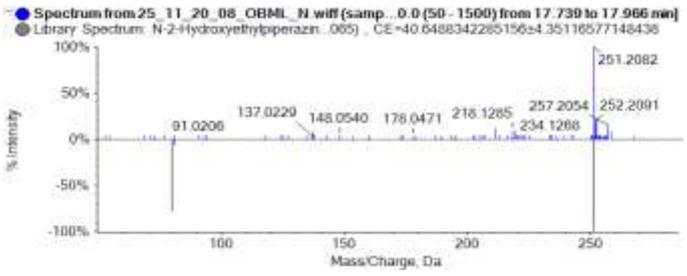

**Acquired / Theoretical MS**

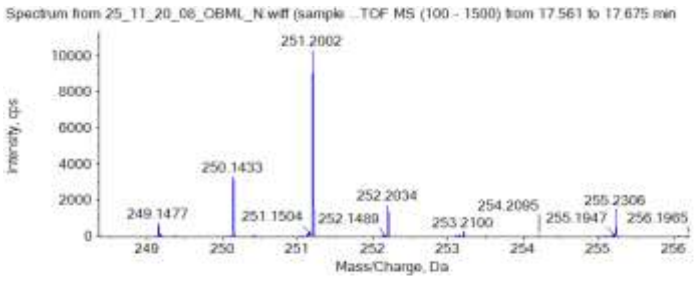

## 307.1899 / 17.79 [M-H]- (Mass/FragMass/RT/Isotope/Library/Formula/Ion Ratio)

**Retention Time:** 17.79 minutes
**Precursor m/z :** 307.1899
**Fit (%)** 50.4% **RFit (%)** 96.1%

**Exp RT:** 17.79 minutes
**Analyte Name:**
307.1899 / 17.79 [M-H]-

**Collision Energy = 35 ± 15 eV**

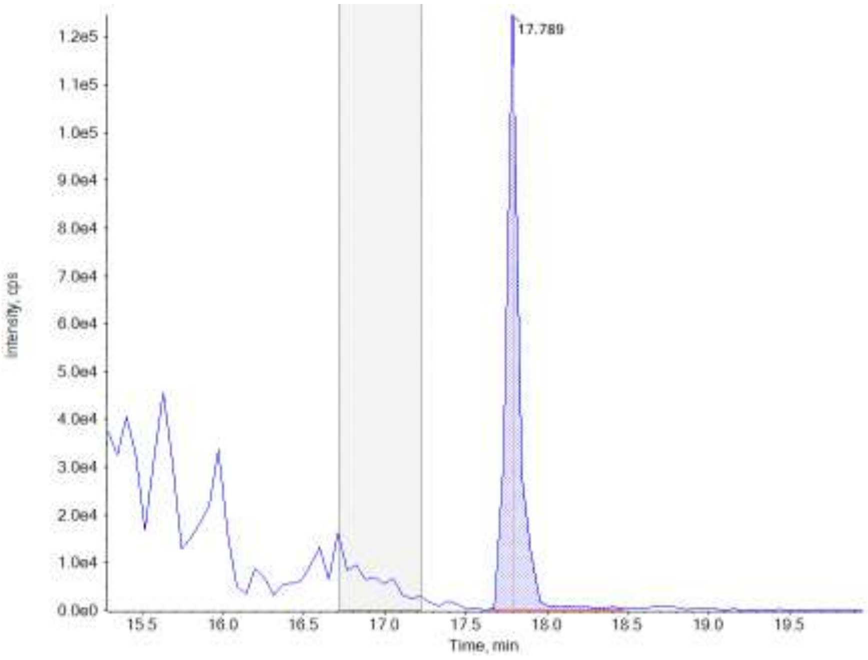


**Acquired / Library MSMS**

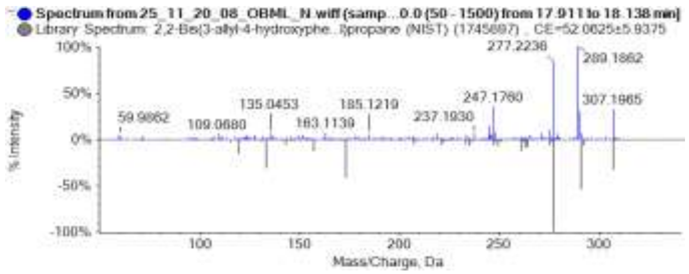


**Acquired / Theoretical MS**

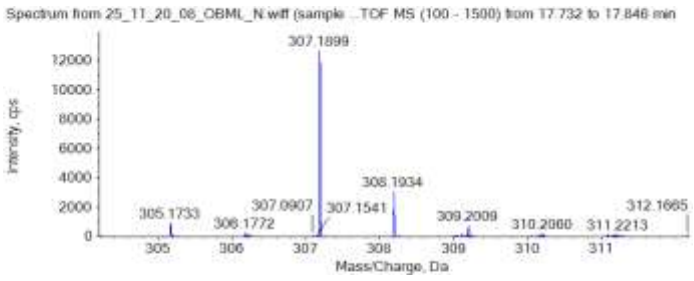

## 277.2279 / 17.85 (Mass/FragMass/RT/Isotope/Library/Formula/Ion Ratio)

**Retention Time:** 17.87 minutes
**Precursor m/z :** 277.2279
**Fit (%)** 92.3% **RFit (%)** 100.0%

**Exp RT:** 17.85 minutes
**Analyte Name:**
277.2279 / 17.85

**Collision Energy = 35 ± 15 eV**

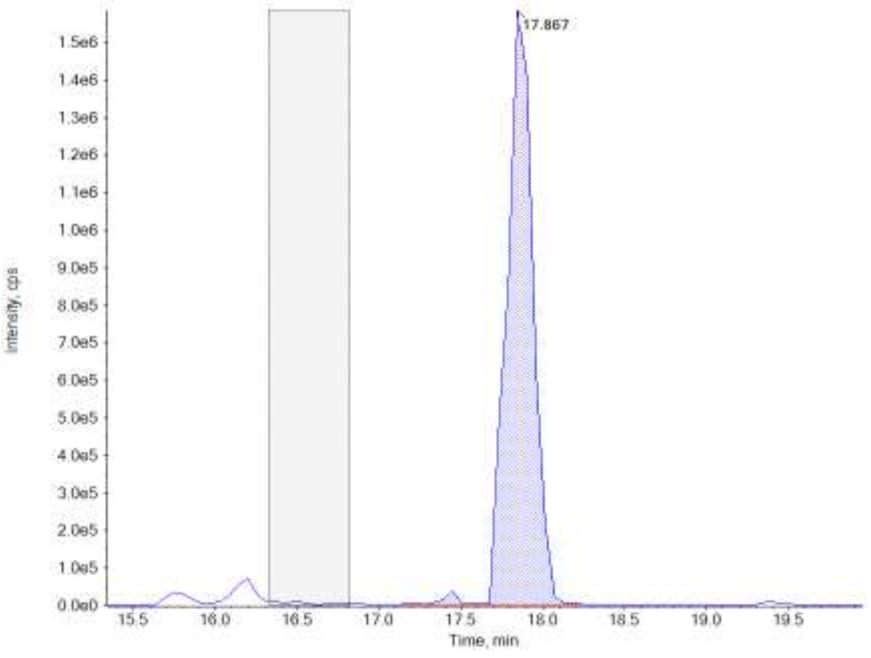


**Acquired / Library MSMS**

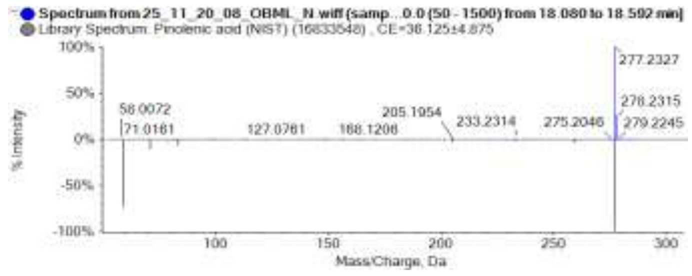


**Acquired / Theoretical MS**

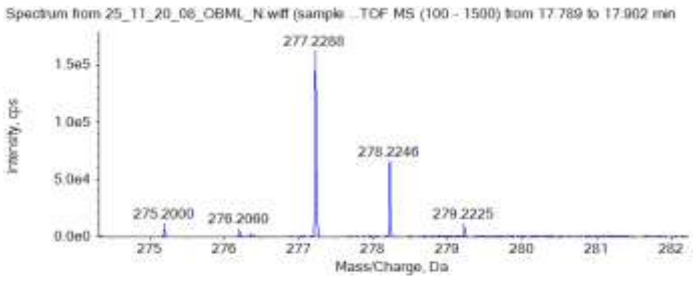

**590.4449 / 17.85** (Mass/FragMass/RT/Isotope/Library/Formula/Ion Ratio)

**Retention Time:** 17.87 minutes
**Precursor m/z :** 590.4449
**Fit (%)** 35.9% **RFit (%)** 99.6%

**Exp RT:** 17.85 minutes
**Analyte Name:**
590.4449 / 17.85

**Collision Energy = 35 ± 15 eV**

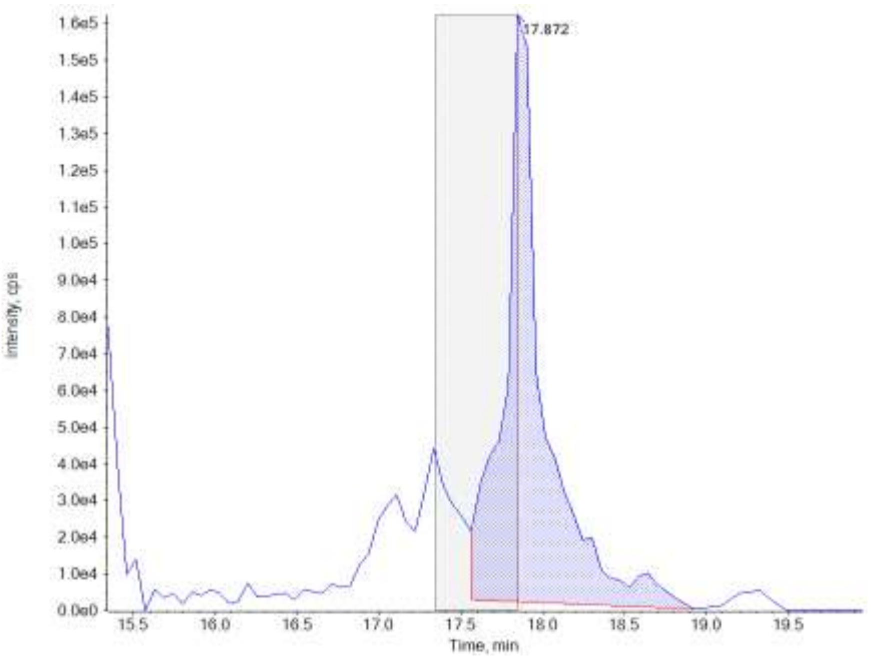


**Acquired / Library MSMS**

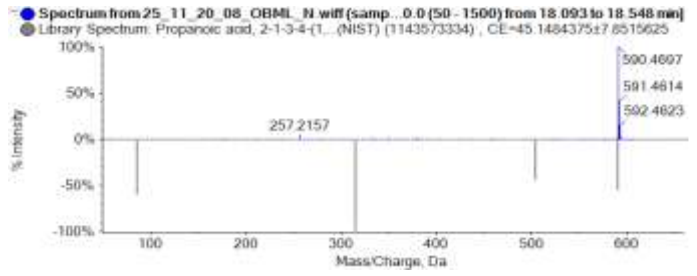


**Acquired / Theoretical MS**

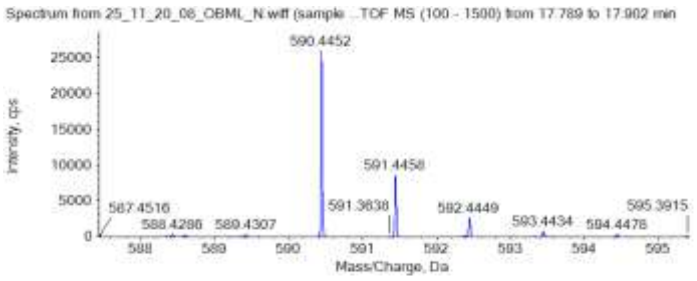

**831.4934 / 18.02** (Mass/FragMass/RT/Isotope/Library/Formula/Ion Ratio)

**Retention Time:** 17.57 minutes
**Precursor m/z :** 831.4934
**Fit (%)** 93.5% **RFit (%)** 93.5%

**Exp RT:** 18.02 minutes
**Analyte Name:**
831.4934 / 18.02

**Collision Energy = 35 ± 15 eV**

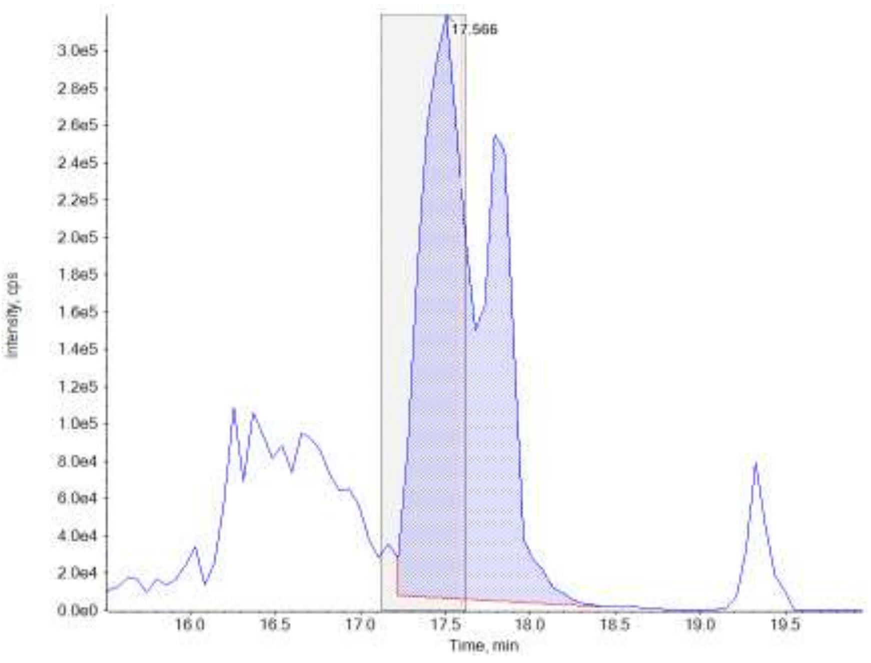


**Acquired / Library MSMS**

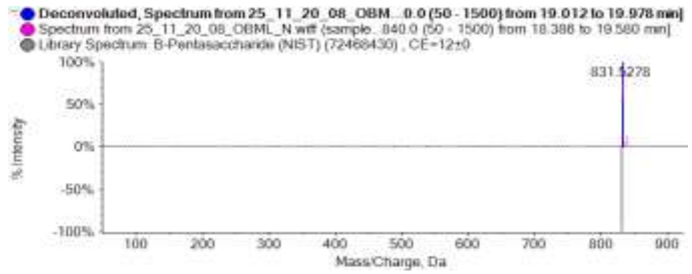


**Acquired / Theoretical MS**

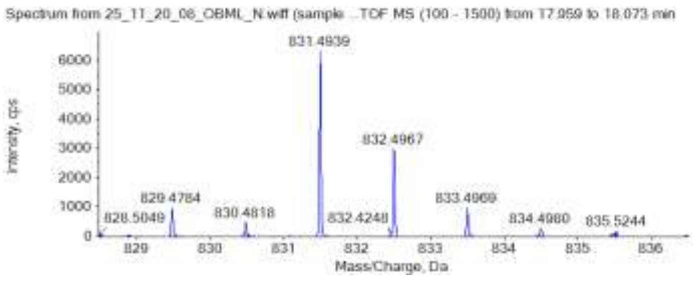

## 253.2232 / 18.13 [M-H2O-H]- (Mass/FragMass/RT/Isotope/Library/Formula/Ion Ratio)

| | |
|---|---|
| **Retention Time:** 18.19 minutes | **Exp RT:** 18.13 minutes |
| **Precursor m/z :** 253.2232 | **Analyte Name:** |
| **Fit (%)** 98.9% **RFit (%)** 98.9% | 253.2232 / 18.13 [M-H2O-H]- |

**Collision Energy = 35 ± 15 eV**

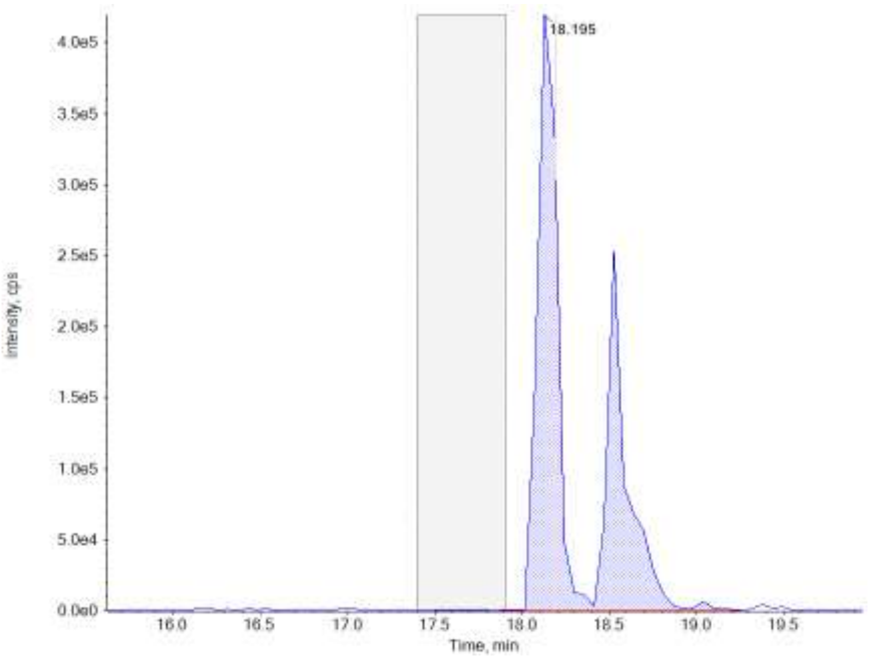


**Acquired / Library MSMS**

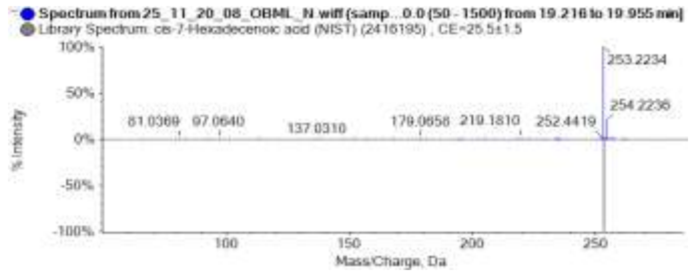


**Acquired / Theoretical MS**

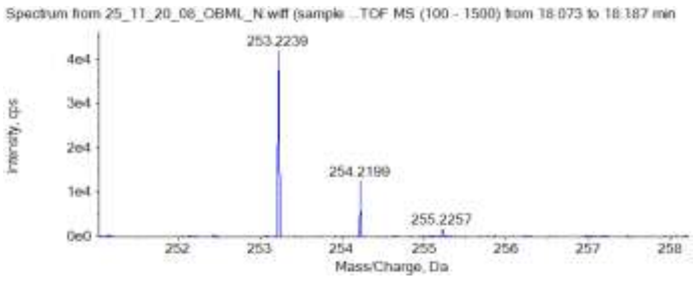

## 271.2331 / 17.96 [M-H]- (Mass/FragMass/RT/Isotope/Library/Formula/Ion Ratio)

| | |
|---|---|
| **Retention Time:** 17.97 minutes | **Exp RT:** 17.96 minutes |
| **Precursor m/z :** 271.2331 | **Analyte Name:** |
| **Fit (%)** 87.2% **RFit (%)** 98.3% | 271.2331 / 17.96 [M-H]- |

**Collision Energy = 35 ± 15 eV**

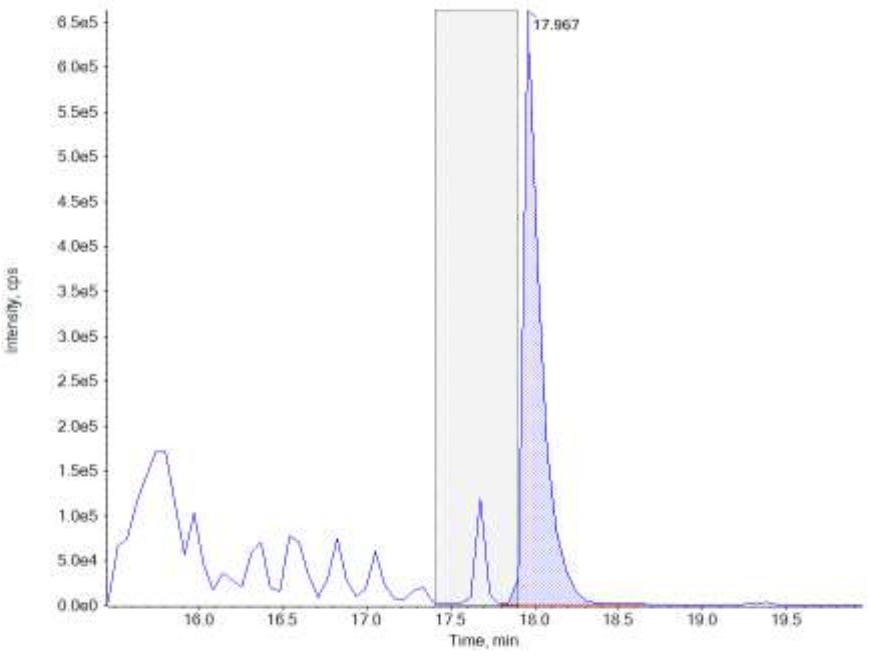


**Acquired / Library MSMS**

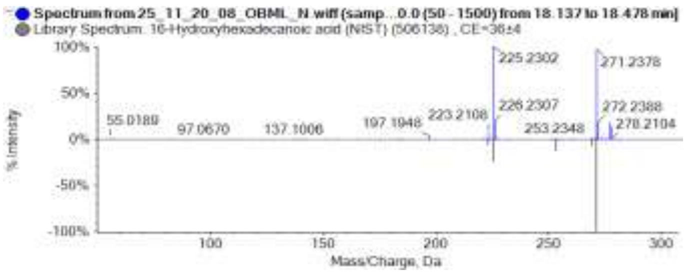


**Acquired / Theoretical MS**

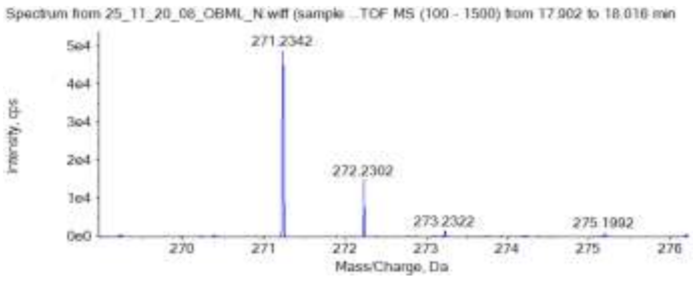

## 279.2401 / 18.30

(Mass/FragMass/RT/Isotope/Library/Formula/Ion Ratio)

| | |
|---|---|
| **Retention Time:** 18.28 minutes | **Exp RT:** 18.30 minutes |
| **Precursor m/z :** 279.2401 | **Analyte Name:** |
| **Fit (%)** 100.0% **RFit (%)** 100.0% | 279.2401 / 18.30 |

**Collision Energy = 35 ± 15 eV**

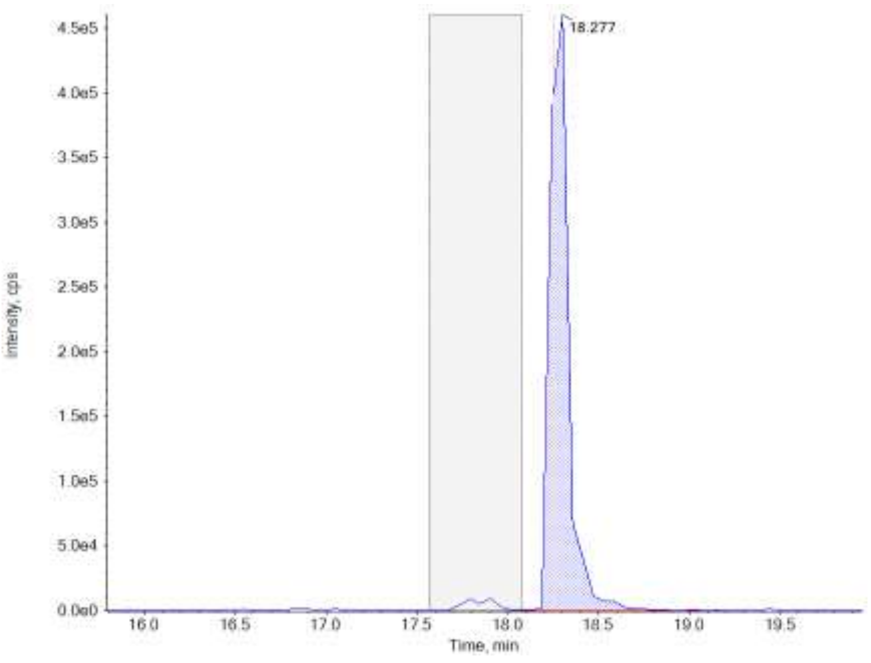


**Acquired / Library MSMS**

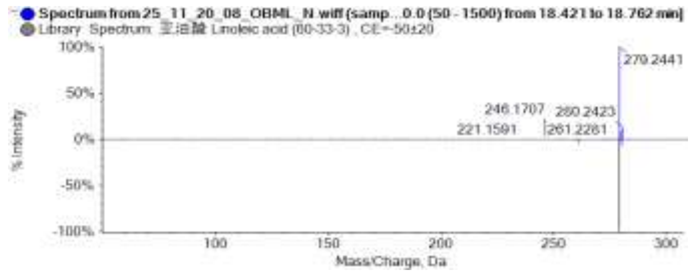


**Acquired / Theoretical MS**

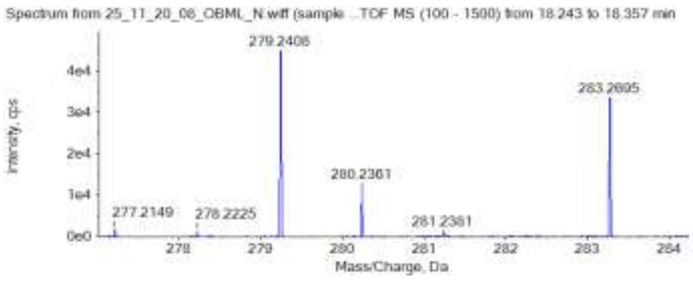

**255.2390 / 18.58** (Mass/FragMass/RT/Isotope/Library/Formula/Ion Ratio)

**Retention Time:** 18.57 minutes
**Precursor m/z :** 255.2390
**Fit (%)** 76.4% **RFit (%)** 97.1%

**Exp RT:** 18.58 minutes
**Analyte Name:**
255.2390 / 18.58

**Collision Energy = 35 ± 15 eV**

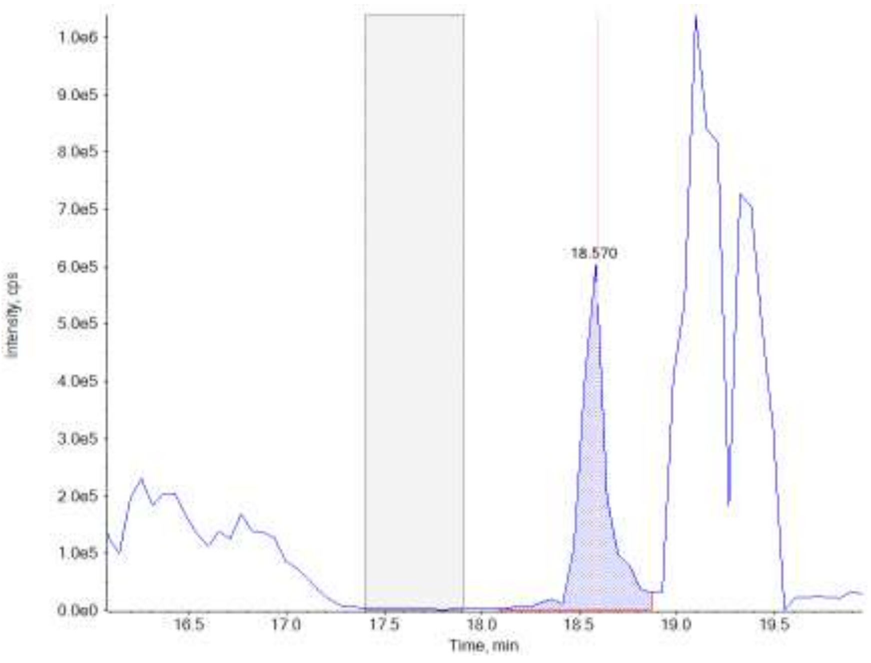


**Acquired / Library MSMS**

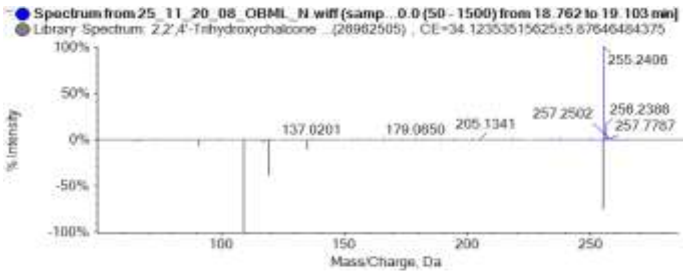


**Acquired / Theoretical MS**

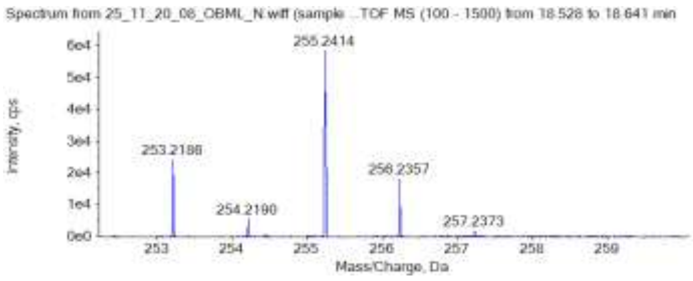

**311.2933 / 18.81** (Mass/FragMass/RT/Isotope/Library/Formula/Ion Ratio)

**Retention Time:** 18.83 minutes
**Precursor m/z :** 311.2933
**Fit (%)** 75.8% **RFit (%)** 99.1%

**Exp RT:** 18.81 minutes
**Analyte Name:**
311.2933 / 18.81

**Collision Energy = 35 ± 15 eV**

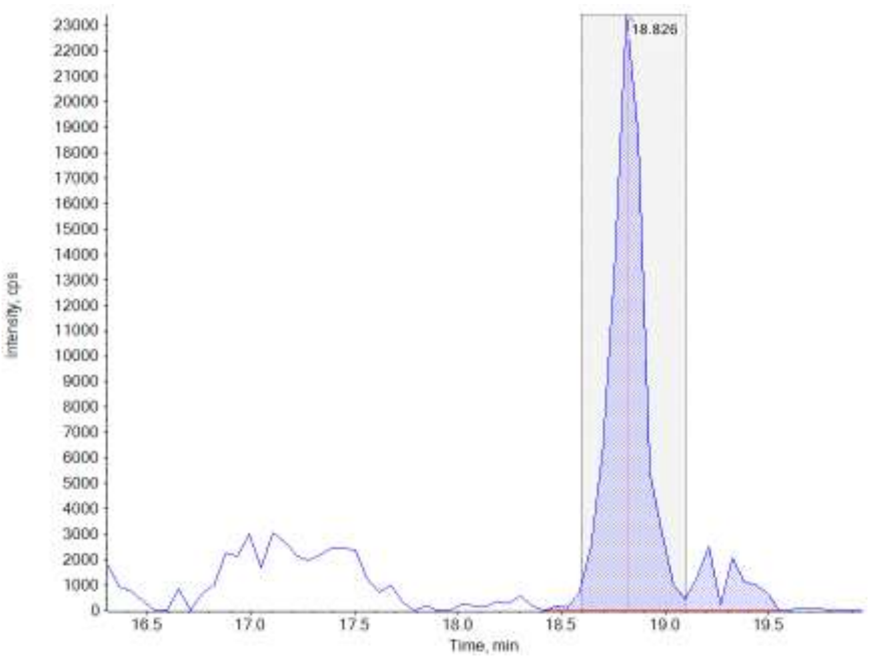


**Acquired / Library MSMS**

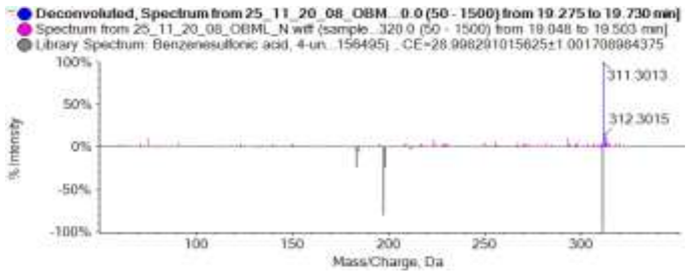


**Acquired / Theoretical MS**

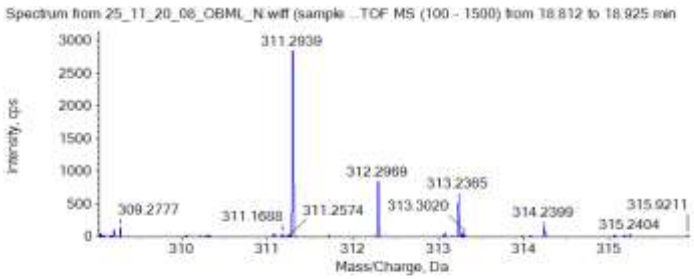

Created with SCIEX OS 3.3.0
Printed:12/9/2025 2:26:41 PM

## 281.2487 / 19.15 (Mass/FragMass/RT/Isotope/Library/Formula/Ion Ratio)

**Retention Time:** 19.15 minutes
**Precursor m/z :** 281.2487
**Fit (%)** 98.5% **RFit (%)** 98.5%

**Exp RT:** 19.15 minutes
**Analyte Name:**
281.2487 / 19.15

**Collision Energy = 35 ± 15 eV**

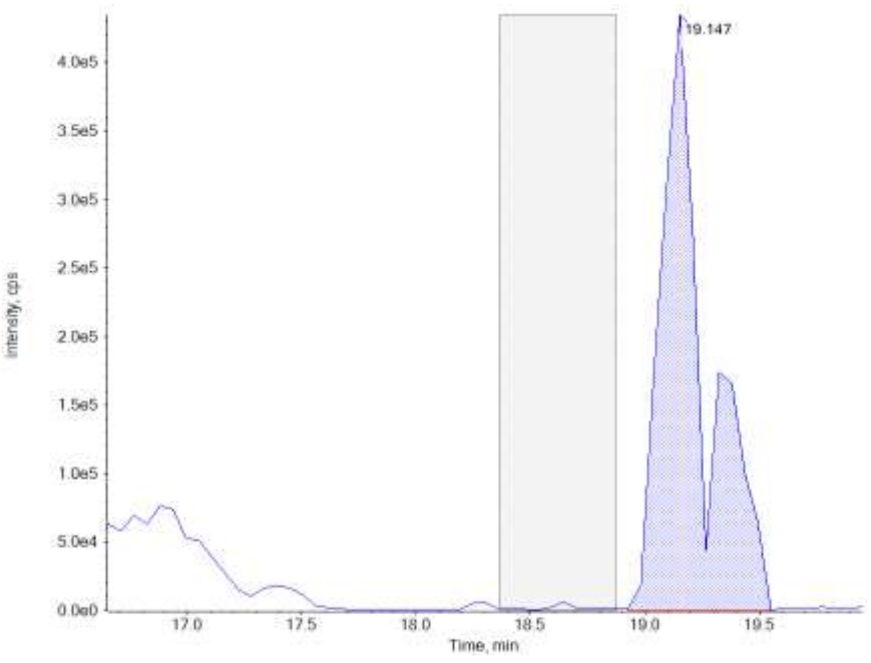


**Acquired / Library MSMS**

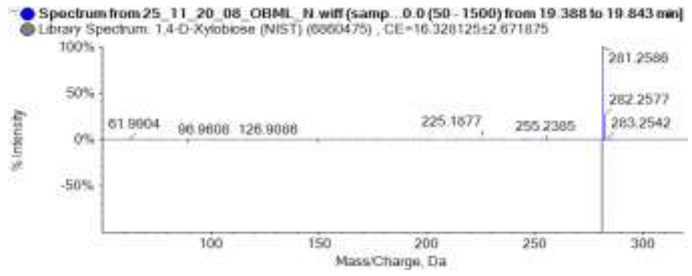


**Acquired / Theoretical MS**

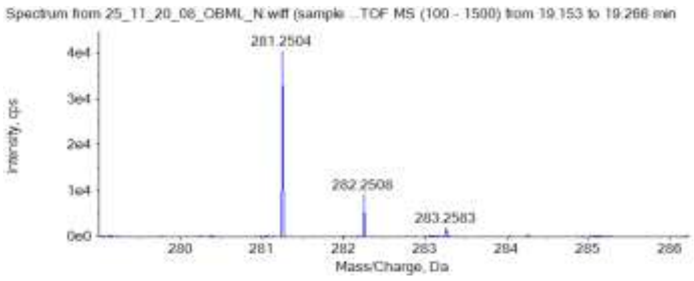

## 349.2733 / 19.15 (Mass/FragMass/RT/Isotope/Library/Formula/Ion Ratio)

**Retention Time:** 19.16 minutes | **Exp RT:** 19.15 minutes
**Precursor m/z :** 349.2733 | **Analyte Name:** 349.2733 / 19.15
**Fit (%)** 72.8% **RFit (%)** 99.6%

**Collision Energy = 35 ± 15 eV**

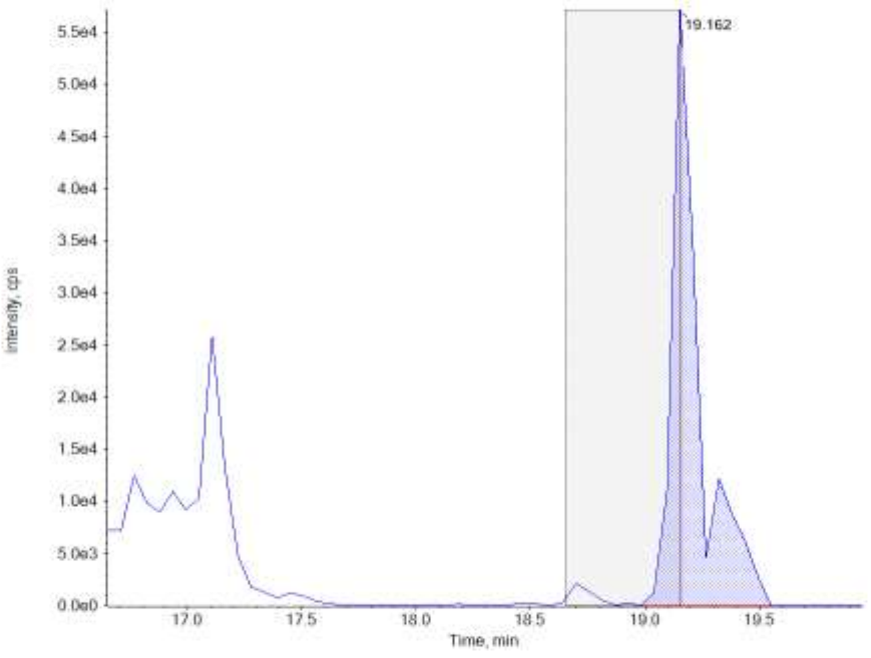


**Acquired / Library MSMS**

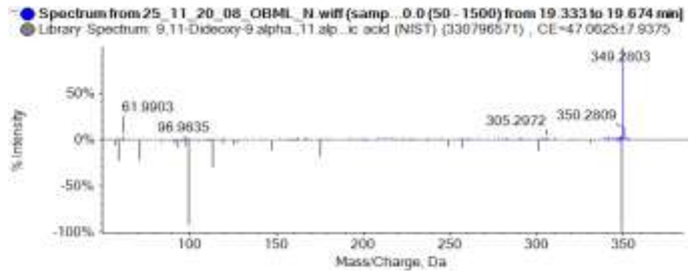


**Acquired / Theoretical MS**

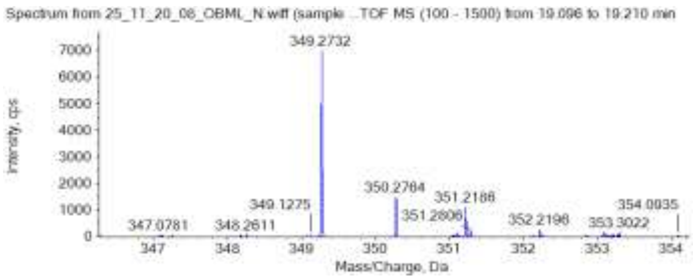

**609.3920 / 19.27** (Mass/FragMass/RT/Isotope/Library/Formula/Ion Ratio)

**Retention Time:** 19.27 minutes
**Precursor m/z :** 609.3920
**Fit (%)** 90.3% **RFit (%)** 97.6%

**Exp RT:** 19.27 minutes
**Analyte Name:**
609.3920 / 19.27

**Collision Energy = 35 ± 15 eV**

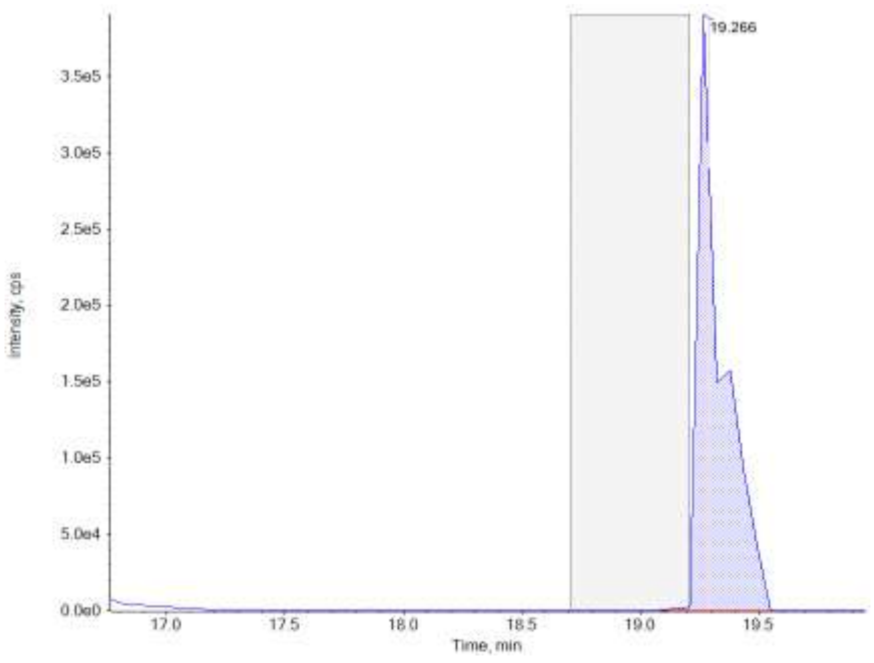


**Acquired / Library MSMS**

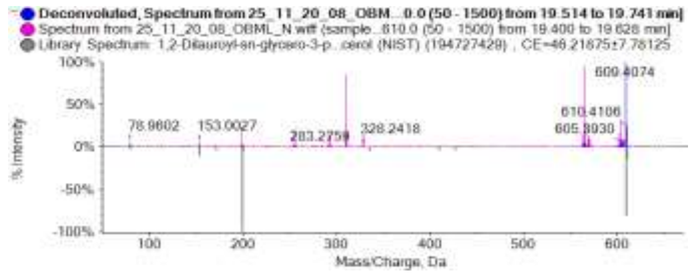


**Acquired / Theoretical MS**

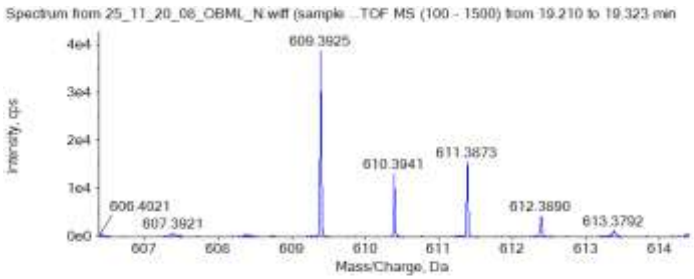

Created with SCIEX OS 3.3.0
Printed:12/9/2025 2:26:41 PM

## 255.2372 / 19.38 (Mass/FragMass/RT/Isotope/Library/Formula/Ion Ratio)

**Retention Time:** 19.22 minutes
**Precursor m/z :** 255.2372
**Fit (%)** 71.3% **RFit (%)** 99.6%

**Exp RT:** 19.38 minutes
**Analyte Name:**
255.2372 / 19.38

**Collision Energy = 35 ± 15 eV**

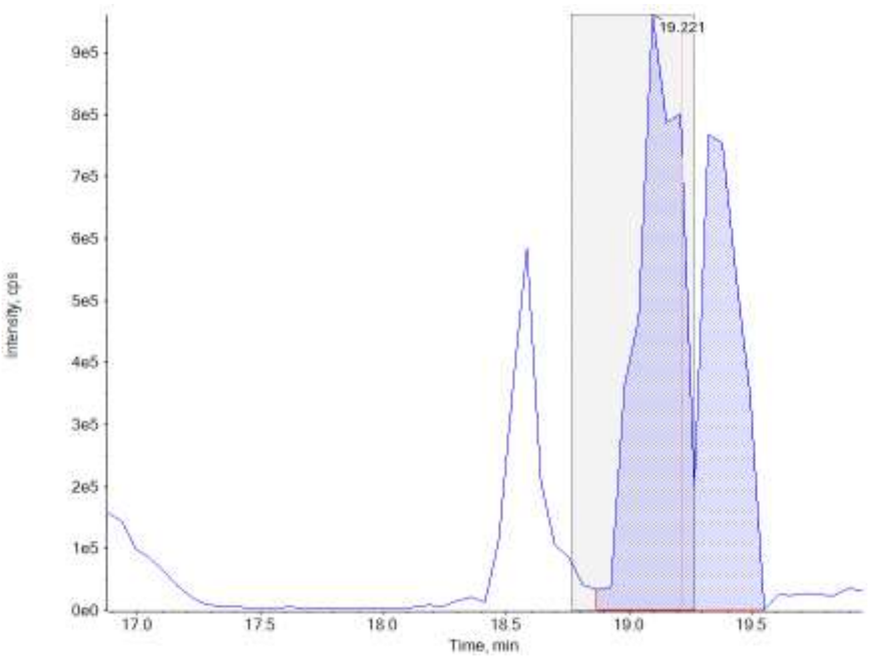


**Acquired / Library MSMS**

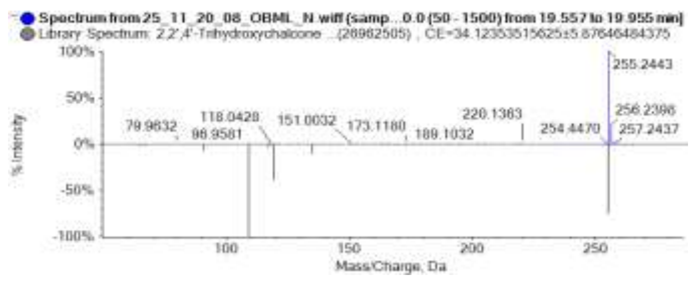


**Acquired / Theoretical MS**

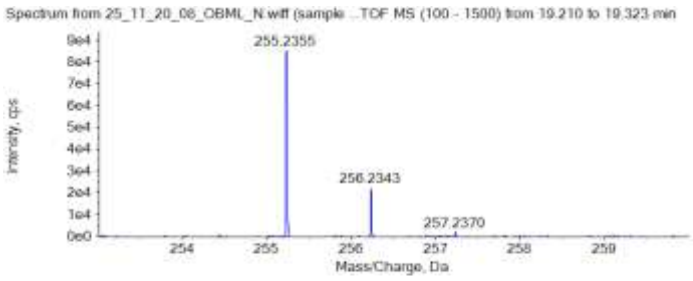

Supplement: Supplementary file 1 [file ijms-27-04945-s001.zip › 2.IJMS-4262115 Supplementary Data OBMLE-negative mode.pdf]
