# Supplementary material for: Box–Behnken Design Optimization of High-Pressure Processed Bitter Melon (Momordica charantia) Leaf Extract Enhancing Phytochemicals, Anticancer, and Anti-Inflammatory Activities
Source: Int J Mol Sci. 2026 May 29;27(11):4945. doi: 10.3390/ijms27114945 (PMC13256669; doi:10.3390/ijms27114945)

O-BMLE (positive mode)

25_12_08_05_OBML_P.wiff - OBML_P

Data File	25_12_08_05_OBML_P.wiff	Result Table	OBML-P-CE40
Acquisition Date	2025-12-08T13:28:49	Algorithm Used	MQ4
Acquisition Method	Pos_DIA_20min_251218.dam	Instrument Name	TripleTOF 6600
Project	Untargeted	Processing Method	

Extracted Ion Chromatogram

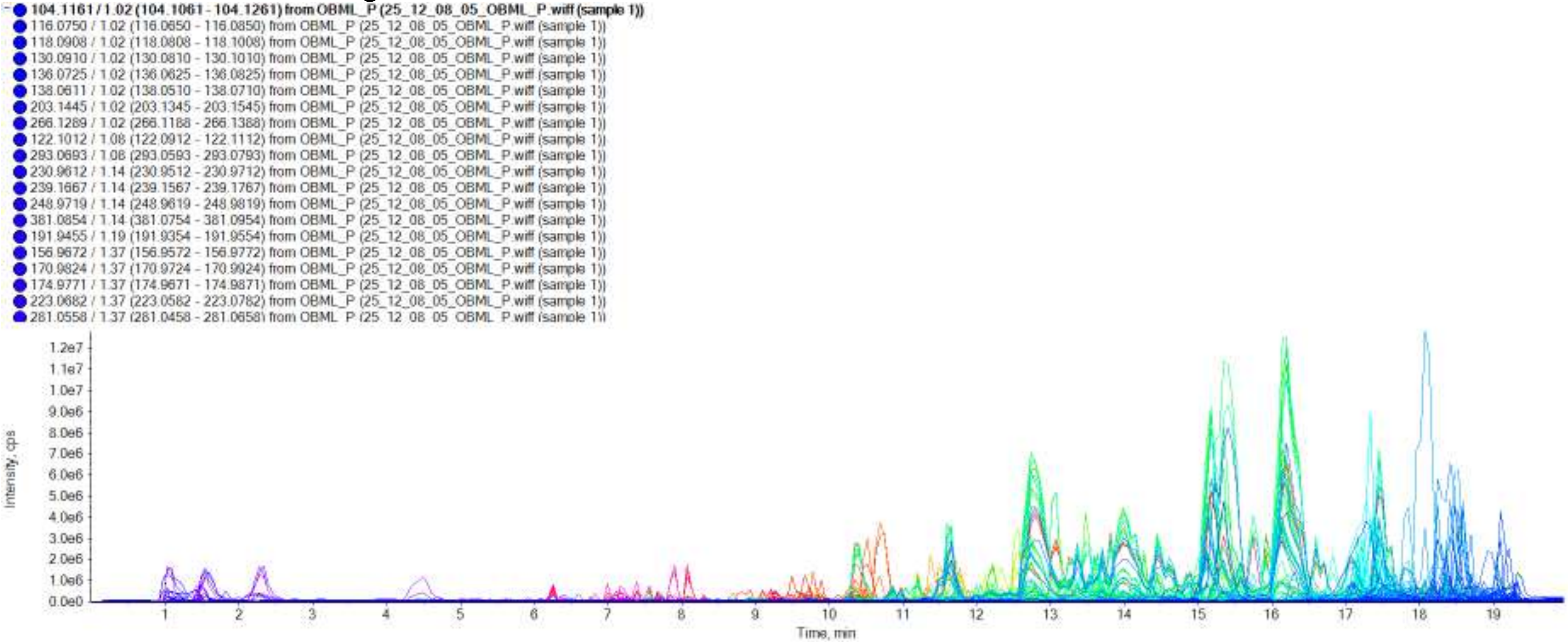

Summary

#	Analyte Peak Name	Mass Error Confidence	Fragment Mass Error Confidence	RT Confidence	Isotope Confidence	Library Confidence	Formula Confidence	Ion Ratio Confidence	Sample Name
1	104.1161 / 1.02					✓			OBML_P
2	116.0750 / 1.02					●			OBML_P
3	118.0908 / 1.02					▲			OBML_P
5	136.0725 / 1.02					✓			OBML_P
14	381.0854 / 1.14					✓			OBML_P
21	337.0945 / 1.37					✓			OBML_P
24	262.1396 / 1.42 [M+H]+					●			OBML_P
26	152.0636 / 1.48					✓			OBML_P
27	182.0869 / 1.48					✓			OBML_P
28	268.1102 / 1.48 [M]+					✓			OBML_P
29	286.1454 / 1.76 [M+NH4]+					✓			OBML_P
30	284.1065 / 1.48 [M+H]+					✓			OBML_P
31	322.0612 / 1.54 [M+K]+					✓			OBML_P
32	276.1546 / 1.54					▲			OBML_P
33	332.1165 / 1.54					✓			OBML_P
34	132.1118 / 1.59					✓			OBML_P
39	204.1281 / 1.82					●			OBML_P
40	282.1284 / 1.82					✓			OBML_P
42	182.0977 / 1.93 [M+H]+					✓			OBML_P
44	247.1344 / 1.99					●			OBML_P

47	121.0899 / 2.27	●	●	●	●	✓	●	●	OBML_P
48	166.0966 / 2.27	●	●	●	●	✓	●	●	OBML_P
51	120.0918 / 2.33	●	●	●	●	✓	●	●	OBML_P
52	328.1472 / 2.33	●	●	●	●	▲	●	●	OBML_P
56	170.0859 / 2.67	●	●	●	●	✓	●	●	OBML_P
57	186.1290 / 2.73	●	●	●	●	🔴	●	●	OBML_P
59	232.1256 / 3.13	●	●	●	●	🔴	●	●	OBML_P
60	102.0960 / 3.64	●	●	●	●	🔴	●	●	OBML_P
66	268.1593 / 4.04	●	●	●	●	▲	●	●	OBML_P
70	144.0852 / 4.49	●	●	●	●	🔴	●	●	OBML_P
71	146.0652 / 4.49	●	●	●	●	🔴	●	●	OBML_P
72	170.0647 / 4.49	●	●	●	●	✓	●	●	OBML_P
73	188.0842 / 4.49	●	●	●	●	✓	●	●	OBML_P
74	205.1047 / 4.49	●	●	●	●	✓	●	●	OBML_P
78	139.0442 / 5.00	●	●	●	●	✓	●	●	OBML_P
79	223.1382 / 5.00	●	●	●	●	🔴	●	●	OBML_P
84	441.1576 / 5.06 [M+K]+	●	●	●	●	✓	●	●	OBML_P
85	205.1273 / 5.06	●	●	●	●	✓	●	●	OBML_P
88	172.1013 / 5.34	●	●	●	●	🔴	●	●	OBML_P
89	295.1345 / 5.40	●	●	●	●	▲	●	●	OBML_P
90	282.1752 / 5.51	●	●	●	●	✓	●	●	OBML_P
94	175.1533 / 5.63	●	●	●	●	🔴	●	●	OBML_P
96	389.1619 / 5.63	●	●	●	●	✓	●	●	OBML_P

97	427.1763 / 5.63					✓			OBML_P
100	231.1180 / 5.91					●			OBML_P
103	136.0804 / 5.97					✓			OBML_P
104	166.0912 / 6.08					●			OBML_P
106	266.1804 / 6.20					✓			OBML_P
110	425.1641 / 6.25					✓			OBML_P
113	296.1899 / 6.31 [M+H]+					✓			OBML_P
114	294.1766 / 6.42					●			OBML_P
115	359.1745 / 6.42					✓			OBML_P
118	331.1210 / 6.59					●			OBML_P
122	264.1510 / 6.99					✓			OBML_P
125	227.1708 / 7.10					●			OBML_P
126	389.2230 / 7.10					●			OBML_P
128	438.2454 / 7.16					▲			OBML_P
129	585.2929 / 7.16 [M+K]+					✓			OBML_P
130	547.3369 / 7.16 [M+H]+					✓			OBML_P
141	629.3214 / 7.39					✓			OBML_P
142	636.4251 / 7.39					▲			OBML_P
148	652.4213 / 7.56					●			OBML_P
149	673.3463 / 7.56					✓			OBML_P
150	680.4509 / 7.56					●			OBML_P
152	322.2076 / 7.62					▲			OBML_P
153	227.1707 / 7.67					✓			OBML_P

159	209.1588 / 7.73					✓			OBML_P
160	364.2035 / 7.73					●			OBML_P
161	371.2118 / 7.73					▲			OBML_P
166	346.1943 / 7.84					✓			OBML_P
167	179.1121 / 7.90					✓			OBML_P
175	193.1645 / 8.07					●			OBML_P
176	211.1794 / 8.07					●			OBML_P
181	146.0658 / 8.13					✓			OBML_P
188	225.1536 / 8.30					●			OBML_P
203	213.1548 / 8.70					▲			OBML_P
213	370.2071 / 8.92					✓			OBML_P
218	115.0812 / 9.04					▲			OBML_P
219	291.2011 / 9.04					●			OBML_P
225	414.2916 / 9.21					✓			OBML_P
226	426.2901 / 9.21					✓			OBML_P
234	209.1590 / 9.38					✓			OBML_P
235	434.2593 / 9.38					●			OBML_P
236	444.2670 / 9.49					●			OBML_P
239	257.1339 / 9.55					●			OBML_P
242	135.1216 / 9.66					✓			OBML_P
243	175.1529 / 9.66					▲			OBML_P
244	193.1641 / 9.66					✓			OBML_P
245	211.1751 / 9.66					✓			OBML_P

246	446.2851 / 9.66					✓			OBML_P
247	191.1489 / 9.72					▲			OBML_P
250	747.5549 / 9.72					✓			OBML_P
251	275.2067 / 9.78					●			OBML_P
256	439.3270 / 9.83					✓			OBML_P
257	137.1376 / 9.89					✓			OBML_P
261	391.3407 / 10.29					✓			OBML_P
263	437.3462 / 10.29					▲			OBML_P
266	195.1430 / 10.34					▲			OBML_P
269	275.2117 / 10.34					✓			OBML_P
270	293.2221 / 10.34					✓			OBML_P
271	311.2287 / 10.34					▲			OBML_P
273	367.1934 / 10.34					▲			OBML_P
293	595.3154 / 10.86					●			OBML_P
304	309.2117 / 11.03					●			OBML_P
311	195.1427 / 11.20					▲			OBML_P
314	389.3274 / 11.20					✓			OBML_P
315	407.3386 / 11.20					●			OBML_P
318	746.4889 / 11.25					●			OBML_P
323	181.1297 / 11.59					✓			OBML_P
324	391.3432 / 11.59					✓			OBML_P
327	419.3413 / 11.59					●			OBML_P
332	291.2017 / 11.71					●			OBML_P

334	730.4933 / 11.71					✓			OBML_P
336	428.2737 / 11.76					✓			OBML_P
347	439.3265 / 11.99					✓			OBML_P
352	373.1373 / 12.11					●			OBML_P
357	273.1906 / 12.22					✓			OBML_P
358	291.2039 / 12.22					●			OBML_P
360	389.3271 / 12.22					✓			OBML_P
362	425.3462 / 12.22					✓			OBML_P
368	498.4000 / 12.33					✓			OBML_P
371	283.1061 / 12.50					✓			OBML_P
376	540.4138 / 12.56					✓			OBML_P
379	313.1138 / 12.62					✓			OBML_P
380	355.2683 / 12.73					●			OBML_P
381	391.3422 / 12.73 [M+H]+					●			OBML_P
382	423.3679 / 12.45 [M+CH3OH+H]+					✓			OBML_P
383	401.3255 / 12.73					●			OBML_P
385	419.3446 / 12.73					●			OBML_P
391	337.2580 / 12.79					●			OBML_P
404	343.1245 / 13.13					●			OBML_P
409	271.1494 / 13.30					●			OBML_P
417	405.3573 / 13.47					✓			OBML_P
421	453.3436 / 13.64					●			OBML_P
428	554.4237 / 13.70 [M+H]+					●			OBML_P

431	195.1426 / 13.81					✓			OBML_P
432	199.1381 / 13.81					●			OBML_P
433	275.2088 / 13.81					▲			OBML_P
434	293.2187 / 13.81					●			OBML_P
438	337.2582 / 13.98					●			OBML_P
439	391.3411 / 13.98					▲			OBML_P
442	441.3775 / 13.98 [M+CH3OH+H]+					▲			OBML_P
446	621.4403 / 13.98					▲			OBML_P
456	668.4407 / 14.21					▲			OBML_P
459	407.3353 / 14.32 [M+H]+					●			OBML_P
460	353.2772 / 14.38					✓			OBML_P
461	421.3510 / 14.38					✓			OBML_P
465	291.2028 / 14.44					●			OBML_P
477	293.2189 / 14.83					●			OBML_P
482	236.1122 / 15.06					●			OBML_P
483	309.2623 / 15.12					●			OBML_P
484	391.3427 / 15.12 [M+H]+					●			OBML_P
485	423.3673 / 14.72 [M+CH3OH+H]+					▲			OBML_P
488	419.3409 / 15.12					●			OBML_P
490	455.3593 / 15.12					▲			OBML_P
494	279.2285 / 15.29					✓			OBML_P
497	439.3605 / 15.35					✓			OBML_P
499	277.2312 / 15.40					✓			OBML_P

503	219.1802 / 15.69	○	○	○	○	✓	○	○	OBML_P
505	261.2277 / 15.74	○	○	○	○	●	○	○	OBML_P
506	335.2635 / 15.74	○	○	○	○	✓	○	○	OBML_P
507	353.2820 / 15.74	○	○	○	○	✓	○	○	OBML_P
508	497.3167 / 15.74	○	○	○	○	▲	○	○	OBML_P
511	275.2092 / 15.91	○	○	○	○	▲	○	○	OBML_P
512	293.2237 / 15.91	○	○	○	○	●	○	○	OBML_P
513	341.3109 / 15.97	○	○	○	○	✓	○	○	OBML_P
517	203.1843 / 16.14	○	○	○	○	●	○	○	OBML_P
518	391.3438 / 16.14	○	○	○	○	●	○	○	OBML_P
521	419.3442 / 16.14	○	○	○	○	●	○	○	OBML_P
522	455.3604 / 16.14	○	○	○	○	▲	○	○	OBML_P
530	365.2737 / 16.42	○	○	○	○	✓	○	○	OBML_P
531	311.2634 / 16.48	○	○	○	○	✓	○	○	OBML_P
533	423.3667 / 16.54	○	○	○	○	●	○	○	OBML_P
534	447.2565 / 16.54	○	○	○	○	✓	○	○	OBML_P
539	179.1481 / 16.60	○	○	○	○	●	○	○	OBML_P
540	277.2256 / 16.60	○	○	○	○	✓	○	○	OBML_P
541	295.2359 / 16.60	○	○	○	○	✓	○	○	OBML_P
542	454.2976 / 16.77	○	○	○	○	✓	○	○	OBML_P
545	423.3201 / 16.82	○	○	○	○	●	○	○	OBML_P
547	305.2533 / 16.88	○	○	○	○	✓	○	○	OBML_P
548	653.3003 / 16.88	○	○	○	○	✓	○	○	OBML_P

563	453.3428 / 17.16	●	●	●	●	🔴	●	●	OBML_P
568	338.3514 / 17.28	●	●	●	●	✓	●	●	OBML_P
576	359.3228 / 17.39	●	●	●	●	✓	●	●	OBML_P
580	391.3412 / 17.45	●	●	●	●	✓	●	●	OBML_P
582	419.3373 / 17.45	●	●	●	●	🔴	●	●	OBML_P
584	353.2759 / 17.50	●	●	●	●	✓	●	●	OBML_P
585	599.4148 / 17.50	●	●	●	●	🔴	●	●	OBML_P
588	367.2695 / 17.56	●	●	●	●	▲	●	●	OBML_P
589	609.4562 / 17.56	●	●	●	●	✓	●	●	OBML_P
594	667.3163 / 17.62	●	●	●	●	🔴	●	●	OBML_P
595	615.4079 / 17.68	●	●	●	●	✓	●	●	OBML_P
596	623.2557 / 17.68	●	●	●	●	🔴	●	●	OBML_P
599	375.2739 / 17.73	●	●	●	●	▲	●	●	OBML_P
602	280.2434 / 17.79	●	●	●	●	🔴	●	●	OBML_P
607	279.2414 / 17.85	●	●	●	●	▲	●	●	OBML_P
608	734.5957 / 17.85	●	●	●	●	▲	●	●	OBML_P
616	583.4185 / 18.19	●	●	●	●	✓	●	●	OBML_P
625	419.3381 / 18.36	●	●	●	●	▲	●	●	OBML_P
634	439.3618 / 18.53	●	●	●	●	✓	●	●	OBML_P
642	429.3781 / 18.70	●	●	●	●	✓	●	●	OBML_P
644	344.3376 / 18.81	●	●	●	●	✓	●	●	OBML_P
649	293.2537 / 18.93	●	●	●	●	✓	●	●	OBML_P
655	277.2237 / 19.04	●	●	●	●	✓	●	●	OBML_P

658	553.4305 / 19.04 [M+NH4]+								OBML_P
661	309.2898 / 19.10								OBML_P
662	351.2953 / 19.10								OBML_P
663	567.4237 / 19.10								OBML_P
668	311.2415 / 19.15								OBML_P
671	565.2491 / 19.21								OBML_P
679	535.2756 / 19.32								OBML_P
680	584.4255 / 19.32								OBML_P

#	Analyte Peak Name	Component Name	Component Type	Component Group Name	Expected RT	Area	Retention Time	Retention Time Delta (min)	Formula	Precursor Mass	Found At Mass
1	104.1161 / 1.02	104.1161 / 1.02	Quantifiers	[No data for] Component Group Name	1.02	2.437e+07	1.07	N/A	{103.10941}	104.116	104.1160
2	116.0750 / 1.02	116.0750 / 1.02	Quantifiers	[No data for] Component Group Name	1.02	2.249e+06	1.05	N/A	{115.06832}	116.075	116.0753
3	118.0908 / 1.02	118.0908 / 1.02	Quantifiers	[No data for] Component Group Name	1.02	2.473e+06	1.05	N/A	{117.08412}	118.091	118.0911
5	136.0725 / 1.02	136.0725 / 1.02	Quantifiers	[No data for] Component Group Name	1.02	1.571e+07	1.05	N/A	{135.06578}	136.073	136.0732
14	381.0854 / 1.14	381.0854 / 1.14	Quantifiers	[No data for] Component Group Name	1.14	9.515e+06	1.09	N/A	{380.07872}	381.085	381.0853
21	337.0945 / 1.37	337.0945 / 1.37	Quantifiers	[No data for] Component Group Name	1.37	8.853e+05	1.31	N/A	{336.08774}	337.094	337.0951
24	262.1396 / 1.42 [M+H]+	262.1396 / 1.42 [M+H]+	Qualifiers	294.1659 / 1.54	1.42	2.560e+06	1.42	N/A	{261.13283}	262.140	262.1393
26	152.0636 / 1.48	152.0636 / 1.48	Quantifiers	[No data for] Component Group Name	1.48	5.998e+06	1.50	N/A	{151.05687}	152.064	152.0638
27	182.0869 / 1.48	182.0869 / 1.48	Quantifiers	[No data for] Component Group Name	1.48	1.525e+06	1.48	N/A	{181.08017}	182.087	182.0868
28	268.1102 / 1.48 [M]+	268.1102 / 1.48 [M]+	Quantifiers	268.1102 / 1.48	1.48	1.061e+06	1.48	N/A	{268.11129}	268.110	268.1102
29	286.1454 / 1.76	286.1454 / 1.76	Qualifiers	268.1102 / 1.48	1.76	8.871e+05	1.77	N/A	{268.11217}	286.145	286.1458

	**[M+NH4]+**	**[M+NH4]+**									

30	284.1065 / 1.48 [M+H]+	284.1065 / 1.48 [M+H]+	Quantifiers	284.1065 / 1.48	1.48	4.646e+06	1.50	N/A	{283.09979}	284.107	284.1068
31	322.0612 / 1.54 [M+K]+	322.0612 / 1.54 [M+K]+	Qualifiers	284.1065 / 1.48	1.54	2.862e+06	1.51	N/A	{283.09854}	322.061	322.0613
32	276.1546 / 1.54	276.1546 / 1.54	Quantifiers	[No data for] Component Group Name	1.54	1.635e+07	1.53	N/A	{275.14782}	276.155	276.1549
33	332.1165 / 1.54	332.1165 / 1.54	Quantifiers	[No data for] Component Group Name	1.54	2.422e+06	1.55	N/A	{331.10973}	332.116	332.1165
34	132.1118 / 1.59	132.1118 / 1.59	Quantifiers	[No data for] Component Group Name	1.59	1.661e+07	1.59	N/A	{131.10507}	132.112	132.1122
39	204.1281 / 1.82	204.1281 / 1.82	Quantifiers	[No data for] Component Group Name	1.82	8.810e+05	1.88	N/A	{203.12139}	204.128	204.1285
40	282.1284 / 1.82	282.1284 / 1.82	Quantifiers	[No data for] Component Group Name	1.82	4.907e+06	1.82	N/A	{281.12167}	282.128	282.1292
42	182.0977 / 1.93 [M+H]+	182.0977 / 1.93 [M+H]+	Qualifiers	214.1241 / 1.88	1.93	7.093e+05	1.91	N/A	{181.09099}	182.098	182.0978
44	247.1344 / 1.99	247.1344 / 1.99	Quantifiers	[No data for] Component Group Name	1.99	5.690e+05	1.99	N/A	{246.12765}	247.134	247.1346
47	121.0899 / 2.27	121.0899 / 2.27	Quantifiers	[No data for] Component Group Name	2.27	3.043e+06	2.28	N/A	{120.08314}	121.090	121.0898
48	166.0966 / 2.27	166.0966 / 2.27	Quantifiers	[No data for] Component Group Name	2.27	1.914e+07	2.28	N/A	{165.08983}	166.097	166.0962
51	120.0918 / 2.33	120.0918 / 2.33	Quantifiers	[No data for] Component Group Name	2.33	2.138e+07	2.31	N/A	{119.08511}	120.092	120.0924
52	328.1472 / 2.33	328.1472 / 2.33	Quantifiers	[No data for] Component Group Name	2.33	9.847e+06	2.24	N/A	{327.14045}	328.147	328.1469
56	170.0859 / 2.67	170.0859 / 2.67	Quantifiers	[No data for] Component Group Name	2.67	6.943e+05	2.65	N/A	{169.07916}	170.086	170.0861
57	186.1290 / 2.73	186.1290 / 2.73	Quantifiers	[No data for] Component Group Name	2.73	1.226e+06	2.68	N/A	{185.12228}	186.129	186.1290
59	232.1256 / 3.13	232.1256 / 3.13	Quantifiers	[No data for] Component Group Name	3.13	2.260e+06	3.10	N/A	{231.11891}	232.126	232.1259
60	102.0960 / 3.64	102.0960 / 3.64	Quantifiers	[No data for]	3.64	4.356e+05	3.61	N/A	{101.08931}	102.096	102.0961

				Component Group Name								
66	268.1593 / 4.04	268.1593 / 4.04	Quantifiers	[No data for] Component Group Name	4.04	6.089e+05	4.01	N/A	{267.15254}	268.159	268.1597
70	144.0852 / 4.49	144.0852 / 4.49	Quantifiers	[No data for] Component Group Name	4.49	7.167e+05	4.45	N/A	{143.07849}	144.085	144.0855
71	146.0652 / 4.49	146.0652 / 4.49	Quantifiers	[No data for] Component Group Name	4.49	2.437e+06	4.46	N/A	{145.05845}	146.065	146.0653
72	170.0647 / 4.49	170.0647 / 4.49	Quantifiers	[No data for] Component Group Name	4.49	5.387e+05	4.46	N/A	{169.05799}	170.065	170.0651
73	188.0842 / 4.49	188.0842 / 4.49	Quantifiers	[No data for] Component Group Name	4.49	1.999e+07	4.46	N/A	{187.07748}	188.084	188.0846
74	205.1047 / 4.49	205.1047 / 4.49	Quantifiers	[No data for] Component Group Name	4.49	7.168e+06	4.46	N/A	{204.09799}	205.105	205.1046
78	139.0442 / 5.00	139.0442 / 5.00	Quantifiers	[No data for] Component Group Name	5.00	1.025e+06	4.98	N/A	{138.03749}	139.044	139.0443
79	223.1382 / 5.00	223.1382 / 5.00	Quantifiers	[No data for] Component Group Name	5.00	7.967e+05	5.03	N/A	{222.13148}	223.138	223.1384
84	441.1576 / 5.06 [M+K]+	441.1576 / 5.06 [M+K]+	Qualifiers	403.2019 / 5.00	5.06	1.003e+06	5.02	N/A	{402.19502}	441.158	441.1576
85	205.1273 / 5.06	205.1273 / 5.06	Quantifiers	[No data for] Component Group Name	5.06	8.827e+05	5.06	N/A	{204.12056}	205.127	205.1274
88	172.1013 / 5.34	172.1013 / 5.34	Quantifiers	[No data for] Component Group Name	5.34	3.333e+05	5.32	N/A	{171.09461}	172.101	172.1014
89	295.1345 / 5.40	295.1345 / 5.40	Quantifiers	[No data for] Component Group Name	5.40	9.062e+05	5.41	N/A	{294.12782}	295.135	295.1346
90	282.1752 / 5.51	282.1752 / 5.51	Quantifiers	[No data for] Component Group Name	5.51	5.470e+05	5.49	N/A	{281.16842}	282.175	282.1753
94	175.1533 / 5.63	175.1533 / 5.63	Quantifiers	[No data for] Component Group Name	5.63	9.002e+05	5.62	N/A	{174.14661}	175.153	175.1533
96	389.1619 / 5.63	389.1619 / 5.63	Quantifiers	[No data for] Component Group Name	5.63	1.085e+06	5.55	N/A	{388.15520}	389.162	389.1621

97	427.1763 / 5.63	427.1763 / 5.63	Quantifiers	[No data for] Component Group Name	5.63	3.763e+05	5.63	N/A	{426.16956}	427.176	427.1774
100	231.1180 / 5.91	231.1180 / 5.91	Quantifiers	[No data for] Component Group Name	5.91	7.258e+05	5.91	N/A	{230.11128}	231.118	231.1180
103	136.0804 / 5.97	136.0804 / 5.97	Quantifiers	[No data for] Component Group Name	5.97	8.373e+05	5.97	N/A	{135.07367}	136.080	136.0807
104	166.0912 / 6.08	166.0912 / 6.08	Quantifiers	[No data for] Component Group Name	6.08	1.296e+06	6.08	N/A	{165.08452}	166.091	166.0915
106	266.1804 / 6.20	266.1804 / 6.20	Quantifiers	[No data for] Component Group Name	6.20	8.068e+05	6.22	N/A	{265.17366}	266.180	266.1806
110	425.1641 / 6.25	425.1641 / 6.25	Quantifiers	[No data for] Component Group Name	6.25	1.990e+06	6.25	N/A	{424.15738}	425.164	425.1643
113	296.1899 / 6.31 [M+H]+	296.1899 / 6.31 [M+H]+	Qualifiers	295.1807 / 6.37	6.31	9.531e+05	6.30	N/A	{295.18313}	296.190	296.1898
114	294.1766 / 6.42	294.1766 / 6.42	Quantifiers	[No data for] Component Group Name	6.42	2.558e+06	6.40	N/A	{293.16988}	294.177	294.1766
115	359.1745 / 6.42	359.1745 / 6.42	Quantifiers	[No data for] Component Group Name	6.42	8.771e+05	6.42	N/A	{358.16777}	359.174	359.1745
118	331.1210 / 6.59	331.1210 / 6.59	Quantifiers	[No data for] Component Group Name	6.59	6.949e+05	6.59	N/A	{330.11431}	331.121	331.1212
122	264.1510 / 6.99	264.1510 / 6.99	Quantifiers	[No data for] Component Group Name	6.99	8.178e+05	7.02	N/A	{263.14425}	264.151	264.1511
125	227.1708 / 7.10	227.1708 / 7.10	Quantifiers	[No data for] Component Group Name	7.11	3.040e+06	7.10	N/A	{226.16407}	227.171	227.1708
126	389.2230 / 7.10	389.2230 / 7.10	Quantifiers	[No data for] Component Group Name	7.11	2.333e+06	7.10	N/A	{388.21629}	389.223	389.2230
128	438.2454 / 7.16	438.2454 / 7.16	Quantifiers	[No data for] Component Group Name	7.16	5.235e+06	7.16	N/A	{437.23866}	438.245	438.2460
129	585.2929 / 7.16 [M+K]+	585.2929 / 7.16 [M+K]+	Quantifiers	585.2929 / 7.16	7.16	1.173e+06	7.18	N/A	{546.33031}	585.293	585.2929
130	547.3369 / 7.16 [M+H]+	547.3369 / 7.16 [M+H]+	Qualifiers	585.2929 / 7.16	7.16	9.270e+05	7.18	N/A	{546.33015}	547.337	547.3367
141	629.3214 / 7.39	629.3214 / 7.39	Quantifiers	[No data for]	7.39	1.262e+06	7.39	N/A	{628.31470}	629.321	629.3210

				Component Group Name								
142	636.4251 / 7.39	636.4251 / 7.39	Quantifiers	[No data for] Component Group Name	7.39	2.657e+06	7.39	N/A	{635.41841}	636.425	636.4256
148	652.4213 / 7.56	652.4213 / 7.56	Quantifiers	[No data for] Component Group Name	7.56	3.899e+06	7.56	N/A	{651.41461}	652.421	652.4216
149	673.3463 / 7.56	673.3463 / 7.56	Quantifiers	[No data for] Component Group Name	7.56	1.124e+06	7.56	N/A	{672.33958}	673.346	673.3461
150	680.4509 / 7.56	680.4509 / 7.56	Quantifiers	[No data for] Component Group Name	7.56	2.687e+06	7.56	N/A	{679.44415}	680.451	680.4509
152	322.2076 / 7.62	322.2076 / 7.62	Quantifiers	[No data for] Component Group Name	7.62	1.239e+06	7.62	N/A	{321.20085}	322.208	322.2073
153	227.1707 / 7.67	227.1707 / 7.67	Quantifiers	[No data for] Component Group Name	7.67	2.279e+06	7.67	N/A	{226.16398}	227.171	227.1705
159	209.1588 / 7.73	209.1588 / 7.73	Quantifiers	[No data for] Component Group Name	7.73	3.730e+06	7.79	N/A	{208.15210}	209.159	209.1590
160	364.2035 / 7.73	364.2035 / 7.73	Quantifiers	[No data for] Component Group Name	7.73	6.345e+06	7.51	N/A	{363.19677}	364.203	364.2037
161	371.2118 / 7.73	371.2118 / 7.73	Quantifiers	[No data for] Component Group Name	7.73	1.907e+06	7.73	N/A	{370.20504}	371.212	371.2117
166	346.1943 / 7.84	346.1943 / 7.84	Quantifiers	[No data for] Component Group Name	7.84	1.712e+06	7.84	N/A	{345.18758}	346.194	346.1942
167	179.1121 / 7.90	179.1121 / 7.90	Quantifiers	[No data for] Component Group Name	7.90	1.897e+06	7.90	N/A	{178.10541}	179.112	179.1123
175	193.1645 / 8.07	193.1645 / 8.07	Quantifiers	[No data for] Component Group Name	8.07	1.630e+06	8.07	N/A	{192.15782}	193.165	193.1646
176	211.1794 / 8.07	211.1794 / 8.07	Quantifiers	[No data for] Component Group Name	8.07	8.734e+06	8.07	N/A	{210.17266}	211.179	211.1798
181	146.0658 / 8.13	146.0658 / 8.13	Quantifiers	[No data for] Component Group Name	8.13	1.420e+06	8.13	N/A	{145.05910}	146.066	146.0658
188	225.1536 / 8.30	225.1536 / 8.30	Quantifiers	[No data for] Component Group	8.30	9.274e+05	8.28	N/A	{224.14687}	225.154	225.1539

				Name							
203	213.1548 / 8.70	213.1548 / 8.70	Quantifiers	[No data for] Component Group Name	8.70	2.391e+06	8.70	N/A	{212.14805}	213.155	213.1548
213	370.2071 / 8.92	370.2071 / 8.92	Quantifiers	[No data for] Component Group Name	8.92	1.120e+06	8.92	N/A	{369.20038}	370.207	370.2073
218	115.0812 / 9.04	115.0812 / 9.04	Quantifiers	[No data for] Component Group Name	9.04	1.354e+06	9.04	N/A	{114.07446}	115.081	115.0812
219	291.2011 / 9.04	291.2011 / 9.04	Quantifiers	[No data for] Component Group Name	9.04	3.191e+06	8.87	N/A	{290.19435}	291.201	291.2012
225	414.2916 / 9.21	414.2916 / 9.21	Quantifiers	[No data for] Component Group Name	9.21	8.597e+06	9.15	N/A	{413.28486}	414.292	414.2919
226	426.2901 / 9.21	426.2901 / 9.21	Quantifiers	[No data for] Component Group Name	9.21	3.281e+06	9.29	N/A	{425.28341}	426.290	426.2903
234	209.1590 / 9.38	209.1590 / 9.38	Quantifiers	[No data for] Component Group Name	9.38	2.534e+06	9.48	N/A	{208.15231}	209.159	209.1588
235	434.2593 / 9.38	434.2593 / 9.38	Quantifiers	[No data for] Component Group Name	9.38	3.150e+06	9.30	N/A	{433.25259}	434.259	434.2597
236	444.2670 / 9.49	444.2670 / 9.49	Quantifiers	[No data for] Component Group Name	9.49	3.320e+06	9.46	N/A	{443.26026}	444.267	444.2667
239	257.1339 / 9.55	257.1339 / 9.55	Quantifiers	[No data for] Component Group Name	9.55	9.962e+05	9.56	N/A	{256.12713}	257.134	257.1342
242	135.1216 / 9.66	135.1216 / 9.66	Quantifiers	[No data for] Component Group Name	9.66	1.320e+06	9.64	N/A	{134.11486}	135.122	135.1216
243	175.1529 / 9.66	175.1529 / 9.66	Quantifiers	[No data for] Component Group Name	9.66	1.068e+06	9.64	N/A	{174.14620}	175.153	175.1532
244	193.1641 / 9.66	193.1641 / 9.66	Quantifiers	[No data for] Component Group Name	9.66	2.323e+06	9.64	N/A	{192.15734}	193.164	193.1641
245	211.1751 / 9.66	211.1751 / 9.66	Quantifiers	[No data for] Component Group Name	9.66	2.792e+06	9.65	N/A	{210.16835}	211.175	211.1751
246	446.2851 / 9.66	446.2851 / 9.66	Quantifiers	[No data for] Component Group Name	9.66	2.514e+07	9.59	N/A	{445.27836}	446.285	446.2856

247	191.1489 / 9.72	191.1489 / 9.72	Quantifiers	[No data for] Component Group Name	9.72	4.082e+06	9.70	N/A	{190.14218}	191.149	191.1489
250	747.5549 / 9.72	747.5549 / 9.72	Quantifiers	[No data for] Component Group Name	9.72	1.667e+06	9.72	N/A	{746.54812}	747.555	747.5545
251	275.2067 / 9.78	275.2067 / 9.78	Quantifiers	[No data for] Component Group Name	9.78	1.353e+06	9.76	N/A	{274.19993}	275.207	275.2067
256	439.3270 / 9.83	439.3270 / 9.83	Quantifiers	[No data for] Component Group Name	9.83	3.239e+06	9.83	N/A	{438.32028}	439.327	439.3269
257	137.1376 / 9.89	137.1376 / 9.89	Quantifiers	[No data for] Component Group Name	9.89	2.059e+06	9.89	N/A	{136.13092}	137.138	137.1377
261	391.3407 / 10.29	391.3407 / 10.29	Quantifiers	[No data for] Component Group Name	10.29	7.401e+05	10.28	N/A	{390.33400}	391.341	391.3406
263	437.3462 / 10.29	437.3462 / 10.29	Quantifiers	[No data for] Component Group Name	10.29	9.602e+05	10.28	N/A	{436.33947}	437.346	437.3458
266	195.1430 / 10.34	195.1430 / 10.34	Quantifiers	[No data for] Component Group Name	10.34	3.716e+06	10.38	N/A	{194.13625}	195.143	195.1433
269	275.2117 / 10.34	275.2117 / 10.34	Quantifiers	[No data for] Component Group Name	10.34	5.395e+07	10.37	N/A	{274.20499}	275.212	275.2131
270	293.2221 / 10.34	293.2221 / 10.34	Quantifiers	[No data for] Component Group Name	10.34	4.614e+07	10.37	N/A	{292.21535}	293.222	293.2236
271	311.2287 / 10.34	311.2287 / 10.34	Quantifiers	[No data for] Component Group Name	10.34	1.786e+07	10.49	N/A	{310.22194}	311.229	311.2285
273	367.1934 / 10.34	367.1934 / 10.34	Quantifiers	[No data for] Component Group Name	10.34	3.065e+06	10.37	N/A	{366.18672}	367.193	367.1933
293	595.3154 / 10.86	595.3154 / 10.86	Quantifiers	[No data for] Component Group Name	10.86	1.200e+06	10.83	N/A	{594.30870}	595.315	595.3162
304	309.2117 / 11.03	309.2117 / 11.03	Quantifiers	[No data for] Component Group Name	11.03	2.454e+06	11.03	N/A	{308.20501}	309.212	309.2116
311	195.1427 / 11.20	195.1427 / 11.20	Quantifiers	[No data for] Component Group Name	11.20	3.971e+06	11.16	N/A	{194.13600}	195.143	195.1428
314	389.3274 / 11.20	389.3274 / 11.20	Quantifiers	[No data for]	11.20	5.756e+06	11.20	N/A	{388.32072}	389.327	389.3277

				Component Group Name							
315	407.3386 / 11.20	407.3386 / 11.20	Quantifiers	[No data for] Component Group Name	11.20	7.600e+06	11.20	N/A	{406.33187}	407.339	407.3391
318	746.4889 / 11.25	746.4889 / 11.25	Quantifiers	[No data for] Component Group Name	11.25	3.223e+06	11.27	N/A	{745.48218}	746.489	746.4890
323	181.1297 / 11.59	181.1297 / 11.59	Quantifiers	[No data for] Component Group Name	11.59	1.698e+07	11.59	N/A	{180.12295}	181.130	181.1304
324	391.3432 / 11.59	391.3432 / 11.59	Quantifiers	[No data for] Component Group Name	11.59	1.313e+07	11.61	N/A	{390.33651}	391.343	391.3440
327	419.3413 / 11.59	419.3413 / 11.59	Quantifiers	[No data for] Component Group Name	11.59	2.910e+07	11.62	N/A	{418.33455}	419.341	419.3443
332	291.2017 / 11.71	291.2017 / 11.71	Quantifiers	[No data for] Component Group Name	11.71	2.607e+07	12.20	N/A	{290.19495}	291.202	291.2053
334	730.4933 / 11.71	730.4933 / 11.71	Quantifiers	[No data for] Component Group Name	11.71	7.330e+06	11.77	N/A	{729.48655}	730.493	730.4930
336	428.2737 / 11.76	428.2737 / 11.76	Quantifiers	[No data for] Component Group Name	11.77	1.005e+07	11.77	N/A	{427.26695}	428.274	428.2742
347	439.3265 / 11.99	439.3265 / 11.99	Quantifiers	[No data for] Component Group Name	11.99	1.332e+06	12.00	N/A	{438.31981}	439.327	439.3269
352	373.1373 / 12.11	373.1373 / 12.11	Quantifiers	[No data for] Component Group Name	12.11	6.362e+06	12.11	N/A	{372.13057}	373.137	373.1373
357	273.1906 / 12.22	273.1906 / 12.22	Quantifiers	[No data for] Component Group Name	12.22	4.981e+06	12.01	N/A	{272.18382}	273.191	273.1905
358	291.2039 / 12.22	291.2039 / 12.22	Quantifiers	[No data for] Component Group Name	12.22	3.050e+07	12.20	N/A	{290.19712}	291.204	291.2053
360	389.3271 / 12.22	389.3271 / 12.22	Quantifiers	[No data for] Component Group Name	12.22	7.627e+06	12.19	N/A	{388.32042}	389.327	389.3275
362	425.3462 / 12.22	425.3462 / 12.22	Quantifiers	[No data for] Component Group Name	12.22	2.630e+06	12.19	N/A	{424.33944}	425.346	425.3462
368	498.4000 / 12.33	498.4000 / 12.33	Quantifiers	[No data for] Component Group	12.33	5.678e+06	12.33	N/A	{497.39323}	498.400	498.4003

				Name							
371	283.1061 / 12.50	283.1061 / 12.50	Quantifiers	[No data for] Component Group Name	12.50	1.084e+07	12.52	N/A	{282.09936}	283.106	283.1062
376	540.4138 / 12.56	540.4138 / 12.56	Quantifiers	[No data for] Component Group Name	12.56	9.669e+06	12.54	N/A	{539.40702}	540.414	540.4139
379	313.1138 / 12.62	313.1138 / 12.62	Quantifiers	[No data for] Component Group Name	12.62	6.545e+06	12.62	N/A	{312.10704}	313.114	313.1138
380	355.2683 / 12.73	355.2683 / 12.73	Quantifiers	[No data for] Component Group Name	12.73	9.944e+06	12.77	N/A	{354.26153}	355.268	355.2683
381	391.3422 / 12.73 [M+H]+	391.3422 / 12.73 [M+H]+	Quantifiers	391.3422 / 12.73	12.73	2.989e+07	12.75	N/A	{390.33552}	391.342	391.3421
382	423.3679 / 12.45 [M+CH3OH+H]+	423.3679 / 12.45 [M+CH3OH+H]+	Qualifiers	391.3422 / 12.73	12.45	4.466e+06	12.45	N/A	{390.33495}	423.368	423.3680
383	401.3255 / 12.73	401.3255 / 12.73	Quantifiers	[No data for] Component Group Name	12.73	1.447e+07	12.77	N/A	{400.31873}	401.325	401.3255
385	419.3446 / 12.73	419.3446 / 12.73	Quantifiers	[No data for] Component Group Name	12.73	1.362e+08	12.78	N/A	{418.33791}	419.345	419.3443
391	337.2580 / 12.79	337.2580 / 12.79	Quantifiers	[No data for] Component Group Name	12.79	1.294e+07	12.76	N/A	{336.25127}	337.258	337.2580
404	343.1245 / 13.13	343.1245 / 13.13	Quantifiers	[No data for] Component Group Name	13.13	5.572e+06	13.13	N/A	{342.11774}	343.124	343.1245
409	271.1494 / 13.30	271.1494 / 13.30	Quantifiers	[No data for] Component Group Name	13.30	2.333e+06	13.28	N/A	{270.14265}	271.149	271.1496
417	405.3573 / 13.47	405.3573 / 13.47	Quantifiers	[No data for] Component Group Name	13.47	5.777e+06	13.47	N/A	{404.35054}	405.357	405.3575
421	453.3436 / 13.64	453.3436 / 13.64	Quantifiers	[No data for] Component Group Name	13.64	3.090e+07	13.62	N/A	{452.33688}	453.344	453.3446
428	554.4237 / 13.70 [M+H]+	554.4237 / 13.70 [M+H]+	Qualifiers	586.4508 / 14.15	13.70	4.986e+06	13.69	N/A	{553.41698}	554.424	554.4245
431	195.1426 / 13.81	195.1426 / 13.81	Quantifiers	[No data for] Component Group Name	13.81	6.123e+06	13.81	N/A	{194.13590}	195.143	195.1428
432	199.1381 / 13.81	199.1381 / 13.81	Quantifiers	[No data for] Component Group Name	13.81	5.660e+06	13.81	N/A	{198.13136}	199.138	199.1384

433	275.2088 / 13.81	275.2088 / 13.81	Quantifiers	[No data for] Component Group Name	13.81	7.667e+07	13.60	N/A	{274.20205}	275.209	275.2088
434	293.2187 / 13.81	293.2187 / 13.81	Quantifiers	[No data for] Component Group Name	13.81	4.879e+07	13.72	N/A	{292.21193}	293.219	293.2181
438	337.2582 / 13.98	337.2582 / 13.98	Quantifiers	[No data for] Component Group Name	13.98	7.547e+06	14.02	N/A	{336.25143}	337.258	337.2581
439	391.3411 / 13.98	391.3411 / 13.98	Quantifiers	[No data for] Component Group Name	13.98	2.189e+07	14.00	N/A	{390.33437}	391.341	391.3413
442	441.3775 / 13.98 [M+CH3OH+H]+	441.3775 / 13.98 [M+CH3OH+H]+	Qualifiers	409.3521 / 13.98	13.98	6.053e+06	13.97	N/A	{408.34455}	441.377	441.3777
446	621.4403 / 13.98	621.4403 / 13.98	Quantifiers	[No data for] Component Group Name	13.98	4.965e+06	13.97	N/A	{620.43360}	621.440	621.4401
456	668.4407 / 14.21	668.4407 / 14.21	Quantifiers	[No data for] Component Group Name	14.21	3.417e+06	14.19	N/A	{667.43397}	668.441	668.4407
459	407.3353 / 14.32 [M+H]+	407.3353 / 14.32 [M+H]+	Qualifiers	439.3618 / 14.38	14.32	1.852e+06	14.66	N/A	{406.32856}	407.335	407.3357
460	353.2772 / 14.38	353.2772 / 14.38	Quantifiers	[No data for] Component Group Name	14.38	1.083e+07	14.38	N/A	{352.27043}	353.277	353.2774
461	421.3510 / 14.38	421.3510 / 14.38	Quantifiers	[No data for] Component Group Name	14.38	8.780e+06	14.38	N/A	{420.34424}	421.351	421.3509
465	291.2028 / 14.44	291.2028 / 14.44	Quantifiers	[No data for] Component Group Name	14.44	1.526e+07	14.44	N/A	{290.19604}	291.203	291.2032
477	293.2189 / 14.83	293.2189 / 14.83	Quantifiers	[No data for] Component Group Name	14.83	1.032e+07	14.83	N/A	{292.21213}	293.219	293.2193
482	236.1122 / 15.06	236.1122 / 15.06	Quantifiers	[No data for] Component Group Name	15.06	1.429e+06	15.06	N/A	{235.10543}	236.112	236.1124
483	309.2623 / 15.12	309.2623 / 15.12	Quantifiers	[No data for] Component Group Name	15.12	4.669e+06	15.13	N/A	{308.25562}	309.262	309.2626
484	391.3427 / 15.12 [M+H]+	391.3427 / 15.12 [M+H]+	Quantifiers	391.3427 / 15.12	15.12	2.301e+07	15.12	N/A	{390.33597}	391.343	391.3430
485	423.3673 / 14.72 [M+CH3OH+H]+	423.3673 / 14.72 [M+CH3OH+H]+	Qualifiers	391.3427 / 15.12	14.72	4.599e+06	14.72	N/A	{390.33432}	423.367	423.3675
488	419.3409 / 15.12	419.3409 / 15.12	Quantifiers	[No data for] Component Group	15.12	3.957e+07	15.13	N/A	{418.33420}	419.341	419.3423

				Name							
490	455.3593 / 15.12	455.3593 / 15.12	Quantifiers	[No data for] Component Group Name	15.12	3.031e+07	15.13	N/A	{454.35255}	455.359	455.3589
494	279.2285 / 15.29	279.2285 / 15.29	Quantifiers	[No data for] Component Group Name	15.29	7.469e+06	15.33	N/A	{278.22173}	279.228	279.2284
497	439.3605 / 15.35	439.3605 / 15.35	Quantifiers	[No data for] Component Group Name	15.35	2.964e+07	15.33	N/A	{438.35382}	439.361	439.3624
499	277.2312 / 15.40	277.2312 / 15.40	Quantifiers	[No data for] Component Group Name	15.40	2.182e+08	15.37	N/A	{276.22450}	277.231	277.2315
503	219.1802 / 15.69	219.1802 / 15.69	Quantifiers	[No data for] Component Group Name	15.69	3.534e+06	15.69	N/A	{218.17346}	219.180	219.1800
505	261.2277 / 15.74	261.2277 / 15.74	Quantifiers	[No data for] Component Group Name	15.74	7.823e+06	15.74	N/A	{260.22096}	261.228	261.2280
506	335.2635 / 15.74	335.2635 / 15.74	Quantifiers	[No data for] Component Group Name	15.74	4.295e+06	15.74	N/A	{334.25682}	335.264	335.2637
507	353.2820 / 15.74	353.2820 / 15.74	Quantifiers	[No data for] Component Group Name	15.74	4.838e+07	15.75	N/A	{352.27531}	353.282	353.2836
508	497.3167 / 15.74	497.3167 / 15.74	Quantifiers	[No data for] Component Group Name	15.74	5.027e+06	15.67	N/A	{496.31002}	497.317	497.3162
511	275.2092 / 15.91	275.2092 / 15.91	Quantifiers	[No data for] Component Group Name	15.91	3.183e+07	15.92	N/A	{274.20250}	275.209	275.2105
512	293.2237 / 15.91	293.2237 / 15.91	Quantifiers	[No data for] Component Group Name	15.91	3.341e+07	15.93	N/A	{292.21694}	293.224	293.2241
513	341.3109 / 15.97	341.3109 / 15.97	Quantifiers	[No data for] Component Group Name	15.97	9.580e+06	15.90	N/A	{340.30422}	341.311	341.3111
517	203.1843 / 16.14	203.1843 / 16.14	Quantifiers	[No data for] Component Group Name	16.14	7.679e+06	16.14	N/A	{202.17757}	203.184	203.1844
518	391.3438 / 16.14	391.3438 / 16.14	Quantifiers	[No data for] Component Group Name	16.14	5.266e+07	16.15	N/A	{390.33705}	391.344	391.3435
521	419.3442 / 16.14	419.3442 / 16.14	Quantifiers	[No data for] Component Group Name	16.14	1.275e+08	16.16	N/A	{418.33744}	419.344	419.3445

522	455.3604 / 16.14	455.3604 / 16.14	Quantifiers	[No data for] Component Group Name	16.14	6.740e+07	16.16	N/A	{454.35370}	455.360	455.3607
530	365.2737 / 16.42	365.2737 / 16.42	Quantifiers	[No data for] Component Group Name	16.43	6.849e+06	16.55	N/A	{364.26698}	365.274	365.2738
531	311.2634 / 16.48	311.2634 / 16.48	Quantifiers	[No data for] Component Group Name	16.48	6.388e+06	16.52	N/A	{310.25662}	311.263	311.2637
533	423.3667 / 16.54	423.3667 / 16.54	Quantifiers	[No data for] Component Group Name	16.54	4.419e+06	16.54	N/A	{422.35992}	423.367	423.3669
534	447.2565 / 16.54	447.2565 / 16.54	Quantifiers	[No data for] Component Group Name	16.54	8.296e+06	16.52	N/A	{446.24977}	447.256	447.2567
539	179.1481 / 16.60	179.1481 / 16.60	Quantifiers	[No data for] Component Group Name	16.60	4.112e+06	16.60	N/A	{178.14138}	179.148	179.1485
540	277.2256 / 16.60	277.2256 / 16.60	Quantifiers	[No data for] Component Group Name	16.60	4.460e+07	16.63	N/A	{276.21888}	277.226	277.2247
541	295.2359 / 16.60	295.2359 / 16.60	Quantifiers	[No data for] Component Group Name	16.60	2.714e+07	16.64	N/A	{294.22919}	295.236	295.2357
542	454.2976 / 16.77	454.2976 / 16.77	Quantifiers	[No data for] Component Group Name	16.77	2.127e+06	16.77	N/A	{453.29083}	454.298	454.2982
545	423.3201 / 16.82	423.3201 / 16.82	Quantifiers	[No data for] Component Group Name	16.82	2.132e+07	16.83	N/A	{422.31334}	423.320	423.3214
547	305.2533 / 16.88	305.2533 / 16.88	Quantifiers	[No data for] Component Group Name	16.88	2.937e+06	16.88	N/A	{304.24661}	305.253	305.2534
548	653.3003 / 16.88	653.3003 / 16.88	Quantifiers	[No data for] Component Group Name	16.88	8.062e+06	16.91	N/A	{652.29361}	653.300	653.3009
563	453.3428 / 17.16	453.3428 / 17.16	Quantifiers	[No data for] Component Group Name	17.16	1.698e+07	17.16	N/A	{452.33611}	453.343	453.3430
568	338.3514 / 17.28	338.3514 / 17.28	Quantifiers	[No data for] Component Group Name	17.28	1.142e+08	17.25	N/A	{337.34471}	338.351	338.3510
576	359.3228 / 17.39	359.3228 / 17.39	Quantifiers	[No data for] Component Group Name	17.39	5.305e+07	17.38	N/A	{358.31603}	359.323	359.3232
580	391.3412 / 17.45	391.3412 / 17.45	Quantifiers	[No data for]	17.45	1.985e+07	17.46	N/A	{390.33444}	391.341	391.3411

Created with SCIEX OS 3.3.0
Printed:12/9/2025 2:05:30 PM

				Component Group Name							
582	419.3373 / 17.45	419.3373 / 17.45	Quantifiers	[No data for] Component Group Name	17.45	5.335e+07	17.46	N/A	{418.33056}	419.337	419.3387
584	353.2759 / 17.50	353.2759 / 17.50	Quantifiers	[No data for] Component Group Name	17.51	2.513e+07	17.47	N/A	{352.26919}	353.276	353.2764
585	599.4148 / 17.50	599.4148 / 17.50	Quantifiers	[No data for] Component Group Name	17.51	3.391e+07	17.52	N/A	{598.40804}	599.415	599.4145
588	367.2695 / 17.56	367.2695 / 17.56	Quantifiers	[No data for] Component Group Name	17.56	9.661e+06	17.56	N/A	{366.26275}	367.269	367.2695
589	609.4562 / 17.56	609.4562 / 17.56	Quantifiers	[No data for] Component Group Name	17.56	1.098e+07	17.56	N/A	{608.44952}	609.456	609.4565
594	667.3163 / 17.62	667.3163 / 17.62	Quantifiers	[No data for] Component Group Name	17.62	9.380e+06	17.63	N/A	{666.30952}	667.316	667.3166
595	615.4079 / 17.68	615.4079 / 17.68	Quantifiers	[No data for] Component Group Name	17.68	1.171e+07	17.67	N/A	{614.40121}	615.408	615.4082
596	623.2557 / 17.68	623.2557 / 17.68	Quantifiers	[No data for] Component Group Name	17.68	2.258e+07	17.58	N/A	{622.24900}	623.256	623.2563
599	375.2739 / 17.73	375.2739 / 17.73	Quantifiers	[No data for] Component Group Name	17.73	6.146e+06	17.73	N/A	{374.26717}	375.274	375.2739
602	280.2434 / 17.79	280.2434 / 17.79	Quantifiers	[No data for] Component Group Name	17.79	8.666e+06	17.82	N/A	{279.23669}	280.243	280.2434
607	279.2414 / 17.85	279.2414 / 17.85	Quantifiers	[No data for] Component Group Name	17.85	3.994e+07	17.82	N/A	{278.23470}	279.241	279.2420
608	734.5957 / 17.85	734.5957 / 17.85	Quantifiers	[No data for] Component Group Name	17.85	1.459e+07	17.83	N/A	{733.58901}	734.596	734.5961
616	583.4185 / 18.19	583.4185 / 18.19	Quantifiers	[No data for] Component Group Name	18.19	2.690e+07	18.17	N/A	{582.41179}	583.419	583.4192
625	419.3381 / 18.36	419.3381 / 18.36	Quantifiers	[No data for] Component Group Name	18.36	1.750e+07	18.36	N/A	{418.33142}	419.338	419.3385
634	439.3618 / 18.53	439.3618 / 18.53	Quantifiers	[No data for] Component Group	18.53	1.478e+07	18.54	N/A	{438.35509}	439.362	439.3621

				Name							
642	429.3781 / 18.70	429.3781 / 18.70	Quantifiers	[No data for] Component Group Name	18.70	7.987e+06	18.68	N/A	{428.37133}	429.378	429.3782
644	344.3376 / 18.81	344.3376 / 18.81	Quantifiers	[No data for] Component Group Name	18.81	1.531e+07	18.80	N/A	{343.33090}	344.338	344.3379
649	293.2537 / 18.93	293.2537 / 18.93	Quantifiers	[No data for] Component Group Name	18.93	2.960e+06	18.93	N/A	{292.24702}	293.254	293.2537
655	277.2237 / 19.04	277.2237 / 19.04	Quantifiers	[No data for] Component Group Name	19.04	1.273e+07	19.05	N/A	{276.21697}	277.224	277.2241
658	553.4305 / 19.04 [M+NH4]+	553.4305 / 19.04 [M+NH4]+	Qualifiers	568.4316 / 19.27	19.04	5.162e+06	19.05	N/A	{535.39722}	553.430	553.4306
661	309.2898 / 19.10	309.2898 / 19.10	Quantifiers	[No data for] Component Group Name	19.10	2.952e+07	19.11	N/A	{308.28307}	309.290	309.2909
662	351.2953 / 19.10	351.2953 / 19.10	Quantifiers	[No data for] Component Group Name	19.10	5.562e+06	19.10	N/A	{350.28861}	351.295	351.2954
663	567.4237 / 19.10	567.4237 / 19.10	Quantifiers	[No data for] Component Group Name	19.10	7.952e+06	19.13	N/A	{566.41695}	567.424	567.4240
668	311.2415 / 19.15	311.2415 / 19.15	Quantifiers	[No data for] Component Group Name	19.15	3.602e+06	19.16	N/A	{310.23479}	311.242	311.2423
671	565.2491 / 19.21	565.2491 / 19.21	Quantifiers	[No data for] Component Group Name	19.21	2.472e+06	19.23	N/A	{564.24242}	565.249	565.2492
679	535.2756 / 19.32	535.2756 / 19.32	Quantifiers	[No data for] Component Group Name	19.32	7.216e+06	19.32	N/A	{534.26892}	535.276	535.2755
680	584.4255 / 19.32	584.4255 / 19.32	Quantifiers	[No data for] Component Group Name	19.32	4.818e+06	19.31	N/A	{583.41880}	584.426	584.4258

#	Analyte Peak Name	Library Hit	Library Score
1	104.1161 / 1.02	Choline cation (NIST) [Smart Confirmation]	96.6
2	116.0750 / 1.02	脯氨酸 Proline [Smart Confirmation]	48.8
3	118.0908 / 1.02	N-Methyl-.alpha.-aminoisobutyric acid (NIST) [Smart Confirmation]	54.8
5	136.0725 / 1.02	Adenine (NIST) [Smart Confirmation]	82.0
14	381.0854 / 1.14	Lactobionic acid (NIST) [Smart Confirmation]	80.2
21	337.0945 / 1.37	Isoprostaglandin-F2.alpha.-IV (NIST) [Smart Confirmation]	84.3
24	262.1396 / 1.42 [M+H]+	O-Phospho-L-tyrosine (NIST) [Smart Confirmation]	32.9

26	152.0636 / 1.48	O-Methyltyramine (NIST) [Smart Confirmation]	81.5
27	182.0869 / 1.48	L-Tyrosine (NIST) [Smart Confirmation]	97.8
28	268.1102 / 1.48 [M]+	腺苷 Adenosine [Smart Confirmation]	86.9
29	286.1454 / 1.76 [M+NH4]+	DL-Homocystine (NIST) [Smart Confirmation]	95.4
30	284.1065 / 1.48 [M+H]+	Guanosine (NIST) [Smart Confirmation]	100.0
31	322.0612 / 1.54 [M+K]+	2,5-Dimethoxy-4-iodoamphetamine (NIST) [Smart Confirmation]	74.0
32	276.1546 / 1.54	Gln-Phe (NIST) [Smart Confirmation]	64.2
33	332.1165 / 1.54	Nizatidine (NIST) [Smart Confirmation]	90.3
34	132.1118 / 1.59	异亮氨酸Isoleucine [Smart Confirmation]	100.0
39	204.1281 / 1.82	Indole-3-pyruvic acid (NIST) [Smart Confirmation]	30.6
40	282.1284 / 1.82	2'-O-Methyladenosine (NIST) [Smart Confirmation]	96.8
42	182.0977 / 1.93 [M+H]+	L-Tyrosine (NIST) [Smart Confirmation]	83.7
44	247.1344 / 1.99	Val-Glu (NIST) [Smart Confirmation]	21.2
47	121.0899 / 2.27	2-(3-Hydroxyphenyl)ethanol (NIST) [Smart Confirmation]	95.8
48	166.0966 / 2.27	苯丙氨酸Phenylalanine [Smart Confirmation]	99.8
51	120.0918 / 2.33	S-(2-Aminoethyl)isothiourea (NIST) [Smart Confirmation]	88.0
52	328.1472 / 2.33	10-Nitrooleic acid (NIST) [Smart Confirmation]	59.4
56	170.0859 / 2.67	Cysteic acid (NIST) [Smart Confirmation]	95.1
57	186.1290 / 2.73	Phosphoserine (NIST) [Smart Confirmation]	33.2
59	232.1256 / 3.13	Asn-Val (NIST) [Smart Confirmation]	29.0
60	102.0960 / 3.64	2-Ethylbutan-1-amine (NIST) [Smart Confirmation]	20.9
66	268.1593 / 4.04	Diethofencarb (NIST) [Smart Confirmation]	50.4
70	144.0852 / 4.49	1-Naphthalenamine (NIST) [Smart Confirmation]	36.7
71	146.0652 / 4.49	Indole-6-carboxaldehyde (NIST) [Smart Confirmation]	21.7
72	170.0647 / 4.49	4-Chloroamphetamine (NIST) [Smart Confirmation]	100.0
73	188.0842 / 4.49	3-Indoleacrylic acid (NIST) [Smart Confirmation]	97.7
74	205.1047 / 4.49	L-Tryptophan (NIST) [Smart Confirmation]	97.9
78	139.0442 / 5.00	Salicylic acid (NIST) [Smart Confirmation]	92.7
79	223.1382 / 5.00	Monobutyl phthalate (NIST) [Smart Confirmation]	23.6
84	441.1576 / 5.06 [M+K]+	Desmethylverapamil (NIST) [Smart Confirmation]	79.5
85	205.1273 / 5.06	Valeroyl salicylate (NIST) [Smart Confirmation]	96.9
88	172.1013 / 5.34	N.alpha.-Acetyl-L-lysine (NIST) [Smart Confirmation]	36.6
89	295.1345 / 5.40	Glu-Phe (NIST) [Smart Confirmation]	61.5
90	282.1752 / 5.51	4-Diphenylmethoxymethylpiperidine (NIST) [Smart Confirmation]	94.7
94	175.1533 / 5.63	trans-Aconitic acid (NIST) [Smart Confirmation]	34.3
96	389.1619 / 5.63	Methyl deoxycholate (NIST) [Smart Confirmation]	100.0
97	427.1763 / 5.63	去乙酰车叶草苷酸甲酯 Deacetyl asperulosidic acid methyl ester +Na [Smart Confirmation]	90.8
100	231.1180 / 5.91	2-Acetyl-5-(tetrahydroxybutyl)imidazole (NIST) [Smart Confirmation]	33.9
103	136.0804 / 5.97	2-Phenylacetamide (NIST) [Smart Confirmation]	91.5
104	166.0912 / 6.08	(-)-Pseudoephedrine (NIST) [Smart Confirmation]	23.9
106	266.1804 / 6.20	N-Acetyl-2-carboxybenzenesulfonamide (NIST) [Smart Confirmation]	75.1
110	425.1641 / 6.25	Benazepril (NIST) [Smart Confirmation]	78.9
113	296.1899 / 6.31 [M+H]+	Nimetazepam (NIST) [Smart Confirmation]	75.1
114	294.1766 / 6.42	Oxaprozin (NIST) [Smart Confirmation]	36.6

115	359.1745 / 6.42	当药苷 sweroside [Smart Confirmation]	97.4
118	331.1210 / 6.59	香紫苏醇 Sclareol +Na [Smart Confirmation]	22.0
122	264.1510 / 6.99	(-)-Neplanocin A (NIST) [Smart Confirmation]	75.6
125	227.1708 / 7.10	2,5-Dimethoxy-4-nitrophenethylamine (NIST) [Smart Confirmation]	38.2
126	389.2230 / 7.10	Secologanin (NIST) [Smart Confirmation]	43.8
128	438.2454 / 7.16	Tyr-Lys-Lys (NIST) [Smart Confirmation]	61.3
129	585.2929 / 7.16 [M+K]+	Hemslecin A (NIST) [Smart Confirmation]	70.0
130	547.3369 / 7.16 [M+H]+	Trp-Trp-Arg (NIST) [Smart Confirmation]	96.6
141	629.3214 / 7.39	Octadecamethyloctasiloxane (NIST) [Smart Confirmation]	97.1
142	636.4251 / 7.39	4-Quinazolinamine, N-1-(cyclohexylmethyl)-4-piperidinyl-2-hexahydro-4-(1-methylethyl)-1H-1,4-diazepin-1-yl-6-methoxy-7-3-(1-piperidinyl)propoxy- (NIST) [Smart Confirmation]	59.9
148	652.4213 / 7.56	1-Hexadecyl-2-azelaoyl-sn-glycero-3-phosphocholine (NIST) [Smart Confirmation]	38.7
149	673.3463 / 7.56	Thapsigargin (NIST) [Smart Confirmation]	91.5
150	680.4509 / 7.56	1,2-Dimyristoyl-sn-glycero-3-phospho-L-serine (NIST) [Smart Confirmation]	22.5
152	322.2076 / 7.62	2,5-Dimethoxy-4-iodoamphetamine (NIST) [Smart Confirmation]	64.0
153	227.1707 / 7.67	Myristoleic acid (NIST) [Smart Confirmation]	70.9
159	209.1588 / 7.73	Ethyl trans-caffeate (NIST) [Smart Confirmation]	76.5
160	364.2035 / 7.73	Prostaglandin F2.alpha. ethylamide (NIST) [Smart Confirmation]	32.2
161	371.2118 / 7.73	秦皮苷 Fraxin [Smart Confirmation]	66.8
166	346.1943 / 7.84	Adenosine-2',3'-monophosphorothioate (NIST) [Smart Confirmation]	85.5
167	179.1121 / 7.90	Hydrocinnamic acid ethyl ester (NIST) [Smart Confirmation]	97.4
175	193.1645 / 8.07	.alpha.-Ionone (NIST) [Smart Confirmation]	34.1
176	211.1794 / 8.07	(.+-.)7-epi-Jasmonic acid (NIST) [Smart Confirmation]	42.1
181	146.0658 / 8.13	Indole-6-carboxaldehyde (NIST) [Smart Confirmation]	96.8
188	225.1536 / 8.30	Pentadecanoic acid (NIST) [Smart Confirmation]	45.2
203	213.1548 / 8.70	n-Propyl gallate (NIST) [Smart Confirmation]	53.2
213	370.2071 / 8.92	Bimatoprost amide (NIST) [Smart Confirmation]	96.8
218	115.0812 / 9.04	Dihydrouracil (NIST) [Smart Confirmation]	56.0
219	291.2011 / 9.04	Isoprothiolane (NIST) [Smart Confirmation]	40.5
225	414.2916 / 9.21	17-Phenoxytrinorprostaglandin F2.alpha. ethylamide (NIST) [Smart Confirmation]	70.1
226	426.2901 / 9.21	Prostaglandin E2 serinolamide (NIST) [Smart Confirmation]	91.9
234	209.1590 / 9.38	O,O-Diethyl thiophosphate (NIST) [Smart Confirmation]	87.7
235	434.2593 / 9.38	N-(3-Pyridyl)indomethacinamide (NIST) [Smart Confirmation]	28.0
236	444.2670 / 9.49	Propaquizafop (NIST) [Smart Confirmation]	42.3
239	257.1339 / 9.55	Indolo3,2-bcarbazole (NIST) [Smart Confirmation]	25.6
242	135.1216 / 9.66	3,4-Dimethylbenzaldehyde (NIST) [Smart Confirmation]	89.4
243	175.1529 / 9.66	trans-Aconitic acid (NIST) [Smart Confirmation]	56.9
244	193.1641 / 9.66	.alpha.-Ionone (NIST) [Smart Confirmation]	87.2
245	211.1751 / 9.66	(.+-.)7-epi-Jasmonic acid (NIST) [Smart Confirmation]	70.9
246	446.2851 / 9.66	L-Serine, N-(1-oxohexadecyl)-O-phosphono- (NIST) [Smart Confirmation]	70.5
247	191.1489 / 9.72	Z-Ligustilide (NIST) [Smart Confirmation]	62.0
250	747.5549 / 9.72	Nigericin (NIST) [Smart Confirmation]	100.0
251	275.2067 / 9.78	Lys-Lys (NIST) [Smart Confirmation]	24.3
256	439.3270 / 9.83	17-Trifluoromethylphenyltrinorprostaglandin F2.alpha. (NIST) [Smart Confirmation]	70.7

257	137.1376 / 9.89	乙酸龙脑酯 Bomyl acetate [Smart Confirmation]	93.2
261	391.3407 / 10.29	.gamma.-Muricholic acid (NIST) [Smart Confirmation]	97.2
263	437.3462 / 10.29	Esfenvalerate (NIST) [Smart Confirmation]	64.7
266	195.1430 / 10.34	Sedanolide (NIST) [Smart Confirmation]	58.1
269	275.2117 / 10.34	17.alpha.-Nandrolone (NIST) [Smart Confirmation]	78.6
270	293.2221 / 10.34	9-Oxo-10E,12Z,15Z-octadecatrienoic acid (NIST) [Smart Confirmation]	91.4
271	311.2287 / 10.34	.alpha.-L-Glu-L-Tyr (NIST) [Smart Confirmation]	51.1
273	367.1934 / 10.34	Previtamin D3 (NIST) [Smart Confirmation]	54.9
293	595.3154 / 10.86	Pelargonidin 3,5-diglucoside cation (NIST) [Smart Confirmation]	21.0
304	309.2117 / 11.03	8Z,14Z-Eicosadienoic acid (NIST) [Smart Confirmation]	28.3
311	195.1427 / 11.20	Sedanolide (NIST) [Smart Confirmation]	52.3
314	389.3274 / 11.20	Methyl deoxycholate (NIST) [Smart Confirmation]	97.5
315	407.3386 / 11.20	Unoprostone isopropyl ester (NIST) [Smart Confirmation]	45.8
318	746.4889 / 11.25	1-Hexadecyl-2-(9Z-octadecenoyl)-sn-glycero-3-phosphocholine (NIST) [Smart Confirmation]	49.8
323	181.1297 / 11.59	4-Hydroxyphenylpyruvic acid (NIST) [Smart Confirmation]	76.2
324	391.3432 / 11.59	.gamma.-Muricholic acid (NIST) [Smart Confirmation]	79.3
327	419.3413 / 11.59	5.alpha.-Hydroxy-6-ketocholesterol (NIST) [Smart Confirmation]	30.5
332	291.2017 / 11.71	5.beta.-Dihydrotestosterone (NIST) [Smart Confirmation]	35.7
334	730.4933 / 11.71	G0-2AB (NIST) [Smart Confirmation]	77.7
336	428.2737 / 11.76	Adenosine 5'-diphosphate (NIST) [Smart Confirmation]	75.0
347	439.3265 / 11.99	17-Trifluoromethylphenyltrinorprostaglandin F2.alpha. (NIST) [Smart Confirmation]	80.2
352	373.1373 / 12.11	10-姜酚 10-Gingerol +Na [Smart Confirmation]	29.6
357	273.1906 / 12.22	4-Methoxytriphenylmethyl cation (NIST) [Smart Confirmation]	100.0
358	291.2039 / 12.22	5.beta.-Dihydrotestosterone (NIST) [Smart Confirmation]	35.7
360	389.3271 / 12.22	Methyl deoxycholate (NIST) [Smart Confirmation]	77.6
362	425.3462 / 12.22	Cyclopentaneheptanoic acid, 2-(3R)-4-(2-fluorophenyl)thio-3-hydroxybutyl-3,5-dihydroxy-, methyl ester, (1R,2R,3R,5S)- (NIST) [Smart Confirmation]	75.3
368	498.4000 / 12.33	Microcystin LR (NIST) [Smart Confirmation]	76.5
371	283.1061 / 12.50	5,7-Dimethoxyflavone (NIST) [Smart Confirmation]	81.3
376	540.4138 / 12.56	Nintedanib (NIST) [Smart Confirmation]	72.4
379	313.1138 / 12.62	Minaprine, lactame (NIST) [Smart Confirmation]	70.7
380	355.2683 / 12.73	13,14-Dihydro-15-ketoprostaglandin D1 (NIST) [Smart Confirmation]	25.4
381	391.3422 / 12.73 [M+H]+	(R)-Butaprost (NIST) [Smart Confirmation]	23.2
382	423.3679 / 12.45 [M+CH3OH+H]+	1,2-Didecanoyl-sn-glycerol (NIST) [Smart Confirmation]	83.6
383	401.3255 / 12.73	7-Oxocholesterol (NIST) [Smart Confirmation]	22.1
385	419.3446 / 12.73	5.alpha.-Hydroxy-6-ketocholesterol (NIST) [Smart Confirmation]	43.4
391	337.2580 / 12.79	Fluoxymesterone (NIST) [Smart Confirmation]	25.9
404	343.1245 / 13.13	Scutellarein tetramethyl ether (NIST) [Smart Confirmation]	37.9
409	271.1494 / 13.30	Estra-1,3,5(10),7-tetraene-3,17.beta.-diol (NIST) [Smart Confirmation]	34.2
417	405.3573 / 13.47	Lovastatin hydroxy acid (NIST) [Smart Confirmation]	97.7
421	453.3436 / 13.64	Met(O)-Met-Arg (NIST) [Smart Confirmation]	20.3
428	554.4237 / 13.70 [M+H]+	3,5,9-Trioxa-4-phosphatetracosan-1-aminium, 7-(acetyloxy)-24-carboxy-4-hydroxy-N,N,N-trimethyl-, inner salt, 4-oxide, (R)- (NIST) [Smart Confirmation]	27.6

431	195.1426 / 13.81	7,8-Dihydro-.alpha.-ionone (NIST) [Smart Confirmation]	78.9
432	199.1381 / 13.81	.gamma.-Dodecalactone (NIST) [Smart Confirmation]	33.6
433	275.2088 / 13.81	17.alpha.-Nandrolone (NIST) [Smart Confirmation]	59.2
434	293.2187 / 13.81	Picrotoxinin (NIST) [Smart Confirmation]	45.5
438	337.2582 / 13.98	Fluoxymesterone (NIST) [Smart Confirmation]	27.1
439	391.3411 / 13.98	.gamma.-Muricholic acid (NIST) [Smart Confirmation]	53.2
442	441.3775 / 13.98 [M+CH3OH+H]+	Soyasapogenol B (NIST) [Smart Confirmation]	60.9
446	621.4403 / 13.98	Ginsenoside Rh1 (NIST) [Smart Confirmation]	60.5
456	668.4407 / 14.21	长梗冬青苷 Pedunculoside +NH3 [Smart Confirmation]	50.1
459	407.3353 / 14.32 [M+H]+	7-Keto-3.alpha.,12-.alpha.-dihydroxycholanic acid (NIST) [Smart Confirmation]	20.9
460	353.2772 / 14.38	Monolinolenin (9c,12c,15c) (NIST) [Smart Confirmation]	76.8
461	421.3510 / 14.38	Ouabagenin (NIST) [Smart Confirmation]	71.3
465	291.2028 / 14.44	5.alpha.-Androsterone (NIST) [Smart Confirmation]	33.9
477	293.2189 / 14.83	Cyclopentanehexanoic acid, 2-(2Z)-4-carboxy-2-buten-1-yl-3,5-dihydroxy-.gamma.-oxo-, (1R,2R,3S,5S)- (NIST) [Smart Confirmation]	49.1
482	236.1122 / 15.06	D,L-N,N-Didesmethyl-O-desmethylvenlafaxine (NIST) [Smart Confirmation]	29.8
483	309.2623 / 15.12	Heptaethylene glycol (NIST) [Smart Confirmation]	28.8
484	391.3427 / 15.12 [M+H]+	.gamma.-Muricholic acid (NIST) [Smart Confirmation]	49.0
485	423.3673 / 14.72 [M+CH3OH+H]+	1,2-Didecanoyl-sn-glycerol (NIST) [Smart Confirmation]	58.8
488	419.3409 / 15.12	5.alpha.-Hydroxy-6-ketocholesterol (NIST) [Smart Confirmation]	23.7
490	455.3593 / 15.12	路路通酸 Betulonicacid [Smart Confirmation]	55.3
494	279.2285 / 15.29	Oxymetazoline impurity A (NIST) [Smart Confirmation]	77.1
497	439.3605 / 15.35	17-Trifluoromethylphenyltrinorprostaglandin F2.alpha. (NIST) [Smart Confirmation]	100.0
499	277.2312 / 15.40	Stearidonic acid (NIST) [Smart Confirmation]	96.4
503	219.1802 / 15.69	5-Sulfosalicylic acid (NIST) [Smart Confirmation]	98.5
505	261.2277 / 15.74	Oxymetazoline (NIST) [Smart Confirmation]	42.7
506	335.2635 / 15.74	9,11-Methane-epoxyprostaglandin F1.alpha. (NIST) [Smart Confirmation]	74.7
507	353.2820 / 15.74	Monolinolenin (9c,12c,15c) (NIST) [Smart Confirmation]	96.8
508	497.3167 / 15.74	菊苣酸 Cichoric acid +Na [Smart Confirmation]	63.8
511	275.2092 / 15.91	5.beta.-Androstane-3.alpha.,17.beta.-diol (NIST) [Smart Confirmation]	50.2
512	293.2237 / 15.91	9-Oxo-10E,12Z,15Z-octadecatrienoic acid (NIST) [Smart Confirmation]	41.7
513	341.3109 / 15.97	(+)-Pinoresinol (NIST) [Smart Confirmation]	90.6
517	203.1843 / 16.14	Bergaptol (NIST) [Smart Confirmation]	37.8
518	391.3438 / 16.14	Digoxigenin (NIST) [Smart Confirmation]	30.2
521	419.3442 / 16.14	5.alpha.-Hydroxy-6-ketocholesterol (NIST) [Smart Confirmation]	25.2
522	455.3604 / 16.14	路路通酸 Betulonicacid [Smart Confirmation]	55.5
530	365.2737 / 16.42	Nigerose (NIST) [Smart Confirmation]	74.4
531	311.2634 / 16.48	Avobenzone (NIST) [Smart Confirmation]	77.8
533	423.3667 / 16.54	1,2-Didecanoyl-sn-glycerol (NIST) [Smart Confirmation]	30.1
534	447.2565 / 16.54	Cefuroxime (NIST) [Smart Confirmation]	75.1
539	179.1481 / 16.60	3,4-Methylenedioxyphenylacetone (NIST) [Smart Confirmation]	26.4
540	277.2256 / 16.60	6-Gingerol (NIST) [Smart Confirmation]	98.1
541	295.2359 / 16.60	13-Keto-9Z,11E-octadecadienoic acid (NIST) [Smart Confirmation]	77.6

542	454.2976 / 16.77	1-Palmitoyl-2-hydroxy-sn-glycero-3-phosphoethanolamine (NIST) [Smart Confirmation]	82.3
545	423.3201 / 16.82	1,2-Didecanoyl-sn-glycerol (NIST) [Smart Confirmation]	25.4
547	305.2533 / 16.88	.omega.-3 Arachidonic acid (NIST) [Smart Confirmation]	90.9
548	653.3003 / 16.88	Leiocarposide (NIST) [Smart Confirmation]	98.6
563	453.3428 / 17.16	Met(O)-Met-Arg (NIST) [Smart Confirmation]	24.6
568	338.3514 / 17.28	Erucamide (NIST) [Smart Confirmation]	90.7
576	359.3228 / 17.39	1-Stearoyl-rac-glycerol (NIST) [Smart Confirmation]	93.2
580	391.3412 / 17.45	.gamma.-Muricholic acid (NIST) [Smart Confirmation]	81.9
582	419.3373 / 17.45	5.alpha.-Hydroxy-6-ketocholesterol (NIST) [Smart Confirmation]	43.2
584	353.2759 / 17.50	Monolinolenin (9c,12c,15c) (NIST) [Smart Confirmation]	91.0
585	599.4148 / 17.50	Phe-Met-Arg-Phe-amide (NIST) [Smart Confirmation]	33.2
588	367.2695 / 17.56	Diacetoxyscirpenol (NIST) [Smart Confirmation]	57.7
589	609.4562 / 17.56	1-(1,2-Dioctanoylphosphatidyl)inositol (NIST) [Smart Confirmation]	88.9
594	667.3163 / 17.62	Syrosingopine (NIST) [Smart Confirmation]	21.1
595	615.4079 / 17.68	16,16-Dimethylprostaglandin E2 p-(p-acetamidobenzamido) phenyl ester (NIST) [Smart Confirmation]	73.8
596	623.2557 / 17.68	6,10,11-Triethylcarbonate-1-demethyl daunomycinone (NIST) [Smart Confirmation]	30.4
599	375.2739 / 17.73	6.alpha.-Methylprednisolone (NIST) [Smart Confirmation]	62.5
602	280.2434 / 17.79	Cidofovir (NIST) [Smart Confirmation]	20.2
607	279.2414 / 17.85	Pinolenic acid (NIST) [Smart Confirmation]	61.6
608	734.5957 / 17.85	1,2-Dihexadecanoyl-sn-glycero-3-phosphocholine (NIST) [Smart Confirmation]	53.2
616	583.4185 / 18.19	Geldanamycin (NIST) [Smart Confirmation]	90.8
625	419.3381 / 18.36	5.alpha.-Hydroxy-6-ketocholesterol (NIST) [Smart Confirmation]	55.4
634	439.3618 / 18.53	.alpha.,.alpha.'-Dilaurin (NIST) [Smart Confirmation]	93.3
642	429.3781 / 18.70	17-Phenoxyprostaglandin F2.alpha. isopropyl ester (NIST) [Smart Confirmation]	95.0
644	344.3376 / 18.81	Lauroyl-L-carnitine (NIST) [Smart Confirmation]	72.9
649	293.2537 / 18.93	9Z,11E,13E-Octadecatrienoic acid methyl ester (NIST) [Smart Confirmation]	87.2
655	277.2237 / 19.04	9,12-Octadecadiynoic acid (NIST) [Smart Confirmation]	97.7
658	553.4305 / 19.04 [M+NH4]+	1-Stearoyl-2-hydroxy-sn-glycero-3-phosphate (NIST) [Smart Confirmation]	64.3
661	309.2898 / 19.10	8Z,14Z-Eicosadienoic acid (NIST) [Smart Confirmation]	76.6
662	351.2953 / 19.10	15(R),19(R)-Hydroxyprostaglandin E2 (NIST) [Smart Confirmation]	100.0
663	567.4237 / 19.10	Mesoporphyrin IX (NIST) [Smart Confirmation]	23.5
668	311.2415 / 19.15	Picrotin (NIST) [Smart Confirmation]	64.9
671	565.2491 / 19.21	Cornuside (NIST) [Smart Confirmation]	80.8
679	535.2756 / 19.32	Kahweol palmitate (NIST) [Smart Confirmation]	80.5
680	584.4255 / 19.32	Isoglobotriaose-.beta.-N(Acetyl)-Propargyl (NIST) [Smart Confirmation]	78.9

End of Table

104.1161 / 1.02 (Mass/FragMass/RT/Isotope/Library/Formula/Ion Ratio)

**Retention Time:** 1.07 minutes	**Exp RT:** 1.02 minutes
**Precursor m/z :** 104.1161	**Analyte Name:**
**Fit (%)** 96.6% **RFit (%)** 100.0%	104.1161 / 1.02

Collision Energy = 35 ± 15 eV

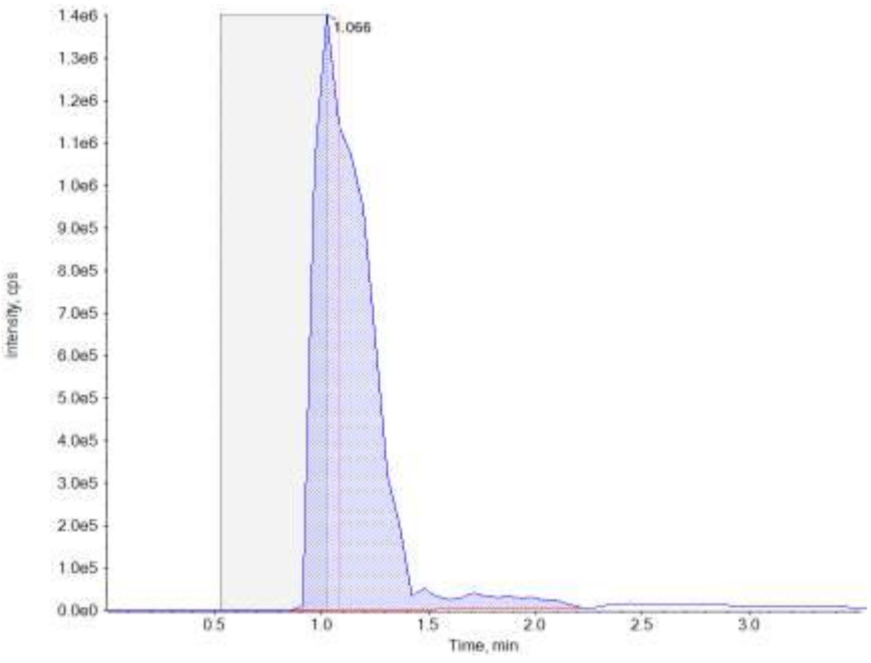

Acquired / Library MSMS

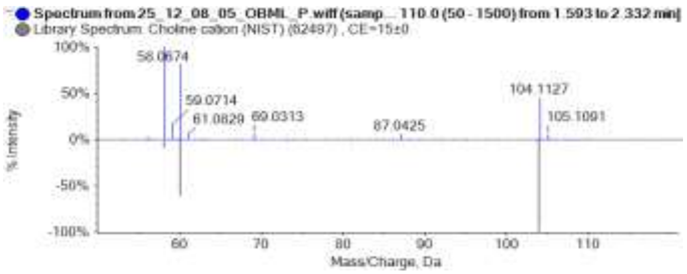

Acquired / Theoretical MS

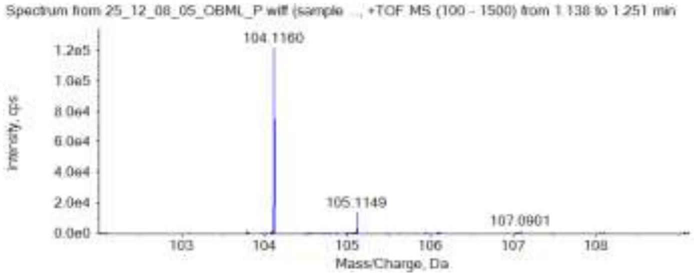

116.0750 / 1.02 (Mass/FragMass/RT/Isotope/Library/Formula/Ion Ratio)

Retention Time: 1.05 minutes
Precursor m/z : 116.0750
Fit (%) 98.5% **RFit (%)** 56.4%

Exp RT: 1.02 minutes
Analyte Name:
116.0750 / 1.02

Collision Energy = 35 ± 15 eV

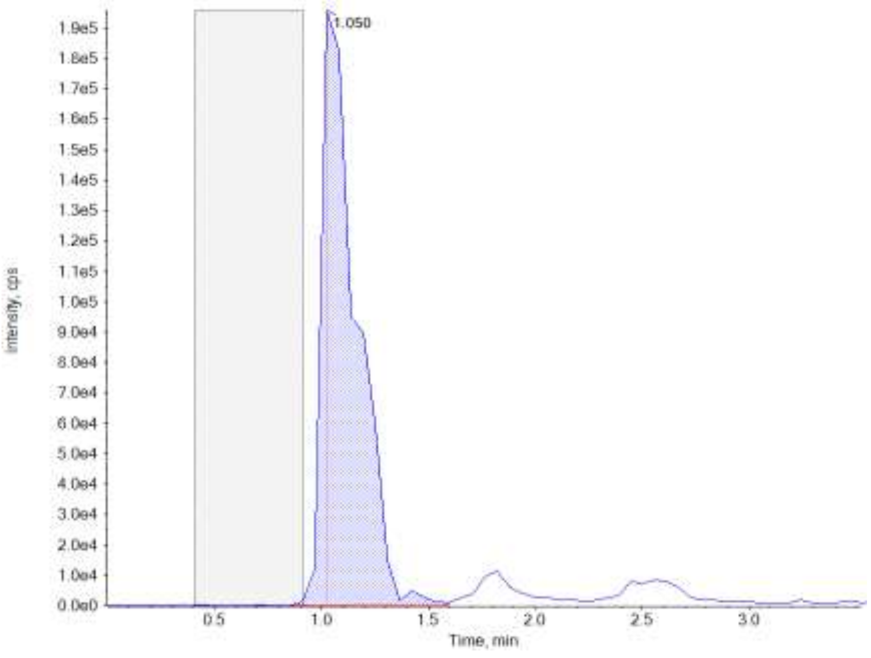

Acquired / Library MSMS

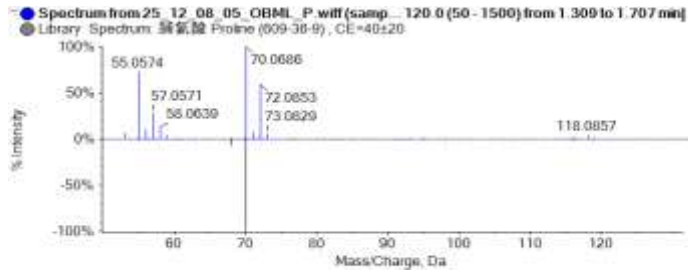

Acquired / Theoretical MS

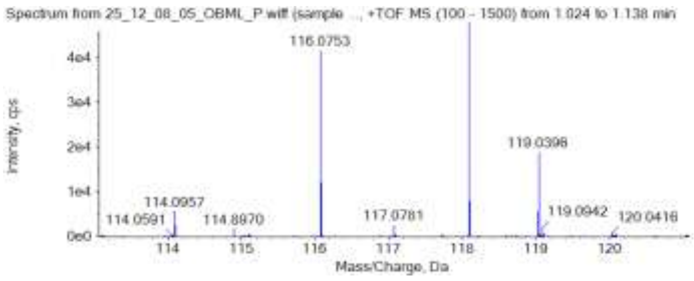

118.0908 / 1.02 (Mass/FragMass/RT/Isotope/Library/Formula/Ion Ratio)

**Retention Time:** 1.05 minutes	**Exp RT:** 1.02 minutes
**Precursor m/z :** 118.0908	**Analyte Name:**
**Fit (%)** 86.3% **RFit (%)** 87.6%	118.0908 / 1.02

Collision Energy = 35 ± 15 eV

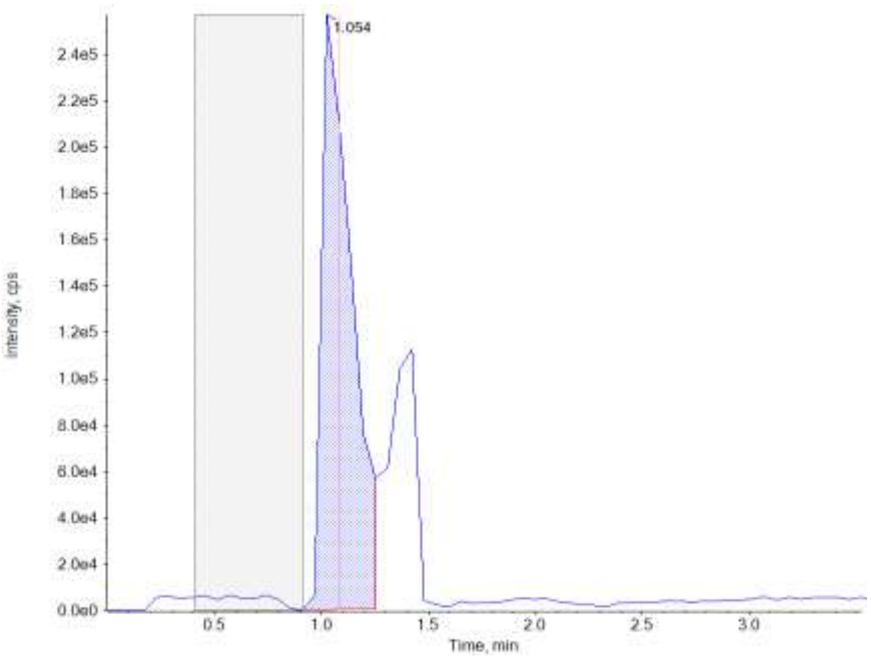

Acquired / Library MSMS

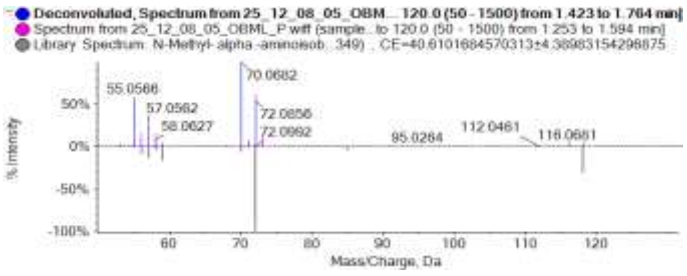

Acquired / Theoretical MS

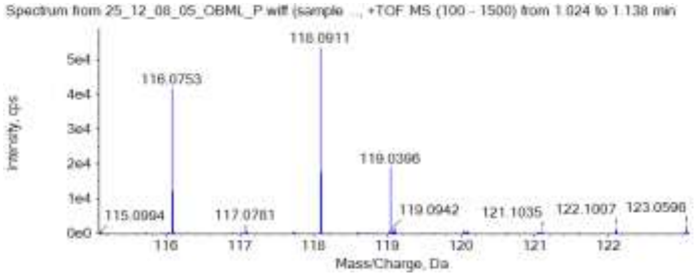

136.0725 / 1.02 (Mass/FragMass/RT/Isotope/Library/Formula/Ion Ratio)

**Retention Time:** 1.05 minutes	**Exp RT:** 1.02 minutes
**Precursor m/z :** 136.0725	**Analyte Name:**
**Fit (%)** 99.1% **RFit (%)** 85.0%	136.0725 / 1.02

Collision Energy = 35 ± 15 eV

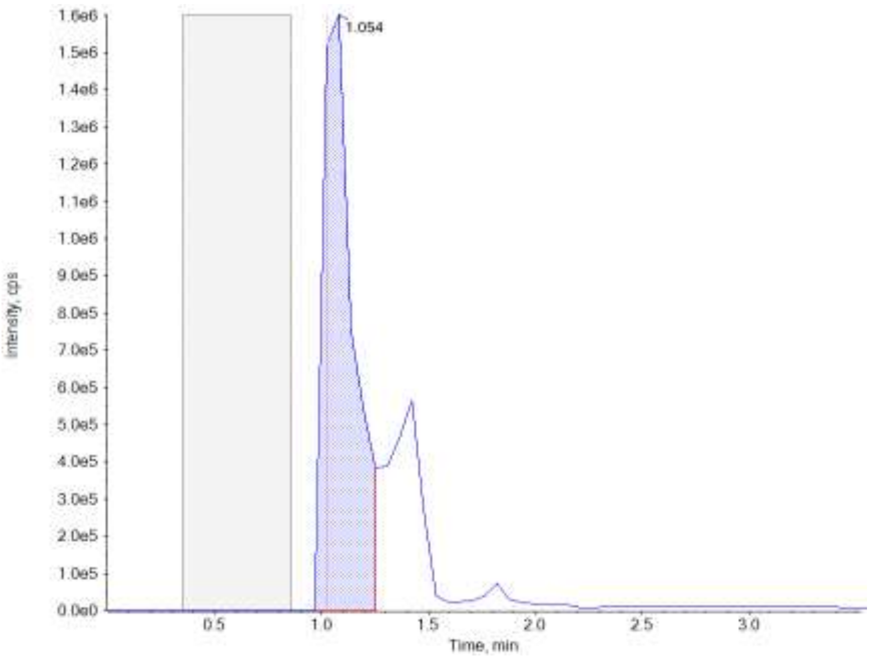

Acquired / Library MSMS

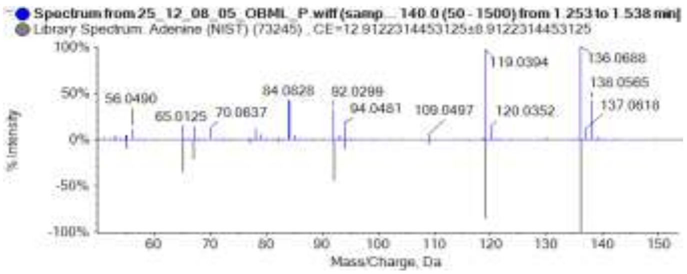

Acquired / Theoretical MS

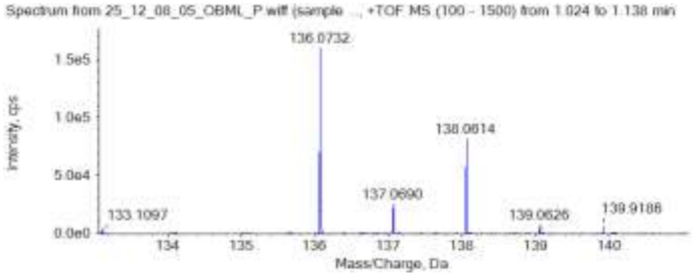

381.0854 / 1.14 (Mass/FragMass/RT/Isotope/Library/Formula/Ion Ratio)

**Retention Time:** 1.09 minutes	**Exp RT:** 1.14 minutes
**Precursor m/z :** 381.0854	**Analyte Name:**
**Fit (%)** 93.2% **RFit (%)** 94.1%	381.0854 / 1.14

Collision Energy = 35 ± 15 eV

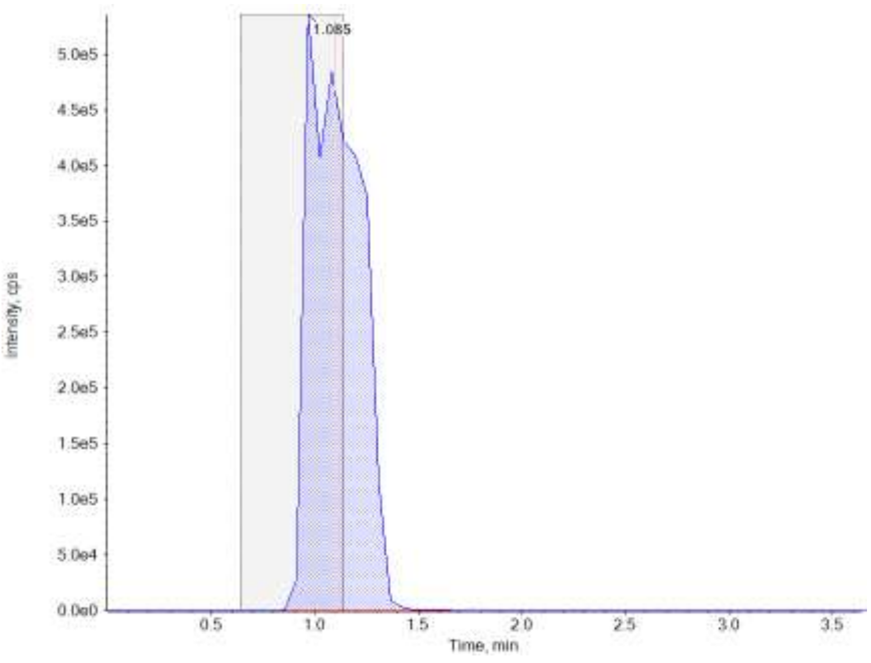

Acquired / Library MSMS

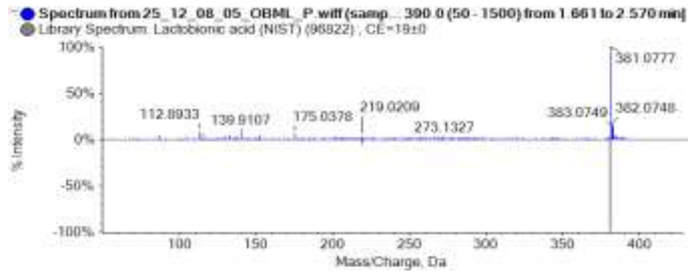

Acquired / Theoretical MS

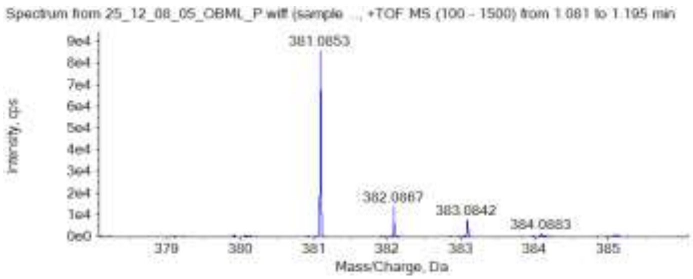

Created with SCIEX OS 3.3.0
Printed:12/9/2025 2:05:30 PM

337.0945 / 1.37 (Mass/FragMass/RT/Isotope/Library/Formula/Ion Ratio)

Retention Time: 1.31 minutes
Precursor m/z : 337.0945
Fit (%) 100.0% **RFit (%)** 84.3%

Exp RT: 1.37 minutes
Analyte Name:
337.0945 / 1.37

Collision Energy = 35 ± 15 eV

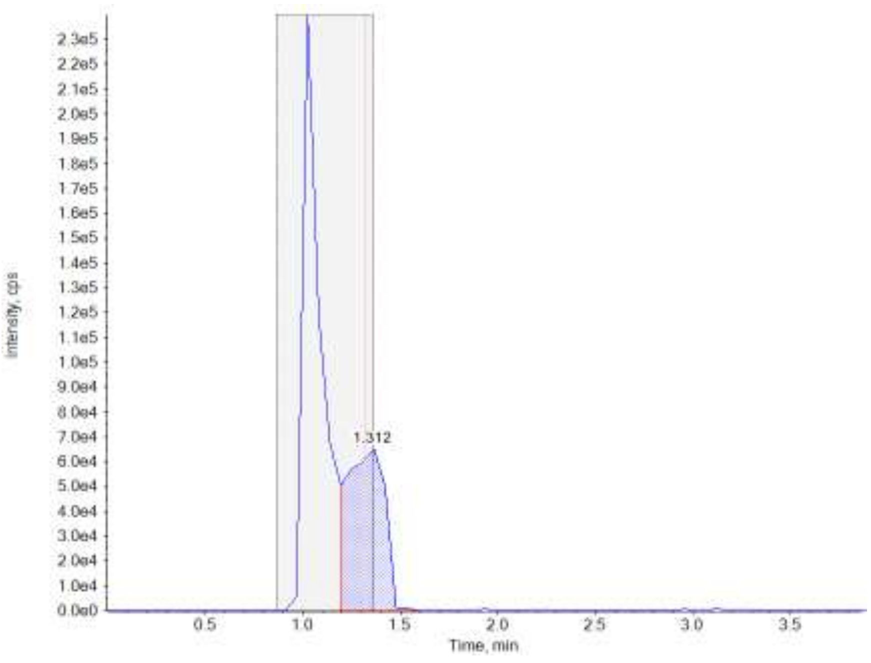

Acquired / Library MSMS

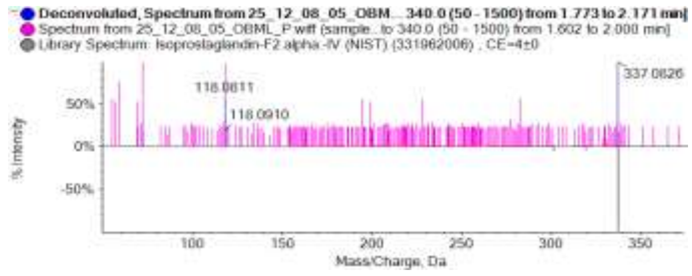

Acquired / Theoretical MS

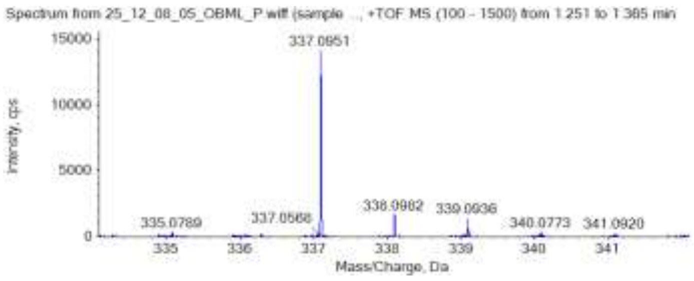

262.1396 / 1.42 [M+H]+

(Mass/FragMass/RT/Isotope/Library/Formula/Ion Ratio)

Retention Time: 1.42 minutes
Precursor m/z : 262.1396
Fit (%) 58.6% **RFit (%)** 90.7%

Exp RT: 1.42 minutes
Analyte Name:
262.1396 / 1.42 [M+H]+

Collision Energy = 35 ± 15 eV

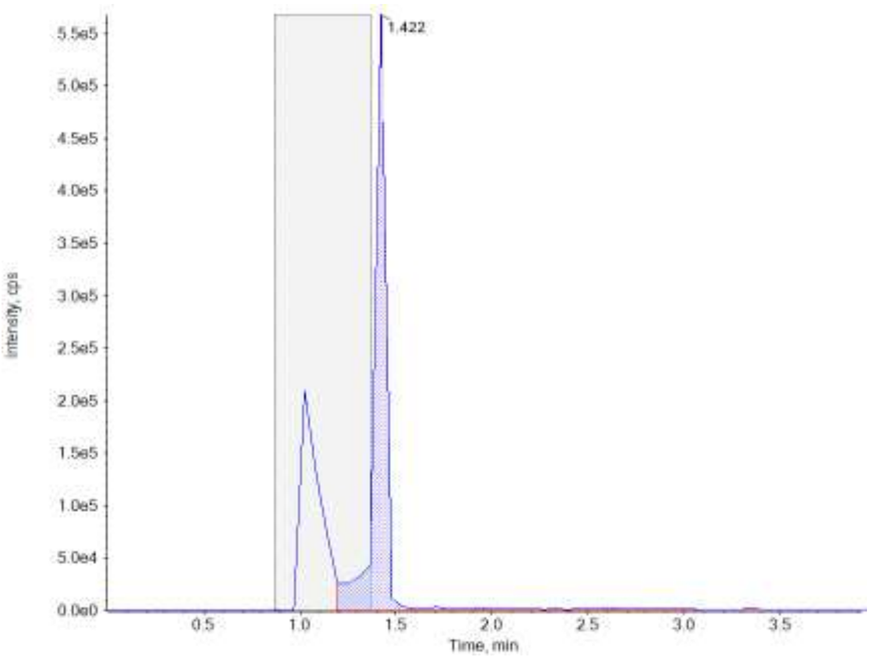

Acquired / Library MSMS

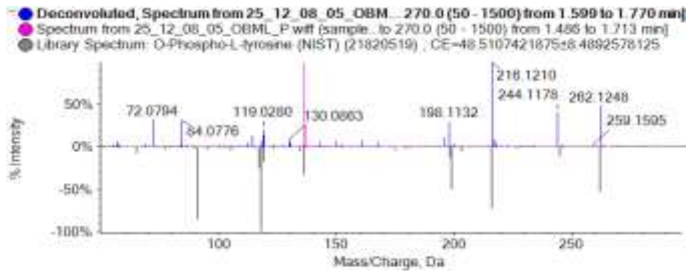

Acquired / Theoretical MS

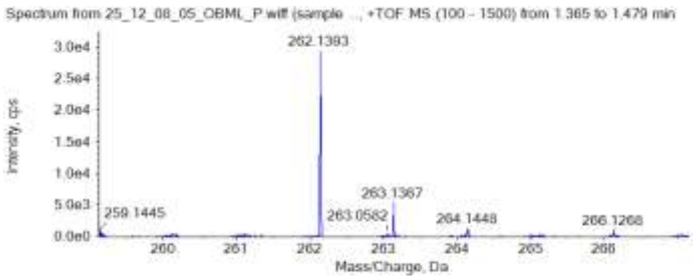

152.0636 / 1.48

(Mass/FragMass/RT/Isotope/Library/Formula/Ion Ratio)

Retention Time: 1.50 minutes
Precursor m/z : 152.0636
Fit (%) 100.0% **RFit (%)** 81.5%

Exp RT: 1.48 minutes
Analyte Name:
152.0636 / 1.48

Collision Energy = 35 ± 15 eV

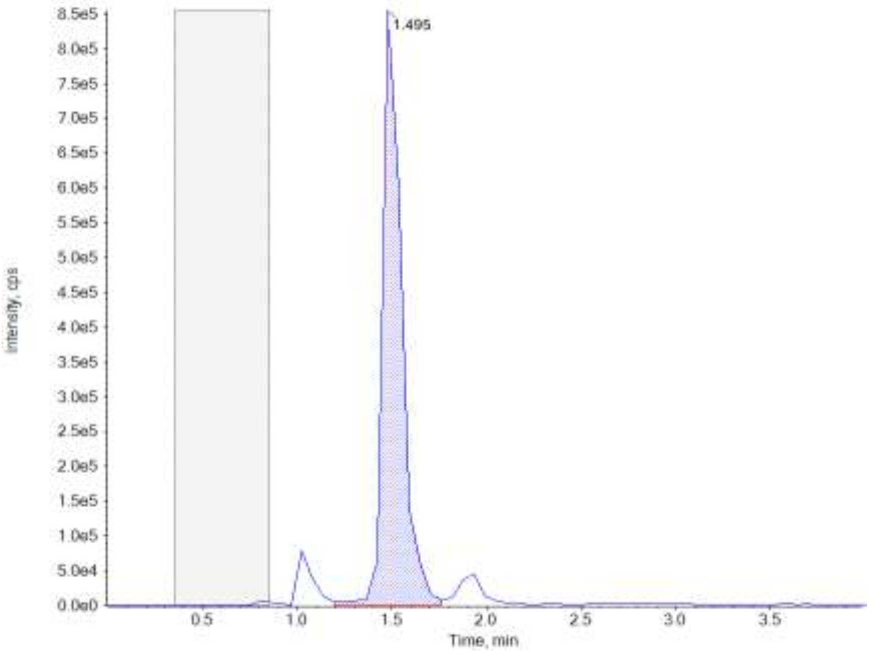

Acquired / Library MSMS

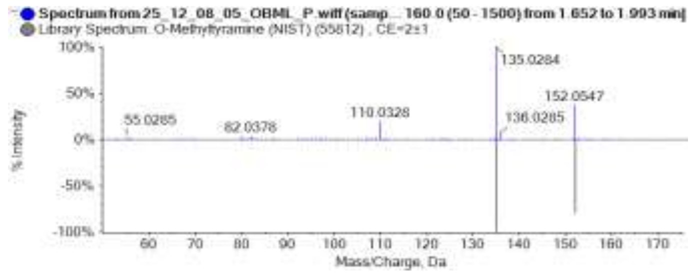

Acquired / Theoretical MS

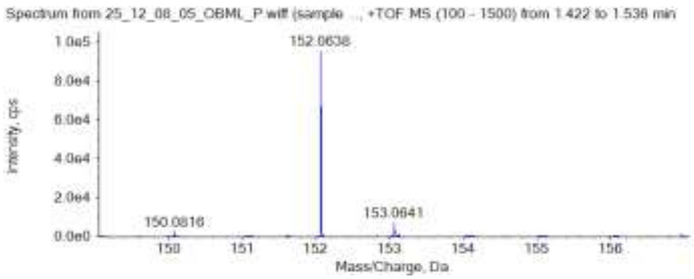

182.0869 / 1.48 (Mass/FragMass/RT/Isotope/Library/Formula/Ion Ratio)

Retention Time: 1.48 minutes
Precursor m/z : 182.0869
Fit (%) 98.1% **RFit (%)** 99.7%

Exp RT: 1.48 minutes
Analyte Name:
182.0869 / 1.48

Collision Energy = 35 ± 15 eV

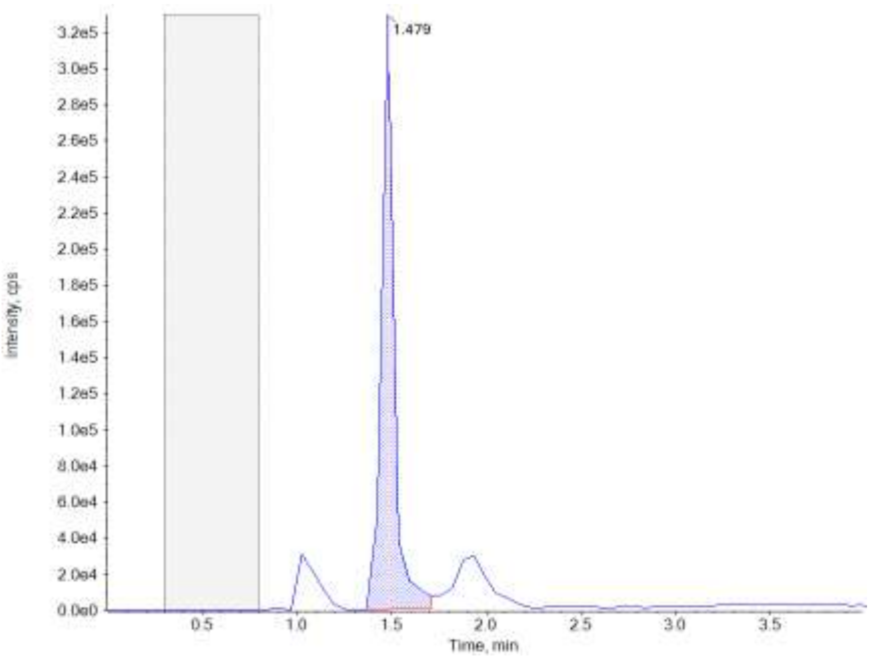

Acquired / Library MSMS

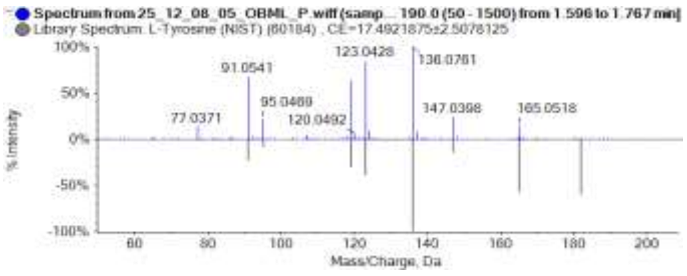

Acquired / Theoretical MS

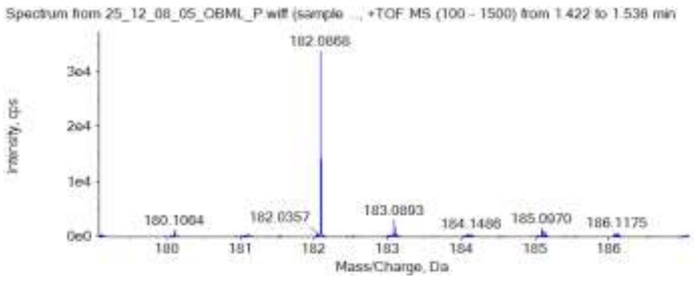

268.1102 / 1.48 [M]+ (Mass/FragMass/RT/Isotope/Library/Formula/Ion Ratio)

Retention Time: 1.48 minutes
Precursor m/z : 268.1102
Fit (%) 98.0% **RFit (%)** 91.8%

Exp RT: 1.48 minutes
Analyte Name:
268.1102 / 1.48 [M]+

Collision Energy = 35 ± 15 eV

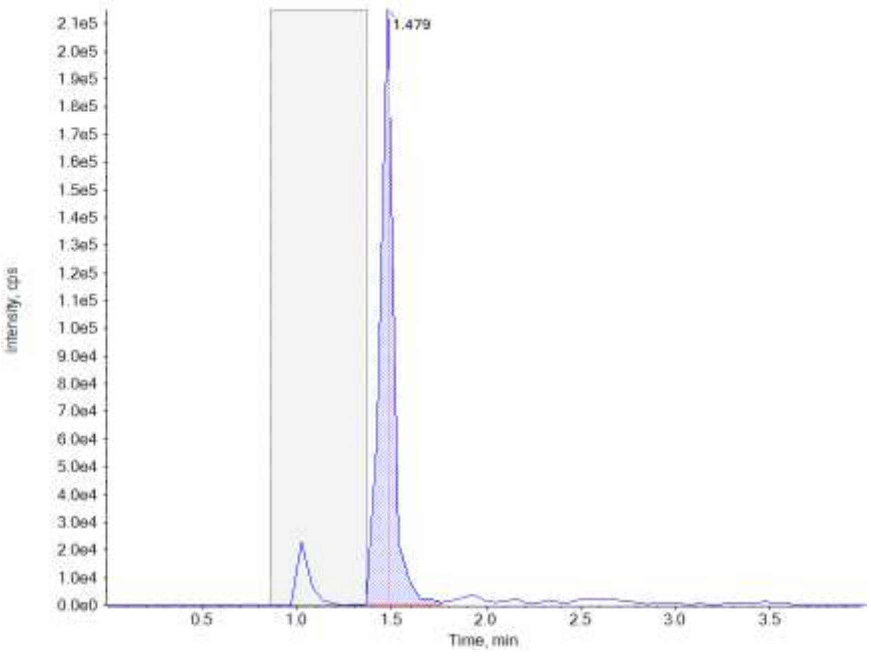

Acquired / Library MSMS

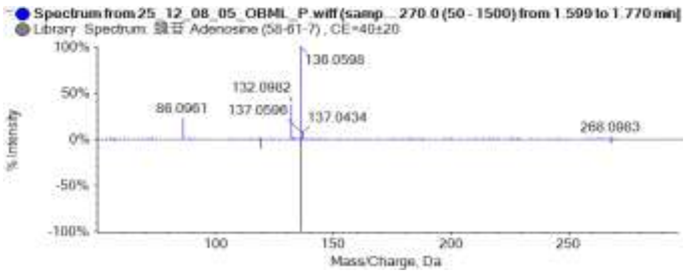

Acquired / Theoretical MS

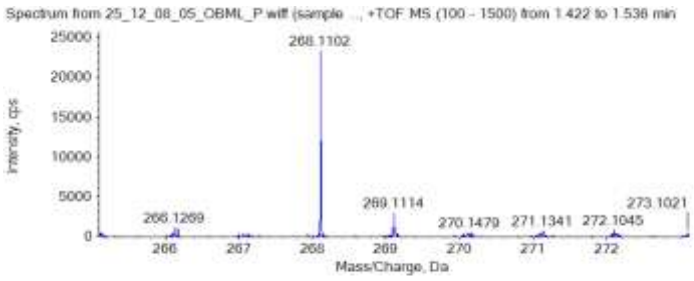

286.1454 / 1.76 [M+NH4]+ (Mass/FragMass/RT/Isotope/Library/Formula/Ion Ratio)

Retention Time: 1.77 minutes
Precursor m/z : 286.1454
Fit (%) 97.9% **RFit (%)** 98.2%

Exp RT: 1.76 minutes
Analyte Name:
286.1454 / 1.76 [M+NH4]+

Collision Energy = 35 ± 15 eV

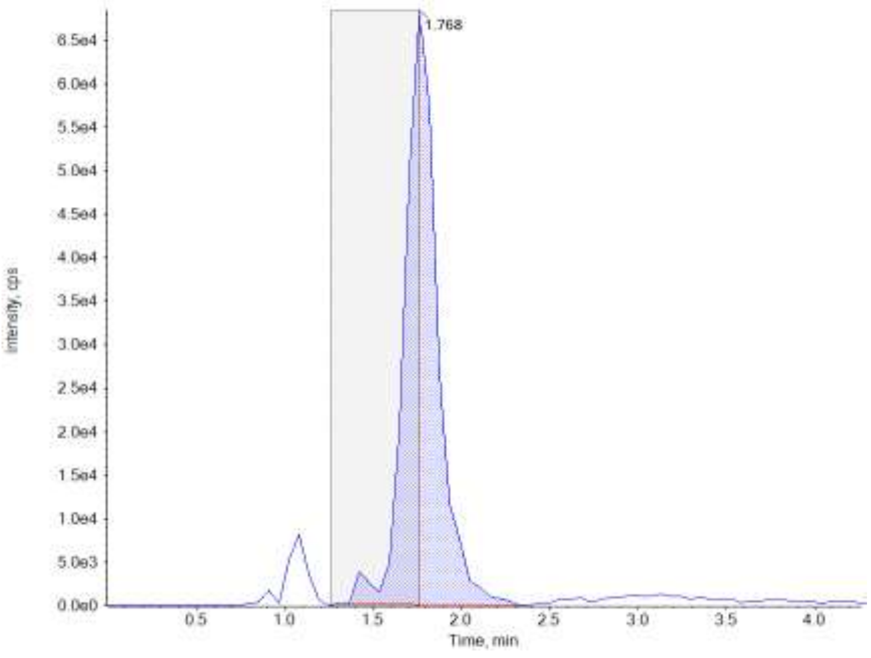

Acquired / Library MSMS

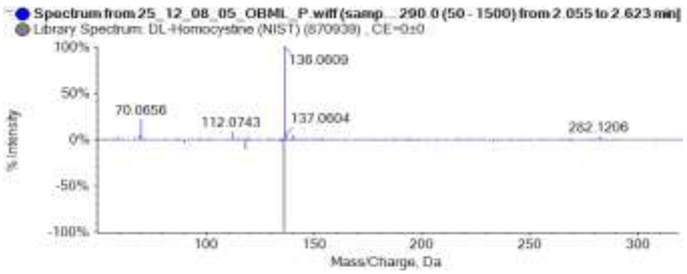

Acquired / Theoretical MS

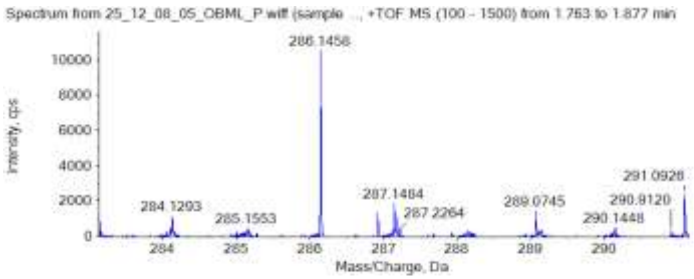

284.1065 / 1.48 [M+H]+ (Mass/FragMass/RT/Isotope/Library/Formula/Ion Ratio)

**Retention Time:** 1.50 minutes	**Exp RT:** 1.48 minutes
**Precursor m/z :** 284.1065	**Analyte Name:**
**Fit (%)** 100.0% **RFit (%)** 100.0%	284.1065 / 1.48 [M+H]+

Collision Energy = 35 ± 15 eV

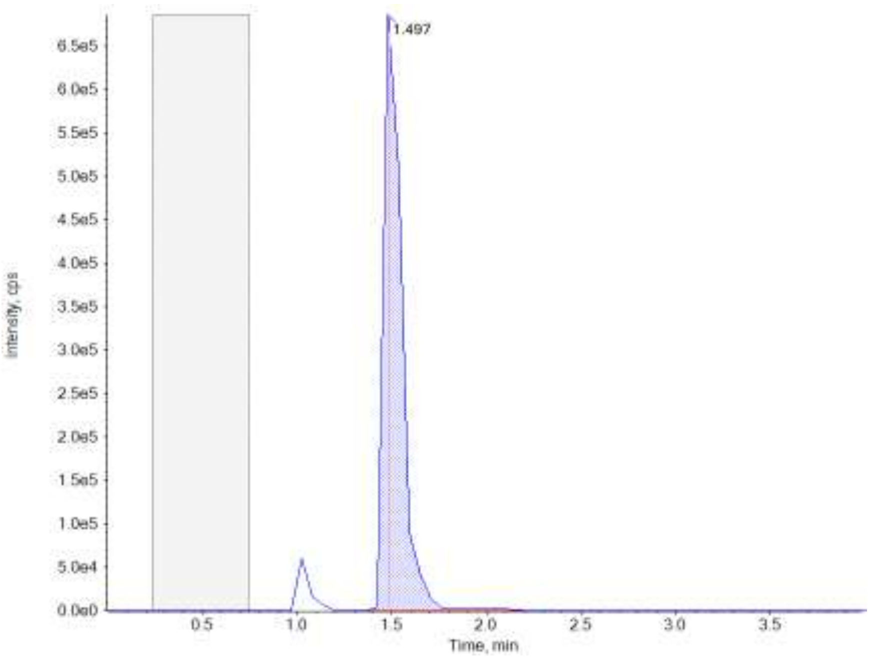

Acquired / Library MSMS

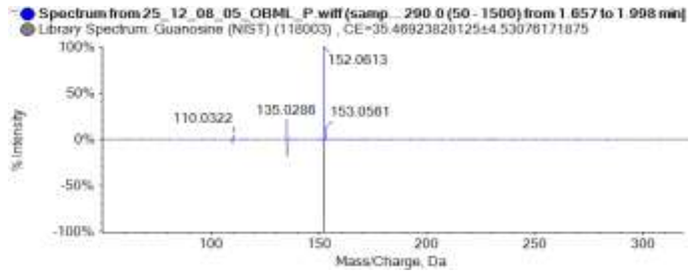

Acquired / Theoretical MS

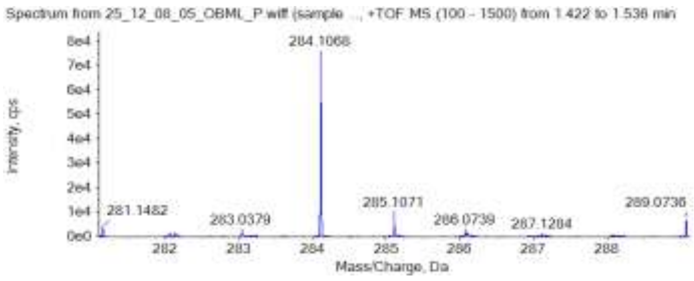

322.0612 / 1.54 [M+K]+ (Mass/FragMass/RT/Isotope/Library/Formula/Ion Ratio)

Retention Time: 1.51 minutes
Precursor m/z : 322.0611
Fit (%) 100.0% **RFit (%)** 74.0%

Exp RT: 1.54 minutes
Analyte Name:
322.0612 / 1.54 [M+K]+

Collision Energy = 35 ± 15 eV

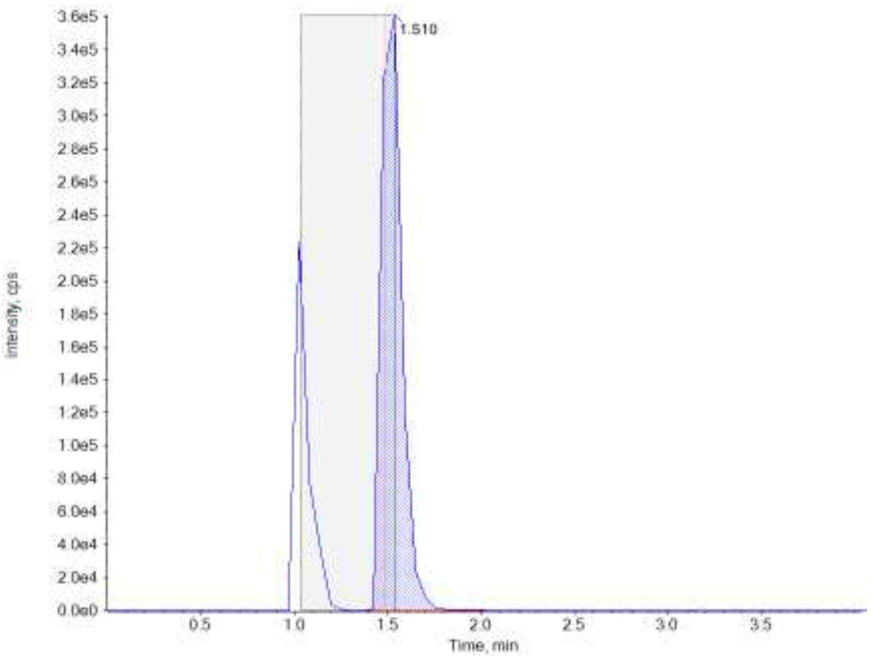

Acquired / Library MSMS

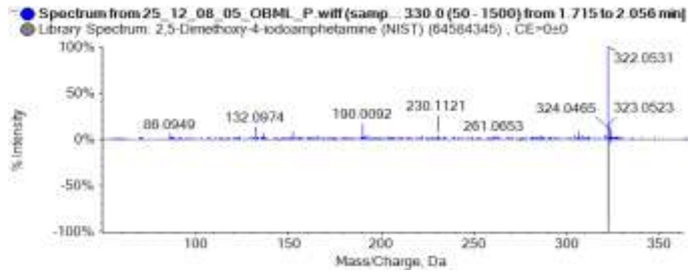

Acquired / Theoretical MS

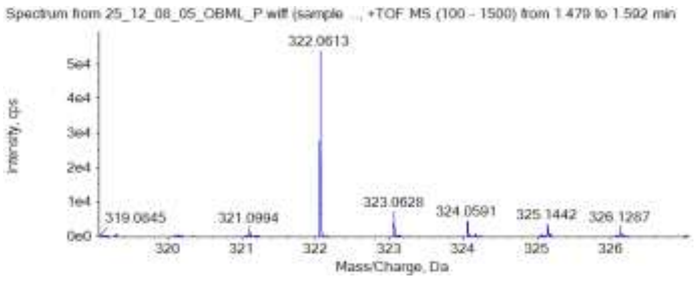

276.1546 / 1.54 (Mass/FragMass/RT/Isotope/Library/Formula/Ion Ratio)

Retention Time: 1.53 minutes
Precursor m/z : 276.1545
Fit (%) 96.0% **RFit (%)** 79.6%

Exp RT: 1.54 minutes
Analyte Name:
276.1546 / 1.54

Collision Energy = 35 ± 15 eV

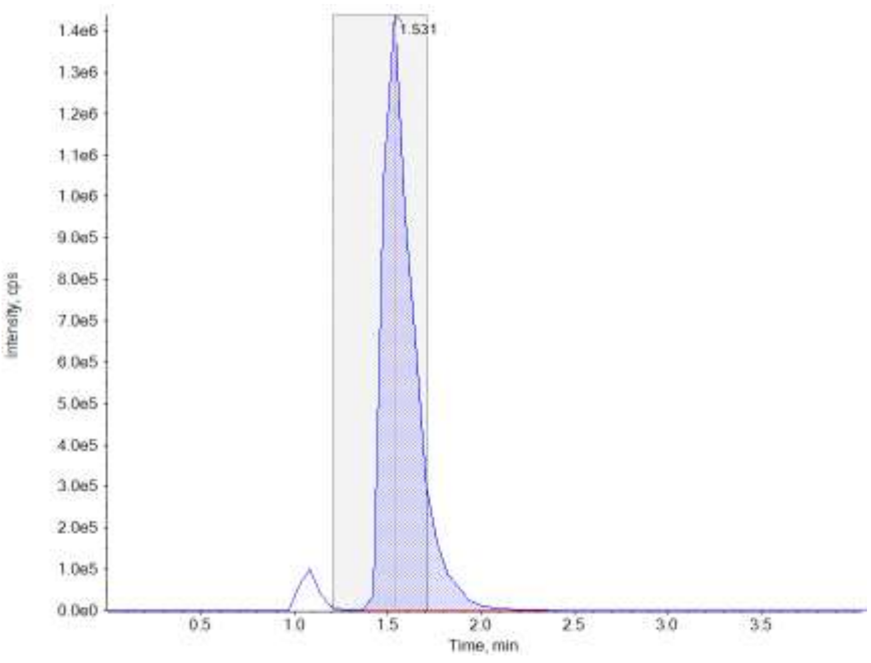

Acquired / Library MSMS

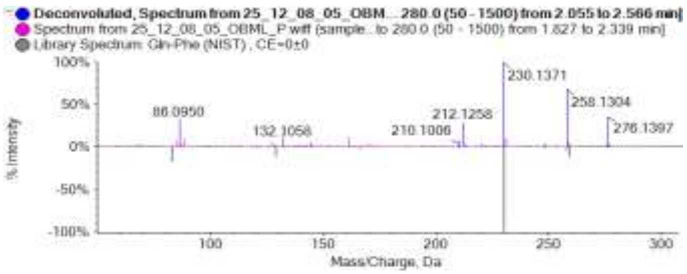

Acquired / Theoretical MS

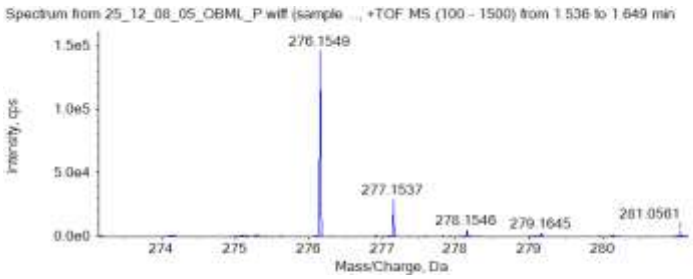

Created with SCIEX OS 3.3.0
Printed:12/9/2025 2:05:30 PM

332.1165 / 1.54 (Mass/FragMass/RT/Isotope/Library/Formula/Ion Ratio)

**Retention Time:** 1.55 minutes	**Exp RT:** 1.54 minutes
**Precursor m/z :** 332.1165	**Analyte Name:**
**Fit (%)** 100.0% **RFit (%)** 90.3%	332.1165 / 1.54

Collision Energy = 35 ± 15 eV

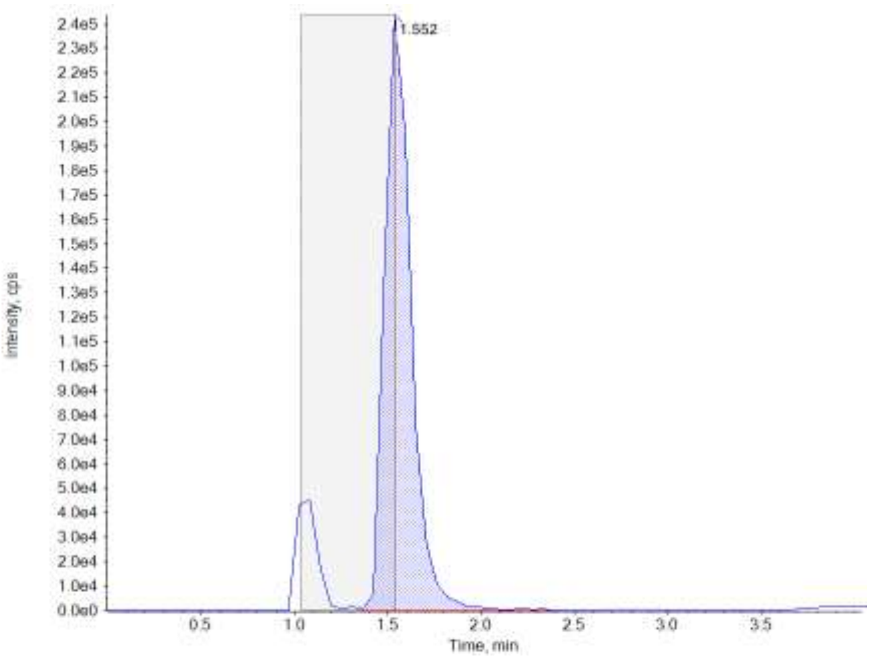

Acquired / Library MSMS

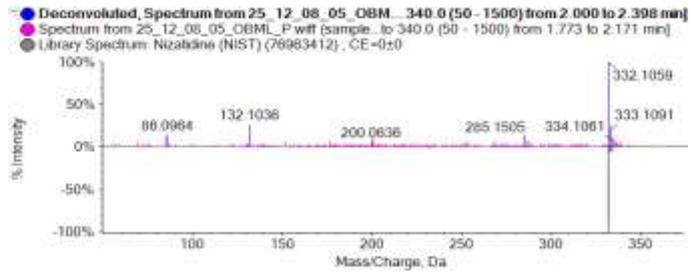

Acquired / Theoretical MS

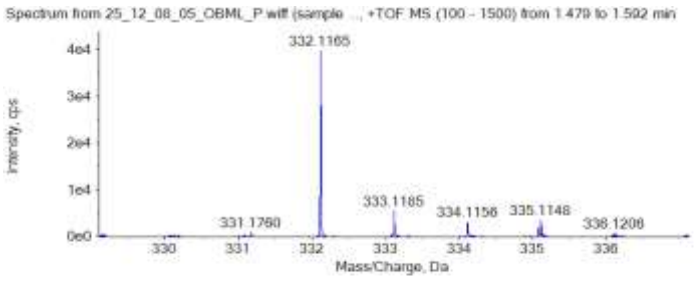

132.1118 / 1.59 (Mass/FragMass/RT/Isotope/Library/Formula/Ion Ratio)

Retention Time: 1.59 minutes | **Exp RT:** 1.59 minutes
Precursor m/z : 132.1118 | **Analyte Name:**
Fit (%) 100.0% **RFit (%)** 100.0% | 132.1118 / 1.59

Collision Energy = 35 ± 15 eV

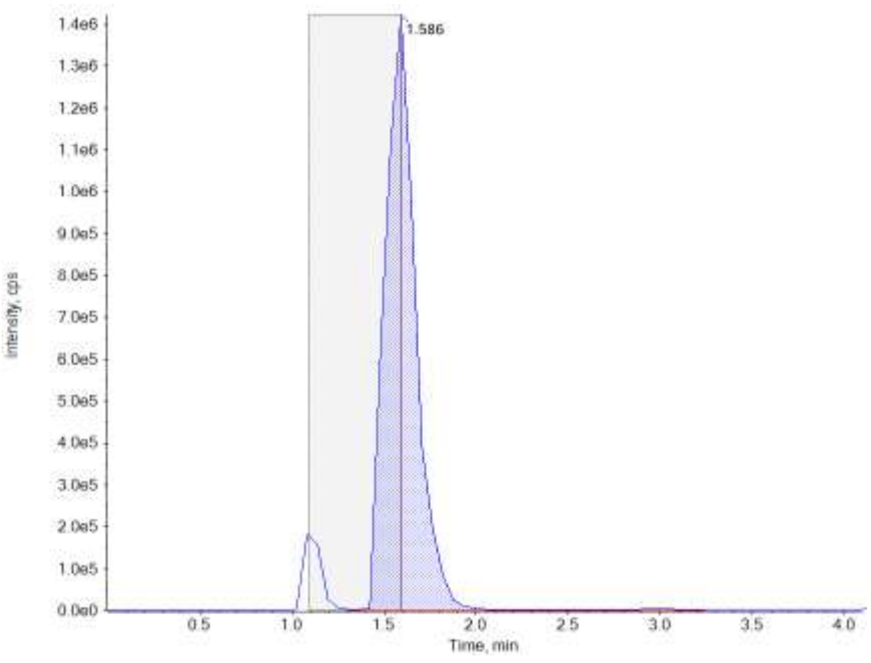

Acquired / Library MSMS

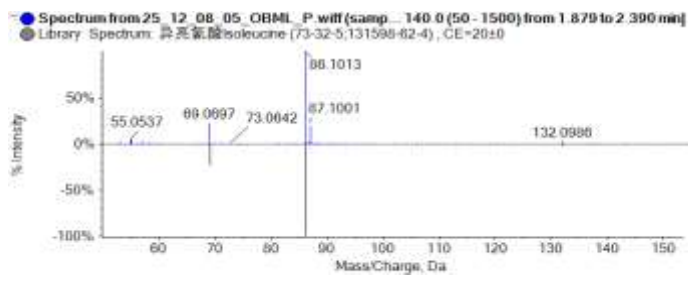

Acquired / Theoretical MS

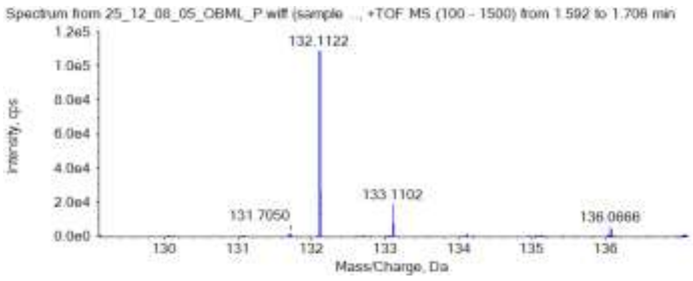

204.1281 / 1.82 (Mass/FragMass/RT/Isotope/Library/Formula/Ion Ratio)

Retention Time: 1.88 minutes
Precursor m/z : 204.1281
Fit (%) 100.0% **RFit (%)** 30.6%

Exp RT: 1.82 minutes
Analyte Name:
204.1281 / 1.82

Collision Energy = 35 ± 15 eV

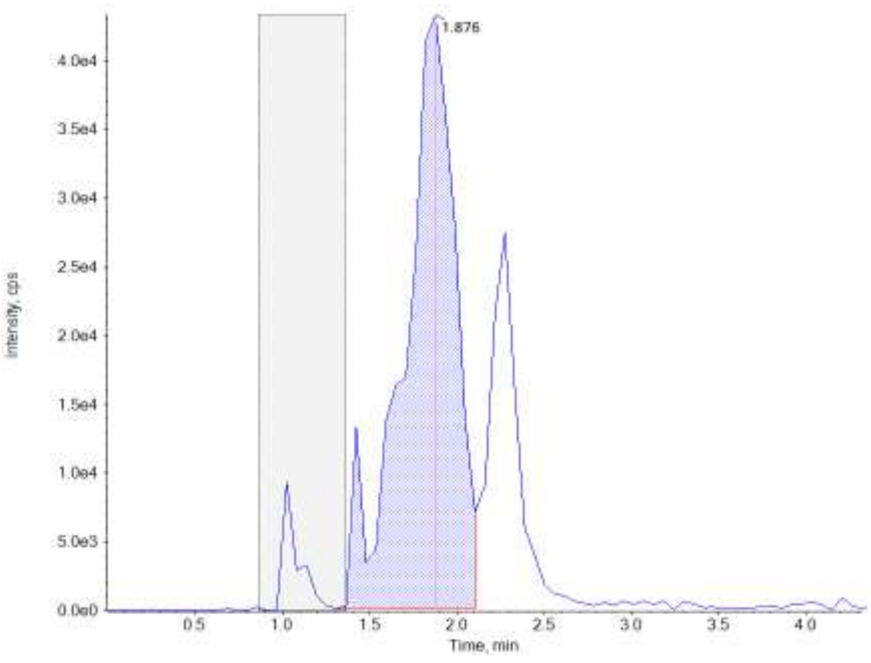

Acquired / Library MSMS

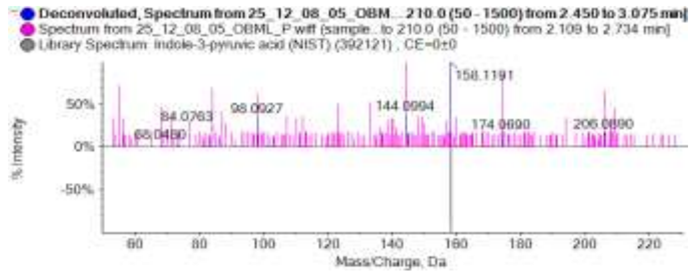

Acquired / Theoretical MS

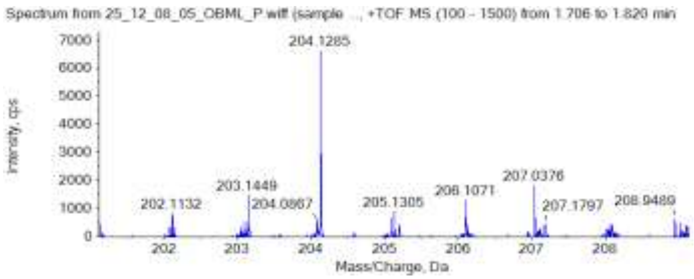

282.1284 / 1.82 (Mass/FragMass/RT/Isotope/Library/Formula/Ion Ratio)

Retention Time: 1.82 minutes **Exp RT:** 1.82 minutes
Precursor m/z : 282.1284 **Analyte Name:**
Fit (%) 96.8% **RFit (%)** 100.0% 282.1284 / 1.82

Collision Energy = 35 ± 15 eV

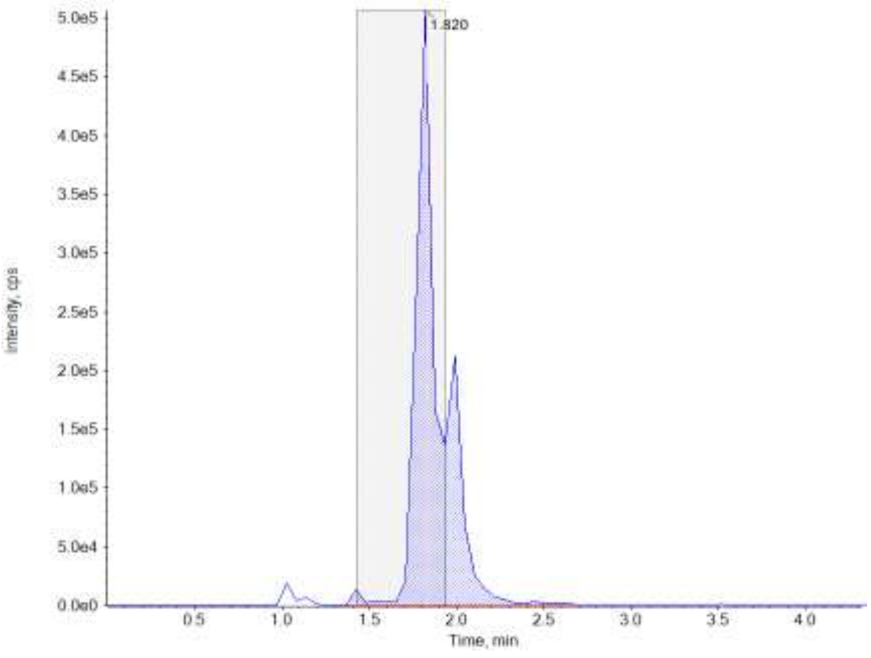

Acquired / Library MSMS

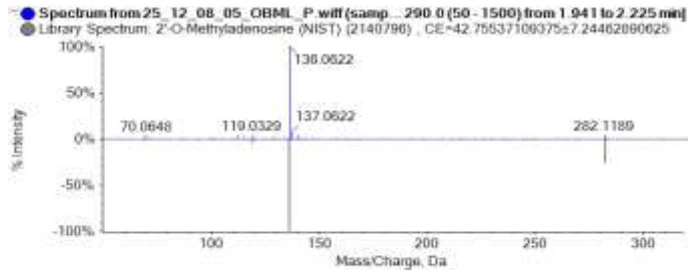

Acquired / Theoretical MS

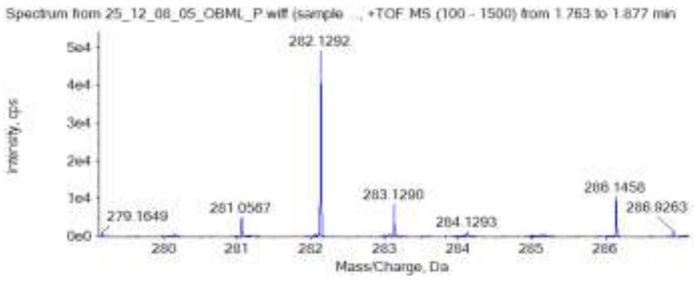

182.0977 / 1.93 [M+H]+ (Mass/FragMass/RT/Isotope/Library/Formula/Ion Ratio)

**Retention Time:** 1.91 minutes<br>**Precursor m/z :** 182.0977<br>**Fit (%)** 95.4% **RFit (%)** 93.6%	**Exp RT:** 1.93 minutes<br>**Analyte Name:**<br>182.0977 / 1.93 [M+H]+

Collision Energy = 35 ± 15 eV

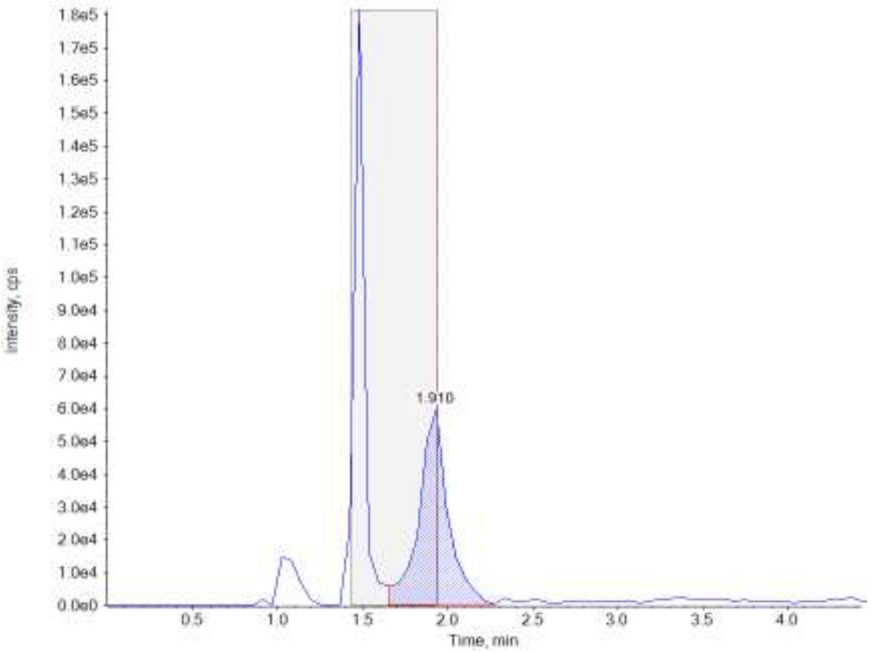

Acquired / Library MSMS

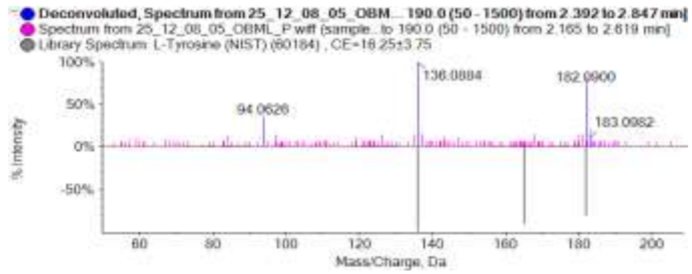

Acquired / Theoretical MS

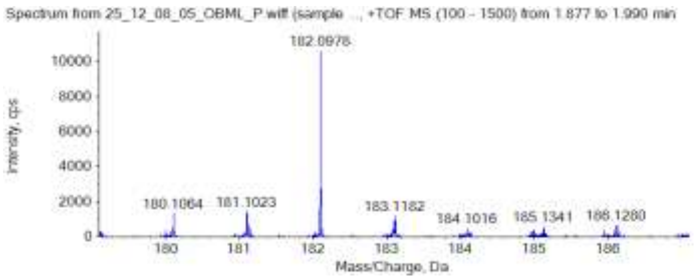

247.1344 / 1.99 (Mass/FragMass/RT/Isotope/Library/Formula/Ion Ratio)

Retention Time: 1.99 minutes
Precursor m/z : 247.1344
Fit (%) 85.3% **RFit (%)** 49.3%

Exp RT: 1.99 minutes
Analyte Name:
247.1344 / 1.99

Collision Energy = 35 ± 15 eV

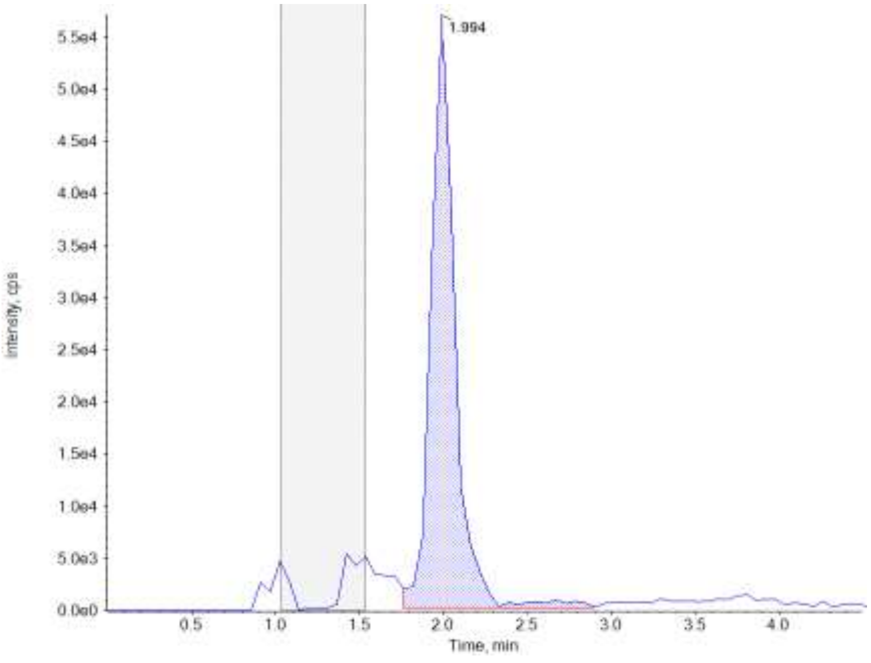

Acquired / Library MSMS

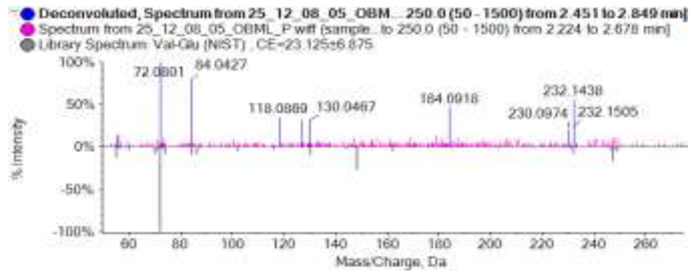

Acquired / Theoretical MS

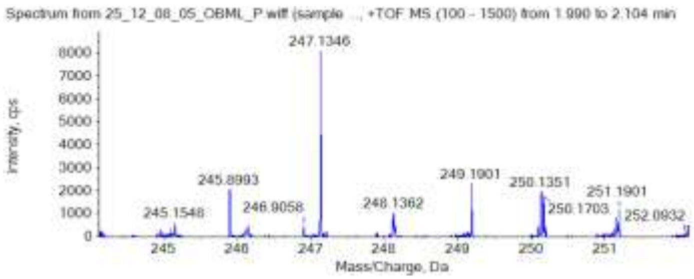

121.0899 / 2.27 (Mass/FragMass/RT/Isotope/Library/Formula/Ion Ratio)

<table>
<tr><td>Retention Time: 2.28 minutes
Precursor m/z : 121.0899
Fit (%) 100.0% RFit (%) 95.8%</td><td colspan="2">Exp RT: 2.27 minutes
Analyte Name:
121.0899 / 2.27</td></tr>
<tr><td></td><td colspan="2">Collision Energy = 35 ± 15 eV</td></tr>
<tr><td rowspan="2">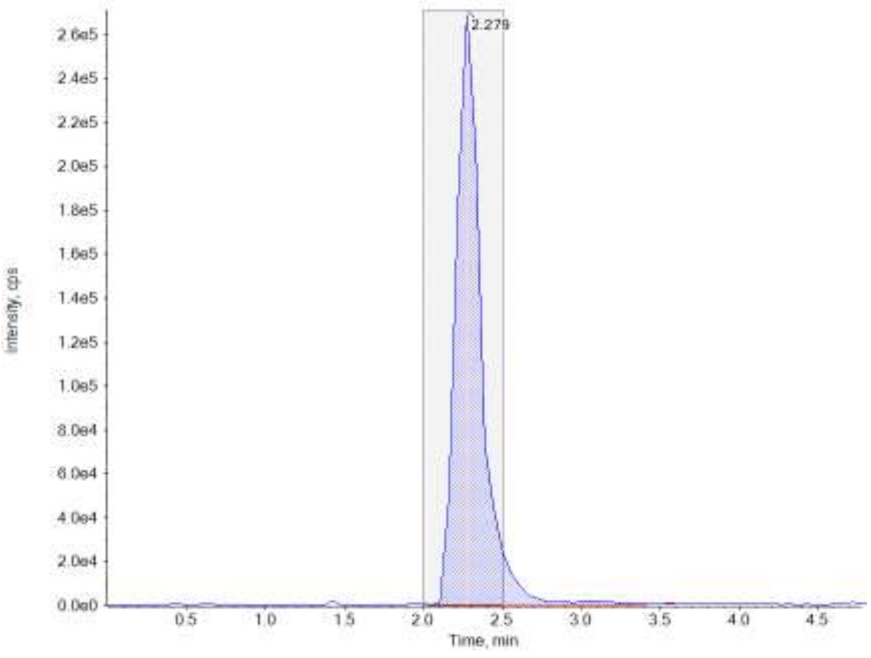</td><td>Acquired / Library MSMS</td><td>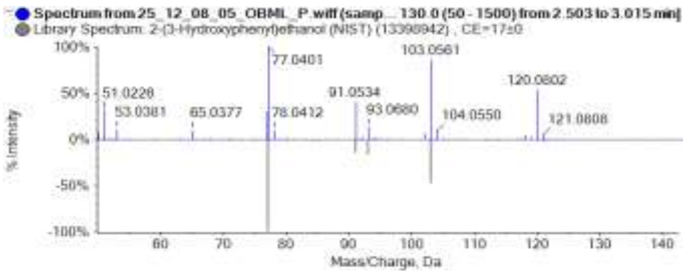</td></tr>
<tr><td>Acquired / Theoretical MS</td><td>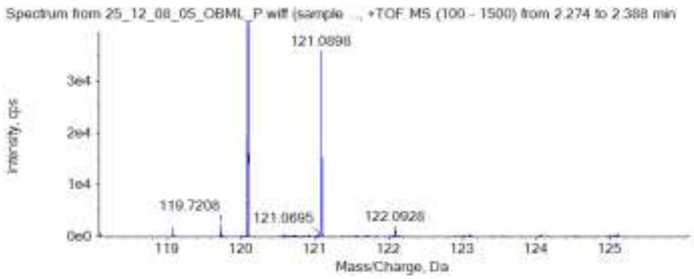</td></tr>
</table>

166.0966 / 2.27 (Mass/FragMass/RT/Isotope/Library/Formula/Ion Ratio)

Retention Time: 2.28 minutes
Precursor m/z : 166.0966
Fit (%) 99.8% **RFit (%)** 100.0%

Exp RT: 2.27 minutes
Analyte Name:
166.0966 / 2.27

Collision Energy = 35 ± 15 eV

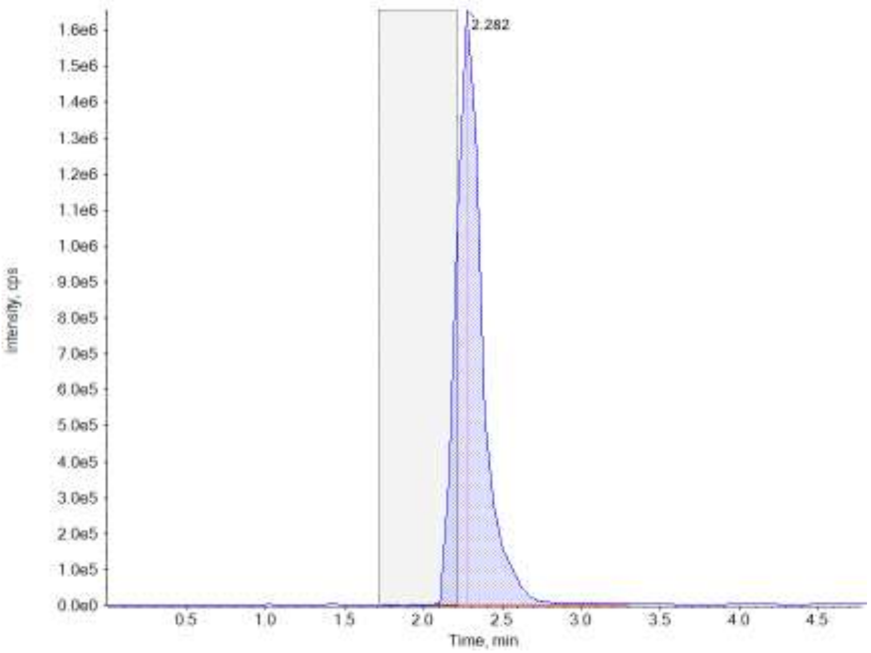

Acquired / Library MSMS

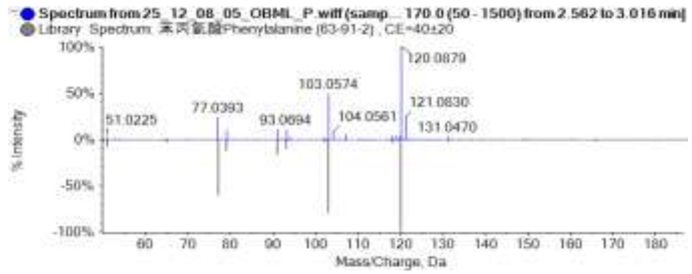

Acquired / Theoretical MS

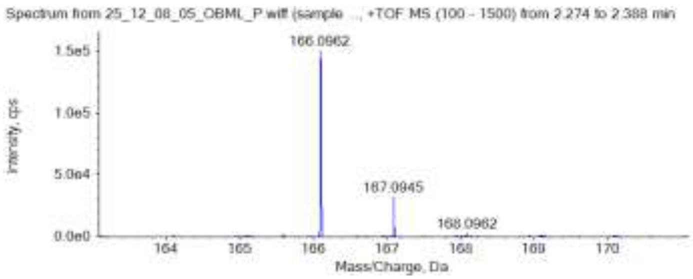

120.0918 / 2.33

(Mass/FragMass/RT/Isotope/Library/Formula/Ion Ratio)

**Retention Time:** 2.31 minutes	**Exp RT:** 2.33 minutes
**Precursor m/z :** 120.0918	**Analyte Name:**
**Fit (%)** 96.0% **RFit (%)** 95.4%	120.0918 / 2.33

Collision Energy = 35 ± 15 eV

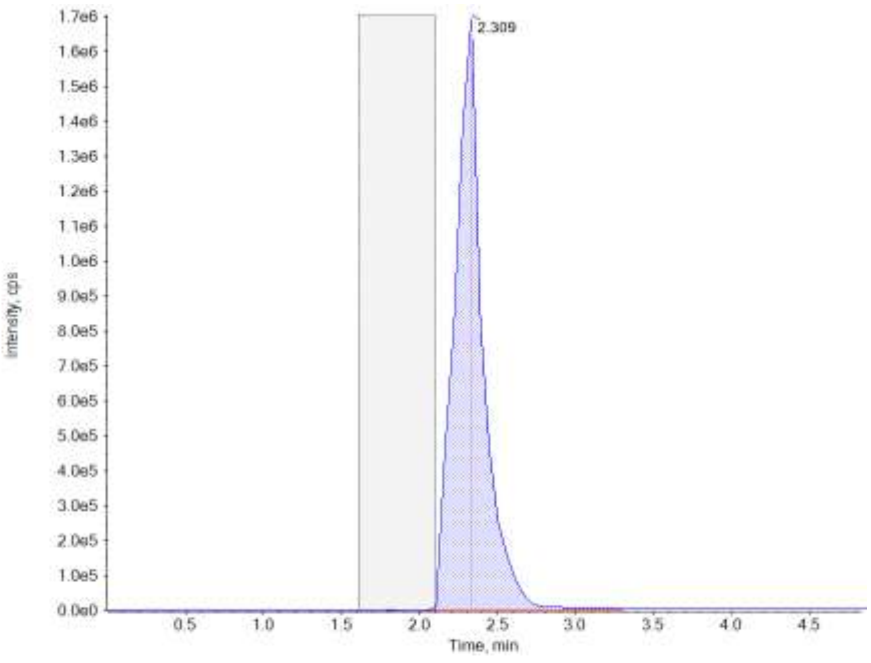

Acquired / Library MSMS

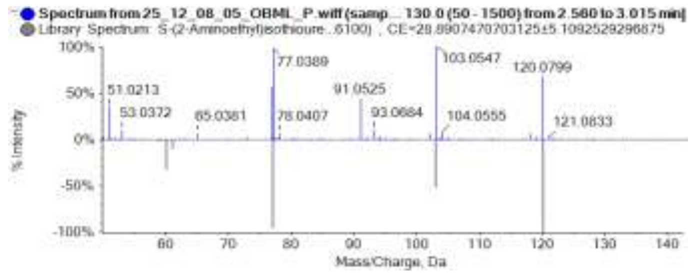

Acquired / Theoretical MS

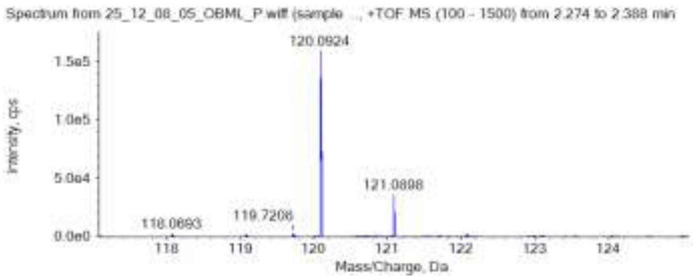

328.1472 / 2.33 (Mass/FragMass/RT/Isotope/Library/Formula/Ion Ratio)

Retention Time: 2.24 minutes
Precursor m/z : 328.1472
Fit (%) 100.0% **RFit (%)** 59.4%

Exp RT: 2.33 minutes
Analyte Name:
328.1472 / 2.33

Collision Energy = 35 ± 15 eV

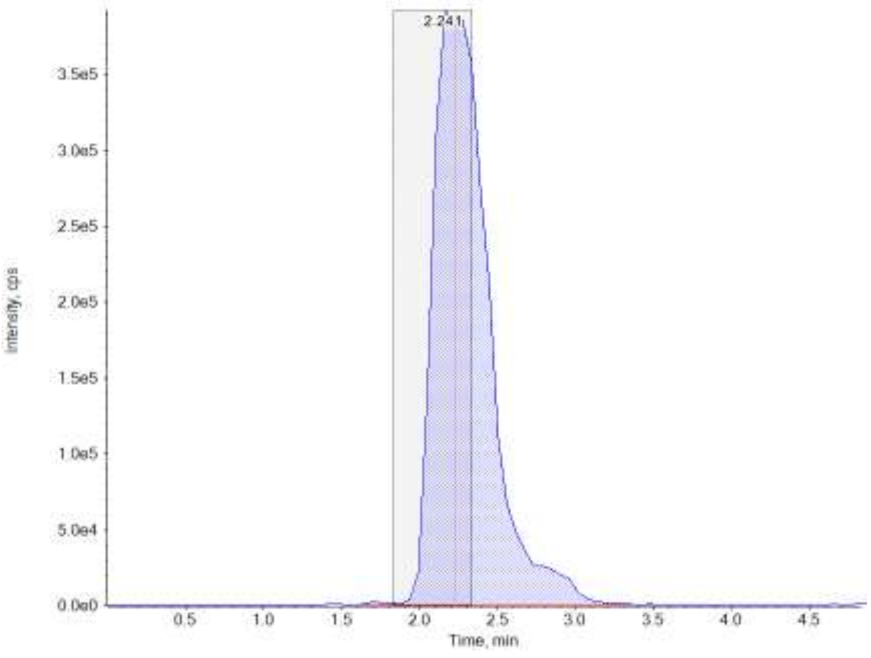

Acquired / Library MSMS

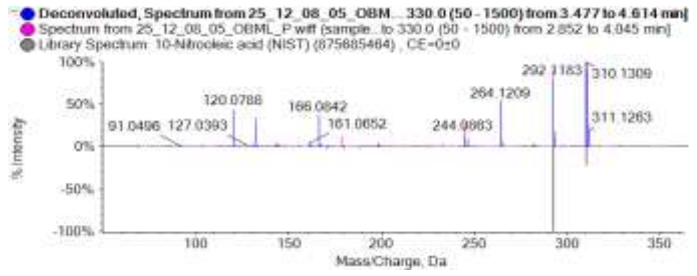

Acquired / Theoretical MS

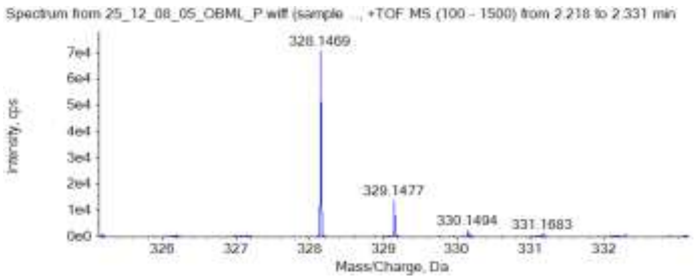

170.0859 / 2.67 (Mass/FragMass/RT/Isotope/Library/Formula/Ion Ratio)

Retention Time: 2.65 minutes **Exp RT:** 2.67 minutes
Precursor m/z : 170.0859 **Analyte Name:** 170.0859 / 2.67
Fit (%) 100.0% **RFit (%)** 95.1%

Collision Energy = 35 ± 15 eV

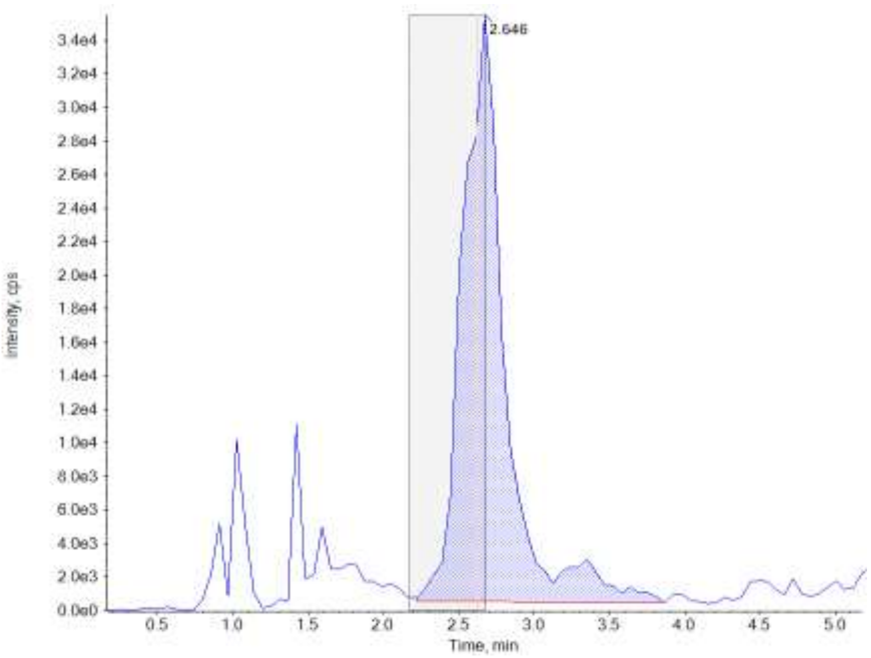

Acquired / Library MSMS

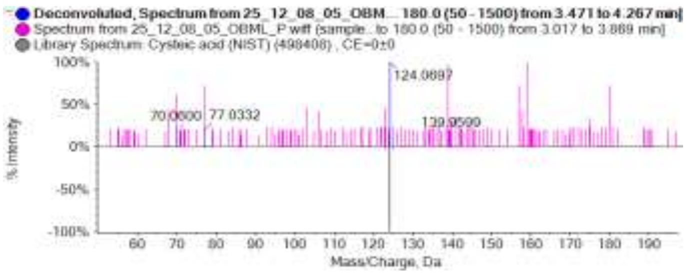

Acquired / Theoretical MS

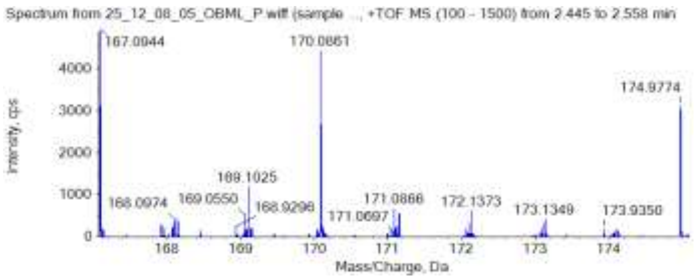

186.1290 / 2.73 (Mass/FragMass/RT/Isotope/Library/Formula/Ion Ratio)

Retention Time: 2.68 minutes
Precursor m/z : 186.1290
Fit (%) 33.2% **RFit (%)** 100.0%

Exp RT: 2.73 minutes
Analyte Name:
186.1290 / 2.73

Collision Energy = 35 ± 15 eV

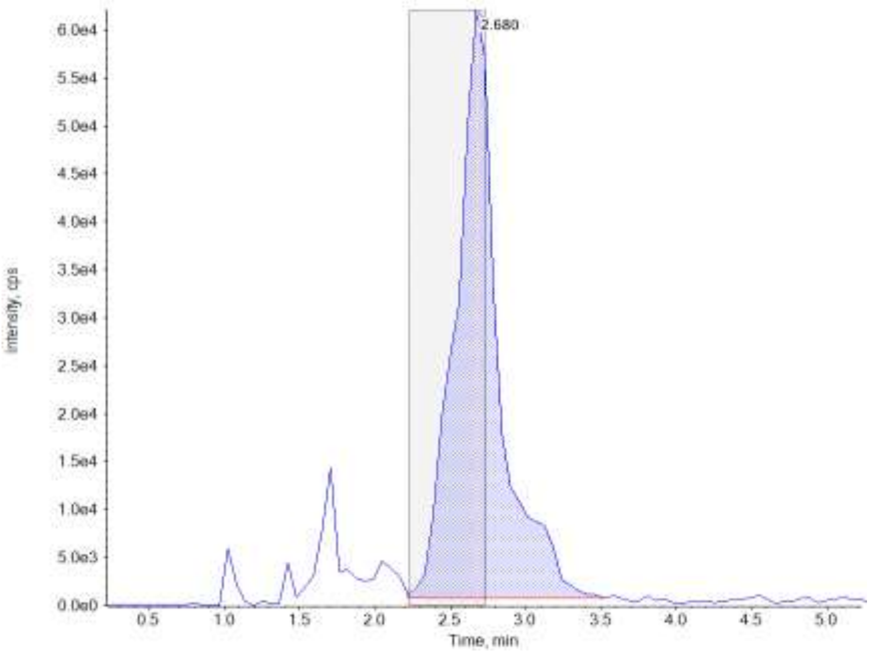

Acquired / Library MSMS

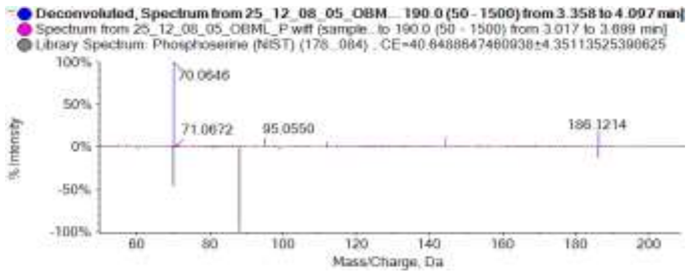

Acquired / Theoretical MS

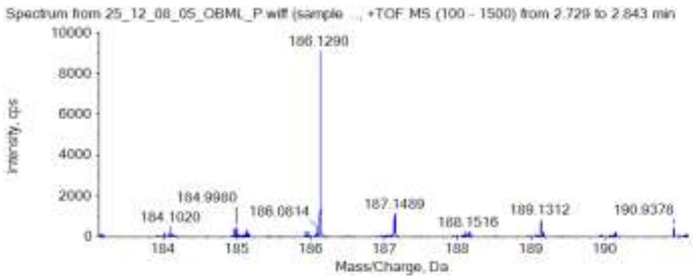

Created with SCIEX OS 3.3.0
Printed:12/9/2025 2:05:30 PM

232.1256 / 3.13 (Mass/FragMass/RT/Isotope/Library/Formula/Ion Ratio)

Retention Time: 3.10 minutes
Precursor m/z : 232.1256
Fit (%) 67.1% **RFit (%)** 83.5%

Exp RT: 3.13 minutes
Analyte Name:
232.1256 / 3.13

Collision Energy = 35 ± 15 eV

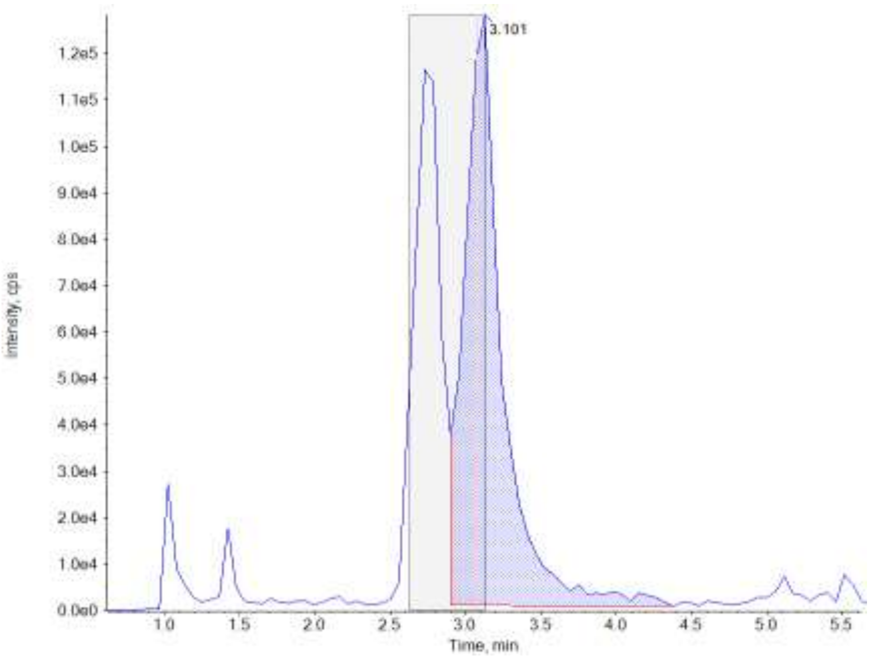

Acquired / Library MSMS

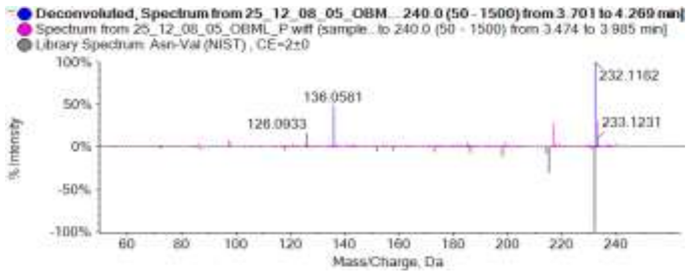

Acquired / Theoretical MS

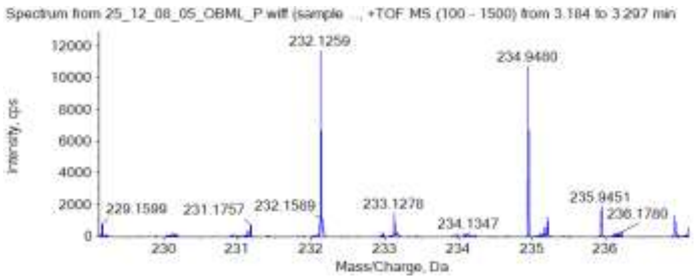

Created with SCIEX OS 3.3.0
Printed:12/9/2025 2:05:30 PM

102.0960 / 3.64 (Mass/FragMass/RT/Isotope/Library/Formula/Ion Ratio)

Retention Time: 3.61 minutes
Precursor m/z : 102.0960
Fit (%) 87.2% **RFit (%)** 45.6%

Exp RT: 3.64 minutes
Analyte Name:
102.0960 / 3.64

Collision Energy = 35 ± 15 eV

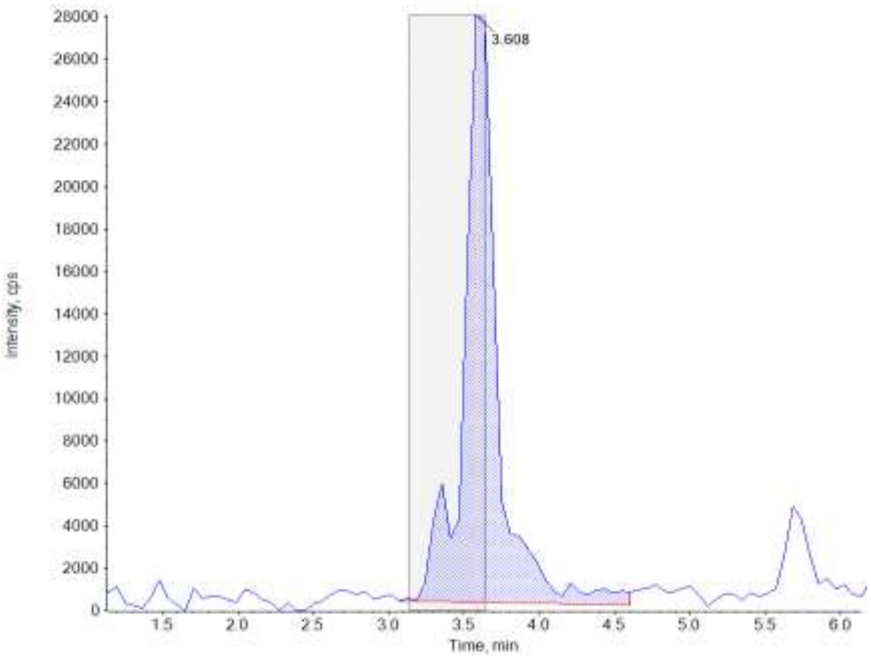

Acquired / Library MSMS

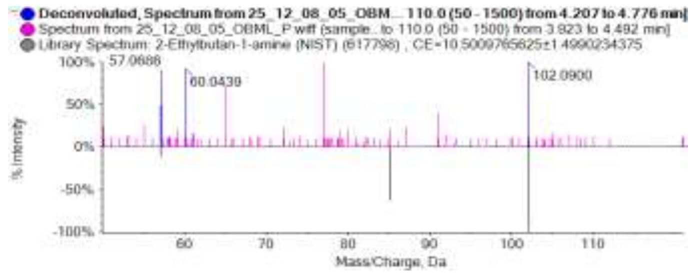

Acquired / Theoretical MS

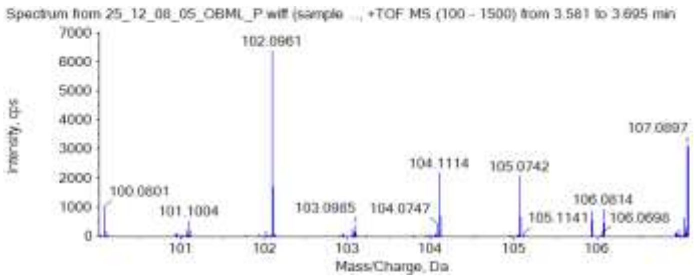

268.1593 / 4.04 (Mass/FragMass/RT/Isotope/Library/Formula/Ion Ratio)

Retention Time: 4.01 minutes
Precursor m/z : 268.1593
Fit (%) 85.8% **RFit (%)** 85.8%

Exp RT: 4.04 minutes
Analyte Name:
268.1593 / 4.04

Collision Energy = 35 ± 15 eV

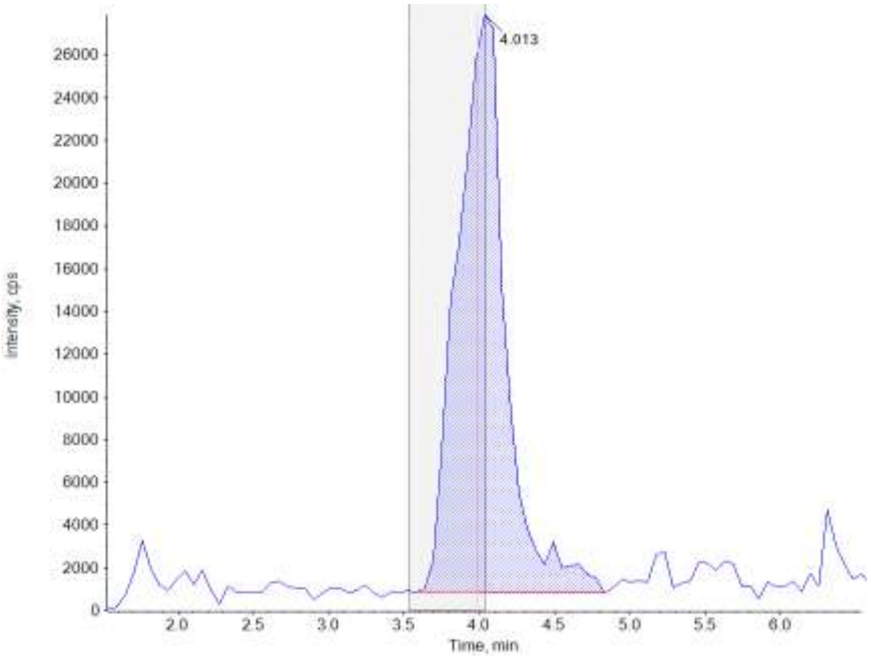

Acquired / Library MSMS

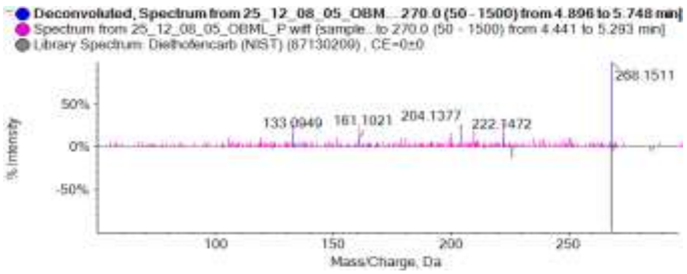

Acquired / Theoretical MS

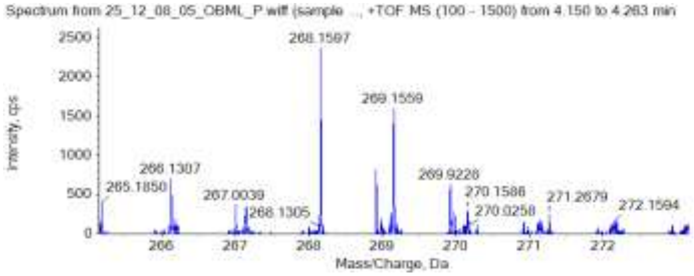

144.0852 / 4.49 (Mass/FragMass/RT/Isotope/Library/Formula/Ion Ratio)

Retention Time: 4.45 minutes
Precursor m/z : 144.0852
Fit (%) 97.3% **RFit (%)** 47.5%

Exp RT: 4.49 minutes
Analyte Name:
144.0852 / 4.49

Collision Energy = 35 ± 15 eV

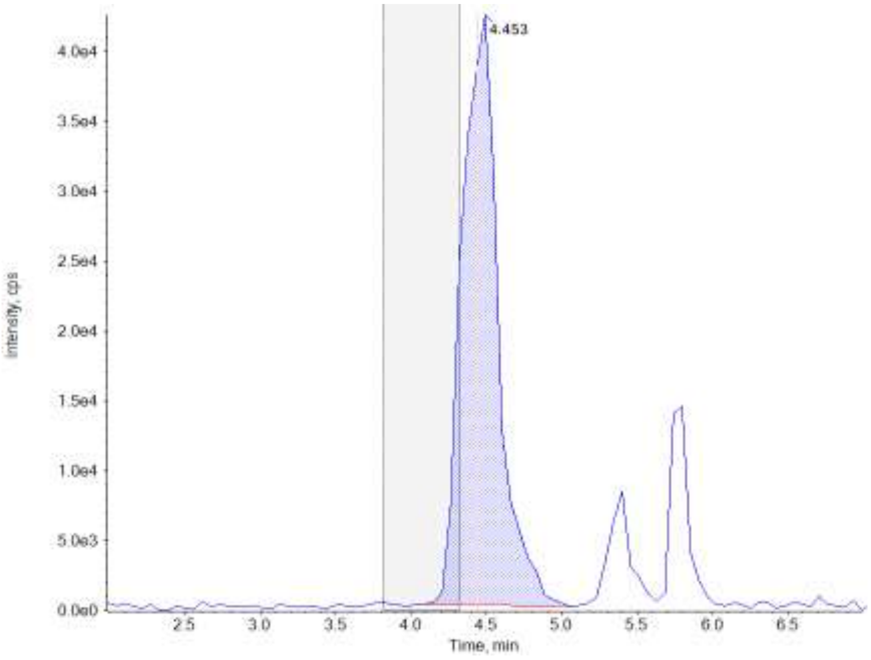

Acquired / Library MSMS

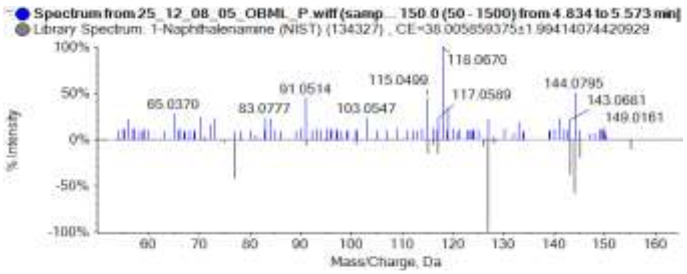

Acquired / Theoretical MS

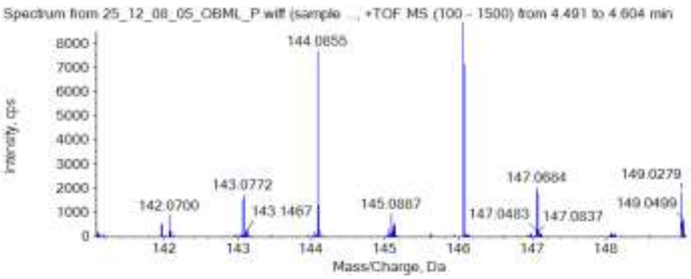

146.0652 / 4.49 (Mass/FragMass/RT/Isotope/Library/Formula/Ion Ratio)

**Retention Time:** 4.46 minutes<br>**Precursor m/z :** 146.0652<br>**Fit (%)** 97.5% **RFit (%)** 27.8%	**Exp RT:** 4.49 minutes<br>**Analyte Name:**<br>146.0652 / 4.49

	**Collision Energy = 35 ± 15 eV**
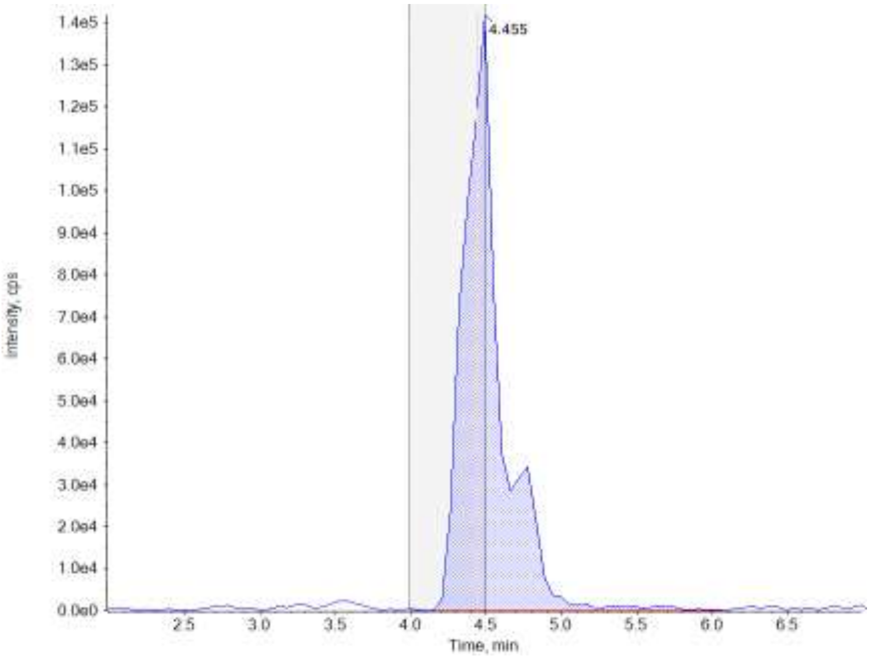	**Acquired / Library MSMS** 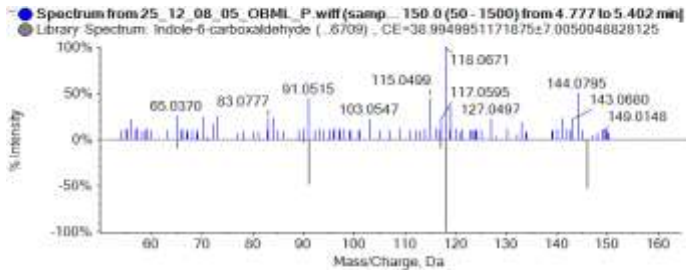
	**Acquired / Theoretical MS** 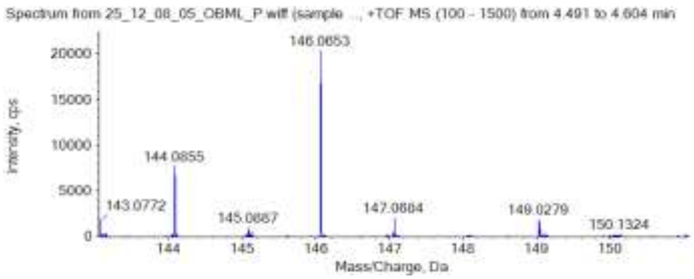

170.0647 / 4.49 (Mass/FragMass/RT/Isotope/Library/Formula/Ion Ratio)

Retention Time: 4.46 minutes
Precursor m/z : 170.0647
Fit (%) 100.0% **RFit (%)** 100.0%

Exp RT: 4.49 minutes
Analyte Name:
170.0647 / 4.49

Collision Energy = 35 ± 15 eV

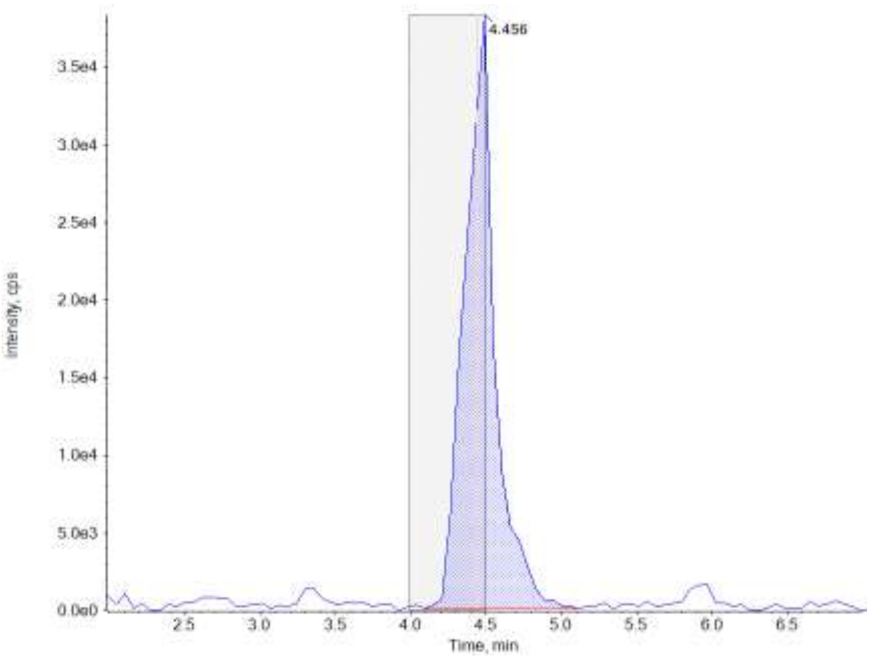

Acquired / Library MSMS

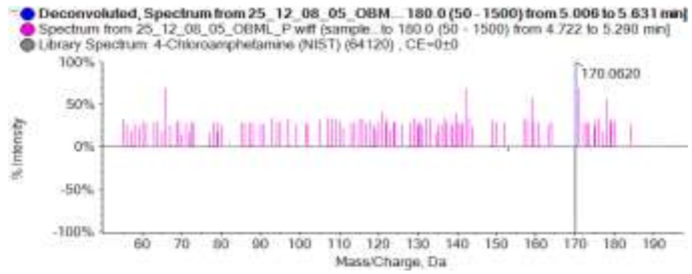

Acquired / Theoretical MS

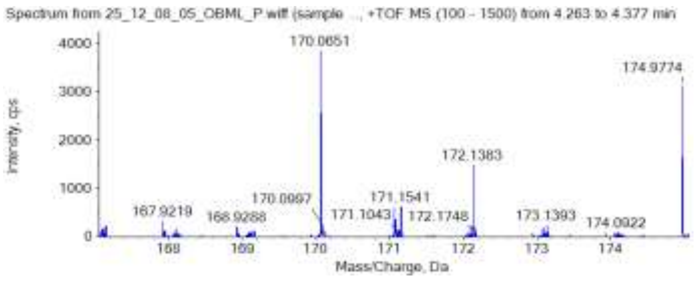

188.0842 / 4.49 (Mass/FragMass/RT/Isotope/Library/Formula/Ion Ratio)

Retention Time: 4.46 minutes
Precursor m/z : 188.0842
Fit (%) 97.7% **RFit (%)** 99.9%

Exp RT: 4.49 minutes
Analyte Name:
188.0842 / 4.49

Collision Energy = 35 ± 15 eV

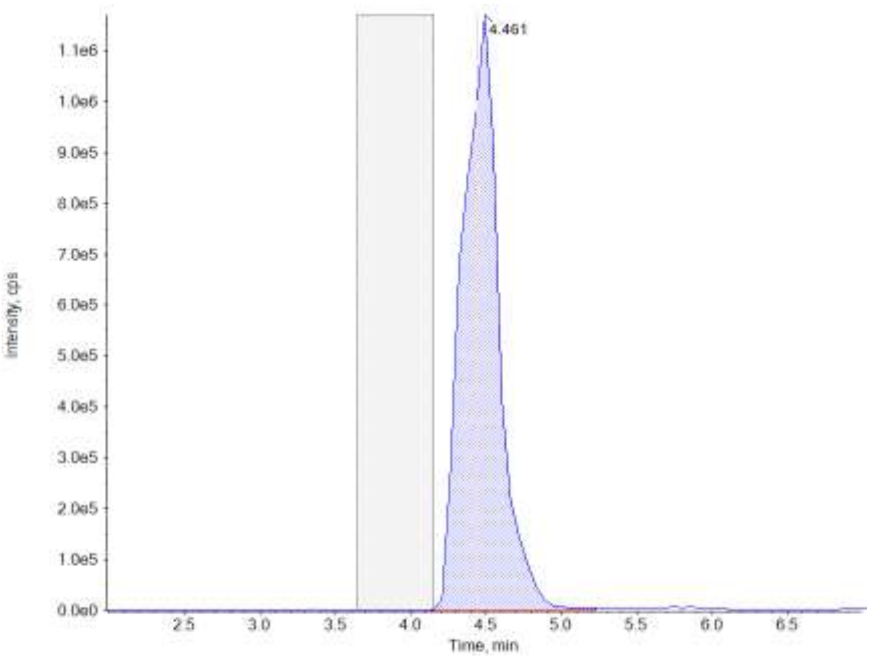

Acquired / Library MSMS

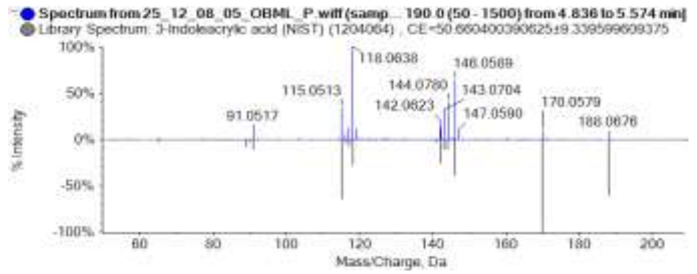

Acquired / Theoretical MS

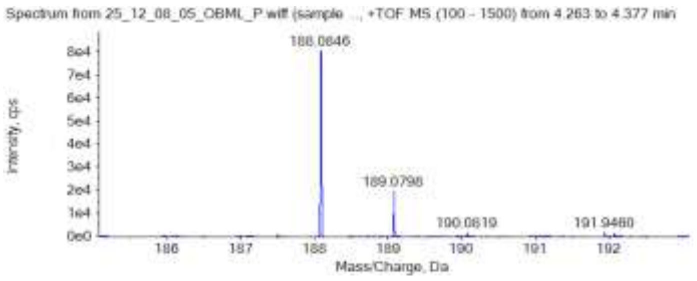

205.1047 / 4.49 (Mass/FragMass/RT/Isotope/Library/Formula/Ion Ratio)

Retention Time: 4.46 minutes
Precursor m/z : 205.1047
Fit (%) 97.9% **RFit (%)** 100.0%

Exp RT: 4.49 minutes
Analyte Name:
205.1047 / 4.49

Collision Energy = 35 ± 15 eV

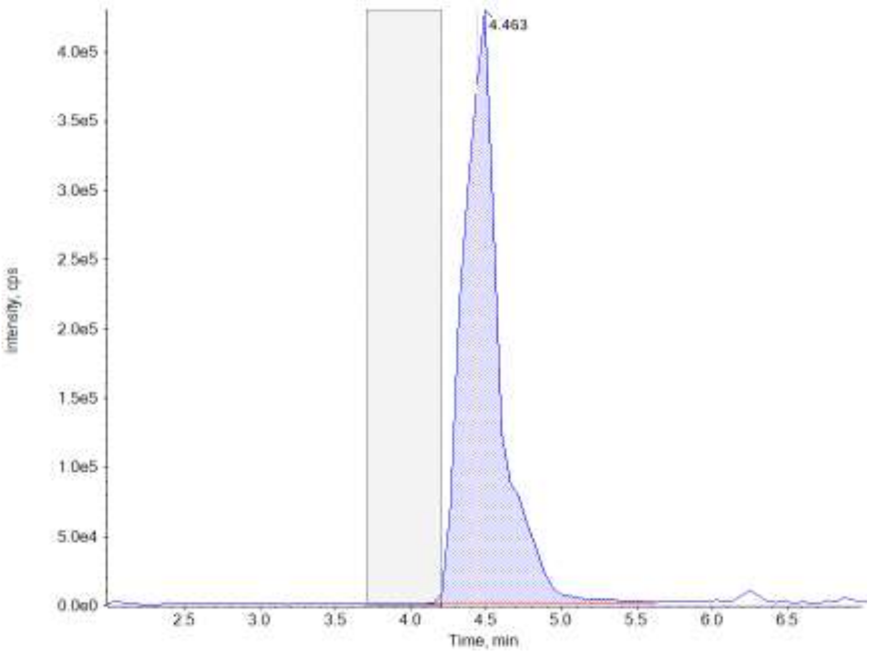

Acquired / Library MSMS

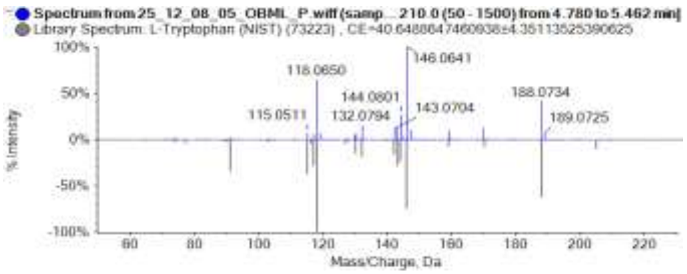

Acquired / Theoretical MS

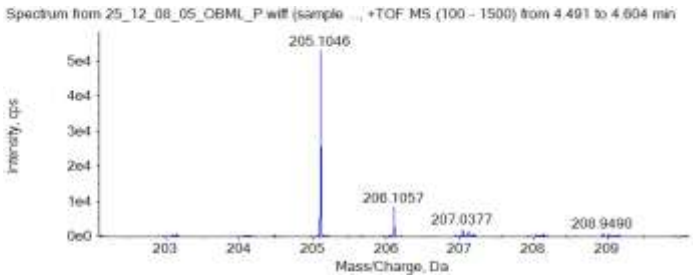

139.0442 / 5.00 (Mass/FragMass/RT/Isotope/Library/Formula/Ion Ratio)

Retention Time: 4.98 minutes
Precursor m/z : 139.0442
Fit (%) 92.7% **RFit (%)** 100.0%

Exp RT: 5.00 minutes
Analyte Name:
139.0442 / 5.00

Collision Energy = 35 ± 15 eV

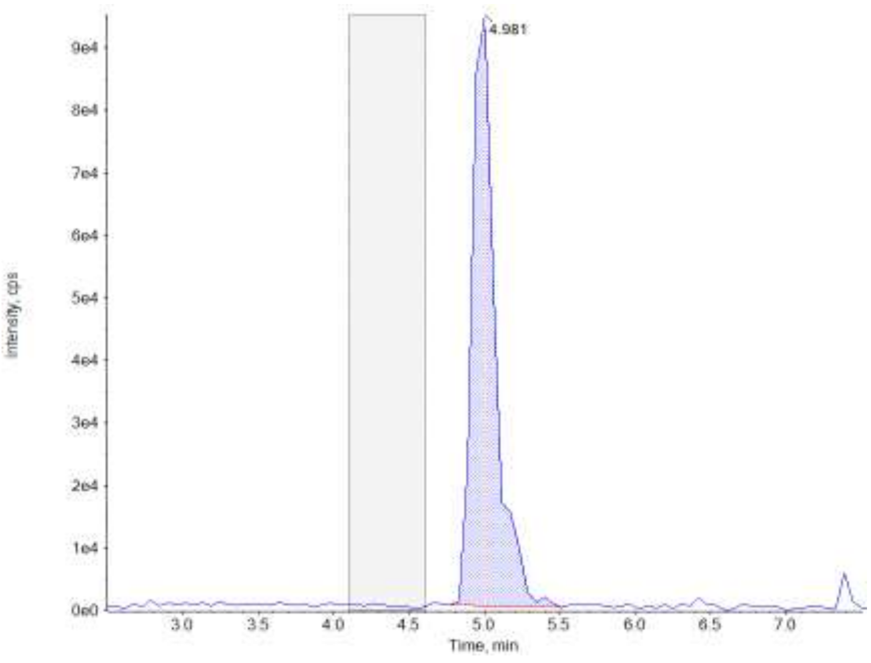

Acquired / Library MSMS

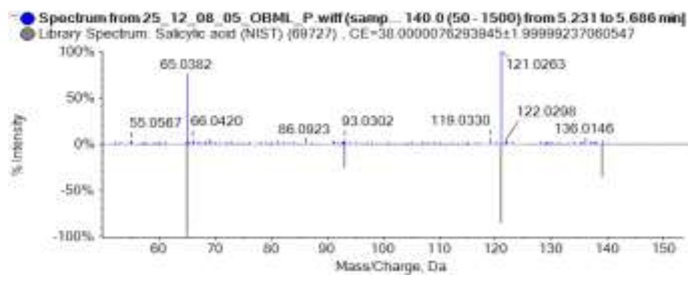

Acquired / Theoretical MS

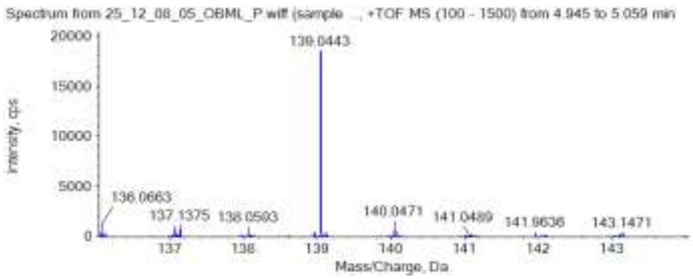

223.1382 / 5.00 (Mass/FragMass/RT/Isotope/Library/Formula/Ion Ratio)

Retention Time: 5.03 minutes
Precursor m/z : 223.1382
Fit (%) 33.3% **RFit (%)** 96.3%

Exp RT: 5.00 minutes
Analyte Name:
223.1382 / 5.00

Collision Energy = 35 ± 15 eV

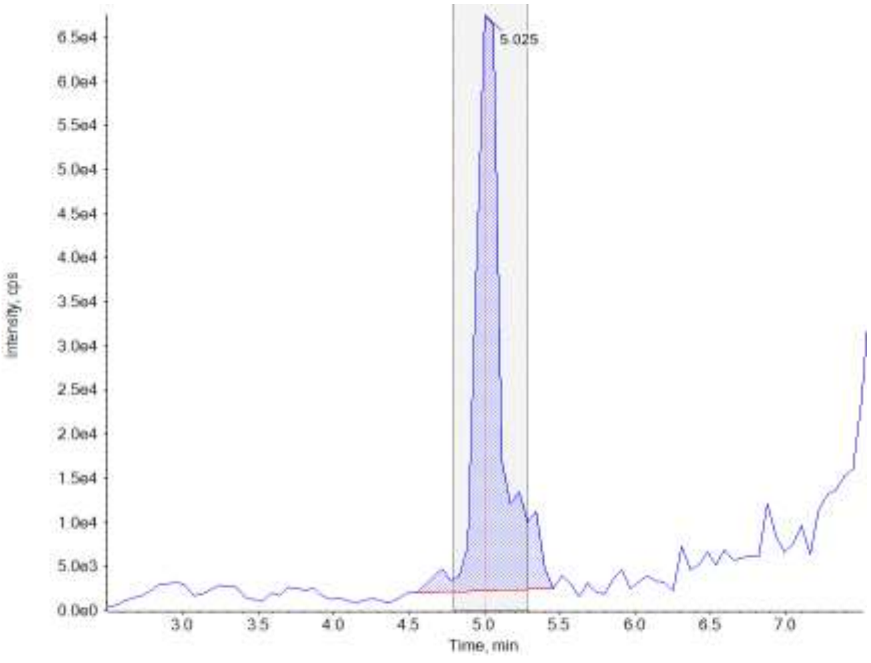

Acquired / Library MSMS

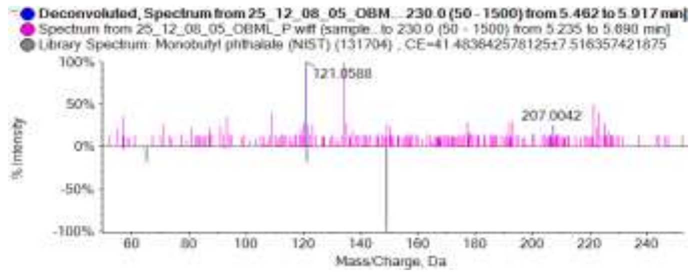

Acquired / Theoretical MS

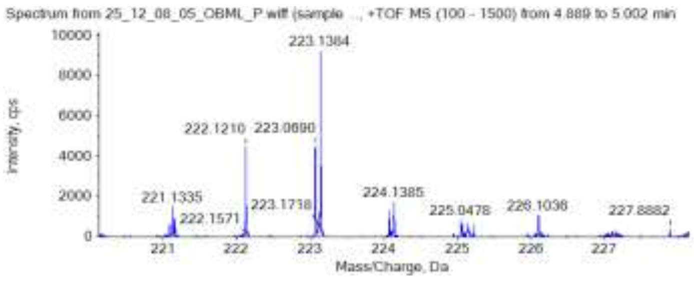

441.1576 / 5.06 [M+K]+ (Mass/FragMass/RT/Isotope/Library/Formula/Ion Ratio)

**Retention Time:** 5.02 minutes<br>**Precursor m/z :** 441.1576<br>**Fit (%)** 100.0% **RFit (%)** 79.5%	**Exp RT:** 5.06 minutes<br>**Analyte Name:**<br>441.1576 / 5.06 [M+K]+

Collision Energy = 35 ± 15 eV

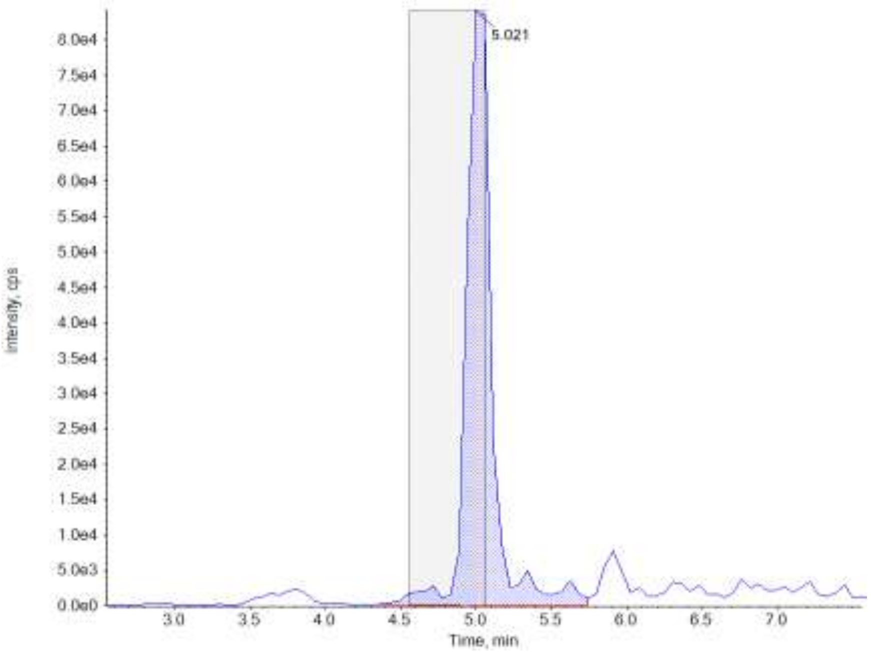

Acquired / Library MSMS

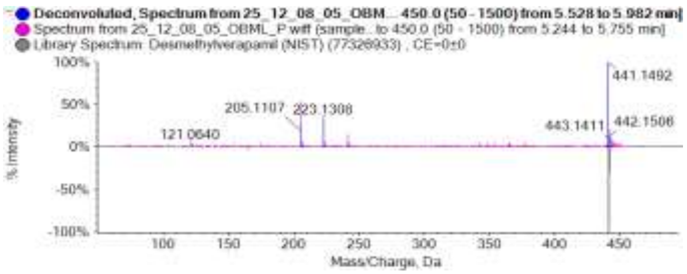

Acquired / Theoretical MS

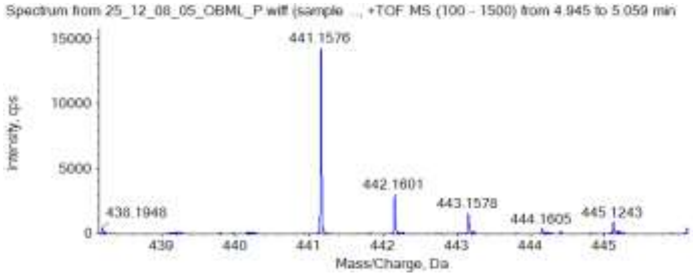

205.1273 / 5.06 (Mass/FragMass/RT/Isotope/Library/Formula/Ion Ratio)

**Retention Time:** 5.06 minutes	**Exp RT:** 5.06 minutes
**Precursor m/z :** 205.1273	**Analyte Name:**
**Fit (%)** 100.0% **RFit (%)** 96.9%	205.1273 / 5.06

Collision Energy = 35 ± 15 eV

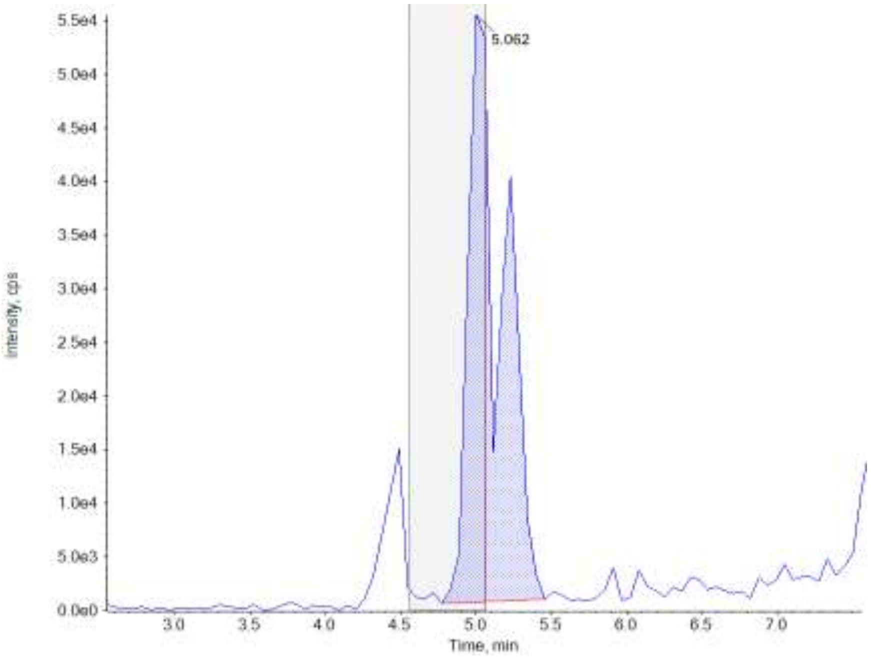

Acquired / Library MSMS

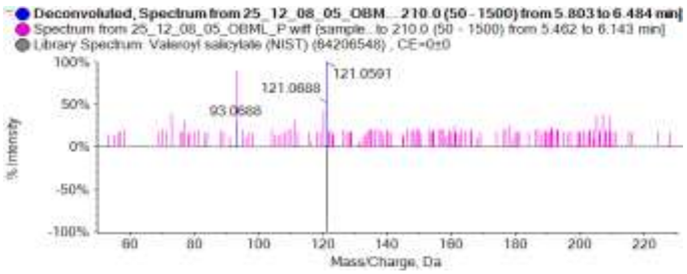

Acquired / Theoretical MS

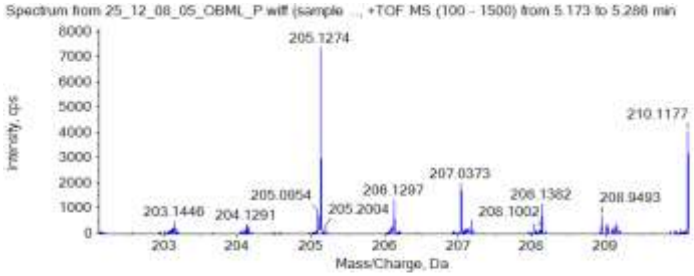

172.1013 / 5.34 (Mass/FragMass/RT/Isotope/Library/Formula/Ion Ratio)

Retention Time: 5.32 minutes
Precursor m/z : 172.1013
Fit (%) 39.1% **RFit (%)** 99.4%

Exp RT: 5.34 minutes
Analyte Name:
172.1013 / 5.34

Collision Energy = 35 ± 15 eV

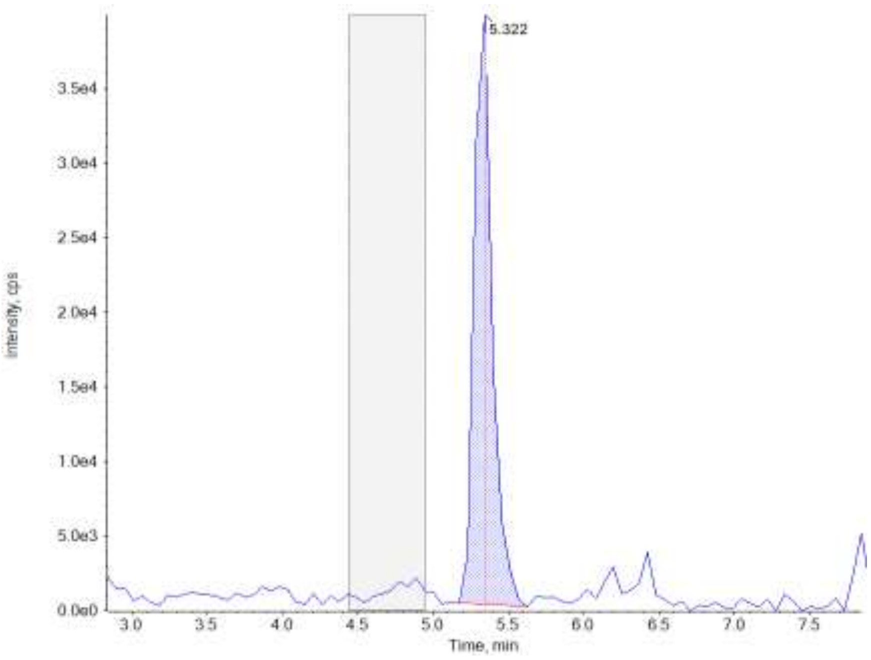

Acquired / Library MSMS

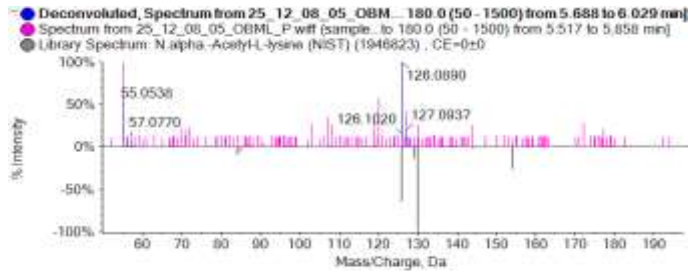

Acquired / Theoretical MS

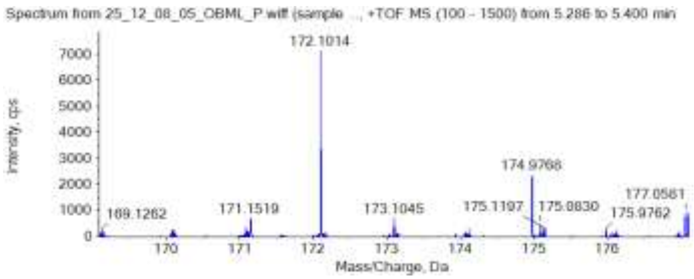

295.1345 / 5.40

(Mass/FragMass/RT/Isotope/Library/Formula/Ion Ratio)

Retention Time: 5.41 minutes
Precursor m/z : 295.1345
Fit (%) 80.0% **RFit (%)** 95.0%

Exp RT: 5.40 minutes
Analyte Name:
295.1345 / 5.40

Collision Energy = 35 ± 15 eV

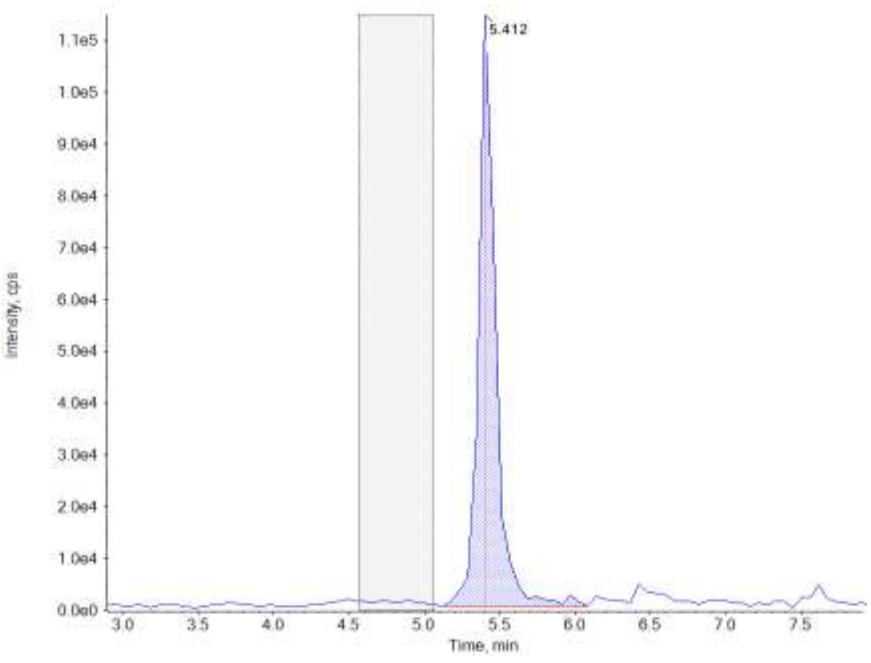

Acquired / Library MSMS

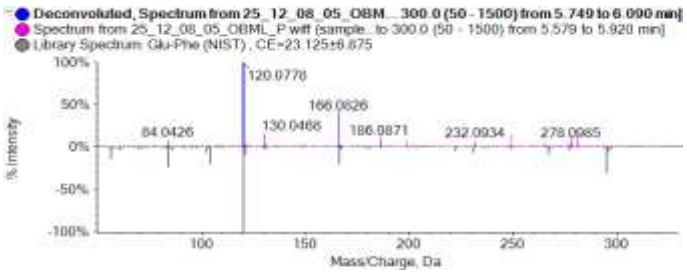

Acquired / Theoretical MS

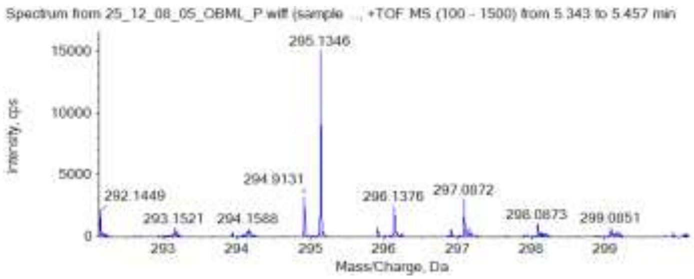

282.1752 / 5.51 (Mass/FragMass/RT/Isotope/Library/Formula/Ion Ratio)

Retention Time: 5.49 minutes
Precursor m/z : 282.1751
Fit (%) 100.0% **RFit (%)** 94.7%

Exp RT: 5.51 minutes
Analyte Name:
282.1752 / 5.51

Collision Energy = 35 ± 15 eV

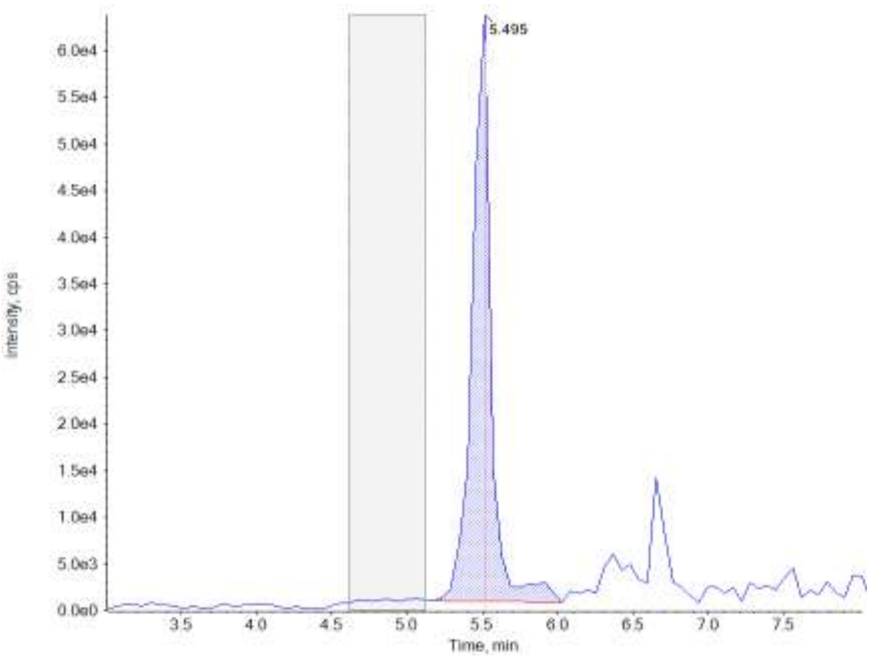

Acquired / Library MSMS

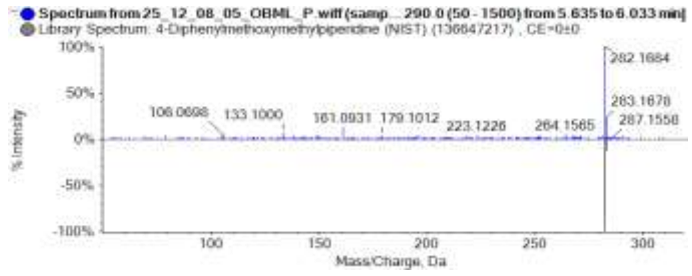

Acquired / Theoretical MS

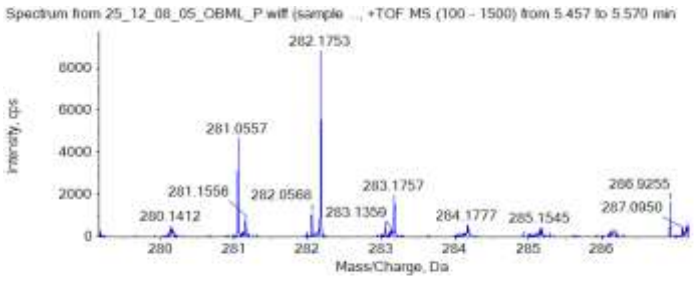

Created with SCIEX OS 3.3.0
Printed:12/9/2025 2:05:30 PM

175.1533 / 5.63 (Mass/FragMass/RT/Isotope/Library/Formula/Ion Ratio)

Retention Time: 5.62 minutes
Precursor m/z : 175.1533
Fit (%) 76.0% **RFit (%)** 81.6%

Exp RT: 5.63 minutes
Analyte Name:
175.1533 / 5.63

Collision Energy = 35 ± 15 eV

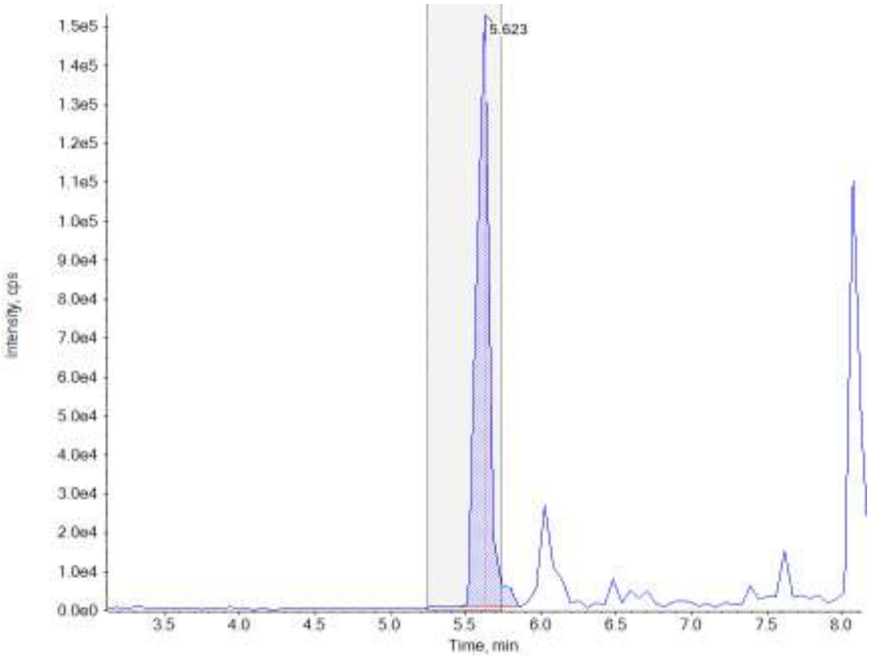

Acquired / Library MSMS

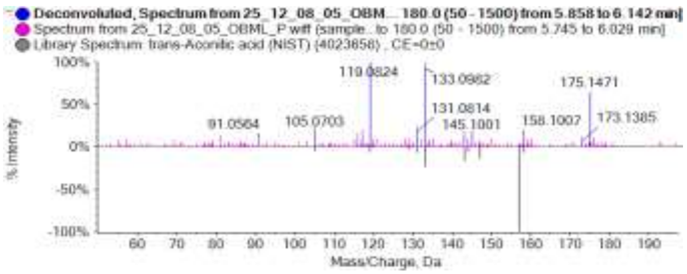

Acquired / Theoretical MS

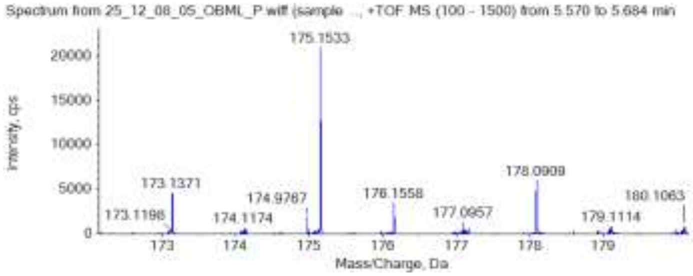

Created with SCIEX OS 3.3.0
Printed:12/9/2025 2:05:30 PM

389.1619 / 5.63 (Mass/FragMass/RT/Isotope/Library/Formula/Ion Ratio)

Retention Time: 5.55 minutes
Precursor m/z : 389.1619
Fit (%) 100.0% **RFit (%)** 100.0%

Exp RT: 5.63 minutes
Analyte Name:
389.1619 / 5.63

Collision Energy = 35 ± 15 eV

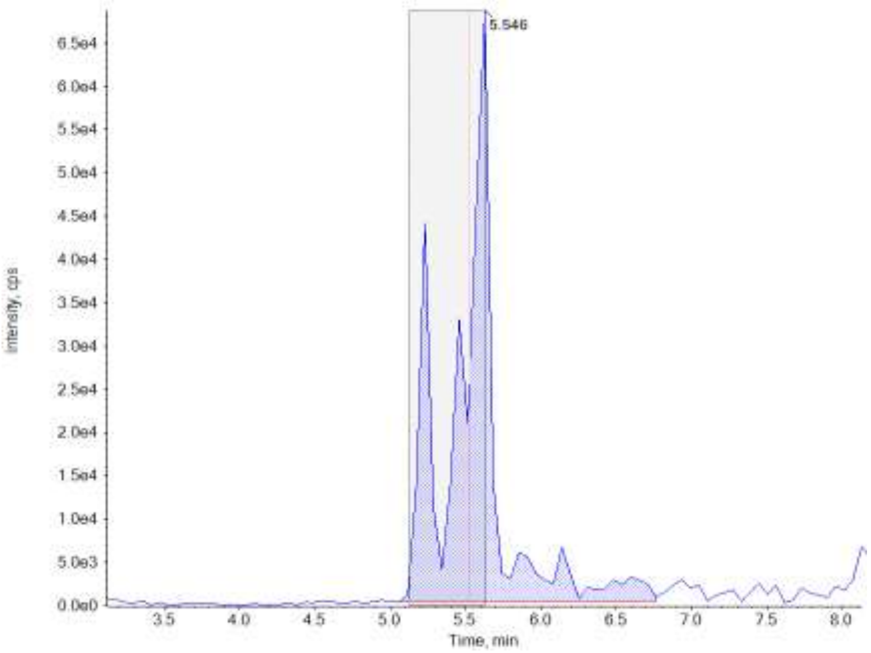

Acquired / Library MSMS

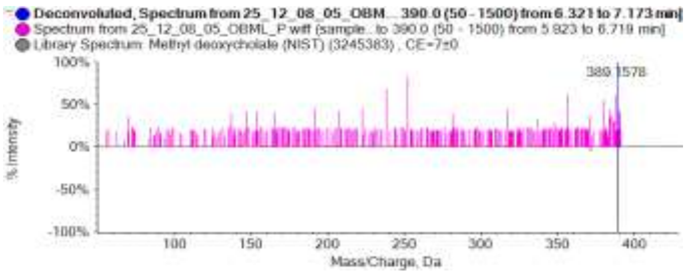

Acquired / Theoretical MS

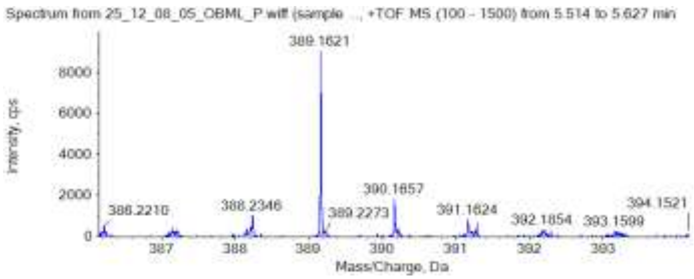

Created with SCIEX OS 3.3.0
Printed:12/9/2025 2:05:30 PM

427.1763 / 5.63 (Mass/FragMass/RT/Isotope/Library/Formula/Ion Ratio)

Retention Time: 5.63 minutes
Precursor m/z : 427.1763
Fit (%) 90.8% **RFit (%)** 100.0%

Exp RT: 5.63 minutes
Analyte Name:
427.1763 / 5.63

Collision Energy = 35 ± 15 eV

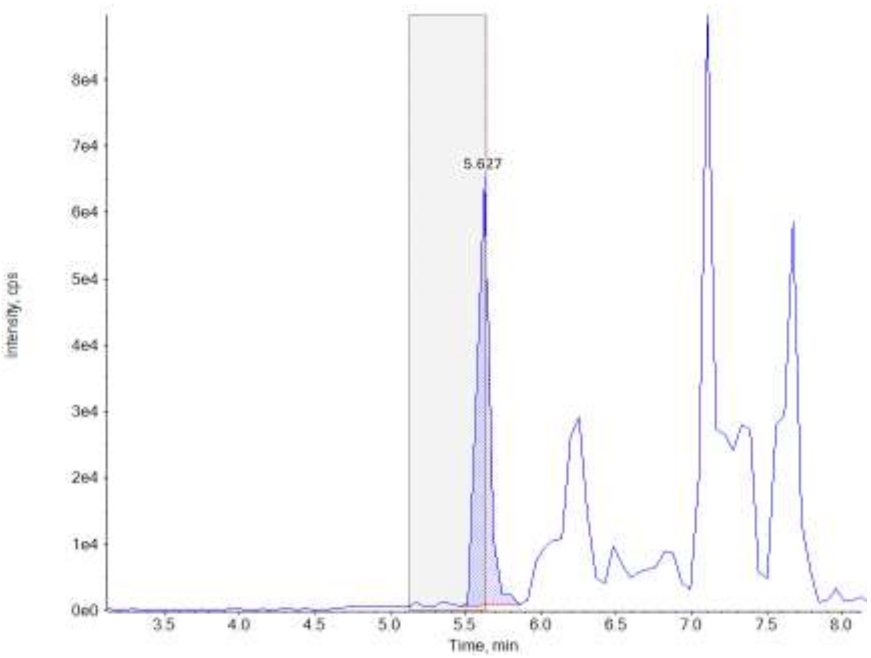

Acquired / Library MSMS

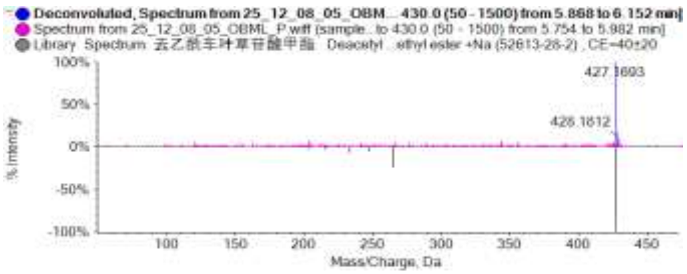

Acquired / Theoretical MS

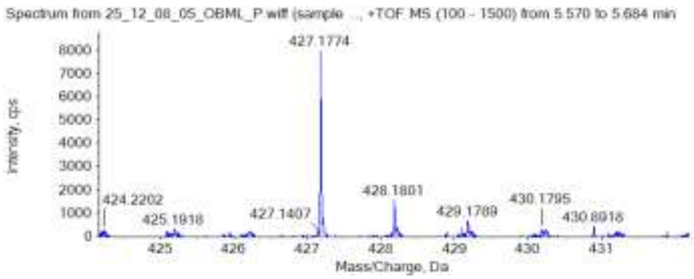

231.1180 / 5.91 (Mass/FragMass/RT/Isotope/Library/Formula/Ion Ratio)

**Retention Time:** 5.91 minutes<br>**Precursor m/z :** 231.1180<br>**Fit (%)** 95.6% **RFit (%)** 49.6%	**Exp RT:** 5.91 minutes<br>**Analyte Name:**<br>231.1180 / 5.91

Collision Energy = 35 ± 15 eV

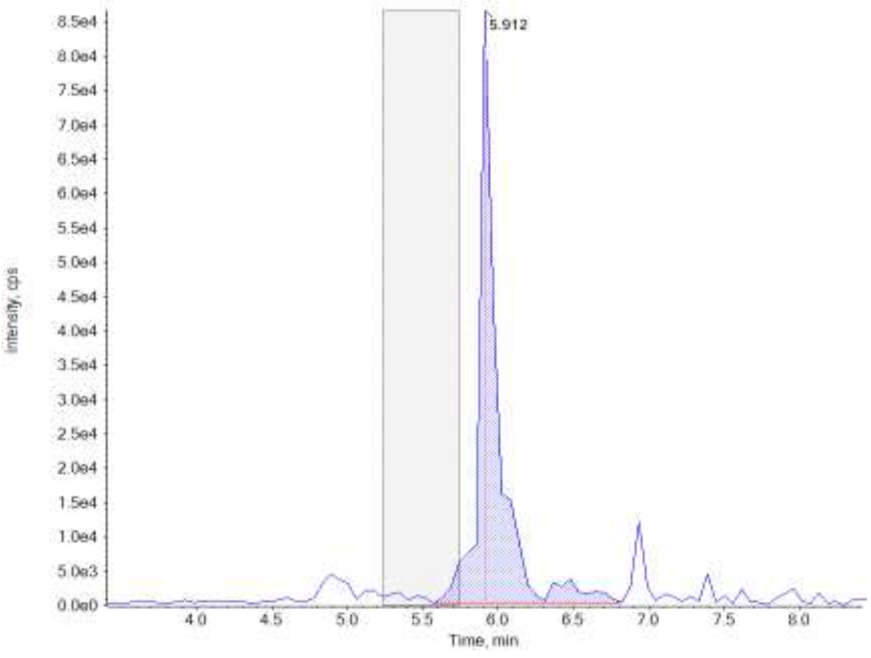

Acquired / Library MSMS

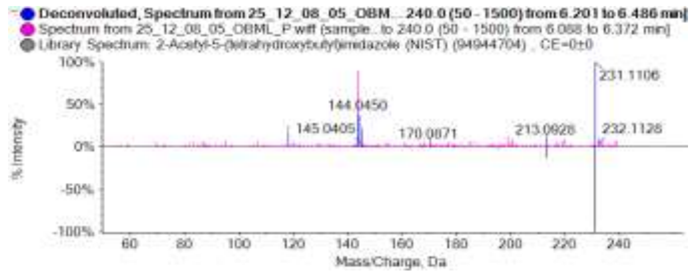

Acquired / Theoretical MS

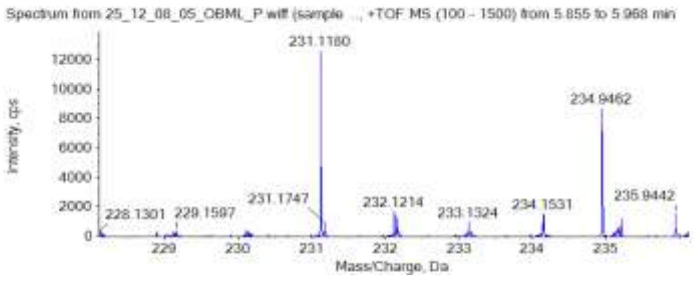

136.0804 / 5.97 (Mass/FragMass/RT/Isotope/Library/Formula/Ion Ratio)

**Retention Time:** 5.97 minutes	**Exp RT:** 5.97 minutes
**Precursor m/z :** 136.0804	**Analyte Name:**
**Fit (%)** 91.5% **RFit (%)** 100.0%	136.0804 / 5.97

Collision Energy = 35 ± 15 eV

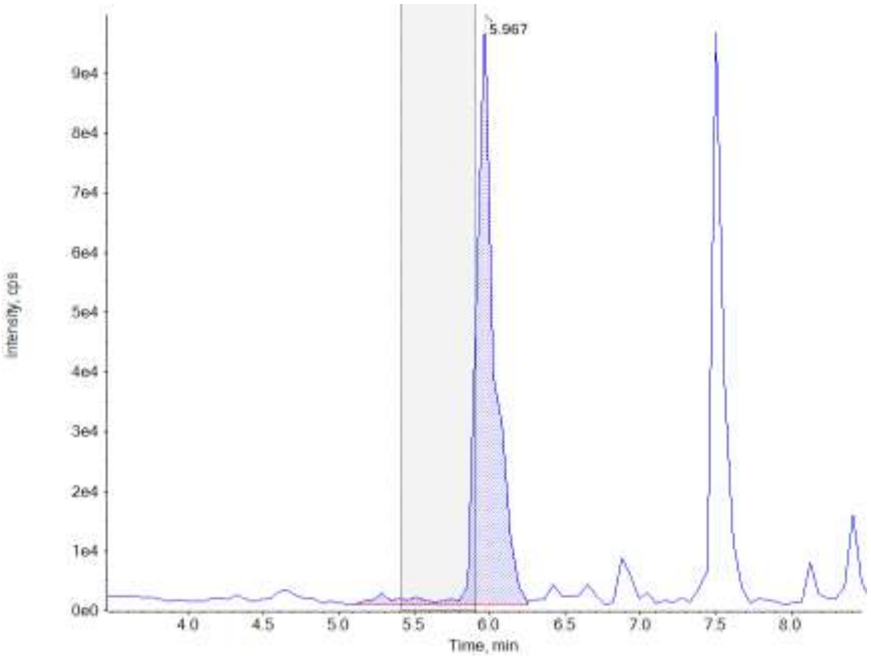

Acquired / Library MSMS

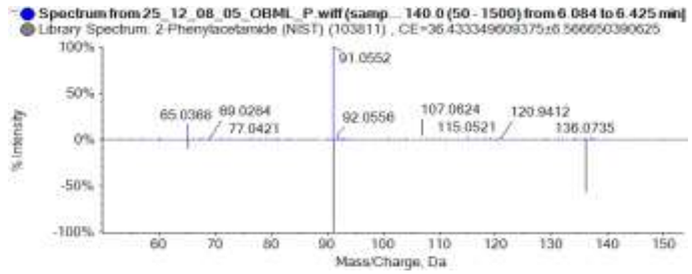

Acquired / Theoretical MS

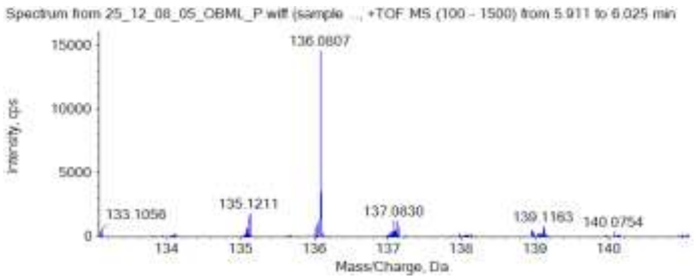

166.0912 / 6.08 (Mass/FragMass/RT/Isotope/Library/Formula/Ion Ratio)

**Retention Time:** 6.08 minutes<br>**Precursor m/z :** 166.0912<br>**Fit (%)** 100.0% **RFit (%)** 23.9%	**Exp RT:** 6.08 minutes<br>**Analyte Name:**<br>166.0912 / 6.08

Collision Energy = 35 ± 15 eV

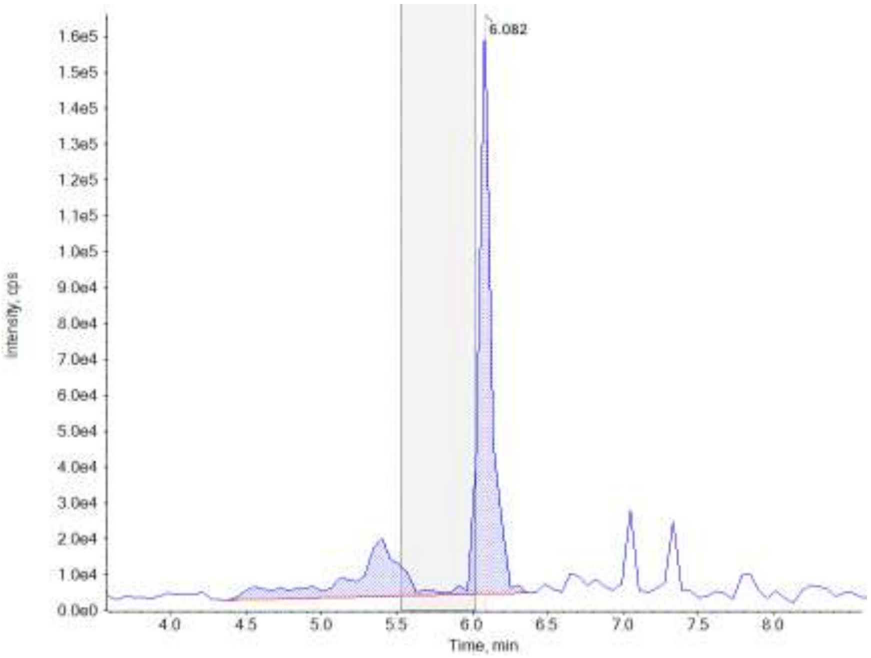

Acquired / Library MSMS

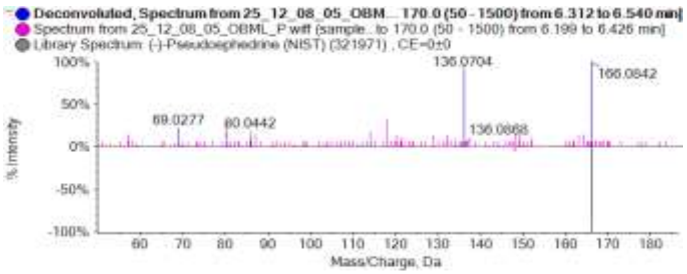

Acquired / Theoretical MS

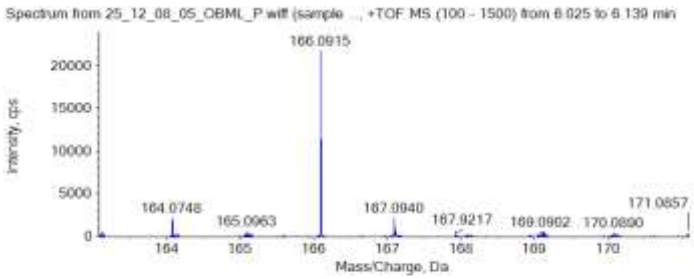

266.1804 / 6.20 (Mass/FragMass/RT/Isotope/Library/Formula/Ion Ratio)

Retention Time: 6.22 minutes
Precursor m/z : 266.1804
Fit (%) 99.8% **RFit (%)** 75.9%

Exp RT: 6.20 minutes
Analyte Name:
266.1804 / 6.20

Collision Energy = 35 ± 15 eV

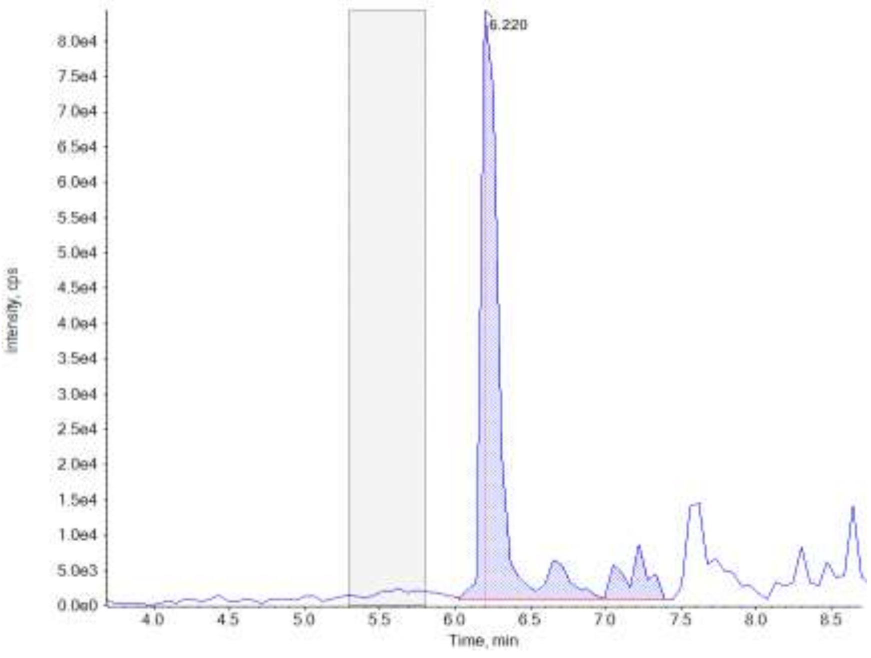

Acquired / Library MSMS

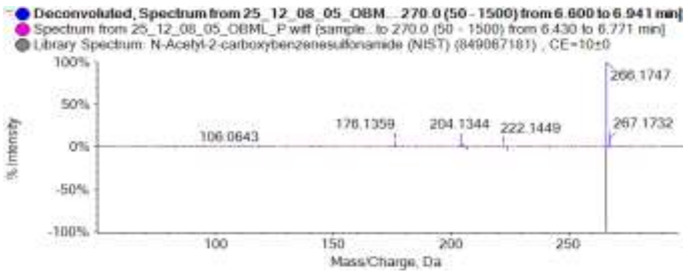

Acquired / Theoretical MS

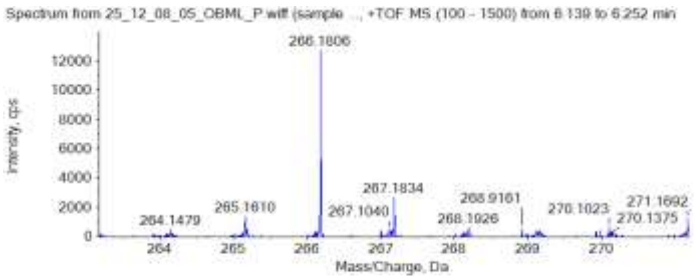

425.1641 / 6.25 (Mass/FragMass/RT/Isotope/Library/Formula/Ion Ratio)

Retention Time: 6.25 minutes
Precursor m/z : 425.1641
Fit (%) 78.9% **RFit (%)** 100.0%

Exp RT: 6.25 minutes
Analyte Name:
425.1641 / 6.25

Collision Energy = 35 ± 15 eV

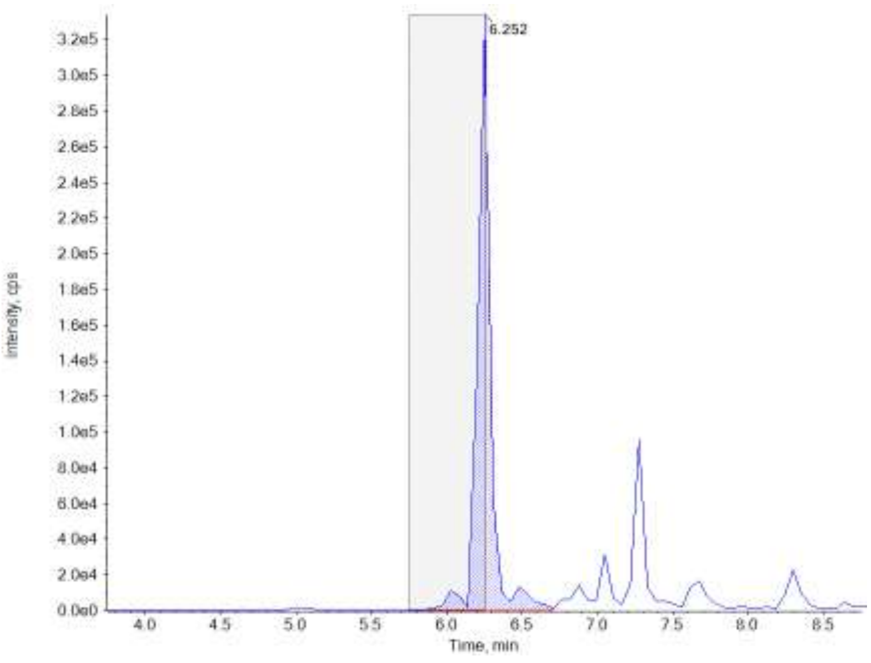

Acquired / Library MSMS

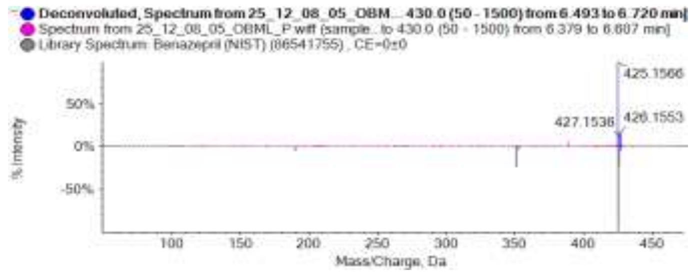

Acquired / Theoretical MS

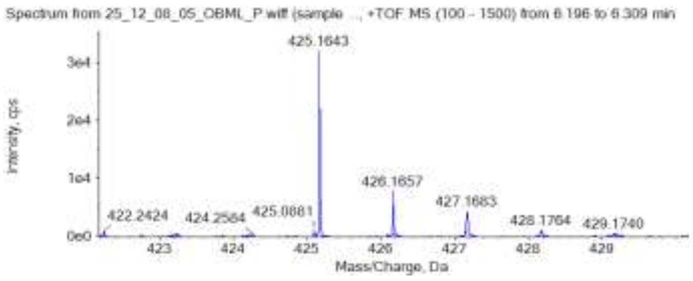

296.1899 / 6.31 [M+H]+

(Mass/FragMass/RT/Isotope/Library/Formula/Ion Ratio)

**Retention Time:** 6.30 minutes	**Exp RT:** 6.31 minutes
**Precursor m/z :** 296.1899	**Analyte Name:**
**Fit (%)** 97.9% **RFit (%)** 83.0%	296.1899 / 6.31 [M+H]+

Collision Energy = 35 ± 15 eV

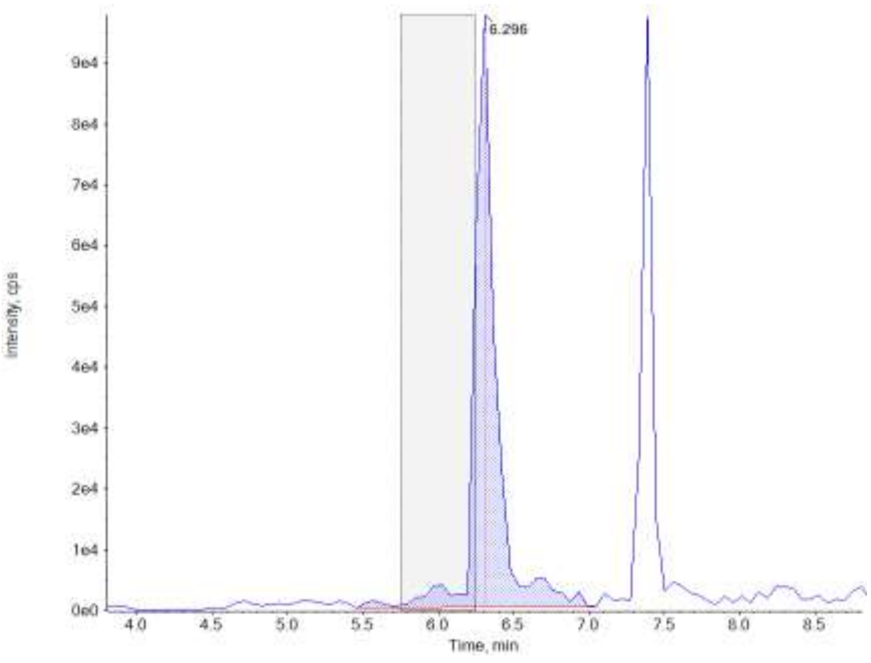

Acquired / Library MSMS

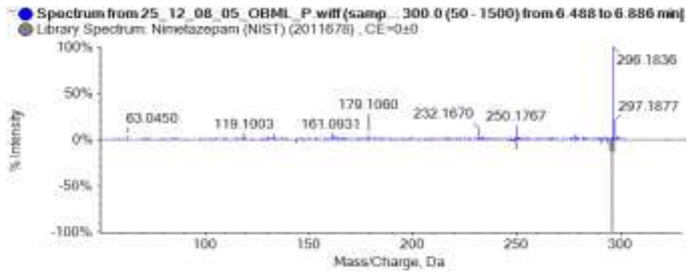

Acquired / Theoretical MS

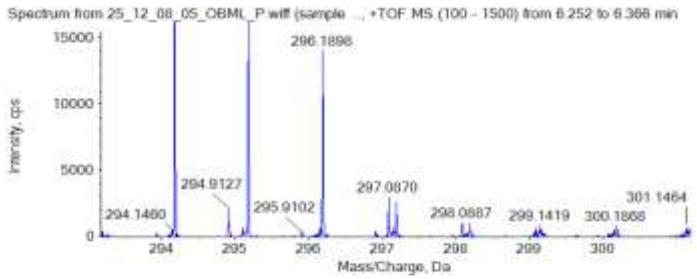

294.1766 / 6.42 (Mass/FragMass/RT/Isotope/Library/Formula/Ion Ratio)

Retention Time: 6.40 minutes
Precursor m/z : 294.1766
Fit (%) 52.9% **RFit (%)** 95.7%

Exp RT: 6.42 minutes
Analyte Name:
294.1766 / 6.42

Collision Energy = 35 ± 15 eV

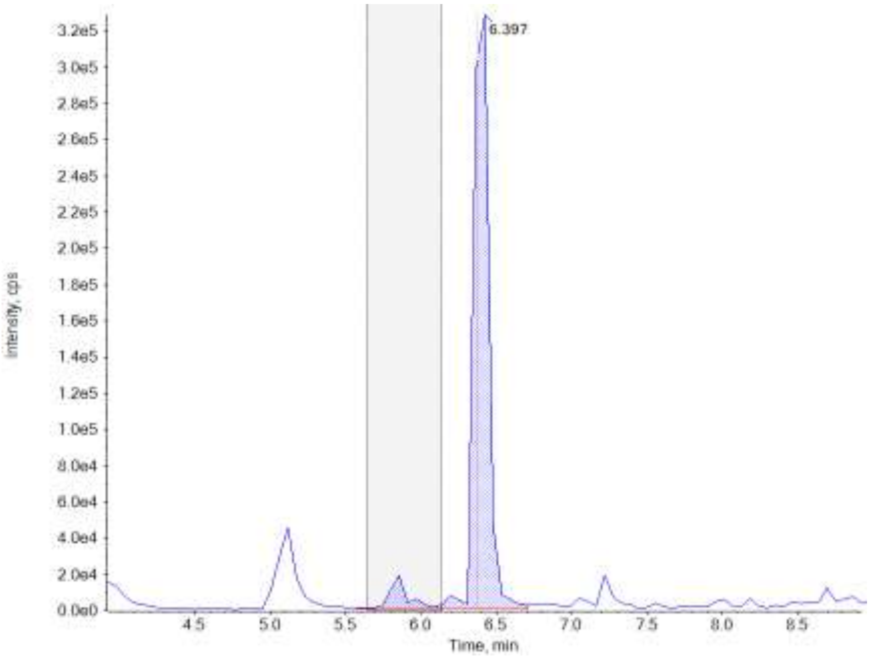

Acquired / Library MSMS

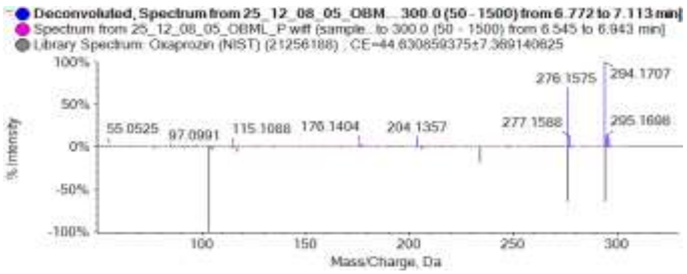

Acquired / Theoretical MS

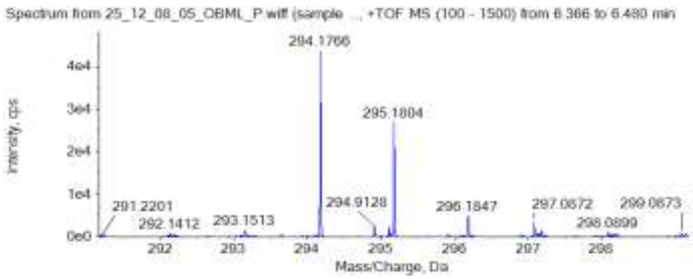

359.1745 / 6.42 (Mass/FragMass/RT/Isotope/Library/Formula/Ion Ratio)

Retention Time: 6.42 minutes
Precursor m/z : 359.1745
Fit (%) 98.4% **RFit (%)** 99.3%

Exp RT: 6.42 minutes
Analyte Name:
359.1745 / 6.42

Collision Energy = 35 ± 15 eV

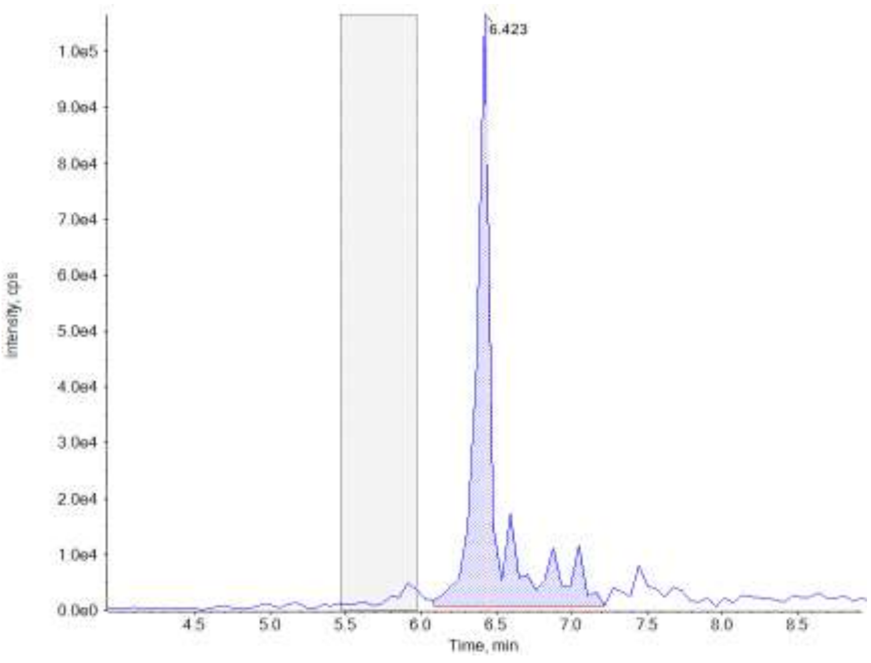

Acquired / Library MSMS

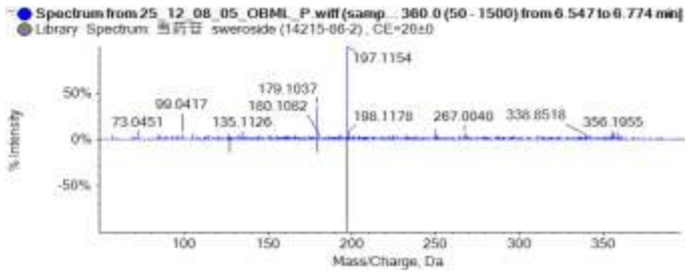

Acquired / Theoretical MS

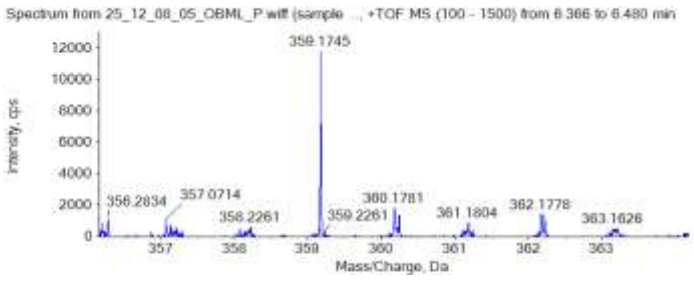

331.1210 / 6.59 (Mass/FragMass/RT/Isotope/Library/Formula/Ion Ratio)

Retention Time: 6.59 minutes
Precursor m/z : 331.1210
Fit (%) 100.0% **RFit (%)** 22.0%

Exp RT: 6.59 minutes
Analyte Name:
331.1210 / 6.59

Collision Energy = 35 ± 15 eV

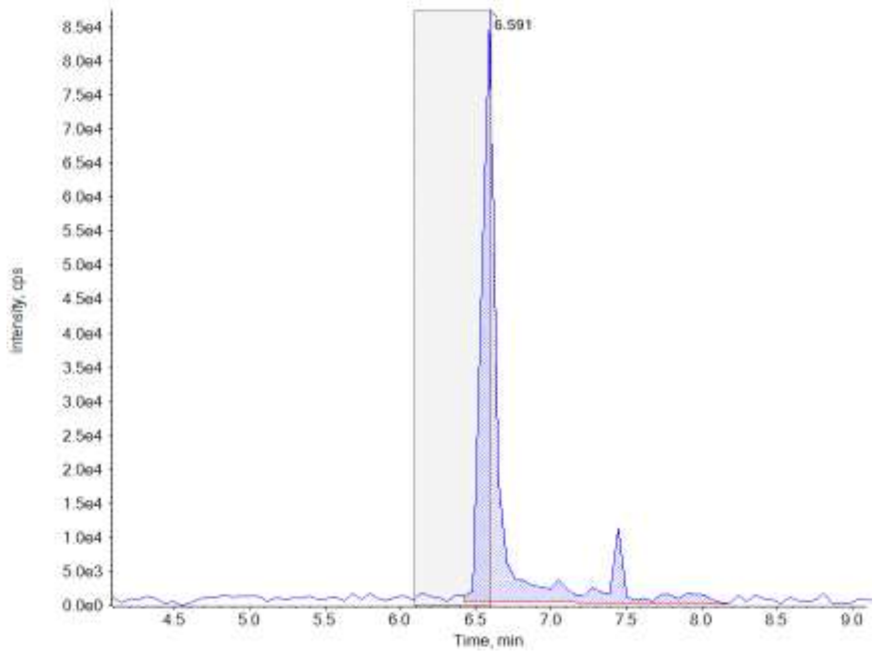

Acquired / Library MSMS

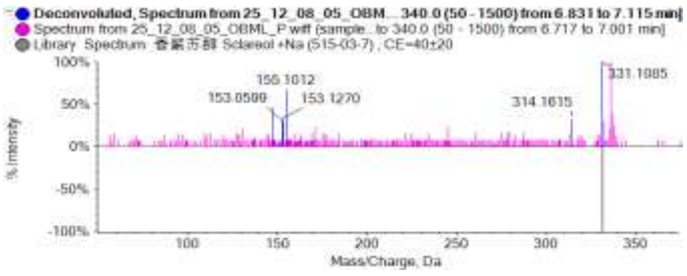

Acquired / Theoretical MS

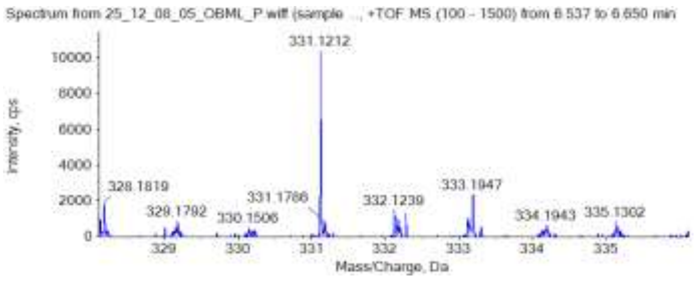

264.1510 / 6.99 (Mass/FragMass/RT/Isotope/Library/Formula/Ion Ratio)

Retention Time: 7.02 minutes
Precursor m/z : 264.1510
Fit (%) 87.0% **RFit (%)** 96.4%

Exp RT: 6.99 minutes
Analyte Name:
264.1510 / 6.99

Collision Energy = 35 ± 15 eV

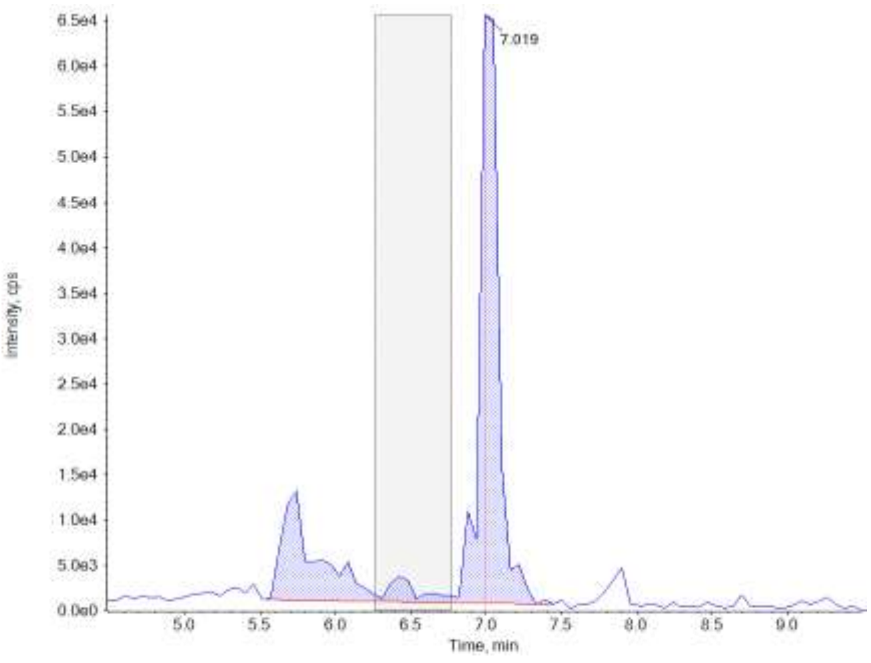

Acquired / Library MSMS

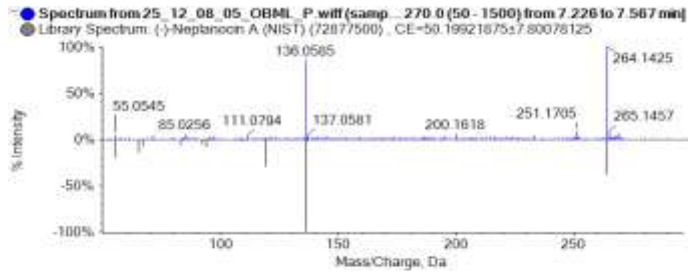

Acquired / Theoretical MS

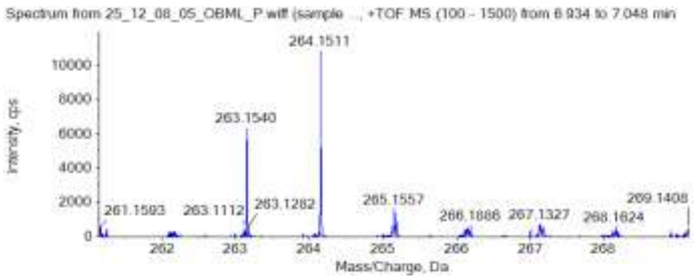

227.1708 / 7.10 (Mass/FragMass/RT/Isotope/Library/Formula/Ion Ratio)

Retention Time: 7.10 minutes
Precursor m/z : 227.1708
Fit (%) 98.4% **RFit (%)** 44.8%

Exp RT: 7.11 minutes
Analyte Name:
227.1708 / 7.10

Collision Energy = 35 ± 15 eV

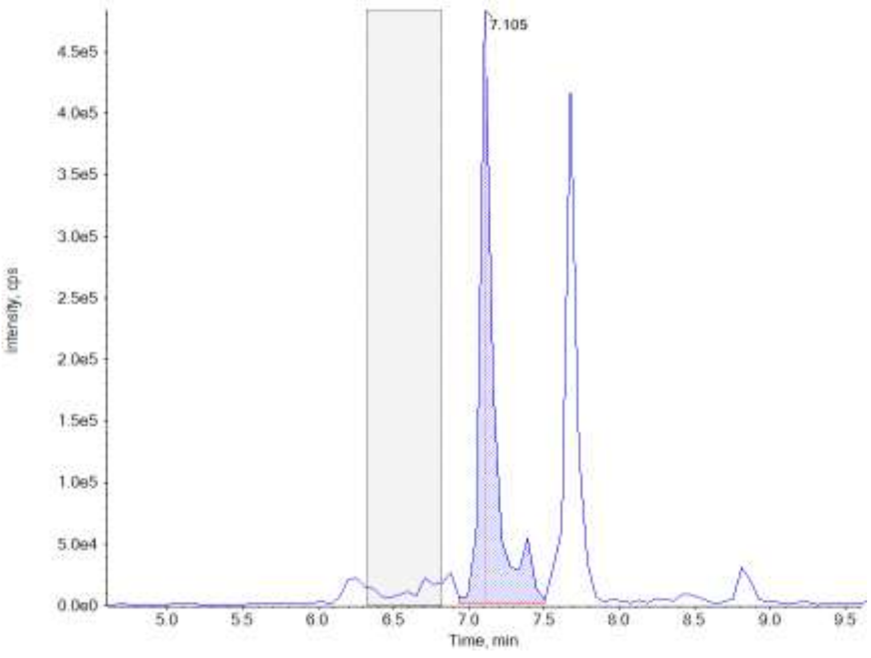

Acquired / Library MSMS

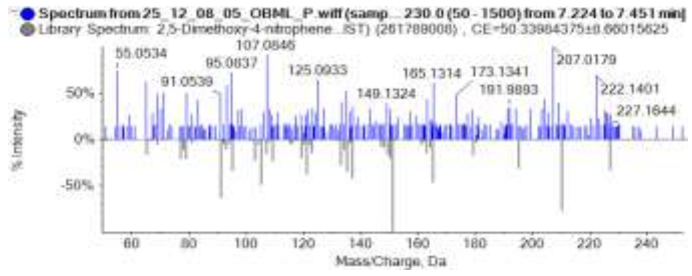

Acquired / Theoretical MS

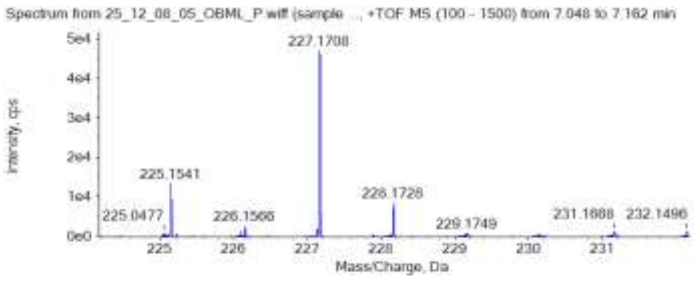

389.2230 / 7.10 (Mass/FragMass/RT/Isotope/Library/Formula/Ion Ratio)

Retention Time: 7.10 minutes
Precursor m/z : 389.2230
Fit (%) 79.8% **RFit (%)** 86.6%

Exp RT: 7.11 minutes
Analyte Name:
389.2230 / 7.10

Collision Energy = 35 ± 15 eV

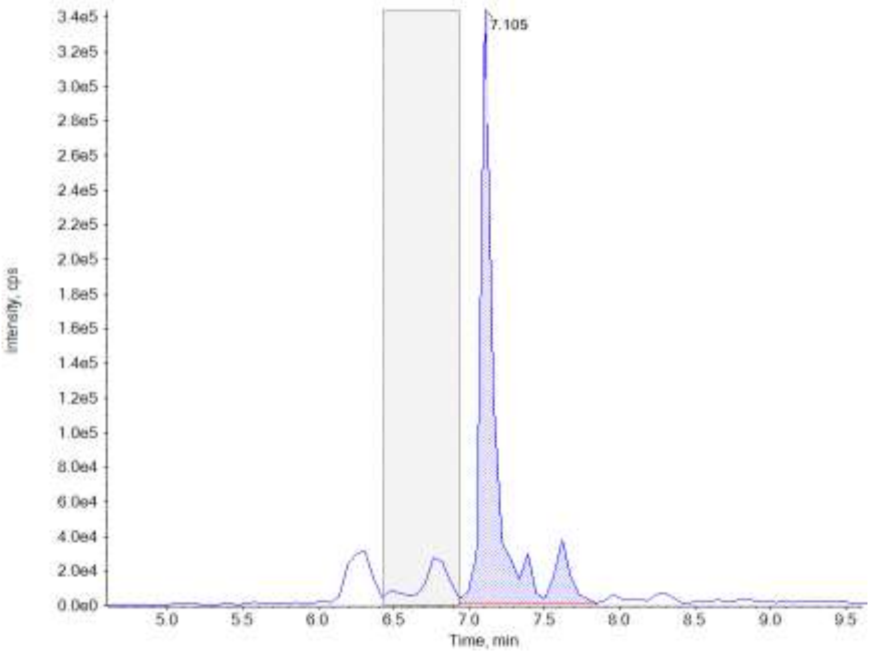

Acquired / Library MSMS

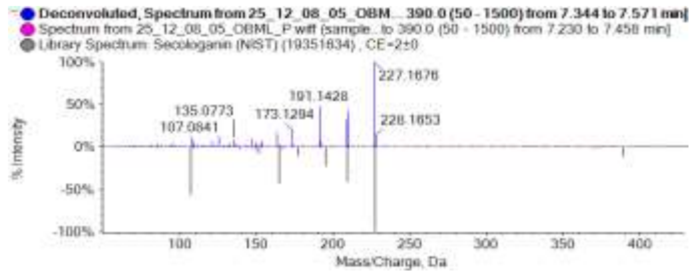

Acquired / Theoretical MS

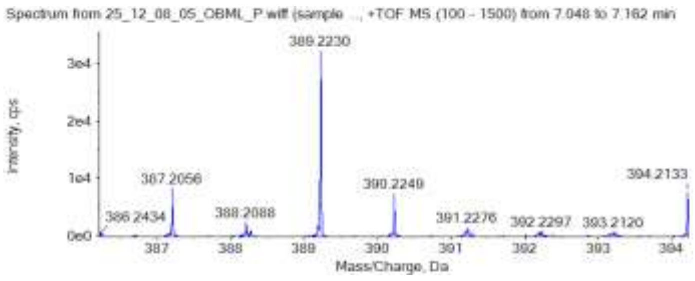

438.2454 / 7.16 (Mass/FragMass/RT/Isotope/Library/Formula/Ion Ratio)

Retention Time: 7.16 minutes
Precursor m/z : 438.2454
Fit (%) 87.7% **RFit (%)** 90.1%

Exp RT: 7.16 minutes
Analyte Name:
438.2454 / 7.16

Collision Energy = 35 ± 15 eV

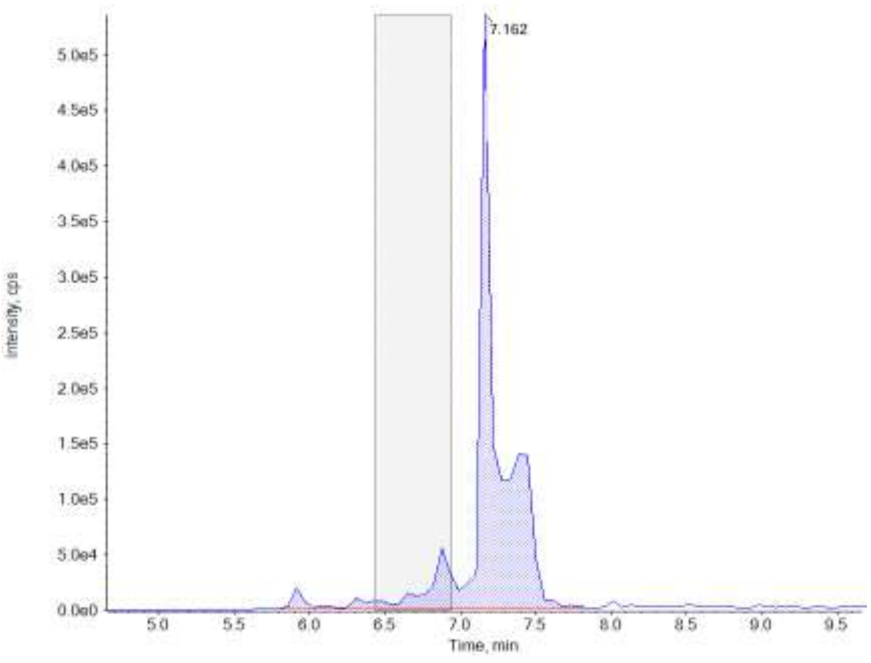

Acquired / Library MSMS

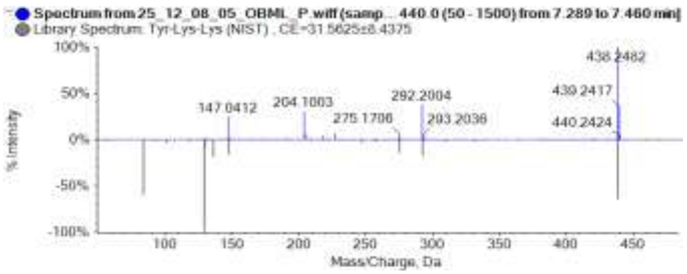

Acquired / Theoretical MS

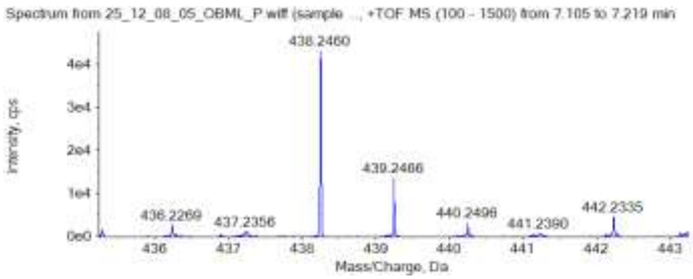

585.2929 / 7.16 [M+K]+ (Mass/FragMass/RT/Isotope/Library/Formula/Ion Ratio)

Retention Time: 7.18 minutes
Precursor m/z : 585.2929
Fit (%) 70.0% **RFit (%)** 100.0%

Exp RT: 7.16 minutes
Analyte Name:
585.2929 / 7.16 [M+K]+

Collision Energy = 35 ± 15 eV

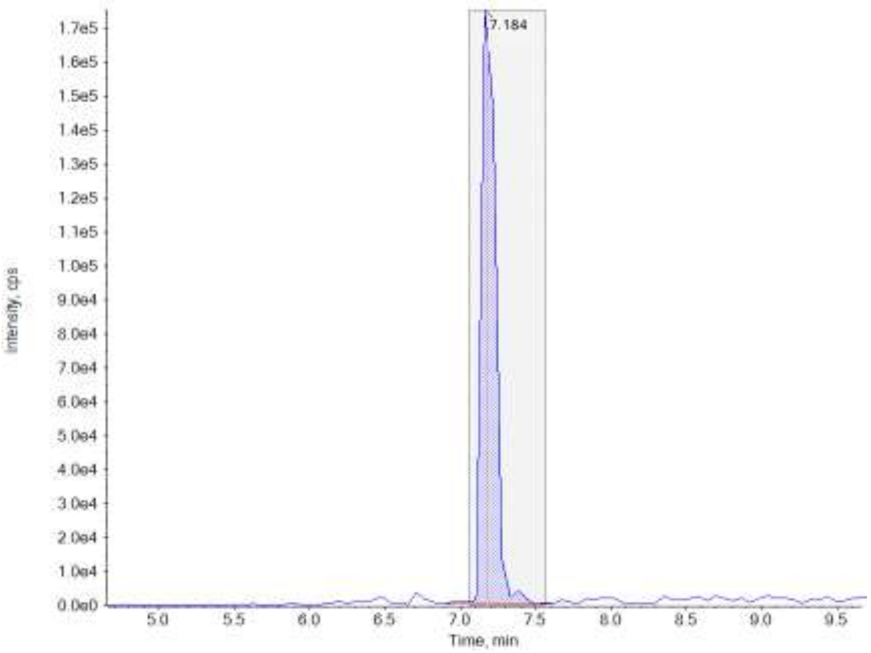

Acquired / Library MSMS

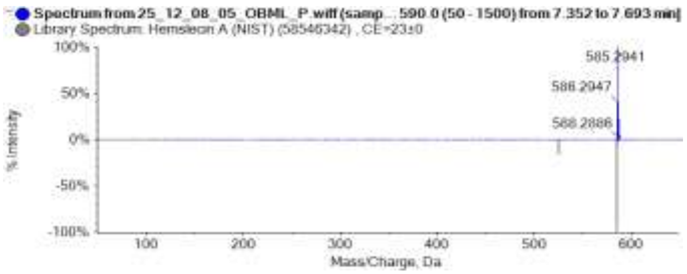

Acquired / Theoretical MS

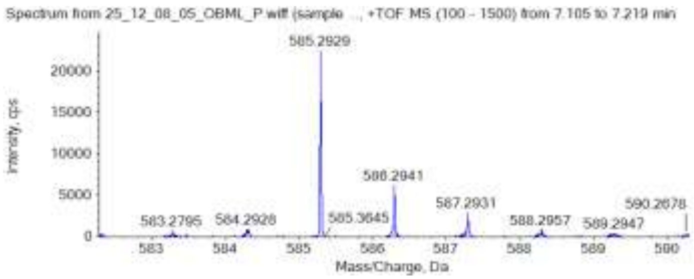

547.3369 / 7.16 [M+H]+ (Mass/FragMass/RT/Isotope/Library/Formula/Ion Ratio)

Retention Time: 7.18 minutes
Precursor m/z : 547.3369
Fit (%) 96.6% **RFit (%)** 100.0%

Exp RT: 7.16 minutes
Analyte Name:
547.3369 / 7.16 [M+H]+

Collision Energy = 35 ± 15 eV

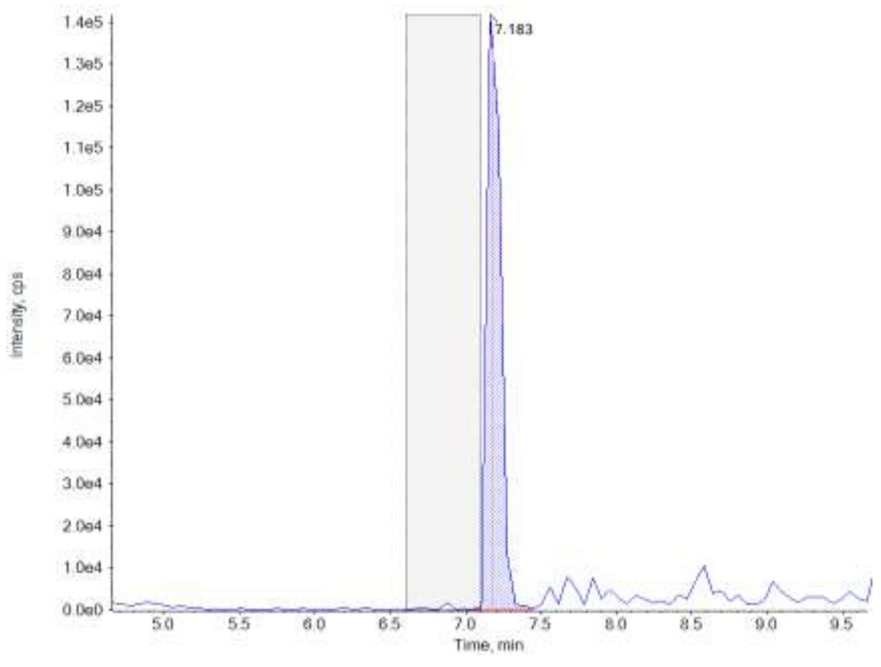

Acquired / Library MSMS

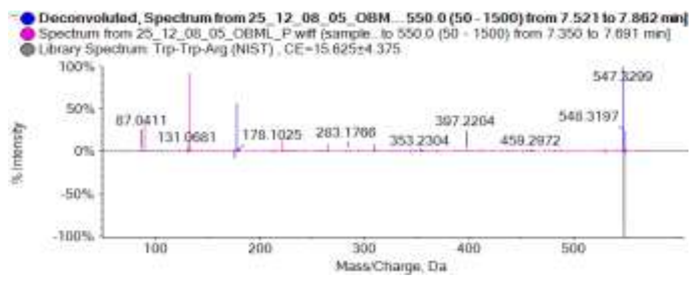

Acquired / Theoretical MS

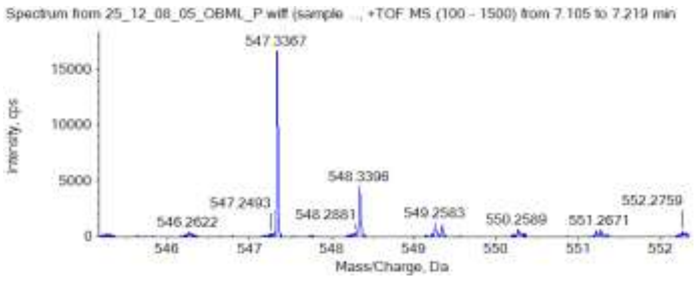

629.3214 / 7.39 (Mass/FragMass/RT/Isotope/Library/Formula/Ion Ratio)

Retention Time: 7.39 minutes
Precursor m/z : 629.3214
Fit (%) 98.7% **RFit (%)** 98.7%

Exp RT: 7.39 minutes
Analyte Name:
629.3214 / 7.39

Collision Energy = 35 ± 15 eV

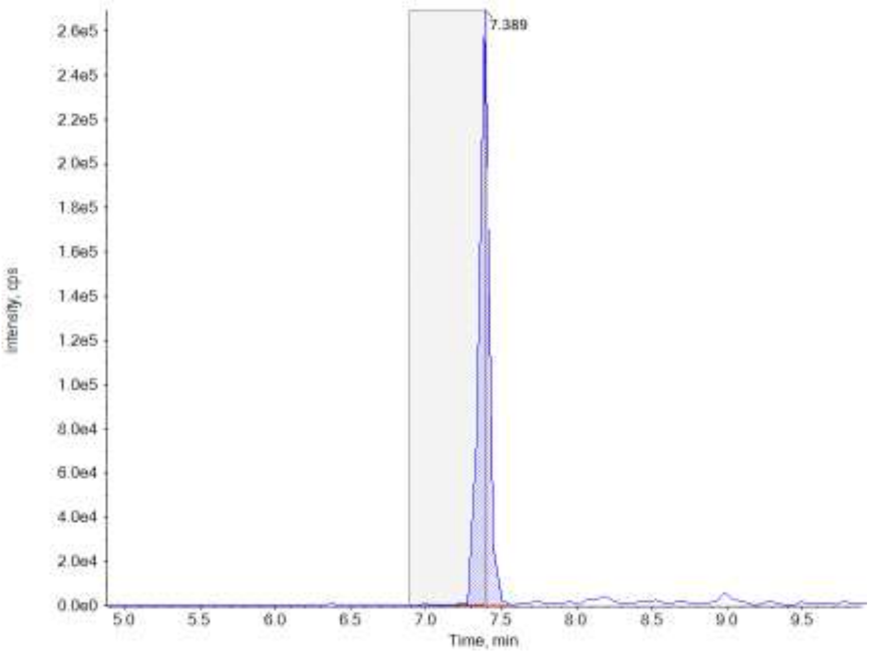

Acquired / Library MSMS

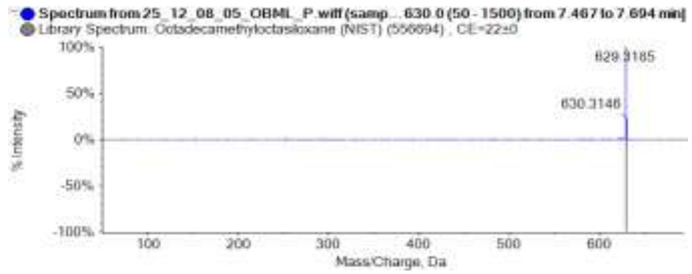

Acquired / Theoretical MS

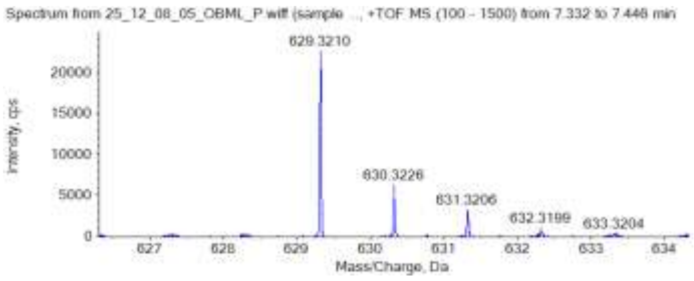

636.4251 / 7.39 (Mass/FragMass/RT/Isotope/Library/Formula/Ion Ratio)

Retention Time: 7.39 minutes
Precursor m/z : 636.4251
Fit (%) 90.4% **RFit (%)** 86.5%

Exp RT: 7.39 minutes
Analyte Name:
636.4251 / 7.39

Collision Energy = 35 ± 15 eV

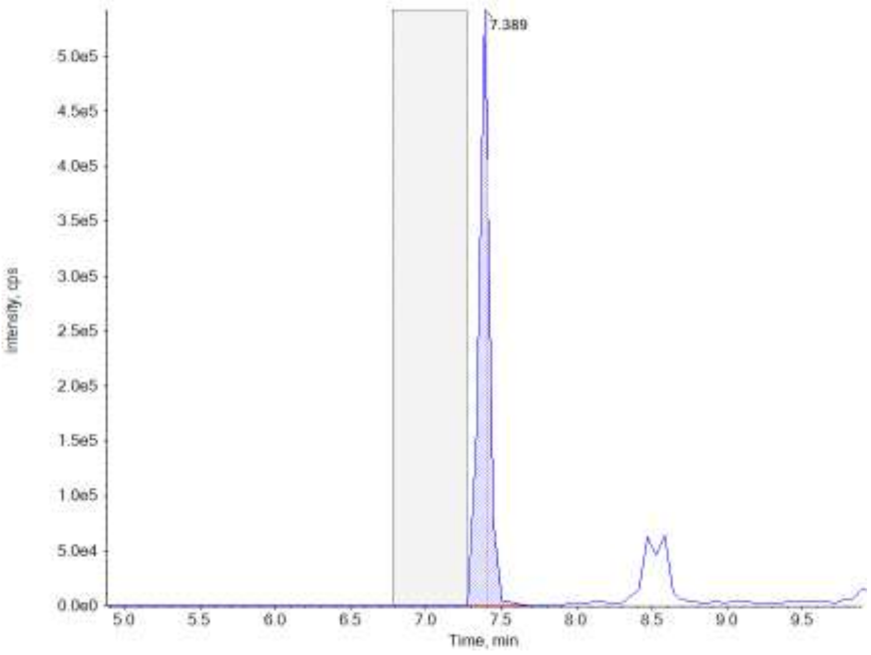

Acquired / Library MSMS

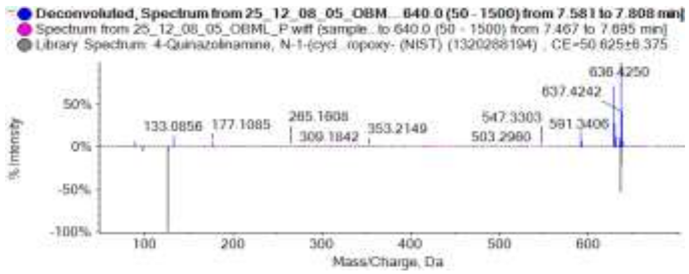

Acquired / Theoretical MS

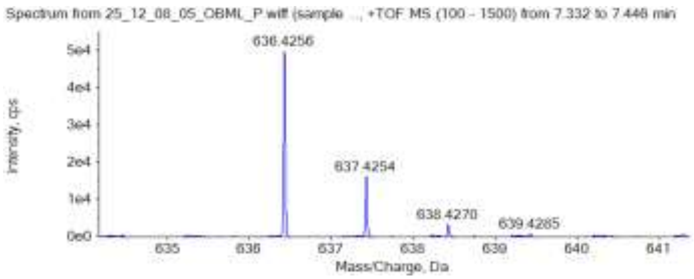

652.4213 / 7.56 (Mass/FragMass/RT/Isotope/Library/Formula/Ion Ratio)

**Retention Time:** 7.56 minutes	**Exp RT:** 7.56 minutes
**Precursor m/z :** 652.4213	**Analyte Name:**
**Fit (%)** 79.0% **RFit (%)** 83.6%	652.4213 / 7.56

Collision Energy = 35 ± 15 eV

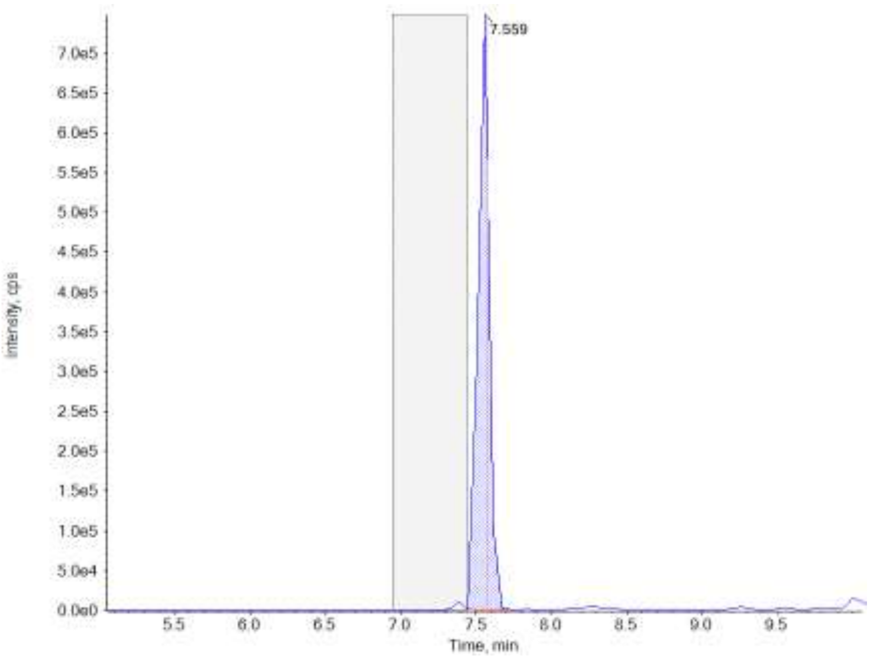

Acquired / Library MSMS

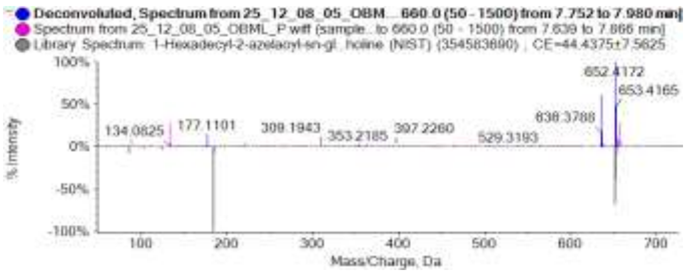

Acquired / Theoretical MS

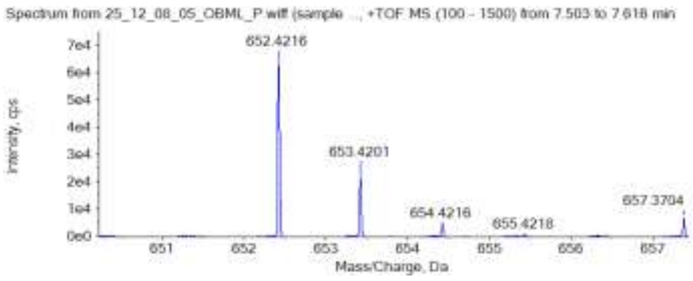

673.3463 / 7.56 (Mass/FragMass/RT/Isotope/Library/Formula/Ion Ratio)

**Retention Time:** 7.56 minutes	**Exp RT:** 7.56 minutes
**Precursor m/z :** 673.3463	**Analyte Name:**
**Fit (%)** 100.0% **RFit (%)** 91.5%	673.3463 / 7.56

Collision Energy = 35 ± 15 eV

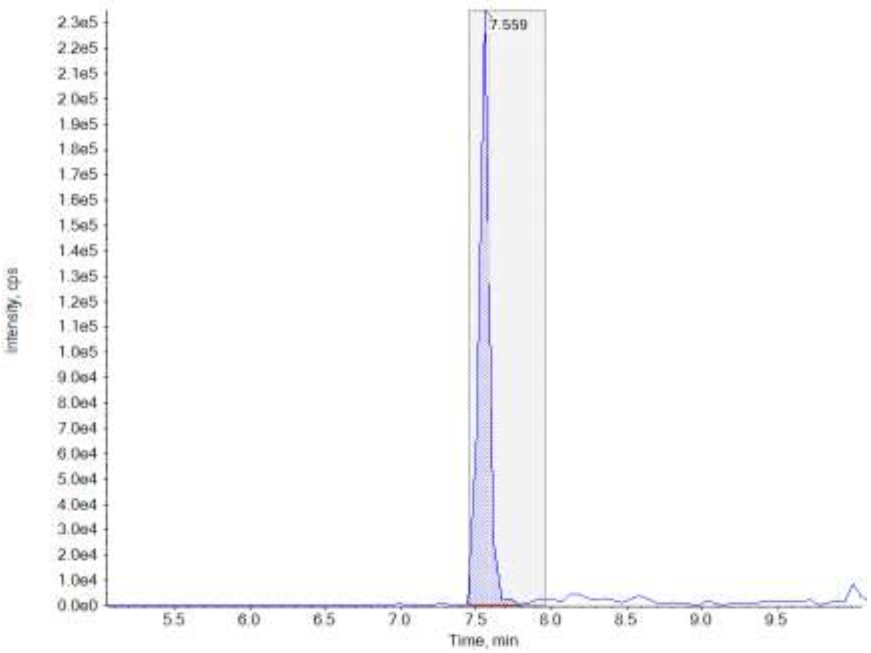

Acquired / Library MSMS

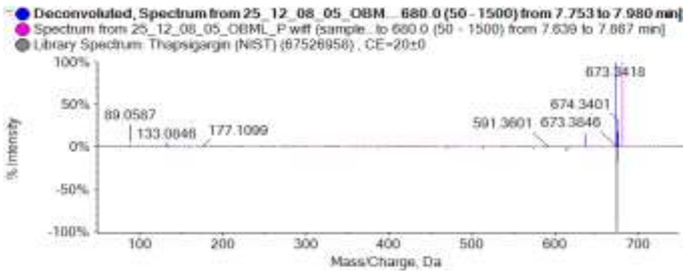

Acquired / Theoretical MS

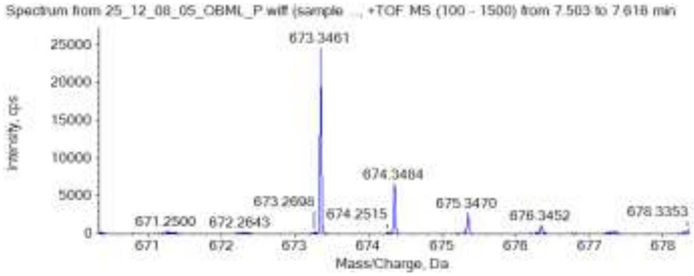

680.4509 / 7.56 (Mass/FragMass/RT/Isotope/Library/Formula/Ion Ratio)

Retention Time: 7.56 minutes
Precursor m/z : 680.4509
Fit (%) 28.2% **RFit (%)** 97.7%

Exp RT: 7.56 minutes
Analyte Name:
680.4509 / 7.56

Collision Energy = 35 ± 15 eV

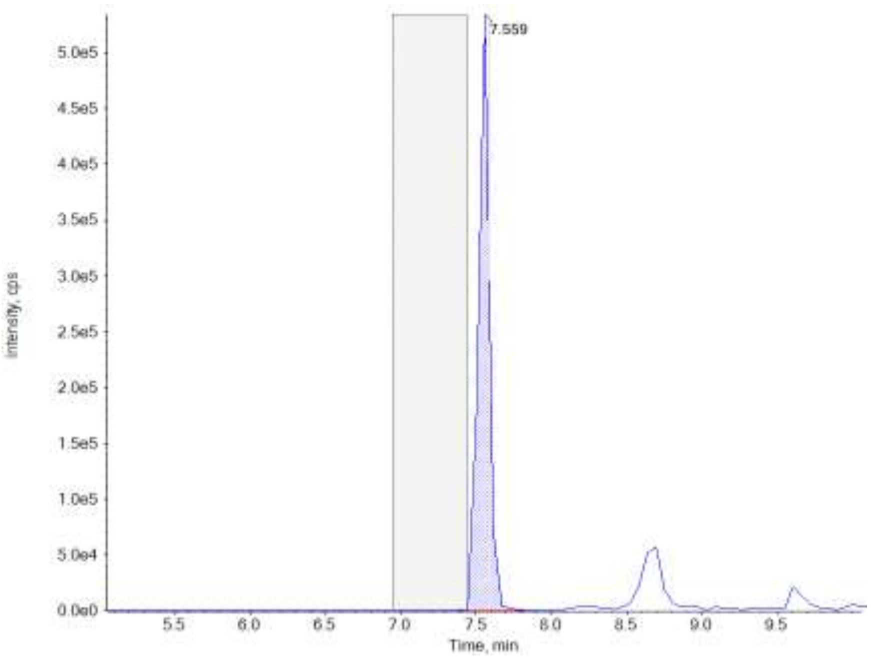

Acquired / Library MSMS

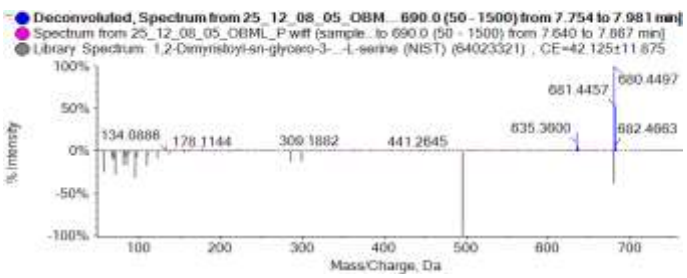

Acquired / Theoretical MS

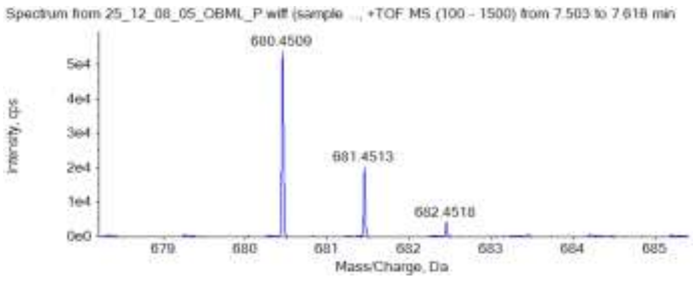

322.2076 / 7.62 (Mass/FragMass/RT/Isotope/Library/Formula/Ion Ratio)

Retention Time: 7.62 minutes
Precursor m/z : 322.2076
Fit (%) 99.9% **RFit (%)** 64.7%

Exp RT: 7.62 minutes
Analyte Name:
322.2076 / 7.62

Collision Energy = 35 ± 15 eV

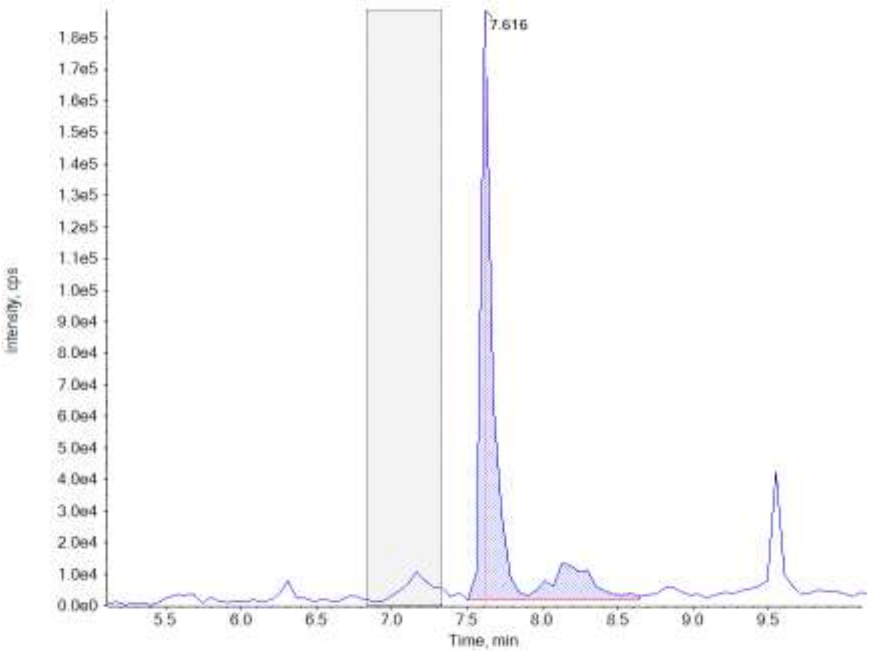

Acquired / Library MSMS

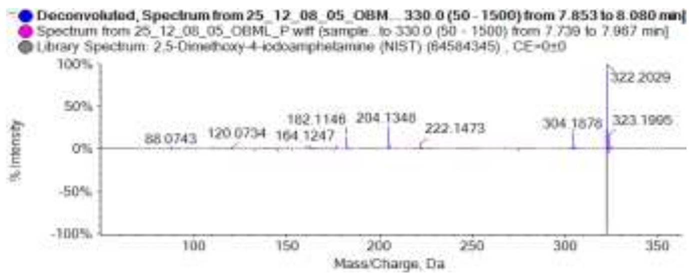

Acquired / Theoretical MS

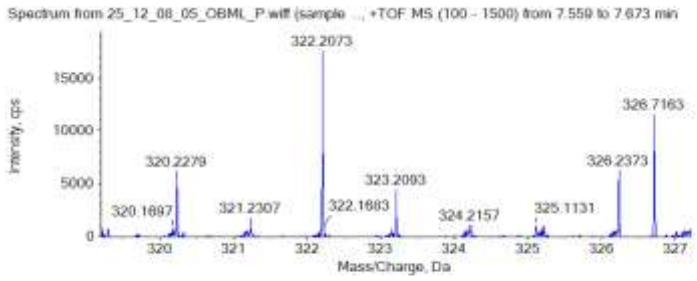

227.1707 / 7.67 (Mass/FragMass/RT/Isotope/Library/Formula/Ion Ratio)

Retention Time: 7.67 minutes
Precursor m/z : 227.1707
Fit (%) 97.7% **RFit (%)** 80.3%

Exp RT: 7.67 minutes
Analyte Name:
227.1707 / 7.67

Collision Energy = 35 ± 15 eV

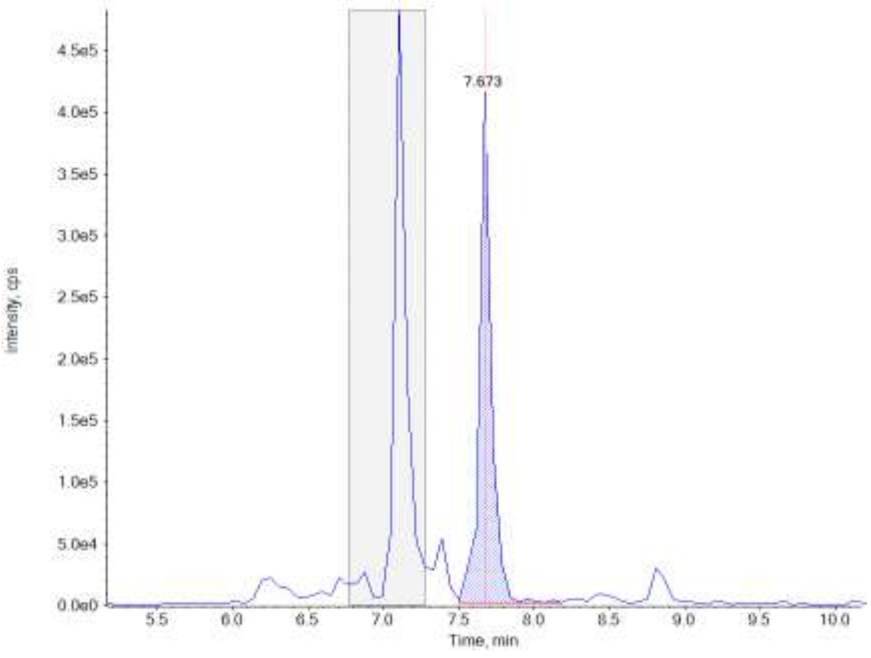

Acquired / Library MSMS

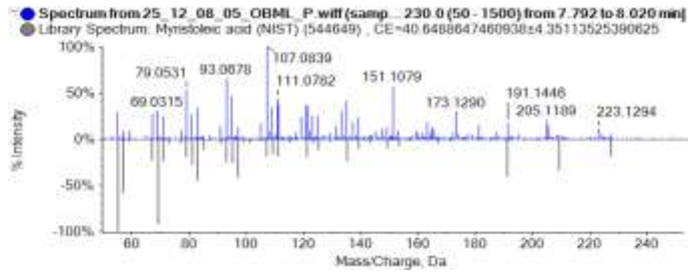

Acquired / Theoretical MS

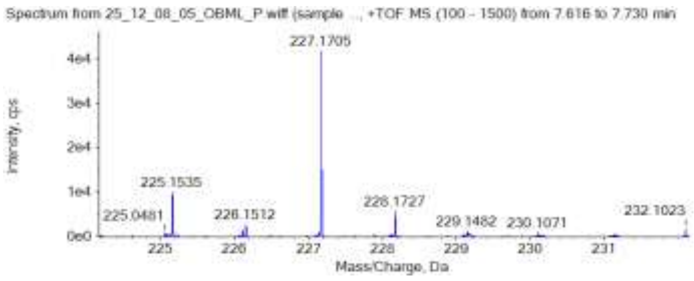

209.1588 / 7.73 (Mass/FragMass/RT/Isotope/Library/Formula/Ion Ratio)

Retention Time: 7.79 minutes
Precursor m/z : 209.1588
Fit (%) 76.5% **RFit (%)** 100.0%

Exp RT: 7.73 minutes
Analyte Name:
209.1588 / 7.73

Collision Energy = 35 ± 15 eV

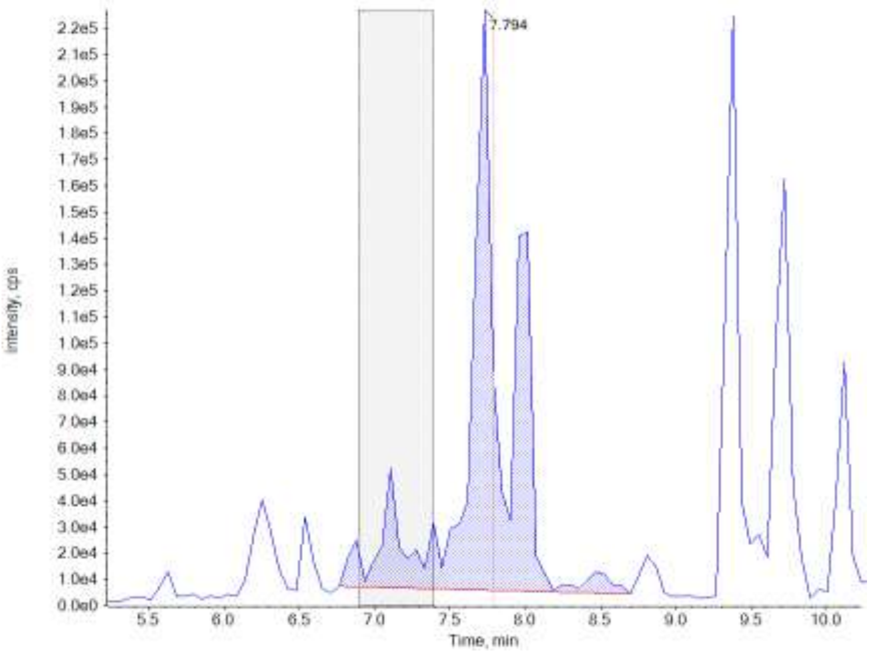

Acquired / Library MSMS

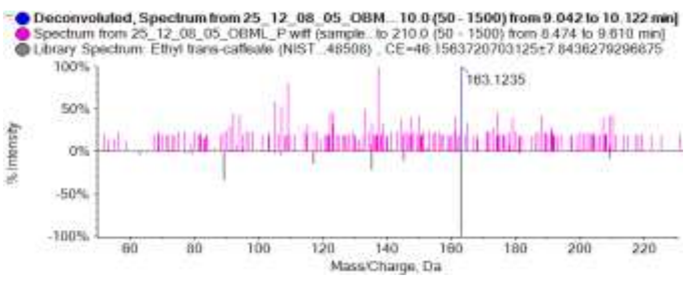

Acquired / Theoretical MS

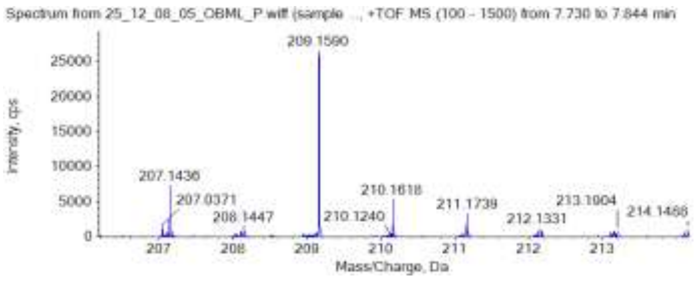

364.2035 / 7.73 (Mass/FragMass/RT/Isotope/Library/Formula/Ion Ratio)

**Retention Time:** 7.51 minutes<br>**Precursor m/z :** 364.2035<br>**Fit (%)** 100.0% **RFit (%)** 32.2%	**Exp RT:** 7.73 minutes<br>**Analyte Name:**<br>364.2035 / 7.73

Collision Energy = 35 ± 15 eV

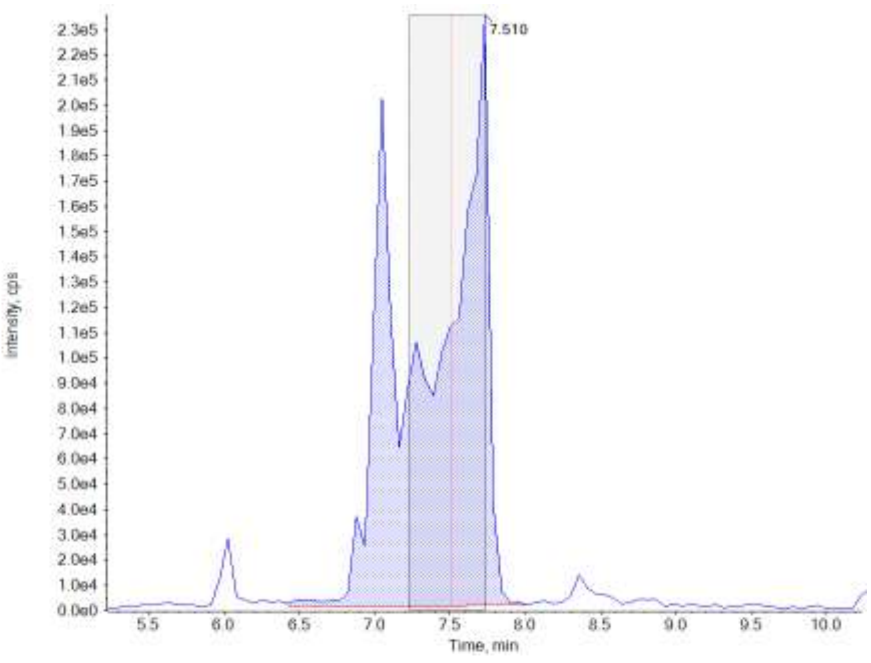

Acquired / Library MSMS

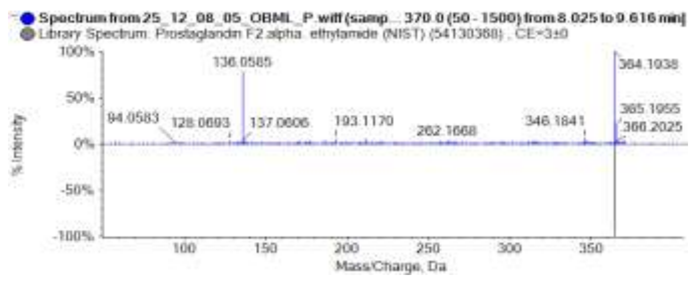

Acquired / Theoretical MS

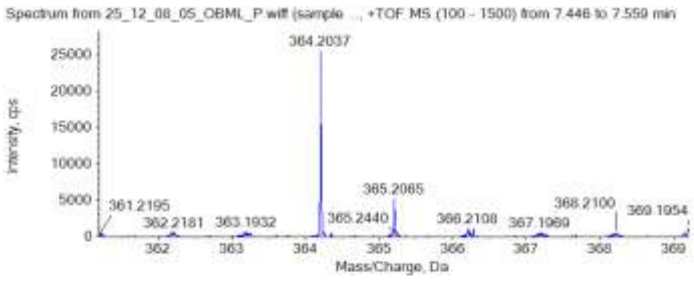

371.2118 / 7.73 (Mass/FragMass/RT/Isotope/Library/Formula/Ion Ratio)

Retention Time: 7.73 minutes
Precursor m/z : 371.2118
Fit (%) 79.8% **RFit (%)** 96.7%

Exp RT: 7.73 minutes
Analyte Name:
371.2118 / 7.73

Collision Energy = 35 ± 15 eV

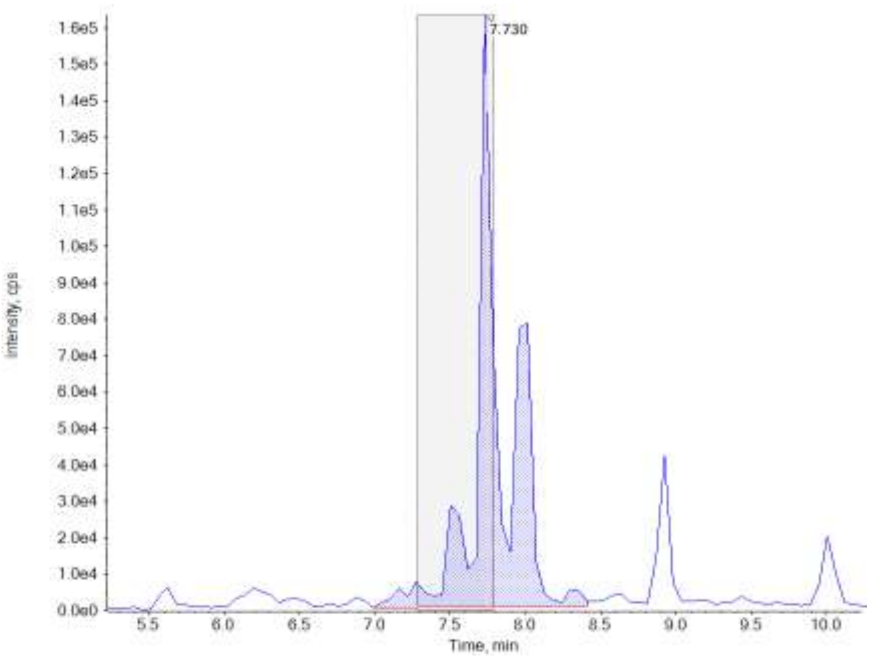

Acquired / Library MSMS

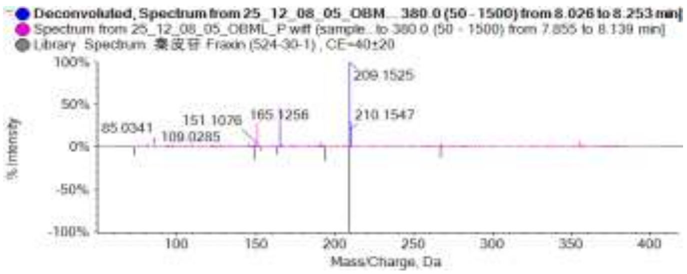

Acquired / Theoretical MS

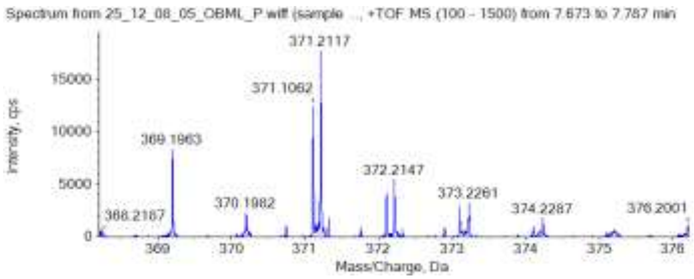

346.1943 / 7.84 (Mass/FragMass/RT/Isotope/Library/Formula/Ion Ratio)

Retention Time: 7.84 minutes
Precursor m/z : 346.1943
Fit (%) 99.4% **RFit (%)** 87.6%

Exp RT: 7.84 minutes
Analyte Name:
346.1943 / 7.84

Collision Energy = 35 ± 15 eV

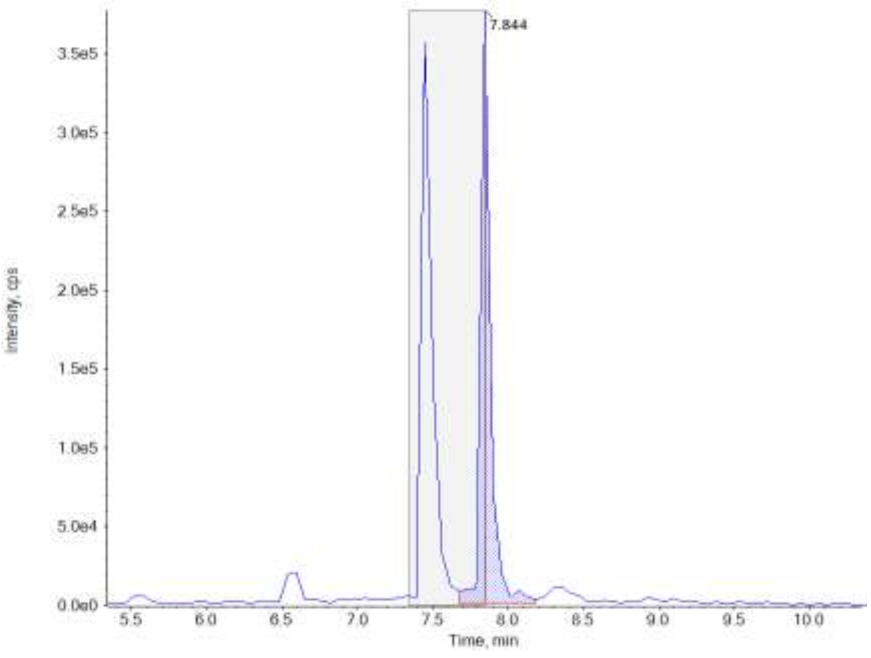

Acquired / Library MSMS

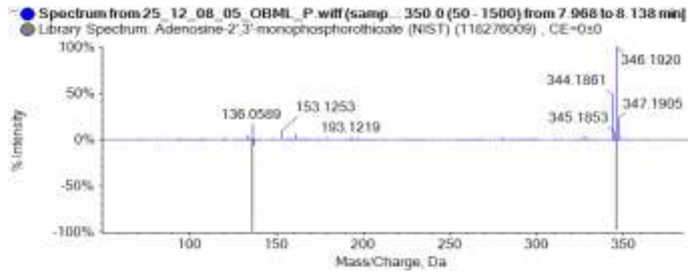

Acquired / Theoretical MS

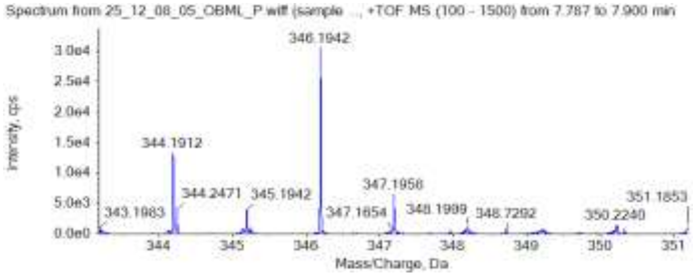

179.1121 / 7.90 (Mass/FragMass/RT/Isotope/Library/Formula/Ion Ratio)

Retention Time: 7.90 minutes
Precursor m/z : 179.1121
Fit (%) 100.0% **RFit (%)** 97.5%

Exp RT: 7.90 minutes
Analyte Name:
179.1121 / 7.90

Collision Energy = 35 ± 15 eV

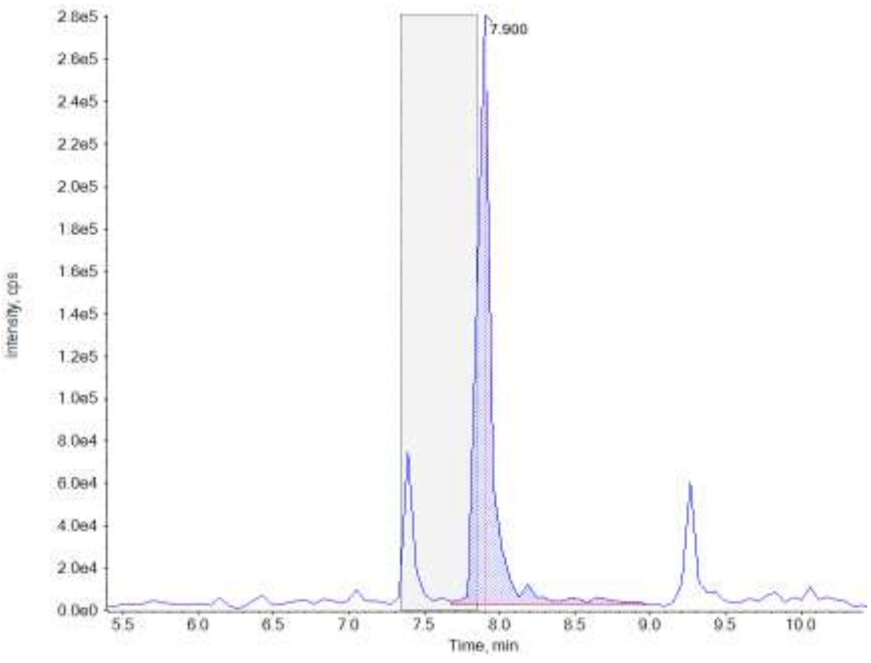

Acquired / Library MSMS

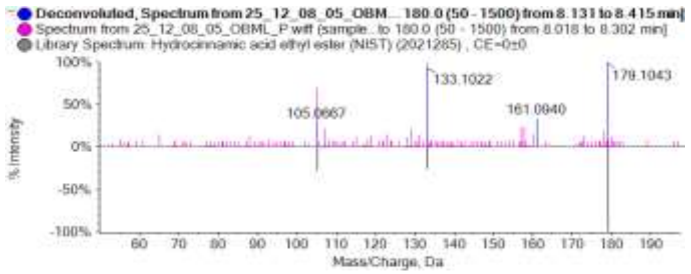

Acquired / Theoretical MS

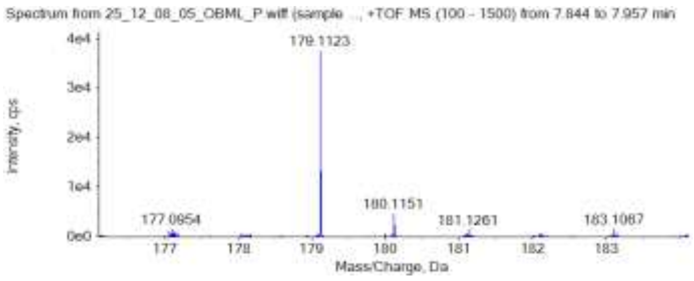

193.1645 / 8.07 (Mass/FragMass/RT/Isotope/Library/Formula/Ion Ratio)

Retention Time: 8.07 minutes
Precursor m/z : 193.1645
Fit (%) 93.7% **RFit (%)** 55.3%

Exp RT: 8.07 minutes
Analyte Name:
193.1645 / 8.07

Collision Energy = 35 ± 15 eV

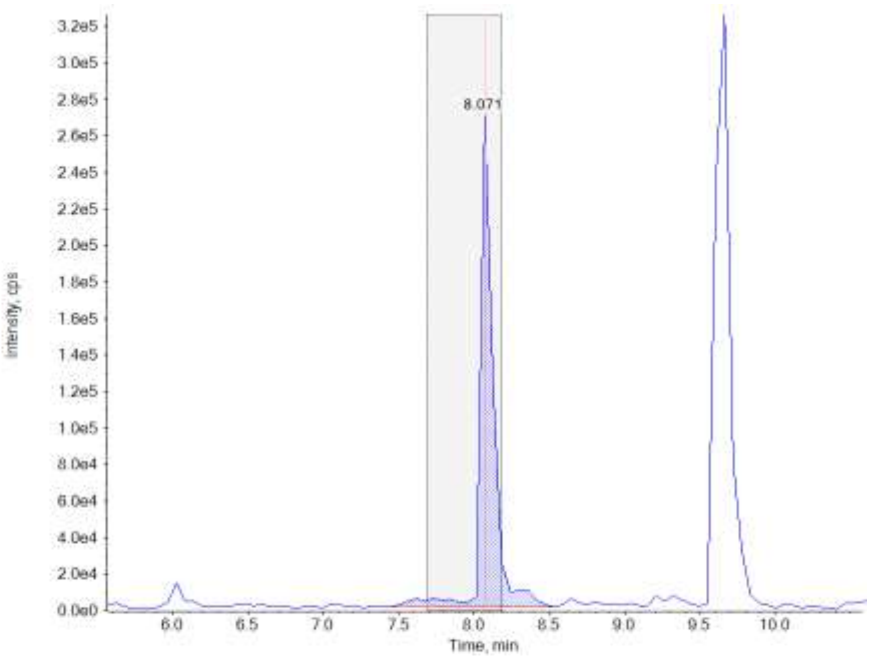

Acquired / Library MSMS

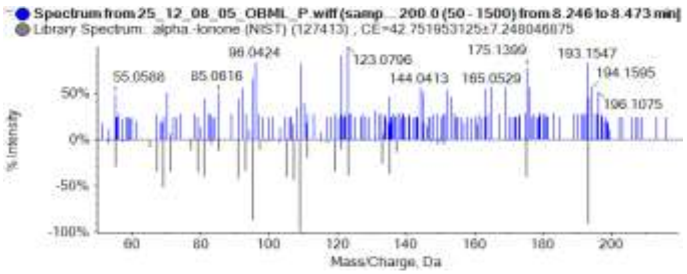

Acquired / Theoretical MS

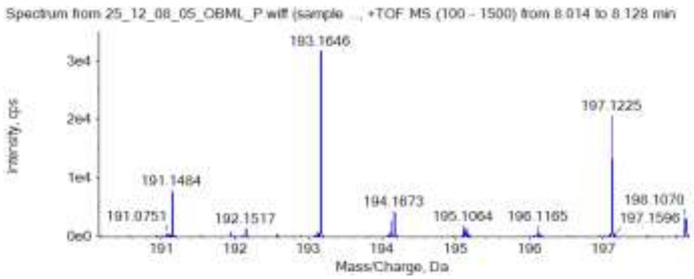

211.1794 / 8.07

(Mass/FragMass/RT/Isotope/Library/Formula/Ion Ratio)

Retention Time: 8.07 minutes
Precursor m/z : 211.1794
Fit (%) 62.4% **RFit (%)** 94.5%

Exp RT: 8.07 minutes
Analyte Name:
211.1794 / 8.07

Collision Energy = 35 ± 15 eV

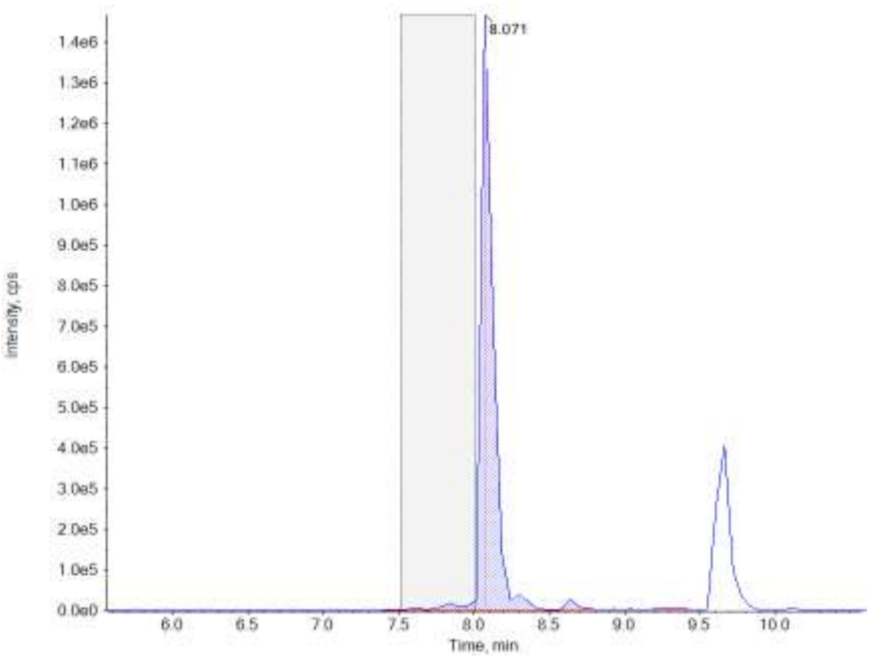

Acquired / Library MSMS

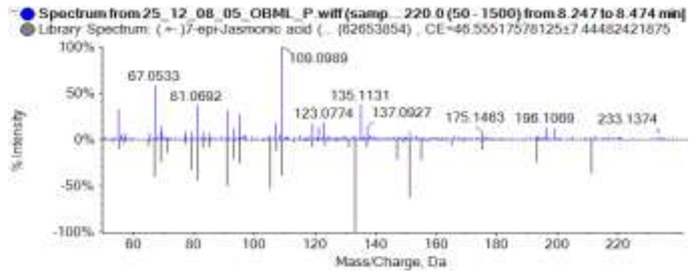

Acquired / Theoretical MS

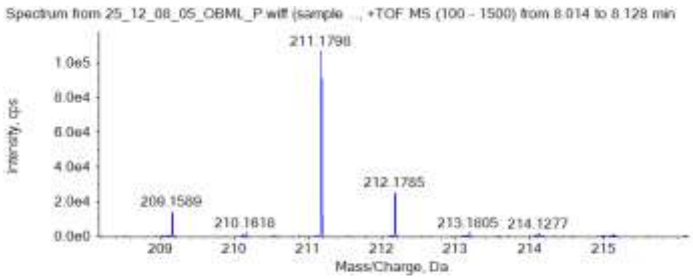

146.0658 / 8.13 (Mass/FragMass/RT/Isotope/Library/Formula/Ion Ratio)

**Retention Time:** 8.13 minutes	**Exp RT:** 8.13 minutes
**Precursor m/z :** 146.0658	**Analyte Name:**
**Fit (%)** 96.8% **RFit (%)** 100.0%	146.0658 / 8.13

Collision Energy = 35 ± 15 eV

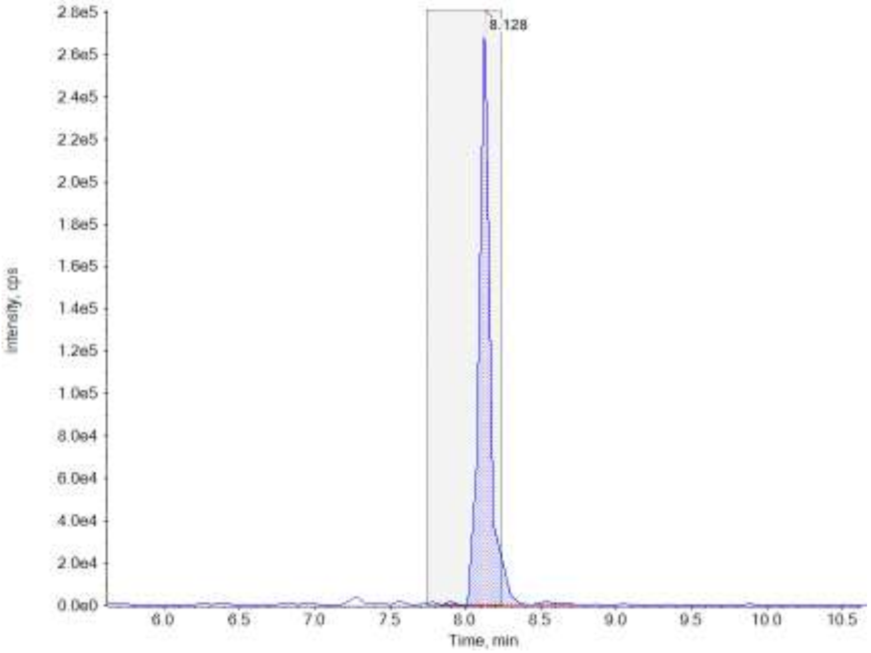

Acquired / Library MSMS

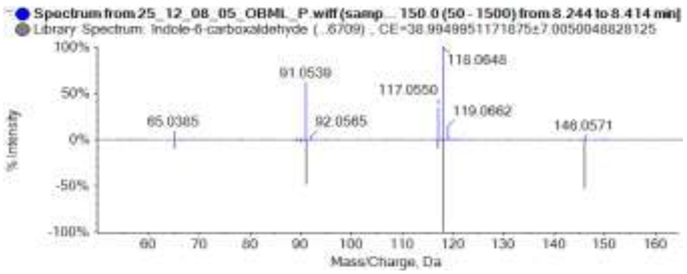

Acquired / Theoretical MS

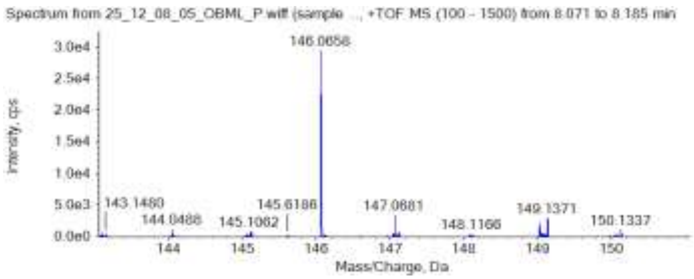

225.1536 / 8.30 (Mass/FragMass/RT/Isotope/Library/Formula/Ion Ratio)

**Retention Time:** 8.28 minutes	**Exp RT:** 8.30 minutes
**Precursor m/z :** 225.1536	**Analyte Name:**
**Fit (%)** 91.0% **RFit (%)** 74.4%	225.1536 / 8.30

Collision Energy = 35 ± 15 eV

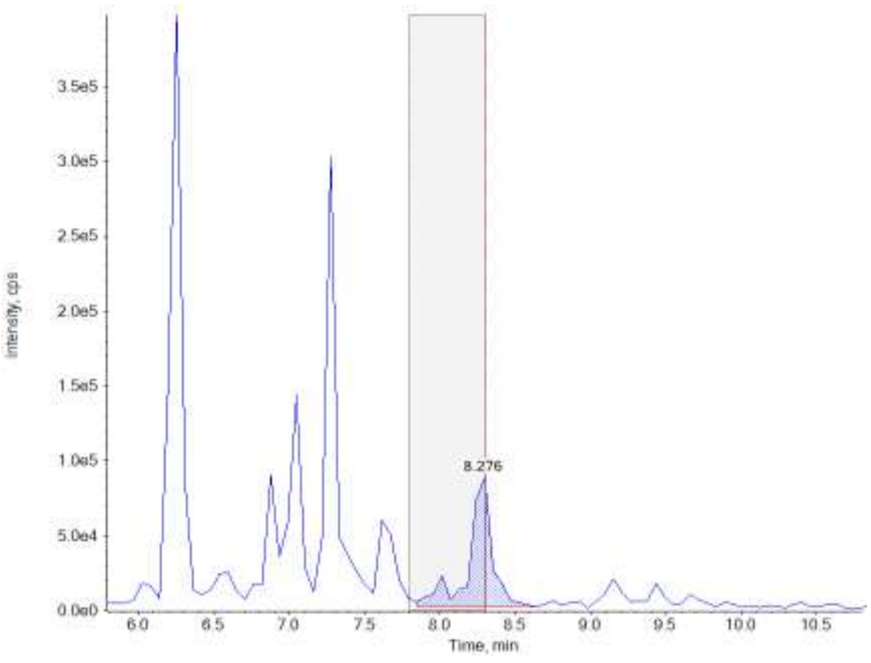

Acquired / Library MSMS

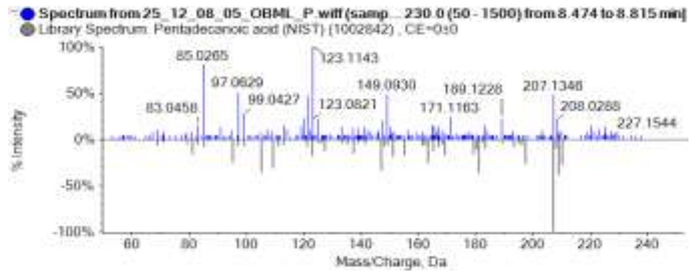

Acquired / Theoretical MS

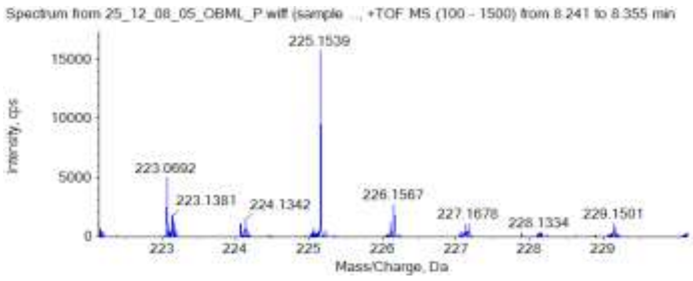

213.1548 / 8.70 (Mass/FragMass/RT/Isotope/Library/Formula/Ion Ratio)

Retention Time: 8.70 minutes
Precursor m/z : 213.1548
Fit (%) 98.3% **RFit (%)** 61.0%

Exp RT: 8.70 minutes
Analyte Name:
213.1548 / 8.70

Collision Energy = 35 ± 15 eV

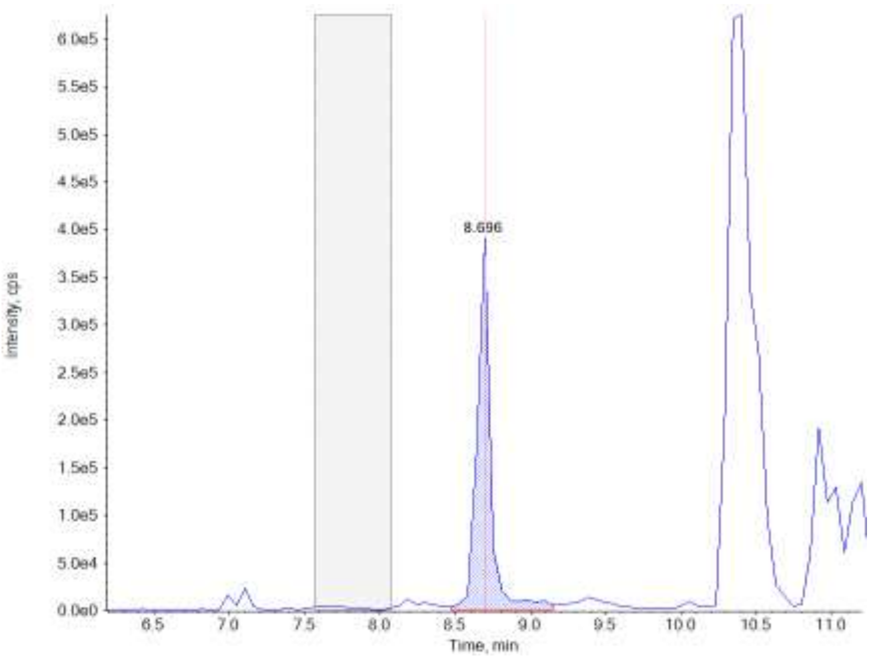

Acquired / Library MSMS

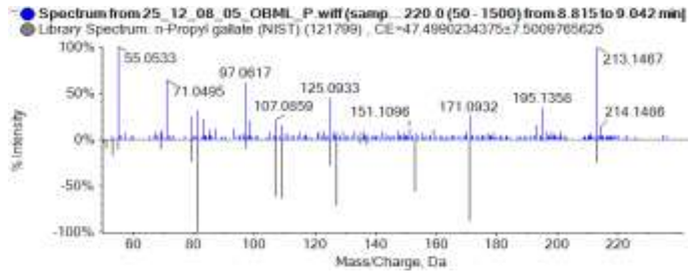

Acquired / Theoretical MS

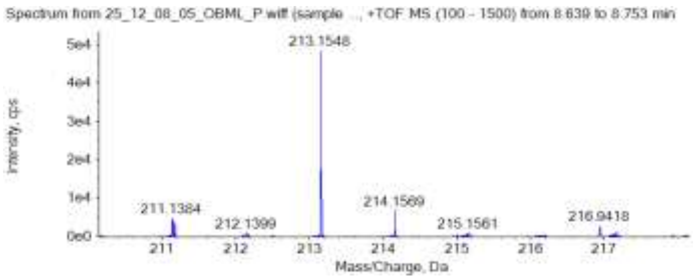

370.2071 / 8.92 (Mass/FragMass/RT/Isotope/Library/Formula/Ion Ratio)

Retention Time: 8.92 minutes
Precursor m/z : 370.2071
Fit (%) 100.0% **RFit (%)** 96.8%

Exp RT: 8.92 minutes
Analyte Name:
370.2071 / 8.92

Collision Energy = 35 ± 15 eV

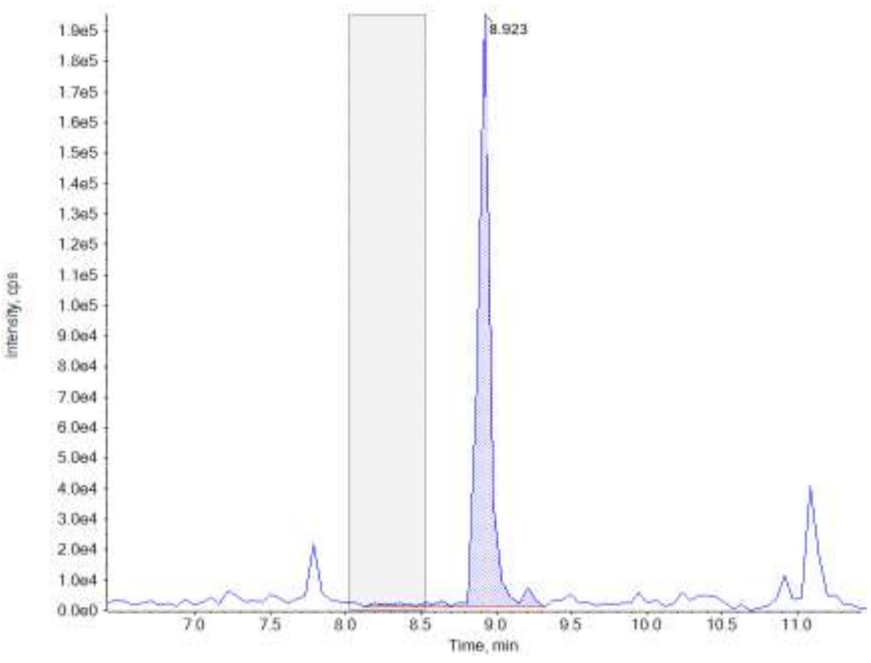

Acquired / Library MSMS

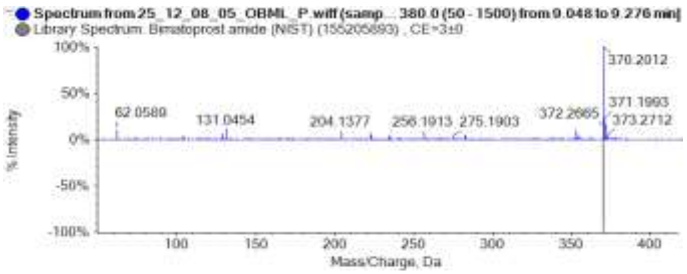

Acquired / Theoretical MS

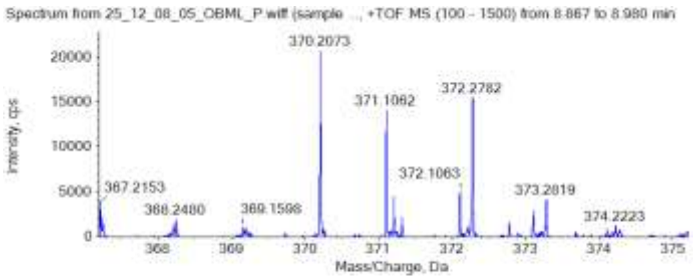

115.0812 / 9.04 (Mass/FragMass/RT/Isotope/Library/Formula/Ion Ratio)

Retention Time: 9.04 minutes | **Exp RT:** 9.04 minutes
Precursor m/z : 115.0812 | **Analyte Name:**
Fit (%) 61.1% **RFit (%)** 99.1% | 115.0812 / 9.04

Collision Energy = 35 ± 15 eV

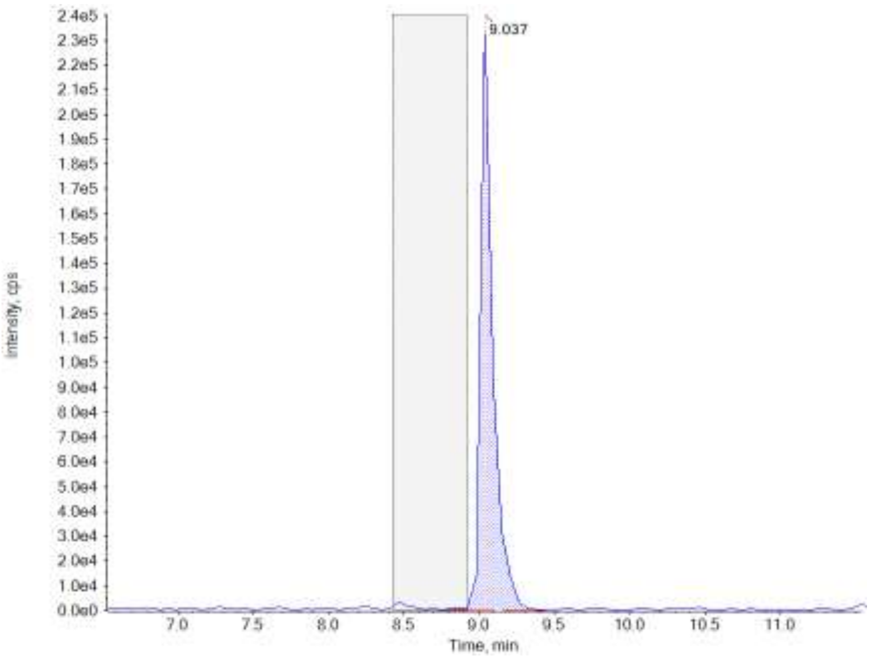

Acquired / Library MSMS

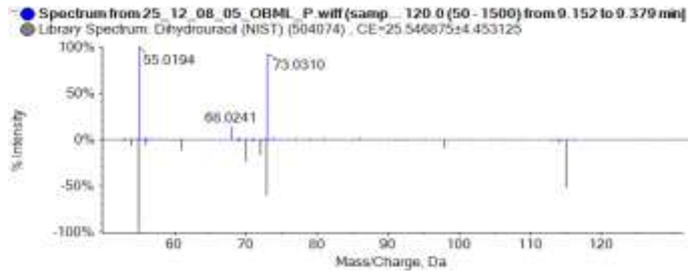

Acquired / Theoretical MS

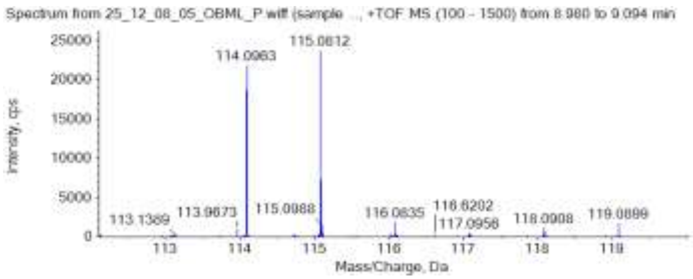

291.2011 / 9.04 (Mass/FragMass/RT/Isotope/Library/Formula/Ion Ratio)

**Retention Time:** 8.87 minutes	**Exp RT:** 9.04 minutes
**Precursor m/z :** 291.2011	**Analyte Name:**
**Fit (%)** 56.0% **RFit (%)** 96.2%	291.2011 / 9.04

Collision Energy = 35 ± 15 eV

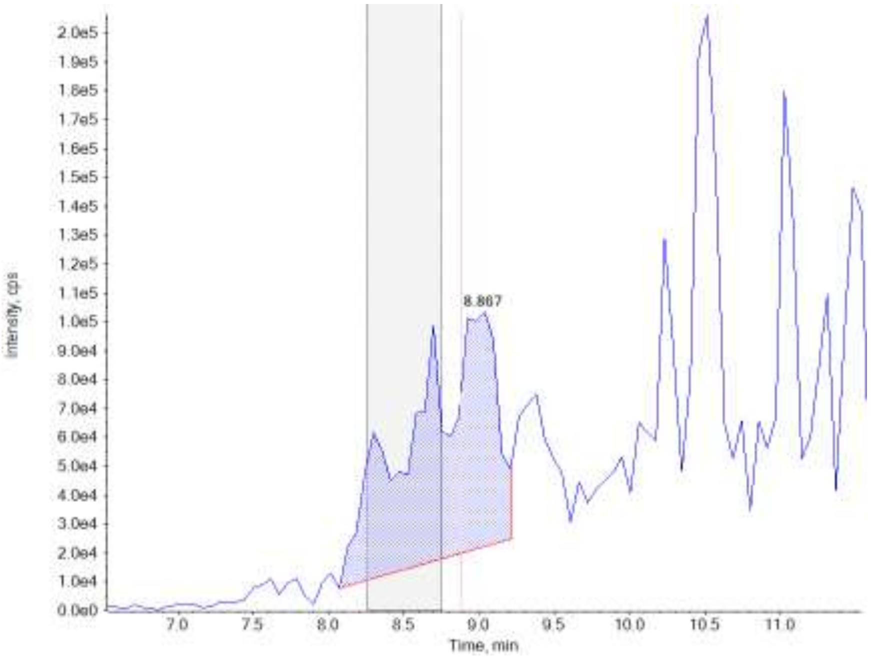

Acquired / Library MSMS

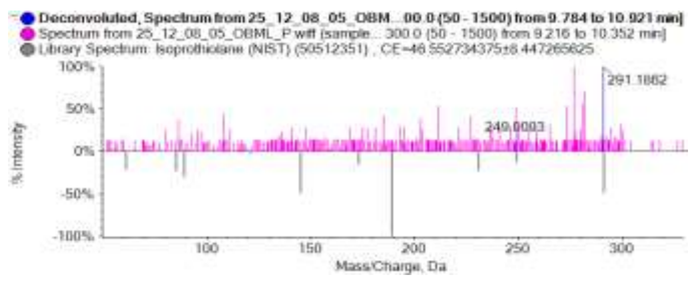

Acquired / Theoretical MS

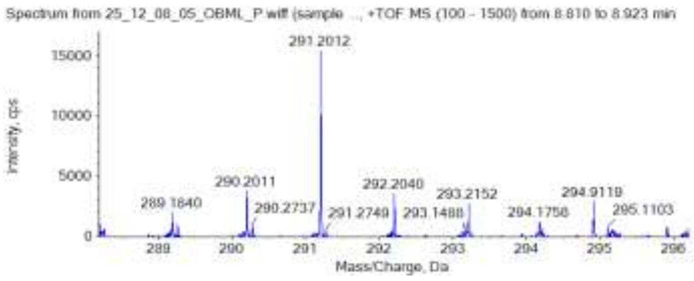

414.2916 / 9.21 (Mass/FragMass/RT/Isotope/Library/Formula/Ion Ratio)

Retention Time: 9.15 minutes
Precursor m/z : 414.2916
Fit (%) 99.4% **RFit (%)** 73.1%

Exp RT: 9.21 minutes
Analyte Name:
414.2916 / 9.21

Collision Energy = 35 ± 15 eV

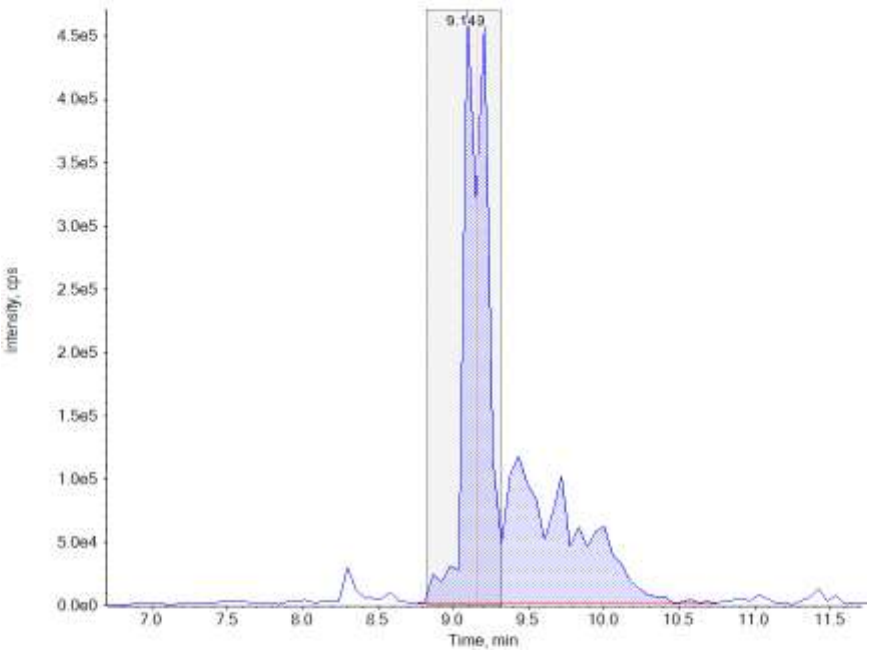

Acquired / Library MSMS

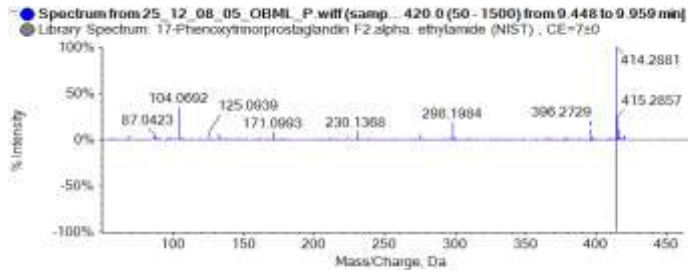

Acquired / Theoretical MS

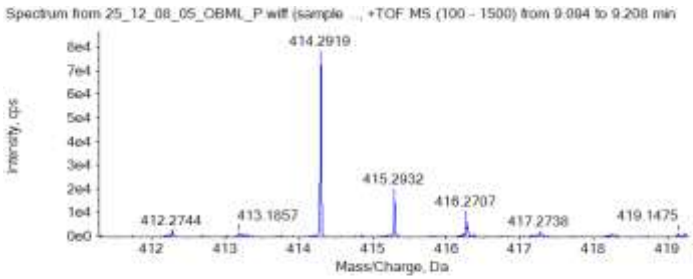

426.2901 / 9.21 (Mass/FragMass/RT/Isotope/Library/Formula/Ion Ratio)

**Retention Time:** 9.29 minutes	**Exp RT:** 9.21 minutes
**Precursor m/z :** 426.2901	**Analyte Name:**
**Fit (%)** 93.4% **RFit (%)** 99.4%	426.2901 / 9.21

Collision Energy = 35 ± 15 eV

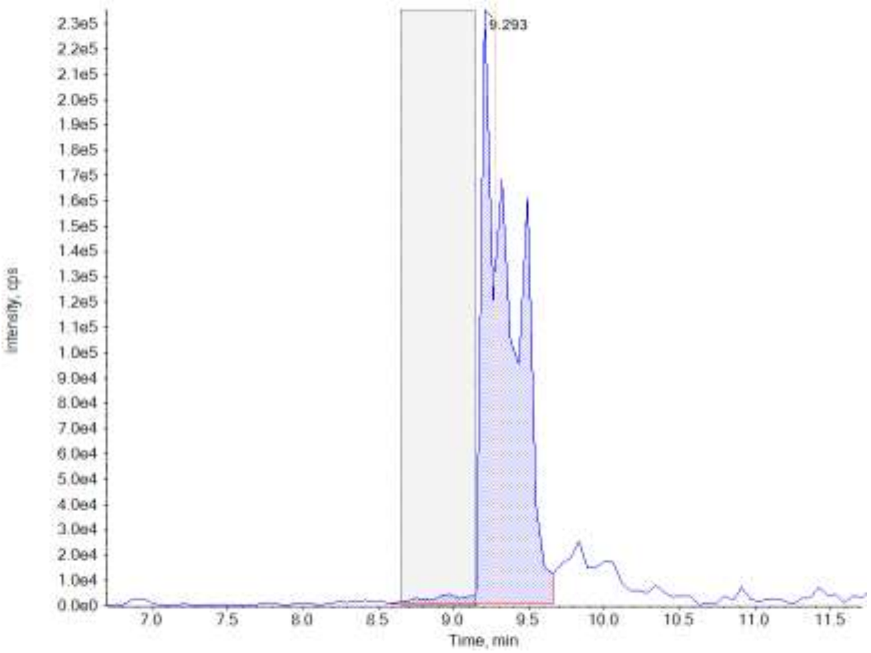

Acquired / Library MSMS

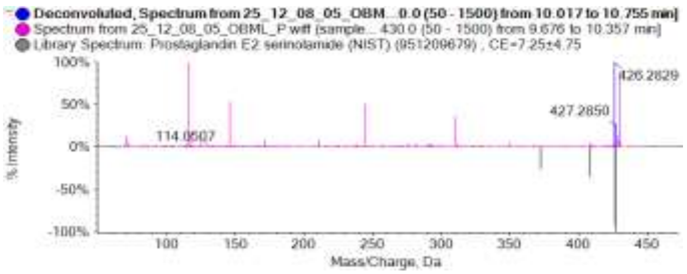

Acquired / Theoretical MS

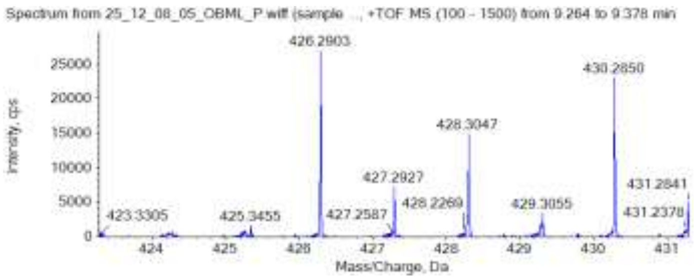

209.1590 / 9.38 (Mass/FragMass/RT/Isotope/Library/Formula/Ion Ratio)

Retention Time: 9.48 minutes
Precursor m/z : 209.1590
Fit (%) 87.7% **RFit (%)** 100.0%

Exp RT: 9.38 minutes
Analyte Name:
209.1590 / 9.38

Collision Energy = 35 ± 15 eV

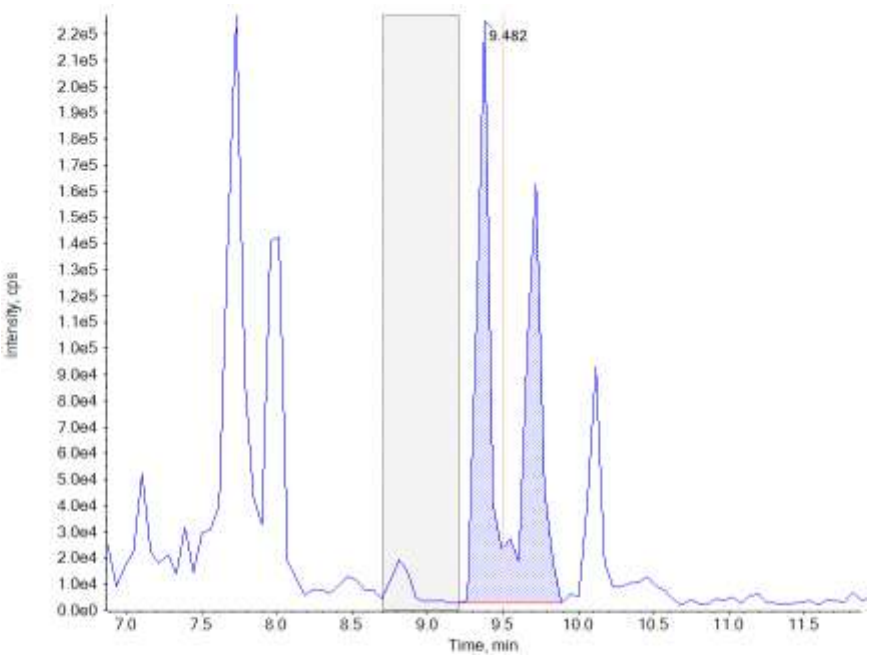

Acquired / Library MSMS

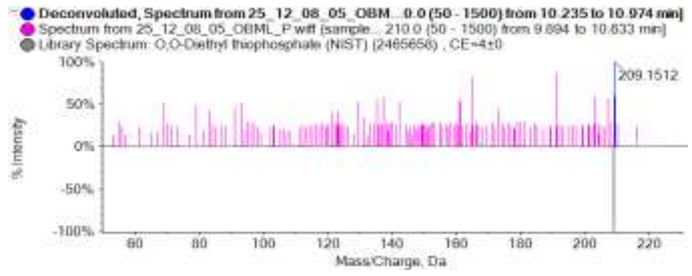

Acquired / Theoretical MS

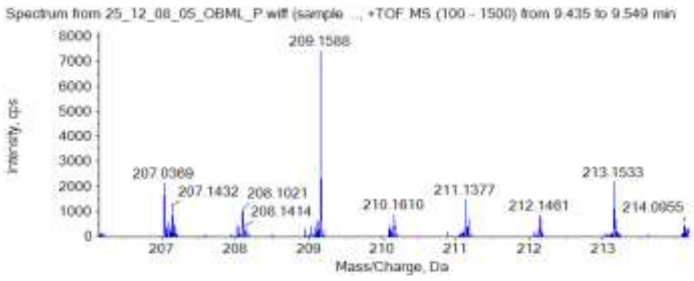

434.2593 / 9.38 (Mass/FragMass/RT/Isotope/Library/Formula/Ion Ratio)

**Retention Time:** 9.30 minutes	**Exp RT:** 9.38 minutes
**Precursor m/z :** 434.2593	**Analyte Name:**
**Fit (%)** 28.0% **RFit (%)** 100.0%	434.2593 / 9.38

Collision Energy = 35 ± 15 eV

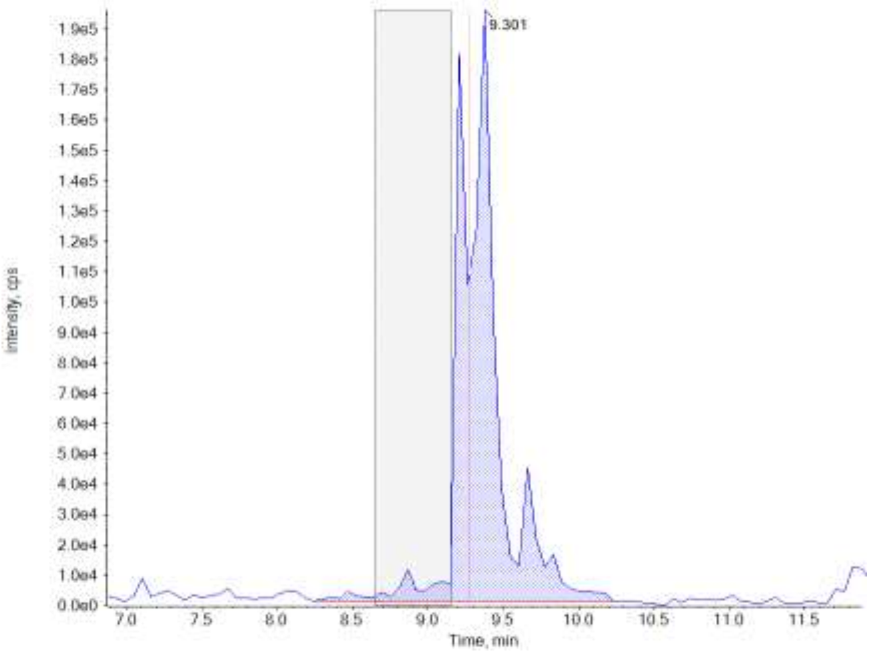

Acquired / Library MSMS

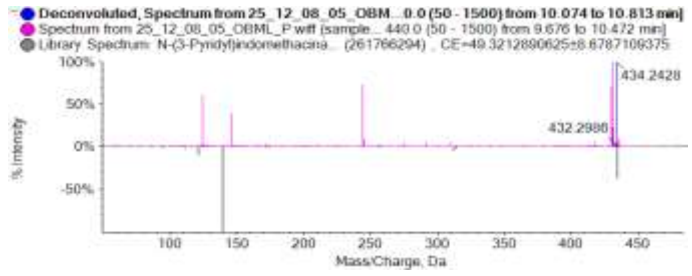

Acquired / Theoretical MS

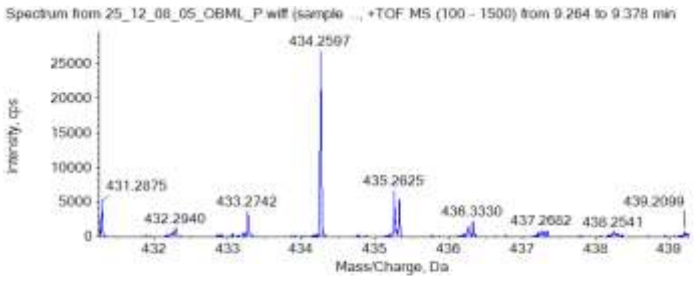

444.2670 / 9.49 (Mass/FragMass/RT/Isotope/Library/Formula/Ion Ratio)

Retention Time: 9.46 minutes **Exp RT:** 9.49 minutes
Precursor m/z : 444.2670 **Analyte Name:**
Fit (%) 59.8% **RFit (%)** 95.7% 444.2670 / 9.49

Collision Energy = 35 ± 15 eV

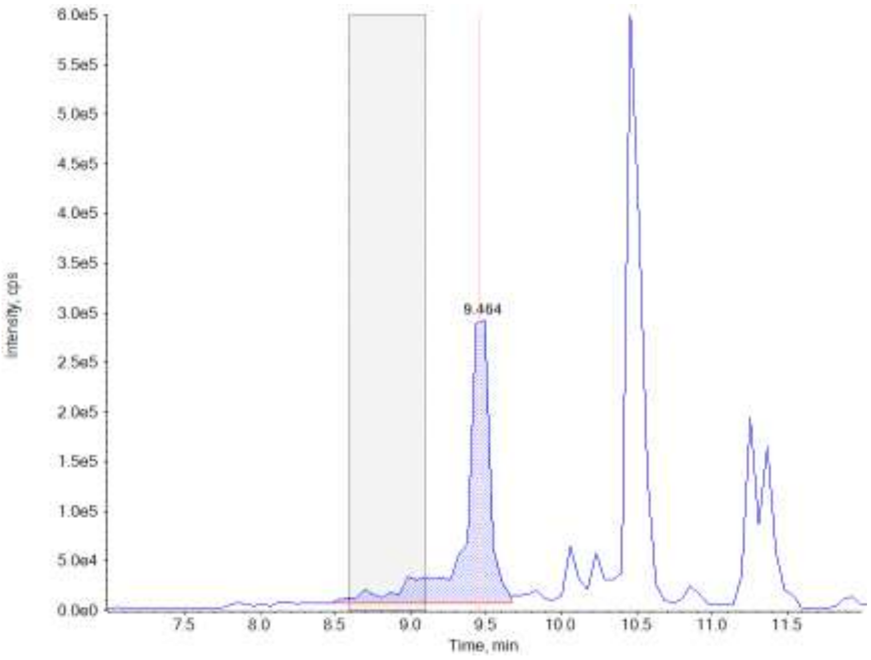

Acquired / Library MSMS

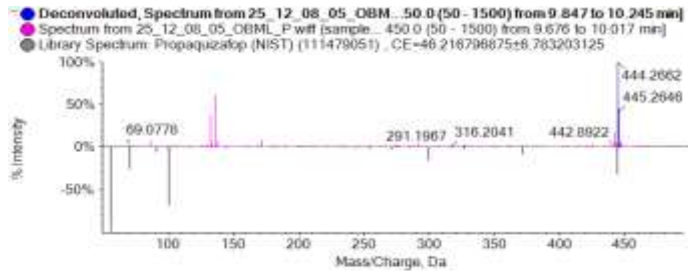

Acquired / Theoretical MS

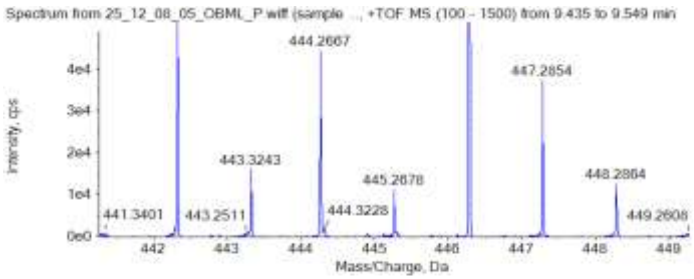

257.1339 / 9.55 (Mass/FragMass/RT/Isotope/Library/Formula/Ion Ratio)

Retention Time: 9.56 minutes
Precursor m/z : 257.1339
Fit (%) 66.0% **RFit (%)** 80.1%

Exp RT: 9.55 minutes
Analyte Name:
257.1339 / 9.55

Collision Energy = 35 ± 15 eV

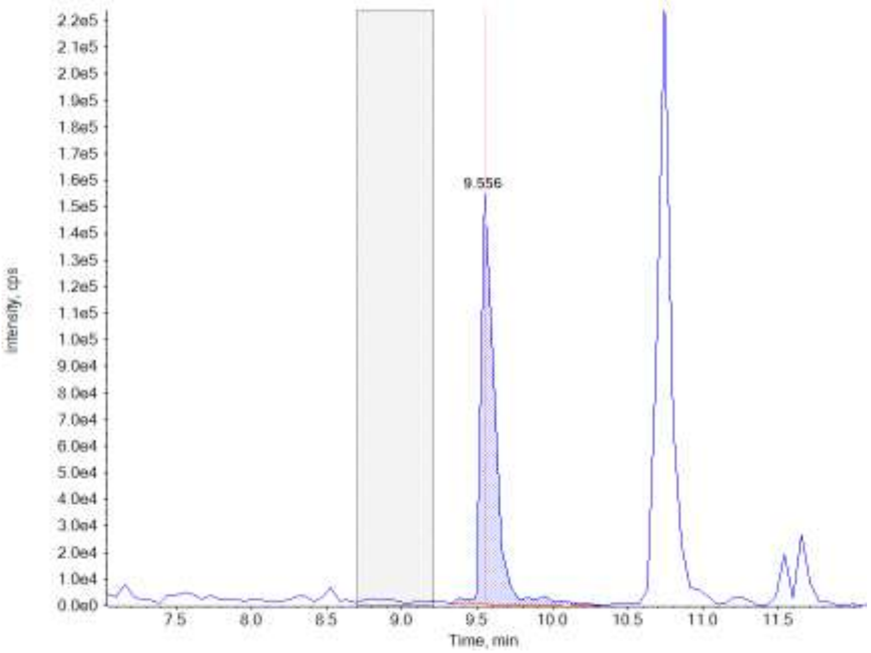

Acquired / Library MSMS

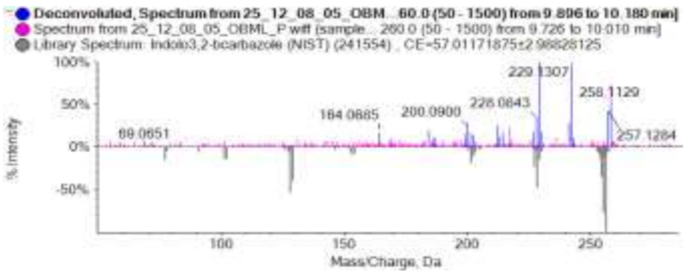

Acquired / Theoretical MS

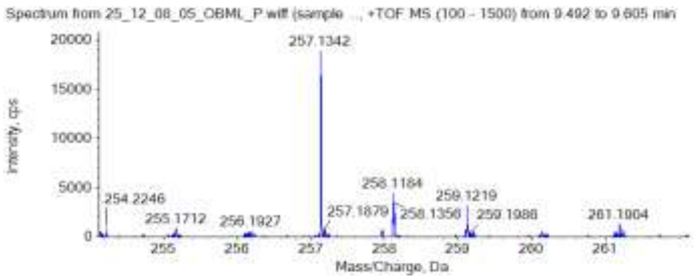

135.1216 / 9.66 (Mass/FragMass/RT/Isotope/Library/Formula/Ion Ratio)

**Retention Time:** 9.64 minutes<br>**Precursor m/z :** 135.1216<br>**Fit (%)** 95.7% **RFit (%)** 96.5%	**Exp RT:** 9.66 minutes<br>**Analyte Name:**<br>135.1216 / 9.66

	**Collision Energy = 35 ± 15 eV**	
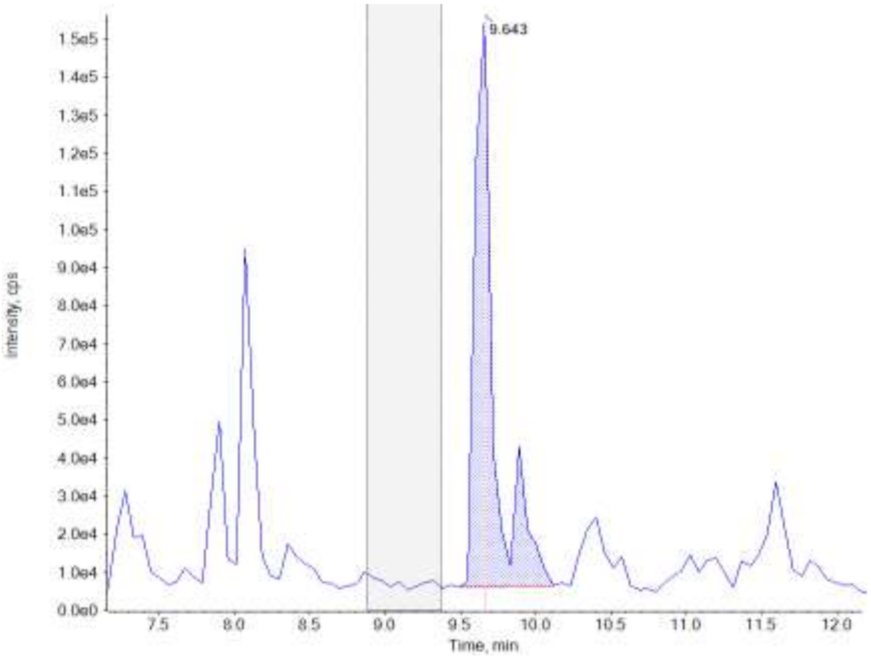	**Acquired / Library MSMS**	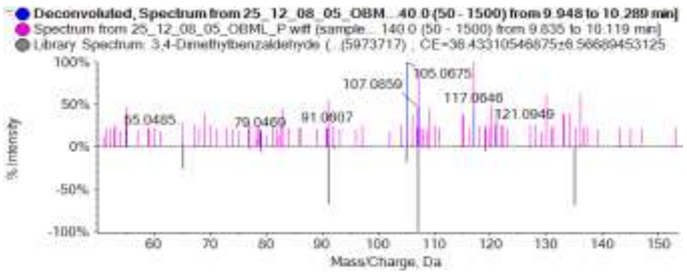
	**Acquired / Theoretical MS**	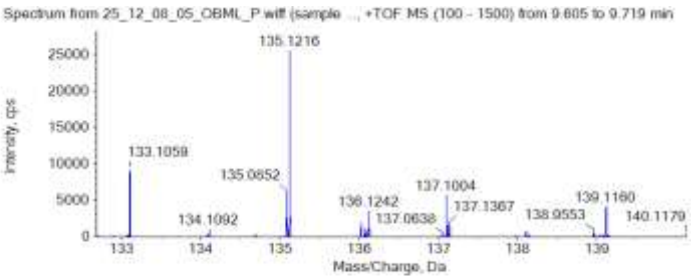

175.1529 / 9.66 (Mass/FragMass/RT/Isotope/Library/Formula/Ion Ratio)

Retention Time: 9.64 minutes
Precursor m/z : 175.1529
Fit (%) 76.0% **RFit (%)** 95.1%

Exp RT: 9.66 minutes
Analyte Name:
175.1529 / 9.66

Collision Energy = 35 ± 15 eV

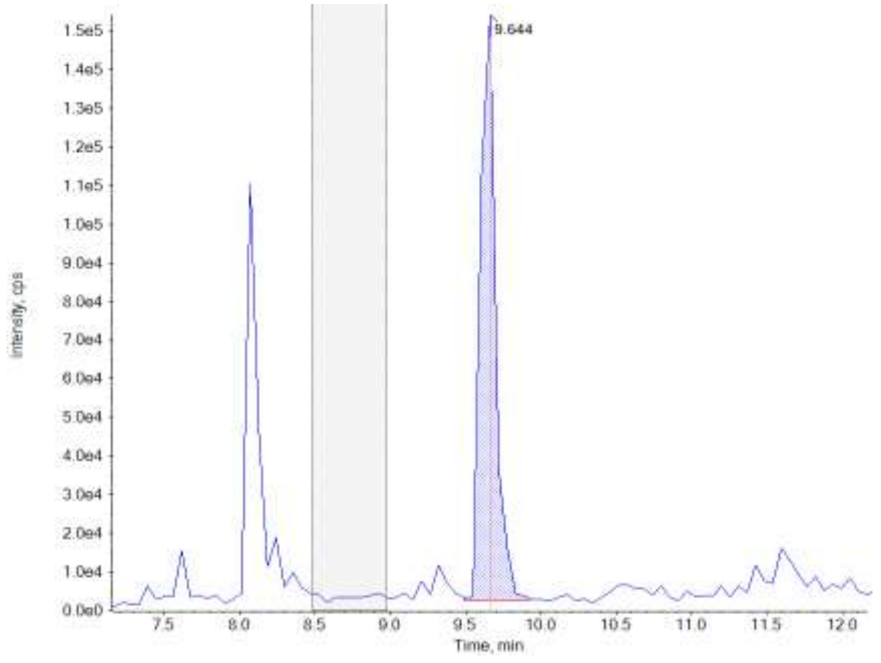

Acquired / Library MSMS

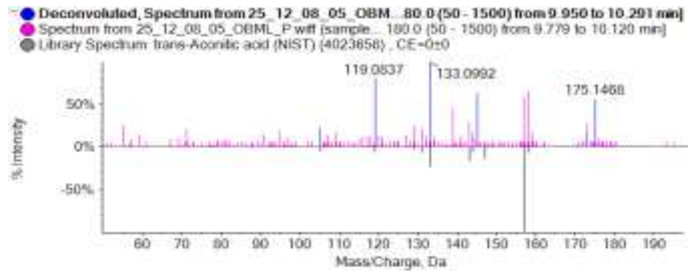

Acquired / Theoretical MS

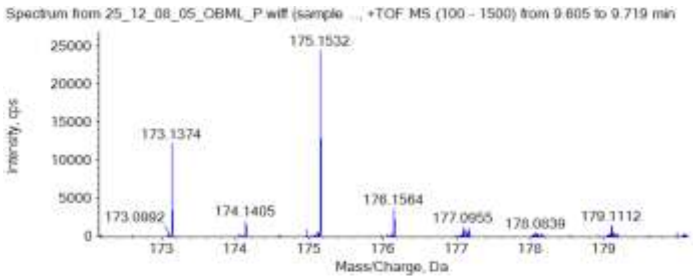

193.1641 / 9.66 (Mass/FragMass/RT/Isotope/Library/Formula/Ion Ratio)

Retention Time: 9.64 minutes
Precursor m/z : 193.1641
Fit (%) 99.1% **RFit (%)** 89.6%

Exp RT: 9.66 minutes
Analyte Name:
193.1641 / 9.66

Collision Energy = 35 ± 15 eV

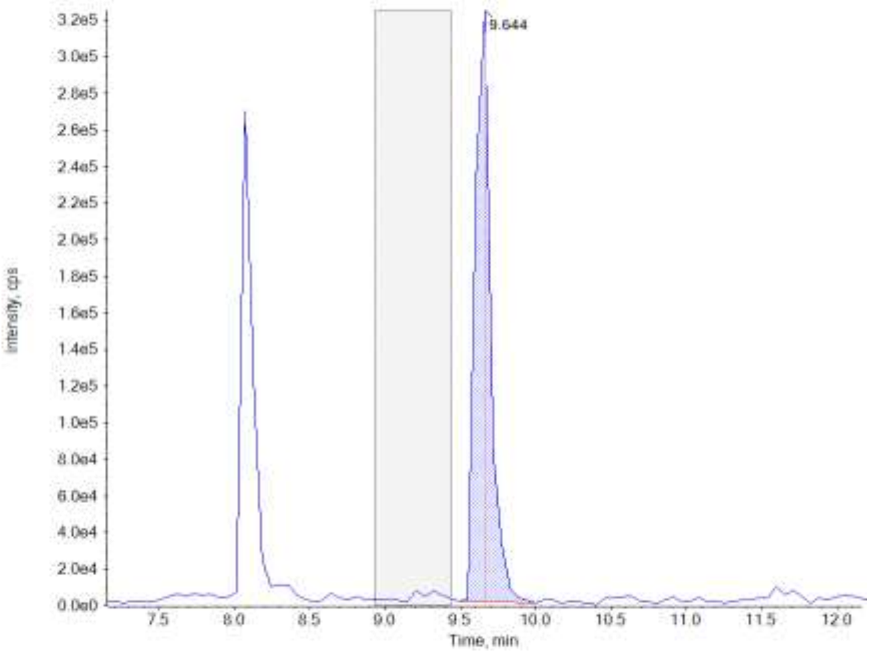

Acquired / Library MSMS

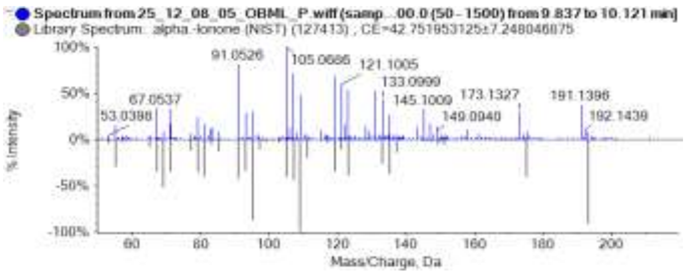

Acquired / Theoretical MS

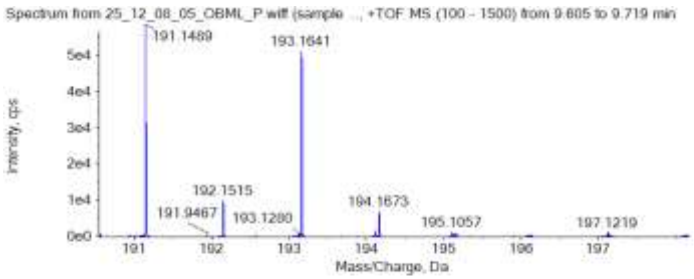

211.1751 / 9.66 (Mass/FragMass/RT/Isotope/Library/Formula/Ion Ratio)

Retention Time: 9.65 minutes **Exp RT:** 9.66 minutes
Precursor m/z : 211.1751 **Analyte Name:**
Fit (%) 92.7% **RFit (%)** 89.9% 211.1751 / 9.66

Collision Energy = 35 ± 15 eV

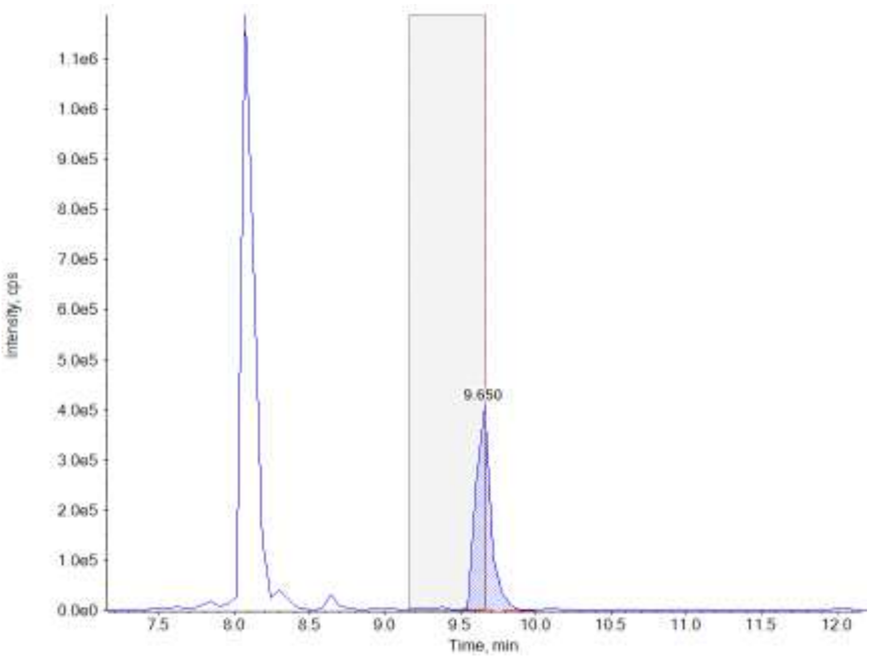

Acquired / Library MSMS

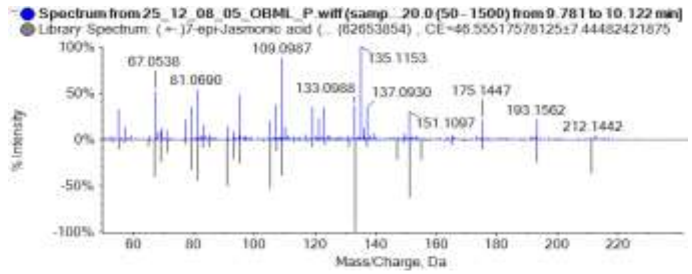

Acquired / Theoretical MS

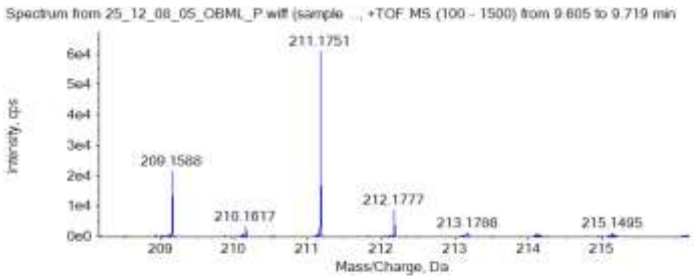

446.2851 / 9.66 (Mass/FragMass/RT/Isotope/Library/Formula/Ion Ratio)

**Retention Time:** 9.59 minutes	**Exp RT:** 9.66 minutes
**Precursor m/z :** 446.2851	**Analyte Name:**
**Fit (%)** 70.5% **RFit (%)** 100.0%	446.2851 / 9.66

Collision Energy = 35 ± 15 eV

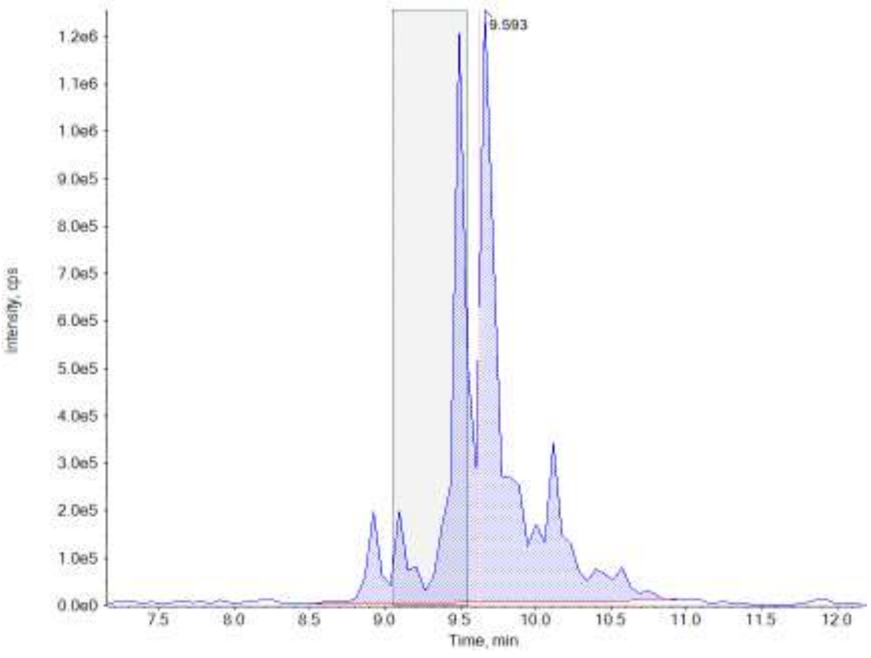

Acquired / Library MSMS

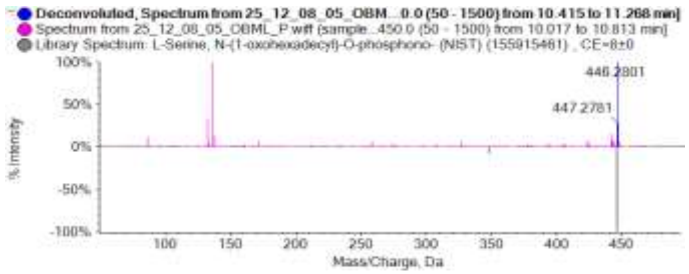

Acquired / Theoretical MS

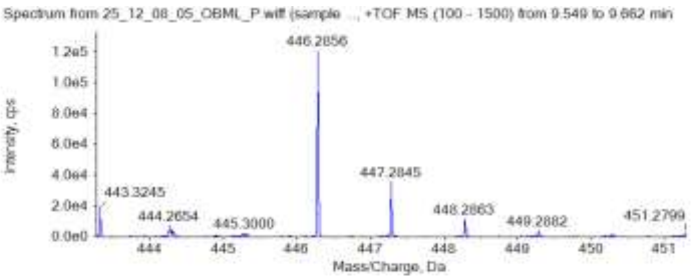

191.1489 / 9.72 (Mass/FragMass/RT/Isotope/Library/Formula/Ion Ratio)

Retention Time: 9.70 minutes **Exp RT:** 9.72 minutes
Precursor m/z : 191.1489 **Analyte Name:**
Fit (%) 97.4% **RFit (%)** 73.6% 191.1489 / 9.72

Collision Energy = 35 ± 15 eV

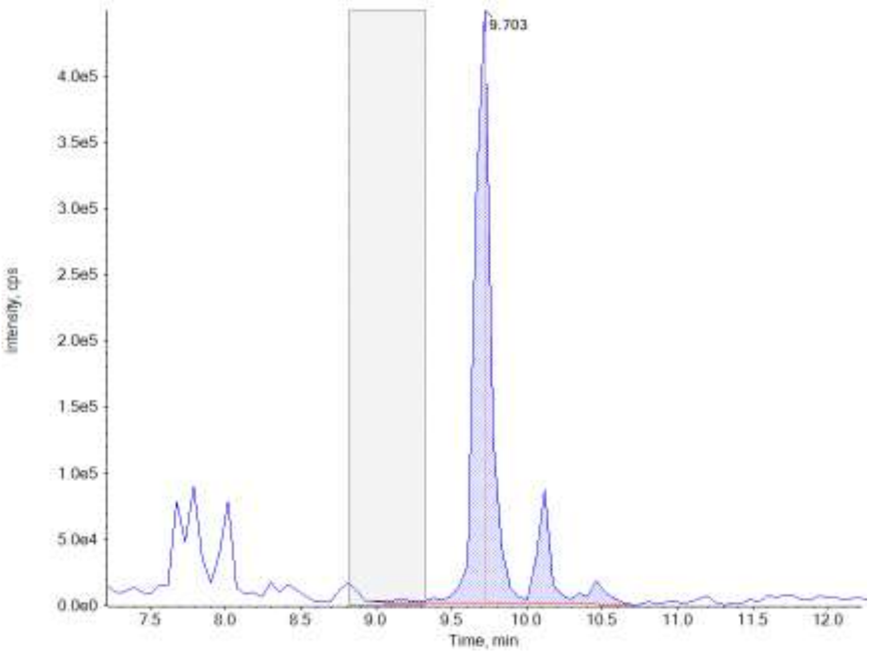

Acquired / Library MSMS

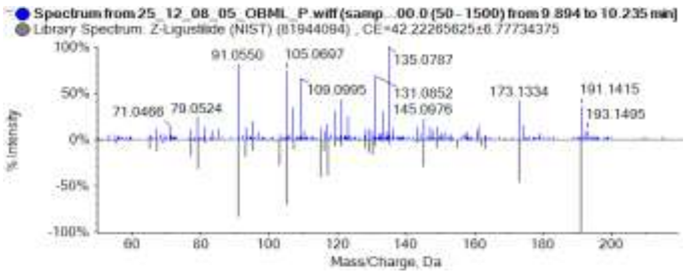

Acquired / Theoretical MS

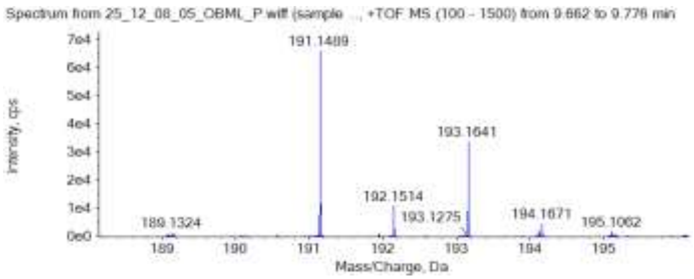

747.5549 / 9.72 (Mass/FragMass/RT/Isotope/Library/Formula/Ion Ratio)

Retention Time: 9.72 minutes
Precursor m/z : 747.5548
Fit (%) 100.0% **RFit (%)** 100.0%

Exp RT: 9.72 minutes
Analyte Name:
747.5549 / 9.72

Collision Energy = 35 ± 15 eV

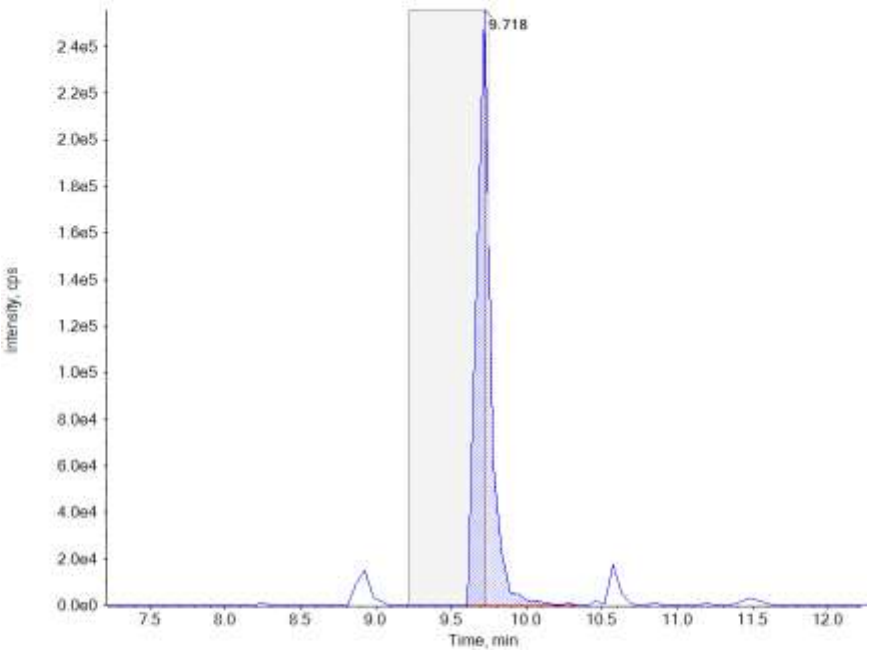

Acquired / Library MSMS

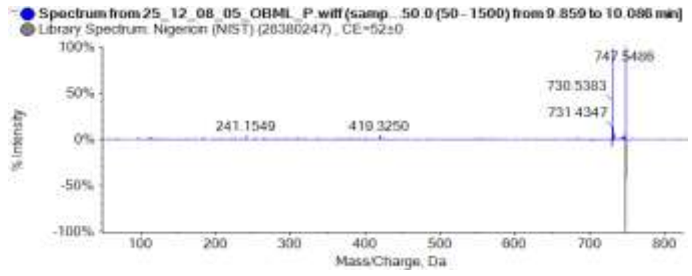

Acquired / Theoretical MS

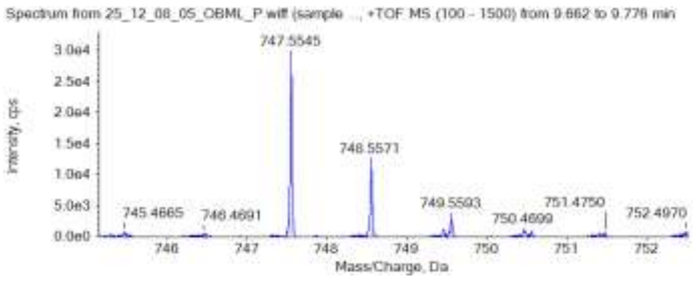

275.2067 / 9.78 (Mass/FragMass/RT/Isotope/Library/Formula/Ion Ratio)

Retention Time: 9.76 minutes
Precursor m/z : 275.2067
Fit (%) 24.3% **RFit (%)** 100.0%

Exp RT: 9.78 minutes
Analyte Name:
275.2067 / 9.78

Collision Energy = 35 ± 15 eV

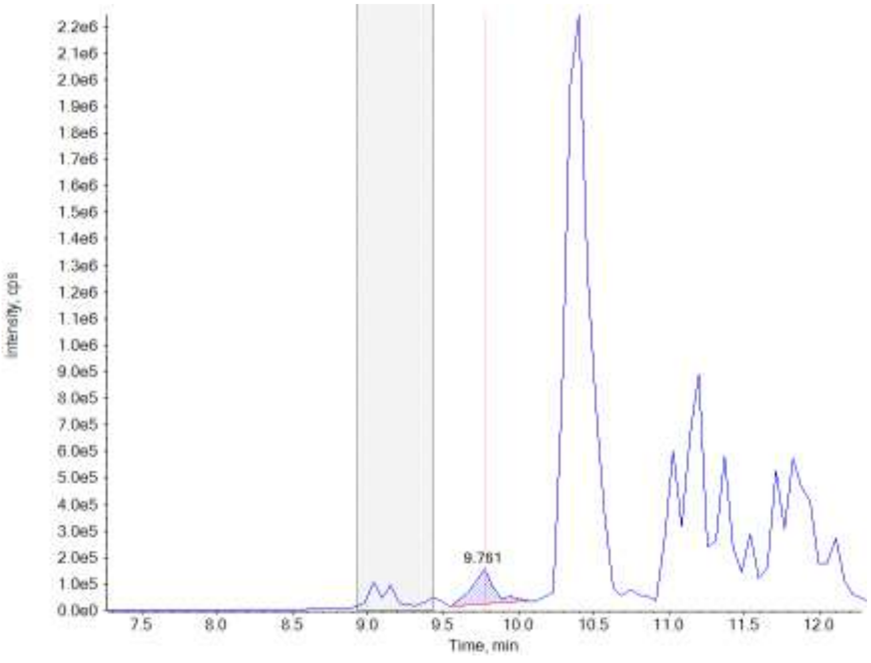

Acquired / Library MSMS

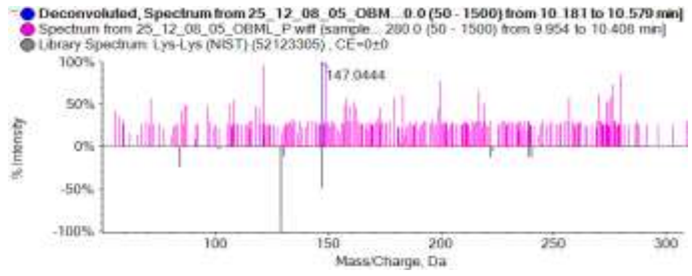

Acquired / Theoretical MS

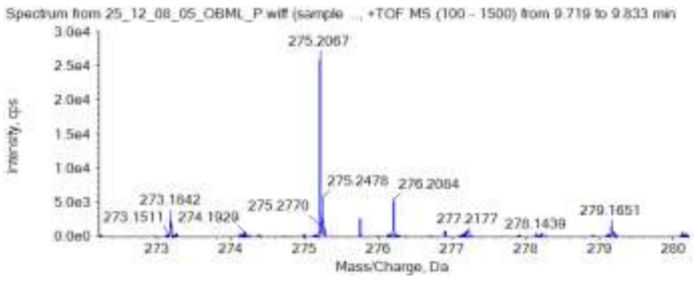

439.3270 / 9.83 (Mass/FragMass/RT/Isotope/Library/Formula/Ion Ratio)

**Retention Time:** 9.83 minutes	**Exp RT:** 9.83 minutes
**Precursor m/z :** 439.3270	**Analyte Name:**
**Fit (%)** 99.4% **RFit (%)** 73.1%	439.3270 / 9.83

Collision Energy = 35 ± 15 eV

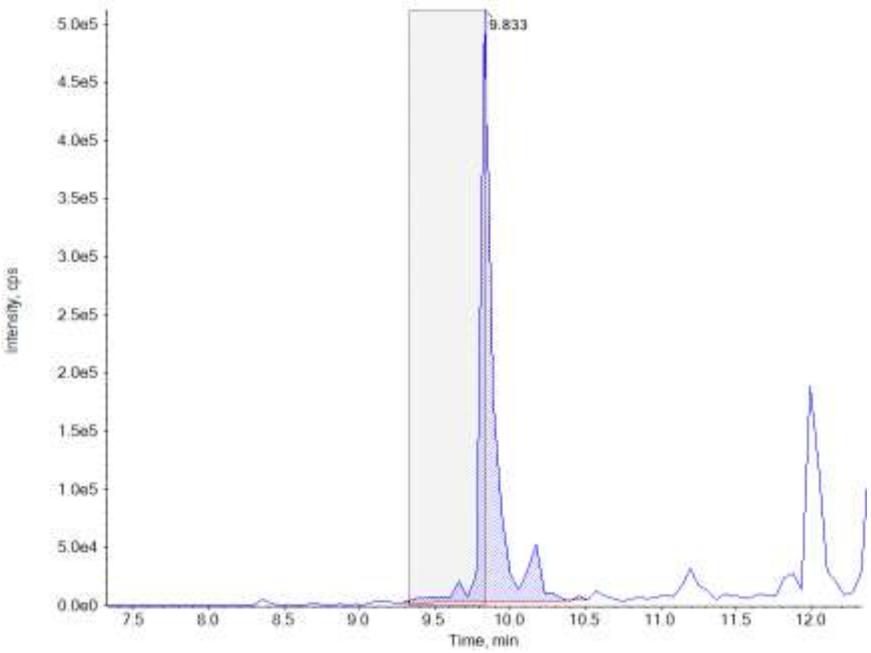

Acquired / Library MSMS

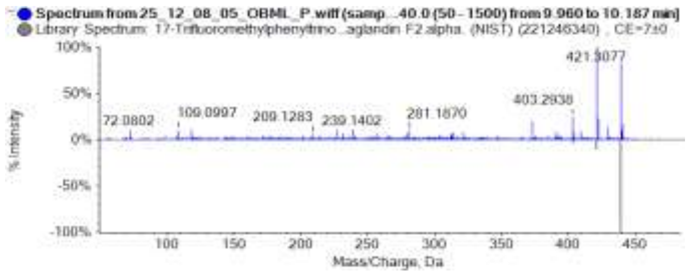

Acquired / Theoretical MS

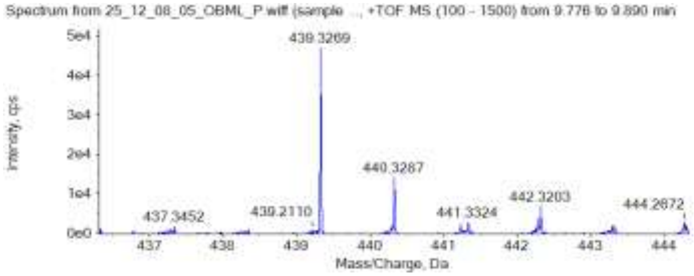

137.1376 / 9.89 (Mass/FragMass/RT/Isotope/Library/Formula/Ion Ratio)

**Retention Time:** 9.89 minutes	**Exp RT:** 9.89 minutes
**Precursor m/z :** 137.1376	**Analyte Name:**
**Fit (%)** 96.9% **RFit (%)** 97.7%	137.1376 / 9.89

Collision Energy = 35 ± 15 eV

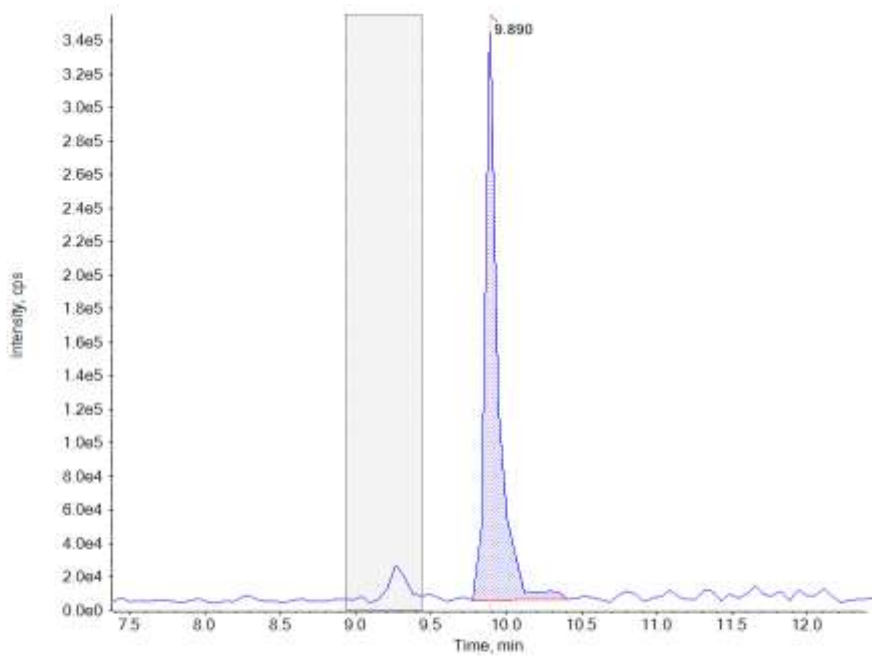

Acquired / Library MSMS

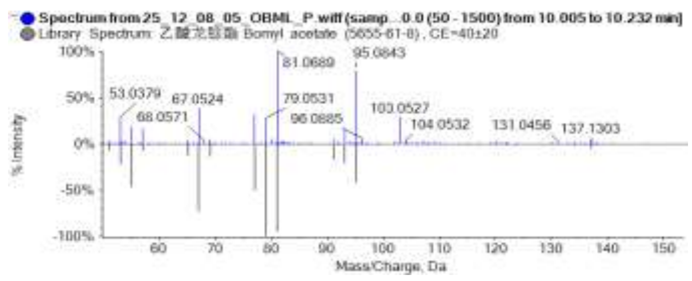

Acquired / Theoretical MS

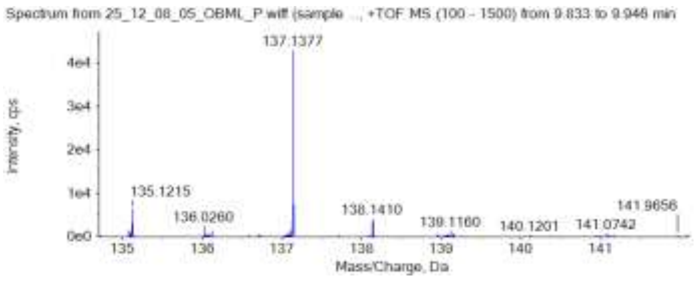

Created with SCIEX OS 3.3.0
Printed:12/9/2025 2:05:30 PM

391.3407 / 10.29 (Mass/FragMass/RT/Isotope/Library/Formula/Ion Ratio)

**Retention Time:** 10.28 minutes	**Exp RT:** 10.29 minutes
**Precursor m/z :** 391.3407	**Analyte Name:**
**Fit (%)** 99.6% **RFit (%)** 97.7%	391.3407 / 10.29

Collision Energy = 35 ± 15 eV

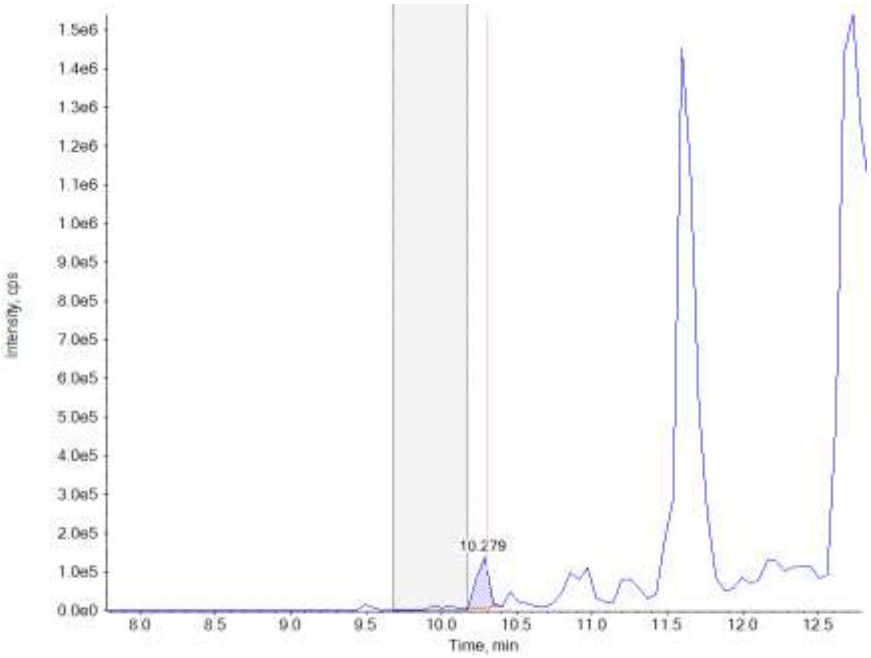

Acquired / Library MSMS

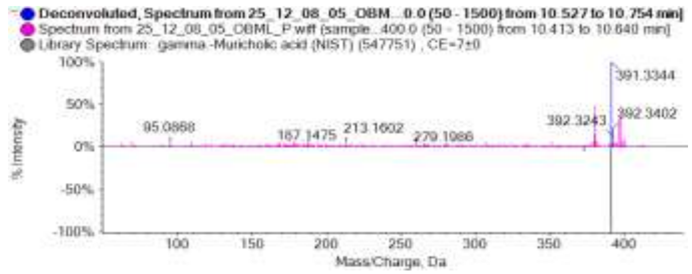

Acquired / Theoretical MS

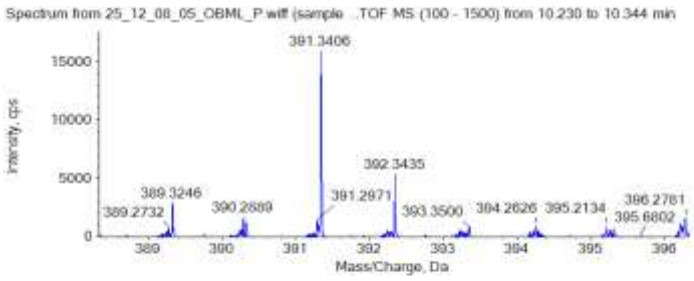

437.3462 / 10.29 (Mass/FragMass/RT/Isotope/Library/Formula/Ion Ratio)

**Retention Time:** 10.28 minutes	**Exp RT:** 10.29 minutes
**Precursor m/z :** 437.3462	**Analyte Name:**
**Fit (%)** 86.3% **RFit (%)** 92.7%	437.3462 / 10.29

Collision Energy = 35 ± 15 eV

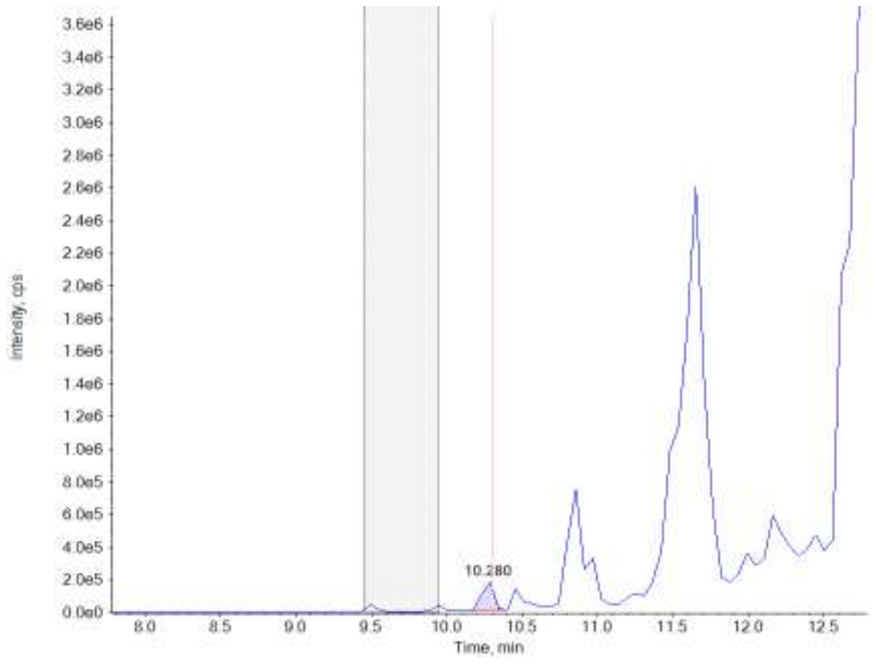

Acquired / Library MSMS

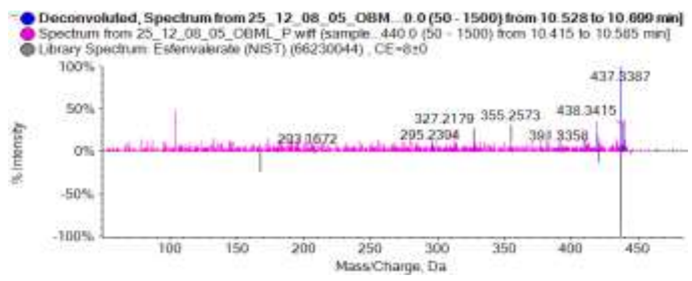

Acquired / Theoretical MS

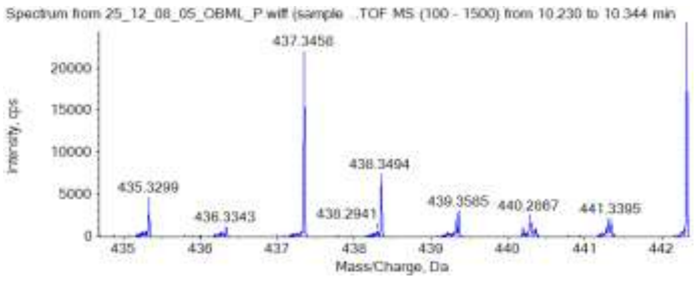

195.1430 / 10.34 (Mass/FragMass/RT/Isotope/Library/Formula/Ion Ratio)

**Retention Time:** 10.38 minutes	**Exp RT:** 10.34 minutes
**Precursor m/z :** 195.1430	**Analyte Name:**
**Fit (%)** 91.6% **RFit (%)** 83.8%	195.1430 / 10.34

Collision Energy = 35 ± 15 eV

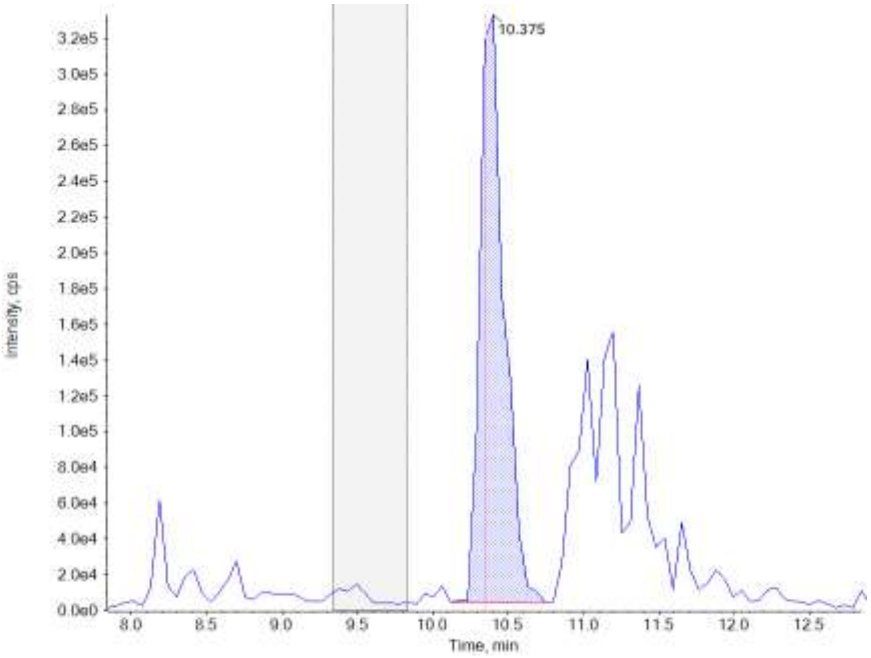

Acquired / Library MSMS

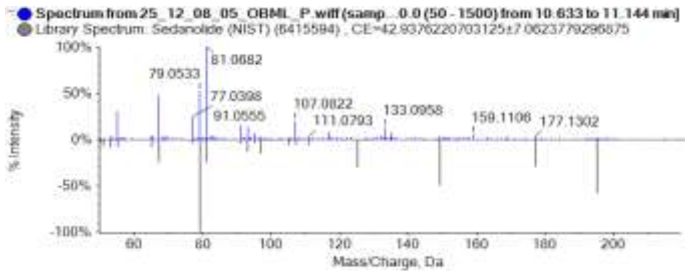

Acquired / Theoretical MS

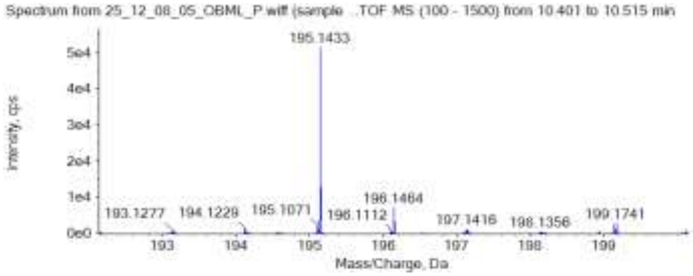

275.2117 / 10.34 (Mass/FragMass/RT/Isotope/Library/Formula/Ion Ratio)

**Retention Time:** 10.37 minutes	**Exp RT:** 10.34 minutes
**Precursor m/z :** 275.2117	**Analyte Name:**
**Fit (%)** 81.2% **RFit (%)** 99.4%	275.2117 / 10.34

Collision Energy = 35 ± 15 eV

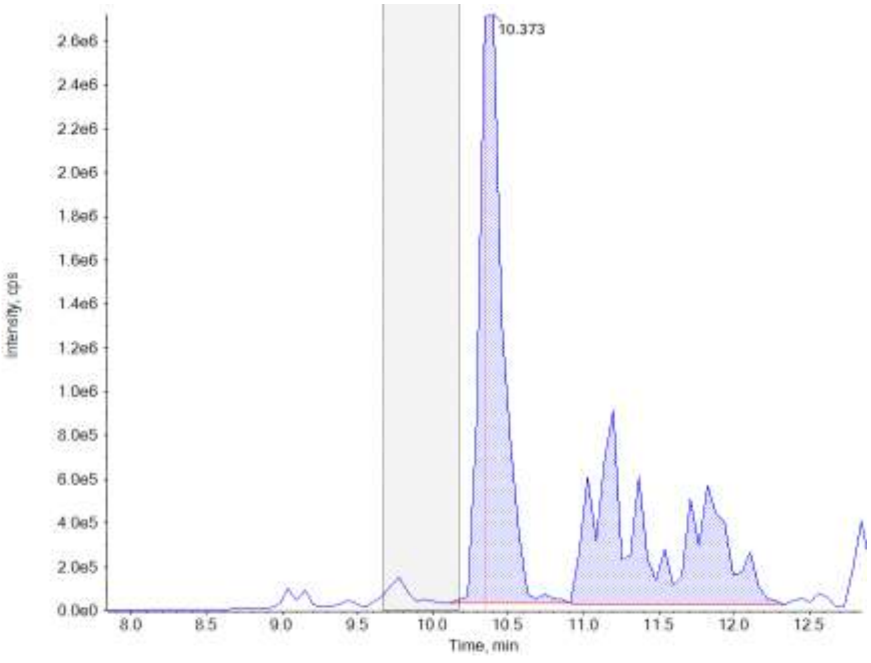

Acquired / Library MSMS

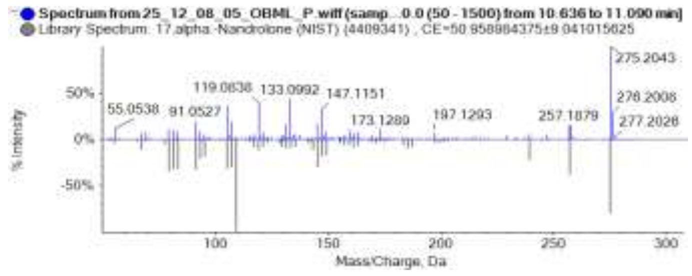

Acquired / Theoretical MS

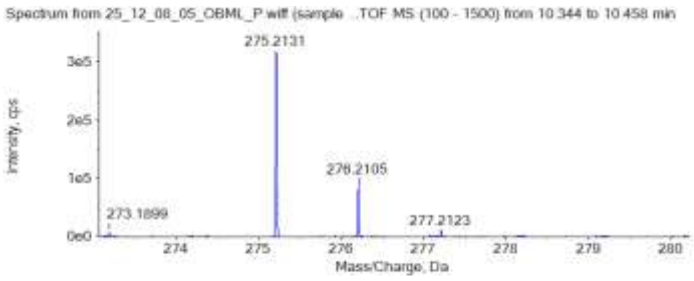

293.2221 / 10.34

(Mass/FragMass/RT/Isotope/Library/Formula/Ion Ratio)

**Retention Time:** 10.37 minutes	**Exp RT:** 10.34 minutes
**Precursor m/z :** 293.2221	**Analyte Name:**
**Fit (%)** 97.0% **RFit (%)** 96.4%	293.2221 / 10.34

Collision Energy = 35 ± 15 eV

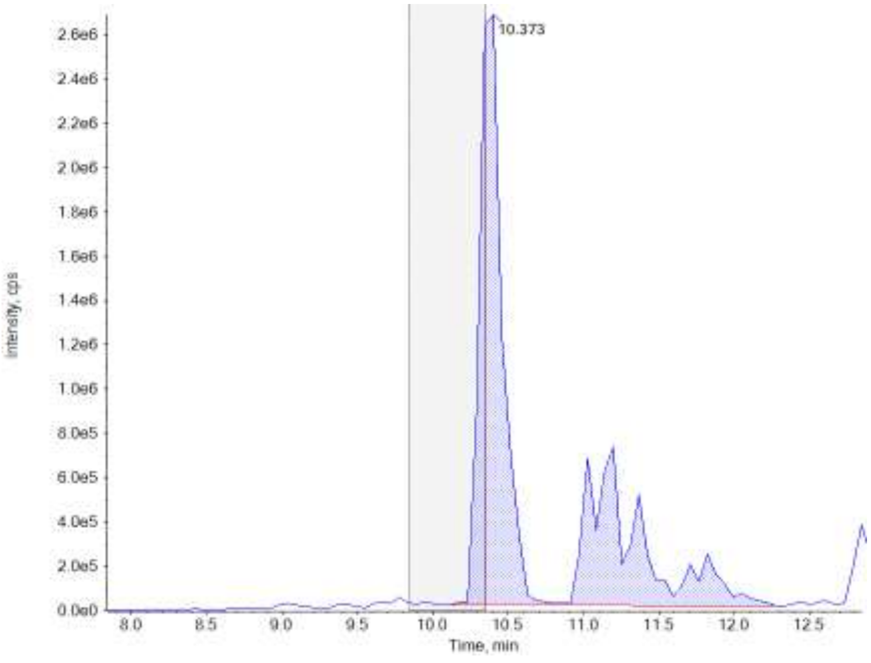

Acquired / Library MSMS

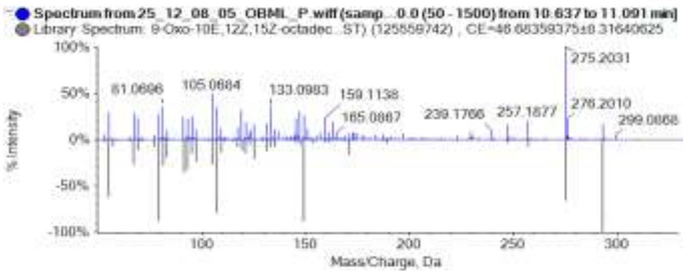

Acquired / Theoretical MS

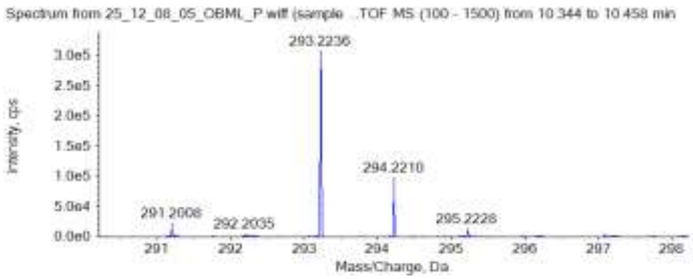

311.2287 / 10.34

(Mass/FragMass/RT/Isotope/Library/Formula/Ion Ratio)

**Retention Time:** 10.49 minutes	**Exp RT:** 10.34 minutes
**Precursor m/z :** 311.2287	**Analyte Name:**
**Fit (%)** 80.5% **RFit (%)** 86.9%	311.2287 / 10.34

Collision Energy = 35 ± 15 eV

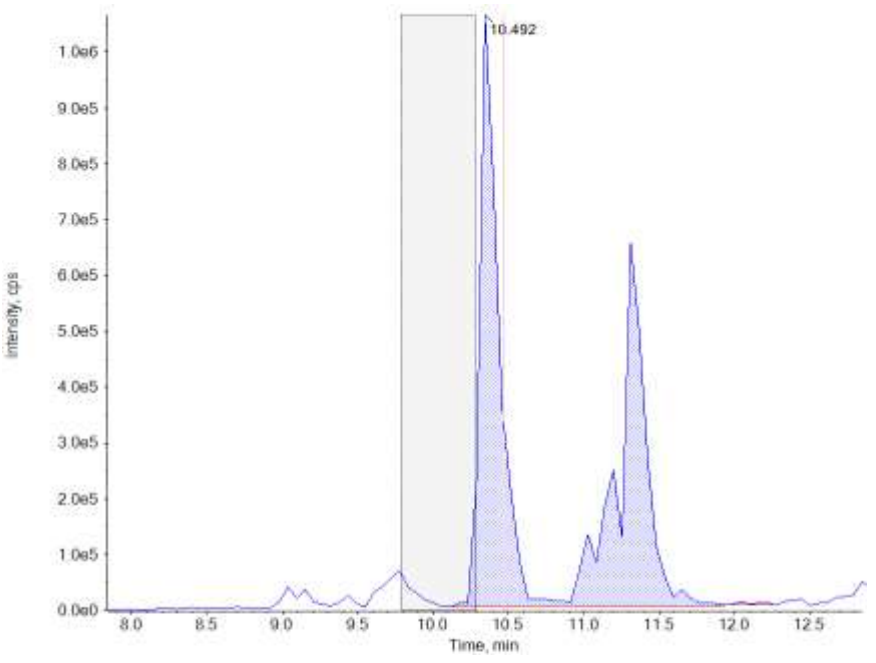

Acquired / Library MSMS

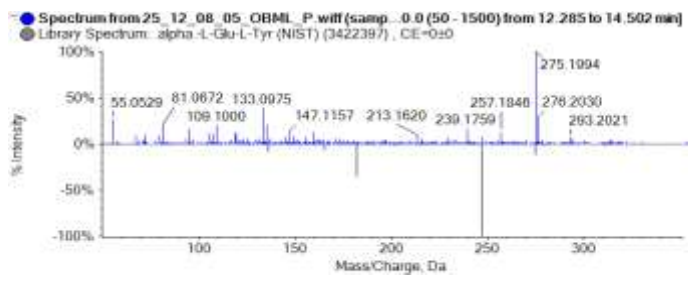

Acquired / Theoretical MS

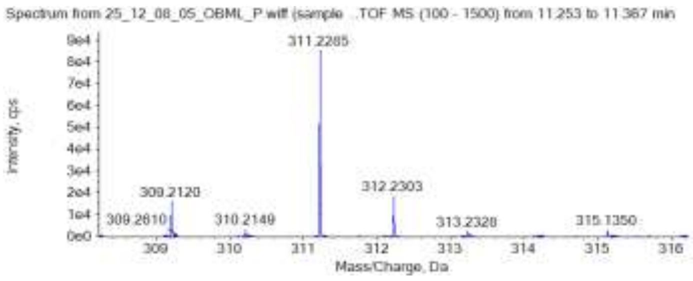

367.1934 / 10.34 (Mass/FragMass/RT/Isotope/Library/Formula/Ion Ratio)

Retention Time: 10.37 minutes
Precursor m/z : 367.1934
Fit (%) 100.0% **RFit (%)** 54.9%

Exp RT: 10.34 minutes
Analyte Name:
367.1934 / 10.34

Collision Energy = 35 ± 15 eV

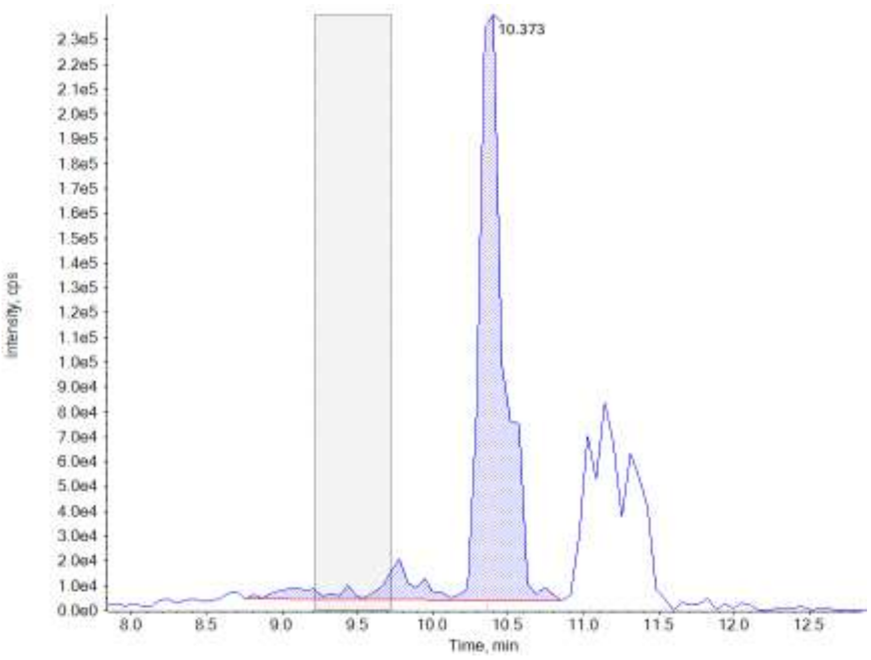

Acquired / Library MSMS

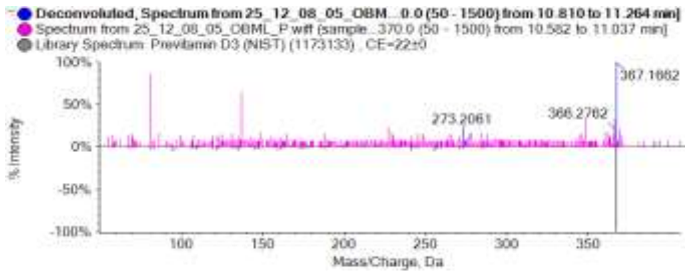

Acquired / Theoretical MS

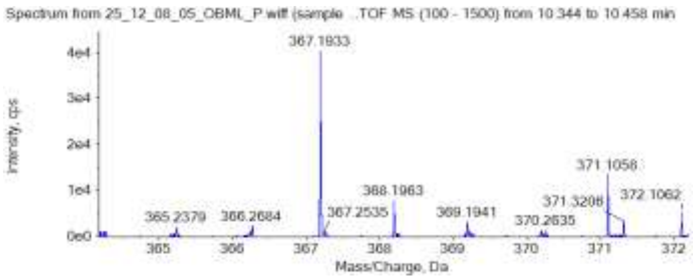

Created with SCIEX OS 3.3.0
Printed:12/9/2025 2:05:30 PM

595.3154 / 10.86 (Mass/FragMass/RT/Isotope/Library/Formula/Ion Ratio)

Retention Time: 10.83 minutes
Precursor m/z : 595.3154
Fit (%) 100.0% **RFit (%)** 21.0%

Exp RT: 10.86 minutes
Analyte Name:
595.3154 / 10.86

Collision Energy = 35 ± 15 eV

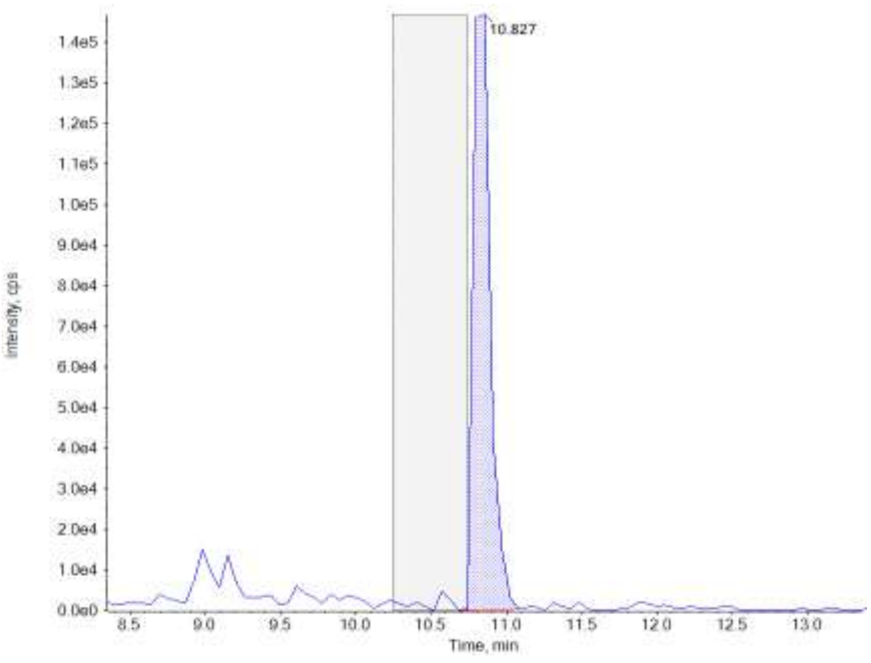

Acquired / Library MSMS

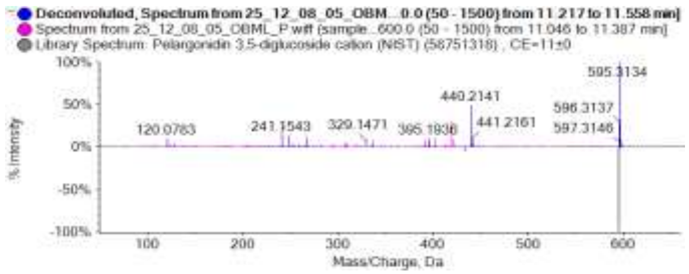

Acquired / Theoretical MS

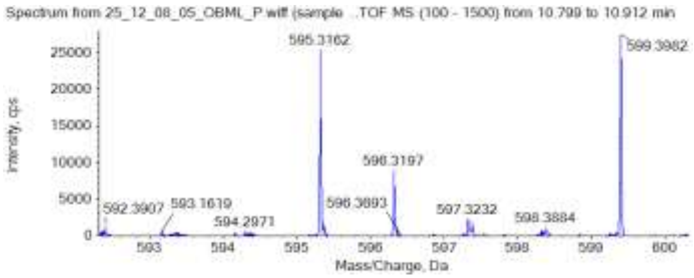

309.2117 / 11.03

(Mass/FragMass/RT/Isotope/Library/Formula/Ion Ratio)

Retention Time: 11.03 minutes **Exp RT:** 11.03 minutes
Precursor m/z : 309.2117 **Analyte Name:**
Fit (%) 98.8% **RFit (%)** 31.5% 309.2117 / 11.03

Collision Energy = 35 ± 15 eV

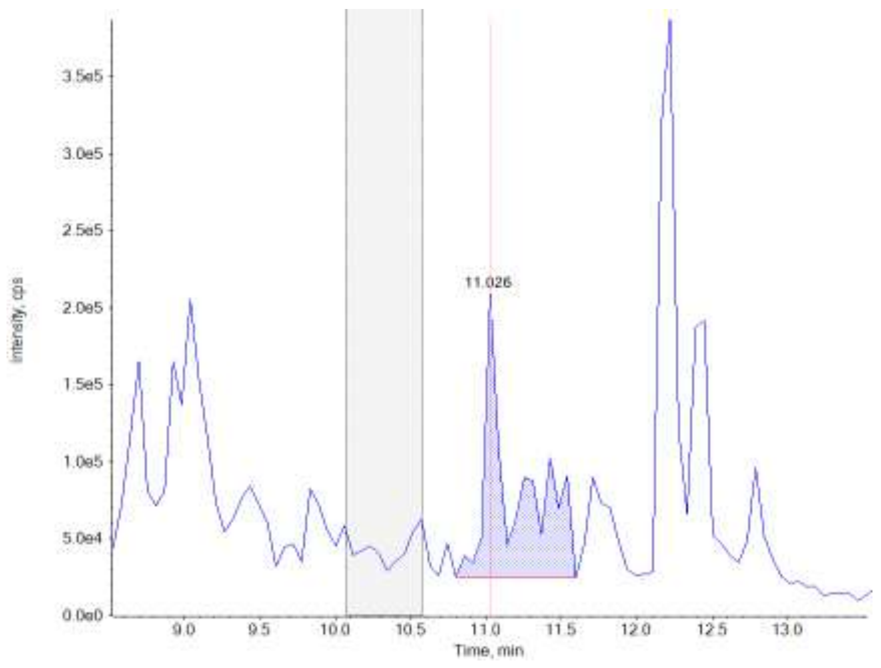

Acquired / Library MSMS

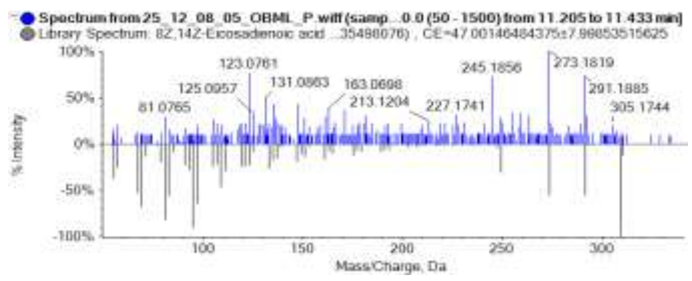

Acquired / Theoretical MS

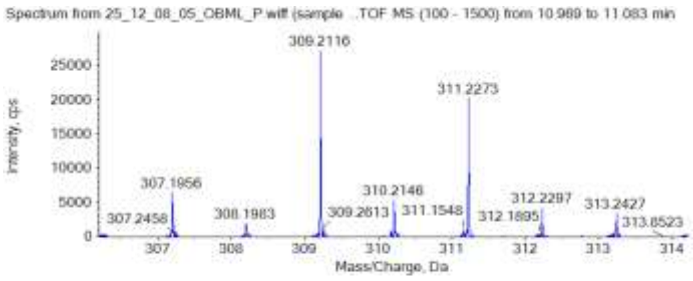

195.1427 / 11.20

(Mass/FragMass/RT/Isotope/Library/Formula/Ion Ratio)

Retention Time: 11.16 minutes
Precursor m/z : 195.1427
Fit (%) 97.5% **RFit (%)** 63.9%

Exp RT: 11.20 minutes
Analyte Name:
195.1427 / 11.20

Collision Energy = 35 ± 15 eV

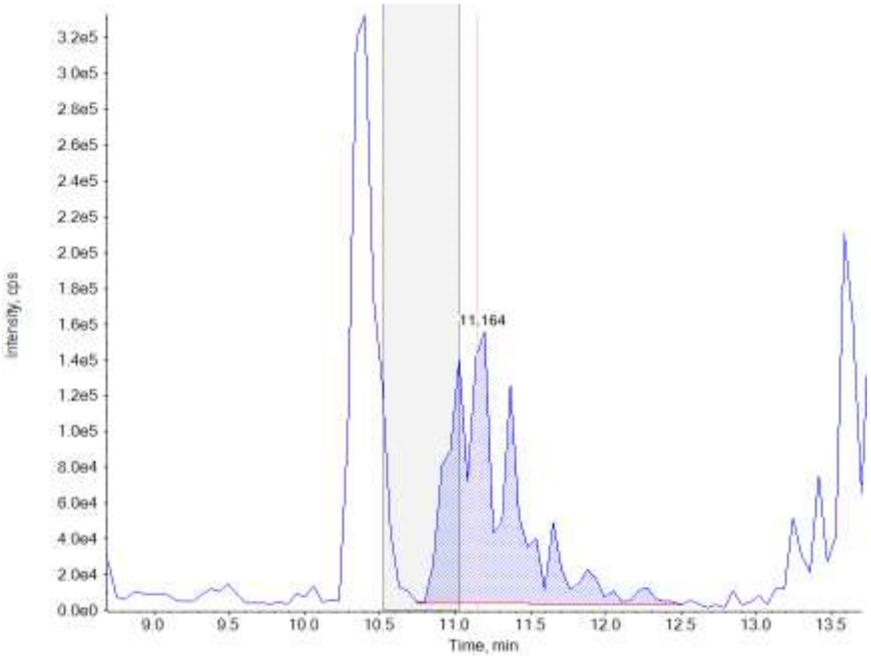

Acquired / Library MSMS

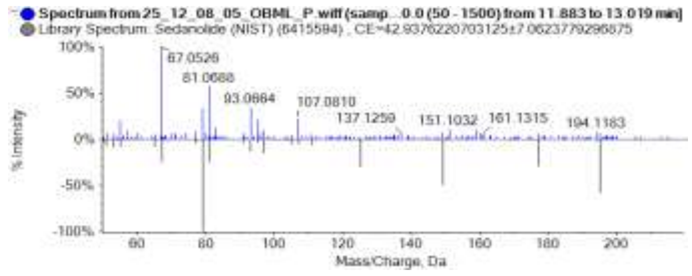

Acquired / Theoretical MS

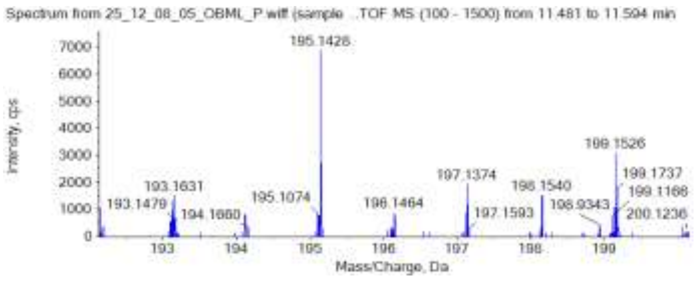

Created with SCIEX OS 3.3.0
Printed:12/9/2025 2:05:30 PM

389.3274 / 11.20

(Mass/FragMass/RT/Isotope/Library/Formula/Ion Ratio)

Retention Time: 11.20 minutes
Precursor m/z : 389.3274
Fit (%) 100.0% **RFit (%)** 97.5%

Exp RT: 11.20 minutes
Analyte Name:
389.3274 / 11.20

Collision Energy = 35 ± 15 eV

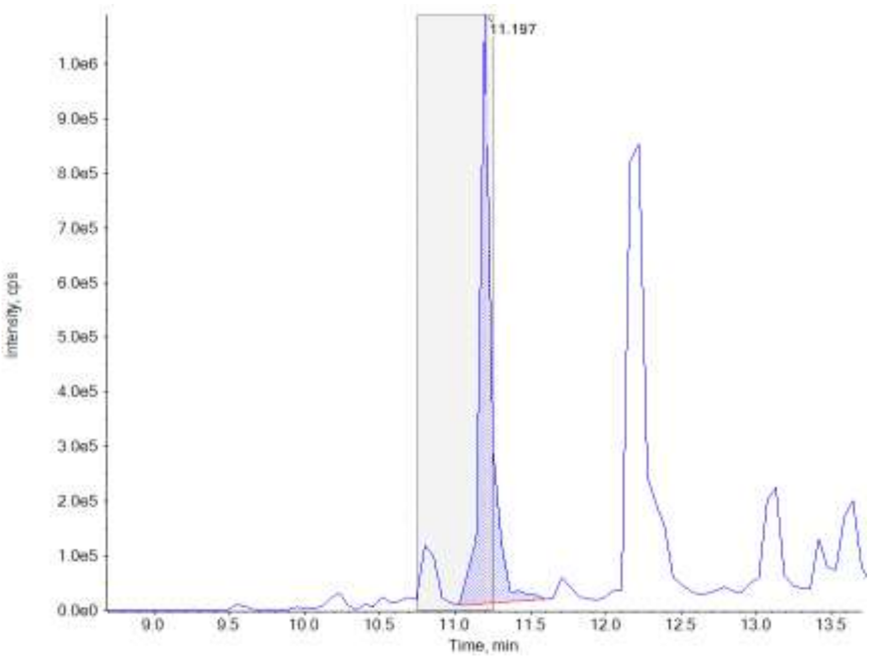

Acquired / Library MSMS

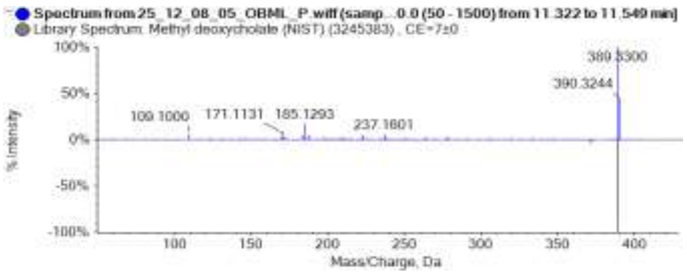

Acquired / Theoretical MS

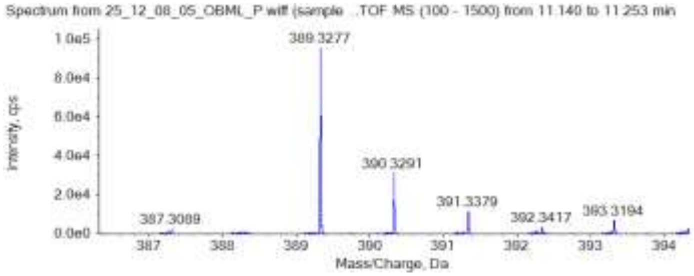

407.3386 / 11.20 (Mass/FragMass/RT/Isotope/Library/Formula/Ion Ratio)

Retention Time: 11.20 minutes
Precursor m/z : 407.3386
Fit (%) 100.0% **RFit (%)** 45.8%

Exp RT: 11.20 minutes
Analyte Name:
407.3386 / 11.20

Collision Energy = 35 ± 15 eV

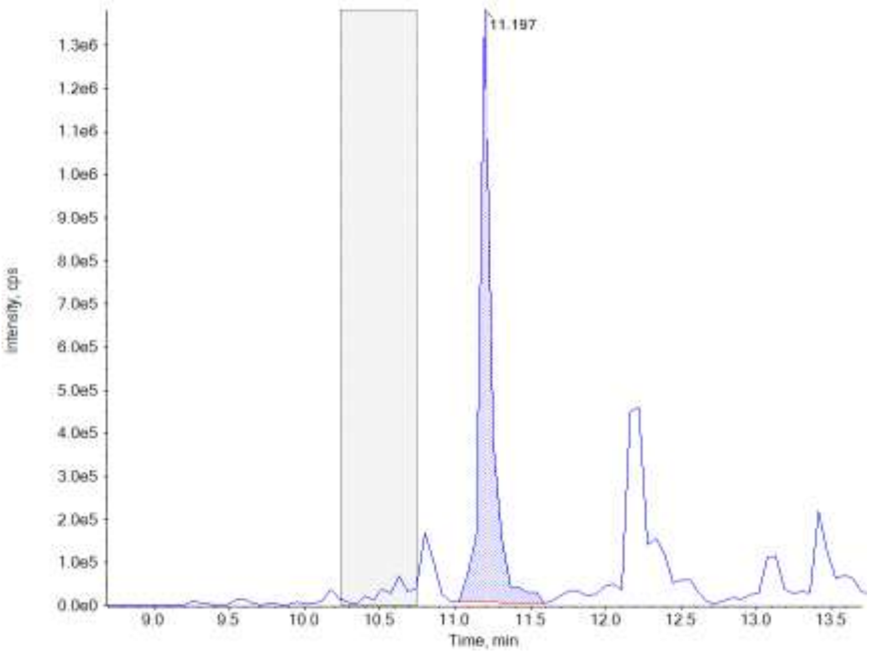

Acquired / Library MSMS

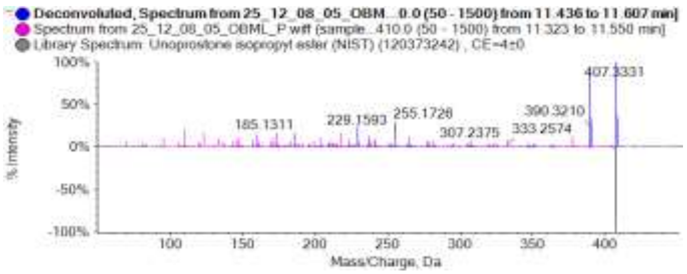

Acquired / Theoretical MS

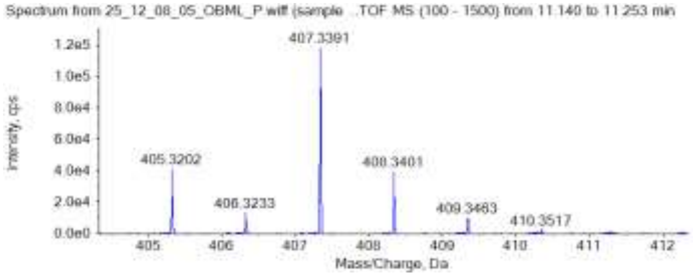

746.4889 / 11.25 (Mass/FragMass/RT/Isotope/Library/Formula/Ion Ratio)

**Retention Time:** 11.27 minutes	**Exp RT:** 11.25 minutes
**Precursor m/z :** 746.4889	**Analyte Name:**
**Fit (%)** 74.8% **RFit (%)** 92.9%	746.4889 / 11.25

Collision Energy = 35 ± 15 eV

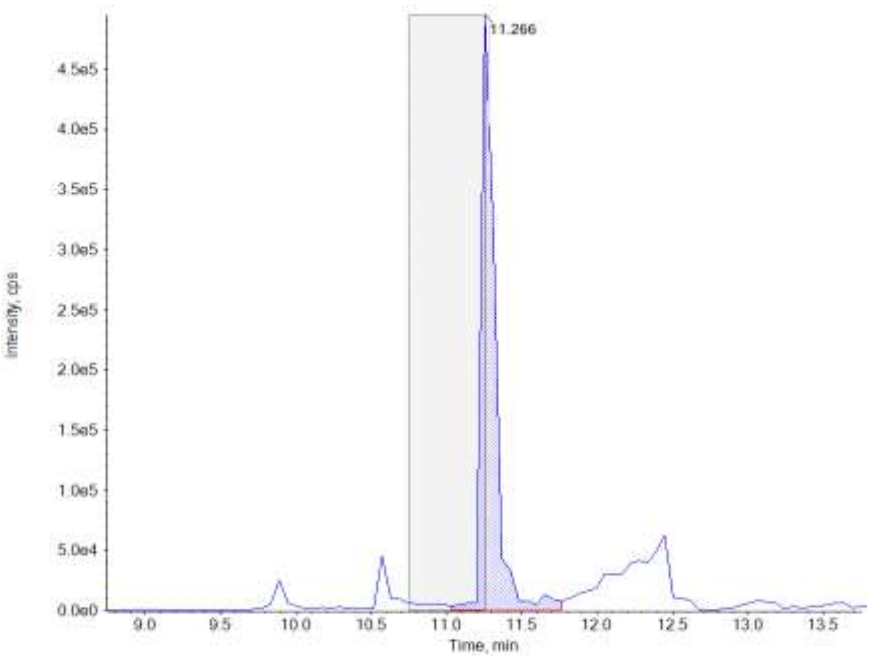

Acquired / Library MSMS

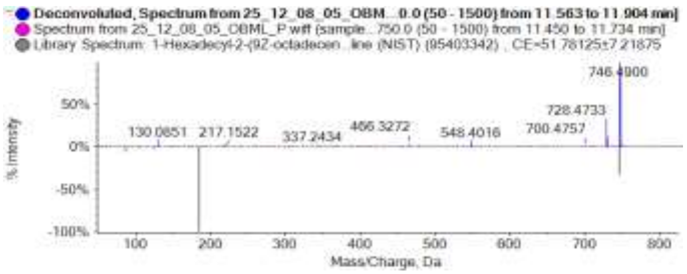

Acquired / Theoretical MS

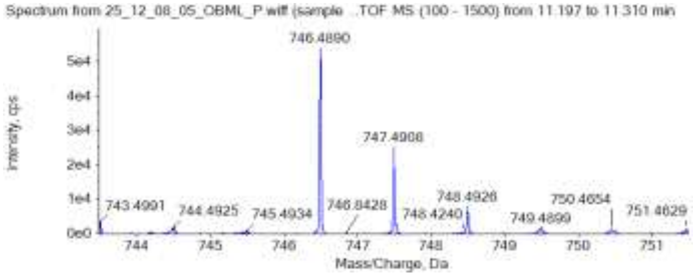

181.1297 / 11.59 (Mass/FragMass/RT/Isotope/Library/Formula/Ion Ratio)

Retention Time: 11.59 minutes
Precursor m/z : 181.1297
Fit (%) 96.2% **RFit (%)** 87.9%

Exp RT: 11.59 minutes
Analyte Name:
181.1297 / 11.59

Collision Energy = 35 ± 15 eV

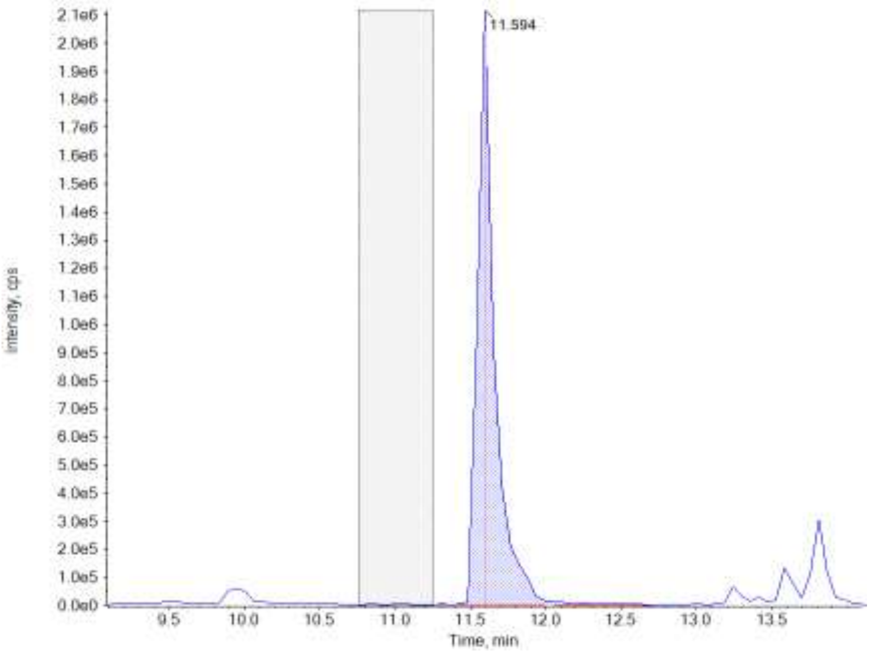

Acquired / Library MSMS

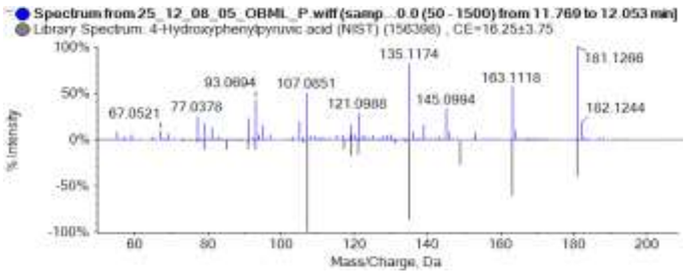

Acquired / Theoretical MS

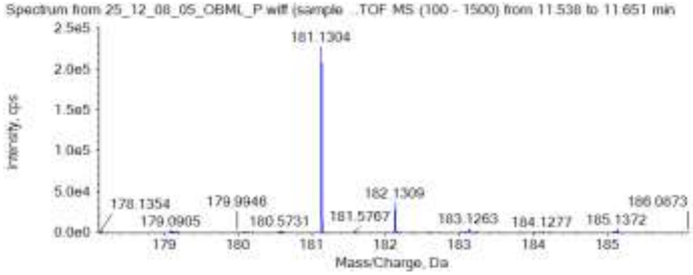

391.3432 / 11.59 (Mass/FragMass/RT/Isotope/Library/Formula/Ion Ratio)

**Retention Time:** 11.61 minutes	**Exp RT:** 11.59 minutes
**Precursor m/z :** 391.3432	**Analyte Name:**
**Fit (%)** 100.0% **RFit (%)** 79.3%	391.3432 / 11.59

Collision Energy = 35 ± 15 eV

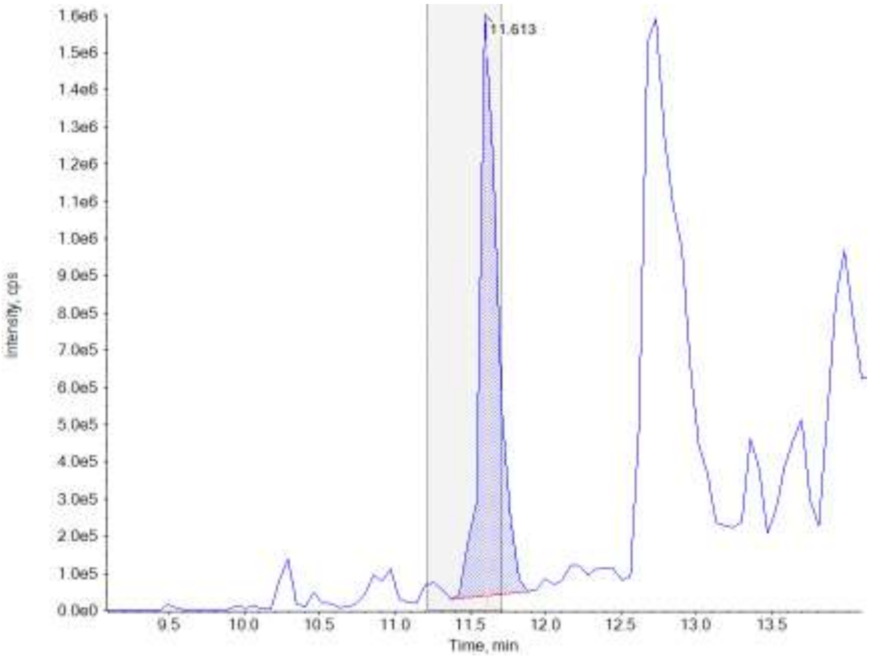

Acquired / Library MSMS

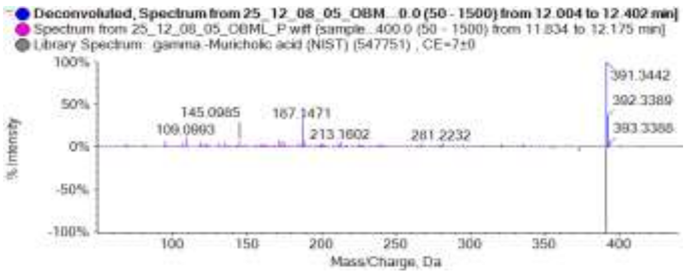

Acquired / Theoretical MS

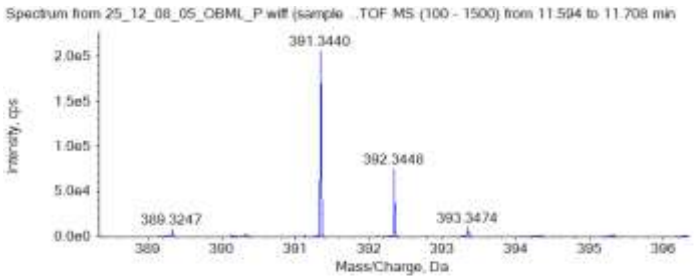

Created with SCIEX OS 3.3.0
Printed:12/9/2025 2:05:30 PM

419.3413 / 11.59 (Mass/FragMass/RT/Isotope/Library/Formula/Ion Ratio)

Retention Time: 11.62 minutes
Precursor m/z : 419.3413
Fit (%) 74.6% **RFit (%)** 79.3%

Exp RT: 11.59 minutes
Analyte Name:
419.3413 / 11.59

Collision Energy = 35 ± 15 eV

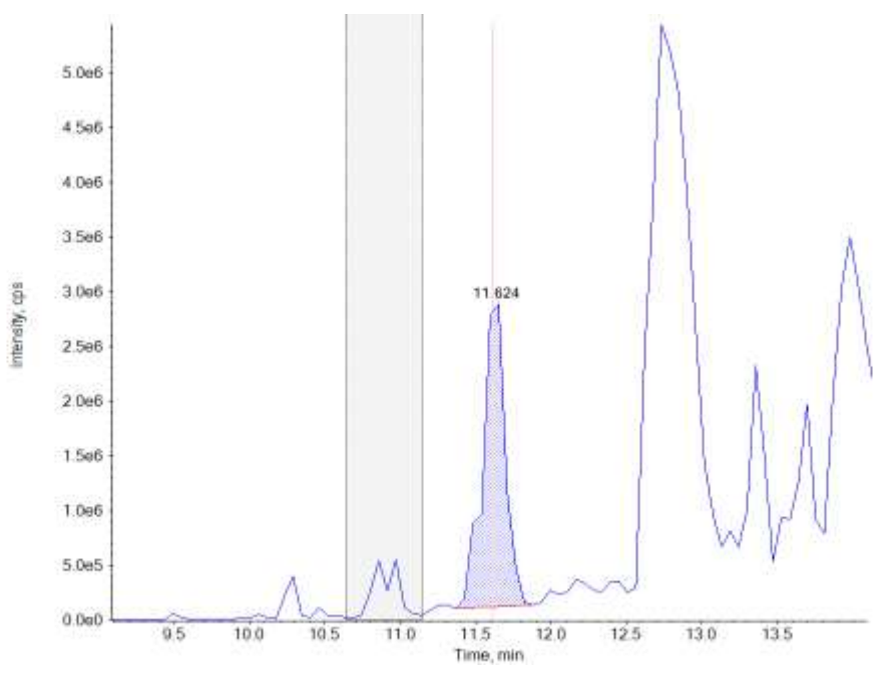

Acquired / Library MSMS

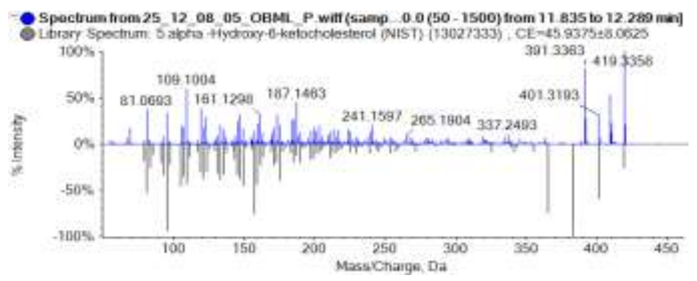

Acquired / Theoretical MS

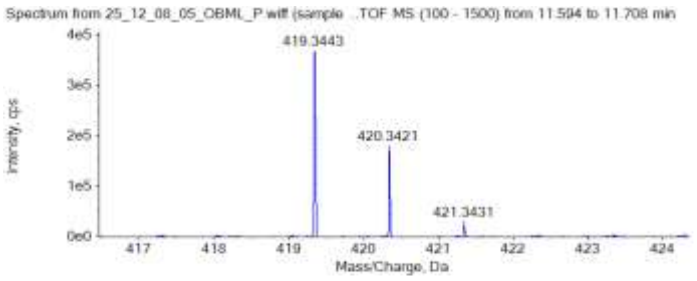

291.2017 / 11.71 (Mass/FragMass/RT/Isotope/Library/Formula/Ion Ratio)

Retention Time: 12.20 minutes
Precursor m/z : 291.2017
Fit (%) 49.1% **RFit (%)** 96.5%

Exp RT: 11.71 minutes
Analyte Name:
291.2017 / 11.71

Collision Energy = 35 ± 15 eV

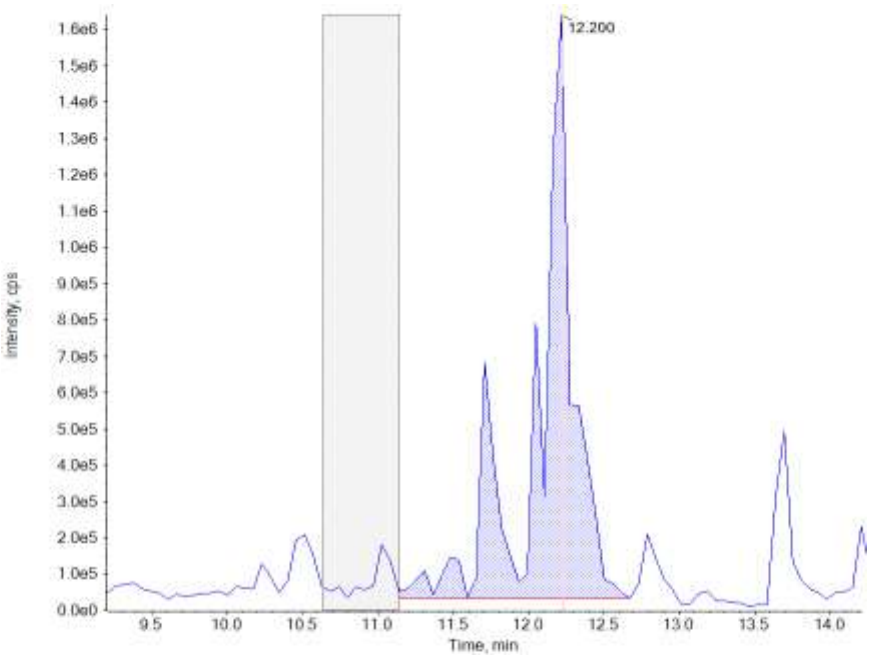

Acquired / Library MSMS

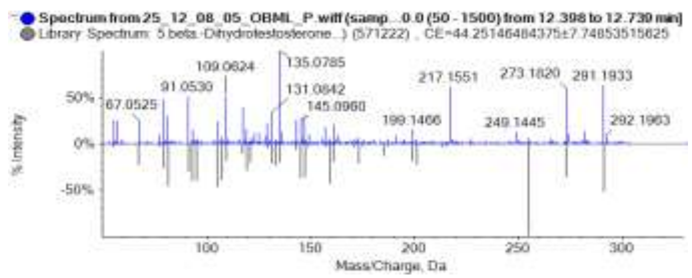

Acquired / Theoretical MS

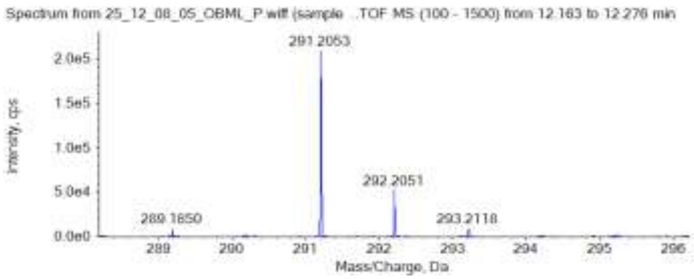

730.4933 / 11.71 (Mass/FragMass/RT/Isotope/Library/Formula/Ion Ratio)

Retention Time: 11.77 minutes
Precursor m/z : 730.4933
Fit (%) 97.8% **RFit (%)** 85.4%

Exp RT: 11.71 minutes
Analyte Name:
730.4933 / 11.71

Collision Energy = 35 ± 15 eV

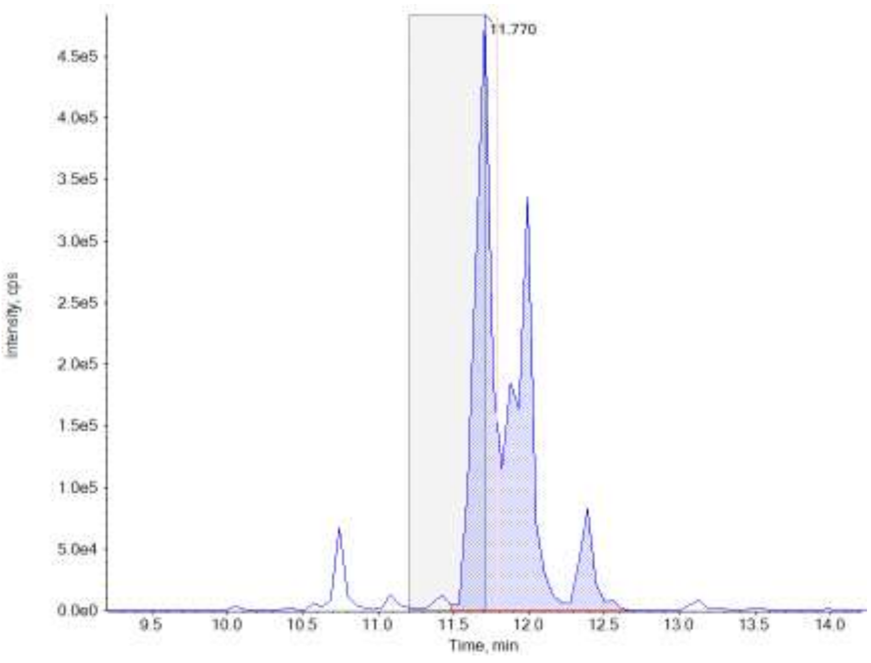

Acquired / Library MSMS

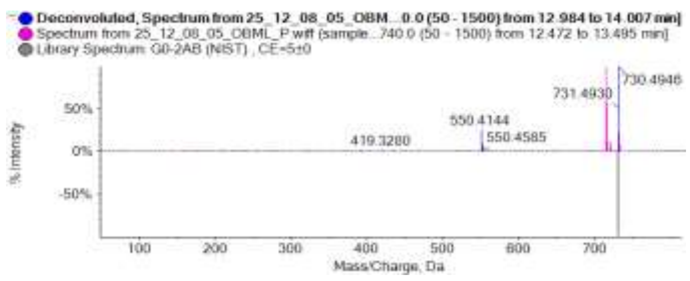

Acquired / Theoretical MS

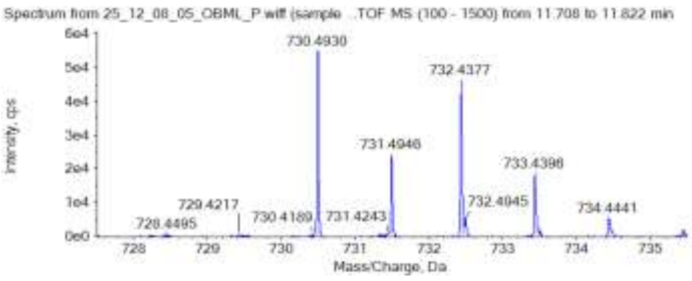

428.2737 / 11.76 (Mass/FragMass/RT/Isotope/Library/Formula/Ion Ratio)

Retention Time: 11.77 minutes
Precursor m/z : 428.2737
Fit (%) 95.3% **RFit (%)** 88.7%

Exp RT: 11.77 minutes
Analyte Name:
428.2737 / 11.76

Collision Energy = 35 ± 15 eV

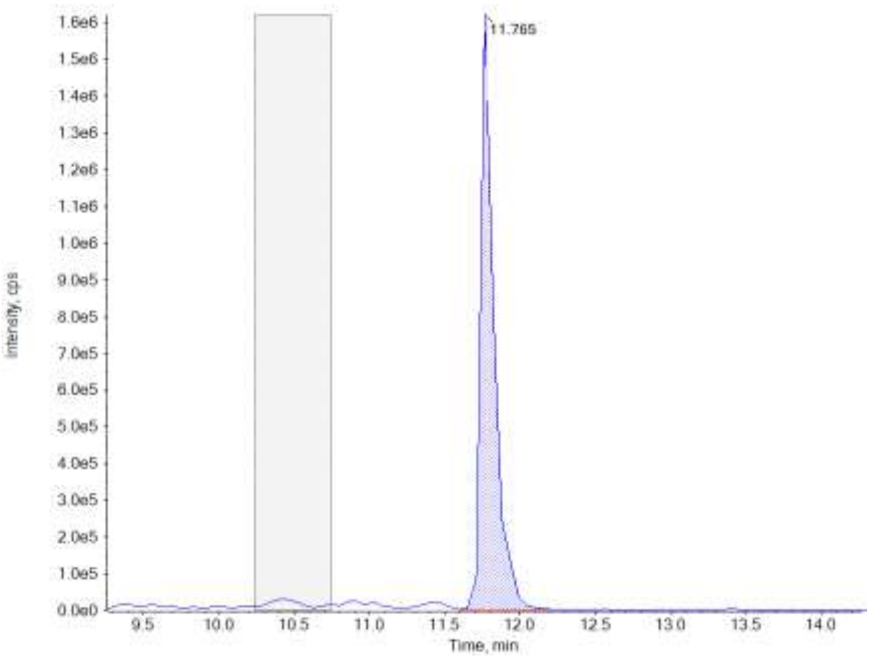

Acquired / Library MSMS

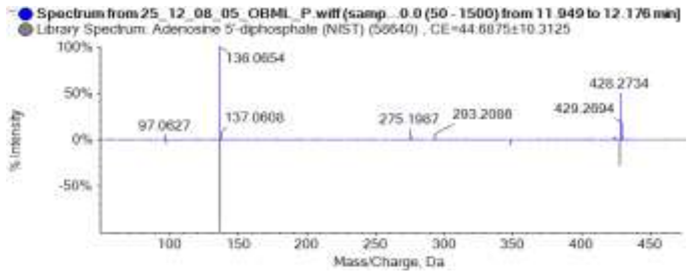

Acquired / Theoretical MS

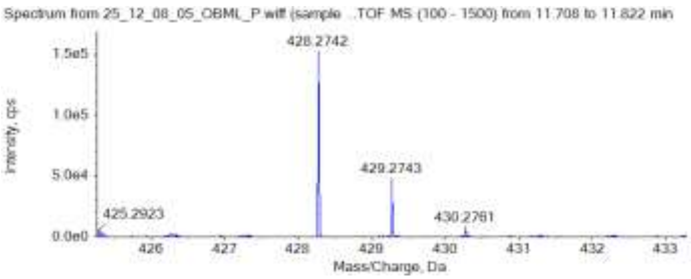

439.3265 / 11.99 (Mass/FragMass/RT/Isotope/Library/Formula/Ion Ratio)

Retention Time: 12.00 minutes
Precursor m/z : 439.3265
Fit (%) 93.4% **RFit (%)** 89.2%

Exp RT: 11.99 minutes
Analyte Name:
439.3265 / 11.99

Collision Energy = 35 ± 15 eV

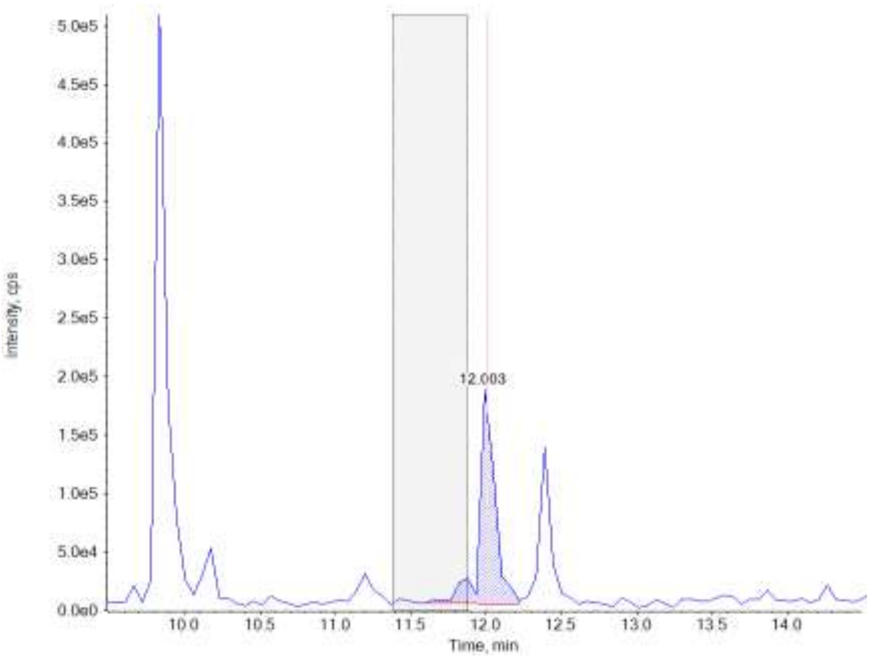

Acquired / Library MSMS

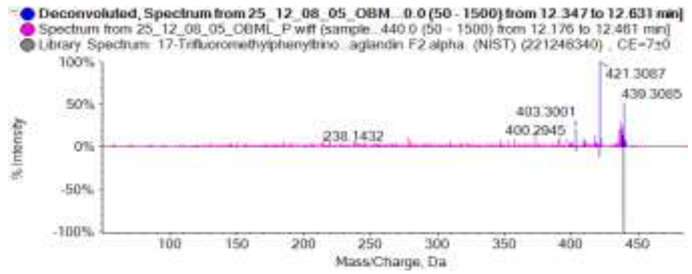

Acquired / Theoretical MS

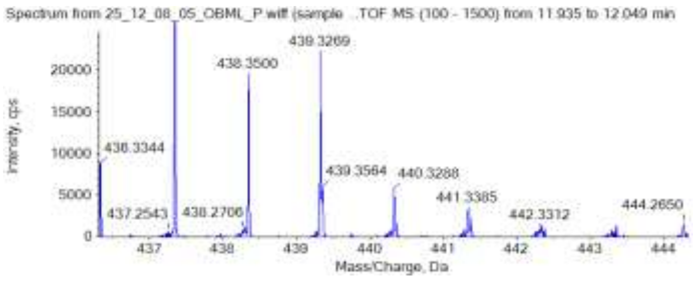

373.1373 / 12.11 (Mass/FragMass/RT/Isotope/Library/Formula/Ion Ratio)

Retention Time: 12.11 minutes **Exp RT:** 12.11 minutes
Precursor m/z : 373.1373 **Analyte Name:**
Fit (%) 100.0% **RFit (%)** 29.6% 373.1373 / 12.11

Collision Energy = 35 ± 15 eV

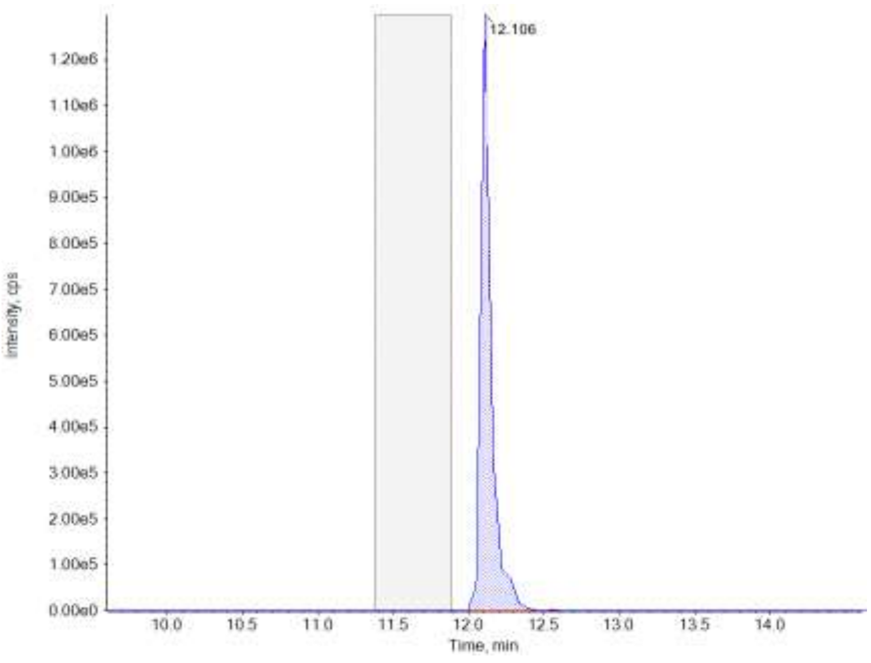

Acquired / Library MSMS

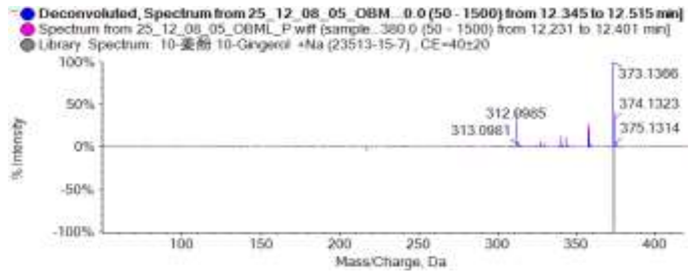

Acquired / Theoretical MS

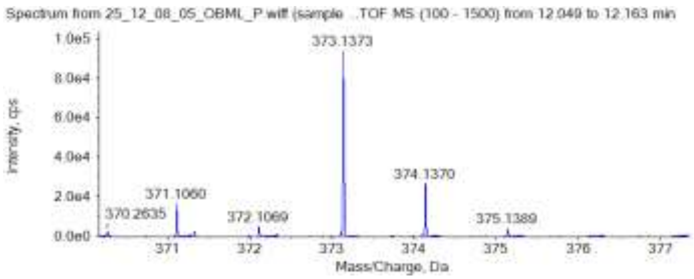

273.1906 / 12.22 (Mass/FragMass/RT/Isotope/Library/Formula/Ion Ratio)

Retention Time: 12.01 minutes | **Exp RT:** 12.22 minutes
Precursor m/z : 273.1905 | **Analyte Name:**
Fit (%) 100.0% **RFit (%)** 100.0% | 273.1906 / 12.22

Collision Energy = 35 ± 15 eV

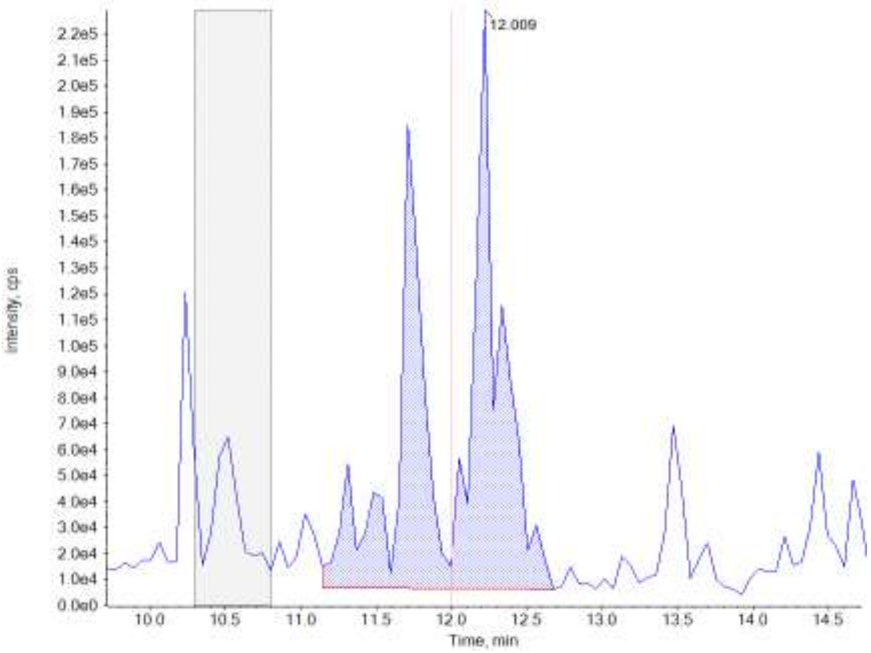

Acquired / Library MSMS

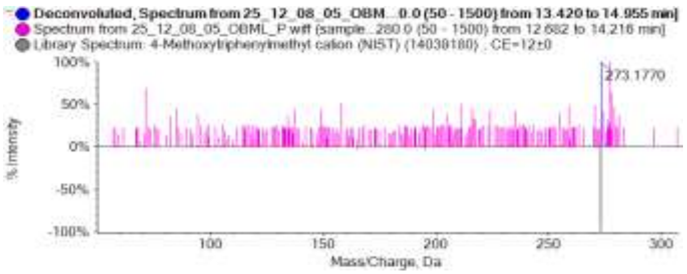

Acquired / Theoretical MS

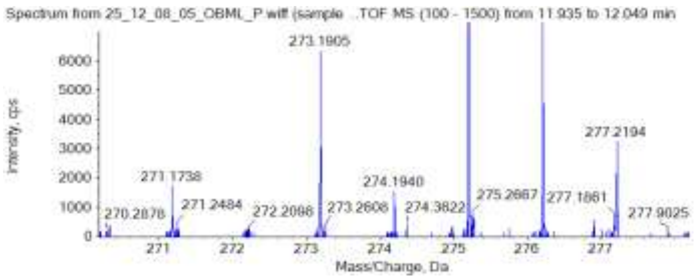

291.2039 / 12.22 (Mass/FragMass/RT/Isotope/Library/Formula/Ion Ratio)

Retention Time: 12.20 minutes
Precursor m/z : 291.2038
Fit (%) 49.1% **RFit (%)** 96.5%

Exp RT: 12.22 minutes
Analyte Name:
291.2039 / 12.22

Collision Energy = 35 ± 15 eV

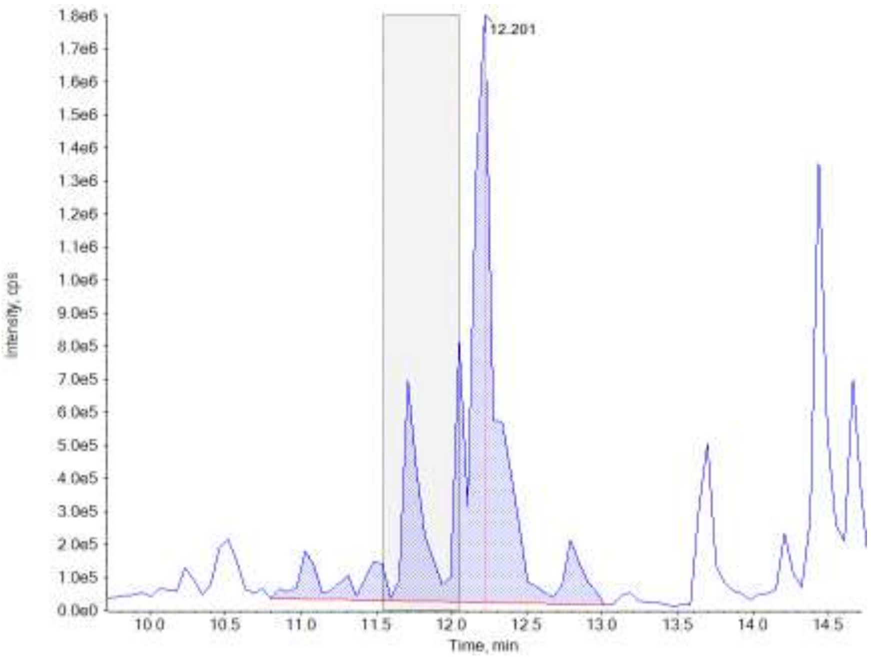

Acquired / Library MSMS

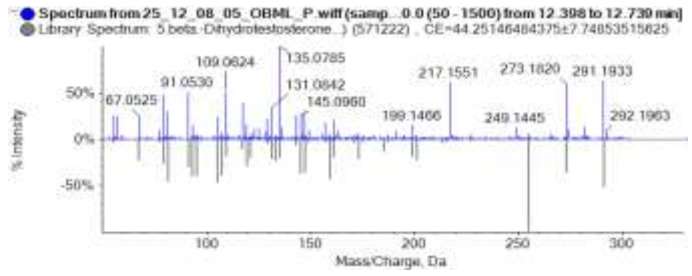

Acquired / Theoretical MS

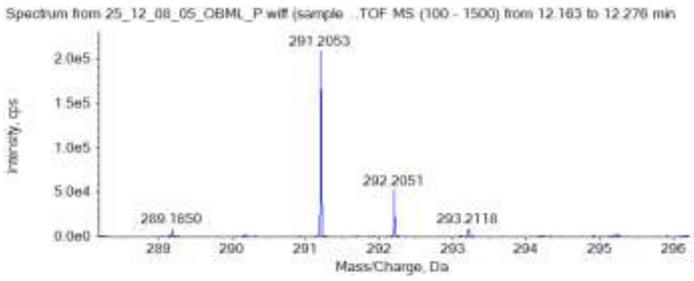

389.3271 / 12.22 (Mass/FragMass/RT/Isotope/Library/Formula/Ion Ratio)

Retention Time: 12.19 minutes
Precursor m/z : 389.3271
Fit (%) 100.0% **RFit (%)** 77.6%

Exp RT: 12.22 minutes
Analyte Name:
389.3271 / 12.22

Collision Energy = 35 ± 15 eV

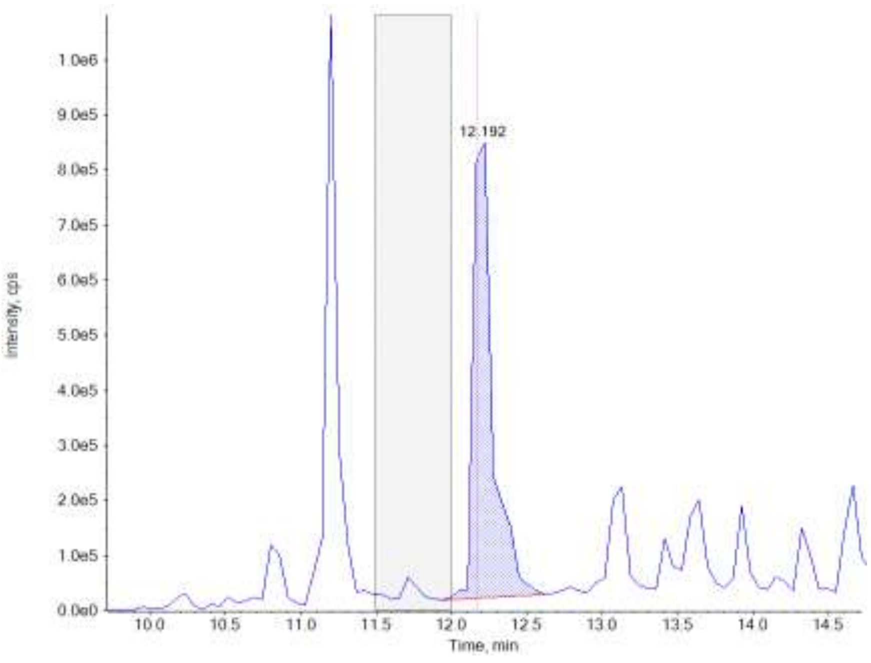

Acquired / Library MSMS

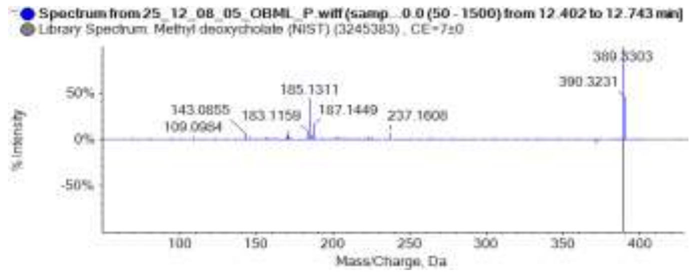

Acquired / Theoretical MS

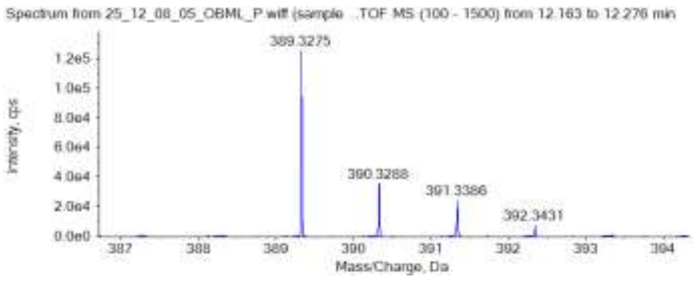

425.3462 / 12.22

(Mass/FragMass/RT/Isotope/Library/Formula/Ion Ratio)

Retention Time: 12.19 minutes
Precursor m/z : 425.3462
Fit (%) 75.3% **RFit (%)** 100.0%

Exp RT: 12.22 minutes
Analyte Name:
425.3462 / 12.22

Collision Energy = 35 ± 15 eV

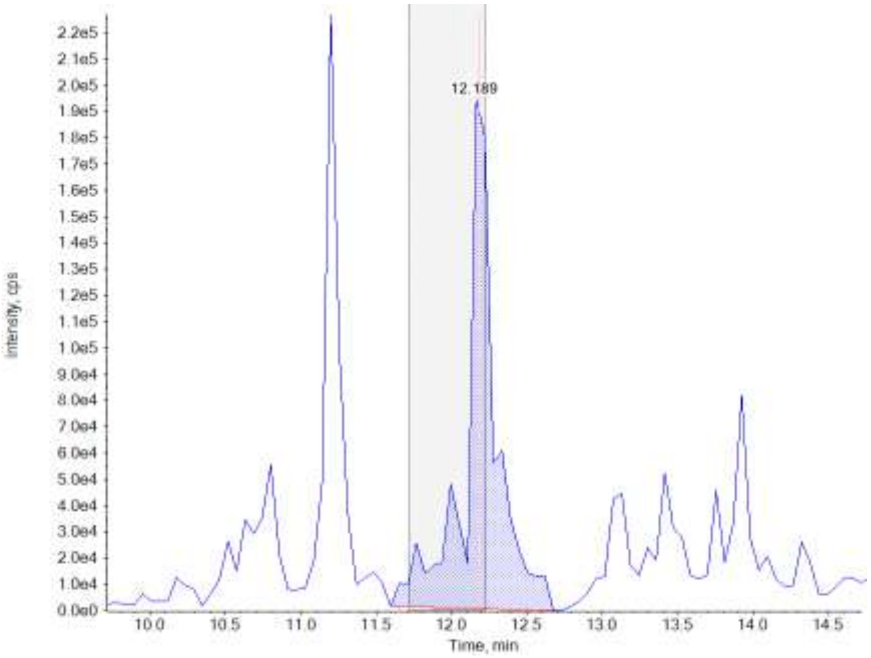

Acquired / Library MSMS

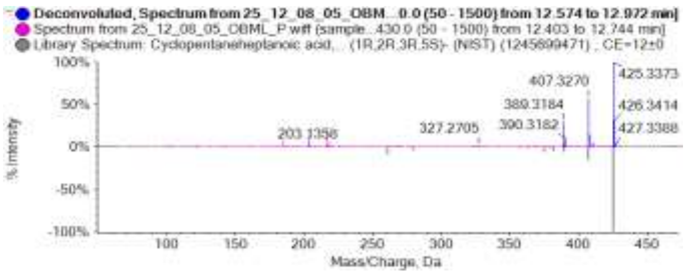

Acquired / Theoretical MS

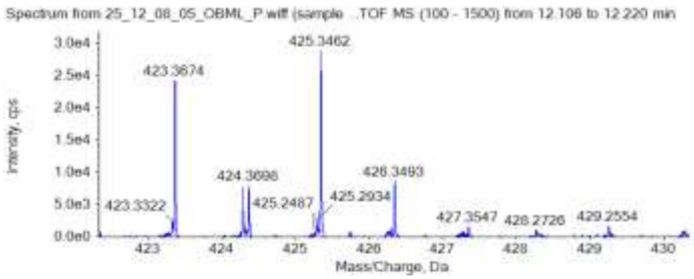

Created with SCIEX OS 3.3.0
Printed:12/9/2025 2:05:30 PM

498.4000 / 12.33 (Mass/FragMass/RT/Isotope/Library/Formula/Ion Ratio)

**Retention Time:** 12.33 minutes	**Exp RT:** 12.33 minutes
**Precursor m/z :** 498.4000	**Analyte Name:**
**Fit (%)** 76.5% **RFit (%)** 100.0%	498.4000 / 12.33

Collision Energy = 35 ± 15 eV

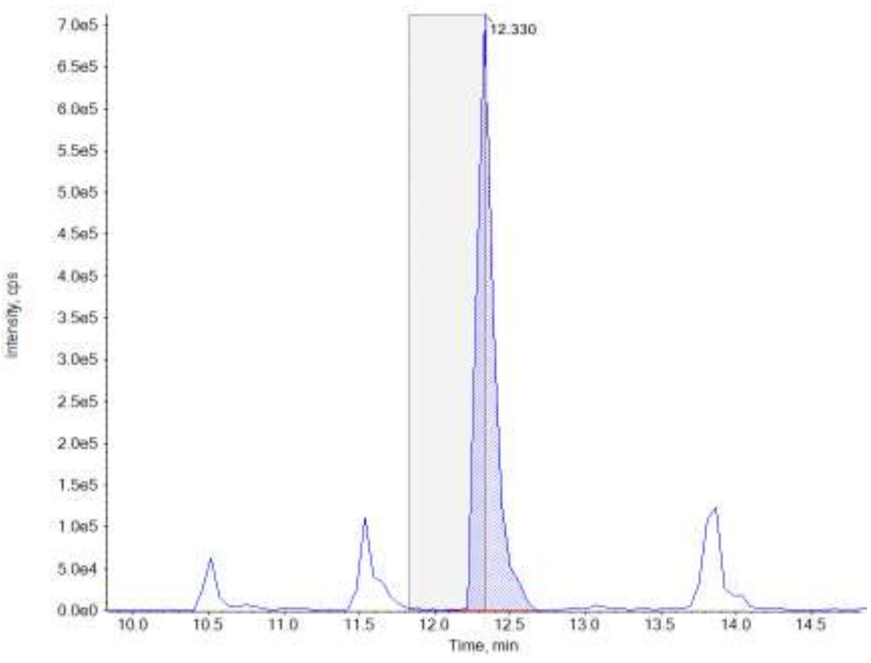

Acquired / Library MSMS

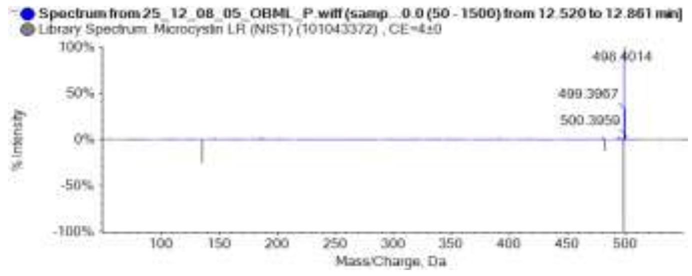

Acquired / Theoretical MS

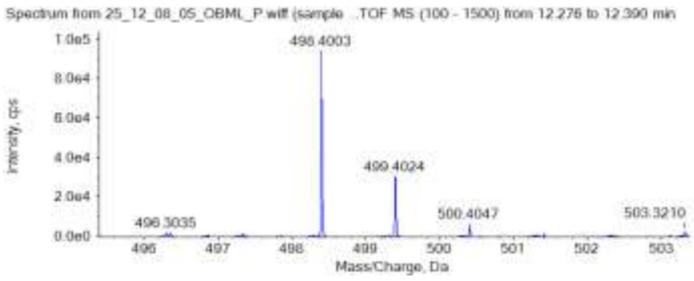

283.1061 / 12.50

(Mass/FragMass/RT/Isotope/Library/Formula/Ion Ratio)

Retention Time: 12.52 minutes
Precursor m/z : 283.1061
Fit (%) 81.9% **RFit (%)** 99.9%

Exp RT: 12.50 minutes
Analyte Name:
283.1061 / 12.50

Collision Energy = 35 ± 15 eV

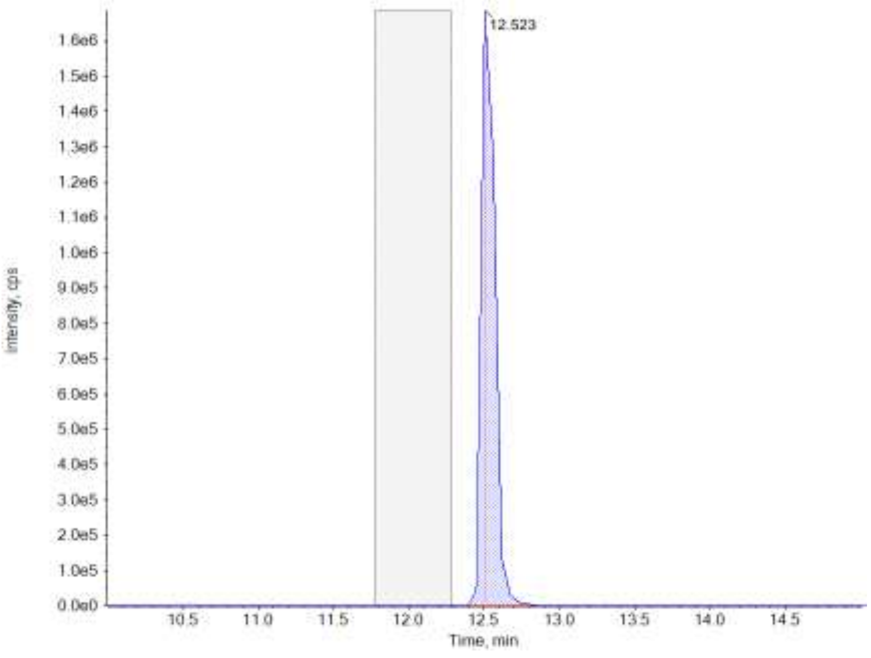

Acquired / Library MSMS

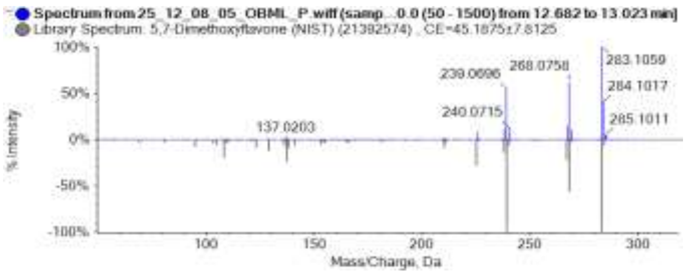

Acquired / Theoretical MS

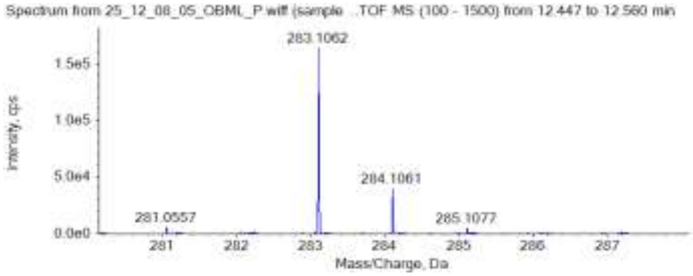

540.4138 / 12.56 (Mass/FragMass/RT/Isotope/Library/Formula/Ion Ratio)

Retention Time: 12.54 minutes
Precursor m/z : 540.4137
Fit (%) 75.5% **RFit (%)** 99.4%

Exp RT: 12.56 minutes
Analyte Name:
540.4138 / 12.56

Collision Energy = 35 ± 15 eV

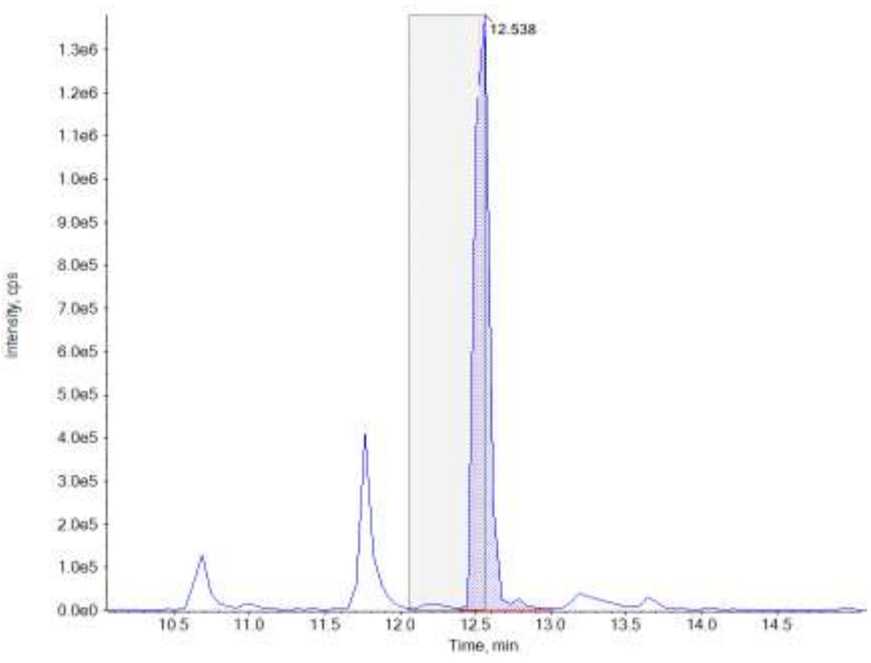

Acquired / Library MSMS

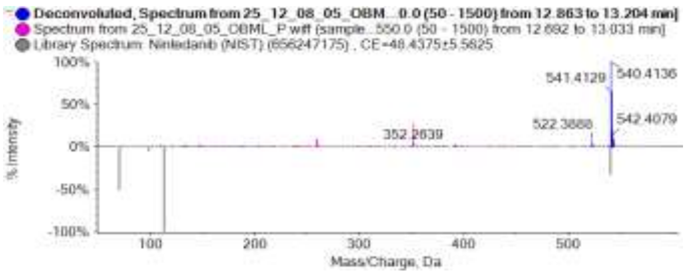

Acquired / Theoretical MS

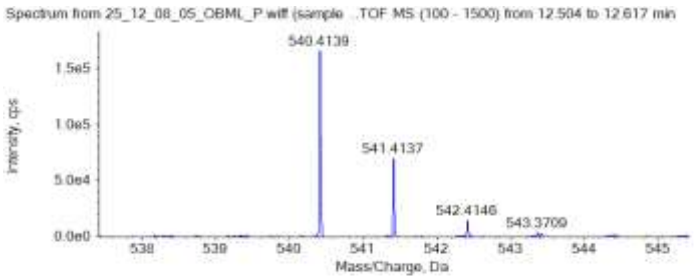

313.1138 / 12.62

(Mass/FragMass/RT/Isotope/Library/Formula/Ion Ratio)

Retention Time: 12.62 minutes
Precursor m/z : 313.1138
Fit (%) 100.0% **RFit (%)** 70.7%

Exp RT: 12.62 minutes
Analyte Name:
313.1138 / 12.62

Collision Energy = 35 ± 15 eV

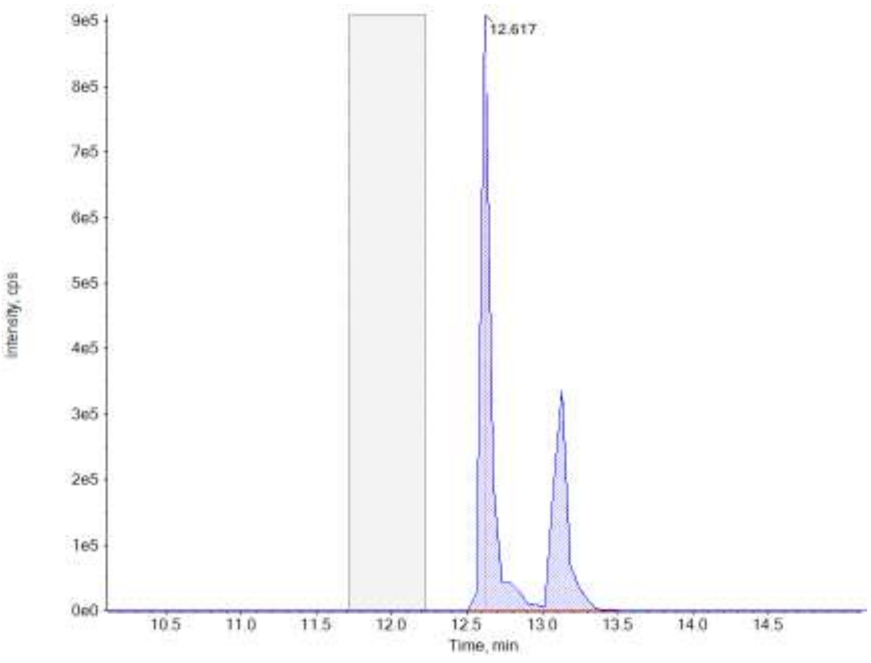

Acquired / Library MSMS

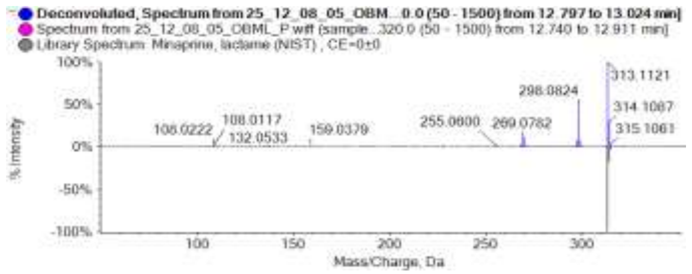

Acquired / Theoretical MS

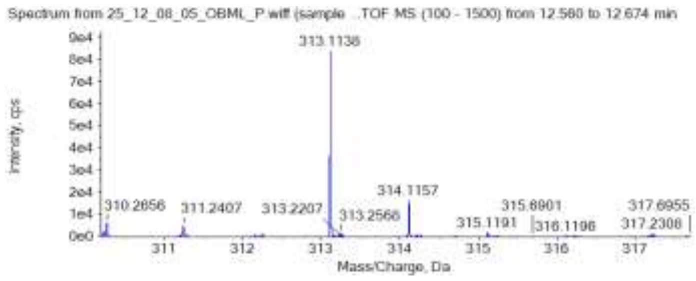

355.2683 / 12.73 (Mass/FragMass/RT/Isotope/Library/Formula/Ion Ratio)

Retention Time: 12.77 minutes
Precursor m/z : 355.2683
Fit (%) 66.9% **RFit (%)** 78.6%

Exp RT: 12.73 minutes
Analyte Name:
355.2683 / 12.73

Collision Energy = 35 ± 15 eV

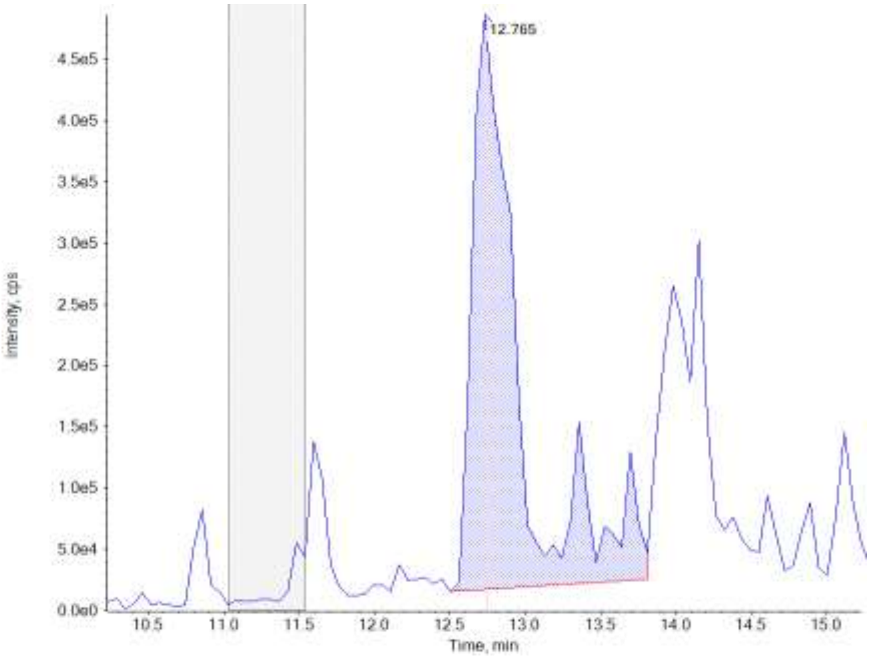

Acquired / Library MSMS

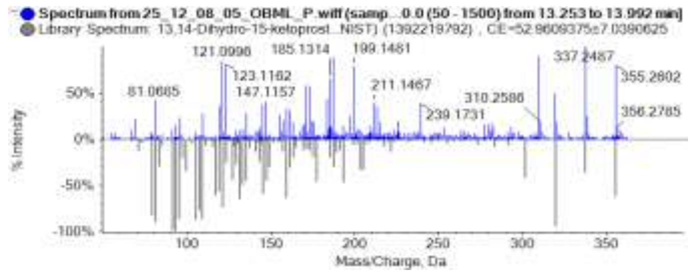

Acquired / Theoretical MS

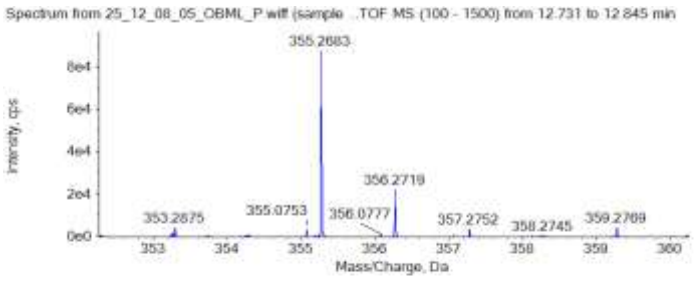

Created with SCIEX OS 3.3.0
Printed:12/9/2025 2:05:30 PM

391.3422 / 12.73 [M+H]+ (Mass/FragMass/RT/Isotope/Library/Formula/Ion Ratio)

Retention Time: 12.75 minutes
Precursor m/z : 391.3422
Fit (%) 100.0% **RFit (%)** 23.2%

Exp RT: 12.73 minutes
Analyte Name:
391.3422 / 12.73 [M+H]+

Collision Energy = 35 ± 15 eV

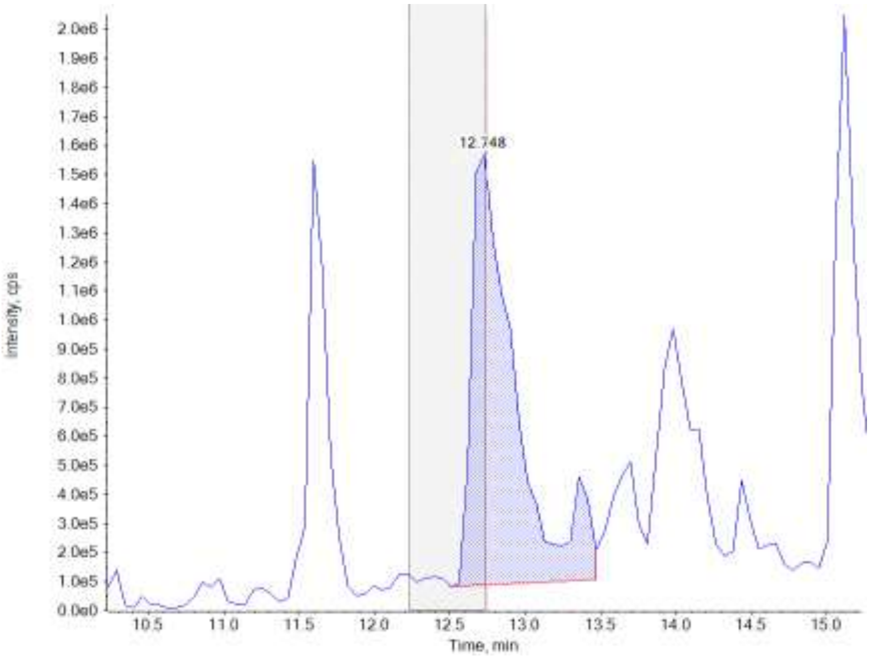

Acquired / Library MSMS

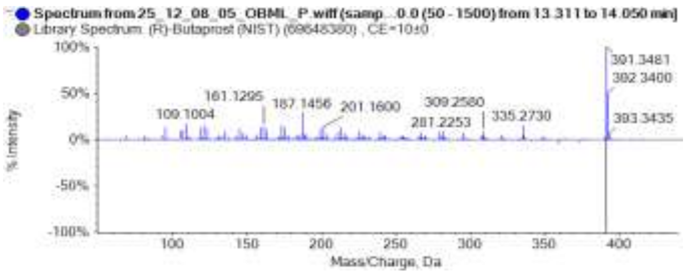

Acquired / Theoretical MS

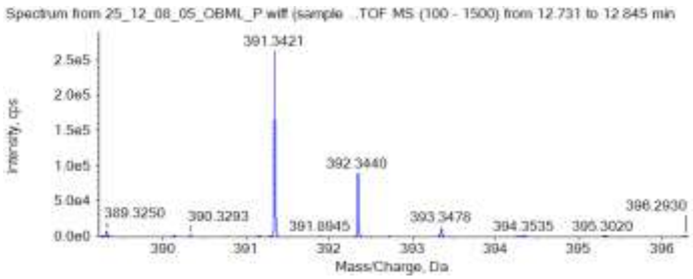

423.3679 / 12.45 [M+CH3OH+H]+ (Mass/FragMass/RT/Isotope/Library/Formula/Ion Ratio)

**Retention Time:** 12.45 minutes	**Exp RT:** 12.45 minutes
**Precursor m/z :** 423.3679	**Analyte Name:**
**Fit (%)** 100.0% **RFit (%)** 83.6%	423.3679 / 12.45 [M+CH3OH+H]+

Collision Energy = 35 ± 15 eV

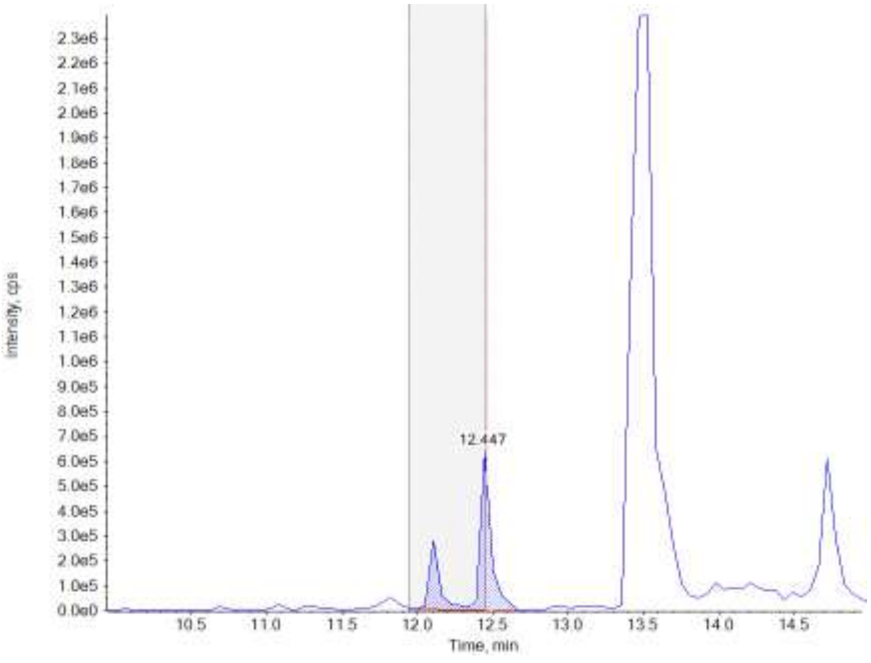

Acquired / Library MSMS

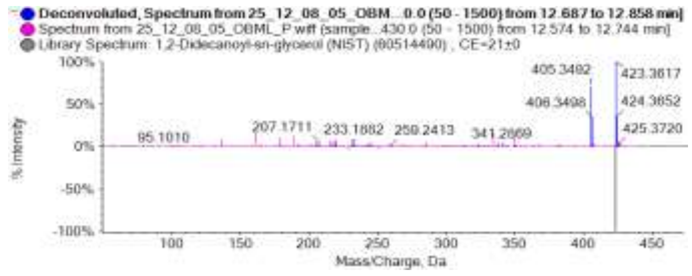

Acquired / Theoretical MS

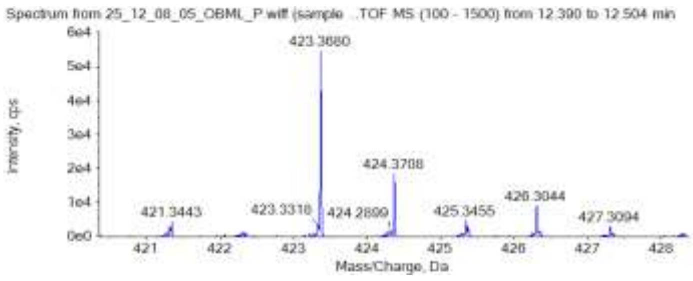

401.3255 / 12.73 (Mass/FragMass/RT/Isotope/Library/Formula/Ion Ratio)

Retention Time: 12.77 minutes
Precursor m/z : 401.3255
Fit (%) 61.5% **RFit (%)** 77.9%

Exp RT: 12.73 minutes
Analyte Name:
401.3255 / 12.73

Collision Energy = 35 ± 15 eV

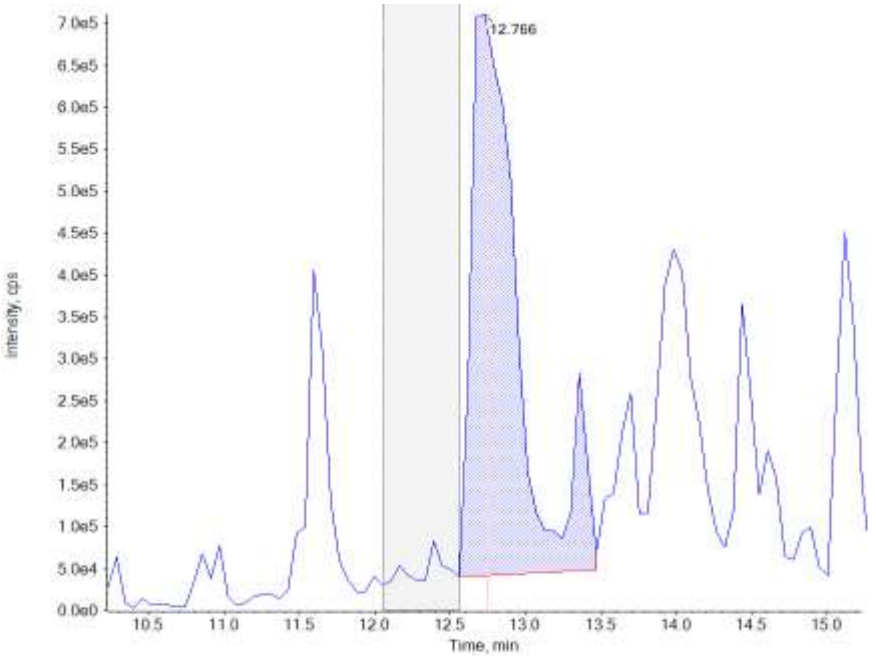

Acquired / Library MSMS

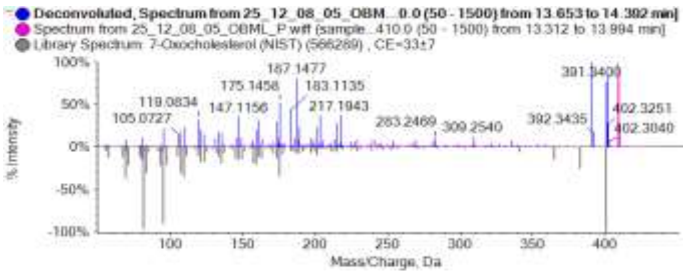

Acquired / Theoretical MS

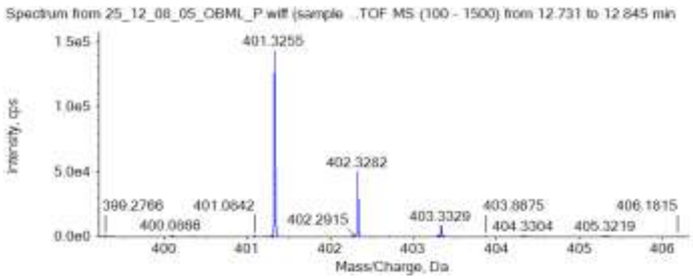

Created with SCIEX OS 3.3.0
Printed:12/9/2025 2:05:30 PM

419.3446 / 12.73 (Mass/FragMass/RT/Isotope/Library/Formula/Ion Ratio)

Retention Time: 12.78 minutes
Precursor m/z : 419.3446
Fit (%) 88.4% **RFit (%)** 77.1%

Exp RT: 12.73 minutes
Analyte Name:
419.3446 / 12.73

Collision Energy = 35 ± 15 eV

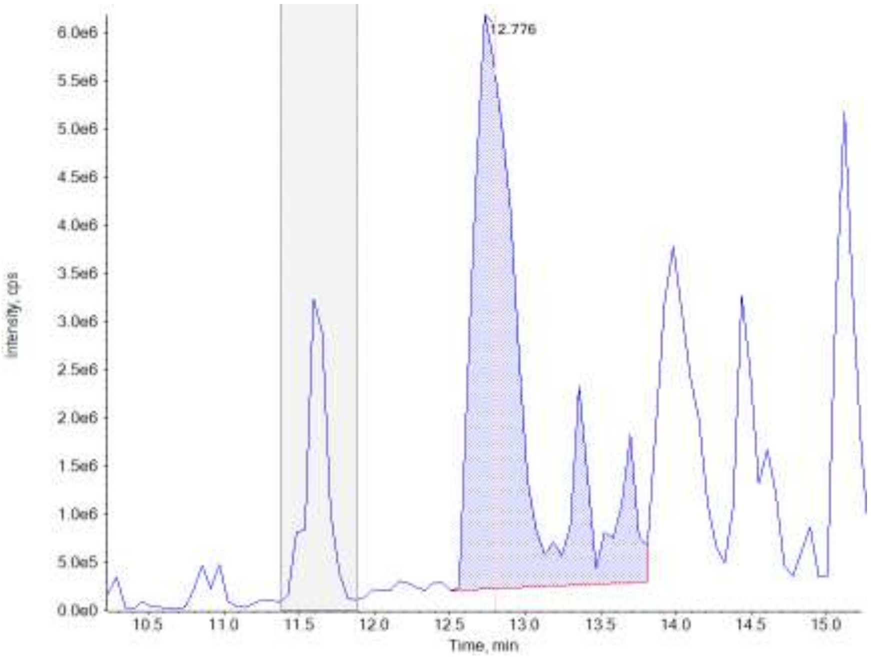

Acquired / Library MSMS

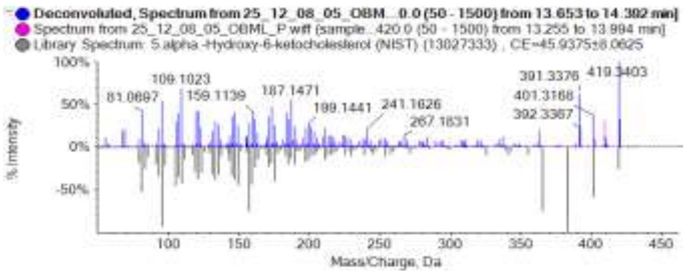

Acquired / Theoretical MS

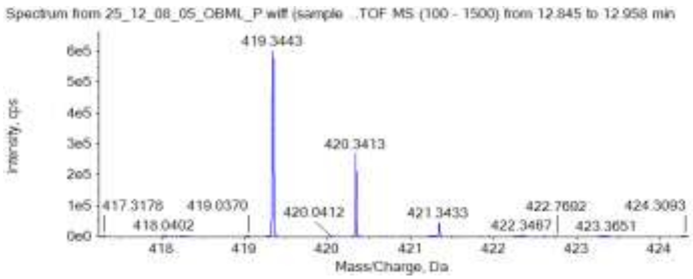

337.2580 / 12.79 (Mass/FragMass/RT/Isotope/Library/Formula/Ion Ratio)

Retention Time: 12.76 minutes
Precursor m/z : 337.2580
Fit (%) 47.5% **RFit (%)** 91.6%

Exp RT: 12.79 minutes
Analyte Name:
337.2580 / 12.79

Collision Energy = 35 ± 15 eV

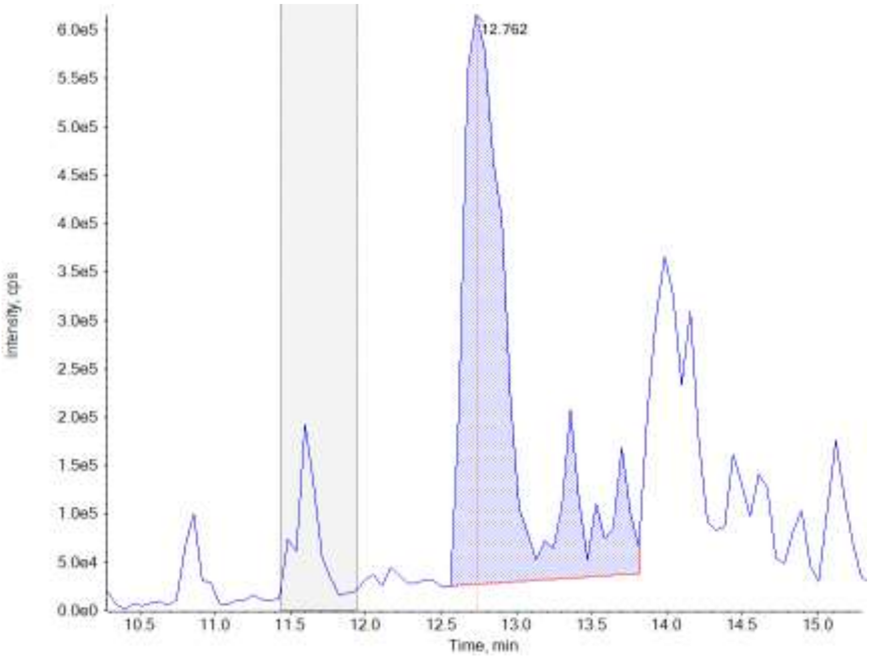

Acquired / Library MSMS

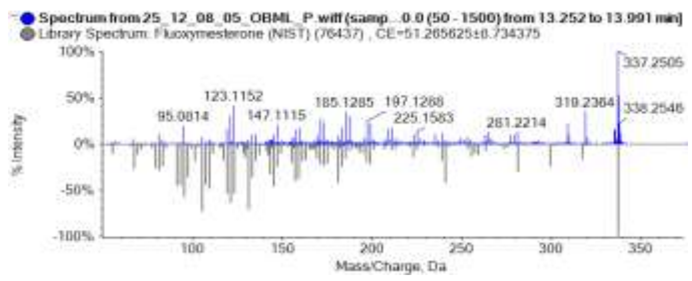

Acquired / Theoretical MS

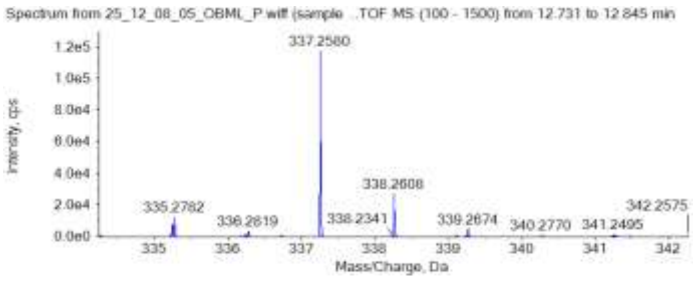

Created with SCIEX OS 3.3.0
Printed:12/9/2025 2:05:30 PM

343.1245 / 13.13 (Mass/FragMass/RT/Isotope/Library/Formula/Ion Ratio)

Retention Time: 13.13 minutes
Precursor m/z : 343.1245
Fit (%) 37.9% **RFit (%)** 100.0%

Exp RT: 13.13 minutes
Analyte Name:
343.1245 / 13.13

Collision Energy = 35 ± 15 eV

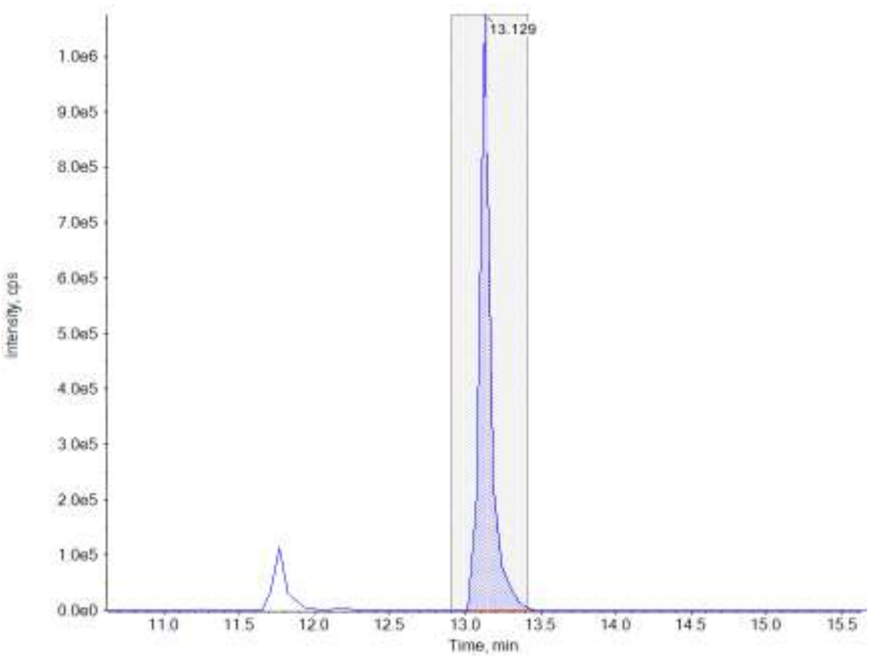

Acquired / Library MSMS

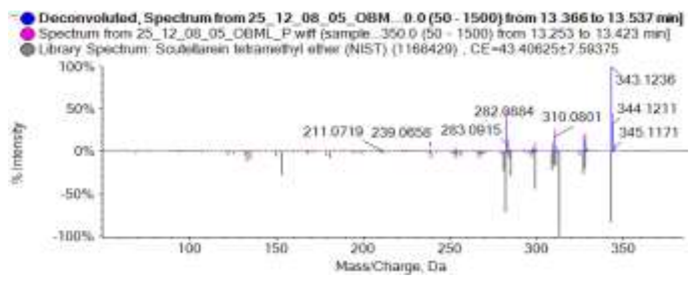

Acquired / Theoretical MS

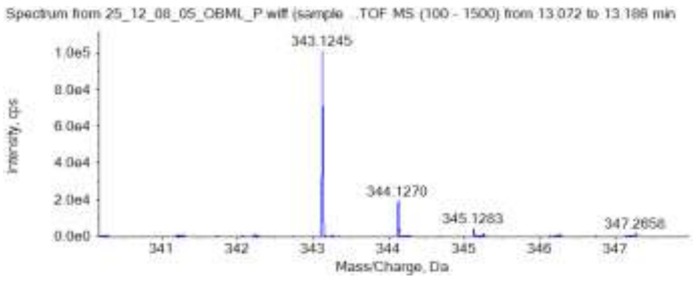

271.1494 / 13.30 (Mass/FragMass/RT/Isotope/Library/Formula/Ion Ratio)

**Retention Time:** 13.28 minutes<br>**Precursor m/z :** 271.1494<br>**Fit (%)** 90.5% **RFit (%)** 62.8%	**Exp RT:** 13.30 minutes<br>**Analyte Name:**<br>271.1494 / 13.30

Collision Energy = 35 ± 15 eV

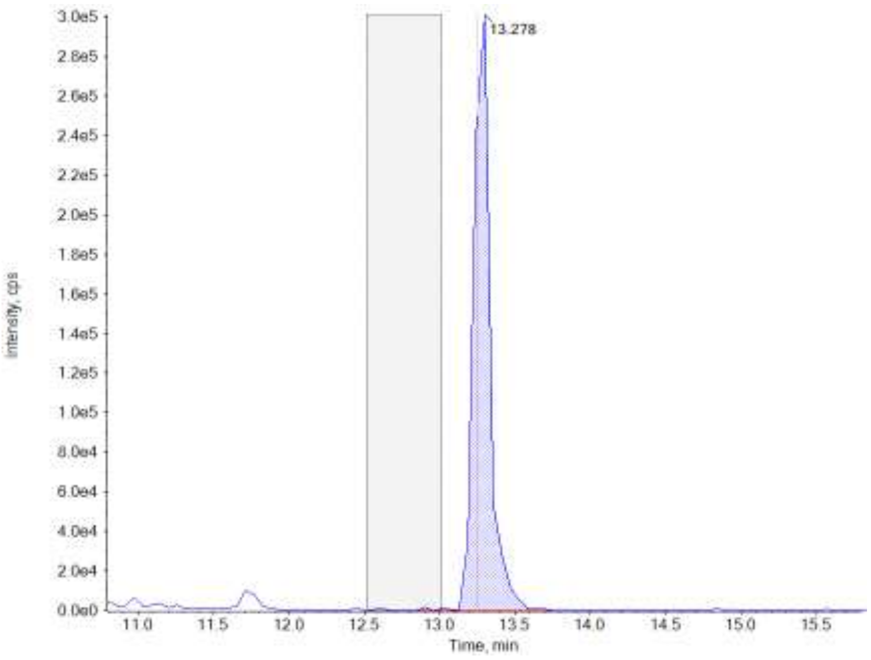

Acquired / Library MSMS

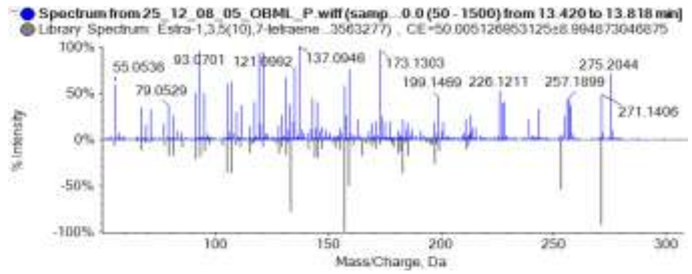

Acquired / Theoretical MS

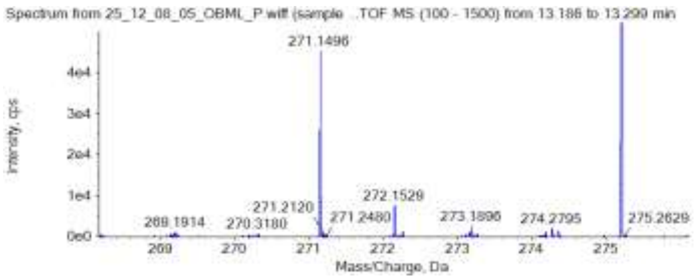

405.3573 / 13.47

(Mass/FragMass/RT/Isotope/Library/Formula/Ion Ratio)

Retention Time: 13.47 minutes
Precursor m/z : 405.3573
Fit (%) 100.0% **RFit (%)** 97.7%

Exp RT: 13.47 minutes
Analyte Name:
405.3573 / 13.47

Collision Energy = 35 ± 15 eV

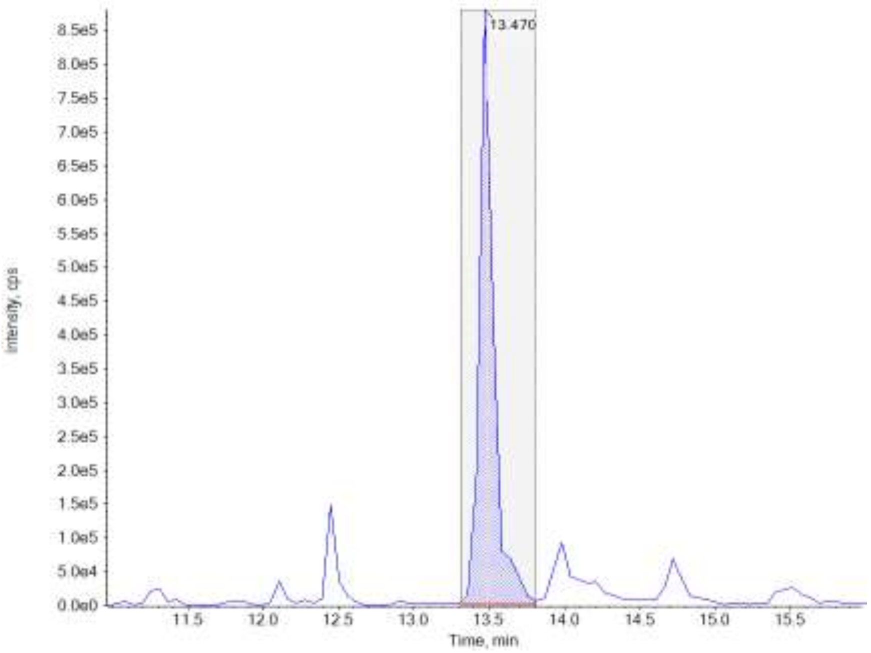

Acquired / Library MSMS

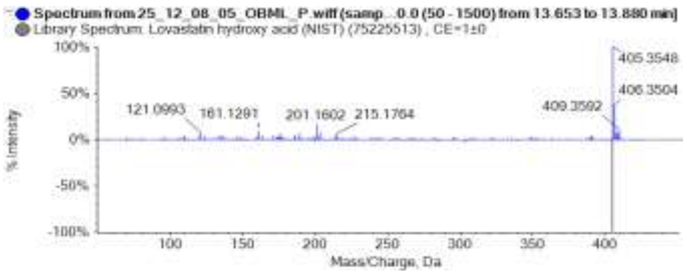

Acquired / Theoretical MS

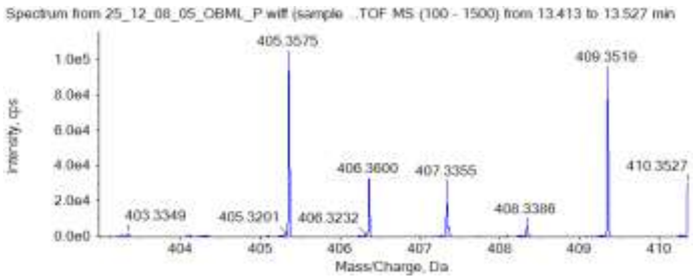

453.3436 / 13.64

(Mass/FragMass/RT/Isotope/Library/Formula/Ion Ratio)

Retention Time: 13.62 minutes
Precursor m/z : 453.3436
Fit (%) 100.0% **RFit (%)** 20.3%

Exp RT: 13.64 minutes
Analyte Name:
453.3436 / 13.64

Collision Energy = 35 ± 15 eV

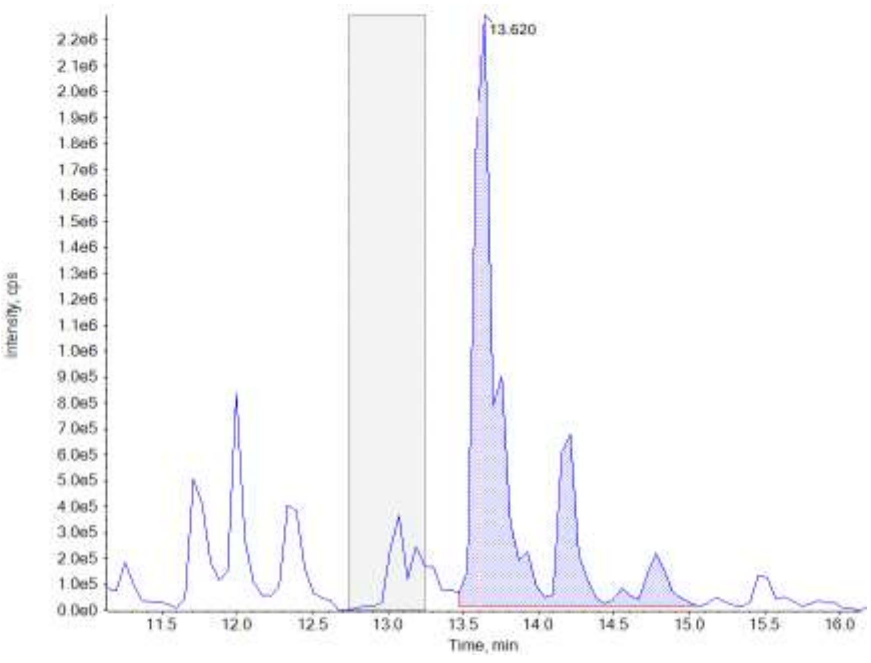

Acquired / Library MSMS

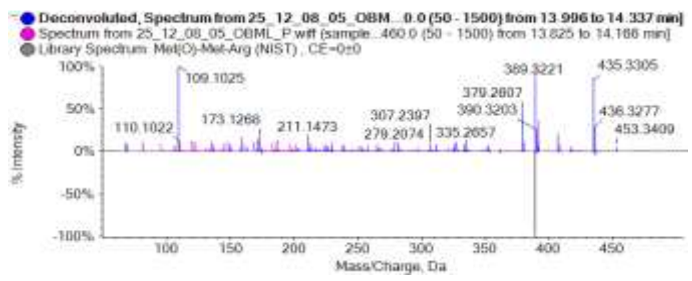

Acquired / Theoretical MS

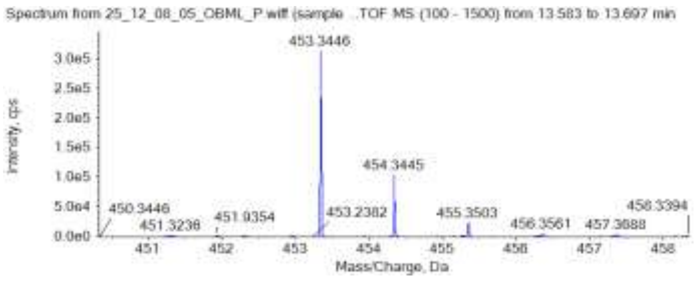

554.4237 / 13.70 [M+H]+

(Mass/FragMass/RT/Isotope/Library/Formula/Ion Ratio)

Retention Time: 13.69 minutes
Precursor m/z : 554.4237
Fit (%) 67.1% **RFit (%)** 81.8%

Exp RT: 13.70 minutes
Analyte Name:
554.4237 / 13.70 [M+H]+

Collision Energy = 35 ± 15 eV

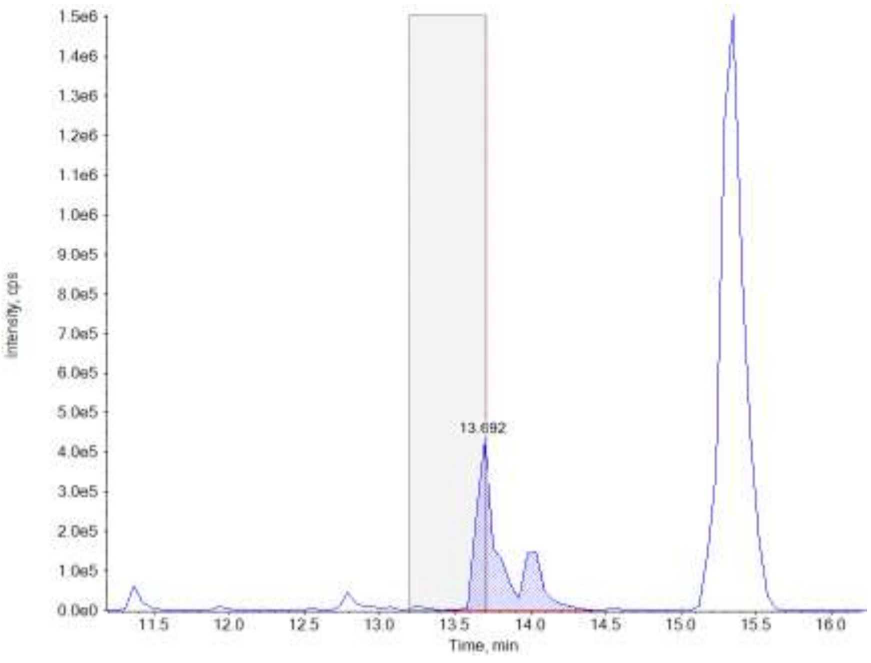

Acquired / Library MSMS

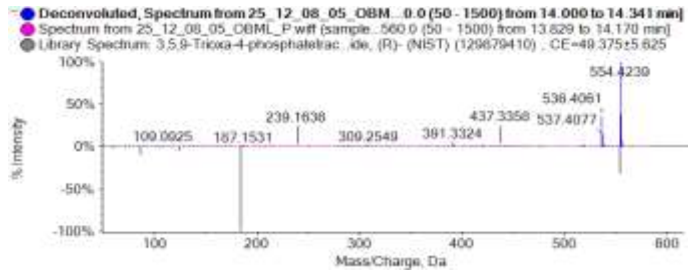

Acquired / Theoretical MS

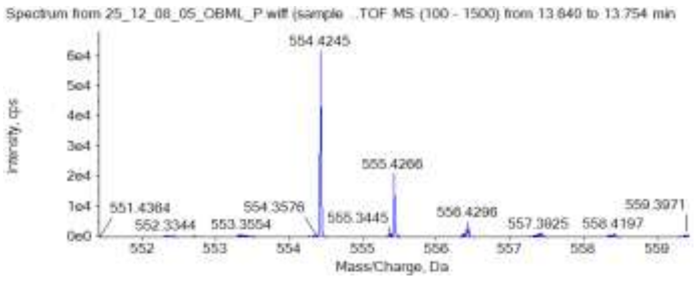

195.1426 / 13.81 (Mass/FragMass/RT/Isotope/Library/Formula/Ion Ratio)

**Retention Time:** 13.81 minutes	**Exp RT:** 13.81 minutes
**Precursor m/z :** 195.1426	**Analyte Name:**
**Fit (%)** 84.7% **RFit (%)** 98.1%	195.1426 / 13.81

Collision Energy = 35 ± 15 eV

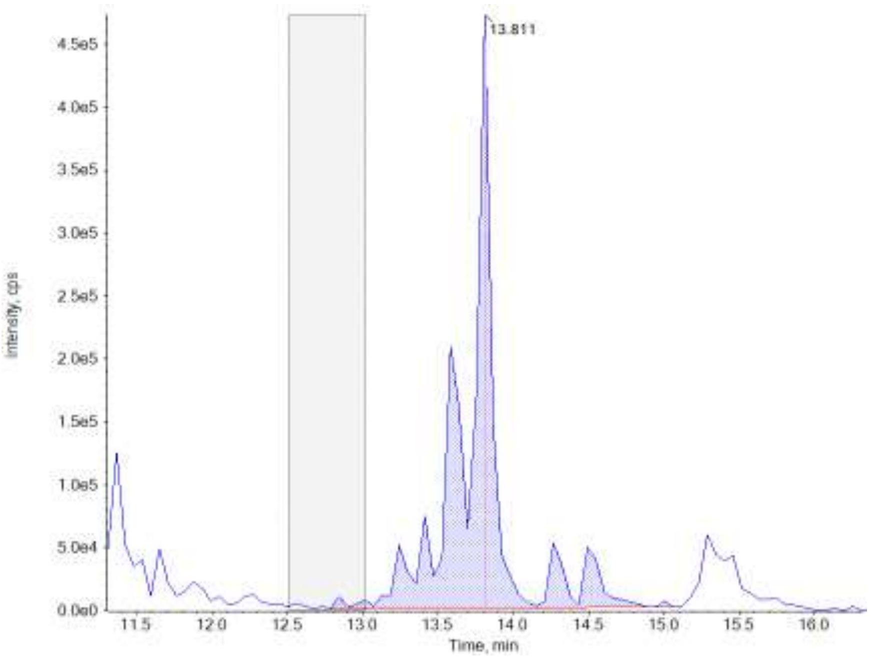

Acquired / Library MSMS

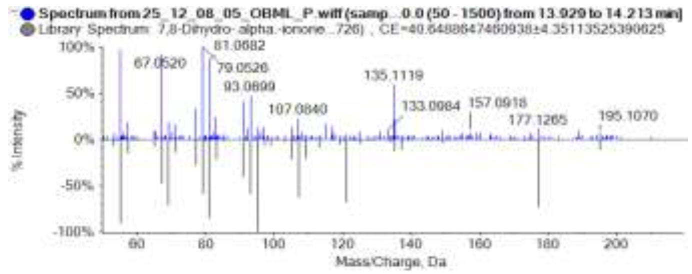

Acquired / Theoretical MS

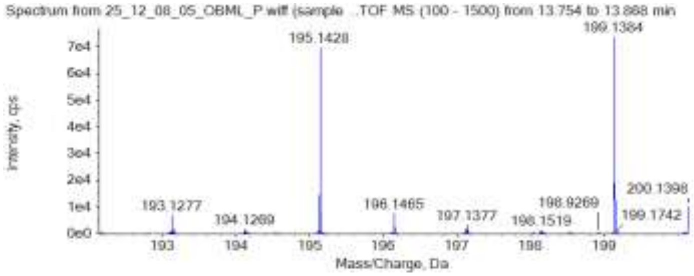

199.1381 / 13.81 (Mass/FragMass/RT/Isotope/Library/Formula/Ion Ratio)

**Retention Time:** 13.81 minutes	**Exp RT:** 13.81 minutes
**Precursor m/z :** 199.1381	**Analyte Name:**
**Fit (%)** 54.1% **RFit (%)** 93.3%	199.1381 / 13.81

Collision Energy = 35 ± 15 eV

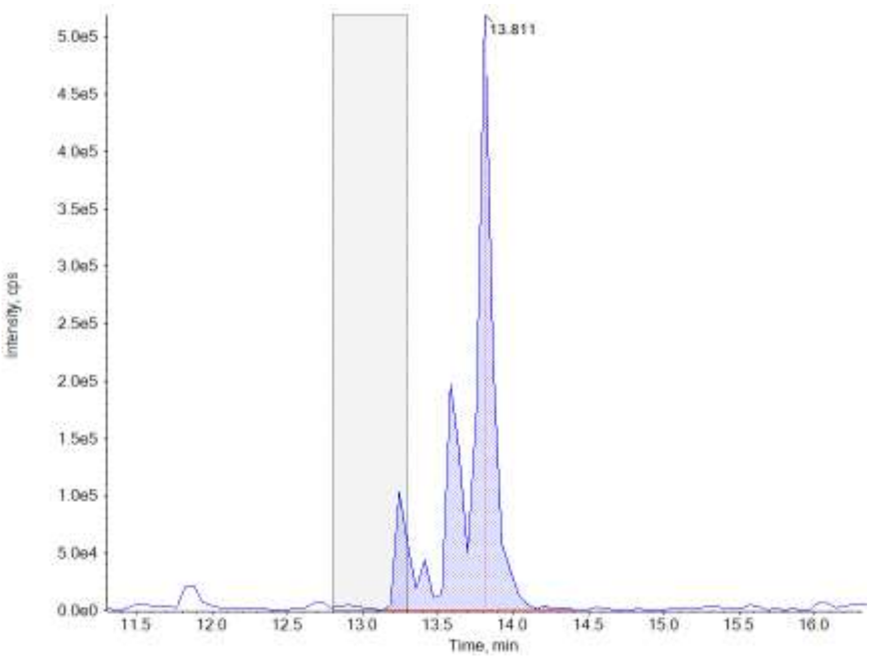

Acquired / Library MSMS

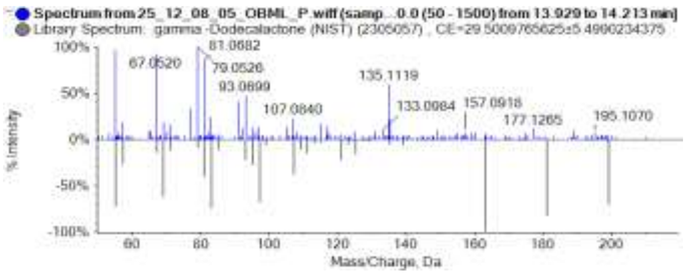

Acquired / Theoretical MS

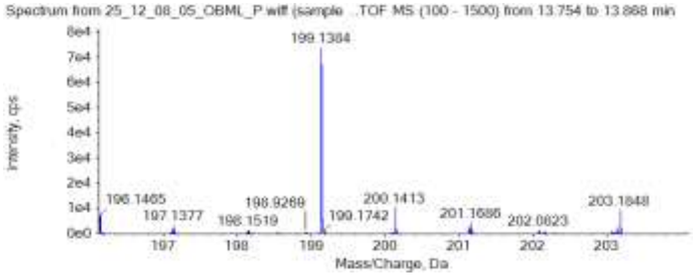

275.2088 / 13.81 (Mass/FragMass/RT/Isotope/Library/Formula/Ion Ratio)

**Retention Time:** 13.60 minutes	**Exp RT:** 13.81 minutes
**Precursor m/z :** 275.2088	**Analyte Name:**
**Fit (%)** 60.5% **RFit (%)** 99.8%	275.2088 / 13.81

Collision Energy = 35 ± 15 eV

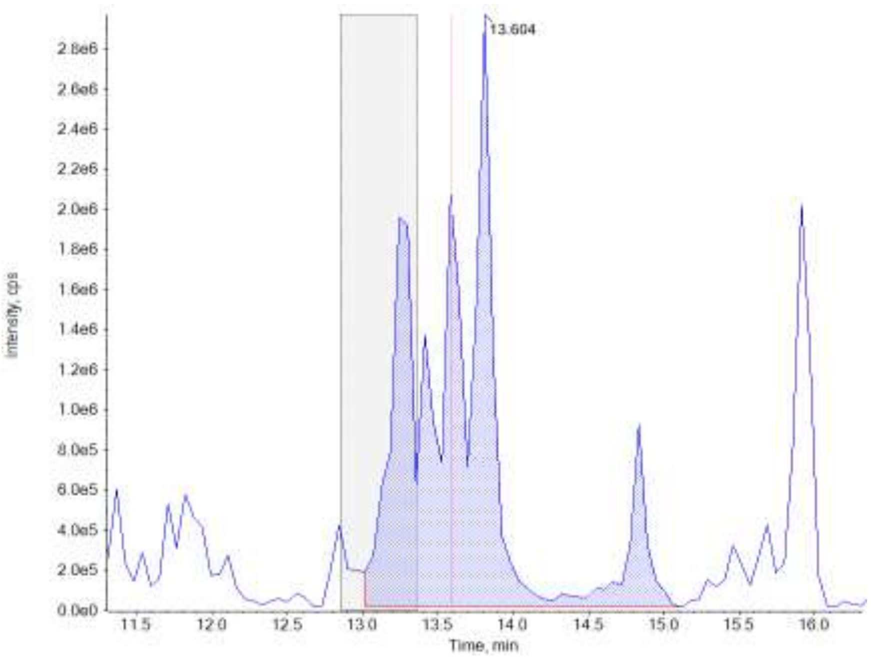

Acquired / Library MSMS

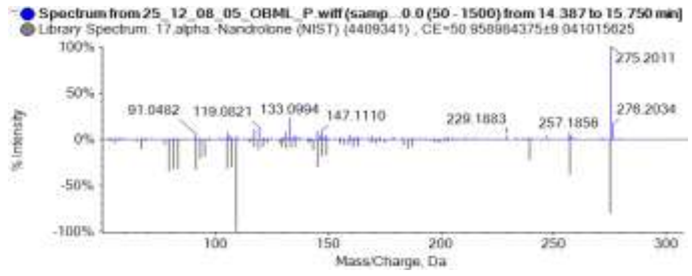

Acquired / Theoretical MS

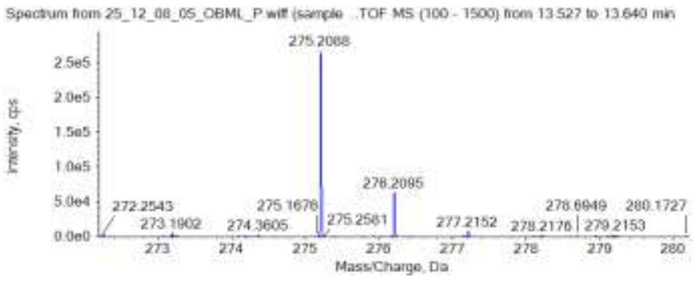

293.2187 / 13.81 (Mass/FragMass/RT/Isotope/Library/Formula/Ion Ratio)

Retention Time: 13.72 minutes
Precursor m/z : 293.2187
Fit (%) 96.3% **RFit (%)** 60.8%

Exp RT: 13.81 minutes
Analyte Name:
293.2187 / 13.81

Collision Energy = 35 ± 15 eV

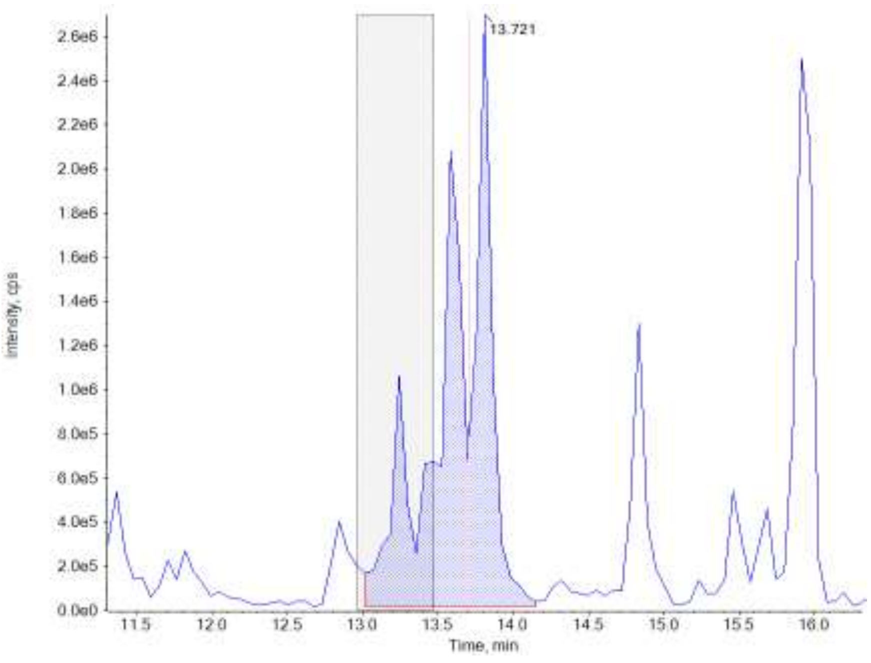

Acquired / Library MSMS

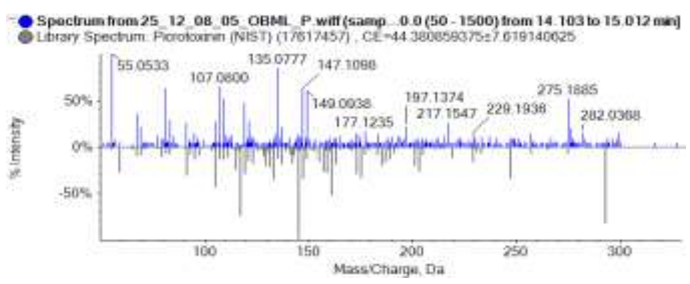

Acquired / Theoretical MS

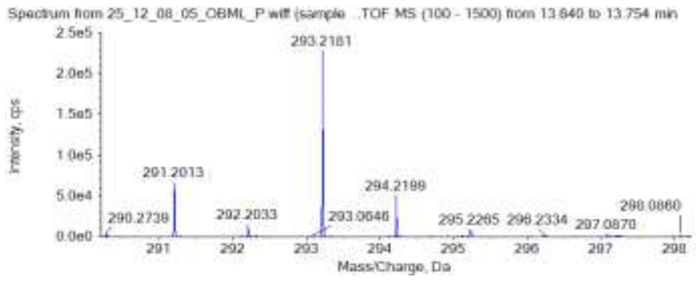

Created with SCIEX OS 3.3.0
Printed:12/9/2025 2:05:30 PM

337.2582 / 13.98 (Mass/FragMass/RT/Isotope/Library/Formula/Ion Ratio)

Retention Time: 14.02 minutes
Precursor m/z : 337.2582
Fit (%) 55.2% **RFit (%)** 88.8%

Exp RT: 13.98 minutes
Analyte Name:
337.2582 / 13.98

Collision Energy = 35 ± 15 eV

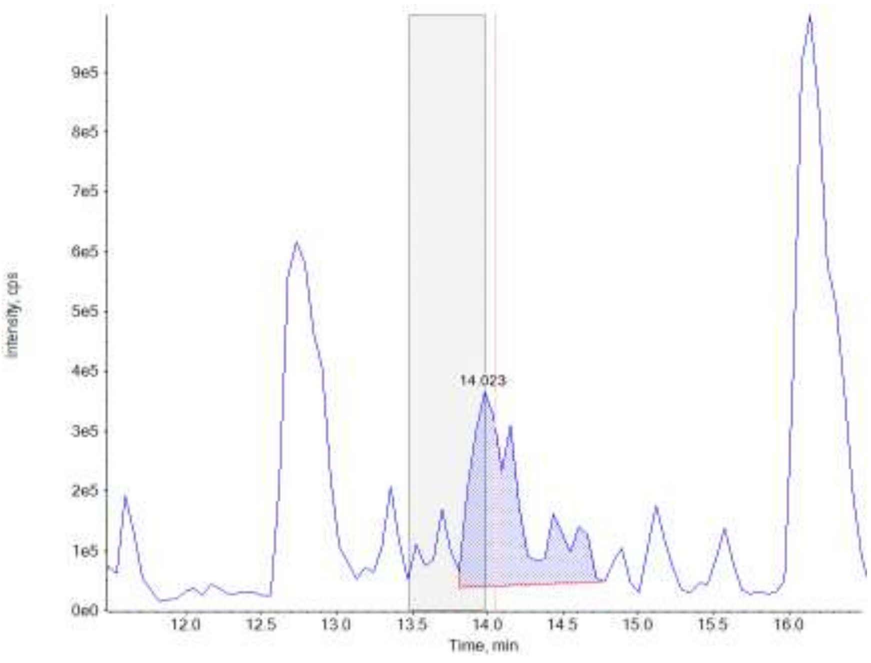

Acquired / Library MSMS

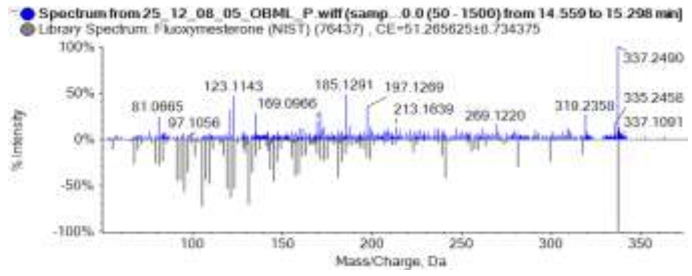

Acquired / Theoretical MS

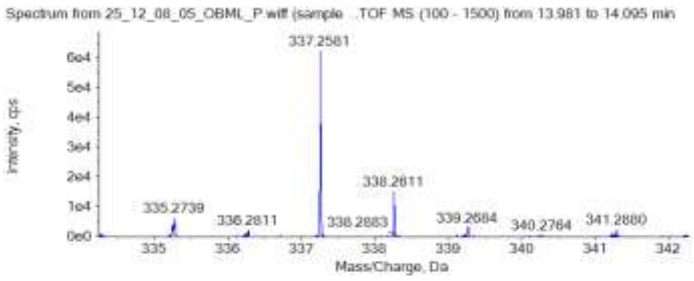

391.3411 / 13.98

(Mass/FragMass/RT/Isotope/Library/Formula/Ion Ratio)

Retention Time: 14.00 minutes **Exp RT:** 13.98 minutes
Precursor m/z : 391.3411 **Analyte Name:**
Fit (%) 100.0% **RFit (%)** 53.2% 391.3411 / 13.98

Collision Energy = 35 ± 15 eV

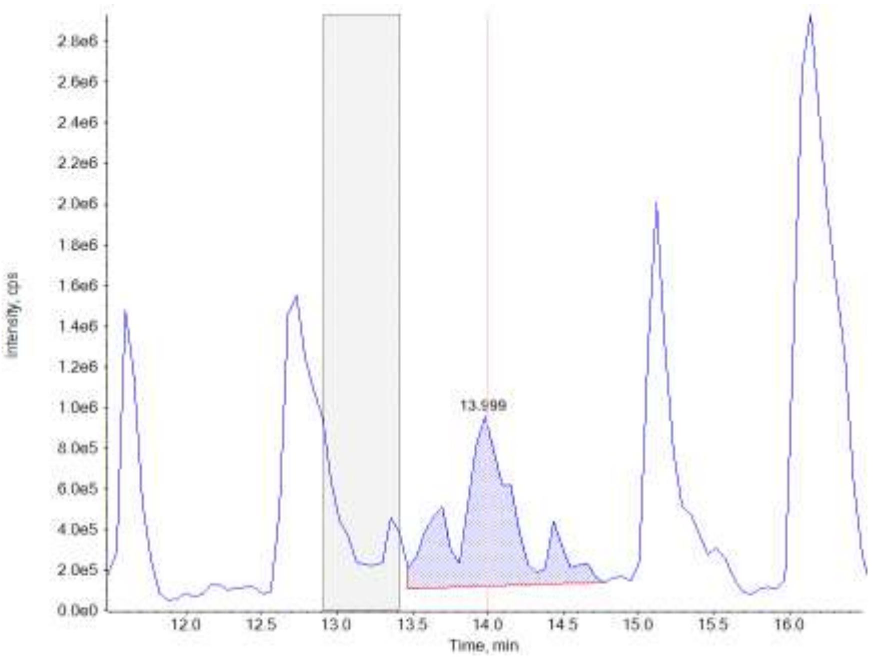

Acquired / Library MSMS

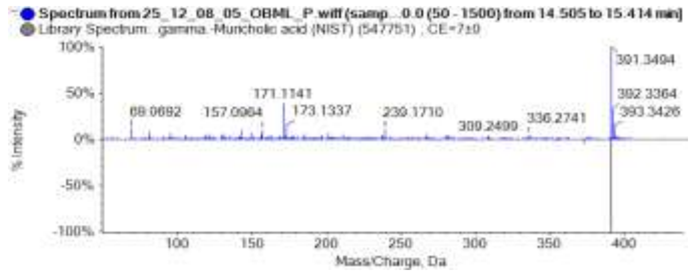

Acquired / Theoretical MS

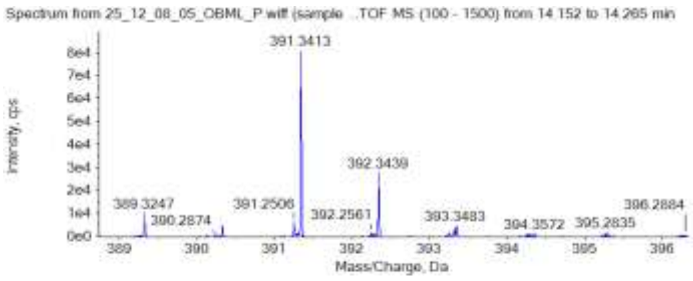

441.3775 / 13.98 [M+CH3OH+H]+ (Mass/FragMass/RT/Isotope/Library/Formula/Ion Ratio)

Retention Time: 13.97 minutes
Precursor m/z : 441.3775
Fit (%) 91.4% **RFit (%)** 85.7%

Exp RT: 13.98 minutes
Analyte Name:
441.3775 / 13.98 [M+CH3OH+H]+

Collision Energy = 35 ± 15 eV

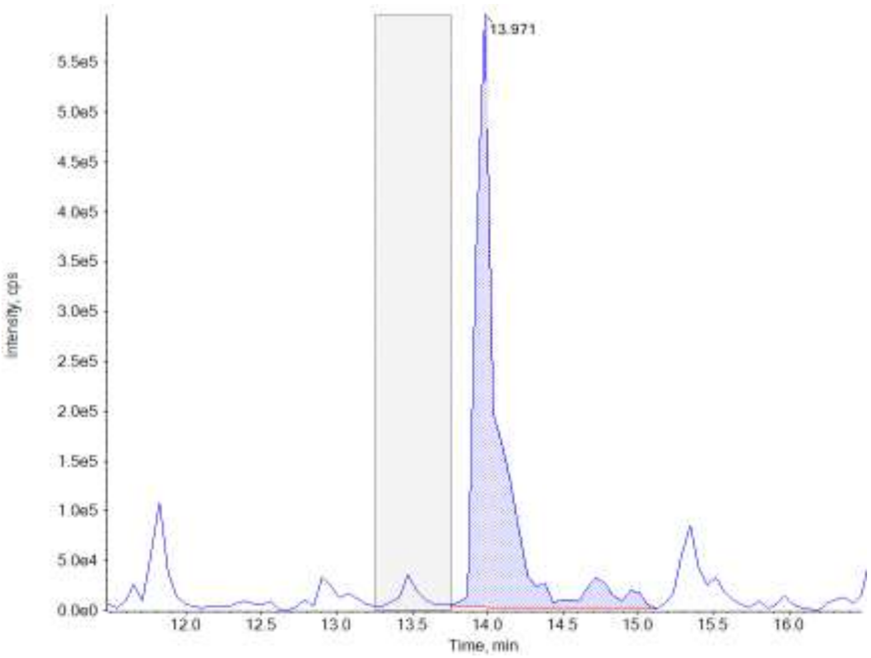

Acquired / Library MSMS

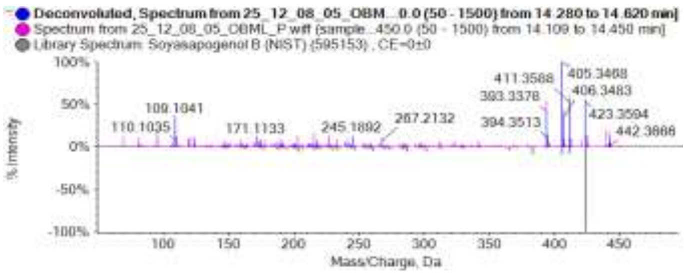

Acquired / Theoretical MS

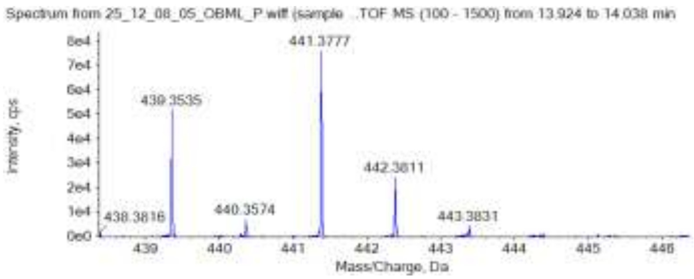

621.4403 / 13.98 (Mass/FragMass/RT/Isotope/Library/Formula/Ion Ratio)

Retention Time: 13.97 minutes
Precursor m/z : 621.4403
Fit (%) 93.2% **RFit (%)** 82.7%

Exp RT: 13.98 minutes
Analyte Name:
621.4403 / 13.98

Collision Energy = 35 ± 15 eV

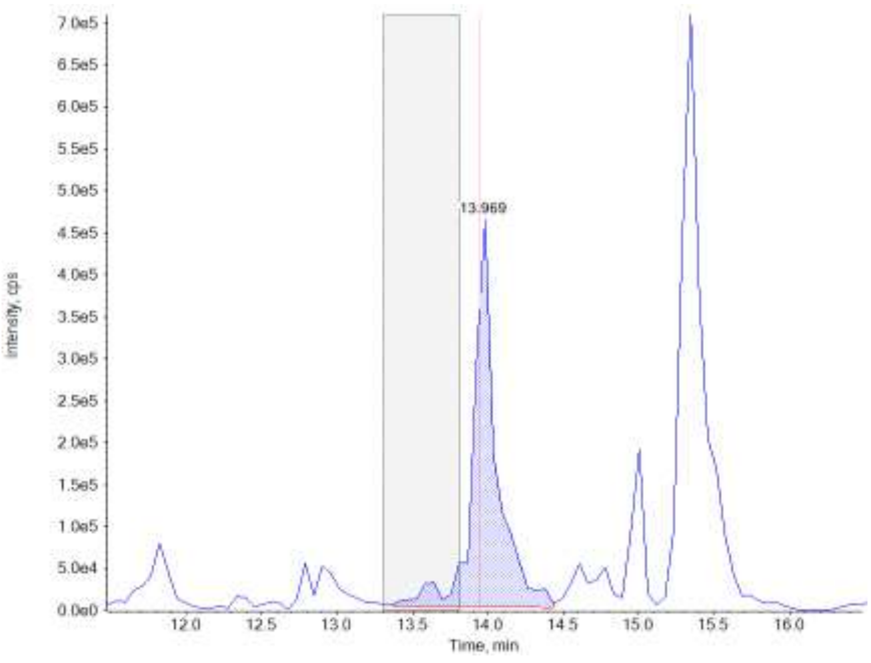

Acquired / Library MSMS

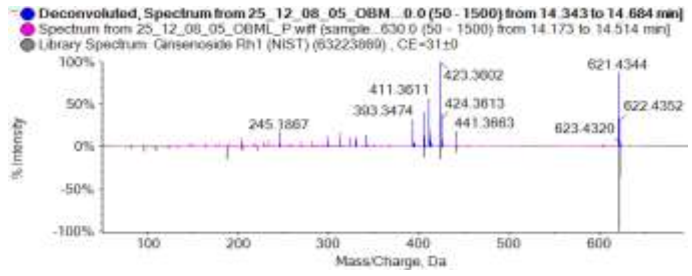

Acquired / Theoretical MS

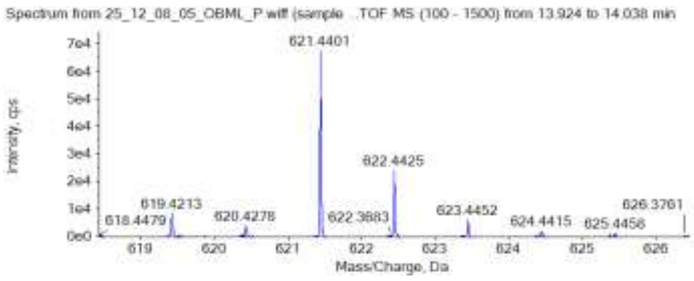

668.4407 / 14.21 (Mass/FragMass/RT/Isotope/Library/Formula/Ion Ratio)

**Retention Time:** 14.19 minutes<br>**Precursor m/z :** 668.4407<br>**Fit (%)** 72.7% **RFit (%)** 94.0%	**Exp RT:** 14.21 minutes<br>**Analyte Name:**<br>668.4407 / 14.21

	**Collision Energy = 35 ± 15 eV**
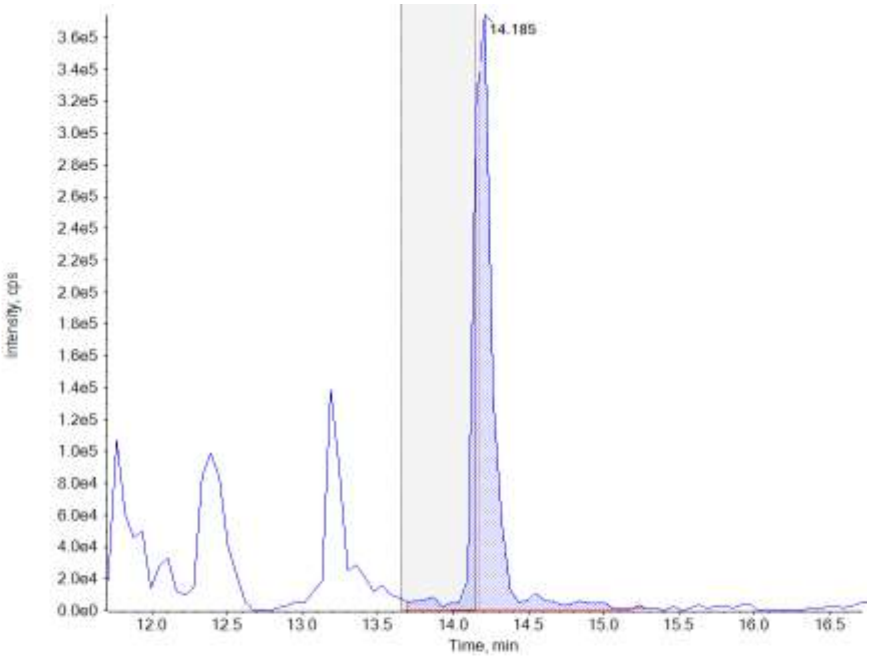	**Acquired / Library MSMS**<br>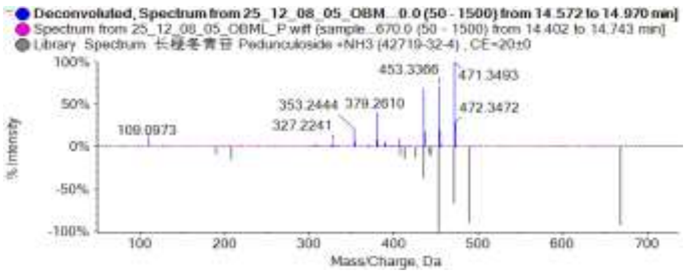
	**Acquired / Theoretical MS**<br>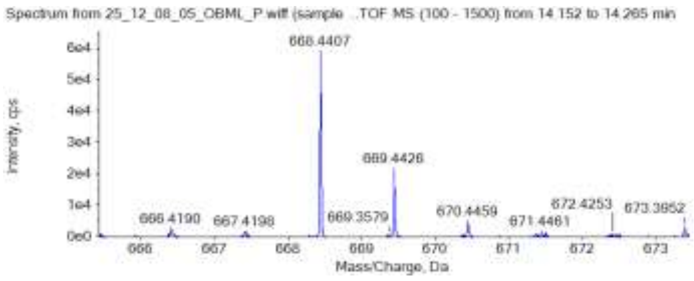

407.3353 / 14.32 [M+H]+ (Mass/FragMass/RT/Isotope/Library/Formula/Ion Ratio)

Retention Time: 14.66 minutes
Precursor m/z : 407.3353
Fit (%) 21.0% **RFit (%)** 99.9%

Exp RT: 14.32 minutes
Analyte Name:
407.3353 / 14.32 [M+H]+

Collision Energy = 35 ± 15 eV

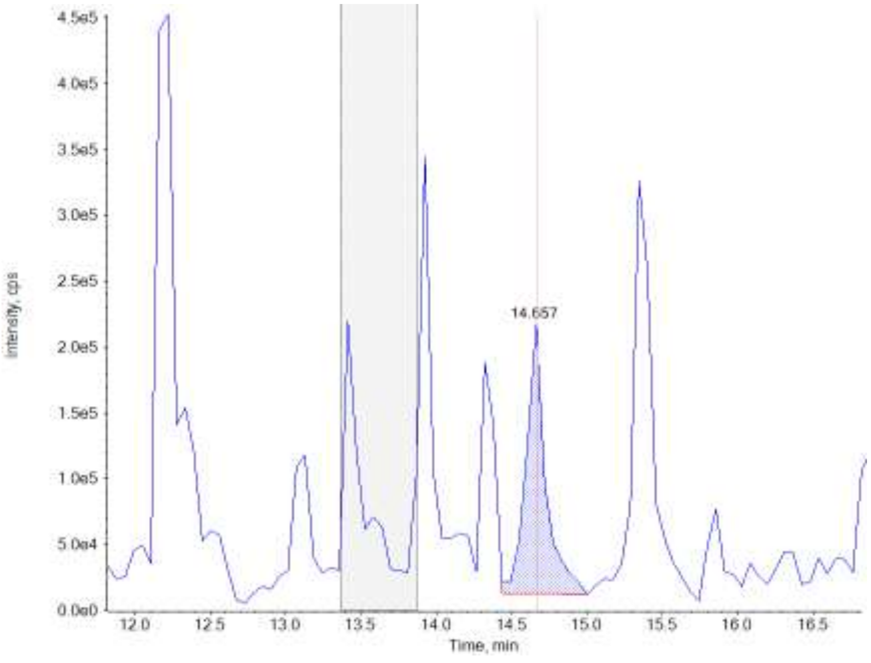

Acquired / Library MSMS

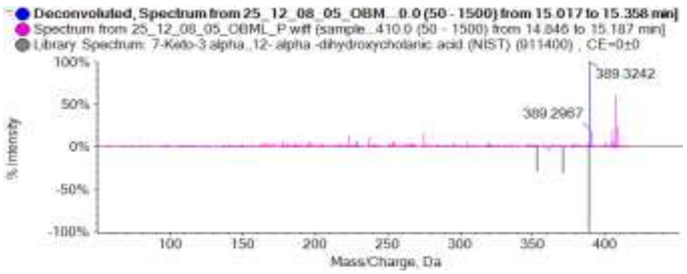

Acquired / Theoretical MS

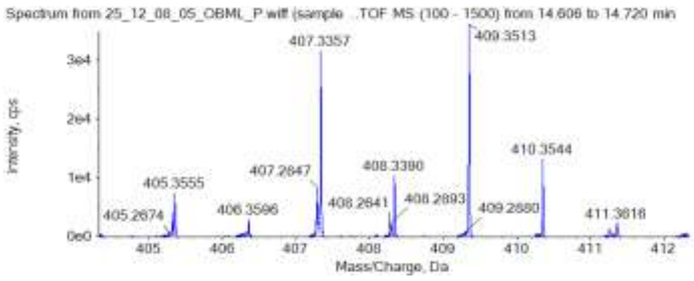

Created with SCIEX OS 3.3.0
Printed:12/9/2025 2:05:30 PM

353.2772 / 14.38

(Mass/FragMass/RT/Isotope/Library/Formula/Ion Ratio)

Retention Time: 14.38 minutes
Precursor m/z : 353.2772
Fit (%) 78.8% **RFit (%)** 99.5%

Exp RT: 14.38 minutes
Analyte Name:
353.2772 / 14.38

Collision Energy = 35 ± 15 eV

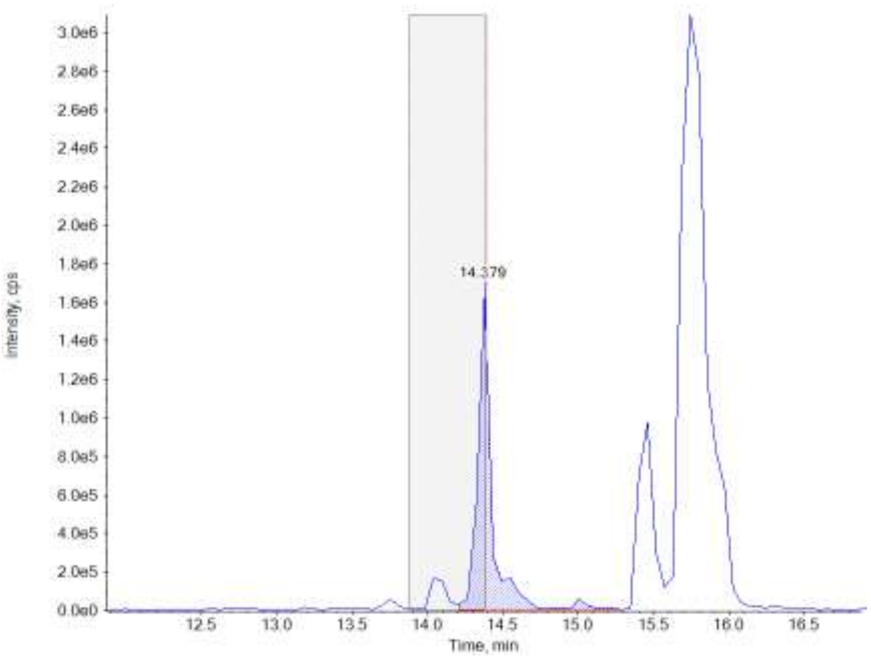

Acquired / Library MSMS

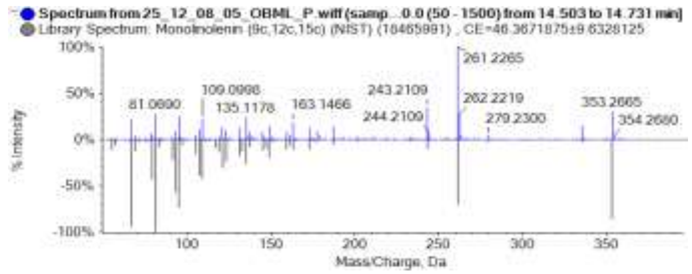

Acquired / Theoretical MS

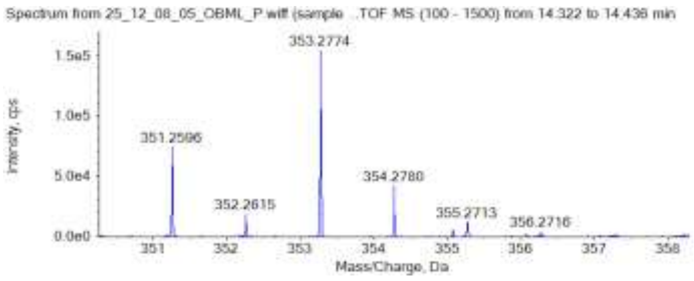

Created with SCIEX OS 3.3.0
Printed:12/9/2025 2:05:30 PM

421.3510 / 14.38 (Mass/FragMass/RT/Isotope/Library/Formula/Ion Ratio)

Retention Time: 14.38 minutes
Precursor m/z : 421.3510
Fit (%) 90.2% **RFit (%)** 92.6%

Exp RT: 14.38 minutes
Analyte Name:
421.3510 / 14.38

Collision Energy = 35 ± 15 eV

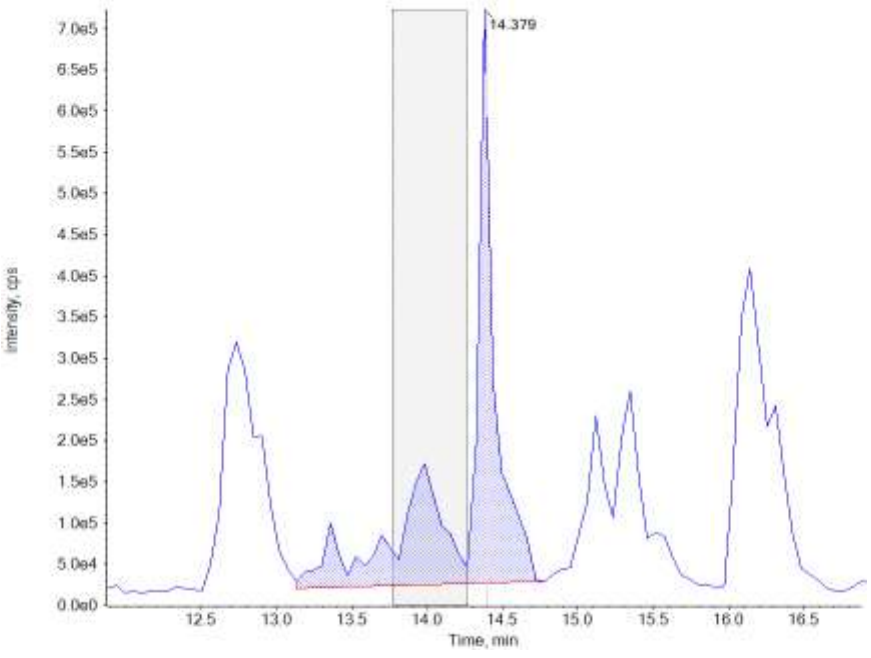

Acquired / Library MSMS

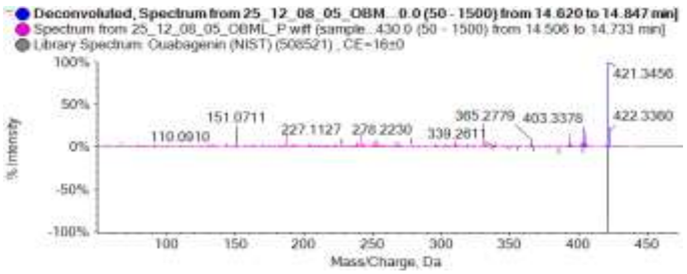

Acquired / Theoretical MS

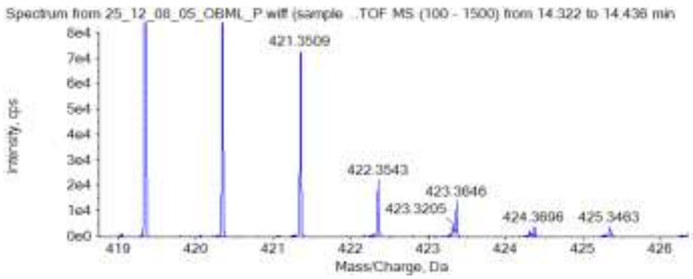

291.2028 / 14.44 (Mass/FragMass/RT/Isotope/Library/Formula/Ion Ratio)

Retention Time: 14.44 minutes
Precursor m/z : 291.2028
Fit (%) 75.4% **RFit (%)** 82.1%

Exp RT: 14.44 minutes
Analyte Name:
291.2028 / 14.44

Collision Energy = 35 ± 15 eV

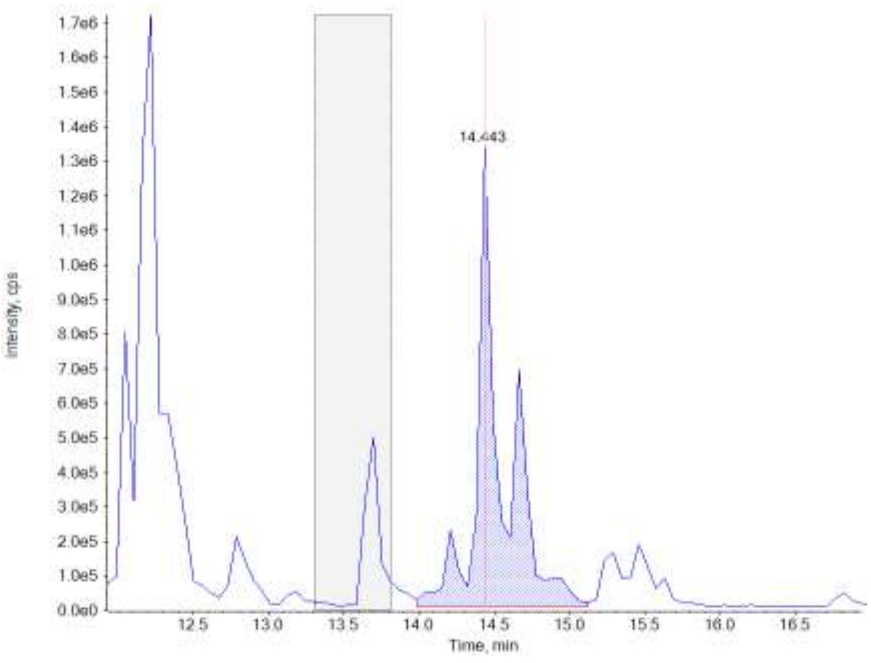

Acquired / Library MSMS

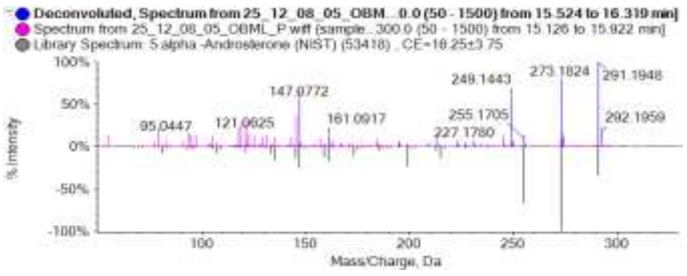

Acquired / Theoretical MS

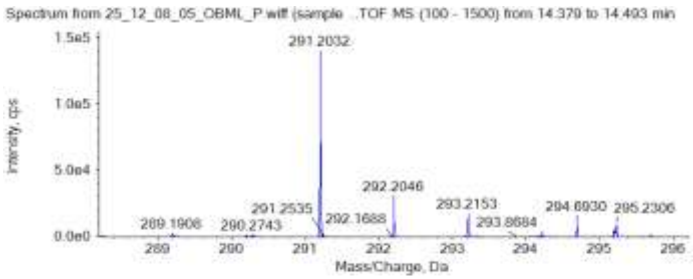

293.2189 / 14.83

(Mass/FragMass/RT/Isotope/Library/Formula/Ion Ratio)

Retention Time: 14.83 minutes
Precursor m/z : 293.2189
Fit (%) 83.5% **RFit (%)** 87.1%

Exp RT: 14.83 minutes
Analyte Name:
293.2189 / 14.83

Collision Energy = 35 ± 15 eV

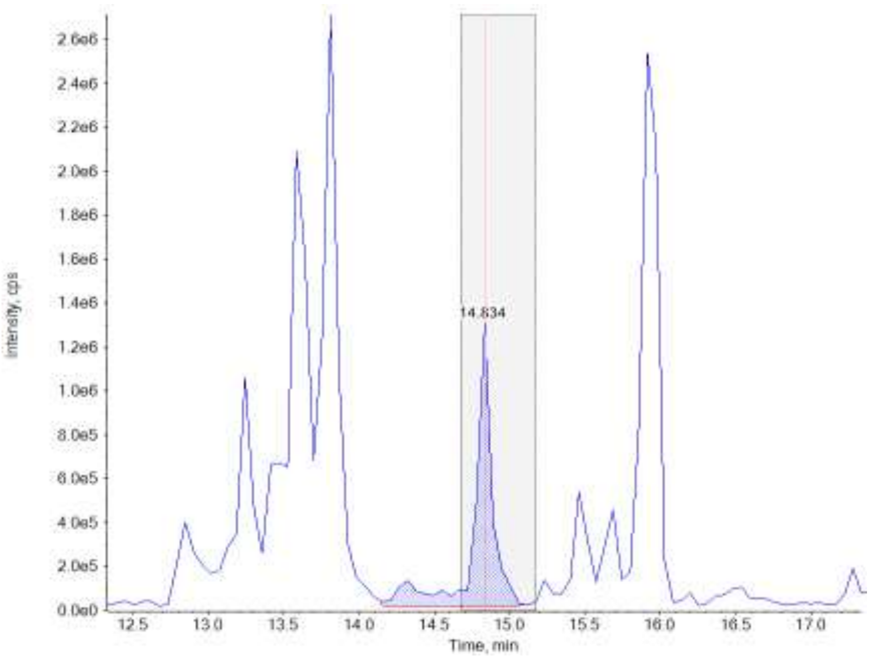

Acquired / Library MSMS

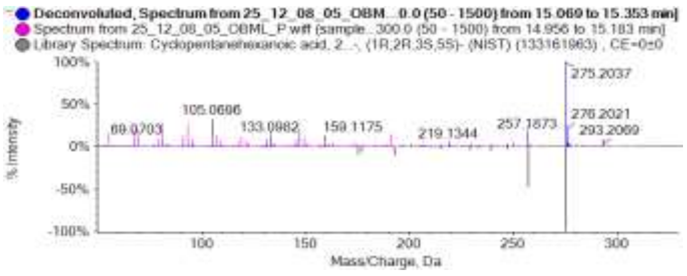

Acquired / Theoretical MS

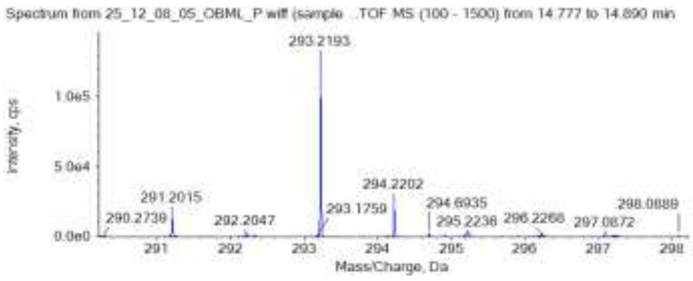

236.1122 / 15.06 (Mass/FragMass/RT/Isotope/Library/Formula/Ion Ratio)

**Retention Time:** 15.06 minutes	**Exp RT:** 15.06 minutes
**Precursor m/z :** 236.1122	**Analyte Name:**
**Fit (%)** 90.6% **RFit (%)** 56.1%	236.1122 / 15.06

Collision Energy = 35 ± 15 eV

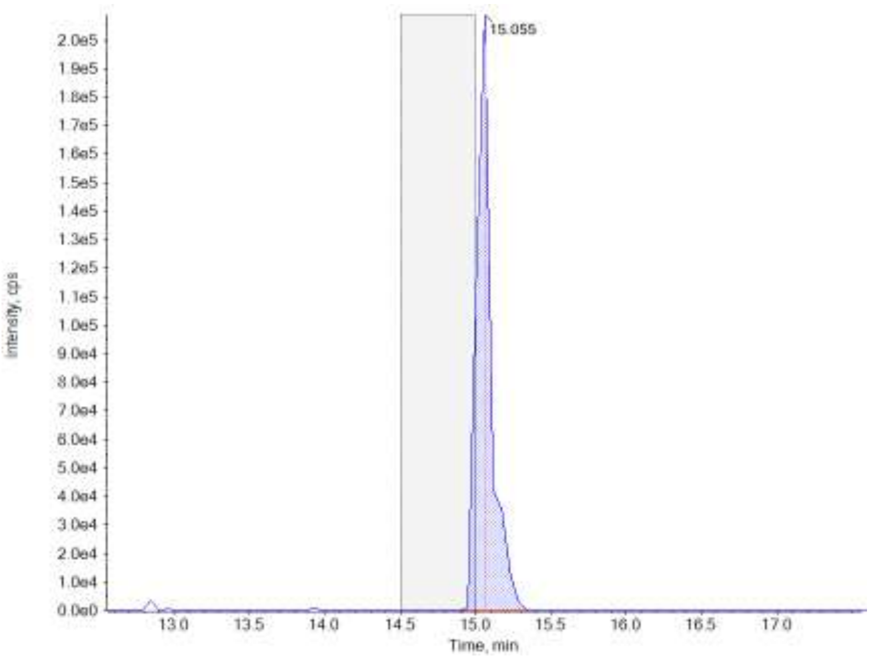

Acquired / Library MSMS

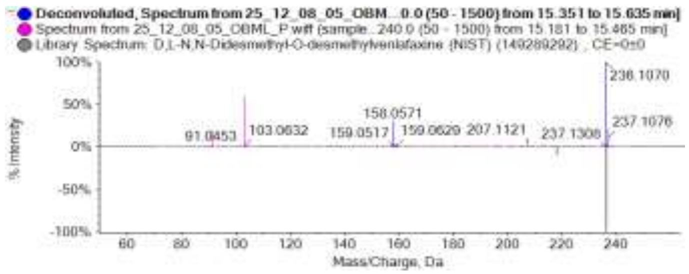

Acquired / Theoretical MS

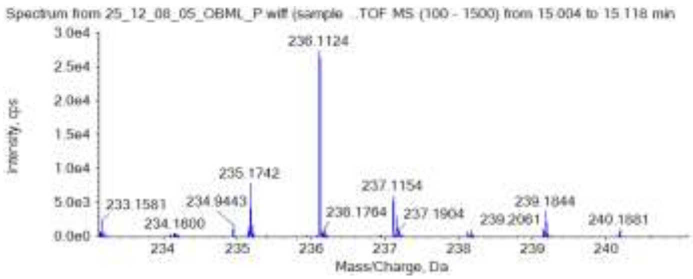

309.2623 / 15.12 (Mass/FragMass/RT/Isotope/Library/Formula/Ion Ratio)

Retention Time: 15.13 minutes **Exp RT:** 15.12 minutes
Precursor m/z : 309.2623 **Analyte Name:**
Fit (%) 100.0% **RFit (%)** 28.8% 309.2623 / 15.12

Collision Energy = 35 ± 15 eV

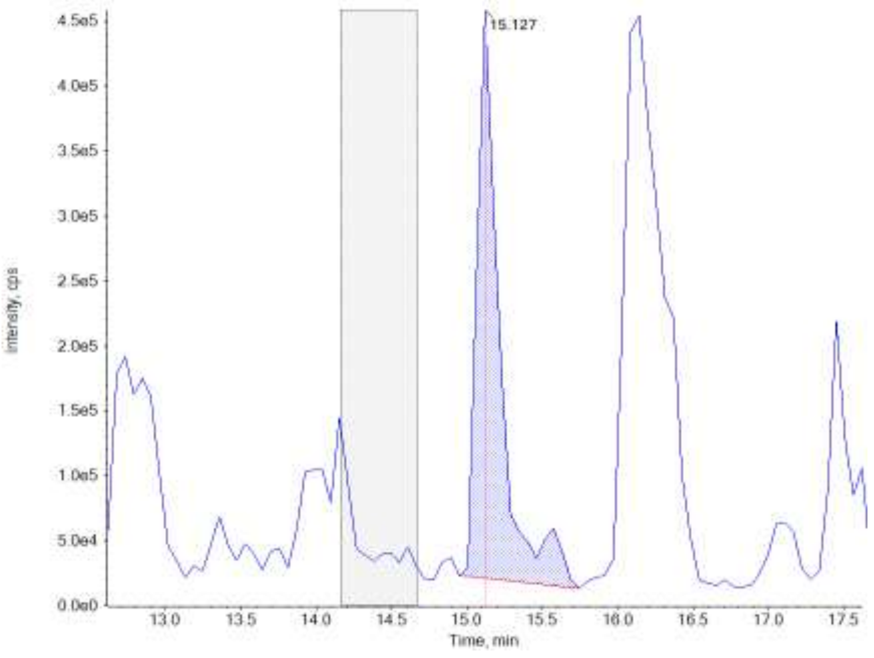

Acquired / Library MSMS

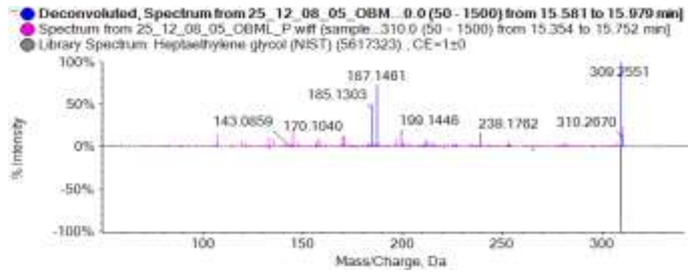

Acquired / Theoretical MS

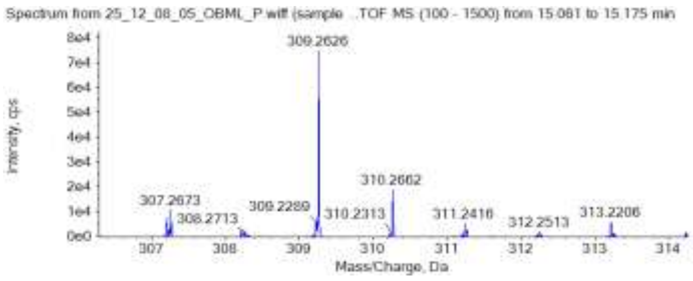

391.3427 / 15.12 [M+H]+

(Mass/FragMass/RT/Isotope/Library/Formula/Ion Ratio)

Retention Time: 15.12 minutes
Precursor m/z : 391.3427
Fit (%) 100.0% **RFit (%)** 49.0%

Exp RT: 15.12 minutes
Analyte Name:
391.3427 / 15.12 [M+H]+

Collision Energy = 35 ± 15 eV

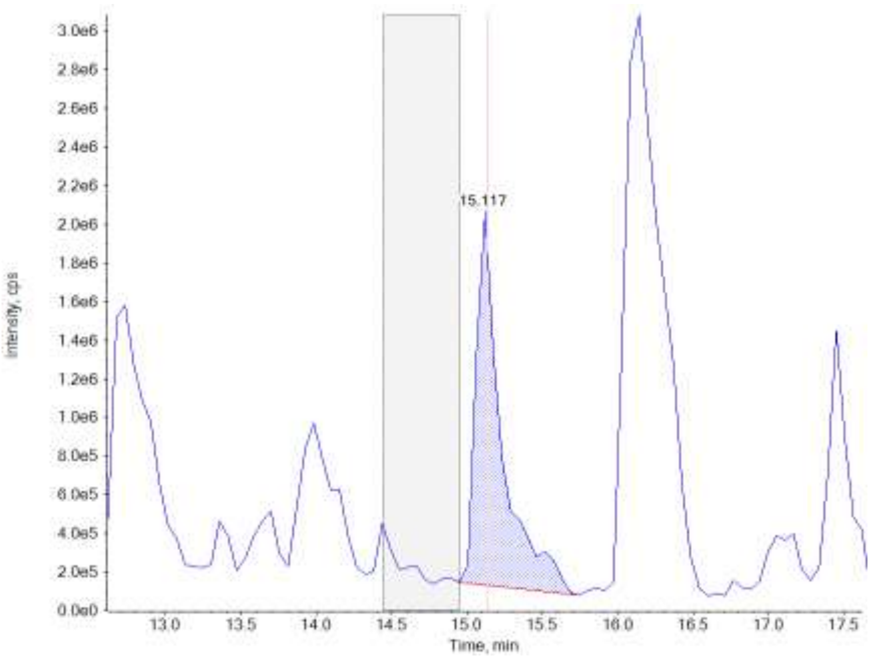

Acquired / Library MSMS

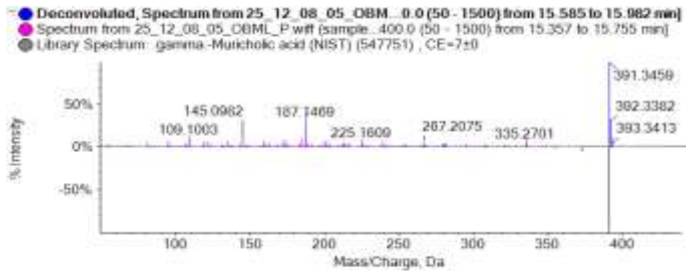

Acquired / Theoretical MS

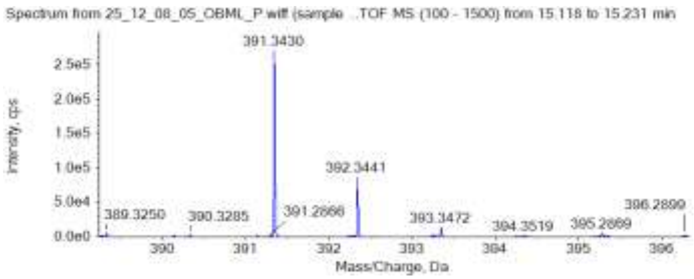

423.3673 / 14.72 [M+CH3OH+H]+ (Mass/FragMass/RT/Isotope/Library/Formula/Ion Ratio)

Retention Time: 14.72 minutes
Precursor m/z : 423.3673
Fit (%) 100.0% **RFit (%)** 58.8%

Exp RT: 14.72 minutes
Analyte Name:
423.3673 / 14.72 [M+CH3OH+H]+

Collision Energy = 35 ± 15 eV

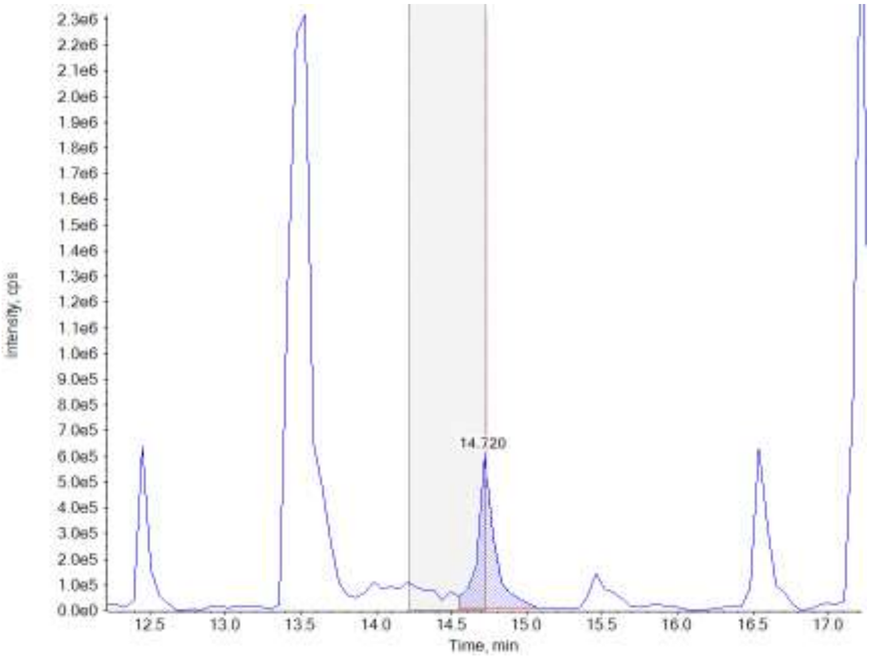

Acquired / Library MSMS

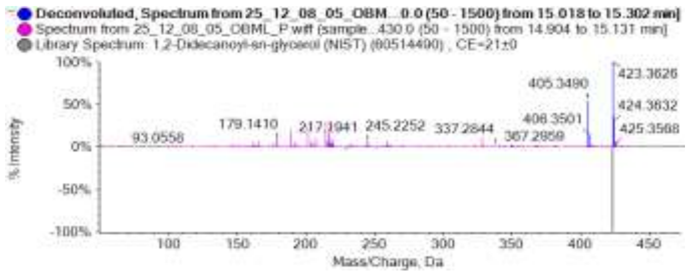

Acquired / Theoretical MS

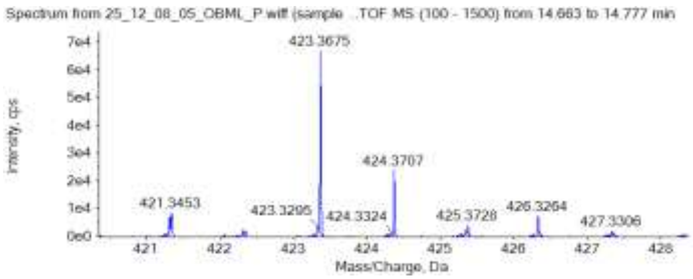

Created with SCIEX OS 3.3.0
Printed:12/9/2025 2:05:30 PM

419.3409 / 15.12 (Mass/FragMass/RT/Isotope/Library/Formula/Ion Ratio)

Retention Time: 15.13 minutes
Precursor m/z : 419.3409
Fit (%) 52.4% **RFit (%)** 86.8%

Exp RT: 15.12 minutes
Analyte Name:
419.3409 / 15.12

Collision Energy = 35 ± 15 eV

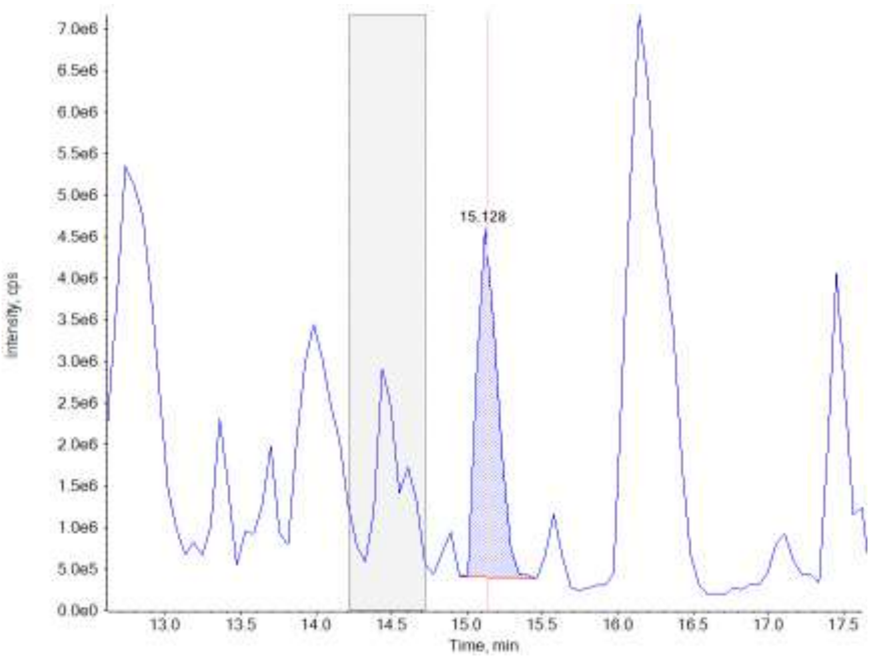

Acquired / Library MSMS

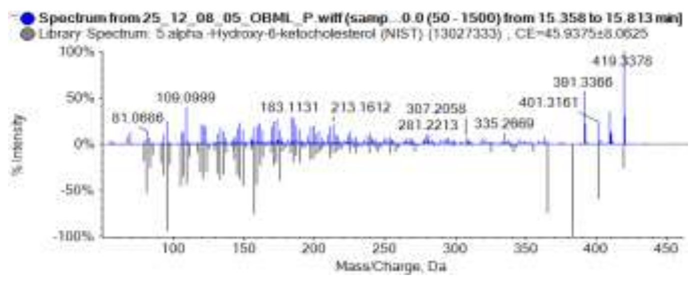

Acquired / Theoretical MS

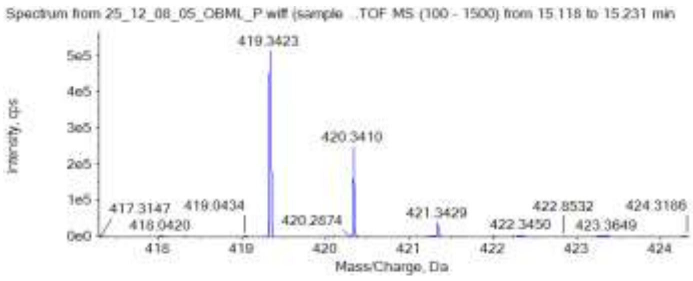

455.3593 / 15.12 (Mass/FragMass/RT/Isotope/Library/Formula/Ion Ratio)

Retention Time: 15.13 minutes
Precursor m/z : 455.3593
Fit (%) 91.8% **RFit (%)** 81.5%

Exp RT: 15.12 minutes
Analyte Name:
455.3593 / 15.12

Collision Energy = 35 ± 15 eV

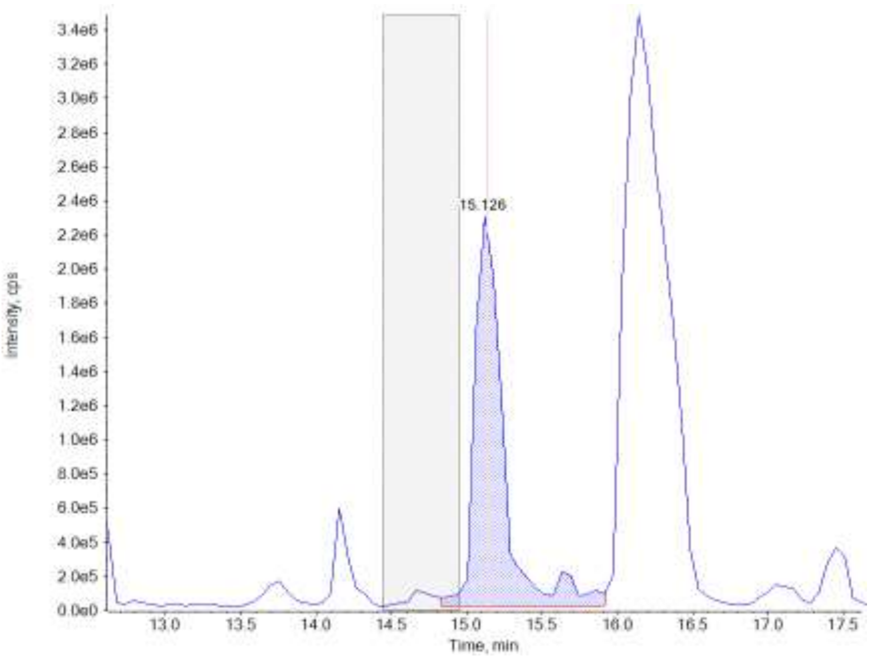

Acquired / Library MSMS

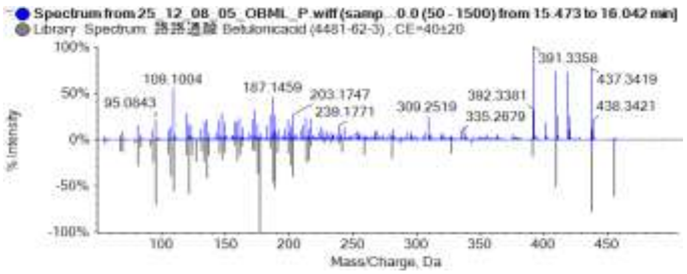

Acquired / Theoretical MS

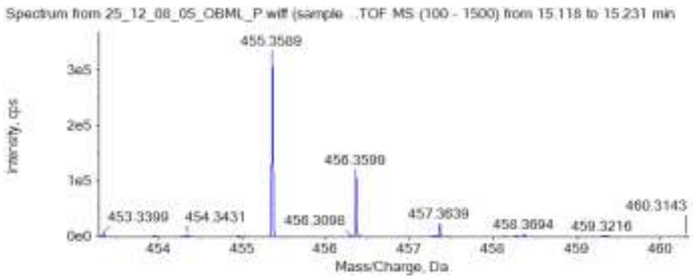

279.2285 / 15.29

(Mass/FragMass/RT/Isotope/Library/Formula/Ion Ratio)

Retention Time: 15.33 minutes
Precursor m/z : 279.2285
Fit (%) 97.4% **RFit (%)** 86.0%

Exp RT: 15.29 minutes
Analyte Name:
279.2285 / 15.29

Collision Energy = 35 ± 15 eV

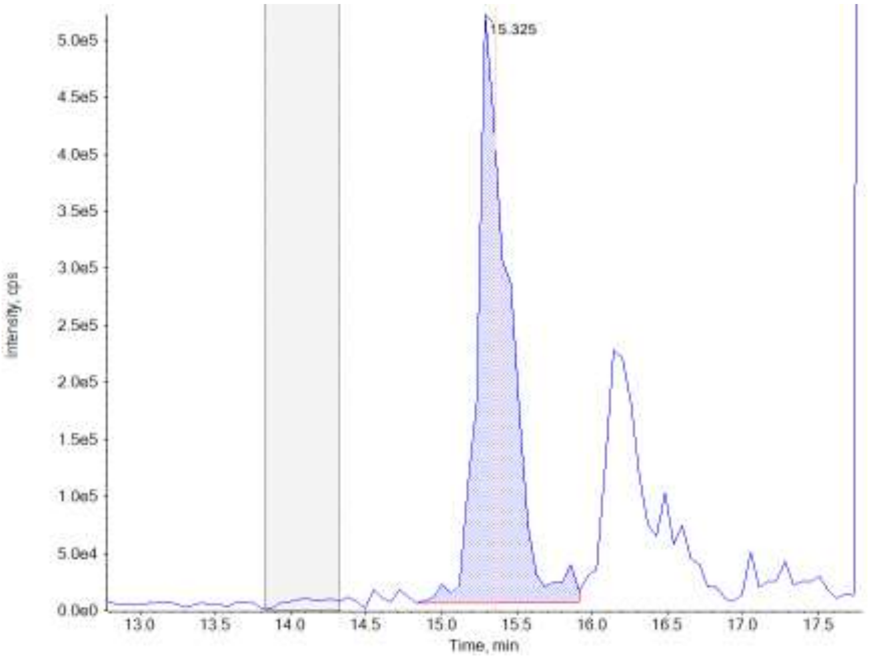

Acquired / Library MSMS

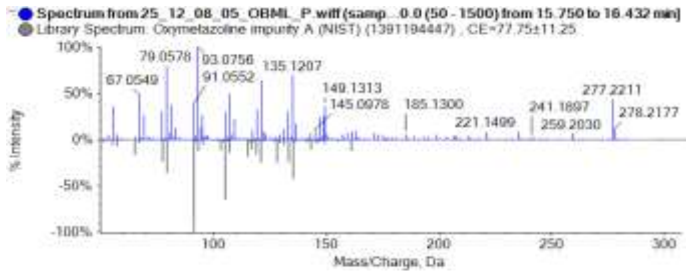

Acquired / Theoretical MS

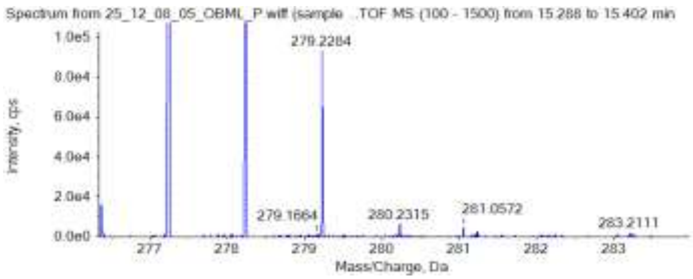

439.3605 / 15.35 (Mass/FragMass/RT/Isotope/Library/Formula/Ion Ratio)

**Retention Time:** 15.33 minutes	**Exp RT:** 15.35 minutes
**Precursor m/z :** 439.3605	**Analyte Name:**
**Fit (%)** 100.0% **RFit (%)** 100.0%	439.3605 / 15.35

Collision Energy = 35 ± 15 eV

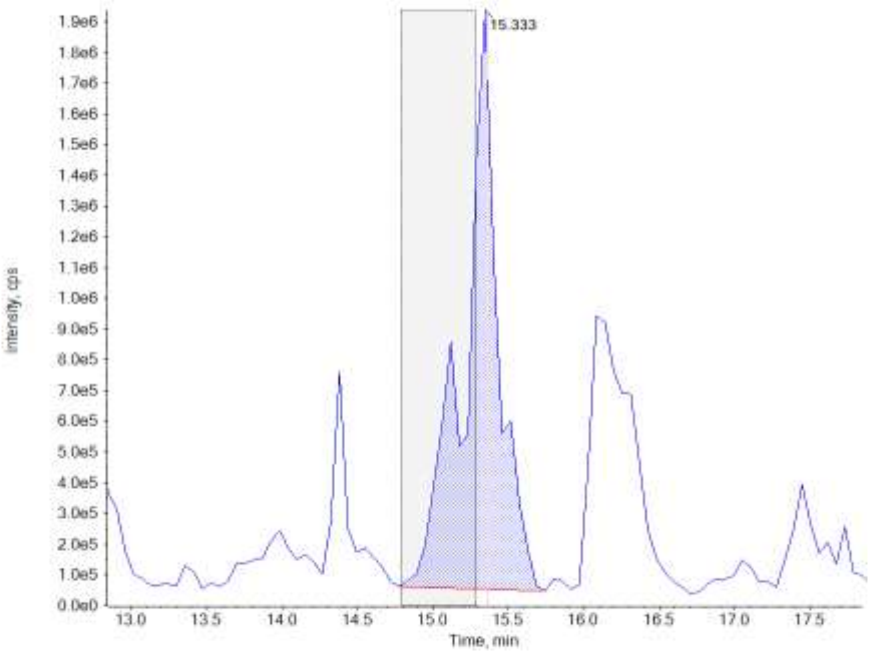

Acquired / Library MSMS

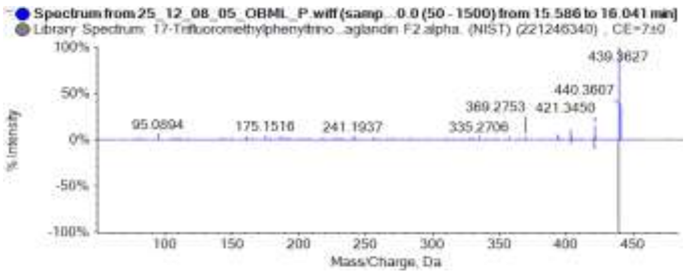

Acquired / Theoretical MS

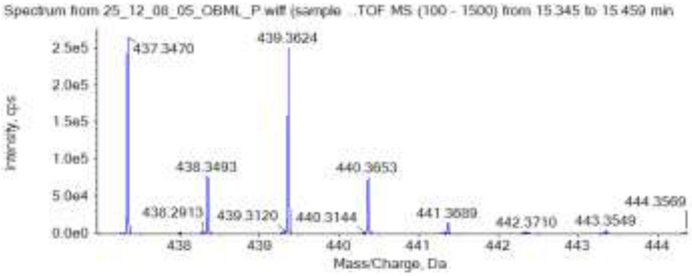

277.2312 / 15.40 (Mass/FragMass/RT/Isotope/Library/Formula/Ion Ratio)

Retention Time: 15.37 minutes **Exp RT:** 15.40 minutes
Precursor m/z : 277.2312 **Analyte Name:**
Fit (%) 99.3% **RFit (%)** 97.4% 277.2312 / 15.40

Collision Energy = 35 ± 15 eV

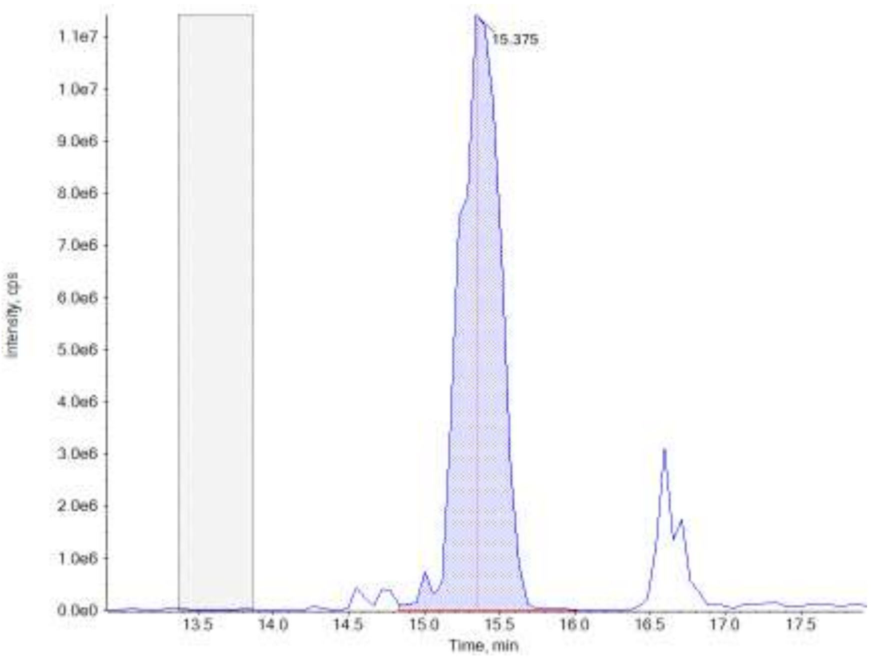

Acquired / Library MSMS

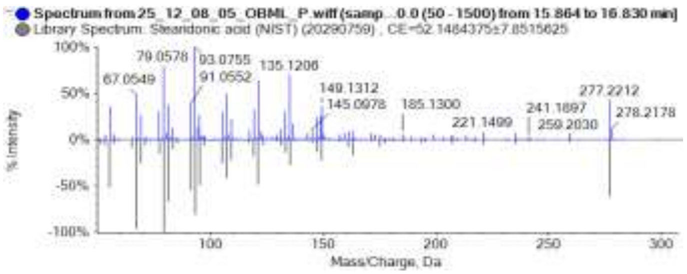

Acquired / Theoretical MS

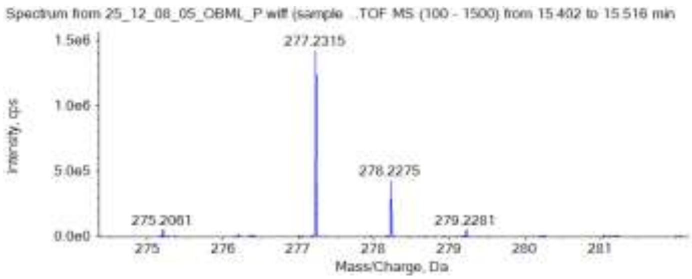

219.1802 / 15.69 (Mass/FragMass/RT/Isotope/Library/Formula/Ion Ratio)

Retention Time: 15.69 minutes
Precursor m/z : 219.1802
Fit (%) 98.5% **RFit (%)** 100.0%

Exp RT: 15.69 minutes
Analyte Name:
219.1802 / 15.69

Collision Energy = 35 ± 15 eV

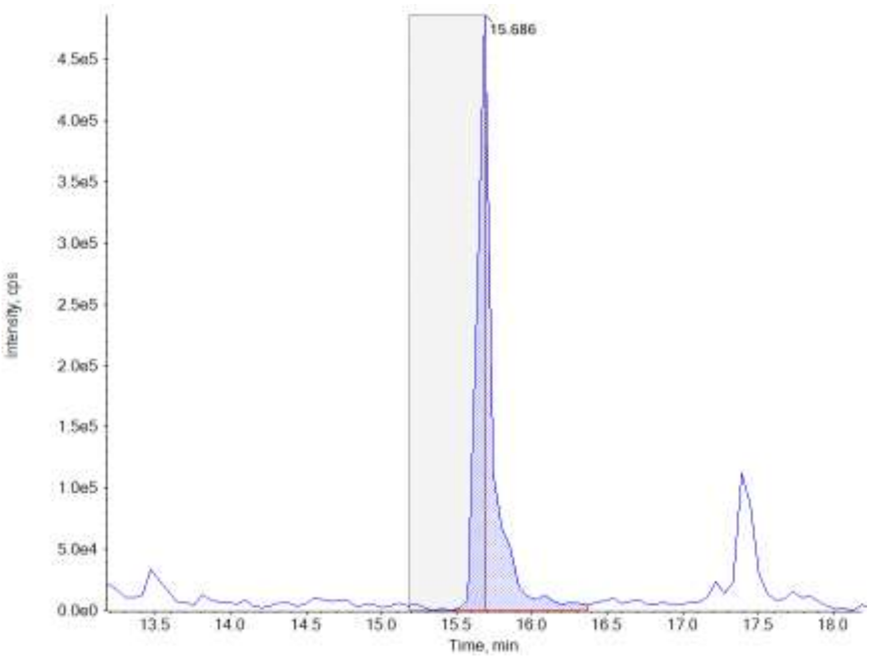

Acquired / Library MSMS

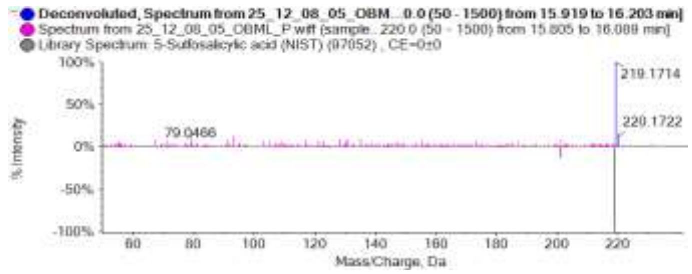

Acquired / Theoretical MS

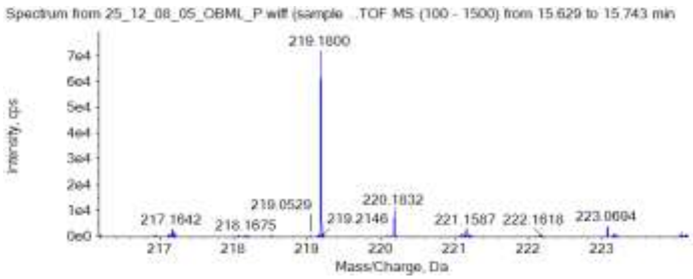

Created with SCIEX OS 3.3.0
Printed:12/9/2025 2:05:30 PM

261.2277 / 15.74 (Mass/FragMass/RT/Isotope/Library/Formula/Ion Ratio)

Retention Time: 15.74 minutes
Precursor m/z : 261.2277
Fit (%) 70.6% **RFit (%)** 91.7%

Exp RT: 15.74 minutes
Analyte Name:
261.2277 / 15.74

Collision Energy = 35 ± 15 eV

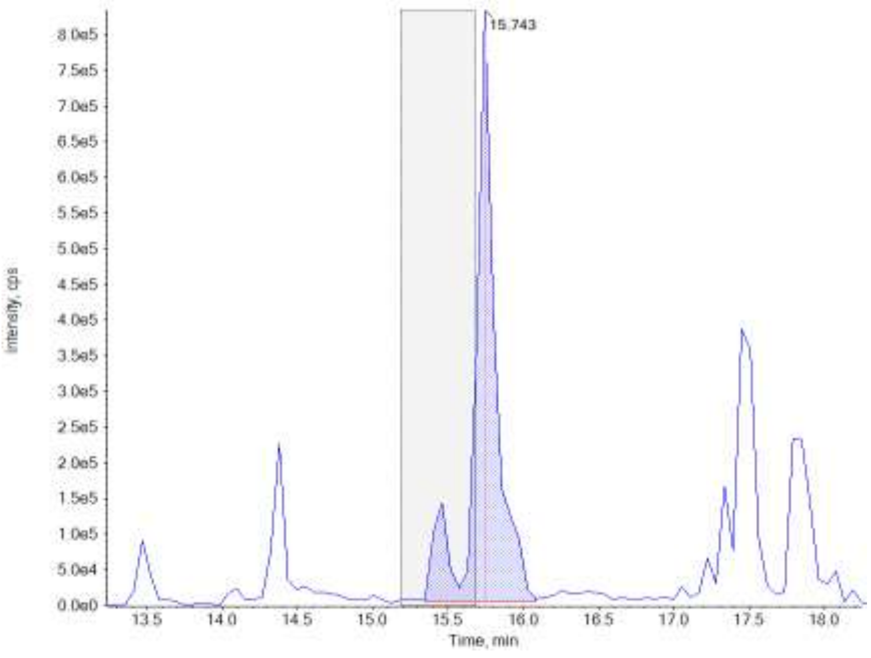

Acquired / Library MSMS

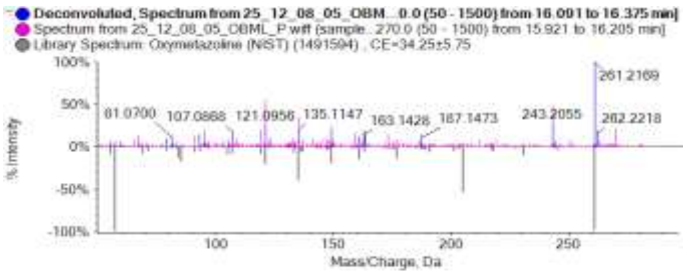

Acquired / Theoretical MS

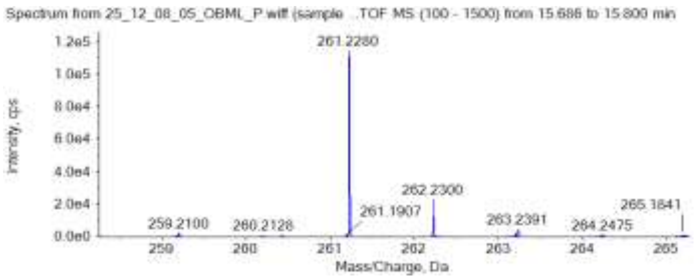

335.2635 / 15.74 (Mass/FragMass/RT/Isotope/Library/Formula/Ion Ratio)

**Retention Time:** 15.74 minutes	**Exp RT:** 15.74 minutes
**Precursor m/z :** 335.2635	**Analyte Name:**
**Fit (%)** 100.0% **RFit (%)** 74.7%	335.2635 / 15.74

Collision Energy = 35 ± 15 eV

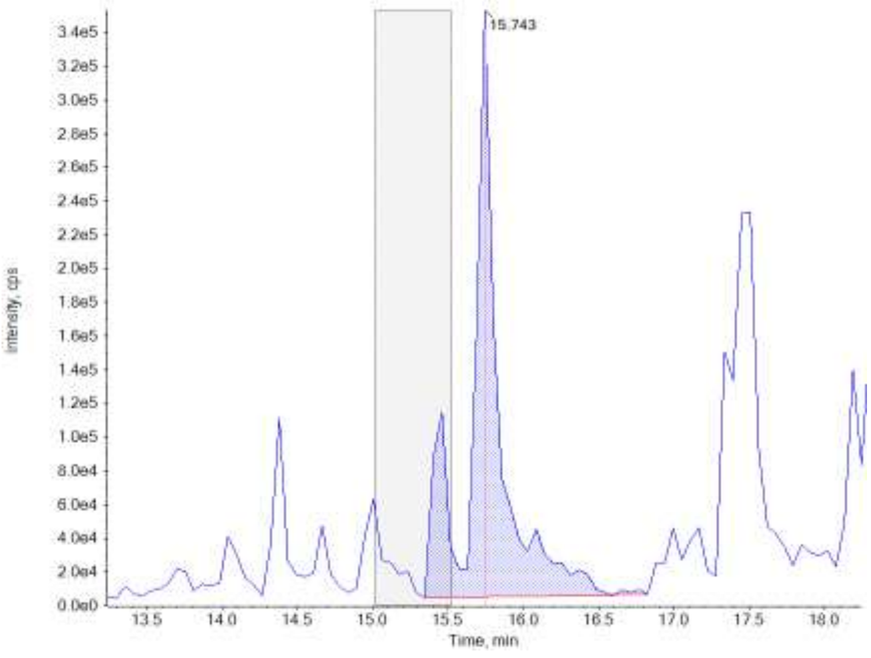

Acquired / Library MSMS

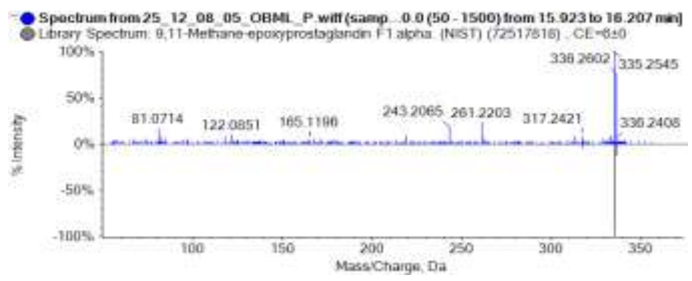

Acquired / Theoretical MS

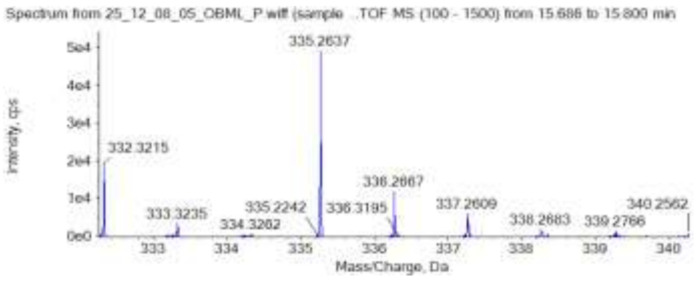

SCIEX

353.2820 / 15.74 (Mass/FragMass/RT/Isotope/Library/Formula/Ion Ratio)

**Retention Time:** 15.75 minutes	**Exp RT:** 15.74 minutes
**Precursor m/z :** 353.2820	**Analyte Name:**
**Fit (%)** 97.1% **RFit (%)** 99.8%	353.2820 / 15.74

Collision Energy = 35 ± 15 eV

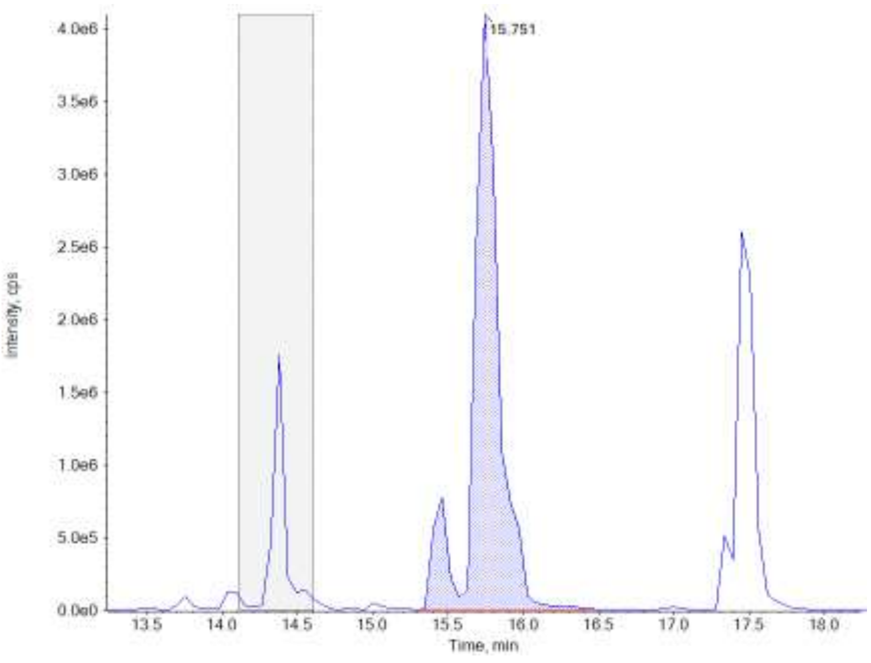

Acquired / Library MSMS

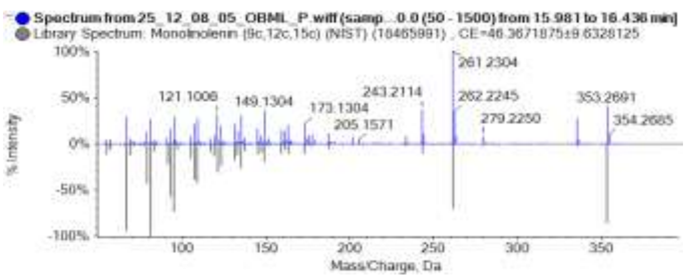

Acquired / Theoretical MS

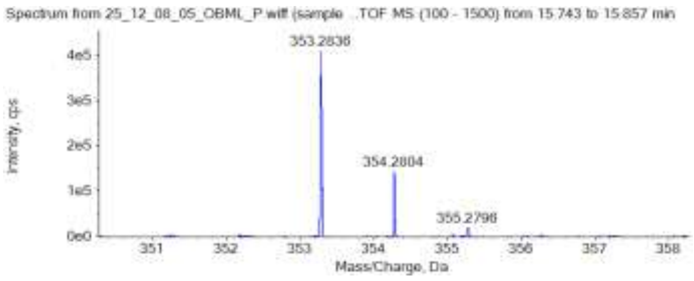

497.3167 / 15.74

(Mass/FragMass/RT/Isotope/Library/Formula/Ion Ratio)

Retention Time: 15.67 minutes
Precursor m/z : 497.3167
Fit (%) 78.9% **RFit (%)** 96.1%

Exp RT: 15.74 minutes
Analyte Name:
497.3167 / 15.74

Collision Energy = 35 ± 15 eV

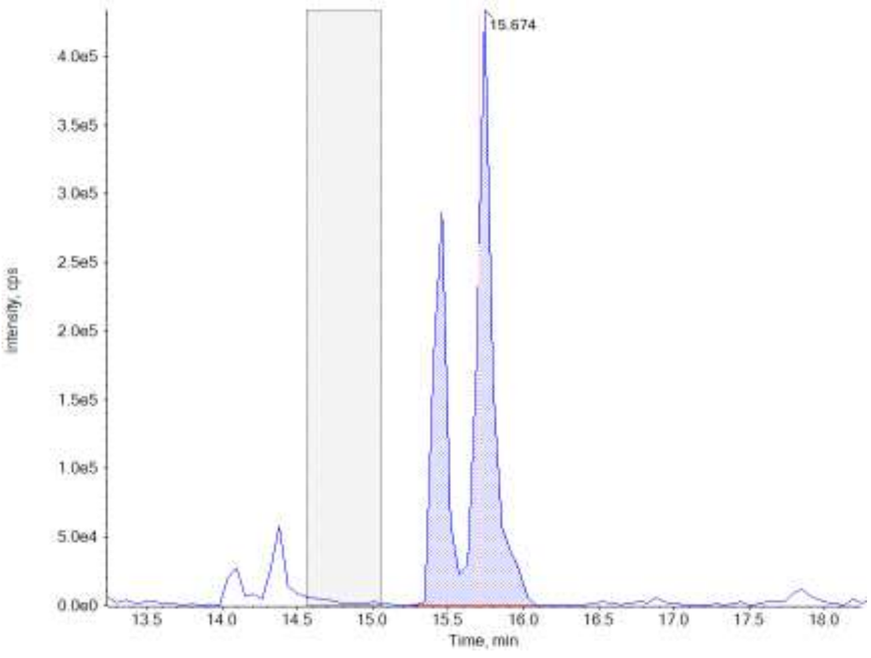

Acquired / Library MSMS

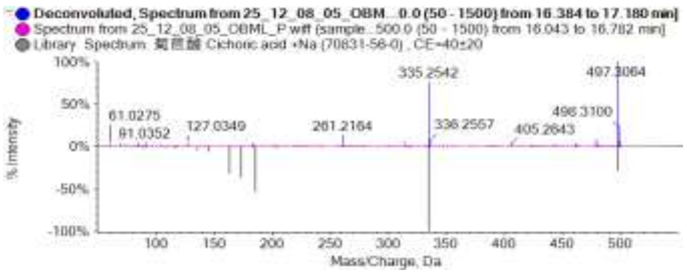

Acquired / Theoretical MS

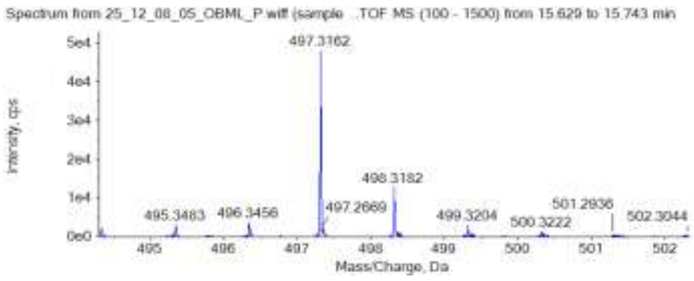

275.2092 / 15.91 (Mass/FragMass/RT/Isotope/Library/Formula/Ion Ratio)

**Retention Time:** 15.92 minutes	**Exp RT:** 15.91 minutes
**Precursor m/z :** 275.2092	**Analyte Name:**
**Fit (%)** 93.6% **RFit (%)** 73.7%	275.2092 / 15.91

Collision Energy = 35 ± 15 eV

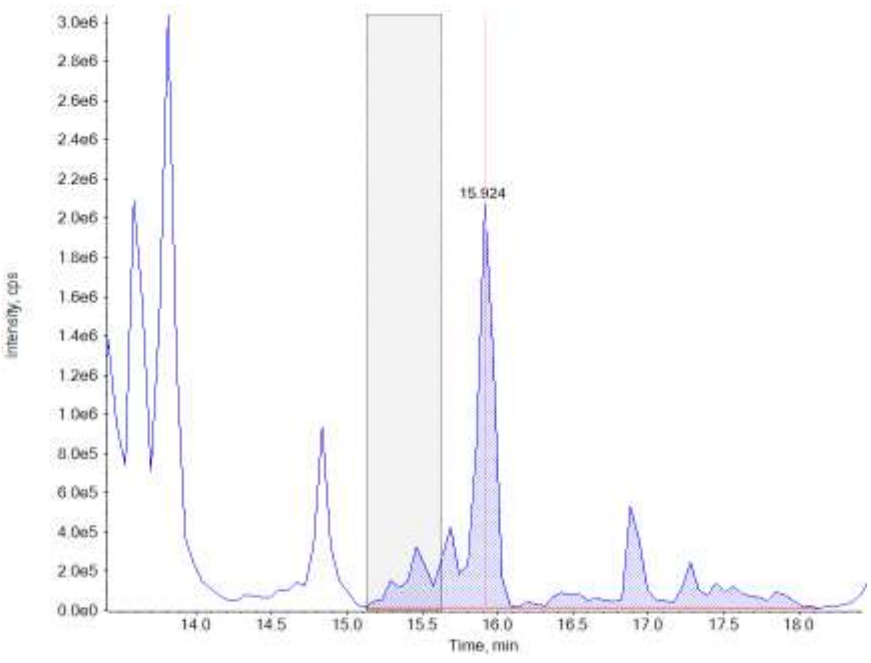

Acquired / Library MSMS

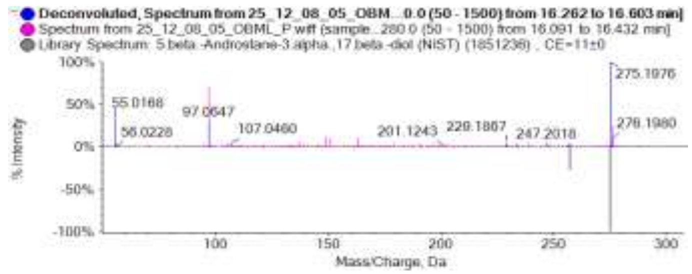

Acquired / Theoretical MS

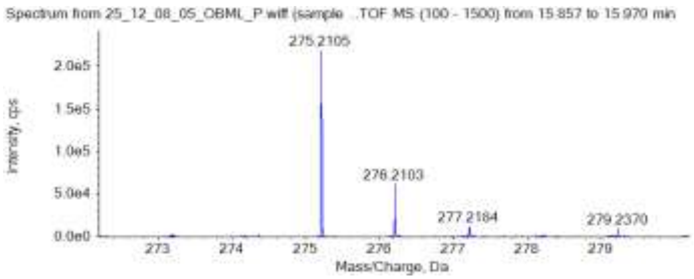

293.2237 / 15.91 (Mass/FragMass/RT/Isotope/Library/Formula/Ion Ratio)

Retention Time: 15.93 minutes **Exp RT:** 15.91 minutes
Precursor m/z : 293.2237 **Analyte Name:**
Fit (%) 89.9% **RFit (%)** 72.0% 293.2237 / 15.91

Collision Energy = 35 ± 15 eV

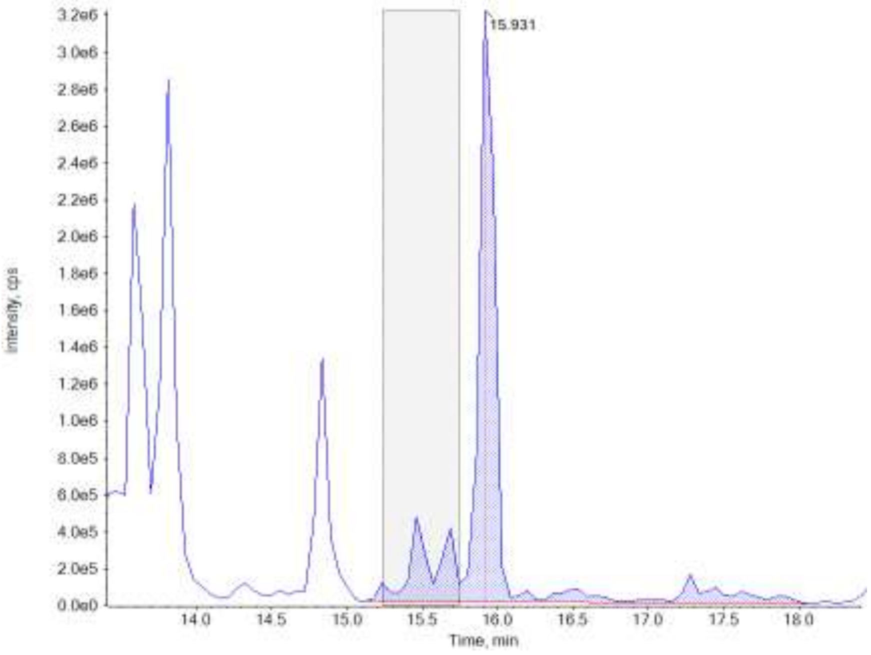

Acquired / Library MSMS

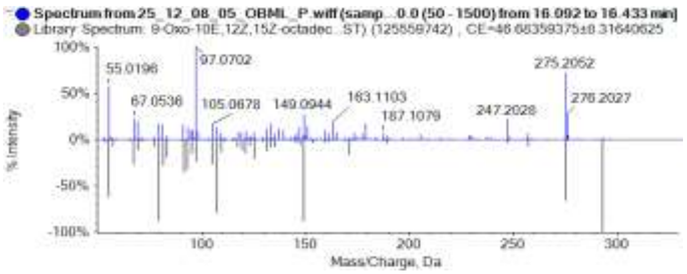

Acquired / Theoretical MS

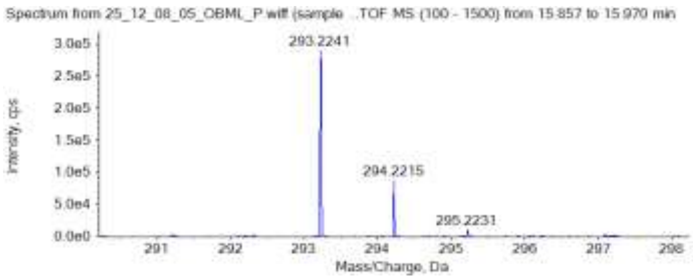

Created with SCIEX OS 3.3.0
Printed:12/9/2025 2:05:30 PM

341.3109 / 15.97 (Mass/FragMass/RT/Isotope/Library/Formula/Ion Ratio)

**Retention Time:** 15.90 minutes	**Exp RT:** 15.97 minutes
**Precursor m/z :** 341.3109	**Analyte Name:**
**Fit (%)** 90.6% **RFit (%)** 100.0%	341.3109 / 15.97

Collision Energy = 35 ± 15 eV

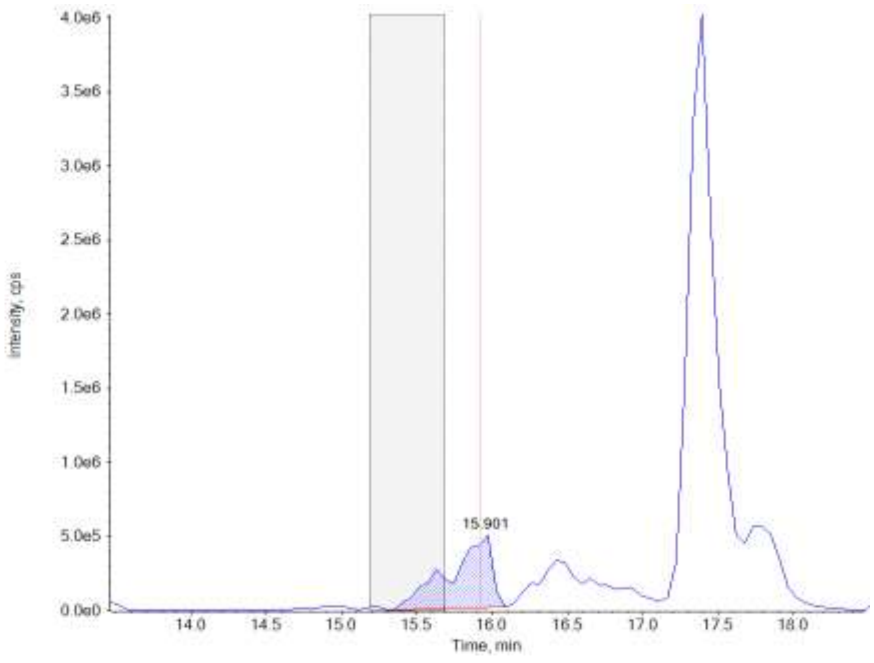

Acquired / Library MSMS

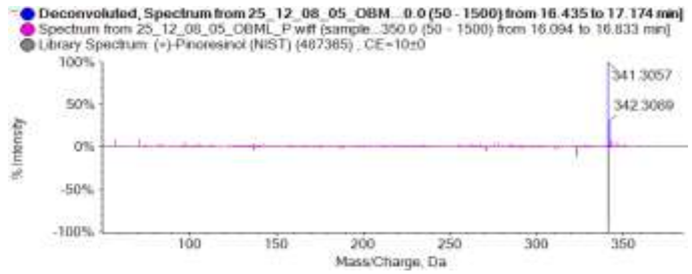

Acquired / Theoretical MS

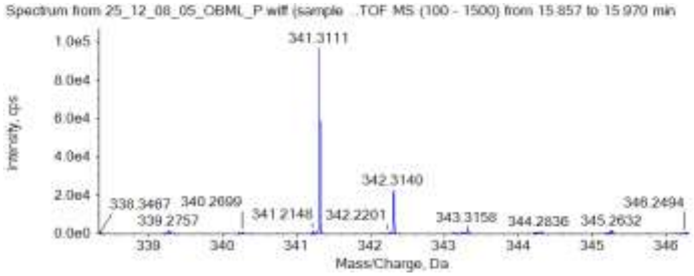

203.1843 / 16.14 (Mass/FragMass/RT/Isotope/Library/Formula/Ion Ratio)

**Retention Time:** 16.14 minutes	**Exp RT:** 16.14 minutes
**Precursor m/z :** 203.1843	**Analyte Name:**
**Fit (%)** 95.7% **RFit (%)** 54.2%	203.1843 / 16.14

Collision Energy = 35 ± 15 eV

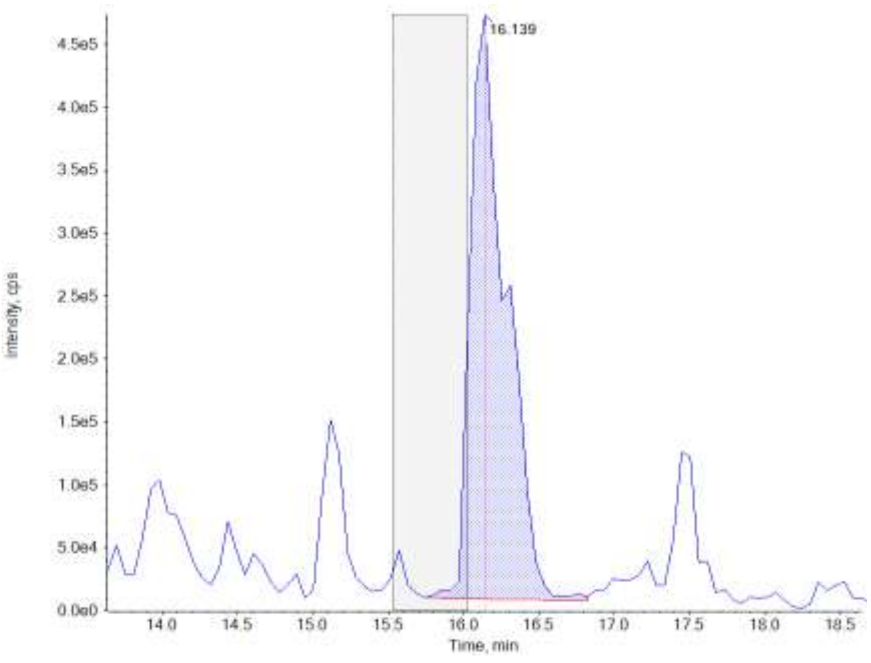

Acquired / Library MSMS

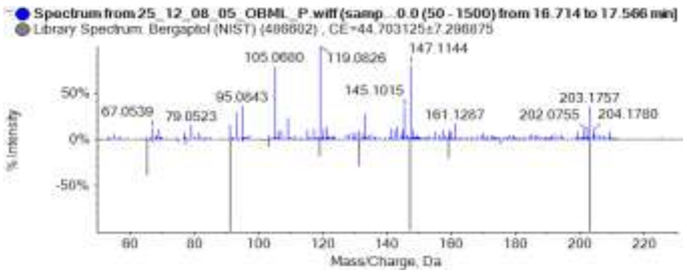

Acquired / Theoretical MS

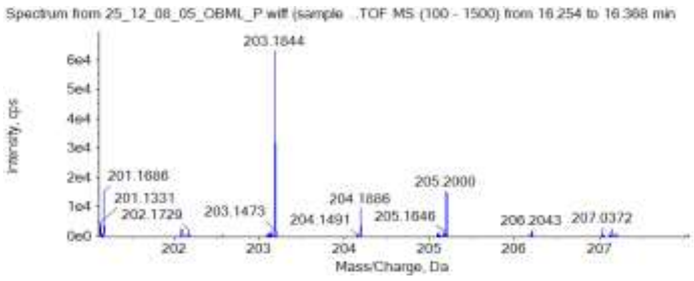

391.3438 / 16.14 (Mass/FragMass/RT/Isotope/Library/Formula/Ion Ratio)

Retention Time: 16.15 minutes **Exp RT:** 16.14 minutes
Precursor m/z : 391.3438 **Analyte Name:**
Fit (%) 56.7% **RFit (%)** 90.6% 391.3438 / 16.14

Collision Energy = 35 ± 15 eV

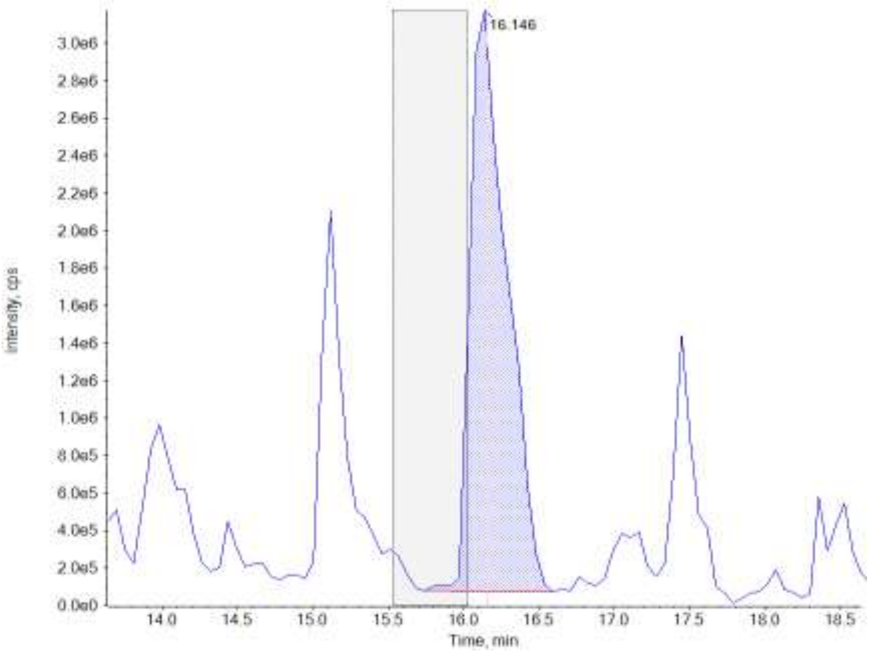

Acquired / Library MSMS

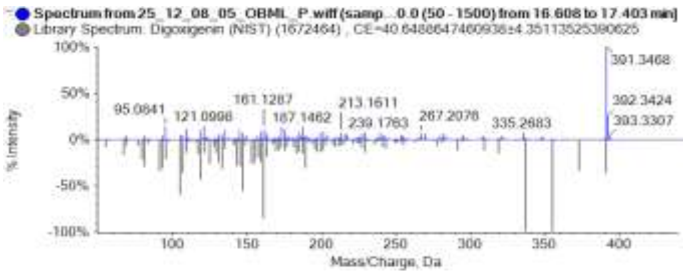

Acquired / Theoretical MS

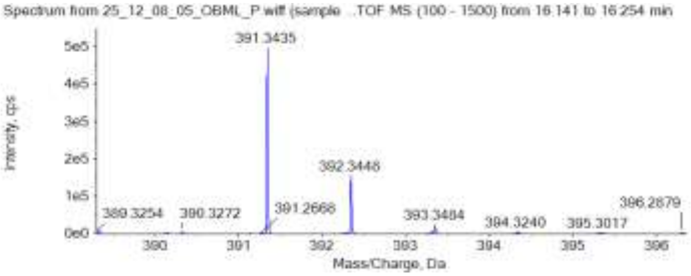

419.3442 / 16.14 (Mass/FragMass/RT/Isotope/Library/Formula/Ion Ratio)

Retention Time: 16.16 minutes **Exp RT:** 16.14 minutes
Precursor m/z : 419.3442 **Analyte Name:**
Fit (%) 62.2% **RFit (%)** 82.3% 419.3442 / 16.14

Collision Energy = 35 ± 15 eV

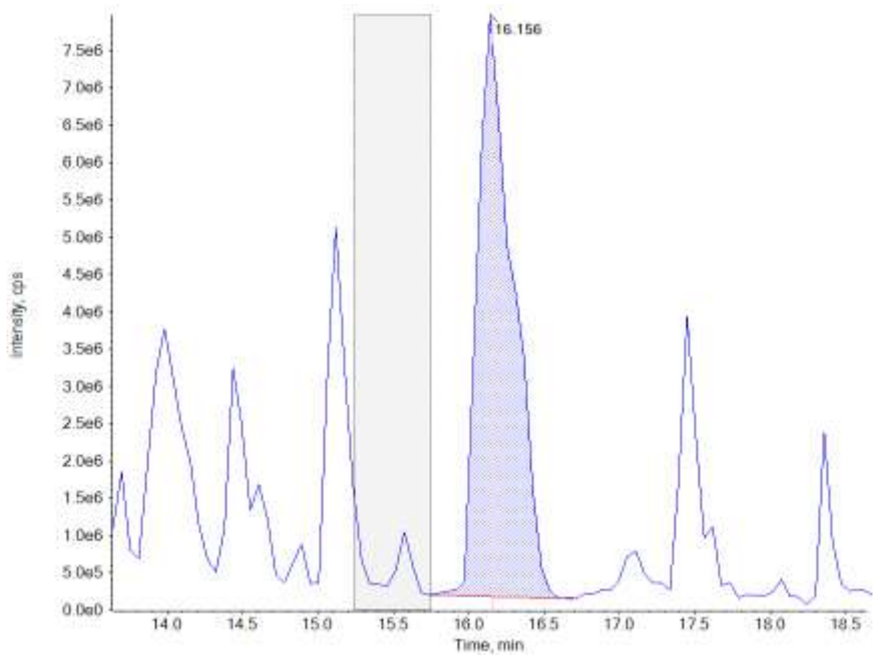

Acquired / Library MSMS

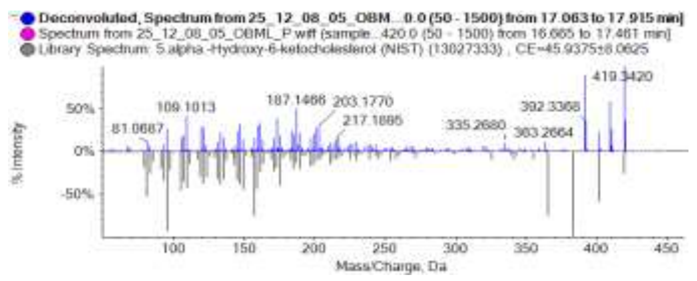

Acquired / Theoretical MS

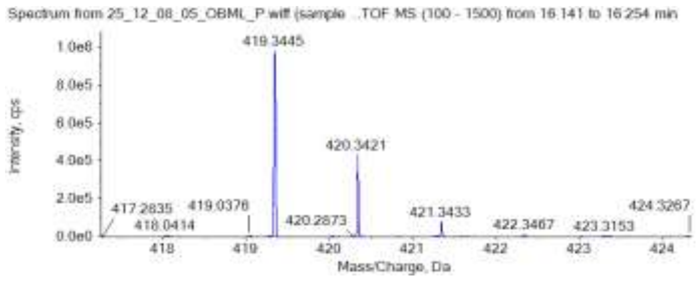

455.3604 / 16.14 (Mass/FragMass/RT/Isotope/Library/Formula/Ion Ratio)

Retention Time: 16.16 minutes
Precursor m/z : 455.3604
Fit (%) 93.7% **RFit (%)** 78.0%

Exp RT: 16.14 minutes
Analyte Name:
455.3604 / 16.14

Collision Energy = 35 ± 15 eV

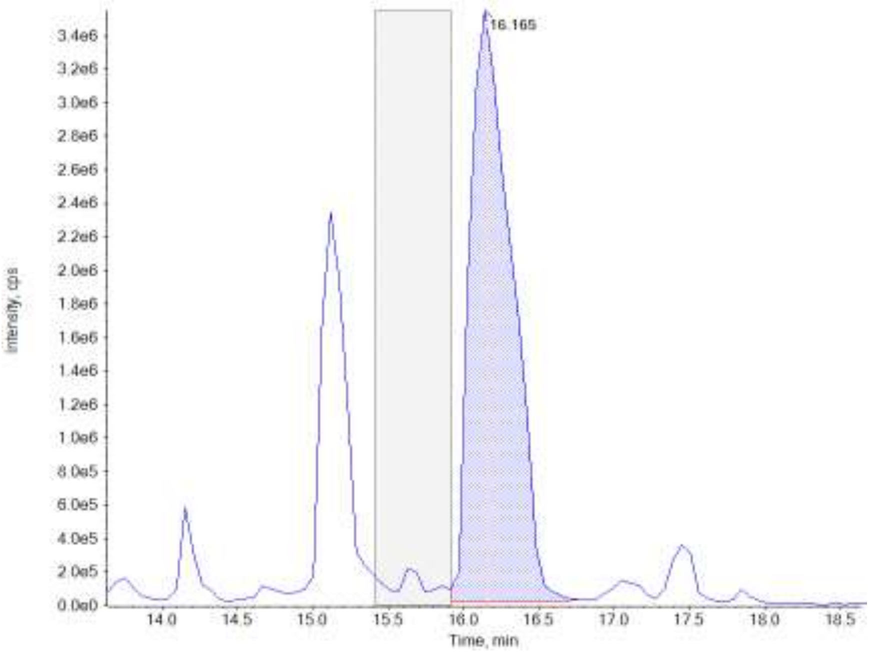

Acquired / Library MSMS

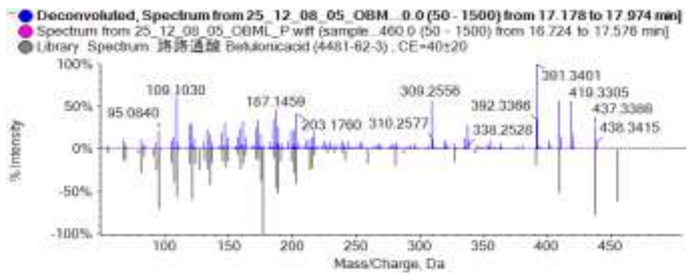

Acquired / Theoretical MS

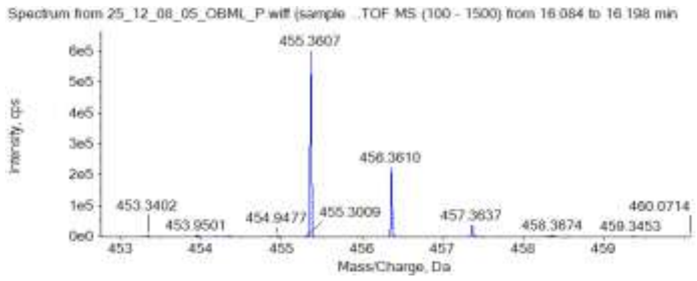

365.2737 / 16.42 (Mass/FragMass/RT/Isotope/Library/Formula/Ion Ratio)

**Retention Time:** 16.55 minutes	**Exp RT:** 16.43 minutes
**Precursor m/z :** 365.2737	**Analyte Name:**
**Fit (%)** 95.5% **RFit (%)** 88.0%	365.2737 / 16.42

Collision Energy = 35 ± 15 eV

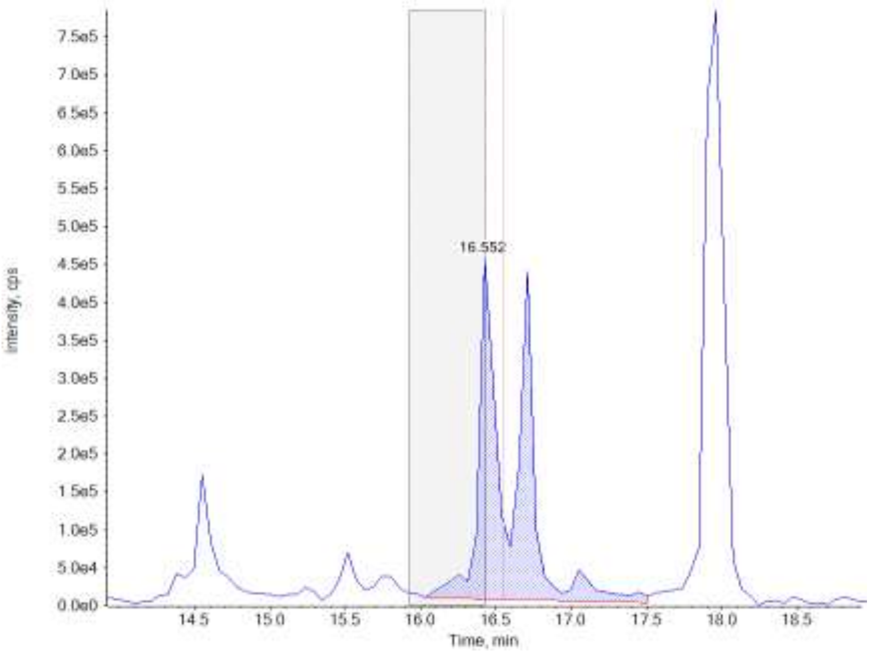

Acquired / Library MSMS

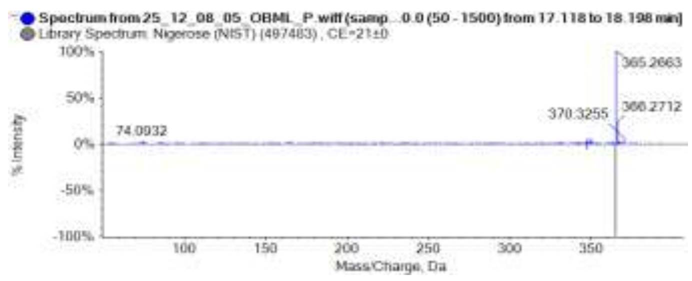

Acquired / Theoretical MS

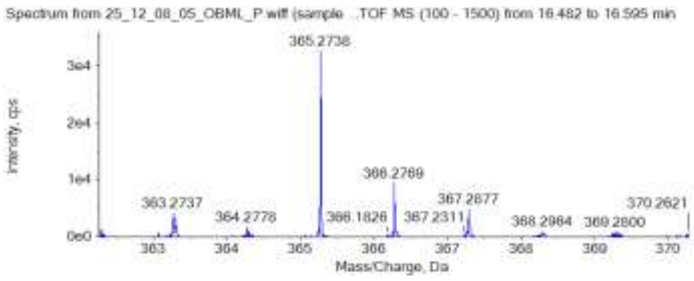

311.2634 / 16.48 (Mass/FragMass/RT/Isotope/Library/Formula/Ion Ratio)

Retention Time: 16.52 minutes
Precursor m/z : 311.2633
Fit (%) 93.4% **RFit (%)** 92.7%

Exp RT: 16.48 minutes
Analyte Name:
311.2634 / 16.48

Collision Energy = 35 ± 15 eV

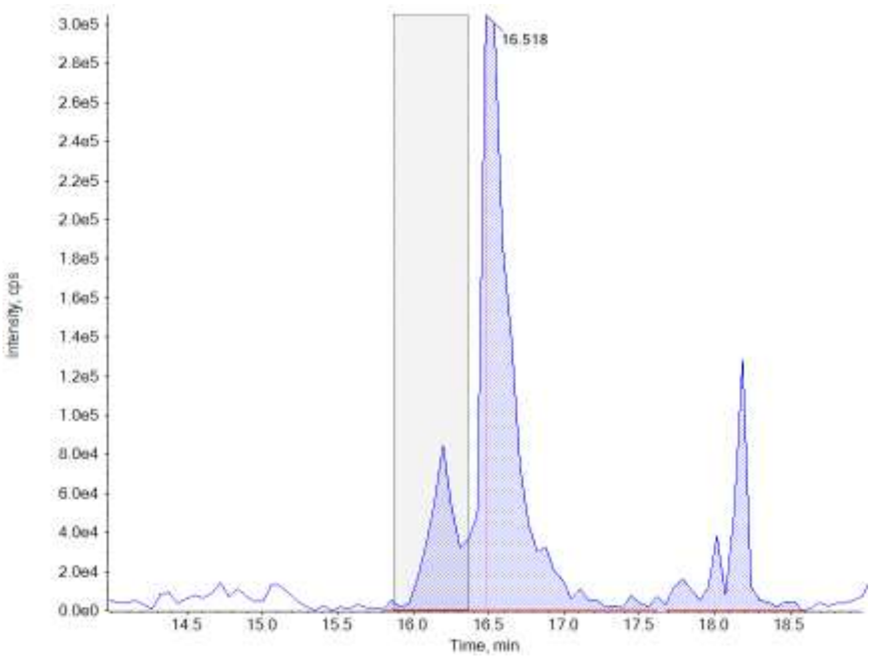

Acquired / Library MSMS

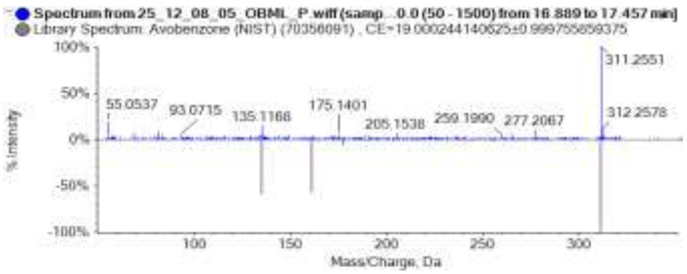

Acquired / Theoretical MS

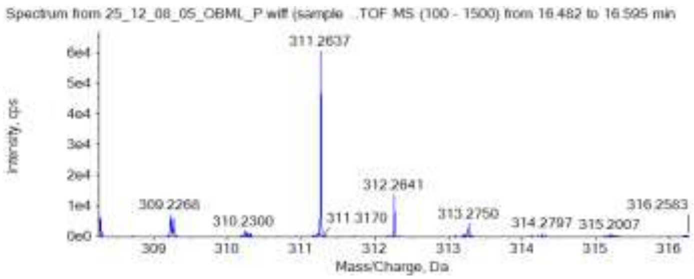

Created with SCIEX OS 3.3.0
Printed:12/9/2025 2:05:30 PM

423.3667 / 16.54 (Mass/FragMass/RT/Isotope/Library/Formula/Ion Ratio)

Retention Time: 16.54 minutes
Precursor m/z : 423.3666
Fit (%) 100.0% **RFit (%)** 30.1%

Exp RT: 16.54 minutes
Analyte Name:
423.3667 / 16.54

Collision Energy = 35 ± 15 eV

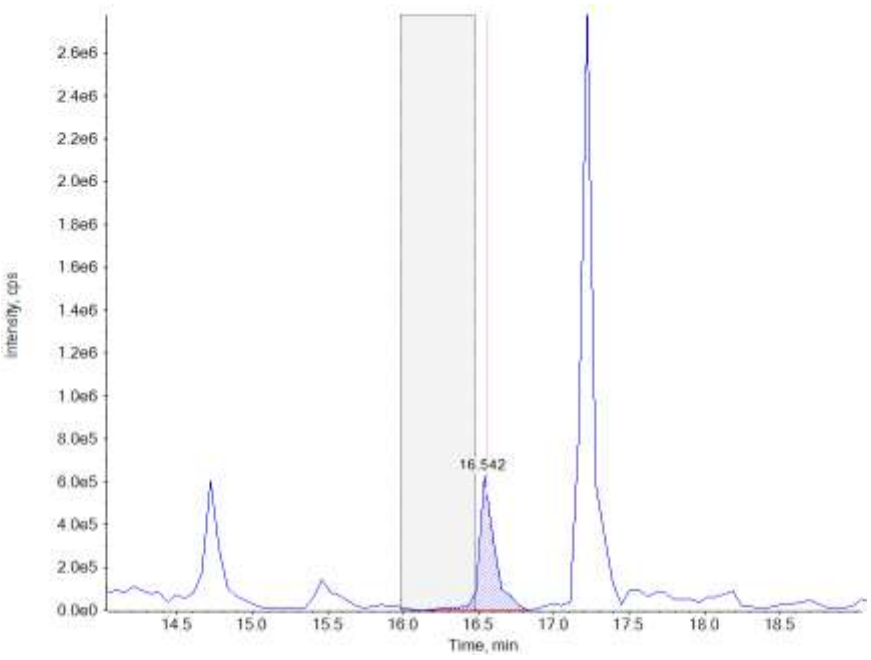

Acquired / Library MSMS

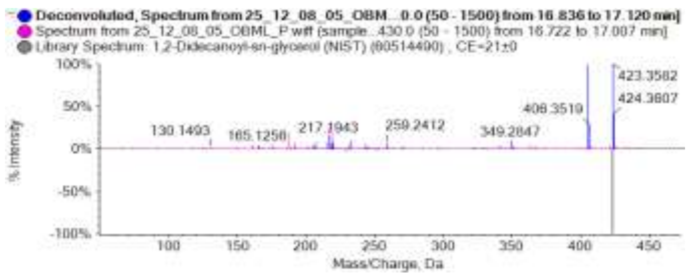

Acquired / Theoretical MS

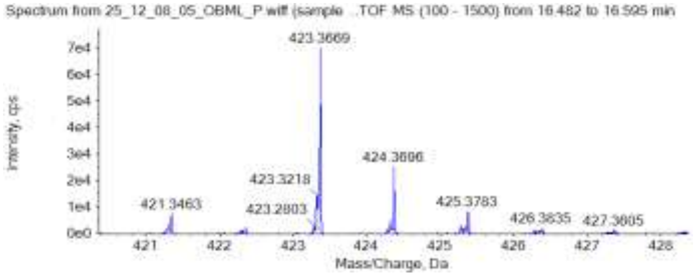

447.2565 / 16.54 (Mass/FragMass/RT/Isotope/Library/Formula/Ion Ratio)

Retention Time: 16.52 minutes
Precursor m/z : 447.2565
Fit (%) 99.2% **RFit (%)** 78.6%

Exp RT: 16.54 minutes
Analyte Name:
447.2565 / 16.54

Collision Energy = 35 ± 15 eV

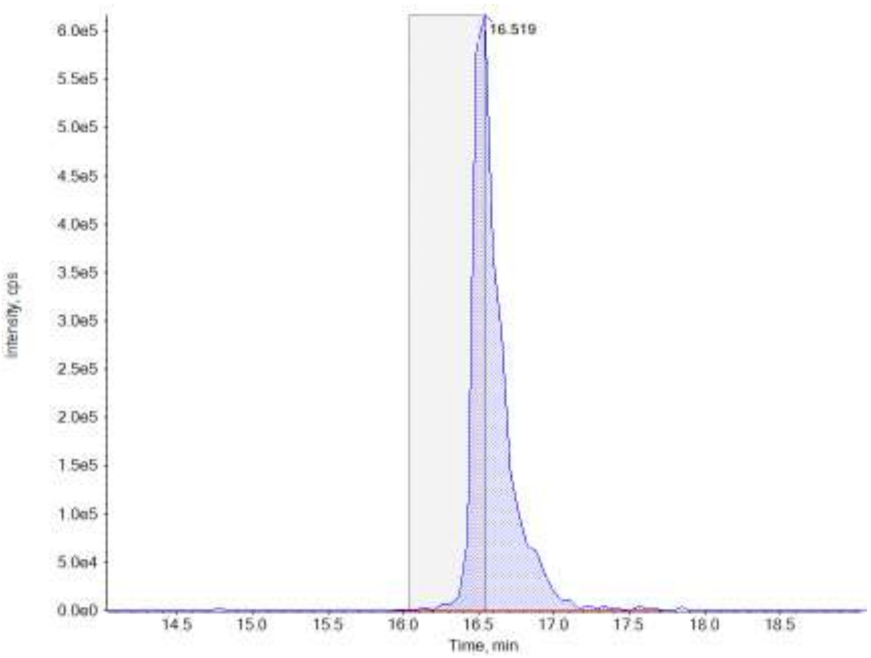

Acquired / Library MSMS

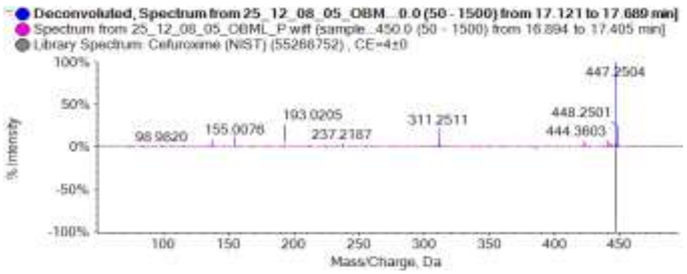

Acquired / Theoretical MS

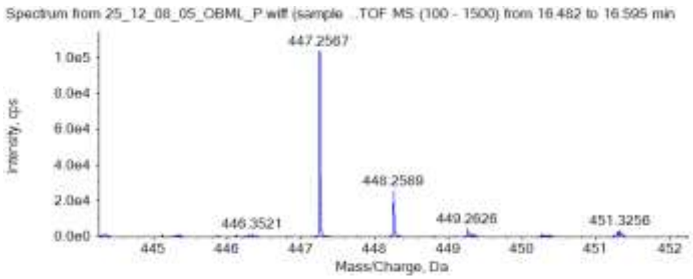

179.1481 / 16.60 (Mass/FragMass/RT/Isotope/Library/Formula/Ion Ratio)

**Retention Time:** 16.60 minutes	**Exp RT:** 16.60 minutes
**Precursor m/z :** 179.1481	**Analyte Name:**
**Fit (%)** 96.7% **RFit (%)** 35.8%	179.1481 / 16.60

Collision Energy = 35 ± 15 eV

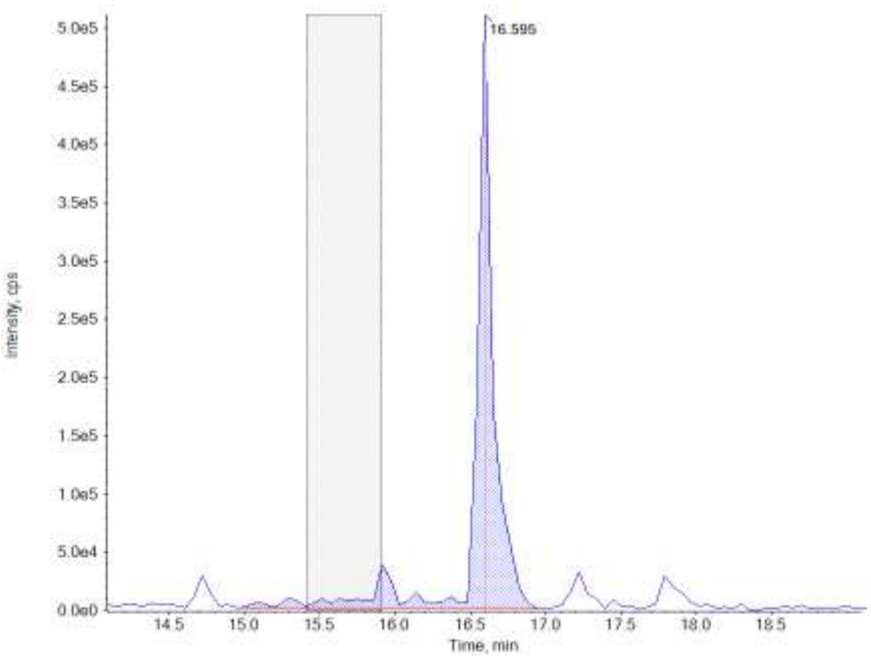

Acquired / Library MSMS

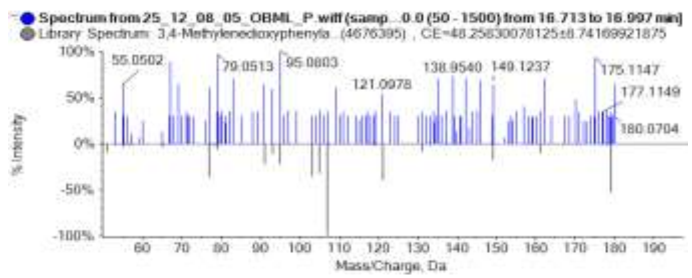

Acquired / Theoretical MS

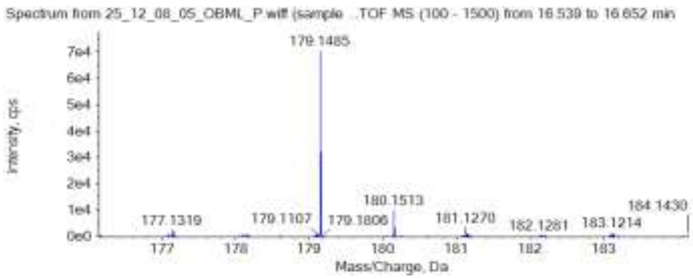

277.2256 / 16.60 (Mass/FragMass/RT/Isotope/Library/Formula/Ion Ratio)

**Retention Time:** 16.63 minutes	**Exp RT:** 16.60 minutes
**Precursor m/z :** 277.2256	**Analyte Name:**
**Fit (%)** 99.1% **RFit (%)** 99.1%	277.2256 / 16.60

Collision Energy = 35 ± 15 eV

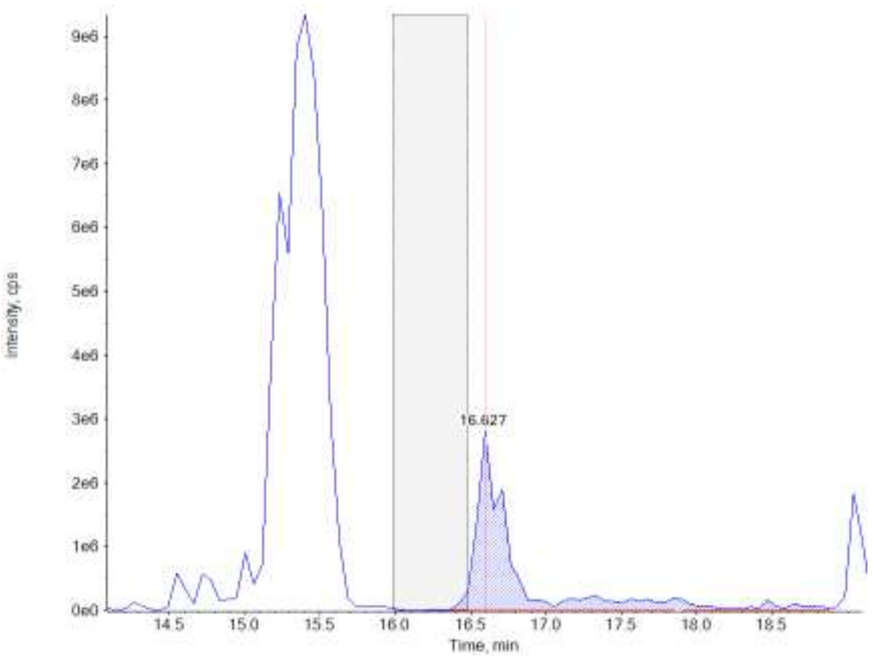

Acquired / Library MSMS

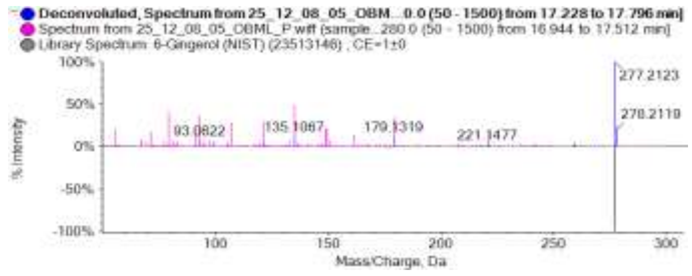

Acquired / Theoretical MS

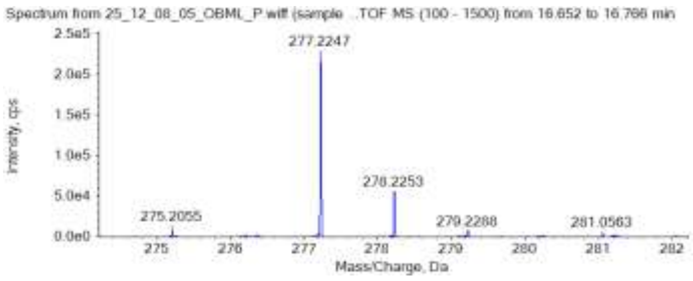

295.2359 / 16.60 (Mass/FragMass/RT/Isotope/Library/Formula/Ion Ratio)

Retention Time: 16.64 minutes
Precursor m/z : 295.2359
Fit (%) 82.2% **RFit (%)** 98.7%

Exp RT: 16.60 minutes
Analyte Name:
295.2359 / 16.60

Collision Energy = 35 ± 15 eV

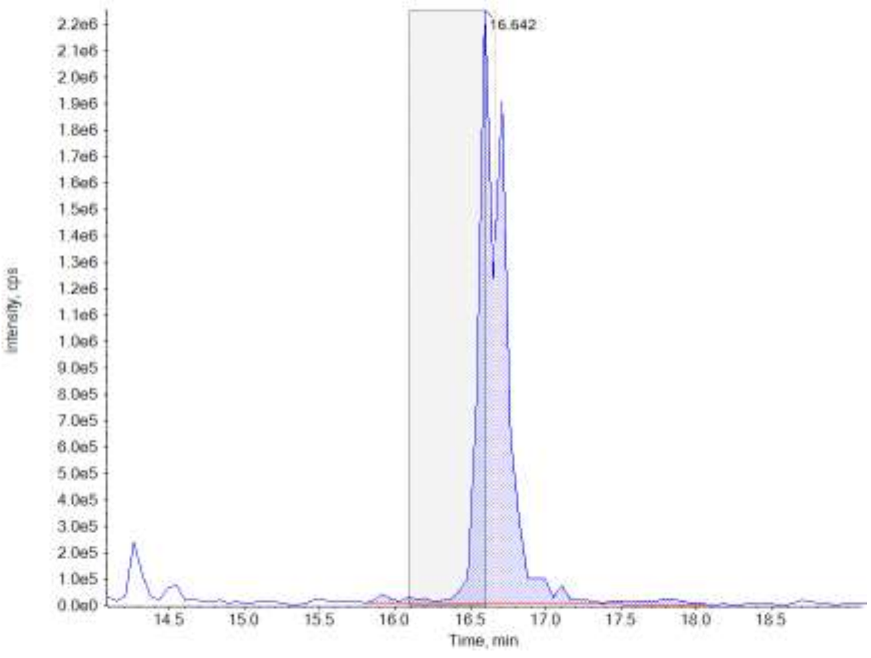

Acquired / Library MSMS

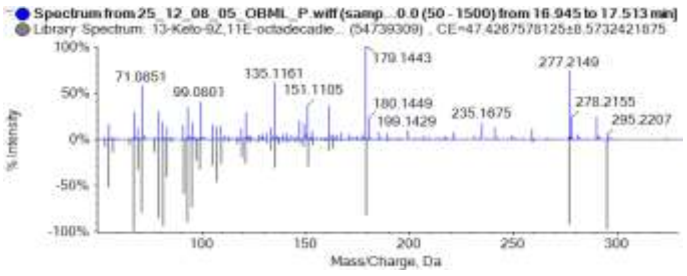

Acquired / Theoretical MS

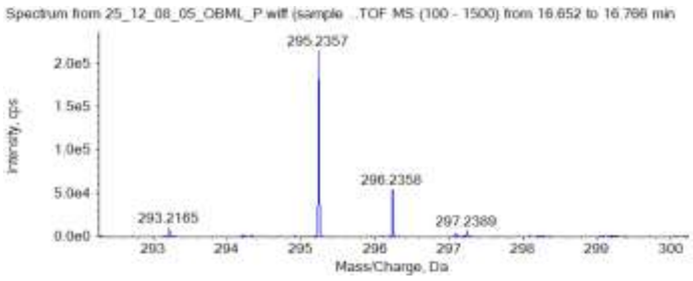

454.2976 / 16.77

(Mass/FragMass/RT/Isotope/Library/Formula/Ion Ratio)

**Retention Time:** 16.77 minutes	**Exp RT:** 16.77 minutes
**Precursor m/z :** 454.2976	**Analyte Name:**
**Fit (%)** 82.3% **RFit (%)** 100.0%	454.2976 / 16.77

Collision Energy = 35 ± 15 eV

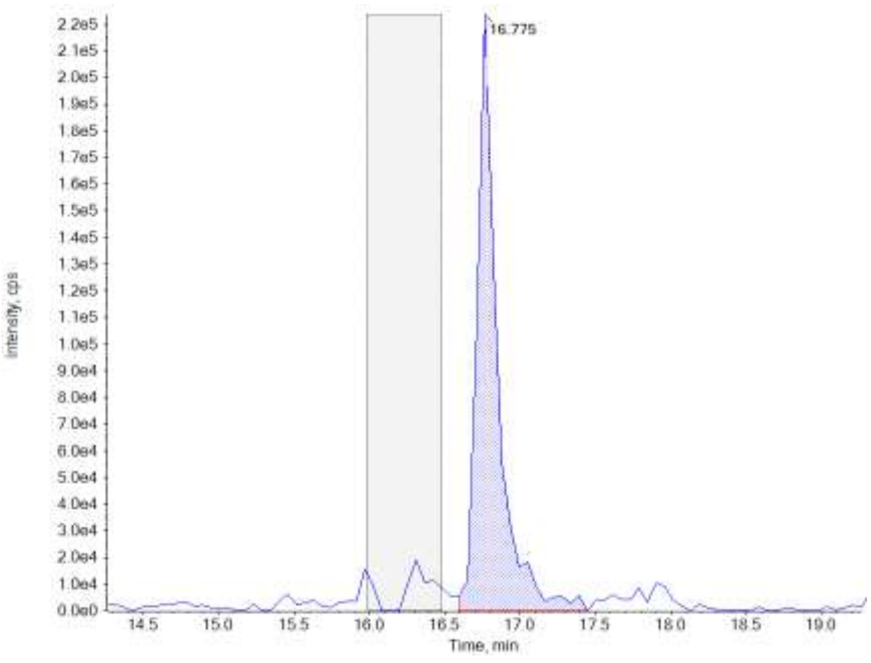

Acquired / Library MSMS

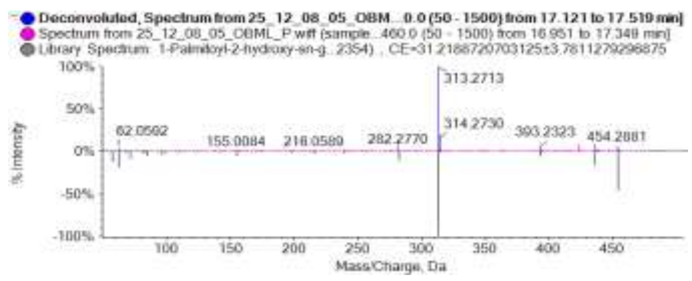

Acquired / Theoretical MS

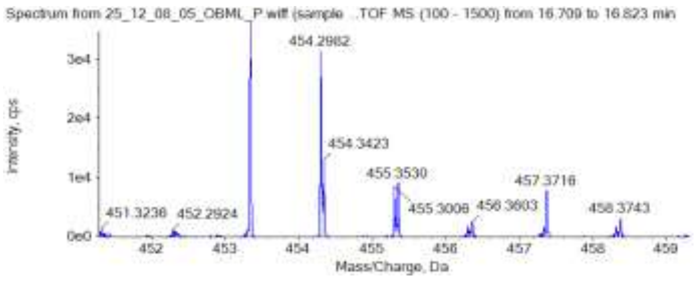

423.3201 / 16.82 (Mass/FragMass/RT/Isotope/Library/Formula/Ion Ratio)

Retention Time: 16.83 minutes
Precursor m/z : 423.3201
Fit (%) 100.0% **RFit (%)** 25.4%

Exp RT: 16.82 minutes
Analyte Name:
423.3201 / 16.82

Collision Energy = 35 ± 15 eV

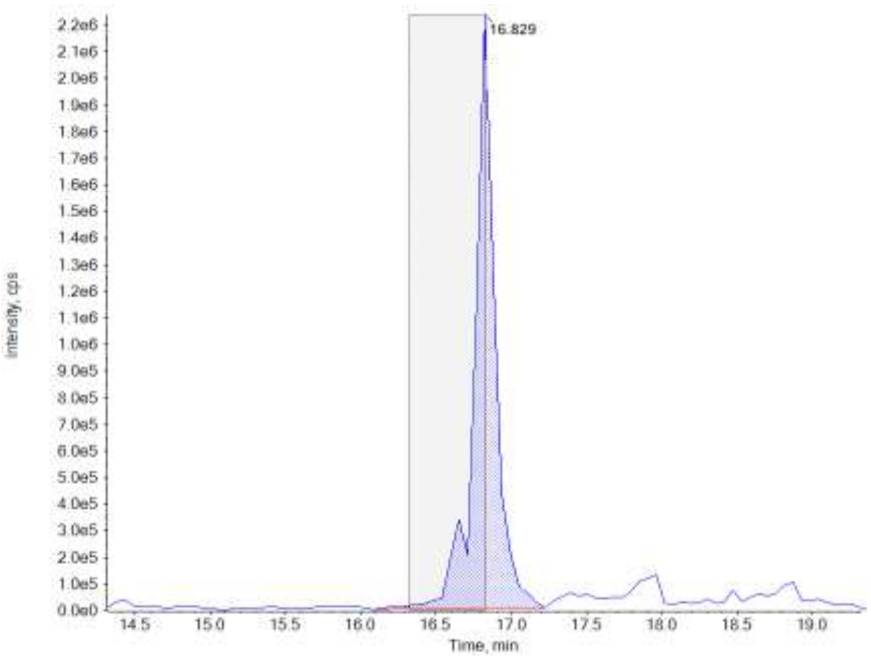

Acquired / Library MSMS

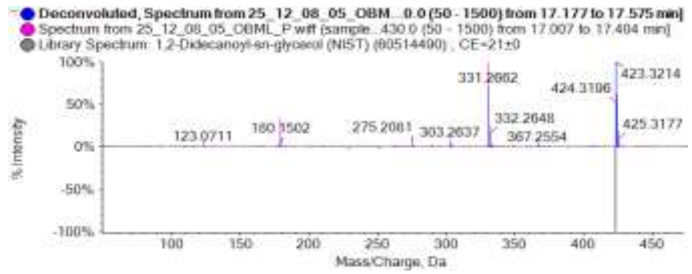

Acquired / Theoretical MS

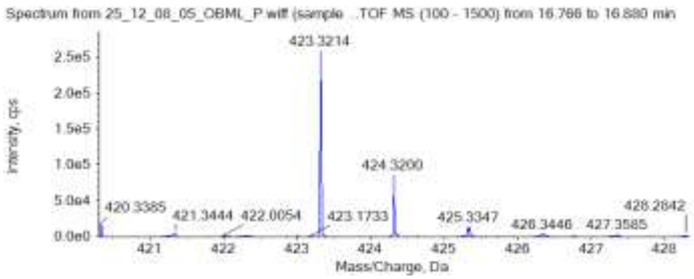

Created with SCIEX OS 3.3.0
Printed:12/9/2025 2:05:30 PM

305.2533 / 16.88 (Mass/FragMass/RT/Isotope/Library/Formula/Ion Ratio)

Retention Time: 16.88 minutes
Precursor m/z : 305.2533
Fit (%) 97.1% **RFit (%)** 96.0%

Exp RT: 16.88 minutes
Analyte Name:
305.2533 / 16.88

Collision Energy = 35 ± 15 eV

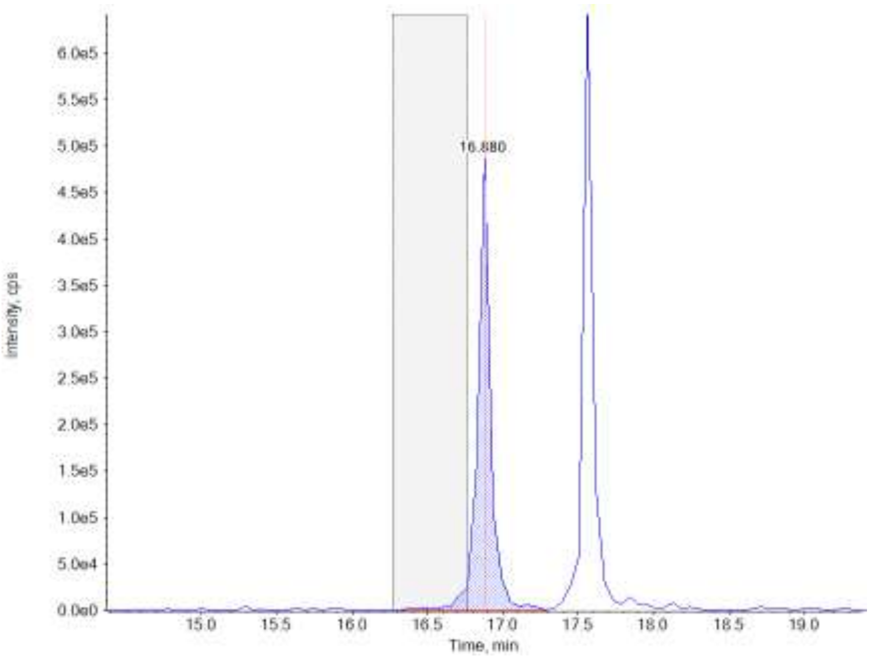

Acquired / Library MSMS

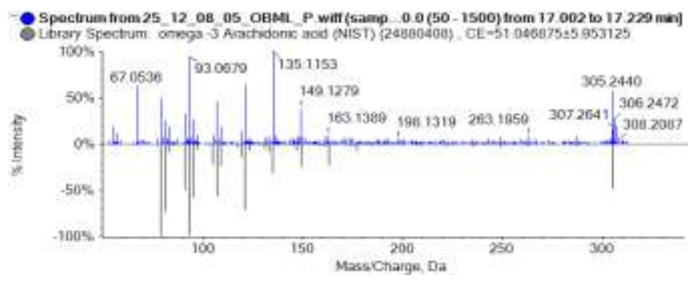

Acquired / Theoretical MS

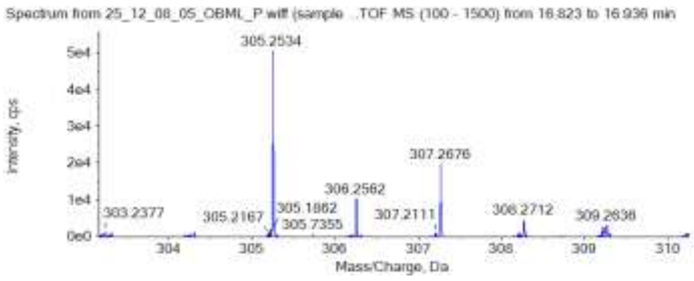

653.3003 / 16.88 (Mass/FragMass/RT/Isotope/Library/Formula/Ion Ratio)

Retention Time: 16.91 minutes
Precursor m/z : 653.3003
Fit (%) 100.0% **RFit (%)** 98.6%

Exp RT: 16.88 minutes
Analyte Name:
653.3003 / 16.88

Collision Energy = 35 ± 15 eV

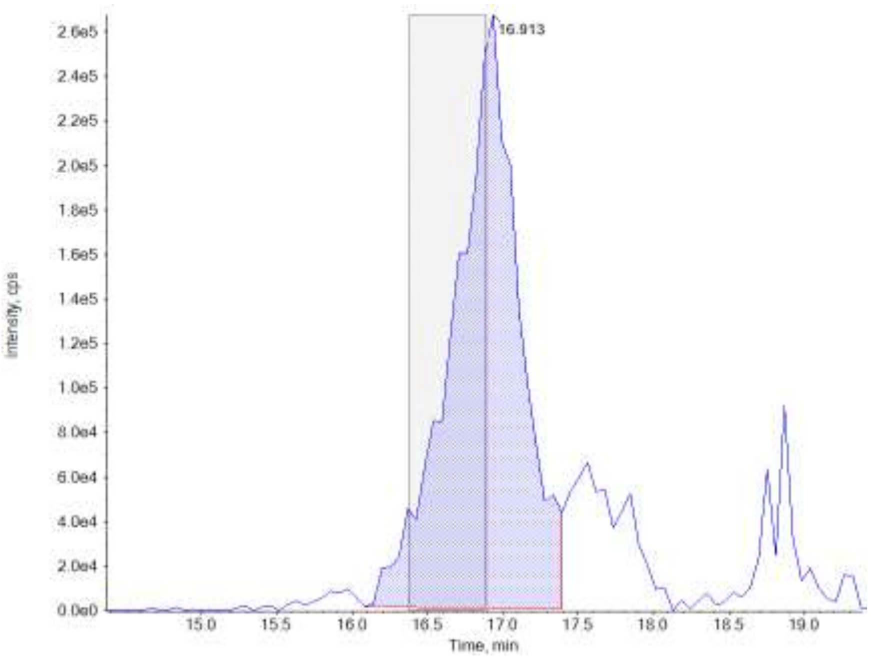

Acquired / Library MSMS

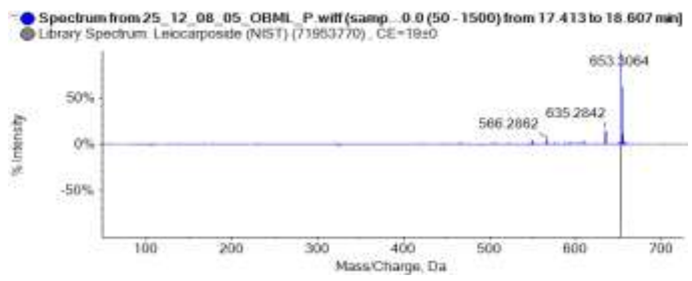

Acquired / Theoretical MS

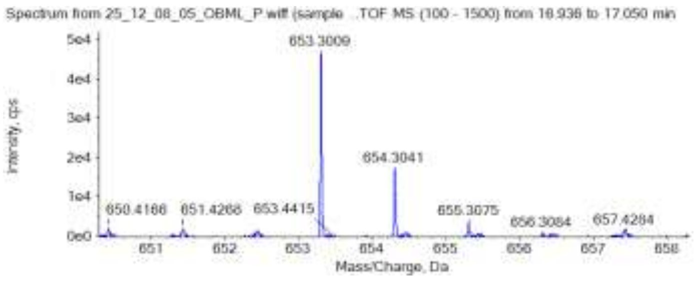

453.3428 / 17.16 (Mass/FragMass/RT/Isotope/Library/Formula/Ion Ratio)

Retention Time: 17.16 minutes | **Exp RT:** 17.16 minutes
Precursor m/z : 453.3428 | **Analyte Name:**
Fit (%) 100.0% **RFit (%)** 24.6% | 453.3428 / 17.16

Collision Energy = 35 ± 15 eV

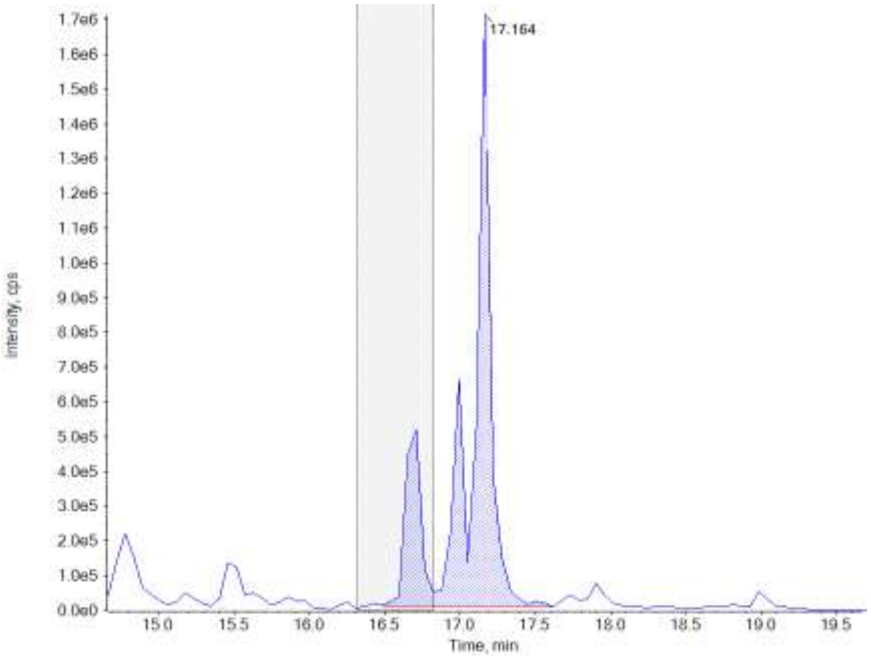

Acquired / Library MSMS

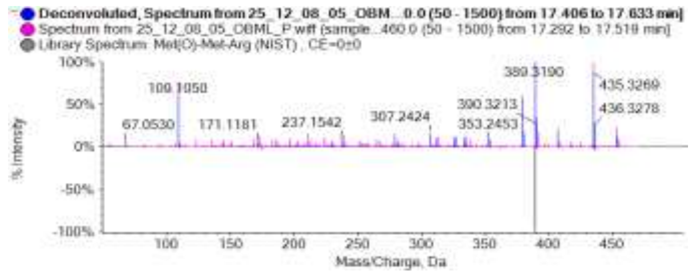

Acquired / Theoretical MS

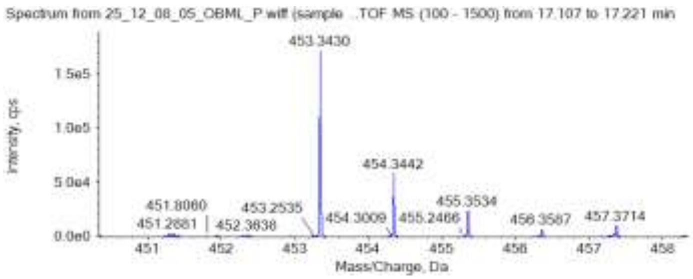

338.3514 / 17.28 (Mass/FragMass/RT/Isotope/Library/Formula/Ion Ratio)

Retention Time: 17.25 minutes
Precursor m/z : 338.3514
Fit (%) 95.1% **RFit (%)** 97.7%

Exp RT: 17.28 minutes
Analyte Name:
338.3514 / 17.28

Collision Energy = 35 ± 15 eV

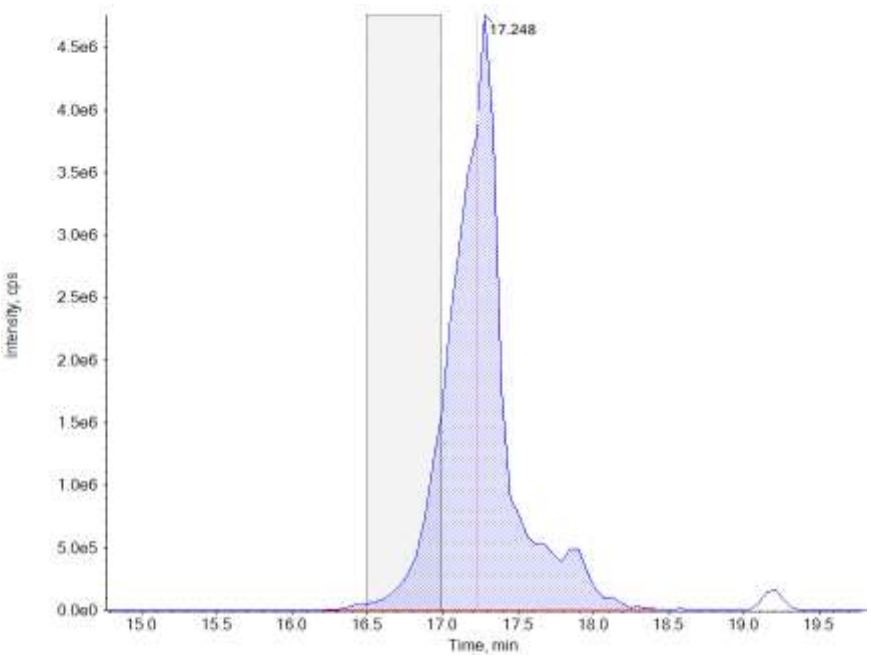

Acquired / Library MSMS

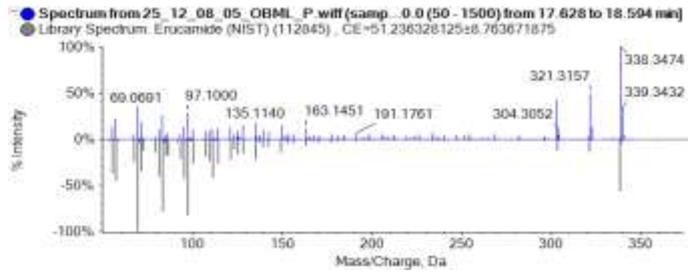

Acquired / Theoretical MS

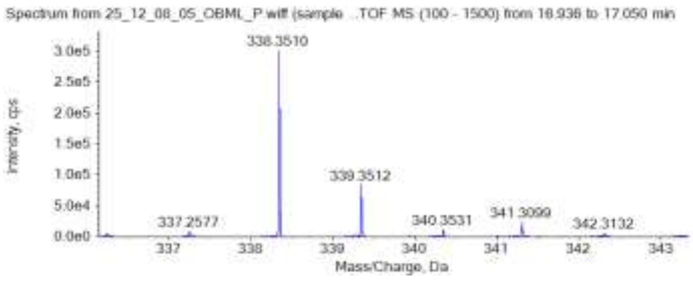

359.3228 / 17.39 (Mass/FragMass/RT/Isotope/Library/Formula/Ion Ratio)

Retention Time: 17.38 minutes
Precursor m/z : 359.3228
Fit (%) 98.5% **RFit (%)** 95.9%

Exp RT: 17.39 minutes
Analyte Name:
359.3228 / 17.39

Collision Energy = 35 ± 15 eV

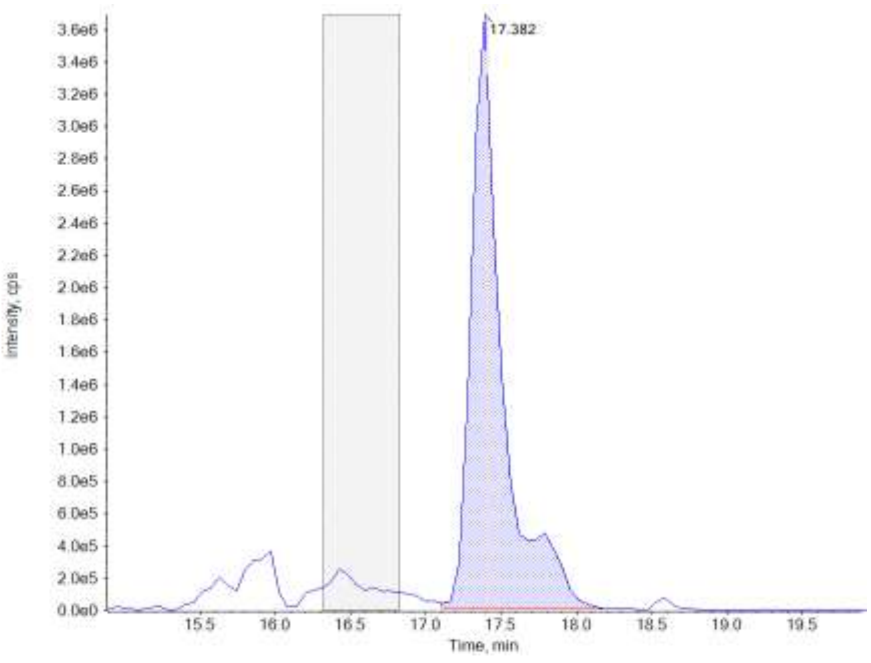

Acquired / Library MSMS

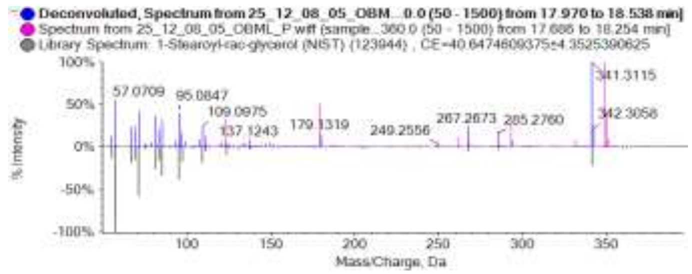

Acquired / Theoretical MS

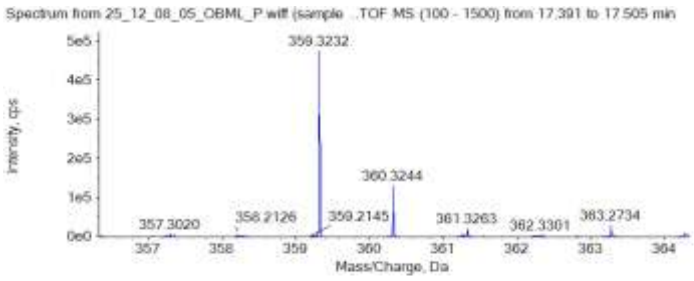

391.3412 / 17.45 (Mass/FragMass/RT/Isotope/Library/Formula/Ion Ratio)

Retention Time: 17.46 minutes
Precursor m/z : 391.3412
Fit (%) 100.0% **RFit (%)** 81.9%

Exp RT: 17.45 minutes
Analyte Name:
391.3412 / 17.45

Collision Energy = 35 ± 15 eV

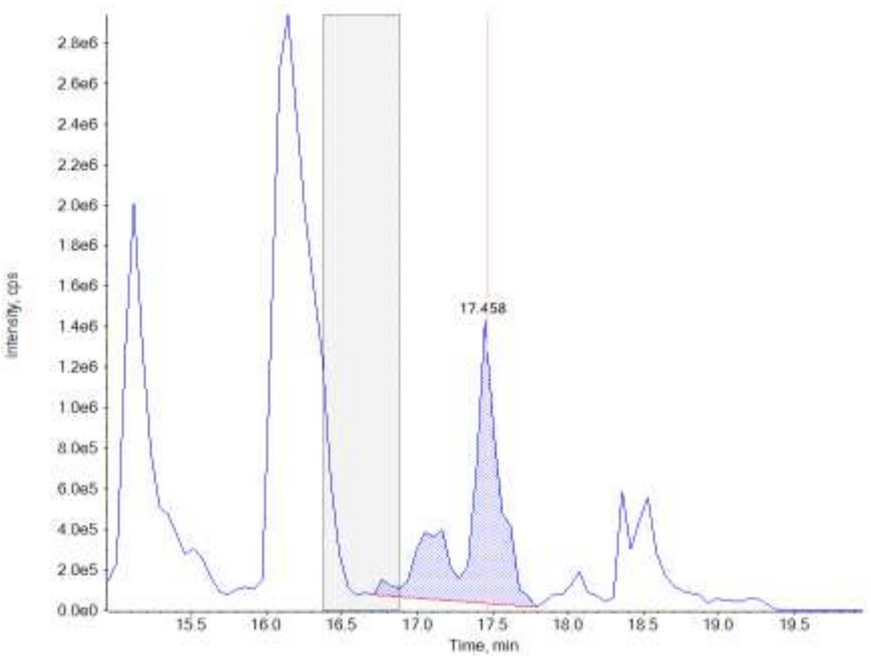

Acquired / Library MSMS

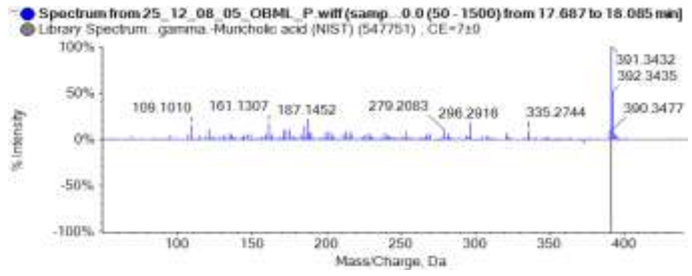

Acquired / Theoretical MS

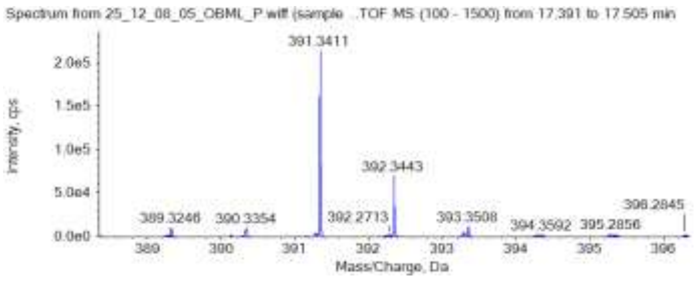

419.3373 / 17.45 (Mass/FragMass/RT/Isotope/Library/Formula/Ion Ratio)

Retention Time: 17.46 minutes **Exp RT:** 17.45 minutes
Precursor m/z : 419.3373 **Analyte Name:**
Fit (%) 73.8% **RFit (%)** 90.3% 419.3373 / 17.45

Collision Energy = 35 ± 15 eV

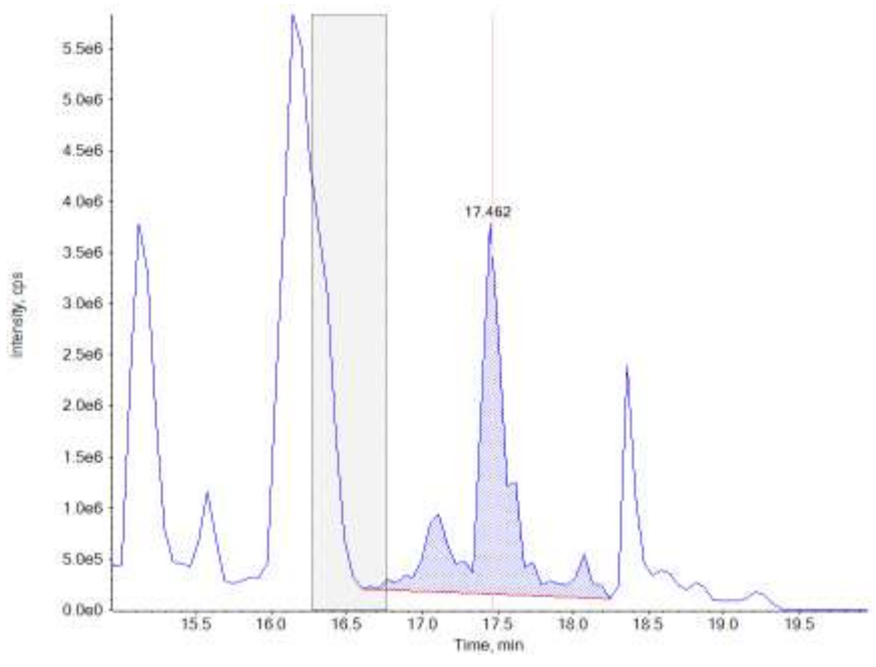

Acquired / Library MSMS

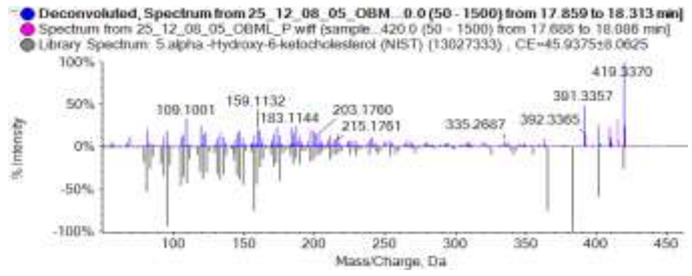

Acquired / Theoretical MS

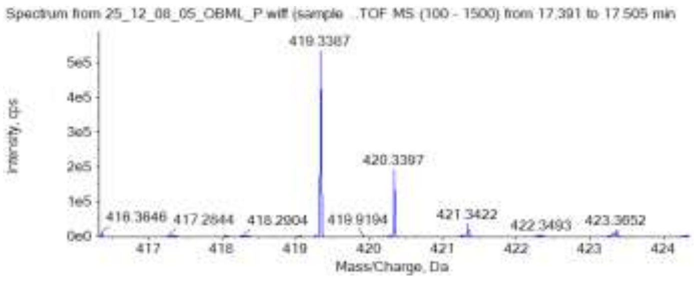

353.2759 / 17.50 (Mass/FragMass/RT/Isotope/Library/Formula/Ion Ratio)

**Retention Time:** 17.47 minutes	**Exp RT:** 17.51 minutes
**Precursor m/z :** 353.2759	**Analyte Name:**
**Fit (%)** 96.5% **RFit (%)** 96.6%	353.2759 / 17.50

Collision Energy = 35 ± 15 eV

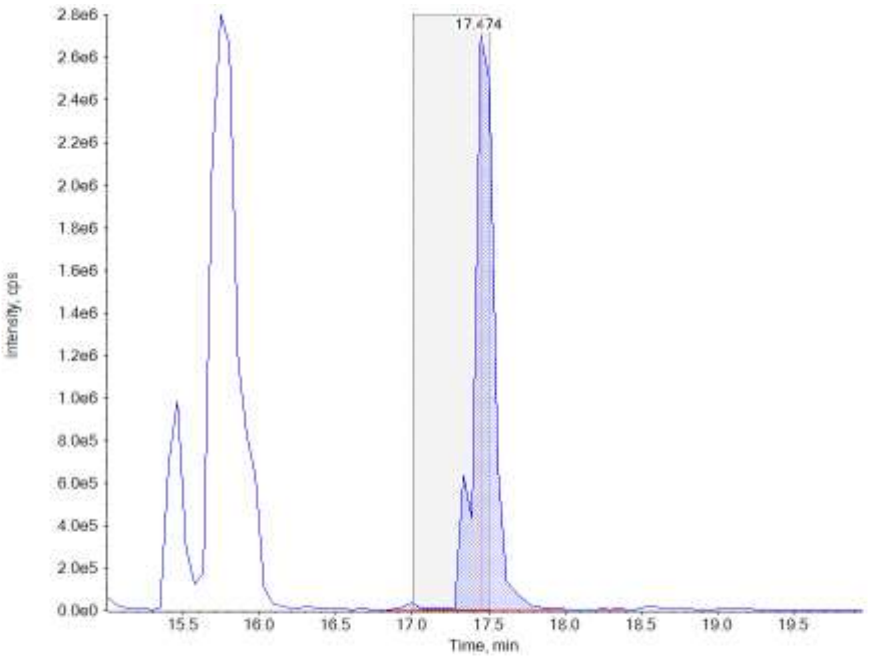

Acquired / Library MSMS

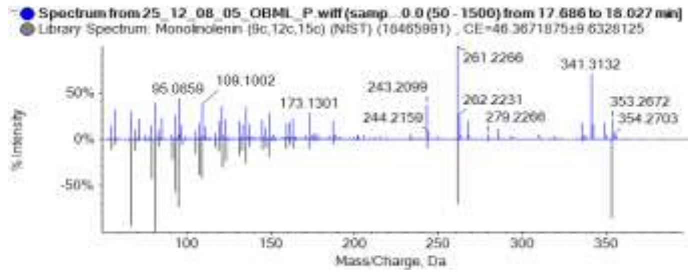

Acquired / Theoretical MS

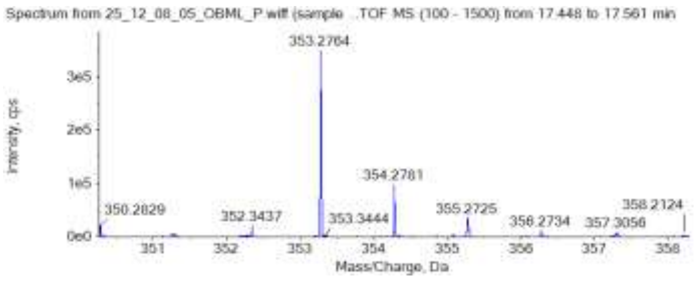

599.4148 / 17.50 (Mass/FragMass/RT/Isotope/Library/Formula/Ion Ratio)

Retention Time: 17.52 minutes
Precursor m/z : 599.4148
Fit (%) 42.4% **RFit (%)** 97.5%

Exp RT: 17.51 minutes
Analyte Name:
599.4148 / 17.50

Collision Energy = 35 ± 15 eV

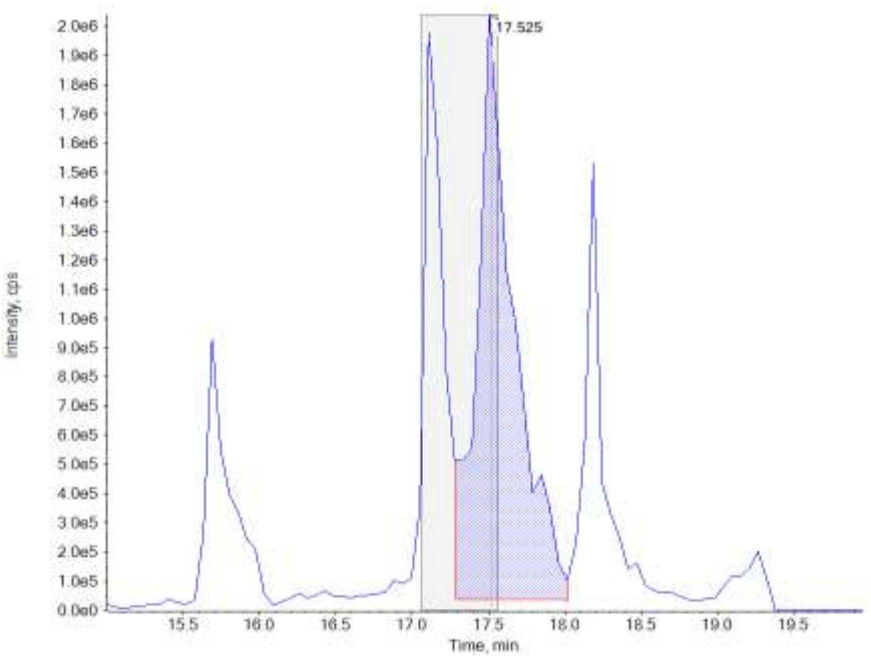

Acquired / Library MSMS

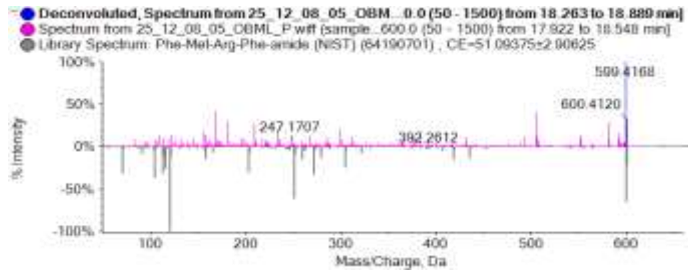

Acquired / Theoretical MS

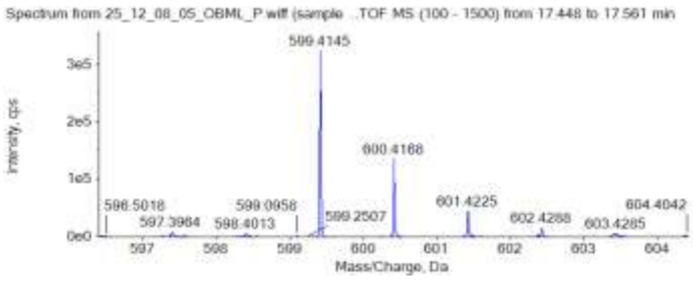

367.2695 / 17.56

(Mass/FragMass/RT/Isotope/Library/Formula/Ion Ratio)

Retention Time: 17.56 minutes
Precursor m/z : 367.2695
Fit (%) 77.6% **RFit (%)** 94.7%

Exp RT: 17.56 minutes
Analyte Name:
367.2695 / 17.56

Collision Energy = 35 ± 15 eV

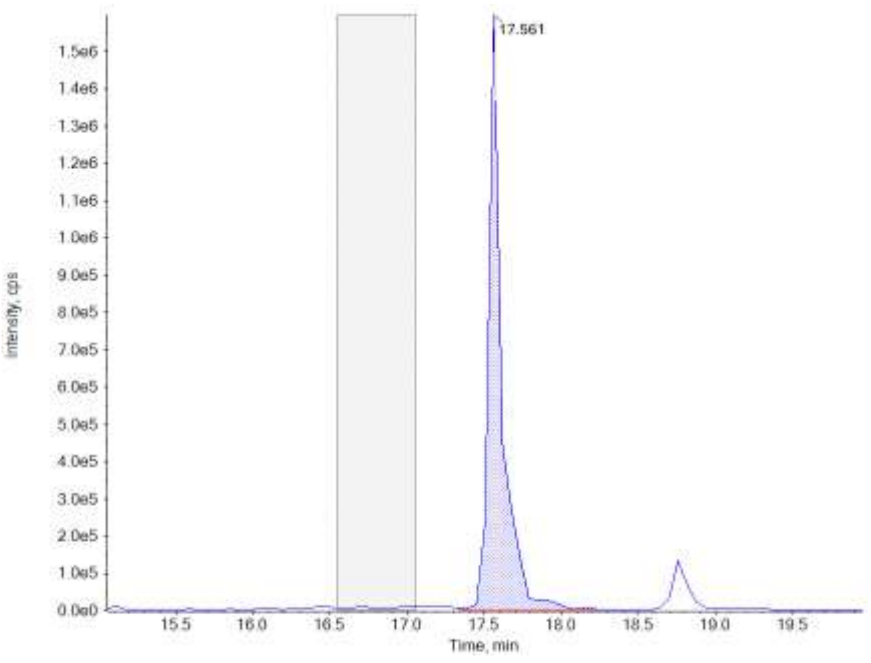

Acquired / Library MSMS

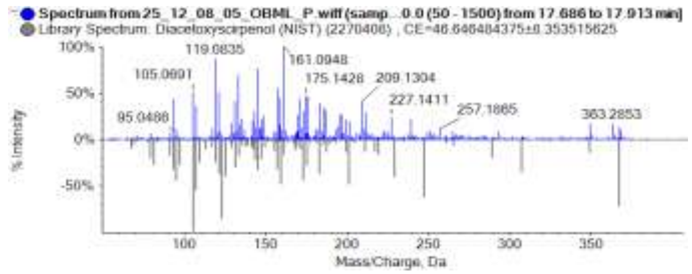

Acquired / Theoretical MS

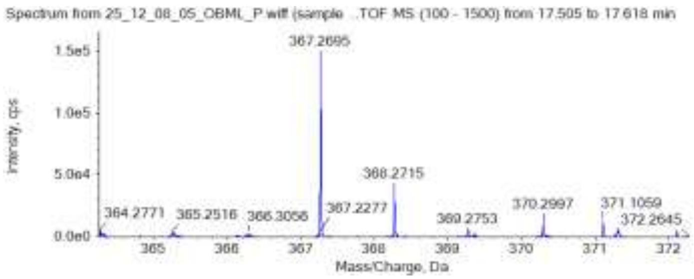

609.4562 / 17.56

(Mass/FragMass/RT/Isotope/Library/Formula/Ion Ratio)

Retention Time: 17.56 minutes
Precursor m/z : 609.4562
Fit (%) 98.8% **RFit (%)** 92.0%

Exp RT: 17.56 minutes
Analyte Name:
609.4562 / 17.56

Collision Energy = 35 ± 15 eV

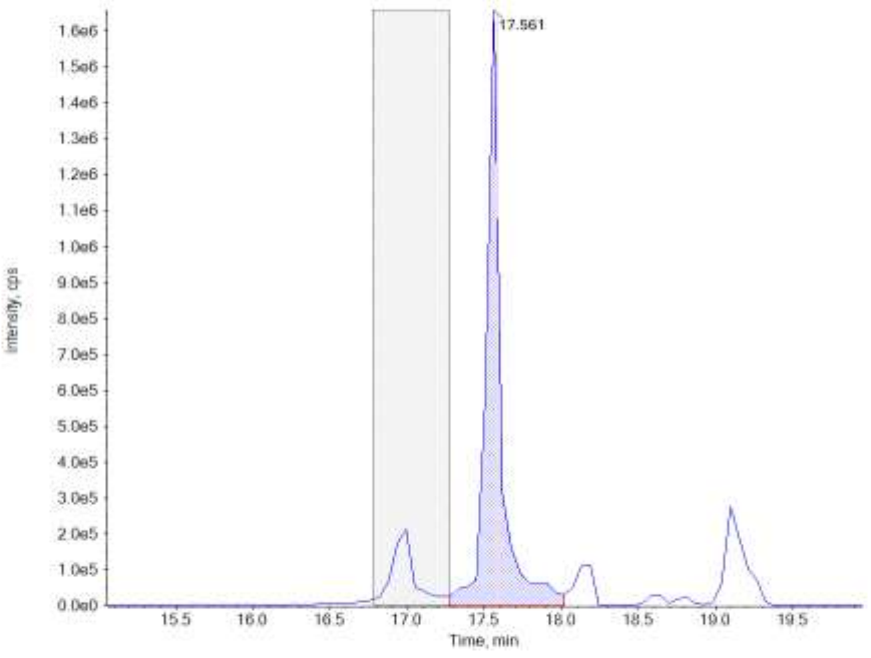

Acquired / Library MSMS

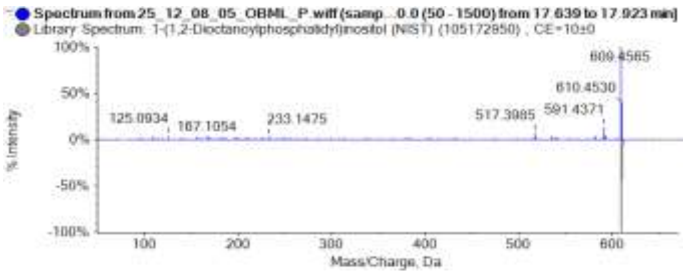

Acquired / Theoretical MS

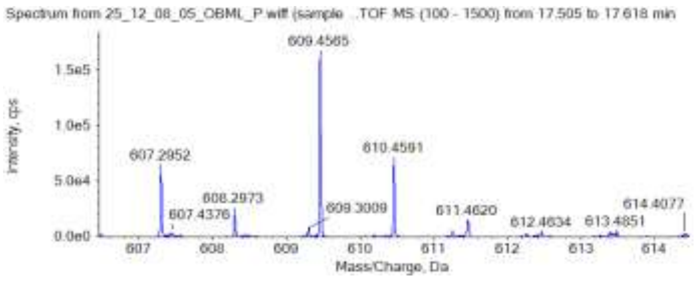

667.3163 / 17.62 (Mass/FragMass/RT/Isotope/Library/Formula/Ion Ratio)

Retention Time: 17.63 minutes
Precursor m/z : 667.3162
Fit (%) 21.1% **RFit (%)** 100.0%

Exp RT: 17.62 minutes
Analyte Name:
667.3163 / 17.62

Collision Energy = 35 ± 15 eV

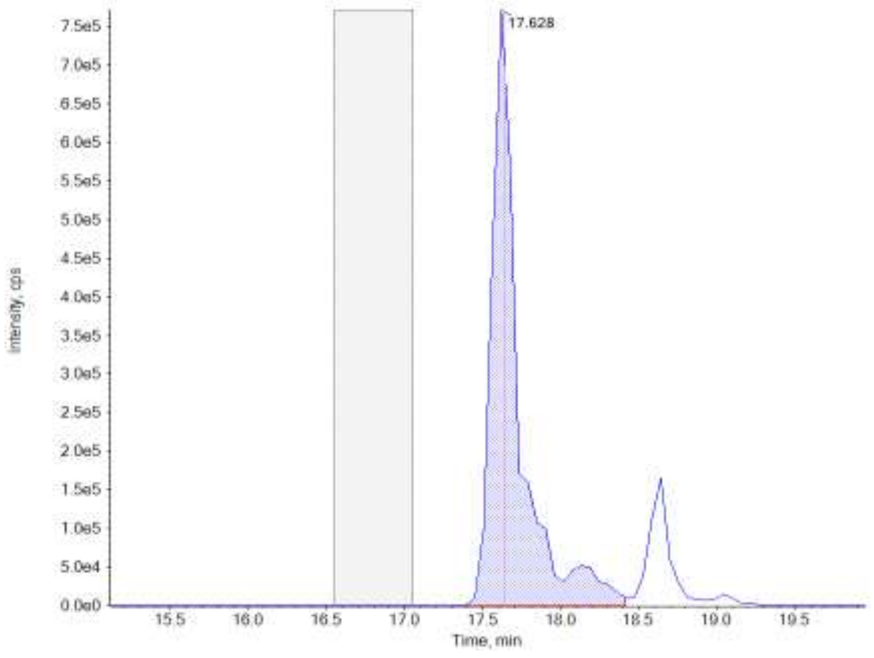

Acquired / Library MSMS

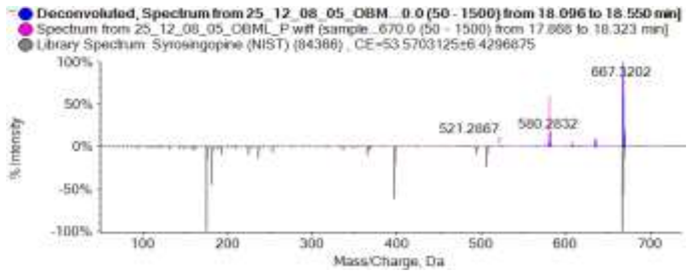

Acquired / Theoretical MS

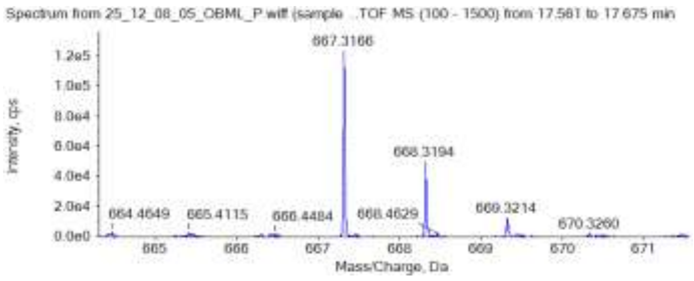

615.4079 / 17.68 (Mass/FragMass/RT/Isotope/Library/Formula/Ion Ratio)

**Retention Time:** 17.67 minutes	**Exp RT:** 17.68 minutes
**Precursor m/z :** 615.4079	**Analyte Name:**
**Fit (%)** 100.0% **RFit (%)** 73.8%	615.4079 / 17.68

Collision Energy = 35 ± 15 eV

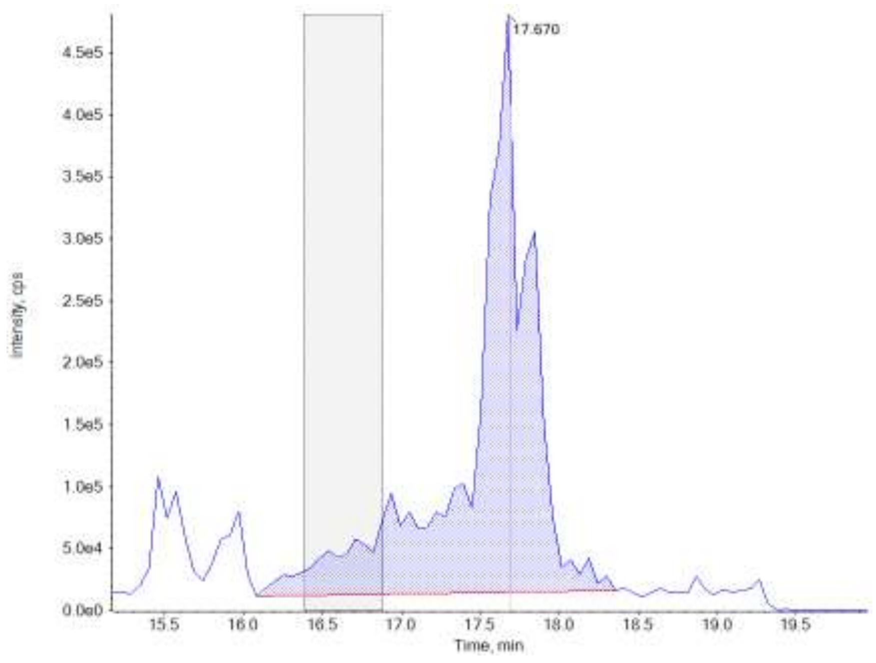

Acquired / Library MSMS

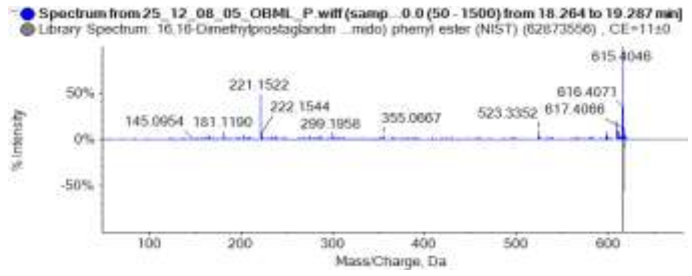

Acquired / Theoretical MS

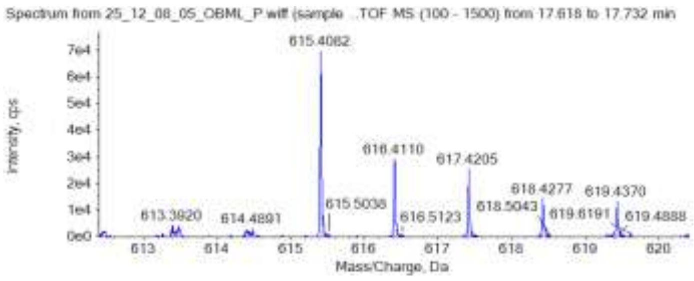

623.2557 / 17.68

(Mass/FragMass/RT/Isotope/Library/Formula/Ion Ratio)

Retention Time: 17.58 minutes
Precursor m/z : 623.2557
Fit (%) 30.4% **RFit (%)** 100.0%

Exp RT: 17.68 minutes
Analyte Name:
623.2557 / 17.68

Collision Energy = 35 ± 15 eV

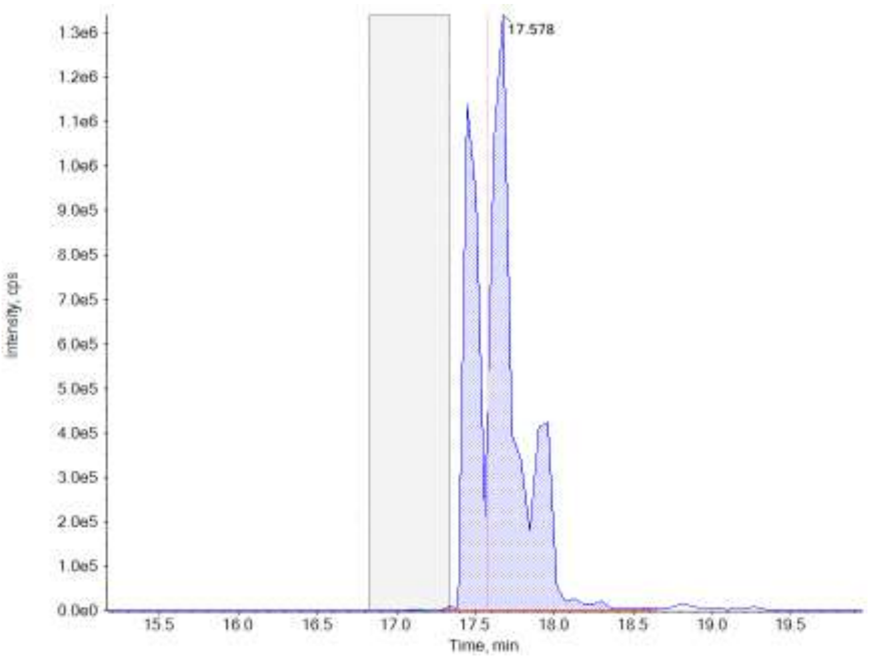

Acquired / Library MSMS

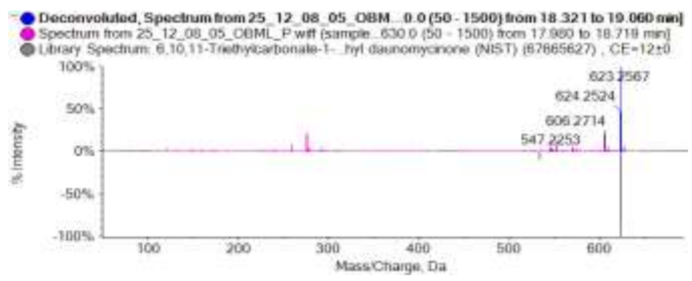

Acquired / Theoretical MS

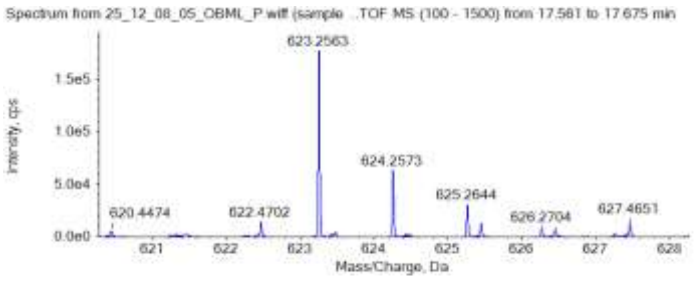

375.2739 / 17.73 (Mass/FragMass/RT/Isotope/Library/Formula/Ion Ratio)

Retention Time: 17.73 minutes
Precursor m/z : 375.2739
Fit (%) 89.5% **RFit (%)** 89.0%

Exp RT: 17.73 minutes
Analyte Name:
375.2739 / 17.73

Collision Energy = 35 ± 15 eV

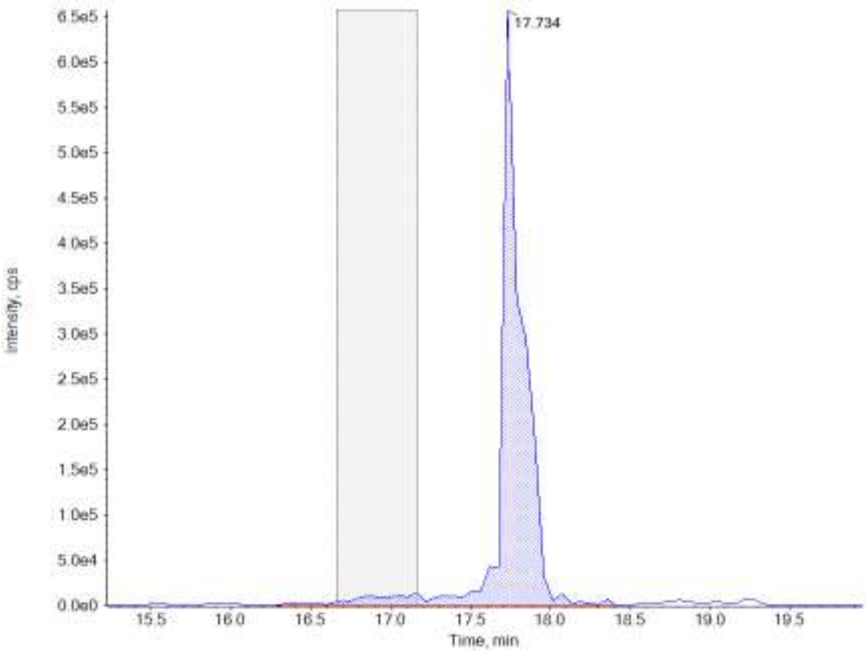

Acquired / Library MSMS

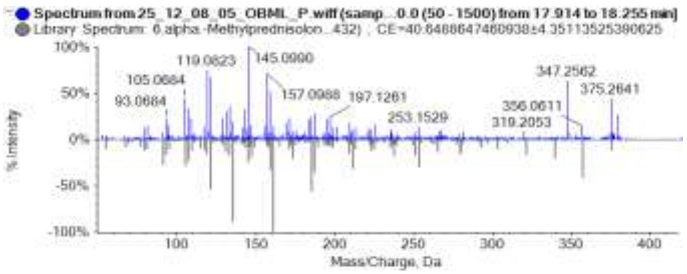

Acquired / Theoretical MS

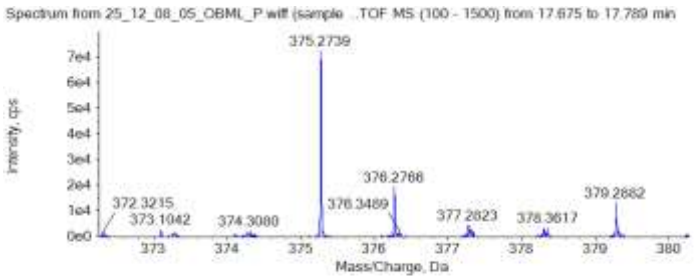

280.2434 / 17.79 (Mass/FragMass/RT/Isotope/Library/Formula/Ion Ratio)

**Retention Time:** 17.82 minutes<br>**Precursor m/z :** 280.2434<br>**Fit (%)** 57.7% **RFit (%)** 77.4%	**Exp RT:** 17.79 minutes<br>**Analyte Name:**<br>280.2434 / 17.79

Collision Energy = 35 ± 15 eV

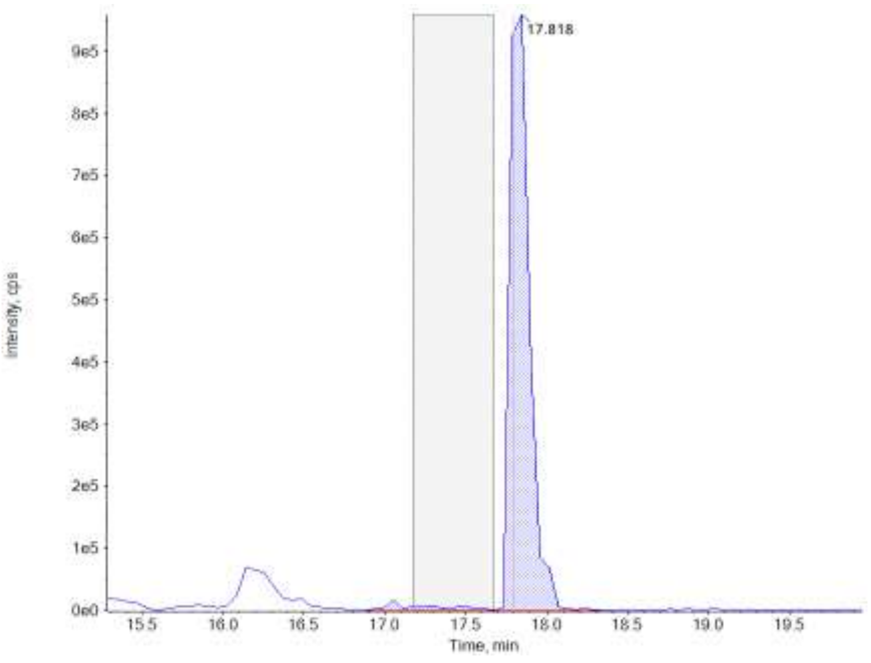

Acquired / Library MSMS

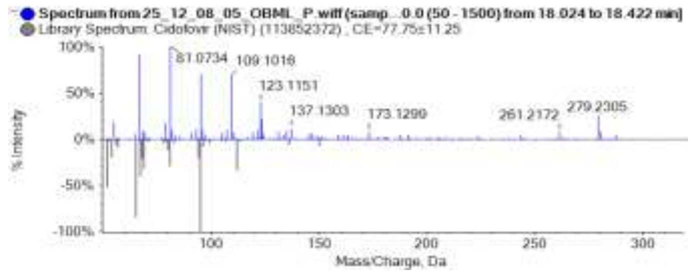

Acquired / Theoretical MS

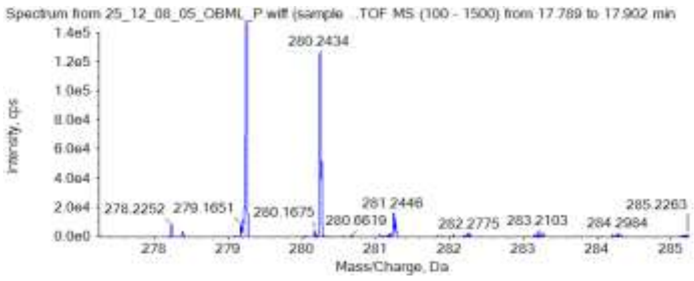

Created with SCIEX OS 3.3.0
Printed:12/9/2025 2:05:30 PM

279.2414 / 17.85 (Mass/FragMass/RT/Isotope/Library/Formula/Ion Ratio)

Retention Time: 17.82 minutes
Precursor m/z : 279.2414
Fit (%) 85.9% **RFit (%)** 91.6%

Exp RT: 17.85 minutes
Analyte Name:
279.2414 / 17.85

Collision Energy = 35 ± 15 eV

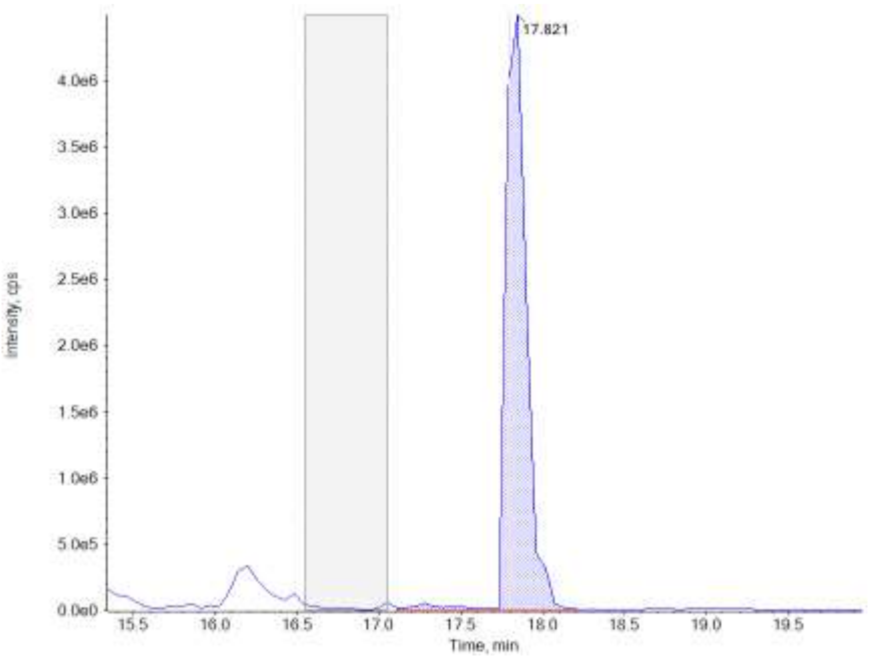

Acquired / Library MSMS

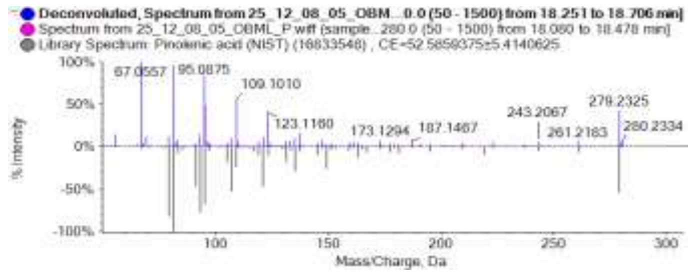

Acquired / Theoretical MS

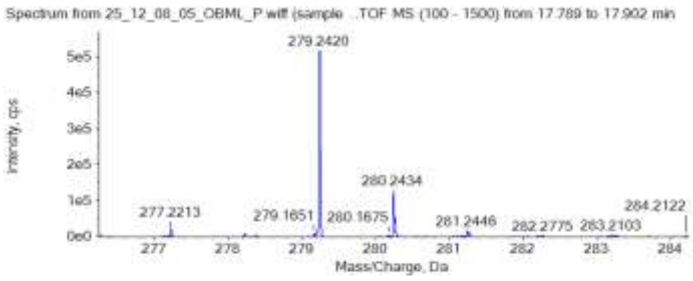

Created with SCIEX OS 3.3.0
Printed:12/9/2025 2:05:30 PM

734.5957 / 17.85 (Mass/FragMass/RT/Isotope/Library/Formula/Ion Ratio)

Retention Time: 17.83 minutes
Precursor m/z : 734.5957
Fit (%) 68.3% **RFit (%)** 96.6%

Exp RT: 17.85 minutes
Analyte Name:
734.5957 / 17.85

Collision Energy = 35 ± 15 eV

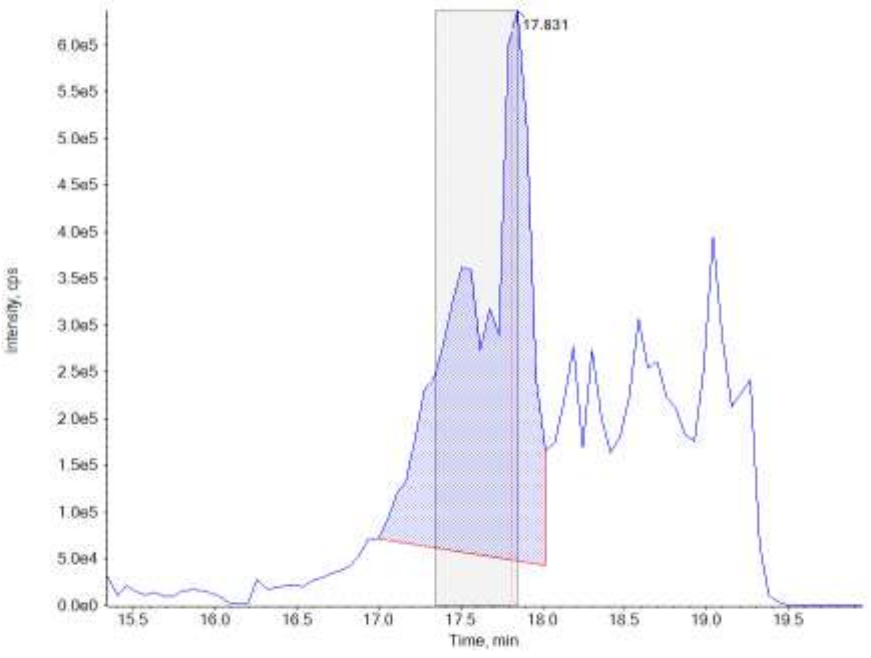

Acquired / Library MSMS

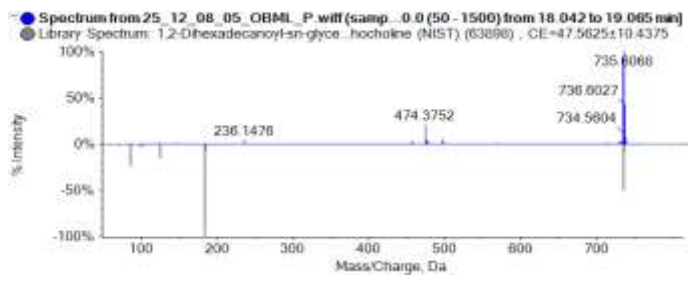

Acquired / Theoretical MS

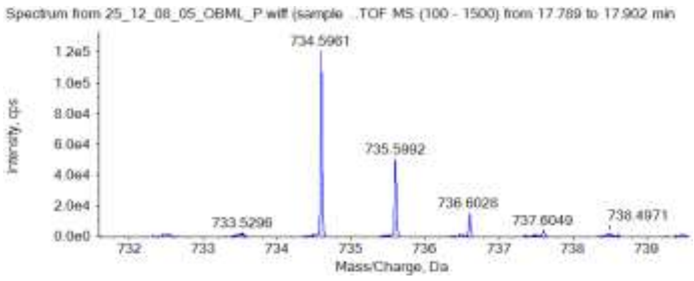

583.4185 / 18.19 (Mass/FragMass/RT/Isotope/Library/Formula/Ion Ratio)

Retention Time: 18.17 minutes **Exp RT:** 18.19 minutes
Precursor m/z : 583.4185 **Analyte Name:**
Fit (%) 99.9% **RFit (%)** 91.0% 583.4185 / 18.19

Collision Energy = 35 ± 15 eV

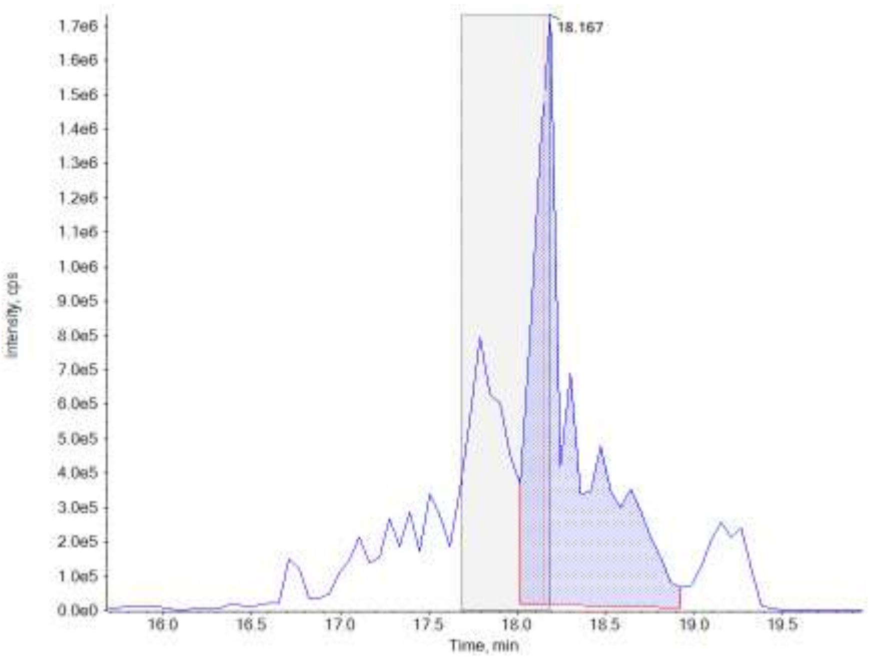

Acquired / Library MSMS

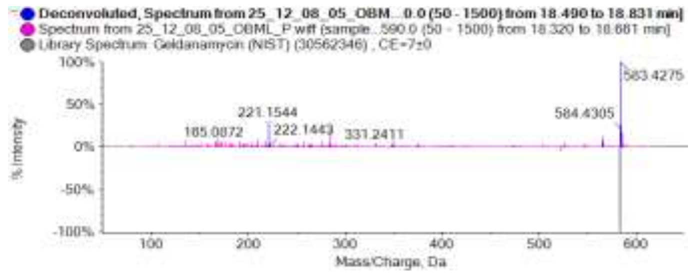

Acquired / Theoretical MS

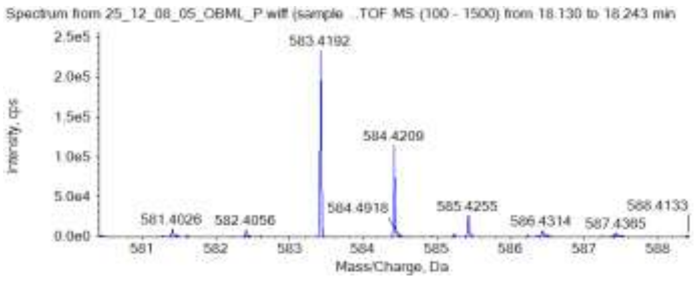

419.3381 / 18.36 (Mass/FragMass/RT/Isotope/Library/Formula/Ion Ratio)

Retention Time: 18.36 minutes
Precursor m/z : 419.3381
Fit (%) 82.6% **RFit (%)** 91.2%

Exp RT: 18.36 minutes
Analyte Name:
419.3381 / 18.36

Collision Energy = 35 ± 15 eV

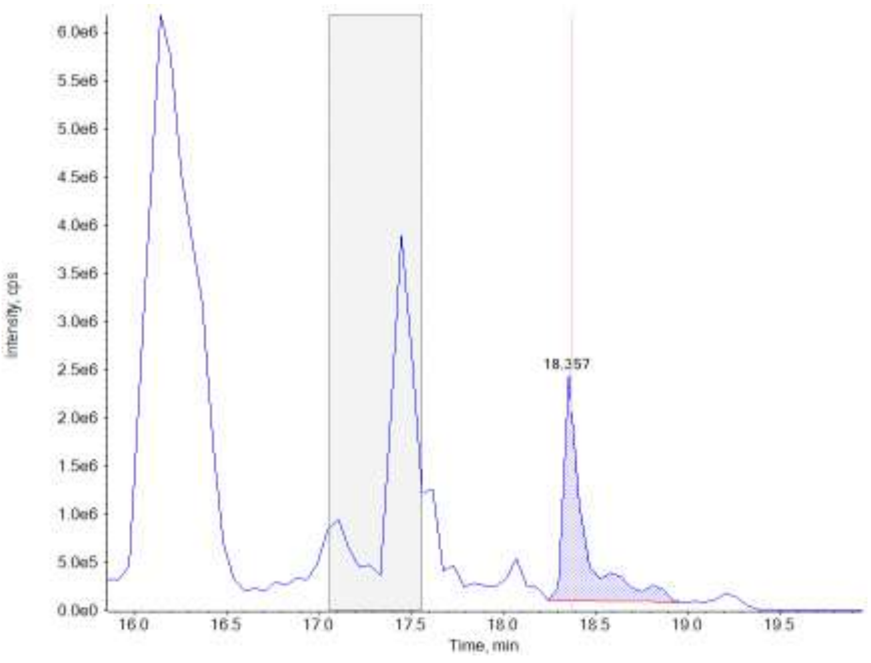

Acquired / Library MSMS

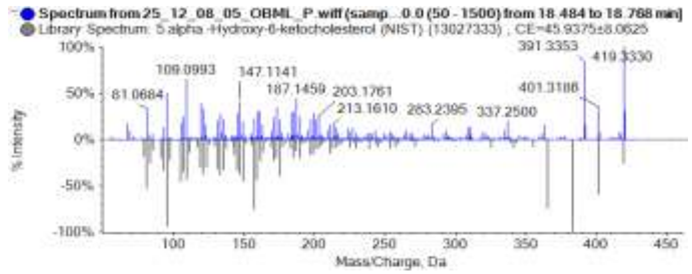

Acquired / Theoretical MS

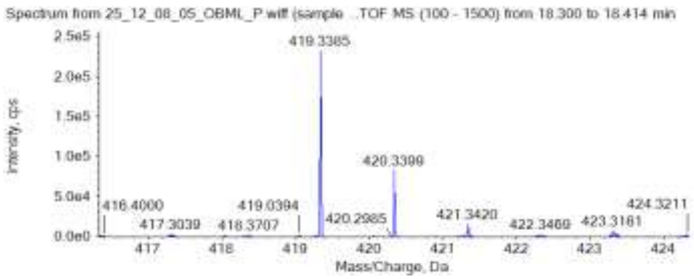

439.3618 / 18.53

(Mass/FragMass/RT/Isotope/Library/Formula/Ion Ratio)

**Retention Time:** 18.54 minutes	**Exp RT:** 18.53 minutes
**Precursor m/z :** 439.3618	**Analyte Name:**
**Fit (%)** 97.5% **RFit (%)** 97.2%	439.3618 / 18.53

Collision Energy = 35 ± 15 eV

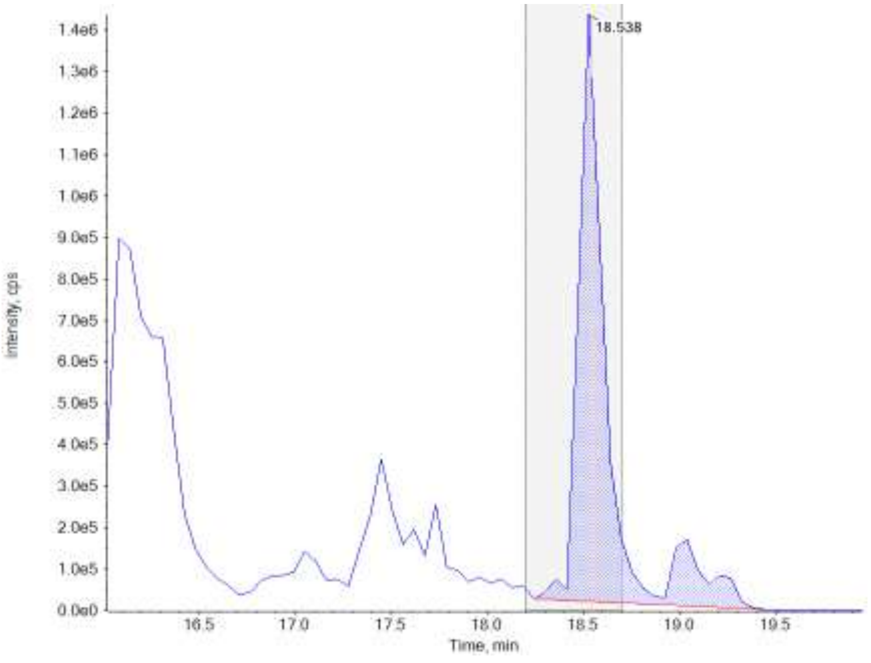

Acquired / Library MSMS

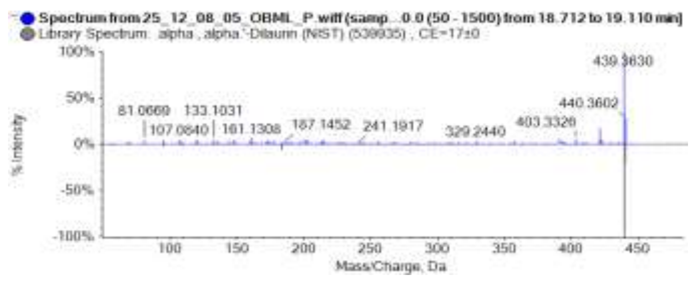

Acquired / Theoretical MS

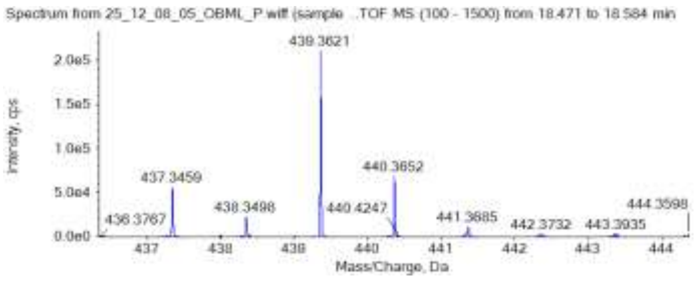

429.3781 / 18.70 (Mass/FragMass/RT/Isotope/Library/Formula/Ion Ratio)

Retention Time: 18.68 minutes **Exp RT:** 18.70 minutes
Precursor m/z : 429.3781 **Analyte Name:**
Fit (%) 95.0% **RFit (%)** 100.0% 429.3781 / 18.70

Collision Energy = 35 ± 15 eV

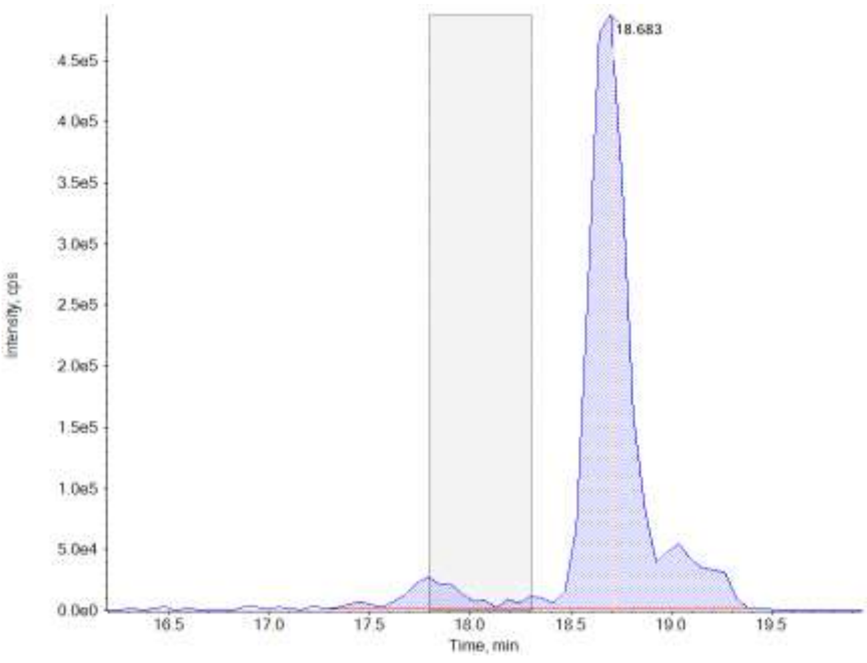

Acquired / Library MSMS

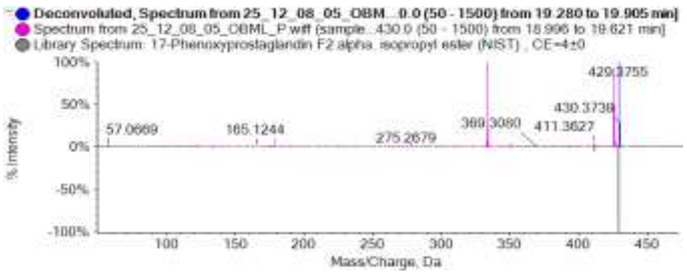

Acquired / Theoretical MS

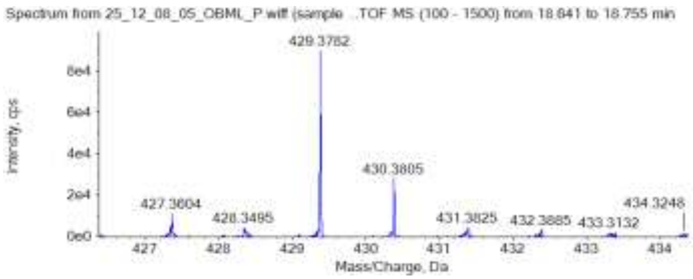

344.3376 / 18.81 (Mass/FragMass/RT/Isotope/Library/Formula/Ion Ratio)

**Retention Time:** 18.80 minutes	**Exp RT:** 18.81 minutes
**Precursor m/z :** 344.3376	**Analyte Name:**
**Fit (%)** 72.9% **RFit (%)** 100.0%	344.3376 / 18.81

Collision Energy = 35 ± 15 eV

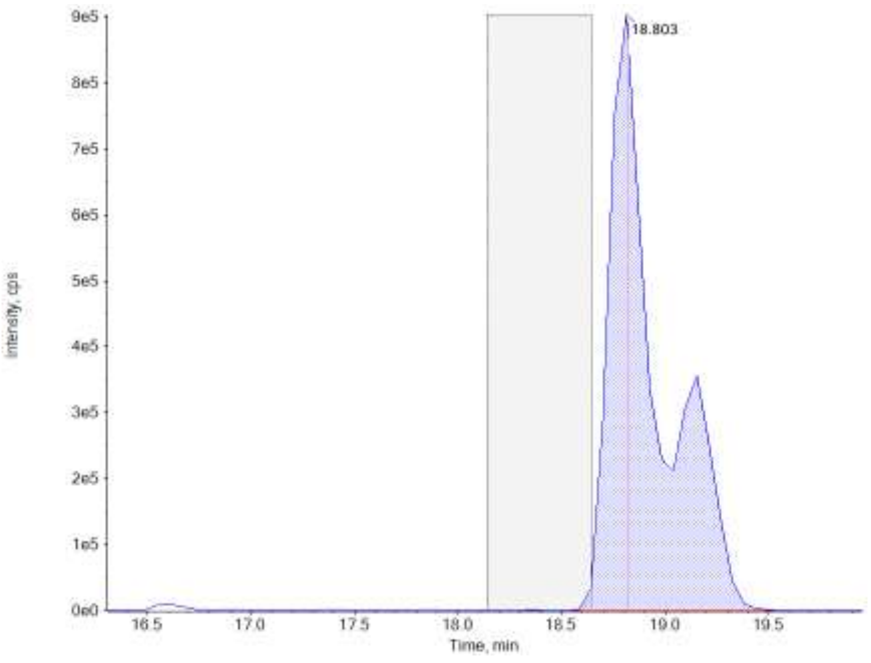

Acquired / Library MSMS

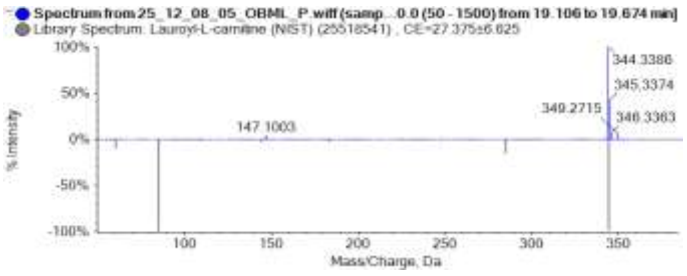

Acquired / Theoretical MS

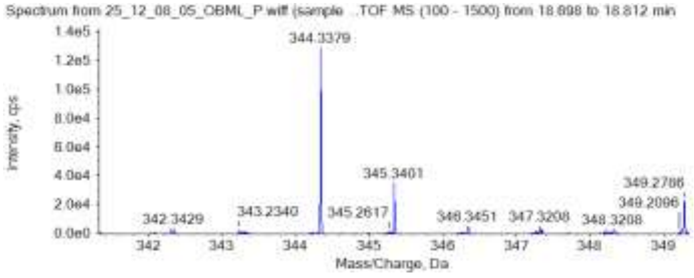

293.2537 / 18.93 (Mass/FragMass/RT/Isotope/Library/Formula/Ion Ratio)

Retention Time: 18.93 minutes
Precursor m/z : 293.2537
Fit (%) 96.8% **RFit (%)** 94.0%

Exp RT: 18.93 minutes
Analyte Name:
293.2537 / 18.93

Collision Energy = 35 ± 15 eV

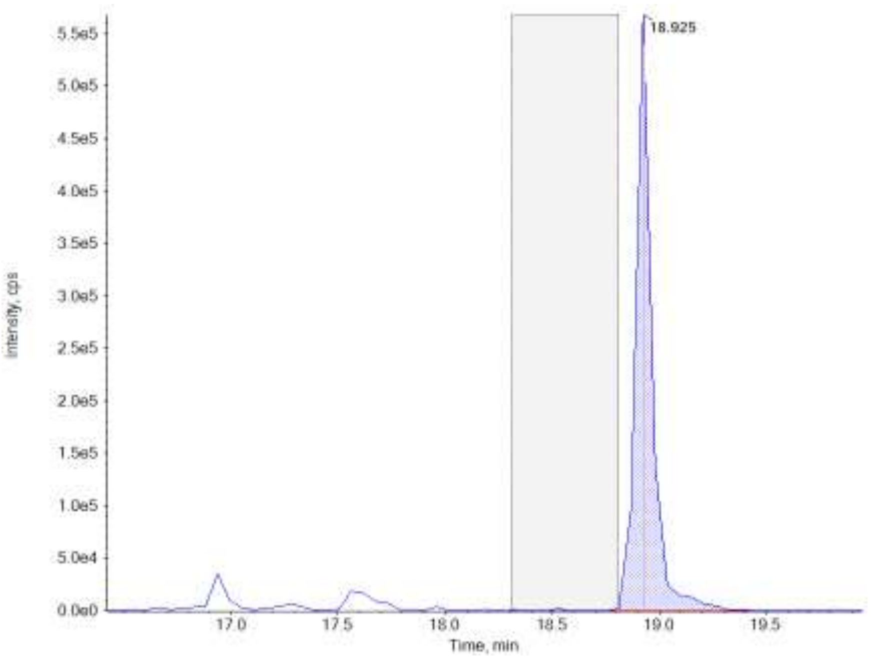

Acquired / Library MSMS

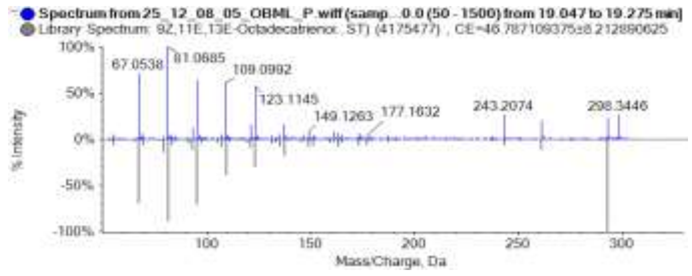

Acquired / Theoretical MS

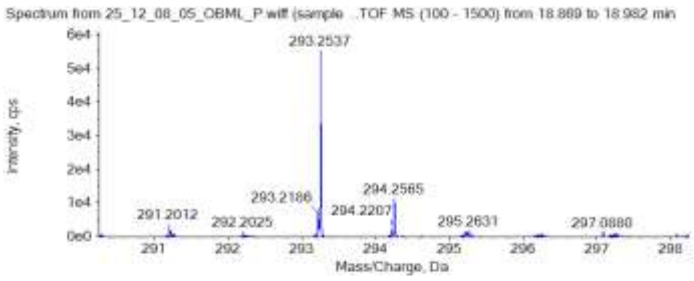

277.2237 / 19.04 (Mass/FragMass/RT/Isotope/Library/Formula/Ion Ratio)

**Retention Time:** 19.05 minutes	**Exp RT:** 19.04 minutes
**Precursor m/z :** 277.2237	**Analyte Name:**
**Fit (%)** 98.8% **RFit (%)** 99.1%	277.2237 / 19.04

Collision Energy = 35 ± 15 eV

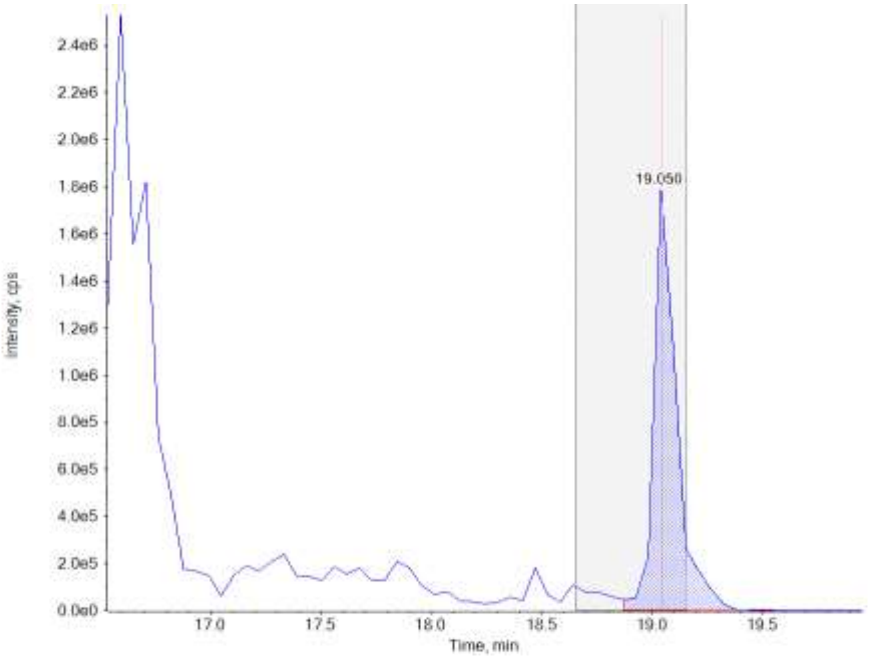

Acquired / Library MSMS

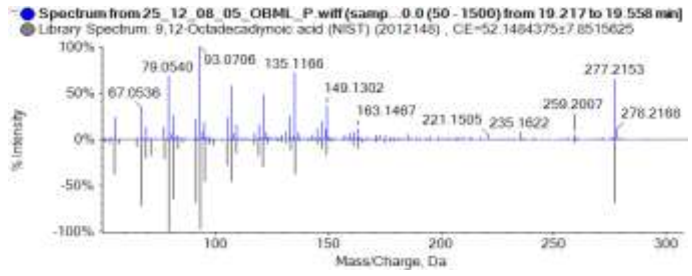

Acquired / Theoretical MS

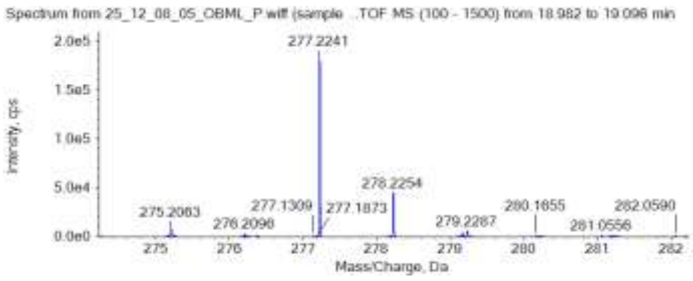

553.4305 / 19.04 [M+NH4]+ (Mass/FragMass/RT/Isotope/Library/Formula/Ion Ratio)

Retention Time: 19.05 minutes
Precursor m/z : 553.4305
Fit (%) 92.9% **RFit (%)** 85.8%

Exp RT: 19.04 minutes
Analyte Name:
553.4305 / 19.04 [M+NH4]+

Collision Energy = 35 ± 15 eV

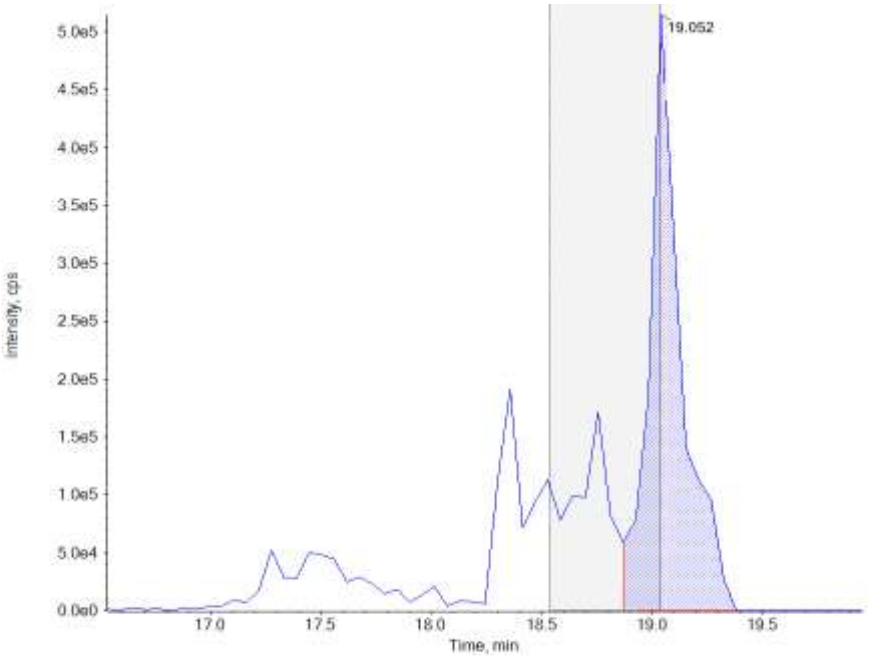

Acquired / Library MSMS

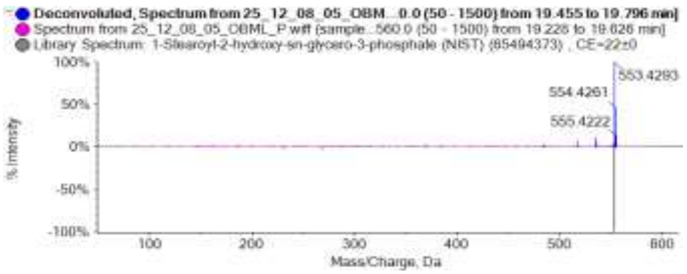

Acquired / Theoretical MS

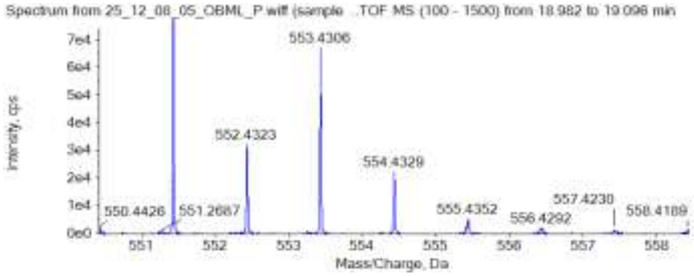

309.2898 / 19.10

(Mass/FragMass/RT/Isotope/Library/Formula/Ion Ratio)

Retention Time: 19.11 minutes
Precursor m/z : 309.2898
Fit (%) 88.1% **RFit (%)** 96.1%

Exp RT: 19.10 minutes
Analyte Name:
309.2898 / 19.10

Collision Energy = 35 ± 15 eV

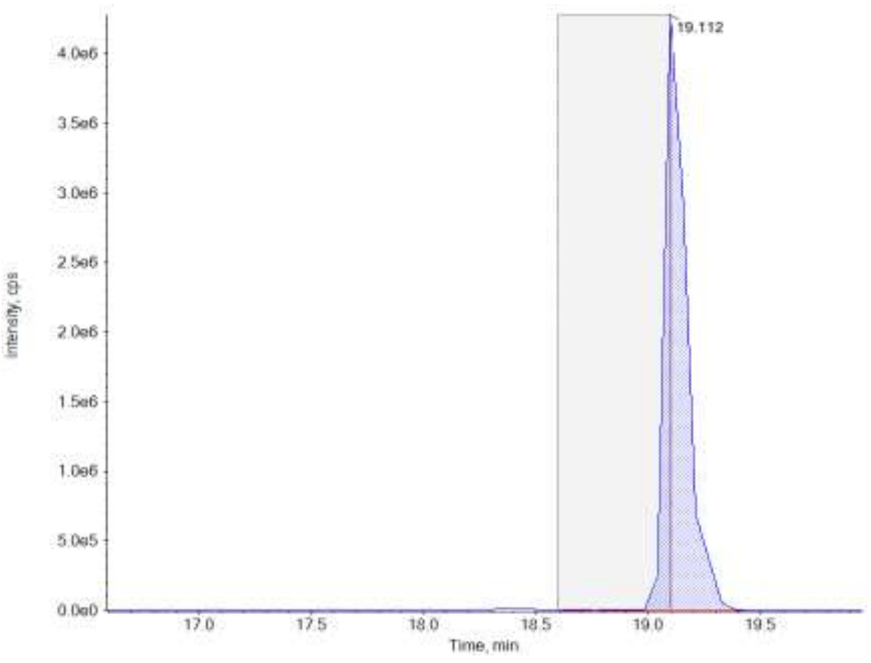

Acquired / Library MSMS

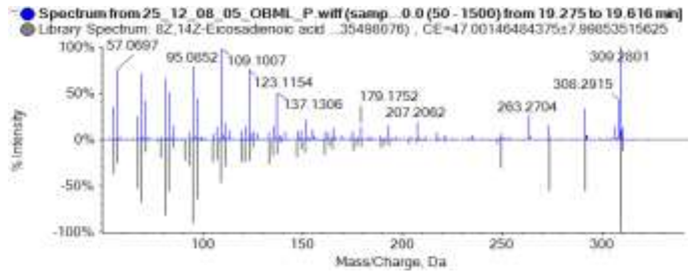

Acquired / Theoretical MS

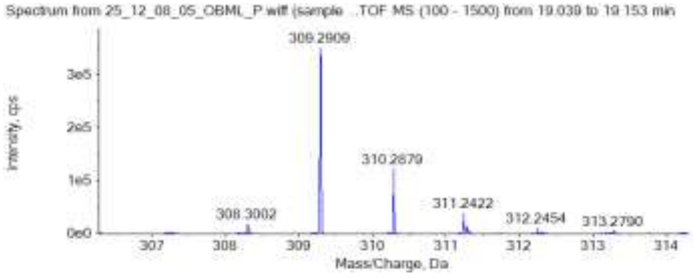

Created with SCIEX OS 3.3.0
Printed:12/9/2025 2:05:30 PM

351.2953 / 19.10 (Mass/FragMass/RT/Isotope/Library/Formula/Ion Ratio)

Retention Time: 19.10 minutes
Precursor m/z : 351.2953
Fit (%) 100.0% **RFit (%)** 100.0%

Exp RT: 19.10 minutes
Analyte Name:
351.2953 / 19.10

Collision Energy = 35 ± 15 eV

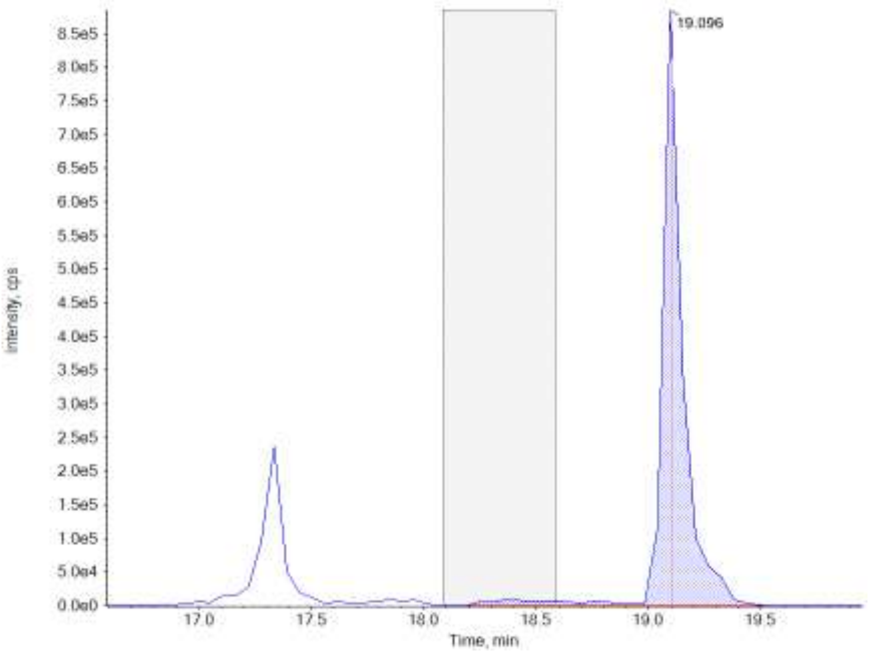

Acquired / Library MSMS

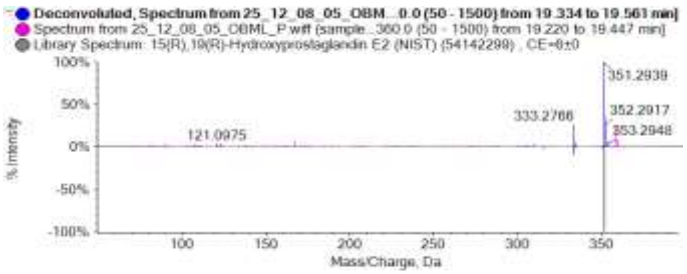

Acquired / Theoretical MS

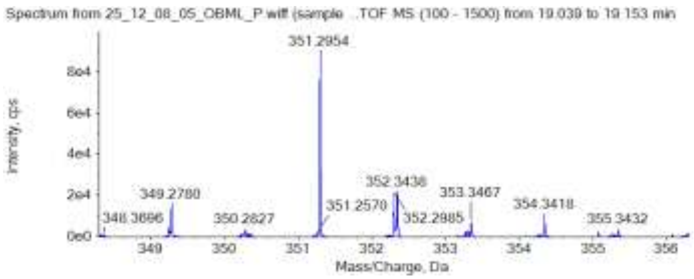

567.4237 / 19.10 (Mass/FragMass/RT/Isotope/Library/Formula/Ion Ratio)

Retention Time: 19.13 minutes
Precursor m/z : 567.4237
Fit (%) 77.5% **RFit (%)** 65.0%

Exp RT: 19.10 minutes
Analyte Name:
567.4237 / 19.10

Collision Energy = 35 ± 15 eV

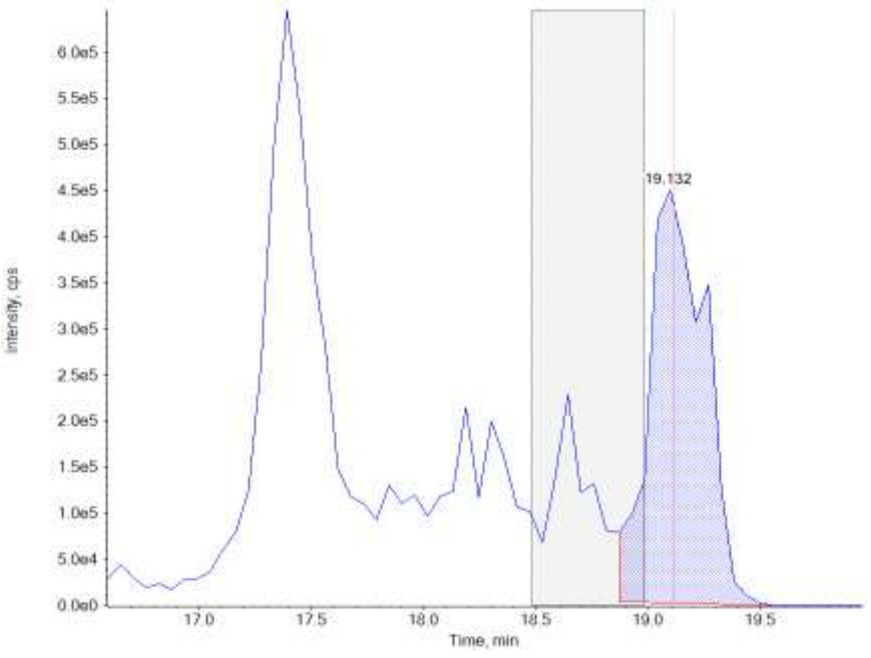

Acquired / Library MSMS

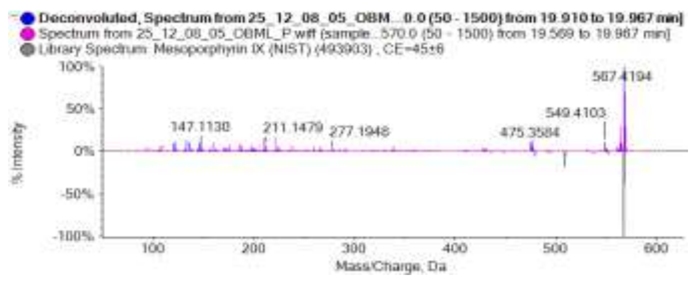

Acquired / Theoretical MS

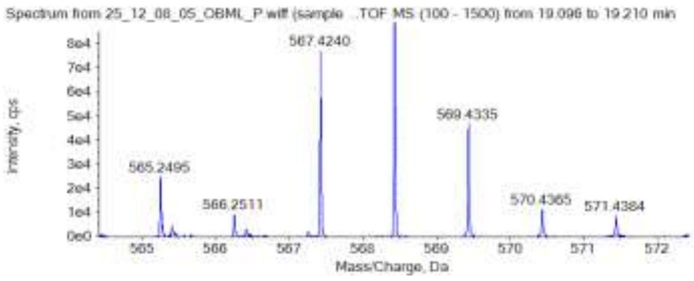

311.2415 / 19.15 (Mass/FragMass/RT/Isotope/Library/Formula/Ion Ratio)

**Retention Time:** 19.16 minutes	**Exp RT:** 19.15 minutes
**Precursor m/z :** 311.2415	**Analyte Name:**
**Fit (%)** 73.8% **RFit (%)** 98.1%	311.2415 / 19.15

Collision Energy = 35 ± 15 eV

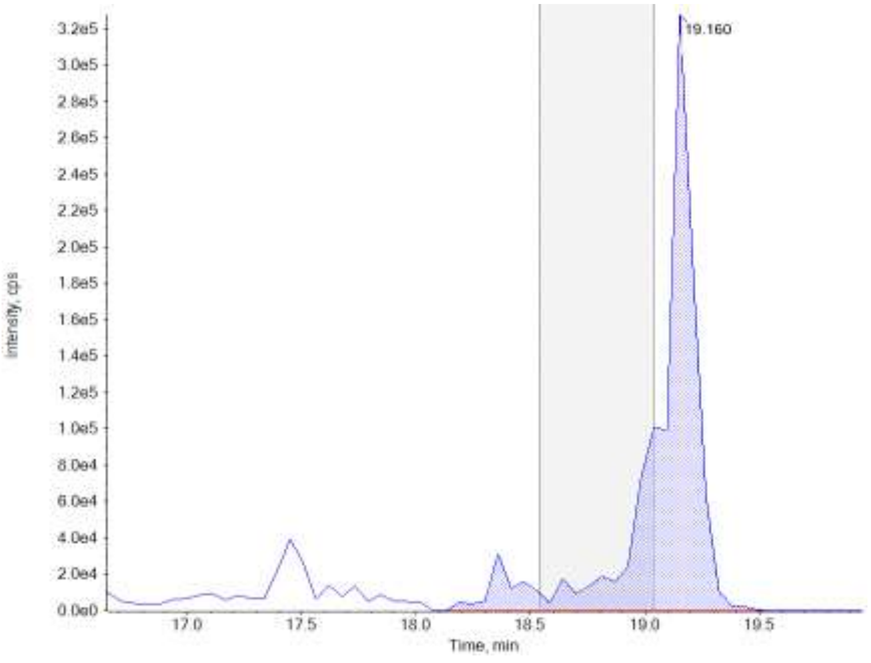

Acquired / Library MSMS

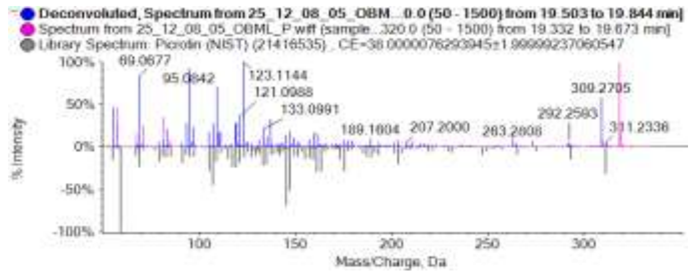

Acquired / Theoretical MS

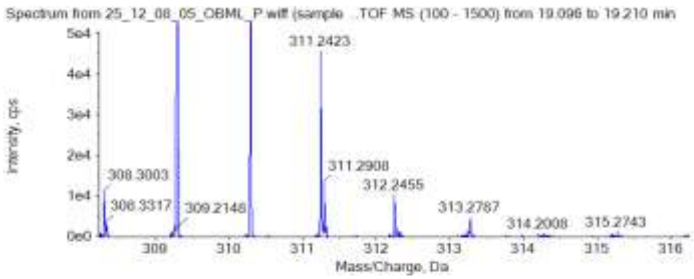

565.2491 / 19.21 (Mass/FragMass/RT/Isotope/Library/Formula/Ion Ratio)

Retention Time: 19.23 minutes **Exp RT:** 19.21 minutes
Precursor m/z : 565.2491 **Analyte Name:**
Fit (%) 100.0% **RFit (%)** 80.8% 565.2491 / 19.21

Collision Energy = 35 ± 15 eV

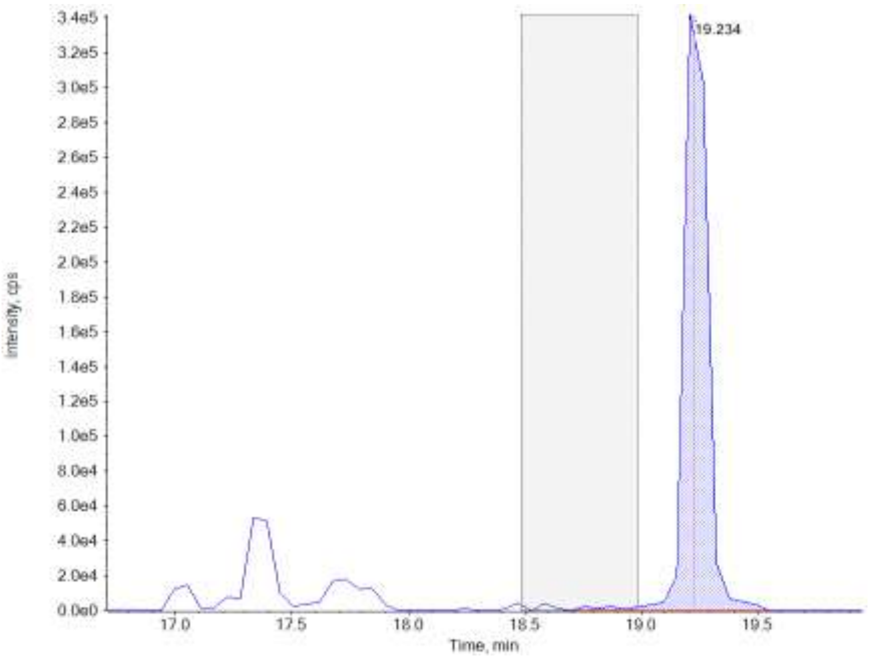

Acquired / Library MSMS

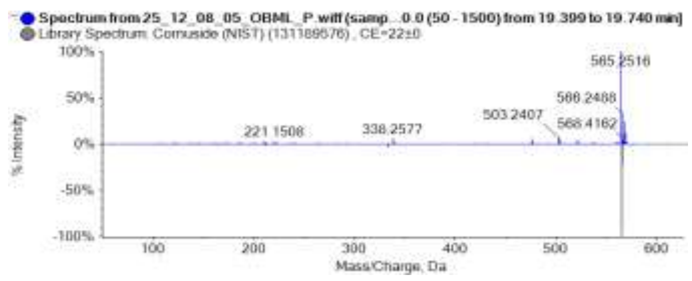

Acquired / Theoretical MS

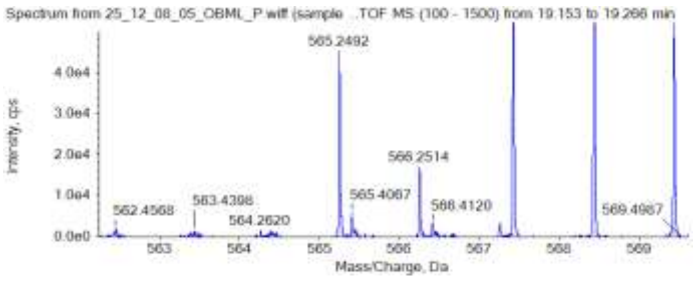

Created with SCIEX OS 3.3.0
Printed:12/9/2025 2:05:30 PM

535.2756 / 19.32 (Mass/FragMass/RT/Isotope/Library/Formula/Ion Ratio)

Retention Time: 19.32 minutes
Precursor m/z : 535.2756
Fit (%) 80.5% **RFit (%)** 100.0%

Exp RT: 19.32 minutes
Analyte Name:
535.2756 / 19.32

Collision Energy = 35 ± 15 eV

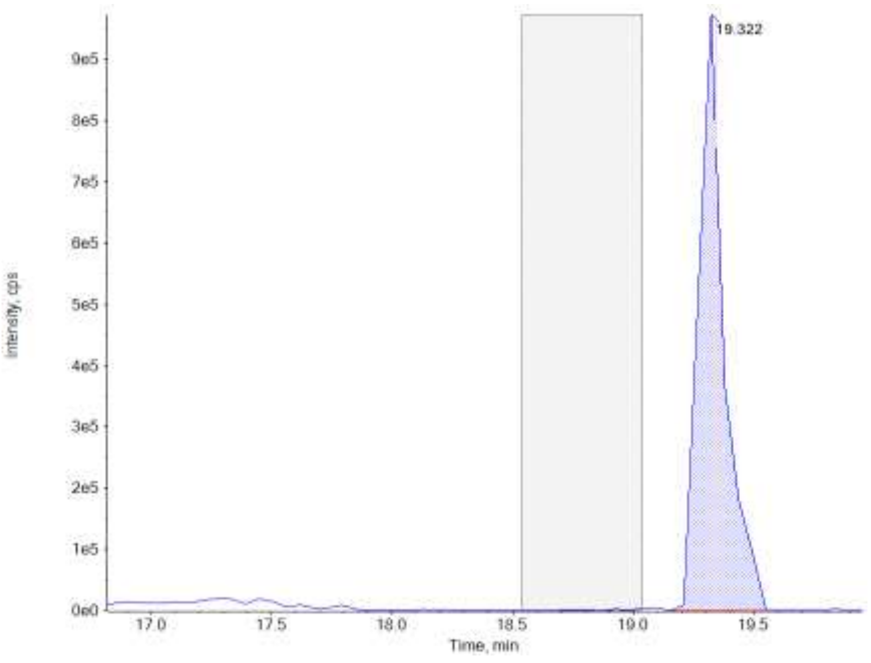

Acquired / Library MSMS

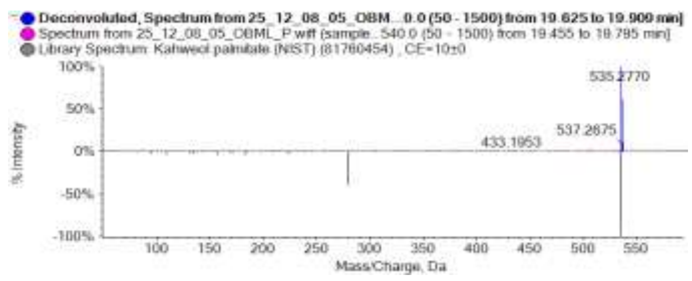

Acquired / Theoretical MS

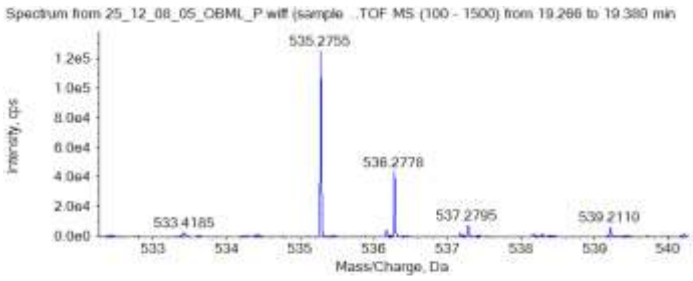

SCIEX

Created with SCIEX OS 3.3.0
Printed:12/9/2025 2:05:30 PM

584.4255 / 19.32 (Mass/FragMass/RT/Isotope/Library/Formula/Ion Ratio)

Retention Time: 19.31 minutes
Precursor m/z : 584.4255
Fit (%) 80.5% **RFit (%)** 99.6%

Exp RT: 19.32 minutes
Analyte Name:
584.4255 / 19.32

Collision Energy = 35 ± 15 eV

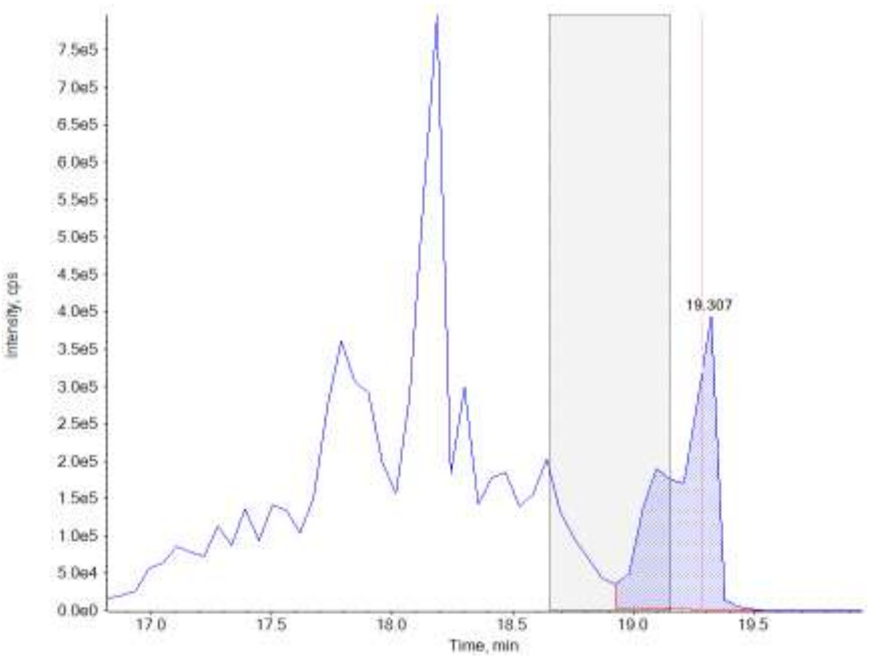

Acquired / Library MSMS

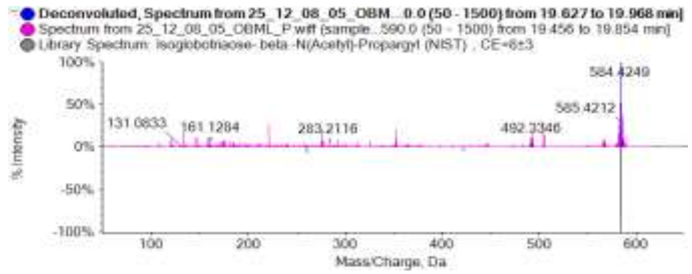

Acquired / Theoretical MS

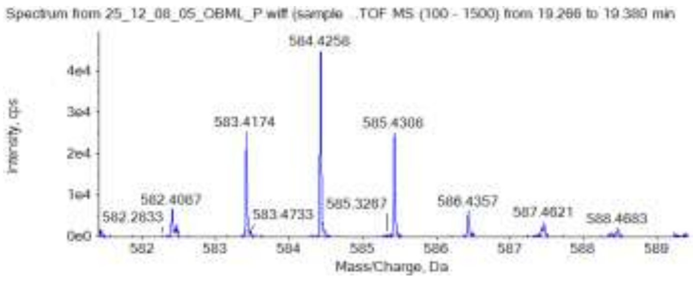

Supplement: Supplementary file 1 [file ijms-27-04945-s001.zip › 2.IJMS-4262115 Supplementary Data OBMLE-positive mode.pdf]
