# Supplementary material for: Box–Behnken Design Optimization of High-Pressure Processed Bitter Melon (Momordica charantia) Leaf Extract Enhancing Phytochemicals, Anticancer, and Anti-Inflammatory Activities
Source: Int J Mol Sci. 2026 May 29;27(11):4945. doi: 10.3390/ijms27114945 (PMC13256669; doi:10.3390/ijms27114945)
Supplement: Supplementary file 1 [file ijms-27-04945-s001.zip › 2.IJMS-4262115-supplementary.pdf]

International Journal of *Molecular Sciences*

MDPI

Supplementary Materials

Box–Behnken Design Optimization of High-Pressure Processed Bitter Melon (*Momordica charantia*) Leaf Extract Enhancing Phytochemicals, Anticancer, and Anti-Inflammatory Activities

Kongsakon Kulchim [1,2], Sukan Braspaiboon [3], Pornsiri Pitchakarn [2], Arisa Imsumran [2], Pensiri Buacheen [2], Tanongsak Laowanitwattana [2], Piya Temviriyanukul [4], Kongthawat Chairatvit [5] and Ariyaphong Wongnoppavich [2,*]

[1] Graduate/ Ph.D. Program in Biochemistry, Faculty of Medicine, Chiang Mai University, Chiang Mai 50200, Thailand; kongsakon_kulchim@cmu.ac.th (K.K.)
[2] Department of Biochemistry, Faculty of Medicine, Chiang Mai University, Chiang Mai 50200, Thailand; pornsiri.p@cmu.ac.th (P.P.); arisa.bonness@cmu.ac.th (A.I.); pensiri.bua@cmu.ac.th (P.B.); tanongsak.l@cmu.ac.th (T.L.)
[3] Faculty of Agro-Industry, Chiang Mai University, Chiang Mai 50100, Thailand; sukan.bras@cmu.ac.th (S.B.)
[4] Institute of Nutrition, Mahidol University, Salaya, Nakhon Pathom 73170, Thailand; piya.tem@mahidol.ac.th (P.T.)
[5] Department of Oral Biology, Faculty of Dentistry, Mahidol University, Bangkok 10400, Thailand; kongthawat.cha@mahidol.ac.th (K.C.)
* Correspondence: ariyaphong.w@cmu.ac.th

Supplementary Table S1. Independent Variables (Pressure (A), Time (B), Ethanol Concentration (C), and L:S Ratio (D)) and Dependent Variable (% Inhibition of A549 Cell Growth) of BMLE Derived from a Four-Factor, Three-Level BBD Consisting of 29 Experimental Runs

Run	X_1: Pressure (MPa)	X_2: Time (Min)	X_3: Ethanol concentration (%)	X_4: L:S ratio (Fold)	Response Anti-cancer (% Inhibition of A549 cell growth)
1	400	90	72.5	10	43.42±5.21
2	400	60	72.5	20	30.56±8.89
3	300	60	50	20	24.84±1.50
4	400	30	72.5	30	37.00±13.17
5	400	90	72.5	30	50.10±12.50
6	400	60	72.5	20	65.84±3.14
7	400	30	50	20	20.94±3.53
8	300	60	95	20	50.25±11.25
9	400	60	50	10	12.31±1.21
10	400	60	72.5	20	40.16±2.73
11	500	60	72.5	30	65.56±0.34
12	400	30	72.5	10	50.21±4.46
13	400	90	50	20	17.93±2.06
14	500	60	72.5	10	54.06±7.73
15	500	60	50	20	29.38±1.95

16	400	60	72.5	20	29.20±3.66
17	300	30	72.5	20	34.15±1.84
18	300	90	72.5	20	27.33±4.00
19	500	90	72.5	20	17.19±6.53
20	400	60	95	30	34.58±6.81
21	500	60	95	20	65.22±2.98
22	500	30	72.5	20	37.70±5.65
23	300	60	72.5	10	64.38±2.75
24	400	90	95	20	73.39±1.78
25	300	60	72.5	30	40.60±4.72
26	400	30	95	20	65.83±2.99
27	400	60	72.5	20	38.55±8.44
28	400	60	50	30	20.12±1.54
29	400	60	95	10	61.48±4.19

Supplementary Table S2. Analysis of Variance, Regression Coefficients, and P-value of the Second-Order Polynomial Model for % Inhibition of A549 Cell Growth, Derived from the Initial Four-Factor BBD

**Anti-cancer activity (% Inhibition of A549 cell growth)**						
**Source**	**Sum of Squares**	**DF**	**Mean Square**	**F-Value**	**p Value**	**Significant**
**Model**	5551.90	14	396.56	1.84	0.1339	
**A: Pressure**	63.31	1	63.31	0.29	0.5968	
**B: Time**	22.62	1	22.62	0.10	0.7510	
**C: %EtOH**	4226.68	1	4226.68	19.57	0.0006	***
**D: L:S ratio**	119.76	1	119.76	0.55	0.4688	
**A^2**	21.47	1	21.47	0.099	0.7572	
**B^2**	44.38	1	44.38	0.21	0.6573	
**C^2**	39.85	1	39.85	0.18	0.6741	
**D^2**	144.24	1	144.24	0.67	0.4275	
**AB**	46.89	1	46.89	0.22	0.6484	
**AC**	27.16	1	27.16	0.13	0.7282	
**AD**	311.31	1	311.31	1.44	0.2498	
**BC**	27.94	1	27.94	0.13	0.7244	
**BD**	98.86	1	98.86	0.46	0.5097	
**CD**	301.11	1	301.11	1.39	0.2574	
**Residual**	3023.88	14	215.99			
**Lack of Fit**	2151.91	10	215.19	0.99	0.5543	NS

**Pure Error**	871.97	4	217.99		
**Cor Total**	8575.78	28			
**R^2**					0.6474
**Adjusted R^2**					0.2948

Differences in response were evaluated using ANOVA at a 95% confidence level. Statistical analyses were conducted using Design-Expert software (version 13) . ***p<0.001, and NS: not statistically significant.

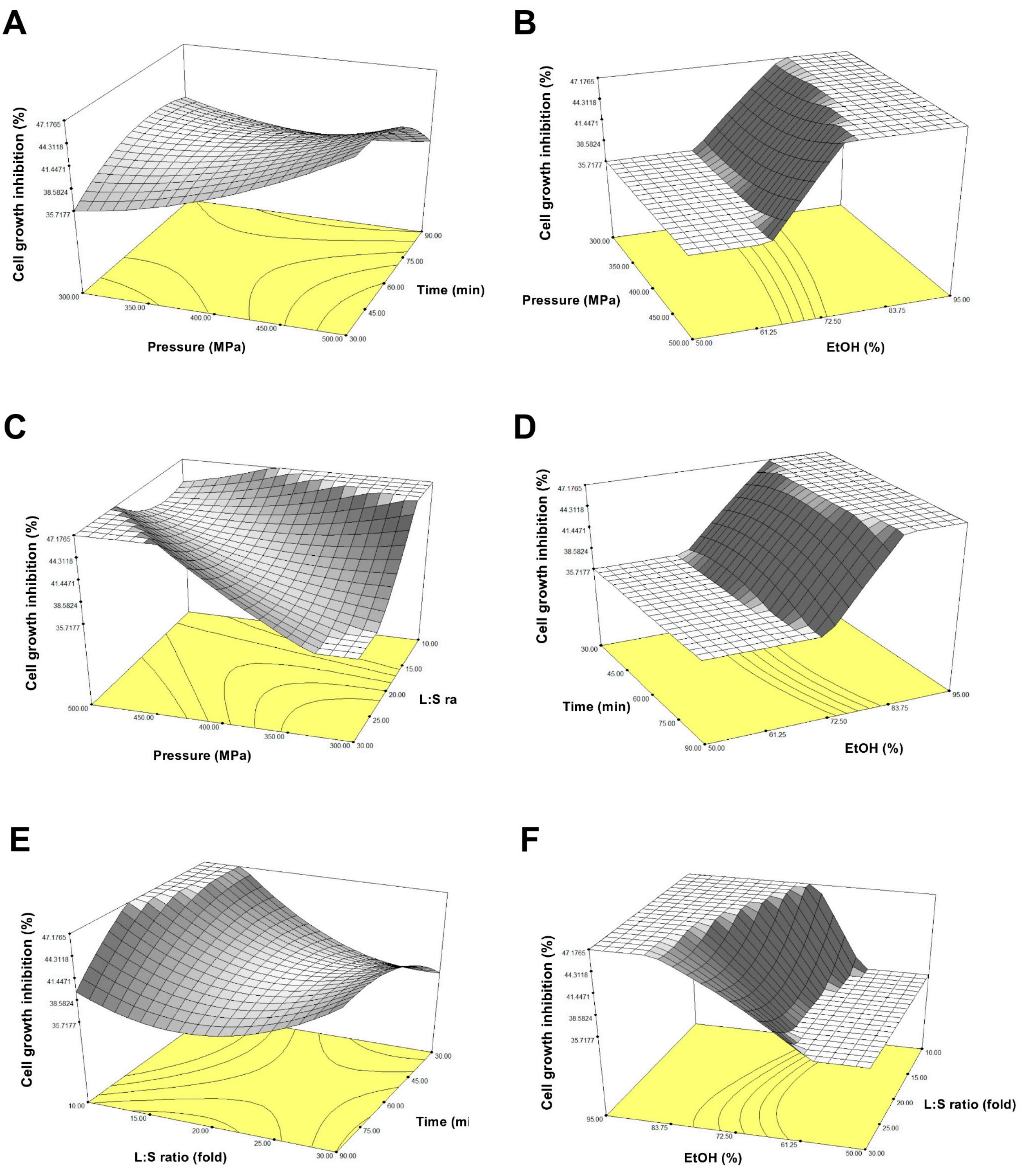

Supplementary Figure S1. Contour Plots Illustrate How HPP Parameters Interactively Influence Biological Activities of BMLE during BBD Optimization. (A-F) Effects on A549 lung cancer cell growth inhibition (%). Independent variables: pressure (A; 300-500 MPa), extraction time (B; 30-90 min), ethanol concentration (C; 50-95%), and L:S ratio (D; 10-30-fold)

Supplementary Table S3. Metabolite Profiling of O-BMLE in Positive Ionization mode, Showing Compounds with a Library Score of ≥90%, Ranked from Highest to Lowest Relative Peak Area, as Determined by HPLC-qTOF-MS

Rank	RT (min)	Mode	Tentatively Identified Metabolites	Precursor Mass	Library Score (%)	Relative Area (%)
1	15.37	M+	Stearidonic acid	277.2315	96.4	27.8278
2	17.25	M+	Erucamide	338.351	90.7	14.5643
3	17.38	M+	1-Stearoyl-rac-glycerol	359.3232	93.2	6.7656
4	15.75	M+	Monolinolenin	353.2836	96.8	6.1701
5	10.37	M+	9-Oxo-octadecatrienoic acid	293.2236	91.4	5.8844
6	16.63	M+	6-Gingerol	277.2247	98.1	5.6880
7	15.33	M+	17-Trifluoromethyl Phenyl Trinor Prostaglandin F2α	439.3624	100	3.7801
8	18.17	M+	Geldanamycin	583.4192	90.8	3.4306
9	1.07	M+	Choline	104.116	96.6	3.1080
10	4.46	M+	3-Indoleacrylic acid	188.0846	97.7	2.5494
11	2.28	M+	Phenylalanine	166.0962	99.8	2.4410
12	1.59	M+	Isoleucine	132.1122	100	2.1183
13	18.54	M+	α,α′-Dilaurin	439.3621	93.3	1.8849
14	19.05	M+	9,12-Octadecadiynoic acid	277.2241	97.7	1.6235
15	15.90	M+	Pinoresinol	341.3111	90.6	1.2218
16	16.91	M+	Leiocarposide	653.3009	98.6	1.0282
17	18.68	M+	17-Phenoxy Prostaglandin F2α isopropyl ester	429.3782	95	1.0186
18	4.46	M+	L-tryptophan	205.1046	97.9	0.9142
19	13.47	M+	Lovastatin hydroxy acid	405.3575	97.7	0.7368
20	19.10	M+	15(R),19(R)-Hydroxyprostaglandin E2	351.2954	100	0.7093
21	12.01	M+	4-Methoxytriphenylmethyl cation	273.1905	100	0.6352
22	1.82	M+	2'-O-Methyladenosine	282.1292	96.8	0.6258
23	1.50	[M+H]+	Guanosine	284.1068	100	0.5925
24	15.69	M+	5-Sulfosalicylic acid	219.18	98.5	0.4507
25	9.29	M+	Prostaglandin E2 serinolamide	426.2903	91.9	0.4184

26	2.28	M+	2-(3-Hydroxyphenyl)ethanol	121.0898	95.8	0.3881
27	16.88	M+	Ω-3 Arachidonic acid	305.2534	90.9	0.3746
28	1.55	M+	Nizatidine	332.1165	90.3	0.3089
29	9.89	M+	Bomyl acetate	137.1377	93.2	0.2626
30	7.90	M+	Hydrocinnamic acid ethyl ester	179.1123	97.4	0.2419
31	9.72	M+	Nigericin	747.5545	100	0.2126
32	1.48	M+	L-Tyrosine	182.0868	97.8	0.1945
33	8.13	M+	Indole-6-carboxaldehyde	146.0658	96.8	0.1811
34	7.39	M+	Octadecamethyloctasiloxane	629.321	97.1	0.1609
35	7.56	M+	Thapsigargin	673.3461	91.5	0.1433
36	8.92	M+	Bimatoprost amide	370.2073	96.8	0.1428
37	5.55	M+	Methyl deoxycholate	389.1621	100	0.1384
38	4.98	M+	Salicylic acid	139.0443	92.7	0.1307
39	7.18	[M+H]+	Trp-Trp-Arg	547.3367	96.6	0.1182
40	1.77	[M+NH4]+	DL-Homocystine	286.1458	95.4	0.1131
41	5.06	M+	Valeroyl salicylate	205.1274	96.9	0.1126
42	6.42	M+	Sweroside	359.1745	97.4	0.1119
43	5.97	M+	2-Phenylacetamide	136.0807	91.5	0.1068
44	10.28	M+	γ-Muricholic acid	391.3406	97.2	0.0944
45	2.65	M+	Cysteic acid	170.0861	95.1	0.0885
46	5.49	M+	4-Diphenylmethoxymethylpiperidine	282.1753	94.7	0.0698
47	4.46	M+	4-Chloroamphetamine	170.0651	100	0.0687
48	5.63	M+	Deacetyl asperulosidic acid methyl ester +Na	427.1774	90.8	0.0480

Supplementary Table S4. Metabolite Profiling of O-BMLE in Negative Ionization Mode, Showing Compounds with a Library Score of ≥90%, Ranked from Highest to Lowest Relative Peak Area, as Determined by HPLC-qTOF-MS

Rank	RT (min)	Mode	Tentatively Identified Metabolites	Precursor Mass	Library Score (%)	Relative Area (%)
1	2.23	M-	4-Hydroxybenzoic acid	137.028	93.9	19.5128
2	1.02	M-	D-Pyroglutamic acid	128.034	99.7	14.4272
3	1.71	M-	Adenine	134.052	93.9	10.9716
4	17.34	M-	Heptadecanoic acid	269.246	92.0	7.1793
5	1.17	M-	D-(+)-Mannose	179.055	99.2	4.0335

6	19.15	M-	1,4-D-Xylobiose	281.249	98.6	3.6177
7	1.48	[M-H]-	Asp-Asp	247.117	100	3.5699
8	17.88	M-	Pinolenic acid	277.216	92.3	2.3058
9	4.84	M-	Esculin	339.071	95.0	2.1935
10	2.81	M-	Vanillic acid	167.034	98.1	2.1831
11	17.28	M-	17-Phenyltrinor-8-iso-prostaglandin E2	365.273	91.0	2.1103
12	1.41	M-	Uridine	243.068	98.5	2.0937
13	14.19	M-	Pedunculoside +HCOOH	695.402	99.1	1.9633
14	13.30	M-	1-Hexadecanoyl-sn-glycero-3-phospho-(1'-myo-inositol)	571.293	91.0	1.8558
15	15.15	M-	Asp-Cys	235.169	95.1	1.8324
16	6.99	M-	PolygalaxanthoneⅨ	551.235	95.5	1.6937
17	9.61	[M+FA-H]-	20(R)-Ginsenoside Rh1 +HCOOH	683.403	100	1.6833
18	14.41	M-	10E,12Z-octadecadienoic acid	279.195	91.7	1.6209
19	13.38	M-	Palmitelaidic acid	253.179	100	1.4564
20	6.93	M-	Asp-Asp	247.154	100	1.4188
21	13.7	M-	Jasmonic acid	209.117	96.6	1.2500
22	9.62	M-	Eriodictyol-7-O-glucoside	449.274	100	1.2458
23	6.25	M-	Naphthofluorescein	431.201	97.5	1.1148
24	9.09	M-	O,O-Diethyl phosphate	153.09	99.4	0.9776
25	19.22	M-	2,2',4'-Trihydroxychalcone	255.237	100	0.9768
26	1.20	[M-H]-	D-(+)-Trehalose	341.108	90.3	0.9171
27	1.83	M-	2-Isopropylmalic acid	175.098	98.7	0.7119
28	1.82	M-	5-Methyluridine	257.077	100	0.7030
29	9.38	M-	Furosemide	329.16	98.7	0.5842
30	1.45	M-	1-(2,8-Dihydroxyquinolin-5-yl)ethan-1-one	202.107	92.1	0.5410
31	16.11	M-	Pseudoginsenoside-RT5 +HCOOH	699.383	94.1	0.5179
32	17.87	M-	Propanoic acid, 2-1-3-4-(1,1'-biphenyl-4-ylcarbonyl)-2-propylphenoxy propyl-1,2,3,4-tetrahydro-5-quinolinyloxy-2-methyl-	590.445	95.0	0.4375
33	11.52	M-	α-hederin +HCOOH	795.455	97.5	0.4100
34	11.03	M-	Ionomycin	707.402	96.4	0.3900
35	16.31	M-	Man7-2AA	849.505	99.2	0.3734
36	8.84	M-	Cinnabarinic acid	299.184	100	0.2474

37	1.59	M-	13,14-Dihydro-15-ketotetranorprostaglandin F1.α	299.082	95.7	0.2275
38	8.41	M-	trans-1,4-Cyclohexanedicarboxylic acid	171.102	91.7	0.2039
39	10.58	[M-H2O-H]-	N-Lauroyl-D-erythro-sphingosylphosphorylcholine	645.364	92.2	0.1656
40	8.76	M-	Lactitol	343.215	94.1	0.1379
41	9.28	[M+FA-H]-	Glucobrassicin	493.229	97.1	0.0796
42	17.35	M-	Crocin II	813.484	91.8	0.0640

Supplementary Table S5. Metabolite Profiling of BMLE in Positive Ionization Mode, Showing Compounds with a Library Score of ≥90%, Ranked from Highest to Lowest Relative Peak Area, as Determined by HPLC-qTOF-MS

Rank	RT (min)	Mode	Tentatively Identified Metabolites	Precursor Mass	Library Score (%)	Relative Area (%)
1	15.36	M+	Stearidonic acid	277.2313	97.5	16.7398
2	15.38	M+	9,12-Octadecadiynoic acid	277.228	96.1	11.4770
3	4.46	M+	3-Indoleacrylic acid	188.0848	96.8	8.2965
4	18.36	M+	1-(1,2-Dioctanoylphosphatidyl)inositol	609.2844	99.1	8.2309
5	18.57	M+	1-Palmitoylglycerol	331.2927	94.6	6.3351
6	2.28	M+	Phenylalanine	166.0964	99.8	5.0280
7	1.12	M+	Lactobionic acid	381.0883	95	4.2406
8	1.08	M+	Choline cation	104.1156	95.2	4.0818
9	4.43	M+	L-Tryptophan	205.1059	98	3.7123
10	14.76	M+	1-Palmitoyl-sn-glycero-3-phosphocholine	496.3453	97.2	3.1995
11	18.53	M+	α,α'-Dilaurin	439.3627	95.2	2.9854
12	17.47	M+	Monolinolenin (9c,12c,15c)	353.2766	96.4	2.9802
13	18.37	M+	γ-Muricholic acid	391.3418	100	2.8783
14	18.17	M+	Geldanamycin	583.4191	91.9	2.7316
15	17.38	M+	(+)-Pinoresinol	341.3108	92.8	2.5036
16	1.57	M+	Isoleucine	132.1109	100	1.8423
17	17.56	M+	Stearidonic acid ethyl ester	305.2558	97.4	1.8389
18	13.91	M+	Glu-Arg	304.2905	100	1.7629
19	17.8	M+	Phthalic anhydride	149.0284	95	1.3071
20	1.49	M+	Guanosine	284.108	100	1.2309
21	2.27	M+	2-(3-Hydroxyphenyl)ethanol	121.09	95.3	0.7609

22	15.77	M+	Benzyldimethyltetradecylammonium cation	332.3213	91.5	0.7010
23	7.39	[M+K]+	Octadecamethyloctasiloxane	629.3218	100	0.5658
24	1.82	M+	2'-O-Methyladenosine	282.1277	96.8	0.5610
25	9.55	M+	Sulprostone	448.2861	94.2	0.5233
26	4.97	M+	Tetraphenylphosphonium cation	339.055	95.7	0.4769
27	4.97	M+	Salicylic acid	139.045	95.5	0.4662
28	8.41	M+	7α,25-Dihydroxycholesterol	383.1757	96.3	0.4356
29	11.23	M+	1-Palmitoyl-2-stearoyl-sn-glycero-3-phospho-rac-1-glycerol	769.4771	90.8	0.3576
30	14.83	M+	5β-Androstane-3β,17β-diol	275.2062	97.8	0.3165
31	4.43	M+	Indole	118.0697	95.2	0.2927
32	4.29	M+	5'-S-Methyl-5'-thioadenosine	298.1028	100	0.2902
33	8.13	M+	Indole-6-carboxaldehyde	146.0652	98	0.2048
34	1.92	M+	Indole-3-pyruvic acid	204.1283	99.1	0.1944
35	1.48	M+	L-Tyrosine	182.0867	97.8	0.1559
36	1.78	[M+K]+	Cyamemazine	324.1013	98.4	0.1493
37	1.31	M+	Isoprostaglandin-F2α-IV	337.0953	100	0.1450

Supplementary Table S6. Metabolite Profiling of BMLE in Negative Ionization Mode, Showing Compounds with a Library Score of ≥90%, Ranked from Highest to Lowest Relative Peak Area, as Determined by HPLC-qTOF-MS

Rank	RT (min)	Mode	Tentatively Identified Metabolites	Precursor Mass	Library Score (%)	Relative Area (%)
1	15.39	M-	13S-Hydroxy-9Z,11E,15Z-octadecatrienoic acid	293.223	97.8	33.0637
2	15.41	[M-H]-	13S-Hydroxy-9Z,11E,15Z-octadecatrienoic acid	293.223	97.8	21.0483
3	16.14	M-	13R-Hydroxy-9Z,11E-octadecadienoic acid	295.2358	97.1	5.8622
4	16.63	M-	13-Keto-9Z,11E-octadecadienoic acid	293.2202	98.2	5.1930
5	13.94	M-	1-Palmitoyl-2-hydroxy-sn-glycero-3-phospho-(1'-rac-glycerol)	483.2785	99.1	4.5516
6	1.95	M-	Phenprobamate	164.0775	96.6	2.7971
7	3.13	M-	L-Tryptophan	203.0887	98.6	2.7268
8	16.85	M-	Chikusetsusponin IVa	793.5156	100	2.5377
9	15.42	M-	Asiatic acid	487.3415	100	1.9545
10	14.27	M-	1-Oleoyl-2-hydroxy-sn-glycero-3-phospho-(1'-rac-glycerol)	509.2892	98.6	1.8417
11	8.01	M-	Azelaic acid	187.1029	98.4	1.7399

12	1.67	M-	Adenine	134.0498	97.9	1.4113
13	7.51	M-	Hyperin	463.0889	98.7	1.2610
14	9.09	M-	(+)-Abscisic acid	263.1304	97.7	1.2464
15	10.79	M-	Butanedioic acid, 2-(4,4-dimethyl-2-methylenepen-tyl)	227.1273	99.1	1.2173
16	7.97	M-	Astragalin	447.0968	99.1	1.1417
17	7.01	M-	PolygalaxanthoneⅨ	551.2335	93.9	1.0377
18	11.2	M-	Pachymic acid	527.3352	98.7	1.0266
19	14.18	[M+AcO-H]-	Pedunculoside +HCOOH	695.4021	92.4	0.9170
20	1.2	M-	Melibiose	341.11	96.2	0.8824
21	17.79	M-	Pinolenic acid	277.2284	97.1	0.7645
22	8.26	M-	(.+-.)-Camphor-10-sulfonic acid	231.1598	94.1	0.7417
23	11.03	M-	Saikosaponin B4	811.4124	92.7	0.5672
24	6.65	M-	Isoferulic acid	193.0489	97.8	0.5564
25	7.05	M-	Peltatoside	595.1297	99.4	0.4773
26	1.96	M-	Daidzin	415.1597	91.9	0.4212
27	14.48	M-	Linoleic acid	279.1943	100	0.3596
28	14.51	M-	13S-Hydroperoxy-6Z,9Z,11E-octadecatrienoic acid	309.2046	91.8	0.3482
29	1.73	M-	N-Acetyl-L-phenylalanine	206.0812	98.0	0.3343
30	9.47	M-	Cinnamic acid	147.0443	99.5	0.3253
31	7.28	M-	Rutin	609.1449	97.8	0.3028
32	1.18	M-	D-(+)-Mannose	179.055	97.4	0.2871
33	4.81	M-	Esculin	339.0703	97.2	0.2739
34	18.14	M-	δ2-cis-Hexadecenoic acid	253.2181	97.3	0.2205
35	1.65	[M+AcO-H]-	Pantothenic acid	218.1027	100	0.2174
36	1.22	M-	3-Aminopentanoic acid	116.0703	95.1	0.1914
37	2.41	M-	5-Amino-2-methoxyphenol	138.0546	100	0.0767
38	2.69	M-	2,6-Dihydroxybenzoic acid	153.018	98.0	0.0767

Table S7. Primer Sequences.

**Genes**	**Primers**	**Sequences**
TNF-α	Sense	5′-TCTCATGCACCACCATCAAGGACT-3′

	Antisense	5′-CCACTCTCCCTTTGCAGAACTCA-3′
IL-1β	Sense	5′-AAGGGCTGCTTCCCAACCTTTGAC-3′
	Antisense	5′-ATACTGCCTGCCTGAAGCTCTTGT-3′
IL-6	Sense	5′-ATCCAGTTGCCTTCTTGGGACTGA-3′
	Antisense	5′-TAAGCCTCCGACTTGTGAAGTGGT-3′
GAPDH	Sense	5′-ACCACAGTCCATGCCATCAC-3′
	Antisense	5′-TCCACCACCCTGTTGCTGTA-3′
